# Supplementary material for: Seminoma and Embryonal Carcinoma Footprints Identified by Analysis of Integrated Genome-Wide Epigenetic and Expression Profiles of Germ Cell Cancer Cell Lines
Source: PLoS One. 2014 Jun 2;9(6):e98330. doi: 10.1371/journal.pone.0098330 (PMC4041891; doi:10.1371/journal.pone.0098330)
Supplement: File S1 — ZIP file containing DMRforPairs output for significant regions. Please start from the html files. (ZIP) [file pone.0098330.s008.zip › all.html]

 
 
 DMRforPairs output generated on Sun Oct 20 16:11:16 2013. Identified regions were set to contain at least 4 probes with a maximum distance of 200 bp between individual probes (n=30306). Regions in which median methylation levels (M-values) between the samples differed at least |1.4| (n=5139) (=relevant) were tested for statistical significance (significant: p 2). p.adj denotes the multiple testing corrected p-value (method:bonferroni). Gene symbols of overlapping transcripts are listed for the exact region and within a margin of 10000 bp of the region. 

	  
	  
		 Chr   
		 Start   
		 End   
		 Links   
		 Length   
		 n   
		 ID   
		 Class   
		 TCam2   
		 NCCIT   
		 Gene.Symbol   
		 dM   
		 p   
		 p.adj  
	  
  
 20
 
  61446962
 
  61448325
 
  PDF   STATS   ENSEMBL   UCSC 
 
 1364
 
 42
 
 25843
 
 tss;island
 
 0.04147
 
 0.74284
 
 OGFR; COL9A3 (margin: OGFR; COL9A3; OGFR-AS1)
 
  6.4503563
 
 1.19e-24
 
 6.12e-21
  
 
  
 6
 
  29973988
 
  29975427
 
  PDF   STATS   ENSEMBL   UCSC 
 
 1440
 
 47
 
 33643
 
 gene;island
 
 0.16563
 
 0.69279
 
 HLA-J; ZNRD1-AS1; HCG4P3 (margin: HLA-J; MCCD1P2; ZNRD1-AS1; HCG4P3)
 
  3.2184720
 
 8.15e-23
 
 4.19e-19
  
 
  
 10
 
 134599783
 
 134600998
 
  PDF   STATS   ENSEMBL   UCSC 
 
 1216
 
 30
 
 5432
 
 tss;island
 
 0.03311
 
 0.93434
 
 RP11-288G11.3; NKX6-2 (margin: INPP5A; RP11-288G11.3; NKX6-2)
 
  8.6746985
 
 1.69e-17
 
 8.69e-14
  
 
  
 20
 
  57425979
 
  57428032
 
  PDF   STATS   ENSEMBL   UCSC 
 
 2054
 
 65
 
 25433
 
 gene;tss;island
 
 0.49326
 
 0.03771
 
 GNAS; GNAS-AS1 (margin: GNAS; GNAS-AS1; RP1-309F20.3)
 
  4.4439975
 
 6.13e-17
 
 3.15e-13
  
 
  
 6
 
  32133929
 
  32135396
 
  PDF   STATS   ENSEMBL   UCSC 
 
 1468
 
 44
 
 34002
 
 gene;island
 
 0.30898
 
 0.88071
 
 PPT2; PPT2-EGFL8; EGFL8; AGPAT1 (margin: PPT2; PPT2-EGFL8; EGFL8; RNF5; PRRT1; AGPAT1)
 
  4.2625491
 
 1.57e-13
 
 8.07e-10
  
 
  
 10
 
 135050619
 
 135051351
 
  PDF   STATS   ENSEMBL   UCSC 
 
  733
 
 25
 
 5438
 
 tss
 
 0.32901
 
 0.02832
 
 VENTX (margin: KNDC1; UTF1; VENTX; MIR202)
 
  4.1355655
 
 1.90e-13
 
 9.76e-10
  
 
  
 11
 
   2721207
 
   2722713
 
  PDF   STATS   ENSEMBL   UCSC 
 
 1507
 
 41
 
 6448
 
 gene;island
 
 0.55738
 
 0.04265
 
 KCNQ1; KCNQ1OT1 (margin: KCNQ1; KCNQ1OT1)
 
  4.7930858
 
 4.09e-13
 
 2.10e-09
  
 
  
 11
 
   2019436
 
   2020560
 
  PDF   STATS   ENSEMBL   UCSC 
 
 1125
 
 36
 
 7035
 
 tss;island
 
 0.02256
 
 0.46131
 
 H19 (margin: AC051649.6; MRPL23-AS1; H19)
 
  4.8050808
 
 9.15e-13
 
 4.70e-09
  
 
  
 11
 
   2721243
 
   2722713
 
  PDF   STATS   ENSEMBL   UCSC 
 
 1471
 
 40
 
 7057
 
 tss
 
 0.55688
 
 0.04538
 
 KCNQ1; KCNQ1OT1 (margin: KCNQ1; KCNQ1OT1)
 
  4.7076717
 
 1.16e-12
 
 5.98e-09
  
 
  
 3
 
  37033625
 
  37034787
 
  PDF   STATS   ENSEMBL   UCSC 
 
 1163
 
 22
 
 27674
 
 gene
 
 0.39558
 
 0.01455
 
 MLH1; EPM2AIP1 (margin: MLH1; RP11-129K12.4; RP11-129K12.3; EPM2AIP1)
 
  5.5206075
 
 6.65e-12
 
 3.42e-08
  
 
  
 6
 
  31165290
 
  31166174
 
  PDF   STATS   ENSEMBL   UCSC 
 
  885
 
 29
 
 35694
 
 island
 
 0.02763
 
 0.27578
 
 HCG27; XXbac-BPG299F13.17; XXbac-BPG299F13.14 (margin: HCG27; XXbac-BPG299F13.17; XXbac-BPG299F13.14)
 
  3.8609353
 
 1.43e-11
 
 7.33e-08
  
 
  
 7
 
  95025611
 
  95026248
 
  PDF   STATS   ENSEMBL   UCSC 
 
  638
 
 20
 
 38614
 
 island
 
 0.02405
 
 0.93699
 
 PON1; PON3 (margin: PON1; PON3; PON2)
 
  9.5962745
 
 1.45e-11
 
 7.46e-08
  
 
  
 6
 
  30139421
 
  30140417
 
  PDF   STATS   ENSEMBL   UCSC 
 
  997
 
 26
 
 33676
 
 gene;island
 
 0.33140
 
 0.78489
 
 TRIM15 (margin: TRIM15; TRIM10; TRIM26)
 
  2.8841530
 
 4.73e-11
 
 2.43e-07
  
 
  
 7
 
  95025736
 
  95026248
 
  PDF   STATS   ENSEMBL   UCSC 
 
  513
 
 19
 
 37661
 
 tss
 
 0.02143
 
 0.93631
 
 PON1; PON3 (margin: PON1; PON3; PON2)
 
  9.4994230
 
 5.66e-11
 
 2.91e-07
  
 
  
 14
 
  65006688
 
  65007512
 
  PDF   STATS   ENSEMBL   UCSC 
 
  825
 
 18
 
 12907
 
 island
 
 0.03705
 
 0.90166
 
 HSPA2; RP11-973N13.4 (margin: ZBTB1; HSPA2; PPP1R36; RP11-973N13.4; RP11-973N13.5; RP11-973N13.3)
 
  9.0745610
 
 2.20e-10
 
 1.13e-06
  
 
  
 20
 
  61051317
 
  61052060
 
  PDF   STATS   ENSEMBL   UCSC 
 
  744
 
 18
 
 25833
 
 tss;island
 
 0.07097
 
 0.83112
 
 RP13-379O24.3; GATA5 (margin: RP13-379O24.3; GATA5)
 
  6.0445256
 
 2.20e-10
 
 1.13e-06
  
 
  
 6
 
  32116538
 
  32117725
 
  PDF   STATS   ENSEMBL   UCSC 
 
 1188
 
 27
 
 33995
 
 gene;island
 
 0.04216
 
 0.83493
 
 PRRT1 (margin: PPT2; PPT2-EGFL8; PRRT1)
 
  6.0977436
 
 7.75e-10
 
 3.98e-06
  
 
  
 7
 
  27204663
 
  27205658
 
  PDF   STATS   ENSEMBL   UCSC 
 
  996
 
 17
 
 38292
 
 island
 
 0.02568
 
 0.77553
 
 HOXA9; RP1-170O19.20 (margin: HOXA-AS3; RP1-170O19.21; HOXA-AS4; HOXA7; HOXA9; RP1-170O19.20; MIR196B; HOXA10)
 
  7.4569217
 
 8.57e-10
 
 4.40e-06
  
 
  
 1
 
  68515788
 
  68516713
 
  PDF   STATS   ENSEMBL   UCSC 
 
  926
 
 17
 
 3362
 
 island
 
 0.05192
 
 0.72874
 
 GNG12-AS1; DIRAS3; ARL5AP3 (margin: GNG12-AS1; RP4-609E1.2; AL157407.1; DIRAS3; ARL5AP3)
 
  5.7006948
 
 8.57e-10
 
 4.40e-06
  
 
  
 1
 
 111506233
 
 111507160
 
  PDF   STATS   ENSEMBL   UCSC 
 
  928
 
 17
 
 3576
 
 island
 
 0.01602
 
 0.31724
 
 LRIF1 (margin: RP11-96K19.2; LRIF1)
 
  4.9352237
 
 8.57e-10
 
 4.40e-06
  
 
  
 10
 
 135050619
 
 135051581
 
  PDF   STATS   ENSEMBL   UCSC 
 
  963
 
 28
 
 6330
 
 island
 
 0.33540
 
 0.02906
 
 VENTX (margin: KNDC1; UTF1; VENTX; MIR202)
 
  4.0939154
 
 1.03e-09
 
 5.31e-06
  
 
  
 3
 
  37033625
 
  37035399
 
  PDF   STATS   ENSEMBL   UCSC 
 
 1775
 
 43
 
 28178
 
 tss;island
 
 0.15867
 
 0.01963
 
 MLH1; EPM2AIP1 (margin: MLH1; RP11-129K12.4; RP11-129K12.3; EPM2AIP1)
 
  2.1706526
 
 1.87e-09
 
 9.60e-06
  
 
  
 X
 
 153775262
 
 153776107
 
  PDF   STATS   ENSEMBL   UCSC 
 
  846
 
 16
 
 41164
 
 gene;tss;island
 
 0.02333
 
 0.59012
 
 IKBKG; G6PD (margin: IKBKG; G6PD)
 
  5.7669406
 
 3.33e-09
 
 1.71e-05
  
 
  
 2
 
 198364525
 
 198365150
 
  PDF   STATS   ENSEMBL   UCSC 
 
  626
 
 16
 
 22931
 
 gene;tss;island
 
 0.02383
 
 0.47477
 
 HSPE1; MOB4; HSPD1 (margin: HSPE1; MOB4; HSPD1)
 
  5.4482660
 
 3.33e-09
 
 1.71e-05
  
 
  
 5
 
 135415693
 
 135416613
 
  PDF   STATS   ENSEMBL   UCSC 
 
  921
 
 16
 
 33084
 
 island
 
 0.05114
 
 0.53300
 
 VTRNA2-1 (margin: VTRNA2-1)
 
  4.4170546
 
 3.33e-09
 
 1.71e-05
  
 
  
 X
 
  47518016
 
  47518849
 
  PDF   STATS   ENSEMBL   UCSC 
 
  834
 
 17
 
 40982
 
 gene;island
 
 0.02432
 
 0.52067
 
 RP1-212G6.7; UXT (margin: RP1-212G6.7; ELK1; UXT)
 
  5.7362727
 
 3.43e-09
 
 1.76e-05
  
 
  
 12
 
  54410251
 
  54410727
 
  PDF   STATS   ENSEMBL   UCSC 
 
  477
 
 17
 
 10467
 
 island
 
 0.01517
 
 0.30179
 
 HOXC5; HOXC6; HOXC4 (margin: HOXC5; HOXC6; HOXC8; HOXC4; AC012531.25)
 
  4.9817744
 
 6.00e-09
 
 3.08e-05
  
 
  
 6
 
  32063394
 
  32064258
 
  PDF   STATS   ENSEMBL   UCSC 
 
  865
 
 28
 
 33984
 
 gene;island
 
 0.37747
 
 0.92532
 
 TNXB; ATF6B (margin: TNXB; ATF6B)
 
  4.1207349
 
 7.11e-09
 
 3.65e-05
  
 
  
 7
 
  27154262
 
  27155548
 
  PDF   STATS   ENSEMBL   UCSC 
 
 1287
 
 18
 
 36868
 
 gene;island
 
 0.70517
 
 0.08093
 
 HOXA-AS2; HOXA3 (margin: HOXA-AS2; HOXA2; HOXA3)
 
  4.1382084
 
 9.92e-09
 
 5.10e-05
  
 
  
 14
 
  65006688
 
  65007171
 
  PDF   STATS   ENSEMBL   UCSC 
 
  484
 
 15
 
 12431
 
 tss
 
 0.04363
 
 0.90142
 
 HSPA2; RP11-973N13.4 (margin: ZBTB1; HSPA2; PPP1R36; RP11-973N13.4; RP11-973N13.5)
 
  8.9444274
 
 1.29e-08
 
 6.63e-05
  
 
  
 3
 
 181428046
 
 181428697
 
  PDF   STATS   ENSEMBL   UCSC 
 
  652
 
 15
 
 27998
 
 gene;island
 
 0.43117
 
 0.04126
 
 SOX2-OT; SOX2 (margin: SOX2-OT; SOX2)
 
  4.3707113
 
 1.29e-08
 
 6.63e-05
  
 
  
 5
 
 140810051
 
 140811312
 
  PDF   STATS   ENSEMBL   UCSC 
 
 1262
 
 16
 
 31779
 
 gene;island
 
 0.34269
 
 0.89506
 
 PCDHGA1; PCDHGA2; PCDHGA3; PCDHGB1; PCDHGA4; PCDHGB2; PCDHGA5; PCDHGB3; PCDHGA6; PCDHGA7; PCDHGB4; PCDHGA8; PCDHGA9; PCDHGB6; PCDHGA10; PCDHGB7; PCDHGA11; PCDHGB8P; PCDHGA12 (margin: PCDHGA1; PCDHGA2; PCDHGA3; PCDHGB1; PCDHGA4; PCDHGB2; PCDHGA5; PCDHGB3; PCDHGA6; PCDHGA7; PCDHGB4; PCDHGA8; PCDHGA9; PCDHGB6; PCDHGA10; PCDHGB7; PCDHGA11; PCDHGB8P; PCDHGA12)
 
  4.2836194
 
 1.33e-08
 
 6.84e-05
  
 
  
 4
 
  89618324
 
  89619573
 
  PDF   STATS   ENSEMBL   UCSC 
 
 1250
 
 20
 
 30009
 
 gene
 
 0.04982
 
 0.91616
 
 HERC3; NAP1L5 (margin: HERC3; FAM13A-AS1; NAP1L5)
 
  7.1432720
 
 2.32e-08
 
 1.19e-04
  
 
  
 1
 
  25257505
 
  25258332
 
  PDF   STATS   ENSEMBL   UCSC 
 
  828
 
 21
 
 330
 
 gene;island
 
 0.33984
 
 0.09938
 
 RUNX3 (margin: RUNX3)
 
  1.9779462
 
 3.44e-08
 
 1.77e-04
  
 
  
 1
 
 165737775
 
 165738563
 
  PDF   STATS   ENSEMBL   UCSC 
 
  789
 
 14
 
 3921
 
 island
 
 0.89099
 
 0.01537
 
 RP11-466F5.8; TMCO1 (margin: RP11-466F5.8; TMCO1)
 
  9.3248973
 
 4.99e-08
 
 2.56e-04
  
 
  
 13
 
  36871646
 
  36872346
 
  PDF   STATS   ENSEMBL   UCSC 
 
  701
 
 14
 
 11639
 
 island
 
 0.94549
 
 0.09210
 
 SOHLH2; CCDC169-SOHLH2; CCDC169 (margin: SOHLH2; CCDC169-SOHLH2; CCDC169; SPG20)
 
  7.7474803
 
 4.99e-08
 
 2.56e-04
  
 
  
 X
 
 119005413
 
 119006122
 
  PDF   STATS   ENSEMBL   UCSC 
 
  710
 
 14
 
 41095
 
 gene;tss;island
 
 0.01651
 
 0.62465
 
 NDUFA1; RNF113A (margin: NDUFA1; RNF113A)
 
  6.6246427
 
 4.99e-08
 
 2.56e-04
  
 
  
 14
 
  75593334
 
  75594058
 
  PDF   STATS   ENSEMBL   UCSC 
 
  725
 
 14
 
 12984
 
 island
 
 0.69509
 
 0.02418
 
 RP11-950C14.7; NEK9 (margin: RP11-950C14.7; NEK9; HIF1AP1; TMED10)
 
  6.5505692
 
 4.99e-08
 
 2.56e-04
  
 
  
 10
 
  93392644
 
  93393509
 
  PDF   STATS   ENSEMBL   UCSC 
 
  866
 
 14
 
 5913
 
 island
 
 0.03365
 
 0.75510
 
 PPP1R3C (margin: PPP1R3C)
 
  6.4114792
 
 4.99e-08
 
 2.56e-04
  
 
  
 11
 
  47270041
 
  47270669
 
  PDF   STATS   ENSEMBL   UCSC 
 
  629
 
 14
 
 6586
 
 gene;tss;island
 
 0.02928
 
 0.45909
 
 NR1H3; ACP2 (margin: DDB2; NR1H3; ACP2)
 
  4.9582125
 
 4.99e-08
 
 2.56e-04
  
 
  
 3
 
 181428242
 
 181428697
 
  PDF   STATS   ENSEMBL   UCSC 
 
  456
 
 14
 
 28696
 
 tss
 
 0.41932
 
 0.04117
 
 SOX2-OT; SOX2 (margin: SOX2-OT; SOX2)
 
  4.3112893
 
 4.99e-08
 
 2.56e-04
  
 
  
 8
 
  97657072
 
  97657833
 
  PDF   STATS   ENSEMBL   UCSC 
 
  762
 
 14
 
 40410
 
 island
 
 0.07953
 
 0.57201
 
 CPQ (margin: CPQ)
 
  4.1257207
 
 4.99e-08
 
 2.56e-04
  
 
  
 X
 
  46404528
 
  46405125
 
  PDF   STATS   ENSEMBL   UCSC 
 
  598
 
 14
 
 41271
 
 tss;island
 
 0.08432
 
 0.59461
 
 ZNF674-AS1; ZNF674 (margin: ZNF674-AS1; ZNF674)
 
  3.7995064
 
 4.99e-08
 
 2.56e-04
  
 
  
 3
 
  50388115
 
  50388670
 
  PDF   STATS   ENSEMBL   UCSC 
 
  556
 
 14
 
 27766
 
 gene;island
 
 0.18697
 
 0.02753
 
 CYB561D2; XXcos-LUCA11.5; NPRL2 (margin: ZMYND10-AS1; CYB561D2; XXcos-LUCA11.5; RASSF1; ZMYND10; NPRL2; TMEM115; CACNA2D2)
 
  3.0848812
 
 4.99e-08
 
 2.56e-04
  
 
  
 11
 
   2889809
 
   2890725
 
  PDF   STATS   ENSEMBL   UCSC 
 
  917
 
 32
 
 7058
 
 tss
 
 0.34222
 
 0.86620
 
 KCNQ1DN (margin: KCNQ1DN; KCNQ1-AS1)
 
  3.3500619
 
 5.08e-08
 
 2.61e-04
  
 
  
 11
 
   2889602
 
   2890725
 
  PDF   STATS   ENSEMBL   UCSC 
 
 1124
 
 35
 
 7955
 
 island
 
 0.33717
 
 0.85799
 
 KCNQ1DN (margin: KCNQ1DN; KCNQ1-AS1)
 
  3.2575680
 
 7.38e-08
 
 3.79e-04
  
 
  
 1
 
  25257505
 
  25258264
 
  PDF   STATS   ENSEMBL   UCSC 
 
  760
 
 20
 
 1361
 
 tss
 
 0.35193
 
 0.08869
 
 RUNX3 (margin: RUNX3)
 
  2.0531215
 
 8.34e-08
 
 4.29e-04
  
 
  
 X
 
  48754982
 
  48755797
 
  PDF   STATS   ENSEMBL   UCSC 
 
  816
 
 21
 
 40988
 
 gene;tss;island
 
 0.24712
 
 0.02750
 
 PQBP1; TIMM17B (margin: PQBP1; TIMM17B; SLC35A2)
 
  3.0649730
 
 8.47e-08
 
 4.35e-04
  
 
  
 17
 
  17109570
 
  17110353
 
  PDF   STATS   ENSEMBL   UCSC 
 
  784
 
 14
 
 18027
 
 island
 
 0.05499
 
 0.94317
 
 MPRIP; PLD6; RP11-45M22.4 (margin: MPRIP; PLD6; RP11-45M22.4; FLCN)
 
  7.9799405
 
 9.97e-08
 
 5.12e-04
  
 
  
 11
 
  44331905
 
  44333192
 
  PDF   STATS   ENSEMBL   UCSC 
 
 1288
 
 39
 
 7240
 
 tss;island
 
 0.45237
 
 0.12446
 
 ALX4 (margin: ALX4)
 
  2.3384598
 
 1.44e-07
 
 7.40e-04
  
 
  
 6
 
  31165567
 
  31166174
 
  PDF   STATS   ENSEMBL   UCSC 
 
  608
 
 20
 
 33800
 
 gene
 
 0.02592
 
 0.28245
 
 HCG27; XXbac-BPG299F13.17; XXbac-BPG299F13.14 (margin: HCG27; XXbac-BPG299F13.17; XXbac-BPG299F13.14)
 
  3.8923894
 
 1.69e-07
 
 8.68e-04
  
 
  
 6
 
  32064491
 
  32065211
 
  PDF   STATS   ENSEMBL   UCSC 
 
  721
 
 29
 
 33985
 
 gene;island
 
 0.43415
 
 0.82096
 
 TNXB; ATF6B (margin: TNXB; ATF6B)
 
  2.7480875
 
 1.79e-07
 
 9.18e-04
  
 
  
 6
 
  26033394
 
  26034145
 
  PDF   STATS   ENSEMBL   UCSC 
 
  752
 
 13
 
 34618
 
 tss;island
 
 0.01093
 
 0.81336
 
 HIST1H3B; HIST1H2AB (margin: HIST1H4A; U91328.2; HIST1H3C; HIST1H4B; HIST1H3B; HIST1H2AB; HIST1H2BB)
 
  8.1533539
 
 1.92e-07
 
 9.88e-04
  
 
  
 19
 
  52391078
 
  52391789
 
  PDF   STATS   ENSEMBL   UCSC 
 
  712
 
 13
 
 22309
 
 island
 
 0.17681
 
 0.95794
 
 CTC-429C10.2; ZNF577; ZNF649 (margin: CTC-429C10.2; ZNF577; ZNF649)
 
  6.9022460
 
 1.92e-07
 
 9.88e-04
  
 
  
 1
 
 209979111
 
 209979779
 
  PDF   STATS   ENSEMBL   UCSC 
 
  669
 
 13
 
 4154
 
 island
 
 0.02320
 
 0.66796
 
 IRF6 (margin: IRF6)
 
  6.4912674
 
 1.92e-07
 
 9.88e-04
  
 
  
 10
 
  49812686
 
  49813434
 
  PDF   STATS   ENSEMBL   UCSC 
 
  749
 
 13
 
 5708
 
 island
 
 0.03878
 
 0.81259
 
 ARHGAP22 (margin: ARHGAP22)
 
  6.4085636
 
 1.92e-07
 
 9.88e-04
  
 
  
 3
 
 130745442
 
 130745959
 
  PDF   STATS   ENSEMBL   UCSC 
 
  518
 
 13
 
 28549
 
 tss;island
 
 0.02093
 
 0.59228
 
 NEK11; ASTE1 (margin: ATP2C1; NEK11; ASTE1)
 
  6.0674161
 
 1.92e-07
 
 9.88e-04
  
 
  
 19
 
  12146404
 
  12147014
 
  PDF   STATS   ENSEMBL   UCSC 
 
  611
 
 13
 
 21553
 
 island
 
 0.02165
 
 0.52029
 
 CTD-2006C1.2; ZNF433; CTD-2006C1.10 (margin: CTD-2006C1.2; ZNF433; CTD-2006C1.10; RNA5SP465; ZNF878)
 
  5.9833656
 
 1.92e-07
 
 9.88e-04
  
 
  
 5
 
  80597143
 
  80597828
 
  PDF   STATS   ENSEMBL   UCSC 
 
  686
 
 13
 
 31610
 
 gene;island
 
 0.44632
 
 0.02171
 
 ZCCHC9; CTC-281B15.1 (margin: ZCCHC9; CTC-281B15.1)
 
  4.9013482
 
 1.92e-07
 
 9.88e-04
  
 
  
 17
 
  46688182
 
  46688845
 
  PDF   STATS   ENSEMBL   UCSC 
 
  664
 
 13
 
 18455
 
 island
 
 0.01769
 
 0.24952
 
 HOXB7; HOXB8 (margin: HOXB-AS3; HOXB3; HOXB6; HOXB7; HOXB8; HOXB9)
 
  4.5123709
 
 1.92e-07
 
 9.88e-04
  
 
  
 19
 
  11784246
 
  11785337
 
  PDF   STATS   ENSEMBL   UCSC 
 
 1092
 
 15
 
 21544
 
 island
 
 0.03751
 
 0.51186
 
 ZNF833P (margin: ZNF833P; CTC-499B15.4; HNRNPA1P10)
 
  4.2370445
 
 2.45e-07
 
 1.26e-03
  
 
  
 20
 
  36148133
 
  36149455
 
  PDF   STATS   ENSEMBL   UCSC 
 
 1323
 
 41
 
 25378
 
 gene;tss;island
 
 0.93272
 
 0.67052
 
 NNAT; BLCAP (margin: NNAT; PPIAP3; RP11-425M5.5; BLCAP)
 
  2.5156524
 
 3.32e-07
 
 1.71e-03
  
 
  
 6
 
  27832728
 
  27833555
 
  PDF   STATS   ENSEMBL   UCSC 
 
  828
 
 13
 
 35507
 
 island
 
 0.02640
 
 0.94173
 
 HIST1H2AL; HIST1H2BPS2; HIST1H1B (margin: HIST1H2BN; HIST1H2AL; HIST1H2BPS2; HIST1H1B; HIST1H3I; HIST1H4L)
 
  8.8965867
 
 3.85e-07
 
 1.98e-03
  
 
  
 16
 
  88717374
 
  88717989
 
  PDF   STATS   ENSEMBL   UCSC 
 
  616
 
 13
 
 16176
 
 island
 
 0.78964
 
 0.09482
 
 CYBA; MVD (margin: IL17C; SNAI3-AS1; CYBA; MVD)
 
  4.8271486
 
 3.85e-07
 
 1.98e-03
  
 
  
 6
 
  30923241
 
  30924004
 
  PDF   STATS   ENSEMBL   UCSC 
 
  764
 
 17
 
 35684
 
 island
 
 0.35674
 
 0.04498
 
 DPCR1; HCG21 (margin: DPCR1; HCG21)
 
  3.6508076
 
 5.86e-07
 
 3.01e-03
  
 
  
 6
 
  28601269
 
  28601519
 
  PDF   STATS   ENSEMBL   UCSC 
 
  251
 
 14
 
 35529
 
 island
 
 0.89665
 
 0.45887
 
 RP11-373N24.2 (margin: RP11-373N24.2)
 
  3.3067822
 
 5.98e-07
 
 3.07e-03
  
 
  
 4
 
 144480206
 
 144480740
 
  PDF   STATS   ENSEMBL   UCSC 
 
  535
 
 12
 
 31283
 
 island
 
 0.00838
 
 0.85083
 
 SMARCA5; GUSBP5 (margin: SMARCA5; GUSBP5; RP11-481K16.2)
 
  9.8348486
 
 7.40e-07
 
 3.80e-03
  
 
  
 2
 
 187713554
 
 187714279
 
  PDF   STATS   ENSEMBL   UCSC 
 
  726
 
 12
 
 24912
 
 island
 
 0.03341
 
 0.91428
 
 ZSWIM2 (margin: ZSWIM2)
 
  8.0950021
 
 7.40e-07
 
 3.80e-03
  
 
  
 2
 
 113341711
 
 113342262
 
  PDF   STATS   ENSEMBL   UCSC 
 
  552
 
 12
 
 24586
 
 island
 
 0.02745
 
 0.85022
 
 CHCHD5 (margin: POLR1B; CHCHD5; AC012442.5; AC012442.6; Y_RNA)
 
  7.6092442
 
 7.40e-07
 
 3.80e-03
  
 
  
 12
 
   3862221
 
   3862597
 
  PDF   STATS   ENSEMBL   UCSC 
 
  377
 
 12
 
 10140
 
 island
 
 0.04487
 
 0.81025
 
 EFCAB4B (margin: EFCAB4B)
 
  6.7269686
 
 7.40e-07
 
 3.80e-03
  
 
  
 7
 
  99517136
 
  99517509
 
  PDF   STATS   ENSEMBL   UCSC 
 
  374
 
 12
 
 38677
 
 island
 
 0.80869
 
 0.03291
 
 TRIM4 (margin: RP4-604G5.1; TRIM4; GJC3)
 
  6.4912279
 
 7.40e-07
 
 3.80e-03
  
 
  
 3
 
  44753865
 
  44754587
 
  PDF   STATS   ENSEMBL   UCSC 
 
  723
 
 12
 
 28997
 
 island
 
 0.55584
 
 0.03533
 
 ZNF502; SOCS5P3 (margin: ZNF502; SOCS5P3)
 
  5.3032427
 
 7.40e-07
 
 3.80e-03
  
 
  
 8
 
   1764878
 
   1765477
 
  PDF   STATS   ENSEMBL   UCSC 
 
  600
 
 12
 
 39974
 
 island
 
 0.03227
 
 0.53461
 
 MIR596 (margin: MIR596; ARHGEF10)
 
  5.1649735
 
 7.40e-07
 
 3.80e-03
  
 
  
 22
 
  45680498
 
  45681036
 
  PDF   STATS   ENSEMBL   UCSC 
 
  539
 
 12
 
 27531
 
 island
 
 0.87809
 
 0.17172
 
 UPK3A (margin: CTA-268H5.9; UPK3A)
 
  4.9830655
 
 7.40e-07
 
 3.80e-03
  
 
  
 20
 
  61148178
 
  61149009
 
  PDF   STATS   ENSEMBL   UCSC 
 
  832
 
 12
 
 25449
 
 gene;island
 
 0.14751
 
 0.82802
 
 C20orf166; C20orf166-AS1 (margin: C20orf166; MIR1-1; C20orf166-AS1)
 
  4.9045080
 
 7.40e-07
 
 3.80e-03
  
 
  
 1
 
 233086060
 
 233086622
 
  PDF   STATS   ENSEMBL   UCSC 
 
  563
 
 12
 
 4318
 
 island
 
 0.38720
 
 0.02586
 
 NTPCR (margin: NTPCR)
 
  4.6239574
 
 7.40e-07
 
 3.80e-03
  
 
  
 11
 
  86383182
 
  86383585
 
  PDF   STATS   ENSEMBL   UCSC 
 
  404
 
 12
 
 6835
 
 gene
 
 0.05540
 
 0.56346
 
 ME3 (margin: ME3)
 
  4.4388528
 
 7.40e-07
 
 3.80e-03
  
 
  
 8
 
  11560299
 
  11560851
 
  PDF   STATS   ENSEMBL   UCSC 
 
  553
 
 12
 
 39489
 
 tss;island
 
 0.73098
 
 0.16740
 
 GATA4 (margin: GATA4)
 
  4.3119086
 
 7.40e-07
 
 3.80e-03
  
 
  
 1
 
  67217673
 
  67218275
 
  PDF   STATS   ENSEMBL   UCSC 
 
  603
 
 12
 
 3354
 
 island
 
 0.02380
 
 0.35889
 
 TCTEX1D1 (margin: SGIP1; TCTEX1D1)
 
  4.1607753
 
 7.40e-07
 
 3.80e-03
  
 
  
 10
 
 105212056
 
 105212813
 
  PDF   STATS   ENSEMBL   UCSC 
 
  758
 
 12
 
 6084
 
 island
 
 0.38751
 
 0.84230
 
 RP11-225H22.4; CALHM2; CALHM1 (margin: PDCD11; RP11-225H22.4; CALHM2; CALHM1)
 
  3.3984987
 
 7.40e-07
 
 3.80e-03
  
 
  
 17
 
  33775295
 
  33776116
 
  PDF   STATS   ENSEMBL   UCSC 
 
  822
 
 12
 
 18181
 
 island
 
 0.45261
 
 0.85822
 
 SLFN13 (margin: SLFN13; RP11-1094M14.7)
 
  2.7785276
 
 7.40e-07
 
 3.80e-03
  
 
  
 6
 
  30881112
 
  30881842
 
  PDF   STATS   ENSEMBL   UCSC 
 
  731
 
 23
 
 33772
 
 gene
 
 0.07611
 
 0.70181
 
 GTF2H4; VARS2 (margin: RN7SL175P; GTF2H4; VARS2)
 
  3.1918496
 
 1.18e-06
 
 6.04e-03
  
 
  
 19
 
  34174665
 
  34175554
 
  PDF   STATS   ENSEMBL   UCSC 
 
  890
 
 21
 
 19939
 
 gene;island
 
 0.08304
 
 0.73978
 
 CHST8 (margin: CHST8)
 
  4.9154038
 
 1.29e-06
 
 6.63e-03
  
 
  
 10
 
 135049991
 
 135050355
 
  PDF   STATS   ENSEMBL   UCSC 
 
  365
 
 13
 
 5437
 
 tss;island
 
 0.33678
 
 0.03069
 
 VENTX (margin: KNDC1; UTF1; VENTX; MIR202)
 
  3.4799025
 
 1.35e-06
 
 6.92e-03
  
 
  
 17
 
  17109651
 
  17110353
 
  PDF   STATS   ENSEMBL   UCSC 
 
  703
 
 12
 
 17129
 
 tss
 
 0.11191
 
 0.94317
 
 MPRIP; PLD6; RP11-45M22.4 (margin: MPRIP; PLD6; RP11-45M22.4; FLCN)
 
  7.0678872
 
 1.48e-06
 
 7.60e-03
  
 
  
 12
 
  54331892
 
  54332695
 
  PDF   STATS   ENSEMBL   UCSC 
 
  804
 
 12
 
 10451
 
 island
 
 0.01790
 
 0.54688
 
 HOXC13; HOXC-AS5 (margin: HOXC13; HOXC-AS5)
 
  5.9629610
 
 1.48e-06
 
 7.60e-03
  
 
  
 X
 
  48931388
 
  48932122
 
  PDF   STATS   ENSEMBL   UCSC 
 
  735
 
 12
 
 41781
 
 island
 
 0.20282
 
 0.02440
 
 PRAF2; WDR45 (margin: CCDC120; RNU4-52P; PRAF2; WDR45)
 
  3.5929592
 
 1.48e-06
 
 7.60e-03
  
 
  
 7
 
  27154262
 
  27155039
 
  PDF   STATS   ENSEMBL   UCSC 
 
  778
 
 14
 
 37425
 
 tss
 
 0.64307
 
 0.07005
 
 HOXA-AS2; HOXA3 (margin: HOXA-AS2; HOXA2; HOXA3)
 
  3.6498420
 
 1.50e-06
 
 7.69e-03
  
 
  
 4
 
 144480206
 
 144480561
 
  PDF   STATS   ENSEMBL   UCSC 
 
  356
 
 11
 
 30566
 
 tss
 
 0.00812
 
 0.80744
 
 SMARCA5; GUSBP5 (margin: SMARCA5; GUSBP5; RP11-481K16.2)
 
 10.0261262
 
 2.84e-06
 
 1.46e-02
  
 
  
 4
 
 103940711
 
 103941300
 
  PDF   STATS   ENSEMBL   UCSC 
 
  590
 
 11
 
 31159
 
 island
 
 0.01663
 
 0.89135
 
 SLC9B1; SLC9B2 (margin: SLC9B1; SLC9B2)
 
  9.6177656
 
 2.84e-06
 
 1.46e-02
  
 
  
 1
 
 161008127
 
 161008977
 
  PDF   STATS   ENSEMBL   UCSC 
 
  851
 
 11
 
 3884
 
 island
 
 0.03083
 
 0.96123
 
 F11R; TSTD1; USF1 (margin: RP11-544M22.3; F11R; TSTD1; USF1; ARHGAP30)
 
  9.5881658
 
 2.84e-06
 
 1.46e-02
  
 
  
 19
 
  54290737
 
  54291194
 
  PDF   STATS   ENSEMBL   UCSC 
 
  458
 
 11
 
 21003
 
 tss
 
 0.02519
 
 0.93216
 
 MIR371A; MIR372; MIR373; AC008753.4; MIR371B (margin: AC008753.3; MIR371A; MIR372; MIR373; AC008753.4; MIR371B; NLRP12)
 
  8.9918130
 
 2.84e-06
 
 1.46e-02
  
 
  
 15
 
  40544855
 
  40545511
 
  PDF   STATS   ENSEMBL   UCSC 
 
  657
 
 11
 
 13261
 
 gene;island
 
 0.01977
 
 0.89700
 
 RP11-133K1.2; PAK6; C15orf56 (margin: RP11-133K1.2; PAK6; C15orf56)
 
  8.7288591
 
 2.84e-06
 
 1.46e-02
  
 
  
 4
 
 188916496
 
 188916943
 
  PDF   STATS   ENSEMBL   UCSC 
 
  448
 
 11
 
 31437
 
 island
 
 0.96312
 
 0.11854
 
 ZFP42 (margin: ZFP42)
 
  7.6924825
 
 2.84e-06
 
 1.46e-02
  
 
  
 6
 
  30698584
 
  30698987
 
  PDF   STATS   ENSEMBL   UCSC 
 
  404
 
 11
 
 33755
 
 gene
 
 0.04332
 
 0.87591
 
 FLOT1 (margin: TUBB; XXbac-BPG252P9.10; XXbac-BPG252P9.9; FLOT1; Y_RNA; IER3)
 
  6.9424563
 
 2.84e-06
 
 1.46e-02
  
 
  
 19
 
  15342749
 
  15343425
 
  PDF   STATS   ENSEMBL   UCSC 
 
  677
 
 11
 
 19867
 
 gene;island
 
 0.75907
 
 0.04659
 
 EPHX3 (margin: AC004257.1; EPHX3; AC004257.3; BRD4)
 
  6.7635185
 
 2.84e-06
 
 1.46e-02
  
 
  
 19
 
  52900882
 
  52901307
 
  PDF   STATS   ENSEMBL   UCSC 
 
  426
 
 11
 
 22322
 
 island
 
 0.02292
 
 0.83850
 
 ZNF528; CTD-3018O17.3 (margin: ZNF880; ZNF528; CTD-3018O17.3)
 
  6.7313271
 
 2.84e-06
 
 1.46e-02
  
 
  
 6
 
  79576851
 
  79577260
 
  PDF   STATS   ENSEMBL   UCSC 
 
  410
 
 11
 
 36209
 
 island
 
 0.04090
 
 0.71060
 
 IRAK1BP1 (margin: IRAK1BP1)
 
  6.6960302
 
 2.84e-06
 
 1.46e-02
  
 
  
 19
 
  12075601
 
  12076348
 
  PDF   STATS   ENSEMBL   UCSC 
 
  748
 
 11
 
 21552
 
 island
 
 0.03051
 
 0.67328
 
 ZNF763 (margin: ZNF763; CTD-2006C1.12)
 
  6.6274624
 
 2.84e-06
 
 1.46e-02
  
 
  
 3
 
  57541583
 
  57542027
 
  PDF   STATS   ENSEMBL   UCSC 
 
  445
 
 11
 
 29184
 
 island
 
 0.01697
 
 0.56060
 
 PDE12 (margin: RNU6-483P; PDE12; DNAH12)
 
  6.3173231
 
 2.84e-06
 
 1.46e-02
  
 
  
 21
 
  39493025
 
  39493629
 
  PDF   STATS   ENSEMBL   UCSC 
 
  605
 
 11
 
 26504
 
 tss
 
 0.03988
 
 0.79839
 
 DSCR8; DSCR4 (margin: DSCR8; DSCR4)
 
  6.2863091
 
 2.84e-06
 
 1.46e-02
  
 
  
 5
 
  94982161
 
  94982715
 
  PDF   STATS   ENSEMBL   UCSC 
 
  555
 
 11
 
 32907
 
 island
 
 0.02558
 
 0.66273
 
 RFESD (margin: RFESD; SPATA9)
 
  6.1799987
 
 2.84e-06
 
 1.46e-02
  
 
  
 X
 
 134186198
 
 134186640
 
  PDF   STATS   ENSEMBL   UCSC 
 
  443
 
 11
 
 42048
 
 island
 
 0.01875
 
 0.54118
 
 FAM127B (margin: FAM127B)
 
  6.1499384
 
 2.84e-06
 
 1.46e-02
  
 
  
 13
 
 111267667
 
 111268031
 
  PDF   STATS   ENSEMBL   UCSC 
 
  365
 
 11
 
 11851
 
 island
 
 0.68442
 
 0.03776
 
 CARKD (margin: CARKD)
 
  6.0355614
 
 2.84e-06
 
 1.46e-02
  
 
  
 18
 
  22006316
 
  22006735
 
  PDF   STATS   ENSEMBL   UCSC 
 
  420
 
 11
 
 19403
 
 island
 
 0.02611
 
 0.60460
 
 IMPACT (margin: IMPACT; Y_RNA; RP11-178F10.1)
 
  6.0126704
 
 2.84e-06
 
 1.46e-02
  
 
  
 3
 
  44770851
 
  44771316
 
  PDF   STATS   ENSEMBL   UCSC 
 
  466
 
 11
 
 28998
 
 island
 
 0.54117
 
 0.01985
 
 ZNF501 (margin: ZNF502; ZNF501; KIAA1143)
 
  6.0018577
 
 2.84e-06
 
 1.46e-02
  
 
  
 X
 
  77150966
 
  77151316
 
  PDF   STATS   ENSEMBL   UCSC 
 
  351
 
 11
 
 41884
 
 island
 
 0.02388
 
 0.58420
 
 MAGT1 (margin: RN7SL460P; COX7B; MAGT1)
 
  5.9926617
 
 2.84e-06
 
 1.46e-02
  
 
  
 6
 
  18122938
 
  18123232
 
  PDF   STATS   ENSEMBL   UCSC 
 
  295
 
 11
 
 34594
 
 tss;island
 
 0.03106
 
 0.66955
 
 NHLRC1 (margin: NHLRC1; TPMT)
 
  5.9817945
 
 2.84e-06
 
 1.46e-02
  
 
  
 22
 
  22901569
 
  22902237
 
  PDF   STATS   ENSEMBL   UCSC 
 
  669
 
 11
 
 26963
 
 tss;island
 
 0.03729
 
 0.77074
 
 LL22NC03-63E9.3; PRAME (margin: LL22NC03-63E9.3; PRAME)
 
  5.9653167
 
 2.84e-06
 
 1.46e-02
  
 
  
 X
 
 153059760
 
 153060546
 
  PDF   STATS   ENSEMBL   UCSC 
 
  787
 
 11
 
 41587
 
 tss;island
 
 0.01792
 
 0.55873
 
 SSR4; IDH3G (margin: SRPK3; SSR4; IDH3G; PDZD4)
 
  5.9192423
 
 2.84e-06
 
 1.46e-02
  
 
  
 2
 
  24232603
 
  24232974
 
  PDF   STATS   ENSEMBL   UCSC 
 
  372
 
 11
 
 24087
 
 island
 
 0.25470
 
 0.94043
 
 MFSD2B (margin: UBXN2A; MFSD2B)
 
  5.7527291
 
 2.84e-06
 
 1.46e-02
  
 
  
 20
 
  32307885
 
  32308529
 
  PDF   STATS   ENSEMBL   UCSC 
 
  645
 
 11
 
 26062
 
 island
 
 0.02402
 
 0.61683
 
 PXMP4 (margin: ZNF341; PXMP4; RP4-553F4.2)
 
  5.7120271
 
 2.84e-06
 
 1.46e-02
  
 
  
 1
 
  68515788
 
  68516453
 
  PDF   STATS   ENSEMBL   UCSC 
 
  666
 
 11
 
 573
 
 gene
 
 0.05192
 
 0.66770
 
 GNG12-AS1; DIRAS3; ARL5AP3 (margin: GNG12-AS1; RP4-609E1.2; AL157407.1; DIRAS3; ARL5AP3)
 
  5.7006948
 
 2.84e-06
 
 1.46e-02
  
 
  
 17
 
   5094881
 
   5095455
 
  PDF   STATS   ENSEMBL   UCSC 
 
  575
 
 11
 
 17858
 
 island
 
 0.02901
 
 0.53716
 
 RP11-333E1.1; ZNF594 (margin: RP11-333E1.1; ZNF594)
 
  5.3598603
 
 2.84e-06
 
 1.46e-02
  
 
  
 5
 
 114937535
 
 114938459
 
  PDF   STATS   ENSEMBL   UCSC 
 
  925
 
 11
 
 31664
 
 gene;island
 
 0.12126
 
 0.88478
 
 AC010226.4; TICAM2; TMED7-TICAM2 (margin: AC010226.4; TICAM2; TMED7-TICAM2; TMED7)
 
  5.3465036
 
 2.84e-06
 
 1.46e-02
  
 
  
 18
 
  71815073
 
  71815372
 
  PDF   STATS   ENSEMBL   UCSC 
 
  300
 
 11
 
 19285
 
 tss;island
 
 0.01393
 
 0.37993
 
 TIMM21; FBXO15 (margin: TIMM21; FBXO15)
 
  5.3354094
 
 2.84e-06
 
 1.46e-02
  
 
  
 15
 
  98503714
 
  98503952
 
  PDF   STATS   ENSEMBL   UCSC 
 
  239
 
 11
 
 14465
 
 island
 
 0.02180
 
 0.46601
 
 ARRDC4 (margin: ARRDC4)
 
  5.2895525
 
 2.84e-06
 
 1.46e-02
  
 
  
 1
 
  74663583
 
  74664160
 
  PDF   STATS   ENSEMBL   UCSC 
 
  578
 
 11
 
 1692
 
 tss;island
 
 0.02126
 
 0.51291
 
 FPGT; FPGT-TNNI3K; TNNI3K; LRRIQ3 (margin: FPGT; FPGT-TNNI3K; TNNI3K; LRRIQ3)
 
  5.2542615
 
 2.84e-06
 
 1.46e-02
  
 
  
 7
 
  27195918
 
  27196555
 
  PDF   STATS   ENSEMBL   UCSC 
 
  638
 
 11
 
 38289
 
 island
 
 0.02090
 
 0.34711
 
 HOXA-AS3; RP1-170O19.21; HOXA7 (margin: HOXA-AS3; RP1-170O19.21; HOXA-AS4; HOXA3; HOXA6; HOXA7; HOXA9; RP1-170O19.20)
 
  4.7489723
 
 2.84e-06
 
 1.46e-02
  
 
  
 1
 
 111506577
 
 111507160
 
  PDF   STATS   ENSEMBL   UCSC 
 
  584
 
 11
 
 1817
 
 tss
 
 0.01602
 
 0.28924
 
 LRIF1 (margin: RP11-96K19.2; LRIF1)
 
  4.6632305
 
 2.84e-06
 
 1.46e-02
  
 
  
 3
 
  49066699
 
  49067151
 
  PDF   STATS   ENSEMBL   UCSC 
 
  453
 
 11
 
 29063
 
 island
 
 0.02423
 
 0.33064
 
 RP13-131K19.6; IMPDH2; QRICH1 (margin: NDUFAF3; RP13-131K19.6; DALRD3; MIR425; MIR191; IMPDH2; QRICH1)
 
  4.5444186
 
 2.84e-06
 
 1.46e-02
  
 
  
 1
 
  26233332
 
  26233709
 
  PDF   STATS   ENSEMBL   UCSC 
 
  378
 
 11
 
 1376
 
 tss;island
 
 0.04786
 
 0.49919
 
 STMN1; MIR3917 (margin: AL033528.1; STMN1; MIR3917)
 
  4.3360159
 
 2.84e-06
 
 1.46e-02
  
 
  
 6
 
 122931154
 
 122931702
 
  PDF   STATS   ENSEMBL   UCSC 
 
  549
 
 11
 
 34398
 
 gene;island
 
 0.06450
 
 0.57169
 
 PKIB (margin: PKIB)
 
  4.2750360
 
 2.84e-06
 
 1.46e-02
  
 
  
 1
 
  19615303
 
  19615702
 
  PDF   STATS   ENSEMBL   UCSC 
 
  400
 
 11
 
 1298
 
 tss;island
 
 0.11750
 
 0.81759
 
 AKR7A3 (margin: RP4-657E11.10; AKR7A3)
 
  4.2597936
 
 2.84e-06
 
 1.46e-02
  
 
  
 7
 
 150497065
 
 150497760
 
  PDF   STATS   ENSEMBL   UCSC 
 
  696
 
 11
 
 37214
 
 gene;tss;island
 
 0.30002
 
 0.85608
 
 TMEM176A; TMEM176B (margin: RP5-1051J4.4; TMEM176A; TMEM176B)
 
  4.0591203
 
 2.84e-06
 
 1.46e-02
  
 
  
 12
 
  51566379
 
  51567112
 
  PDF   STATS   ENSEMBL   UCSC 
 
  734
 
 11
 
 10396
 
 island
 
 0.44877
 
 0.04101
 
 TFCP2 (margin: RNU6-199P; TFCP2)
 
  4.0243918
 
 2.84e-06
 
 1.46e-02
  
 
  
 10
 
 105212236
 
 105212813
 
  PDF   STATS   ENSEMBL   UCSC 
 
  578
 
 11
 
 5321
 
 tss
 
 0.46931
 
 0.84486
 
 RP11-225H22.4; CALHM2; CALHM1 (margin: PDCD11; RP11-225H22.4; CALHM2; CALHM1)
 
  3.0306286
 
 2.84e-06
 
 1.46e-02
  
 
  
 1
 
   2344979
 
   2345475
 
  PDF   STATS   ENSEMBL   UCSC 
 
  497
 
 11
 
 1170
 
 tss;island
 
 0.55131
 
 0.16669
 
 PEX10 (margin: RER1; PLCH2; PEX10)
 
  2.5212420
 
 2.84e-06
 
 1.46e-02
  
 
  
 19
 
  38182773
 
  38183456
 
  PDF   STATS   ENSEMBL   UCSC 
 
  684
 
 11
 
 21925
 
 island
 
 0.16685
 
 0.04677
 
 ZFP30; ZNF781; CTD-2528L19.4; CTD-2528L19.3 (margin: ZFP30; ZNF781; CTD-2528L19.4; CTD-2528L19.3; ZNF607)
 
  2.2766135
 
 2.84e-06
 
 1.46e-02
  
 
  
 2
 
  27851409
 
  27852231
 
  PDF   STATS   ENSEMBL   UCSC 
 
  823
 
 16
 
 22595
 
 gene;tss
 
 0.42328
 
 0.06330
 
 ZNF512; RP11-158I13.2; GPN1; CCDC121 (margin: ZNF512; RP11-158I13.2; GPN1; CCDC121)
 
  2.9240664
 
 3.04e-06
 
 1.56e-02
  
 
  
 6
 
  30131001
 
  30132226
 
  PDF   STATS   ENSEMBL   UCSC 
 
 1226
 
 24
 
 33673
 
 gene
 
 0.36161
 
 0.63172
 
 TRIM15 (margin: TRIM15; TRIM10)
 
  1.6225805
 
 4.20e-06
 
 2.16e-02
  
 
  
 12
 
  54410251
 
  54410632
 
  PDF   STATS   ENSEMBL   UCSC 
 
  382
 
 12
 
 9729
 
 tss
 
 0.02116
 
 0.35661
 
 HOXC5; HOXC6; HOXC4 (margin: HOXC5; HOXC6; HOXC8; HOXC4; AC012531.25)
 
  5.0392270
 
 5.18e-06
 
 2.66e-02
  
 
  
 16
 
  88717464
 
  88717989
 
  PDF   STATS   ENSEMBL   UCSC 
 
  526
 
 11
 
 15380
 
 tss
 
 0.67959
 
 0.10529
 
 CYBA; MVD (margin: IL17C; SNAI3-AS1; CYBA; MVD)
 
  4.5981526
 
 5.67e-06
 
 2.91e-02
  
 
  
 3
 
  44626029
 
  44626860
 
  PDF   STATS   ENSEMBL   UCSC 
 
  832
 
 11
 
 28993
 
 island
 
 0.31663
 
 0.03272
 
 ZKSCAN7; ZNF660; ZNF197; RP11-944L7.4 (margin: ZKSCAN7; ZNF660; ZNF197; RP11-944L7.4; MPRIPP1)
 
  4.2749877
 
 5.67e-06
 
 2.91e-02
  
 
  
 2
 
  27805536
 
  27806117
 
  PDF   STATS   ENSEMBL   UCSC 
 
  582
 
 11
 
 24151
 
 island
 
 0.92048
 
 0.49255
 
 C2orf16; ZNF512; RP11-158I13.2; AC074091.1 (margin: C2orf16; ZNF512; RP11-158I13.2; AC074091.1)
 
  3.6634067
 
 5.67e-06
 
 2.91e-02
  
 
  
 3
 
  48632484
 
  48633144
 
  PDF   STATS   ENSEMBL   UCSC 
 
  661
 
 11
 
 29047
 
 island
 
 0.38028
 
 0.84593
 
 COL7A1 (margin: COL7A1; UQCRC1)
 
  3.5028384
 
 5.67e-06
 
 2.91e-02
  
 
  
 10
 
 106399877
 
 106400880
 
  PDF   STATS   ENSEMBL   UCSC 
 
 1004
 
 11
 
 6101
 
 island
 
 0.05418
 
 0.26667
 
 SORCS3 (margin: SORCS3)
 
  2.9596557
 
 5.67e-06
 
 2.91e-02
  
 
  
 6
 
 111580120
 
 111580939
 
  PDF   STATS   ENSEMBL   UCSC 
 
  820
 
 15
 
 36357
 
 island
 
 0.42121
 
 0.02368
 
 KIAA1919; RP11-428F8.2 (margin: KIAA1919; RP11-428F8.2)
 
  4.4708454
 
 6.55e-06
 
 3.37e-02
  
 
  
 15
 
  79383385
 
  79383980
 
  PDF   STATS   ENSEMBL   UCSC 
 
  596
 
 14
 
 13814
 
 tss;island
 
 0.02511
 
 0.18780
 
 RASGRF1 (margin: RASGRF1)
 
  2.8928587
 
 6.93e-06
 
 3.56e-02
  
 
  
 15
 
  45670865
 
  45671347
 
  PDF   STATS   ENSEMBL   UCSC 
 
  483
 
 13
 
 13304
 
 gene;tss;island
 
 0.91080
 
 0.06551
 
 GATM (margin: GATM)
 
  5.7891210
 
 8.65e-06
 
 4.45e-02
  
 
  
 3
 
  49026934
 
  49027409
 
  PDF   STATS   ENSEMBL   UCSC 
 
  476
 
 13
 
 29059
 
 island
 
 0.53558
 
 0.90155
 
 P4HTM; RP13-131K19.2 (margin: ARIH2; P4HTM; RP13-131K19.1; RP13-131K19.2)
 
  2.8137988
 
 8.65e-06
 
 4.45e-02
  
 
  
 6
 
  33160067
 
  33160604
 
  PDF   STATS   ENSEMBL   UCSC 
 
  538
 
 15
 
 35906
 
 island
 
 0.12477
 
 0.38724
 
 COL11A2; RXRB (margin: RNY4P10; SLC39A7; HSD17B8; COL11A2; RXRB)
 
  2.1706889
 
 8.82e-06
 
 4.53e-02
  
 
  
 19
 
  34174665
 
  34175419
 
  PDF   STATS   ENSEMBL   UCSC 
 
  755
 
 19
 
 20652
 
 tss
 
 0.08304
 
 0.73978
 
 CHST8 (margin: CHST8)
 
  4.3051983
 
 9.13e-06
 
 4.69e-02
  
 
  
 6
 
 160182438
 
 160182856
 
  PDF   STATS   ENSEMBL   UCSC 
 
  419
 
 10
 
 35257
 
 tss;island
 
 0.95376
 
 0.02036
 
 Not queried
 
  9.8521801
 
 1.08e-05
 
 5.56e-02
  
 
  
 18
 
  21718735
 
  21718979
 
  PDF   STATS   ENSEMBL   UCSC 
 
  245
 
 10
 
 19176
 
 tss;island
 
 0.03836
 
 0.94397
 
 Not queried
 
  9.2118827
 
 1.08e-05
 
 5.56e-02
  
 
  
 6
 
  27839751
 
  27840257
 
  PDF   STATS   ENSEMBL   UCSC 
 
  507
 
 10
 
 35509
 
 island
 
 0.02719
 
 0.94949
 
 Not queried
 
  9.0853110
 
 1.08e-05
 
 5.56e-02
  
 
  
 7
 
  27205159
 
  27205658
 
  PDF   STATS   ENSEMBL   UCSC 
 
  500
 
 10
 
 37431
 
 tss
 
 0.01815
 
 0.89898
 
 Not queried
 
  8.7055108
 
 1.08e-05
 
 5.56e-02
  
 
  
 20
 
  30134929
 
  30135362
 
  PDF   STATS   ENSEMBL   UCSC 
 
  434
 
 10
 
 25341
 
 gene;island
 
 0.97013
 
 0.08380
 
 Not queried
 
  8.5101569
 
 1.08e-05
 
 5.56e-02
  
 
  
 1
 
  59042745
 
  59043370
 
  PDF   STATS   ENSEMBL   UCSC 
 
  626
 
 10
 
 3307
 
 island
 
 0.02713
 
 0.88002
 
 Not queried
 
  7.8658141
 
 1.08e-05
 
 5.56e-02
  
 
  
 14
 
  55907007
 
  55907501
 
  PDF   STATS   ENSEMBL   UCSC 
 
  495
 
 10
 
 12841
 
 island
 
 0.04414
 
 0.82780
 
 Not queried
 
  7.6203332
 
 1.08e-05
 
 5.56e-02
  
 
  
 10
 
  50887472
 
  50887934
 
  PDF   STATS   ENSEMBL   UCSC 
 
  463
 
 10
 
 5719
 
 island
 
 0.02436
 
 0.84748
 
 Not queried
 
  7.6133624
 
 1.08e-05
 
 5.56e-02
  
 
  
 4
 
 188916496
 
 188916875
 
  PDF   STATS   ENSEMBL   UCSC 
 
  380
 
 10
 
 30662
 
 tss
 
 0.96089
 
 0.11016
 
 Not queried
 
  7.4709954
 
 1.08e-05
 
 5.56e-02
  
 
  
 11
 
  34460107
 
  34460557
 
  PDF   STATS   ENSEMBL   UCSC 
 
  451
 
 10
 
 8182
 
 island
 
 0.02558
 
 0.81088
 
 Not queried
 
  7.2314545
 
 1.08e-05
 
 5.56e-02
  
 
  
 14
 
  55493573
 
  55494452
 
  PDF   STATS   ENSEMBL   UCSC 
 
  880
 
 10
 
 12058
 
 gene;tss;island
 
 0.71947
 
 0.02434
 
 Not queried
 
  6.9498593
 
 1.08e-05
 
 5.56e-02
  
 
  
 10
 
  93392870
 
  93393509
 
  PDF   STATS   ENSEMBL   UCSC 
 
  640
 
 10
 
 5206
 
 tss
 
 0.03171
 
 0.72871
 
 Not queried
 
  6.7136384
 
 1.08e-05
 
 5.56e-02
  
 
  
 X
 
 134478461
 
 134479169
 
  PDF   STATS   ENSEMBL   UCSC 
 
  709
 
 10
 
 41541
 
 tss;island
 
 0.01536
 
 0.60682
 
 Not queried
 
  6.6754336
 
 1.08e-05
 
 5.56e-02
  
 
  
 4
 
  52917271
 
  52917660
 
  PDF   STATS   ENSEMBL   UCSC 
 
  390
 
 10
 
 30988
 
 island
 
 0.91120
 
 0.11379
 
 Not queried
 
  6.4966761
 
 1.08e-05
 
 5.56e-02
  
 
  
 7
 
  99517192
 
  99517509
 
  PDF   STATS   ENSEMBL   UCSC 
 
  318
 
 10
 
 37697
 
 tss
 
 0.82922
 
 0.04160
 
 Not queried
 
  6.4912279
 
 1.08e-05
 
 5.56e-02
  
 
  
 X
 
  79590789
 
  79591129
 
  PDF   STATS   ENSEMBL   UCSC 
 
  341
 
 10
 
 41889
 
 island
 
 0.02028
 
 0.57700
 
 Not queried
 
  6.2750854
 
 1.08e-05
 
 5.56e-02
  
 
  
 X
 
 134186228
 
 134186640
 
  PDF   STATS   ENSEMBL   UCSC 
 
  413
 
 10
 
 41538
 
 tss
 
 0.01861
 
 0.54315
 
 Not queried
 
  6.2304013
 
 1.08e-05
 
 5.56e-02
  
 
  
 6
 
  29599012
 
  29599390
 
  PDF   STATS   ENSEMBL   UCSC 
 
  379
 
 10
 
 33618
 
 gene;island
 
 0.63939
 
 0.03038
 
 Not queried
 
  6.2183201
 
 1.08e-05
 
 5.56e-02
  
 
  
 19
 
  52391234
 
  52391789
 
  PDF   STATS   ENSEMBL   UCSC 
 
  556
 
 10
 
 20963
 
 tss
 
 0.22893
 
 0.95652
 
 Not queried
 
  6.1564612
 
 1.08e-05
 
 5.56e-02
  
 
  
 10
 
  38299242
 
  38299653
 
  PDF   STATS   ENSEMBL   UCSC 
 
  412
 
 10
 
 5654
 
 island
 
 0.02191
 
 0.54101
 
 Not queried
 
  6.0616757
 
 1.08e-05
 
 5.56e-02
  
 
  
 22
 
  45608345
 
  45608713
 
  PDF   STATS   ENSEMBL   UCSC 
 
  369
 
 10
 
 26860
 
 gene
 
 0.12166
 
 0.88573
 
 Not queried
 
  5.9016746
 
 1.08e-05
 
 5.56e-02
  
 
  
 11
 
 118781408
 
 118781978
 
  PDF   STATS   ENSEMBL   UCSC 
 
  571
 
 10
 
 8838
 
 island
 
 0.04933
 
 0.74742
 
 Not queried
 
  5.8984067
 
 1.08e-05
 
 5.56e-02
  
 
  
 19
 
  12146565
 
  12147014
 
  PDF   STATS   ENSEMBL   UCSC 
 
  450
 
 10
 
 20451
 
 tss
 
 0.02184
 
 0.51254
 
 Not queried
 
  5.8927610
 
 1.08e-05
 
 5.56e-02
  
 
  
 5
 
 169659618
 
 169659950
 
  PDF   STATS   ENSEMBL   UCSC 
 
  333
 
 10
 
 33313
 
 island
 
 0.14094
 
 0.89681
 
 Not queried
 
  5.8292356
 
 1.08e-05
 
 5.56e-02
  
 
  
 19
 
  38754529
 
  38755330
 
  PDF   STATS   ENSEMBL   UCSC 
 
  802
 
 10
 
 21934
 
 island
 
 0.02182
 
 0.55594
 
 Not queried
 
  5.7751470
 
 1.08e-05
 
 5.56e-02
  
 
  
 8
 
  82192519
 
  82192931
 
  PDF   STATS   ENSEMBL   UCSC 
 
  413
 
 10
 
 40352
 
 island
 
 0.08027
 
 0.85785
 
 Not queried
 
  5.6659815
 
 1.08e-05
 
 5.56e-02
  
 
  
 X
 
 153626255
 
 153626754
 
  PDF   STATS   ENSEMBL   UCSC 
 
  500
 
 10
 
 42138
 
 island
 
 0.02379
 
 0.56661
 
 Not queried
 
  5.6454482
 
 1.08e-05
 
 5.56e-02
  
 
  
 12
 
   3862417
 
   3862597
 
  PDF   STATS   ENSEMBL   UCSC 
 
  181
 
 10
 
 9470
 
 tss
 
 0.07107
 
 0.80539
 
 Not queried
 
  5.6336400
 
 1.08e-05
 
 5.56e-02
  
 
  
 8
 
   1764878
 
   1765387
 
  PDF   STATS   ENSEMBL   UCSC 
 
  510
 
 10
 
 39472
 
 tss
 
 0.02434
 
 0.52256
 
 Not queried
 
  5.5187107
 
 1.08e-05
 
 5.56e-02
  
 
  
 X
 
  70752639
 
  70753023
 
  PDF   STATS   ENSEMBL   UCSC 
 
  385
 
 10
 
 41026
 
 gene;island
 
 0.01573
 
 0.37933
 
 Not queried
 
  5.4562237
 
 1.08e-05
 
 5.56e-02
  
 
  
 2
 
  24232603
 
  24232891
 
  PDF   STATS   ENSEMBL   UCSC 
 
  289
 
 10
 
 23182
 
 tss
 
 0.26393
 
 0.94448
 
 Not queried
 
  5.3520231
 
 1.08e-05
 
 5.56e-02
  
 
  
 20
 
  57089933
 
  57090317
 
  PDF   STATS   ENSEMBL   UCSC 
 
  385
 
 10
 
 26251
 
 island
 
 0.03950
 
 0.59734
 
 Not queried
 
  5.3369620
 
 1.08e-05
 
 5.56e-02
  
 
  
 5
 
 178487123
 
 178487576
 
  PDF   STATS   ENSEMBL   UCSC 
 
  454
 
 10
 
 32488
 
 tss
 
 0.01117
 
 0.33814
 
 Not queried
 
  5.2349706
 
 1.08e-05
 
 5.56e-02
  
 
  
 13
 
  20805094
 
  20805679
 
  PDF   STATS   ENSEMBL   UCSC 
 
  586
 
 10
 
 11068
 
 gene;island
 
 0.67702
 
 0.12682
 
 Not queried
 
  5.0889476
 
 1.08e-05
 
 5.56e-02
  
 
  
 12
 
  49110427
 
  49110861
 
  PDF   STATS   ENSEMBL   UCSC 
 
  435
 
 10
 
 10346
 
 island
 
 0.38376
 
 0.02198
 
 Not queried
 
  4.8484171
 
 1.08e-05
 
 5.56e-02
  
 
  
 19
 
  35454891
 
  35455389
 
  PDF   STATS   ENSEMBL   UCSC 
 
  499
 
 10
 
 21858
 
 island
 
 0.54793
 
 0.03754
 
 Not queried
 
  4.8409101
 
 1.08e-05
 
 5.56e-02
  
 
  
 1
 
 156163644
 
 156164205
 
  PDF   STATS   ENSEMBL   UCSC 
 
  562
 
 10
 
 3835
 
 island
 
 0.02918
 
 0.37322
 
 Not queried
 
  4.6831602
 
 1.08e-05
 
 5.56e-02
  
 
  
 5
 
  63257499
 
  63257941
 
  PDF   STATS   ENSEMBL   UCSC 
 
  443
 
 10
 
 32764
 
 island
 
 0.07590
 
 0.61682
 
 Not queried
 
  4.6734969
 
 1.08e-05
 
 5.56e-02
  
 
  
 X
 
  21676344
 
  21677134
 
  PDF   STATS   ENSEMBL   UCSC 
 
  791
 
 10
 
 41681
 
 island
 
 0.31919
 
 0.91372
 
 Not queried
 
  4.5450668
 
 1.08e-05
 
 5.56e-02
  
 
  
 1
 
  67217673
 
  67218135
 
  PDF   STATS   ENSEMBL   UCSC 
 
  463
 
 10
 
 1677
 
 tss
 
 0.02380
 
 0.35693
 
 Not queried
 
  4.1607753
 
 1.08e-05
 
 5.56e-02
  
 
  
 4
 
     53010
 
     53548
 
  PDF   STATS   ENSEMBL   UCSC 
 
  539
 
 10
 
 30665
 
 island
 
 0.03290
 
 0.40757
 
 Not queried
 
  4.1186395
 
 1.08e-05
 
 5.56e-02
  
 
  
 6
 
 160210606
 
 160211010
 
  PDF   STATS   ENSEMBL   UCSC 
 
  405
 
 10
 
 35258
 
 tss;island
 
 0.49134
 
 0.05351
 
 Not queried
 
  4.1118891
 
 1.08e-05
 
 5.56e-02
  
 
  
 8
 
  97657072
 
  97657432
 
  PDF   STATS   ENSEMBL   UCSC 
 
  361
 
 10
 
 39764
 
 tss
 
 0.07953
 
 0.54536
 
 Not queried
 
  4.0465896
 
 1.08e-05
 
 5.56e-02
  
 
  
 X
 
  53461213
 
  53461709
 
  PDF   STATS   ENSEMBL   UCSC 
 
  497
 
 10
 
 41809
 
 island
 
 0.03236
 
 0.37159
 
 Not queried
 
  4.0303692
 
 1.08e-05
 
 5.56e-02
  
 
  
 19
 
  52552025
 
  52552461
 
  PDF   STATS   ENSEMBL   UCSC 
 
  437
 
 10
 
 22313
 
 island
 
 0.09973
 
 0.55622
 
 Not queried
 
  3.7166485
 
 1.08e-05
 
 5.56e-02
  
 
  
 2
 
 177053274
 
 177053568
 
  PDF   STATS   ENSEMBL   UCSC 
 
  295
 
 10
 
 24876
 
 island
 
 0.02860
 
 0.25482
 
 Not queried
 
  3.6642071
 
 1.08e-05
 
 5.56e-02
  
 
  
 11
 
  65816463
 
  65816985
 
  PDF   STATS   ENSEMBL   UCSC 
 
  523
 
 10
 
 8475
 
 island
 
 0.84637
 
 0.22051
 
 Not queried
 
  3.6223724
 
 1.08e-05
 
 5.56e-02
  
 
  
 1
 
 153508511
 
 153508875
 
  PDF   STATS   ENSEMBL   UCSC 
 
  365
 
 10
 
 3752
 
 island
 
 0.42961
 
 0.88247
 
 Not queried
 
  3.0911794
 
 1.08e-05
 
 5.56e-02
  
 
  
 10
 
 106399877
 
 106400824
 
  PDF   STATS   ENSEMBL   UCSC 
 
  948
 
 10
 
 5333
 
 tss
 
 0.05861
 
 0.30550
 
 Not queried
 
  2.9686473
 
 1.08e-05
 
 5.56e-02
  
 
  
 3
 
  46506104
 
  46506554
 
  PDF   STATS   ENSEMBL   UCSC 
 
  451
 
 10
 
 29011
 
 island
 
 0.55056
 
 0.87391
 
 Not queried
 
  2.8876180
 
 1.08e-05
 
 5.56e-02
  
 
  
 6
 
 150346721
 
 150347053
 
  PDF   STATS   ENSEMBL   UCSC 
 
  333
 
 10
 
 35230
 
 tss
 
 0.17539
 
 0.67174
 
 Not queried
 
  2.7584383
 
 1.08e-05
 
 5.56e-02
  
 
  
 2
 
  26785120
 
  26785531
 
  PDF   STATS   ENSEMBL   UCSC 
 
  412
 
 10
 
 24116
 
 island
 
 0.50175
 
 0.85125
 
 Not queried
 
  2.7285445
 
 1.08e-05
 
 5.56e-02
  
 
  
 19
 
   9473565
 
   9473880
 
  PDF   STATS   ENSEMBL   UCSC 
 
  316
 
 10
 
 21461
 
 island
 
 0.90469
 
 0.61371
 
 Not queried
 
  2.7074373
 
 1.08e-05
 
 5.56e-02
  
 
  
 X
 
  48768632
 
  48769107
 
  PDF   STATS   ENSEMBL   UCSC 
 
  476
 
 10
 
 40989
 
 gene;island
 
 0.16511
 
 0.59385
 
 Not queried
 
  2.5064631
 
 1.08e-05
 
 5.56e-02
  
 
  
 X
 
  47863193
 
  47863707
 
  PDF   STATS   ENSEMBL   UCSC 
 
  515
 
 10
 
 41287
 
 tss;island
 
 0.10260
 
 0.02159
 
 Not queried
 
  2.3099071
 
 1.08e-05
 
 5.56e-02
  
 
  
 5
 
   1201359
 
   1201713
 
  PDF   STATS   ENSEMBL   UCSC 
 
  355
 
 10
 
 32553
 
 island
 
 0.47858
 
 0.79186
 
 Not queried
 
  1.9262145
 
 1.08e-05
 
 5.56e-02
  
 
  
 6
 
  31164776
 
  31164994
 
  PDF   STATS   ENSEMBL   UCSC 
 
  219
 
 11
 
 34739
 
 tss;island
 
 0.03173
 
 0.57107
 
 Not queried
 
  5.2089222
 
 1.13e-05
 
 5.83e-02
  
 
  
 17
 
  46608099
 
  46608570
 
  PDF   STATS   ENSEMBL   UCSC 
 
  472
 
 11
 
 18440
 
 island
 
 0.83132
 
 0.20544
 
 Not queried
 
  4.6683651
 
 1.13e-05
 
 5.83e-02
  
 
  
 6
 
  31275551
 
  31275881
 
  PDF   STATS   ENSEMBL   UCSC 
 
  331
 
 11
 
 35700
 
 island
 
 0.41452
 
 0.64915
 
 Not queried
 
  1.5567750
 
 1.13e-05
 
 5.83e-02
  
 
  
 7
 
  45961078
 
  45961569
 
  PDF   STATS   ENSEMBL   UCSC 
 
  492
 
 16
 
 37522
 
 tss;island
 
 0.06329
 
 0.55247
 
 Not queried
 
  3.5706045
 
 1.15e-05
 
 5.91e-02
  
 
  
 6
 
  30431563
 
  30431860
 
  PDF   STATS   ENSEMBL   UCSC 
 
  298
 
 15
 
 35620
 
 island
 
 0.19471
 
 0.48145
 
 Not queried
 
  2.1469557
 
 1.18e-05
 
 6.05e-02
  
 
  
 17
 
  46691820
 
  46692592
 
  PDF   STATS   ENSEMBL   UCSC 
 
  773
 
 12
 
 18456
 
 island
 
 0.03498
 
 0.34582
 
 Not queried
 
  3.6022461
 
 1.41e-05
 
 7.22e-02
  
 
  
 3
 
 147125712
 
 147127143
 
  PDF   STATS   ENSEMBL   UCSC 
 
 1432
 
 27
 
 28609
 
 tss
 
 0.02342
 
 0.08333
 
 Not queried
 
  2.3888091
 
 1.59e-05
 
 8.16e-02
  
 
  
 6
 
  30881112
 
  30882094
 
  PDF   STATS   ENSEMBL   UCSC 
 
  983
 
 28
 
 34729
 
 tss
 
 0.06824
 
 0.59315
 
 Not queried
 
  2.6650692
 
 1.77e-05
 
 9.08e-02
  
 
  
 6
 
  27840957
 
  27841423
 
  PDF   STATS   ENSEMBL   UCSC 
 
  467
 
 11
 
 34664
 
 tss;island
 
 0.45763
 
 0.93338
 
 Not queried
 
  3.8561089
 
 1.98e-05
 
 1.02e-01
  
 
  
 2
 
  25391505
 
  25391911
 
  PDF   STATS   ENSEMBL   UCSC 
 
  407
 
 11
 
 24101
 
 island
 
 0.50087
 
 0.05588
 
 Not queried
 
  3.4390528
 
 1.98e-05
 
 1.02e-01
  
 
  
 11
 
  71954982
 
  71955599
 
  PDF   STATS   ENSEMBL   UCSC 
 
  618
 
 11
 
 8607
 
 island
 
 0.21203
 
 0.01931
 
 Not queried
 
  2.7284235
 
 1.98e-05
 
 1.02e-01
  
 
  
 3
 
 147125712
 
 147127193
 
  PDF   STATS   ENSEMBL   UCSC 
 
 1482
 
 28
 
 29528
 
 island
 
 0.02220
 
 0.08050
 
 Not queried
 
  2.3141191
 
 2.10e-05
 
 1.08e-01
  
 
  
 5
 
 140753415
 
 140753924
 
  PDF   STATS   ENSEMBL   UCSC 
 
  510
 
 10
 
 31768
 
 gene;island
 
 0.04916
 
 0.84635
 
 Not queried
 
  6.2748130
 
 2.17e-05
 
 1.11e-01
  
 
  
 12
 
  54331892
 
  54332530
 
  PDF   STATS   ENSEMBL   UCSC 
 
  639
 
 10
 
 9723
 
 tss
 
 0.02034
 
 0.54688
 
 Not queried
 
  5.9629610
 
 2.17e-05
 
 1.11e-01
  
 
  
 11
 
  66360492
 
  66360803
 
  PDF   STATS   ENSEMBL   UCSC 
 
  312
 
 10
 
 7454
 
 tss;island
 
 0.02479
 
 0.39124
 
 Not queried
 
  4.5910503
 
 2.17e-05
 
 1.11e-01
  
 
  
 5
 
 122429966
 
 122430821
 
  PDF   STATS   ENSEMBL   UCSC 
 
  856
 
 10
 
 31673
 
 gene;island
 
 0.50959
 
 0.07284
 
 Not queried
 
  3.9001668
 
 2.17e-05
 
 1.11e-01
  
 
  
 6
 
  85474028
 
  85474595
 
  PDF   STATS   ENSEMBL   UCSC 
 
  568
 
 10
 
 35037
 
 tss;island
 
 0.01535
 
 0.09425
 
 Not queried
 
  3.1941686
 
 2.17e-05
 
 1.11e-01
  
 
  
 18
 
    904963
 
    905611
 
  PDF   STATS   ENSEMBL   UCSC 
 
  649
 
 10
 
 19000
 
 gene
 
 0.07955
 
 0.41910
 
 Not queried
 
  2.9125935
 
 2.17e-05
 
 1.11e-01
  
 
  
 17
 
   6679254
 
   6679781
 
  PDF   STATS   ENSEMBL   UCSC 
 
  528
 
 12
 
 17877
 
 island
 
 0.53709
 
 0.23442
 
 Not queried
 
  1.5730899
 
 2.22e-05
 
 1.14e-01
  
 
  
 7
 
  50861418
 
  50862266
 
  PDF   STATS   ENSEMBL   UCSC 
 
  849
 
 20
 
 37533
 
 tss;island
 
 0.30910
 
 0.04255
 
 Not queried
 
  3.9705790
 
 2.49e-05
 
 1.28e-01
  
 
  
 X
 
  71458680
 
  71459274
 
  PDF   STATS   ENSEMBL   UCSC 
 
  595
 
 11
 
 41031
 
 gene;island
 
 0.03580
 
 0.42108
 
 Not queried
 
  3.7308343
 
 3.40e-05
 
 1.75e-01
  
 
  
 6
 
  32119616
 
  32120324
 
  PDF   STATS   ENSEMBL   UCSC 
 
  709
 
 11
 
 35840
 
 island
 
 0.24073
 
 0.64990
 
 Not queried
 
  3.5435243
 
 3.40e-05
 
 1.75e-01
  
 
  
 X
 
  47695789
 
  47696363
 
  PDF   STATS   ENSEMBL   UCSC 
 
  575
 
 11
 
 41757
 
 island
 
 0.03819
 
 0.31500
 
 Not queried
 
  3.4600237
 
 3.40e-05
 
 1.75e-01
  
 
  
 1
 
 229569877
 
 229570288
 
  PDF   STATS   ENSEMBL   UCSC 
 
  412
 
 11
 
 2307
 
 tss
 
 0.06065
 
 0.44192
 
 Not queried
 
  2.6237794
 
 3.40e-05
 
 1.75e-01
  
 
  
 6
 
  28973318
 
  28973524
 
  PDF   STATS   ENSEMBL   UCSC 
 
  207
 
 11
 
 34682
 
 tss
 
 0.02329
 
 0.10097
 
 Not queried
 
  2.2062807
 
 3.40e-05
 
 1.75e-01
  
 
  
 4
 
 103940876
 
 103941300
 
  PDF   STATS   ENSEMBL   UCSC 
 
  425
 
  9
 
 30476
 
 tss
 
 0.01663
 
 0.90335
 
 Not queried
 
  9.6177656
 
 4.11e-05
 
 2.11e-01
  
 
  
 12
 
  89748726
 
  89749142
 
  PDF   STATS   ENSEMBL   UCSC 
 
  417
 
  9
 
 10639
 
 island
 
 0.89799
 
 0.04113
 
 Not queried
 
  9.3898814
 
 4.11e-05
 
 2.11e-01
  
 
  
 15
 
  91473059
 
  91473569
 
  PDF   STATS   ENSEMBL   UCSC 
 
  511
 
  9
 
 14433
 
 island
 
 0.01579
 
 0.88853
 
 Not queried
 
  8.7183897
 
 4.11e-05
 
 2.11e-01
  
 
  
 2
 
 166650606
 
 166650965
 
  PDF   STATS   ENSEMBL   UCSC 
 
  360
 
  9
 
 24794
 
 island
 
 0.03001
 
 0.83333
 
 Not queried
 
  8.0854998
 
 4.11e-05
 
 2.11e-01
  
 
  
 3
 
   9404422
 
   9404837
 
  PDF   STATS   ENSEMBL   UCSC 
 
  416
 
  9
 
 28812
 
 island
 
 0.87427
 
 0.03068
 
 Not queried
 
  7.9753462
 
 4.11e-05
 
 2.11e-01
  
 
  
 15
 
  40545143
 
  40545511
 
  PDF   STATS   ENSEMBL   UCSC 
 
  369
 
  9
 
 13556
 
 tss
 
 0.01759
 
 0.85795
 
 Not queried
 
  7.8219430
 
 4.11e-05
 
 2.11e-01
  
 
  
 2
 
 187713902
 
 187714279
 
  PDF   STATS   ENSEMBL   UCSC 
 
  378
 
  9
 
 23737
 
 tss
 
 0.03151
 
 0.82817
 
 Not queried
 
  7.7456128
 
 4.11e-05
 
 2.11e-01
  
 
  
 4
 
  52917271
 
  52917567
 
  PDF   STATS   ENSEMBL   UCSC 
 
  297
 
  9
 
 30343
 
 tss
 
 0.90564
 
 0.07837
 
 Not queried
 
  7.3399882
 
 4.11e-05
 
 2.11e-01
  
 
  
 6
 
  26021768
 
  26022273
 
  PDF   STATS   ENSEMBL   UCSC 
 
  506
 
  9
 
 35443
 
 island
 
 0.12071
 
 0.94958
 
 Not queried
 
  7.2331127
 
 4.11e-05
 
 2.11e-01
  
 
  
 14
 
  75593812
 
  75594058
 
  PDF   STATS   ENSEMBL   UCSC 
 
  247
 
  9
 
 12488
 
 tss
 
 0.76159
 
 0.02069
 
 Not queried
 
  7.1453620
 
 4.11e-05
 
 2.11e-01
  
 
  
 5
 
   1594579
 
   1595048
 
  PDF   STATS   ENSEMBL   UCSC 
 
  470
 
  9
 
 32575
 
 island
 
 0.96122
 
 0.14631
 
 Not queried
 
  7.0718567
 
 4.11e-05
 
 2.11e-01
  
 
  
 7
 
 138720786
 
 138721065
 
  PDF   STATS   ENSEMBL   UCSC 
 
  280
 
  9
 
 37862
 
 tss;island
 
 0.04033
 
 0.83668
 
 Not queried
 
  6.8945628
 
 4.11e-05
 
 2.11e-01
  
 
  
 6
 
  88031886
 
  88032455
 
  PDF   STATS   ENSEMBL   UCSC 
 
  570
 
  9
 
 34344
 
 gene;island
 
 0.03509
 
 0.83561
 
 Not queried
 
  6.7818546
 
 4.11e-05
 
 2.11e-01
  
 
  
 X
 
 134555540
 
 134555839
 
  PDF   STATS   ENSEMBL   UCSC 
 
  300
 
  9
 
 41542
 
 tss;island
 
 0.01719
 
 0.60411
 
 Not queried
 
  6.6940377
 
 4.11e-05
 
 2.11e-01
  
 
  
 5
 
 140207460
 
 140208201
 
  PDF   STATS   ENSEMBL   UCSC 
 
  742
 
  9
 
 31740
 
 gene;island
 
 0.08208
 
 0.85401
 
 Not queried
 
  6.6513936
 
 4.11e-05
 
 2.11e-01
  
 
  
 16
 
  67906784
 
  67907373
 
  PDF   STATS   ENSEMBL   UCSC 
 
  590
 
  9
 
 15999
 
 island
 
 0.01766
 
 0.57928
 
 Not queried
 
  6.5050831
 
 4.11e-05
 
 2.11e-01
  
 
  
 19
 
  44324747
 
  44325008
 
  PDF   STATS   ENSEMBL   UCSC 
 
  262
 
  9
 
 22061
 
 island
 
 0.01949
 
 0.66345
 
 Not queried
 
  6.4019501
 
 4.11e-05
 
 2.11e-01
  
 
  
 12
 
 123215151
 
 123215684
 
  PDF   STATS   ENSEMBL   UCSC 
 
  534
 
  9
 
 10060
 
 tss
 
 0.03539
 
 0.73595
 
 Not queried
 
  6.3173463
 
 4.11e-05
 
 2.11e-01
  
 
  
 3
 
  57541583
 
  57541949
 
  PDF   STATS   ENSEMBL   UCSC 
 
  367
 
  9
 
 28366
 
 tss
 
 0.01697
 
 0.56060
 
 Not queried
 
  6.3173231
 
 4.11e-05
 
 2.11e-01
  
 
  
 22
 
  19929066
 
  19929557
 
  PDF   STATS   ENSEMBL   UCSC 
 
  492
 
  9
 
 26751
 
 gene;island
 
 0.01904
 
 0.59052
 
 Not queried
 
  6.2744575
 
 4.11e-05
 
 2.11e-01
  
 
  
 3
 
  44770851
 
  44771113
 
  PDF   STATS   ENSEMBL   UCSC 
 
  263
 
  9
 
 28219
 
 tss
 
 0.58714
 
 0.01584
 
 Not queried
 
  6.2183211
 
 4.11e-05
 
 2.11e-01
  
 
  
 5
 
  94982161
 
  94982558
 
  PDF   STATS   ENSEMBL   UCSC 
 
  398
 
  9
 
 32126
 
 tss
 
 0.02727
 
 0.66651
 
 Not queried
 
  6.1799987
 
 4.11e-05
 
 2.11e-01
  
 
  
 10
 
  49813142
 
  49813434
 
  PDF   STATS   ENSEMBL   UCSC 
 
  293
 
  9
 
 5050
 
 tss
 
 0.05145
 
 0.79903
 
 Not queried
 
  6.1736148
 
 4.11e-05
 
 2.11e-01
  
 
  
 2
 
  27531124
 
  27531535
 
  PDF   STATS   ENSEMBL   UCSC 
 
  412
 
  9
 
 24138
 
 island
 
 0.05322
 
 0.80085
 
 Not queried
 
  6.1606333
 
 4.11e-05
 
 2.11e-01
  
 
  
 22
 
  45608428
 
  45608713
 
  PDF   STATS   ENSEMBL   UCSC 
 
  286
 
  9
 
 27177
 
 tss
 
 0.10123
 
 0.88663
 
 Not queried
 
  6.1544626
 
 4.11e-05
 
 2.11e-01
  
 
  
 10
 
  38299242
 
  38299574
 
  PDF   STATS   ENSEMBL   UCSC 
 
  333
 
  9
 
 5018
 
 tss
 
 0.01959
 
 0.60107
 
 Not queried
 
  6.0691511
 
 4.11e-05
 
 2.11e-01
  
 
  
 X
 
  47518552
 
  47518849
 
  PDF   STATS   ENSEMBL   UCSC 
 
  298
 
  9
 
 41285
 
 tss
 
 0.02006
 
 0.56523
 
 Not queried
 
  5.9889824
 
 4.11e-05
 
 2.11e-01
  
 
  
 X
 
  25033767
 
  25034241
 
  PDF   STATS   ENSEMBL   UCSC 
 
  475
 
  9
 
 41694
 
 island
 
 0.62523
 
 0.01895
 
 Not queried
 
  5.9134430
 
 4.11e-05
 
 2.11e-01
  
 
  
 X
 
 101854121
 
 101854594
 
  PDF   STATS   ENSEMBL   UCSC 
 
  474
 
  9
 
 41055
 
 gene
 
 0.01498
 
 0.54054
 
 Not queried
 
  5.8124664
 
 4.11e-05
 
 2.11e-01
  
 
  
 2
 
 214148856
 
 214149200
 
  PDF   STATS   ENSEMBL   UCSC 
 
  345
 
  9
 
 25026
 
 island
 
 0.01541
 
 0.47248
 
 Not queried
 
  5.8083462
 
 4.11e-05
 
 2.11e-01
  
 
  
 16
 
  82044738
 
  82045222
 
  PDF   STATS   ENSEMBL   UCSC 
 
  485
 
  9
 
 16093
 
 island
 
 0.05212
 
 0.88106
 
 Not queried
 
  5.8019874
 
 4.11e-05
 
 2.11e-01
  
 
  
 X
 
 153656771
 
 153657411
 
  PDF   STATS   ENSEMBL   UCSC 
 
  641
 
  9
 
 42141
 
 island
 
 0.03345
 
 0.61658
 
 Not queried
 
  5.7734750
 
 4.11e-05
 
 2.11e-01
  
 
  
 5
 
 169659618
 
 169659863
 
  PDF   STATS   ENSEMBL   UCSC 
 
  246
 
  9
 
 32424
 
 tss
 
 0.21994
 
 0.89731
 
 Not queried
 
  5.7179027
 
 4.11e-05
 
 2.11e-01
  
 
  
 13
 
 111267667
 
 111267991
 
  PDF   STATS   ENSEMBL   UCSC 
 
  325
 
  9
 
 11506
 
 tss
 
 0.68442
 
 0.04301
 
 Not queried
 
  5.6962011
 
 4.11e-05
 
 2.11e-01
  
 
  
 X
 
  46306474
 
  46306863
 
  PDF   STATS   ENSEMBL   UCSC 
 
  390
 
  9
 
 41737
 
 island
 
 0.03519
 
 0.59478
 
 Not queried
 
  5.4468927
 
 4.11e-05
 
 2.11e-01
  
 
  
 20
 
  57089953
 
  57090317
 
  PDF   STATS   ENSEMBL   UCSC 
 
  365
 
  9
 
 25811
 
 tss
 
 0.04436
 
 0.61360
 
 Not queried
 
  5.4012476
 
 4.11e-05
 
 2.11e-01
  
 
  
 17
 
   6899085
 
   6899380
 
  PDF   STATS   ENSEMBL   UCSC 
 
  296
 
  9
 
 17023
 
 tss
 
 0.03741
 
 0.56382
 
 Not queried
 
  5.3918714
 
 4.11e-05
 
 2.11e-01
  
 
  
 22
 
  45680498
 
  45680844
 
  PDF   STATS   ENSEMBL   UCSC 
 
  347
 
  9
 
 27178
 
 tss
 
 0.90286
 
 0.16456
 
 Not queried
 
  5.3916920
 
 4.11e-05
 
 2.11e-01
  
 
  
 15
 
  98503714
 
  98503878
 
  PDF   STATS   ENSEMBL   UCSC 
 
  165
 
  9
 
 13893
 
 tss
 
 0.02180
 
 0.46571
 
 Not queried
 
  5.2800086
 
 4.11e-05
 
 2.11e-01
  
 
  
 2
 
 228736089
 
 228736459
 
  PDF   STATS   ENSEMBL   UCSC 
 
  371
 
  9
 
 25131
 
 island
 
 0.02907
 
 0.46405
 
 Not queried
 
  4.9253857
 
 4.11e-05
 
 2.11e-01
  
 
  
 8
 
 144640306
 
 144640755
 
  PDF   STATS   ENSEMBL   UCSC 
 
  450
 
  9
 
 39420
 
 gene;island
 
 0.16314
 
 0.82852
 
 Not queried
 
  4.8248911
 
 4.11e-05
 
 2.11e-01
  
 
  
 5
 
  63257554
 
  63257941
 
  PDF   STATS   ENSEMBL   UCSC 
 
  388
 
  9
 
 32028
 
 tss
 
 0.07176
 
 0.59787
 
 Not queried
 
  4.8196334
 
 4.11e-05
 
 2.11e-01
  
 
  
 14
 
  45722289
 
  45722745
 
  PDF   STATS   ENSEMBL   UCSC 
 
  457
 
  9
 
 12788
 
 island
 
 0.01834
 
 0.36478
 
 Not queried
 
  4.7120737
 
 4.11e-05
 
 2.11e-01
  
 
  
 X
 
 134165954
 
 134166347
 
  PDF   STATS   ENSEMBL   UCSC 
 
  394
 
  9
 
 42047
 
 island
 
 0.03946
 
 0.52308
 
 Not queried
 
  4.6933363
 
 4.11e-05
 
 2.11e-01
  
 
  
 19
 
   9649241
 
   9649542
 
  PDF   STATS   ENSEMBL   UCSC 
 
  302
 
  9
 
 21464
 
 island
 
 0.37765
 
 0.02568
 
 Not queried
 
  4.5750779
 
 4.11e-05
 
 2.11e-01
  
 
  
 X
 
  21676481
 
  21677134
 
  PDF   STATS   ENSEMBL   UCSC 
 
  654
 
  9
 
 41220
 
 tss
 
 0.24764
 
 0.90738
 
 Not queried
 
  4.5406360
 
 4.11e-05
 
 2.11e-01
  
 
  
 17
 
  46688405
 
  46688845
 
  PDF   STATS   ENSEMBL   UCSC 
 
  441
 
  9
 
 17433
 
 tss
 
 0.02149
 
 0.33391
 
 Not queried
 
  4.5123709
 
 4.11e-05
 
 2.11e-01
  
 
  
 5
 
 140235435
 
 140236081
 
  PDF   STATS   ENSEMBL   UCSC 
 
  647
 
  9
 
 31743
 
 gene;island
 
 0.08028
 
 0.62538
 
 Not queried
 
  4.4880282
 
 4.11e-05
 
 2.11e-01
  
 
  
 1
 
  41444651
 
  41444871
 
  PDF   STATS   ENSEMBL   UCSC 
 
  221
 
  9
 
 1540
 
 tss;island
 
 0.34793
 
 0.05910
 
 Not queried
 
  4.4239383
 
 4.11e-05
 
 2.11e-01
  
 
  
 7
 
   5085252
 
   5085745
 
  PDF   STATS   ENSEMBL   UCSC 
 
  494
 
  9
 
 38145
 
 island
 
 0.26526
 
 0.02759
 
 Not queried
 
  4.4195377
 
 4.11e-05
 
 2.11e-01
  
 
  
 22
 
  42896105
 
  42896985
 
  PDF   STATS   ENSEMBL   UCSC 
 
  881
 
  9
 
 27500
 
 island
 
 0.13709
 
 0.72908
 
 Not queried
 
  4.4138613
 
 4.11e-05
 
 2.11e-01
  
 
  
 8
 
  57358130
 
  57358713
 
  PDF   STATS   ENSEMBL   UCSC 
 
  584
 
  9
 
 39251
 
 gene;island
 
 0.04290
 
 0.51867
 
 Not queried
 
  4.3774859
 
 4.11e-05
 
 2.11e-01
  
 
  
 7
 
  22539703
 
  22540154
 
  PDF   STATS   ENSEMBL   UCSC 
 
  452
 
  9
 
 38236
 
 island
 
 0.03317
 
 0.27603
 
 Not queried
 
  4.3467048
 
 4.11e-05
 
 2.11e-01
  
 
  
 12
 
  51566731
 
  51567112
 
  PDF   STATS   ENSEMBL   UCSC 
 
  382
 
  9
 
 9687
 
 tss
 
 0.44980
 
 0.03709
 
 Not queried
 
  4.2469036
 
 4.11e-05
 
 2.11e-01
  
 
  
 19
 
  56915403
 
  56915879
 
  PDF   STATS   ENSEMBL   UCSC 
 
  477
 
  9
 
 20199
 
 gene;island
 
 0.53309
 
 0.04938
 
 Not queried
 
  4.1606535
 
 4.11e-05
 
 2.11e-01
  
 
  
 3
 
 147077127
 
 147077774
 
  PDF   STATS   ENSEMBL   UCSC 
 
  648
 
  9
 
 29522
 
 island
 
 0.66074
 
 0.23629
 
 Not queried
 
  4.1472471
 
 4.11e-05
 
 2.11e-01
  
 
  
 5
 
 135416331
 
 135416613
 
  PDF   STATS   ENSEMBL   UCSC 
 
  283
 
  9
 
 32251
 
 tss
 
 0.06942
 
 0.55838
 
 Not queried
 
  4.0931389
 
 4.11e-05
 
 2.11e-01
  
 
  
 6
 
  26103952
 
  26104560
 
  PDF   STATS   ENSEMBL   UCSC 
 
  609
 
  9
 
 35451
 
 island
 
 0.03477
 
 0.31535
 
 Not queried
 
  4.0571282
 
 4.11e-05
 
 2.11e-01
  
 
  
 6
 
 151560831
 
 151561236
 
  PDF   STATS   ENSEMBL   UCSC 
 
  406
 
  9
 
 36508
 
 island
 
 0.68217
 
 0.10177
 
 Not queried
 
  3.9914448
 
 4.11e-05
 
 2.11e-01
  
 
  
 4
 
 118006405
 
 118006832
 
  PDF   STATS   ENSEMBL   UCSC 
 
  428
 
  9
 
 31210
 
 island
 
 0.02384
 
 0.34489
 
 Not queried
 
  3.9124841
 
 4.11e-05
 
 2.11e-01
  
 
  
 19
 
  52552120
 
  52552461
 
  PDF   STATS   ENSEMBL   UCSC 
 
  342
 
  9
 
 20969
 
 tss
 
 0.09424
 
 0.58127
 
 Not queried
 
  3.9050252
 
 4.11e-05
 
 2.11e-01
  
 
  
 4
 
 175135048
 
 175135997
 
  PDF   STATS   ENSEMBL   UCSC 
 
  950
 
  9
 
 31378
 
 island
 
 0.51892
 
 0.06967
 
 Not queried
 
  3.7185155
 
 4.11e-05
 
 2.11e-01
  
 
  
 1
 
 201368613
 
 201369031
 
  PDF   STATS   ENSEMBL   UCSC 
 
  419
 
  9
 
 4062
 
 island
 
 0.02824
 
 0.30520
 
 Not queried
 
  3.7101352
 
 4.11e-05
 
 2.11e-01
  
 
  
 2
 
  29337946
 
  29338258
 
  PDF   STATS   ENSEMBL   UCSC 
 
  313
 
  9
 
 23244
 
 tss
 
 0.54966
 
 0.08649
 
 Not queried
 
  3.6923928
 
 4.11e-05
 
 2.11e-01
  
 
  
 5
 
  72596646
 
  72597316
 
  PDF   STATS   ENSEMBL   UCSC 
 
  671
 
  9
 
 32807
 
 island
 
 0.84537
 
 0.25666
 
 Not queried
 
  3.6919189
 
 4.11e-05
 
 2.11e-01
  
 
  
 5
 
  78985425
 
  78985592
 
  PDF   STATS   ENSEMBL   UCSC 
 
  168
 
  9
 
 32088
 
 tss
 
 0.01945
 
 0.18971
 
 Not queried
 
  3.6888513
 
 4.11e-05
 
 2.11e-01
  
 
  
 6
 
  31165290
 
  31165512
 
  PDF   STATS   ENSEMBL   UCSC 
 
  223
 
  9
 
 34740
 
 tss
 
 0.02763
 
 0.23511
 
 Not queried
 
  3.5972134
 
 4.11e-05
 
 2.11e-01
  
 
  
 6
 
  30652202
 
  30652399
 
  PDF   STATS   ENSEMBL   UCSC 
 
  198
 
  9
 
 33739
 
 gene;island
 
 0.09131
 
 0.58464
 
 Not queried
 
  3.5475848
 
 4.11e-05
 
 2.11e-01
  
 
  
 1
 
  32827707
 
  32828191
 
  PDF   STATS   ENSEMBL   UCSC 
 
  485
 
  9
 
 394
 
 gene;tss;island
 
 0.04014
 
 0.32549
 
 Not queried
 
  3.4426254
 
 4.11e-05
 
 2.11e-01
  
 
  
 1
 
  19600673
 
  19601069
 
  PDF   STATS   ENSEMBL   UCSC 
 
  397
 
  9
 
 1297
 
 tss;island
 
 0.39067
 
 0.86334
 
 Not queried
 
  3.3329770
 
 4.11e-05
 
 2.11e-01
  
 
  
 X
 
 153637497
 
 153637827
 
  PDF   STATS   ENSEMBL   UCSC 
 
  331
 
  9
 
 41157
 
 gene;island
 
 0.37282
 
 0.04746
 
 Not queried
 
  3.2507621
 
 4.11e-05
 
 2.11e-01
  
 
  
 2
 
 220299484
 
 220299900
 
  PDF   STATS   ENSEMBL   UCSC 
 
  417
 
  9
 
 25086
 
 island
 
 0.32963
 
 0.81484
 
 Not queried
 
  3.1873779
 
 4.11e-05
 
 2.11e-01
  
 
  
 16
 
  31227884
 
  31228555
 
  PDF   STATS   ENSEMBL   UCSC 
 
  672
 
  9
 
 14705
 
 gene;island
 
 0.07693
 
 0.33924
 
 Not queried
 
  3.0760786
 
 4.11e-05
 
 2.11e-01
  
 
  
 X
 
  73834295
 
  73834821
 
  PDF   STATS   ENSEMBL   UCSC 
 
  527
 
  9
 
 41878
 
 island
 
 0.20476
 
 0.62604
 
 Not queried
 
  2.3445411
 
 4.11e-05
 
 2.11e-01
  
 
  
 16
 
  66586309
 
  66586745
 
  PDF   STATS   ENSEMBL   UCSC 
 
  437
 
  9
 
 15955
 
 island
 
 0.13606
 
 0.03255
 
 Not queried
 
  2.3364259
 
 4.11e-05
 
 2.11e-01
  
 
  
 5
 
   1201359
 
   1201698
 
  PDF   STATS   ENSEMBL   UCSC 
 
  340
 
  9
 
 31908
 
 tss
 
 0.46545
 
 0.77147
 
 Not queried
 
  1.8664184
 
 4.11e-05
 
 2.11e-01
  
 
  
 14
 
  96180576
 
  96181044
 
  PDF   STATS   ENSEMBL   UCSC 
 
  469
 
  9
 
 12562
 
 tss
 
 0.71469
 
 0.90315
 
 Not queried
 
  1.7329601
 
 4.11e-05
 
 2.11e-01
  
 
  
 1
 
  55266578
 
  55267293
 
  PDF   STATS   ENSEMBL   UCSC 
 
  716
 
 10
 
 3295
 
 island
 
 0.28248
 
 0.76152
 
 Not queried
 
  3.6007328
 
 4.33e-05
 
 2.23e-01
  
 
  
 2
 
  25391656
 
  25391911
 
  PDF   STATS   ENSEMBL   UCSC 
 
  256
 
 10
 
 23193
 
 tss
 
 0.51045
 
 0.05862
 
 Not queried
 
  3.4814437
 
 4.33e-05
 
 2.23e-01
  
 
  
 15
 
  83953690
 
  83954395
 
  PDF   STATS   ENSEMBL   UCSC 
 
  706
 
 18
 
 13837
 
 tss;island
 
 0.49770
 
 0.10274
 
 Not queried
 
  3.5951192
 
 4.89e-05
 
 2.51e-01
  
 
  
 12
 
  54409207
 
  54409770
 
  PDF   STATS   ENSEMBL   UCSC 
 
  564
 
 12
 
 9728
 
 tss;island
 
 0.91803
 
 0.42467
 
 Not queried
 
  3.9416192
 
 4.96e-05
 
 2.55e-01
  
 
  
 1
 
 235813839
 
 235814462
 
  PDF   STATS   ENSEMBL   UCSC 
 
  624
 
 11
 
 2332
 
 tss;island
 
 0.46118
 
 0.04185
 
 Not queried
 
  3.8721317
 
 5.39e-05
 
 2.77e-01
  
 
  
 19
 
  11689488
 
  11689997
 
  PDF   STATS   ENSEMBL   UCSC 
 
  510
 
 12
 
 20441
 
 tss;island
 
 0.63166
 
 0.92566
 
 Not queried
 
  2.8151347
 
 7.17e-05
 
 3.69e-01
  
 
  
 17
 
   7757933
 
   7758854
 
  PDF   STATS   ENSEMBL   UCSC 
 
  922
 
 17
 
 17941
 
 island
 
 0.57199
 
 0.87623
 
 Not queried
 
  2.0093468
 
 7.44e-05
 
 3.82e-01
  
 
  
 19
 
   6463949
 
   6464275
 
  PDF   STATS   ENSEMBL   UCSC 
 
  327
 
 10
 
 21394
 
 island
 
 0.02358
 
 0.42233
 
 Not queried
 
  4.0861601
 
 7.58e-05
 
 3.89e-01
  
 
  
 14
 
 106938234
 
 106938621
 
  PDF   STATS   ENSEMBL   UCSC 
 
  388
 
 10
 
 13219
 
 island
 
 0.49415
 
 0.89807
 
 Not queried
 
  3.4428283
 
 7.58e-05
 
 3.89e-01
  
 
  
 1
 
 222763046
 
 222763513
 
  PDF   STATS   ENSEMBL   UCSC 
 
  468
 
 10
 
 4216
 
 island
 
 0.20606
 
 0.03510
 
 Not queried
 
  2.7594074
 
 7.58e-05
 
 3.89e-01
  
 
  
 14
 
  24803679
 
  24804342
 
  PDF   STATS   ENSEMBL   UCSC 
 
  664
 
 10
 
 12718
 
 island
 
 0.03237
 
 0.14360
 
 Not queried
 
  1.7484829
 
 7.58e-05
 
 3.89e-01
  
 
  
 5
 
 137610127
 
 137610645
 
  PDF   STATS   ENSEMBL   UCSC 
 
  519
 
  9
 
 33092
 
 island
 
 0.01411
 
 0.49104
 
 Not queried
 
  6.2479812
 
 8.23e-05
 
 4.23e-01
  
 
  
 5
 
 140800398
 
 140800983
 
  PDF   STATS   ENSEMBL   UCSC 
 
  586
 
  9
 
 31777
 
 gene;island
 
 0.09843
 
 0.85198
 
 Not queried
 
  5.3009498
 
 8.23e-05
 
 4.23e-01
  
 
  
 5
 
 140723331
 
 140723899
 
  PDF   STATS   ENSEMBL   UCSC 
 
  569
 
  9
 
 31761
 
 gene;island
 
 0.25800
 
 0.85295
 
 Not queried
 
  4.7311711
 
 8.23e-05
 
 4.23e-01
  
 
  
 19
 
   6463949
 
   6464214
 
  PDF   STATS   ENSEMBL   UCSC 
 
  266
 
  9
 
 20354
 
 tss
 
 0.02467
 
 0.46467
 
 Not queried
 
  4.2269952
 
 8.23e-05
 
 4.23e-01
  
 
  
 10
 
 134901199
 
 134901689
 
  PDF   STATS   ENSEMBL   UCSC 
 
  491
 
  9
 
 6313
 
 island
 
 0.02691
 
 0.47961
 
 Not queried
 
  4.0256704
 
 8.23e-05
 
 4.23e-01
  
 
  
 2
 
 237077920
 
 237078733
 
  PDF   STATS   ENSEMBL   UCSC 
 
  814
 
  9
 
 25187
 
 island
 
 0.31656
 
 0.03653
 
 Not queried
 
  3.6107211
 
 8.23e-05
 
 4.23e-01
  
 
  
 X
 
  47695789
 
  47696239
 
  PDF   STATS   ENSEMBL   UCSC 
 
  451
 
  9
 
 41286
 
 tss
 
 0.06467
 
 0.31500
 
 Not queried
 
  3.4600237
 
 8.23e-05
 
 4.23e-01
  
 
  
 X
 
  48931699
 
  48932122
 
  PDF   STATS   ENSEMBL   UCSC 
 
  424
 
  9
 
 41306
 
 tss
 
 0.20012
 
 0.02953
 
 Not queried
 
  3.0286417
 
 8.23e-05
 
 4.23e-01
  
 
  
 X
 
  72782624
 
  72783164
 
  PDF   STATS   ENSEMBL   UCSC 
 
  541
 
  9
 
 41388
 
 tss;island
 
 0.09375
 
 0.34120
 
 Not queried
 
  2.3237810
 
 8.23e-05
 
 4.23e-01
  
 
  
 7
 
  27186993
 
  27187691
 
  PDF   STATS   ENSEMBL   UCSC 
 
  699
 
 11
 
 38285
 
 island
 
 0.05266
 
 0.60707
 
 Not queried
 
  4.8081116
 
 8.51e-05
 
 4.37e-01
  
 
  
 15
 
  45421578
 
  45422095
 
  PDF   STATS   ENSEMBL   UCSC 
 
  518
 
 11
 
 13618
 
 tss;island
 
 0.08782
 
 0.58619
 
 Not queried
 
  4.1565904
 
 8.51e-05
 
 4.37e-01
  
 
  
 3
 
  49026934
 
  49027283
 
  PDF   STATS   ENSEMBL   UCSC 
 
  350
 
 11
 
 28270
 
 tss
 
 0.53278
 
 0.90155
 
 Not queried
 
  2.8137988
 
 8.51e-05
 
 4.37e-01
  
 
  
 12
 
   9217390
 
   9218039
 
  PDF   STATS   ENSEMBL   UCSC 
 
  650
 
 11
 
 10209
 
 island
 
 0.53775
 
 0.83036
 
 Not queried
 
  2.5687366
 
 8.51e-05
 
 4.37e-01
  
 
  
 14
 
  96180319
 
  96181044
 
  PDF   STATS   ENSEMBL   UCSC 
 
  726
 
 11
 
 13084
 
 island
 
 0.68556
 
 0.88042
 
 Not queried
 
  1.7329601
 
 8.51e-05
 
 4.37e-01
  
 
  
 11
 
   2292361
 
   2293309
 
  PDF   STATS   ENSEMBL   UCSC 
 
  949
 
 32
 
 7044
 
 tss;island
 
 0.71152
 
 0.84319
 
 Not queried
 
  1.4508354
 
 9.20e-05
 
 4.73e-01
  
 
  
 18
 
  12253749
 
  12254566
 
  PDF   STATS   ENSEMBL   UCSC 
 
  818
 
 18
 
 19153
 
 tss
 
 0.32709
 
 0.64075
 
 Not queried
 
  1.9857354
 
 9.59e-05
 
 4.93e-01
  
 
  
 4
 
  89619023
 
  89619573
 
  PDF   STATS   ENSEMBL   UCSC 
 
  551
 
 12
 
 30448
 
 tss
 
 0.24005
 
 0.92819
 
 Not queried
 
  4.6516216
 
 1.03e-04
 
 5.28e-01
  
 
  
 2
 
   3642098
 
   3642967
 
  PDF   STATS   ENSEMBL   UCSC 
 
  870
 
 11
 
 23997
 
 island
 
 0.17632
 
 0.79224
 
 Not queried
 
  3.7218621
 
 1.28e-04
 
 6.56e-01
  
 
  
 17
 
  72889323
 
  72890001
 
  PDF   STATS   ENSEMBL   UCSC 
 
  679
 
 11
 
 18693
 
 island
 
 0.08564
 
 0.34967
 
 Not queried
 
  2.8916784
 
 1.28e-04
 
 6.56e-01
  
 
  
 20
 
  57414039
 
  57414596
 
  PDF   STATS   ENSEMBL   UCSC 
 
  558
 
 11
 
 25431
 
 gene;tss;island
 
 0.62223
 
 0.91910
 
 Not queried
 
  2.6466669
 
 1.28e-04
 
 6.56e-01
  
 
  
 20
 
  56725695
 
  56726185
 
  PDF   STATS   ENSEMBL   UCSC 
 
  491
 
 11
 
 26246
 
 island
 
 0.40501
 
 0.80101
 
 Not queried
 
  2.2314726
 
 1.28e-04
 
 6.56e-01
  
 
  
 4
 
 190861788
 
 190862268
 
  PDF   STATS   ENSEMBL   UCSC 
 
  481
 
 10
 
 31440
 
 island
 
 0.02123
 
 0.34614
 
 Not queried
 
  4.1291187
 
 1.30e-04
 
 6.68e-01
  
 
  
 11
 
 124735089
 
 124735554
 
  PDF   STATS   ENSEMBL   UCSC 
 
  466
 
 10
 
 8893
 
 island
 
 0.03569
 
 0.40253
 
 Not queried
 
  3.9731953
 
 1.30e-04
 
 6.68e-01
  
 
  
 11
 
  89224288
 
  89224799
 
  PDF   STATS   ENSEMBL   UCSC 
 
  512
 
 10
 
 6838
 
 gene;island
 
 0.14020
 
 0.02035
 
 Not queried
 
  3.2379082
 
 1.30e-04
 
 6.68e-01
  
 
  
 7
 
 150019955
 
 150020401
 
  PDF   STATS   ENSEMBL   UCSC 
 
  447
 
 10
 
 37207
 
 gene;island
 
 0.34360
 
 0.16759
 
 Not queried
 
  1.5668473
 
 1.30e-04
 
 6.68e-01
  
 
  
 5
 
 126409007
 
 126409573
 
  PDF   STATS   ENSEMBL   UCSC 
 
  567
 
 12
 
 33003
 
 island
 
 0.45541
 
 0.93733
 
 Not queried
 
  4.5734833
 
 1.44e-04
 
 7.41e-01
  
 
  
 16
 
  58548873
 
  58549320
 
  PDF   STATS   ENSEMBL   UCSC 
 
  448
 
  8
 
 15244
 
 tss;island
 
 0.01536
 
 0.94265
 
 Not queried
 
 10.0989693
 
 1.55e-04
 
 7.99e-01
  
 
  
 X
 
 133941107
 
 133941225
 
  PDF   STATS   ENSEMBL   UCSC 
 
  119
 
  8
 
 41113
 
 gene
 
 0.91639
 
 0.01550
 
 Not queried
 
  9.9503789
 
 1.55e-04
 
 7.99e-01
  
 
  
 1
 
 165738180
 
 165738563
 
  PDF   STATS   ENSEMBL   UCSC 
 
  384
 
  8
 
 2065
 
 tss
 
 0.95631
 
 0.02225
 
 Not queried
 
  9.3012773
 
 1.55e-04
 
 7.99e-01
  
 
  
 X
 
  20134346
 
  20134908
 
  PDF   STATS   ENSEMBL   UCSC 
 
  563
 
  8
 
 40946
 
 gene;island
 
 0.02038
 
 0.92385
 
 Not queried
 
  9.1731208
 
 1.55e-04
 
 7.99e-01
  
 
  
 6
 
  27832728
 
  27833095
 
  PDF   STATS   ENSEMBL   UCSC 
 
  368
 
  8
 
 34661
 
 tss
 
 0.03343
 
 0.95911
 
 Not queried
 
  8.9353346
 
 1.55e-04
 
 7.99e-01
  
 
  
 20
 
  30134929
 
  30135177
 
  PDF   STATS   ENSEMBL   UCSC 
 
  249
 
  8
 
 25623
 
 tss
 
 0.97013
 
 0.08380
 
 Not queried
 
  8.6844877
 
 1.55e-04
 
 7.99e-01
  
 
  
 7
 
  45026259
 
  45026583
 
  PDF   STATS   ENSEMBL   UCSC 
 
  325
 
  8
 
 37511
 
 tss;island
 
 0.93583
 
 0.02678
 
 Not queried
 
  8.5315561
 
 1.55e-04
 
 7.99e-01
  
 
  
 4
 
  48491941
 
  48492344
 
  PDF   STATS   ENSEMBL   UCSC 
 
  404
 
  8
 
 30981
 
 island
 
 0.05676
 
 0.90610
 
 Not queried
 
  8.1346431
 
 1.55e-04
 
 7.99e-01
  
 
  
 2
 
  73429084
 
  73429539
 
  PDF   STATS   ENSEMBL   UCSC 
 
  456
 
  8
 
 24374
 
 island
 
 0.95476
 
 0.07225
 
 Not queried
 
  7.9169084
 
 1.55e-04
 
 7.99e-01
  
 
  
 6
 
  84140564
 
  84140997
 
  PDF   STATS   ENSEMBL   UCSC 
 
  434
 
  8
 
 36228
 
 island
 
 0.03057
 
 0.91471
 
 Not queried
 
  7.7168090
 
 1.55e-04
 
 7.99e-01
  
 
  
 2
 
  45159894
 
  45160554
 
  PDF   STATS   ENSEMBL   UCSC 
 
  661
 
  8
 
 24222
 
 island
 
 0.90392
 
 0.06398
 
 Not queried
 
  7.7088224
 
 1.55e-04
 
 7.99e-01
  
 
  
 2
 
 113341711
 
 113341977
 
  PDF   STATS   ENSEMBL   UCSC 
 
  267
 
  8
 
 23541
 
 tss
 
 0.03128
 
 0.85022
 
 Not queried
 
  7.6015184
 
 1.55e-04
 
 7.99e-01
  
 
  
 13
 
  36872048
 
  36872346
 
  PDF   STATS   ENSEMBL   UCSC 
 
  299
 
  8
 
 11365
 
 tss
 
 0.94021
 
 0.06589
 
 Not queried
 
  7.5711392
 
 1.55e-04
 
 7.99e-01
  
 
  
 6
 
  10419684
 
  10420079
 
  PDF   STATS   ENSEMBL   UCSC 
 
  396
 
  8
 
 35368
 
 island
 
 0.04219
 
 0.79468
 
 Not queried
 
  7.3584041
 
 1.55e-04
 
 7.99e-01
  
 
  
 X
 
  83757470
 
  83757693
 
  PDF   STATS   ENSEMBL   UCSC 
 
  224
 
  8
 
 41408
 
 tss
 
 0.02390
 
 0.71093
 
 Not queried
 
  7.2995511
 
 1.55e-04
 
 7.99e-01
  
 
  
 22
 
  21386798
 
  21387058
 
  PDF   STATS   ENSEMBL   UCSC 
 
  261
 
  8
 
 27288
 
 island
 
 0.02648
 
 0.75974
 
 Not queried
 
  7.2599999
 
 1.55e-04
 
 7.99e-01
  
 
  
 6
 
 164392967
 
 164393860
 
  PDF   STATS   ENSEMBL   UCSC 
 
  894
 
  8
 
 36557
 
 island
 
 0.07239
 
 0.85147
 
 Not queried
 
  7.2166024
 
 1.55e-04
 
 7.99e-01
  
 
  
 11
 
  34460107
 
  34460386
 
  PDF   STATS   ENSEMBL   UCSC 
 
  280
 
  8
 
 7221
 
 tss
 
 0.02742
 
 0.81088
 
 Not queried
 
  7.1739237
 
 1.55e-04
 
 7.99e-01
  
 
  
 5
 
   1594676
 
   1595048
 
  PDF   STATS   ENSEMBL   UCSC 
 
  373
 
  8
 
 31912
 
 tss
 
 0.96342
 
 0.14518
 
 Not queried
 
  7.1371122
 
 1.55e-04
 
 7.99e-01
  
 
  
 8
 
  24772137
 
  24772513
 
  PDF   STATS   ENSEMBL   UCSC 
 
  377
 
  8
 
 39183
 
 gene;island
 
 0.06134
 
 0.91791
 
 Not queried
 
  7.0172843
 
 1.55e-04
 
 7.99e-01
  
 
  
 17
 
  21030030
 
  21030370
 
  PDF   STATS   ENSEMBL   UCSC 
 
  341
 
  8
 
 18082
 
 island
 
 0.01579
 
 0.66413
 
 Not queried
 
  6.8585462
 
 1.55e-04
 
 7.99e-01
  
 
  
 6
 
  79576851
 
  79577177
 
  PDF   STATS   ENSEMBL   UCSC 
 
  327
 
  8
 
 35015
 
 tss
 
 0.03229
 
 0.70641
 
 Not queried
 
  6.8276923
 
 1.55e-04
 
 7.99e-01
  
 
  
 1
 
  68512539
 
  68513063
 
  PDF   STATS   ENSEMBL   UCSC 
 
  525
 
  8
 
 572
 
 gene;island
 
 0.05520
 
 0.88016
 
 Not queried
 
  6.7386239
 
 1.55e-04
 
 7.99e-01
  
 
  
 19
 
   1132256
 
   1132537
 
  PDF   STATS   ENSEMBL   UCSC 
 
  282
 
  8
 
 19609
 
 gene
 
 0.02886
 
 0.80334
 
 Not queried
 
  6.7309294
 
 1.55e-04
 
 7.99e-01
  
 
  
 X
 
  92928508
 
  92928730
 
  PDF   STATS   ENSEMBL   UCSC 
 
  223
 
  8
 
 41413
 
 tss;island
 
 0.01513
 
 0.56524
 
 Not queried
 
  6.6510724
 
 1.55e-04
 
 7.99e-01
  
 
  
 17
 
  75315486
 
  75315868
 
  PDF   STATS   ENSEMBL   UCSC 
 
  383
 
  8
 
 16768
 
 gene
 
 0.03709
 
 0.79106
 
 Not queried
 
  6.6101744
 
 1.55e-04
 
 7.99e-01
  
 
  
 19
 
  52900882
 
  52900988
 
  PDF   STATS   ENSEMBL   UCSC 
 
  107
 
  8
 
 20977
 
 tss
 
 0.04823
 
 0.84052
 
 Not queried
 
  6.4719875
 
 1.55e-04
 
 7.99e-01
  
 
  
 19
 
  44324856
 
  44325008
 
  PDF   STATS   ENSEMBL   UCSC 
 
  153
 
  8
 
 20799
 
 tss
 
 0.01834
 
 0.65284
 
 Not queried
 
  6.4548823
 
 1.55e-04
 
 7.99e-01
  
 
  
 8
 
  82633454
 
  82633764
 
  PDF   STATS   ENSEMBL   UCSC 
 
  311
 
  8
 
 40356
 
 island
 
 0.01710
 
 0.50073
 
 Not queried
 
  6.3800065
 
 1.55e-04
 
 7.99e-01
  
 
  
 2
 
 172952883
 
 172953630
 
  PDF   STATS   ENSEMBL   UCSC 
 
  748
 
  8
 
 22886
 
 gene;island
 
 0.91054
 
 0.19184
 
 Not queried
 
  6.3716036
 
 1.55e-04
 
 7.99e-01
  
 
  
 1
 
  43751270
 
  43751563
 
  PDF   STATS   ENSEMBL   UCSC 
 
  294
 
  8
 
 1555
 
 tss
 
 0.02392
 
 0.56091
 
 Not queried
 
  6.3595522
 
 1.55e-04
 
 7.99e-01
  
 
  
 20
 
  29611652
 
  29611924
 
  PDF   STATS   ENSEMBL   UCSC 
 
  273
 
  8
 
 26029
 
 island
 
 0.02003
 
 0.61983
 
 Not queried
 
  6.3429648
 
 1.55e-04
 
 7.99e-01
  
 
  
 X
 
  77151065
 
  77151316
 
  PDF   STATS   ENSEMBL   UCSC 
 
  252
 
  8
 
 41400
 
 tss
 
 0.02757
 
 0.64422
 
 Not queried
 
  6.3381237
 
 1.55e-04
 
 7.99e-01
  
 
  
 2
 
  27531163
 
  27531535
 
  PDF   STATS   ENSEMBL   UCSC 
 
  373
 
  8
 
 23225
 
 tss
 
 0.05425
 
 0.75659
 
 Not queried
 
  6.2366131
 
 1.55e-04
 
 7.99e-01
  
 
  
 17
 
   4802692
 
   4803053
 
  PDF   STATS   ENSEMBL   UCSC 
 
  362
 
  8
 
 16323
 
 gene;island
 
 0.01948
 
 0.60940
 
 Not queried
 
  6.1435770
 
 1.55e-04
 
 7.99e-01
  
 
  
 2
 
  63285365
 
  63286049
 
  PDF   STATS   ENSEMBL   UCSC 
 
  685
 
  8
 
 24304
 
 island
 
 0.90755
 
 0.09797
 
 Not queried
 
  6.1185051
 
 1.55e-04
 
 7.99e-01
  
 
  
 4
 
  54959419
 
  54960259
 
  PDF   STATS   ENSEMBL   UCSC 
 
  841
 
  8
 
 30998
 
 island
 
 0.78149
 
 0.05652
 
 Not queried
 
  6.0597194
 
 1.55e-04
 
 7.99e-01
  
 
  
 3
 
 138658243
 
 138659021
 
  PDF   STATS   ENSEMBL   UCSC 
 
  779
 
  8
 
 29486
 
 island
 
 0.81503
 
 0.06970
 
 Not queried
 
  6.0531117
 
 1.55e-04
 
 7.99e-01
  
 
  
 3
 
  44753865
 
  44754131
 
  PDF   STATS   ENSEMBL   UCSC 
 
  267
 
  8
 
 28218
 
 tss
 
 0.57126
 
 0.02730
 
 Not queried
 
  6.0305538
 
 1.55e-04
 
 7.99e-01
  
 
  
 1
 
 209957965
 
 209958343
 
  PDF   STATS   ENSEMBL   UCSC 
 
  379
 
  8
 
 2228
 
 tss;island
 
 0.02006
 
 0.58789
 
 Not queried
 
  6.0284285
 
 1.55e-04
 
 7.99e-01
  
 
  
 21
 
  36041334
 
  36041699
 
  PDF   STATS   ENSEMBL   UCSC 
 
  366
 
  8
 
 26621
 
 island
 
 0.64654
 
 0.02389
 
 Not queried
 
  5.9458677
 
 1.55e-04
 
 7.99e-01
  
 
  
 11
 
  65554042
 
  65554410
 
  PDF   STATS   ENSEMBL   UCSC 
 
  369
 
  8
 
 7420
 
 tss;island
 
 0.03046
 
 0.62839
 
 Not queried
 
  5.9361904
 
 1.55e-04
 
 7.99e-01
  
 
  
 1
 
   2985218
 
   2985720
 
  PDF   STATS   ENSEMBL   UCSC 
 
  503
 
  8
 
 1176
 
 tss;island
 
 0.03953
 
 0.64337
 
 Not queried
 
  5.9247330
 
 1.55e-04
 
 7.99e-01
  
 
  
 19
 
   4831543
 
   4832024
 
  PDF   STATS   ENSEMBL   UCSC 
 
  482
 
  8
 
 21354
 
 island
 
 0.06593
 
 0.80716
 
 Not queried
 
  5.9073906
 
 1.55e-04
 
 7.99e-01
  
 
  
 4
 
  15704393
 
  15704844
 
  PDF   STATS   ENSEMBL   UCSC 
 
  452
 
  8
 
 30888
 
 island
 
 0.12819
 
 0.87699
 
 Not queried
 
  5.8457246
 
 1.55e-04
 
 7.99e-01
  
 
  
 11
 
  62474725
 
  62475078
 
  PDF   STATS   ENSEMBL   UCSC 
 
  354
 
  8
 
 6625
 
 gene;tss;island
 
 0.72171
 
 0.04626
 
 Not queried
 
  5.7952804
 
 1.55e-04
 
 7.99e-01
  
 
  
 1
 
 209979487
 
 209979779
 
  PDF   STATS   ENSEMBL   UCSC 
 
  293
 
  8
 
 2229
 
 tss
 
 0.02740
 
 0.59386
 
 Not queried
 
  5.7082250
 
 1.55e-04
 
 7.99e-01
  
 
  
 18
 
  22006316
 
  22006514
 
  PDF   STATS   ENSEMBL   UCSC 
 
  199
 
  8
 
 19180
 
 tss
 
 0.02790
 
 0.60540
 
 Not queried
 
  5.6675076
 
 1.55e-04
 
 7.99e-01
  
 
  
 11
 
  45825356
 
  45825579
 
  PDF   STATS   ENSEMBL   UCSC 
 
  224
 
  8
 
 7245
 
 tss;island
 
 0.85411
 
 0.08061
 
 Not queried
 
  5.5738386
 
 1.55e-04
 
 7.99e-01
  
 
  
 6
 
 166876490
 
 166877038
 
  PDF   STATS   ENSEMBL   UCSC 
 
  549
 
  8
 
 34480
 
 gene
 
 0.16334
 
 0.84051
 
 Not queried
 
  5.5441381
 
 1.55e-04
 
 7.99e-01
  
 
  
 17
 
   5095195
 
   5095455
 
  PDF   STATS   ENSEMBL   UCSC 
 
  261
 
  8
 
 17009
 
 tss
 
 0.02335
 
 0.54302
 
 Not queried
 
  5.4407169
 
 1.55e-04
 
 7.99e-01
  
 
  
 19
 
  12267308
 
  12267796
 
  PDF   STATS   ENSEMBL   UCSC 
 
  489
 
  8
 
 21559
 
 island
 
 0.03159
 
 0.51812
 
 Not queried
 
  5.3375466
 
 1.55e-04
 
 7.99e-01
  
 
  
 7
 
 105221826
 
 105222180
 
  PDF   STATS   ENSEMBL   UCSC 
 
  355
 
  8
 
 38757
 
 island
 
 0.03006
 
 0.53788
 
 Not queried
 
  5.3206474
 
 1.55e-04
 
 7.99e-01
  
 
  
 X
 
  70712215
 
  70712810
 
  PDF   STATS   ENSEMBL   UCSC 
 
  596
 
  8
 
 41025
 
 gene;island
 
 0.96031
 
 0.36695
 
 Not queried
 
  5.3205029
 
 1.55e-04
 
 7.99e-01
  
 
  
 6
 
 117086850
 
 117087238
 
  PDF   STATS   ENSEMBL   UCSC 
 
  389
 
  8
 
 36371
 
 island
 
 0.03472
 
 0.62871
 
 Not queried
 
  5.2904405
 
 1.55e-04
 
 7.99e-01
  
 
  
 16
 
   5121709
 
   5122251
 
  PDF   STATS   ENSEMBL   UCSC 
 
  543
 
  8
 
 15650
 
 island
 
 0.02279
 
 0.52561
 
 Not queried
 
  5.2860064
 
 1.55e-04
 
 7.99e-01
  
 
  
 2
 
  74153795
 
  74154363
 
  PDF   STATS   ENSEMBL   UCSC 
 
  569
 
  8
 
 24384
 
 island
 
 0.40705
 
 0.01616
 
 Not queried
 
  5.2833858
 
 1.55e-04
 
 7.99e-01
  
 
  
 13
 
  20805380
 
  20805679
 
  PDF   STATS   ENSEMBL   UCSC 
 
  300
 
  8
 
 11302
 
 tss
 
 0.63613
 
 0.07719
 
 Not queried
 
  5.2458078
 
 1.55e-04
 
 7.99e-01
  
 
  
 19
 
  35454992
 
  35455389
 
  PDF   STATS   ENSEMBL   UCSC 
 
  398
 
  8
 
 20661
 
 tss
 
 0.54793
 
 0.02517
 
 Not queried
 
  5.1739123
 
 1.55e-04
 
 7.99e-01
  
 
  
 4
 
  74847646
 
  74848016
 
  PDF   STATS   ENSEMBL   UCSC 
 
  371
 
  8
 
 31047
 
 island
 
 0.08150
 
 0.84832
 
 Not queried
 
  5.1229158
 
 1.55e-04
 
 7.99e-01
  
 
  
 2
 
  31361593
 
  31361818
 
  PDF   STATS   ENSEMBL   UCSC 
 
  226
 
  8
 
 23249
 
 tss;island
 
 0.66135
 
 0.07143
 
 Not queried
 
  5.1010195
 
 1.55e-04
 
 7.99e-01
  
 
  
 8
 
  27182871
 
  27183342
 
  PDF   STATS   ENSEMBL   UCSC 
 
  472
 
  8
 
 39192
 
 gene;island
 
 0.03322
 
 0.57475
 
 Not queried
 
  5.0625462
 
 1.55e-04
 
 7.99e-01
  
 
  
 3
 
 157261004
 
 157261273
 
  PDF   STATS   ENSEMBL   UCSC 
 
  270
 
  8
 
 29571
 
 island
 
 0.06279
 
 0.71330
 
 Not queried
 
  5.0617045
 
 1.55e-04
 
 7.99e-01
  
 
  
 3
 
  42306385
 
  42306974
 
  PDF   STATS   ENSEMBL   UCSC 
 
  590
 
  8
 
 28969
 
 island
 
 0.06999
 
 0.75632
 
 Not queried
 
  4.9950108
 
 1.55e-04
 
 7.99e-01
  
 
  
 1
 
 156163755
 
 156164205
 
  PDF   STATS   ENSEMBL   UCSC 
 
  451
 
  8
 
 814
 
 gene
 
 0.02517
 
 0.38752
 
 Not queried
 
  4.9895609
 
 1.55e-04
 
 7.99e-01
  
 
  
 16
 
   1030383
 
   1030619
 
  PDF   STATS   ENSEMBL   UCSC 
 
  237
 
  8
 
 14955
 
 tss;island
 
 0.63301
 
 0.04742
 
 Not queried
 
  4.9363179
 
 1.55e-04
 
 7.99e-01
  
 
  
 8
 
  48172283
 
  48172992
 
  PDF   STATS   ENSEMBL   UCSC 
 
  710
 
  8
 
 39624
 
 tss;island
 
 0.38817
 
 0.02241
 
 Not queried
 
  4.9112399
 
 1.55e-04
 
 7.99e-01
  
 
  
 12
 
   2800055
 
   2800500
 
  PDF   STATS   ENSEMBL   UCSC 
 
  446
 
  8
 
 8982
 
 gene;island
 
 0.49089
 
 0.04000
 
 Not queried
 
  4.8970596
 
 1.55e-04
 
 7.99e-01
  
 
  
 7
 
  27187372
 
  27187691
 
  PDF   STATS   ENSEMBL   UCSC 
 
  320
 
  8
 
 37428
 
 tss
 
 0.03858
 
 0.59822
 
 Not queried
 
  4.8868398
 
 1.55e-04
 
 7.99e-01
  
 
  
 5
 
 172661803
 
 172662463
 
  PDF   STATS   ENSEMBL   UCSC 
 
  661
 
  8
 
 33343
 
 island
 
 0.12725
 
 0.87347
 
 Not queried
 
  4.8331736
 
 1.55e-04
 
 7.99e-01
  
 
  
 8
 
 104153592
 
 104153829
 
  PDF   STATS   ENSEMBL   UCSC 
 
  238
 
  8
 
 39326
 
 gene;tss;island
 
 0.55128
 
 0.06016
 
 Not queried
 
  4.6971438
 
 1.55e-04
 
 7.99e-01
  
 
  
 1
 
 233086060
 
 233086346
 
  PDF   STATS   ENSEMBL   UCSC 
 
  287
 
  8
 
 2321
 
 tss
 
 0.39822
 
 0.02603
 
 Not queried
 
  4.5284826
 
 1.55e-04
 
 7.99e-01
  
 
  
 6
 
 122931154
 
 122931308
 
  PDF   STATS   ENSEMBL   UCSC 
 
  155
 
  8
 
 35142
 
 tss
 
 0.05218
 
 0.57989
 
 Not queried
 
  4.5177587
 
 1.55e-04
 
 7.99e-01
  
 
  
 3
 
  49314595
 
  49314920
 
  PDF   STATS   ENSEMBL   UCSC 
 
  326
 
  8
 
 27737
 
 gene
 
 0.51334
 
 0.96028
 
 Not queried
 
  4.4957551
 
 1.55e-04
 
 7.99e-01
  
 
  
 6
 
 101328941
 
 101329504
 
  PDF   STATS   ENSEMBL   UCSC 
 
  564
 
  8
 
 36307
 
 island
 
 0.53440
 
 0.04743
 
 Not queried
 
  4.4087518
 
 1.55e-04
 
 7.99e-01
  
 
  
 14
 
  52734156
 
  52734529
 
  PDF   STATS   ENSEMBL   UCSC 
 
  374
 
  8
 
 12814
 
 island
 
 0.32329
 
 0.90351
 
 Not queried
 
  4.4016684
 
 1.55e-04
 
 7.99e-01
  
 
  
 10
 
  43818243
 
  43818911
 
  PDF   STATS   ENSEMBL   UCSC 
 
  669
 
  8
 
 5675
 
 island
 
 0.94531
 
 0.37936
 
 Not queried
 
  4.3307382
 
 1.55e-04
 
 7.99e-01
  
 
  
 5
 
  80597143
 
  80597395
 
  PDF   STATS   ENSEMBL   UCSC 
 
  253
 
  8
 
 32095
 
 tss
 
 0.44845
 
 0.03230
 
 Not queried
 
  4.3270840
 
 1.55e-04
 
 7.99e-01
  
 
  
 4
 
  41753439
 
  41754125
 
  PDF   STATS   ENSEMBL   UCSC 
 
  687
 
  8
 
 30953
 
 island
 
 0.63366
 
 0.10676
 
 Not queried
 
  4.3182747
 
 1.55e-04
 
 7.99e-01
  
 
  
 5
 
 140613740
 
 140614106
 
  PDF   STATS   ENSEMBL   UCSC 
 
  367
 
  8
 
 33164
 
 island
 
 0.02224
 
 0.32781
 
 Not queried
 
  4.3015901
 
 1.55e-04
 
 7.99e-01
  
 
  
 5
 
 178368071
 
 178368415
 
  PDF   STATS   ENSEMBL   UCSC 
 
  345
 
  8
 
 33412
 
 island
 
 0.39418
 
 0.02452
 
 Not queried
 
  4.1413564
 
 1.55e-04
 
 7.99e-01
  
 
  
 1
 
 156182601
 
 156183160
 
  PDF   STATS   ENSEMBL   UCSC 
 
  560
 
  8
 
 3836
 
 island
 
 0.44794
 
 0.03817
 
 Not queried
 
  4.1233894
 
 1.55e-04
 
 7.99e-01
  
 
  
 4
 
     53010
 
     53270
 
  PDF   STATS   ENSEMBL   UCSC 
 
  261
 
  8
 
 30174
 
 tss
 
 0.03290
 
 0.40757
 
 Not queried
 
  4.1186395
 
 1.55e-04
 
 7.99e-01
  
 
  
 1
 
 201368738
 
 201369031
 
  PDF   STATS   ENSEMBL   UCSC 
 
  294
 
  8
 
 2162
 
 tss
 
 0.02285
 
 0.29455
 
 Not queried
 
  4.0950971
 
 1.55e-04
 
 7.99e-01
  
 
  
 4
 
   1166755
 
   1166990
 
  PDF   STATS   ENSEMBL   UCSC 
 
  236
 
  8
 
 29814
 
 gene;tss;island
 
 0.28190
 
 0.86806
 
 Not queried
 
  3.9792124
 
 1.55e-04
 
 7.99e-01
  
 
  
 X
 
  49126087
 
  49126243
 
  PDF   STATS   ENSEMBL   UCSC 
 
  157
 
  8
 
 41316
 
 tss;island
 
 0.20982
 
 0.75873
 
 Not queried
 
  3.9610803
 
 1.55e-04
 
 7.99e-01
  
 
  
 17
 
  76128481
 
  76128683
 
  PDF   STATS   ENSEMBL   UCSC 
 
  203
 
  8
 
 16779
 
 gene;island
 
 0.12202
 
 0.70566
 
 Not queried
 
  3.9555157
 
 1.55e-04
 
 7.99e-01
  
 
  
 12
 
 114846162
 
 114846503
 
  PDF   STATS   ENSEMBL   UCSC 
 
  342
 
  8
 
 10813
 
 island
 
 0.93782
 
 0.45921
 
 Not queried
 
  3.9310971
 
 1.55e-04
 
 7.99e-01
  
 
  
 X
 
  53461386
 
  53461709
 
  PDF   STATS   ENSEMBL   UCSC 
 
  324
 
  8
 
 41331
 
 tss
 
 0.04037
 
 0.37159
 
 Not queried
 
  3.9117740
 
 1.55e-04
 
 7.99e-01
  
 
  
 17
 
  80797881
 
  80798078
 
  PDF   STATS   ENSEMBL   UCSC 
 
  198
 
  8
 
 16926
 
 gene
 
 0.39354
 
 0.91585
 
 Not queried
 
  3.9000979
 
 1.55e-04
 
 7.99e-01
  
 
  
 2
 
  21022431
 
  21023042
 
  PDF   STATS   ENSEMBL   UCSC 
 
  612
 
  8
 
 24079
 
 island
 
 0.92281
 
 0.49336
 
 Not queried
 
  3.8696645
 
 1.55e-04
 
 7.99e-01
  
 
  
 X
 
 135578793
 
 135579224
 
  PDF   STATS   ENSEMBL   UCSC 
 
  432
 
  8
 
 41549
 
 tss;island
 
 0.04097
 
 0.52580
 
 Not queried
 
  3.8506196
 
 1.55e-04
 
 7.99e-01
  
 
  
 17
 
  73583839
 
  73584111
 
  PDF   STATS   ENSEMBL   UCSC 
 
  273
 
  8
 
 17623
 
 tss;island
 
 0.52697
 
 0.09306
 
 Not queried
 
  3.8104114
 
 1.55e-04
 
 7.99e-01
  
 
  
 X
 
  82763219
 
  82763706
 
  PDF   STATS   ENSEMBL   UCSC 
 
  488
 
  8
 
 41892
 
 island
 
 0.36379
 
 0.90835
 
 Not queried
 
  3.8057632
 
 1.55e-04
 
 7.99e-01
  
 
  
 1
 
  84326462
 
  84326856
 
  PDF   STATS   ENSEMBL   UCSC 
 
  395
 
  8
 
 3399
 
 island
 
 0.03716
 
 0.35114
 
 Not queried
 
  3.7926831
 
 1.55e-04
 
 7.99e-01
  
 
  
 3
 
  49395574
 
  49395991
 
  PDF   STATS   ENSEMBL   UCSC 
 
  418
 
  8
 
 29073
 
 island
 
 0.86272
 
 0.34741
 
 Not queried
 
  3.7781457
 
 1.55e-04
 
 7.99e-01
  
 
  
 22
 
  19743759
 
  19744239
 
  PDF   STATS   ENSEMBL   UCSC 
 
  481
 
  8
 
 27262
 
 island
 
 0.24666
 
 0.77682
 
 Not queried
 
  3.7660454
 
 1.55e-04
 
 7.99e-01
  
 
  
 3
 
  48632625
 
  48633144
 
  PDF   STATS   ENSEMBL   UCSC 
 
  520
 
  8
 
 28259
 
 tss
 
 0.31949
 
 0.82358
 
 Not queried
 
  3.5148986
 
 1.55e-04
 
 7.99e-01
  
 
  
 11
 
  93583509
 
  93583973
 
  PDF   STATS   ENSEMBL   UCSC 
 
  465
 
  8
 
 8709
 
 island
 
 0.31969
 
 0.85026
 
 Not queried
 
  3.4801934
 
 1.55e-04
 
 7.99e-01
  
 
  
 11
 
   6341717
 
   6341908
 
  PDF   STATS   ENSEMBL   UCSC 
 
  192
 
  8
 
 7992
 
 island
 
 0.30693
 
 0.04727
 
 Not queried
 
  3.4598732
 
 1.55e-04
 
 7.99e-01
  
 
  
 6
 
 106433792
 
 106434623
 
  PDF   STATS   ENSEMBL   UCSC 
 
  832
 
  8
 
 36316
 
 island
 
 0.50244
 
 0.87459
 
 Not queried
 
  3.3986333
 
 1.55e-04
 
 7.99e-01
  
 
  
 22
 
  50585197
 
  50585427
 
  PDF   STATS   ENSEMBL   UCSC 
 
  231
 
  8
 
 26883
 
 gene;tss
 
 0.13325
 
 0.63293
 
 Not queried
 
  3.2439782
 
 1.55e-04
 
 7.99e-01
  
 
  
 X
 
 153637603
 
 153637827
 
  PDF   STATS   ENSEMBL   UCSC 
 
  225
 
  8
 
 41597
 
 tss
 
 0.29747
 
 0.04919
 
 Not queried
 
  3.2079357
 
 1.55e-04
 
 7.99e-01
  
 
  
 12
 
   9217390
 
   9217769
 
  PDF   STATS   ENSEMBL   UCSC 
 
  380
 
  8
 
 9531
 
 tss
 
 0.50364
 
 0.90017
 
 Not queried
 
  3.2026398
 
 1.55e-04
 
 7.99e-01
  
 
  
 2
 
 223164459
 
 223164925
 
  PDF   STATS   ENSEMBL   UCSC 
 
  467
 
  8
 
 23012
 
 gene;tss;island
 
 0.33603
 
 0.04084
 
 Not queried
 
  3.1948185
 
 1.55e-04
 
 7.99e-01
  
 
  
 3
 
 196366191
 
 196366669
 
  PDF   STATS   ENSEMBL   UCSC 
 
  479
 
  8
 
 29766
 
 island
 
 0.29483
 
 0.77169
 
 Not queried
 
  3.1823767
 
 1.55e-04
 
 7.99e-01
  
 
  
 19
 
  10736006
 
  10736448
 
  PDF   STATS   ENSEMBL   UCSC 
 
  443
 
  8
 
 19782
 
 gene;island
 
 0.03415
 
 0.20919
 
 Not queried
 
  3.1547072
 
 1.55e-04
 
 7.99e-01
  
 
  
 12
 
 114841536
 
 114842031
 
  PDF   STATS   ENSEMBL   UCSC 
 
  496
 
  8
 
 9299
 
 gene;island
 
 0.94251
 
 0.52838
 
 Not queried
 
  3.0498183
 
 1.55e-04
 
 7.99e-01
  
 
  
 5
 
 140562562
 
 140563400
 
  PDF   STATS   ENSEMBL   UCSC 
 
  839
 
  8
 
 31756
 
 gene;island
 
 0.52252
 
 0.87881
 
 Not queried
 
  3.0380489
 
 1.55e-04
 
 7.99e-01
  
 
  
 2
 
  26785120
 
  26785460
 
  PDF   STATS   ENSEMBL   UCSC 
 
  341
 
  8
 
 23206
 
 tss
 
 0.48658
 
 0.86936
 
 Not queried
 
  3.0232917
 
 1.55e-04
 
 7.99e-01
  
 
  
 17
 
  72889708
 
  72890001
 
  PDF   STATS   ENSEMBL   UCSC 
 
  294
 
  8
 
 17601
 
 tss
 
 0.07101
 
 0.32841
 
 Not queried
 
  2.9744288
 
 1.55e-04
 
 7.99e-01
  
 
  
 2
 
  74875227
 
  74875548
 
  PDF   STATS   ENSEMBL   UCSC 
 
  322
 
  8
 
 23427
 
 tss;island
 
 0.34925
 
 0.78496
 
 Not queried
 
  2.8820843
 
 1.55e-04
 
 7.99e-01
  
 
  
 6
 
  29555799
 
  29556415
 
  PDF   STATS   ENSEMBL   UCSC 
 
  617
 
  8
 
 33599
 
 gene
 
 0.34332
 
 0.70904
 
 Not queried
 
  2.5712695
 
 1.55e-04
 
 7.99e-01
  
 
  
 13
 
 113776873
 
 113777109
 
  PDF   STATS   ENSEMBL   UCSC 
 
  237
 
  8
 
 11520
 
 tss
 
 0.39161
 
 0.75438
 
 Not queried
 
  2.4532979
 
 1.55e-04
 
 7.99e-01
  
 
  
 7
 
 120968877
 
 120969174
 
  PDF   STATS   ENSEMBL   UCSC 
 
  298
 
  8
 
 37140
 
 gene;island
 
 0.13917
 
 0.43901
 
 Not queried
 
  2.4371662
 
 1.55e-04
 
 7.99e-01
  
 
  
 11
 
   1036677
 
   1036938
 
  PDF   STATS   ENSEMBL   UCSC 
 
  262
 
  8
 
 7876
 
 island
 
 0.65043
 
 0.85649
 
 Not queried
 
  1.8689487
 
 1.55e-04
 
 7.99e-01
  
 
  
 10
 
 102893925
 
 102894639
 
  PDF   STATS   ENSEMBL   UCSC 
 
  715
 
  8
 
 4710
 
 gene;island
 
 0.55757
 
 0.79745
 
 Not queried
 
  1.6287468
 
 1.55e-04
 
 7.99e-01
  
 
  
 4
 
  41882564
 
  41883172
 
  PDF   STATS   ENSEMBL   UCSC 
 
  609
 
  9
 
 30958
 
 island
 
 0.94065
 
 0.28028
 
 Not queried
 
  5.8884909
 
 1.65e-04
 
 8.46e-01
  
 
  
 17
 
  46608277
 
  46608570
 
  PDF   STATS   ENSEMBL   UCSC 
 
  294
 
  9
 
 17423
 
 tss
 
 0.83132
 
 0.08350
 
 Not queried
 
  4.9770178
 
 1.65e-04
 
 8.46e-01
  
 
  
 2
 
 220378996
 
 220379260
 
  PDF   STATS   ENSEMBL   UCSC 
 
  265
 
  9
 
 22999
 
 gene;island
 
 0.19029
 
 0.68151
 
 Not queried
 
  3.9530557
 
 1.65e-04
 
 8.46e-01
  
 
  
 22
 
  40766469
 
  40766683
 
  PDF   STATS   ENSEMBL   UCSC 
 
  215
 
  9
 
 27469
 
 island
 
 0.02881
 
 0.14278
 
 Not queried
 
  2.8481885
 
 1.65e-04
 
 8.46e-01
  
 
  
 6
 
 151646540
 
 151647133
 
  PDF   STATS   ENSEMBL   UCSC 
 
  594
 
  9
 
 34444
 
 gene;island
 
 0.24827
 
 0.03246
 
 Not queried
 
  2.4964219
 
 1.65e-04
 
 8.46e-01
  
 
  
 18
 
    904851
 
    905611
 
  PDF   STATS   ENSEMBL   UCSC 
 
  761
 
 15
 
 19317
 
 island
 
 0.06583
 
 0.31619
 
 Not queried
 
  2.5140707
 
 1.74e-04
 
 8.95e-01
  
 
  
 6
 
  33084420
 
  33085063
 
  PDF   STATS   ENSEMBL   UCSC 
 
  644
 
 20
 
 34123
 
 gene;island
 
 0.20694
 
 0.42774
 
 Not queried
 
  1.8178165
 
 1.77e-04
 
 9.07e-01
  
 
  
 6
 
  26273029
 
  26273611
 
  PDF   STATS   ENSEMBL   UCSC 
 
  583
 
 11
 
 35471
 
 island
 
 0.57117
 
 0.93176
 
 Not queried
 
  3.1363836
 
 1.90e-04
 
 9.76e-01
  
 
  
 6
 
  27840105
 
  27840257
 
  PDF   STATS   ENSEMBL   UCSC 
 
  153
 
  6
 
 34663
 
 tss
 
 0.01102
 
 0.96427
 
 Not queried
 
 11.7968560
 
 2.16e-03
 
 1.00e+00
  
 
  
 12
 
  75785089
 
  75785295
 
  PDF   STATS   ENSEMBL   UCSC 
 
  207
 
  5
 
 9197
 
 gene;island
 
 0.01220
 
 0.96990
 
 Not queried
 
 11.3890386
 
 7.94e-03
 
 1.00e+00
  
 
  
 15
 
  89089914
 
  89090195
 
  PDF   STATS   ENSEMBL   UCSC 
 
  282
 
  4
 
 13852
 
 tss
 
 0.98129
 
 0.04995
 
 Not queried
 
 10.6623904
 
 2.86e-02
 
 1.00e+00
  
 
  
 19
 
    291239
 
    291315
 
  PDF   STATS   ENSEMBL   UCSC 
 
   77
 
  5
 
 19587
 
 gene;tss;island
 
 0.02355
 
 0.97214
 
 Not queried
 
 10.5903980
 
 7.94e-03
 
 1.00e+00
  
 
  
 1
 
 150947832
 
 150948030
 
  PDF   STATS   ENSEMBL   UCSC 
 
  199
 
  5
 
 1912
 
 tss;island
 
 0.96070
 
 0.02463
 
 Not queried
 
 10.4973414
 
 7.94e-03
 
 1.00e+00
  
 
  
 1
 
 161008811
 
 161008977
 
  PDF   STATS   ENSEMBL   UCSC 
 
  167
 
  4
 
 2038
 
 tss
 
 0.02429
 
 0.96836
 
 Not queried
 
 10.4762993
 
 2.86e-02
 
 1.00e+00
  
 
  
 16
 
   3414983
 
   3415311
 
  PDF   STATS   ENSEMBL   UCSC 
 
  329
 
  5
 
 15616
 
 island
 
 0.00936
 
 0.91281
 
 Not queried
 
 10.4153455
 
 7.94e-03
 
 1.00e+00
  
 
  
 X
 
 133941107
 
 133941194
 
  PDF   STATS   ENSEMBL   UCSC 
 
   88
 
  7
 
 41533
 
 tss
 
 0.92463
 
 0.01010
 
 Not queried
 
 10.3942148
 
 5.83e-04
 
 1.00e+00
  
 
  
 2
 
  63283806
 
  63284132
 
  PDF   STATS   ENSEMBL   UCSC 
 
  327
 
  5
 
 22668
 
 gene;island
 
 0.97646
 
 0.03421
 
 Not queried
 
 10.3617411
 
 7.94e-03
 
 1.00e+00
  
 
  
 12
 
  75784855
 
  75784884
 
  PDF   STATS   ENSEMBL   UCSC 
 
   30
 
  4
 
 9856
 
 tss;island
 
 0.01659
 
 0.95414
 
 Not queried
 
  9.9548061
 
 2.86e-02
 
 1.00e+00
  
 
  
 7
 
 154684327
 
 154684562
 
  PDF   STATS   ENSEMBL   UCSC 
 
  236
 
  4
 
 37241
 
 gene;island
 
 0.02386
 
 0.95080
 
 Not queried
 
  9.6606317
 
 2.86e-02
 
 1.00e+00
  
 
  
 6
 
  24358236
 
  24358327
 
  PDF   STATS   ENSEMBL   UCSC 
 
   92
 
  5
 
 33526
 
 gene;island
 
 0.01419
 
 0.93329
 
 Not queried
 
  9.6288656
 
 7.94e-03
 
 1.00e+00
  
 
  
 1
 
 161008127
 
 161008750
 
  PDF   STATS   ENSEMBL   UCSC 
 
  624
 
  7
 
 844
 
 gene
 
 0.03121
 
 0.91634
 
 Not queried
 
  9.5485994
 
 5.83e-04
 
 1.00e+00
  
 
  
 16
 
  29796620
 
  29796798
 
  PDF   STATS   ENSEMBL   UCSC 
 
  179
 
  4
 
 15773
 
 island
 
 0.96569
 
 0.04814
 
 Not queried
 
  9.4311750
 
 2.86e-02
 
 1.00e+00
  
 
  
 5
 
 172662315
 
 172662463
 
  PDF   STATS   ENSEMBL   UCSC 
 
  149
 
  4
 
 32442
 
 tss
 
 0.02210
 
 0.89061
 
 Not queried
 
  9.3993595
 
 2.86e-02
 
 1.00e+00
  
 
  
 1
 
 165737775
 
 165738087
 
  PDF   STATS   ENSEMBL   UCSC 
 
  313
 
  6
 
 864
 
 gene
 
 0.77665
 
 0.01158
 
 Not queried
 
  9.3248973
 
 2.16e-03
 
 1.00e+00
  
 
  
 1
 
 162337134
 
 162337375
 
  PDF   STATS   ENSEMBL   UCSC 
 
  242
 
  4
 
 857
 
 gene;island
 
 0.03948
 
 0.95308
 
 Not queried
 
  9.3216364
 
 2.86e-02
 
 1.00e+00
  
 
  
 4
 
  85414016
 
  85414194
 
  PDF   STATS   ENSEMBL   UCSC 
 
  179
 
  4
 
 31106
 
 island
 
 0.03414
 
 0.93475
 
 Not queried
 
  9.3018367
 
 2.86e-02
 
 1.00e+00
  
 
  
 7
 
 149389669
 
 149389941
 
  PDF   STATS   ENSEMBL   UCSC 
 
  273
 
  4
 
 38937
 
 island
 
 0.96657
 
 0.04933
 
 Not queried
 
  9.2850891
 
 2.86e-02
 
 1.00e+00
  
 
  
 X
 
  20134778
 
  20134908
 
  PDF   STATS   ENSEMBL   UCSC 
 
  131
 
  4
 
 41217
 
 tss
 
 0.01745
 
 0.91853
 
 Not queried
 
  9.1731208
 
 2.86e-02
 
 1.00e+00
  
 
  
 8
 
   6692785
 
   6693125
 
  PDF   STATS   ENSEMBL   UCSC 
 
  341
 
  4
 
 39128
 
 gene
 
 0.02536
 
 0.87165
 
 Not queried
 
  9.1433734
 
 2.86e-02
 
 1.00e+00
  
 
  
 19
 
  21646329
 
  21646781
 
  PDF   STATS   ENSEMBL   UCSC 
 
  453
 
  5
 
 21799
 
 island
 
 0.79716
 
 0.01123
 
 Not queried
 
  9.0433068
 
 7.94e-03
 
 1.00e+00
  
 
  
 19
 
  50979561
 
  50979637
 
  PDF   STATS   ENSEMBL   UCSC 
 
   77
 
  4
 
 20943
 
 tss
 
 0.02362
 
 0.85525
 
 Not queried
 
  9.0428764
 
 2.86e-02
 
 1.00e+00
  
 
  
 4
 
 187985253
 
 187985472
 
  PDF   STATS   ENSEMBL   UCSC 
 
  220
 
  4
 
 31435
 
 island
 
 0.03285
 
 0.91228
 
 Not queried
 
  9.0419551
 
 2.86e-02
 
 1.00e+00
  
 
  
 3
 
  39424697
 
  39424992
 
  PDF   STATS   ENSEMBL   UCSC 
 
  296
 
  6
 
 28954
 
 island
 
 0.01773
 
 0.95343
 
 Not queried
 
  8.9917225
 
 2.16e-03
 
 1.00e+00
  
 
  
 2
 
 139537745
 
 139537845
 
  PDF   STATS   ENSEMBL   UCSC 
 
  101
 
  4
 
 24726
 
 island
 
 0.01400
 
 0.87401
 
 Not queried
 
  8.9784978
 
 2.86e-02
 
 1.00e+00
  
 
  
 3
 
  39425775
 
  39426101
 
  PDF   STATS   ENSEMBL   UCSC 
 
  327
 
  4
 
 27683
 
 gene;island
 
 0.05556
 
 0.96319
 
 Not queried
 
  8.9202931
 
 2.86e-02
 
 1.00e+00
  
 
  
 5
 
   8457548
 
   8458089
 
  PDF   STATS   ENSEMBL   UCSC 
 
  542
 
  6
 
 32622
 
 island
 
 0.02126
 
 0.91076
 
 Not queried
 
  8.9006468
 
 2.16e-03
 
 1.00e+00
  
 
  
 6
 
  27833121
 
  27833555
 
  PDF   STATS   ENSEMBL   UCSC 
 
  435
 
  5
 
 33558
 
 gene
 
 0.02246
 
 0.91631
 
 Not queried
 
  8.8965867
 
 7.94e-03
 
 1.00e+00
  
 
  
 8
 
  37605552
 
  37605978
 
  PDF   STATS   ENSEMBL   UCSC 
 
  427
 
  5
 
 39582
 
 tss
 
 0.03565
 
 0.94799
 
 Not queried
 
  8.8936134
 
 7.94e-03
 
 1.00e+00
  
 
  
 3
 
 186857284
 
 186857593
 
  PDF   STATS   ENSEMBL   UCSC 
 
  310
 
  7
 
 28735
 
 tss;island
 
 0.01799
 
 0.90171
 
 Not queried
 
  8.8914387
 
 5.83e-04
 
 1.00e+00
  
 
  
 10
 
  50887472
 
  50887632
 
  PDF   STATS   ENSEMBL   UCSC 
 
  161
 
  7
 
 5059
 
 tss
 
 0.01532
 
 0.85417
 
 Not queried
 
  8.8434672
 
 5.83e-04
 
 1.00e+00
  
 
  
 19
 
  11530021
 
  11530065
 
  PDF   STATS   ENSEMBL   UCSC 
 
   45
 
  4
 
 20435
 
 tss
 
 0.94156
 
 0.04473
 
 Not queried
 
  8.8212514
 
 2.86e-02
 
 1.00e+00
  
 
  
 8
 
  80803196
 
  80803526
 
  PDF   STATS   ENSEMBL   UCSC 
 
  331
 
  5
 
 40345
 
 island
 
 0.02697
 
 0.91067
 
 Not queried
 
  8.7779967
 
 7.94e-03
 
 1.00e+00
  
 
  
 15
 
  78556834
 
  78557094
 
  PDF   STATS   ENSEMBL   UCSC 
 
  261
 
  6
 
 13408
 
 gene;tss;island
 
 0.04285
 
 0.93962
 
 Not queried
 
  8.7670148
 
 2.16e-03
 
 1.00e+00
  
 
  
 12
 
   7781004
 
   7781431
 
  PDF   STATS   ENSEMBL   UCSC 
 
  428
 
  5
 
 10201
 
 island
 
 0.03621
 
 0.93350
 
 Not queried
 
  8.7330377
 
 1.51e-01
 
 1.00e+00
  
 
  
 5
 
 179740743
 
 179741120
 
  PDF   STATS   ENSEMBL   UCSC 
 
  378
 
  4
 
 31883
 
 gene;island
 
 0.01214
 
 0.83445
 
 Not queried
 
  8.7241414
 
 2.86e-02
 
 1.00e+00
  
 
  
 3
 
 109035381
 
 109035781
 
  PDF   STATS   ENSEMBL   UCSC 
 
  401
 
  6
 
 28450
 
 tss
 
 0.01786
 
 0.86308
 
 Not queried
 
  8.6890221
 
 2.16e-03
 
 1.00e+00
  
 
  
 19
 
  50861774
 
  50862121
 
  PDF   STATS   ENSEMBL   UCSC 
 
  348
 
  5
 
 20137
 
 gene;island
 
 0.03010
 
 0.85631
 
 Not queried
 
  8.6881577
 
 7.94e-03
 
 1.00e+00
  
 
  
 6
 
  10390811
 
  10391412
 
  PDF   STATS   ENSEMBL   UCSC 
 
  602
 
  6
 
 35360
 
 island
 
 0.93433
 
 0.03855
 
 Not queried
 
  8.6849213
 
 2.16e-03
 
 1.00e+00
  
 
  
 11
 
  15095017
 
  15095178
 
  PDF   STATS   ENSEMBL   UCSC 
 
  162
 
  6
 
 8061
 
 island
 
 0.02096
 
 0.86387
 
 Not queried
 
  8.6198653
 
 2.16e-03
 
 1.00e+00
  
 
  
 X
 
 113815930
 
 113816304
 
  PDF   STATS   ENSEMBL   UCSC 
 
  375
 
  5
 
 41966
 
 island
 
 0.03224
 
 0.92793
 
 Not queried
 
  8.6145594
 
 7.94e-03
 
 1.00e+00
  
 
  
 1
 
 179560700
 
 179561111
 
  PDF   STATS   ENSEMBL   UCSC 
 
  412
 
  6
 
 3988
 
 island
 
 0.01445
 
 0.86061
 
 Not queried
 
  8.5761559
 
 2.16e-03
 
 1.00e+00
  
 
  
 18
 
  21719263
 
  21719433
 
  PDF   STATS   ENSEMBL   UCSC 
 
  171
 
  5
 
 19177
 
 tss
 
 0.06129
 
 0.93350
 
 Not queried
 
  8.5714586
 
 7.94e-03
 
 1.00e+00
  
 
  
 6
 
  27835363
 
  27835460
 
  PDF   STATS   ENSEMBL   UCSC 
 
   98
 
  6
 
 34662
 
 tss
 
 0.03731
 
 0.92585
 
 Not queried
 
  8.5504481
 
 2.16e-03
 
 1.00e+00
  
 
  
 6
 
  24358304
 
  24358327
 
  PDF   STATS   ENSEMBL   UCSC 
 
   24
 
  4
 
 34602
 
 tss
 
 0.02476
 
 0.92632
 
 Not queried
 
  8.5502862
 
 2.86e-02
 
 1.00e+00
  
 
  
 16
 
  82044738
 
  82045078
 
  PDF   STATS   ENSEMBL   UCSC 
 
  341
 
  5
 
 14826
 
 gene
 
 0.03669
 
 0.91932
 
 Not queried
 
  8.5388602
 
 7.94e-03
 
 1.00e+00
  
 
  
 13
 
 113420153
 
 113420371
 
  PDF   STATS   ENSEMBL   UCSC 
 
  219
 
  4
 
 11222
 
 gene;island
 
 0.03990
 
 0.94757
 
 Not queried
 
  8.5295232
 
 2.86e-02
 
 1.00e+00
  
 
  
 2
 
 234776807
 
 234777085
 
  PDF   STATS   ENSEMBL   UCSC 
 
  279
 
  5
 
 25174
 
 island
 
 0.01852
 
 0.87342
 
 Not queried
 
  8.5148040
 
 7.94e-03
 
 1.00e+00
  
 
  
 1
 
 234367145
 
 234367586
 
  PDF   STATS   ENSEMBL   UCSC 
 
  442
 
  5
 
 1082
 
 gene;island
 
 0.01665
 
 0.92147
 
 Not queried
 
  8.4849516
 
 7.94e-03
 
 1.00e+00
  
 
  
 6
 
  27835191
 
  27835460
 
  PDF   STATS   ENSEMBL   UCSC 
 
  270
 
  7
 
 35508
 
 island
 
 0.03571
 
 0.92826
 
 Not queried
 
  8.4842641
 
 5.83e-04
 
 1.00e+00
  
 
  
 5
 
 176559881
 
 176560265
 
  PDF   STATS   ENSEMBL   UCSC 
 
  385
 
  4
 
 32462
 
 tss;island
 
 0.01656
 
 0.84381
 
 Not queried
 
  8.4308908
 
 2.86e-02
 
 1.00e+00
  
 
  
 1
 
 150254280
 
 150254546
 
  PDF   STATS   ENSEMBL   UCSC 
 
  267
 
  5
 
 1901
 
 tss;island
 
 0.04870
 
 0.94628
 
 Not queried
 
  8.4267120
 
 7.94e-03
 
 1.00e+00
  
 
  
 7
 
 111368367
 
 111368847
 
  PDF   STATS   ENSEMBL   UCSC 
 
  481
 
  4
 
 37125
 
 gene;island
 
 0.04517
 
 0.91340
 
 Not queried
 
  8.4034800
 
 2.86e-02
 
 1.00e+00
  
 
  
 15
 
  99409194
 
  99409506
 
  PDF   STATS   ENSEMBL   UCSC 
 
  313
 
  4
 
 13468
 
 gene
 
 0.02855
 
 0.89758
 
 Not queried
 
  8.3899365
 
 2.86e-02
 
 1.00e+00
  
 
  
 5
 
 180258557
 
 180258852
 
  PDF   STATS   ENSEMBL   UCSC 
 
  296
 
  5
 
 31889
 
 gene;island
 
 0.91704
 
 0.03020
 
 Not queried
 
  8.3625549
 
 7.94e-03
 
 1.00e+00
  
 
  
 13
 
 100547192
 
 100547367
 
  PDF   STATS   ENSEMBL   UCSC 
 
  176
 
  5
 
 11179
 
 gene;island
 
 0.04541
 
 0.94878
 
 Not queried
 
  8.3593366
 
 7.94e-03
 
 1.00e+00
  
 
  
 12
 
  64784070
 
  64784252
 
  PDF   STATS   ENSEMBL   UCSC 
 
  183
 
  4
 
 9178
 
 gene;island
 
 0.03331
 
 0.89519
 
 Not queried
 
  8.3225637
 
 2.86e-02
 
 1.00e+00
  
 
  
 1
 
 209979111
 
 209979470
 
  PDF   STATS   ENSEMBL   UCSC 
 
  360
 
  5
 
 985
 
 gene
 
 0.00816
 
 0.82463
 
 Not queried
 
  8.3108587
 
 7.94e-03
 
 1.00e+00
  
 
  
 6
 
  27782484
 
  27782658
 
  PDF   STATS   ENSEMBL   UCSC 
 
  175
 
  5
 
 34656
 
 tss;island
 
 0.00755
 
 0.54945
 
 Not queried
 
  8.2721259
 
 7.94e-03
 
 1.00e+00
  
 
  
 7
 
 121651766
 
 121652024
 
  PDF   STATS   ENSEMBL   UCSC 
 
  259
 
  4
 
 37141
 
 gene
 
 0.05188
 
 0.93791
 
 Not queried
 
  8.2698249
 
 2.86e-02
 
 1.00e+00
  
 
  
 7
 
  23287222
 
  23287560
 
  PDF   STATS   ENSEMBL   UCSC 
 
  339
 
  4
 
 36851
 
 gene;island
 
 0.01997
 
 0.86601
 
 Not queried
 
  8.2688011
 
 2.86e-02
 
 1.00e+00
  
 
  
 8
 
  37605359
 
  37605978
 
  PDF   STATS   ENSEMBL   UCSC 
 
  620
 
  7
 
 39216
 
 gene
 
 0.04379
 
 0.94799
 
 Not queried
 
  8.2556995
 
 5.83e-04
 
 1.00e+00
  
 
  
 17
 
  36997420
 
  36997627
 
  PDF   STATS   ENSEMBL   UCSC 
 
  208
 
  4
 
 16507
 
 gene
 
 0.04557
 
 0.94266
 
 Not queried
 
  8.2485040
 
 2.86e-02
 
 1.00e+00
  
 
  
 18
 
  21719263
 
  21719568
 
  PDF   STATS   ENSEMBL   UCSC 
 
  306
 
  7
 
 19044
 
 gene;island
 
 0.06207
 
 0.91153
 
 Not queried
 
  8.2441441
 
 5.83e-04
 
 1.00e+00
  
 
  
 1
 
 179560700
 
 179560983
 
  PDF   STATS   ENSEMBL   UCSC 
 
  284
 
  5
 
 2115
 
 tss
 
 0.01450
 
 0.85319
 
 Not queried
 
  8.2261068
 
 7.94e-03
 
 1.00e+00
  
 
  
 11
 
   7110074
 
   7110196
 
  PDF   STATS   ENSEMBL   UCSC 
 
  123
 
  5
 
 8004
 
 island
 
 0.02345
 
 0.86500
 
 Not queried
 
  8.2054513
 
 7.94e-03
 
 1.00e+00
  
 
  
 4
 
 187422005
 
 187422119
 
  PDF   STATS   ENSEMBL   UCSC 
 
  115
 
  5
 
 31427
 
 island
 
 0.04050
 
 0.87308
 
 Not queried
 
  8.1982156
 
 7.94e-03
 
 1.00e+00
  
 
  
 12
 
 131303016
 
 131303247
 
  PDF   STATS   ENSEMBL   UCSC 
 
  232
 
  4
 
 9392
 
 gene;island
 
 0.01976
 
 0.85662
 
 Not queried
 
  8.1805821
 
 2.86e-02
 
 1.00e+00
  
 
  
 16
 
  29296614
 
  29296797
 
  PDF   STATS   ENSEMBL   UCSC 
 
  184
 
  4
 
 15769
 
 island
 
 0.01862
 
 0.67507
 
 Not queried
 
  8.1387921
 
 2.86e-02
 
 1.00e+00
  
 
  
 11
 
 130029961
 
 130030459
 
  PDF   STATS   ENSEMBL   UCSC 
 
  499
 
  4
 
 6956
 
 gene;island
 
 0.02321
 
 0.82595
 
 Not queried
 
  8.1386401
 
 2.86e-02
 
 1.00e+00
  
 
  
 2
 
 166650805
 
 166650965
 
  PDF   STATS   ENSEMBL   UCSC 
 
  161
 
  6
 
 23657
 
 tss
 
 0.02316
 
 0.85930
 
 Not queried
 
  8.1181806
 
 2.16e-03
 
 1.00e+00
  
 
  
 2
 
 113342070
 
 113342262
 
  PDF   STATS   ENSEMBL   UCSC 
 
  193
 
  4
 
 22793
 
 gene
 
 0.02042
 
 0.86764
 
 Not queried
 
  8.1168022
 
 2.86e-02
 
 1.00e+00
  
 
  
 1
 
  59042745
 
  59043070
 
  PDF   STATS   ENSEMBL   UCSC 
 
  326
 
  4
 
 556
 
 gene
 
 0.06667
 
 0.90849
 
 Not queried
 
  8.1085431
 
 2.86e-02
 
 1.00e+00
  
 
  
 6
 
  24877462
 
  24877729
 
  PDF   STATS   ENSEMBL   UCSC 
 
  268
 
  4
 
 33530
 
 gene
 
 0.03440
 
 0.91979
 
 Not queried
 
  8.0857580
 
 2.86e-02
 
 1.00e+00
  
 
  
 3
 
 170746029
 
 170746265
 
  PDF   STATS   ENSEMBL   UCSC 
 
  237
 
  5
 
 28674
 
 tss
 
 0.03112
 
 0.88366
 
 Not queried
 
  8.0818909
 
 7.94e-03
 
 1.00e+00
  
 
  
 18
 
   7011217
 
   7011463
 
  PDF   STATS   ENSEMBL   UCSC 
 
  247
 
  4
 
 19018
 
 gene;island
 
 0.09564
 
 0.93804
 
 Not queried
 
  8.0083981
 
 2.86e-02
 
 1.00e+00
  
 
  
 2
 
 225266733
 
 225266880
 
  PDF   STATS   ENSEMBL   UCSC 
 
  148
 
  6
 
 23887
 
 tss
 
 0.01542
 
 0.81557
 
 Not queried
 
  8.0007927
 
 2.16e-03
 
 1.00e+00
  
 
  
 4
 
  48491941
 
  48492304
 
  PDF   STATS   ENSEMBL   UCSC 
 
  364
 
  7
 
 30336
 
 tss
 
 0.07113
 
 0.90283
 
 Not queried
 
  8.0006180
 
 5.83e-04
 
 1.00e+00
  
 
  
 7
 
  97602481
 
  97602715
 
  PDF   STATS   ENSEMBL   UCSC 
 
  235
 
  4
 
 37676
 
 tss;island
 
 0.95573
 
 0.07921
 
 Not queried
 
  7.9948134
 
 2.86e-02
 
 1.00e+00
  
 
  
 4
 
  56023751
 
  56024045
 
  PDF   STATS   ENSEMBL   UCSC 
 
  295
 
  4
 
 31006
 
 island
 
 0.05930
 
 0.92843
 
 Not queried
 
  7.9772747
 
 2.86e-02
 
 1.00e+00
  
 
  
 3
 
   9404422
 
   9404684
 
  PDF   STATS   ENSEMBL   UCSC 
 
  263
 
  7
 
 28082
 
 tss
 
 0.88224
 
 0.03068
 
 Not queried
 
  7.9753462
 
 5.83e-04
 
 1.00e+00
  
 
  
 19
 
   1074727
 
   1075104
 
  PDF   STATS   ENSEMBL   UCSC 
 
  378
 
  4
 
 19606
 
 gene;island
 
 0.02600
 
 0.84611
 
 Not queried
 
  7.9486021
 
 2.86e-02
 
 1.00e+00
  
 
  
 14
 
  61748243
 
  61748440
 
  PDF   STATS   ENSEMBL   UCSC 
 
  198
 
  4
 
 12077
 
 gene;island
 
 0.02814
 
 0.85989
 
 Not queried
 
  7.9480009
 
 2.86e-02
 
 1.00e+00
  
 
  
 15
 
  91473059
 
  91473386
 
  PDF   STATS   ENSEMBL   UCSC 
 
  328
 
  6
 
 13878
 
 tss
 
 0.03576
 
 0.89739
 
 Not queried
 
  7.9332543
 
 2.16e-03
 
 1.00e+00
  
 
  
 20
 
  52199520
 
  52199778
 
  PDF   STATS   ENSEMBL   UCSC 
 
  259
 
  5
 
 26223
 
 island
 
 0.08439
 
 0.90248
 
 Not queried
 
  7.8953826
 
 7.94e-03
 
 1.00e+00
  
 
  
 12
 
  54332992
 
  54333323
 
  PDF   STATS   ENSEMBL   UCSC 
 
  332
 
  4
 
 9129
 
 gene;island
 
 0.01084
 
 0.67248
 
 Not queried
 
  7.8849737
 
 2.86e-02
 
 1.00e+00
  
 
  
 7
 
  48495072
 
  48495333
 
  PDF   STATS   ENSEMBL   UCSC 
 
  262
 
  4
 
 36945
 
 gene;island
 
 0.04772
 
 0.91243
 
 Not queried
 
  7.8563462
 
 2.86e-02
 
 1.00e+00
  
 
  
 20
 
  61051029
 
  61051039
 
  PDF   STATS   ENSEMBL   UCSC 
 
   11
 
  4
 
 25832
 
 tss
 
 0.01815
 
 0.80397
 
 Not queried
 
  7.8562673
 
 2.86e-02
 
 1.00e+00
  
 
  
 6
 
  34724739
 
  34724845
 
  PDF   STATS   ENSEMBL   UCSC 
 
  107
 
  5
 
 34858
 
 tss;island
 
 0.02667
 
 0.89123
 
 Not queried
 
  7.8517066
 
 7.94e-03
 
 1.00e+00
  
 
  
 12
 
  64784483
 
  64784686
 
  PDF   STATS   ENSEMBL   UCSC 
 
  204
 
  5
 
 9816
 
 tss;island
 
 0.04889
 
 0.90648
 
 Not queried
 
  7.8441670
 
 7.94e-03
 
 1.00e+00
  
 
  
 5
 
 179779115
 
 179779237
 
  PDF   STATS   ENSEMBL   UCSC 
 
  123
 
  4
 
 31885
 
 gene;island
 
 0.86507
 
 0.04894
 
 Not queried
 
  7.8425074
 
 2.86e-02
 
 1.00e+00
  
 
  
 19
 
  33210498
 
  33210851
 
  PDF   STATS   ENSEMBL   UCSC 
 
  354
 
  5
 
 21834
 
 island
 
 0.03319
 
 0.89451
 
 Not queried
 
  7.8410635
 
 7.94e-03
 
 1.00e+00
  
 
  
 4
 
    779480
 
    779880
 
  PDF   STATS   ENSEMBL   UCSC 
 
  401
 
  4
 
 29793
 
 gene;island
 
 0.03548
 
 0.89383
 
 Not queried
 
  7.8363620
 
 2.86e-02
 
 1.00e+00
  
 
  
 3
 
 170746029
 
 170746291
 
  PDF   STATS   ENSEMBL   UCSC 
 
  263
 
  6
 
 29617
 
 island
 
 0.03472
 
 0.88057
 
 Not queried
 
  7.8284735
 
 2.16e-03
 
 1.00e+00
  
 
  
 3
 
  87138203
 
  87138700
 
  PDF   STATS   ENSEMBL   UCSC 
 
  498
 
  6
 
 29257
 
 island
 
 0.03926
 
 0.90923
 
 Not queried
 
  7.8176281
 
 2.16e-03
 
 1.00e+00
  
 
  
 11
 
 130029478
 
 130029684
 
  PDF   STATS   ENSEMBL   UCSC 
 
  207
 
  5
 
 8933
 
 island
 
 0.04930
 
 0.92126
 
 Not queried
 
  7.8162870
 
 7.94e-03
 
 1.00e+00
  
 
  
 10
 
  49812686
 
  49813128
 
  PDF   STATS   ENSEMBL   UCSC 
 
  443
 
  4
 
 4567
 
 gene
 
 0.02834
 
 0.83465
 
 Not queried
 
  7.8154983
 
 2.86e-02
 
 1.00e+00
  
 
  
 1
 
 209848845
 
 209849185
 
  PDF   STATS   ENSEMBL   UCSC 
 
  341
 
  5
 
 983
 
 gene;island
 
 0.87628
 
 0.03055
 
 Not queried
 
  7.8123643
 
 7.94e-03
 
 1.00e+00
  
 
  
 13
 
  36871646
 
  36871962
 
  PDF   STATS   ENSEMBL   UCSC 
 
  317
 
  6
 
 11101
 
 gene
 
 0.96347
 
 0.10961
 
 Not queried
 
  7.8122120
 
 2.16e-03
 
 1.00e+00
  
 
  
 1
 
   8013974
 
   8014650
 
  PDF   STATS   ENSEMBL   UCSC 
 
  677
 
  6
 
 2625
 
 island
 
 0.04738
 
 0.87225
 
 Not queried
 
  7.8087341
 
 2.16e-03
 
 1.00e+00
  
 
  
 X
 
   8751190
 
   8751687
 
  PDF   STATS   ENSEMBL   UCSC 
 
  498
 
  7
 
 41624
 
 island
 
 0.02642
 
 0.91226
 
 Not queried
 
  7.8025545
 
 5.83e-04
 
 1.00e+00
  
 
  
 2
 
  73429084
 
  73429371
 
  PDF   STATS   ENSEMBL   UCSC 
 
  288
 
  7
 
 23399
 
 tss
 
 0.94896
 
 0.05955
 
 Not queried
 
  7.7982372
 
 5.83e-04
 
 1.00e+00
  
 
  
 7
 
  48494362
 
  48494734
 
  PDF   STATS   ENSEMBL   UCSC 
 
  373
 
  5
 
 36944
 
 gene;island
 
 0.02817
 
 0.87326
 
 Not queried
 
  7.7590733
 
 7.94e-03
 
 1.00e+00
  
 
  
 5
 
 156696185
 
 156696520
 
  PDF   STATS   ENSEMBL   UCSC 
 
  336
 
  4
 
 31816
 
 gene;island
 
 0.04131
 
 0.83740
 
 Not queried
 
  7.7520108
 
 2.86e-02
 
 1.00e+00
  
 
  
 11
 
 130029478
 
 130029615
 
  PDF   STATS   ENSEMBL   UCSC 
 
  138
 
  4
 
 7795
 
 tss
 
 0.05268
 
 0.92436
 
 Not queried
 
  7.7193905
 
 2.86e-02
 
 1.00e+00
  
 
  
 X
 
 153284899
 
 153285158
 
  PDF   STATS   ENSEMBL   UCSC 
 
  260
 
  4
 
 41154
 
 gene;island
 
 0.01487
 
 0.80619
 
 Not queried
 
  7.7178075
 
 2.86e-02
 
 1.00e+00
  
 
  
 19
 
  12306136
 
  12306497
 
  PDF   STATS   ENSEMBL   UCSC 
 
  362
 
  4
 
 21561
 
 island
 
 0.01954
 
 0.87090
 
 Not queried
 
  7.7022088
 
 2.86e-02
 
 1.00e+00
  
 
  
 19
 
  12075601
 
  12075848
 
  PDF   STATS   ENSEMBL   UCSC 
 
  248
 
  5
 
 20450
 
 tss
 
 0.01073
 
 0.67328
 
 Not queried
 
  7.6877903
 
 7.94e-03
 
 1.00e+00
  
 
  
 X
 
 149716155
 
 149716393
 
  PDF   STATS   ENSEMBL   UCSC 
 
  239
 
  4
 
 42095
 
 island
 
 0.07104
 
 0.92328
 
 Not queried
 
  7.6833156
 
 2.86e-02
 
 1.00e+00
  
 
  
 22
 
  36903287
 
  36903611
 
  PDF   STATS   ENSEMBL   UCSC 
 
  325
 
  6
 
 27067
 
 tss;island
 
 0.02539
 
 0.88235
 
 Not queried
 
  7.6812740
 
 1.52e-02
 
 1.00e+00
  
 
  
 6
 
  26240307
 
  26240579
 
  PDF   STATS   ENSEMBL   UCSC 
 
  273
 
  5
 
 34635
 
 tss;island
 
 0.01173
 
 0.70687
 
 Not queried
 
  7.6671140
 
 7.94e-03
 
 1.00e+00
  
 
  
 17
 
  74533845
 
  74534090
 
  PDF   STATS   ENSEMBL   UCSC 
 
  246
 
  7
 
 17648
 
 tss;island
 
 0.05128
 
 0.87093
 
 Not queried
 
  7.6513685
 
 5.83e-04
 
 1.00e+00
  
 
  
 22
 
  50064736
 
  50065058
 
  PDF   STATS   ENSEMBL   UCSC 
 
  323
 
  4
 
 27563
 
 island
 
 0.04304
 
 0.87097
 
 Not queried
 
  7.6501018
 
 2.86e-02
 
 1.00e+00
  
 
  
 12
 
   7072696
 
   7072891
 
  PDF   STATS   ENSEMBL   UCSC 
 
  196
 
  4
 
 9514
 
 tss
 
 0.07813
 
 0.90443
 
 Not queried
 
  7.6246397
 
 2.86e-02
 
 1.00e+00
  
 
  
 19
 
  21657529
 
  21658001
 
  PDF   STATS   ENSEMBL   UCSC 
 
  473
 
  6
 
 21800
 
 island
 
 0.89143
 
 0.05281
 
 Not queried
 
  7.6165865
 
 2.16e-03
 
 1.00e+00
  
 
  
 22
 
  22901569
 
  22901661
 
  PDF   STATS   ENSEMBL   UCSC 
 
   93
 
  4
 
 26767
 
 gene
 
 0.02123
 
 0.83234
 
 Not queried
 
  7.6135504
 
 2.86e-02
 
 1.00e+00
  
 
  
 8
 
  58055876
 
  58056175
 
  PDF   STATS   ENSEMBL   UCSC 
 
  300
 
  4
 
 40254
 
 island
 
 0.09566
 
 0.92403
 
 Not queried
 
  7.5904028
 
 2.86e-02
 
 1.00e+00
  
 
  
 2
 
 131046122
 
 131046551
 
  PDF   STATS   ENSEMBL   UCSC 
 
  430
 
  4
 
 24680
 
 island
 
 0.03290
 
 0.88335
 
 Not queried
 
  7.5880256
 
 2.86e-02
 
 1.00e+00
  
 
  
 X
 
 153285622
 
 153285934
 
  PDF   STATS   ENSEMBL   UCSC 
 
  313
 
  5
 
 41590
 
 tss;island
 
 0.05775
 
 0.86536
 
 Not queried
 
  7.5828289
 
 7.94e-03
 
 1.00e+00
  
 
  
 19
 
  58520847
 
  58521273
 
  PDF   STATS   ENSEMBL   UCSC 
 
  427
 
  6
 
 22453
 
 island
 
 0.03253
 
 0.88397
 
 Not queried
 
  7.5802632
 
 2.16e-03
 
 1.00e+00
  
 
  
 5
 
   3188066
 
   3188374
 
  PDF   STATS   ENSEMBL   UCSC 
 
  309
 
  5
 
 32601
 
 island
 
 0.05160
 
 0.93482
 
 Not queried
 
  7.5579560
 
 7.94e-03
 
 1.00e+00
  
 
  
 17
 
  43246883
 
  43247290
 
  PDF   STATS   ENSEMBL   UCSC 
 
  408
 
  4
 
 16597
 
 gene;island
 
 0.05336
 
 0.89771
 
 Not queried
 
  7.5566750
 
 2.86e-02
 
 1.00e+00
  
 
  
 6
 
  27258460
 
  27258767
 
  PDF   STATS   ENSEMBL   UCSC 
 
  308
 
  6
 
 35489
 
 island
 
 0.92281
 
 0.03937
 
 Not queried
 
  7.5426942
 
 2.16e-03
 
 1.00e+00
  
 
  
 14
 
 104583632
 
 104584279
 
  PDF   STATS   ENSEMBL   UCSC 
 
  648
 
  6
 
 13173
 
 island
 
 0.05562
 
 0.87820
 
 Not queried
 
  7.5318782
 
 2.16e-03
 
 1.00e+00
  
 
  
 1
 
 236686564
 
 236686804
 
  PDF   STATS   ENSEMBL   UCSC 
 
  241
 
  5
 
 1095
 
 gene;tss;island
 
 0.06307
 
 0.86925
 
 Not queried
 
  7.5231652
 
 7.94e-03
 
 1.00e+00
  
 
  
 2
 
   3260576
 
   3261155
 
  PDF   STATS   ENSEMBL   UCSC 
 
  580
 
  5
 
 22506
 
 gene
 
 0.13322
 
 0.82791
 
 Not queried
 
  7.5222691
 
 7.94e-03
 
 1.00e+00
  
 
  
 6
 
  16306377
 
  16306857
 
  PDF   STATS   ENSEMBL   UCSC 
 
  481
 
  4
 
 33522
 
 gene
 
 0.06106
 
 0.90297
 
 Not queried
 
  7.5149321
 
 2.86e-02
 
 1.00e+00
  
 
  
 6
 
  26021916
 
  26022273
 
  PDF   STATS   ENSEMBL   UCSC 
 
  358
 
  5
 
 33532
 
 gene
 
 0.12071
 
 0.94958
 
 Not queried
 
  7.5056158
 
 7.94e-03
 
 1.00e+00
  
 
  
 6
 
  10419835
 
  10420079
 
  PDF   STATS   ENSEMBL   UCSC 
 
  245
 
  7
 
 34560
 
 tss
 
 0.03384
 
 0.80483
 
 Not queried
 
  7.5014191
 
 5.83e-04
 
 1.00e+00
  
 
  
 14
 
  55907299
 
  55907501
 
  PDF   STATS   ENSEMBL   UCSC 
 
  203
 
  7
 
 12386
 
 tss
 
 0.05232
 
 0.82192
 
 Not queried
 
  7.5000832
 
 5.83e-04
 
 1.00e+00
  
 
  
 14
 
  54430382
 
  54430831
 
  PDF   STATS   ENSEMBL   UCSC 
 
  450
 
  4
 
 12826
 
 island
 
 0.02445
 
 0.87721
 
 Not queried
 
  7.4995059
 
 2.86e-02
 
 1.00e+00
  
 
  
 15
 
  23810163
 
  23810378
 
  PDF   STATS   ENSEMBL   UCSC 
 
  216
 
  5
 
 13486
 
 tss
 
 0.07822
 
 0.93121
 
 Not queried
 
  7.4869514
 
 7.94e-03
 
 1.00e+00
  
 
  
 20
 
  61050885
 
  61051039
 
  PDF   STATS   ENSEMBL   UCSC 
 
  155
 
  7
 
 26280
 
 island
 
 0.02368
 
 0.81008
 
 Not queried
 
  7.4824754
 
 5.83e-04
 
 1.00e+00
  
 
  
 7
 
  27138712
 
  27138974
 
  PDF   STATS   ENSEMBL   UCSC 
 
  263
 
  4
 
 38273
 
 island
 
 0.93916
 
 0.08941
 
 Not queried
 
  7.4603982
 
 2.86e-02
 
 1.00e+00
  
 
  
 6
 
  10416337
 
  10416531
 
  PDF   STATS   ENSEMBL   UCSC 
 
  195
 
  4
 
 33498
 
 gene;tss;island
 
 0.84675
 
 0.03795
 
 Not queried
 
  7.4575734
 
 2.86e-02
 
 1.00e+00
  
 
  
 20
 
  61002595
 
  61002866
 
  PDF   STATS   ENSEMBL   UCSC 
 
  272
 
  6
 
 25831
 
 tss
 
 0.02276
 
 0.80987
 
 Not queried
 
  7.4465497
 
 2.16e-03
 
 1.00e+00
  
 
  
 8
 
  55533655
 
  55534103
 
  PDF   STATS   ENSEMBL   UCSC 
 
  449
 
  7
 
 39243
 
 gene;island
 
 0.03814
 
 0.84950
 
 Not queried
 
  7.4277902
 
 5.83e-04
 
 1.00e+00
  
 
  
 8
 
  24772137
 
  24772435
 
  PDF   STATS   ENSEMBL   UCSC 
 
  299
 
  7
 
 39541
 
 tss
 
 0.05704
 
 0.91136
 
 Not queried
 
  7.4090208
 
 5.83e-04
 
 1.00e+00
  
 
  
 11
 
  15095017
 
  15095070
 
  PDF   STATS   ENSEMBL   UCSC 
 
   54
 
  5
 
 7148
 
 tss
 
 0.03234
 
 0.85084
 
 Not queried
 
  7.4023070
 
 7.94e-03
 
 1.00e+00
  
 
  
 7
 
  65878734
 
  65879115
 
  PDF   STATS   ENSEMBL   UCSC 
 
  382
 
  4
 
 38470
 
 island
 
 0.01518
 
 0.66878
 
 Not queried
 
  7.3994647
 
 2.86e-02
 
 1.00e+00
  
 
  
 6
 
 160211355
 
 160211577
 
  PDF   STATS   ENSEMBL   UCSC 
 
  223
 
  6
 
 35259
 
 tss;island
 
 0.76892
 
 0.01876
 
 Not queried
 
  7.3956472
 
 2.16e-03
 
 1.00e+00
  
 
  
 17
 
  43221220
 
  43221575
 
  PDF   STATS   ENSEMBL   UCSC 
 
  356
 
  4
 
 18391
 
 island
 
 0.02670
 
 0.84002
 
 Not queried
 
  7.3834752
 
 2.86e-02
 
 1.00e+00
  
 
  
 2
 
  74357527
 
  74357872
 
  PDF   STATS   ENSEMBL   UCSC 
 
  346
 
  4
 
 24387
 
 island
 
 0.02233
 
 0.81291
 
 Not queried
 
  7.3820314
 
 2.86e-02
 
 1.00e+00
  
 
  
 8
 
 143781297
 
 143781406
 
  PDF   STATS   ENSEMBL   UCSC 
 
  110
 
  4
 
 39869
 
 tss;island
 
 0.02975
 
 0.77073
 
 Not queried
 
  7.3341618
 
 2.86e-02
 
 1.00e+00
  
 
  
 10
 
  21797112
 
  21797191
 
  PDF   STATS   ENSEMBL   UCSC 
 
   80
 
  4
 
 5575
 
 island
 
 0.78928
 
 0.02256
 
 Not queried
 
  7.3316219
 
 2.86e-02
 
 1.00e+00
  
 
  
 7
 
   1113014
 
   1113237
 
  PDF   STATS   ENSEMBL   UCSC 
 
  224
 
  4
 
 36683
 
 gene
 
 0.04396
 
 0.90399
 
 Not queried
 
  7.3200246
 
 2.86e-02
 
 1.00e+00
  
 
  
 1
 
  59043173
 
  59043370
 
  PDF   STATS   ENSEMBL   UCSC 
 
  198
 
  6
 
 1651
 
 tss
 
 0.02555
 
 0.78057
 
 Not queried
 
  7.3186397
 
 2.16e-03
 
 1.00e+00
  
 
  
 13
 
 100547728
 
 100548078
 
  PDF   STATS   ENSEMBL   UCSC 
 
  351
 
  5
 
 11180
 
 gene;island
 
 0.06000
 
 0.93890
 
 Not queried
 
  7.3109829
 
 7.94e-03
 
 1.00e+00
  
 
  
 6
 
  26033394
 
  26033786
 
  PDF   STATS   ENSEMBL   UCSC 
 
  393
 
  6
 
 33534
 
 gene
 
 0.01307
 
 0.69523
 
 Not queried
 
  7.2977697
 
 2.16e-03
 
 1.00e+00
  
 
  
 X
 
 119133700
 
 119134082
 
  PDF   STATS   ENSEMBL   UCSC 
 
  383
 
  5
 
 41994
 
 island
 
 0.91256
 
 0.06222
 
 Not queried
 
  7.2974307
 
 7.94e-03
 
 1.00e+00
  
 
  
 12
 
  52626814
 
  52627047
 
  PDF   STATS   ENSEMBL   UCSC 
 
  234
 
  6
 
 10411
 
 island
 
 0.03118
 
 0.83634
 
 Not queried
 
  7.2836768
 
 2.16e-03
 
 1.00e+00
  
 
  
 1
 
  91192451
 
  91192803
 
  PDF   STATS   ENSEMBL   UCSC 
 
  353
 
  5
 
 3457
 
 island
 
 0.82731
 
 0.05999
 
 Not queried
 
  7.2831468
 
 7.94e-03
 
 1.00e+00
  
 
  
 1
 
   2980380
 
   2980598
 
  PDF   STATS   ENSEMBL   UCSC 
 
  219
 
  4
 
 94
 
 gene;island
 
 0.03522
 
 0.83688
 
 Not queried
 
  7.2821075
 
 2.86e-02
 
 1.00e+00
  
 
  
 22
 
  21386885
 
  21387058
 
  PDF   STATS   ENSEMBL   UCSC 
 
  174
 
  7
 
 26950
 
 tss
 
 0.02559
 
 0.74364
 
 Not queried
 
  7.2776498
 
 5.83e-04
 
 1.00e+00
  
 
  
 19
 
   2289982
 
   2290107
 
  PDF   STATS   ENSEMBL   UCSC 
 
  126
 
  4
 
 19650
 
 gene;island
 
 0.04571
 
 0.87808
 
 Not queried
 
  7.2775704
 
 2.86e-02
 
 1.00e+00
  
 
  
 1
 
  63249197
 
  63249213
 
  PDF   STATS   ENSEMBL   UCSC 
 
   17
 
  4
 
 1663
 
 tss
 
 0.02830
 
 0.84162
 
 Not queried
 
  7.2738520
 
 2.86e-02
 
 1.00e+00
  
 
  
 11
 
 117069780
 
 117069966
 
  PDF   STATS   ENSEMBL   UCSC 
 
  187
 
  7
 
 7695
 
 tss
 
 0.02835
 
 0.84932
 
 Not queried
 
  7.2661735
 
 5.83e-04
 
 1.00e+00
  
 
  
 X
 
 134429653
 
 134429972
 
  PDF   STATS   ENSEMBL   UCSC 
 
  320
 
  4
 
 41114
 
 gene;island
 
 0.04585
 
 0.88283
 
 Not queried
 
  7.2534378
 
 2.86e-02
 
 1.00e+00
  
 
  
 22
 
  37212787
 
  37213130
 
  PDF   STATS   ENSEMBL   UCSC 
 
  344
 
  4
 
 26819
 
 gene;island
 
 0.91572
 
 0.03503
 
 Not queried
 
  7.2473192
 
 2.86e-02
 
 1.00e+00
  
 
  
 2
 
 106886503
 
 106886729
 
  PDF   STATS   ENSEMBL   UCSC 
 
  227
 
  4
 
 24553
 
 island
 
 0.01299
 
 0.50599
 
 Not queried
 
  7.2354078
 
 2.86e-02
 
 1.00e+00
  
 
  
 8
 
  52722023
 
  52722237
 
  PDF   STATS   ENSEMBL   UCSC 
 
  215
 
  5
 
 39631
 
 tss
 
 0.03736
 
 0.83653
 
 Not queried
 
  7.2308292
 
 7.94e-03
 
 1.00e+00
  
 
  
 14
 
 104583048
 
 104583245
 
  PDF   STATS   ENSEMBL   UCSC 
 
  198
 
  4
 
 12629
 
 tss;island
 
 0.03992
 
 0.82290
 
 Not queried
 
  7.2229157
 
 2.86e-02
 
 1.00e+00
  
 
  
 5
 
 140530028
 
 140530606
 
  PDF   STATS   ENSEMBL   UCSC 
 
  579
 
  5
 
 31752
 
 gene;island
 
 0.10467
 
 0.95127
 
 Not queried
 
  7.1915846
 
 7.94e-03
 
 1.00e+00
  
 
  
 6
 
  76203530
 
  76203675
 
  PDF   STATS   ENSEMBL   UCSC 
 
  146
 
  6
 
 35013
 
 tss
 
 0.04415
 
 0.87764
 
 Not queried
 
  7.1585397
 
 2.16e-03
 
 1.00e+00
  
 
  
 7
 
    914964
 
    915203
 
  PDF   STATS   ENSEMBL   UCSC 
 
  240
 
  4
 
 37325
 
 tss;island
 
 0.07134
 
 0.92817
 
 Not queried
 
  7.1572095
 
 2.86e-02
 
 1.00e+00
  
 
  
 22
 
  24181212
 
  24181270
 
  PDF   STATS   ENSEMBL   UCSC 
 
   59
 
  4
 
 26971
 
 tss
 
 0.67259
 
 0.01448
 
 Not queried
 
  7.1476677
 
 2.86e-02
 
 1.00e+00
  
 
  
 22
 
  24180995
 
  24181270
 
  PDF   STATS   ENSEMBL   UCSC 
 
  276
 
  6
 
 27312
 
 island
 
 0.64213
 
 0.01448
 
 Not queried
 
  7.1476677
 
 2.16e-03
 
 1.00e+00
  
 
  
 13
 
  41768782
 
  41768978
 
  PDF   STATS   ENSEMBL   UCSC 
 
  197
 
  5
 
 11382
 
 tss
 
 0.02159
 
 0.73505
 
 Not queried
 
  7.1466731
 
 7.94e-03
 
 1.00e+00
  
 
  
 12
 
   6649733
 
   6649994
 
  PDF   STATS   ENSEMBL   UCSC 
 
  262
 
  4
 
 9004
 
 gene;island
 
 0.04367
 
 0.85483
 
 Not queried
 
  7.1222645
 
 2.86e-02
 
 1.00e+00
  
 
  
 22
 
  29711916
 
  29712139
 
  PDF   STATS   ENSEMBL   UCSC 
 
  224
 
  6
 
 27005
 
 tss;island
 
 0.93169
 
 0.07224
 
 Not queried
 
  7.1218178
 
 2.16e-03
 
 1.00e+00
  
 
  
 19
 
  15343235
 
  15343425
 
  PDF   STATS   ENSEMBL   UCSC 
 
  191
 
  6
 
 20521
 
 tss
 
 0.85072
 
 0.04046
 
 Not queried
 
  7.1158339
 
 2.16e-03
 
 1.00e+00
  
 
  
 12
 
  52626814
 
  52626904
 
  PDF   STATS   ENSEMBL   UCSC 
 
   91
 
  5
 
 9700
 
 tss
 
 0.03210
 
 0.80271
 
 Not queried
 
  7.1133491
 
 7.94e-03
 
 1.00e+00
  
 
  
 17
 
  36997420
 
  36997740
 
  PDF   STATS   ENSEMBL   UCSC 
 
  321
 
  7
 
 18228
 
 island
 
 0.06639
 
 0.91160
 
 Not queried
 
  7.1051238
 
 5.83e-04
 
 1.00e+00
  
 
  
 12
 
  65562903
 
  65563329
 
  PDF   STATS   ENSEMBL   UCSC 
 
  427
 
  7
 
 9819
 
 tss;island
 
 0.71037
 
 0.01763
 
 Not queried
 
  7.0945444
 
 4.08e-03
 
 1.00e+00
  
 
  
 11
 
  75139390
 
  75139736
 
  PDF   STATS   ENSEMBL   UCSC 
 
  347
 
  4
 
 6817
 
 gene;island
 
 0.01376
 
 0.62436
 
 Not queried
 
  7.0866390
 
 2.86e-02
 
 1.00e+00
  
 
  
 X
 
 100353031
 
 100353462
 
  PDF   STATS   ENSEMBL   UCSC 
 
  432
 
  6
 
 41907
 
 island
 
 0.02274
 
 0.68991
 
 Not queried
 
  7.0851812
 
 2.16e-03
 
 1.00e+00
  
 
  
 6
 
  27259010
 
  27259237
 
  PDF   STATS   ENSEMBL   UCSC 
 
  228
 
  5
 
 35490
 
 island
 
 0.88516
 
 0.05714
 
 Not queried
 
  7.0849833
 
 7.94e-03
 
 1.00e+00
  
 
  
 7
 
  39649224
 
  39649443
 
  PDF   STATS   ENSEMBL   UCSC 
 
  220
 
  5
 
 36911
 
 gene;island
 
 0.01812
 
 0.77272
 
 Not queried
 
  7.0793176
 
 7.94e-03
 
 1.00e+00
  
 
  
 1
 
 236687053
 
 236687277
 
  PDF   STATS   ENSEMBL   UCSC 
 
  225
 
  4
 
 1096
 
 gene;island
 
 0.04696
 
 0.79982
 
 Not queried
 
  7.0366983
 
 2.86e-02
 
 1.00e+00
  
 
  
 8
 
 114389383
 
 114389479
 
  PDF   STATS   ENSEMBL   UCSC 
 
   97
 
  4
 
 39812
 
 tss
 
 0.05779
 
 0.84163
 
 Not queried
 
  7.0280782
 
 2.86e-02
 
 1.00e+00
  
 
  
 5
 
 140207460
 
 140207636
 
  PDF   STATS   ENSEMBL   UCSC 
 
  177
 
  5
 
 32293
 
 tss
 
 0.06297
 
 0.86472
 
 Not queried
 
  7.0143903
 
 7.94e-03
 
 1.00e+00
  
 
  
 22
 
  36877697
 
  36877737
 
  PDF   STATS   ENSEMBL   UCSC 
 
   41
 
  4
 
 27066
 
 tss
 
 0.01260
 
 0.59779
 
 Not queried
 
  6.9922388
 
 2.86e-02
 
 1.00e+00
  
 
  
 5
 
  54826547
 
  54826768
 
  PDF   STATS   ENSEMBL   UCSC 
 
  222
 
  4
 
 31562
 
 gene;island
 
 0.01582
 
 0.63050
 
 Not queried
 
  6.9919491
 
 5.71e-02
 
 1.00e+00
  
 
  
 10
 
 118900324
 
 118900492
 
  PDF   STATS   ENSEMBL   UCSC 
 
  169
 
  4
 
 6154
 
 island
 
 0.91452
 
 0.09738
 
 Not queried
 
  6.9847451
 
 2.86e-02
 
 1.00e+00
  
 
  
 12
 
  66583029
 
  66583431
 
  PDF   STATS   ENSEMBL   UCSC 
 
  403
 
  5
 
 9182
 
 gene;island
 
 0.03316
 
 0.84151
 
 Not queried
 
  6.9748837
 
 7.94e-03
 
 1.00e+00
  
 
  
 14
 
 105714153
 
 105714589
 
  PDF   STATS   ENSEMBL   UCSC 
 
  437
 
  6
 
 12230
 
 gene;tss;island
 
 0.10548
 
 0.91835
 
 Not queried
 
  6.9704860
 
 2.16e-03
 
 1.00e+00
  
 
  
 1
 
 158119395
 
 158119673
 
  PDF   STATS   ENSEMBL   UCSC 
 
  279
 
  5
 
 3865
 
 island
 
 0.04065
 
 0.85340
 
 Not queried
 
  6.9623435
 
 7.94e-03
 
 1.00e+00
  
 
  
 6
 
  26021768
 
  26021843
 
  PDF   STATS   ENSEMBL   UCSC 
 
   76
 
  4
 
 34615
 
 tss
 
 0.14909
 
 0.95043
 
 Not queried
 
  6.9505862
 
 2.86e-02
 
 1.00e+00
  
 
  
 2
 
 128458240
 
 128458556
 
  PDF   STATS   ENSEMBL   UCSC 
 
  317
 
  6
 
 23578
 
 tss
 
 0.91678
 
 0.05747
 
 Not queried
 
  6.9197549
 
 2.16e-03
 
 1.00e+00
  
 
  
 5
 
  76010444
 
  76010658
 
  PDF   STATS   ENSEMBL   UCSC 
 
  215
 
  4
 
 32075
 
 tss;island
 
 0.93070
 
 0.08555
 
 Not queried
 
  6.9113225
 
 2.86e-02
 
 1.00e+00
  
 
  
 7
 
  50132541
 
  50132764
 
  PDF   STATS   ENSEMBL   UCSC 
 
  224
 
  4
 
 36947
 
 gene
 
 0.04805
 
 0.80515
 
 Not queried
 
  6.9109009
 
 2.86e-02
 
 1.00e+00
  
 
  
 7
 
 124404244
 
 124404472
 
  PDF   STATS   ENSEMBL   UCSC 
 
  229
 
  4
 
 37144
 
 gene;island
 
 0.02319
 
 0.65492
 
 Not queried
 
  6.9063927
 
 2.86e-02
 
 1.00e+00
  
 
  
 2
 
 128458240
 
 128458845
 
  PDF   STATS   ENSEMBL   UCSC 
 
  606
 
  8
 
 24662
 
 island
 
 0.89523
 
 0.04643
 
 Not queried
 
  6.8942259
 
 1.04e-02
 
 1.00e+00
  
 
  
 2
 
  11051728
 
  11052102
 
  PDF   STATS   ENSEMBL   UCSC 
 
  375
 
  7
 
 24036
 
 island
 
 0.02830
 
 0.88806
 
 Not queried
 
  6.8935994
 
 5.83e-04
 
 1.00e+00
  
 
  
 16
 
   2902830
 
   2903392
 
  PDF   STATS   ENSEMBL   UCSC 
 
  563
 
  5
 
 14586
 
 gene;island
 
 0.06340
 
 0.75620
 
 Not queried
 
  6.8753667
 
 7.94e-03
 
 1.00e+00
  
 
  
 3
 
 169530268
 
 169530621
 
  PDF   STATS   ENSEMBL   UCSC 
 
  354
 
  6
 
 29606
 
 island
 
 0.02610
 
 0.80172
 
 Not queried
 
  6.8731844
 
 2.16e-03
 
 1.00e+00
  
 
  
 7
 
  12151221
 
  12151638
 
  PDF   STATS   ENSEMBL   UCSC 
 
  418
 
  4
 
 38200
 
 island
 
 0.06686
 
 0.83198
 
 Not queried
 
  6.8610480
 
 2.86e-02
 
 1.00e+00
  
 
  
 5
 
 140254793
 
 140255069
 
  PDF   STATS   ENSEMBL   UCSC 
 
  277
 
  4
 
 31745
 
 gene;island
 
 0.06048
 
 0.91864
 
 Not queried
 
  6.8553653
 
 2.86e-02
 
 1.00e+00
  
 
  
 15
 
  28016437
 
  28016654
 
  PDF   STATS   ENSEMBL   UCSC 
 
  218
 
  4
 
 13234
 
 gene;island
 
 0.08077
 
 0.81648
 
 Not queried
 
  6.8472722
 
 2.86e-02
 
 1.00e+00
  
 
  
 6
 
  44265644
 
  44265696
 
  PDF   STATS   ENSEMBL   UCSC 
 
   53
 
  4
 
 34947
 
 tss;island
 
 0.18847
 
 0.97050
 
 Not queried
 
  6.8438712
 
 2.86e-02
 
 1.00e+00
  
 
  
 17
 
  21030030
 
  21030248
 
  PDF   STATS   ENSEMBL   UCSC 
 
  219
 
  7
 
 17167
 
 tss
 
 0.01598
 
 0.65584
 
 Not queried
 
  6.8360730
 
 5.83e-04
 
 1.00e+00
  
 
  
 10
 
  38692145
 
  38692317
 
  PDF   STATS   ENSEMBL   UCSC 
 
  173
 
  4
 
 5021
 
 tss;island
 
 0.03423
 
 0.58912
 
 Not queried
 
  6.8257281
 
 5.71e-02
 
 1.00e+00
  
 
  
 17
 
  79031376
 
  79031712
 
  PDF   STATS   ENSEMBL   UCSC 
 
  337
 
  5
 
 16857
 
 gene
 
 0.03776
 
 0.85479
 
 Not queried
 
  6.8215930
 
 7.94e-03
 
 1.00e+00
  
 
  
 11
 
    393938
 
    394289
 
  PDF   STATS   ENSEMBL   UCSC 
 
  352
 
  5
 
 7825
 
 island
 
 0.04189
 
 0.78778
 
 Not queried
 
  6.8215214
 
 7.94e-03
 
 1.00e+00
  
 
  
 16
 
  67906784
 
  67906986
 
  PDF   STATS   ENSEMBL   UCSC 
 
  203
 
  5
 
 15283
 
 tss
 
 0.01766
 
 0.65219
 
 Not queried
 
  6.8146199
 
 7.94e-03
 
 1.00e+00
  
 
  
 4
 
  75719957
 
  75720078
 
  PDF   STATS   ENSEMBL   UCSC 
 
  122
 
  5
 
 30389
 
 tss;island
 
 0.02845
 
 0.76156
 
 Not queried
 
  6.8080280
 
 7.94e-03
 
 1.00e+00
  
 
  
 6
 
  32977400
 
  32977708
 
  PDF   STATS   ENSEMBL   UCSC 
 
  309
 
  4
 
 34822
 
 tss
 
 0.48113
 
 0.01932
 
 Not queried
 
  6.8080259
 
 1.14e-01
 
 1.00e+00
  
 
  
 8
 
  82633568
 
  82633764
 
  PDF   STATS   ENSEMBL   UCSC 
 
  197
 
  7
 
 39721
 
 tss
 
 0.01386
 
 0.48673
 
 Not queried
 
  6.8072081
 
 5.83e-04
 
 1.00e+00
  
 
  
 X
 
  21776242
 
  21776494
 
  PDF   STATS   ENSEMBL   UCSC 
 
  253
 
  6
 
 41221
 
 tss
 
 0.10051
 
 0.90202
 
 Not queried
 
  6.7942066
 
 2.16e-03
 
 1.00e+00
  
 
  
 2
 
 208635549
 
 208636363
 
  PDF   STATS   ENSEMBL   UCSC 
 
  815
 
 10
 
 25005
 
 island
 
 0.85574
 
 0.04958
 
 Not queried
 
  6.7925346
 
 4.87e-04
 
 1.00e+00
  
 
  
 10
 
  38645346
 
  38645450
 
  PDF   STATS   ENSEMBL   UCSC 
 
  105
 
  4
 
 4547
 
 gene;island
 
 0.03040
 
 0.64559
 
 Not queried
 
  6.7918378
 
 2.86e-02
 
 1.00e+00
  
 
  
 1
 
 209848306
 
 209848577
 
  PDF   STATS   ENSEMBL   UCSC 
 
  272
 
  5
 
 2227
 
 tss;island
 
 0.87335
 
 0.05872
 
 Not queried
 
  6.7883848
 
 7.94e-03
 
 1.00e+00
  
 
  
 6
 
  88031886
 
  88032210
 
  PDF   STATS   ENSEMBL   UCSC 
 
  325
 
  7
 
 35044
 
 tss
 
 0.03456
 
 0.83561
 
 Not queried
 
  6.7818546
 
 5.83e-04
 
 1.00e+00
  
 
  
 11
 
  67442067
 
  67442408
 
  PDF   STATS   ENSEMBL   UCSC 
 
  342
 
  6
 
 6746
 
 gene
 
 0.02979
 
 0.71849
 
 Not queried
 
  6.7813249
 
 2.16e-03
 
 1.00e+00
  
 
  
 3
 
 169530417
 
 169530621
 
  PDF   STATS   ENSEMBL   UCSC 
 
  205
 
  5
 
 28666
 
 tss
 
 0.02835
 
 0.77760
 
 Not queried
 
  6.7785307
 
 7.94e-03
 
 1.00e+00
  
 
  
 6
 
  79577539
 
  79577801
 
  PDF   STATS   ENSEMBL   UCSC 
 
  263
 
  4
 
 34332
 
 gene;island
 
 0.01672
 
 0.62111
 
 Not queried
 
  6.7656675
 
 5.71e-02
 
 1.00e+00
  
 
  
 X
 
 119134323
 
 119134684
 
  PDF   STATS   ENSEMBL   UCSC 
 
  362
 
  4
 
 41995
 
 island
 
 0.95402
 
 0.16257
 
 Not queried
 
  6.7637114
 
 2.86e-02
 
 1.00e+00
  
 
  
 1
 
  74663583
 
  74663827
 
  PDF   STATS   ENSEMBL   UCSC 
 
  245
 
  5
 
 582
 
 gene
 
 0.01411
 
 0.53298
 
 Not queried
 
  6.7534832
 
 7.94e-03
 
 1.00e+00
  
 
  
 20
 
  22559305
 
  22559456
 
  PDF   STATS   ENSEMBL   UCSC 
 
  152
 
  4
 
 25593
 
 tss;island
 
 0.02419
 
 0.69319
 
 Not queried
 
  6.7439198
 
 2.86e-02
 
 1.00e+00
  
 
  
 15
 
  31372742
 
  31373178
 
  PDF   STATS   ENSEMBL   UCSC 
 
  437
 
  4
 
 13243
 
 gene
 
 0.87098
 
 0.07111
 
 Not queried
 
  6.7408259
 
 5.71e-02
 
 1.00e+00
  
 
  
 X
 
 154299274
 
 154299395
 
  PDF   STATS   ENSEMBL   UCSC 
 
  122
 
  4
 
 41166
 
 gene;tss;island
 
 0.01371
 
 0.60990
 
 Not queried
 
  6.7318599
 
 2.86e-02
 
 1.00e+00
  
 
  
 2
 
 176981328
 
 176981469
 
  PDF   STATS   ENSEMBL   UCSC 
 
  142
 
  4
 
 23697
 
 tss
 
 0.01257
 
 0.56786
 
 Not queried
 
  6.7229592
 
 2.86e-02
 
 1.00e+00
  
 
  
 7
 
 157809235
 
 157809596
 
  PDF   STATS   ENSEMBL   UCSC 
 
  362
 
  6
 
 37293
 
 gene
 
 0.77168
 
 0.04734
 
 Not queried
 
  6.6974400
 
 2.16e-03
 
 1.00e+00
  
 
  
 19
 
  37742739
 
  37742956
 
  PDF   STATS   ENSEMBL   UCSC 
 
  218
 
  4
 
 21916
 
 island
 
 0.76963
 
 0.03306
 
 Not queried
 
  6.6961474
 
 2.86e-02
 
 1.00e+00
  
 
  
 17
 
  48172598
 
  48172668
 
  PDF   STATS   ENSEMBL   UCSC 
 
   71
 
  4
 
 17459
 
 tss;island
 
 0.02749
 
 0.69301
 
 Not queried
 
  6.6956182
 
 2.86e-02
 
 1.00e+00
  
 
  
 12
 
   7858592
 
   7858848
 
  PDF   STATS   ENSEMBL   UCSC 
 
  257
 
  6
 
 10202
 
 island
 
 0.09536
 
 0.90491
 
 Not queried
 
  6.6954925
 
 2.16e-03
 
 1.00e+00
  
 
  
 Y
 
   2655740
 
   2655977
 
  PDF   STATS   ENSEMBL   UCSC 
 
  238
 
  5
 
 42175
 
 island
 
 0.01549
 
 0.30053
 
 Not queried
 
  6.6792630
 
 4.21e-01
 
 1.00e+00
  
 
  
 15
 
  25018132
 
  25018449
 
  PDF   STATS   ENSEMBL   UCSC 
 
  318
 
  4
 
 13923
 
 island
 
 0.13870
 
 0.93237
 
 Not queried
 
  6.6711273
 
 2.86e-02
 
 1.00e+00
  
 
  
 2
 
  66667039
 
  66667101
 
  PDF   STATS   ENSEMBL   UCSC 
 
   63
 
  5
 
 22674
 
 gene
 
 0.64999
 
 0.02538
 
 Not queried
 
  6.6701626
 
 7.94e-03
 
 1.00e+00
  
 
  
 7
 
  27204663
 
  27205114
 
  PDF   STATS   ENSEMBL   UCSC 
 
  452
 
  7
 
 36875
 
 gene
 
 0.04069
 
 0.74520
 
 Not queried
 
  6.6667050
 
 5.83e-04
 
 1.00e+00
  
 
  
 X
 
 153059760
 
 153059963
 
  PDF   STATS   ENSEMBL   UCSC 
 
  204
 
  4
 
 41147
 
 gene
 
 0.00796
 
 0.44839
 
 Not queried
 
  6.6529008
 
 2.86e-02
 
 1.00e+00
  
 
  
 8
 
  18541446
 
  18541627
 
  PDF   STATS   ENSEMBL   UCSC 
 
  182
 
  5
 
 39156
 
 gene
 
 0.04223
 
 0.81606
 
 Not queried
 
  6.6526697
 
 7.94e-03
 
 1.00e+00
  
 
  
 13
 
 103426286
 
 103426481
 
  PDF   STATS   ENSEMBL   UCSC 
 
  196
 
  7
 
 11495
 
 tss;island
 
 0.01398
 
 0.58667
 
 Not queried
 
  6.6449334
 
 1.17e-03
 
 1.00e+00
  
 
  
 15
 
  39871808
 
  39872186
 
  PDF   STATS   ENSEMBL   UCSC 
 
  379
 
  7
 
 13547
 
 tss;island
 
 0.04241
 
 0.85604
 
 Not queried
 
  6.6292377
 
 1.17e-03
 
 1.00e+00
  
 
  
 5
 
 140213783
 
 140213924
 
  PDF   STATS   ENSEMBL   UCSC 
 
  142
 
  5
 
 31741
 
 gene;tss;island
 
 0.02658
 
 0.91968
 
 Not queried
 
  6.6290947
 
 7.94e-03
 
 1.00e+00
  
 
  
 2
 
 176981328
 
 176981654
 
  PDF   STATS   ENSEMBL   UCSC 
 
  327
 
  5
 
 24860
 
 island
 
 0.01516
 
 0.60302
 
 Not queried
 
  6.6242560
 
 9.52e-02
 
 1.00e+00
  
 
  
 7
 
  56515510
 
  56515846
 
  PDF   STATS   ENSEMBL   UCSC 
 
  337
 
  5
 
 36959
 
 gene;island
 
 0.02224
 
 0.69929
 
 Not queried
 
  6.6210518
 
 7.94e-03
 
 1.00e+00
  
 
  
 19
 
  15344046
 
  15344364
 
  PDF   STATS   ENSEMBL   UCSC 
 
  319
 
  4
 
 20522
 
 tss;island
 
 0.75708
 
 0.04558
 
 Not queried
 
  6.6128499
 
 2.86e-02
 
 1.00e+00
  
 
  
 12
 
  29302016
 
  29302279
 
  PDF   STATS   ENSEMBL   UCSC 
 
  264
 
  5
 
 10278
 
 island
 
 0.01646
 
 0.59787
 
 Not queried
 
  6.6088143
 
 7.94e-03
 
 1.00e+00
  
 
  
 11
 
 117800233
 
 117800571
 
  PDF   STATS   ENSEMBL   UCSC 
 
  339
 
  5
 
 7702
 
 tss
 
 0.05404
 
 0.86424
 
 Not queried
 
  6.6048977
 
 7.94e-03
 
 1.00e+00
  
 
  
 19
 
  50194120
 
  50194252
 
  PDF   STATS   ENSEMBL   UCSC 
 
  133
 
  5
 
 20925
 
 tss;island
 
 0.82439
 
 0.05214
 
 Not queried
 
  6.5935483
 
 7.94e-03
 
 1.00e+00
  
 
  
 1
 
  91185028
 
  91185441
 
  PDF   STATS   ENSEMBL   UCSC 
 
  414
 
  6
 
 3453
 
 island
 
 0.80804
 
 0.04225
 
 Not queried
 
  6.5890960
 
 2.16e-03
 
 1.00e+00
  
 
  
 X
 
 135579980
 
 135580181
 
  PDF   STATS   ENSEMBL   UCSC 
 
  202
 
  4
 
 41120
 
 gene;island
 
 0.01592
 
 0.57619
 
 Not queried
 
  6.5819424
 
 2.86e-02
 
 1.00e+00
  
 
  
 17
 
  75315486
 
  75315567
 
  PDF   STATS   ENSEMBL   UCSC 
 
   82
 
  5
 
 17663
 
 tss
 
 0.03891
 
 0.78937
 
 Not queried
 
  6.5618303
 
 7.94e-03
 
 1.00e+00
  
 
  
 14
 
 105714833
 
 105715025
 
  PDF   STATS   ENSEMBL   UCSC 
 
  193
 
  4
 
 12231
 
 gene;tss;island
 
 0.07014
 
 0.84429
 
 Not queried
 
  6.5601455
 
 2.86e-02
 
 1.00e+00
  
 
  
 19
 
   1132294
 
   1132537
 
  PDF   STATS   ENSEMBL   UCSC 
 
  244
 
  7
 
 20256
 
 tss
 
 0.02918
 
 0.79081
 
 Not queried
 
  6.5514378
 
 5.83e-04
 
 1.00e+00
  
 
  
 13
 
  41768693
 
  41768978
 
  PDF   STATS   ENSEMBL   UCSC 
 
  286
 
  6
 
 11663
 
 island
 
 0.05191
 
 0.72537
 
 Not queried
 
  6.5505384
 
 2.16e-03
 
 1.00e+00
  
 
  
 12
 
  51236582
 
  51236768
 
  PDF   STATS   ENSEMBL   UCSC 
 
  187
 
  6
 
 10392
 
 island
 
 0.09338
 
 0.91108
 
 Not queried
 
  6.5504598
 
 2.16e-03
 
 1.00e+00
  
 
  
 17
 
  19437691
 
  19437929
 
  PDF   STATS   ENSEMBL   UCSC 
 
  239
 
  4
 
 16445
 
 gene;island
 
 0.77999
 
 0.08404
 
 Not queried
 
  6.5409379
 
 2.86e-02
 
 1.00e+00
  
 
  
 8
 
 114389168
 
 114389479
 
  PDF   STATS   ENSEMBL   UCSC 
 
  312
 
  6
 
 39338
 
 gene
 
 0.08705
 
 0.87580
 
 Not queried
 
  6.5403956
 
 2.16e-03
 
 1.00e+00
  
 
  
 1
 
 165567312
 
 165567529
 
  PDF   STATS   ENSEMBL   UCSC 
 
  218
 
  4
 
 3917
 
 island
 
 0.02010
 
 0.58561
 
 Not queried
 
  6.5346148
 
 2.86e-02
 
 1.00e+00
  
 
  
 1
 
  29138919
 
  29139278
 
  PDF   STATS   ENSEMBL   UCSC 
 
  360
 
  5
 
 370
 
 gene;island
 
 0.01203
 
 0.51739
 
 Not queried
 
  6.5334328
 
 7.94e-03
 
 1.00e+00
  
 
  
 X
 
 134477802
 
 134478126
 
  PDF   STATS   ENSEMBL   UCSC 
 
  325
 
  5
 
 41540
 
 tss;island
 
 0.01845
 
 0.71486
 
 Not queried
 
  6.5308005
 
 7.94e-03
 
 1.00e+00
  
 
  
 10
 
 115860606
 
 115860959
 
  PDF   STATS   ENSEMBL   UCSC 
 
  354
 
  4
 
 6130
 
 island
 
 0.01864
 
 0.69297
 
 Not queried
 
  6.5260473
 
 2.86e-02
 
 1.00e+00
  
 
  
 6
 
  32977138
 
  32977708
 
  PDF   STATS   ENSEMBL   UCSC 
 
  571
 
  9
 
 35893
 
 island
 
 0.73315
 
 0.05162
 
 Not queried
 
  6.5139256
 
 1.85e-03
 
 1.00e+00
  
 
  
 2
 
  61371880
 
  61372226
 
  PDF   STATS   ENSEMBL   UCSC 
 
  347
 
  7
 
 23339
 
 tss
 
 0.07409
 
 0.86546
 
 Not queried
 
  6.5133601
 
 5.83e-04
 
 1.00e+00
  
 
  
 3
 
  49236800
 
  49236946
 
  PDF   STATS   ENSEMBL   UCSC 
 
  147
 
  6
 
 27736
 
 gene;island
 
 0.11060
 
 0.89288
 
 Not queried
 
  6.5038102
 
 2.16e-03
 
 1.00e+00
  
 
  
 13
 
  48895173
 
  48895478
 
  PDF   STATS   ENSEMBL   UCSC 
 
  306
 
  4
 
 11133
 
 gene;island
 
 0.04537
 
 0.81251
 
 Not queried
 
  6.5006648
 
 2.86e-02
 
 1.00e+00
  
 
  
 3
 
 147142102
 
 147142415
 
  PDF   STATS   ENSEMBL   UCSC 
 
  314
 
  5
 
 29533
 
 island
 
 0.82789
 
 0.05037
 
 Not queried
 
  6.5005863
 
 7.94e-03
 
 1.00e+00
  
 
  
 2
 
 186603398
 
 186603639
 
  PDF   STATS   ENSEMBL   UCSC 
 
  242
 
  4
 
 24908
 
 island
 
 0.00422
 
 0.26337
 
 Not queried
 
  6.4898631
 
 2.86e-02
 
 1.00e+00
  
 
  
 2
 
  11051728
 
  11051991
 
  PDF   STATS   ENSEMBL   UCSC 
 
  264
 
  5
 
 23148
 
 tss
 
 0.06255
 
 0.88806
 
 Not queried
 
  6.4869148
 
 7.94e-03
 
 1.00e+00
  
 
  
 1
 
  11752109
 
  11752494
 
  PDF   STATS   ENSEMBL   UCSC 
 
  386
 
  4
 
 225
 
 gene;tss;island
 
 0.69754
 
 0.01792
 
 Not queried
 
  6.4754427
 
 2.86e-02
 
 1.00e+00
  
 
  
 14
 
  23937965
 
  23938207
 
  PDF   STATS   ENSEMBL   UCSC 
 
  243
 
  4
 
 12276
 
 tss
 
 0.12323
 
 0.89202
 
 Not queried
 
  6.4712170
 
 2.86e-02
 
 1.00e+00
  
 
  
 17
 
  72352688
 
  72352968
 
  PDF   STATS   ENSEMBL   UCSC 
 
  281
 
  4
 
 16717
 
 gene;island
 
 0.07471
 
 0.76928
 
 Not queried
 
  6.4597661
 
 2.86e-02
 
 1.00e+00
  
 
  
 X
 
  50024233
 
  50024550
 
  PDF   STATS   ENSEMBL   UCSC 
 
  318
 
  4
 
 41795
 
 island
 
 0.32943
 
 0.95726
 
 Not queried
 
  6.4577330
 
 2.86e-02
 
 1.00e+00
  
 
  
 16
 
   4311628
 
   4311941
 
  PDF   STATS   ENSEMBL   UCSC 
 
  314
 
  5
 
 14606
 
 gene;island
 
 0.06305
 
 0.74432
 
 Not queried
 
  6.4566695
 
 7.94e-03
 
 1.00e+00
  
 
  
 X
 
  79590789
 
  79590966
 
  PDF   STATS   ENSEMBL   UCSC 
 
  178
 
  7
 
 41404
 
 tss
 
 0.01492
 
 0.52131
 
 Not queried
 
  6.4470742
 
 5.83e-04
 
 1.00e+00
  
 
  
 21
 
  38593636
 
  38593754
 
  PDF   STATS   ENSEMBL   UCSC 
 
  119
 
  4
 
 26637
 
 island
 
 0.01831
 
 0.61807
 
 Not queried
 
  6.4423017
 
 2.86e-02
 
 1.00e+00
  
 
  
 17
 
  46682390
 
  46682413
 
  PDF   STATS   ENSEMBL   UCSC 
 
   24
 
  4
 
 17432
 
 tss
 
 0.06695
 
 0.87101
 
 Not queried
 
  6.4387914
 
 2.86e-02
 
 1.00e+00
  
 
  
 14
 
 103674159
 
 103674342
 
  PDF   STATS   ENSEMBL   UCSC 
 
  184
 
  4
 
 13155
 
 island
 
 0.83611
 
 0.10448
 
 Not queried
 
  6.4348038
 
 2.86e-02
 
 1.00e+00
  
 
  
 12
 
  98850691
 
  98851021
 
  PDF   STATS   ENSEMBL   UCSC 
 
  331
 
  5
 
 10676
 
 island
 
 0.16316
 
 0.88762
 
 Not queried
 
  6.4308977
 
 7.94e-03
 
 1.00e+00
  
 
  
 1
 
  86174060
 
  86174240
 
  PDF   STATS   ENSEMBL   UCSC 
 
  181
 
  7
 
 3421
 
 island
 
 0.01027
 
 0.53554
 
 Not queried
 
  6.4239798
 
 5.83e-04
 
 1.00e+00
  
 
  
 13
 
  46742691
 
  46742802
 
  PDF   STATS   ENSEMBL   UCSC 
 
  112
 
  4
 
 11128
 
 gene
 
 0.01522
 
 0.56788
 
 Not queried
 
  6.4165421
 
 2.86e-02
 
 1.00e+00
  
 
  
 6
 
  24357720
 
  24357985
 
  PDF   STATS   ENSEMBL   UCSC 
 
  266
 
  4
 
 33525
 
 gene;island
 
 0.05582
 
 0.86875
 
 Not queried
 
  6.4133251
 
 2.86e-02
 
 1.00e+00
  
 
  
 9
 
  89763370
 
  89763570
 
  PDF   STATS   ENSEMBL   UCSC 
 
  201
 
  4
 
 40836
 
 island
 
 0.09329
 
 0.91583
 
 Not queried
 
  6.4069965
 
 2.86e-02
 
 1.00e+00
  
 
  
 6
 
  27647713
 
  27648057
 
  PDF   STATS   ENSEMBL   UCSC 
 
  345
 
  5
 
 35497
 
 island
 
 0.00566
 
 0.29604
 
 Not queried
 
  6.4005532
 
 7.94e-03
 
 1.00e+00
  
 
  
 11
 
  67442126
 
  67442408
 
  PDF   STATS   ENSEMBL   UCSC 
 
  283
 
  4
 
 7480
 
 tss
 
 0.02947
 
 0.69940
 
 Not queried
 
  6.3946761
 
 2.86e-02
 
 1.00e+00
  
 
  
 10
 
  94825547
 
  94825848
 
  PDF   STATS   ENSEMBL   UCSC 
 
  302
 
  4
 
 4665
 
 gene;island
 
 0.10428
 
 0.88325
 
 Not queried
 
  6.3940138
 
 2.86e-02
 
 1.00e+00
  
 
  
 X
 
 154299647
 
 154299853
 
  PDF   STATS   ENSEMBL   UCSC 
 
  207
 
  5
 
 41612
 
 tss;island
 
 0.01874
 
 0.57887
 
 Not queried
 
  6.3936350
 
 7.94e-03
 
 1.00e+00
  
 
  
 15
 
  91427821
 
  91428056
 
  PDF   STATS   ENSEMBL   UCSC 
 
  236
 
  5
 
 13449
 
 gene;tss;island
 
 0.06320
 
 0.85316
 
 Not queried
 
  6.3884027
 
 7.94e-03
 
 1.00e+00
  
 
  
 2
 
 219866424
 
 219866628
 
  PDF   STATS   ENSEMBL   UCSC 
 
  205
 
  6
 
 25064
 
 island
 
 0.01417
 
 0.52500
 
 Not queried
 
  6.3783157
 
 2.16e-03
 
 1.00e+00
  
 
  
 22
 
  19929066
 
  19929254
 
  PDF   STATS   ENSEMBL   UCSC 
 
  189
 
  5
 
 26926
 
 tss
 
 0.01684
 
 0.57003
 
 Not queried
 
  6.3679554
 
 7.94e-03
 
 1.00e+00
  
 
  
 8
 
   1649758
 
   1650309
 
  PDF   STATS   ENSEMBL   UCSC 
 
  552
 
  5
 
 39110
 
 gene;island
 
 0.08054
 
 0.93941
 
 Not queried
 
  6.3648597
 
 7.94e-03
 
 1.00e+00
  
 
  
 X
 
 134478751
 
 134479169
 
  PDF   STATS   ENSEMBL   UCSC 
 
  419
 
  5
 
 41115
 
 gene
 
 0.01346
 
 0.52729
 
 Not queried
 
  6.3529280
 
 7.94e-03
 
 1.00e+00
  
 
  
 2
 
  43903470
 
  43904011
 
  PDF   STATS   ENSEMBL   UCSC 
 
  542
 
  8
 
 22623
 
 gene;tss
 
 0.09554
 
 0.88369
 
 Not queried
 
  6.3476038
 
 3.11e-04
 
 1.00e+00
  
 
  
 1
 
  86174125
 
  86174240
 
  PDF   STATS   ENSEMBL   UCSC 
 
  116
 
  6
 
 1720
 
 tss
 
 0.01142
 
 0.51742
 
 Not queried
 
  6.3416911
 
 2.16e-03
 
 1.00e+00
  
 
  
 X
 
  13835198
 
  13835295
 
  PDF   STATS   ENSEMBL   UCSC 
 
   98
 
  4
 
 40941
 
 gene
 
 0.75524
 
 0.03341
 
 Not queried
 
  6.3347409
 
 2.86e-02
 
 1.00e+00
  
 
  
 16
 
  57836242
 
  57836706
 
  PDF   STATS   ENSEMBL   UCSC 
 
  465
 
  6
 
 15936
 
 island
 
 0.03709
 
 0.68130
 
 Not queried
 
  6.3341668
 
 2.16e-03
 
 1.00e+00
  
 
  
 16
 
  20360206
 
  20360497
 
  PDF   STATS   ENSEMBL   UCSC 
 
  292
 
  4
 
 14653
 
 gene;island
 
 0.03436
 
 0.67096
 
 Not queried
 
  6.3332774
 
 2.86e-02
 
 1.00e+00
  
 
  
 4
 
  85422998
 
  85423302
 
  PDF   STATS   ENSEMBL   UCSC 
 
  305
 
  4
 
 31109
 
 island
 
 0.11847
 
 0.87313
 
 Not queried
 
  6.3331096
 
 2.86e-02
 
 1.00e+00
  
 
  
 2
 
 214148856
 
 214149035
 
  PDF   STATS   ENSEMBL   UCSC 
 
  180
 
  7
 
 23821
 
 tss
 
 0.01209
 
 0.47248
 
 Not queried
 
  6.3285562
 
 5.83e-04
 
 1.00e+00
  
 
  
 10
 
   2357224
 
   2357587
 
  PDF   STATS   ENSEMBL   UCSC 
 
  364
 
  5
 
 5483
 
 island
 
 0.06932
 
 0.89598
 
 Not queried
 
  6.3262553
 
 7.94e-03
 
 1.00e+00
  
 
  
 2
 
   3642710
 
   3642967
 
  PDF   STATS   ENSEMBL   UCSC 
 
  258
 
  5
 
 22516
 
 gene
 
 0.04520
 
 0.79070
 
 Not queried
 
  6.3184280
 
 7.94e-03
 
 1.00e+00
  
 
  
 3
 
  38035463
 
  38036060
 
  PDF   STATS   ENSEMBL   UCSC 
 
  598
 
  7
 
 27676
 
 gene;island
 
 0.06738
 
 0.76059
 
 Not queried
 
  6.3163618
 
 5.83e-04
 
 1.00e+00
  
 
  
 18
 
  43608309
 
  43608548
 
  PDF   STATS   ENSEMBL   UCSC 
 
  240
 
  4
 
 19065
 
 gene;island
 
 0.90787
 
 0.05268
 
 Not queried
 
  6.3083390
 
 2.86e-02
 
 1.00e+00
  
 
  
 7
 
 128470217
 
 128470614
 
  PDF   STATS   ENSEMBL   UCSC 
 
  398
 
  5
 
 38835
 
 island
 
 0.05841
 
 0.88401
 
 Not queried
 
  6.3064531
 
 7.94e-03
 
 1.00e+00
  
 
  
 X
 
 114524264
 
 114524286
 
  PDF   STATS   ENSEMBL   UCSC 
 
   23
 
  4
 
 41477
 
 tss
 
 0.10952
 
 0.92458
 
 Not queried
 
  6.3021709
 
 2.86e-02
 
 1.00e+00
  
 
  
 4
 
  85402870
 
  85403409
 
  PDF   STATS   ENSEMBL   UCSC 
 
  540
 
  5
 
 31105
 
 island
 
 0.12134
 
 0.93920
 
 Not queried
 
  6.2895143
 
 7.94e-03
 
 1.00e+00
  
 
  
 6
 
  74104388
 
  74104600
 
  PDF   STATS   ENSEMBL   UCSC 
 
  213
 
  4
 
 34323
 
 gene;island
 
 0.02474
 
 0.62052
 
 Not queried
 
  6.2862212
 
 2.86e-02
 
 1.00e+00
  
 
  
 6
 
  84140785
 
  84140997
 
  PDF   STATS   ENSEMBL   UCSC 
 
  213
 
  6
 
 35031
 
 tss
 
 0.05223
 
 0.88075
 
 Not queried
 
  6.2825093
 
 2.16e-03
 
 1.00e+00
  
 
  
 X
 
 153657001
 
 153657411
 
  PDF   STATS   ENSEMBL   UCSC 
 
  411
 
  5
 
 41159
 
 gene
 
 0.02108
 
 0.62538
 
 Not queried
 
  6.2765311
 
 7.94e-03
 
 1.00e+00
  
 
  
 13
 
  47012103
 
  47012543
 
  PDF   STATS   ENSEMBL   UCSC 
 
  441
 
  4
 
 11686
 
 island
 
 0.11147
 
 0.88343
 
 Not queried
 
  6.2709931
 
 2.86e-02
 
 1.00e+00
  
 
  
 6
 
 125284203
 
 125284359
 
  PDF   STATS   ENSEMBL   UCSC 
 
  157
 
  6
 
 35144
 
 tss;island
 
 0.67986
 
 0.02681
 
 Not queried
 
  6.2687940
 
 2.16e-03
 
 1.00e+00
  
 
  
 12
 
  25102311
 
  25102521
 
  PDF   STATS   ENSEMBL   UCSC 
 
  211
 
  4
 
 9583
 
 tss
 
 0.86091
 
 0.06660
 
 Not queried
 
  6.2637655
 
 2.86e-02
 
 1.00e+00
  
 
  
 X
 
 154299737
 
 154299853
 
  PDF   STATS   ENSEMBL   UCSC 
 
  117
 
  4
 
 41167
 
 gene
 
 0.01901
 
 0.56749
 
 Not queried
 
  6.2604834
 
 2.86e-02
 
 1.00e+00
  
 
  
 19
 
  38755103
 
  38755330
 
  PDF   STATS   ENSEMBL   UCSC 
 
  228
 
  5
 
 19978
 
 gene
 
 0.01491
 
 0.56187
 
 Not queried
 
  6.2602762
 
 7.94e-03
 
 1.00e+00
  
 
  
 21
 
  39493025
 
  39493391
 
  PDF   STATS   ENSEMBL   UCSC 
 
  367
 
  5
 
 26384
 
 gene
 
 0.03988
 
 0.79839
 
 Not queried
 
  6.2575302
 
 7.94e-03
 
 1.00e+00
  
 
  
 21
 
  45789122
 
  45789379
 
  PDF   STATS   ENSEMBL   UCSC 
 
  258
 
  4
 
 26414
 
 gene;island
 
 0.09428
 
 0.82133
 
 Not queried
 
  6.2516873
 
 2.86e-02
 
 1.00e+00
  
 
  
 20
 
  29611652
 
  29611810
 
  PDF   STATS   ENSEMBL   UCSC 
 
  159
 
  5
 
 25617
 
 tss
 
 0.01882
 
 0.61314
 
 Not queried
 
  6.2516375
 
 7.94e-03
 
 1.00e+00
  
 
  
 22
 
  28010359
 
  28010598
 
  PDF   STATS   ENSEMBL   UCSC 
 
  240
 
  4
 
 27337
 
 island
 
 0.08407
 
 0.87020
 
 Not queried
 
  6.2426197
 
 2.86e-02
 
 1.00e+00
  
 
  
 19
 
  50979561
 
  50979755
 
  PDF   STATS   ENSEMBL   UCSC 
 
  195
 
  6
 
 20140
 
 gene;island
 
 0.03604
 
 0.81659
 
 Not queried
 
  6.2419596
 
 2.16e-03
 
 1.00e+00
  
 
  
 6
 
  31096861
 
  31097078
 
  PDF   STATS   ENSEMBL   UCSC 
 
  218
 
  5
 
 33790
 
 gene
 
 0.04149
 
 0.76612
 
 Not queried
 
  6.2417876
 
 3.17e-02
 
 1.00e+00
  
 
  
 1
 
 207818395
 
 207818449
 
  PDF   STATS   ENSEMBL   UCSC 
 
   55
 
  4
 
 2220
 
 tss
 
 0.01361
 
 0.47717
 
 Not queried
 
  6.2350930
 
 2.86e-02
 
 1.00e+00
  
 
  
 19
 
  50194120
 
  50194228
 
  PDF   STATS   ENSEMBL   UCSC 
 
  109
 
  4
 
 20125
 
 gene
 
 0.78840
 
 0.05997
 
 Not queried
 
  6.2333243
 
 2.86e-02
 
 1.00e+00
  
 
  
 9
 
    841340
 
    841636
 
  PDF   STATS   ENSEMBL   UCSC 
 
  297
 
  5
 
 40736
 
 tss;island
 
 0.02696
 
 0.70565
 
 Not queried
 
  6.2266603
 
 7.94e-03
 
 1.00e+00
  
 
  
 2
 
 219866437
 
 219866628
 
  PDF   STATS   ENSEMBL   UCSC 
 
  192
 
  5
 
 23850
 
 tss
 
 0.01611
 
 0.54949
 
 Not queried
 
  6.2265816
 
 7.94e-03
 
 1.00e+00
  
 
  
 4
 
  79472605
 
  79472939
 
  PDF   STATS   ENSEMBL   UCSC 
 
  335
 
  5
 
 31075
 
 island
 
 0.82870
 
 0.04403
 
 Not queried
 
  6.2185189
 
 7.94e-03
 
 1.00e+00
  
 
  
 1
 
  12655992
 
  12656315
 
  PDF   STATS   ENSEMBL   UCSC 
 
  324
 
  4
 
 233
 
 gene;island
 
 0.86837
 
 0.06318
 
 Not queried
 
  6.2180990
 
 2.86e-02
 
 1.00e+00
  
 
  
 4
 
  15704599
 
  15704844
 
  PDF   STATS   ENSEMBL   UCSC 
 
  246
 
  4
 
 29914
 
 gene
 
 0.11117
 
 0.90815
 
 Not queried
 
  6.2175827
 
 2.86e-02
 
 1.00e+00
  
 
  
 10
 
 128077307
 
 128077376
 
  PDF   STATS   ENSEMBL   UCSC 
 
   70
 
  6
 
 5415
 
 tss;island
 
 0.83355
 
 0.08434
 
 Not queried
 
  6.2172255
 
 2.16e-03
 
 1.00e+00
  
 
  
 14
 
 104551867
 
 104552034
 
  PDF   STATS   ENSEMBL   UCSC 
 
  168
 
  4
 
 12628
 
 tss
 
 0.17507
 
 0.93144
 
 Not queried
 
  6.2064598
 
 2.86e-02
 
 1.00e+00
  
 
  
 19
 
  49713645
 
  49714044
 
  PDF   STATS   ENSEMBL   UCSC 
 
  400
 
  4
 
 20111
 
 gene;island
 
 0.10181
 
 0.86379
 
 Not queried
 
  6.2062094
 
 2.86e-02
 
 1.00e+00
  
 
  
 19
 
  23653837
 
  23654274
 
  PDF   STATS   ENSEMBL   UCSC 
 
  438
 
  5
 
 21812
 
 island
 
 0.10525
 
 0.89238
 
 Not queried
 
  6.1986200
 
 7.94e-03
 
 1.00e+00
  
 
  
 1
 
  38412519
 
  38412711
 
  PDF   STATS   ENSEMBL   UCSC 
 
  193
 
  4
 
 438
 
 gene
 
 0.67862
 
 0.02911
 
 Not queried
 
  6.1970228
 
 2.86e-02
 
 1.00e+00
  
 
  
 3
 
 129693370
 
 129693791
 
  PDF   STATS   ENSEMBL   UCSC 
 
  422
 
  6
 
 27902
 
 gene;island
 
 0.87825
 
 0.10174
 
 Not queried
 
  6.1949960
 
 2.16e-03
 
 1.00e+00
  
 
  
 X
 
  25033767
 
  25034037
 
  PDF   STATS   ENSEMBL   UCSC 
 
  271
 
  4
 
 40953
 
 gene
 
 0.75015
 
 0.03966
 
 Not queried
 
  6.1931929
 
 2.86e-02
 
 1.00e+00
  
 
  
 19
 
   1467921
 
   1467979
 
  PDF   STATS   ENSEMBL   UCSC 
 
   59
 
  4
 
 19621
 
 gene;island
 
 0.03197
 
 0.74112
 
 Not queried
 
  6.1841954
 
 2.86e-02
 
 1.00e+00
  
 
  
 16
 
  75150744
 
  75150880
 
  PDF   STATS   ENSEMBL   UCSC 
 
  137
 
  6
 
 15328
 
 tss
 
 0.78758
 
 0.06221
 
 Not queried
 
  6.1815815
 
 2.16e-03
 
 1.00e+00
  
 
  
 5
 
 178986131
 
 178986412
 
  PDF   STATS   ENSEMBL   UCSC 
 
  282
 
  4
 
 31873
 
 gene;tss;island
 
 0.49461
 
 0.01871
 
 Not queried
 
  6.1803897
 
 2.86e-02
 
 1.00e+00
  
 
  
 13
 
 110521956
 
 110522297
 
  PDF   STATS   ENSEMBL   UCSC 
 
  342
 
  5
 
 11844
 
 island
 
 0.02726
 
 0.80883
 
 Not queried
 
  6.1736221
 
 7.94e-03
 
 1.00e+00
  
 
  
 4
 
  74847758
 
  74848016
 
  PDF   STATS   ENSEMBL   UCSC 
 
  259
 
  6
 
 30384
 
 tss
 
 0.08150
 
 0.84832
 
 Not queried
 
  6.1731963
 
 2.16e-03
 
 1.00e+00
  
 
  
 11
 
  78673073
 
  78673290
 
  PDF   STATS   ENSEMBL   UCSC 
 
  218
 
  4
 
 6828
 
 gene;island
 
 0.75302
 
 0.03105
 
 Not queried
 
  6.1717408
 
 2.86e-02
 
 1.00e+00
  
 
  
 13
 
  50707427
 
  50708043
 
  PDF   STATS   ENSEMBL   UCSC 
 
  617
 
  5
 
 11715
 
 island
 
 0.74653
 
 0.03943
 
 Not queried
 
  6.1696623
 
 7.94e-03
 
 1.00e+00
  
 
  
 10
 
  94451351
 
  94451736
 
  PDF   STATS   ENSEMBL   UCSC 
 
  386
 
  4
 
 4662
 
 gene;island
 
 0.77371
 
 0.04152
 
 Not queried
 
  6.1678357
 
 2.86e-02
 
 1.00e+00
  
 
  
 12
 
  95942761
 
  95943114
 
  PDF   STATS   ENSEMBL   UCSC 
 
  354
 
  5
 
 9228
 
 gene;tss;island
 
 0.67512
 
 0.06442
 
 Not queried
 
  6.1652987
 
 7.94e-03
 
 1.00e+00
  
 
  
 X
 
  73438299
 
  73438541
 
  PDF   STATS   ENSEMBL   UCSC 
 
  243
 
  4
 
 41036
 
 gene;tss
 
 0.24541
 
 0.91871
 
 Not queried
 
  6.1644691
 
 2.86e-02
 
 1.00e+00
  
 
  
 X
 
 114546190
 
 114546624
 
  PDF   STATS   ENSEMBL   UCSC 
 
  435
 
  4
 
 41971
 
 island
 
 0.81043
 
 0.09166
 
 Not queried
 
  6.1620802
 
 2.86e-02
 
 1.00e+00
  
 
  
 X
 
  77394971
 
  77395543
 
  PDF   STATS   ENSEMBL   UCSC 
 
  573
 
  8
 
 41888
 
 island
 
 0.01955
 
 0.56855
 
 Not queried
 
  6.1615327
 
 3.11e-04
 
 1.00e+00
  
 
  
 6
 
   5085404
 
   5085713
 
  PDF   STATS   ENSEMBL   UCSC 
 
  310
 
  5
 
 34543
 
 tss;island
 
 0.02101
 
 0.60385
 
 Not queried
 
  6.1505303
 
 7.94e-03
 
 1.00e+00
  
 
  
 11
 
 118781633
 
 118781978
 
  PDF   STATS   ENSEMBL   UCSC 
 
  346
 
  7
 
 7719
 
 tss
 
 0.04448
 
 0.81207
 
 Not queried
 
  6.1490780
 
 5.83e-04
 
 1.00e+00
  
 
  
 19
 
  48216390
 
  48216502
 
  PDF   STATS   ENSEMBL   UCSC 
 
  113
 
  5
 
 20872
 
 tss
 
 0.72825
 
 0.02960
 
 Not queried
 
  6.1375774
 
 7.94e-03
 
 1.00e+00
  
 
  
 5
 
 140777344
 
 140777611
 
  PDF   STATS   ENSEMBL   UCSC 
 
  268
 
  6
 
 31772
 
 gene;tss;island
 
 0.02786
 
 0.63231
 
 Not queried
 
  6.1338783
 
 2.16e-03
 
 1.00e+00
  
 
  
 3
 
 184971652
 
 184971844
 
  PDF   STATS   ENSEMBL   UCSC 
 
  193
 
  4
 
 28025
 
 gene;island
 
 0.02075
 
 0.58099
 
 Not queried
 
  6.1305952
 
 2.86e-02
 
 1.00e+00
  
 
  
 8
 
 143859669
 
 143859990
 
  PDF   STATS   ENSEMBL   UCSC 
 
  322
 
  7
 
 39874
 
 tss;island
 
 0.96274
 
 0.26979
 
 Not queried
 
  6.1285337
 
 5.83e-04
 
 1.00e+00
  
 
  
 13
 
  26760422
 
  26760793
 
  PDF   STATS   ENSEMBL   UCSC 
 
  372
 
  4
 
 11588
 
 island
 
 0.01844
 
 0.45006
 
 Not queried
 
  6.1201650
 
 2.86e-02
 
 1.00e+00
  
 
  
 11
 
    504784
 
    504937
 
  PDF   STATS   ENSEMBL   UCSC 
 
  154
 
  5
 
 6358
 
 gene;island
 
 0.17197
 
 0.94748
 
 Not queried
 
  6.1180841
 
 7.94e-03
 
 1.00e+00
  
 
  
 21
 
  42792127
 
  42792516
 
  PDF   STATS   ENSEMBL   UCSC 
 
  390
 
  4
 
 26516
 
 tss
 
 0.00895
 
 0.32052
 
 Not queried
 
  6.1154067
 
 1.14e-01
 
 1.00e+00
  
 
  
 17
 
  19883326
 
  19883716
 
  PDF   STATS   ENSEMBL   UCSC 
 
  391
 
  4
 
 18075
 
 island
 
 0.09622
 
 0.85840
 
 Not queried
 
  6.1111675
 
 2.86e-02
 
 1.00e+00
  
 
  
 2
 
 145147129
 
 145147558
 
  PDF   STATS   ENSEMBL   UCSC 
 
  430
 
  5
 
 22852
 
 gene;island
 
 0.20321
 
 0.92247
 
 Not queried
 
  6.1092156
 
 7.94e-03
 
 1.00e+00
  
 
  
 3
 
 139257713
 
 139257907
 
  PDF   STATS   ENSEMBL   UCSC 
 
  195
 
  5
 
 27927
 
 gene;island
 
 0.90253
 
 0.11829
 
 Not queried
 
  6.1089173
 
 7.94e-03
 
 1.00e+00
  
 
  
 5
 
 115297690
 
 115298079
 
  PDF   STATS   ENSEMBL   UCSC 
 
  390
 
  5
 
 32173
 
 tss;island
 
 0.06065
 
 0.89840
 
 Not queried
 
  6.1086975
 
 7.94e-03
 
 1.00e+00
  
 
  
 11
 
  66824138
 
  66824219
 
  PDF   STATS   ENSEMBL   UCSC 
 
   82
 
  5
 
 7462
 
 tss;island
 
 0.03881
 
 0.87586
 
 Not queried
 
  6.1072328
 
 7.94e-03
 
 1.00e+00
  
 
  
 16
 
  70285806
 
  70285978
 
  PDF   STATS   ENSEMBL   UCSC 
 
  173
 
  5
 
 16032
 
 island
 
 0.01688
 
 0.55139
 
 Not queried
 
  6.0992147
 
 7.94e-03
 
 1.00e+00
  
 
  
 X
 
 154444356
 
 154444619
 
  PDF   STATS   ENSEMBL   UCSC 
 
  264
 
  7
 
 41613
 
 tss
 
 0.01828
 
 0.58242
 
 Not queried
 
  6.0941042
 
 5.83e-04
 
 1.00e+00
  
 
  
 1
 
  28286348
 
  28286411
 
  PDF   STATS   ENSEMBL   UCSC 
 
   64
 
  4
 
 1419
 
 tss;island
 
 0.58884
 
 0.02321
 
 Not queried
 
  6.0936033
 
 2.86e-02
 
 1.00e+00
  
 
  
 16
 
  57831745
 
  57832309
 
  PDF   STATS   ENSEMBL   UCSC 
 
  565
 
  6
 
 14738
 
 gene
 
 0.03555
 
 0.71958
 
 Not queried
 
  6.0928479
 
 2.16e-03
 
 1.00e+00
  
 
  
 Y
 
  21239219
 
  21239607
 
  PDF   STATS   ENSEMBL   UCSC 
 
  389
 
  5
 
 42182
 
 island
 
 0.96934
 
 0.45050
 
 Not queried
 
  6.0815796
 
 7.94e-03
 
 1.00e+00
  
 
  
 16
 
  57832005
 
  57832309
 
  PDF   STATS   ENSEMBL   UCSC 
 
  305
 
  4
 
 15236
 
 tss;island
 
 0.03555
 
 0.71958
 
 Not queried
 
  6.0652007
 
 2.86e-02
 
 1.00e+00
  
 
  
 X
 
  77395258
 
  77395543
 
  PDF   STATS   ENSEMBL   UCSC 
 
  286
 
  6
 
 41403
 
 tss
 
 0.01814
 
 0.56593
 
 Not queried
 
  6.0582249
 
 4.33e-03
 
 1.00e+00
  
 
  
 1
 
  63785867
 
  63786306
 
  PDF   STATS   ENSEMBL   UCSC 
 
  440
 
  5
 
 3334
 
 island
 
 0.80044
 
 0.05076
 
 Not queried
 
  6.0572637
 
 7.94e-03
 
 1.00e+00
  
 
  
 14
 
  67878973
 
  67879166
 
  PDF   STATS   ENSEMBL   UCSC 
 
  194
 
  4
 
 12442
 
 tss;island
 
 0.12088
 
 0.87448
 
 Not queried
 
  6.0494791
 
 2.86e-02
 
 1.00e+00
  
 
  
 X
 
 134232821
 
 134233109
 
  PDF   STATS   ENSEMBL   UCSC 
 
  289
 
  5
 
 41539
 
 tss;island
 
 0.02416
 
 0.65074
 
 Not queried
 
  6.0465400
 
 7.94e-03
 
 1.00e+00
  
 
  
 5
 
 140614369
 
 140614765
 
  PDF   STATS   ENSEMBL   UCSC 
 
  397
 
  4
 
 31758
 
 gene;island
 
 0.18048
 
 0.93208
 
 Not queried
 
  6.0434961
 
 2.86e-02
 
 1.00e+00
  
 
  
 22
 
  18923787
 
  18923986
 
  PDF   STATS   ENSEMBL   UCSC 
 
  200
 
  5
 
 27245
 
 island
 
 0.60396
 
 0.02260
 
 Not queried
 
  6.0434849
 
 7.94e-03
 
 1.00e+00
  
 
  
 16
 
  28634766
 
  28635053
 
  PDF   STATS   ENSEMBL   UCSC 
 
  288
 
  4
 
 15757
 
 island
 
 0.02539
 
 0.66314
 
 Not queried
 
  6.0418379
 
 2.86e-02
 
 1.00e+00
  
 
  
 3
 
 130745442
 
 130745618
 
  PDF   STATS   ENSEMBL   UCSC 
 
  177
 
  7
 
 27907
 
 gene
 
 0.02231
 
 0.59228
 
 Not queried
 
  6.0390107
 
 5.83e-04
 
 1.00e+00
  
 
  
 20
 
  32307885
 
  32308119
 
  PDF   STATS   ENSEMBL   UCSC 
 
  235
 
  4
 
 25356
 
 gene
 
 0.02686
 
 0.62879
 
 Not queried
 
  6.0350287
 
 2.86e-02
 
 1.00e+00
  
 
  
 X
 
  77041722
 
  77042040
 
  PDF   STATS   ENSEMBL   UCSC 
 
  319
 
  7
 
 41399
 
 tss
 
 0.01537
 
 0.49969
 
 Not queried
 
  6.0334779
 
 1.11e-02
 
 1.00e+00
  
 
  
 X
 
  77041452
 
  77042040
 
  PDF   STATS   ENSEMBL   UCSC 
 
  589
 
  9
 
 41883
 
 island
 
 0.01537
 
 0.49969
 
 Not queried
 
  6.0334779
 
 1.85e-03
 
 1.00e+00
  
 
  
 19
 
   3801294
 
   3801727
 
  PDF   STATS   ENSEMBL   UCSC 
 
  434
 
  4
 
 19688
 
 gene
 
 0.04971
 
 0.80067
 
 Not queried
 
  6.0244865
 
 2.86e-02
 
 1.00e+00
  
 
  
 4
 
 111540884
 
 111541339
 
  PDF   STATS   ENSEMBL   UCSC 
 
  456
 
  4
 
 30047
 
 gene;island
 
 0.91897
 
 0.17917
 
 Not queried
 
  6.0231512
 
 2.86e-02
 
 1.00e+00
  
 
  
 16
 
  67907176
 
  67907373
 
  PDF   STATS   ENSEMBL   UCSC 
 
  198
 
  4
 
 14775
 
 gene
 
 0.02196
 
 0.57672
 
 Not queried
 
  6.0169916
 
 2.86e-02
 
 1.00e+00
  
 
  
 12
 
  25102274
 
  25102521
 
  PDF   STATS   ENSEMBL   UCSC 
 
  248
 
  5
 
 10264
 
 island
 
 0.83844
 
 0.02830
 
 Not queried
 
  6.0141674
 
 7.94e-03
 
 1.00e+00
  
 
  
 19
 
  48216390
 
  48216613
 
  PDF   STATS   ENSEMBL   UCSC 
 
  224
 
  6
 
 22165
 
 island
 
 0.67313
 
 0.02855
 
 Not queried
 
  5.9881741
 
 2.16e-03
 
 1.00e+00
  
 
  
 1
 
  68516463
 
  68516713
 
  PDF   STATS   ENSEMBL   UCSC 
 
  251
 
  6
 
 1683
 
 tss
 
 0.05951
 
 0.73903
 
 Not queried
 
  5.9691544
 
 2.16e-03
 
 1.00e+00
  
 
  
 5
 
   2755820
 
   2756113
 
  PDF   STATS   ENSEMBL   UCSC 
 
  294
 
  4
 
 32598
 
 island
 
 0.64216
 
 0.02669
 
 Not queried
 
  5.9684275
 
 2.86e-02
 
 1.00e+00
  
 
  
 1
 
 244624521
 
 244624883
 
  PDF   STATS   ENSEMBL   UCSC 
 
  363
 
  6
 
 4381
 
 island
 
 0.04035
 
 0.66690
 
 Not queried
 
  5.9603643
 
 2.16e-03
 
 1.00e+00
  
 
  
 10
 
  27541298
 
  27541418
 
  PDF   STATS   ENSEMBL   UCSC 
 
  121
 
  4
 
 4984
 
 tss
 
 0.06844
 
 0.82123
 
 Not queried
 
  5.9593178
 
 2.86e-02
 
 1.00e+00
  
 
  
 X
 
 149861526
 
 149861779
 
  PDF   STATS   ENSEMBL   UCSC 
 
  254
 
  7
 
 41566
 
 tss
 
 0.01672
 
 0.54082
 
 Not queried
 
  5.9461379
 
 5.83e-04
 
 1.00e+00
  
 
  
 22
 
  42394590
 
  42394853
 
  PDF   STATS   ENSEMBL   UCSC 
 
  264
 
  4
 
 27495
 
 island
 
 0.14866
 
 0.85934
 
 Not queried
 
  5.9418553
 
 2.86e-02
 
 1.00e+00
  
 
  
 1
 
 247802703
 
 247803166
 
  PDF   STATS   ENSEMBL   UCSC 
 
  464
 
  7
 
 4407
 
 island
 
 0.02625
 
 0.59201
 
 Not queried
 
  5.9326978
 
 5.83e-04
 
 1.00e+00
  
 
  
 11
 
  64739253
 
  64739484
 
  PDF   STATS   ENSEMBL   UCSC 
 
  232
 
  6
 
 8409
 
 island
 
 0.05017
 
 0.78044
 
 Not queried
 
  5.9304588
 
 2.16e-03
 
 1.00e+00
  
 
  
 6
 
 169653905
 
 169654035
 
  PDF   STATS   ENSEMBL   UCSC 
 
  131
 
  7
 
 34502
 
 gene
 
 0.89263
 
 0.03694
 
 Not queried
 
  5.9288937
 
 5.83e-04
 
 1.00e+00
  
 
  
 19
 
  12075882
 
  12076348
 
  PDF   STATS   ENSEMBL   UCSC 
 
  467
 
  6
 
 19815
 
 gene
 
 0.03524
 
 0.70038
 
 Not queried
 
  5.9260211
 
 2.16e-03
 
 1.00e+00
  
 
  
 17
 
     31528
 
     31552
 
  PDF   STATS   ENSEMBL   UCSC 
 
   25
 
  5
 
 16944
 
 tss;island
 
 0.03373
 
 0.66559
 
 Not queried
 
  5.9245378
 
 7.94e-03
 
 1.00e+00
  
 
  
 X
 
 153626255
 
 153626527
 
  PDF   STATS   ENSEMBL   UCSC 
 
  273
 
  7
 
 41596
 
 tss
 
 0.01956
 
 0.57489
 
 Not queried
 
  5.9184173
 
 5.83e-04
 
 1.00e+00
  
 
  
 X
 
  25034093
 
  25034241
 
  PDF   STATS   ENSEMBL   UCSC 
 
  149
 
  5
 
 41235
 
 tss
 
 0.55025
 
 0.01895
 
 Not queried
 
  5.9134430
 
 7.94e-03
 
 1.00e+00
  
 
  
 1
 
 207818395
 
 207818493
 
  PDF   STATS   ENSEMBL   UCSC 
 
   99
 
  5
 
 4141
 
 island
 
 0.01508
 
 0.47950
 
 Not queried
 
  5.9110485
 
 7.94e-03
 
 1.00e+00
  
 
  
 3
 
  49236800
 
  49236918
 
  PDF   STATS   ENSEMBL   UCSC 
 
  119
 
  5
 
 28282
 
 tss
 
 0.11693
 
 0.88644
 
 Not queried
 
  5.9033722
 
 7.94e-03
 
 1.00e+00
  
 
  
 1
 
 146543992
 
 146544267
 
  PDF   STATS   ENSEMBL   UCSC 
 
  276
 
  4
 
 3679
 
 island
 
 0.60622
 
 0.02707
 
 Not queried
 
  5.8933504
 
 2.86e-02
 
 1.00e+00
  
 
  
 16
 
  80574553
 
  80575018
 
  PDF   STATS   ENSEMBL   UCSC 
 
  466
 
  6
 
 16083
 
 island
 
 0.00881
 
 0.42182
 
 Not queried
 
  5.8902527
 
 2.16e-03
 
 1.00e+00
  
 
  
 7
 
  94023308
 
  94023869
 
  PDF   STATS   ENSEMBL   UCSC 
 
  562
 
  9
 
 37653
 
 tss
 
 0.03720
 
 0.73981
 
 Not queried
 
  5.8901984
 
 2.76e-03
 
 1.00e+00
  
 
  
 7
 
 158789723
 
 158790115
 
  PDF   STATS   ENSEMBL   UCSC 
 
  393
 
  4
 
 39095
 
 island
 
 0.07696
 
 0.84161
 
 Not queried
 
  5.8833509
 
 2.86e-02
 
 1.00e+00
  
 
  
 1
 
 171220575
 
 171220819
 
  PDF   STATS   ENSEMBL   UCSC 
 
  245
 
  4
 
 882
 
 gene
 
 0.71638
 
 0.06310
 
 Not queried
 
  5.8775005
 
 2.86e-02
 
 1.00e+00
  
 
  
 X
 
 153060150
 
 153060546
 
  PDF   STATS   ENSEMBL   UCSC 
 
  397
 
  5
 
 41148
 
 gene
 
 0.02271
 
 0.57701
 
 Not queried
 
  5.8755001
 
 7.94e-03
 
 1.00e+00
  
 
  
 1
 
  15930094
 
  15930386
 
  PDF   STATS   ENSEMBL   UCSC 
 
  293
 
  4
 
 2717
 
 island
 
 0.01698
 
 0.44524
 
 Not queried
 
  5.8714586
 
 2.86e-02
 
 1.00e+00
  
 
  
 5
 
 140552347
 
 140552600
 
  PDF   STATS   ENSEMBL   UCSC 
 
  254
 
  4
 
 31754
 
 gene
 
 0.23944
 
 0.91761
 
 Not queried
 
  5.8690958
 
 2.86e-02
 
 1.00e+00
  
 
  
 11
 
  34535357
 
  34535579
 
  PDF   STATS   ENSEMBL   UCSC 
 
  223
 
  7
 
 7222
 
 tss
 
 0.11876
 
 0.93865
 
 Not queried
 
  5.8644469
 
 5.83e-04
 
 1.00e+00
  
 
  
 11
 
 125757328
 
 125757753
 
  PDF   STATS   ENSEMBL   UCSC 
 
  426
 
  7
 
 6940
 
 gene;island
 
 0.02372
 
 0.55294
 
 Not queried
 
  5.8614356
 
 5.83e-04
 
 1.00e+00
  
 
  
 5
 
  43396802
 
  43397288
 
  PDF   STATS   ENSEMBL   UCSC 
 
  487
 
  7
 
 31547
 
 gene;island
 
 0.01005
 
 0.49202
 
 Not queried
 
  5.8612133
 
 5.83e-04
 
 1.00e+00
  
 
  
 X
 
 150565243
 
 150565466
 
  PDF   STATS   ENSEMBL   UCSC 
 
  224
 
  4
 
 41569
 
 tss;island
 
 0.02493
 
 0.55674
 
 Not queried
 
  5.8583657
 
 2.86e-02
 
 1.00e+00
  
 
  
 15
 
  93652578
 
  93653073
 
  PDF   STATS   ENSEMBL   UCSC 
 
  496
 
  6
 
 14454
 
 island
 
 0.24191
 
 0.84751
 
 Not queried
 
  5.8499573
 
 4.33e-03
 
 1.00e+00
  
 
  
 8
 
 105479058
 
 105479420
 
  PDF   STATS   ENSEMBL   UCSC 
 
  363
 
  7
 
 40459
 
 island
 
 0.04915
 
 0.88359
 
 Not queried
 
  5.8469383
 
 5.83e-04
 
 1.00e+00
  
 
  
 3
 
 194117018
 
 194117403
 
  PDF   STATS   ENSEMBL   UCSC 
 
  386
 
  4
 
 28047
 
 gene;island
 
 0.03917
 
 0.69287
 
 Not queried
 
  5.8453870
 
 2.00e-01
 
 1.00e+00
  
 
  
 22
 
  22901830
 
  22902237
 
  PDF   STATS   ENSEMBL   UCSC 
 
  408
 
  6
 
 26768
 
 gene
 
 0.05465
 
 0.76276
 
 Not queried
 
  5.8424365
 
 2.16e-03
 
 1.00e+00
  
 
  
 19
 
   4831885
 
   4832024
 
  PDF   STATS   ENSEMBL   UCSC 
 
  140
 
  5
 
 20337
 
 tss
 
 0.06890
 
 0.82429
 
 Not queried
 
  5.8391047
 
 7.94e-03
 
 1.00e+00
  
 
  
 19
 
   3801294
 
   3801922
 
  PDF   STATS   ENSEMBL   UCSC 
 
  629
 
  7
 
 21312
 
 island
 
 0.06036
 
 0.76262
 
 Not queried
 
  5.8382116
 
 5.83e-04
 
 1.00e+00
  
 
  
 1
 
 155579369
 
 155579964
 
  PDF   STATS   ENSEMBL   UCSC 
 
  596
 
  9
 
 1997
 
 tss;island
 
 0.77826
 
 0.04673
 
 Not queried
 
  5.8284397
 
 2.76e-03
 
 1.00e+00
  
 
  
 8
 
  33457483
 
  33457822
 
  PDF   STATS   ENSEMBL   UCSC 
 
  340
 
  6
 
 39578
 
 tss
 
 0.04862
 
 0.84913
 
 Not queried
 
  5.8207578
 
 4.33e-03
 
 1.00e+00
  
 
  
 4
 
 113970617
 
 113970745
 
  PDF   STATS   ENSEMBL   UCSC 
 
  129
 
  4
 
 30507
 
 tss
 
 0.72974
 
 0.06165
 
 Not queried
 
  5.8169878
 
 2.86e-02
 
 1.00e+00
  
 
  
 X
 
 144903565
 
 144903748
 
  PDF   STATS   ENSEMBL   UCSC 
 
  184
 
  4
 
 41132
 
 gene;island
 
 0.08342
 
 0.85653
 
 Not queried
 
  5.8128247
 
 2.86e-02
 
 1.00e+00
  
 
  
 1
 
 228651708
 
 228652067
 
  PDF   STATS   ENSEMBL   UCSC 
 
  360
 
  4
 
 4288
 
 island
 
 0.02608
 
 0.56200
 
 Not queried
 
  5.8105114
 
 2.86e-02
 
 1.00e+00
  
 
  
 1
 
 152161237
 
 152161683
 
  PDF   STATS   ENSEMBL   UCSC 
 
  447
 
  4
 
 3749
 
 island
 
 0.02853
 
 0.69180
 
 Not queried
 
  5.8102465
 
 1.14e-01
 
 1.00e+00
  
 
  
 8
 
  41733475
 
  41733931
 
  PDF   STATS   ENSEMBL   UCSC 
 
  457
 
  5
 
 39232
 
 gene;island
 
 0.09504
 
 0.79989
 
 Not queried
 
  5.8048262
 
 7.94e-03
 
 1.00e+00
  
 
  
 X
 
  24691042
 
  24691232
 
  PDF   STATS   ENSEMBL   UCSC 
 
  191
 
  6
 
 41233
 
 tss
 
 0.13248
 
 0.90735
 
 Not queried
 
  5.8036094
 
 4.33e-03
 
 1.00e+00
  
 
  
 19
 
  10698020
 
  10698098
 
  PDF   STATS   ENSEMBL   UCSC 
 
   79
 
  4
 
 20417
 
 tss
 
 0.02326
 
 0.53073
 
 Not queried
 
  5.8007148
 
 2.86e-02
 
 1.00e+00
  
 
  
 X
 
 125300035
 
 125300111
 
  PDF   STATS   ENSEMBL   UCSC 
 
   77
 
  5
 
 41506
 
 tss;island
 
 0.01976
 
 0.51858
 
 Not queried
 
  5.7972017
 
 7.94e-03
 
 1.00e+00
  
 
  
 5
 
 140800398
 
 140800495
 
  PDF   STATS   ENSEMBL   UCSC 
 
   98
 
  4
 
 32323
 
 tss
 
 0.08682
 
 0.88669
 
 Not queried
 
  5.7855725
 
 2.86e-02
 
 1.00e+00
  
 
  
 X
 
 144902897
 
 144903125
 
  PDF   STATS   ENSEMBL   UCSC 
 
  229
 
  4
 
 41131
 
 gene;island
 
 0.05102
 
 0.77755
 
 Not queried
 
  5.7835537
 
 2.86e-02
 
 1.00e+00
  
 
  
 16
 
  31146999
 
  31147054
 
  PDF   STATS   ENSEMBL   UCSC 
 
   56
 
  4
 
 14702
 
 gene
 
 0.02117
 
 0.57553
 
 Not queried
 
  5.7792157
 
 2.86e-02
 
 1.00e+00
  
 
  
 10
 
  49674251
 
  49674530
 
  PDF   STATS   ENSEMBL   UCSC 
 
  280
 
  4
 
 4566
 
 gene;island
 
 0.22414
 
 0.92817
 
 Not queried
 
  5.7782336
 
 2.86e-02
 
 1.00e+00
  
 
  
 6
 
 119669112
 
 119669372
 
  PDF   STATS   ENSEMBL   UCSC 
 
  261
 
  4
 
 34394
 
 gene;island
 
 0.60723
 
 0.02803
 
 Not queried
 
  5.7752849
 
 2.86e-02
 
 1.00e+00
  
 
  
 21
 
  36041334
 
  36041683
 
  PDF   STATS   ENSEMBL   UCSC 
 
  350
 
  7
 
 26490
 
 tss
 
 0.66612
 
 0.03003
 
 Not queried
 
  5.7624057
 
 5.83e-04
 
 1.00e+00
  
 
  
 2
 
 131010423
 
 131010871
 
  PDF   STATS   ENSEMBL   UCSC 
 
  449
 
  5
 
 24679
 
 island
 
 0.05326
 
 0.70387
 
 Not queried
 
  5.7503589
 
 7.94e-03
 
 1.00e+00
  
 
  
 14
 
  75593334
 
  75593738
 
  PDF   STATS   ENSEMBL   UCSC 
 
  405
 
  5
 
 12116
 
 gene
 
 0.60249
 
 0.02742
 
 Not queried
 
  5.7483238
 
 7.94e-03
 
 1.00e+00
  
 
  
 16
 
  68481049
 
  68481543
 
  PDF   STATS   ENSEMBL   UCSC 
 
  495
 
  6
 
 14785
 
 gene;island
 
 0.06177
 
 0.75884
 
 Not queried
 
  5.7421203
 
 2.16e-03
 
 1.00e+00
  
 
  
 12
 
  30948636
 
  30948794
 
  PDF   STATS   ENSEMBL   UCSC 
 
  159
 
  5
 
 10282
 
 island
 
 0.87502
 
 0.11301
 
 Not queried
 
  5.7420496
 
 7.94e-03
 
 1.00e+00
  
 
  
 12
 
  51236582
 
  51236693
 
  PDF   STATS   ENSEMBL   UCSC 
 
  112
 
  4
 
 9683
 
 tss
 
 0.11415
 
 0.91108
 
 Not queried
 
  5.7414250
 
 2.86e-02
 
 1.00e+00
  
 
  
 X
 
 135579428
 
 135579487
 
  PDF   STATS   ENSEMBL   UCSC 
 
   60
 
  5
 
 41119
 
 gene;tss;island
 
 0.01687
 
 0.43278
 
 Not queried
 
  5.7383453
 
 7.94e-03
 
 1.00e+00
  
 
  
 X
 
 152939154
 
 152939615
 
  PDF   STATS   ENSEMBL   UCSC 
 
  462
 
  4
 
 41143
 
 gene;tss;island
 
 0.15826
 
 0.94389
 
 Not queried
 
  5.7368479
 
 1.14e-01
 
 1.00e+00
  
 
  
 6
 
  33043762
 
  33044012
 
  PDF   STATS   ENSEMBL   UCSC 
 
  251
 
  6
 
 34120
 
 gene
 
 0.72126
 
 0.04786
 
 Not queried
 
  5.7317292
 
 2.16e-03
 
 1.00e+00
  
 
  
 8
 
  99959430
 
  99959723
 
  PDF   STATS   ENSEMBL   UCSC 
 
  294
 
  4
 
 39311
 
 gene;island
 
 0.85236
 
 0.04641
 
 Not queried
 
  5.7204158
 
 2.86e-02
 
 1.00e+00
  
 
  
 5
 
  68628240
 
  68628738
 
  PDF   STATS   ENSEMBL   UCSC 
 
  499
 
  5
 
 32788
 
 island
 
 0.02457
 
 0.60054
 
 Not queried
 
  5.7201363
 
 7.94e-03
 
 1.00e+00
  
 
  
 20
 
  47896769
 
  47897451
 
  PDF   STATS   ENSEMBL   UCSC 
 
  683
 
  7
 
 25409
 
 gene;island
 
 0.88857
 
 0.15732
 
 Not queried
 
  5.7172119
 
 5.83e-04
 
 1.00e+00
  
 
  
 20
 
  47896769
 
  47897207
 
  PDF   STATS   ENSEMBL   UCSC 
 
  439
 
  5
 
 25768
 
 tss
 
 0.88857
 
 0.15732
 
 Not queried
 
  5.7172119
 
 7.94e-03
 
 1.00e+00
  
 
  
 8
 
  97171668
 
  97172012
 
  PDF   STATS   ENSEMBL   UCSC 
 
  345
 
  4
 
 39304
 
 gene;island
 
 0.12110
 
 0.76955
 
 Not queried
 
  5.7120611
 
 2.86e-02
 
 1.00e+00
  
 
  
 20
 
  32308170
 
  32308529
 
  PDF   STATS   ENSEMBL   UCSC 
 
  360
 
  7
 
 25646
 
 tss
 
 0.02402
 
 0.57988
 
 Not queried
 
  5.7120271
 
 5.83e-04
 
 1.00e+00
  
 
  
 5
 
 140749735
 
 140749834
 
  PDF   STATS   ENSEMBL   UCSC 
 
  100
 
  4
 
 31767
 
 gene;tss;island
 
 0.05757
 
 0.70866
 
 Not queried
 
  5.7015641
 
 2.86e-02
 
 1.00e+00
  
 
  
 4
 
  26030463
 
  26030691
 
  PDF   STATS   ENSEMBL   UCSC 
 
  229
 
  4
 
 30914
 
 island
 
 0.03189
 
 0.60315
 
 Not queried
 
  5.6980184
 
 2.86e-02
 
 1.00e+00
  
 
  
 1
 
  46859671
 
  46859845
 
  PDF   STATS   ENSEMBL   UCSC 
 
  175
 
  6
 
 1597
 
 tss;island
 
 0.93230
 
 0.27915
 
 Not queried
 
  5.6963366
 
 2.60e-02
 
 1.00e+00
  
 
  
 6
 
 157876915
 
 157877218
 
  PDF   STATS   ENSEMBL   UCSC 
 
  304
 
  5
 
 34458
 
 gene
 
 0.04447
 
 0.75577
 
 Not queried
 
  5.6941268
 
 1.59e-02
 
 1.00e+00
  
 
  
 1
 
  87617142
 
  87617350
 
  PDF   STATS   ENSEMBL   UCSC 
 
  209
 
  4
 
 612
 
 gene;island
 
 0.77020
 
 0.06188
 
 Not queried
 
  5.6938701
 
 2.00e-01
 
 1.00e+00
  
 
  
 8
 
 105479318
 
 105479420
 
  PDF   STATS   ENSEMBL   UCSC 
 
  103
 
  4
 
 39797
 
 tss
 
 0.12715
 
 0.89580
 
 Not queried
 
  5.6883648
 
 2.86e-02
 
 1.00e+00
  
 
  
 11
 
  65816463
 
  65816646
 
  PDF   STATS   ENSEMBL   UCSC 
 
  184
 
  4
 
 6705
 
 gene
 
 0.75030
 
 0.04526
 
 Not queried
 
  5.6881365
 
 2.86e-02
 
 1.00e+00
  
 
  
 2
 
  63284481
 
  63284784
 
  PDF   STATS   ENSEMBL   UCSC 
 
  304
 
  5
 
 24303
 
 island
 
 0.95404
 
 0.23909
 
 Not queried
 
  5.6843663
 
 7.94e-03
 
 1.00e+00
  
 
  
 17
 
   7287652
 
   7288001
 
  PDF   STATS   ENSEMBL   UCSC 
 
  350
 
  4
 
 16357
 
 gene;island
 
 0.87216
 
 0.13268
 
 Not queried
 
  5.6841582
 
 2.86e-02
 
 1.00e+00
  
 
  
 5
 
    524679
 
    524941
 
  PDF   STATS   ENSEMBL   UCSC 
 
  263
 
  4
 
 31901
 
 tss
 
 0.03871
 
 0.68803
 
 Not queried
 
  5.6824314
 
 2.86e-02
 
 1.00e+00
  
 
  
 5
 
 167956306
 
 167956425
 
  PDF   STATS   ENSEMBL   UCSC 
 
  120
 
  4
 
 32419
 
 tss
 
 0.02433
 
 0.51330
 
 Not queried
 
  5.6816156
 
 2.86e-02
 
 1.00e+00
  
 
  
 22
 
  18923876
 
  18923986
 
  PDF   STATS   ENSEMBL   UCSC 
 
  111
 
  4
 
 26910
 
 tss
 
 0.62437
 
 0.02772
 
 Not queried
 
  5.6808046
 
 2.86e-02
 
 1.00e+00
  
 
  
 5
 
 100239919
 
 100240223
 
  PDF   STATS   ENSEMBL   UCSC 
 
  305
 
  4
 
 32138
 
 tss;island
 
 0.67800
 
 0.03512
 
 Not queried
 
  5.6730479
 
 2.86e-02
 
 1.00e+00
  
 
  
 4
 
 113739249
 
 113739379
 
  PDF   STATS   ENSEMBL   UCSC 
 
  131
 
  5
 
 30055
 
 gene
 
 0.70384
 
 0.02297
 
 Not queried
 
  5.6655185
 
 7.94e-03
 
 1.00e+00
  
 
  
 19
 
  50191342
 
  50191683
 
  PDF   STATS   ENSEMBL   UCSC 
 
  342
 
  5
 
 20124
 
 gene
 
 0.12211
 
 0.89265
 
 Not queried
 
  5.6649398
 
 7.94e-03
 
 1.00e+00
  
 
  
 8
 
  82543035
 
  82543431
 
  PDF   STATS   ENSEMBL   UCSC 
 
  397
 
  5
 
 40354
 
 island
 
 0.10437
 
 0.72090
 
 Not queried
 
  5.6606273
 
 7.94e-03
 
 1.00e+00
  
 
  
 7
 
  57484635
 
  57484821
 
  PDF   STATS   ENSEMBL   UCSC 
 
  187
 
  4
 
 38455
 
 island
 
 0.05658
 
 0.72984
 
 Not queried
 
  5.6600404
 
 2.86e-02
 
 1.00e+00
  
 
  
 5
 
 140536175
 
 140536496
 
  PDF   STATS   ENSEMBL   UCSC 
 
  322
 
  4
 
 31753
 
 gene;island
 
 0.04401
 
 0.77251
 
 Not queried
 
  5.6586547
 
 2.86e-02
 
 1.00e+00
  
 
  
 6
 
  32977138
 
  32977349
 
  PDF   STATS   ENSEMBL   UCSC 
 
  212
 
  5
 
 34114
 
 gene
 
 0.73315
 
 0.14884
 
 Not queried
 
  5.6574616
 
 1.59e-02
 
 1.00e+00
  
 
  
 18
 
  42643730
 
  42643936
 
  PDF   STATS   ENSEMBL   UCSC 
 
  207
 
  5
 
 19064
 
 gene;island
 
 0.22393
 
 0.91121
 
 Not queried
 
  5.6564371
 
 7.94e-03
 
 1.00e+00
  
 
  
 1
 
 208132388
 
 208132787
 
  PDF   STATS   ENSEMBL   UCSC 
 
  400
 
  4
 
 4145
 
 island
 
 0.55861
 
 0.03149
 
 Not queried
 
  5.6521826
 
 2.86e-02
 
 1.00e+00
  
 
  
 7
 
   1609793
 
   1609908
 
  PDF   STATS   ENSEMBL   UCSC 
 
  116
 
  7
 
 36702
 
 gene;tss;island
 
 0.02857
 
 0.63603
 
 Not queried
 
  5.6502425
 
 5.83e-04
 
 1.00e+00
  
 
  
 18
 
  32173084
 
  32173227
 
  PDF   STATS   ENSEMBL   UCSC 
 
  144
 
  4
 
 19052
 
 gene;tss
 
 0.13720
 
 0.88435
 
 Not queried
 
  5.6491914
 
 2.86e-02
 
 1.00e+00
  
 
  
 6
 
  27114864
 
  27115249
 
  PDF   STATS   ENSEMBL   UCSC 
 
  386
 
  5
 
 34649
 
 tss;island
 
 0.02662
 
 0.66394
 
 Not queried
 
  5.6486599
 
 7.94e-03
 
 1.00e+00
  
 
  
 19
 
  10697913
 
  10698098
 
  PDF   STATS   ENSEMBL   UCSC 
 
  186
 
  5
 
 21504
 
 island
 
 0.02797
 
 0.51433
 
 Not queried
 
  5.6452078
 
 7.94e-03
 
 1.00e+00
  
 
  
 5
 
  42995123
 
  42995527
 
  PDF   STATS   ENSEMBL   UCSC 
 
  405
 
  5
 
 32701
 
 island
 
 0.69890
 
 0.07248
 
 Not queried
 
  5.6447530
 
 7.94e-03
 
 1.00e+00
  
 
  
 X
 
  70752639
 
  70752883
 
  PDF   STATS   ENSEMBL   UCSC 
 
  245
 
  7
 
 41378
 
 tss
 
 0.01412
 
 0.40644
 
 Not queried
 
  5.6409556
 
 5.83e-04
 
 1.00e+00
  
 
  
 5
 
 140535215
 
 140535810
 
  PDF   STATS   ENSEMBL   UCSC 
 
  596
 
  6
 
 33153
 
 island
 
 0.01953
 
 0.43143
 
 Not queried
 
  5.6389308
 
 2.16e-03
 
 1.00e+00
  
 
  
 17
 
   4802692
 
   4802906
 
  PDF   STATS   ENSEMBL   UCSC 
 
  215
 
  6
 
 17000
 
 tss
 
 0.02428
 
 0.58387
 
 Not queried
 
  5.6385261
 
 2.16e-03
 
 1.00e+00
  
 
  
 5
 
 140753415
 
 140753570
 
  PDF   STATS   ENSEMBL   UCSC 
 
  156
 
  7
 
 32318
 
 tss
 
 0.03385
 
 0.66696
 
 Not queried
 
  5.6308349
 
 1.17e-03
 
 1.00e+00
  
 
  
 17
 
  15686294
 
  15686473
 
  PDF   STATS   ENSEMBL   UCSC 
 
  180
 
  4
 
 18010
 
 island
 
 0.26759
 
 0.94356
 
 Not queried
 
  5.6268676
 
 2.86e-02
 
 1.00e+00
  
 
  
 4
 
  46995637
 
  46995821
 
  PDF   STATS   ENSEMBL   UCSC 
 
  185
 
  5
 
 30328
 
 tss
 
 0.01761
 
 0.47590
 
 Not queried
 
  5.6221169
 
 7.94e-03
 
 1.00e+00
  
 
  
 19
 
  48947560
 
  48947631
 
  PDF   STATS   ENSEMBL   UCSC 
 
   72
 
  4
 
 20088
 
 gene;tss;island
 
 0.09967
 
 0.78671
 
 Not queried
 
  5.6183564
 
 2.86e-02
 
 1.00e+00
  
 
  
 8
 
  65284438
 
  65284519
 
  PDF   STATS   ENSEMBL   UCSC 
 
   82
 
  4
 
 40275
 
 island
 
 0.82342
 
 0.08892
 
 Not queried
 
  5.6176835
 
 2.86e-02
 
 1.00e+00
  
 
  
 5
 
    524503
 
    524941
 
  PDF   STATS   ENSEMBL   UCSC 
 
  439
 
  5
 
 32527
 
 island
 
 0.04405
 
 0.68382
 
 Not queried
 
  5.6092619
 
 7.94e-03
 
 1.00e+00
  
 
  
 Y
 
  21239332
 
  21239607
 
  PDF   STATS   ENSEMBL   UCSC 
 
  276
 
  4
 
 42170
 
 tss
 
 0.96958
 
 0.46444
 
 Not queried
 
  5.6065483
 
 2.86e-02
 
 1.00e+00
  
 
  
 6
 
 111580510
 
 111580939
 
  PDF   STATS   ENSEMBL   UCSC 
 
  430
 
  6
 
 34380
 
 gene
 
 0.40420
 
 0.01636
 
 Not queried
 
  5.6030207
 
 2.16e-03
 
 1.00e+00
  
 
  
 X
 
 153672702
 
 153672898
 
  PDF   STATS   ENSEMBL   UCSC 
 
  197
 
  4
 
 41161
 
 gene;island
 
 0.01841
 
 0.50491
 
 Not queried
 
  5.5988512
 
 2.86e-02
 
 1.00e+00
  
 
  
 17
 
  16593426
 
  16593613
 
  PDF   STATS   ENSEMBL   UCSC 
 
  188
 
  6
 
 17128
 
 tss;island
 
 0.06719
 
 0.84711
 
 Not queried
 
  5.5977129
 
 4.33e-03
 
 1.00e+00
  
 
  
 5
 
  20305576
 
  20305935
 
  PDF   STATS   ENSEMBL   UCSC 
 
  360
 
  4
 
 32648
 
 island
 
 0.10775
 
 0.87741
 
 Not queried
 
  5.5969618
 
 2.86e-02
 
 1.00e+00
  
 
  
 1
 
   2986362
 
   2986566
 
  PDF   STATS   ENSEMBL   UCSC 
 
  205
 
  4
 
 96
 
 gene;island
 
 0.03018
 
 0.60900
 
 Not queried
 
  5.5907331
 
 2.86e-02
 
 1.00e+00
  
 
  
 3
 
  42306467
 
  42306974
 
  PDF   STATS   ENSEMBL   UCSC 
 
  508
 
  7
 
 28200
 
 tss
 
 0.07905
 
 0.75688
 
 Not queried
 
  5.5846630
 
 5.83e-04
 
 1.00e+00
  
 
  
 9
 
 137392877
 
 137393288
 
  PDF   STATS   ENSEMBL   UCSC 
 
  412
 
  5
 
 40905
 
 island
 
 0.23018
 
 0.90683
 
 Not queried
 
  5.5823613
 
 7.94e-03
 
 1.00e+00
  
 
  
 6
 
  49681299
 
  49681391
 
  PDF   STATS   ENSEMBL   UCSC 
 
   93
 
  5
 
 34963
 
 tss
 
 0.01624
 
 0.44944
 
 Not queried
 
  5.5822920
 
 7.94e-03
 
 1.00e+00
  
 
  
 6
 
  27114943
 
  27115249
 
  PDF   STATS   ENSEMBL   UCSC 
 
  307
 
  4
 
 33554
 
 gene
 
 0.03402
 
 0.62118
 
 Not queried
 
  5.5819038
 
 2.86e-02
 
 1.00e+00
  
 
  
 6
 
 168435914
 
 168436099
 
  PDF   STATS   ENSEMBL   UCSC 
 
  186
 
  4
 
 34493
 
 gene;island
 
 0.10466
 
 0.75112
 
 Not queried
 
  5.5798414
 
 2.86e-02
 
 1.00e+00
  
 
  
 6
 
 161100017
 
 161100672
 
  PDF   STATS   ENSEMBL   UCSC 
 
  656
 
  6
 
 36552
 
 island
 
 0.11688
 
 0.89567
 
 Not queried
 
  5.5748352
 
 2.16e-03
 
 1.00e+00
  
 
  
 12
 
 103344336
 
 103344685
 
  PDF   STATS   ENSEMBL   UCSC 
 
  350
 
  5
 
 10700
 
 island
 
 0.07534
 
 0.73371
 
 Not queried
 
  5.5692892
 
 7.94e-03
 
 1.00e+00
  
 
  
 X
 
 125686801
 
 125687207
 
  PDF   STATS   ENSEMBL   UCSC 
 
  407
 
  8
 
 42013
 
 island
 
 0.12927
 
 0.91288
 
 Not queried
 
  5.5640601
 
 3.11e-04
 
 1.00e+00
  
 
  
 2
 
   2193511
 
   2193665
 
  PDF   STATS   ENSEMBL   UCSC 
 
  155
 
  4
 
 22505
 
 gene;island
 
 0.10342
 
 0.82006
 
 Not queried
 
  5.5591397
 
 2.86e-02
 
 1.00e+00
  
 
  
 10
 
 101874829
 
 101875138
 
  PDF   STATS   ENSEMBL   UCSC 
 
  310
 
  5
 
 5999
 
 island
 
 0.27193
 
 0.94399
 
 Not queried
 
  5.5560432
 
 7.94e-03
 
 1.00e+00
  
 
  
 19
 
  41106155
 
  41106351
 
  PDF   STATS   ENSEMBL   UCSC 
 
  197
 
  4
 
 20011
 
 gene;tss;island
 
 0.03612
 
 0.68406
 
 Not queried
 
  5.5527316
 
 2.86e-02
 
 1.00e+00
  
 
  
 10
 
  95361132
 
  95361386
 
  PDF   STATS   ENSEMBL   UCSC 
 
  255
 
  5
 
 5222
 
 tss;island
 
 0.13955
 
 0.82050
 
 Not queried
 
  5.5521359
 
 7.94e-03
 
 1.00e+00
  
 
  
 2
 
 154336045
 
 154336401
 
  PDF   STATS   ENSEMBL   UCSC 
 
  357
 
  5
 
 23631
 
 tss;island
 
 0.50764
 
 0.01701
 
 Not queried
 
  5.5508735
 
 7.94e-03
 
 1.00e+00
  
 
  
 5
 
 151066268
 
 151066486
 
  PDF   STATS   ENSEMBL   UCSC 
 
  219
 
  4
 
 31809
 
 gene
 
 0.01575
 
 0.42653
 
 Not queried
 
  5.5496576
 
 2.86e-02
 
 1.00e+00
  
 
  
 1
 
 240160972
 
 240161447
 
  PDF   STATS   ENSEMBL   UCSC 
 
  476
 
  5
 
 4359
 
 island
 
 0.02548
 
 0.54339
 
 Not queried
 
  5.5481437
 
 7.94e-03
 
 1.00e+00
  
 
  
 19
 
   4304933
 
   4305239
 
  PDF   STATS   ENSEMBL   UCSC 
 
  307
 
  4
 
 19695
 
 gene;island
 
 0.92437
 
 0.08067
 
 Not queried
 
  5.5473867
 
 1.14e-01
 
 1.00e+00
  
 
  
 8
 
  82192519
 
  82192777
 
  PDF   STATS   ENSEMBL   UCSC 
 
  259
 
  7
 
 39720
 
 tss
 
 0.05601
 
 0.83799
 
 Not queried
 
  5.5457075
 
 5.83e-04
 
 1.00e+00
  
 
  
 X
 
  17755642
 
  17755839
 
  PDF   STATS   ENSEMBL   UCSC 
 
  198
 
  4
 
 40945
 
 gene
 
 0.31724
 
 0.01331
 
 Not queried
 
  5.5448083
 
 2.86e-02
 
 1.00e+00
  
 
  
 8
 
  22960461
 
  22960619
 
  PDF   STATS   ENSEMBL   UCSC 
 
  159
 
  4
 
 39176
 
 gene
 
 0.02882
 
 0.56932
 
 Not queried
 
  5.5435441
 
 2.86e-02
 
 1.00e+00
  
 
  
 4
 
 190862026
 
 190862268
 
  PDF   STATS   ENSEMBL   UCSC 
 
  243
 
  4
 
 30173
 
 gene
 
 0.01871
 
 0.41459
 
 Not queried
 
  5.5393238
 
 2.86e-02
 
 1.00e+00
  
 
  
 10
 
   2815004
 
   2815547
 
  PDF   STATS   ENSEMBL   UCSC 
 
  544
 
  6
 
 5484
 
 island
 
 0.02801
 
 0.56235
 
 Not queried
 
  5.5376478
 
 2.16e-03
 
 1.00e+00
  
 
  
 15
 
  26874098
 
  26874515
 
  PDF   STATS   ENSEMBL   UCSC 
 
  418
 
  4
 
 13232
 
 gene;island
 
 0.23718
 
 0.93045
 
 Not queried
 
  5.5372324
 
 2.86e-02
 
 1.00e+00
  
 
  
 17
 
   7791882
 
   7792134
 
  PDF   STATS   ENSEMBL   UCSC 
 
  253
 
  5
 
 17069
 
 tss
 
 0.06522
 
 0.87052
 
 Not queried
 
  5.5361322
 
 7.94e-03
 
 1.00e+00
  
 
  
 4
 
   8271196
 
   8271414
 
  PDF   STATS   ENSEMBL   UCSC 
 
  219
 
  6
 
 30249
 
 tss
 
 0.04365
 
 0.71309
 
 Not queried
 
  5.5339027
 
 8.66e-03
 
 1.00e+00
  
 
  
 1
 
  53067637
 
  53067965
 
  PDF   STATS   ENSEMBL   UCSC 
 
  329
 
  5
 
 1629
 
 tss;island
 
 0.06473
 
 0.79736
 
 Not queried
 
  5.5182532
 
 7.94e-03
 
 1.00e+00
  
 
  
 13
 
  79181285
 
  79181613
 
  PDF   STATS   ENSEMBL   UCSC 
 
  329
 
  4
 
 11766
 
 island
 
 0.74525
 
 0.03678
 
 Not queried
 
  5.5152413
 
 2.86e-02
 
 1.00e+00
  
 
  
 10
 
 134838014
 
 134838656
 
  PDF   STATS   ENSEMBL   UCSC 
 
  643
 
  7
 
 6307
 
 island
 
 0.15922
 
 0.84655
 
 Not queried
 
  5.5112047
 
 5.83e-04
 
 1.00e+00
  
 
  
 1
 
 243646235
 
 243646402
 
  PDF   STATS   ENSEMBL   UCSC 
 
  168
 
  4
 
 1109
 
 gene;island
 
 0.06440
 
 0.80293
 
 Not queried
 
  5.5095592
 
 2.86e-02
 
 1.00e+00
  
 
  
 10
 
 134901199
 
 134901305
 
  PDF   STATS   ENSEMBL   UCSC 
 
  107
 
  7
 
 5434
 
 tss
 
 0.02691
 
 0.48235
 
 Not queried
 
  5.5080133
 
 5.83e-04
 
 1.00e+00
  
 
  
 15
 
  25841831
 
  25842111
 
  PDF   STATS   ENSEMBL   UCSC 
 
  281
 
  4
 
 13928
 
 island
 
 0.18517
 
 0.87033
 
 Not queried
 
  5.5064196
 
 2.86e-02
 
 1.00e+00
  
 
  
 7
 
  27195918
 
  27196286
 
  PDF   STATS   ENSEMBL   UCSC 
 
  369
 
  4
 
 36874
 
 gene
 
 0.01727
 
 0.40674
 
 Not queried
 
  5.5018560
 
 2.86e-02
 
 1.00e+00
  
 
  
 8
 
  10683044
 
  10683266
 
  PDF   STATS   ENSEMBL   UCSC 
 
  223
 
  5
 
 39137
 
 gene;tss
 
 0.01389
 
 0.35042
 
 Not queried
 
  5.5011197
 
 9.52e-02
 
 1.00e+00
  
 
  
 2
 
 119599459
 
 119600002
 
  PDF   STATS   ENSEMBL   UCSC 
 
  544
 
  6
 
 24606
 
 island
 
 0.92159
 
 0.15741
 
 Not queried
 
  5.5002451
 
 2.16e-03
 
 1.00e+00
  
 
  
 19
 
  37096010
 
  37096588
 
  PDF   STATS   ENSEMBL   UCSC 
 
  579
 
  7
 
 19969
 
 gene;tss;island
 
 0.03953
 
 0.62178
 
 Not queried
 
  5.4993893
 
 5.83e-04
 
 1.00e+00
  
 
  
 16
 
  89883988
 
  89884347
 
  PDF   STATS   ENSEMBL   UCSC 
 
  360
 
  5
 
 15402
 
 tss;island
 
 0.93390
 
 0.24414
 
 Not queried
 
  5.4977010
 
 7.94e-03
 
 1.00e+00
  
 
  
 20
 
  29535007
 
  29535150
 
  PDF   STATS   ENSEMBL   UCSC 
 
  144
 
  4
 
 26028
 
 island
 
 0.02981
 
 0.57516
 
 Not queried
 
  5.4858364
 
 2.86e-02
 
 1.00e+00
  
 
  
 1
 
  20669724
 
  20669975
 
  PDF   STATS   ENSEMBL   UCSC 
 
  252
 
  4
 
 286
 
 gene;island
 
 0.03384
 
 0.56648
 
 Not queried
 
  5.4828062
 
 2.86e-02
 
 1.00e+00
  
 
  
 7
 
 105221985
 
 105222180
 
  PDF   STATS   ENSEMBL   UCSC 
 
  196
 
  7
 
 37754
 
 tss
 
 0.03022
 
 0.55437
 
 Not queried
 
  5.4812238
 
 5.83e-04
 
 1.00e+00
  
 
  
 17
 
  45401148
 
  45401312
 
  PDF   STATS   ENSEMBL   UCSC 
 
  165
 
  4
 
 17408
 
 tss
 
 0.02482
 
 0.55740
 
 Not queried
 
  5.4762807
 
 2.86e-02
 
 1.00e+00
  
 
  
 8
 
  33457226
 
  33457822
 
  PDF   STATS   ENSEMBL   UCSC 
 
  597
 
  8
 
 40150
 
 island
 
 0.04635
 
 0.75722
 
 Not queried
 
  5.4756808
 
 3.11e-04
 
 1.00e+00
  
 
  
 8
 
 145016475
 
 145016828
 
  PDF   STATS   ENSEMBL   UCSC 
 
  354
 
  4
 
 39435
 
 gene;island
 
 0.01771
 
 0.38141
 
 Not queried
 
  5.4690983
 
 2.86e-02
 
 1.00e+00
  
 
  
 X
 
 153640221
 
 153640674
 
  PDF   STATS   ENSEMBL   UCSC 
 
  454
 
  7
 
 41158
 
 gene;island
 
 0.03801
 
 0.62181
 
 Not queried
 
  5.4629783
 
 5.83e-04
 
 1.00e+00
  
 
  
 X
 
 153640466
 
 153640674
 
  PDF   STATS   ENSEMBL   UCSC 
 
  209
 
  5
 
 41598
 
 tss
 
 0.03801
 
 0.62423
 
 Not queried
 
  5.4629783
 
 7.94e-03
 
 1.00e+00
  
 
  
 5
 
  64398797
 
  64399099
 
  PDF   STATS   ENSEMBL   UCSC 
 
  303
 
  4
 
 32771
 
 island
 
 0.10179
 
 0.83685
 
 Not queried
 
  5.4602139
 
 2.86e-02
 
 1.00e+00
  
 
  
 3
 
  49449687
 
  49449964
 
  PDF   STATS   ENSEMBL   UCSC 
 
  278
 
  6
 
 27742
 
 gene
 
 0.02184
 
 0.52005
 
 Not queried
 
  5.4560025
 
 2.16e-03
 
 1.00e+00
  
 
  
 X
 
 125686936
 
 125687207
 
  PDF   STATS   ENSEMBL   UCSC 
 
  272
 
  7
 
 41507
 
 tss
 
 0.13699
 
 0.89008
 
 Not queried
 
  5.4554007
 
 1.17e-03
 
 1.00e+00
  
 
  
 5
 
 167956306
 
 167956581
 
  PDF   STATS   ENSEMBL   UCSC 
 
  276
 
  5
 
 33308
 
 island
 
 0.02809
 
 0.51901
 
 Not queried
 
  5.4537117
 
 7.94e-03
 
 1.00e+00
  
 
  
 X
 
  46306474
 
  46306568
 
  PDF   STATS   ENSEMBL   UCSC 
 
   95
 
  5
 
 41270
 
 tss
 
 0.03519
 
 0.59478
 
 Not queried
 
  5.4468927
 
 7.94e-03
 
 1.00e+00
  
 
  
 22
 
  17602561
 
  17602681
 
  PDF   STATS   ENSEMBL   UCSC 
 
  121
 
  4
 
 26900
 
 tss;island
 
 0.53575
 
 0.02949
 
 Not queried
 
  5.4461431
 
 2.86e-02
 
 1.00e+00
  
 
  
 5
 
 140718283
 
 140718405
 
  PDF   STATS   ENSEMBL   UCSC 
 
  123
 
  4
 
 31759
 
 gene;island
 
 0.03517
 
 0.57160
 
 Not queried
 
  5.4444414
 
 1.14e-01
 
 1.00e+00
  
 
  
 1
 
 207224510
 
 207224740
 
  PDF   STATS   ENSEMBL   UCSC 
 
  231
 
  4
 
 2213
 
 tss
 
 0.40079
 
 0.00873
 
 Not queried
 
  5.4390189
 
 5.71e-02
 
 1.00e+00
  
 
  
 5
 
 178986620
 
 178986906
 
  PDF   STATS   ENSEMBL   UCSC 
 
  287
 
  5
 
 31874
 
 gene;island
 
 0.48789
 
 0.01809
 
 Not queried
 
  5.4356729
 
 7.94e-03
 
 1.00e+00
  
 
  
 4
 
  15704393
 
  15704550
 
  PDF   STATS   ENSEMBL   UCSC 
 
  158
 
  4
 
 30264
 
 tss
 
 0.19572
 
 0.84256
 
 Not queried
 
  5.4354609
 
 2.86e-02
 
 1.00e+00
  
 
  
 7
 
 155283738
 
 155284062
 
  PDF   STATS   ENSEMBL   UCSC 
 
  325
 
  4
 
 39008
 
 island
 
 0.04801
 
 0.69853
 
 Not queried
 
  5.4293287
 
 2.86e-02
 
 1.00e+00
  
 
  
 3
 
  49066699
 
  49066825
 
  PDF   STATS   ENSEMBL   UCSC 
 
  127
 
  4
 
 27735
 
 gene
 
 0.02266
 
 0.49902
 
 Not queried
 
  5.4282866
 
 2.86e-02
 
 1.00e+00
  
 
  
 7
 
  65970924
 
  65971372
 
  PDF   STATS   ENSEMBL   UCSC 
 
  449
 
  4
 
 38472
 
 island
 
 0.07290
 
 0.81089
 
 Not queried
 
  5.4280679
 
 5.71e-02
 
 1.00e+00
  
 
  
 7
 
 127744210
 
 127744617
 
  PDF   STATS   ENSEMBL   UCSC 
 
  408
 
  4
 
 38826
 
 island
 
 0.03570
 
 0.60708
 
 Not queried
 
  5.4259099
 
 2.86e-02
 
 1.00e+00
  
 
  
 1
 
  38022522
 
  38022718
 
  PDF   STATS   ENSEMBL   UCSC 
 
  197
 
  5
 
 433
 
 gene
 
 0.76331
 
 0.06046
 
 Not queried
 
  5.4202639
 
 1.51e-01
 
 1.00e+00
  
 
  
 3
 
 193922037
 
 193922406
 
  PDF   STATS   ENSEMBL   UCSC 
 
  370
 
  5
 
 29740
 
 island
 
 0.01836
 
 0.40112
 
 Not queried
 
  5.4186970
 
 7.94e-03
 
 1.00e+00
  
 
  
 10
 
  73847963
 
  73848463
 
  PDF   STATS   ENSEMBL   UCSC 
 
  501
 
  5
 
 4612
 
 gene;island
 
 0.20133
 
 0.90147
 
 Not queried
 
  5.4181775
 
 7.94e-03
 
 1.00e+00
  
 
  
 Y
 
  14774019
 
  14774198
 
  PDF   STATS   ENSEMBL   UCSC 
 
  180
 
  4
 
 42167
 
 tss
 
 0.01982
 
 0.47730
 
 Not queried
 
  5.4123403
 
 2.86e-02
 
 1.00e+00
  
 
  
 19
 
  50191342
 
  50191882
 
  PDF   STATS   ENSEMBL   UCSC 
 
  541
 
  6
 
 20924
 
 tss;island
 
 0.15584
 
 0.88427
 
 Not queried
 
  5.4083068
 
 2.16e-03
 
 1.00e+00
  
 
  
 7
 
     31288
 
     31497
 
  PDF   STATS   ENSEMBL   UCSC 
 
  210
 
  5
 
 37962
 
 island
 
 0.04579
 
 0.61482
 
 Not queried
 
  5.4057528
 
 7.94e-03
 
 1.00e+00
  
 
  
 1
 
 228652338
 
 228652581
 
  PDF   STATS   ENSEMBL   UCSC 
 
  244
 
  4
 
 4289
 
 island
 
 0.04690
 
 0.66470
 
 Not queried
 
  5.4030180
 
 2.86e-02
 
 1.00e+00
  
 
  
 19
 
   2525231
 
   2525634
 
  PDF   STATS   ENSEMBL   UCSC 
 
  404
 
  5
 
 19655
 
 gene;island
 
 0.11105
 
 0.80684
 
 Not queried
 
  5.4020059
 
 7.94e-03
 
 1.00e+00
  
 
  
 22
 
  31364274
 
  31364356
 
  PDF   STATS   ENSEMBL   UCSC 
 
   83
 
  5
 
 27029
 
 tss;island
 
 0.72786
 
 0.05566
 
 Not queried
 
  5.3999485
 
 7.94e-03
 
 1.00e+00
  
 
  
 7
 
 156796967
 
 156797410
 
  PDF   STATS   ENSEMBL   UCSC 
 
  444
 
  6
 
 39029
 
 island
 
 0.96932
 
 0.32360
 
 Not queried
 
  5.3980392
 
 2.16e-03
 
 1.00e+00
  
 
  
 22
 
  17489112
 
  17489367
 
  PDF   STATS   ENSEMBL   UCSC 
 
  256
 
  7
 
 26897
 
 tss;island
 
 0.10394
 
 0.89829
 
 Not queried
 
  5.3961543
 
 5.83e-04
 
 1.00e+00
  
 
  
 16
 
  89440878
 
  89441217
 
  PDF   STATS   ENSEMBL   UCSC 
 
  340
 
  4
 
 14911
 
 gene;island
 
 0.24255
 
 0.88746
 
 Not queried
 
  5.3923550
 
 1.14e-01
 
 1.00e+00
  
 
  
 4
 
     53233
 
     53548
 
  PDF   STATS   ENSEMBL   UCSC 
 
  316
 
  5
 
 29781
 
 gene
 
 0.02069
 
 0.46989
 
 Not queried
 
  5.3910134
 
 7.94e-03
 
 1.00e+00
  
 
  
 16
 
  78601767
 
  78601961
 
  PDF   STATS   ENSEMBL   UCSC 
 
  195
 
  4
 
 14819
 
 gene
 
 0.23665
 
 0.93499
 
 Not queried
 
  5.3893335
 
 2.86e-02
 
 1.00e+00
  
 
  
 11
 
   7695165
 
   7695528
 
  PDF   STATS   ENSEMBL   UCSC 
 
  364
 
  7
 
 7101
 
 tss;island
 
 0.03705
 
 0.61656
 
 Not queried
 
  5.3851367
 
 5.83e-04
 
 1.00e+00
  
 
  
 3
 
 157261004
 
 157261106
 
  PDF   STATS   ENSEMBL   UCSC 
 
  103
 
  6
 
 28638
 
 tss
 
 0.05086
 
 0.71330
 
 Not queried
 
  5.3761658
 
 2.16e-03
 
 1.00e+00
  
 
  
 5
 
 139525919
 
 139526226
 
  PDF   STATS   ENSEMBL   UCSC 
 
  308
 
  4
 
 33121
 
 island
 
 0.76434
 
 0.11878
 
 Not queried
 
  5.3669040
 
 2.86e-02
 
 1.00e+00
  
 
  
 11
 
  57414401
 
  57414626
 
  PDF   STATS   ENSEMBL   UCSC 
 
  226
 
  4
 
 6597
 
 gene;island
 
 0.05444
 
 0.69055
 
 Not queried
 
  5.3656133
 
 2.86e-02
 
 1.00e+00
  
 
  
 17
 
  46682308
 
  46682413
 
  PDF   STATS   ENSEMBL   UCSC 
 
  106
 
  6
 
 16622
 
 gene;island
 
 0.05041
 
 0.73489
 
 Not queried
 
  5.3432682
 
 2.16e-03
 
 1.00e+00
  
 
  
 13
 
 112725903
 
 112726124
 
  PDF   STATS   ENSEMBL   UCSC 
 
  222
 
  4
 
 11883
 
 island
 
 0.83980
 
 0.11115
 
 Not queried
 
  5.3394384
 
 2.86e-02
 
 1.00e+00
  
 
  
 13
 
  23489817
 
  23490053
 
  PDF   STATS   ENSEMBL   UCSC 
 
  237
 
  4
 
 11566
 
 island
 
 0.02264
 
 0.47176
 
 Not queried
 
  5.3385135
 
 2.86e-02
 
 1.00e+00
  
 
  
 X
 
  46306719
 
  46306863
 
  PDF   STATS   ENSEMBL   UCSC 
 
  145
 
  4
 
 40969
 
 gene
 
 0.03902
 
 0.59825
 
 Not queried
 
  5.3332933
 
 2.86e-02
 
 1.00e+00
  
 
  
 10
 
 103043991
 
 103044385
 
  PDF   STATS   ENSEMBL   UCSC 
 
  395
 
  5
 
 6047
 
 island
 
 0.86592
 
 0.09068
 
 Not queried
 
  5.3326063
 
 7.94e-03
 
 1.00e+00
  
 
  
 2
 
  75185439
 
  75185645
 
  PDF   STATS   ENSEMBL   UCSC 
 
  207
 
  6
 
 23429
 
 tss;island
 
 0.02718
 
 0.48256
 
 Not queried
 
  5.3324955
 
 2.16e-03
 
 1.00e+00
  
 
  
 19
 
  20277919
 
  20278059
 
  PDF   STATS   ENSEMBL   UCSC 
 
  141
 
  6
 
 20620
 
 tss
 
 0.73329
 
 0.06211
 
 Not queried
 
  5.3322764
 
 2.16e-03
 
 1.00e+00
  
 
  
 11
 
  66360492
 
  66360551
 
  PDF   STATS   ENSEMBL   UCSC 
 
   60
 
  4
 
 6715
 
 gene
 
 0.02222
 
 0.45549
 
 Not queried
 
  5.3314946
 
 2.86e-02
 
 1.00e+00
  
 
  
 21
 
  37442104
 
  37442476
 
  PDF   STATS   ENSEMBL   UCSC 
 
  373
 
  4
 
 26627
 
 island
 
 0.03289
 
 0.56448
 
 Not queried
 
  5.3249086
 
 2.86e-02
 
 1.00e+00
  
 
  
 7
 
 156400711
 
 156400990
 
  PDF   STATS   ENSEMBL   UCSC 
 
  280
 
  4
 
 39022
 
 island
 
 0.19116
 
 0.91125
 
 Not queried
 
  5.3231412
 
 2.86e-02
 
 1.00e+00
  
 
  
 1
 
   3634811
 
   3635210
 
  PDF   STATS   ENSEMBL   UCSC 
 
  400
 
  6
 
 141
 
 gene;island
 
 0.02173
 
 0.50013
 
 Not queried
 
  5.3202879
 
 4.11e-02
 
 1.00e+00
  
 
  
 17
 
  45401148
 
  45401397
 
  PDF   STATS   ENSEMBL   UCSC 
 
  250
 
  5
 
 18420
 
 island
 
 0.02686
 
 0.55826
 
 Not queried
 
  5.3186722
 
 7.94e-03
 
 1.00e+00
  
 
  
 11
 
  73358518
 
  73358657
 
  PDF   STATS   ENSEMBL   UCSC 
 
  140
 
  4
 
 6804
 
 gene;tss
 
 0.41256
 
 0.03387
 
 Not queried
 
  5.3154872
 
 2.86e-02
 
 1.00e+00
  
 
  
 12
 
  63025490
 
  63025948
 
  PDF   STATS   ENSEMBL   UCSC 
 
  459
 
  5
 
 10558
 
 island
 
 0.62432
 
 0.04018
 
 Not queried
 
  5.3108578
 
 7.94e-03
 
 1.00e+00
  
 
  
 X
 
 122866753
 
 122867073
 
  PDF   STATS   ENSEMBL   UCSC 
 
  321
 
  4
 
 42005
 
 island
 
 0.02628
 
 0.61043
 
 Not queried
 
  5.3100687
 
 2.86e-02
 
 1.00e+00
  
 
  
 17
 
  36719518
 
  36719937
 
  PDF   STATS   ENSEMBL   UCSC 
 
  420
 
  5
 
 16504
 
 gene;island
 
 0.84569
 
 0.14879
 
 Not queried
 
  5.3097516
 
 7.94e-03
 
 1.00e+00
  
 
  
 1
 
 207842833
 
 207843084
 
  PDF   STATS   ENSEMBL   UCSC 
 
  252
 
  4
 
 976
 
 gene;island
 
 0.28445
 
 0.94028
 
 Not queried
 
  5.3073497
 
 2.86e-02
 
 1.00e+00
  
 
  
 4
 
   8271196
 
   8271507
 
  PDF   STATS   ENSEMBL   UCSC 
 
  312
 
  7
 
 30867
 
 island
 
 0.04791
 
 0.71275
 
 Not queried
 
  5.3024435
 
 4.08e-03
 
 1.00e+00
  
 
  
 2
 
  74153979
 
  74154363
 
  PDF   STATS   ENSEMBL   UCSC 
 
  385
 
  5
 
 22698
 
 gene
 
 0.38850
 
 0.01384
 
 Not queried
 
  5.3009449
 
 7.94e-03
 
 1.00e+00
  
 
  
 5
 
 140551968
 
 140552600
 
  PDF   STATS   ENSEMBL   UCSC 
 
  633
 
  8
 
 33155
 
 island
 
 0.08111
 
 0.88734
 
 Not queried
 
  5.2998424
 
 2.07e-02
 
 1.00e+00
  
 
  
 X
 
  70712619
 
  70712810
 
  PDF   STATS   ENSEMBL   UCSC 
 
  192
 
  5
 
 41377
 
 tss
 
 0.95871
 
 0.38382
 
 Not queried
 
  5.2996154
 
 7.94e-03
 
 1.00e+00
  
 
  
 22
 
  43253517
 
  43253559
 
  PDF   STATS   ENSEMBL   UCSC 
 
   43
 
  5
 
 27156
 
 tss;island
 
 0.57571
 
 0.01766
 
 Not queried
 
  5.2990605
 
 7.94e-03
 
 1.00e+00
  
 
  
 12
 
  56040025
 
  56040336
 
  PDF   STATS   ENSEMBL   UCSC 
 
  312
 
  5
 
 10480
 
 island
 
 0.05326
 
 0.65034
 
 Not queried
 
  5.2948451
 
 7.94e-03
 
 1.00e+00
  
 
  
 16
 
   5121855
 
   5122251
 
  PDF   STATS   ENSEMBL   UCSC 
 
  397
 
  6
 
 14617
 
 gene
 
 0.02279
 
 0.47849
 
 Not queried
 
  5.2860064
 
 2.16e-03
 
 1.00e+00
  
 
  
 5
 
  77146197
 
  77146318
 
  PDF   STATS   ENSEMBL   UCSC 
 
  122
 
  4
 
 32843
 
 island
 
 0.92984
 
 0.30043
 
 Not queried
 
  5.2832132
 
 2.86e-02
 
 1.00e+00
  
 
  
 15
 
  25093244
 
  25093521
 
  PDF   STATS   ENSEMBL   UCSC 
 
  278
 
  4
 
 13226
 
 gene;island
 
 0.89043
 
 0.15319
 
 Not queried
 
  5.2793137
 
 2.86e-02
 
 1.00e+00
  
 
  
 7
 
 158750244
 
 158750607
 
  PDF   STATS   ENSEMBL   UCSC 
 
  364
 
  4
 
 39094
 
 island
 
 0.09592
 
 0.76919
 
 Not queried
 
  5.2777096
 
 2.86e-02
 
 1.00e+00
  
 
  
 4
 
 107956955
 
 107957430
 
  PDF   STATS   ENSEMBL   UCSC 
 
  476
 
  5
 
 30035
 
 gene;island
 
 0.02886
 
 0.53496
 
 Not queried
 
  5.2745623
 
 7.94e-03
 
 1.00e+00
  
 
  
 17
 
   7791882
 
   7792191
 
  PDF   STATS   ENSEMBL   UCSC 
 
  310
 
  6
 
 16384
 
 gene;island
 
 0.12391
 
 0.79373
 
 Not queried
 
  5.2662341
 
 2.16e-03
 
 1.00e+00
  
 
  
 16
 
  82045140
 
  82045222
 
  PDF   STATS   ENSEMBL   UCSC 
 
   83
 
  4
 
 15353
 
 tss
 
 0.06416
 
 0.71238
 
 Not queried
 
  5.2621832
 
 2.86e-02
 
 1.00e+00
  
 
  
 10
 
 134775542
 
 134776050
 
  PDF   STATS   ENSEMBL   UCSC 
 
  509
 
  5
 
 6302
 
 island
 
 0.04993
 
 0.67461
 
 Not queried
 
  5.2550256
 
 7.94e-03
 
 1.00e+00
  
 
  
 5
 
 176170138
 
 176170795
 
  PDF   STATS   ENSEMBL   UCSC 
 
  658
 
  7
 
 33369
 
 island
 
 0.05230
 
 0.52083
 
 Not queried
 
  5.2542764
 
 5.83e-04
 
 1.00e+00
  
 
  
 12
 
 121688317
 
 121688459
 
  PDF   STATS   ENSEMBL   UCSC 
 
  143
 
  4
 
 9337
 
 gene
 
 0.11272
 
 0.87355
 
 Not queried
 
  5.2515195
 
 2.86e-02
 
 1.00e+00
  
 
  
 5
 
 140180921
 
 140181074
 
  PDF   STATS   ENSEMBL   UCSC 
 
  154
 
  4
 
 31737
 
 gene;island
 
 0.05731
 
 0.63485
 
 Not queried
 
  5.2445032
 
 2.86e-02
 
 1.00e+00
  
 
  
 2
 
 200468445
 
 200469026
 
  PDF   STATS   ENSEMBL   UCSC 
 
  582
 
  5
 
 24955
 
 island
 
 0.18162
 
 0.87295
 
 Not queried
 
  5.2436546
 
 7.94e-03
 
 1.00e+00
  
 
  
 19
 
  12759034
 
  12759156
 
  PDF   STATS   ENSEMBL   UCSC 
 
  123
 
  4
 
 19822
 
 gene;island
 
 0.06518
 
 0.65823
 
 Not queried
 
  5.2413053
 
 2.86e-02
 
 1.00e+00
  
 
  
 14
 
 101175970
 
 101176310
 
  PDF   STATS   ENSEMBL   UCSC 
 
  341
 
  4
 
 13112
 
 island
 
 0.07453
 
 0.84869
 
 Not queried
 
  5.2372728
 
 1.14e-01
 
 1.00e+00
  
 
  
 8
 
  43132125
 
  43132507
 
  PDF   STATS   ENSEMBL   UCSC 
 
  383
 
  4
 
 40203
 
 island
 
 0.08722
 
 0.78831
 
 Not queried
 
  5.2354725
 
 2.86e-02
 
 1.00e+00
  
 
  
 16
 
  89913618
 
  89913972
 
  PDF   STATS   ENSEMBL   UCSC 
 
  355
 
  4
 
 14919
 
 gene;island
 
 0.24470
 
 0.91369
 
 Not queried
 
  5.2345681
 
 2.86e-02
 
 1.00e+00
  
 
  
 4
 
   3873884
 
   3874143
 
  PDF   STATS   ENSEMBL   UCSC 
 
  260
 
  4
 
 30807
 
 island
 
 0.26627
 
 0.93261
 
 Not queried
 
  5.2334965
 
 2.86e-02
 
 1.00e+00
  
 
  
 1
 
 151103869
 
 151104367
 
  PDF   STATS   ENSEMBL   UCSC 
 
  499
 
  4
 
 3728
 
 island
 
 0.04618
 
 0.52680
 
 Not queried
 
  5.2275588
 
 2.86e-02
 
 1.00e+00
  
 
  
 1
 
  23521272
 
  23521417
 
  PDF   STATS   ENSEMBL   UCSC 
 
  146
 
  4
 
 1332
 
 tss
 
 0.19284
 
 0.89243
 
 Not queried
 
  5.2235651
 
 2.86e-02
 
 1.00e+00
  
 
  
 11
 
 123430575
 
 123430949
 
  PDF   STATS   ENSEMBL   UCSC 
 
  375
 
  4
 
 6930
 
 gene
 
 0.05724
 
 0.67401
 
 Not queried
 
  5.2217154
 
 2.86e-02
 
 1.00e+00
  
 
  
 1
 
  52498502
 
  52498993
 
  PDF   STATS   ENSEMBL   UCSC 
 
  492
 
  5
 
 528
 
 gene;island
 
 0.58113
 
 0.04148
 
 Not queried
 
  5.2134346
 
 7.94e-03
 
 1.00e+00
  
 
  
 X
 
  11445498
 
  11445818
 
  PDF   STATS   ENSEMBL   UCSC 
 
  321
 
  4
 
 40934
 
 gene;island
 
 0.09777
 
 0.82598
 
 Not queried
 
  5.2073705
 
 5.71e-02
 
 1.00e+00
  
 
  
 15
 
  57998711
 
  57998894
 
  PDF   STATS   ENSEMBL   UCSC 
 
  184
 
  5
 
 13668
 
 tss
 
 0.59031
 
 0.03703
 
 Not queried
 
  5.2069675
 
 4.21e-01
 
 1.00e+00
  
 
  
 6
 
 117086931
 
 117087238
 
  PDF   STATS   ENSEMBL   UCSC 
 
  308
 
  7
 
 35129
 
 tss
 
 0.03779
 
 0.59565
 
 Not queried
 
  5.2041214
 
 5.83e-04
 
 1.00e+00
  
 
  
 1
 
 153508511
 
 153508713
 
  PDF   STATS   ENSEMBL   UCSC 
 
  203
 
  6
 
 758
 
 gene
 
 0.31338
 
 0.92518
 
 Not queried
 
  5.2040872
 
 2.16e-03
 
 1.00e+00
  
 
  
 13
 
  36728869
 
  36729247
 
  PDF   STATS   ENSEMBL   UCSC 
 
  379
 
  6
 
 11637
 
 island
 
 0.02856
 
 0.54651
 
 Not queried
 
  5.2040543
 
 2.16e-03
 
 1.00e+00
  
 
  
 7
 
 128578023
 
 128578297
 
  PDF   STATS   ENSEMBL   UCSC 
 
  275
 
  4
 
 37155
 
 gene
 
 0.03490
 
 0.59695
 
 Not queried
 
  5.2000892
 
 2.86e-02
 
 1.00e+00
  
 
  
 5
 
 172665707
 
 172666228
 
  PDF   STATS   ENSEMBL   UCSC 
 
  522
 
  4
 
 33345
 
 island
 
 0.03855
 
 0.65150
 
 Not queried
 
  5.1932709
 
 2.86e-02
 
 1.00e+00
  
 
  
 3
 
  44626492
 
  44626860
 
  PDF   STATS   ENSEMBL   UCSC 
 
  369
 
  4
 
 27698
 
 gene
 
 0.41589
 
 0.03080
 
 Not queried
 
  5.1929095
 
 2.86e-02
 
 1.00e+00
  
 
  
 15
 
  66947392
 
  66947617
 
  PDF   STATS   ENSEMBL   UCSC 
 
  226
 
  4
 
 14217
 
 island
 
 0.05105
 
 0.63832
 
 Not queried
 
  5.1885746
 
 2.86e-02
 
 1.00e+00
  
 
  
 1
 
 209957601
 
 209957750
 
  PDF   STATS   ENSEMBL   UCSC 
 
  150
 
  5
 
 984
 
 gene;island
 
 0.03921
 
 0.61169
 
 Not queried
 
  5.1869996
 
 7.94e-03
 
 1.00e+00
  
 
  
 2
 
  39005158
 
  39005322
 
  PDF   STATS   ENSEMBL   UCSC 
 
  165
 
  4
 
 23276
 
 tss
 
 0.01092
 
 0.31125
 
 Not queried
 
  5.1828112
 
 2.86e-02
 
 1.00e+00
  
 
  
 16
 
  75150456
 
  75150880
 
  PDF   STATS   ENSEMBL   UCSC 
 
  425
 
  8
 
 16062
 
 island
 
 0.82506
 
 0.06482
 
 Not queried
 
  5.1827710
 
 6.22e-04
 
 1.00e+00
  
 
  
 17
 
   3716214
 
   3716558
 
  PDF   STATS   ENSEMBL   UCSC 
 
  345
 
  6
 
 16312
 
 gene
 
 0.32282
 
 0.91927
 
 Not queried
 
  5.1827365
 
 2.16e-03
 
 1.00e+00
  
 
  
 5
 
   2753852
 
   2754322
 
  PDF   STATS   ENSEMBL   UCSC 
 
  471
 
  8
 
 31494
 
 gene;island
 
 0.65068
 
 0.05224
 
 Not queried
 
  5.1818994
 
 1.09e-03
 
 1.00e+00
  
 
  
 5
 
  54518469
 
  54519307
 
  PDF   STATS   ENSEMBL   UCSC 
 
  839
 
  7
 
 32738
 
 island
 
 0.86138
 
 0.07167
 
 Not queried
 
  5.1788049
 
 5.83e-04
 
 1.00e+00
  
 
  
 Y
 
   6778543
 
   6778695
 
  PDF   STATS   ENSEMBL   UCSC 
 
  153
 
  4
 
 42166
 
 tss;island
 
 0.95581
 
 0.32469
 
 Not queried
 
  5.1746947
 
 2.86e-02
 
 1.00e+00
  
 
  
 20
 
  57581903
 
  57582292
 
  PDF   STATS   ENSEMBL   UCSC 
 
  390
 
  6
 
 25440
 
 gene
 
 0.03119
 
 0.56531
 
 Not queried
 
  5.1678681
 
 2.16e-03
 
 1.00e+00
  
 
  
 5
 
 178487123
 
 178488100
 
  PDF   STATS   ENSEMBL   UCSC 
 
  978
 
 14
 
 33415
 
 island
 
 0.01466
 
 0.37227
 
 Not queried
 
  5.1655194
 
 4.21e-04
 
 1.00e+00
  
 
  
 1
 
   1713944
 
   1714271
 
  PDF   STATS   ENSEMBL   UCSC 
 
  328
 
  5
 
 2458
 
 island
 
 0.11169
 
 0.82404
 
 Not queried
 
  5.1655052
 
 7.94e-03
 
 1.00e+00
  
 
  
 15
 
  76005189
 
  76005446
 
  PDF   STATS   ENSEMBL   UCSC 
 
  258
 
  4
 
 13789
 
 tss
 
 0.04299
 
 0.60868
 
 Not queried
 
  5.1615520
 
 2.86e-02
 
 1.00e+00
  
 
  
 4
 
   2765767
 
   2766135
 
  PDF   STATS   ENSEMBL   UCSC 
 
  369
 
  5
 
 30771
 
 island
 
 0.14153
 
 0.85446
 
 Not queried
 
  5.1542453
 
 3.17e-02
 
 1.00e+00
  
 
  
 14
 
  76451771
 
  76452033
 
  PDF   STATS   ENSEMBL   UCSC 
 
  263
 
  4
 
 12495
 
 tss
 
 0.54446
 
 0.04765
 
 Not queried
 
  5.1530567
 
 1.14e-01
 
 1.00e+00
  
 
  
 11
 
   5617703
 
   5617926
 
  PDF   STATS   ENSEMBL   UCSC 
 
  224
 
  5
 
 6467
 
 gene
 
 0.02437
 
 0.50760
 
 Not queried
 
  5.1489539
 
 7.94e-03
 
 1.00e+00
  
 
  
 12
 
  52627272
 
  52627576
 
  PDF   STATS   ENSEMBL   UCSC 
 
  305
 
  4
 
 9110
 
 gene;island
 
 0.11993
 
 0.81190
 
 Not queried
 
  5.1482124
 
 2.86e-02
 
 1.00e+00
  
 
  
 15
 
  36871420
 
  36871800
 
  PDF   STATS   ENSEMBL   UCSC 
 
  381
 
  5
 
 13538
 
 tss
 
 0.01537
 
 0.45608
 
 Not queried
 
  5.1403935
 
 7.94e-03
 
 1.00e+00
  
 
  
 2
 
 152118408
 
 152118835
 
  PDF   STATS   ENSEMBL   UCSC 
 
  428
 
  8
 
 23620
 
 tss;island
 
 0.03048
 
 0.52754
 
 Not queried
 
  5.1290290
 
 4.99e-02
 
 1.00e+00
  
 
  
 2
 
 162930175
 
 162930454
 
  PDF   STATS   ENSEMBL   UCSC 
 
  280
 
  4
 
 22873
 
 gene;island
 
 0.02359
 
 0.50376
 
 Not queried
 
  5.1284432
 
 2.86e-02
 
 1.00e+00
  
 
  
 12
 
  54343506
 
  54343835
 
  PDF   STATS   ENSEMBL   UCSC 
 
  330
 
  5
 
 10453
 
 island
 
 0.02502
 
 0.47936
 
 Not queried
 
  5.1277501
 
 3.17e-02
 
 1.00e+00
  
 
  
 13
 
  50706583
 
  50707065
 
  PDF   STATS   ENSEMBL   UCSC 
 
  483
 
  6
 
 11714
 
 island
 
 0.47568
 
 0.03176
 
 Not queried
 
  5.1273900
 
 2.16e-03
 
 1.00e+00
  
 
  
 6
 
  31111076
 
  31111270
 
  PDF   STATS   ENSEMBL   UCSC 
 
  195
 
  4
 
 33792
 
 gene
 
 0.08609
 
 0.74340
 
 Not queried
 
  5.1267032
 
 2.86e-02
 
 1.00e+00
  
 
  
 13
 
 113424938
 
 113425298
 
  PDF   STATS   ENSEMBL   UCSC 
 
  361
 
  6
 
 11223
 
 gene;island
 
 0.08682
 
 0.72233
 
 Not queried
 
  5.1227238
 
 2.16e-03
 
 1.00e+00
  
 
  
 4
 
  74864596
 
  74864725
 
  PDF   STATS   ENSEMBL   UCSC 
 
  130
 
  4
 
 30385
 
 tss;island
 
 0.11110
 
 0.72426
 
 Not queried
 
  5.1158526
 
 2.86e-02
 
 1.00e+00
  
 
  
 16
 
  31483137
 
  31483534
 
  PDF   STATS   ENSEMBL   UCSC 
 
  398
 
  5
 
 15181
 
 tss;island
 
 0.36742
 
 0.05707
 
 Not queried
 
  5.1139911
 
 9.52e-02
 
 1.00e+00
  
 
  
 14
 
 105173643
 
 105173980
 
  PDF   STATS   ENSEMBL   UCSC 
 
  338
 
  5
 
 12220
 
 gene
 
 0.16342
 
 0.89625
 
 Not queried
 
  5.1121922
 
 7.94e-03
 
 1.00e+00
  
 
  
 12
 
 130822603
 
 130822818
 
  PDF   STATS   ENSEMBL   UCSC 
 
  216
 
  4
 
 10973
 
 island
 
 0.41364
 
 0.94691
 
 Not queried
 
  5.1081074
 
 2.86e-02
 
 1.00e+00
  
 
  
 16
 
  51183988
 
  51184583
 
  PDF   STATS   ENSEMBL   UCSC 
 
  596
 
  8
 
 14718
 
 gene;island
 
 0.61592
 
 0.02418
 
 Not queried
 
  5.1044684
 
 1.86e-03
 
 1.00e+00
  
 
  
 16
 
  68270129
 
  68270387
 
  PDF   STATS   ENSEMBL   UCSC 
 
  259
 
  5
 
 16008
 
 island
 
 0.02886
 
 0.51379
 
 Not queried
 
  5.1039406
 
 7.94e-03
 
 1.00e+00
  
 
  
 5
 
 137610255
 
 137610645
 
  PDF   STATS   ENSEMBL   UCSC 
 
  391
 
  7
 
 32258
 
 tss
 
 0.02524
 
 0.49104
 
 Not queried
 
  5.1010304
 
 1.17e-03
 
 1.00e+00
  
 
  
 2
 
 161127463
 
 161127884
 
  PDF   STATS   ENSEMBL   UCSC 
 
  422
 
  5
 
 24777
 
 island
 
 0.16314
 
 0.86991
 
 Not queried
 
  5.1003030
 
 7.94e-03
 
 1.00e+00
  
 
  
 7
 
   1121657
 
   1121929
 
  PDF   STATS   ENSEMBL   UCSC 
 
  273
 
  4
 
 36685
 
 gene;island
 
 0.21229
 
 0.89319
 
 Not queried
 
  5.0995191
 
 2.86e-02
 
 1.00e+00
  
 
  
 X
 
 148713660
 
 148713830
 
  PDF   STATS   ENSEMBL   UCSC 
 
  171
 
  7
 
 41563
 
 tss;island
 
 0.03933
 
 0.58303
 
 Not queried
 
  5.0992642
 
 5.83e-04
 
 1.00e+00
  
 
  
 7
 
  87848143
 
  87848358
 
  PDF   STATS   ENSEMBL   UCSC 
 
  216
 
  4
 
 37013
 
 gene;island
 
 0.56612
 
 0.04496
 
 Not queried
 
  5.0970534
 
 2.86e-02
 
 1.00e+00
  
 
  
 2
 
   1286216
 
   1286641
 
  PDF   STATS   ENSEMBL   UCSC 
 
  426
 
  5
 
 22491
 
 gene;island
 
 0.07673
 
 0.75063
 
 Not queried
 
  5.0914674
 
 7.94e-03
 
 1.00e+00
  
 
  
 4
 
   3748154
 
   3748347
 
  PDF   STATS   ENSEMBL   UCSC 
 
  194
 
  4
 
 30804
 
 island
 
 0.11688
 
 0.83903
 
 Not queried
 
  5.0893771
 
 2.86e-02
 
 1.00e+00
  
 
  
 6
 
   2842146
 
   2842550
 
  PDF   STATS   ENSEMBL   UCSC 
 
  405
 
  7
 
 34527
 
 tss
 
 0.08252
 
 0.72869
 
 Not queried
 
  5.0887315
 
 2.62e-02
 
 1.00e+00
  
 
  
 6
 
   2841875
 
   2842550
 
  PDF   STATS   ENSEMBL   UCSC 
 
  676
 
  9
 
 35313
 
 island
 
 0.08252
 
 0.70478
 
 Not queried
 
  5.0887315
 
 3.99e-03
 
 1.00e+00
  
 
  
 6
 
 151560831
 
 151561055
 
  PDF   STATS   ENSEMBL   UCSC 
 
  225
 
  7
 
 35234
 
 tss
 
 0.69047
 
 0.12180
 
 Not queried
 
  5.0853035
 
 5.83e-04
 
 1.00e+00
  
 
  
 16
 
  85362963
 
  85363209
 
  PDF   STATS   ENSEMBL   UCSC 
 
  247
 
  4
 
 16119
 
 island
 
 0.48562
 
 0.03669
 
 Not queried
 
  5.0822450
 
 2.86e-02
 
 1.00e+00
  
 
  
 X
 
  73164102
 
  73164145
 
  PDF   STATS   ENSEMBL   UCSC 
 
   44
 
  4
 
 41389
 
 tss
 
 0.02693
 
 0.39853
 
 Not queried
 
  5.0804497
 
 2.86e-02
 
 1.00e+00
  
 
  
 10
 
  50886878
 
  50887212
 
  PDF   STATS   ENSEMBL   UCSC 
 
  335
 
  5
 
 5058
 
 tss;island
 
 0.19102
 
 0.78274
 
 Not queried
 
  5.0784539
 
 7.94e-03
 
 1.00e+00
  
 
  
 15
 
  89921672
 
  89922071
 
  PDF   STATS   ENSEMBL   UCSC 
 
  400
 
  4
 
 13439
 
 gene;island
 
 0.19201
 
 0.87603
 
 Not queried
 
  5.0748894
 
 2.86e-02
 
 1.00e+00
  
 
  
 6
 
  66497586
 
  66498032
 
  PDF   STATS   ENSEMBL   UCSC 
 
  447
 
  4
 
 36169
 
 island
 
 0.08101
 
 0.69084
 
 Not queried
 
  5.0715924
 
 2.86e-02
 
 1.00e+00
  
 
  
 10
 
  50506748
 
  50507092
 
  PDF   STATS   ENSEMBL   UCSC 
 
  345
 
  7
 
 5054
 
 tss
 
 0.08385
 
 0.80601
 
 Not queried
 
  5.0713589
 
 2.33e-03
 
 1.00e+00
  
 
  
 21
 
  47394354
 
  47394659
 
  PDF   STATS   ENSEMBL   UCSC 
 
  306
 
  4
 
 26723
 
 island
 
 0.04576
 
 0.59328
 
 Not queried
 
  5.0657598
 
 2.86e-02
 
 1.00e+00
  
 
  
 19
 
  59074265
 
  59074507
 
  PDF   STATS   ENSEMBL   UCSC 
 
  243
 
  4
 
 20228
 
 gene;island
 
 0.04060
 
 0.58931
 
 Not queried
 
  5.0616657
 
 2.86e-02
 
 1.00e+00
  
 
  
 10
 
  27541213
 
  27541418
 
  PDF   STATS   ENSEMBL   UCSC 
 
  206
 
  5
 
 5609
 
 island
 
 0.08830
 
 0.90683
 
 Not queried
 
  5.0594058
 
 7.94e-03
 
 1.00e+00
  
 
  
 6
 
 111408745
 
 111408765
 
  PDF   STATS   ENSEMBL   UCSC 
 
   21
 
  5
 
 35111
 
 tss
 
 0.53416
 
 0.03326
 
 Not queried
 
  5.0588451
 
 7.94e-03
 
 1.00e+00
  
 
  
 5
 
 157098338
 
 157098555
 
  PDF   STATS   ENSEMBL   UCSC 
 
  218
 
  6
 
 32398
 
 tss
 
 0.04443
 
 0.59059
 
 Not queried
 
  5.0585213
 
 2.16e-03
 
 1.00e+00
  
 
  
 2
 
 198651303
 
 198651576
 
  PDF   STATS   ENSEMBL   UCSC 
 
  274
 
  4
 
 23765
 
 tss;island
 
 0.03292
 
 0.50989
 
 Not queried
 
  5.0561144
 
 2.86e-02
 
 1.00e+00
  
 
  
 2
 
 235406275
 
 235406540
 
  PDF   STATS   ENSEMBL   UCSC 
 
  266
 
  4
 
 23923
 
 tss;island
 
 0.79686
 
 0.10859
 
 Not queried
 
  5.0556905
 
 5.71e-02
 
 1.00e+00
  
 
  
 X
 
  73642618
 
  73642885
 
  PDF   STATS   ENSEMBL   UCSC 
 
  268
 
  4
 
 41037
 
 gene;island
 
 0.60737
 
 0.04548
 
 Not queried
 
  5.0423790
 
 2.86e-02
 
 1.00e+00
  
 
  
 22
 
  35937058
 
  35937531
 
  PDF   STATS   ENSEMBL   UCSC 
 
  474
 
  8
 
 27405
 
 island
 
 0.63018
 
 0.04151
 
 Not queried
 
  5.0397445
 
 6.22e-04
 
 1.00e+00
  
 
  
 16
 
  68270252
 
  68270387
 
  PDF   STATS   ENSEMBL   UCSC 
 
  136
 
  4
 
 15288
 
 tss
 
 0.03348
 
 0.53686
 
 Not queried
 
  5.0345616
 
 2.86e-02
 
 1.00e+00
  
 
  
 14
 
 104551867
 
 104552397
 
  PDF   STATS   ENSEMBL   UCSC 
 
  531
 
  6
 
 13170
 
 island
 
 0.29586
 
 0.93061
 
 Not queried
 
  5.0334151
 
 2.16e-03
 
 1.00e+00
  
 
  
 5
 
  38445522
 
  38445916
 
  PDF   STATS   ENSEMBL   UCSC 
 
  395
 
  7
 
 31539
 
 gene
 
 0.04780
 
 0.61240
 
 Not queried
 
  5.0308140
 
 5.83e-04
 
 1.00e+00
  
 
  
 17
 
  79393463
 
  79393718
 
  PDF   STATS   ENSEMBL   UCSC 
 
  256
 
  5
 
 16875
 
 gene;island
 
 0.21180
 
 0.78186
 
 Not queried
 
  5.0290483
 
 7.94e-03
 
 1.00e+00
  
 
  
 16
 
  14397576
 
  14397766
 
  PDF   STATS   ENSEMBL   UCSC 
 
  191
 
  5
 
 15070
 
 tss
 
 0.43334
 
 0.03145
 
 Not queried
 
  5.0259296
 
 7.94e-03
 
 1.00e+00
  
 
  
 5
 
 140723331
 
 140723583
 
  PDF   STATS   ENSEMBL   UCSC 
 
  253
 
  6
 
 32314
 
 tss
 
 0.19234
 
 0.84771
 
 Not queried
 
  5.0246396
 
 2.16e-03
 
 1.00e+00
  
 
  
 6
 
  27839751
 
  27840068
 
  PDF   STATS   ENSEMBL   UCSC 
 
  318
 
  4
 
 33559
 
 gene
 
 0.22634
 
 0.90049
 
 Not queried
 
  5.0174783
 
 2.86e-02
 
 1.00e+00
  
 
  
 3
 
 172166182
 
 172166258
 
  PDF   STATS   ENSEMBL   UCSC 
 
   77
 
  4
 
 29622
 
 island
 
 0.12926
 
 0.86002
 
 Not queried
 
  5.0173143
 
 2.86e-02
 
 1.00e+00
  
 
  
 8
 
  22960177
 
  22960619
 
  PDF   STATS   ENSEMBL   UCSC 
 
  443
 
 10
 
 40081
 
 island
 
 0.04635
 
 0.56908
 
 Not queried
 
  5.0066349
 
 2.88e-03
 
 1.00e+00
  
 
  
 20
 
  61148764
 
  61149009
 
  PDF   STATS   ENSEMBL   UCSC 
 
  246
 
  7
 
 25835
 
 tss
 
 0.16365
 
 0.87094
 
 Not queried
 
  5.0045200
 
 5.83e-04
 
 1.00e+00
  
 
  
 5
 
  38445522
 
  38445614
 
  PDF   STATS   ENSEMBL   UCSC 
 
   93
 
  4
 
 31970
 
 tss
 
 0.09368
 
 0.59260
 
 Not queried
 
  5.0034163
 
 2.86e-02
 
 1.00e+00
  
 
  
 17
 
  33775295
 
  33775779
 
  PDF   STATS   ENSEMBL   UCSC 
 
  485
 
  5
 
 16485
 
 gene
 
 0.17234
 
 0.86962
 
 Not queried
 
  5.0015486
 
 7.94e-03
 
 1.00e+00
  
 
  
 2
 
 162930666
 
 162931175
 
  PDF   STATS   ENSEMBL   UCSC 
 
  510
 
  5
 
 24787
 
 island
 
 0.02912
 
 0.45262
 
 Not queried
 
  5.0015387
 
 7.94e-03
 
 1.00e+00
  
 
  
 5
 
  76934327
 
  76934696
 
  PDF   STATS   ENSEMBL   UCSC 
 
  370
 
  6
 
 32839
 
 island
 
 0.48674
 
 0.02617
 
 Not queried
 
  5.0008761
 
 4.33e-03
 
 1.00e+00
  
 
  
 3
 
  55508379
 
  55508686
 
  PDF   STATS   ENSEMBL   UCSC 
 
  308
 
  4
 
 27795
 
 gene;island
 
 0.12732
 
 0.90904
 
 Not queried
 
  5.0006975
 
 2.86e-02
 
 1.00e+00
  
 
  
 6
 
 167508001
 
 167508226
 
  PDF   STATS   ENSEMBL   UCSC 
 
  226
 
  6
 
 36580
 
 island
 
 0.31775
 
 0.91141
 
 Not queried
 
  4.9962796
 
 8.66e-03
 
 1.00e+00
  
 
  
 8
 
  24771273
 
  24771645
 
  PDF   STATS   ENSEMBL   UCSC 
 
  373
 
  4
 
 39182
 
 gene
 
 0.11955
 
 0.71503
 
 Not queried
 
  4.9961978
 
 2.86e-02
 
 1.00e+00
  
 
  
 7
 
  27134109
 
  27134369
 
  PDF   STATS   ENSEMBL   UCSC 
 
  261
 
  4
 
 36863
 
 gene;island
 
 0.84593
 
 0.19557
 
 Not queried
 
  4.9949091
 
 2.86e-02
 
 1.00e+00
  
 
  
 16
 
  90113893
 
  90114173
 
  PDF   STATS   ENSEMBL   UCSC 
 
  281
 
  4
 
 14925
 
 gene
 
 0.03378
 
 0.52693
 
 Not queried
 
  4.9943349
 
 2.86e-02
 
 1.00e+00
  
 
  
 12
 
 131438523
 
 131438788
 
  PDF   STATS   ENSEMBL   UCSC 
 
  266
 
  6
 
 9394
 
 gene
 
 0.10806
 
 0.81799
 
 Not queried
 
  4.9891261
 
 2.16e-03
 
 1.00e+00
  
 
  
 2
 
  91777099
 
  91777482
 
  PDF   STATS   ENSEMBL   UCSC 
 
  384
 
  4
 
 24470
 
 island
 
 0.15980
 
 0.87096
 
 Not queried
 
  4.9855756
 
 2.86e-02
 
 1.00e+00
  
 
  
 15
 
  36871420
 
  36871852
 
  PDF   STATS   ENSEMBL   UCSC 
 
  433
 
  7
 
 13979
 
 island
 
 0.02049
 
 0.40110
 
 Not queried
 
  4.9854208
 
 5.83e-04
 
 1.00e+00
  
 
  
 8
 
 143695822
 
 143696144
 
  PDF   STATS   ENSEMBL   UCSC 
 
  323
 
  7
 
 40573
 
 island
 
 0.06485
 
 0.56400
 
 Not queried
 
  4.9850348
 
 5.83e-04
 
 1.00e+00
  
 
  
 12
 
  54410657
 
  54410727
 
  PDF   STATS   ENSEMBL   UCSC 
 
   71
 
  5
 
 9133
 
 gene
 
 0.00703
 
 0.21760
 
 Not queried
 
  4.9817744
 
 7.94e-03
 
 1.00e+00
  
 
  
 X
 
 153990478
 
 153991165
 
  PDF   STATS   ENSEMBL   UCSC 
 
  688
 
 10
 
 42154
 
 island
 
 0.02604
 
 0.54607
 
 Not queried
 
  4.9805902
 
 1.05e-03
 
 1.00e+00
  
 
  
 X
 
  23350313
 
  23350651
 
  PDF   STATS   ENSEMBL   UCSC 
 
  339
 
  4
 
 41684
 
 island
 
 0.49805
 
 0.02660
 
 Not queried
 
  4.9800857
 
 2.86e-02
 
 1.00e+00
  
 
  
 4
 
 113432595
 
 113432911
 
  PDF   STATS   ENSEMBL   UCSC 
 
  317
 
  4
 
 31201
 
 island
 
 0.61177
 
 0.04168
 
 Not queried
 
  4.9792174
 
 2.86e-02
 
 1.00e+00
  
 
  
 2
 
   9614471
 
   9614744
 
  PDF   STATS   ENSEMBL   UCSC 
 
  274
 
  7
 
 24018
 
 island
 
 0.43700
 
 0.90900
 
 Not queried
 
  4.9781732
 
 5.83e-04
 
 1.00e+00
  
 
  
 19
 
  38754529
 
  38754930
 
  PDF   STATS   ENSEMBL   UCSC 
 
  402
 
  5
 
 20712
 
 tss
 
 0.04250
 
 0.55001
 
 Not queried
 
  4.9718615
 
 7.94e-03
 
 1.00e+00
  
 
  
 1
 
 111506233
 
 111506560
 
  PDF   STATS   ENSEMBL   UCSC 
 
  328
 
  6
 
 677
 
 gene
 
 0.01721
 
 0.33124
 
 Not queried
 
  4.9688139
 
 2.16e-03
 
 1.00e+00
  
 
  
 20
 
  57581903
 
  57582371
 
  PDF   STATS   ENSEMBL   UCSC 
 
  469
 
  7
 
 26259
 
 island
 
 0.03205
 
 0.56709
 
 Not queried
 
  4.9678786
 
 5.83e-04
 
 1.00e+00
  
 
  
 10
 
  38691787
 
  38691801
 
  PDF   STATS   ENSEMBL   UCSC 
 
   15
 
  4
 
 5020
 
 tss;island
 
 0.02971
 
 0.52951
 
 Not queried
 
  4.9672791
 
 2.86e-02
 
 1.00e+00
  
 
  
 8
 
  24771114
 
  24771645
 
  PDF   STATS   ENSEMBL   UCSC 
 
  532
 
  7
 
 39540
 
 tss;island
 
 0.12077
 
 0.71551
 
 Not queried
 
  4.9672604
 
 5.83e-04
 
 1.00e+00
  
 
  
 16
 
  80574553
 
  80574757
 
  PDF   STATS   ENSEMBL   UCSC 
 
  205
 
  4
 
 15344
 
 tss
 
 0.01387
 
 0.39083
 
 Not queried
 
  4.9672309
 
 2.86e-02
 
 1.00e+00
  
 
  
 6
 
    291687
 
    291909
 
  PDF   STATS   ENSEMBL   UCSC 
 
  223
 
  4
 
 34520
 
 tss;island
 
 0.02223
 
 0.47868
 
 Not queried
 
  4.9623535
 
 2.86e-02
 
 1.00e+00
  
 
  
 8
 
  11559039
 
  11559488
 
  PDF   STATS   ENSEMBL   UCSC 
 
  450
 
  4
 
 40021
 
 island
 
 0.94472
 
 0.44559
 
 Not queried
 
  4.9612258
 
 2.00e-01
 
 1.00e+00
  
 
  
 6
 
  85482570
 
  85483055
 
  PDF   STATS   ENSEMBL   UCSC 
 
  486
 
  5
 
 36236
 
 island
 
 0.01750
 
 0.10203
 
 Not queried
 
  4.9555974
 
 3.17e-02
 
 1.00e+00
  
 
  
 X
 
  17755531
 
  17755839
 
  PDF   STATS   ENSEMBL   UCSC 
 
  309
 
  6
 
 41669
 
 island
 
 0.25757
 
 0.01468
 
 Not queried
 
  4.9535621
 
 2.16e-03
 
 1.00e+00
  
 
  
 16
 
  30198370
 
  30198517
 
  PDF   STATS   ENSEMBL   UCSC 
 
  148
 
  4
 
 14688
 
 gene;island
 
 0.12803
 
 0.70923
 
 Not queried
 
  4.9522935
 
 5.71e-02
 
 1.00e+00
  
 
  
 12
 
  54088760
 
  54088972
 
  PDF   STATS   ENSEMBL   UCSC 
 
  213
 
  4
 
 10444
 
 island
 
 0.70431
 
 0.09034
 
 Not queried
 
  4.9494124
 
 2.86e-02
 
 1.00e+00
  
 
  
 2
 
 162930666
 
 162931003
 
  PDF   STATS   ENSEMBL   UCSC 
 
  338
 
  4
 
 22874
 
 gene
 
 0.03566
 
 0.50837
 
 Not queried
 
  4.9435018
 
 2.86e-02
 
 1.00e+00
  
 
  
 6
 
  27064705
 
  27065003
 
  PDF   STATS   ENSEMBL   UCSC 
 
  299
 
  4
 
 35482
 
 island
 
 0.02443
 
 0.44102
 
 Not queried
 
  4.9427221
 
 2.86e-02
 
 1.00e+00
  
 
  
 2
 
 109647956
 
 109648208
 
  PDF   STATS   ENSEMBL   UCSC 
 
  253
 
  4
 
 24566
 
 island
 
 0.49103
 
 0.03005
 
 Not queried
 
  4.9383953
 
 2.86e-02
 
 1.00e+00
  
 
  
 10
 
  29698152
 
  29698470
 
  PDF   STATS   ENSEMBL   UCSC 
 
  319
 
  8
 
 4991
 
 tss;island
 
 0.30536
 
 0.94316
 
 Not queried
 
  4.9383913
 
 1.04e-02
 
 1.00e+00
  
 
  
 19
 
  36822311
 
  36822770
 
  PDF   STATS   ENSEMBL   UCSC 
 
  460
 
  6
 
 21902
 
 island
 
 0.31672
 
 0.01191
 
 Not queried
 
  4.9338971
 
 2.16e-03
 
 1.00e+00
  
 
  
 16
 
  30572739
 
  30573013
 
  PDF   STATS   ENSEMBL   UCSC 
 
  275
 
  5
 
 15804
 
 island
 
 0.34318
 
 0.94746
 
 Not queried
 
  4.9306043
 
 7.94e-03
 
 1.00e+00
  
 
  
 X
 
 138773906
 
 138774368
 
  PDF   STATS   ENSEMBL   UCSC 
 
  463
 
  5
 
 41128
 
 gene;island
 
 0.03831
 
 0.61008
 
 Not queried
 
  4.9300056
 
 7.94e-03
 
 1.00e+00
  
 
  
 7
 
  74020711
 
  74021054
 
  PDF   STATS   ENSEMBL   UCSC 
 
  344
 
  5
 
 38516
 
 island
 
 0.06315
 
 0.67253
 
 Not queried
 
  4.9292892
 
 7.94e-03
 
 1.00e+00
  
 
  
 12
 
  54378736
 
  54379056
 
  PDF   STATS   ENSEMBL   UCSC 
 
  321
 
  8
 
 10459
 
 island
 
 0.01768
 
 0.34366
 
 Not queried
 
  4.9260025
 
 6.22e-04
 
 1.00e+00
  
 
  
 16
 
  33483244
 
  33483591
 
  PDF   STATS   ENSEMBL   UCSC 
 
  348
 
  5
 
 15856
 
 island
 
 0.09168
 
 0.72276
 
 Not queried
 
  4.9259957
 
 7.94e-03
 
 1.00e+00
  
 
  
 3
 
 112359488
 
 112359652
 
  PDF   STATS   ENSEMBL   UCSC 
 
  165
 
  6
 
 27854
 
 gene
 
 0.12764
 
 0.80963
 
 Not queried
 
  4.9189072
 
 2.16e-03
 
 1.00e+00
  
 
  
 8
 
  24813866
 
  24814137
 
  PDF   STATS   ENSEMBL   UCSC 
 
  272
 
  4
 
 40097
 
 island
 
 0.02576
 
 0.45845
 
 Not queried
 
  4.9171236
 
 2.86e-02
 
 1.00e+00
  
 
  
 15
 
  74658547
 
  74658653
 
  PDF   STATS   ENSEMBL   UCSC 
 
  107
 
  4
 
 13393
 
 gene;island
 
 0.04869
 
 0.49971
 
 Not queried
 
  4.9156962
 
 2.86e-02
 
 1.00e+00
  
 
  
 15
 
  57668039
 
  57668261
 
  PDF   STATS   ENSEMBL   UCSC 
 
  223
 
  4
 
 13666
 
 tss;island
 
 0.93002
 
 0.19687
 
 Not queried
 
  4.9141940
 
 1.14e-01
 
 1.00e+00
  
 
  
 12
 
  49110781
 
  49110861
 
  PDF   STATS   ENSEMBL   UCSC 
 
   81
 
  6
 
 9647
 
 tss
 
 0.36807
 
 0.02198
 
 Not queried
 
  4.9139344
 
 2.16e-03
 
 1.00e+00
  
 
  
 X
 
 101853857
 
 101854594
 
  PDF   STATS   ENSEMBL   UCSC 
 
  738
 
 13
 
 41917
 
 island
 
 0.02866
 
 0.58891
 
 Not queried
 
  4.9131311
 
 7.95e-04
 
 1.00e+00
  
 
  
 5
 
 157098338
 
 157098619
 
  PDF   STATS   ENSEMBL   UCSC 
 
  282
 
  7
 
 33283
 
 island
 
 0.04908
 
 0.58845
 
 Not queried
 
  4.9097769
 
 5.83e-04
 
 1.00e+00
  
 
  
 1
 
 166890293
 
 166890436
 
  PDF   STATS   ENSEMBL   UCSC 
 
  144
 
  4
 
 869
 
 gene;island
 
 0.02190
 
 0.43800
 
 Not queried
 
  4.9086361
 
 2.86e-02
 
 1.00e+00
  
 
  
 7
 
  38350921
 
  38351226
 
  PDF   STATS   ENSEMBL   UCSC 
 
  306
 
  5
 
 38358
 
 island
 
 0.28084
 
 0.90684
 
 Not queried
 
  4.9080713
 
 7.94e-03
 
 1.00e+00
  
 
  
 22
 
  22862802
 
  22863293
 
  PDF   STATS   ENSEMBL   UCSC 
 
  492
 
  5
 
 26766
 
 gene;island
 
 0.56317
 
 0.04384
 
 Not queried
 
  4.9068411
 
 7.94e-03
 
 1.00e+00
  
 
  
 1
 
 233086372
 
 233086622
 
  PDF   STATS   ENSEMBL   UCSC 
 
  251
 
  4
 
 1079
 
 gene
 
 0.37201
 
 0.02550
 
 Not queried
 
  4.9035237
 
 2.86e-02
 
 1.00e+00
  
 
  
 4
 
   4228571
 
   4228650
 
  PDF   STATS   ENSEMBL   UCSC 
 
   80
 
  4
 
 30808
 
 island
 
 0.14744
 
 0.72326
 
 Not queried
 
  4.9025852
 
 2.86e-02
 
 1.00e+00
  
 
  
 14
 
  45722617
 
  45722745
 
  PDF   STATS   ENSEMBL   UCSC 
 
  129
 
  6
 
 12346
 
 tss
 
 0.01784
 
 0.39791
 
 Not queried
 
  4.8996202
 
 2.16e-03
 
 1.00e+00
  
 
  
 1
 
  39546511
 
  39546688
 
  PDF   STATS   ENSEMBL   UCSC 
 
  178
 
  4
 
 1517
 
 tss;island
 
 0.70300
 
 0.06981
 
 Not queried
 
  4.8969393
 
 2.86e-02
 
 1.00e+00
  
 
  
 19
 
  36246395
 
  36246408
 
  PDF   STATS   ENSEMBL   UCSC 
 
   14
 
  4
 
 19958
 
 gene;island
 
 0.18921
 
 0.89072
 
 Not queried
 
  4.8958255
 
 2.86e-02
 
 1.00e+00
  
 
  
 X
 
 108868237
 
 108868465
 
  PDF   STATS   ENSEMBL   UCSC 
 
  229
 
  4
 
 41956
 
 island
 
 0.19841
 
 0.84814
 
 Not queried
 
  4.8942228
 
 2.86e-02
 
 1.00e+00
  
 
  
 8
 
   1796429
 
   1796676
 
  PDF   STATS   ENSEMBL   UCSC 
 
  248
 
  4
 
 39116
 
 gene;island
 
 0.27243
 
 0.91410
 
 Not queried
 
  4.8931272
 
 2.86e-02
 
 1.00e+00
  
 
  
 6
 
  50818685
 
  50819236
 
  PDF   STATS   ENSEMBL   UCSC 
 
  552
 
  4
 
 36138
 
 island
 
 0.89086
 
 0.24117
 
 Not queried
 
  4.8859654
 
 2.86e-02
 
 1.00e+00
  
 
  
 7
 
 128577593
 
 128578297
 
  PDF   STATS   ENSEMBL   UCSC 
 
  705
 
 13
 
 38839
 
 island
 
 0.04883
 
 0.63166
 
 Not queried
 
  4.8834469
 
 4.93e-04
 
 1.00e+00
  
 
  
 1
 
 240656217
 
 240656737
 
  PDF   STATS   ENSEMBL   UCSC 
 
  521
 
  6
 
 1103
 
 gene;island
 
 0.05184
 
 0.55862
 
 Not queried
 
  4.8817189
 
 2.16e-03
 
 1.00e+00
  
 
  
 17
 
  17910235
 
  17910425
 
  PDF   STATS   ENSEMBL   UCSC 
 
  191
 
  4
 
 16434
 
 gene;island
 
 0.05758
 
 0.66351
 
 Not queried
 
  4.8812023
 
 2.86e-02
 
 1.00e+00
  
 
  
 19
 
  11529133
 
  11530065
 
  PDF   STATS   ENSEMBL   UCSC 
 
  933
 
 11
 
 21532
 
 island
 
 0.77825
 
 0.04542
 
 Not queried
 
  4.8782316
 
 2.45e-03
 
 1.00e+00
  
 
  
 7
 
  22539703
 
  22539822
 
  PDF   STATS   ENSEMBL   UCSC 
 
  120
 
  4
 
 36849
 
 gene
 
 0.02233
 
 0.24140
 
 Not queried
 
  4.8762284
 
 2.86e-02
 
 1.00e+00
  
 
  
 16
 
   2801668
 
   2801952
 
  PDF   STATS   ENSEMBL   UCSC 
 
  285
 
  5
 
 14583
 
 gene;tss;island
 
 0.68681
 
 0.05416
 
 Not queried
 
  4.8760980
 
 7.94e-03
 
 1.00e+00
  
 
  
 12
 
  11422897
 
  11423120
 
  PDF   STATS   ENSEMBL   UCSC 
 
  224
 
  4
 
 9541
 
 tss
 
 0.05582
 
 0.60120
 
 Not queried
 
  4.8739054
 
 5.71e-02
 
 1.00e+00
  
 
  
 16
 
  23765810
 
  23766116
 
  PDF   STATS   ENSEMBL   UCSC 
 
  307
 
  5
 
 15737
 
 island
 
 0.06244
 
 0.66120
 
 Not queried
 
  4.8730884
 
 7.94e-03
 
 1.00e+00
  
 
  
 4
 
 113970617
 
 113970935
 
  PDF   STATS   ENSEMBL   UCSC 
 
  319
 
  6
 
 30056
 
 gene
 
 0.76709
 
 0.09501
 
 Not queried
 
  4.8698557
 
 2.16e-03
 
 1.00e+00
  
 
  
 19
 
  11784246
 
  11784774
 
  PDF   STATS   ENSEMBL   UCSC 
 
  529
 
  9
 
 20443
 
 tss
 
 0.02711
 
 0.47910
 
 Not queried
 
  4.8560749
 
 4.94e-04
 
 1.00e+00
  
 
  
 10
 
  72141375
 
  72141625
 
  PDF   STATS   ENSEMBL   UCSC 
 
  251
 
  4
 
 4604
 
 gene;island
 
 0.86035
 
 0.14899
 
 Not queried
 
  4.8521694
 
 2.86e-02
 
 1.00e+00
  
 
  
 8
 
  11555178
 
  11555548
 
  PDF   STATS   ENSEMBL   UCSC 
 
  371
 
  4
 
 40020
 
 island
 
 0.68799
 
 0.09130
 
 Not queried
 
  4.8480820
 
 2.86e-02
 
 1.00e+00
  
 
  
 8
 
 144640306
 
 144640403
 
  PDF   STATS   ENSEMBL   UCSC 
 
   98
 
  6
 
 39888
 
 tss
 
 0.18166
 
 0.86626
 
 Not queried
 
  4.8448773
 
 2.16e-03
 
 1.00e+00
  
 
  
 X
 
 101853857
 
 101854377
 
  PDF   STATS   ENSEMBL   UCSC 
 
  521
 
 11
 
 41431
 
 tss
 
 0.04539
 
 0.58891
 
 Not queried
 
  4.8443319
 
 5.24e-03
 
 1.00e+00
  
 
  
 2
 
  45397776
 
  45398036
 
  PDF   STATS   ENSEMBL   UCSC 
 
  261
 
  4
 
 24232
 
 island
 
 0.90480
 
 0.18618
 
 Not queried
 
  4.8438132
 
 2.86e-02
 
 1.00e+00
  
 
  
 3
 
 124448994
 
 124449432
 
  PDF   STATS   ENSEMBL   UCSC 
 
  439
 
  7
 
 29374
 
 island
 
 0.57160
 
 0.04655
 
 Not queried
 
  4.8317837
 
 5.83e-04
 
 1.00e+00
  
 
  
 4
 
   1049609
 
   1049922
 
  PDF   STATS   ENSEMBL   UCSC 
 
  314
 
  4
 
 30717
 
 island
 
 0.09937
 
 0.82922
 
 Not queried
 
  4.8296617
 
 5.71e-02
 
 1.00e+00
  
 
  
 10
 
  88730324
 
  88730599
 
  PDF   STATS   ENSEMBL   UCSC 
 
  276
 
  4
 
 4650
 
 gene;island
 
 0.42144
 
 0.93714
 
 Not queried
 
  4.8251608
 
 2.86e-02
 
 1.00e+00
  
 
  
 8
 
  27182871
 
  27182965
 
  PDF   STATS   ENSEMBL   UCSC 
 
   95
 
  5
 
 39549
 
 tss
 
 0.03169
 
 0.53656
 
 Not queried
 
  4.8238628
 
 7.94e-03
 
 1.00e+00
  
 
  
 1
 
  33592870
 
  33593154
 
  PDF   STATS   ENSEMBL   UCSC 
 
  285
 
  5
 
 3002
 
 island
 
 0.13170
 
 0.89095
 
 Not queried
 
  4.8230492
 
 7.94e-03
 
 1.00e+00
  
 
  
 11
 
  58912052
 
  58912327
 
  PDF   STATS   ENSEMBL   UCSC 
 
  276
 
  6
 
 6601
 
 gene;island
 
 0.70944
 
 0.11941
 
 Not queried
 
  4.8226461
 
 2.16e-03
 
 1.00e+00
  
 
  
 19
 
  12267592
 
  12267796
 
  PDF   STATS   ENSEMBL   UCSC 
 
  205
 
  6
 
 20454
 
 tss
 
 0.03679
 
 0.47268
 
 Not queried
 
  4.8215029
 
 2.16e-03
 
 1.00e+00
  
 
  
 14
 
  80328091
 
  80328595
 
  PDF   STATS   ENSEMBL   UCSC 
 
  505
 
  5
 
 12132
 
 gene;island
 
 0.32891
 
 0.92483
 
 Not queried
 
  4.8187679
 
 3.17e-02
 
 1.00e+00
  
 
  
 13
 
  69558928
 
  69559303
 
  PDF   STATS   ENSEMBL   UCSC 
 
  376
 
  4
 
 11741
 
 island
 
 0.08541
 
 0.72925
 
 Not queried
 
  4.8155609
 
 2.86e-02
 
 1.00e+00
  
 
  
 2
 
 172378774
 
 172379087
 
  PDF   STATS   ENSEMBL   UCSC 
 
  314
 
  4
 
 24816
 
 island
 
 0.47261
 
 0.02671
 
 Not queried
 
  4.8155012
 
 2.86e-02
 
 1.00e+00
  
 
  
 19
 
  54676948
 
  54677232
 
  PDF   STATS   ENSEMBL   UCSC 
 
  285
 
  6
 
 21019
 
 tss
 
 0.05519
 
 0.51226
 
 Not queried
 
  4.8143889
 
 1.52e-02
 
 1.00e+00
  
 
  
 19
 
  12969204
 
  12969611
 
  PDF   STATS   ENSEMBL   UCSC 
 
  408
 
  5
 
 19834
 
 gene;island
 
 0.27616
 
 0.91550
 
 Not queried
 
  4.8067632
 
 7.94e-03
 
 1.00e+00
  
 
  
 11
 
 124823738
 
 124823917
 
  PDF   STATS   ENSEMBL   UCSC 
 
  180
 
  7
 
 7766
 
 tss;island
 
 0.01361
 
 0.27852
 
 Not queried
 
  4.8066668
 
 5.83e-04
 
 1.00e+00
  
 
  
 16
 
    745663
 
    745765
 
  PDF   STATS   ENSEMBL   UCSC 
 
  103
 
  4
 
 14510
 
 gene;island
 
 0.05459
 
 0.53827
 
 Not queried
 
  4.8064973
 
 2.86e-02
 
 1.00e+00
  
 
  
 17
 
  14207530
 
  14207968
 
  PDF   STATS   ENSEMBL   UCSC 
 
  439
 
  5
 
 16415
 
 gene;island
 
 0.54779
 
 0.07530
 
 Not queried
 
  4.8051825
 
 5.56e-02
 
 1.00e+00
  
 
  
 17
 
  46688182
 
  46688329
 
  PDF   STATS   ENSEMBL   UCSC 
 
  148
 
  4
 
 16624
 
 gene
 
 0.00602
 
 0.09668
 
 Not queried
 
  4.8024376
 
 2.86e-02
 
 1.00e+00
  
 
  
 17
 
  37895771
 
  37896175
 
  PDF   STATS   ENSEMBL   UCSC 
 
  405
 
  5
 
 16521
 
 gene
 
 0.02879
 
 0.33431
 
 Not queried
 
  4.7983838
 
 3.17e-02
 
 1.00e+00
  
 
  
 11
 
  68924577
 
  68924956
 
  PDF   STATS   ENSEMBL   UCSC 
 
  380
 
  5
 
 8564
 
 island
 
 0.12108
 
 0.81873
 
 Not queried
 
  4.7945138
 
 7.94e-03
 
 1.00e+00
  
 
  
 5
 
    533963
 
    534332
 
  PDF   STATS   ENSEMBL   UCSC 
 
  370
 
  4
 
 32528
 
 island
 
 0.13286
 
 0.85909
 
 Not queried
 
  4.7936973
 
 2.86e-02
 
 1.00e+00
  
 
  
 12
 
 131303478
 
 131303774
 
  PDF   STATS   ENSEMBL   UCSC 
 
  297
 
  4
 
 9393
 
 gene;island
 
 0.04748
 
 0.24729
 
 Not queried
 
  4.7934142
 
 2.86e-02
 
 1.00e+00
  
 
  
 7
 
  45197251
 
  45197346
 
  PDF   STATS   ENSEMBL   UCSC 
 
   96
 
  4
 
 37518
 
 tss
 
 0.06826
 
 0.77055
 
 Not queried
 
  4.7892480
 
 2.86e-02
 
 1.00e+00
  
 
  
 10
 
  22624663
 
  22625015
 
  PDF   STATS   ENSEMBL   UCSC 
 
  353
 
  4
 
 5583
 
 island
 
 0.93178
 
 0.25957
 
 Not queried
 
  4.7862120
 
 2.86e-02
 
 1.00e+00
  
 
  
 5
 
 140810051
 
 140810137
 
  PDF   STATS   ENSEMBL   UCSC 
 
   87
 
  5
 
 32324
 
 tss
 
 0.16812
 
 0.88544
 
 Not queried
 
  4.7797385
 
 7.94e-03
 
 1.00e+00
  
 
  
 5
 
 137225191
 
 137225509
 
  PDF   STATS   ENSEMBL   UCSC 
 
  319
 
  5
 
 31706
 
 gene;island
 
 0.01617
 
 0.45407
 
 Not queried
 
  4.7780865
 
 4.21e-01
 
 1.00e+00
  
 
  
 2
 
  27805893
 
  27806117
 
  PDF   STATS   ENSEMBL   UCSC 
 
  225
 
  4
 
 22594
 
 gene
 
 0.95876
 
 0.39622
 
 Not queried
 
  4.7774759
 
 2.86e-02
 
 1.00e+00
  
 
  
 5
 
 162931471
 
 162931829
 
  PDF   STATS   ENSEMBL   UCSC 
 
  359
 
  4
 
 31823
 
 gene;tss;island
 
 0.23648
 
 0.01250
 
 Not queried
 
  4.7769555
 
 2.86e-02
 
 1.00e+00
  
 
  
 19
 
  51171640
 
  51172144
 
  PDF   STATS   ENSEMBL   UCSC 
 
  505
 
  5
 
 20145
 
 gene;island
 
 0.07810
 
 0.69250
 
 Not queried
 
  4.7750397
 
 7.94e-03
 
 1.00e+00
  
 
  
 2
 
    264120
 
    264204
 
  PDF   STATS   ENSEMBL   UCSC 
 
   85
 
  6
 
 23115
 
 tss;island
 
 0.03705
 
 0.45629
 
 Not queried
 
  4.7744755
 
 2.16e-03
 
 1.00e+00
  
 
  
 5
 
 145713607
 
 145713882
 
  PDF   STATS   ENSEMBL   UCSC 
 
  276
 
  6
 
 33217
 
 island
 
 0.03759
 
 0.54499
 
 Not queried
 
  4.7743873
 
 2.16e-03
 
 1.00e+00
  
 
  
 2
 
 162272107
 
 162272687
 
  PDF   STATS   ENSEMBL   UCSC 
 
  581
 
  7
 
 24783
 
 island
 
 0.06224
 
 0.71162
 
 Not queried
 
  4.7719837
 
 5.83e-04
 
 1.00e+00
  
 
  
 6
 
 111408745
 
 111408840
 
  PDF   STATS   ENSEMBL   UCSC 
 
   96
 
  6
 
 36356
 
 island
 
 0.55064
 
 0.03444
 
 Not queried
 
  4.7699964
 
 2.16e-03
 
 1.00e+00
  
 
  
 11
 
 119293601
 
 119293869
 
  PDF   STATS   ENSEMBL   UCSC 
 
  269
 
  5
 
 6921
 
 gene;island
 
 0.72680
 
 0.04512
 
 Not queried
 
  4.7695543
 
 1.59e-02
 
 1.00e+00
  
 
  
 15
 
  40268421
 
  40268777
 
  PDF   STATS   ENSEMBL   UCSC 
 
  357
 
  4
 
 13257
 
 gene;island
 
 0.02310
 
 0.37012
 
 Not queried
 
  4.7677795
 
 1.14e-01
 
 1.00e+00
  
 
  
 7
 
  98245716
 
  98246006
 
  PDF   STATS   ENSEMBL   UCSC 
 
  291
 
  4
 
 37682
 
 tss;island
 
 0.05664
 
 0.53646
 
 Not queried
 
  4.7635903
 
 2.86e-02
 
 1.00e+00
  
 
  
 6
 
  24494462
 
  24494793
 
  PDF   STATS   ENSEMBL   UCSC 
 
  332
 
  5
 
 34604
 
 tss;island
 
 0.74351
 
 0.09131
 
 Not queried
 
  4.7633408
 
 7.94e-03
 
 1.00e+00
  
 
  
 3
 
 124449238
 
 124449432
 
  PDF   STATS   ENSEMBL   UCSC 
 
  195
 
  4
 
 27882
 
 gene
 
 0.55171
 
 0.04977
 
 Not queried
 
  4.7627762
 
 2.86e-02
 
 1.00e+00
  
 
  
 4
 
 174459743
 
 174460124
 
  PDF   STATS   ENSEMBL   UCSC 
 
  382
 
  4
 
 30135
 
 gene;island
 
 0.60329
 
 0.05360
 
 Not queried
 
  4.7577786
 
 2.86e-02
 
 1.00e+00
  
 
  
 17
 
  46622454
 
  46622899
 
  PDF   STATS   ENSEMBL   UCSC 
 
  446
 
  8
 
 17424
 
 tss;island
 
 0.75709
 
 0.08148
 
 Not queried
 
  4.7513990
 
 2.95e-03
 
 1.00e+00
  
 
  
 14
 
  61789466
 
  61789733
 
  PDF   STATS   ENSEMBL   UCSC 
 
  268
 
  5
 
 12079
 
 gene;island
 
 0.02199
 
 0.28817
 
 Not queried
 
  4.7497609
 
 7.94e-03
 
 1.00e+00
  
 
  
 X
 
 153084485
 
 153085101
 
  PDF   STATS   ENSEMBL   UCSC 
 
  617
 
  5
 
 41150
 
 gene;island
 
 0.05975
 
 0.56275
 
 Not queried
 
  4.7445311
 
 5.56e-02
 
 1.00e+00
  
 
  
 Y
 
   2655866
 
   2655977
 
  PDF   STATS   ENSEMBL   UCSC 
 
  112
 
  4
 
 42163
 
 tss
 
 0.02194
 
 0.15051
 
 Not queried
 
  4.7428352
 
 6.86e-01
 
 1.00e+00
  
 
  
 1
 
 108023249
 
 108023482
 
  PDF   STATS   ENSEMBL   UCSC 
 
  234
 
  5
 
 659
 
 gene;island
 
 0.28696
 
 0.91002
 
 Not queried
 
  4.7426634
 
 7.94e-03
 
 1.00e+00
  
 
  
 5
 
 140767197
 
 140767412
 
  PDF   STATS   ENSEMBL   UCSC 
 
  216
 
  7
 
 31770
 
 gene;tss;island
 
 0.02196
 
 0.44439
 
 Not queried
 
  4.7411959
 
 1.17e-03
 
 1.00e+00
  
 
  
 6
 
   5851644
 
   5851994
 
  PDF   STATS   ENSEMBL   UCSC 
 
  351
 
  5
 
 35337
 
 island
 
 0.79716
 
 0.09337
 
 Not queried
 
  4.7402400
 
 7.94e-03
 
 1.00e+00
  
 
  
 22
 
  44258572
 
  44258712
 
  PDF   STATS   ENSEMBL   UCSC 
 
  141
 
  5
 
 27163
 
 tss;island
 
 0.03767
 
 0.70854
 
 Not queried
 
  4.7383501
 
 7.94e-03
 
 1.00e+00
  
 
  
 13
 
  50070436
 
  50070766
 
  PDF   STATS   ENSEMBL   UCSC 
 
  331
 
  8
 
 11138
 
 gene;island
 
 0.57020
 
 0.06222
 
 Not queried
 
  4.7373574
 
 1.86e-03
 
 1.00e+00
  
 
  
 17
 
  22016686
 
  22017365
 
  PDF   STATS   ENSEMBL   UCSC 
 
  680
 
  6
 
 18096
 
 island
 
 0.04587
 
 0.48073
 
 Not queried
 
  4.7365554
 
 2.16e-03
 
 1.00e+00
  
 
  
 4
 
  85417312
 
  85417755
 
  PDF   STATS   ENSEMBL   UCSC 
 
  444
 
  4
 
 30001
 
 gene;island
 
 0.64838
 
 0.06623
 
 Not queried
 
  4.7324399
 
 2.86e-02
 
 1.00e+00
  
 
  
 X
 
  74376241
 
  74376313
 
  PDF   STATS   ENSEMBL   UCSC 
 
   73
 
  4
 
 41394
 
 tss
 
 0.04589
 
 0.59846
 
 Not queried
 
  4.7309289
 
 2.86e-02
 
 1.00e+00
  
 
  
 6
 
 170578592
 
 170578855
 
  PDF   STATS   ENSEMBL   UCSC 
 
  264
 
  4
 
 36622
 
 island
 
 0.16276
 
 0.78834
 
 Not queried
 
  4.7264495
 
 2.86e-02
 
 1.00e+00
  
 
  
 X
 
  73164102
 
  73164390
 
  PDF   STATS   ENSEMBL   UCSC 
 
  289
 
  6
 
 41873
 
 island
 
 0.02724
 
 0.32240
 
 Not queried
 
  4.7243948
 
 2.16e-03
 
 1.00e+00
  
 
  
 2
 
  63281069
 
  63281383
 
  PDF   STATS   ENSEMBL   UCSC 
 
  315
 
  4
 
 22667
 
 gene;island
 
 0.84976
 
 0.16793
 
 Not queried
 
  4.7237336
 
 2.86e-02
 
 1.00e+00
  
 
  
 7
 
 128577593
 
 128578176
 
  PDF   STATS   ENSEMBL   UCSC 
 
  584
 
 12
 
 37822
 
 tss
 
 0.06221
 
 0.63632
 
 Not queried
 
  4.7237058
 
 1.11e-03
 
 1.00e+00
  
 
  
 6
 
 138866865
 
 138867125
 
  PDF   STATS   ENSEMBL   UCSC 
 
  261
 
  7
 
 34424
 
 gene
 
 0.67034
 
 0.09471
 
 Not queried
 
  4.7166899
 
 4.08e-03
 
 1.00e+00
  
 
  
 14
 
  37051693
 
  37051949
 
  PDF   STATS   ENSEMBL   UCSC 
 
  257
 
  7
 
 12759
 
 island
 
 0.03948
 
 0.57869
 
 Not queried
 
  4.7160790
 
 5.83e-04
 
 1.00e+00
  
 
  
 1
 
   6526049
 
   6526583
 
  PDF   STATS   ENSEMBL   UCSC 
 
  535
 
 12
 
 173
 
 gene;island
 
 0.09128
 
 0.73248
 
 Not queried
 
  4.7125456
 
 3.64e-03
 
 1.00e+00
  
 
  
 16
 
  14397576
 
  14397892
 
  PDF   STATS   ENSEMBL   UCSC 
 
  317
 
  6
 
 15689
 
 island
 
 0.45233
 
 0.03277
 
 Not queried
 
  4.7121470
 
 2.16e-03
 
 1.00e+00
  
 
  
 15
 
  68117903
 
  68118127
 
  PDF   STATS   ENSEMBL   UCSC 
 
  225
 
  4
 
 14224
 
 island
 
 0.04174
 
 0.48739
 
 Not queried
 
  4.7066281
 
 2.86e-02
 
 1.00e+00
  
 
  
 13
 
 111268673
 
 111268714
 
  PDF   STATS   ENSEMBL   UCSC 
 
   42
 
  4
 
 11204
 
 gene;island
 
 0.60239
 
 0.06773
 
 Not queried
 
  4.7014483
 
 2.86e-02
 
 1.00e+00
  
 
  
 10
 
 118386951
 
 118387518
 
  PDF   STATS   ENSEMBL   UCSC 
 
  568
 
  5
 
 4761
 
 gene;island
 
 0.13292
 
 0.79943
 
 Not queried
 
  4.7003790
 
 7.94e-03
 
 1.00e+00
  
 
  
 12
 
  49110427
 
  49110646
 
  PDF   STATS   ENSEMBL   UCSC 
 
  220
 
  4
 
 9088
 
 gene
 
 0.38727
 
 0.01959
 
 Not queried
 
  4.6977352
 
 2.86e-02
 
 1.00e+00
  
 
  
 6
 
  26020672
 
  26020714
 
  PDF   STATS   ENSEMBL   UCSC 
 
   43
 
  4
 
 34614
 
 tss;island
 
 0.58043
 
 0.96888
 
 Not queried
 
  4.6948728
 
 2.86e-02
 
 1.00e+00
  
 
  
 X
 
 134165954
 
 134166287
 
  PDF   STATS   ENSEMBL   UCSC 
 
  334
 
  7
 
 41537
 
 tss
 
 0.03946
 
 0.52308
 
 Not queried
 
  4.6933363
 
 5.83e-04
 
 1.00e+00
  
 
  
 X
 
  49022793
 
  49023115
 
  PDF   STATS   ENSEMBL   UCSC 
 
  323
 
  5
 
 40993
 
 gene;island
 
 0.43453
 
 0.04132
 
 Not queried
 
  4.6921926
 
 7.94e-03
 
 1.00e+00
  
 
  
 10
 
  93392644
 
  93392839
 
  PDF   STATS   ENSEMBL   UCSC 
 
  196
 
  4
 
 4659
 
 gene
 
 0.14399
 
 0.79183
 
 Not queried
 
  4.6893608
 
 2.86e-02
 
 1.00e+00
  
 
  
 2
 
 219773416
 
 219774183
 
  PDF   STATS   ENSEMBL   UCSC 
 
  768
 
  8
 
 25057
 
 island
 
 0.02239
 
 0.38885
 
 Not queried
 
  4.6858648
 
 3.11e-04
 
 1.00e+00
  
 
  
 15
 
  29033879
 
  29033901
 
  PDF   STATS   ENSEMBL   UCSC 
 
   23
 
  6
 
 13943
 
 island
 
 0.35560
 
 0.90957
 
 Not queried
 
  4.6854136
 
 2.16e-03
 
 1.00e+00
  
 
  
 10
 
  47151300
 
  47151589
 
  PDF   STATS   ENSEMBL   UCSC 
 
  290
 
  5
 
 5702
 
 island
 
 0.02682
 
 0.39541
 
 Not queried
 
  4.6853112
 
 7.94e-03
 
 1.00e+00
  
 
  
 X
 
 153656771
 
 153656930
 
  PDF   STATS   ENSEMBL   UCSC 
 
  160
 
  4
 
 41599
 
 tss
 
 0.04837
 
 0.57339
 
 Not queried
 
  4.6840829
 
 2.86e-02
 
 1.00e+00
  
 
  
 12
 
  54070891
 
  54071194
 
  PDF   STATS   ENSEMBL   UCSC 
 
  304
 
  7
 
 9721
 
 tss;island
 
 0.01587
 
 0.29912
 
 Not queried
 
  4.6776339
 
 5.83e-04
 
 1.00e+00
  
 
  
 2
 
 203103432
 
 203103763
 
  PDF   STATS   ENSEMBL   UCSC 
 
  332
 
  5
 
 23786
 
 tss;island
 
 0.00950
 
 0.17276
 
 Not queried
 
  4.6759361
 
 1.51e-01
 
 1.00e+00
  
 
  
 2
 
 131185333
 
 131185700
 
  PDF   STATS   ENSEMBL   UCSC 
 
  368
 
  6
 
 24682
 
 island
 
 0.08931
 
 0.65969
 
 Not queried
 
  4.6721966
 
 2.16e-03
 
 1.00e+00
  
 
  
 3
 
  50275394
 
  50275694
 
  PDF   STATS   ENSEMBL   UCSC 
 
  301
 
  5
 
 27754
 
 gene;island
 
 0.32729
 
 0.01950
 
 Not queried
 
  4.6678415
 
 7.94e-03
 
 1.00e+00
  
 
  
 3
 
 192444985
 
 192445380
 
  PDF   STATS   ENSEMBL   UCSC 
 
  396
 
  5
 
 28044
 
 gene
 
 0.02891
 
 0.46506
 
 Not queried
 
  4.6667041
 
 3.17e-02
 
 1.00e+00
  
 
  
 20
 
  44451667
 
  44451973
 
  PDF   STATS   ENSEMBL   UCSC 
 
  307
 
  4
 
 26163
 
 island
 
 0.07554
 
 0.70694
 
 Not queried
 
  4.6647461
 
 2.86e-02
 
 1.00e+00
  
 
  
 1
 
  55445834
 
  55446387
 
  PDF   STATS   ENSEMBL   UCSC 
 
  554
 
  7
 
 1647
 
 tss
 
 0.07771
 
 0.76319
 
 Not queried
 
  4.6635416
 
 2.62e-02
 
 1.00e+00
  
 
  
 12
 
 133177383
 
 133177827
 
  PDF   STATS   ENSEMBL   UCSC 
 
  445
 
  4
 
 11036
 
 island
 
 0.10846
 
 0.71139
 
 Not queried
 
  4.6599184
 
 2.86e-02
 
 1.00e+00
  
 
  
 19
 
  56915403
 
  56915658
 
  PDF   STATS   ENSEMBL   UCSC 
 
  256
 
  6
 
 21061
 
 tss
 
 0.53482
 
 0.04028
 
 Not queried
 
  4.6545983
 
 2.16e-03
 
 1.00e+00
  
 
  
 4
 
 174427388
 
 174428191
 
  PDF   STATS   ENSEMBL   UCSC 
 
  804
 
  9
 
 31370
 
 island
 
 0.14619
 
 0.74207
 
 Not queried
 
  4.6536796
 
 4.94e-04
 
 1.00e+00
  
 
  
 7
 
 157690609
 
 157690769
 
  PDF   STATS   ENSEMBL   UCSC 
 
  161
 
  4
 
 37286
 
 gene;island
 
 0.32626
 
 0.83345
 
 Not queried
 
  4.6500936
 
 2.86e-02
 
 1.00e+00
  
 
  
 7
 
   3033408
 
   3033939
 
  PDF   STATS   ENSEMBL   UCSC 
 
  532
 
  5
 
 36760
 
 gene
 
 0.27220
 
 0.90148
 
 Not queried
 
  4.6500114
 
 7.94e-03
 
 1.00e+00
  
 
  
 5
 
 134181484
 
 134181607
 
  PDF   STATS   ENSEMBL   UCSC 
 
  124
 
  6
 
 32240
 
 tss
 
 0.01147
 
 0.31932
 
 Not queried
 
  4.6486266
 
 2.16e-03
 
 1.00e+00
  
 
  
 17
 
  56738915
 
  56739129
 
  PDF   STATS   ENSEMBL   UCSC 
 
  215
 
  5
 
 16676
 
 gene;island
 
 0.22515
 
 0.94241
 
 Not queried
 
  4.6438566
 
 7.94e-03
 
 1.00e+00
  
 
  
 3
 
   8810980
 
   8811279
 
  PDF   STATS   ENSEMBL   UCSC 
 
  300
 
  4
 
 27605
 
 gene
 
 0.02427
 
 0.36286
 
 Not queried
 
  4.6432207
 
 3.43e-01
 
 1.00e+00
  
 
  
 10
 
  22726610
 
  22726885
 
  PDF   STATS   ENSEMBL   UCSC 
 
  276
 
  6
 
 5587
 
 island
 
 0.54736
 
 0.04777
 
 Not queried
 
  4.6404821
 
 2.16e-03
 
 1.00e+00
  
 
  
 3
 
  54157557
 
  54157900
 
  PDF   STATS   ENSEMBL   UCSC 
 
  344
 
  5
 
 27793
 
 gene;island
 
 0.02001
 
 0.31685
 
 Not queried
 
  4.6348769
 
 7.94e-03
 
 1.00e+00
  
 
  
 5
 
 151304770
 
 151305143
 
  PDF   STATS   ENSEMBL   UCSC 
 
  374
 
  6
 
 32386
 
 tss;island
 
 0.04860
 
 0.53672
 
 Not queried
 
  4.6342189
 
 8.66e-03
 
 1.00e+00
  
 
  
 12
 
 115124584
 
 115125098
 
  PDF   STATS   ENSEMBL   UCSC 
 
  515
 
  5
 
 10820
 
 island
 
 0.73025
 
 0.11167
 
 Not queried
 
  4.6296736
 
 7.94e-03
 
 1.00e+00
  
 
  
 12
 
 107348689
 
 107349191
 
  PDF   STATS   ENSEMBL   UCSC 
 
  503
 
  7
 
 9946
 
 tss;island
 
 0.68126
 
 0.02613
 
 Not queried
 
  4.6287298
 
 2.59e-01
 
 1.00e+00
  
 
  
 X
 
 153990478
 
 153991009
 
  PDF   STATS   ENSEMBL   UCSC 
 
  532
 
  9
 
 41608
 
 tss
 
 0.02661
 
 0.54119
 
 Not queried
 
  4.6281191
 
 2.76e-03
 
 1.00e+00
  
 
  
 5
 
 172661803
 
 172662130
 
  PDF   STATS   ENSEMBL   UCSC 
 
  328
 
  4
 
 31845
 
 gene
 
 0.18195
 
 0.85955
 
 Not queried
 
  4.6271342
 
 2.86e-02
 
 1.00e+00
  
 
  
 6
 
  34206400
 
  34206683
 
  PDF   STATS   ENSEMBL   UCSC 
 
  284
 
  4
 
 34237
 
 gene;island
 
 0.51585
 
 0.02690
 
 Not queried
 
  4.6268459
 
 1.14e-01
 
 1.00e+00
  
 
  
 5
 
  44388799
 
  44389062
 
  PDF   STATS   ENSEMBL   UCSC 
 
  264
 
  6
 
 31993
 
 tss
 
 0.04644
 
 0.52527
 
 Not queried
 
  4.6256839
 
 2.16e-03
 
 1.00e+00
  
 
  
 22
 
  33195343
 
  33195688
 
  PDF   STATS   ENSEMBL   UCSC 
 
  346
 
  6
 
 26808
 
 gene;tss;island
 
 0.53274
 
 0.95858
 
 Not queried
 
  4.6226698
 
 2.16e-03
 
 1.00e+00
  
 
  
 7
 
  92672812
 
  92673176
 
  PDF   STATS   ENSEMBL   UCSC 
 
  365
 
  5
 
 38601
 
 island
 
 0.51182
 
 0.01859
 
 Not queried
 
  4.6202970
 
 7.94e-03
 
 1.00e+00
  
 
  
 17
 
   8230130
 
   8230940
 
  PDF   STATS   ENSEMBL   UCSC 
 
  811
 
  7
 
 17968
 
 island
 
 0.22522
 
 0.87869
 
 Not queried
 
  4.6201159
 
 6.99e-03
 
 1.00e+00
  
 
  
 10
 
  22634432
 
  22634733
 
  PDF   STATS   ENSEMBL   UCSC 
 
  302
 
  5
 
 4517
 
 gene;island
 
 0.05389
 
 0.58307
 
 Not queried
 
  4.6177291
 
 7.94e-03
 
 1.00e+00
  
 
  
 6
 
  99291343
 
  99291677
 
  PDF   STATS   ENSEMBL   UCSC 
 
  335
 
  4
 
 36284
 
 island
 
 0.54792
 
 0.03721
 
 Not queried
 
  4.6142261
 
 2.86e-02
 
 1.00e+00
  
 
  
 11
 
  35547074
 
  35547311
 
  PDF   STATS   ENSEMBL   UCSC 
 
  238
 
  4
 
 8188
 
 island
 
 0.61139
 
 0.07318
 
 Not queried
 
  4.6131791
 
 2.86e-02
 
 1.00e+00
  
 
  
 X
 
  68835489
 
  68835895
 
  PDF   STATS   ENSEMBL   UCSC 
 
  407
 
  7
 
 41362
 
 tss
 
 0.09679
 
 0.00772
 
 Not queried
 
  4.6081307
 
 1.11e-02
 
 1.00e+00
  
 
  
 5
 
 140098174
 
 140098496
 
  PDF   STATS   ENSEMBL   UCSC 
 
  323
 
  6
 
 32291
 
 tss
 
 0.02740
 
 0.29454
 
 Not queried
 
  4.6070965
 
 2.16e-03
 
 1.00e+00
  
 
  
 2
 
 162273011
 
 162273648
 
  PDF   STATS   ENSEMBL   UCSC 
 
  638
 
  7
 
 22872
 
 gene;island
 
 0.13539
 
 0.80642
 
 Not queried
 
  4.6060379
 
 5.83e-04
 
 1.00e+00
  
 
  
 12
 
  63195695
 
  63195954
 
  PDF   STATS   ENSEMBL   UCSC 
 
  260
 
  4
 
 9175
 
 gene;island
 
 0.36942
 
 0.92528
 
 Not queried
 
  4.6036593
 
 2.86e-02
 
 1.00e+00
  
 
  
 13
 
  20392406
 
  20392706
 
  PDF   STATS   ENSEMBL   UCSC 
 
  301
 
  5
 
 11534
 
 island
 
 0.06805
 
 0.66666
 
 Not queried
 
  4.6000069
 
 7.94e-03
 
 1.00e+00
  
 
  
 12
 
   6484521
 
   6484729
 
  PDF   STATS   ENSEMBL   UCSC 
 
  209
 
  4
 
 9002
 
 gene;tss
 
 0.05482
 
 0.55220
 
 Not queried
 
  4.5993204
 
 2.86e-02
 
 1.00e+00
  
 
  
 2
 
  33701120
 
  33701319
 
  PDF   STATS   ENSEMBL   UCSC 
 
  200
 
  4
 
 22605
 
 gene;tss
 
 0.02913
 
 0.41149
 
 Not queried
 
  4.5989179
 
 2.86e-02
 
 1.00e+00
  
 
  
 10
 
 118429481
 
 118429952
 
  PDF   STATS   ENSEMBL   UCSC 
 
  472
 
  7
 
 5369
 
 tss
 
 0.05003
 
 0.57135
 
 Not queried
 
  4.5949159
 
 5.83e-04
 
 1.00e+00
  
 
  
 17
 
  36105117
 
  36105517
 
  PDF   STATS   ENSEMBL   UCSC 
 
  401
 
  6
 
 17258
 
 tss
 
 0.66036
 
 0.08689
 
 Not queried
 
  4.5935393
 
 2.16e-03
 
 1.00e+00
  
 
  
 8
 
 145027948
 
 145028505
 
  PDF   STATS   ENSEMBL   UCSC 
 
  558
 
 10
 
 39441
 
 gene;island
 
 0.56490
 
 0.03744
 
 Not queried
 
  4.5928725
 
 3.89e-03
 
 1.00e+00
  
 
  
 19
 
  58570419
 
  58570491
 
  PDF   STATS   ENSEMBL   UCSC 
 
   73
 
  6
 
 21095
 
 tss;island
 
 0.02544
 
 0.38647
 
 Not queried
 
  4.5926627
 
 2.16e-03
 
 1.00e+00
  
 
  
 3
 
  35720994
 
  35721284
 
  PDF   STATS   ENSEMBL   UCSC 
 
  291
 
  5
 
 27670
 
 gene;tss
 
 0.04314
 
 0.50425
 
 Not queried
 
  4.5911648
 
 7.94e-03
 
 1.00e+00
  
 
  
 11
 
  66360740
 
  66360803
 
  PDF   STATS   ENSEMBL   UCSC 
 
   64
 
  4
 
 6716
 
 gene
 
 0.01831
 
 0.27685
 
 Not queried
 
  4.5910503
 
 2.86e-02
 
 1.00e+00
  
 
  
 21
 
  26934197
 
  26934682
 
  PDF   STATS   ENSEMBL   UCSC 
 
  486
 
  6
 
 26574
 
 island
 
 0.04629
 
 0.60903
 
 Not queried
 
  4.5900743
 
 2.16e-03
 
 1.00e+00
  
 
  
 21
 
  42792127
 
  42792703
 
  PDF   STATS   ENSEMBL   UCSC 
 
  577
 
  6
 
 26648
 
 island
 
 0.03277
 
 0.35871
 
 Not queried
 
  4.5897067
 
 8.66e-03
 
 1.00e+00
  
 
  
 13
 
  51417686
 
  51418221
 
  PDF   STATS   ENSEMBL   UCSC 
 
  536
 
 10
 
 11716
 
 island
 
 0.06150
 
 0.54352
 
 Not queried
 
  4.5885045
 
 1.50e-03
 
 1.00e+00
  
 
  
 8
 
  56433594
 
  56433717
 
  PDF   STATS   ENSEMBL   UCSC 
 
  124
 
  4
 
 39246
 
 gene
 
 0.02747
 
 0.27553
 
 Not queried
 
  4.5884609
 
 2.86e-02
 
 1.00e+00
  
 
  
 5
 
   3590820
 
   3591171
 
  PDF   STATS   ENSEMBL   UCSC 
 
  352
 
  4
 
 32604
 
 island
 
 0.05826
 
 0.63142
 
 Not queried
 
  4.5842037
 
 2.86e-02
 
 1.00e+00
  
 
  
 22
 
  17850431
 
  17850690
 
  PDF   STATS   ENSEMBL   UCSC 
 
  260
 
  4
 
 27231
 
 island
 
 0.39155
 
 0.04515
 
 Not queried
 
  4.5825576
 
 2.86e-02
 
 1.00e+00
  
 
  
 7
 
 143105826
 
 143105970
 
  PDF   STATS   ENSEMBL   UCSC 
 
  145
 
  4
 
 37192
 
 gene
 
 0.04484
 
 0.48612
 
 Not queried
 
  4.5817083
 
 2.86e-02
 
 1.00e+00
  
 
  
 2
 
 241395181
 
 241395351
 
  PDF   STATS   ENSEMBL   UCSC 
 
  171
 
  4
 
 23084
 
 gene;tss;island
 
 0.08182
 
 0.71385
 
 Not queried
 
  4.5775550
 
 2.86e-02
 
 1.00e+00
  
 
  
 1
 
 156182812
 
 156183160
 
  PDF   STATS   ENSEMBL   UCSC 
 
  349
 
  4
 
 815
 
 gene
 
 0.44794
 
 0.03324
 
 Not queried
 
  4.5773541
 
 2.86e-02
 
 1.00e+00
  
 
  
 19
 
   9649310
 
   9649542
 
  PDF   STATS   ENSEMBL   UCSC 
 
  233
 
  7
 
 20390
 
 tss
 
 0.40138
 
 0.02568
 
 Not queried
 
  4.5750779
 
 5.83e-04
 
 1.00e+00
  
 
  
 6
 
  99292170
 
  99292438
 
  PDF   STATS   ENSEMBL   UCSC 
 
  269
 
  4
 
 36285
 
 island
 
 0.37899
 
 0.04969
 
 Not queried
 
  4.5738061
 
 2.86e-02
 
 1.00e+00
  
 
  
 5
 
   1633830
 
   1634218
 
  PDF   STATS   ENSEMBL   UCSC 
 
  389
 
  5
 
 32576
 
 island
 
 0.51457
 
 0.06570
 
 Not queried
 
  4.5701866
 
 7.94e-03
 
 1.00e+00
  
 
  
 1
 
  74663927
 
  74664160
 
  PDF   STATS   ENSEMBL   UCSC 
 
  234
 
  4
 
 583
 
 gene
 
 0.03001
 
 0.41188
 
 Not queried
 
  4.5642792
 
 2.86e-02
 
 1.00e+00
  
 
  
 3
 
  44754201
 
  44754587
 
  PDF   STATS   ENSEMBL   UCSC 
 
  387
 
  4
 
 27700
 
 gene
 
 0.51965
 
 0.04417
 
 Not queried
 
  4.5540096
 
 2.86e-02
 
 1.00e+00
  
 
  
 20
 
  33999839
 
  33999889
 
  PDF   STATS   ENSEMBL   UCSC 
 
   51
 
  6
 
 25666
 
 tss
 
 0.32733
 
 0.02852
 
 Not queried
 
  4.5525397
 
 2.16e-03
 
 1.00e+00
  
 
  
 6
 
  27648317
 
  27648605
 
  PDF   STATS   ENSEMBL   UCSC 
 
  289
 
  5
 
 35498
 
 island
 
 0.03380
 
 0.48728
 
 Not queried
 
  4.5473395
 
 7.94e-03
 
 1.00e+00
  
 
  
 6
 
  32407689
 
  32407838
 
  PDF   STATS   ENSEMBL   UCSC 
 
  150
 
  4
 
 34050
 
 gene
 
 0.55115
 
 0.06893
 
 Not queried
 
  4.5469803
 
 2.00e-01
 
 1.00e+00
  
 
  
 X
 
  53254643
 
  53254818
 
  PDF   STATS   ENSEMBL   UCSC 
 
  176
 
  7
 
 41328
 
 tss;island
 
 0.03297
 
 0.26112
 
 Not queried
 
  4.5458635
 
 5.83e-04
 
 1.00e+00
  
 
  
 1
 
 154474344
 
 154474530
 
  PDF   STATS   ENSEMBL   UCSC 
 
  187
 
  4
 
 776
 
 gene;tss;island
 
 0.03175
 
 0.38735
 
 Not queried
 
  4.5429542
 
 2.86e-02
 
 1.00e+00
  
 
  
 1
 
 172113756
 
 172114068
 
  PDF   STATS   ENSEMBL   UCSC 
 
  313
 
  4
 
 883
 
 gene
 
 0.31775
 
 0.91485
 
 Not queried
 
  4.5417211
 
 2.86e-02
 
 1.00e+00
  
 
  
 12
 
 114838567
 
 114839007
 
  PDF   STATS   ENSEMBL   UCSC 
 
  441
 
  5
 
 9298
 
 gene;island
 
 0.91183
 
 0.30763
 
 Not queried
 
  4.5407302
 
 7.94e-03
 
 1.00e+00
  
 
  
 20
 
  44657637
 
  44657948
 
  PDF   STATS   ENSEMBL   UCSC 
 
  312
 
  7
 
 25400
 
 gene;island
 
 0.22158
 
 0.82960
 
 Not queried
 
  4.5386300
 
 5.83e-04
 
 1.00e+00
  
 
  
 Y
 
  22737896
 
  22738029
 
  PDF   STATS   ENSEMBL   UCSC 
 
  134
 
  4
 
 42162
 
 gene;island
 
 0.05432
 
 0.43069
 
 Not queried
 
  4.5381983
 
 2.86e-02
 
 1.00e+00
  
 
  
 6
 
  36355488
 
  36355825
 
  PDF   STATS   ENSEMBL   UCSC 
 
  338
 
  6
 
 34872
 
 tss
 
 0.08580
 
 0.75344
 
 Not queried
 
  4.5369935
 
 2.16e-03
 
 1.00e+00
  
 
  
 5
 
 140220686
 
 140220822
 
  PDF   STATS   ENSEMBL   UCSC 
 
  137
 
  4
 
 32295
 
 tss
 
 0.34159
 
 0.87197
 
 Not queried
 
  4.5368043
 
 2.86e-02
 
 1.00e+00
  
 
  
 12
 
   6665108
 
   6665447
 
  PDF   STATS   ENSEMBL   UCSC 
 
  340
 
  7
 
 10173
 
 island
 
 0.66848
 
 0.07365
 
 Not queried
 
  4.5342416
 
 5.83e-04
 
 1.00e+00
  
 
  
 7
 
  28319343
 
  28319676
 
  PDF   STATS   ENSEMBL   UCSC 
 
  334
 
  6
 
 37439
 
 tss
 
 0.38014
 
 0.93616
 
 Not queried
 
  4.5312011
 
 1.32e-01
 
 1.00e+00
  
 
  
 14
 
  77737495
 
  77737800
 
  PDF   STATS   ENSEMBL   UCSC 
 
  306
 
  6
 
 13003
 
 island
 
 0.23837
 
 0.88984
 
 Not queried
 
  4.5241732
 
 2.16e-03
 
 1.00e+00
  
 
  
 5
 
   2754753
 
   2755158
 
  PDF   STATS   ENSEMBL   UCSC 
 
  406
 
  4
 
 31495
 
 gene;island
 
 0.05119
 
 0.01891
 
 Not queried
 
  4.5241486
 
 2.86e-02
 
 1.00e+00
  
 
  
 12
 
   6665288
 
   6665447
 
  PDF   STATS   ENSEMBL   UCSC 
 
  160
 
  6
 
 9491
 
 tss
 
 0.66558
 
 0.07841
 
 Not queried
 
  4.5234280
 
 2.16e-03
 
 1.00e+00
  
 
  
 2
 
 162272107
 
 162272604
 
  PDF   STATS   ENSEMBL   UCSC 
 
  498
 
  6
 
 23649
 
 tss
 
 0.07254
 
 0.64519
 
 Not queried
 
  4.5218114
 
 2.16e-03
 
 1.00e+00
  
 
  
 10
 
 118429452
 
 118429952
 
  PDF   STATS   ENSEMBL   UCSC 
 
  501
 
  8
 
 6147
 
 island
 
 0.05070
 
 0.60738
 
 Not queried
 
  4.5173859
 
 6.99e-03
 
 1.00e+00
  
 
  
 5
 
  10307544
 
  10307831
 
  PDF   STATS   ENSEMBL   UCSC 
 
  288
 
  4
 
 31504
 
 gene;island
 
 0.02424
 
 0.33822
 
 Not queried
 
  4.5106919
 
 2.86e-02
 
 1.00e+00
  
 
  
 1
 
 178994243
 
 178994355
 
  PDF   STATS   ENSEMBL   UCSC 
 
  113
 
  4
 
 2110
 
 tss;island
 
 0.54357
 
 0.04792
 
 Not queried
 
  4.5032717
 
 2.86e-02
 
 1.00e+00
  
 
  
 19
 
   7553986
 
   7554201
 
  PDF   STATS   ENSEMBL   UCSC 
 
  216
 
  6
 
 20362
 
 tss
 
 0.03361
 
 0.46488
 
 Not queried
 
  4.5023771
 
 2.16e-03
 
 1.00e+00
  
 
  
 18
 
  48085671
 
  48085844
 
  PDF   STATS   ENSEMBL   UCSC 
 
  174
 
  4
 
 19242
 
 tss;island
 
 0.53860
 
 0.04163
 
 Not queried
 
  4.4992609
 
 2.86e-02
 
 1.00e+00
  
 
  
 16
 
  90114063
 
  90114307
 
  PDF   STATS   ENSEMBL   UCSC 
 
  245
 
  6
 
 15407
 
 tss
 
 0.03378
 
 0.47087
 
 Not queried
 
  4.4964143
 
 4.33e-03
 
 1.00e+00
  
 
  
 13
 
  99630462
 
  99630583
 
  PDF   STATS   ENSEMBL   UCSC 
 
  122
 
  4
 
 11175
 
 gene;tss
 
 0.03092
 
 0.48906
 
 Not queried
 
  4.4947654
 
 2.86e-02
 
 1.00e+00
  
 
  
 11
 
  70917159
 
  70917533
 
  PDF   STATS   ENSEMBL   UCSC 
 
  375
 
  4
 
 6782
 
 gene
 
 0.31167
 
 0.94607
 
 Not queried
 
  4.4940281
 
 5.71e-02
 
 1.00e+00
  
 
  
 15
 
  93633145
 
  93633408
 
  PDF   STATS   ENSEMBL   UCSC 
 
  264
 
  5
 
 13888
 
 tss;island
 
 0.74837
 
 0.04680
 
 Not queried
 
  4.4906329
 
 7.94e-03
 
 1.00e+00
  
 
  
 18
 
  32398024
 
  32398287
 
  PDF   STATS   ENSEMBL   UCSC 
 
  264
 
  4
 
 19053
 
 gene;tss
 
 0.27133
 
 0.92034
 
 Not queried
 
  4.4892966
 
 5.71e-02
 
 1.00e+00
  
 
  
 17
 
  46805785
 
  46806056
 
  PDF   STATS   ENSEMBL   UCSC 
 
  272
 
  4
 
 16631
 
 gene
 
 0.03378
 
 0.32355
 
 Not queried
 
  4.4852676
 
 2.86e-02
 
 1.00e+00
  
 
  
 10
 
  63809073
 
  63809170
 
  PDF   STATS   ENSEMBL   UCSC 
 
   98
 
  6
 
 4586
 
 gene
 
 0.02540
 
 0.37797
 
 Not queried
 
  4.4843698
 
 2.16e-03
 
 1.00e+00
  
 
  
 6
 
   4282075
 
   4282346
 
  PDF   STATS   ENSEMBL   UCSC 
 
  272
 
  4
 
 35330
 
 island
 
 0.19794
 
 0.79521
 
 Not queried
 
  4.4799893
 
 2.86e-02
 
 1.00e+00
  
 
  
 X
 
  47518016
 
  47518238
 
  PDF   STATS   ENSEMBL   UCSC 
 
  223
 
  6
 
 41284
 
 tss
 
 0.02701
 
 0.45464
 
 Not queried
 
  4.4763129
 
 2.16e-03
 
 1.00e+00
  
 
  
 17
 
   6899085
 
   6899888
 
  PDF   STATS   ENSEMBL   UCSC 
 
  804
 
 14
 
 17880
 
 island
 
 0.05150
 
 0.56600
 
 Not queried
 
  4.4745782
 
 3.52e-03
 
 1.00e+00
  
 
  
 2
 
 136743411
 
 136743698
 
  PDF   STATS   ENSEMBL   UCSC 
 
  288
 
  5
 
 23610
 
 tss;island
 
 0.02016
 
 0.47865
 
 Not queried
 
  4.4737749
 
 7.94e-03
 
 1.00e+00
  
 
  
 6
 
  33401480
 
  33401542
 
  PDF   STATS   ENSEMBL   UCSC 
 
   63
 
  5
 
 34227
 
 gene
 
 0.80676
 
 0.16131
 
 Not queried
 
  4.4724639
 
 7.94e-03
 
 1.00e+00
  
 
  
 10
 
  14050305
 
  14050521
 
  PDF   STATS   ENSEMBL   UCSC 
 
  217
 
  4
 
 4494
 
 gene
 
 0.25064
 
 0.86800
 
 Not queried
 
  4.4720320
 
 2.86e-02
 
 1.00e+00
  
 
  
 3
 
  49395138
 
  49395303
 
  PDF   STATS   ENSEMBL   UCSC 
 
  166
 
  4
 
 27738
 
 gene;island
 
 0.89328
 
 0.30875
 
 Not queried
 
  4.4702882
 
 2.86e-02
 
 1.00e+00
  
 
  
 X
 
 100446220
 
 100446736
 
  PDF   STATS   ENSEMBL   UCSC 
 
  517
 
  5
 
 41908
 
 island
 
 0.05323
 
 0.55734
 
 Not queried
 
  4.4668120
 
 7.94e-03
 
 1.00e+00
  
 
  
 10
 
 131843517
 
 131843798
 
  PDF   STATS   ENSEMBL   UCSC 
 
  282
 
  4
 
 6251
 
 island
 
 0.23413
 
 0.80118
 
 Not queried
 
  4.4645206
 
 2.86e-02
 
 1.00e+00
  
 
  
 14
 
 103294363
 
 103294740
 
  PDF   STATS   ENSEMBL   UCSC 
 
  378
 
  5
 
 12205
 
 gene
 
 0.11337
 
 0.85966
 
 Not queried
 
  4.4601985
 
 7.94e-03
 
 1.00e+00
  
 
  
 15
 
  75918608
 
  75918757
 
  PDF   STATS   ENSEMBL   UCSC 
 
  150
 
  4
 
 13787
 
 tss;island
 
 0.42084
 
 0.03430
 
 Not queried
 
  4.4585967
 
 2.86e-02
 
 1.00e+00
  
 
  
 19
 
  49655176
 
  49655517
 
  PDF   STATS   ENSEMBL   UCSC 
 
  342
 
  4
 
 20108
 
 gene;island
 
 0.02909
 
 0.38853
 
 Not queried
 
  4.4581266
 
 2.86e-02
 
 1.00e+00
  
 
  
 2
 
 228736089
 
 228736324
 
  PDF   STATS   ENSEMBL   UCSC 
 
  236
 
  6
 
 23894
 
 tss
 
 0.04927
 
 0.46116
 
 Not queried
 
  4.4576305
 
 2.16e-03
 
 1.00e+00
  
 
  
 2
 
 229046325
 
 229046785
 
  PDF   STATS   ENSEMBL   UCSC 
 
  461
 
  5
 
 25132
 
 island
 
 0.67879
 
 0.07967
 
 Not queried
 
  4.4568486
 
 9.52e-02
 
 1.00e+00
  
 
  
 11
 
  57364841
 
  57365088
 
  PDF   STATS   ENSEMBL   UCSC 
 
  248
 
  6
 
 7283
 
 tss
 
 0.76968
 
 0.08072
 
 Not queried
 
  4.4563300
 
 2.16e-03
 
 1.00e+00
  
 
  
 16
 
  89573312
 
  89573631
 
  PDF   STATS   ENSEMBL   UCSC 
 
  320
 
  5
 
 15394
 
 tss;island
 
 0.09915
 
 0.76786
 
 Not queried
 
  4.4536645
 
 7.94e-03
 
 1.00e+00
  
 
  
 6
 
  31237013
 
  31237199
 
  PDF   STATS   ENSEMBL   UCSC 
 
  187
 
  4
 
 33801
 
 gene;island
 
 0.10794
 
 0.68423
 
 Not queried
 
  4.4528980
 
 5.71e-02
 
 1.00e+00
  
 
  
 5
 
 140772182
 
 140772859
 
  PDF   STATS   ENSEMBL   UCSC 
 
  678
 
  7
 
 31771
 
 gene;island
 
 0.43160
 
 0.92864
 
 Not queried
 
  4.4528328
 
 5.83e-04
 
 1.00e+00
  
 
  
 2
 
 115419537
 
 115419846
 
  PDF   STATS   ENSEMBL   UCSC 
 
  310
 
  4
 
 22801
 
 gene;island
 
 0.05122
 
 0.53643
 
 Not queried
 
  4.4510857
 
 2.86e-02
 
 1.00e+00
  
 
  
 10
 
  88391658
 
  88392088
 
  PDF   STATS   ENSEMBL   UCSC 
 
  431
 
  4
 
 5888
 
 island
 
 0.06956
 
 0.58392
 
 Not queried
 
  4.4506101
 
 2.00e-01
 
 1.00e+00
  
 
  
 7
 
   5085252
 
   5085485
 
  PDF   STATS   ENSEMBL   UCSC 
 
  234
 
  7
 
 37354
 
 tss
 
 0.37357
 
 0.02759
 
 Not queried
 
  4.4486222
 
 5.83e-04
 
 1.00e+00
  
 
  
 19
 
  39894559
 
  39894767
 
  PDF   STATS   ENSEMBL   UCSC 
 
  209
 
  4
 
 21959
 
 island
 
 0.51853
 
 0.05908
 
 Not queried
 
  4.4484232
 
 2.86e-02
 
 1.00e+00
  
 
  
 10
 
  22633916
 
  22634226
 
  PDF   STATS   ENSEMBL   UCSC 
 
  311
 
  8
 
 4968
 
 tss;island
 
 0.04690
 
 0.48472
 
 Not queried
 
  4.4465439
 
 3.11e-04
 
 1.00e+00
  
 
  
 7
 
 140103545
 
 140103783
 
  PDF   STATS   ENSEMBL   UCSC 
 
  239
 
  7
 
 37873
 
 tss
 
 0.31510
 
 0.85610
 
 Not queried
 
  4.4455408
 
 5.83e-04
 
 1.00e+00
  
 
  
 16
 
  31159558
 
  31159920
 
  PDF   STATS   ENSEMBL   UCSC 
 
  363
 
  5
 
 14703
 
 gene;island
 
 0.03265
 
 0.41291
 
 Not queried
 
  4.4430301
 
 7.94e-03
 
 1.00e+00
  
 
  
 7
 
 158059704
 
 158060003
 
  PDF   STATS   ENSEMBL   UCSC 
 
  300
 
  5
 
 37299
 
 gene;island
 
 0.22085
 
 0.86715
 
 Not queried
 
  4.4415191
 
 9.52e-02
 
 1.00e+00
  
 
  
 7
 
  88425077
 
  88425148
 
  PDF   STATS   ENSEMBL   UCSC 
 
   72
 
  5
 
 37628
 
 tss
 
 0.19341
 
 0.68169
 
 Not queried
 
  4.4394727
 
 7.94e-03
 
 1.00e+00
  
 
  
 3
 
  49314539
 
  49314920
 
  PDF   STATS   ENSEMBL   UCSC 
 
  382
 
  9
 
 28283
 
 tss
 
 0.53903
 
 0.95567
 
 Not queried
 
  4.4368256
 
 2.76e-03
 
 1.00e+00
  
 
  
 3
 
  49314370
 
  49314920
 
  PDF   STATS   ENSEMBL   UCSC 
 
  551
 
 11
 
 29070
 
 island
 
 0.48764
 
 0.94217
 
 Not queried
 
  4.4368256
 
 3.94e-04
 
 1.00e+00
  
 
  
 16
 
  67876973
 
  67877413
 
  PDF   STATS   ENSEMBL   UCSC 
 
  441
 
  4
 
 14773
 
 gene;island
 
 0.45727
 
 0.04507
 
 Not queried
 
  4.4345268
 
 1.14e-01
 
 1.00e+00
  
 
  
 17
 
  36105064
 
  36105517
 
  PDF   STATS   ENSEMBL   UCSC 
 
  454
 
  7
 
 18217
 
 island
 
 0.65114
 
 0.08575
 
 Not queried
 
  4.4334039
 
 5.83e-04
 
 1.00e+00
  
 
  
 2
 
 165812079
 
 165812297
 
  PDF   STATS   ENSEMBL   UCSC 
 
  219
 
  4
 
 23656
 
 tss
 
 0.04314
 
 0.57353
 
 Not queried
 
  4.4331961
 
 5.71e-02
 
 1.00e+00
  
 
  
 2
 
    583115
 
    583384
 
  PDF   STATS   ENSEMBL   UCSC 
 
  270
 
  5
 
 23969
 
 island
 
 0.38865
 
 0.94310
 
 Not queried
 
  4.4323175
 
 7.94e-03
 
 1.00e+00
  
 
  
 22
 
  38479181
 
  38479332
 
  PDF   STATS   ENSEMBL   UCSC 
 
  152
 
  6
 
 27088
 
 tss
 
 0.31065
 
 0.84218
 
 Not queried
 
  4.4302960
 
 2.16e-03
 
 1.00e+00
  
 
  
 20
 
  62406677
 
  62406964
 
  PDF   STATS   ENSEMBL   UCSC 
 
  288
 
  4
 
 25473
 
 gene;island
 
 0.10595
 
 0.72733
 
 Not queried
 
  4.4274152
 
 5.71e-02
 
 1.00e+00
  
 
  
 17
 
  74075092
 
  74075195
 
  PDF   STATS   ENSEMBL   UCSC 
 
  104
 
  4
 
 17637
 
 tss
 
 0.19680
 
 0.73880
 
 Not queried
 
  4.4250393
 
 2.86e-02
 
 1.00e+00
  
 
  
 X
 
  53350498
 
  53350693
 
  PDF   STATS   ENSEMBL   UCSC 
 
  196
 
  6
 
 41807
 
 island
 
 0.03304
 
 0.44140
 
 Not queried
 
  4.4245816
 
 2.16e-03
 
 1.00e+00
  
 
  
 10
 
  97850047
 
  97850343
 
  PDF   STATS   ENSEMBL   UCSC 
 
  297
 
  5
 
 5952
 
 island
 
 0.40319
 
 0.93526
 
 Not queried
 
  4.4183288
 
 1.51e-01
 
 1.00e+00
  
 
  
 2
 
  39005329
 
  39005834
 
  PDF   STATS   ENSEMBL   UCSC 
 
  506
 
  5
 
 22614
 
 gene
 
 0.02556
 
 0.35928
 
 Not queried
 
  4.4177462
 
 7.94e-03
 
 1.00e+00
  
 
  
 12
 
   7073179
 
   7073247
 
  PDF   STATS   ENSEMBL   UCSC 
 
   69
 
  4
 
 9515
 
 tss
 
 0.31314
 
 0.86460
 
 Not queried
 
  4.4166827
 
 2.86e-02
 
 1.00e+00
  
 
  
 2
 
   6121909
 
   6122107
 
  PDF   STATS   ENSEMBL   UCSC 
 
  199
 
  4
 
 23127
 
 tss
 
 0.11130
 
 0.74197
 
 Not queried
 
  4.4141712
 
 2.86e-02
 
 1.00e+00
  
 
  
 5
 
  76925380
 
  76925623
 
  PDF   STATS   ENSEMBL   UCSC 
 
  244
 
  4
 
 31598
 
 gene;island
 
 0.44053
 
 0.05510
 
 Not queried
 
  4.4112806
 
 2.00e-01
 
 1.00e+00
  
 
  
 5
 
 140624869
 
 140625389
 
  PDF   STATS   ENSEMBL   UCSC 
 
  521
 
 10
 
 33166
 
 island
 
 0.01933
 
 0.35252
 
 Not queried
 
  4.4101813
 
 1.05e-03
 
 1.00e+00
  
 
  
 6
 
  26103952
 
  26104165
 
  PDF   STATS   ENSEMBL   UCSC 
 
  214
 
  5
 
 34621
 
 tss
 
 0.03477
 
 0.35770
 
 Not queried
 
  4.4094190
 
 7.94e-03
 
 1.00e+00
  
 
  
 20
 
  44258197
 
  44258437
 
  PDF   STATS   ENSEMBL   UCSC 
 
  241
 
  5
 
 25393
 
 gene
 
 0.04004
 
 0.45483
 
 Not queried
 
  4.4050873
 
 7.94e-03
 
 1.00e+00
  
 
  
 8
 
  97657553
 
  97657833
 
  PDF   STATS   ENSEMBL   UCSC 
 
  281
 
  4
 
 39307
 
 gene
 
 0.06588
 
 0.60095
 
 Not queried
 
  4.4040113
 
 2.86e-02
 
 1.00e+00
  
 
  
 1
 
 231296640
 
 231297103
 
  PDF   STATS   ENSEMBL   UCSC 
 
  464
 
  5
 
 4310
 
 island
 
 0.85234
 
 0.17515
 
 Not queried
 
  4.4037608
 
 9.52e-02
 
 1.00e+00
  
 
  
 8
 
 143695893
 
 143696144
 
  PDF   STATS   ENSEMBL   UCSC 
 
  252
 
  5
 
 39867
 
 tss
 
 0.08110
 
 0.64401
 
 Not queried
 
  4.4024498
 
 7.94e-03
 
 1.00e+00
  
 
  
 14
 
  52734156
 
  52734397
 
  PDF   STATS   ENSEMBL   UCSC 
 
  242
 
  6
 
 12368
 
 tss
 
 0.33859
 
 0.90351
 
 Not queried
 
  4.4016684
 
 2.16e-03
 
 1.00e+00
  
 
  
 7
 
  27291346
 
  27291695
 
  PDF   STATS   ENSEMBL   UCSC 
 
  350
 
  4
 
 38303
 
 island
 
 0.84929
 
 0.20045
 
 Not queried
 
  4.4013033
 
 2.86e-02
 
 1.00e+00
  
 
  
 22
 
  32599511
 
  32599648
 
  PDF   STATS   ENSEMBL   UCSC 
 
  138
 
  5
 
 26805
 
 gene;tss
 
 0.23905
 
 0.86877
 
 Not queried
 
  4.3973790
 
 7.94e-03
 
 1.00e+00
  
 
  
 1
 
  46958475
 
  46958856
 
  PDF   STATS   ENSEMBL   UCSC 
 
  382
 
  4
 
 3214
 
 island
 
 0.82148
 
 0.17650
 
 Not queried
 
  4.3930592
 
 2.86e-02
 
 1.00e+00
  
 
  
 14
 
  37125292
 
  37125997
 
  PDF   STATS   ENSEMBL   UCSC 
 
  706
 
  8
 
 12332
 
 tss
 
 0.04101
 
 0.61578
 
 Not queried
 
  4.3926403
 
 2.95e-03
 
 1.00e+00
  
 
  
 14
 
  37125004
 
  37125997
 
  PDF   STATS   ENSEMBL   UCSC 
 
  994
 
 10
 
 12763
 
 island
 
 0.04101
 
 0.61578
 
 Not queried
 
  4.3926403
 
 2.88e-03
 
 1.00e+00
  
 
  
 1
 
  16970920
 
  16971585
 
  PDF   STATS   ENSEMBL   UCSC 
 
  666
 
  8
 
 1282
 
 tss;island
 
 0.02026
 
 0.29688
 
 Not queried
 
  4.3915269
 
 3.11e-04
 
 1.00e+00
  
 
  
 17
 
   7284255
 
   7284351
 
  PDF   STATS   ENSEMBL   UCSC 
 
   97
 
  4
 
 17044
 
 tss;island
 
 0.01943
 
 0.33526
 
 Not queried
 
  4.3909779
 
 2.86e-02
 
 1.00e+00
  
 
  
 13
 
  50070436
 
  50070520
 
  PDF   STATS   ENSEMBL   UCSC 
 
   85
 
  5
 
 11413
 
 tss
 
 0.51665
 
 0.03036
 
 Not queried
 
  4.3820061
 
 7.94e-03
 
 1.00e+00
  
 
  
 5
 
 140578896
 
 140579303
 
  PDF   STATS   ENSEMBL   UCSC 
 
  408
 
  7
 
 32307
 
 tss
 
 0.02412
 
 0.24048
 
 Not queried
 
  4.3769414
 
 1.11e-02
 
 1.00e+00
  
 
  
 5
 
 140578896
 
 140579644
 
  PDF   STATS   ENSEMBL   UCSC 
 
  749
 
  9
 
 33161
 
 island
 
 0.02451
 
 0.41143
 
 Not queried
 
  4.3769414
 
 1.85e-03
 
 1.00e+00
  
 
  
 21
 
  26934197
 
  26934455
 
  PDF   STATS   ENSEMBL   UCSC 
 
  259
 
  4
 
 26455
 
 tss
 
 0.03410
 
 0.47925
 
 Not queried
 
  4.3761853
 
 2.86e-02
 
 1.00e+00
  
 
  
 6
 
  13873924
 
  13874251
 
  PDF   STATS   ENSEMBL   UCSC 
 
  328
 
  4
 
 35395
 
 island
 
 0.04969
 
 0.49992
 
 Not queried
 
  4.3759835
 
 2.86e-02
 
 1.00e+00
  
 
  
 1
 
  50886782
 
  50886969
 
  PDF   STATS   ENSEMBL   UCSC 
 
  188
 
  4
 
 524
 
 gene;island
 
 0.60432
 
 0.08390
 
 Not queried
 
  4.3753399
 
 2.86e-02
 
 1.00e+00
  
 
  
 22
 
  50523686
 
  50524124
 
  PDF   STATS   ENSEMBL   UCSC 
 
  439
 
  8
 
 26881
 
 gene
 
 0.12856
 
 0.59126
 
 Not queried
 
  4.3659347
 
 3.11e-04
 
 1.00e+00
  
 
  
 1
 
 144994542
 
 144995101
 
  PDF   STATS   ENSEMBL   UCSC 
 
  560
 
  6
 
 718
 
 gene
 
 0.09677
 
 0.69588
 
 Not queried
 
  4.3635152
 
 2.16e-03
 
 1.00e+00
  
 
  
 16
 
  80966033
 
  80966491
 
  PDF   STATS   ENSEMBL   UCSC 
 
  459
 
  6
 
 16084
 
 island
 
 0.38661
 
 0.02757
 
 Not queried
 
  4.3609275
 
 2.16e-03
 
 1.00e+00
  
 
  
 1
 
 217263507
 
 217263847
 
  PDF   STATS   ENSEMBL   UCSC 
 
  341
 
  4
 
 1008
 
 gene;tss;island
 
 0.61107
 
 0.13041
 
 Not queried
 
  4.3605522
 
 1.14e-01
 
 1.00e+00
  
 
  
 11
 
  76838498
 
  76838749
 
  PDF   STATS   ENSEMBL   UCSC 
 
  252
 
  5
 
 7561
 
 tss
 
 0.01002
 
 0.02887
 
 Not queried
 
  4.3604837
 
 1.59e-02
 
 1.00e+00
  
 
  
 5
 
 140529499
 
 140529819
 
  PDF   STATS   ENSEMBL   UCSC 
 
  321
 
  6
 
 32304
 
 tss;island
 
 0.03619
 
 0.37679
 
 Not queried
 
  4.3580081
 
 2.16e-03
 
 1.00e+00
  
 
  
 10
 
 104535792
 
 104536121
 
  PDF   STATS   ENSEMBL   UCSC 
 
  330
 
  9
 
 4723
 
 gene
 
 0.03283
 
 0.46505
 
 Not queried
 
  4.3568927
 
 1.23e-03
 
 1.00e+00
  
 
  
 8
 
  10586678
 
  10587055
 
  PDF   STATS   ENSEMBL   UCSC 
 
  378
 
  7
 
 39136
 
 gene;island
 
 0.80246
 
 0.14538
 
 Not queried
 
  4.3564718
 
 5.83e-04
 
 1.00e+00
  
 
  
 13
 
 114780772
 
 114780992
 
  PDF   STATS   ENSEMBL   UCSC 
 
  221
 
  4
 
 11276
 
 gene;island
 
 0.19267
 
 0.82753
 
 Not queried
 
  4.3522840
 
 2.86e-02
 
 1.00e+00
  
 
  
 1
 
  20878760
 
  20878928
 
  PDF   STATS   ENSEMBL   UCSC 
 
  169
 
  4
 
 1311
 
 tss
 
 0.52427
 
 0.05778
 
 Not queried
 
  4.3516155
 
 5.71e-02
 
 1.00e+00
  
 
  
 1
 
  53387430
 
  53387719
 
  PDF   STATS   ENSEMBL   UCSC 
 
  290
 
  4
 
 3269
 
 island
 
 0.03856
 
 0.49980
 
 Not queried
 
  4.3491015
 
 2.00e-01
 
 1.00e+00
  
 
  
 5
 
 143978290
 
 143978420
 
  PDF   STATS   ENSEMBL   UCSC 
 
  131
 
  4
 
 33213
 
 island
 
 0.04528
 
 0.49472
 
 Not queried
 
  4.3480385
 
 2.86e-02
 
 1.00e+00
  
 
  
 2
 
  66664535
 
  66664960
 
  PDF   STATS   ENSEMBL   UCSC 
 
  426
 
  5
 
 22673
 
 gene;island
 
 0.69264
 
 0.10669
 
 Not queried
 
  4.3467792
 
 3.17e-02
 
 1.00e+00
  
 
  
 19
 
  53811704
 
  53812098
 
  PDF   STATS   ENSEMBL   UCSC 
 
  395
 
  4
 
 22338
 
 island
 
 0.11449
 
 0.63778
 
 Not queried
 
  4.3441861
 
 2.86e-02
 
 1.00e+00
  
 
  
 10
 
  37414506
 
  37414802
 
  PDF   STATS   ENSEMBL   UCSC 
 
  297
 
  7
 
 5650
 
 island
 
 0.14907
 
 0.88112
 
 Not queried
 
  4.3433732
 
 5.83e-04
 
 1.00e+00
  
 
  
 5
 
 134181484
 
 134181707
 
  PDF   STATS   ENSEMBL   UCSC 
 
  224
 
  7
 
 31695
 
 gene;island
 
 0.01110
 
 0.29763
 
 Not queried
 
  4.3431399
 
 5.83e-04
 
 1.00e+00
  
 
  
 10
 
 118890977
 
 118891355
 
  PDF   STATS   ENSEMBL   UCSC 
 
  379
 
  4
 
 4762
 
 gene;island
 
 0.66866
 
 0.06663
 
 Not queried
 
  4.3413836
 
 2.86e-02
 
 1.00e+00
  
 
  
 11
 
  76839129
 
  76839217
 
  PDF   STATS   ENSEMBL   UCSC 
 
   89
 
  4
 
 7562
 
 tss
 
 0.03751
 
 0.38745
 
 Not queried
 
  4.3372544
 
 2.86e-02
 
 1.00e+00
  
 
  
 11
 
  58912052
 
  58912240
 
  PDF   STATS   ENSEMBL   UCSC 
 
  189
 
  5
 
 7294
 
 tss
 
 0.73243
 
 0.16012
 
 Not queried
 
  4.3365840
 
 7.94e-03
 
 1.00e+00
  
 
  
 X
 
 153707447
 
 153707810
 
  PDF   STATS   ENSEMBL   UCSC 
 
  364
 
  6
 
 42147
 
 island
 
 0.07427
 
 0.60370
 
 Not queried
 
  4.3356138
 
 2.16e-03
 
 1.00e+00
  
 
  
 2
 
 134326055
 
 134326366
 
  PDF   STATS   ENSEMBL   UCSC 
 
  312
 
  5
 
 23603
 
 tss
 
 0.21414
 
 0.66624
 
 Not queried
 
  4.3346075
 
 7.94e-03
 
 1.00e+00
  
 
  
 11
 
 113258210
 
 113258455
 
  PDF   STATS   ENSEMBL   UCSC 
 
  246
 
  6
 
 7681
 
 tss;island
 
 0.23875
 
 0.84583
 
 Not queried
 
  4.3321138
 
 2.16e-03
 
 1.00e+00
  
 
  
 15
 
 101587506
 
 101587971
 
  PDF   STATS   ENSEMBL   UCSC 
 
  466
 
  5
 
 13478
 
 gene
 
 0.91970
 
 0.13343
 
 Not queried
 
  4.3272548
 
 3.17e-02
 
 1.00e+00
  
 
  
 X
 
  63615185
 
  63615495
 
  PDF   STATS   ENSEMBL   UCSC 
 
  311
 
  7
 
 41828
 
 island
 
 0.05103
 
 0.51885
 
 Not queried
 
  4.3258056
 
 5.83e-04
 
 1.00e+00
  
 
  
 10
 
  57390536
 
  57391055
 
  PDF   STATS   ENSEMBL   UCSC 
 
  520
 
  4
 
 5736
 
 island
 
 0.11382
 
 0.59593
 
 Not queried
 
  4.3251473
 
 2.86e-02
 
 1.00e+00
  
 
  
 14
 
  64855023
 
  64855353
 
  PDF   STATS   ENSEMBL   UCSC 
 
  331
 
  4
 
 12083
 
 gene;island
 
 0.56960
 
 0.04603
 
 Not queried
 
  4.3223236
 
 2.86e-02
 
 1.00e+00
  
 
  
 19
 
  51231557
 
  51232007
 
  PDF   STATS   ENSEMBL   UCSC 
 
  451
 
  5
 
 22287
 
 island
 
 0.07933
 
 0.63269
 
 Not queried
 
  4.3213434
 
 1.51e-01
 
 1.00e+00
  
 
  
 1
 
  63795636
 
  63795970
 
  PDF   STATS   ENSEMBL   UCSC 
 
  335
 
  5
 
 3337
 
 island
 
 0.38572
 
 0.04211
 
 Not queried
 
  4.3196060
 
 7.94e-03
 
 1.00e+00
  
 
  
 22
 
  38479099
 
  38479332
 
  PDF   STATS   ENSEMBL   UCSC 
 
  234
 
  7
 
 27438
 
 island
 
 0.35993
 
 0.85333
 
 Not queried
 
  4.3140526
 
 5.83e-04
 
 1.00e+00
  
 
  
 X
 
  54070741
 
  54070878
 
  PDF   STATS   ENSEMBL   UCSC 
 
  138
 
  4
 
 41333
 
 tss;island
 
 0.03744
 
 0.42020
 
 Not queried
 
  4.3140460
 
 2.86e-02
 
 1.00e+00
  
 
  
 3
 
  53190593
 
  53190763
 
  PDF   STATS   ENSEMBL   UCSC 
 
  171
 
  4
 
 29155
 
 island
 
 0.09276
 
 0.61233
 
 Not queried
 
  4.3114621
 
 5.71e-02
 
 1.00e+00
  
 
  
 2
 
  54342584
 
  54342961
 
  PDF   STATS   ENSEMBL   UCSC 
 
  378
 
  4
 
 22645
 
 gene;island
 
 0.01963
 
 0.28192
 
 Not queried
 
  4.3099411
 
 2.86e-02
 
 1.00e+00
  
 
  
 13
 
  28365587
 
  28365853
 
  PDF   STATS   ENSEMBL   UCSC 
 
  267
 
  4
 
 11335
 
 tss;island
 
 0.03894
 
 0.44491
 
 Not queried
 
  4.3062444
 
 5.71e-02
 
 1.00e+00
  
 
  
 X
 
  68835489
 
  68836202
 
  PDF   STATS   ENSEMBL   UCSC 
 
  714
 
 11
 
 41843
 
 island
 
 0.10783
 
 0.01045
 
 Not queried
 
  4.3039825
 
 3.94e-04
 
 1.00e+00
  
 
  
 3
 
 148847007
 
 148847295
 
  PDF   STATS   ENSEMBL   UCSC 
 
  289
 
  6
 
 28613
 
 tss;island
 
 0.03103
 
 0.35905
 
 Not queried
 
  4.3024688
 
 2.16e-03
 
 1.00e+00
  
 
  
 11
 
 120008944
 
 120009135
 
  PDF   STATS   ENSEMBL   UCSC 
 
  192
 
  5
 
 7736
 
 tss
 
 0.07884
 
 0.62897
 
 Not queried
 
  4.3005867
 
 9.52e-02
 
 1.00e+00
  
 
  
 5
 
 179588440
 
 179588838
 
  PDF   STATS   ENSEMBL   UCSC 
 
  399
 
  4
 
 31881
 
 gene;island
 
 0.24953
 
 0.79882
 
 Not queried
 
  4.2990309
 
 2.86e-02
 
 1.00e+00
  
 
  
 18
 
  20735537
 
  20735693
 
  PDF   STATS   ENSEMBL   UCSC 
 
  157
 
  4
 
 19038
 
 gene;tss
 
 0.13797
 
 0.79952
 
 Not queried
 
  4.2988274
 
 2.86e-02
 
 1.00e+00
  
 
  
 X
 
 146992908
 
 146993175
 
  PDF   STATS   ENSEMBL   UCSC 
 
  268
 
  5
 
 41133
 
 gene;tss;island
 
 0.04368
 
 0.63945
 
 Not queried
 
  4.2966679
 
 9.52e-02
 
 1.00e+00
  
 
  
 15
 
  89910632
 
  89910860
 
  PDF   STATS   ENSEMBL   UCSC 
 
  229
 
  4
 
 13858
 
 tss;island
 
 0.05553
 
 0.54814
 
 Not queried
 
  4.2960584
 
 5.71e-02
 
 1.00e+00
  
 
  
 X
 
  63615339
 
  63615495
 
  PDF   STATS   ENSEMBL   UCSC 
 
  157
 
  6
 
 41350
 
 tss
 
 0.05196
 
 0.52857
 
 Not queried
 
  4.2952260
 
 2.16e-03
 
 1.00e+00
  
 
  
 X
 
  53350524
 
  53350693
 
  PDF   STATS   ENSEMBL   UCSC 
 
  170
 
  5
 
 41329
 
 tss
 
 0.03147
 
 0.42557
 
 Not queried
 
  4.2948123
 
 7.94e-03
 
 1.00e+00
  
 
  
 11
 
   1660843
 
   1661201
 
  PDF   STATS   ENSEMBL   UCSC 
 
  359
 
  4
 
 6408
 
 gene
 
 0.08136
 
 0.69897
 
 Not queried
 
  4.2921358
 
 2.86e-02
 
 1.00e+00
  
 
  
 6
 
  49755009
 
  49755190
 
  PDF   STATS   ENSEMBL   UCSC 
 
  182
 
  4
 
 34964
 
 tss
 
 0.17584
 
 0.76777
 
 Not queried
 
  4.2903020
 
 2.86e-02
 
 1.00e+00
  
 
  
 10
 
 130063978
 
 130064296
 
  PDF   STATS   ENSEMBL   UCSC 
 
  319
 
  4
 
 6236
 
 island
 
 0.60455
 
 0.09218
 
 Not queried
 
  4.2889464
 
 2.86e-02
 
 1.00e+00
  
 
  
 6
 
 138200071
 
 138200387
 
  PDF   STATS   ENSEMBL   UCSC 
 
  317
 
  6
 
 34422
 
 gene
 
 0.10684
 
 0.78070
 
 Not queried
 
  4.2860039
 
 1.52e-02
 
 1.00e+00
  
 
  
 6
 
  32116207
 
  32116317
 
  PDF   STATS   ENSEMBL   UCSC 
 
  111
 
  4
 
 33994
 
 gene
 
 0.08125
 
 0.58213
 
 Not queried
 
  4.2849727
 
 2.86e-02
 
 1.00e+00
  
 
  
 11
 
  65222227
 
  65222786
 
  PDF   STATS   ENSEMBL   UCSC 
 
  560
 
  6
 
 8435
 
 island
 
 0.23480
 
 0.69788
 
 Not queried
 
  4.2775378
 
 6.49e-02
 
 1.00e+00
  
 
  
 5
 
 140624869
 
 140625130
 
  PDF   STATS   ENSEMBL   UCSC 
 
  262
 
  7
 
 32310
 
 tss
 
 0.02196
 
 0.21950
 
 Not queried
 
  4.2719677
 
 5.83e-04
 
 1.00e+00
  
 
  
 15
 
  51385812
 
  51385970
 
  PDF   STATS   ENSEMBL   UCSC 
 
  159
 
  4
 
 13319
 
 gene;island
 
 0.86648
 
 0.21100
 
 Not queried
 
  4.2714733
 
 5.71e-02
 
 1.00e+00
  
 
  
 2
 
  19551750
 
  19551944
 
  PDF   STATS   ENSEMBL   UCSC 
 
  195
 
  4
 
 22549
 
 gene;island
 
 0.81797
 
 0.13045
 
 Not queried
 
  4.2711625
 
 2.86e-02
 
 1.00e+00
  
 
  
 17
 
  55938748
 
  55939089
 
  PDF   STATS   ENSEMBL   UCSC 
 
  342
 
  4
 
 18553
 
 island
 
 0.06814
 
 0.59346
 
 Not queried
 
  4.2689704
 
 2.86e-02
 
 1.00e+00
  
 
  
 12
 
  46663270
 
  46663407
 
  PDF   STATS   ENSEMBL   UCSC 
 
  138
 
  5
 
 9632
 
 tss;island
 
 0.36027
 
 0.91368
 
 Not queried
 
  4.2686080
 
 7.94e-03
 
 1.00e+00
  
 
  
 17
 
  45949677
 
  45949878
 
  PDF   STATS   ENSEMBL   UCSC 
 
  202
 
  5
 
 18430
 
 island
 
 0.58980
 
 0.08294
 
 Not queried
 
  4.2671774
 
 7.94e-03
 
 1.00e+00
  
 
  
 8
 
  70946891
 
  70947218
 
  PDF   STATS   ENSEMBL   UCSC 
 
  328
 
  4
 
 40308
 
 island
 
 0.60007
 
 0.08043
 
 Not queried
 
  4.2657986
 
 2.86e-02
 
 1.00e+00
  
 
  
 2
 
 198650603
 
 198651076
 
  PDF   STATS   ENSEMBL   UCSC 
 
  474
 
  6
 
 23764
 
 tss;island
 
 0.05090
 
 0.49960
 
 Not queried
 
  4.2628608
 
 4.11e-02
 
 1.00e+00
  
 
  
 11
 
  61159602
 
  61159837
 
  PDF   STATS   ENSEMBL   UCSC 
 
  236
 
  7
 
 7313
 
 tss;island
 
 0.79836
 
 0.12041
 
 Not queried
 
  4.2620189
 
 1.17e-03
 
 1.00e+00
  
 
  
 2
 
    628881
 
    629121
 
  PDF   STATS   ENSEMBL   UCSC 
 
  241
 
  5
 
 23971
 
 island
 
 0.02553
 
 0.37299
 
 Not queried
 
  4.2615260
 
 7.94e-03
 
 1.00e+00
  
 
  
 2
 
 171568791
 
 171569381
 
  PDF   STATS   ENSEMBL   UCSC 
 
  591
 
  7
 
 24807
 
 island
 
 0.69116
 
 0.08981
 
 Not queried
 
  4.2600415
 
 1.75e-02
 
 1.00e+00
  
 
  
 2
 
   9614471
 
   9614578
 
  PDF   STATS   ENSEMBL   UCSC 
 
  108
 
  5
 
 23135
 
 tss
 
 0.44460
 
 0.93695
 
 Not queried
 
  4.2589022
 
 7.94e-03
 
 1.00e+00
  
 
  
 X
 
  46404528
 
  46404864
 
  PDF   STATS   ENSEMBL   UCSC 
 
  337
 
  6
 
 40970
 
 gene
 
 0.06298
 
 0.60108
 
 Not queried
 
  4.2580784
 
 2.16e-03
 
 1.00e+00
  
 
  
 19
 
  31210456
 
  31210714
 
  PDF   STATS   ENSEMBL   UCSC 
 
  259
 
  4
 
 21826
 
 island
 
 0.12683
 
 0.68635
 
 Not queried
 
  4.2571162
 
 2.86e-02
 
 1.00e+00
  
 
  
 6
 
    292329
 
    292596
 
  PDF   STATS   ENSEMBL   UCSC 
 
  268
 
  4
 
 33465
 
 gene;island
 
 0.05499
 
 0.48873
 
 Not queried
 
  4.2500805
 
 2.86e-02
 
 1.00e+00
  
 
  
 1
 
  55445834
 
  55446708
 
  PDF   STATS   ENSEMBL   UCSC 
 
  875
 
 10
 
 3297
 
 island
 
 0.10712
 
 0.74370
 
 Not queried
 
  4.2470668
 
 2.09e-03
 
 1.00e+00
  
 
  
 11
 
  65190135
 
  65190227
 
  PDF   STATS   ENSEMBL   UCSC 
 
   93
 
  7
 
 7408
 
 tss;island
 
 0.10632
 
 0.77132
 
 Not queried
 
  4.2411517
 
 5.83e-04
 
 1.00e+00
  
 
  
 5
 
 140220686
 
 140221106
 
  PDF   STATS   ENSEMBL   UCSC 
 
  421
 
  6
 
 31742
 
 gene;island
 
 0.27128
 
 0.86740
 
 Not queried
 
  4.2361048
 
 2.16e-03
 
 1.00e+00
  
 
  
 12
 
  97300410
 
  97300582
 
  PDF   STATS   ENSEMBL   UCSC 
 
  173
 
  4
 
 9907
 
 tss;island
 
 0.67846
 
 0.96519
 
 Not queried
 
  4.2313628
 
 2.86e-02
 
 1.00e+00
  
 
  
 2
 
 229046406
 
 229046785
 
  PDF   STATS   ENSEMBL   UCSC 
 
  380
 
  4
 
 23895
 
 tss
 
 0.66705
 
 0.08830
 
 Not queried
 
  4.2261884
 
 2.00e-01
 
 1.00e+00
  
 
  
 17
 
  46659751
 
  46660002
 
  PDF   STATS   ENSEMBL   UCSC 
 
  252
 
  4
 
 18449
 
 island
 
 0.50626
 
 0.11141
 
 Not queried
 
  4.2227371
 
 1.14e-01
 
 1.00e+00
  
 
  
 3
 
 140813769
 
 140814133
 
  PDF   STATS   ENSEMBL   UCSC 
 
  365
 
  4
 
 27929
 
 gene;island
 
 0.03059
 
 0.39665
 
 Not queried
 
  4.2225210
 
 1.14e-01
 
 1.00e+00
  
 
  
 5
 
  54281198
 
  54281362
 
  PDF   STATS   ENSEMBL   UCSC 
 
  165
 
  4
 
 31558
 
 gene
 
 0.02976
 
 0.37237
 
 Not queried
 
  4.2198274
 
 2.86e-02
 
 1.00e+00
  
 
  
 20
 
  61847579
 
  61847878
 
  PDF   STATS   ENSEMBL   UCSC 
 
  300
 
  8
 
 25851
 
 tss;island
 
 0.02385
 
 0.23316
 
 Not queried
 
  4.2174757
 
 1.09e-03
 
 1.00e+00
  
 
  
 3
 
  62355319
 
  62355525
 
  PDF   STATS   ENSEMBL   UCSC 
 
  207
 
  4
 
 29203
 
 island
 
 0.77491
 
 0.13829
 
 Not queried
 
  4.2146093
 
 2.86e-02
 
 1.00e+00
  
 
  
 12
 
 122231614
 
 122231927
 
  PDF   STATS   ENSEMBL   UCSC 
 
  314
 
  6
 
 10051
 
 tss
 
 0.03116
 
 0.40493
 
 Not queried
 
  4.2136116
 
 2.16e-03
 
 1.00e+00
  
 
  
 8
 
 144852570
 
 144853338
 
  PDF   STATS   ENSEMBL   UCSC 
 
  769
 
  7
 
 40622
 
 island
 
 0.64413
 
 0.05249
 
 Not queried
 
  4.2088651
 
 2.33e-03
 
 1.00e+00
  
 
  
 7
 
   1329387
 
   1329592
 
  PDF   STATS   ENSEMBL   UCSC 
 
  206
 
  5
 
 38033
 
 island
 
 0.33030
 
 0.93582
 
 Not queried
 
  4.2087784
 
 7.94e-03
 
 1.00e+00
  
 
  
 6
 
  25992047
 
  25992401
 
  PDF   STATS   ENSEMBL   UCSC 
 
  355
 
  4
 
 35440
 
 island
 
 0.07156
 
 0.45830
 
 Not queried
 
  4.2073233
 
 2.86e-02
 
 1.00e+00
  
 
  
 17
 
  46805785
 
  46806134
 
  PDF   STATS   ENSEMBL   UCSC 
 
  350
 
  5
 
 18469
 
 island
 
 0.03782
 
 0.28130
 
 Not queried
 
  4.2036108
 
 5.56e-02
 
 1.00e+00
  
 
  
 17
 
  40274524
 
  40274811
 
  PDF   STATS   ENSEMBL   UCSC 
 
  288
 
  7
 
 17321
 
 tss;island
 
 0.09753
 
 0.66538
 
 Not queried
 
  4.2016952
 
 5.83e-04
 
 1.00e+00
  
 
  
 2
 
 241497412
 
 241497846
 
  PDF   STATS   ENSEMBL   UCSC 
 
  435
 
  8
 
 23943
 
 tss;island
 
 0.57153
 
 0.07070
 
 Not queried
 
  4.1996622
 
 4.66e-03
 
 1.00e+00
  
 
  
 5
 
 140235435
 
 140235607
 
  PDF   STATS   ENSEMBL   UCSC 
 
  173
 
  5
 
 32296
 
 tss
 
 0.08742
 
 0.62538
 
 Not queried
 
  4.1959848
 
 7.94e-03
 
 1.00e+00
  
 
  
 6
 
  90660913
 
  90661373
 
  PDF   STATS   ENSEMBL   UCSC 
 
  461
 
  5
 
 34351
 
 gene
 
 0.32682
 
 0.82532
 
 Not queried
 
  4.1941168
 
 7.94e-03
 
 1.00e+00
  
 
  
 6
 
  29717917
 
  29718119
 
  PDF   STATS   ENSEMBL   UCSC 
 
  203
 
  4
 
 34690
 
 tss;island
 
 0.71414
 
 0.10953
 
 Not queried
 
  4.1919616
 
 2.86e-02
 
 1.00e+00
  
 
  
 1
 
 228225533
 
 228225886
 
  PDF   STATS   ENSEMBL   UCSC 
 
  354
 
  5
 
 1042
 
 gene;island
 
 0.06634
 
 0.54695
 
 Not queried
 
  4.1867630
 
 7.94e-03
 
 1.00e+00
  
 
  
 9
 
 130700866
 
 130701076
 
  PDF   STATS   ENSEMBL   UCSC 
 
  211
 
  4
 
 40776
 
 tss;island
 
 0.03151
 
 0.61917
 
 Not queried
 
  4.1806699
 
 5.71e-02
 
 1.00e+00
  
 
  
 18
 
  52626476
 
  52626848
 
  PDF   STATS   ENSEMBL   UCSC 
 
  373
 
  5
 
 19252
 
 tss;island
 
 0.03715
 
 0.44394
 
 Not queried
 
  4.1796653
 
 5.56e-02
 
 1.00e+00
  
 
  
 2
 
 232260116
 
 232260305
 
  PDF   STATS   ENSEMBL   UCSC 
 
  190
 
  4
 
 23901
 
 tss;island
 
 0.04722
 
 0.46027
 
 Not queried
 
  4.1790501
 
 2.86e-02
 
 1.00e+00
  
 
  
 16
 
  90148388
 
  90148962
 
  PDF   STATS   ENSEMBL   UCSC 
 
  575
 
  5
 
 16247
 
 island
 
 0.01243
 
 0.39955
 
 Not queried
 
  4.1740905
 
 3.17e-02
 
 1.00e+00
  
 
  
 2
 
  85811292
 
  85811471
 
  PDF   STATS   ENSEMBL   UCSC 
 
  180
 
  4
 
 23445
 
 tss
 
 0.81584
 
 0.23183
 
 Not queried
 
  4.1731024
 
 2.86e-02
 
 1.00e+00
  
 
  
 6
 
 111580120
 
 111580469
 
  PDF   STATS   ENSEMBL   UCSC 
 
  350
 
  9
 
 35112
 
 tss
 
 0.43511
 
 0.02931
 
 Not queried
 
  4.1715717
 
 2.76e-03
 
 1.00e+00
  
 
  
 17
 
  46641708
 
  46642104
 
  PDF   STATS   ENSEMBL   UCSC 
 
  397
 
  4
 
 16617
 
 gene;island
 
 0.46726
 
 0.04857
 
 Not queried
 
  4.1702344
 
 2.86e-02
 
 1.00e+00
  
 
  
 22
 
  39378224
 
  39378382
 
  PDF   STATS   ENSEMBL   UCSC 
 
  159
 
  4
 
 27106
 
 tss
 
 0.81294
 
 0.27464
 
 Not queried
 
  4.1676735
 
 2.86e-02
 
 1.00e+00
  
 
  
 5
 
 140857474
 
 140858107
 
  PDF   STATS   ENSEMBL   UCSC 
 
  634
 
  8
 
 31781
 
 gene;island
 
 0.05060
 
 0.66636
 
 Not queried
 
  4.1669000
 
 3.79e-02
 
 1.00e+00
  
 
  
 1
 
  91191583
 
  91192162
 
  PDF   STATS   ENSEMBL   UCSC 
 
  580
 
  6
 
 3456
 
 island
 
 0.26052
 
 0.01147
 
 Not queried
 
  4.1614643
 
 1.52e-02
 
 1.00e+00
  
 
  
 5
 
   1295737
 
   1296007
 
  PDF   STATS   ENSEMBL   UCSC 
 
  271
 
  5
 
 31910
 
 tss;island
 
 0.84446
 
 0.35519
 
 Not queried
 
  4.1614512
 
 3.17e-02
 
 1.00e+00
  
 
  
 8
 
 145028093
 
 145028505
 
  PDF   STATS   ENSEMBL   UCSC 
 
  413
 
  9
 
 39903
 
 tss
 
 0.58388
 
 0.04045
 
 Not queried
 
  4.1609341
 
 7.77e-03
 
 1.00e+00
  
 
  
 7
 
  55000889
 
  55001207
 
  PDF   STATS   ENSEMBL   UCSC 
 
  319
 
  4
 
 38438
 
 island
 
 0.02463
 
 0.41266
 
 Not queried
 
  4.1598347
 
 5.71e-02
 
 1.00e+00
  
 
  
 3
 
 133464463
 
 133464971
 
  PDF   STATS   ENSEMBL   UCSC 
 
  509
 
  6
 
 28559
 
 tss;island
 
 0.41679
 
 0.91096
 
 Not queried
 
  4.1561302
 
 2.60e-02
 
 1.00e+00
  
 
  
 17
 
  76210025
 
  76210520
 
  PDF   STATS   ENSEMBL   UCSC 
 
  496
 
  6
 
 18784
 
 island
 
 0.03765
 
 0.45910
 
 Not queried
 
  4.1558249
 
 1.52e-02
 
 1.00e+00
  
 
  
 11
 
  49455851
 
  49456196
 
  PDF   STATS   ENSEMBL   UCSC 
 
  346
 
  4
 
 8254
 
 island
 
 0.21625
 
 0.82617
 
 Not queried
 
  4.1555105
 
 5.71e-02
 
 1.00e+00
  
 
  
 3
 
   8809306
 
   8809715
 
  PDF   STATS   ENSEMBL   UCSC 
 
  410
 
  4
 
 27604
 
 gene;island
 
 0.18181
 
 0.86461
 
 Not queried
 
  4.1536733
 
 2.86e-02
 
 1.00e+00
  
 
  
 16
 
  56701994
 
  56702158
 
  PDF   STATS   ENSEMBL   UCSC 
 
  165
 
  5
 
 15220
 
 tss
 
 0.13489
 
 0.73306
 
 Not queried
 
  4.1506439
 
 7.94e-03
 
 1.00e+00
  
 
  
 16
 
  56701868
 
  56702158
 
  PDF   STATS   ENSEMBL   UCSC 
 
  291
 
  7
 
 15921
 
 island
 
 0.13489
 
 0.73306
 
 Not queried
 
  4.1506439
 
 5.83e-04
 
 1.00e+00
  
 
  
 8
 
   6692785
 
   6693540
 
  PDF   STATS   ENSEMBL   UCSC 
 
  756
 
 10
 
 39990
 
 island
 
 0.10103
 
 0.79271
 
 Not queried
 
  4.1485806
 
 7.25e-04
 
 1.00e+00
  
 
  
 12
 
 122231455
 
 122231927
 
  PDF   STATS   ENSEMBL   UCSC 
 
  473
 
  7
 
 10890
 
 island
 
 0.03483
 
 0.43080
 
 Not queried
 
  4.1448127
 
 5.83e-04
 
 1.00e+00
  
 
  
 4
 
  38869436
 
  38869779
 
  PDF   STATS   ENSEMBL   UCSC 
 
  344
 
  8
 
 29934
 
 gene
 
 0.37171
 
 0.02956
 
 Not queried
 
  4.1446699
 
 2.95e-03
 
 1.00e+00
  
 
  
 19
 
  40972228
 
  40972683
 
  PDF   STATS   ENSEMBL   UCSC 
 
  456
 
  4
 
 20755
 
 tss;island
 
 0.08485
 
 0.68587
 
 Not queried
 
  4.1431992
 
 2.00e-01
 
 1.00e+00
  
 
  
 5
 
 178368071
 
 178368205
 
  PDF   STATS   ENSEMBL   UCSC 
 
  135
 
  6
 
 32485
 
 tss
 
 0.37367
 
 0.02452
 
 Not queried
 
  4.1413564
 
 2.16e-03
 
 1.00e+00
  
 
  
 12
 
 115105220
 
 115105710
 
  PDF   STATS   ENSEMBL   UCSC 
 
  491
 
  5
 
 10817
 
 island
 
 0.55553
 
 0.04133
 
 Not queried
 
  4.1410344
 
 1.59e-02
 
 1.00e+00
  
 
  
 19
 
   7553700
 
   7554201
 
  PDF   STATS   ENSEMBL   UCSC 
 
  502
 
  9
 
 21413
 
 island
 
 0.04998
 
 0.47408
 
 Not queried
 
  4.1386402
 
 1.06e-02
 
 1.00e+00
  
 
  
 4
 
   1167230
 
   1167423
 
  PDF   STATS   ENSEMBL   UCSC 
 
  194
 
  5
 
 29815
 
 gene;tss;island
 
 0.14766
 
 0.88294
 
 Not queried
 
  4.1373029
 
 7.94e-03
 
 1.00e+00
  
 
  
 8
 
   8580376
 
   8580960
 
  PDF   STATS   ENSEMBL   UCSC 
 
  585
 
  6
 
 39993
 
 island
 
 0.06475
 
 0.60774
 
 Not queried
 
  4.1367877
 
 4.11e-02
 
 1.00e+00
  
 
  
 X
 
 152938750
 
 152938907
 
  PDF   STATS   ENSEMBL   UCSC 
 
  158
 
  4
 
 41582
 
 tss
 
 0.25675
 
 0.80746
 
 Not queried
 
  4.1336359
 
 2.86e-02
 
 1.00e+00
  
 
  
 3
 
 159756648
 
 159757071
 
  PDF   STATS   ENSEMBL   UCSC 
 
  424
 
  6
 
 29582
 
 island
 
 0.44445
 
 0.06295
 
 Not queried
 
  4.1322633
 
 2.16e-03
 
 1.00e+00
  
 
  
 1
 
 176998705
 
 176999034
 
  PDF   STATS   ENSEMBL   UCSC 
 
  330
 
  4
 
 894
 
 gene;tss
 
 0.18178
 
 0.67190
 
 Not queried
 
  4.1317687
 
 1.14e-01
 
 1.00e+00
  
 
  
 Y
 
  22737391
 
  22737594
 
  PDF   STATS   ENSEMBL   UCSC 
 
  204
 
  6
 
 42173
 
 tss
 
 0.02924
 
 0.33019
 
 Not queried
 
  4.1289676
 
 2.16e-03
 
 1.00e+00
  
 
  
 1
 
  12538341
 
  12538678
 
  PDF   STATS   ENSEMBL   UCSC 
 
  338
 
  4
 
 232
 
 gene
 
 0.17887
 
 0.78850
 
 Not queried
 
  4.1233263
 
 2.86e-02
 
 1.00e+00
  
 
  
 11
 
  62211990
 
  62212195
 
  PDF   STATS   ENSEMBL   UCSC 
 
  206
 
  4
 
 6618
 
 gene;island
 
 0.70585
 
 0.13263
 
 Not queried
 
  4.1208525
 
 2.86e-02
 
 1.00e+00
  
 
  
 6
 
  24583477
 
  24583848
 
  PDF   STATS   ENSEMBL   UCSC 
 
  372
 
  5
 
 33528
 
 gene
 
 0.27676
 
 0.03913
 
 Not queried
 
  4.1196369
 
 9.52e-02
 
 1.00e+00
  
 
  
 15
 
  52404887
 
  52405162
 
  PDF   STATS   ENSEMBL   UCSC 
 
  276
 
  8
 
 14119
 
 island
 
 0.15477
 
 0.87139
 
 Not queried
 
  4.1194840
 
 2.07e-02
 
 1.00e+00
  
 
  
 6
 
  78176473
 
  78176847
 
  PDF   STATS   ENSEMBL   UCSC 
 
  375
 
  6
 
 36208
 
 island
 
 0.51367
 
 0.05423
 
 Not queried
 
  4.1144280
 
 2.60e-02
 
 1.00e+00
  
 
  
 3
 
  21447391
 
  21447667
 
  PDF   STATS   ENSEMBL   UCSC 
 
  277
 
  4
 
 27657
 
 gene
 
 0.22866
 
 0.86197
 
 Not queried
 
  4.1122294
 
 2.86e-02
 
 1.00e+00
  
 
  
 1
 
  26856408
 
  26856648
 
  PDF   STATS   ENSEMBL   UCSC 
 
  241
 
  4
 
 341
 
 gene;island
 
 0.03046
 
 0.33597
 
 Not queried
 
  4.1119265
 
 2.86e-02
 
 1.00e+00
  
 
  
 14
 
  24563410
 
  24563578
 
  PDF   STATS   ENSEMBL   UCSC 
 
  169
 
  4
 
 12697
 
 island
 
 0.03033
 
 0.24694
 
 Not queried
 
  4.1117614
 
 2.86e-02
 
 1.00e+00
  
 
  
 7
 
  22539908
 
  22540154
 
  PDF   STATS   ENSEMBL   UCSC 
 
  247
 
  5
 
 37400
 
 tss
 
 0.03317
 
 0.37217
 
 Not queried
 
  4.1109042
 
 7.94e-03
 
 1.00e+00
  
 
  
 7
 
 101005832
 
 101006089
 
  PDF   STATS   ENSEMBL   UCSC 
 
  258
 
  9
 
 37737
 
 tss;island
 
 0.01202
 
 0.18574
 
 Not queried
 
  4.1103556
 
 2.88e-04
 
 1.00e+00
  
 
  
 7
 
   2208345
 
   2208908
 
  PDF   STATS   ENSEMBL   UCSC 
 
  564
 
  6
 
 36732
 
 gene
 
 0.46695
 
 0.93835
 
 Not queried
 
  4.1094762
 
 2.16e-03
 
 1.00e+00
  
 
  
 6
 
 168418276
 
 168418527
 
  PDF   STATS   ENSEMBL   UCSC 
 
  252
 
  7
 
 35282
 
 tss
 
 0.38375
 
 0.87094
 
 Not queried
 
  4.1038854
 
 3.79e-02
 
 1.00e+00
  
 
  
 19
 
  49340489
 
  49340765
 
  PDF   STATS   ENSEMBL   UCSC 
 
  277
 
  4
 
 20102
 
 gene;tss;island
 
 0.07746
 
 0.50836
 
 Not queried
 
  4.1025427
 
 2.86e-02
 
 1.00e+00
  
 
  
 4
 
    206112
 
    206562
 
  PDF   STATS   ENSEMBL   UCSC 
 
  451
 
  8
 
 30666
 
 island
 
 0.82672
 
 0.18385
 
 Not queried
 
  4.1025399
 
 1.86e-03
 
 1.00e+00
  
 
  
 22
 
  17565455
 
  17565904
 
  PDF   STATS   ENSEMBL   UCSC 
 
  450
 
  9
 
 27229
 
 island
 
 0.33490
 
 0.03178
 
 Not queried
 
  4.1003608
 
 2.44e-02
 
 1.00e+00
  
 
  
 6
 
  29855110
 
  29855347
 
  PDF   STATS   ENSEMBL   UCSC 
 
  238
 
  7
 
 34692
 
 tss
 
 0.85167
 
 0.16228
 
 Not queried
 
  4.0997754
 
 4.08e-03
 
 1.00e+00
  
 
  
 1
 
 117112616
 
 117112854
 
  PDF   STATS   ENSEMBL   UCSC 
 
  239
 
  4
 
 707
 
 gene;island
 
 0.60279
 
 0.06211
 
 Not queried
 
  4.0975603
 
 2.86e-02
 
 1.00e+00
  
 
  
 19
 
  14016603
 
  14016822
 
  PDF   STATS   ENSEMBL   UCSC 
 
  220
 
  7
 
 19851
 
 gene
 
 0.52197
 
 0.02171
 
 Not queried
 
  4.0946972
 
 3.18e-01
 
 1.00e+00
  
 
  
 17
 
  18011879
 
  18012014
 
  PDF   STATS   ENSEMBL   UCSC 
 
  136
 
  5
 
 17139
 
 tss
 
 0.10198
 
 0.65898
 
 Not queried
 
  4.0946107
 
 7.94e-03
 
 1.00e+00
  
 
  
 7
 
  20818657
 
  20818928
 
  PDF   STATS   ENSEMBL   UCSC 
 
  272
 
  6
 
 38228
 
 island
 
 0.53274
 
 0.06662
 
 Not queried
 
  4.0925044
 
 2.16e-03
 
 1.00e+00
  
 
  
 16
 
  88590325
 
  88590467
 
  PDF   STATS   ENSEMBL   UCSC 
 
  143
 
  5
 
 14877
 
 gene;island
 
 0.32381
 
 0.89093
 
 Not queried
 
  4.0923476
 
 7.94e-03
 
 1.00e+00
  
 
  
 17
 
  39041110
 
  39041266
 
  PDF   STATS   ENSEMBL   UCSC 
 
  157
 
  4
 
 16534
 
 gene
 
 0.09222
 
 0.61049
 
 Not queried
 
  4.0922566
 
 2.86e-02
 
 1.00e+00
  
 
  
 17
 
  48585858
 
  48586147
 
  PDF   STATS   ENSEMBL   UCSC 
 
  290
 
  4
 
 16653
 
 gene;island
 
 0.41344
 
 0.04342
 
 Not queried
 
  4.0922084
 
 2.86e-02
 
 1.00e+00
  
 
  
 1
 
  13909522
 
  13909821
 
  PDF   STATS   ENSEMBL   UCSC 
 
  300
 
  4
 
 1257
 
 tss;island
 
 0.08018
 
 0.50670
 
 Not queried
 
  4.0919573
 
 2.86e-02
 
 1.00e+00
  
 
  
 20
 
  44657637
 
  44657812
 
  PDF   STATS   ENSEMBL   UCSC 
 
  176
 
  5
 
 25748
 
 tss
 
 0.22158
 
 0.82960
 
 Not queried
 
  4.0872813
 
 7.94e-03
 
 1.00e+00
  
 
  
 7
 
  88424985
 
  88425148
 
  PDF   STATS   ENSEMBL   UCSC 
 
  164
 
  6
 
 37016
 
 gene
 
 0.18686
 
 0.71301
 
 Not queried
 
  4.0828230
 
 2.16e-03
 
 1.00e+00
  
 
  
 3
 
  99904510
 
  99904650
 
  PDF   STATS   ENSEMBL   UCSC 
 
  141
 
  4
 
 28422
 
 tss
 
 0.68247
 
 0.95697
 
 Not queried
 
  4.0819557
 
 2.86e-02
 
 1.00e+00
  
 
  
 6
 
  41528198
 
  41528785
 
  PDF   STATS   ENSEMBL   UCSC 
 
  588
 
  8
 
 34264
 
 gene;island
 
 0.51785
 
 0.07539
 
 Not queried
 
  4.0811663
 
 6.99e-03
 
 1.00e+00
  
 
  
 19
 
  11784955
 
  11785337
 
  PDF   STATS   ENSEMBL   UCSC 
 
  383
 
  6
 
 19812
 
 gene
 
 0.06941
 
 0.57172
 
 Not queried
 
  4.0756067
 
 2.16e-03
 
 1.00e+00
  
 
  
 19
 
  52035146
 
  52035287
 
  PDF   STATS   ENSEMBL   UCSC 
 
  142
 
  4
 
 20961
 
 tss
 
 0.06897
 
 0.46861
 
 Not queried
 
  4.0753125
 
 1.14e-01
 
 1.00e+00
  
 
  
 3
 
  46742703
 
  46743199
 
  PDF   STATS   ENSEMBL   UCSC 
 
  497
 
  5
 
 29015
 
 island
 
 0.36378
 
 0.91206
 
 Not queried
 
  4.0741222
 
 7.94e-03
 
 1.00e+00
  
 
  
 7
 
 102553293
 
 102553457
 
  PDF   STATS   ENSEMBL   UCSC 
 
  165
 
  5
 
 37106
 
 gene
 
 0.30607
 
 0.87535
 
 Not queried
 
  4.0733660
 
 7.94e-03
 
 1.00e+00
  
 
  
 3
 
  62362660
 
  62363248
 
  PDF   STATS   ENSEMBL   UCSC 
 
  589
 
  5
 
 29207
 
 island
 
 0.86910
 
 0.16967
 
 Not queried
 
  4.0727283
 
 1.59e-02
 
 1.00e+00
  
 
  
 14
 
 104928289
 
 104928675
 
  PDF   STATS   ENSEMBL   UCSC 
 
  387
 
  4
 
 13176
 
 island
 
 0.03611
 
 0.52953
 
 Not queried
 
  4.0724252
 
 5.71e-02
 
 1.00e+00
  
 
  
 3
 
  44626029
 
  44626453
 
  PDF   STATS   ENSEMBL   UCSC 
 
  425
 
  7
 
 28215
 
 tss
 
 0.28186
 
 0.03272
 
 Not queried
 
  4.0700959
 
 1.17e-03
 
 1.00e+00
  
 
  
 X
 
  75392480
 
  75392755
 
  PDF   STATS   ENSEMBL   UCSC 
 
  276
 
  4
 
 41398
 
 tss
 
 0.47927
 
 0.04004
 
 Not queried
 
  4.0670152
 
 3.43e-01
 
 1.00e+00
  
 
  
 2
 
 164204628
 
 164205343
 
  PDF   STATS   ENSEMBL   UCSC 
 
  716
 
  7
 
 24789
 
 island
 
 0.04254
 
 0.52157
 
 Not queried
 
  4.0659038
 
 5.83e-04
 
 1.00e+00
  
 
  
 18
 
  19752292
 
  19752414
 
  PDF   STATS   ENSEMBL   UCSC 
 
  123
 
  4
 
 19035
 
 gene;island
 
 0.90414
 
 0.37035
 
 Not queried
 
  4.0641648
 
 2.86e-02
 
 1.00e+00
  
 
  
 22
 
  35937058
 
  35937319
 
  PDF   STATS   ENSEMBL   UCSC 
 
  262
 
  6
 
 27058
 
 tss
 
 0.59852
 
 0.03261
 
 Not queried
 
  4.0633010
 
 8.66e-03
 
 1.00e+00
  
 
  
 17
 
  33760230
 
  33760527
 
  PDF   STATS   ENSEMBL   UCSC 
 
  298
 
  5
 
 17237
 
 tss
 
 0.23982
 
 0.77188
 
 Not queried
 
  4.0583110
 
 7.94e-03
 
 1.00e+00
  
 
  
 X
 
  49042873
 
  49043355
 
  PDF   STATS   ENSEMBL   UCSC 
 
  483
 
  6
 
 41313
 
 tss;island
 
 0.31017
 
 0.01775
 
 Not queried
 
  4.0582642
 
 8.66e-03
 
 1.00e+00
  
 
  
 Y
 
  22737391
 
  22737663
 
  PDF   STATS   ENSEMBL   UCSC 
 
  273
 
  7
 
 42184
 
 island
 
 0.02270
 
 0.30004
 
 Not queried
 
  4.0516629
 
 5.83e-04
 
 1.00e+00
  
 
  
 7
 
  27196296
 
  27196555
 
  PDF   STATS   ENSEMBL   UCSC 
 
  260
 
  7
 
 37429
 
 tss
 
 0.03109
 
 0.33114
 
 Not queried
 
  4.0505165
 
 5.83e-04
 
 1.00e+00
  
 
  
 16
 
  88832476
 
  88832700
 
  PDF   STATS   ENSEMBL   UCSC 
 
  225
 
  6
 
 14887
 
 gene
 
 0.33568
 
 0.90007
 
 Not queried
 
  4.0504017
 
 2.16e-03
 
 1.00e+00
  
 
  
 11
 
   1320329
 
   1320634
 
  PDF   STATS   ENSEMBL   UCSC 
 
  306
 
  4
 
 6395
 
 gene;island
 
 0.36077
 
 0.88477
 
 Not queried
 
  4.0494868
 
 5.71e-02
 
 1.00e+00
  
 
  
 11
 
 124735089
 
 124735227
 
  PDF   STATS   ENSEMBL   UCSC 
 
  139
 
  7
 
 7764
 
 tss
 
 0.06791
 
 0.40594
 
 Not queried
 
  4.0458662
 
 2.33e-03
 
 1.00e+00
  
 
  
 1
 
 179050673
 
 179051253
 
  PDF   STATS   ENSEMBL   UCSC 
 
  581
 
  9
 
 3980
 
 island
 
 0.04981
 
 0.46360
 
 Not queried
 
  4.0431560
 
 1.85e-03
 
 1.00e+00
  
 
  
 6
 
  32119616
 
  32119691
 
  PDF   STATS   ENSEMBL   UCSC 
 
   76
 
  4
 
 33997
 
 gene
 
 0.24126
 
 0.83164
 
 Not queried
 
  4.0426096
 
 2.86e-02
 
 1.00e+00
  
 
  
 7
 
  25898206
 
  25898770
 
  PDF   STATS   ENSEMBL   UCSC 
 
  565
 
  7
 
 38261
 
 island
 
 0.73052
 
 0.12485
 
 Not queried
 
  4.0425157
 
 5.83e-04
 
 1.00e+00
  
 
  
 19
 
  35940425
 
  35940593
 
  PDF   STATS   ENSEMBL   UCSC 
 
  169
 
  4
 
 20667
 
 tss
 
 0.20292
 
 0.75996
 
 Not queried
 
  4.0367920
 
 2.86e-02
 
 1.00e+00
  
 
  
 17
 
  35300448
 
  35300874
 
  PDF   STATS   ENSEMBL   UCSC 
 
  427
 
  5
 
 18205
 
 island
 
 0.70351
 
 0.06500
 
 Not queried
 
  4.0366940
 
 1.59e-02
 
 1.00e+00
  
 
  
 8
 
 110987530
 
 110988444
 
  PDF   STATS   ENSEMBL   UCSC 
 
  915
 
  9
 
 39811
 
 tss;island
 
 0.03210
 
 0.27702
 
 Not queried
 
  4.0359549
 
 3.99e-03
 
 1.00e+00
  
 
  
 19
 
  12946231
 
  12946465
 
  PDF   STATS   ENSEMBL   UCSC 
 
  235
 
  6
 
 20479
 
 tss
 
 0.03281
 
 0.22419
 
 Not queried
 
  4.0343701
 
 2.16e-03
 
 1.00e+00
  
 
  
 13
 
  31506685
 
  31506773
 
  PDF   STATS   ENSEMBL   UCSC 
 
   89
 
  5
 
 11349
 
 tss
 
 0.84188
 
 0.22216
 
 Not queried
 
  4.0336085
 
 7.94e-03
 
 1.00e+00
  
 
  
 12
 
  99139301
 
  99139867
 
  PDF   STATS   ENSEMBL   UCSC 
 
  567
 
  6
 
 9236
 
 gene;island
 
 0.11185
 
 0.65121
 
 Not queried
 
  4.0327236
 
 2.16e-03
 
 1.00e+00
  
 
  
 14
 
  26674046
 
  26674563
 
  PDF   STATS   ENSEMBL   UCSC 
 
  518
 
  7
 
 12725
 
 island
 
 0.03684
 
 0.50617
 
 Not queried
 
  4.0315039
 
 2.33e-03
 
 1.00e+00
  
 
  
 10
 
  98144309
 
  98144512
 
  PDF   STATS   ENSEMBL   UCSC 
 
  204
 
  5
 
 4676
 
 gene;island
 
 0.33779
 
 0.89253
 
 Not queried
 
  4.0251617
 
 1.59e-02
 
 1.00e+00
  
 
  
 7
 
  33080496
 
  33080615
 
  PDF   STATS   ENSEMBL   UCSC 
 
  120
 
  4
 
 36897
 
 gene
 
 0.31453
 
 0.88060
 
 Not queried
 
  4.0245794
 
 2.86e-02
 
 1.00e+00
  
 
  
 20
 
  33146263
 
  33146515
 
  PDF   STATS   ENSEMBL   UCSC 
 
  253
 
  4
 
 25654
 
 tss
 
 0.02560
 
 0.29900
 
 Not queried
 
  4.0245141
 
 2.86e-02
 
 1.00e+00
  
 
  
 2
 
  96192273
 
  96192699
 
  PDF   STATS   ENSEMBL   UCSC 
 
  427
 
  4
 
 24479
 
 island
 
 0.21181
 
 0.74391
 
 Not queried
 
  4.0223403
 
 2.86e-02
 
 1.00e+00
  
 
  
 19
 
  38794554
 
  38794845
 
  PDF   STATS   ENSEMBL   UCSC 
 
  292
 
  6
 
 19979
 
 gene
 
 0.21134
 
 0.78404
 
 Not queried
 
  4.0172272
 
 2.16e-03
 
 1.00e+00
  
 
  
 14
 
  70316898
 
  70317239
 
  PDF   STATS   ENSEMBL   UCSC 
 
  342
 
  5
 
 12944
 
 island
 
 0.28112
 
 0.84244
 
 Not queried
 
  4.0140246
 
 1.59e-02
 
 1.00e+00
  
 
  
 6
 
 101329304
 
 101329504
 
  PDF   STATS   ENSEMBL   UCSC 
 
  201
 
  5
 
 35078
 
 tss
 
 0.53212
 
 0.05276
 
 Not queried
 
  4.0110773
 
 7.94e-03
 
 1.00e+00
  
 
  
 17
 
  48610072
 
  48610255
 
  PDF   STATS   ENSEMBL   UCSC 
 
  184
 
  4
 
 16654
 
 gene
 
 0.03490
 
 0.41122
 
 Not queried
 
  4.0073336
 
 2.86e-02
 
 1.00e+00
  
 
  
 22
 
  45072455
 
  45072724
 
  PDF   STATS   ENSEMBL   UCSC 
 
  270
 
  4
 
 26851
 
 gene
 
 0.23751
 
 0.78664
 
 Not queried
 
  4.0044295
 
 2.86e-02
 
 1.00e+00
  
 
  
 4
 
 147561669
 
 147562073
 
  PDF   STATS   ENSEMBL   UCSC 
 
  405
 
  4
 
 30102
 
 gene;island
 
 0.22227
 
 0.81826
 
 Not queried
 
  4.0009134
 
 2.86e-02
 
 1.00e+00
  
 
  
 17
 
    183931
 
    184131
 
  PDF   STATS   ENSEMBL   UCSC 
 
  201
 
  4
 
 16255
 
 gene;island
 
 0.28787
 
 0.85548
 
 Not queried
 
  3.9997223
 
 2.86e-02
 
 1.00e+00
  
 
  
 16
 
   2569911
 
   2569967
 
  PDF   STATS   ENSEMBL   UCSC 
 
   57
 
  4
 
 14577
 
 gene;tss;island
 
 0.43139
 
 0.92287
 
 Not queried
 
  3.9968910
 
 1.14e-01
 
 1.00e+00
  
 
  
 22
 
  24577142
 
  24577438
 
  PDF   STATS   ENSEMBL   UCSC 
 
  297
 
  6
 
 26975
 
 tss
 
 0.04665
 
 0.45386
 
 Not queried
 
  3.9964416
 
 2.16e-03
 
 1.00e+00
  
 
  
 3
 
 119421553
 
 119421667
 
  PDF   STATS   ENSEMBL   UCSC 
 
  115
 
  4
 
 28483
 
 tss;island
 
 0.50545
 
 0.06518
 
 Not queried
 
  3.9959796
 
 1.14e-01
 
 1.00e+00
  
 
  
 7
 
  75157456
 
  75157765
 
  PDF   STATS   ENSEMBL   UCSC 
 
  310
 
  7
 
 37589
 
 tss
 
 0.32677
 
 0.02228
 
 Not queried
 
  3.9938310
 
 5.83e-04
 
 1.00e+00
  
 
  
 17
 
  48046663
 
  48046859
 
  PDF   STATS   ENSEMBL   UCSC 
 
  197
 
  4
 
 16641
 
 gene;island
 
 0.05096
 
 0.34439
 
 Not queried
 
  3.9926295
 
 2.86e-02
 
 1.00e+00
  
 
  
 1
 
  29460817
 
  29461070
 
  PDF   STATS   ENSEMBL   UCSC 
 
  254
 
  4
 
 2953
 
 island
 
 0.26976
 
 0.81054
 
 Not queried
 
  3.9904077
 
 2.86e-02
 
 1.00e+00
  
 
  
 3
 
  52569026
 
  52569169
 
  PDF   STATS   ENSEMBL   UCSC 
 
  144
 
  5
 
 28342
 
 tss;island
 
 0.06614
 
 0.75540
 
 Not queried
 
  3.9886502
 
 1.51e-01
 
 1.00e+00
  
 
  
 11
 
    417540
 
    417643
 
  PDF   STATS   ENSEMBL   UCSC 
 
  104
 
  4
 
 6991
 
 tss;island
 
 0.09664
 
 0.61549
 
 Not queried
 
  3.9873238
 
 2.86e-02
 
 1.00e+00
  
 
  
 9
 
  95896749
 
  95897015
 
  PDF   STATS   ENSEMBL   UCSC 
 
  267
 
  4
 
 40757
 
 tss;island
 
 0.45250
 
 0.05099
 
 Not queried
 
  3.9825446
 
 2.00e-01
 
 1.00e+00
  
 
  
 2
 
 176956396
 
 176956841
 
  PDF   STATS   ENSEMBL   UCSC 
 
  446
 
  5
 
 23694
 
 tss;island
 
 0.78350
 
 0.24391
 
 Not queried
 
  3.9808305
 
 7.94e-03
 
 1.00e+00
  
 
  
 11
 
  63775071
 
  63775612
 
  PDF   STATS   ENSEMBL   UCSC 
 
  542
 
  5
 
 6640
 
 gene;island
 
 0.30583
 
 0.77957
 
 Not queried
 
  3.9773345
 
 7.94e-03
 
 1.00e+00
  
 
  
 3
 
  53529144
 
  53529655
 
  PDF   STATS   ENSEMBL   UCSC 
 
  512
 
  5
 
 27787
 
 gene;island
 
 0.25751
 
 0.03107
 
 Not queried
 
  3.9773260
 
 1.59e-02
 
 1.00e+00
  
 
  
 17
 
   8702698
 
   8702896
 
  PDF   STATS   ENSEMBL   UCSC 
 
  199
 
  6
 
 17089
 
 tss
 
 0.12502
 
 0.65645
 
 Not queried
 
  3.9748664
 
 2.16e-03
 
 1.00e+00
  
 
  
 16
 
  57125654
 
  57125836
 
  PDF   STATS   ENSEMBL   UCSC 
 
  183
 
  5
 
 15226
 
 tss;island
 
 0.17331
 
 0.02699
 
 Not queried
 
  3.9746512
 
 1.59e-02
 
 1.00e+00
  
 
  
 8
 
 144654887
 
 144655030
 
  PDF   STATS   ENSEMBL   UCSC 
 
  144
 
  4
 
 40607
 
 island
 
 0.23692
 
 0.73239
 
 Not queried
 
  3.9746096
 
 2.86e-02
 
 1.00e+00
  
 
  
 5
 
 147443067
 
 147443347
 
  PDF   STATS   ENSEMBL   UCSC 
 
  281
 
  4
 
 32351
 
 tss
 
 0.44469
 
 0.93019
 
 Not queried
 
  3.9740169
 
 2.86e-02
 
 1.00e+00
  
 
  
 4
 
  41880987
 
  41881417
 
  PDF   STATS   ENSEMBL   UCSC 
 
  431
 
  4
 
 30957
 
 island
 
 0.92186
 
 0.28024
 
 Not queried
 
  3.9729594
 
 2.86e-02
 
 1.00e+00
  
 
  
 14
 
  37051863
 
  37051949
 
  PDF   STATS   ENSEMBL   UCSC 
 
   87
 
  4
 
 12331
 
 tss
 
 0.04162
 
 0.55101
 
 Not queried
 
  3.9699846
 
 2.86e-02
 
 1.00e+00
  
 
  
 19
 
  35531639
 
  35532097
 
  PDF   STATS   ENSEMBL   UCSC 
 
  459
 
  5
 
 19944
 
 gene;island
 
 0.27227
 
 0.73666
 
 Not queried
 
  3.9676864
 
 1.51e-01
 
 1.00e+00
  
 
  
 8
 
 145018010
 
 145018300
 
  PDF   STATS   ENSEMBL   UCSC 
 
  291
 
  5
 
 39436
 
 gene;tss;island
 
 0.04283
 
 0.41171
 
 Not queried
 
  3.9672181
 
 7.94e-03
 
 1.00e+00
  
 
  
 5
 
 140551968
 
 140552163
 
  PDF   STATS   ENSEMBL   UCSC 
 
  196
 
  4
 
 32305
 
 tss
 
 0.04270
 
 0.17673
 
 Not queried
 
  3.9665766
 
 1.14e-01
 
 1.00e+00
  
 
  
 4
 
   1046821
 
   1047097
 
  PDF   STATS   ENSEMBL   UCSC 
 
  277
 
  4
 
 30716
 
 island
 
 0.23812
 
 0.87410
 
 Not queried
 
  3.9648526
 
 2.86e-02
 
 1.00e+00
  
 
  
 13
 
  21277342
 
  21277505
 
  PDF   STATS   ENSEMBL   UCSC 
 
  164
 
  4
 
 11551
 
 island
 
 0.36389
 
 0.03464
 
 Not queried
 
  3.9641044
 
 2.86e-02
 
 1.00e+00
  
 
  
 4
 
  62382932
 
  62383240
 
  PDF   STATS   ENSEMBL   UCSC 
 
  309
 
  4
 
 29967
 
 gene;island
 
 0.03297
 
 0.36076
 
 Not queried
 
  3.9624633
 
 2.86e-02
 
 1.00e+00
  
 
  
 10
 
  37414506
 
  37414768
 
  PDF   STATS   ENSEMBL   UCSC 
 
  263
 
  6
 
 5014
 
 tss
 
 0.25712
 
 0.88236
 
 Not queried
 
  3.9621517
 
 2.16e-03
 
 1.00e+00
  
 
  
 6
 
  29893926
 
  29894341
 
  PDF   STATS   ENSEMBL   UCSC 
 
  416
 
 16
 
 34693
 
 tss;island
 
 0.80704
 
 0.31106
 
 Not queried
 
  3.9593018
 
 2.29e-04
 
 1.00e+00
  
 
  
 3
 
 126853940
 
 126854423
 
  PDF   STATS   ENSEMBL   UCSC 
 
  484
 
  4
 
 29398
 
 island
 
 0.25881
 
 0.91258
 
 Not queried
 
  3.9578604
 
 5.71e-02
 
 1.00e+00
  
 
  
 10
 
   6020330
 
   6020585
 
  PDF   STATS   ENSEMBL   UCSC 
 
  256
 
  4
 
 4914
 
 tss;island
 
 0.76535
 
 0.09201
 
 Not queried
 
  3.9554689
 
 1.14e-01
 
 1.00e+00
  
 
  
 12
 
 114846313
 
 114846503
 
  PDF   STATS   ENSEMBL   UCSC 
 
  191
 
  7
 
 10008
 
 tss
 
 0.94564
 
 0.47037
 
 Not queried
 
  3.9545645
 
 5.83e-04
 
 1.00e+00
  
 
  
 12
 
  54378736
 
  54378896
 
  PDF   STATS   ENSEMBL   UCSC 
 
  161
 
  7
 
 9725
 
 tss
 
 0.01530
 
 0.21773
 
 Not queried
 
  3.9523945
 
 1.17e-03
 
 1.00e+00
  
 
  
 3
 
 192445388
 
 192445594
 
  PDF   STATS   ENSEMBL   UCSC 
 
  207
 
  7
 
 28749
 
 tss
 
 0.01359
 
 0.23222
 
 Not queried
 
  3.9521877
 
 5.83e-04
 
 1.00e+00
  
 
  
 17
 
  45772333
 
  45772800
 
  PDF   STATS   ENSEMBL   UCSC 
 
  468
 
  7
 
 18423
 
 island
 
 0.34597
 
 0.02909
 
 Not queried
 
  3.9500905
 
 5.83e-04
 
 1.00e+00
  
 
  
 2
 
 171568984
 
 171569381
 
  PDF   STATS   ENSEMBL   UCSC 
 
  398
 
  6
 
 22880
 
 gene
 
 0.76761
 
 0.15175
 
 Not queried
 
  3.9499543
 
 4.11e-02
 
 1.00e+00
  
 
  
 1
 
  51810337
 
  51811185
 
  PDF   STATS   ENSEMBL   UCSC 
 
  849
 
  8
 
 3254
 
 island
 
 0.15299
 
 0.65973
 
 Not queried
 
  3.9495524
 
 6.50e-02
 
 1.00e+00
  
 
  
 15
 
  37110774
 
  37110854
 
  PDF   STATS   ENSEMBL   UCSC 
 
   81
 
  4
 
 13539
 
 tss
 
 0.57473
 
 0.96315
 
 Not queried
 
  3.9489516
 
 2.86e-02
 
 1.00e+00
  
 
  
 1
 
 229567914
 
 229568467
 
  PDF   STATS   ENSEMBL   UCSC 
 
  554
 
  5
 
 1061
 
 gene;island
 
 0.20526
 
 0.86072
 
 Not queried
 
  3.9448752
 
 3.17e-02
 
 1.00e+00
  
 
  
 4
 
  41869086
 
  41869611
 
  PDF   STATS   ENSEMBL   UCSC 
 
  526
 
  7
 
 30955
 
 island
 
 0.28725
 
 0.04277
 
 Not queried
 
  3.9393063
 
 4.08e-03
 
 1.00e+00
  
 
  
 10
 
 105037349
 
 105037796
 
  PDF   STATS   ENSEMBL   UCSC 
 
  448
 
  4
 
 4725
 
 gene;island
 
 0.01495
 
 0.28714
 
 Not queried
 
  3.9381116
 
 2.86e-02
 
 1.00e+00
  
 
  
 7
 
  24797102
 
  24797884
 
  PDF   STATS   ENSEMBL   UCSC 
 
  783
 
 11
 
 37413
 
 tss
 
 0.51331
 
 0.03719
 
 Not queried
 
  3.9373602
 
 1.92e-02
 
 1.00e+00
  
 
  
 3
 
 129407365
 
 129407548
 
  PDF   STATS   ENSEMBL   UCSC 
 
  184
 
  5
 
 27901
 
 gene
 
 0.39670
 
 0.04416
 
 Not queried
 
  3.9345541
 
 7.94e-03
 
 1.00e+00
  
 
  
 4
 
   5710319
 
   5710411
 
  PDF   STATS   ENSEMBL   UCSC 
 
   93
 
  6
 
 29876
 
 gene;tss;island
 
 0.35200
 
 0.90102
 
 Not queried
 
  3.9345159
 
 2.16e-03
 
 1.00e+00
  
 
  
 16
 
  58533743
 
  58534157
 
  PDF   STATS   ENSEMBL   UCSC 
 
  415
 
  4
 
 14743
 
 gene;island
 
 0.18507
 
 0.77330
 
 Not queried
 
  3.9340958
 
 2.86e-02
 
 1.00e+00
  
 
  
 22
 
  42896105
 
  42896573
 
  PDF   STATS   ENSEMBL   UCSC 
 
  469
 
  6
 
 27149
 
 tss
 
 0.10396
 
 0.71699
 
 Not queried
 
  3.9320284
 
 2.16e-03
 
 1.00e+00
  
 
  
 8
 
  22926785
 
  22926911
 
  PDF   STATS   ENSEMBL   UCSC 
 
  127
 
  7
 
 39532
 
 tss
 
 0.43370
 
 0.03110
 
 Not queried
 
  3.9306809
 
 5.83e-04
 
 1.00e+00
  
 
  
 2
 
 200524002
 
 200524259
 
  PDF   STATS   ENSEMBL   UCSC 
 
  258
 
  4
 
 24956
 
 island
 
 0.14507
 
 0.69823
 
 Not queried
 
  3.9261823
 
 2.86e-02
 
 1.00e+00
  
 
  
 12
 
  91573135
 
  91573372
 
  PDF   STATS   ENSEMBL   UCSC 
 
  238
 
  4
 
 9217
 
 gene;tss
 
 0.15619
 
 0.73476
 
 Not queried
 
  3.9255508
 
 1.14e-01
 
 1.00e+00
  
 
  
 15
 
  68724690
 
  68725002
 
  PDF   STATS   ENSEMBL   UCSC 
 
  313
 
  4
 
 13731
 
 tss;island
 
 0.75425
 
 0.21941
 
 Not queried
 
  3.9248753
 
 1.14e-01
 
 1.00e+00
  
 
  
 6
 
  26104185
 
  26104560
 
  PDF   STATS   ENSEMBL   UCSC 
 
  376
 
  4
 
 33537
 
 gene
 
 0.03324
 
 0.30436
 
 Not queried
 
  3.9247377
 
 2.86e-02
 
 1.00e+00
  
 
  
 3
 
 138657291
 
 138657774
 
  PDF   STATS   ENSEMBL   UCSC 
 
  484
 
  5
 
 29485
 
 island
 
 0.61953
 
 0.15497
 
 Not queried
 
  3.9238755
 
 7.94e-03
 
 1.00e+00
  
 
  
 7
 
   1126249
 
   1126903
 
  PDF   STATS   ENSEMBL   UCSC 
 
  655
 
  8
 
 36686
 
 gene;tss;island
 
 0.04461
 
 0.50771
 
 Not queried
 
  3.9234738
 
 2.07e-02
 
 1.00e+00
  
 
  
 7
 
  34118464
 
  34118935
 
  PDF   STATS   ENSEMBL   UCSC 
 
  472
 
  5
 
 36898
 
 gene;island
 
 0.27258
 
 0.85040
 
 Not queried
 
  3.9231889
 
 7.94e-03
 
 1.00e+00
  
 
  
 1
 
  47695138
 
  47695475
 
  PDF   STATS   ENSEMBL   UCSC 
 
  338
 
  4
 
 3223
 
 island
 
 0.69923
 
 0.11959
 
 Not queried
 
  3.9225647
 
 2.86e-02
 
 1.00e+00
  
 
  
 12
 
  85673200
 
  85673347
 
  PDF   STATS   ENSEMBL   UCSC 
 
  148
 
  4
 
 9875
 
 tss;island
 
 0.49366
 
 0.04198
 
 Not queried
 
  3.9194418
 
 5.71e-02
 
 1.00e+00
  
 
  
 7
 
   4869981
 
   4870162
 
  PDF   STATS   ENSEMBL   UCSC 
 
  182
 
  4
 
 36796
 
 gene
 
 0.19025
 
 0.84394
 
 Not queried
 
  3.9177747
 
 5.71e-02
 
 1.00e+00
  
 
  
 2
 
  85811292
 
  85811662
 
  PDF   STATS   ENSEMBL   UCSC 
 
  371
 
  5
 
 24444
 
 island
 
 0.82066
 
 0.23241
 
 Not queried
 
  3.9177073
 
 7.94e-03
 
 1.00e+00
  
 
  
 12
 
  52300911
 
  52301163
 
  PDF   STATS   ENSEMBL   UCSC 
 
  253
 
  4
 
 9696
 
 tss
 
 0.10741
 
 0.64187
 
 Not queried
 
  3.9169320
 
 2.86e-02
 
 1.00e+00
  
 
  
 1
 
 175568216
 
 175568710
 
  PDF   STATS   ENSEMBL   UCSC 
 
  495
 
  4
 
 893
 
 gene;island
 
 0.38980
 
 0.88981
 
 Not queried
 
  3.9166790
 
 2.86e-02
 
 1.00e+00
  
 
  
 11
 
  27015656
 
  27015991
 
  PDF   STATS   ENSEMBL   UCSC 
 
  336
 
  5
 
 6523
 
 gene
 
 0.13558
 
 0.71504
 
 Not queried
 
  3.9102984
 
 7.94e-03
 
 1.00e+00
  
 
  
 5
 
 180486604
 
 180486879
 
  PDF   STATS   ENSEMBL   UCSC 
 
  276
 
  4
 
 31890
 
 gene;island
 
 0.07288
 
 0.51942
 
 Not queried
 
  3.9089564
 
 2.86e-02
 
 1.00e+00
  
 
  
 5
 
 139080952
 
 139081136
 
  PDF   STATS   ENSEMBL   UCSC 
 
  185
 
  4
 
 33115
 
 island
 
 0.41472
 
 0.06119
 
 Not queried
 
  3.9058807
 
 2.86e-02
 
 1.00e+00
  
 
  
 10
 
 104613767
 
 104613934
 
  PDF   STATS   ENSEMBL   UCSC 
 
  168
 
  6
 
 5314
 
 tss
 
 0.58593
 
 0.03881
 
 Not queried
 
  3.9035130
 
 1.80e-01
 
 1.00e+00
  
 
  
 4
 
    683060
 
    683240
 
  PDF   STATS   ENSEMBL   UCSC 
 
  181
 
  4
 
 30182
 
 tss;island
 
 0.35882
 
 0.85727
 
 Not queried
 
  3.9016424
 
 2.86e-02
 
 1.00e+00
  
 
  
 6
 
 108492642
 
 108492769
 
  PDF   STATS   ENSEMBL   UCSC 
 
  128
 
  4
 
 34374
 
 gene;island
 
 0.64773
 
 0.18973
 
 Not queried
 
  3.9007117
 
 2.86e-02
 
 1.00e+00
  
 
  
 6
 
  36355343
 
  36355825
 
  PDF   STATS   ENSEMBL   UCSC 
 
  483
 
  7
 
 36019
 
 island
 
 0.08891
 
 0.72764
 
 Not queried
 
  3.8971806
 
 5.83e-04
 
 1.00e+00
  
 
  
 7
 
  27281216
 
  27281964
 
  PDF   STATS   ENSEMBL   UCSC 
 
  749
 
 14
 
 37435
 
 tss;island
 
 0.58450
 
 0.13816
 
 Not queried
 
  3.8950407
 
 2.74e-02
 
 1.00e+00
  
 
  
 7
 
  24796981
 
  24797884
 
  PDF   STATS   ENSEMBL   UCSC 
 
  904
 
 12
 
 38254
 
 island
 
 0.38740
 
 0.03597
 
 Not queried
 
  3.8937239
 
 2.84e-02
 
 1.00e+00
  
 
  
 3
 
  49395574
 
  49395745
 
  PDF   STATS   ENSEMBL   UCSC 
 
  172
 
  4
 
 27739
 
 gene
 
 0.89485
 
 0.34741
 
 Not queried
 
  3.8929904
 
 2.86e-02
 
 1.00e+00
  
 
  
 4
 
   4387697
 
   4387917
 
  PDF   STATS   ENSEMBL   UCSC 
 
  221
 
  6
 
 30220
 
 tss;island
 
 0.33197
 
 0.02104
 
 Not queried
 
  3.8862713
 
 6.49e-02
 
 1.00e+00
  
 
  
 7
 
   1269693
 
   1270134
 
  PDF   STATS   ENSEMBL   UCSC 
 
  442
 
  4
 
 38026
 
 island
 
 0.04620
 
 0.36399
 
 Not queried
 
  3.8845788
 
 2.86e-02
 
 1.00e+00
  
 
  
 X
 
 132547142
 
 132547691
 
  PDF   STATS   ENSEMBL   UCSC 
 
  550
 
  5
 
 41109
 
 gene;island
 
 0.39833
 
 0.03016
 
 Not queried
 
  3.8844482
 
 1.51e-01
 
 1.00e+00
  
 
  
 1
 
 156182601
 
 156182774
 
  PDF   STATS   ENSEMBL   UCSC 
 
  174
 
  4
 
 2006
 
 tss
 
 0.42097
 
 0.04086
 
 Not queried
 
  3.8826691
 
 2.86e-02
 
 1.00e+00
  
 
  
 X
 
  82763219
 
  82763266
 
  PDF   STATS   ENSEMBL   UCSC 
 
   48
 
  5
 
 41406
 
 tss
 
 0.34870
 
 0.92921
 
 Not queried
 
  3.8809823
 
 7.94e-03
 
 1.00e+00
  
 
  
 6
 
  30848807
 
  30848846
 
  PDF   STATS   ENSEMBL   UCSC 
 
   40
 
  4
 
 35668
 
 island
 
 0.79741
 
 0.24249
 
 Not queried
 
  3.8800285
 
 2.86e-02
 
 1.00e+00
  
 
  
 1
 
   8271918
 
   8272277
 
  PDF   STATS   ENSEMBL   UCSC 
 
  360
 
  5
 
 2629
 
 island
 
 0.20812
 
 0.77899
 
 Not queried
 
  3.8788758
 
 7.94e-03
 
 1.00e+00
  
 
  
 2
 
 202483704
 
 202484320
 
  PDF   STATS   ENSEMBL   UCSC 
 
  617
 
  9
 
 24972
 
 island
 
 0.27023
 
 0.75519
 
 Not queried
 
  3.8757716
 
 1.85e-03
 
 1.00e+00
  
 
  
 5
 
  42756786
 
  42756876
 
  PDF   STATS   ENSEMBL   UCSC 
 
   91
 
  4
 
 31982
 
 tss
 
 0.91806
 
 0.37091
 
 Not queried
 
  3.8723967
 
 1.14e-01
 
 1.00e+00
  
 
  
 4
 
   1762881
 
   1763211
 
  PDF   STATS   ENSEMBL   UCSC 
 
  331
 
  4
 
 30749
 
 island
 
 0.07876
 
 0.69043
 
 Not queried
 
  3.8720253
 
 1.14e-01
 
 1.00e+00
  
 
  
 6
 
  35744188
 
  35744355
 
  PDF   STATS   ENSEMBL   UCSC 
 
  168
 
  4
 
 34867
 
 tss;island
 
 0.29183
 
 0.03591
 
 Not queried
 
  3.8707852
 
 2.00e-01
 
 1.00e+00
  
 
  
 X
 
 138276904
 
 138277085
 
  PDF   STATS   ENSEMBL   UCSC 
 
  182
 
  4
 
 41125
 
 gene;island
 
 0.01957
 
 0.24732
 
 Not queried
 
  3.8704862
 
 2.86e-02
 
 1.00e+00
  
 
  
 5
 
  72740460
 
  72740748
 
  PDF   STATS   ENSEMBL   UCSC 
 
  289
 
  5
 
 32810
 
 island
 
 0.74444
 
 0.20036
 
 Not queried
 
  3.8704484
 
 3.17e-02
 
 1.00e+00
  
 
  
 2
 
  21022431
 
  21022699
 
  PDF   STATS   ENSEMBL   UCSC 
 
  269
 
  4
 
 22555
 
 gene
 
 0.92281
 
 0.49336
 
 Not queried
 
  3.8696645
 
 2.86e-02
 
 1.00e+00
  
 
  
 3
 
 157813327
 
 157813810
 
  PDF   STATS   ENSEMBL   UCSC 
 
  484
 
  6
 
 29572
 
 island
 
 0.75843
 
 0.17305
 
 Not queried
 
  3.8683992
 
 4.33e-03
 
 1.00e+00
  
 
  
 10
 
  11783794
 
  11784473
 
  PDF   STATS   ENSEMBL   UCSC 
 
  680
 
 10
 
 5528
 
 island
 
 0.09289
 
 0.63106
 
 Not queried
 
  3.8673689
 
 1.05e-03
 
 1.00e+00
  
 
  
 19
 
  46318633
 
  46318918
 
  PDF   STATS   ENSEMBL   UCSC 
 
  286
 
  4
 
 20845
 
 tss
 
 0.32216
 
 0.04190
 
 Not queried
 
  3.8669636
 
 2.86e-02
 
 1.00e+00
  
 
  
 3
 
  46742865
 
  46743199
 
  PDF   STATS   ENSEMBL   UCSC 
 
  335
 
  4
 
 27712
 
 gene
 
 0.32310
 
 0.85802
 
 Not queried
 
  3.8669550
 
 2.86e-02
 
 1.00e+00
  
 
  
 10
 
 129535138
 
 129535509
 
  PDF   STATS   ENSEMBL   UCSC 
 
  372
 
  6
 
 5418
 
 tss
 
 0.05016
 
 0.44190
 
 Not queried
 
  3.8661072
 
 2.16e-03
 
 1.00e+00
  
 
  
 19
 
  35068555
 
  35068788
 
  PDF   STATS   ENSEMBL   UCSC 
 
  234
 
  4
 
 21853
 
 island
 
 0.23221
 
 0.82786
 
 Not queried
 
  3.8653054
 
 2.86e-02
 
 1.00e+00
  
 
  
 12
 
 103311088
 
 103311761
 
  PDF   STATS   ENSEMBL   UCSC 
 
  674
 
  9
 
 10699
 
 island
 
 0.02814
 
 0.34054
 
 Not queried
 
  3.8631115
 
 1.85e-03
 
 1.00e+00
  
 
  
 1
 
  32741454
 
  32741619
 
  PDF   STATS   ENSEMBL   UCSC 
 
  166
 
  4
 
 392
 
 gene;island
 
 0.34995
 
 0.81320
 
 Not queried
 
  3.8586746
 
 2.86e-02
 
 1.00e+00
  
 
  
 17
 
  57915665
 
  57915773
 
  PDF   STATS   ENSEMBL   UCSC 
 
  109
 
  4
 
 16680
 
 gene
 
 0.03018
 
 0.38278
 
 Not queried
 
  3.8579800
 
 2.86e-02
 
 1.00e+00
  
 
  
 5
 
  72598865
 
  72599228
 
  PDF   STATS   ENSEMBL   UCSC 
 
  364
 
  4
 
 32808
 
 island
 
 0.05872
 
 0.48406
 
 Not queried
 
  3.8491839
 
 2.00e-01
 
 1.00e+00
  
 
  
 7
 
 157294324
 
 157294502
 
  PDF   STATS   ENSEMBL   UCSC 
 
  179
 
  4
 
 39043
 
 island
 
 0.14360
 
 0.76987
 
 Not queried
 
  3.8453669
 
 2.86e-02
 
 1.00e+00
  
 
  
 22
 
  50329860
 
  50330022
 
  PDF   STATS   ENSEMBL   UCSC 
 
  163
 
  4
 
 27566
 
 island
 
 0.03747
 
 0.38346
 
 Not queried
 
  3.8402783
 
 2.86e-02
 
 1.00e+00
  
 
  
 X
 
   2984578
 
   2984799
 
  PDF   STATS   ENSEMBL   UCSC 
 
  222
 
  5
 
 41170
 
 tss
 
 0.04540
 
 0.42273
 
 Not queried
 
  3.8376347
 
 7.94e-03
 
 1.00e+00
  
 
  
 22
 
  18632404
 
  18632618
 
  PDF   STATS   ENSEMBL   UCSC 
 
  215
 
  5
 
 26908
 
 tss
 
 0.02771
 
 0.33411
 
 Not queried
 
  3.8359552
 
 5.56e-02
 
 1.00e+00
  
 
  
 19
 
  12707809
 
  12708001
 
  PDF   STATS   ENSEMBL   UCSC 
 
  193
 
  4
 
 19820
 
 gene;island
 
 0.94764
 
 0.56483
 
 Not queried
 
  3.8358233
 
 2.86e-02
 
 1.00e+00
  
 
  
 3
 
 126113327
 
 126114064
 
  PDF   STATS   ENSEMBL   UCSC 
 
  738
 
 13
 
 29389
 
 island
 
 0.29859
 
 0.86290
 
 Not queried
 
  3.8335390
 
 7.95e-04
 
 1.00e+00
  
 
  
 12
 
  91347805
 
  91348242
 
  PDF   STATS   ENSEMBL   UCSC 
 
  438
 
  4
 
 9215
 
 gene;island
 
 0.62744
 
 0.86892
 
 Not queried
 
  3.8320826
 
 3.43e-01
 
 1.00e+00
  
 
  
 19
 
  17392656
 
  17392923
 
  PDF   STATS   ENSEMBL   UCSC 
 
  268
 
  4
 
 19880
 
 gene;island
 
 0.30166
 
 0.03515
 
 Not queried
 
  3.8304496
 
 2.86e-02
 
 1.00e+00
  
 
  
 2
 
 231712722
 
 231712981
 
  PDF   STATS   ENSEMBL   UCSC 
 
  260
 
  5
 
 25137
 
 island
 
 0.73365
 
 0.16275
 
 Not queried
 
  3.8247915
 
 9.52e-02
 
 1.00e+00
  
 
  
 5
 
    851101
 
    851458
 
  PDF   STATS   ENSEMBL   UCSC 
 
  358
 
  6
 
 31903
 
 tss;island
 
 0.20964
 
 0.75987
 
 Not queried
 
  3.8216559
 
 2.16e-03
 
 1.00e+00
  
 
  
 16
 
   3017902
 
   3018262
 
  PDF   STATS   ENSEMBL   UCSC 
 
  361
 
  4
 
 14589
 
 gene;tss;island
 
 0.06632
 
 0.56673
 
 Not queried
 
  3.8206800
 
 2.86e-02
 
 1.00e+00
  
 
  
 3
 
  46037408
 
  46037608
 
  PDF   STATS   ENSEMBL   UCSC 
 
  201
 
  6
 
 28231
 
 tss;island
 
 0.39792
 
 0.05926
 
 Not queried
 
  3.8193847
 
 8.66e-03
 
 1.00e+00
  
 
  
 2
 
 223916502
 
 223916952
 
  PDF   STATS   ENSEMBL   UCSC 
 
  451
 
  7
 
 25119
 
 island
 
 0.45487
 
 0.93262
 
 Not queried
 
  3.8193138
 
 5.83e-04
 
 1.00e+00
  
 
  
 X
 
  48815643
 
  48815856
 
  PDF   STATS   ENSEMBL   UCSC 
 
  214
 
  7
 
 41303
 
 tss;island
 
 0.24686
 
 0.01270
 
 Not queried
 
  3.8185370
 
 4.08e-03
 
 1.00e+00
  
 
  
 8
 
   1365049
 
   1365906
 
  PDF   STATS   ENSEMBL   UCSC 
 
  858
 
  7
 
 39960
 
 island
 
 0.32627
 
 0.87228
 
 Not queried
 
  3.8179780
 
 6.99e-03
 
 1.00e+00
  
 
  
 10
 
  46993502
 
  46993586
 
  PDF   STATS   ENSEMBL   UCSC 
 
   85
 
  4
 
 5700
 
 island
 
 0.48900
 
 0.06646
 
 Not queried
 
  3.8155388
 
 2.86e-02
 
 1.00e+00
  
 
  
 11
 
  69706831
 
  69706981
 
  PDF   STATS   ENSEMBL   UCSC 
 
  151
 
  4
 
 8578
 
 island
 
 0.11461
 
 0.64538
 
 Not queried
 
  3.8139490
 
 2.86e-02
 
 1.00e+00
  
 
  
 6
 
 159290838
 
 159291228
 
  PDF   STATS   ENSEMBL   UCSC 
 
  391
 
  4
 
 36539
 
 island
 
 0.46801
 
 0.92818
 
 Not queried
 
  3.8107875
 
 2.86e-02
 
 1.00e+00
  
 
  
 11
 
  87908631
 
  87908938
 
  PDF   STATS   ENSEMBL   UCSC 
 
  308
 
  8
 
 7591
 
 tss
 
 0.24226
 
 0.03201
 
 Not queried
 
  3.8082907
 
 4.66e-03
 
 1.00e+00
  
 
  
 17
 
  43506728
 
  43507153
 
  PDF   STATS   ENSEMBL   UCSC 
 
  426
 
  4
 
 16600
 
 gene
 
 0.44085
 
 0.07123
 
 Not queried
 
  3.8080903
 
 5.71e-02
 
 1.00e+00
  
 
  
 1
 
 159750305
 
 159750570
 
  PDF   STATS   ENSEMBL   UCSC 
 
  266
 
  5
 
 2026
 
 tss;island
 
 0.01292
 
 0.11528
 
 Not queried
 
  3.8043015
 
 3.17e-02
 
 1.00e+00
  
 
  
 11
 
  87908558
 
  87908938
 
  PDF   STATS   ENSEMBL   UCSC 
 
  381
 
  9
 
 8695
 
 island
 
 0.20440
 
 0.03294
 
 Not queried
 
  3.8028328
 
 3.99e-03
 
 1.00e+00
  
 
  
 5
 
 170740547
 
 170740937
 
  PDF   STATS   ENSEMBL   UCSC 
 
  391
 
  6
 
 33316
 
 island
 
 0.94813
 
 0.47140
 
 Not queried
 
  3.8002885
 
 2.16e-03
 
 1.00e+00
  
 
  
 2
 
  27346296
 
  27346593
 
  PDF   STATS   ENSEMBL   UCSC 
 
  298
 
  5
 
 23217
 
 tss
 
 0.07527
 
 0.53128
 
 Not queried
 
  3.7996104
 
 1.51e-01
 
 1.00e+00
  
 
  
 2
 
  27346296
 
  27346938
 
  PDF   STATS   ENSEMBL   UCSC 
 
  643
 
  7
 
 24131
 
 island
 
 0.07527
 
 0.51047
 
 Not queried
 
  3.7996104
 
 2.62e-02
 
 1.00e+00
  
 
  
 3
 
 142839837
 
 142840240
 
  PDF   STATS   ENSEMBL   UCSC 
 
  404
 
  5
 
 27938
 
 gene;island
 
 0.05005
 
 0.45878
 
 Not queried
 
  3.7993270
 
 7.94e-03
 
 1.00e+00
  
 
  
 2
 
 132152167
 
 132152543
 
  PDF   STATS   ENSEMBL   UCSC 
 
  377
 
  4
 
 24692
 
 island
 
 0.06867
 
 0.47574
 
 Not queried
 
  3.7987950
 
 2.86e-02
 
 1.00e+00
  
 
  
 4
 
 155413392
 
 155413789
 
  PDF   STATS   ENSEMBL   UCSC 
 
  398
 
  4
 
 30596
 
 tss;island
 
 0.47909
 
 0.06723
 
 Not queried
 
  3.7970573
 
 1.14e-01
 
 1.00e+00
  
 
  
 10
 
  93805441
 
  93805870
 
  PDF   STATS   ENSEMBL   UCSC 
 
  430
 
  5
 
 5917
 
 island
 
 0.25707
 
 0.78654
 
 Not queried
 
  3.7960843
 
 7.94e-03
 
 1.00e+00
  
 
  
 11
 
  63382047
 
  63382095
 
  PDF   STATS   ENSEMBL   UCSC 
 
   49
 
  5
 
 7355
 
 tss;island
 
 0.12710
 
 0.01039
 
 Not queried
 
  3.7936590
 
 7.94e-03
 
 1.00e+00
  
 
  
 3
 
  49066976
 
  49067151
 
  PDF   STATS   ENSEMBL   UCSC 
 
  176
 
  7
 
 28274
 
 tss
 
 0.03096
 
 0.31761
 
 Not queried
 
  3.7931218
 
 5.83e-04
 
 1.00e+00
  
 
  
 19
 
  40324850
 
  40325100
 
  PDF   STATS   ENSEMBL   UCSC 
 
  251
 
  7
 
 20742
 
 tss
 
 0.04368
 
 0.44299
 
 Not queried
 
  3.7930997
 
 1.75e-02
 
 1.00e+00
  
 
  
 1
 
   2324205
 
   2324396
 
  PDF   STATS   ENSEMBL   UCSC 
 
  192
 
  4
 
 81
 
 gene;tss;island
 
 0.31422
 
 0.81457
 
 Not queried
 
  3.7912980
 
 2.86e-02
 
 1.00e+00
  
 
  
 8
 
  99961768
 
  99962118
 
  PDF   STATS   ENSEMBL   UCSC 
 
  351
 
  4
 
 39312
 
 gene;island
 
 0.87490
 
 0.25212
 
 Not queried
 
  3.7860936
 
 2.86e-02
 
 1.00e+00
  
 
  
 10
 
  94180383
 
  94180913
 
  PDF   STATS   ENSEMBL   UCSC 
 
  531
 
  5
 
 5921
 
 island
 
 0.45816
 
 0.06552
 
 Not queried
 
  3.7843370
 
 9.52e-02
 
 1.00e+00
  
 
  
 17
 
  80797948
 
  80798078
 
  PDF   STATS   ENSEMBL   UCSC 
 
  131
 
  7
 
 17748
 
 tss
 
 0.37842
 
 0.90492
 
 Not queried
 
  3.7836680
 
 5.83e-04
 
 1.00e+00
  
 
  
 7
 
 158243369
 
 158243730
 
  PDF   STATS   ENSEMBL   UCSC 
 
  362
 
  4
 
 37308
 
 gene
 
 0.47369
 
 0.89030
 
 Not queried
 
  3.7829409
 
 2.86e-02
 
 1.00e+00
  
 
  
 16
 
  46918956
 
  46919194
 
  PDF   STATS   ENSEMBL   UCSC 
 
  239
 
  5
 
 14712
 
 gene;island
 
 0.33213
 
 0.04392
 
 Not queried
 
  3.7828710
 
 5.56e-02
 
 1.00e+00
  
 
  
 22
 
  18267969
 
  18268249
 
  PDF   STATS   ENSEMBL   UCSC 
 
  281
 
  4
 
 27236
 
 island
 
 0.22822
 
 0.82955
 
 Not queried
 
  3.7793774
 
 2.86e-02
 
 1.00e+00
  
 
  
 2
 
 175208588
 
 175208761
 
  PDF   STATS   ENSEMBL   UCSC 
 
  174
 
  4
 
 24838
 
 island
 
 0.32546
 
 0.03351
 
 Not queried
 
  3.7787582
 
 2.86e-02
 
 1.00e+00
  
 
  
 6
 
  88877064
 
  88877413
 
  PDF   STATS   ENSEMBL   UCSC 
 
  350
 
  4
 
 36254
 
 island
 
 0.35620
 
 0.04668
 
 Not queried
 
  3.7775791
 
 2.86e-02
 
 1.00e+00
  
 
  
 5
 
 115697214
 
 115697696
 
  PDF   STATS   ENSEMBL   UCSC 
 
  483
 
  5
 
 32969
 
 island
 
 0.36984
 
 0.76857
 
 Not queried
 
  3.7764380
 
 7.94e-03
 
 1.00e+00
  
 
  
 7
 
 158854447
 
 158854856
 
  PDF   STATS   ENSEMBL   UCSC 
 
  410
 
  4
 
 37318
 
 gene;island
 
 0.48009
 
 0.96095
 
 Not queried
 
  3.7757452
 
 4.86e-01
 
 1.00e+00
  
 
  
 4
 
  10686394
 
  10686610
 
  PDF   STATS   ENSEMBL   UCSC 
 
  217
 
  4
 
 30254
 
 tss
 
 0.42512
 
 0.91380
 
 Not queried
 
  3.7746174
 
 2.86e-02
 
 1.00e+00
  
 
  
 10
 
   1416546
 
   1416920
 
  PDF   STATS   ENSEMBL   UCSC 
 
  375
 
  6
 
 4449
 
 gene;island
 
 0.26774
 
 0.76757
 
 Not queried
 
  3.7744281
 
 2.16e-03
 
 1.00e+00
  
 
  
 14
 
  77737661
 
  77737800
 
  PDF   STATS   ENSEMBL   UCSC 
 
  140
 
  4
 
 12501
 
 tss
 
 0.26696
 
 0.84202
 
 Not queried
 
  3.7741413
 
 2.86e-02
 
 1.00e+00
  
 
  
 12
 
 132514266
 
 132514508
 
  PDF   STATS   ENSEMBL   UCSC 
 
  243
 
  6
 
 9403
 
 gene;island
 
 0.42861
 
 0.90694
 
 Not queried
 
  3.7727852
 
 2.16e-03
 
 1.00e+00
  
 
  
 16
 
  15489542
 
  15489968
 
  PDF   STATS   ENSEMBL   UCSC 
 
  427
 
  6
 
 15694
 
 island
 
 0.03307
 
 0.35429
 
 Not queried
 
  3.7667160
 
 4.11e-02
 
 1.00e+00
  
 
  
 17
 
  45569959
 
  45570168
 
  PDF   STATS   ENSEMBL   UCSC 
 
  210
 
  4
 
 18421
 
 island
 
 0.01419
 
 0.51074
 
 Not queried
 
  3.7637156
 
 5.71e-02
 
 1.00e+00
  
 
  
 17
 
   1202015
 
   1202252
 
  PDF   STATS   ENSEMBL   UCSC 
 
  238
 
  4
 
 16276
 
 gene
 
 0.16184
 
 0.59440
 
 Not queried
 
  3.7637148
 
 2.86e-02
 
 1.00e+00
  
 
  
 7
 
 156812806
 
 156813602
 
  PDF   STATS   ENSEMBL   UCSC 
 
  797
 
  8
 
 39032
 
 island
 
 0.79079
 
 0.20166
 
 Not queried
 
  3.7623539
 
 3.11e-04
 
 1.00e+00
  
 
  
 17
 
  76128522
 
  76128683
 
  PDF   STATS   ENSEMBL   UCSC 
 
  162
 
  7
 
 17667
 
 tss
 
 0.12705
 
 0.70107
 
 Not queried
 
  3.7597883
 
 5.83e-04
 
 1.00e+00
  
 
  
 6
 
  50813551
 
  50814089
 
  PDF   STATS   ENSEMBL   UCSC 
 
  539
 
  5
 
 34303
 
 gene;island
 
 0.25184
 
 0.05973
 
 Not queried
 
  3.7573140
 
 5.56e-02
 
 1.00e+00
  
 
  
 14
 
  65005951
 
  65006359
 
  PDF   STATS   ENSEMBL   UCSC 
 
  409
 
 10
 
 12430
 
 tss;island
 
 0.54559
 
 0.95729
 
 Not queried
 
  3.7506999
 
 1.50e-03
 
 1.00e+00
  
 
  
 1
 
  68629000
 
  68629133
 
  PDF   STATS   ENSEMBL   UCSC 
 
  134
 
  4
 
 574
 
 gene;island
 
 0.12093
 
 0.58553
 
 Not queried
 
  3.7489064
 
 2.86e-02
 
 1.00e+00
  
 
  
 2
 
   3383078
 
   3383413
 
  PDF   STATS   ENSEMBL   UCSC 
 
  336
 
  5
 
 23122
 
 tss
 
 0.12765
 
 0.02151
 
 Not queried
 
  3.7484708
 
 3.10e-01
 
 1.00e+00
  
 
  
 1
 
 174968745
 
 174968980
 
  PDF   STATS   ENSEMBL   UCSC 
 
  236
 
  8
 
 890
 
 gene;island
 
 0.24101
 
 0.02509
 
 Not queried
 
  3.7465137
 
 1.09e-03
 
 1.00e+00
  
 
  
 4
 
  81106240
 
  81106414
 
  PDF   STATS   ENSEMBL   UCSC 
 
  175
 
  6
 
 30412
 
 tss
 
 0.12008
 
 0.51214
 
 Not queried
 
  3.7417055
 
 2.16e-03
 
 1.00e+00
  
 
  
 4
 
    206112
 
    206339
 
  PDF   STATS   ENSEMBL   UCSC 
 
  228
 
  6
 
 30175
 
 tss
 
 0.69949
 
 0.23895
 
 Not queried
 
  3.7365998
 
 1.52e-02
 
 1.00e+00
  
 
  
 7
 
   4783964
 
   4784485
 
  PDF   STATS   ENSEMBL   UCSC 
 
  522
 
  6
 
 36782
 
 gene;island
 
 0.10170
 
 0.61541
 
 Not queried
 
  3.7358900
 
 8.66e-03
 
 1.00e+00
  
 
  
 2
 
 217560272
 
 217560403
 
  PDF   STATS   ENSEMBL   UCSC 
 
  132
 
  5
 
 23833
 
 tss;island
 
 0.46452
 
 0.04048
 
 Not queried
 
  3.7349363
 
 1.59e-02
 
 1.00e+00
  
 
  
 6
 
 147124984
 
 147124996
 
  PDF   STATS   ENSEMBL   UCSC 
 
   13
 
  4
 
 35214
 
 tss
 
 0.53219
 
 0.95440
 
 Not queried
 
  3.7338115
 
 2.00e-01
 
 1.00e+00
  
 
  
 7
 
 157361396
 
 157361759
 
  PDF   STATS   ENSEMBL   UCSC 
 
  364
 
  6
 
 37264
 
 gene;island
 
 0.33668
 
 0.88917
 
 Not queried
 
  3.7335828
 
 2.16e-03
 
 1.00e+00
  
 
  
 11
 
  61062837
 
  61063144
 
  PDF   STATS   ENSEMBL   UCSC 
 
  308
 
  9
 
 7309
 
 tss
 
 0.85860
 
 0.24345
 
 Not queried
 
  3.7328191
 
 4.94e-04
 
 1.00e+00
  
 
  
 11
 
  61062665
 
  61063144
 
  PDF   STATS   ENSEMBL   UCSC 
 
  480
 
 11
 
 8297
 
 island
 
 0.85120
 
 0.15562
 
 Not queried
 
  3.7328191
 
 3.94e-04
 
 1.00e+00
  
 
  
 X
 
  71458866
 
  71459274
 
  PDF   STATS   ENSEMBL   UCSC 
 
  409
 
  9
 
 41382
 
 tss
 
 0.03580
 
 0.42108
 
 Not queried
 
  3.7308343
 
 2.88e-04
 
 1.00e+00
  
 
  
 20
 
  37230326
 
  37230484
 
  PDF   STATS   ENSEMBL   UCSC 
 
  159
 
  5
 
 25698
 
 tss
 
 0.02786
 
 0.29874
 
 Not queried
 
  3.7256931
 
 7.94e-03
 
 1.00e+00
  
 
  
 11
 
 120008836
 
 120009135
 
  PDF   STATS   ENSEMBL   UCSC 
 
  300
 
  6
 
 8864
 
 island
 
 0.06849
 
 0.61994
 
 Not queried
 
  3.7256707
 
 6.49e-02
 
 1.00e+00
  
 
  
 16
 
  88700622
 
  88700916
 
  PDF   STATS   ENSEMBL   UCSC 
 
  295
 
  4
 
 16175
 
 island
 
 0.03293
 
 0.31081
 
 Not queried
 
  3.7230347
 
 2.86e-02
 
 1.00e+00
  
 
  
 4
 
 132896266
 
 132896736
 
  PDF   STATS   ENSEMBL   UCSC 
 
  471
 
  5
 
 31251
 
 island
 
 0.55047
 
 0.79998
 
 Not queried
 
  3.7194997
 
 7.94e-03
 
 1.00e+00
  
 
  
 10
 
 134043618
 
 134043897
 
  PDF   STATS   ENSEMBL   UCSC 
 
  280
 
  5
 
 4841
 
 gene;island
 
 0.34190
 
 0.89247
 
 Not queried
 
  3.7172244
 
 7.94e-03
 
 1.00e+00
  
 
  
 19
 
  38794554
 
  38794664
 
  PDF   STATS   ENSEMBL   UCSC 
 
  111
 
  4
 
 20713
 
 tss
 
 0.20516
 
 0.76552
 
 Not queried
 
  3.7167273
 
 2.86e-02
 
 1.00e+00
  
 
  
 16
 
  90143751
 
  90144006
 
  PDF   STATS   ENSEMBL   UCSC 
 
  256
 
  5
 
 16246
 
 island
 
 0.01767
 
 0.30471
 
 Not queried
 
  3.7155580
 
 7.94e-03
 
 1.00e+00
  
 
  
 11
 
   2884118
 
   2884460
 
  PDF   STATS   ENSEMBL   UCSC 
 
  343
 
  5
 
 7954
 
 island
 
 0.68877
 
 0.20121
 
 Not queried
 
  3.7151225
 
 7.94e-03
 
 1.00e+00
  
 
  
 21
 
  15645649
 
  15645988
 
  PDF   STATS   ENSEMBL   UCSC 
 
  340
 
  4
 
 26446
 
 tss
 
 0.63777
 
 0.94991
 
 Not queried
 
  3.7149505
 
 1.14e-01
 
 1.00e+00
  
 
  
 12
 
  52300911
 
  52301307
 
  PDF   STATS   ENSEMBL   UCSC 
 
  397
 
  5
 
 10406
 
 island
 
 0.12520
 
 0.65263
 
 Not queried
 
  3.7144624
 
 7.94e-03
 
 1.00e+00
  
 
  
 6
 
  30883762
 
  30884124
 
  PDF   STATS   ENSEMBL   UCSC 
 
  363
 
  4
 
 33776
 
 gene;island
 
 0.32100
 
 0.89693
 
 Not queried
 
  3.7118634
 
 2.86e-02
 
 1.00e+00
  
 
  
 7
 
  87105022
 
  87105416
 
  PDF   STATS   ENSEMBL   UCSC 
 
  395
 
  5
 
 37621
 
 tss
 
 0.47189
 
 0.04710
 
 Not queried
 
  3.7118104
 
 5.56e-02
 
 1.00e+00
  
 
  
 6
 
  39016262
 
  39016479
 
  PDF   STATS   ENSEMBL   UCSC 
 
  218
 
  5
 
 34888
 
 tss
 
 0.56546
 
 0.10291
 
 Not queried
 
  3.7108307
 
 7.94e-03
 
 1.00e+00
  
 
  
 11
 
  17740887
 
  17741070
 
  PDF   STATS   ENSEMBL   UCSC 
 
  184
 
  4
 
 7158
 
 tss
 
 0.05521
 
 0.38981
 
 Not queried
 
  3.7097758
 
 2.86e-02
 
 1.00e+00
  
 
  
 19
 
  44302666
 
  44303058
 
  PDF   STATS   ENSEMBL   UCSC 
 
  393
 
  4
 
 20043
 
 gene;island
 
 0.47159
 
 0.06715
 
 Not queried
 
  3.7094185
 
 5.71e-02
 
 1.00e+00
  
 
  
 2
 
  21022844
 
  21023042
 
  PDF   STATS   ENSEMBL   UCSC 
 
  199
 
  4
 
 23178
 
 tss
 
 0.92329
 
 0.48250
 
 Not queried
 
  3.7092652
 
 2.86e-02
 
 1.00e+00
  
 
  
 12
 
 114846849
 
 114847164
 
  PDF   STATS   ENSEMBL   UCSC 
 
  316
 
  4
 
 10009
 
 tss;island
 
 0.90333
 
 0.33862
 
 Not queried
 
  3.7066228
 
 2.86e-02
 
 1.00e+00
  
 
  
 2
 
  45169447
 
  45169681
 
  PDF   STATS   ENSEMBL   UCSC 
 
  235
 
  4
 
 22627
 
 gene;island
 
 0.96694
 
 0.63610
 
 Not queried
 
  3.7017140
 
 1.14e-01
 
 1.00e+00
  
 
  
 8
 
 101315424
 
 101315560
 
  PDF   STATS   ENSEMBL   UCSC 
 
  137
 
  4
 
 39316
 
 gene
 
 0.39488
 
 0.94388
 
 Not queried
 
  3.6977812
 
 5.71e-02
 
 1.00e+00
  
 
  
 3
 
  64253534
 
  64253818
 
  PDF   STATS   ENSEMBL   UCSC 
 
  285
 
  6
 
 29217
 
 island
 
 0.39519
 
 0.07980
 
 Not queried
 
  3.6946964
 
 2.16e-03
 
 1.00e+00
  
 
  
 17
 
  79503642
 
  79503877
 
  PDF   STATS   ENSEMBL   UCSC 
 
  236
 
  4
 
 16881
 
 gene;island
 
 0.55021
 
 0.92736
 
 Not queried
 
  3.6944987
 
 2.86e-02
 
 1.00e+00
  
 
  
 1
 
  22919873
 
  22920169
 
  PDF   STATS   ENSEMBL   UCSC 
 
  297
 
  4
 
 306
 
 gene;island
 
 0.22878
 
 0.77191
 
 Not queried
 
  3.6922128
 
 2.86e-02
 
 1.00e+00
  
 
  
 8
 
 120651190
 
 120651398
 
  PDF   STATS   ENSEMBL   UCSC 
 
  209
 
  4
 
 39822
 
 tss
 
 0.03557
 
 0.32717
 
 Not queried
 
  3.6918327
 
 2.86e-02
 
 1.00e+00
  
 
  
 4
 
 183721565
 
 183721778
 
  PDF   STATS   ENSEMBL   UCSC 
 
  214
 
  4
 
 30146
 
 gene;island
 
 0.59284
 
 0.92300
 
 Not queried
 
  3.6901255
 
 2.86e-02
 
 1.00e+00
  
 
  
 11
 
  58869663
 
  58870075
 
  PDF   STATS   ENSEMBL   UCSC 
 
  413
 
  5
 
 8279
 
 island
 
 0.03947
 
 0.42111
 
 Not queried
 
  3.6893972
 
 7.94e-03
 
 1.00e+00
  
 
  
 2
 
  29337946
 
  29338432
 
  PDF   STATS   ENSEMBL   UCSC 
 
  487
 
 10
 
 24160
 
 island
 
 0.54946
 
 0.06625
 
 Not queried
 
  3.6891608
 
 7.25e-04
 
 1.00e+00
  
 
  
 1
 
  32708234
 
  32708485
 
  PDF   STATS   ENSEMBL   UCSC 
 
  252
 
  5
 
 1453
 
 tss;island
 
 0.49770
 
 0.04453
 
 Not queried
 
  3.6888483
 
 1.59e-02
 
 1.00e+00
  
 
  
 X
 
 134124839
 
 134124960
 
  PDF   STATS   ENSEMBL   UCSC 
 
  122
 
  6
 
 41535
 
 tss
 
 0.09597
 
 0.61884
 
 Not queried
 
  3.6884402
 
 2.16e-03
 
 1.00e+00
  
 
  
 19
 
  54926277
 
  54926805
 
  PDF   STATS   ENSEMBL   UCSC 
 
  529
 
  7
 
 22370
 
 island
 
 0.03645
 
 0.28555
 
 Not queried
 
  3.6883351
 
 6.99e-03
 
 1.00e+00
  
 
  
 2
 
  26726190
 
  26726691
 
  PDF   STATS   ENSEMBL   UCSC 
 
  502
 
  5
 
 22577
 
 gene;island
 
 0.03542
 
 0.37131
 
 Not queried
 
  3.6847286
 
 1.59e-02
 
 1.00e+00
  
 
  
 8
 
   1960217
 
   1960466
 
  PDF   STATS   ENSEMBL   UCSC 
 
  250
 
  4
 
 39982
 
 island
 
 0.58200
 
 0.92783
 
 Not queried
 
  3.6837693
 
 2.86e-02
 
 1.00e+00
  
 
  
 22
 
  24890690
 
  24890833
 
  PDF   STATS   ENSEMBL   UCSC 
 
  144
 
  7
 
 26978
 
 tss;island
 
 0.48913
 
 0.92695
 
 Not queried
 
  3.6832889
 
 5.83e-04
 
 1.00e+00
  
 
  
 15
 
  76004985
 
  76005171
 
  PDF   STATS   ENSEMBL   UCSC 
 
  187
 
  4
 
 13403
 
 gene
 
 0.13489
 
 0.63512
 
 Not queried
 
  3.6823907
 
 2.86e-02
 
 1.00e+00
  
 
  
 X
 
   9960096
 
   9960578
 
  PDF   STATS   ENSEMBL   UCSC 
 
  483
 
  4
 
 41632
 
 island
 
 0.62027
 
 0.95633
 
 Not queried
 
  3.6820802
 
 2.86e-02
 
 1.00e+00
  
 
  
 21
 
  22369802
 
  22370444
 
  PDF   STATS   ENSEMBL   UCSC 
 
  643
 
  6
 
 26454
 
 tss;island
 
 0.04654
 
 0.52412
 
 Not queried
 
  3.6819437
 
 4.11e-02
 
 1.00e+00
  
 
  
 12
 
 103311462
 
 103311761
 
  PDF   STATS   ENSEMBL   UCSC 
 
  300
 
  6
 
 9926
 
 tss
 
 0.02692
 
 0.31184
 
 Not queried
 
  3.6814566
 
 4.11e-02
 
 1.00e+00
  
 
  
 19
 
  31847893
 
  31848310
 
  PDF   STATS   ENSEMBL   UCSC 
 
  418
 
  6
 
 21828
 
 island
 
 0.31490
 
 0.03605
 
 Not queried
 
  3.6797134
 
 8.66e-03
 
 1.00e+00
  
 
  
 17
 
  46019006
 
  46019184
 
  PDF   STATS   ENSEMBL   UCSC 
 
  179
 
  5
 
 16613
 
 gene
 
 0.44685
 
 0.05946
 
 Not queried
 
  3.6756119
 
 7.94e-03
 
 1.00e+00
  
 
  
 4
 
  74719046
 
  74719289
 
  PDF   STATS   ENSEMBL   UCSC 
 
  244
 
  4
 
 29977
 
 gene
 
 0.21612
 
 0.77717
 
 Not queried
 
  3.6751683
 
 2.86e-02
 
 1.00e+00
  
 
  
 17
 
  21743878
 
  21744126
 
  PDF   STATS   ENSEMBL   UCSC 
 
  249
 
  4
 
 18095
 
 island
 
 0.01761
 
 0.18529
 
 Not queried
 
  3.6748278
 
 2.86e-02
 
 1.00e+00
  
 
  
 21
 
  42797847
 
  42798386
 
  PDF   STATS   ENSEMBL   UCSC 
 
  540
 
  6
 
 26389
 
 gene;island
 
 0.14266
 
 0.66634
 
 Not queried
 
  3.6742887
 
 4.33e-03
 
 1.00e+00
  
 
  
 X
 
  46404957
 
  46405125
 
  PDF   STATS   ENSEMBL   UCSC 
 
  169
 
  7
 
 40971
 
 gene
 
 0.08907
 
 0.57672
 
 Not queried
 
  3.6737343
 
 5.83e-04
 
 1.00e+00
  
 
  
 13
 
 112728799
 
 112728964
 
  PDF   STATS   ENSEMBL   UCSC 
 
  166
 
  4
 
 11884
 
 island
 
 0.54233
 
 0.21208
 
 Not queried
 
  3.6734312
 
 5.71e-02
 
 1.00e+00
  
 
  
 2
 
 161126295
 
 161126857
 
  PDF   STATS   ENSEMBL   UCSC 
 
  563
 
  7
 
 24776
 
 island
 
 0.02470
 
 0.30002
 
 Not queried
 
  3.6718768
 
 5.83e-04
 
 1.00e+00
  
 
  
 17
 
    907643
 
    907886
 
  PDF   STATS   ENSEMBL   UCSC 
 
  244
 
  4
 
 16266
 
 gene;island
 
 0.33173
 
 0.79533
 
 Not queried
 
  3.6663127
 
 2.86e-02
 
 1.00e+00
  
 
  
 2
 
  47499521
 
  47499812
 
  PDF   STATS   ENSEMBL   UCSC 
 
  292
 
  4
 
 24243
 
 island
 
 0.02413
 
 0.28505
 
 Not queried
 
  3.6655316
 
 2.86e-02
 
 1.00e+00
  
 
  
 2
 
 177053274
 
 177053297
 
  PDF   STATS   ENSEMBL   UCSC 
 
   24
 
  6
 
 23706
 
 tss
 
 0.02740
 
 0.25482
 
 Not queried
 
  3.6642071
 
 2.16e-03
 
 1.00e+00
  
 
  
 X
 
 152938698
 
 152938907
 
  PDF   STATS   ENSEMBL   UCSC 
 
  210
 
  5
 
 41142
 
 gene;island
 
 0.33083
 
 0.84460
 
 Not queried
 
  3.6633463
 
 7.94e-03
 
 1.00e+00
  
 
  
 1
 
 233462575
 
 233462803
 
  PDF   STATS   ENSEMBL   UCSC 
 
  229
 
  5
 
 2322
 
 tss;island
 
 0.31616
 
 0.06411
 
 Not queried
 
  3.6631763
 
 5.56e-02
 
 1.00e+00
  
 
  
 11
 
 111170578
 
 111170648
 
  PDF   STATS   ENSEMBL   UCSC 
 
   71
 
  4
 
 7661
 
 tss
 
 0.49594
 
 0.12645
 
 Not queried
 
  3.6624006
 
 2.86e-02
 
 1.00e+00
  
 
  
 8
 
  95653874
 
  95654179
 
  PDF   STATS   ENSEMBL   UCSC 
 
  306
 
  4
 
 39300
 
 gene;island
 
 0.03565
 
 0.32541
 
 Not queried
 
  3.6614433
 
 2.86e-02
 
 1.00e+00
  
 
  
 17
 
  74075092
 
  74075336
 
  PDF   STATS   ENSEMBL   UCSC 
 
  245
 
  5
 
 18737
 
 island
 
 0.25501
 
 0.83705
 
 Not queried
 
  3.6599122
 
 7.94e-03
 
 1.00e+00
  
 
  
 17
 
  37123669
 
  37123949
 
  PDF   STATS   ENSEMBL   UCSC 
 
  281
 
  8
 
 17269
 
 tss
 
 0.29037
 
 0.78032
 
 Not queried
 
  3.6591572
 
 3.11e-04
 
 1.00e+00
  
 
  
 17
 
  48609923
 
  48610255
 
  PDF   STATS   ENSEMBL   UCSC 
 
  333
 
  5
 
 18520
 
 island
 
 0.03998
 
 0.38219
 
 Not queried
 
  3.6585490
 
 7.94e-03
 
 1.00e+00
  
 
  
 1
 
   2918373
 
   2918752
 
  PDF   STATS   ENSEMBL   UCSC 
 
  380
 
  5
 
 2520
 
 island
 
 0.21649
 
 0.82686
 
 Not queried
 
  3.6576847
 
 7.94e-03
 
 1.00e+00
  
 
  
 17
 
  48071685
 
  48071988
 
  PDF   STATS   ENSEMBL   UCSC 
 
  304
 
  4
 
 16643
 
 gene;island
 
 0.02420
 
 0.20344
 
 Not queried
 
  3.6552018
 
 2.86e-02
 
 1.00e+00
  
 
  
 8
 
  22735111
 
  22735478
 
  PDF   STATS   ENSEMBL   UCSC 
 
  368
 
  5
 
 39174
 
 gene
 
 0.17750
 
 0.67686
 
 Not queried
 
  3.6527386
 
 3.17e-02
 
 1.00e+00
  
 
  
 15
 
  43531947
 
  43532243
 
  PDF   STATS   ENSEMBL   UCSC 
 
  297
 
  4
 
 13285
 
 gene
 
 0.40745
 
 0.90303
 
 Not queried
 
  3.6522653
 
 2.86e-02
 
 1.00e+00
  
 
  
 17
 
  64188403
 
  64188644
 
  PDF   STATS   ENSEMBL   UCSC 
 
  242
 
  4
 
 17562
 
 tss;island
 
 0.41098
 
 0.05268
 
 Not queried
 
  3.6512983
 
 3.43e-01
 
 1.00e+00
  
 
  
 6
 
  39281421
 
  39281885
 
  PDF   STATS   ENSEMBL   UCSC 
 
  465
 
  5
 
 34260
 
 gene;island
 
 0.03741
 
 0.15877
 
 Not queried
 
  3.6462532
 
 7.94e-03
 
 1.00e+00
  
 
  
 2
 
 175499113
 
 175499377
 
  PDF   STATS   ENSEMBL   UCSC 
 
  265
 
  4
 
 22893
 
 gene
 
 0.35776
 
 0.87531
 
 Not queried
 
  3.6449687
 
 5.71e-02
 
 1.00e+00
  
 
  
 11
 
  66103823
 
  66104174
 
  PDF   STATS   ENSEMBL   UCSC 
 
  352
 
  7
 
 8487
 
 island
 
 0.20516
 
 0.76316
 
 Not queried
 
  3.6420611
 
 1.75e-02
 
 1.00e+00
  
 
  
 X
 
 153707710
 
 153707810
 
  PDF   STATS   ENSEMBL   UCSC 
 
  101
 
  4
 
 41601
 
 tss
 
 0.10889
 
 0.58485
 
 Not queried
 
  3.6415718
 
 2.86e-02
 
 1.00e+00
  
 
  
 3
 
 129407530
 
 129407548
 
  PDF   STATS   ENSEMBL   UCSC 
 
   19
 
  4
 
 28545
 
 tss
 
 0.41856
 
 0.04549
 
 Not queried
 
  3.6390903
 
 2.86e-02
 
 1.00e+00
  
 
  
 1
 
  55267006
 
  55267293
 
  PDF   STATS   ENSEMBL   UCSC 
 
  288
 
  7
 
 1645
 
 tss
 
 0.28000
 
 0.74490
 
 Not queried
 
  3.6381408
 
 5.83e-04
 
 1.00e+00
  
 
  
 20
 
  30639816
 
  30640041
 
  PDF   STATS   ENSEMBL   UCSC 
 
  226
 
  7
 
 25632
 
 tss
 
 0.34397
 
 0.03529
 
 Not queried
 
  3.6366446
 
 2.33e-03
 
 1.00e+00
  
 
  
 12
 
 119772354
 
 119772577
 
  PDF   STATS   ENSEMBL   UCSC 
 
  224
 
  5
 
 10846
 
 island
 
 0.39044
 
 0.04899
 
 Not queried
 
  3.6363358
 
 9.52e-02
 
 1.00e+00
  
 
  
 15
 
  96960214
 
  96960725
 
  PDF   STATS   ENSEMBL   UCSC 
 
  512
 
  5
 
 14463
 
 island
 
 0.01679
 
 0.23995
 
 Not queried
 
  3.6344860
 
 7.94e-03
 
 1.00e+00
  
 
  
 16
 
   2140326
 
   2140438
 
  PDF   STATS   ENSEMBL   UCSC 
 
  113
 
  4
 
 14571
 
 gene;tss;island
 
 0.67193
 
 0.94646
 
 Not queried
 
  3.6328169
 
 5.71e-02
 
 1.00e+00
  
 
  
 21
 
  19616939
 
  19617145
 
  PDF   STATS   ENSEMBL   UCSC 
 
  207
 
  4
 
 26453
 
 tss;island
 
 0.09572
 
 0.51830
 
 Not queried
 
  3.6287007
 
 3.43e-01
 
 1.00e+00
  
 
  
 18
 
  10588980
 
  10589360
 
  PDF   STATS   ENSEMBL   UCSC 
 
  381
 
  5
 
 19358
 
 island
 
 0.69949
 
 0.92002
 
 Not queried
 
  3.6269642
 
 7.94e-03
 
 1.00e+00
  
 
  
 15
 
  52404977
 
  52405162
 
  PDF   STATS   ENSEMBL   UCSC 
 
  186
 
  7
 
 13649
 
 tss
 
 0.16920
 
 0.89112
 
 Not queried
 
  3.6239977
 
 3.79e-02
 
 1.00e+00
  
 
  
 X
 
 153715055
 
 153715609
 
  PDF   STATS   ENSEMBL   UCSC 
 
  555
 
  8
 
 41602
 
 tss;island
 
 0.11190
 
 0.63818
 
 Not queried
 
  3.6205407
 
 1.04e-02
 
 1.00e+00
  
 
  
 12
 
  66582582
 
  66582805
 
  PDF   STATS   ENSEMBL   UCSC 
 
  224
 
  4
 
 9823
 
 tss;island
 
 0.28219
 
 0.84782
 
 Not queried
 
  3.6198271
 
 2.86e-02
 
 1.00e+00
  
 
  
 16
 
  55542668
 
  55542751
 
  PDF   STATS   ENSEMBL   UCSC 
 
   84
 
  4
 
 15209
 
 tss
 
 0.71354
 
 0.19947
 
 Not queried
 
  3.6161653
 
 2.00e-01
 
 1.00e+00
  
 
  
 17
 
  46692347
 
  46692592
 
  PDF   STATS   ENSEMBL   UCSC 
 
  246
 
  7
 
 17434
 
 tss
 
 0.03055
 
 0.42670
 
 Not queried
 
  3.6150604
 
 4.08e-03
 
 1.00e+00
  
 
  
 20
 
  19915716
 
  19915874
 
  PDF   STATS   ENSEMBL   UCSC 
 
  159
 
  6
 
 25331
 
 gene
 
 0.10944
 
 0.66903
 
 Not queried
 
  3.6131424
 
 2.16e-03
 
 1.00e+00
  
 
  
 17
 
   1012288
 
   1012579
 
  PDF   STATS   ENSEMBL   UCSC 
 
  292
 
  4
 
 16272
 
 gene;island
 
 0.01922
 
 0.21264
 
 Not queried
 
  3.6118488
 
 2.86e-02
 
 1.00e+00
  
 
  
 3
 
 183948058
 
 183948313
 
  PDF   STATS   ENSEMBL   UCSC 
 
  256
 
  5
 
 28711
 
 tss
 
 0.02035
 
 0.19749
 
 Not queried
 
  3.6114573
 
 3.17e-02
 
 1.00e+00
  
 
  
 2
 
 121199631
 
 121199920
 
  PDF   STATS   ENSEMBL   UCSC 
 
  290
 
  4
 
 24628
 
 island
 
 0.05851
 
 0.40190
 
 Not queried
 
  3.6098865
 
 2.86e-02
 
 1.00e+00
  
 
  
 20
 
  25566178
 
  25566563
 
  PDF   STATS   ENSEMBL   UCSC 
 
  386
 
  7
 
 25613
 
 tss;island
 
 0.23822
 
 0.02498
 
 Not queried
 
  3.6093531
 
 2.09e-01
 
 1.00e+00
  
 
  
 16
 
  55866757
 
  55867072
 
  PDF   STATS   ENSEMBL   UCSC 
 
  316
 
  5
 
 14724
 
 gene;island
 
 0.49457
 
 0.04230
 
 Not queried
 
  3.6077584
 
 7.94e-03
 
 1.00e+00
  
 
  
 10
 
  22623821
 
  22624374
 
  PDF   STATS   ENSEMBL   UCSC 
 
  554
 
  5
 
 5582
 
 island
 
 0.90058
 
 0.40594
 
 Not queried
 
  3.6067379
 
 7.94e-03
 
 1.00e+00
  
 
  
 8
 
 116660311
 
 116660697
 
  PDF   STATS   ENSEMBL   UCSC 
 
  387
 
  4
 
 39340
 
 gene;island
 
 0.08027
 
 0.48101
 
 Not queried
 
  3.6063102
 
 5.71e-02
 
 1.00e+00
  
 
  
 3
 
 126113327
 
 126113743
 
  PDF   STATS   ENSEMBL   UCSC 
 
  417
 
 10
 
 28515
 
 tss
 
 0.29857
 
 0.87328
 
 Not queried
 
  3.6053819
 
 8.93e-03
 
 1.00e+00
  
 
  
 17
 
  79881468
 
  79881543
 
  PDF   STATS   ENSEMBL   UCSC 
 
   76
 
  5
 
 16896
 
 gene;tss;island
 
 0.69645
 
 0.95331
 
 Not queried
 
  3.6052885
 
 7.94e-03
 
 1.00e+00
  
 
  
 7
 
    751830
 
    753007
 
  PDF   STATS   ENSEMBL   UCSC 
 
 1178
 
 16
 
 36654
 
 gene;island
 
 0.49666
 
 0.94284
 
 Not queried
 
  3.6040502
 
 1.08e-02
 
 1.00e+00
  
 
  
 7
 
 143106001
 
 143106298
 
  PDF   STATS   ENSEMBL   UCSC 
 
  298
 
  7
 
 37890
 
 tss
 
 0.02210
 
 0.25330
 
 Not queried
 
  3.6038093
 
 5.83e-04
 
 1.00e+00
  
 
  
 22
 
  32600722
 
  32601274
 
  PDF   STATS   ENSEMBL   UCSC 
 
  553
 
  4
 
 27047
 
 tss
 
 0.04825
 
 0.36422
 
 Not queried
 
  3.6029363
 
 2.86e-02
 
 1.00e+00
  
 
  
 X
 
 110513791
 
 110513957
 
  PDF   STATS   ENSEMBL   UCSC 
 
  167
 
  4
 
 41474
 
 tss
 
 0.10728
 
 0.69269
 
 Not queried
 
  3.6022946
 
 2.86e-02
 
 1.00e+00
  
 
  
 7
 
  75157345
 
  75157765
 
  PDF   STATS   ENSEMBL   UCSC 
 
  421
 
  9
 
 38521
 
 island
 
 0.24227
 
 0.02228
 
 Not queried
 
  3.6015338
 
 1.85e-03
 
 1.00e+00
  
 
  
 22
 
  42078217
 
  42078723
 
  PDF   STATS   ENSEMBL   UCSC 
 
  507
 
  7
 
 26842
 
 gene;island
 
 0.01263
 
 0.22660
 
 Not queried
 
  3.6011815
 
 1.75e-02
 
 1.00e+00
  
 
  
 11
 
  73020351
 
  73020729
 
  PDF   STATS   ENSEMBL   UCSC 
 
  379
 
  5
 
 6803
 
 gene;island
 
 0.04201
 
 0.34721
 
 Not queried
 
  3.6004604
 
 7.94e-03
 
 1.00e+00
  
 
  
 7
 
  48019205
 
  48019713
 
  PDF   STATS   ENSEMBL   UCSC 
 
  509
 
  5
 
 38417
 
 island
 
 0.45393
 
 0.94887
 
 Not queried
 
  3.5999516
 
 1.51e-01
 
 1.00e+00
  
 
  
 19
 
  40324746
 
  40325100
 
  PDF   STATS   ENSEMBL   UCSC 
 
  355
 
 10
 
 21972
 
 island
 
 0.04359
 
 0.39772
 
 Not queried
 
  3.5978456
 
 1.05e-03
 
 1.00e+00
  
 
  
 X
 
  54833733
 
  54833994
 
  PDF   STATS   ENSEMBL   UCSC 
 
  262
 
  4
 
 41336
 
 tss;island
 
 0.64376
 
 0.07757
 
 Not queried
 
  3.5975412
 
 1.14e-01
 
 1.00e+00
  
 
  
 4
 
  46995474
 
  46995821
 
  PDF   STATS   ENSEMBL   UCSC 
 
  348
 
  7
 
 30969
 
 island
 
 0.03167
 
 0.47590
 
 Not queried
 
  3.5967286
 
 5.83e-04
 
 1.00e+00
  
 
  
 2
 
  26395359
 
  26395556
 
  PDF   STATS   ENSEMBL   UCSC 
 
  198
 
  4
 
 23199
 
 tss;island
 
 0.27900
 
 0.05456
 
 Not queried
 
  3.5964907
 
 2.86e-02
 
 1.00e+00
  
 
  
 19
 
  46318439
 
  46318918
 
  PDF   STATS   ENSEMBL   UCSC 
 
  480
 
  6
 
 22123
 
 island
 
 0.38145
 
 0.05483
 
 Not queried
 
  3.5958102
 
 2.16e-03
 
 1.00e+00
  
 
  
 6
 
  30882994
 
  30883203
 
  PDF   STATS   ENSEMBL   UCSC 
 
  210
 
  5
 
 33775
 
 gene;island
 
 0.49211
 
 0.93110
 
 Not queried
 
  3.5953274
 
 9.52e-02
 
 1.00e+00
  
 
  
 3
 
  72704324
 
  72704701
 
  PDF   STATS   ENSEMBL   UCSC 
 
  378
 
  5
 
 29238
 
 island
 
 0.43041
 
 0.90118
 
 Not queried
 
  3.5931201
 
 7.94e-03
 
 1.00e+00
  
 
  
 15
 
  37110422
 
  37110854
 
  PDF   STATS   ENSEMBL   UCSC 
 
  433
 
  7
 
 13980
 
 island
 
 0.49537
 
 0.95698
 
 Not queried
 
  3.5930183
 
 2.33e-03
 
 1.00e+00
  
 
  
 7
 
 114561804
 
 114562063
 
  PDF   STATS   ENSEMBL   UCSC 
 
  260
 
  5
 
 37782
 
 tss;island
 
 0.68533
 
 0.06011
 
 Not queried
 
  3.5903145
 
 5.56e-02
 
 1.00e+00
  
 
  
 10
 
  46168377
 
  46168597
 
  PDF   STATS   ENSEMBL   UCSC 
 
  221
 
  6
 
 5044
 
 tss
 
 0.01840
 
 0.16794
 
 Not queried
 
  3.5893663
 
 2.16e-03
 
 1.00e+00
  
 
  
 4
 
  81106240
 
  81106463
 
  PDF   STATS   ENSEMBL   UCSC 
 
  224
 
  7
 
 31079
 
 island
 
 0.10689
 
 0.53127
 
 Not queried
 
  3.5856617
 
 5.83e-04
 
 1.00e+00
  
 
  
 X
 
 101967017
 
 101967307
 
  PDF   STATS   ENSEMBL   UCSC 
 
  291
 
  7
 
 41433
 
 tss;island
 
 0.35719
 
 0.03342
 
 Not queried
 
  3.5811793
 
 3.79e-02
 
 1.00e+00
  
 
  
 1
 
  44398868
 
  44399069
 
  PDF   STATS   ENSEMBL   UCSC 
 
  202
 
  4
 
 3166
 
 island
 
 0.22333
 
 0.83567
 
 Not queried
 
  3.5805898
 
 5.71e-02
 
 1.00e+00
  
 
  
 7
 
  27179161
 
  27179432
 
  PDF   STATS   ENSEMBL   UCSC 
 
  272
 
  4
 
 38282
 
 island
 
 0.41837
 
 0.06203
 
 Not queried
 
  3.5804499
 
 2.86e-02
 
 1.00e+00
  
 
  
 10
 
  61468548
 
  61468757
 
  PDF   STATS   ENSEMBL   UCSC 
 
  210
 
  4
 
 4583
 
 gene;island
 
 0.25755
 
 0.02851
 
 Not queried
 
  3.5803491
 
 2.86e-02
 
 1.00e+00
  
 
  
 6
 
  26273029
 
  26273199
 
  PDF   STATS   ENSEMBL   UCSC 
 
  171
 
  6
 
 34640
 
 tss
 
 0.65967
 
 0.93725
 
 Not queried
 
  3.5793638
 
 2.16e-03
 
 1.00e+00
  
 
  
 3
 
  52479069
 
  52479108
 
  PDF   STATS   ENSEMBL   UCSC 
 
   40
 
  4
 
 28338
 
 tss
 
 0.05321
 
 0.39636
 
 Not queried
 
  3.5778855
 
 2.86e-02
 
 1.00e+00
  
 
  
 X
 
  63425713
 
  63425930
 
  PDF   STATS   ENSEMBL   UCSC 
 
  218
 
  7
 
 41349
 
 tss
 
 0.26076
 
 0.02480
 
 Not queried
 
  3.5767487
 
 5.83e-04
 
 1.00e+00
  
 
  
 12
 
 119772354
 
 119772421
 
  PDF   STATS   ENSEMBL   UCSC 
 
   68
 
  4
 
 10025
 
 tss
 
 0.35924
 
 0.03701
 
 Not queried
 
  3.5763220
 
 2.00e-01
 
 1.00e+00
  
 
  
 7
 
 150020818
 
 150020945
 
  PDF   STATS   ENSEMBL   UCSC 
 
  128
 
  6
 
 37911
 
 tss
 
 0.42384
 
 0.06068
 
 Not queried
 
  3.5761483
 
 8.66e-03
 
 1.00e+00
  
 
  
 4
 
 122853693
 
 122853965
 
  PDF   STATS   ENSEMBL   UCSC 
 
  273
 
  4
 
 30070
 
 gene;island
 
 0.32779
 
 0.87871
 
 Not queried
 
  3.5753581
 
 2.86e-02
 
 1.00e+00
  
 
  
 X
 
  53468600
 
  53468978
 
  PDF   STATS   ENSEMBL   UCSC 
 
  379
 
  4
 
 41810
 
 island
 
 0.04589
 
 0.38209
 
 Not queried
 
  3.5746722
 
 2.86e-02
 
 1.00e+00
  
 
  
 10
 
 134791323
 
 134791660
 
  PDF   STATS   ENSEMBL   UCSC 
 
  338
 
  5
 
 6304
 
 island
 
 0.45129
 
 0.90740
 
 Not queried
 
  3.5746287
 
 7.94e-03
 
 1.00e+00
  
 
  
 11
 
   6341842
 
   6341908
 
  PDF   STATS   ENSEMBL   UCSC 
 
   67
 
  7
 
 7086
 
 tss
 
 0.29122
 
 0.03008
 
 Not queried
 
  3.5735855
 
 5.83e-04
 
 1.00e+00
  
 
  
 1
 
 228291486
 
 228291705
 
  PDF   STATS   ENSEMBL   UCSC 
 
  220
 
  5
 
 2293
 
 tss;island
 
 0.52664
 
 0.06649
 
 Not queried
 
  3.5713078
 
 7.94e-03
 
 1.00e+00
  
 
  
 X
 
 152677200
 
 152677554
 
  PDF   STATS   ENSEMBL   UCSC 
 
  355
 
  4
 
 42108
 
 island
 
 0.49146
 
 0.89301
 
 Not queried
 
  3.5713043
 
 2.86e-02
 
 1.00e+00
  
 
  
 11
 
  17740887
 
  17741243
 
  PDF   STATS   ENSEMBL   UCSC 
 
  357
 
  5
 
 8082
 
 island
 
 0.06168
 
 0.40117
 
 Not queried
 
  3.5709823
 
 9.52e-02
 
 1.00e+00
  
 
  
 5
 
  72594461
 
  72594851
 
  PDF   STATS   ENSEMBL   UCSC 
 
  391
 
  4
 
 32805
 
 island
 
 0.53933
 
 0.09249
 
 Not queried
 
  3.5708670
 
 2.86e-02
 
 1.00e+00
  
 
  
 18
 
  74499372
 
  74499584
 
  PDF   STATS   ENSEMBL   UCSC 
 
  213
 
  5
 
 19546
 
 island
 
 0.55125
 
 0.92224
 
 Not queried
 
  3.5695002
 
 7.94e-03
 
 1.00e+00
  
 
  
 4
 
   6202379
 
   6202609
 
  PDF   STATS   ENSEMBL   UCSC 
 
  231
 
  6
 
 30231
 
 tss;island
 
 0.25372
 
 0.02415
 
 Not queried
 
  3.5673091
 
 1.80e-01
 
 1.00e+00
  
 
  
 5
 
 140561893
 
 140562236
 
  PDF   STATS   ENSEMBL   UCSC 
 
  344
 
  6
 
 31755
 
 gene;island
 
 0.02879
 
 0.22533
 
 Not queried
 
  3.5653161
 
 9.31e-02
 
 1.00e+00
  
 
  
 14
 
  91720227
 
  91720696
 
  PDF   STATS   ENSEMBL   UCSC 
 
  470
 
  5
 
 12531
 
 tss
 
 0.53044
 
 0.11793
 
 Not queried
 
  3.5651500
 
 9.52e-02
 
 1.00e+00
  
 
  
 2
 
 152830505
 
 152830665
 
  PDF   STATS   ENSEMBL   UCSC 
 
  161
 
  5
 
 22860
 
 gene;tss
 
 0.30301
 
 0.06135
 
 Not queried
 
  3.5646918
 
 7.94e-03
 
 1.00e+00
  
 
  
 11
 
  67183078
 
  67183616
 
  PDF   STATS   ENSEMBL   UCSC 
 
  539
 
  8
 
 8523
 
 island
 
 0.04482
 
 0.35674
 
 Not queried
 
  3.5644287
 
 3.79e-02
 
 1.00e+00
  
 
  
 16
 
   2765620
 
   2765900
 
  PDF   STATS   ENSEMBL   UCSC 
 
  281
 
  5
 
 14581
 
 gene;island
 
 0.04634
 
 0.41905
 
 Not queried
 
  3.5643138
 
 3.17e-02
 
 1.00e+00
  
 
  
 5
 
 140729429
 
 140730092
 
  PDF   STATS   ENSEMBL   UCSC 
 
  664
 
  9
 
 31762
 
 gene;island
 
 0.01808
 
 0.30962
 
 Not queried
 
  3.5627331
 
 1.85e-03
 
 1.00e+00
  
 
  
 2
 
  19558375
 
  19558569
 
  PDF   STATS   ENSEMBL   UCSC 
 
  195
 
  4
 
 23167
 
 tss
 
 0.28931
 
 0.04295
 
 Not queried
 
  3.5623150
 
 2.86e-02
 
 1.00e+00
  
 
  
 7
 
  76026853
 
  76027101
 
  PDF   STATS   ENSEMBL   UCSC 
 
  249
 
  4
 
 36993
 
 gene;island
 
 0.04326
 
 0.42570
 
 Not queried
 
  3.5590596
 
 2.86e-02
 
 1.00e+00
  
 
  
 1
 
 106623429
 
 106623967
 
  PDF   STATS   ENSEMBL   UCSC 
 
  539
 
  6
 
 3528
 
 island
 
 0.21946
 
 0.73596
 
 Not queried
 
  3.5587515
 
 4.11e-02
 
 1.00e+00
  
 
  
 5
 
 149792330
 
 149792515
 
  PDF   STATS   ENSEMBL   UCSC 
 
  186
 
  6
 
 32370
 
 tss
 
 0.56700
 
 0.11597
 
 Not queried
 
  3.5552792
 
 2.16e-03
 
 1.00e+00
  
 
  
 6
 
  30614081
 
  30614422
 
  PDF   STATS   ENSEMBL   UCSC 
 
  342
 
  8
 
 34713
 
 tss;island
 
 0.11650
 
 0.73252
 
 Not queried
 
  3.5511855
 
 1.86e-03
 
 1.00e+00
  
 
  
 11
 
 118123012
 
 118123243
 
  PDF   STATS   ENSEMBL   UCSC 
 
  232
 
  7
 
 7706
 
 tss
 
 0.14061
 
 0.77869
 
 Not queried
 
  3.5501059
 
 6.99e-03
 
 1.00e+00
  
 
  
 X
 
  84634308
 
  84634593
 
  PDF   STATS   ENSEMBL   UCSC 
 
  286
 
  5
 
 41044
 
 gene
 
 0.43370
 
 0.84161
 
 Not queried
 
  3.5499167
 
 7.94e-03
 
 1.00e+00
  
 
  
 4
 
 118006747
 
 118006832
 
  PDF   STATS   ENSEMBL   UCSC 
 
   86
 
  6
 
 30511
 
 tss
 
 0.05099
 
 0.33893
 
 Not queried
 
  3.5480213
 
 2.16e-03
 
 1.00e+00
  
 
  
 19
 
   2281919
 
   2282207
 
  PDF   STATS   ENSEMBL   UCSC 
 
  289
 
  5
 
 21241
 
 island
 
 0.31482
 
 0.07555
 
 Not queried
 
  3.5477549
 
 1.59e-02
 
 1.00e+00
  
 
  
 3
 
 129694392
 
 129694683
 
  PDF   STATS   ENSEMBL   UCSC 
 
  292
 
  4
 
 27903
 
 gene;island
 
 0.73879
 
 0.20467
 
 Not queried
 
  3.5476845
 
 2.86e-02
 
 1.00e+00
  
 
  
 5
 
  26669610
 
  26669882
 
  PDF   STATS   ENSEMBL   UCSC 
 
  273
 
  4
 
 32650
 
 island
 
 0.17999
 
 0.70779
 
 Not queried
 
  3.5470059
 
 2.86e-02
 
 1.00e+00
  
 
  
 4
 
  74718978
 
  74719289
 
  PDF   STATS   ENSEMBL   UCSC 
 
  312
 
  5
 
 31044
 
 island
 
 0.18770
 
 0.76356
 
 Not queried
 
  3.5454716
 
 7.94e-03
 
 1.00e+00
  
 
  
 4
 
 123747447
 
 123747750
 
  PDF   STATS   ENSEMBL   UCSC 
 
  304
 
  7
 
 30533
 
 tss;island
 
 0.27870
 
 0.01799
 
 Not queried
 
  3.5446075
 
 4.08e-03
 
 1.00e+00
  
 
  
 2
 
 177053345
 
 177053568
 
  PDF   STATS   ENSEMBL   UCSC 
 
  224
 
  4
 
 22903
 
 gene
 
 0.03280
 
 0.28477
 
 Not queried
 
  3.5412934
 
 2.86e-02
 
 1.00e+00
  
 
  
 X
 
  45060311
 
  45060544
 
  PDF   STATS   ENSEMBL   UCSC 
 
  234
 
  4
 
 41269
 
 tss
 
 0.38219
 
 0.84176
 
 Not queried
 
  3.5396364
 
 5.71e-02
 
 1.00e+00
  
 
  
 11
 
  46940434
 
  46940613
 
  PDF   STATS   ENSEMBL   UCSC 
 
  180
 
  4
 
 7261
 
 tss;island
 
 0.14475
 
 0.02051
 
 Not queried
 
  3.5365999
 
 1.14e-01
 
 1.00e+00
  
 
  
 10
 
 104211296
 
 104211488
 
  PDF   STATS   ENSEMBL   UCSC 
 
  193
 
  6
 
 6071
 
 island
 
 0.07236
 
 0.45042
 
 Not queried
 
  3.5362334
 
 2.16e-03
 
 1.00e+00
  
 
  
 12
 
  56546403
 
  56546720
 
  PDF   STATS   ENSEMBL   UCSC 
 
  318
 
  4
 
 9149
 
 gene
 
 0.10807
 
 0.01612
 
 Not queried
 
  3.5332145
 
 1.14e-01
 
 1.00e+00
  
 
  
 16
 
    215410
 
    215960
 
  PDF   STATS   ENSEMBL   UCSC 
 
  551
 
  7
 
 14931
 
 tss
 
 0.03073
 
 0.26423
 
 Not queried
 
  3.5318249
 
 5.83e-04
 
 1.00e+00
  
 
  
 3
 
  42577680
 
  42577884
 
  PDF   STATS   ENSEMBL   UCSC 
 
  205
 
  5
 
 27690
 
 gene;island
 
 0.25523
 
 0.79853
 
 Not queried
 
  3.5318045
 
 7.94e-03
 
 1.00e+00
  
 
  
 17
 
   8702369
 
   8702896
 
  PDF   STATS   ENSEMBL   UCSC 
 
  528
 
  9
 
 17975
 
 island
 
 0.15990
 
 0.68355
 
 Not queried
 
  3.5303152
 
 7.82e-04
 
 1.00e+00
  
 
  
 18
 
   3011810
 
   3012017
 
  PDF   STATS   ENSEMBL   UCSC 
 
  208
 
  4
 
 19323
 
 island
 
 0.11892
 
 0.59414
 
 Not queried
 
  3.5267459
 
 2.00e-01
 
 1.00e+00
  
 
  
 19
 
  50015511
 
  50015532
 
  PDF   STATS   ENSEMBL   UCSC 
 
   22
 
  4
 
 20917
 
 tss;island
 
 0.25738
 
 0.71777
 
 Not queried
 
  3.5209562
 
 2.86e-02
 
 1.00e+00
  
 
  
 13
 
 100310162
 
 100310405
 
  PDF   STATS   ENSEMBL   UCSC 
 
  244
 
  4
 
 11177
 
 gene;island
 
 0.34840
 
 0.04068
 
 Not queried
 
  3.5173728
 
 2.86e-02
 
 1.00e+00
  
 
  
 14
 
 103655242
 
 103655773
 
  PDF   STATS   ENSEMBL   UCSC 
 
  532
 
  5
 
 13154
 
 island
 
 0.43128
 
 0.92869
 
 Not queried
 
  3.5172392
 
 7.94e-03
 
 1.00e+00
  
 
  
 12
 
 132990805
 
 132991277
 
  PDF   STATS   ENSEMBL   UCSC 
 
  473
 
  5
 
 11023
 
 island
 
 0.44983
 
 0.73973
 
 Not queried
 
  3.5149828
 
 2.22e-01
 
 1.00e+00
  
 
  
 X
 
 153046480
 
 153046894
 
  PDF   STATS   ENSEMBL   UCSC 
 
  415
 
  5
 
 41146
 
 gene
 
 0.30492
 
 0.85861
 
 Not queried
 
  3.5139466
 
 7.94e-03
 
 1.00e+00
  
 
  
 2
 
   3452347
 
   3452624
 
  PDF   STATS   ENSEMBL   UCSC 
 
  278
 
  6
 
 22512
 
 gene;island
 
 0.55304
 
 0.95486
 
 Not queried
 
  3.5127594
 
 4.33e-03
 
 1.00e+00
  
 
  
 4
 
 140586921
 
 140587179
 
  PDF   STATS   ENSEMBL   UCSC 
 
  259
 
  6
 
 30085
 
 gene
 
 0.05406
 
 0.35972
 
 Not queried
 
  3.5121841
 
 2.16e-03
 
 1.00e+00
  
 
  
 X
 
  63425537
 
  63425930
 
  PDF   STATS   ENSEMBL   UCSC 
 
  394
 
  8
 
 41827
 
 island
 
 0.26471
 
 0.02676
 
 Not queried
 
  3.5110127
 
 6.22e-04
 
 1.00e+00
  
 
  
 12
 
  25405085
 
  25405317
 
  PDF   STATS   ENSEMBL   UCSC 
 
  233
 
  5
 
 9587
 
 tss
 
 0.65515
 
 0.18726
 
 Not queried
 
  3.5075827
 
 1.59e-02
 
 1.00e+00
  
 
  
 15
 
  62457456
 
  62457681
 
  PDF   STATS   ENSEMBL   UCSC 
 
  226
 
  4
 
 14171
 
 island
 
 0.48703
 
 0.09367
 
 Not queried
 
  3.5074633
 
 2.86e-02
 
 1.00e+00
  
 
  
 1
 
  35258435
 
  35258933
 
  PDF   STATS   ENSEMBL   UCSC 
 
  499
 
  4
 
 3019
 
 island
 
 0.05129
 
 0.45454
 
 Not queried
 
  3.5044044
 
 2.00e-01
 
 1.00e+00
  
 
  
 12
 
 115104274
 
 115104983
 
  PDF   STATS   ENSEMBL   UCSC 
 
  710
 
  7
 
 10816
 
 island
 
 0.67858
 
 0.09105
 
 Not queried
 
  3.5042512
 
 2.62e-02
 
 1.00e+00
  
 
  
 7
 
  56516076
 
  56516504
 
  PDF   STATS   ENSEMBL   UCSC 
 
  429
 
  9
 
 37546
 
 tss;island
 
 0.07804
 
 0.58951
 
 Not queried
 
  3.5025930
 
 7.77e-03
 
 1.00e+00
  
 
  
 11
 
  64937570
 
  64937697
 
  PDF   STATS   ENSEMBL   UCSC 
 
  128
 
  6
 
 7398
 
 tss
 
 0.22561
 
 0.79159
 
 Not queried
 
  3.5025856
 
 2.16e-03
 
 1.00e+00
  
 
  
 5
 
 140201153
 
 140201746
 
  PDF   STATS   ENSEMBL   UCSC 
 
  594
 
  5
 
 31739
 
 gene;island
 
 0.20369
 
 0.66624
 
 Not queried
 
  3.5024011
 
 7.94e-03
 
 1.00e+00
  
 
  
 11
 
   9635889
 
   9636089
 
  PDF   STATS   ENSEMBL   UCSC 
 
  201
 
  4
 
 8029
 
 island
 
 0.14056
 
 0.56514
 
 Not queried
 
  3.4999394
 
 2.86e-02
 
 1.00e+00
  
 
  
 11
 
    393328
 
    393648
 
  PDF   STATS   ENSEMBL   UCSC 
 
  321
 
  5
 
 6990
 
 tss;island
 
 0.30718
 
 0.75022
 
 Not queried
 
  3.4989959
 
 7.94e-03
 
 1.00e+00
  
 
  
 10
 
   3824387
 
   3824687
 
  PDF   STATS   ENSEMBL   UCSC 
 
  301
 
  4
 
 4463
 
 gene;island
 
 0.16658
 
 0.71206
 
 Not queried
 
  3.4986676
 
 2.86e-02
 
 1.00e+00
  
 
  
 19
 
  51416010
 
  51416213
 
  PDF   STATS   ENSEMBL   UCSC 
 
  204
 
  4
 
 22294
 
 island
 
 0.17141
 
 0.79625
 
 Not queried
 
  3.4979362
 
 1.14e-01
 
 1.00e+00
  
 
  
 3
 
  96533511
 
  96534005
 
  PDF   STATS   ENSEMBL   UCSC 
 
  495
 
  6
 
 27829
 
 gene;island
 
 0.02482
 
 0.30606
 
 Not queried
 
  3.4933012
 
 4.33e-03
 
 1.00e+00
  
 
  
 19
 
  51520093
 
  51520658
 
  PDF   STATS   ENSEMBL   UCSC 
 
  566
 
  6
 
 20150
 
 gene;island
 
 0.06346
 
 0.47685
 
 Not queried
 
  3.4927630
 
 2.16e-03
 
 1.00e+00
  
 
  
 12
 
 117407879
 
 117408186
 
  PDF   STATS   ENSEMBL   UCSC 
 
  308
 
  5
 
 9307
 
 gene
 
 0.43106
 
 0.94099
 
 Not queried
 
  3.4923796
 
 9.52e-02
 
 1.00e+00
  
 
  
 9
 
 140473545
 
 140473875
 
  PDF   STATS   ENSEMBL   UCSC 
 
  331
 
  5
 
 40794
 
 tss;island
 
 0.30322
 
 0.01392
 
 Not queried
 
  3.4921386
 
 2.22e-01
 
 1.00e+00
  
 
  
 8
 
  42234724
 
  42234975
 
  PDF   STATS   ENSEMBL   UCSC 
 
  252
 
  5
 
 39614
 
 tss
 
 0.25761
 
 0.83910
 
 Not queried
 
  3.4920551
 
 7.94e-03
 
 1.00e+00
  
 
  
 19
 
  49198900
 
  49199234
 
  PDF   STATS   ENSEMBL   UCSC 
 
  335
 
  5
 
 22195
 
 island
 
 0.04485
 
 0.36738
 
 Not queried
 
  3.4908413
 
 1.51e-01
 
 1.00e+00
  
 
  
 1
 
  99469577
 
  99469819
 
  PDF   STATS   ENSEMBL   UCSC 
 
  243
 
  4
 
 646
 
 gene;island
 
 0.46760
 
 0.08941
 
 Not queried
 
  3.4894950
 
 1.14e-01
 
 1.00e+00
  
 
  
 10
 
 104211319
 
 104211488
 
  PDF   STATS   ENSEMBL   UCSC 
 
  170
 
  5
 
 5308
 
 tss
 
 0.07646
 
 0.45134
 
 Not queried
 
  3.4888204
 
 7.94e-03
 
 1.00e+00
  
 
  
 11
 
  93583672
 
  93583973
 
  PDF   STATS   ENSEMBL   UCSC 
 
  302
 
  6
 
 7604
 
 tss
 
 0.33996
 
 0.85728
 
 Not queried
 
  3.4801934
 
 2.16e-03
 
 1.00e+00
  
 
  
 3
 
  48694451
 
  48694673
 
  PDF   STATS   ENSEMBL   UCSC 
 
  223
 
  4
 
 27726
 
 gene;island
 
 0.36080
 
 0.90796
 
 Not queried
 
  3.4790991
 
 2.86e-02
 
 1.00e+00
  
 
  
 13
 
  51417923
 
  51418221
 
  PDF   STATS   ENSEMBL   UCSC 
 
  299
 
  8
 
 11420
 
 tss
 
 0.10784
 
 0.54352
 
 Not queried
 
  3.4763443
 
 1.04e-02
 
 1.00e+00
  
 
  
 12
 
   4829115
 
   4829690
 
  PDF   STATS   ENSEMBL   UCSC 
 
  576
 
  7
 
 9476
 
 tss
 
 0.10945
 
 0.61648
 
 Not queried
 
  3.4737436
 
 1.17e-03
 
 1.00e+00
  
 
  
 8
 
  22926478
 
  22926911
 
  PDF   STATS   ENSEMBL   UCSC 
 
  434
 
 10
 
 40080
 
 island
 
 0.35774
 
 0.03085
 
 Not queried
 
  3.4716664
 
 1.85e-02
 
 1.00e+00
  
 
  
 3
 
  62305003
 
  62305285
 
  PDF   STATS   ENSEMBL   UCSC 
 
  283
 
  5
 
 28382
 
 tss;island
 
 0.60053
 
 0.18397
 
 Not queried
 
  3.4716490
 
 9.52e-02
 
 1.00e+00
  
 
  
 X
 
  70442524
 
  70443001
 
  PDF   STATS   ENSEMBL   UCSC 
 
  478
 
  6
 
 41373
 
 tss
 
 0.45870
 
 0.92457
 
 Not queried
 
  3.4713674
 
 1.52e-02
 
 1.00e+00
  
 
  
 13
 
  90015518
 
  90015912
 
  PDF   STATS   ENSEMBL   UCSC 
 
  395
 
  4
 
 11782
 
 island
 
 0.25485
 
 0.76790
 
 Not queried
 
  3.4690748
 
 1.14e-01
 
 1.00e+00
  
 
  
 2
 
 172945058
 
 172945383
 
  PDF   STATS   ENSEMBL   UCSC 
 
  326
 
  5
 
 22884
 
 gene;island
 
 0.79300
 
 0.20638
 
 Not queried
 
  3.4682274
 
 3.17e-02
 
 1.00e+00
  
 
  
 3
 
  49941376
 
  49941613
 
  PDF   STATS   ENSEMBL   UCSC 
 
  238
 
  7
 
 28297
 
 tss;island
 
 0.05276
 
 0.38100
 
 Not queried
 
  3.4660425
 
 6.99e-03
 
 1.00e+00
  
 
  
 20
 
  33146263
 
  33146543
 
  PDF   STATS   ENSEMBL   UCSC 
 
  281
 
  6
 
 25362
 
 gene;island
 
 0.05474
 
 0.35310
 
 Not queried
 
  3.4658960
 
 2.16e-03
 
 1.00e+00
  
 
  
 2
 
  80530701
 
  80530948
 
  PDF   STATS   ENSEMBL   UCSC 
 
  248
 
  4
 
 22718
 
 gene;island
 
 0.04706
 
 0.35017
 
 Not queried
 
  3.4655698
 
 2.86e-02
 
 1.00e+00
  
 
  
 7
 
 121956502
 
 121956890
 
  PDF   STATS   ENSEMBL   UCSC 
 
  389
 
  5
 
 38810
 
 island
 
 0.25978
 
 0.03634
 
 Not queried
 
  3.4646917
 
 3.17e-02
 
 1.00e+00
  
 
  
 5
 
 169532686
 
 169532775
 
  PDF   STATS   ENSEMBL   UCSC 
 
   90
 
  6
 
 32423
 
 tss
 
 0.22349
 
 0.73001
 
 Not queried
 
  3.4628055
 
 2.16e-03
 
 1.00e+00
  
 
  
 1
 
  68517177
 
  68517273
 
  PDF   STATS   ENSEMBL   UCSC 
 
   97
 
  4
 
 1684
 
 tss;island
 
 0.16121
 
 0.68139
 
 Not queried
 
  3.4601979
 
 2.86e-02
 
 1.00e+00
  
 
  
 1
 
 155294935
 
 155295267
 
  PDF   STATS   ENSEMBL   UCSC 
 
  333
 
  4
 
 803
 
 gene;tss;island
 
 0.25487
 
 0.02885
 
 Not queried
 
  3.4597406
 
 1.14e-01
 
 1.00e+00
  
 
  
 8
 
 141599141
 
 141599436
 
  PDF   STATS   ENSEMBL   UCSC 
 
  296
 
  5
 
 39380
 
 gene
 
 0.22532
 
 0.82334
 
 Not queried
 
  3.4594313
 
 7.94e-03
 
 1.00e+00
  
 
  
 8
 
 145925708
 
 145926089
 
  PDF   STATS   ENSEMBL   UCSC 
 
  382
 
  5
 
 40681
 
 island
 
 0.32782
 
 0.04457
 
 Not queried
 
  3.4590509
 
 7.94e-03
 
 1.00e+00
  
 
  
 5
 
  66124395
 
  66124588
 
  PDF   STATS   ENSEMBL   UCSC 
 
  194
 
  5
 
 32043
 
 tss
 
 0.02240
 
 0.24872
 
 Not queried
 
  3.4586564
 
 3.17e-02
 
 1.00e+00
  
 
  
 19
 
  19383613
 
  19383976
 
  PDF   STATS   ENSEMBL   UCSC 
 
  364
 
  4
 
 19914
 
 gene
 
 0.24905
 
 0.04202
 
 Not queried
 
  3.4572562
 
 2.86e-02
 
 1.00e+00
  
 
  
 1
 
  26735846
 
  26736154
 
  PDF   STATS   ENSEMBL   UCSC 
 
  309
 
  7
 
 1387
 
 tss;island
 
 0.43022
 
 0.08338
 
 Not queried
 
  3.4556998
 
 5.83e-04
 
 1.00e+00
  
 
  
 3
 
 147128998
 
 147129278
 
  PDF   STATS   ENSEMBL   UCSC 
 
  281
 
  4
 
 27947
 
 gene;island
 
 0.03094
 
 0.33807
 
 Not queried
 
  3.4553029
 
 2.86e-02
 
 1.00e+00
  
 
  
 14
 
  77767644
 
  77767800
 
  PDF   STATS   ENSEMBL   UCSC 
 
  157
 
  4
 
 12123
 
 gene
 
 0.24281
 
 0.58997
 
 Not queried
 
  3.4537057
 
 1.14e-01
 
 1.00e+00
  
 
  
 10
 
  73136913
 
  73137135
 
  PDF   STATS   ENSEMBL   UCSC 
 
  223
 
  4
 
 5809
 
 island
 
 0.33654
 
 0.86148
 
 Not queried
 
  3.4528988
 
 5.71e-02
 
 1.00e+00
  
 
  
 3
 
 126007325
 
 126007563
 
  PDF   STATS   ENSEMBL   UCSC 
 
  239
 
  4
 
 29386
 
 island
 
 0.68440
 
 0.18713
 
 Not queried
 
  3.4522569
 
 2.86e-02
 
 1.00e+00
  
 
  
 2
 
 241389021
 
 241389258
 
  PDF   STATS   ENSEMBL   UCSC 
 
  238
 
  4
 
 23083
 
 gene;island
 
 0.36614
 
 0.80420
 
 Not queried
 
  3.4500414
 
 3.43e-01
 
 1.00e+00
  
 
  
 19
 
   9473565
 
   9473691
 
  PDF   STATS   ENSEMBL   UCSC 
 
  127
 
  6
 
 20387
 
 tss
 
 0.92704
 
 0.61502
 
 Not queried
 
  3.4500243
 
 2.16e-03
 
 1.00e+00
  
 
  
 11
 
  65816655
 
  65816985
 
  PDF   STATS   ENSEMBL   UCSC 
 
  331
 
  6
 
 7436
 
 tss
 
 0.84637
 
 0.33401
 
 Not queried
 
  3.4467598
 
 2.16e-03
 
 1.00e+00
  
 
  
 11
 
   2891065
 
   2891118
 
  PDF   STATS   ENSEMBL   UCSC 
 
   54
 
  5
 
 7059
 
 tss;island
 
 0.29402
 
 0.80424
 
 Not queried
 
  3.4459573
 
 7.94e-03
 
 1.00e+00
  
 
  
 20
 
  32856747
 
  32857072
 
  PDF   STATS   ENSEMBL   UCSC 
 
  326
 
  4
 
 25359
 
 gene
 
 0.61666
 
 0.94052
 
 Not queried
 
  3.4448682
 
 2.86e-02
 
 1.00e+00
  
 
  
 8
 
  24772878
 
  24773268
 
  PDF   STATS   ENSEMBL   UCSC 
 
  391
 
  4
 
 39184
 
 gene;island
 
 0.38932
 
 0.83321
 
 Not queried
 
  3.4441122
 
 5.71e-02
 
 1.00e+00
  
 
  
 19
 
  11529133
 
  11529947
 
  PDF   STATS   ENSEMBL   UCSC 
 
  815
 
  7
 
 19803
 
 gene
 
 0.28811
 
 0.04635
 
 Not queried
 
  3.4439079
 
 1.28e-01
 
 1.00e+00
  
 
  
 16
 
    215410
 
    216244
 
  PDF   STATS   ENSEMBL   UCSC 
 
  835
 
  9
 
 15413
 
 island
 
 0.03143
 
 0.26423
 
 Not queried
 
  3.4407200
 
 2.76e-03
 
 1.00e+00
  
 
  
 13
 
  61989701
 
  61990025
 
  PDF   STATS   ENSEMBL   UCSC 
 
  325
 
  8
 
 11442
 
 tss
 
 0.24253
 
 0.04022
 
 Not queried
 
  3.4407105
 
 2.95e-03
 
 1.00e+00
  
 
  
 13
 
 113776873
 
 113776964
 
  PDF   STATS   ENSEMBL   UCSC 
 
   92
 
  4
 
 11928
 
 island
 
 0.36943
 
 0.81927
 
 Not queried
 
  3.4389681
 
 2.86e-02
 
 1.00e+00
  
 
  
 22
 
  19743759
 
  19744078
 
  PDF   STATS   ENSEMBL   UCSC 
 
  320
 
  7
 
 26924
 
 tss
 
 0.25675
 
 0.75550
 
 Not queried
 
  3.4361743
 
 5.83e-04
 
 1.00e+00
  
 
  
 14
 
 106857188
 
 106857655
 
  PDF   STATS   ENSEMBL   UCSC 
 
  468
 
  4
 
 13218
 
 island
 
 0.49083
 
 0.89650
 
 Not queried
 
  3.4359978
 
 2.86e-02
 
 1.00e+00
  
 
  
 1
 
 154539974
 
 154540159
 
  PDF   STATS   ENSEMBL   UCSC 
 
  186
 
  4
 
 1963
 
 tss
 
 0.82650
 
 0.34296
 
 Not queried
 
  3.4356512
 
 2.86e-02
 
 1.00e+00
  
 
  
 3
 
 183948058
 
 183948322
 
  PDF   STATS   ENSEMBL   UCSC 
 
  265
 
  6
 
 29672
 
 island
 
 0.02572
 
 0.15891
 
 Not queried
 
  3.4343512
 
 1.52e-02
 
 1.00e+00
  
 
  
 X
 
 153603062
 
 153603268
 
  PDF   STATS   ENSEMBL   UCSC 
 
  207
 
  7
 
 41595
 
 tss
 
 0.05062
 
 0.38745
 
 Not queried
 
  3.4343271
 
 2.62e-02
 
 1.00e+00
  
 
  
 22
 
  17956453
 
  17956560
 
  PDF   STATS   ENSEMBL   UCSC 
 
  108
 
  4
 
 26902
 
 tss
 
 0.46309
 
 0.90955
 
 Not queried
 
  3.4341084
 
 2.86e-02
 
 1.00e+00
  
 
  
 8
 
 144921531
 
 144921852
 
  PDF   STATS   ENSEMBL   UCSC 
 
  322
 
  4
 
 39429
 
 gene;island
 
 0.37872
 
 0.87115
 
 Not queried
 
  3.4315436
 
 2.86e-02
 
 1.00e+00
  
 
  
 17
 
  76102354
 
  76102509
 
  PDF   STATS   ENSEMBL   UCSC 
 
  156
 
  4
 
 16778
 
 gene;island
 
 0.30377
 
 0.81423
 
 Not queried
 
  3.4299033
 
 2.00e-01
 
 1.00e+00
  
 
  
 1
 
  17746286
 
  17746597
 
  PDF   STATS   ENSEMBL   UCSC 
 
  312
 
  6
 
 265
 
 gene;island
 
 0.09777
 
 0.65367
 
 Not queried
 
  3.4298101
 
 9.31e-02
 
 1.00e+00
  
 
  
 18
 
  77289084
 
  77289452
 
  PDF   STATS   ENSEMBL   UCSC 
 
  369
 
  4
 
 19568
 
 island
 
 0.09178
 
 0.52993
 
 Not queried
 
  3.4292434
 
 2.00e-01
 
 1.00e+00
  
 
  
 6
 
 159360705
 
 159360984
 
  PDF   STATS   ENSEMBL   UCSC 
 
  280
 
  4
 
 36540
 
 island
 
 0.08038
 
 0.58731
 
 Not queried
 
  3.4290016
 
 1.14e-01
 
 1.00e+00
  
 
  
 3
 
  48507354
 
  48507618
 
  PDF   STATS   ENSEMBL   UCSC 
 
  265
 
  6
 
 27725
 
 gene
 
 0.35968
 
 0.85690
 
 Not queried
 
  3.4288442
 
 1.52e-02
 
 1.00e+00
  
 
  
 6
 
 166666838
 
 166667648
 
  PDF   STATS   ENSEMBL   UCSC 
 
  811
 
  7
 
 36566
 
 island
 
 0.05232
 
 0.33478
 
 Not queried
 
  3.4260612
 
 2.62e-02
 
 1.00e+00
  
 
  
 19
 
  45720771
 
  45721076
 
  PDF   STATS   ENSEMBL   UCSC 
 
  306
 
  4
 
 20053
 
 gene;island
 
 0.24784
 
 0.76415
 
 Not queried
 
  3.4240699
 
 5.71e-02
 
 1.00e+00
  
 
  
 15
 
  67458370
 
  67458665
 
  PDF   STATS   ENSEMBL   UCSC 
 
  296
 
  4
 
 13364
 
 gene
 
 0.69551
 
 0.21292
 
 Not queried
 
  3.4235429
 
 1.14e-01
 
 1.00e+00
  
 
  
 5
 
 122434178
 
 122434544
 
  PDF   STATS   ENSEMBL   UCSC 
 
  367
 
  5
 
 31674
 
 gene;island
 
 0.03928
 
 0.26204
 
 Not queried
 
  3.4233830
 
 3.17e-02
 
 1.00e+00
  
 
  
 17
 
   7227499
 
   7227737
 
  PDF   STATS   ENSEMBL   UCSC 
 
  239
 
  4
 
 16355
 
 gene;island
 
 0.36702
 
 0.76421
 
 Not queried
 
  3.4218561
 
 5.71e-02
 
 1.00e+00
  
 
  
 12
 
 103358619
 
 103358881
 
  PDF   STATS   ENSEMBL   UCSC 
 
  263
 
  6
 
 10702
 
 island
 
 0.03885
 
 0.15042
 
 Not queried
 
  3.4194009
 
 2.60e-02
 
 1.00e+00
  
 
  
 4
 
   3464653
 
   3465064
 
  PDF   STATS   ENSEMBL   UCSC 
 
  412
 
 10
 
 30790
 
 island
 
 0.44303
 
 0.80568
 
 Not queried
 
  3.4184114
 
 4.33e-02
 
 1.00e+00
  
 
  
 10
 
  73848764
 
  73849167
 
  PDF   STATS   ENSEMBL   UCSC 
 
  404
 
  9
 
 5817
 
 island
 
 0.27862
 
 0.82957
 
 Not queried
 
  3.4179740
 
 1.85e-03
 
 1.00e+00
  
 
  
 16
 
  71659830
 
  71660055
 
  PDF   STATS   ENSEMBL   UCSC 
 
  226
 
  5
 
 15317
 
 tss
 
 0.02788
 
 0.20028
 
 Not queried
 
  3.4172443
 
 9.52e-02
 
 1.00e+00
  
 
  
 10
 
  16561202
 
  16561605
 
  PDF   STATS   ENSEMBL   UCSC 
 
  404
 
  5
 
 4502
 
 gene;island
 
 0.83804
 
 0.37014
 
 Not queried
 
  3.4160908
 
 5.56e-02
 
 1.00e+00
  
 
  
 4
 
  40517938
 
  40518143
 
  PDF   STATS   ENSEMBL   UCSC 
 
  206
 
  5
 
 29942
 
 gene
 
 0.12609
 
 0.57799
 
 Not queried
 
  3.4132873
 
 7.94e-03
 
 1.00e+00
  
 
  
 15
 
  48470606
 
  48470973
 
  PDF   STATS   ENSEMBL   UCSC 
 
  368
 
  6
 
 13626
 
 tss
 
 0.24623
 
 0.03866
 
 Not queried
 
  3.4132050
 
 9.31e-02
 
 1.00e+00
  
 
  
 17
 
  46695192
 
  46695514
 
  PDF   STATS   ENSEMBL   UCSC 
 
  323
 
  4
 
 18457
 
 island
 
 0.03262
 
 0.37632
 
 Not queried
 
  3.4089867
 
 1.14e-01
 
 1.00e+00
  
 
  
 16
 
    691444
 
    691677
 
  PDF   STATS   ENSEMBL   UCSC 
 
  234
 
  5
 
 14940
 
 tss;island
 
 0.34668
 
 0.04632
 
 Not queried
 
  3.4067469
 
 3.17e-02
 
 1.00e+00
  
 
  
 6
 
  73973008
 
  73973128
 
  PDF   STATS   ENSEMBL   UCSC 
 
  121
 
  5
 
 35001
 
 tss
 
 0.24757
 
 0.05621
 
 Not queried
 
  3.4029825
 
 7.94e-03
 
 1.00e+00
  
 
  
 7
 
   4243286
 
   4243734
 
  PDF   STATS   ENSEMBL   UCSC 
 
  449
 
  5
 
 36771
 
 gene
 
 0.36100
 
 0.89170
 
 Not queried
 
  3.4021607
 
 7.94e-03
 
 1.00e+00
  
 
  
 2
 
 157291759
 
 157292127
 
  PDF   STATS   ENSEMBL   UCSC 
 
  369
 
  6
 
 23636
 
 tss;island
 
 0.37567
 
 0.09479
 
 Not queried
 
  3.4017772
 
 3.10e-01
 
 1.00e+00
  
 
  
 2
 
 203036093
 
 203036235
 
  PDF   STATS   ENSEMBL   UCSC 
 
  143
 
  4
 
 24978
 
 island
 
 0.29891
 
 0.02516
 
 Not queried
 
  3.4015657
 
 5.71e-02
 
 1.00e+00
  
 
  
 10
 
  45719751
 
  45720134
 
  PDF   STATS   ENSEMBL   UCSC 
 
  384
 
  5
 
 5693
 
 island
 
 0.31441
 
 0.88327
 
 Not queried
 
  3.4013886
 
 7.94e-03
 
 1.00e+00
  
 
  
 7
 
   4304779
 
   4305061
 
  PDF   STATS   ENSEMBL   UCSC 
 
  283
 
  4
 
 36775
 
 gene;island
 
 0.60874
 
 0.94737
 
 Not queried
 
  3.4013274
 
 2.86e-02
 
 1.00e+00
  
 
  
 6
 
 134497084
 
 134497755
 
  PDF   STATS   ENSEMBL   UCSC 
 
  672
 
  9
 
 34417
 
 gene;tss;island
 
 0.11246
 
 0.05573
 
 Not queried
 
  3.4004316
 
 6.25e-02
 
 1.00e+00
  
 
  
 X
 
  70442524
 
  70443093
 
  PDF   STATS   ENSEMBL   UCSC 
 
  570
 
  7
 
 41021
 
 gene;island
 
 0.45907
 
 0.94116
 
 Not queried
 
  3.3990937
 
 2.62e-02
 
 1.00e+00
  
 
  
 5
 
 140613740
 
 140613884
 
  PDF   STATS   ENSEMBL   UCSC 
 
  145
 
  5
 
 32309
 
 tss
 
 0.04238
 
 0.29488
 
 Not queried
 
  3.3932810
 
 7.94e-03
 
 1.00e+00
  
 
  
 20
 
  30639816
 
  30640256
 
  PDF   STATS   ENSEMBL   UCSC 
 
  441
 
 10
 
 26049
 
 island
 
 0.30958
 
 0.03541
 
 Not queried
 
  3.3930022
 
 3.89e-03
 
 1.00e+00
  
 
  
 12
 
 132514292
 
 132514508
 
  PDF   STATS   ENSEMBL   UCSC 
 
  217
 
  5
 
 10100
 
 tss
 
 0.40485
 
 0.87716
 
 Not queried
 
  3.3919770
 
 7.94e-03
 
 1.00e+00
  
 
  
 6
 
  32123479
 
  32123701
 
  PDF   STATS   ENSEMBL   UCSC 
 
  223
 
  5
 
 33999
 
 gene;island
 
 0.57042
 
 0.95056
 
 Not queried
 
  3.3906924
 
 9.52e-02
 
 1.00e+00
  
 
  
 X
 
  68725052
 
  68725088
 
  PDF   STATS   ENSEMBL   UCSC 
 
   37
 
  4
 
 41842
 
 island
 
 0.29844
 
 0.03661
 
 Not queried
 
  3.3903009
 
 2.86e-02
 
 1.00e+00
  
 
  
 21
 
  34444104
 
  34444382
 
  PDF   STATS   ENSEMBL   UCSC 
 
  279
 
  4
 
 26361
 
 gene;island
 
 0.00308
 
 0.01597
 
 Not queried
 
  3.3900541
 
 3.43e-01
 
 1.00e+00
  
 
  
 19
 
  12946093
 
  12946465
 
  PDF   STATS   ENSEMBL   UCSC 
 
  373
 
  8
 
 21589
 
 island
 
 0.03281
 
 0.22419
 
 Not queried
 
  3.3868208
 
 6.22e-04
 
 1.00e+00
  
 
  
 20
 
  33413560
 
  33413713
 
  PDF   STATS   ENSEMBL   UCSC 
 
  154
 
  6
 
 25657
 
 tss;island
 
 0.03402
 
 0.29144
 
 Not queried
 
  3.3865324
 
 2.60e-02
 
 1.00e+00
  
 
  
 12
 
  58119915
 
  58120011
 
  PDF   STATS   ENSEMBL   UCSC 
 
   97
 
  5
 
 9171
 
 gene;tss;island
 
 0.03468
 
 0.32452
 
 Not queried
 
  3.3850260
 
 7.94e-03
 
 1.00e+00
  
 
  
 1
 
 220921855
 
 220922217
 
  PDF   STATS   ENSEMBL   UCSC 
 
  363
 
  6
 
 1017
 
 gene
 
 0.33196
 
 0.02550
 
 Not queried
 
  3.3799850
 
 8.66e-03
 
 1.00e+00
  
 
  
 7
 
 129422915
 
 129423354
 
  PDF   STATS   ENSEMBL   UCSC 
 
  440
 
  4
 
 38849
 
 island
 
 0.86409
 
 0.43330
 
 Not queried
 
  3.3783307
 
 2.86e-02
 
 1.00e+00
  
 
  
 17
 
   9550137
 
   9550296
 
  PDF   STATS   ENSEMBL   UCSC 
 
  160
 
  4
 
 16401
 
 gene;island
 
 0.06320
 
 0.59252
 
 Not queried
 
  3.3782224
 
 5.71e-02
 
 1.00e+00
  
 
  
 17
 
  75524913
 
  75525151
 
  PDF   STATS   ENSEMBL   UCSC 
 
  239
 
  4
 
 18776
 
 island
 
 0.11068
 
 0.02715
 
 Not queried
 
  3.3763053
 
 1.14e-01
 
 1.00e+00
  
 
  
 X
 
  48814914
 
  48815125
 
  PDF   STATS   ENSEMBL   UCSC 
 
  212
 
  6
 
 41302
 
 tss
 
 0.42605
 
 0.04742
 
 Not queried
 
  3.3758644
 
 6.49e-02
 
 1.00e+00
  
 
  
 16
 
    891260
 
    891400
 
  PDF   STATS   ENSEMBL   UCSC 
 
  141
 
  4
 
 15462
 
 island
 
 0.44162
 
 0.89696
 
 Not queried
 
  3.3754427
 
 2.86e-02
 
 1.00e+00
  
 
  
 7
 
  89783435
 
  89783680
 
  PDF   STATS   ENSEMBL   UCSC 
 
  246
 
  6
 
 37630
 
 tss
 
 0.02629
 
 0.23078
 
 Not queried
 
  3.3749188
 
 1.52e-02
 
 1.00e+00
  
 
  
 3
 
  77147070
 
  77147364
 
  PDF   STATS   ENSEMBL   UCSC 
 
  295
 
  5
 
 27820
 
 gene;island
 
 0.50708
 
 0.91024
 
 Not queried
 
  3.3743922
 
 7.94e-03
 
 1.00e+00
  
 
  
 2
 
 241640149
 
 241640444
 
  PDF   STATS   ENSEMBL   UCSC 
 
  296
 
  4
 
 25240
 
 island
 
 0.48998
 
 0.79592
 
 Not queried
 
  3.3742568
 
 2.86e-02
 
 1.00e+00
  
 
  
 11
 
  31822243
 
  31822526
 
  PDF   STATS   ENSEMBL   UCSC 
 
  284
 
  4
 
 6539
 
 gene;island
 
 0.33649
 
 0.86265
 
 Not queried
 
  3.3727968
 
 2.00e-01
 
 1.00e+00
  
 
  
 10
 
 129948776
 
 129949058
 
  PDF   STATS   ENSEMBL   UCSC 
 
  283
 
  5
 
 6233
 
 island
 
 0.21173
 
 0.01948
 
 Not queried
 
  3.3703618
 
 3.17e-02
 
 1.00e+00
  
 
  
 4
 
 186131234
 
 186131285
 
  PDF   STATS   ENSEMBL   UCSC 
 
   52
 
  4
 
 31419
 
 island
 
 0.05387
 
 0.51774
 
 Not queried
 
  3.3699483
 
 2.86e-02
 
 1.00e+00
  
 
  
 5
 
 114938148
 
 114938459
 
  PDF   STATS   ENSEMBL   UCSC 
 
  312
 
  5
 
 32169
 
 tss
 
 0.37565
 
 0.88478
 
 Not queried
 
  3.3684130
 
 7.94e-03
 
 1.00e+00
  
 
  
 10
 
  85985296
 
  85985448
 
  PDF   STATS   ENSEMBL   UCSC 
 
  153
 
  4
 
 5176
 
 tss
 
 0.16538
 
 0.63219
 
 Not queried
 
  3.3671417
 
 2.00e-01
 
 1.00e+00
  
 
  
 17
 
  46035183
 
  46035343
 
  PDF   STATS   ENSEMBL   UCSC 
 
  161
 
  4
 
 17415
 
 tss
 
 0.07550
 
 0.43164
 
 Not queried
 
  3.3670506
 
 5.71e-02
 
 1.00e+00
  
 
  
 1
 
  98515041
 
  98515272
 
  PDF   STATS   ENSEMBL   UCSC 
 
  232
 
  4
 
 3508
 
 island
 
 0.03770
 
 0.29592
 
 Not queried
 
  3.3665734
 
 1.14e-01
 
 1.00e+00
  
 
  
 X
 
  63263994
 
  63264258
 
  PDF   STATS   ENSEMBL   UCSC 
 
  265
 
  4
 
 41826
 
 island
 
 0.17790
 
 0.64416
 
 Not queried
 
  3.3664744
 
 2.86e-02
 
 1.00e+00
  
 
  
 11
 
  32354958
 
  32355509
 
  PDF   STATS   ENSEMBL   UCSC 
 
  552
 
  6
 
 8153
 
 island
 
 0.19783
 
 0.76284
 
 Not queried
 
  3.3627245
 
 4.33e-03
 
 1.00e+00
  
 
  
 4
 
  40518024
 
  40518143
 
  PDF   STATS   ENSEMBL   UCSC 
 
  120
 
  4
 
 30311
 
 tss
 
 0.09162
 
 0.56911
 
 Not queried
 
  3.3620362
 
 2.86e-02
 
 1.00e+00
  
 
  
 7
 
 150020751
 
 150020945
 
  PDF   STATS   ENSEMBL   UCSC 
 
  195
 
  7
 
 37208
 
 gene;island
 
 0.37984
 
 0.05624
 
 Not queried
 
  3.3614235
 
 2.33e-03
 
 1.00e+00
  
 
  
 11
 
  86383182
 
  86383940
 
  PDF   STATS   ENSEMBL   UCSC 
 
  759
 
 20
 
 8688
 
 island
 
 0.10370
 
 0.60214
 
 Not queried
 
  3.3610975
 
 3.53e-03
 
 1.00e+00
  
 
  
 20
 
  35972924
 
  35973098
 
  PDF   STATS   ENSEMBL   UCSC 
 
  175
 
  5
 
 25687
 
 tss;island
 
 0.33009
 
 0.80432
 
 Not queried
 
  3.3609356
 
 7.94e-03
 
 1.00e+00
  
 
  
 2
 
 133343115
 
 133343373
 
  PDF   STATS   ENSEMBL   UCSC 
 
  259
 
  5
 
 22839
 
 gene;island
 
 0.40761
 
 0.78486
 
 Not queried
 
  3.3569336
 
 7.94e-03
 
 1.00e+00
  
 
  
 6
 
 149772707
 
 149772892
 
  PDF   STATS   ENSEMBL   UCSC 
 
  186
 
  4
 
 34440
 
 gene;island
 
 0.37217
 
 0.82104
 
 Not queried
 
  3.3564160
 
 2.86e-02
 
 1.00e+00
  
 
  
 12
 
  54454163
 
  54454445
 
  PDF   STATS   ENSEMBL   UCSC 
 
  283
 
  5
 
 9138
 
 gene;island
 
 0.91125
 
 0.43001
 
 Not queried
 
  3.3557443
 
 7.94e-03
 
 1.00e+00
  
 
  
 11
 
 126455596
 
 126456119
 
  PDF   STATS   ENSEMBL   UCSC 
 
  524
 
  6
 
 6943
 
 gene
 
 0.26926
 
 0.68543
 
 Not queried
 
  3.3547397
 
 1.52e-02
 
 1.00e+00
  
 
  
 1
 
  20878760
 
  20879029
 
  PDF   STATS   ENSEMBL   UCSC 
 
  270
 
  5
 
 2789
 
 island
 
 0.40664
 
 0.05277
 
 Not queried
 
  3.3543475
 
 1.51e-01
 
 1.00e+00
  
 
  
 17
 
   4486934
 
   4487125
 
  PDF   STATS   ENSEMBL   UCSC 
 
  192
 
  5
 
 16993
 
 tss;island
 
 0.35102
 
 0.78991
 
 Not queried
 
  3.3542201
 
 7.94e-03
 
 1.00e+00
  
 
  
 16
 
    281115
 
    281424
 
  PDF   STATS   ENSEMBL   UCSC 
 
  310
 
  5
 
 15415
 
 island
 
 0.20633
 
 0.85941
 
 Not queried
 
  3.3495041
 
 1.51e-01
 
 1.00e+00
  
 
  
 3
 
  65342546
 
  65342971
 
  PDF   STATS   ENSEMBL   UCSC 
 
  426
 
  5
 
 27810
 
 gene;island
 
 0.78608
 
 0.96955
 
 Not queried
 
  3.3488605
 
 7.94e-03
 
 1.00e+00
  
 
  
 1
 
 179050673
 
 179051076
 
  PDF   STATS   ENSEMBL   UCSC 
 
  404
 
  7
 
 2111
 
 tss
 
 0.05343
 
 0.45913
 
 Not queried
 
  3.3481607
 
 2.62e-02
 
 1.00e+00
  
 
  
 2
 
  45159434
 
  45159663
 
  PDF   STATS   ENSEMBL   UCSC 
 
  230
 
  4
 
 24221
 
 island
 
 0.79454
 
 0.28469
 
 Not queried
 
  3.3480761
 
 2.86e-02
 
 1.00e+00
  
 
  
 1
 
    901685
 
    902025
 
  PDF   STATS   ENSEMBL   UCSC 
 
  341
 
  6
 
 2383
 
 island
 
 0.07560
 
 0.43579
 
 Not queried
 
  3.3477485
 
 2.16e-03
 
 1.00e+00
  
 
  
 3
 
  46506404
 
  46506554
 
  PDF   STATS   ENSEMBL   UCSC 
 
  151
 
  6
 
 28233
 
 tss
 
 0.44082
 
 0.87391
 
 Not queried
 
  3.3470239
 
 2.16e-03
 
 1.00e+00
  
 
  
 10
 
 128193219
 
 128193497
 
  PDF   STATS   ENSEMBL   UCSC 
 
  279
 
  5
 
 4802
 
 gene;island
 
 0.18977
 
 0.70393
 
 Not queried
 
  3.3436463
 
 1.59e-02
 
 1.00e+00
  
 
  
 10
 
 135342218
 
 135342620
 
  PDF   STATS   ENSEMBL   UCSC 
 
  403
 
  4
 
 4891
 
 gene;island
 
 0.75688
 
 0.23343
 
 Not queried
 
  3.3400259
 
 2.86e-02
 
 1.00e+00
  
 
  
 10
 
  73848817
 
  73849167
 
  PDF   STATS   ENSEMBL   UCSC 
 
  351
 
  8
 
 5129
 
 tss
 
 0.28809
 
 0.80512
 
 Not queried
 
  3.3395686
 
 4.66e-03
 
 1.00e+00
  
 
  
 17
 
  41131966
 
  41132169
 
  PDF   STATS   ENSEMBL   UCSC 
 
  204
 
  5
 
 16556
 
 gene;tss;island
 
 0.18491
 
 0.02391
 
 Not queried
 
  3.3395251
 
 7.94e-03
 
 1.00e+00
  
 
  
 6
 
  17988404
 
  17988960
 
  PDF   STATS   ENSEMBL   UCSC 
 
  557
 
  9
 
 34593
 
 tss;island
 
 0.30978
 
 0.03306
 
 Not queried
 
  3.3393398
 
 6.25e-02
 
 1.00e+00
  
 
  
 X
 
 153602509
 
 153603268
 
  PDF   STATS   ENSEMBL   UCSC 
 
  760
 
 11
 
 42136
 
 island
 
 0.05062
 
 0.38745
 
 Not queried
 
  3.3387781
 
 7.65e-04
 
 1.00e+00
  
 
  
 15
 
  75199305
 
  75199493
 
  PDF   STATS   ENSEMBL   UCSC 
 
  189
 
  4
 
 14292
 
 island
 
 0.04177
 
 0.27109
 
 Not queried
 
  3.3379390
 
 2.00e-01
 
 1.00e+00
  
 
  
 1
 
  78511140
 
  78511488
 
  PDF   STATS   ENSEMBL   UCSC 
 
  349
 
  7
 
 1707
 
 tss
 
 0.50316
 
 0.07322
 
 Not queried
 
  3.3366496
 
 5.83e-04
 
 1.00e+00
  
 
  
 7
 
  38468944
 
  38469273
 
  PDF   STATS   ENSEMBL   UCSC 
 
  330
 
  5
 
 36908
 
 gene
 
 0.33235
 
 0.87057
 
 Not queried
 
  3.3346619
 
 1.59e-02
 
 1.00e+00
  
 
  
 7
 
 156795767
 
 156796145
 
  PDF   STATS   ENSEMBL   UCSC 
 
  379
 
  5
 
 39028
 
 island
 
 0.16744
 
 0.04295
 
 Not queried
 
  3.3323135
 
 1.59e-02
 
 1.00e+00
  
 
  
 19
 
  19221316
 
  19221574
 
  PDF   STATS   ENSEMBL   UCSC 
 
  259
 
  4
 
 19911
 
 gene;island
 
 0.24071
 
 0.72848
 
 Not queried
 
  3.3305001
 
 2.86e-02
 
 1.00e+00
  
 
  
 10
 
  38691306
 
  38691563
 
  PDF   STATS   ENSEMBL   UCSC 
 
  258
 
  4
 
 4548
 
 gene;island
 
 0.11157
 
 0.50462
 
 Not queried
 
  3.3302700
 
 2.86e-02
 
 1.00e+00
  
 
  
 1
 
 214152709
 
 214153036
 
  PDF   STATS   ENSEMBL   UCSC 
 
  328
 
  5
 
 4180
 
 island
 
 0.90043
 
 0.39602
 
 Not queried
 
  3.3281495
 
 7.94e-03
 
 1.00e+00
  
 
  
 X
 
 100914892
 
 100915108
 
  PDF   STATS   ENSEMBL   UCSC 
 
  217
 
  4
 
 41427
 
 tss
 
 0.41815
 
 0.05447
 
 Not queried
 
  3.3260209
 
 2.86e-02
 
 1.00e+00
  
 
  
 1
 
  24239820
 
  24240069
 
  PDF   STATS   ENSEMBL   UCSC 
 
  250
 
  4
 
 1348
 
 tss
 
 0.24570
 
 0.59767
 
 Not queried
 
  3.3257799
 
 1.14e-01
 
 1.00e+00
  
 
  
 14
 
  91720173
 
  91720696
 
  PDF   STATS   ENSEMBL   UCSC 
 
  524
 
  6
 
 13049
 
 island
 
 0.52555
 
 0.07616
 
 Not queried
 
  3.3220350
 
 6.49e-02
 
 1.00e+00
  
 
  
 16
 
  29820429
 
  29820592
 
  PDF   STATS   ENSEMBL   UCSC 
 
  164
 
  4
 
 14682
 
 gene;island
 
 0.55742
 
 0.91733
 
 Not queried
 
  3.3214660
 
 2.86e-02
 
 1.00e+00
  
 
  
 20
 
   3713341
 
   3713584
 
  PDF   STATS   ENSEMBL   UCSC 
 
  244
 
  4
 
 25933
 
 island
 
 0.15016
 
 0.61390
 
 Not queried
 
  3.3204181
 
 2.86e-02
 
 1.00e+00
  
 
  
 3
 
 181444368
 
 181445156
 
  PDF   STATS   ENSEMBL   UCSC 
 
  789
 
  9
 
 28002
 
 gene;island
 
 0.05535
 
 0.33505
 
 Not queried
 
  3.3163570
 
 7.82e-04
 
 1.00e+00
  
 
  
 2
 
 238767990
 
 238768104
 
  PDF   STATS   ENSEMBL   UCSC 
 
  115
 
  4
 
 23929
 
 tss;island
 
 0.10227
 
 0.50778
 
 Not queried
 
  3.3156149
 
 2.86e-02
 
 1.00e+00
  
 
  
 5
 
  23507450
 
  23507656
 
  PDF   STATS   ENSEMBL   UCSC 
 
  207
 
  9
 
 31942
 
 tss
 
 0.01873
 
 0.13649
 
 Not queried
 
  3.3120556
 
 7.82e-04
 
 1.00e+00
  
 
  
 X
 
  68836036
 
  68836202
 
  PDF   STATS   ENSEMBL   UCSC 
 
  167
 
  4
 
 41011
 
 gene
 
 0.24792
 
 0.02590
 
 Not queried
 
  3.3115604
 
 2.86e-02
 
 1.00e+00
  
 
  
 10
 
 135043492
 
 135043586
 
  PDF   STATS   ENSEMBL   UCSC 
 
   95
 
  4
 
 5436
 
 tss;island
 
 0.31810
 
 0.04676
 
 Not queried
 
  3.3113619
 
 2.86e-02
 
 1.00e+00
  
 
  
 2
 
  71205052
 
  71205564
 
  PDF   STATS   ENSEMBL   UCSC 
 
  513
 
  6
 
 23386
 
 tss
 
 0.41227
 
 0.03503
 
 Not queried
 
  3.3106022
 
 9.31e-02
 
 1.00e+00
  
 
  
 X
 
  70273027
 
  70273384
 
  PDF   STATS   ENSEMBL   UCSC 
 
  358
 
  5
 
 41854
 
 island
 
 0.53129
 
 0.92601
 
 Not queried
 
  3.3087271
 
 7.94e-03
 
 1.00e+00
  
 
  
 14
 
  74185274
 
  74185511
 
  PDF   STATS   ENSEMBL   UCSC 
 
  238
 
  4
 
 12105
 
 gene;island
 
 0.38240
 
 0.85275
 
 Not queried
 
  3.3086417
 
 5.71e-02
 
 1.00e+00
  
 
  
 5
 
 140729429
 
 140729813
 
  PDF   STATS   ENSEMBL   UCSC 
 
  385
 
  7
 
 32315
 
 tss
 
 0.01348
 
 0.12212
 
 Not queried
 
  3.3083333
 
 2.33e-03
 
 1.00e+00
  
 
  
 6
 
  39016262
 
  39016776
 
  PDF   STATS   ENSEMBL   UCSC 
 
  515
 
  7
 
 36043
 
 island
 
 0.56546
 
 0.10936
 
 Not queried
 
  3.3075980
 
 5.83e-04
 
 1.00e+00
  
 
  
 6
 
  14211743
 
  14212256
 
  PDF   STATS   ENSEMBL   UCSC 
 
  514
 
  5
 
 35400
 
 island
 
 0.54625
 
 0.07642
 
 Not queried
 
  3.3054476
 
 5.48e-01
 
 1.00e+00
  
 
  
 10
 
 131323903
 
 131324189
 
  PDF   STATS   ENSEMBL   UCSC 
 
  287
 
  4
 
 4809
 
 gene
 
 0.59463
 
 0.93361
 
 Not queried
 
  3.3051598
 
 5.71e-02
 
 1.00e+00
  
 
  
 15
 
  96887824
 
  96888024
 
  PDF   STATS   ENSEMBL   UCSC 
 
  201
 
  5
 
 14461
 
 island
 
 0.85707
 
 0.37094
 
 Not queried
 
  3.3049359
 
 1.51e-01
 
 1.00e+00
  
 
  
 3
 
 147112081
 
 147112605
 
  PDF   STATS   ENSEMBL   UCSC 
 
  525
 
  7
 
 27946
 
 gene;island
 
 0.02392
 
 0.18927
 
 Not queried
 
  3.3040815
 
 5.30e-02
 
 1.00e+00
  
 
  
 6
 
  10881782
 
  10882050
 
  PDF   STATS   ENSEMBL   UCSC 
 
  269
 
  6
 
 33503
 
 gene
 
 0.01316
 
 0.17357
 
 Not queried
 
  3.2988656
 
 4.11e-02
 
 1.00e+00
  
 
  
 21
 
  45622096
 
  45622493
 
  PDF   STATS   ENSEMBL   UCSC 
 
  398
 
  5
 
 26691
 
 island
 
 0.24483
 
 0.89514
 
 Not queried
 
  3.2961719
 
 7.94e-03
 
 1.00e+00
  
 
  
 5
 
 140734543
 
 140734758
 
  PDF   STATS   ENSEMBL   UCSC 
 
  216
 
  4
 
 32316
 
 tss
 
 0.25501
 
 0.79603
 
 Not queried
 
  3.2938719
 
 2.00e-01
 
 1.00e+00
  
 
  
 4
 
   3464653
 
   3465004
 
  PDF   STATS   ENSEMBL   UCSC 
 
  352
 
  9
 
 30215
 
 tss
 
 0.58417
 
 0.76126
 
 Not queried
 
  3.2937301
 
 9.39e-02
 
 1.00e+00
  
 
  
 10
 
 134683711
 
 134684094
 
  PDF   STATS   ENSEMBL   UCSC 
 
  384
 
  5
 
 6296
 
 island
 
 0.67461
 
 0.95686
 
 Not queried
 
  3.2915393
 
 7.94e-03
 
 1.00e+00
  
 
  
 12
 
 133022423
 
 133022955
 
  PDF   STATS   ENSEMBL   UCSC 
 
  533
 
  6
 
 11030
 
 island
 
 0.74056
 
 0.95931
 
 Not queried
 
  3.2895878
 
 2.16e-03
 
 1.00e+00
  
 
  
 8
 
 143858490
 
 143858821
 
  PDF   STATS   ENSEMBL   UCSC 
 
  332
 
  9
 
 39872
 
 tss
 
 0.36267
 
 0.02782
 
 Not queried
 
  3.2893985
 
 5.64e-03
 
 1.00e+00
  
 
  
 1
 
  91190005
 
  91190620
 
  PDF   STATS   ENSEMBL   UCSC 
 
  616
 
  7
 
 3454
 
 island
 
 0.37243
 
 0.04340
 
 Not queried
 
  3.2889524
 
 2.62e-02
 
 1.00e+00
  
 
  
 1
 
  27286944
 
  27287541
 
  PDF   STATS   ENSEMBL   UCSC 
 
  598
 
  6
 
 1398
 
 tss
 
 0.03070
 
 0.24352
 
 Not queried
 
  3.2872620
 
 1.32e-01
 
 1.00e+00
  
 
  
 17
 
  45772333
 
  45772523
 
  PDF   STATS   ENSEMBL   UCSC 
 
  191
 
  5
 
 17410
 
 tss
 
 0.29907
 
 0.02320
 
 Not queried
 
  3.2851139
 
 7.94e-03
 
 1.00e+00
  
 
  
 16
 
    450837
 
    451040
 
  PDF   STATS   ENSEMBL   UCSC 
 
  204
 
  4
 
 14934
 
 tss;island
 
 0.11356
 
 0.55482
 
 Not queried
 
  3.2850341
 
 2.86e-02
 
 1.00e+00
  
 
  
 20
 
  57414884
 
  57415177
 
  PDF   STATS   ENSEMBL   UCSC 
 
  294
 
  4
 
 25432
 
 gene;island
 
 0.59659
 
 0.93141
 
 Not queried
 
  3.2821957
 
 2.86e-02
 
 1.00e+00
  
 
  
 11
 
 119227592
 
 119227876
 
  PDF   STATS   ENSEMBL   UCSC 
 
  285
 
  4
 
 6919
 
 gene;island
 
 0.02974
 
 0.06837
 
 Not queried
 
  3.2808371
 
 3.43e-01
 
 1.00e+00
  
 
  
 1
 
  45187551
 
  45187946
 
  PDF   STATS   ENSEMBL   UCSC 
 
  396
 
  4
 
 493
 
 gene;island
 
 0.79882
 
 0.34468
 
 Not queried
 
  3.2798194
 
 2.86e-02
 
 1.00e+00
  
 
  
 5
 
  41509849
 
  41510116
 
  PDF   STATS   ENSEMBL   UCSC 
 
  268
 
  5
 
 31543
 
 gene;island
 
 0.11332
 
 0.55366
 
 Not queried
 
  3.2788409
 
 7.94e-03
 
 1.00e+00
  
 
  
 17
 
  48049952
 
  48049979
 
  PDF   STATS   ENSEMBL   UCSC 
 
   28
 
  4
 
 16642
 
 gene;tss;island
 
 0.74654
 
 0.22823
 
 Not queried
 
  3.2781287
 
 2.86e-02
 
 1.00e+00
  
 
  
 4
 
  46392253
 
  46392657
 
  PDF   STATS   ENSEMBL   UCSC 
 
  405
 
  6
 
 30326
 
 tss;island
 
 0.03657
 
 0.22957
 
 Not queried
 
  3.2776646
 
 1.52e-02
 
 1.00e+00
  
 
  
 19
 
  46999055
 
  46999444
 
  PDF   STATS   ENSEMBL   UCSC 
 
  390
 
 10
 
 22140
 
 island
 
 0.46091
 
 0.07378
 
 Not queried
 
  3.2771350
 
 1.05e-03
 
 1.00e+00
  
 
  
 5
 
  54516779
 
  54516879
 
  PDF   STATS   ENSEMBL   UCSC 
 
  101
 
  4
 
 32737
 
 island
 
 0.20026
 
 0.03711
 
 Not queried
 
  3.2754659
 
 2.86e-02
 
 1.00e+00
  
 
  
 7
 
  12610355
 
  12610833
 
  PDF   STATS   ENSEMBL   UCSC 
 
  479
 
  6
 
 36833
 
 gene
 
 0.02656
 
 0.23662
 
 Not queried
 
  3.2739719
 
 1.52e-02
 
 1.00e+00
  
 
  
 17
 
  37784694
 
  37784895
 
  PDF   STATS   ENSEMBL   UCSC 
 
  202
 
  4
 
 16514
 
 gene;island
 
 0.87685
 
 0.29826
 
 Not queried
 
  3.2727059
 
 2.00e-01
 
 1.00e+00
  
 
  
 17
 
  42081810
 
  42082036
 
  PDF   STATS   ENSEMBL   UCSC 
 
  227
 
  8
 
 17364
 
 tss;island
 
 0.42678
 
 0.04131
 
 Not queried
 
  3.2713508
 
 1.09e-03
 
 1.00e+00
  
 
  
 14
 
  47121033
 
  47121158
 
  PDF   STATS   ENSEMBL   UCSC 
 
  126
 
  5
 
 12347
 
 tss
 
 0.23955
 
 0.85985
 
 Not queried
 
  3.2709086
 
 7.94e-03
 
 1.00e+00
  
 
  
 19
 
  15121204
 
  15121596
 
  PDF   STATS   ENSEMBL   UCSC 
 
  393
 
  9
 
 21655
 
 island
 
 0.22208
 
 0.63215
 
 Not queried
 
  3.2700950
 
 1.23e-03
 
 1.00e+00
  
 
  
 18
 
  11751343
 
  11751566
 
  PDF   STATS   ENSEMBL   UCSC 
 
  224
 
  6
 
 19022
 
 gene;island
 
 0.37196
 
 0.84830
 
 Not queried
 
  3.2691828
 
 8.66e-03
 
 1.00e+00
  
 
  
 8
 
 146277200
 
 146277480
 
  PDF   STATS   ENSEMBL   UCSC 
 
  281
 
  4
 
 39935
 
 tss;island
 
 0.03021
 
 0.23386
 
 Not queried
 
  3.2689070
 
 2.86e-02
 
 1.00e+00
  
 
  
 16
 
    850240
 
    850713
 
  PDF   STATS   ENSEMBL   UCSC 
 
  474
 
  6
 
 14519
 
 gene
 
 0.03933
 
 0.33108
 
 Not queried
 
  3.2677303
 
 1.52e-02
 
 1.00e+00
  
 
  
 4
 
    380157
 
    380529
 
  PDF   STATS   ENSEMBL   UCSC 
 
  373
 
  4
 
 30669
 
 island
 
 0.64997
 
 0.15105
 
 Not queried
 
  3.2663537
 
 2.86e-02
 
 1.00e+00
  
 
  
 11
 
 107582804
 
 107582884
 
  PDF   STATS   ENSEMBL   UCSC 
 
   81
 
  4
 
 7642
 
 tss
 
 0.24728
 
 0.79992
 
 Not queried
 
  3.2655184
 
 3.43e-01
 
 1.00e+00
  
 
  
 17
 
  46657274
 
  46657656
 
  PDF   STATS   ENSEMBL   UCSC 
 
  383
 
  5
 
 18448
 
 island
 
 0.70182
 
 0.24905
 
 Not queried
 
  3.2643373
 
 9.52e-02
 
 1.00e+00
  
 
  
 19
 
  50706387
 
  50706874
 
  PDF   STATS   ENSEMBL   UCSC 
 
  488
 
  7
 
 20937
 
 tss;island
 
 0.03471
 
 0.29067
 
 Not queried
 
  3.2628231
 
 1.65e-01
 
 1.00e+00
  
 
  
 2
 
   3642098
 
   3642634
 
  PDF   STATS   ENSEMBL   UCSC 
 
  537
 
  6
 
 23125
 
 tss
 
 0.32426
 
 0.85675
 
 Not queried
 
  3.2627911
 
 1.52e-02
 
 1.00e+00
  
 
  
 17
 
  79495268
 
  79495348
 
  PDF   STATS   ENSEMBL   UCSC 
 
   81
 
  5
 
 17707
 
 tss
 
 0.28669
 
 0.76852
 
 Not queried
 
  3.2605608
 
 7.94e-03
 
 1.00e+00
  
 
  
 5
 
  78281793
 
  78282099
 
  PDF   STATS   ENSEMBL   UCSC 
 
  307
 
  5
 
 32084
 
 tss
 
 0.50758
 
 0.15007
 
 Not queried
 
  3.2602816
 
 1.59e-02
 
 1.00e+00
  
 
  
 6
 
  50808627
 
  50808813
 
  PDF   STATS   ENSEMBL   UCSC 
 
  187
 
  4
 
 34301
 
 gene;island
 
 0.68594
 
 0.16105
 
 Not queried
 
  3.2566186
 
 2.86e-02
 
 1.00e+00
  
 
  
 1
 
 150947442
 
 150947589
 
  PDF   STATS   ENSEMBL   UCSC 
 
  148
 
  4
 
 1911
 
 tss
 
 0.23967
 
 0.02871
 
 Not queried
 
  3.2539811
 
 2.00e-01
 
 1.00e+00
  
 
  
 1
 
    901685
 
    901799
 
  PDF   STATS   ENSEMBL   UCSC 
 
  115
 
  4
 
 1132
 
 tss
 
 0.07798
 
 0.46000
 
 Not queried
 
  3.2530009
 
 2.86e-02
 
 1.00e+00
  
 
  
 1
 
   6265802
 
   6266099
 
  PDF   STATS   ENSEMBL   UCSC 
 
  298
 
  6
 
 1198
 
 tss;island
 
 0.40243
 
 0.03249
 
 Not queried
 
  3.2512385
 
 8.66e-03
 
 1.00e+00
  
 
  
 4
 
 190861788
 
 190861959
 
  PDF   STATS   ENSEMBL   UCSC 
 
  172
 
  6
 
 30663
 
 tss
 
 0.02771
 
 0.23560
 
 Not queried
 
  3.2492247
 
 1.52e-02
 
 1.00e+00
  
 
  
 10
 
  11783794
 
  11784253
 
  PDF   STATS   ENSEMBL   UCSC 
 
  460
 
  8
 
 4929
 
 tss
 
 0.13076
 
 0.63592
 
 Not queried
 
  3.2485030
 
 6.99e-03
 
 1.00e+00
  
 
  
 6
 
 127796989
 
 127797286
 
  PDF   STATS   ENSEMBL   UCSC 
 
  298
 
  4
 
 34405
 
 gene;island
 
 0.39125
 
 0.86454
 
 Not queried
 
  3.2484777
 
 2.86e-02
 
 1.00e+00
  
 
  
 8
 
 141468467
 
 141468933
 
  PDF   STATS   ENSEMBL   UCSC 
 
  467
 
  7
 
 39861
 
 tss;island
 
 0.88016
 
 0.25057
 
 Not queried
 
  3.2456905
 
 3.79e-02
 
 1.00e+00
  
 
  
 6
 
  31105337
 
  31106302
 
  PDF   STATS   ENSEMBL   UCSC 
 
  966
 
 15
 
 33791
 
 gene
 
 0.29635
 
 0.85954
 
 Not queried
 
  3.2444872
 
 2.70e-03
 
 1.00e+00
  
 
  
 11
 
 132948042
 
 132948244
 
  PDF   STATS   ENSEMBL   UCSC 
 
  203
 
  4
 
 6964
 
 gene;island
 
 0.10641
 
 0.52827
 
 Not queried
 
  3.2410412
 
 5.71e-02
 
 1.00e+00
  
 
  
 3
 
  42846907
 
  42847116
 
  PDF   STATS   ENSEMBL   UCSC 
 
  210
 
  4
 
 28207
 
 tss;island
 
 0.38812
 
 0.04245
 
 Not queried
 
  3.2409004
 
 1.14e-01
 
 1.00e+00
  
 
  
 12
 
   9800259
 
   9800619
 
  PDF   STATS   ENSEMBL   UCSC 
 
  361
 
  9
 
 9533
 
 tss
 
 0.07606
 
 0.03044
 
 Not queried
 
  3.2399960
 
 7.77e-03
 
 1.00e+00
  
 
  
 4
 
 113332969
 
 113333269
 
  PDF   STATS   ENSEMBL   UCSC 
 
  301
 
  6
 
 30052
 
 gene;island
 
 0.04066
 
 0.24309
 
 Not queried
 
  3.2382725
 
 8.66e-03
 
 1.00e+00
  
 
  
 11
 
  89224684
 
  89224799
 
  PDF   STATS   ENSEMBL   UCSC 
 
  116
 
  6
 
 7593
 
 tss
 
 0.22483
 
 0.03941
 
 Not queried
 
  3.2379082
 
 2.16e-03
 
 1.00e+00
  
 
  
 3
 
  46734995
 
  46735154
 
  PDF   STATS   ENSEMBL   UCSC 
 
  160
 
  4
 
 27711
 
 gene
 
 0.02312
 
 0.20158
 
 Not queried
 
  3.2371341
 
 2.86e-02
 
 1.00e+00
  
 
  
 10
 
 127584967
 
 127585144
 
  PDF   STATS   ENSEMBL   UCSC 
 
  178
 
  5
 
 6221
 
 island
 
 0.06693
 
 0.50699
 
 Not queried
 
  3.2364593
 
 5.56e-02
 
 1.00e+00
  
 
  
 10
 
  97889373
 
  97889448
 
  PDF   STATS   ENSEMBL   UCSC 
 
   76
 
  4
 
 5236
 
 tss
 
 0.82150
 
 0.32621
 
 Not queried
 
  3.2340944
 
 2.00e-01
 
 1.00e+00
  
 
  
 17
 
  33416834
 
  33417113
 
  PDF   STATS   ENSEMBL   UCSC 
 
  280
 
  5
 
 17231
 
 tss;island
 
 0.05203
 
 0.62149
 
 Not queried
 
  3.2304842
 
 1.51e-01
 
 1.00e+00
  
 
  
 16
 
  28891176
 
  28891926
 
  PDF   STATS   ENSEMBL   UCSC 
 
  751
 
  6
 
 14676
 
 gene;island
 
 0.57684
 
 0.27549
 
 Not queried
 
  3.2289840
 
 2.40e-01
 
 1.00e+00
  
 
  
 6
 
 147124881
 
 147124996
 
  PDF   STATS   ENSEMBL   UCSC 
 
  116
 
  6
 
 34436
 
 gene
 
 0.42393
 
 0.78824
 
 Not queried
 
  3.2286681
 
 1.32e-01
 
 1.00e+00
  
 
  
 5
 
   7826845
 
   7827133
 
  PDF   STATS   ENSEMBL   UCSC 
 
  289
 
  5
 
 31499
 
 gene
 
 0.29788
 
 0.84269
 
 Not queried
 
  3.2277496
 
 7.94e-03
 
 1.00e+00
  
 
  
 X
 
  73513412
 
  73513464
 
  PDF   STATS   ENSEMBL   UCSC 
 
   53
 
  4
 
 41391
 
 tss;island
 
 0.13341
 
 0.37235
 
 Not queried
 
  3.2268200
 
 5.71e-02
 
 1.00e+00
  
 
  
 6
 
  32489553
 
  32489991
 
  PDF   STATS   ENSEMBL   UCSC 
 
  439
 
  6
 
 34053
 
 gene;island
 
 0.15358
 
 0.54148
 
 Not queried
 
  3.2238937
 
 9.31e-02
 
 1.00e+00
  
 
  
 17
 
  81042782
 
  81043176
 
  PDF   STATS   ENSEMBL   UCSC 
 
  395
 
  4
 
 16942
 
 gene;island
 
 0.47242
 
 0.88909
 
 Not queried
 
  3.2211824
 
 2.86e-02
 
 1.00e+00
  
 
  
 3
 
 142666108
 
 142666476
 
  PDF   STATS   ENSEMBL   UCSC 
 
  369
 
  4
 
 29512
 
 island
 
 0.69044
 
 0.94562
 
 Not queried
 
  3.2210238
 
 2.86e-02
 
 1.00e+00
  
 
  
 11
 
  63827695
 
  63828346
 
  PDF   STATS   ENSEMBL   UCSC 
 
  652
 
  5
 
 6642
 
 gene
 
 0.81477
 
 0.31593
 
 Not queried
 
  3.2208898
 
 1.51e-01
 
 1.00e+00
  
 
  
 12
 
  99548329
 
  99548550
 
  PDF   STATS   ENSEMBL   UCSC 
 
  222
 
  5
 
 9237
 
 gene
 
 0.14010
 
 0.58289
 
 Not queried
 
  3.2180602
 
 7.94e-03
 
 1.00e+00
  
 
  
 20
 
  62318426
 
  62318588
 
  PDF   STATS   ENSEMBL   UCSC 
 
  163
 
  4
 
 25468
 
 gene;island
 
 0.43614
 
 0.89482
 
 Not queried
 
  3.2172675
 
 2.86e-02
 
 1.00e+00
  
 
  
 15
 
  45421578
 
  45422054
 
  PDF   STATS   ENSEMBL   UCSC 
 
  477
 
  8
 
 13298
 
 gene
 
 0.11647
 
 0.52224
 
 Not queried
 
  3.2167501
 
 4.66e-03
 
 1.00e+00
  
 
  
 6
 
  34024220
 
  34024479
 
  PDF   STATS   ENSEMBL   UCSC 
 
  260
 
  4
 
 34236
 
 gene;island
 
 0.13839
 
 0.58637
 
 Not queried
 
  3.2163449
 
 5.71e-02
 
 1.00e+00
  
 
  
 13
 
 112758379
 
 112758625
 
  PDF   STATS   ENSEMBL   UCSC 
 
  247
 
  4
 
 11885
 
 island
 
 0.24698
 
 0.03652
 
 Not queried
 
  3.2149800
 
 2.86e-02
 
 1.00e+00
  
 
  
 15
 
  91208370
 
  91208927
 
  PDF   STATS   ENSEMBL   UCSC 
 
  558
 
  5
 
 14428
 
 island
 
 0.63841
 
 0.82838
 
 Not queried
 
  3.2135350
 
 5.56e-02
 
 1.00e+00
  
 
  
 10
 
 129534107
 
 129534411
 
  PDF   STATS   ENSEMBL   UCSC 
 
  305
 
  4
 
 5417
 
 tss
 
 0.09263
 
 0.47318
 
 Not queried
 
  3.2131107
 
 1.14e-01
 
 1.00e+00
  
 
  
 2
 
 105484357
 
 105484823
 
  PDF   STATS   ENSEMBL   UCSC 
 
  467
 
  4
 
 24542
 
 island
 
 0.73019
 
 0.25659
 
 Not queried
 
  3.2121189
 
 2.86e-02
 
 1.00e+00
  
 
  
 16
 
    809910
 
    810292
 
  PDF   STATS   ENSEMBL   UCSC 
 
  383
 
  5
 
 14950
 
 tss
 
 0.36443
 
 0.86269
 
 Not queried
 
  3.2116017
 
 7.94e-03
 
 1.00e+00
  
 
  
 5
 
  37834672
 
  37835168
 
  PDF   STATS   ENSEMBL   UCSC 
 
  497
 
  7
 
 31534
 
 gene;island
 
 0.11056
 
 0.04859
 
 Not queried
 
  3.2079423
 
 5.83e-04
 
 1.00e+00
  
 
  
 5
 
  37834909
 
  37835168
 
  PDF   STATS   ENSEMBL   UCSC 
 
  260
 
  5
 
 31966
 
 tss
 
 0.19578
 
 0.06467
 
 Not queried
 
  3.2079423
 
 7.94e-03
 
 1.00e+00
  
 
  
 2
 
 176948693
 
 176949017
 
  PDF   STATS   ENSEMBL   UCSC 
 
  325
 
  7
 
 23693
 
 tss
 
 0.04339
 
 0.31878
 
 Not queried
 
  3.2077759
 
 1.11e-02
 
 1.00e+00
  
 
  
 6
 
  27840957
 
  27841230
 
  PDF   STATS   ENSEMBL   UCSC 
 
  274
 
  5
 
 33560
 
 gene
 
 0.45336
 
 0.90027
 
 Not queried
 
  3.2074937
 
 7.94e-03
 
 1.00e+00
  
 
  
 5
 
 140778342
 
 140778546
 
  PDF   STATS   ENSEMBL   UCSC 
 
  205
 
  4
 
 31773
 
 gene;island
 
 0.63566
 
 0.94164
 
 Not queried
 
  3.2071542
 
 5.71e-02
 
 1.00e+00
  
 
  
 15
 
  79574773
 
  79575048
 
  PDF   STATS   ENSEMBL   UCSC 
 
  276
 
  5
 
 13815
 
 tss;island
 
 0.04042
 
 0.27987
 
 Not queried
 
  3.2056308
 
 5.56e-02
 
 1.00e+00
  
 
  
 15
 
  42447840
 
  42448259
 
  PDF   STATS   ENSEMBL   UCSC 
 
  420
 
  5
 
 13279
 
 gene
 
 0.47171
 
 0.90671
 
 Not queried
 
  3.2049284
 
 3.17e-02
 
 1.00e+00
  
 
  
 6
 
  29571363
 
  29571483
 
  PDF   STATS   ENSEMBL   UCSC 
 
  121
 
  7
 
 33601
 
 gene
 
 0.73646
 
 0.94443
 
 Not queried
 
  3.2045401
 
 4.08e-03
 
 1.00e+00
  
 
  
 22
 
  50523790
 
  50524124
 
  PDF   STATS   ENSEMBL   UCSC 
 
  335
 
  4
 
 27202
 
 tss
 
 0.12856
 
 0.59126
 
 Not queried
 
  3.2037939
 
 2.86e-02
 
 1.00e+00
  
 
  
 19
 
  58951434
 
  58951885
 
  PDF   STATS   ENSEMBL   UCSC 
 
  452
 
  9
 
 22472
 
 island
 
 0.18437
 
 0.01689
 
 Not queried
 
  3.2016271
 
 2.88e-04
 
 1.00e+00
  
 
  
 11
 
 134262988
 
 134263349
 
  PDF   STATS   ENSEMBL   UCSC 
 
  362
 
  5
 
 6981
 
 gene
 
 0.43062
 
 0.87427
 
 Not queried
 
  3.2007518
 
 1.59e-02
 
 1.00e+00
  
 
  
 Y
 
  21728952
 
  21729314
 
  PDF   STATS   ENSEMBL   UCSC 
 
  363
 
  8
 
 42183
 
 island
 
 0.01285
 
 0.20272
 
 Not queried
 
  3.1988900
 
 2.34e-01
 
 1.00e+00
  
 
  
 4
 
   1027885
 
   1028067
 
  PDF   STATS   ENSEMBL   UCSC 
 
  183
 
  4
 
 30712
 
 island
 
 0.61013
 
 0.92883
 
 Not queried
 
  3.1961457
 
 1.14e-01
 
 1.00e+00
  
 
  
 4
 
   1243849
 
   1244086
 
  PDF   STATS   ENSEMBL   UCSC 
 
  238
 
  7
 
 30193
 
 tss;island
 
 0.88673
 
 0.42200
 
 Not queried
 
  3.1936888
 
 5.83e-04
 
 1.00e+00
  
 
  
 10
 
 129845375
 
 129846082
 
  PDF   STATS   ENSEMBL   UCSC 
 
  708
 
  8
 
 4806
 
 gene
 
 0.14132
 
 0.61280
 
 Not queried
 
  3.1904594
 
 2.95e-03
 
 1.00e+00
  
 
  
 10
 
 129845375
 
 129845766
 
  PDF   STATS   ENSEMBL   UCSC 
 
  392
 
  6
 
 5420
 
 tss
 
 0.14132
 
 0.59485
 
 Not queried
 
  3.1904594
 
 2.16e-03
 
 1.00e+00
  
 
  
 10
 
  88718164
 
  88718393
 
  PDF   STATS   ENSEMBL   UCSC 
 
  230
 
  4
 
 5184
 
 tss
 
 0.35672
 
 0.88102
 
 Not queried
 
  3.1877029
 
 5.71e-02
 
 1.00e+00
  
 
  
 2
 
 220299484
 
 220299672
 
  PDF   STATS   ENSEMBL   UCSC 
 
  189
 
  7
 
 23868
 
 tss
 
 0.32963
 
 0.81484
 
 Not queried
 
  3.1873779
 
 5.83e-04
 
 1.00e+00
  
 
  
 5
 
 101834365
 
 101834780
 
  PDF   STATS   ENSEMBL   UCSC 
 
  416
 
  5
 
 32924
 
 island
 
 0.22835
 
 0.72909
 
 Not queried
 
  3.1849363
 
 1.59e-02
 
 1.00e+00
  
 
  
 15
 
  96875591
 
  96875830
 
  PDF   STATS   ENSEMBL   UCSC 
 
  240
 
  8
 
 13464
 
 gene;tss;island
 
 0.02136
 
 0.21824
 
 Not queried
 
  3.1844592
 
 1.48e-02
 
 1.00e+00
  
 
  
 13
 
  28503060
 
  28503508
 
  PDF   STATS   ENSEMBL   UCSC 
 
  449
 
  4
 
 11606
 
 island
 
 0.24692
 
 0.73305
 
 Not queried
 
  3.1839295
 
 2.86e-02
 
 1.00e+00
  
 
  
 11
 
  58672702
 
  58673167
 
  PDF   STATS   ENSEMBL   UCSC 
 
  466
 
  5
 
 8275
 
 island
 
 0.04412
 
 0.29507
 
 Not queried
 
  3.1809917
 
 9.52e-02
 
 1.00e+00
  
 
  
 1
 
  47915553
 
  47915951
 
  PDF   STATS   ENSEMBL   UCSC 
 
  399
 
  6
 
 3234
 
 island
 
 0.47396
 
 0.07591
 
 Not queried
 
  3.1797142
 
 4.11e-02
 
 1.00e+00
  
 
  
 12
 
  49726500
 
  49726893
 
  PDF   STATS   ENSEMBL   UCSC 
 
  394
 
  4
 
 9093
 
 gene;island
 
 0.43145
 
 0.84377
 
 Not queried
 
  3.1777402
 
 2.86e-02
 
 1.00e+00
  
 
  
 3
 
 196366191
 
 196366618
 
  PDF   STATS   ENSEMBL   UCSC 
 
  428
 
  7
 
 28773
 
 tss
 
 0.28115
 
 0.77152
 
 Not queried
 
  3.1762661
 
 5.83e-04
 
 1.00e+00
  
 
  
 14
 
  24801616
 
  24802150
 
  PDF   STATS   ENSEMBL   UCSC 
 
  535
 
  4
 
 12018
 
 gene;island
 
 0.06584
 
 0.47200
 
 Not queried
 
  3.1754603
 
 2.86e-02
 
 1.00e+00
  
 
  
 17
 
   7826155
 
   7826413
 
  PDF   STATS   ENSEMBL   UCSC 
 
  259
 
  5
 
 16385
 
 gene;island
 
 0.02403
 
 0.31929
 
 Not queried
 
  3.1742375
 
 3.17e-02
 
 1.00e+00
  
 
  
 4
 
   1407592
 
   1407989
 
  PDF   STATS   ENSEMBL   UCSC 
 
  398
 
  4
 
 30737
 
 island
 
 0.36151
 
 0.86053
 
 Not queried
 
  3.1741875
 
 3.43e-01
 
 1.00e+00
  
 
  
 19
 
  36450121
 
  36450528
 
  PDF   STATS   ENSEMBL   UCSC 
 
  408
 
  4
 
 21894
 
 island
 
 0.04147
 
 0.33172
 
 Not queried
 
  3.1741525
 
 2.86e-02
 
 1.00e+00
  
 
  
 11
 
  15136059
 
  15136475
 
  PDF   STATS   ENSEMBL   UCSC 
 
  417
 
  7
 
 7149
 
 tss
 
 0.02462
 
 0.25368
 
 Not queried
 
  3.1729306
 
 1.17e-03
 
 1.00e+00
  
 
  
 12
 
  71833017
 
  71833193
 
  PDF   STATS   ENSEMBL   UCSC 
 
  177
 
  4
 
 9846
 
 tss;island
 
 0.02288
 
 0.16491
 
 Not queried
 
  3.1705314
 
 2.86e-02
 
 1.00e+00
  
 
  
 4
 
   1107495
 
   1107720
 
  PDF   STATS   ENSEMBL   UCSC 
 
  226
 
  5
 
 30719
 
 island
 
 0.32157
 
 0.92019
 
 Not queried
 
  3.1681104
 
 5.56e-02
 
 1.00e+00
  
 
  
 19
 
  54926277
 
  54926617
 
  PDF   STATS   ENSEMBL   UCSC 
 
  341
 
  5
 
 21025
 
 tss
 
 0.04153
 
 0.28555
 
 Not queried
 
  3.1660981
 
 5.56e-02
 
 1.00e+00
  
 
  
 5
 
  66124395
 
  66124801
 
  PDF   STATS   ENSEMBL   UCSC 
 
  407
 
  7
 
 31579
 
 gene
 
 0.03324
 
 0.24872
 
 Not queried
 
  3.1660251
 
 6.99e-03
 
 1.00e+00
  
 
  
 16
 
   2198395
 
   2198504
 
  PDF   STATS   ENSEMBL   UCSC 
 
  110
 
  4
 
 14987
 
 tss;island
 
 0.52054
 
 0.03573
 
 Not queried
 
  3.1647872
 
 5.71e-02
 
 1.00e+00
  
 
  
 11
 
 111170390
 
 111170648
 
  PDF   STATS   ENSEMBL   UCSC 
 
  259
 
  5
 
 6880
 
 gene;island
 
 0.44513
 
 0.06316
 
 Not queried
 
  3.1635760
 
 3.17e-02
 
 1.00e+00
  
 
  
 12
 
 113229582
 
 113230065
 
  PDF   STATS   ENSEMBL   UCSC 
 
  484
 
  8
 
 9291
 
 gene
 
 0.31180
 
 0.69037
 
 Not queried
 
  3.1629900
 
 6.99e-03
 
 1.00e+00
  
 
  
 8
 
  43131260
 
  43131656
 
  PDF   STATS   ENSEMBL   UCSC 
 
  397
 
  5
 
 40202
 
 island
 
 0.22730
 
 0.76882
 
 Not queried
 
  3.1607312
 
 1.51e-01
 
 1.00e+00
  
 
  
 X
 
  70128591
 
  70128670
 
  PDF   STATS   ENSEMBL   UCSC 
 
   80
 
  6
 
 41369
 
 tss
 
 0.58822
 
 0.89065
 
 Not queried
 
  3.1589160
 
 4.11e-02
 
 1.00e+00
  
 
  
 7
 
  89748633
 
  89748810
 
  PDF   STATS   ENSEMBL   UCSC 
 
  178
 
  7
 
 38580
 
 island
 
 0.06047
 
 0.32246
 
 Not queried
 
  3.1583261
 
 5.83e-04
 
 1.00e+00
  
 
  
 6
 
  34164073
 
  34164564
 
  PDF   STATS   ENSEMBL   UCSC 
 
  492
 
  6
 
 35990
 
 island
 
 0.07720
 
 0.41967
 
 Not queried
 
  3.1565023
 
 3.10e-01
 
 1.00e+00
  
 
  
 17
 
  21367002
 
  21367564
 
  PDF   STATS   ENSEMBL   UCSC 
 
  563
 
  5
 
 18089
 
 island
 
 0.33108
 
 0.81519
 
 Not queried
 
  3.1557657
 
 2.22e-01
 
 1.00e+00
  
 
  
 1
 
  33231070
 
  33231382
 
  PDF   STATS   ENSEMBL   UCSC 
 
  313
 
  8
 
 398
 
 gene
 
 0.68794
 
 0.07372
 
 Not queried
 
  3.1549042
 
 1.04e-02
 
 1.00e+00
  
 
  
 5
 
 140261653
 
 140261810
 
  PDF   STATS   ENSEMBL   UCSC 
 
  158
 
  6
 
 32297
 
 tss
 
 0.05980
 
 0.36902
 
 Not queried
 
  3.1535252
 
 4.33e-03
 
 1.00e+00
  
 
  
 3
 
 183993368
 
 183993726
 
  PDF   STATS   ENSEMBL   UCSC 
 
  359
 
  7
 
 28714
 
 tss
 
 0.50113
 
 0.85795
 
 Not queried
 
  3.1530477
 
 9.73e-02
 
 1.00e+00
  
 
  
 6
 
 142409417
 
 142409831
 
  PDF   STATS   ENSEMBL   UCSC 
 
  415
 
  5
 
 34428
 
 gene
 
 0.03230
 
 0.19875
 
 Not queried
 
  3.1522680
 
 9.52e-02
 
 1.00e+00
  
 
  
 2
 
 102090791
 
 102091129
 
  PDF   STATS   ENSEMBL   UCSC 
 
  339
 
  5
 
 22774
 
 gene
 
 0.02832
 
 0.26616
 
 Not queried
 
  3.1522375
 
 7.94e-03
 
 1.00e+00
  
 
  
 3
 
  69915189
 
  69915449
 
  PDF   STATS   ENSEMBL   UCSC 
 
  261
 
  4
 
 27814
 
 gene
 
 0.49535
 
 0.84383
 
 Not queried
 
  3.1519553
 
 1.14e-01
 
 1.00e+00
  
 
  
 19
 
  14183018
 
  14183212
 
  PDF   STATS   ENSEMBL   UCSC 
 
  195
 
  4
 
 20500
 
 tss;island
 
 0.49613
 
 0.08660
 
 Not queried
 
  3.1495471
 
 5.71e-02
 
 1.00e+00
  
 
  
 4
 
  90031538
 
  90031969
 
  PDF   STATS   ENSEMBL   UCSC 
 
  432
 
  4
 
 31124
 
 island
 
 0.01608
 
 0.08153
 
 Not queried
 
  3.1468941
 
 3.43e-01
 
 1.00e+00
  
 
  
 X
 
 153745603
 
 153745887
 
  PDF   STATS   ENSEMBL   UCSC 
 
  285
 
  4
 
 41605
 
 tss;island
 
 0.31965
 
 0.04226
 
 Not queried
 
  3.1456955
 
 2.86e-02
 
 1.00e+00
  
 
  
 1
 
 113261711
 
 113261894
 
  PDF   STATS   ENSEMBL   UCSC 
 
  184
 
  4
 
 1837
 
 tss
 
 0.17080
 
 0.55955
 
 Not queried
 
  3.1437384
 
 1.14e-01
 
 1.00e+00
  
 
  
 17
 
  19483367
 
  19483721
 
  PDF   STATS   ENSEMBL   UCSC 
 
  355
 
  5
 
 18067
 
 island
 
 0.05240
 
 0.30872
 
 Not queried
 
  3.1389950
 
 7.94e-03
 
 1.00e+00
  
 
  
 13
 
 110918331
 
 110918683
 
  PDF   STATS   ENSEMBL   UCSC 
 
  353
 
  4
 
 11198
 
 gene;island
 
 0.59585
 
 0.89665
 
 Not queried
 
  3.1382019
 
 2.86e-02
 
 1.00e+00
  
 
  
 8
 
 143858414
 
 143858821
 
  PDF   STATS   ENSEMBL   UCSC 
 
  408
 
 10
 
 39405
 
 gene;island
 
 0.37182
 
 0.04745
 
 Not queried
 
  3.1380173
 
 3.89e-03
 
 1.00e+00
  
 
  
 2
 
  65593761
 
  65594021
 
  PDF   STATS   ENSEMBL   UCSC 
 
  261
 
  4
 
 22672
 
 gene
 
 0.78370
 
 0.96659
 
 Not queried
 
  3.1374193
 
 1.14e-01
 
 1.00e+00
  
 
  
 16
 
  84346722
 
  84346930
 
  PDF   STATS   ENSEMBL   UCSC 
 
  209
 
  4
 
 14837
 
 gene;island
 
 0.20125
 
 0.69057
 
 Not queried
 
  3.1356294
 
 2.86e-02
 
 1.00e+00
  
 
  
 7
 
 150706128
 
 150706355
 
  PDF   STATS   ENSEMBL   UCSC 
 
  228
 
  4
 
 37218
 
 gene;island
 
 0.22743
 
 0.62892
 
 Not queried
 
  3.1353269
 
 2.00e-01
 
 1.00e+00
  
 
  
 19
 
  28296844
 
  28297092
 
  PDF   STATS   ENSEMBL   UCSC 
 
  249
 
  4
 
 21816
 
 island
 
 0.41466
 
 0.87352
 
 Not queried
 
  3.1352934
 
 2.86e-02
 
 1.00e+00
  
 
  
 13
 
 112895431
 
 112895794
 
  PDF   STATS   ENSEMBL   UCSC 
 
  364
 
  5
 
 11888
 
 island
 
 0.28780
 
 0.69960
 
 Not queried
 
  3.1336683
 
 9.52e-02
 
 1.00e+00
  
 
  
 22
 
  28198218
 
  28198591
 
  PDF   STATS   ENSEMBL   UCSC 
 
  374
 
  4
 
 26996
 
 tss;island
 
 0.25600
 
 0.03305
 
 Not queried
 
  3.1321991
 
 2.00e-01
 
 1.00e+00
  
 
  
 2
 
    468737
 
    469214
 
  PDF   STATS   ENSEMBL   UCSC 
 
  478
 
  5
 
 23965
 
 island
 
 0.70802
 
 0.96446
 
 Not queried
 
  3.1294241
 
 7.94e-03
 
 1.00e+00
  
 
  
 8
 
    496327
 
    496739
 
  PDF   STATS   ENSEMBL   UCSC 
 
  413
 
  5
 
 39470
 
 tss;island
 
 0.53046
 
 0.10830
 
 Not queried
 
  3.1285708
 
 1.51e-01
 
 1.00e+00
  
 
  
 16
 
  88052922
 
  88053104
 
  PDF   STATS   ENSEMBL   UCSC 
 
  183
 
  4
 
 14867
 
 gene;island
 
 0.38900
 
 0.84857
 
 Not queried
 
  3.1277315
 
 2.86e-02
 
 1.00e+00
  
 
  
 16
 
   4714647
 
   4714815
 
  PDF   STATS   ENSEMBL   UCSC 
 
  169
 
  4
 
 14613
 
 gene
 
 0.48912
 
 0.91510
 
 Not queried
 
  3.1272805
 
 2.86e-02
 
 1.00e+00
  
 
  
 15
 
  68723690
 
  68724065
 
  PDF   STATS   ENSEMBL   UCSC 
 
  376
 
  4
 
 13367
 
 gene;island
 
 0.05050
 
 0.48857
 
 Not queried
 
  3.1265726
 
 5.71e-02
 
 1.00e+00
  
 
  
 16
 
  71659830
 
  71660113
 
  PDF   STATS   ENSEMBL   UCSC 
 
  284
 
  7
 
 16044
 
 island
 
 0.02788
 
 0.16421
 
 Not queried
 
  3.1264037
 
 7.28e-02
 
 1.00e+00
  
 
  
 19
 
  46999224
 
  46999444
 
  PDF   STATS   ENSEMBL   UCSC 
 
  221
 
  7
 
 20857
 
 tss
 
 0.44690
 
 0.09184
 
 Not queried
 
  3.1263182
 
 1.75e-02
 
 1.00e+00
  
 
  
 X
 
  73834484
 
  73834821
 
  PDF   STATS   ENSEMBL   UCSC 
 
  338
 
  6
 
 41393
 
 tss
 
 0.18983
 
 0.62595
 
 Not queried
 
  3.1244421
 
 2.16e-03
 
 1.00e+00
  
 
  
 2
 
 200334402
 
 200334584
 
  PDF   STATS   ENSEMBL   UCSC 
 
  183
 
  4
 
 22938
 
 gene;island
 
 0.20226
 
 0.74409
 
 Not queried
 
  3.1223558
 
 2.86e-02
 
 1.00e+00
  
 
  
 11
 
  64108241
 
  64108550
 
  PDF   STATS   ENSEMBL   UCSC 
 
  310
 
  4
 
 6664
 
 gene;island
 
 0.36507
 
 0.06187
 
 Not queried
 
  3.1218556
 
 2.00e-01
 
 1.00e+00
  
 
  
 17
 
  81047627
 
  81047941
 
  PDF   STATS   ENSEMBL   UCSC 
 
  315
 
  4
 
 16943
 
 gene;island
 
 0.38972
 
 0.82782
 
 Not queried
 
  3.1217092
 
 2.86e-02
 
 1.00e+00
  
 
  
 11
 
  71752705
 
  71752997
 
  PDF   STATS   ENSEMBL   UCSC 
 
  293
 
  5
 
 6790
 
 gene
 
 0.84440
 
 0.31457
 
 Not queried
 
  3.1194470
 
 5.56e-02
 
 1.00e+00
  
 
  
 16
 
  85670135
 
  85670348
 
  PDF   STATS   ENSEMBL   UCSC 
 
  214
 
  4
 
 14846
 
 gene;island
 
 0.67066
 
 0.94372
 
 Not queried
 
  3.1192490
 
 2.86e-02
 
 1.00e+00
  
 
  
 18
 
  61143926
 
  61143993
 
  PDF   STATS   ENSEMBL   UCSC 
 
   68
 
  5
 
 19278
 
 tss
 
 0.41192
 
 0.85952
 
 Not queried
 
  3.1182697
 
 7.94e-03
 
 1.00e+00
  
 
  
 11
 
  67373625
 
  67374388
 
  PDF   STATS   ENSEMBL   UCSC 
 
  764
 
  9
 
 8536
 
 island
 
 0.49671
 
 0.07308
 
 Not queried
 
  3.1173619
 
 9.39e-02
 
 1.00e+00
  
 
  
 5
 
    139839
 
    140289
 
  PDF   STATS   ENSEMBL   UCSC 
 
  451
 
  7
 
 31894
 
 tss
 
 0.69151
 
 0.88364
 
 Not queried
 
  3.1117036
 
 2.62e-02
 
 1.00e+00
  
 
  
 17
 
  72269945
 
  72270201
 
  PDF   STATS   ENSEMBL   UCSC 
 
  257
 
  5
 
 17589
 
 tss;island
 
 0.23247
 
 0.68935
 
 Not queried
 
  3.1108798
 
 7.94e-03
 
 1.00e+00
  
 
  
 19
 
  49944817
 
  49944964
 
  PDF   STATS   ENSEMBL   UCSC 
 
  148
 
  5
 
 20909
 
 tss
 
 0.03615
 
 0.24260
 
 Not queried
 
  3.1106483
 
 7.94e-03
 
 1.00e+00
  
 
  
 10
 
   4868328
 
   4868690
 
  PDF   STATS   ENSEMBL   UCSC 
 
  363
 
  8
 
 5498
 
 island
 
 0.09674
 
 0.49439
 
 Not queried
 
  3.1099916
 
 3.11e-04
 
 1.00e+00
  
 
  
 5
 
 140709983
 
 140710237
 
  PDF   STATS   ENSEMBL   UCSC 
 
  255
 
  4
 
 32313
 
 tss;island
 
 0.03449
 
 0.33385
 
 Not queried
 
  3.1098343
 
 5.71e-02
 
 1.00e+00
  
 
  
 3
 
  50388530
 
  50388670
 
  PDF   STATS   ENSEMBL   UCSC 
 
  141
 
  6
 
 28314
 
 tss
 
 0.14253
 
 0.02454
 
 Not queried
 
  3.1098086
 
 2.16e-03
 
 1.00e+00
  
 
  
 10
 
  18549378
 
  18549778
 
  PDF   STATS   ENSEMBL   UCSC 
 
  401
 
  7
 
 4508
 
 gene
 
 0.18944
 
 0.70510
 
 Not queried
 
  3.1095889
 
 1.75e-02
 
 1.00e+00
  
 
  
 5
 
  76939333
 
  76939558
 
  PDF   STATS   ENSEMBL   UCSC 
 
  226
 
  4
 
 32841
 
 island
 
 0.71088
 
 0.13265
 
 Not queried
 
  3.1065041
 
 2.86e-02
 
 1.00e+00
  
 
  
 12
 
   7167584
 
   7167900
 
  PDF   STATS   ENSEMBL   UCSC 
 
  317
 
  4
 
 9518
 
 tss
 
 0.13881
 
 0.56152
 
 Not queried
 
  3.1064903
 
 2.00e-01
 
 1.00e+00
  
 
  
 1
 
  41350014
 
  41350406
 
  PDF   STATS   ENSEMBL   UCSC 
 
  393
 
  5
 
 3126
 
 island
 
 0.21204
 
 0.71883
 
 Not queried
 
  3.1059011
 
 1.59e-02
 
 1.00e+00
  
 
  
 10
 
 129533998
 
 129534411
 
  PDF   STATS   ENSEMBL   UCSC 
 
  414
 
  5
 
 6229
 
 island
 
 0.12351
 
 0.58517
 
 Not queried
 
  3.1027786
 
 1.51e-01
 
 1.00e+00
  
 
  
 1
 
 214153294
 
 214153472
 
  PDF   STATS   ENSEMBL   UCSC 
 
  179
 
  4
 
 4181
 
 island
 
 0.44044
 
 0.08455
 
 Not queried
 
  3.1024274
 
 2.86e-02
 
 1.00e+00
  
 
  
 14
 
  89028798
 
  89029224
 
  PDF   STATS   ENSEMBL   UCSC 
 
  427
 
  6
 
 12517
 
 tss;island
 
 0.13492
 
 0.13607
 
 Not queried
 
  3.1024194
 
 5.89e-01
 
 1.00e+00
  
 
  
 2
 
  45237689
 
  45237929
 
  PDF   STATS   ENSEMBL   UCSC 
 
  241
 
  4
 
 23297
 
 tss;island
 
 0.41588
 
 0.12915
 
 Not queried
 
  3.1020785
 
 2.86e-02
 
 1.00e+00
  
 
  
 X
 
  54556668
 
  54557002
 
  PDF   STATS   ENSEMBL   UCSC 
 
  335
 
  6
 
 40998
 
 gene
 
 0.25452
 
 0.03520
 
 Not queried
 
  3.1020098
 
 4.33e-03
 
 1.00e+00
  
 
  
 12
 
  91572142
 
  91572276
 
  PDF   STATS   ENSEMBL   UCSC 
 
  135
 
  4
 
 9216
 
 gene
 
 0.10616
 
 0.56251
 
 Not queried
 
  3.1019976
 
 1.14e-01
 
 1.00e+00
  
 
  
 16
 
  34777682
 
  34778087
 
  PDF   STATS   ENSEMBL   UCSC 
 
  406
 
  4
 
 15861
 
 island
 
 0.19438
 
 0.64787
 
 Not queried
 
  3.0981014
 
 2.86e-02
 
 1.00e+00
  
 
  
 5
 
 179634383
 
 179634789
 
  PDF   STATS   ENSEMBL   UCSC 
 
  407
 
  5
 
 31882
 
 gene;island
 
 0.88779
 
 0.40801
 
 Not queried
 
  3.0956385
 
 5.56e-02
 
 1.00e+00
  
 
  
 2
 
 176948544
 
 176949017
 
  PDF   STATS   ENSEMBL   UCSC 
 
  474
 
  9
 
 24850
 
 island
 
 0.03922
 
 0.30252
 
 Not queried
 
  3.0953246
 
 3.99e-03
 
 1.00e+00
  
 
  
 19
 
  11689488
 
  11689726
 
  PDF   STATS   ENSEMBL   UCSC 
 
  239
 
  6
 
 19810
 
 gene
 
 0.61869
 
 0.91956
 
 Not queried
 
  3.0950554
 
 2.16e-03
 
 1.00e+00
  
 
  
 4
 
 165898825
 
 165898967
 
  PDF   STATS   ENSEMBL   UCSC 
 
  143
 
  6
 
 30618
 
 tss
 
 0.93313
 
 0.59684
 
 Not queried
 
  3.0947524
 
 2.16e-03
 
 1.00e+00
  
 
  
 11
 
  34535085
 
  34535281
 
  PDF   STATS   ENSEMBL   UCSC 
 
  197
 
  4
 
 6563
 
 gene
 
 0.45327
 
 0.87790
 
 Not queried
 
  3.0943234
 
 2.86e-02
 
 1.00e+00
  
 
  
 22
 
  31198409
 
  31198724
 
  PDF   STATS   ENSEMBL   UCSC 
 
  316
 
  4
 
 26797
 
 gene;island
 
 0.01876
 
 0.11815
 
 Not queried
 
  3.0931698
 
 5.71e-02
 
 1.00e+00
  
 
  
 18
 
  77138860
 
  77139085
 
  PDF   STATS   ENSEMBL   UCSC 
 
  226
 
  4
 
 19562
 
 island
 
 0.04973
 
 0.34232
 
 Not queried
 
  3.0922932
 
 1.14e-01
 
 1.00e+00
  
 
  
 7
 
  75188876
 
  75189413
 
  PDF   STATS   ENSEMBL   UCSC 
 
  538
 
  7
 
 36984
 
 gene;island
 
 0.44844
 
 0.88188
 
 Not queried
 
  3.0920154
 
 4.08e-03
 
 1.00e+00
  
 
  
 12
 
 124870112
 
 124870394
 
  PDF   STATS   ENSEMBL   UCSC 
 
  283
 
  4
 
 9367
 
 gene;island
 
 0.46321
 
 0.87834
 
 Not queried
 
  3.0911994
 
 2.86e-02
 
 1.00e+00
  
 
  
 19
 
  49198900
 
  49199217
 
  PDF   STATS   ENSEMBL   UCSC 
 
  318
 
  4
 
 20887
 
 tss
 
 0.04694
 
 0.48801
 
 Not queried
 
  3.0910403
 
 3.43e-01
 
 1.00e+00
  
 
  
 1
 
 209605104
 
 209605401
 
  PDF   STATS   ENSEMBL   UCSC 
 
  298
 
  4
 
 980
 
 gene;tss
 
 0.42451
 
 0.87528
 
 Not queried
 
  3.0890111
 
 2.86e-02
 
 1.00e+00
  
 
  
 14
 
 106938234
 
 106938451
 
  PDF   STATS   ENSEMBL   UCSC 
 
  218
 
  7
 
 12648
 
 tss
 
 0.50002
 
 0.90370
 
 Not queried
 
  3.0881341
 
 5.83e-04
 
 1.00e+00
  
 
  
 2
 
 223163725
 
 223163809
 
  PDF   STATS   ENSEMBL   UCSC 
 
   85
 
  4
 
 23878
 
 tss
 
 0.16138
 
 0.02097
 
 Not queried
 
  3.0876370
 
 2.86e-02
 
 1.00e+00
  
 
  
 11
 
  74178684
 
  74179054
 
  PDF   STATS   ENSEMBL   UCSC 
 
  371
 
  7
 
 7537
 
 tss;island
 
 0.39070
 
 0.82375
 
 Not queried
 
  3.0858374
 
 5.30e-02
 
 1.00e+00
  
 
  
 7
 
   1609341
 
   1609515
 
  PDF   STATS   ENSEMBL   UCSC 
 
  175
 
  4
 
 36701
 
 gene;tss;island
 
 0.12882
 
 0.57424
 
 Not queried
 
  3.0840433
 
 2.86e-02
 
 1.00e+00
  
 
  
 7
 
  19146255
 
  19146555
 
  PDF   STATS   ENSEMBL   UCSC 
 
  301
 
  4
 
 38217
 
 island
 
 0.01725
 
 0.14214
 
 Not queried
 
  3.0837776
 
 2.86e-02
 
 1.00e+00
  
 
  
 1
 
  25256939
 
  25257191
 
  PDF   STATS   ENSEMBL   UCSC 
 
  253
 
  6
 
 329
 
 gene;tss;island
 
 0.29946
 
 0.03834
 
 Not queried
 
  3.0836737
 
 2.16e-03
 
 1.00e+00
  
 
  
 10
 
    530635
 
    531584
 
  PDF   STATS   ENSEMBL   UCSC 
 
  950
 
 12
 
 4428
 
 gene;island
 
 0.87083
 
 0.50481
 
 Not queried
 
  3.0797925
 
 2.74e-04
 
 1.00e+00
  
 
  
 22
 
  23522307
 
  23522511
 
  PDF   STATS   ENSEMBL   UCSC 
 
  205
 
  7
 
 26967
 
 tss
 
 0.51825
 
 0.33424
 
 Not queried
 
  3.0794763
 
 9.73e-02
 
 1.00e+00
  
 
  
 6
 
   1384491
 
   1384780
 
  PDF   STATS   ENSEMBL   UCSC 
 
  290
 
  4
 
 35302
 
 island
 
 0.80605
 
 0.43930
 
 Not queried
 
  3.0757383
 
 2.86e-02
 
 1.00e+00
  
 
  
 8
 
  38326358
 
  38326730
 
  PDF   STATS   ENSEMBL   UCSC 
 
  373
 
  6
 
 39594
 
 tss
 
 0.32264
 
 0.03291
 
 Not queried
 
  3.0737116
 
 6.49e-02
 
 1.00e+00
  
 
  
 17
 
  46657346
 
  46657656
 
  PDF   STATS   ENSEMBL   UCSC 
 
  311
 
  4
 
 17428
 
 tss
 
 0.78548
 
 0.32889
 
 Not queried
 
  3.0723845
 
 1.14e-01
 
 1.00e+00
  
 
  
 7
 
   5534412
 
   5534721
 
  PDF   STATS   ENSEMBL   UCSC 
 
  310
 
  4
 
 36816
 
 gene;island
 
 0.04068
 
 0.21457
 
 Not queried
 
  3.0703405
 
 3.43e-01
 
 1.00e+00
  
 
  
 4
 
 153601329
 
 153601466
 
  PDF   STATS   ENSEMBL   UCSC 
 
  138
 
  5
 
 30589
 
 tss
 
 0.02481
 
 0.11861
 
 Not queried
 
  3.0700427
 
 9.52e-02
 
 1.00e+00
  
 
  
 X
 
   9733868
 
   9734312
 
  PDF   STATS   ENSEMBL   UCSC 
 
  445
 
  5
 
 41628
 
 island
 
 0.63107
 
 0.93492
 
 Not queried
 
  3.0700318
 
 2.22e-01
 
 1.00e+00
  
 
  
 12
 
 130529058
 
 130530121
 
  PDF   STATS   ENSEMBL   UCSC 
 
 1064
 
  9
 
 10969
 
 island
 
 0.59184
 
 0.08989
 
 Not queried
 
  3.0699969
 
 1.42e-02
 
 1.00e+00
  
 
  
 2
 
  73298971
 
  73299297
 
  PDF   STATS   ENSEMBL   UCSC 
 
  327
 
  8
 
 23398
 
 tss
 
 0.11563
 
 0.01794
 
 Not queried
 
  3.0698315
 
 1.95e-01
 
 1.00e+00
  
 
  
 5
 
  37209182
 
  37209440
 
  PDF   STATS   ENSEMBL   UCSC 
 
  259
 
  4
 
 31532
 
 gene;island
 
 0.32560
 
 0.77525
 
 Not queried
 
  3.0695546
 
 2.86e-02
 
 1.00e+00
  
 
  
 3
 
 109128550
 
 109128775
 
  PDF   STATS   ENSEMBL   UCSC 
 
  226
 
  5
 
 28451
 
 tss
 
 0.28107
 
 0.78401
 
 Not queried
 
  3.0684718
 
 5.56e-02
 
 1.00e+00
  
 
  
 8
 
 108510286
 
 108510343
 
  PDF   STATS   ENSEMBL   UCSC 
 
   58
 
  4
 
 39801
 
 tss
 
 0.20528
 
 0.65131
 
 Not queried
 
  3.0662528
 
 2.86e-02
 
 1.00e+00
  
 
  
 7
 
  55086091
 
  55086288
 
  PDF   STATS   ENSEMBL   UCSC 
 
  198
 
  4
 
 37538
 
 tss;island
 
 0.34282
 
 0.04136
 
 Not queried
 
  3.0645622
 
 1.14e-01
 
 1.00e+00
  
 
  
 12
 
 130961424
 
 130961986
 
  PDF   STATS   ENSEMBL   UCSC 
 
  563
 
  8
 
 9391
 
 gene
 
 0.41122
 
 0.86601
 
 Not queried
 
  3.0640358
 
 6.99e-03
 
 1.00e+00
  
 
  
 X
 
  48534493
 
  48534872
 
  PDF   STATS   ENSEMBL   UCSC 
 
  380
 
  4
 
 41768
 
 island
 
 0.10901
 
 0.53089
 
 Not queried
 
  3.0627079
 
 2.86e-02
 
 1.00e+00
  
 
  
 Y
 
   2802900
 
   2803127
 
  PDF   STATS   ENSEMBL   UCSC 
 
  228
 
  7
 
 42165
 
 tss;island
 
 0.08610
 
 0.45100
 
 Not queried
 
  3.0616595
 
 2.33e-03
 
 1.00e+00
  
 
  
 10
 
   4868328
 
   4868398
 
  PDF   STATS   ENSEMBL   UCSC 
 
   71
 
  6
 
 4904
 
 tss
 
 0.12662
 
 0.51846
 
 Not queried
 
  3.0564139
 
 2.16e-03
 
 1.00e+00
  
 
  
 6
 
  27100831
 
  27101279
 
  PDF   STATS   ENSEMBL   UCSC 
 
  449
 
  5
 
 33552
 
 gene
 
 0.01770
 
 0.08321
 
 Not queried
 
  3.0561218
 
 2.22e-01
 
 1.00e+00
  
 
  
 11
 
  15136059
 
  15136688
 
  PDF   STATS   ENSEMBL   UCSC 
 
  630
 
  9
 
 6503
 
 gene;island
 
 0.04360
 
 0.25711
 
 Not queried
 
  3.0556563
 
 3.15e-02
 
 1.00e+00
  
 
  
 3
 
  45077649
 
  45078075
 
  PDF   STATS   ENSEMBL   UCSC 
 
  427
 
  6
 
 29003
 
 island
 
 0.21081
 
 0.73172
 
 Not queried
 
  3.0521360
 
 1.80e-01
 
 1.00e+00
  
 
  
 17
 
    154410
 
    154671
 
  PDF   STATS   ENSEMBL   UCSC 
 
  262
 
  4
 
 16252
 
 gene;island
 
 0.25964
 
 0.76254
 
 Not queried
 
  3.0509046
 
 2.00e-01
 
 1.00e+00
  
 
  
 X
 
 102000610
 
 102001318
 
  PDF   STATS   ENSEMBL   UCSC 
 
  709
 
 11
 
 41056
 
 gene;island
 
 0.25503
 
 0.03261
 
 Not queried
 
  3.0500198
 
 8.33e-03
 
 1.00e+00
  
 
  
 12
 
 114841708
 
 114842031
 
  PDF   STATS   ENSEMBL   UCSC 
 
  324
 
  6
 
 10007
 
 tss
 
 0.95191
 
 0.52838
 
 Not queried
 
  3.0498183
 
 2.16e-03
 
 1.00e+00
  
 
  
 4
 
   1005876
 
   1006278
 
  PDF   STATS   ENSEMBL   UCSC 
 
  403
 
 10
 
 29812
 
 gene;island
 
 0.18665
 
 0.06380
 
 Not queried
 
  3.0478329
 
 3.15e-01
 
 1.00e+00
  
 
  
 20
 
  61147572
 
  61147679
 
  PDF   STATS   ENSEMBL   UCSC 
 
  108
 
  6
 
 25448
 
 gene;island
 
 0.40690
 
 0.85146
 
 Not queried
 
  3.0467339
 
 4.33e-03
 
 1.00e+00
  
 
  
 10
 
 105428385
 
 105428818
 
  PDF   STATS   ENSEMBL   UCSC 
 
  434
 
  5
 
 4731
 
 gene;island
 
 0.51135
 
 0.86897
 
 Not queried
 
  3.0465009
 
 1.59e-02
 
 1.00e+00
  
 
  
 7
 
  81240257
 
  81240667
 
  PDF   STATS   ENSEMBL   UCSC 
 
  411
 
  5
 
 38559
 
 island
 
 0.03184
 
 0.17875
 
 Not queried
 
  3.0463416
 
 3.17e-02
 
 1.00e+00
  
 
  
 10
 
  13748985
 
  13749625
 
  PDF   STATS   ENSEMBL   UCSC 
 
  641
 
  9
 
 4491
 
 gene
 
 0.02059
 
 0.19719
 
 Not queried
 
  3.0459560
 
 2.44e-02
 
 1.00e+00
  
 
  
 6
 
  72130539
 
  72130799
 
  PDF   STATS   ENSEMBL   UCSC 
 
  261
 
  9
 
 34997
 
 tss
 
 0.02278
 
 0.16642
 
 Not queried
 
  3.0457701
 
 5.64e-03
 
 1.00e+00
  
 
  
 2
 
  56412924
 
  56413253
 
  PDF   STATS   ENSEMBL   UCSC 
 
  330
 
  5
 
 22655
 
 gene;island
 
 0.05890
 
 0.34060
 
 Not queried
 
  3.0449878
 
 2.22e-01
 
 1.00e+00
  
 
  
 10
 
  64578739
 
  64579140
 
  PDF   STATS   ENSEMBL   UCSC 
 
  402
 
  5
 
 5758
 
 island
 
 0.32802
 
 0.04857
 
 Not queried
 
  3.0439399
 
 7.94e-03
 
 1.00e+00
  
 
  
 8
 
 125985352
 
 125985512
 
  PDF   STATS   ENSEMBL   UCSC 
 
  161
 
  4
 
 39844
 
 tss
 
 0.46781
 
 0.89546
 
 Not queried
 
  3.0423581
 
 2.86e-02
 
 1.00e+00
  
 
  
 6
 
 106546704
 
 106546754
 
  PDF   STATS   ENSEMBL   UCSC 
 
   51
 
  4
 
 35084
 
 tss
 
 0.02474
 
 0.11761
 
 Not queried
 
  3.0398626
 
 2.86e-02
 
 1.00e+00
  
 
  
 4
 
   9782891
 
   9783398
 
  PDF   STATS   ENSEMBL   UCSC 
 
  508
 
 10
 
 30874
 
 island
 
 0.37467
 
 0.89993
 
 Not queried
 
  3.0391109
 
 2.88e-03
 
 1.00e+00
  
 
  
 19
 
  12175107
 
  12175719
 
  PDF   STATS   ENSEMBL   UCSC 
 
  613
 
 10
 
 21555
 
 island
 
 0.05102
 
 0.37859
 
 Not queried
 
  3.0388703
 
 3.55e-02
 
 1.00e+00
  
 
  
 3
 
  50359849
 
  50360176
 
  PDF   STATS   ENSEMBL   UCSC 
 
  328
 
  6
 
 27762
 
 gene;tss;island
 
 0.56465
 
 0.23813
 
 Not queried
 
  3.0377999
 
 4.33e-03
 
 1.00e+00
  
 
  
 5
 
 134783075
 
 134783273
 
  PDF   STATS   ENSEMBL   UCSC 
 
  199
 
  4
 
 32245
 
 tss
 
 0.86167
 
 0.46503
 
 Not queried
 
  3.0377625
 
 2.86e-02
 
 1.00e+00
  
 
  
 5
 
  41870746
 
  41871189
 
  PDF   STATS   ENSEMBL   UCSC 
 
  444
 
 10
 
 32693
 
 island
 
 0.05293
 
 0.36127
 
 Not queried
 
  3.0364764
 
 2.32e-02
 
 1.00e+00
  
 
  
 17
 
  79797038
 
  79797373
 
  PDF   STATS   ENSEMBL   UCSC 
 
  336
 
  5
 
 18908
 
 island
 
 0.31693
 
 0.84953
 
 Not queried
 
  3.0358034
 
 3.17e-02
 
 1.00e+00
  
 
  
 20
 
  61147572
 
  61147652
 
  PDF   STATS   ENSEMBL   UCSC 
 
   81
 
  5
 
 25834
 
 tss
 
 0.41225
 
 0.85186
 
 Not queried
 
  3.0353341
 
 7.94e-03
 
 1.00e+00
  
 
  
 16
 
   9058197
 
   9058365
 
  PDF   STATS   ENSEMBL   UCSC 
 
  169
 
  4
 
 15053
 
 tss;island
 
 0.41717
 
 0.06652
 
 Not queried
 
  3.0346195
 
 2.00e-01
 
 1.00e+00
  
 
  
 20
 
  22567724
 
  22568116
 
  PDF   STATS   ENSEMBL   UCSC 
 
  393
 
  4
 
 26006
 
 island
 
 0.12214
 
 0.56741
 
 Not queried
 
  3.0335843
 
 3.43e-01
 
 1.00e+00
  
 
  
 10
 
  99734416
 
  99735202
 
  PDF   STATS   ENSEMBL   UCSC 
 
  787
 
  8
 
 4688
 
 gene
 
 0.25779
 
 0.73027
 
 Not queried
 
  3.0328016
 
 3.11e-04
 
 1.00e+00
  
 
  
 4
 
  24796689
 
  24797007
 
  PDF   STATS   ENSEMBL   UCSC 
 
  319
 
  5
 
 30280
 
 tss
 
 0.14151
 
 0.61556
 
 Not queried
 
  3.0327486
 
 6.90e-01
 
 1.00e+00
  
 
  
 12
 
  12938082
 
  12938425
 
  PDF   STATS   ENSEMBL   UCSC 
 
  344
 
  6
 
 9035
 
 gene;tss;island
 
 0.73015
 
 0.31762
 
 Not queried
 
  3.0319493
 
 2.16e-03
 
 1.00e+00
  
 
  
 3
 
 113251061
 
 113251417
 
  PDF   STATS   ENSEMBL   UCSC 
 
  357
 
  5
 
 29323
 
 island
 
 0.00594
 
 0.03448
 
 Not queried
 
  3.0314241
 
 4.21e-01
 
 1.00e+00
  
 
  
 17
 
  38334161
 
  38334186
 
  PDF   STATS   ENSEMBL   UCSC 
 
   26
 
  5
 
 17293
 
 tss;island
 
 0.23034
 
 0.02681
 
 Not queried
 
  3.0286327
 
 7.94e-03
 
 1.00e+00
  
 
  
 6
 
 117802401
 
 117802786
 
  PDF   STATS   ENSEMBL   UCSC 
 
  386
 
  6
 
 35132
 
 tss;island
 
 0.37697
 
 0.08241
 
 Not queried
 
  3.0277225
 
 8.66e-03
 
 1.00e+00
  
 
  
 15
 
  41803428
 
  41803850
 
  PDF   STATS   ENSEMBL   UCSC 
 
  423
 
  4
 
 13275
 
 gene;island
 
 0.58595
 
 0.12018
 
 Not queried
 
  3.0257388
 
 2.86e-02
 
 1.00e+00
  
 
  
 5
 
 140261653
 
 140261930
 
  PDF   STATS   ENSEMBL   UCSC 
 
  278
 
  7
 
 31746
 
 gene;island
 
 0.06114
 
 0.43869
 
 Not queried
 
  3.0255504
 
 1.75e-02
 
 1.00e+00
  
 
  
 18
 
  11751343
 
  11751556
 
  PDF   STATS   ENSEMBL   UCSC 
 
  214
 
  5
 
 19150
 
 tss
 
 0.37276
 
 0.82868
 
 Not queried
 
  3.0249279
 
 3.17e-02
 
 1.00e+00
  
 
  
 15
 
  65648103
 
  65648490
 
  PDF   STATS   ENSEMBL   UCSC 
 
  388
 
  4
 
 13350
 
 gene;island
 
 0.23337
 
 0.03469
 
 Not queried
 
  3.0235069
 
 2.00e-01
 
 1.00e+00
  
 
  
 7
 
  54956202
 
  54956598
 
  PDF   STATS   ENSEMBL   UCSC 
 
  397
 
  5
 
 38437
 
 island
 
 0.61365
 
 0.21752
 
 Not queried
 
  3.0229680
 
 3.17e-02
 
 1.00e+00
  
 
  
 22
 
  42469976
 
  42470223
 
  PDF   STATS   ENSEMBL   UCSC 
 
  248
 
  6
 
 27145
 
 tss;island
 
 0.46554
 
 0.89234
 
 Not queried
 
  3.0214018
 
 2.16e-03
 
 1.00e+00
  
 
  
 2
 
 242710665
 
 242711046
 
  PDF   STATS   ENSEMBL   UCSC 
 
  382
 
  5
 
 25266
 
 island
 
 0.50661
 
 0.88863
 
 Not queried
 
  3.0206653
 
 5.56e-02
 
 1.00e+00
  
 
  
 19
 
  58951599
 
  58951885
 
  PDF   STATS   ENSEMBL   UCSC 
 
  287
 
  8
 
 21106
 
 tss
 
 0.22831
 
 0.02388
 
 Not queried
 
  3.0199363
 
 1.09e-03
 
 1.00e+00
  
 
  
 20
 
    982991
 
    983130
 
  PDF   STATS   ENSEMBL   UCSC 
 
  140
 
  4
 
 25492
 
 tss
 
 0.08009
 
 0.39378
 
 Not queried
 
  3.0138779
 
 1.14e-01
 
 1.00e+00
  
 
  
 5
 
 169532686
 
 169533120
 
  PDF   STATS   ENSEMBL   UCSC 
 
  435
 
  8
 
 33312
 
 island
 
 0.30183
 
 0.73001
 
 Not queried
 
  3.0137840
 
 4.66e-03
 
 1.00e+00
  
 
  
 17
 
  43222106
 
  43222258
 
  PDF   STATS   ENSEMBL   UCSC 
 
  153
 
  4
 
 18392
 
 island
 
 0.25781
 
 0.73587
 
 Not queried
 
  3.0074935
 
 2.86e-02
 
 1.00e+00
  
 
  
 15
 
  57998711
 
  57998919
 
  PDF   STATS   ENSEMBL   UCSC 
 
  209
 
  6
 
 13336
 
 gene;island
 
 0.30019
 
 0.02696
 
 Not queried
 
  3.0070002
 
 5.89e-01
 
 1.00e+00
  
 
  
 X
 
 118699184
 
 118699375
 
  PDF   STATS   ENSEMBL   UCSC 
 
  192
 
  4
 
 41091
 
 gene
 
 0.01810
 
 0.12166
 
 Not queried
 
  3.0068403
 
 3.43e-01
 
 1.00e+00
  
 
  
 11
 
   2883529
 
   2883912
 
  PDF   STATS   ENSEMBL   UCSC 
 
  384
 
  4
 
 7953
 
 island
 
 0.53963
 
 0.21852
 
 Not queried
 
  3.0063776
 
 1.14e-01
 
 1.00e+00
  
 
  
 19
 
  12167119
 
  12167313
 
  PDF   STATS   ENSEMBL   UCSC 
 
  195
 
  4
 
 21554
 
 island
 
 0.33506
 
 0.75300
 
 Not queried
 
  3.0062372
 
 2.00e-01
 
 1.00e+00
  
 
  
 20
 
  37230326
 
  37230741
 
  PDF   STATS   ENSEMBL   UCSC 
 
  416
 
  7
 
 26118
 
 island
 
 0.05411
 
 0.29874
 
 Not queried
 
  3.0049029
 
 5.83e-04
 
 1.00e+00
  
 
  
 17
 
  80708279
 
  80708632
 
  PDF   STATS   ENSEMBL   UCSC 
 
  354
 
  4
 
 16923
 
 gene;island
 
 0.93816
 
 0.67332
 
 Not queried
 
  3.0042345
 
 2.86e-02
 
 1.00e+00
  
 
  
 2
 
  71693090
 
  71694016
 
  PDF   STATS   ENSEMBL   UCSC 
 
  927
 
 12
 
 22694
 
 gene;island
 
 0.34590
 
 0.08934
 
 Not queried
 
  3.0024105
 
 4.96e-04
 
 1.00e+00
  
 
  
 4
 
 139481751
 
 139482017
 
  PDF   STATS   ENSEMBL   UCSC 
 
  267
 
  4
 
 31255
 
 island
 
 0.28044
 
 0.95218
 
 Not queried
 
  3.0016509
 
 2.00e-01
 
 1.00e+00
  
 
  
 9
 
  38622294
 
  38622748
 
  PDF   STATS   ENSEMBL   UCSC 
 
  455
 
  5
 
 40701
 
 gene;island
 
 0.09317
 
 0.43584
 
 Not queried
 
  3.0013143
 
 1.51e-01
 
 1.00e+00
  
 
  
 17
 
  77903985
 
  77904396
 
  PDF   STATS   ENSEMBL   UCSC 
 
  412
 
  5
 
 18813
 
 island
 
 0.06447
 
 0.36849
 
 Not queried
 
  3.0007930
 
 7.94e-03
 
 1.00e+00
  
 
  
 13
 
  20735819
 
  20736342
 
  PDF   STATS   ENSEMBL   UCSC 
 
  524
 
  8
 
 11300
 
 tss;island
 
 0.47913
 
 0.06514
 
 Not queried
 
  2.9989225
 
 1.04e-02
 
 1.00e+00
  
 
  
 10
 
 135039948
 
 135040250
 
  PDF   STATS   ENSEMBL   UCSC 
 
  303
 
  4
 
 6327
 
 island
 
 0.49938
 
 0.88339
 
 Not queried
 
  2.9978522
 
 2.86e-02
 
 1.00e+00
  
 
  
 3
 
  49395923
 
  49395991
 
  PDF   STATS   ENSEMBL   UCSC 
 
   69
 
  4
 
 28285
 
 tss
 
 0.84740
 
 0.33922
 
 Not queried
 
  2.9973427
 
 2.86e-02
 
 1.00e+00
  
 
  
 2
 
  43327937
 
  43328657
 
  PDF   STATS   ENSEMBL   UCSC 
 
  721
 
  7
 
 24209
 
 island
 
 0.18874
 
 0.04802
 
 Not queried
 
  2.9966170
 
 5.30e-02
 
 1.00e+00
  
 
  
 10
 
 102976327
 
 102977342
 
  PDF   STATS   ENSEMBL   UCSC 
 
 1016
 
 11
 
 6038
 
 island
 
 0.06850
 
 0.34439
 
 Not queried
 
  2.9952096
 
 7.65e-04
 
 1.00e+00
  
 
  
 17
 
  33787402
 
  33787739
 
  PDF   STATS   ENSEMBL   UCSC 
 
  338
 
  4
 
 18182
 
 island
 
 0.17826
 
 0.57058
 
 Not queried
 
  2.9938431
 
 5.71e-02
 
 1.00e+00
  
 
  
 2
 
 223916502
 
 223916820
 
  PDF   STATS   ENSEMBL   UCSC 
 
  319
 
  5
 
 23883
 
 tss
 
 0.51255
 
 0.93262
 
 Not queried
 
  2.9916900
 
 7.94e-03
 
 1.00e+00
  
 
  
 19
 
  46379763
 
  46380134
 
  PDF   STATS   ENSEMBL   UCSC 
 
  372
 
  4
 
 22125
 
 island
 
 0.26307
 
 0.70536
 
 Not queried
 
  2.9912310
 
 2.86e-02
 
 1.00e+00
  
 
  
 19
 
   1826707
 
   1826957
 
  PDF   STATS   ENSEMBL   UCSC 
 
  251
 
  4
 
 19630
 
 gene;island
 
 0.53280
 
 0.87474
 
 Not queried
 
  2.9910294
 
 2.86e-02
 
 1.00e+00
  
 
  
 19
 
  46181221
 
  46181546
 
  PDF   STATS   ENSEMBL   UCSC 
 
  326
 
  5
 
 20069
 
 gene;island
 
 0.06128
 
 0.35621
 
 Not queried
 
  2.9908044
 
 1.59e-02
 
 1.00e+00
  
 
  
 X
 
 135230594
 
 135230841
 
  PDF   STATS   ENSEMBL   UCSC 
 
  248
 
  5
 
 41118
 
 gene;island
 
 0.39518
 
 0.07596
 
 Not queried
 
  2.9906293
 
 3.17e-02
 
 1.00e+00
  
 
  
 10
 
 135150367
 
 135150517
 
  PDF   STATS   ENSEMBL   UCSC 
 
  151
 
  6
 
 6339
 
 island
 
 0.05108
 
 0.34669
 
 Not queried
 
  2.9903547
 
 4.33e-03
 
 1.00e+00
  
 
  
 X
 
 102000610
 
 102000905
 
  PDF   STATS   ENSEMBL   UCSC 
 
  296
 
  7
 
 41434
 
 tss
 
 0.25718
 
 0.04132
 
 Not queried
 
  2.9896428
 
 2.09e-01
 
 1.00e+00
  
 
  
 7
 
 158936508
 
 158936739
 
  PDF   STATS   ENSEMBL   UCSC 
 
  232
 
  4
 
 37320
 
 gene;island
 
 0.14351
 
 0.01751
 
 Not queried
 
  2.9893541
 
 3.43e-01
 
 1.00e+00
  
 
  
 6
 
 146350041
 
 146350748
 
  PDF   STATS   ENSEMBL   UCSC 
 
  708
 
  8
 
 34435
 
 gene;island
 
 0.26888
 
 0.72036
 
 Not queried
 
  2.9869449
 
 1.04e-02
 
 1.00e+00
  
 
  
 11
 
  10673844
 
  10674182
 
  PDF   STATS   ENSEMBL   UCSC 
 
  339
 
  4
 
 6491
 
 gene
 
 0.45080
 
 0.84231
 
 Not queried
 
  2.9862463
 
 2.86e-02
 
 1.00e+00
  
 
  
 17
 
  77997663
 
  77997997
 
  PDF   STATS   ENSEMBL   UCSC 
 
  335
 
  5
 
 16816
 
 gene;island
 
 0.60166
 
 0.94371
 
 Not queried
 
  2.9854519
 
 5.56e-02
 
 1.00e+00
  
 
  
 1
 
 147737016
 
 147737302
 
  PDF   STATS   ENSEMBL   UCSC 
 
  287
 
  5
 
 3690
 
 island
 
 0.21764
 
 0.02807
 
 Not queried
 
  2.9849431
 
 7.94e-03
 
 1.00e+00
  
 
  
 12
 
  48399140
 
  48399493
 
  PDF   STATS   ENSEMBL   UCSC 
 
  354
 
  5
 
 9641
 
 tss;island
 
 0.85467
 
 0.52211
 
 Not queried
 
  2.9844826
 
 1.59e-02
 
 1.00e+00
  
 
  
 17
 
  56736571
 
  56736918
 
  PDF   STATS   ENSEMBL   UCSC 
 
  348
 
  5
 
 16675
 
 gene;island
 
 0.40805
 
 0.08016
 
 Not queried
 
  2.9837033
 
 7.94e-03
 
 1.00e+00
  
 
  
 16
 
  89114613
 
  89114919
 
  PDF   STATS   ENSEMBL   UCSC 
 
  307
 
  5
 
 16200
 
 island
 
 0.25828
 
 0.72893
 
 Not queried
 
  2.9835071
 
 7.94e-03
 
 1.00e+00
  
 
  
 6
 
   3752754
 
   3753083
 
  PDF   STATS   ENSEMBL   UCSC 
 
  330
 
  4
 
 34536
 
 tss;island
 
 0.61627
 
 0.18342
 
 Not queried
 
  2.9834674
 
 2.86e-02
 
 1.00e+00
  
 
  
 5
 
 140797172
 
 140797286
 
  PDF   STATS   ENSEMBL   UCSC 
 
  115
 
  4
 
 31776
 
 gene;island
 
 0.18622
 
 0.62754
 
 Not queried
 
  2.9798055
 
 2.86e-02
 
 1.00e+00
  
 
  
 19
 
  19383613
 
  19384165
 
  PDF   STATS   ENSEMBL   UCSC 
 
  553
 
  5
 
 21775
 
 island
 
 0.22937
 
 0.03830
 
 Not queried
 
  2.9793999
 
 3.17e-02
 
 1.00e+00
  
 
  
 8
 
 142236917
 
 142237359
 
  PDF   STATS   ENSEMBL   UCSC 
 
  443
 
  5
 
 39388
 
 gene;island
 
 0.50159
 
 0.87568
 
 Not queried
 
  2.9784021
 
 7.94e-03
 
 1.00e+00
  
 
  
 16
 
  52581405
 
  52581746
 
  PDF   STATS   ENSEMBL   UCSC 
 
  342
 
  5
 
 15202
 
 tss;island
 
 0.20817
 
 0.04508
 
 Not queried
 
  2.9780191
 
 3.17e-02
 
 1.00e+00
  
 
  
 10
 
  14014419
 
  14014651
 
  PDF   STATS   ENSEMBL   UCSC 
 
  233
 
  5
 
 4493
 
 gene
 
 0.46718
 
 0.89980
 
 Not queried
 
  2.9757971
 
 9.52e-02
 
 1.00e+00
  
 
  
 16
 
  88803803
 
  88804051
 
  PDF   STATS   ENSEMBL   UCSC 
 
  249
 
  4
 
 14883
 
 gene;island
 
 0.02339
 
 0.31355
 
 Not queried
 
  2.9751884
 
 5.71e-02
 
 1.00e+00
  
 
  
 X
 
  64754701
 
  64754995
 
  PDF   STATS   ENSEMBL   UCSC 
 
  295
 
 10
 
 41352
 
 tss
 
 0.10025
 
 0.02670
 
 Not queried
 
  2.9741163
 
 4.87e-04
 
 1.00e+00
  
 
  
 10
 
  97889373
 
  97889705
 
  PDF   STATS   ENSEMBL   UCSC 
 
  333
 
  6
 
 5953
 
 island
 
 0.55832
 
 0.20331
 
 Not queried
 
  2.9739539
 
 1.80e-01
 
 1.00e+00
  
 
  
 12
 
 124941781
 
 124942248
 
  PDF   STATS   ENSEMBL   UCSC 
 
  468
 
  5
 
 9371
 
 gene;island
 
 0.63002
 
 0.91845
 
 Not queried
 
  2.9734913
 
 7.94e-03
 
 1.00e+00
  
 
  
 19
 
  10736006
 
  10736117
 
  PDF   STATS   ENSEMBL   UCSC 
 
  112
 
  5
 
 20419
 
 tss
 
 0.03223
 
 0.19128
 
 Not queried
 
  2.9733693
 
 7.94e-03
 
 1.00e+00
  
 
  
 4
 
   3486039
 
   3486380
 
  PDF   STATS   ENSEMBL   UCSC 
 
  342
 
  4
 
 29863
 
 gene;island
 
 0.43860
 
 0.79008
 
 Not queried
 
  2.9712077
 
 2.86e-02
 
 1.00e+00
  
 
  
 5
 
 101834365
 
 101834675
 
  PDF   STATS   ENSEMBL   UCSC 
 
  311
 
  4
 
 31646
 
 gene
 
 0.23419
 
 0.74742
 
 Not queried
 
  2.9699881
 
 2.86e-02
 
 1.00e+00
  
 
  
 10
 
   1517083
 
   1517340
 
  PDF   STATS   ENSEMBL   UCSC 
 
  258
 
  5
 
 4454
 
 gene;island
 
 0.11012
 
 0.50012
 
 Not queried
 
  2.9662931
 
 9.52e-02
 
 1.00e+00
  
 
  
 11
 
  62559638
 
  62559882
 
  PDF   STATS   ENSEMBL   UCSC 
 
  245
 
  5
 
 6629
 
 gene
 
 0.08880
 
 0.42805
 
 Not queried
 
  2.9662433
 
 9.52e-02
 
 1.00e+00
  
 
  
 17
 
  75180961
 
  75181373
 
  PDF   STATS   ENSEMBL   UCSC 
 
  413
 
  4
 
 16766
 
 gene
 
 0.34375
 
 0.79088
 
 Not queried
 
  2.9650368
 
 5.71e-02
 
 1.00e+00
  
 
  
 15
 
  42371511
 
  42371967
 
  PDF   STATS   ENSEMBL   UCSC 
 
  457
 
  7
 
 13278
 
 gene;island
 
 0.13195
 
 0.55652
 
 Not queried
 
  2.9635210
 
 5.83e-04
 
 1.00e+00
  
 
  
 10
 
 100227419
 
 100227782
 
  PDF   STATS   ENSEMBL   UCSC 
 
  364
 
  4
 
 4690
 
 gene;island
 
 0.01505
 
 0.14241
 
 Not queried
 
  2.9632638
 
 3.43e-01
 
 1.00e+00
  
 
  
 11
 
   1902923
 
   1903333
 
  PDF   STATS   ENSEMBL   UCSC 
 
  411
 
  4
 
 6418
 
 gene;island
 
 0.33440
 
 0.71032
 
 Not queried
 
  2.9621887
 
 2.00e-01
 
 1.00e+00
  
 
  
 4
 
 183063397
 
 183063637
 
  PDF   STATS   ENSEMBL   UCSC 
 
  241
 
  4
 
 30143
 
 gene;island
 
 0.60285
 
 0.15083
 
 Not queried
 
  2.9616896
 
 5.71e-02
 
 1.00e+00
  
 
  
 7
 
   1083072
 
   1083473
 
  PDF   STATS   ENSEMBL   UCSC 
 
  402
 
  4
 
 36678
 
 gene;island
 
 0.61021
 
 0.91816
 
 Not queried
 
  2.9588172
 
 2.86e-02
 
 1.00e+00
  
 
  
 13
 
  27333803
 
  27334259
 
  PDF   STATS   ENSEMBL   UCSC 
 
  457
 
  4
 
 11085
 
 gene;island
 
 0.04702
 
 0.36447
 
 Not queried
 
  2.9572196
 
 2.86e-02
 
 1.00e+00
  
 
  
 22
 
  27068716
 
  27068761
 
  PDF   STATS   ENSEMBL   UCSC 
 
   46
 
  4
 
 26786
 
 gene
 
 0.05538
 
 0.00722
 
 Not queried
 
  2.9556447
 
 2.86e-02
 
 1.00e+00
  
 
  
 7
 
 100199952
 
 100200009
 
  PDF   STATS   ENSEMBL   UCSC 
 
   58
 
  4
 
 37081
 
 gene
 
 0.91682
 
 0.63387
 
 Not queried
 
  2.9526373
 
 2.86e-02
 
 1.00e+00
  
 
  
 13
 
 113832355
 
 113832595
 
  PDF   STATS   ENSEMBL   UCSC 
 
  241
 
  4
 
 11251
 
 gene
 
 0.62939
 
 0.94695
 
 Not queried
 
  2.9513937
 
 5.71e-02
 
 1.00e+00
  
 
  
 12
 
   7033470
 
   7033563
 
  PDF   STATS   ENSEMBL   UCSC 
 
   94
 
  4
 
 9510
 
 tss
 
 0.03532
 
 0.09014
 
 Not queried
 
  2.9506069
 
 3.43e-01
 
 1.00e+00
  
 
  
 11
 
  65779192
 
  65779343
 
  PDF   STATS   ENSEMBL   UCSC 
 
  152
 
  6
 
 7435
 
 tss
 
 0.25397
 
 0.68469
 
 Not queried
 
  2.9505945
 
 2.16e-03
 
 1.00e+00
  
 
  
 10
 
 135150485
 
 135150517
 
  PDF   STATS   ENSEMBL   UCSC 
 
   33
 
  4
 
 5443
 
 tss
 
 0.05108
 
 0.30469
 
 Not queried
 
  2.9505059
 
 5.71e-02
 
 1.00e+00
  
 
  
 5
 
 139135602
 
 139135961
 
  PDF   STATS   ENSEMBL   UCSC 
 
  360
 
  4
 
 33116
 
 island
 
 0.07511
 
 0.38956
 
 Not queried
 
  2.9502410
 
 5.71e-02
 
 1.00e+00
  
 
  
 20
 
  57471654
 
  57471844
 
  PDF   STATS   ENSEMBL   UCSC 
 
  191
 
  5
 
 25438
 
 gene
 
 0.68934
 
 0.94203
 
 Not queried
 
  2.9489357
 
 1.59e-02
 
 1.00e+00
  
 
  
 3
 
  81810999
 
  81811448
 
  PDF   STATS   ENSEMBL   UCSC 
 
  450
 
  6
 
 28406
 
 tss
 
 0.24867
 
 0.03597
 
 Not queried
 
  2.9458370
 
 4.11e-02
 
 1.00e+00
  
 
  
 6
 
  31685392
 
  31685575
 
  PDF   STATS   ENSEMBL   UCSC 
 
  184
 
  7
 
 33877
 
 gene
 
 0.11494
 
 0.49746
 
 Not queried
 
  2.9451865
 
 5.83e-04
 
 1.00e+00
  
 
  
 X
 
  70288026
 
  70288225
 
  PDF   STATS   ENSEMBL   UCSC 
 
  200
 
  4
 
 41019
 
 gene
 
 0.04226
 
 0.00963
 
 Not queried
 
  2.9450868
 
 1.14e-01
 
 1.00e+00
  
 
  
 19
 
  15121204
 
  15121531
 
  PDF   STATS   ENSEMBL   UCSC 
 
  328
 
  7
 
 20516
 
 tss
 
 0.25359
 
 0.70801
 
 Not queried
 
  2.9431166
 
 4.08e-03
 
 1.00e+00
  
 
  
 1
 
  15250209
 
  15250388
 
  PDF   STATS   ENSEMBL   UCSC 
 
  180
 
  4
 
 238
 
 gene;tss;island
 
 0.59108
 
 0.18364
 
 Not queried
 
  2.9402041
 
 2.00e-01
 
 1.00e+00
  
 
  
 14
 
  60715218
 
  60715614
 
  PDF   STATS   ENSEMBL   UCSC 
 
  397
 
  4
 
 12074
 
 gene;tss;island
 
 0.18187
 
 0.03807
 
 Not queried
 
  2.9350575
 
 5.71e-02
 
 1.00e+00
  
 
  
 2
 
 176973688
 
 176974060
 
  PDF   STATS   ENSEMBL   UCSC 
 
  373
 
  5
 
 22898
 
 gene;island
 
 0.75769
 
 0.19634
 
 Not queried
 
  2.9349206
 
 7.94e-03
 
 1.00e+00
  
 
  
 6
 
 106546704
 
 106546824
 
  PDF   STATS   ENSEMBL   UCSC 
 
  121
 
  5
 
 34369
 
 gene
 
 0.02896
 
 0.13803
 
 Not queried
 
  2.9341732
 
 7.94e-03
 
 1.00e+00
  
 
  
 1
 
 155043322
 
 155043967
 
  PDF   STATS   ENSEMBL   UCSC 
 
  646
 
  7
 
 3795
 
 island
 
 0.04838
 
 0.28717
 
 Not queried
 
  2.9338829
 
 2.09e-01
 
 1.00e+00
  
 
  
 22
 
  37640314
 
  37640514
 
  PDF   STATS   ENSEMBL   UCSC 
 
  201
 
  5
 
 27073
 
 tss
 
 0.06787
 
 0.35746
 
 Not queried
 
  2.9335927
 
 7.94e-03
 
 1.00e+00
  
 
  
 1
 
 228246632
 
 228247135
 
  PDF   STATS   ENSEMBL   UCSC 
 
  504
 
  4
 
 1043
 
 gene;island
 
 0.20196
 
 0.63046
 
 Not queried
 
  2.9326714
 
 5.71e-02
 
 1.00e+00
  
 
  
 10
 
  81075012
 
  81075241
 
  PDF   STATS   ENSEMBL   UCSC 
 
  230
 
  4
 
 4635
 
 gene
 
 0.59432
 
 0.89943
 
 Not queried
 
  2.9322879
 
 2.86e-02
 
 1.00e+00
  
 
  
 13
 
  50570018
 
  50570250
 
  PDF   STATS   ENSEMBL   UCSC 
 
  233
 
  4
 
 11139
 
 gene;tss;island
 
 0.57376
 
 0.14484
 
 Not queried
 
  2.9312541
 
 2.86e-02
 
 1.00e+00
  
 
  
 3
 
    350503
 
    351003
 
  PDF   STATS   ENSEMBL   UCSC 
 
  501
 
  6
 
 27595
 
 gene;island
 
 0.07001
 
 0.47864
 
 Not queried
 
  2.9310674
 
 9.31e-02
 
 1.00e+00
  
 
  
 X
 
  54209594
 
  54209715
 
  PDF   STATS   ENSEMBL   UCSC 
 
  122
 
  4
 
 41813
 
 island
 
 0.17925
 
 0.02232
 
 Not queried
 
  2.9302715
 
 1.14e-01
 
 1.00e+00
  
 
  
 4
 
 186732837
 
 186733060
 
  PDF   STATS   ENSEMBL   UCSC 
 
  224
 
  7
 
 30165
 
 gene;tss
 
 0.02680
 
 0.35192
 
 Not queried
 
  2.9270389
 
 4.08e-03
 
 1.00e+00
  
 
  
 1
 
  45278971
 
  45279349
 
  PDF   STATS   ENSEMBL   UCSC 
 
  379
 
  4
 
 497
 
 gene;island
 
 0.38139
 
 0.09424
 
 Not queried
 
  2.9268778
 
 1.14e-01
 
 1.00e+00
  
 
  
 11
 
  32452038
 
  32452839
 
  PDF   STATS   ENSEMBL   UCSC 
 
  802
 
  8
 
 6548
 
 gene;island
 
 0.81944
 
 0.33938
 
 Not queried
 
  2.9245088
 
 6.22e-04
 
 1.00e+00
  
 
  
 1
 
  38230769
 
  38230887
 
  PDF   STATS   ENSEMBL   UCSC 
 
  119
 
  6
 
 3067
 
 island
 
 0.09610
 
 0.51455
 
 Not queried
 
  2.9240035
 
 2.16e-03
 
 1.00e+00
  
 
  
 2
 
  42719503
 
  42719933
 
  PDF   STATS   ENSEMBL   UCSC 
 
  431
 
  4
 
 22618
 
 gene;island
 
 0.80495
 
 0.33350
 
 Not queried
 
  2.9221629
 
 2.86e-02
 
 1.00e+00
  
 
  
 17
 
  46691820
 
  46692248
 
  PDF   STATS   ENSEMBL   UCSC 
 
  429
 
  5
 
 16625
 
 gene
 
 0.03940
 
 0.25985
 
 Not queried
 
  2.9211041
 
 7.94e-03
 
 1.00e+00
  
 
  
 5
 
  87898489
 
  87898688
 
  PDF   STATS   ENSEMBL   UCSC 
 
  200
 
  4
 
 31617
 
 gene;island
 
 0.13180
 
 0.47794
 
 Not queried
 
  2.9210950
 
 2.00e-01
 
 1.00e+00
  
 
  
 1
 
  29062689
 
  29063076
 
  PDF   STATS   ENSEMBL   UCSC 
 
  388
 
  7
 
 1433
 
 tss;island
 
 0.07939
 
 0.01387
 
 Not queried
 
  2.9206108
 
 1.75e-02
 
 1.00e+00
  
 
  
 11
 
   6291339
 
   6291625
 
  PDF   STATS   ENSEMBL   UCSC 
 
  287
 
  4
 
 6470
 
 gene;island
 
 0.36549
 
 0.79476
 
 Not queried
 
  2.9176630
 
 5.71e-02
 
 1.00e+00
  
 
  
 1
 
 152487844
 
 152488368
 
  PDF   STATS   ENSEMBL   UCSC 
 
  525
 
  8
 
 756
 
 gene;island
 
 0.02565
 
 0.29190
 
 Not queried
 
  2.9142489
 
 1.48e-02
 
 1.00e+00
  
 
  
 X
 
  49687028
 
  49687331
 
  PDF   STATS   ENSEMBL   UCSC 
 
  304
 
 10
 
 41794
 
 island
 
 0.11070
 
 0.01540
 
 Not queried
 
  2.9139169
 
 1.05e-03
 
 1.00e+00
  
 
  
 6
 
  53412803
 
  53413189
 
  PDF   STATS   ENSEMBL   UCSC 
 
  387
 
  5
 
 36153
 
 island
 
 0.02109
 
 0.13960
 
 Not queried
 
  2.9129296
 
 5.56e-02
 
 1.00e+00
  
 
  
 2
 
 132152814
 
 132152960
 
  PDF   STATS   ENSEMBL   UCSC 
 
  147
 
  4
 
 24693
 
 island
 
 0.09925
 
 0.44826
 
 Not queried
 
  2.9121775
 
 2.00e-01
 
 1.00e+00
  
 
  
 15
 
  29407792
 
  29408145
 
  PDF   STATS   ENSEMBL   UCSC 
 
  354
 
  4
 
 13239
 
 gene;island
 
 0.39598
 
 0.80915
 
 Not queried
 
  2.9102528
 
 2.86e-02
 
 1.00e+00
  
 
  
 18
 
  54305957
 
  54306437
 
  PDF   STATS   ENSEMBL   UCSC 
 
  481
 
 10
 
 19254
 
 tss
 
 0.02469
 
 0.13424
 
 Not queried
 
  2.9097684
 
 8.93e-03
 
 1.00e+00
  
 
  
 2
 
  55450577
 
  55450874
 
  PDF   STATS   ENSEMBL   UCSC 
 
  298
 
  4
 
 22649
 
 gene;island
 
 0.01502
 
 0.15236
 
 Not queried
 
  2.9096482
 
 2.86e-02
 
 1.00e+00
  
 
  
 2
 
 109746691
 
 109746754
 
  PDF   STATS   ENSEMBL   UCSC 
 
   64
 
  4
 
 22786
 
 gene;tss;island
 
 0.88949
 
 0.47661
 
 Not queried
 
  2.9082470
 
 2.00e-01
 
 1.00e+00
  
 
  
 X
 
  48693397
 
  48693665
 
  PDF   STATS   ENSEMBL   UCSC 
 
  269
 
  4
 
 40986
 
 gene;island
 
 0.49067
 
 0.18566
 
 Not queried
 
  2.9075632
 
 2.86e-02
 
 1.00e+00
  
 
  
 6
 
  29795815
 
  29796124
 
  PDF   STATS   ENSEMBL   UCSC 
 
  310
 
  9
 
 33633
 
 gene;island
 
 0.02873
 
 0.18162
 
 Not queried
 
  2.9073086
 
 9.39e-02
 
 1.00e+00
  
 
  
 19
 
  33182526
 
  33182834
 
  PDF   STATS   ENSEMBL   UCSC 
 
  309
 
  7
 
 20643
 
 tss;island
 
 0.02750
 
 0.24477
 
 Not queried
 
  2.9065727
 
 6.99e-03
 
 1.00e+00
  
 
  
 3
 
 149374761
 
 149375293
 
  PDF   STATS   ENSEMBL   UCSC 
 
  533
 
  7
 
 27950
 
 gene;island
 
 0.16515
 
 0.02439
 
 Not queried
 
  2.9062742
 
 3.79e-02
 
 1.00e+00
  
 
  
 1
 
 174968745
 
 174968881
 
  PDF   STATS   ENSEMBL   UCSC 
 
  137
 
  4
 
 2099
 
 tss
 
 0.20461
 
 0.02912
 
 Not queried
 
  2.9053789
 
 1.14e-01
 
 1.00e+00
  
 
  
 3
 
  46735315
 
  46735454
 
  PDF   STATS   ENSEMBL   UCSC 
 
  140
 
  5
 
 28236
 
 tss
 
 0.07266
 
 0.38832
 
 Not queried
 
  2.9037979
 
 9.52e-02
 
 1.00e+00
  
 
  
 3
 
  46734995
 
  46735454
 
  PDF   STATS   ENSEMBL   UCSC 
 
  460
 
  9
 
 29014
 
 island
 
 0.05103
 
 0.27711
 
 Not queried
 
  2.9037979
 
 3.99e-03
 
 1.00e+00
  
 
  
 6
 
 110798012
 
 110798323
 
  PDF   STATS   ENSEMBL   UCSC 
 
  312
 
  5
 
 35106
 
 tss;island
 
 0.55913
 
 0.93601
 
 Not queried
 
  2.9036805
 
 5.56e-02
 
 1.00e+00
  
 
  
 1
 
  11898405
 
  11898805
 
  PDF   STATS   ENSEMBL   UCSC 
 
  401
 
  5
 
 227
 
 gene;island
 
 0.16528
 
 0.59704
 
 Not queried
 
  2.9035863
 
 5.56e-02
 
 1.00e+00
  
 
  
 X
 
 153046175
 
 153046894
 
  PDF   STATS   ENSEMBL   UCSC 
 
  720
 
  9
 
 42120
 
 island
 
 0.34260
 
 0.85861
 
 Not queried
 
  2.9034611
 
 7.77e-03
 
 1.00e+00
  
 
  
 17
 
  27346732
 
  27347260
 
  PDF   STATS   ENSEMBL   UCSC 
 
  529
 
  6
 
 18131
 
 island
 
 0.46390
 
 0.86009
 
 Not queried
 
  2.9032796
 
 8.66e-03
 
 1.00e+00
  
 
  
 6
 
  33091111
 
  33092243
 
  PDF   STATS   ENSEMBL   UCSC 
 
 1133
 
 20
 
 34125
 
 gene
 
 0.94655
 
 0.60787
 
 Not queried
 
  2.9023229
 
 1.77e-03
 
 1.00e+00
  
 
  
 2
 
 127963489
 
 127963653
 
  PDF   STATS   ENSEMBL   UCSC 
 
  165
 
  5
 
 23572
 
 tss
 
 0.64478
 
 0.26848
 
 Not queried
 
  2.9019242
 
 7.94e-03
 
 1.00e+00
  
 
  
 2
 
 202484020
 
 202484320
 
  PDF   STATS   ENSEMBL   UCSC 
 
  301
 
  6
 
 23781
 
 tss
 
 0.19298
 
 0.71730
 
 Not queried
 
  2.9014855
 
 2.16e-03
 
 1.00e+00
  
 
  
 1
 
  23763081
 
  23763959
 
  PDF   STATS   ENSEMBL   UCSC 
 
  879
 
  7
 
 313
 
 gene;island
 
 0.16158
 
 0.76055
 
 Not queried
 
  2.9004522
 
 2.62e-02
 
 1.00e+00
  
 
  
 6
 
  10881782
 
  10882336
 
  PDF   STATS   ENSEMBL   UCSC 
 
  555
 
 11
 
 35375
 
 island
 
 0.01716
 
 0.05746
 
 Not queried
 
  2.9003385
 
 5.24e-03
 
 1.00e+00
  
 
  
 13
 
 113540400
 
 113540631
 
  PDF   STATS   ENSEMBL   UCSC 
 
  232
 
  5
 
 11233
 
 gene;island
 
 0.44275
 
 0.85569
 
 Not queried
 
  2.8997384
 
 7.94e-03
 
 1.00e+00
  
 
  
 1
 
 222763258
 
 222763513
 
  PDF   STATS   ENSEMBL   UCSC 
 
  256
 
  8
 
 2262
 
 tss
 
 0.23235
 
 0.03762
 
 Not queried
 
  2.8992592
 
 3.11e-04
 
 1.00e+00
  
 
  
 2
 
 227700458
 
 227700568
 
  PDF   STATS   ENSEMBL   UCSC 
 
  111
 
  6
 
 23890
 
 tss;island
 
 0.23505
 
 0.05419
 
 Not queried
 
  2.8986361
 
 2.16e-03
 
 1.00e+00
  
 
  
 18
 
  49867117
 
  49867488
 
  PDF   STATS   ENSEMBL   UCSC 
 
  372
 
  5
 
 19082
 
 gene;island
 
 0.09268
 
 0.36575
 
 Not queried
 
  2.8985789
 
 5.56e-02
 
 1.00e+00
  
 
  
 1
 
  92012408
 
  92012736
 
  PDF   STATS   ENSEMBL   UCSC 
 
  329
 
  5
 
 3468
 
 island
 
 0.39780
 
 0.79074
 
 Not queried
 
  2.8964113
 
 7.94e-03
 
 1.00e+00
  
 
  
 8
 
 144897555
 
 144897576
 
  PDF   STATS   ENSEMBL   UCSC 
 
   22
 
  5
 
 39897
 
 tss
 
 0.47068
 
 0.10672
 
 Not queried
 
  2.8958428
 
 7.94e-03
 
 1.00e+00
  
 
  
 8
 
 144897297
 
 144897576
 
  PDF   STATS   ENSEMBL   UCSC 
 
  280
 
  7
 
 40625
 
 island
 
 0.47947
 
 0.10672
 
 Not queried
 
  2.8958428
 
 5.83e-04
 
 1.00e+00
  
 
  
 16
 
  31548640
 
  31548908
 
  PDF   STATS   ENSEMBL   UCSC 
 
  269
 
  4
 
 15849
 
 island
 
 0.90562
 
 0.50586
 
 Not queried
 
  2.8953073
 
 5.71e-02
 
 1.00e+00
  
 
  
 11
 
  77185354
 
  77185670
 
  PDF   STATS   ENSEMBL   UCSC 
 
  317
 
  4
 
 7563
 
 tss;island
 
 0.04261
 
 0.30557
 
 Not queried
 
  2.8947598
 
 5.71e-02
 
 1.00e+00
  
 
  
 15
 
 101817896
 
 101818008
 
  PDF   STATS   ENSEMBL   UCSC 
 
  113
 
  5
 
 13905
 
 tss;island
 
 0.01232
 
 0.02375
 
 Not queried
 
  2.8938811
 
 2.22e-01
 
 1.00e+00
  
 
  
 7
 
 127672458
 
 127672658
 
  PDF   STATS   ENSEMBL   UCSC 
 
  201
 
  4
 
 37148
 
 gene;island
 
 0.18099
 
 0.03295
 
 Not queried
 
  2.8927100
 
 2.86e-02
 
 1.00e+00
  
 
  
 12
 
  20094141
 
  20094385
 
  PDF   STATS   ENSEMBL   UCSC 
 
  245
 
  4
 
 10249
 
 island
 
 0.59124
 
 0.92862
 
 Not queried
 
  2.8918367
 
 5.71e-02
 
 1.00e+00
  
 
  
 6
 
  29869838
 
  29870062
 
  PDF   STATS   ENSEMBL   UCSC 
 
  225
 
  5
 
 33638
 
 gene
 
 0.80605
 
 0.47011
 
 Not queried
 
  2.8917948
 
 3.17e-02
 
 1.00e+00
  
 
  
 6
 
  29711249
 
  29711588
 
  PDF   STATS   ENSEMBL   UCSC 
 
  340
 
  4
 
 33627
 
 gene
 
 0.69869
 
 0.26416
 
 Not queried
 
  2.8914619
 
 2.86e-02
 
 1.00e+00
  
 
  
 6
 
 108444791
 
 108444920
 
  PDF   STATS   ENSEMBL   UCSC 
 
  130
 
  4
 
 36330
 
 island
 
 0.85913
 
 0.58373
 
 Not queried
 
  2.8907092
 
 2.86e-02
 
 1.00e+00
  
 
  
 7
 
  96627602
 
  96627821
 
  PDF   STATS   ENSEMBL   UCSC 
 
  220
 
  4
 
 37037
 
 gene;island
 
 0.35452
 
 0.08058
 
 Not queried
 
  2.8893364
 
 2.86e-02
 
 1.00e+00
  
 
  
 2
 
  19558368
 
  19558569
 
  PDF   STATS   ENSEMBL   UCSC 
 
  202
 
  5
 
 24064
 
 island
 
 0.27815
 
 0.04258
 
 Not queried
 
  2.8875152
 
 1.59e-02
 
 1.00e+00
  
 
  
 20
 
  42544648
 
  42544794
 
  PDF   STATS   ENSEMBL   UCSC 
 
  147
 
  8
 
 25384
 
 gene;island
 
 0.24481
 
 0.01828
 
 Not queried
 
  2.8868630
 
 2.07e-02
 
 1.00e+00
  
 
  
 1
 
  67600172
 
  67600835
 
  PDF   STATS   ENSEMBL   UCSC 
 
  664
 
  8
 
 3357
 
 island
 
 0.11250
 
 0.52672
 
 Not queried
 
  2.8860779
 
 2.07e-02
 
 1.00e+00
  
 
  
 12
 
  54321992
 
  54322396
 
  PDF   STATS   ENSEMBL   UCSC 
 
  405
 
  4
 
 10449
 
 island
 
 0.04327
 
 0.20766
 
 Not queried
 
  2.8858272
 
 2.00e-01
 
 1.00e+00
  
 
  
 4
 
  57522493
 
  57523055
 
  PDF   STATS   ENSEMBL   UCSC 
 
  563
 
  9
 
 29965
 
 gene;island
 
 0.59810
 
 0.14641
 
 Not queried
 
  2.8853756
 
 1.88e-02
 
 1.00e+00
  
 
  
 5
 
 180542154
 
 180542623
 
  PDF   STATS   ENSEMBL   UCSC 
 
  470
 
  8
 
 33451
 
 island
 
 0.19909
 
 0.54697
 
 Not queried
 
  2.8850692
 
 1.48e-02
 
 1.00e+00
  
 
  
 11
 
  61519687
 
  61520084
 
  PDF   STATS   ENSEMBL   UCSC 
 
  398
 
  5
 
 7316
 
 tss
 
 0.22110
 
 0.03701
 
 Not queried
 
  2.8847605
 
 5.48e-01
 
 1.00e+00
  
 
  
 19
 
   2543602
 
   2543877
 
  PDF   STATS   ENSEMBL   UCSC 
 
  276
 
  5
 
 19656
 
 gene;island
 
 0.03591
 
 0.11502
 
 Not queried
 
  2.8830697
 
 5.56e-02
 
 1.00e+00
  
 
  
 X
 
 134124839
 
 134125259
 
  PDF   STATS   ENSEMBL   UCSC 
 
  421
 
  9
 
 42045
 
 island
 
 0.10769
 
 0.61884
 
 Not queried
 
  2.8828486
 
 2.88e-04
 
 1.00e+00
  
 
  
 15
 
 101629063
 
 101629375
 
  PDF   STATS   ENSEMBL   UCSC 
 
  313
 
  4
 
 14486
 
 island
 
 0.12414
 
 0.51067
 
 Not queried
 
  2.8824108
 
 2.00e-01
 
 1.00e+00
  
 
  
 22
 
  21369411
 
  21369777
 
  PDF   STATS   ENSEMBL   UCSC 
 
  367
 
  4
 
 26949
 
 tss;island
 
 0.68551
 
 0.29482
 
 Not queried
 
  2.8822274
 
 2.00e-01
 
 1.00e+00
  
 
  
 2
 
 242702553
 
 242702932
 
  PDF   STATS   ENSEMBL   UCSC 
 
  380
 
  4
 
 23107
 
 gene
 
 0.70259
 
 0.94079
 
 Not queried
 
  2.8818319
 
 2.00e-01
 
 1.00e+00
  
 
  
 3
 
 184972080
 
 184972153
 
  PDF   STATS   ENSEMBL   UCSC 
 
   74
 
  4
 
 28723
 
 tss;island
 
 0.16035
 
 0.60478
 
 Not queried
 
  2.8817675
 
 2.86e-02
 
 1.00e+00
  
 
  
 20
 
  32856747
 
  32857227
 
  PDF   STATS   ENSEMBL   UCSC 
 
  481
 
  6
 
 26067
 
 island
 
 0.61666
 
 0.93531
 
 Not queried
 
  2.8810726
 
 2.16e-03
 
 1.00e+00
  
 
  
 11
 
  67373625
 
  67374213
 
  PDF   STATS   ENSEMBL   UCSC 
 
  589
 
  8
 
 7477
 
 tss
 
 0.60138
 
 0.22630
 
 Not queried
 
  2.8807050
 
 1.30e-01
 
 1.00e+00
  
 
  
 17
 
   4545074
 
   4545157
 
  PDF   STATS   ENSEMBL   UCSC 
 
   84
 
  4
 
 16994
 
 tss
 
 0.61187
 
 0.15466
 
 Not queried
 
  2.8800637
 
 5.71e-02
 
 1.00e+00
  
 
  
 1
 
  78511140
 
  78512129
 
  PDF   STATS   ENSEMBL   UCSC 
 
  990
 
 12
 
 3395
 
 island
 
 0.27574
 
 0.06497
 
 Not queried
 
  2.8791629
 
 6.81e-03
 
 1.00e+00
  
 
  
 8
 
 143859143
 
 143859410
 
  PDF   STATS   ENSEMBL   UCSC 
 
  268
 
  5
 
 39406
 
 gene;tss;island
 
 0.31852
 
 0.05979
 
 Not queried
 
  2.8777452
 
 3.17e-02
 
 1.00e+00
  
 
  
 12
 
  72233240
 
  72233472
 
  PDF   STATS   ENSEMBL   UCSC 
 
  233
 
  8
 
 9850
 
 tss
 
 0.25059
 
 0.08339
 
 Not queried
 
  2.8770069
 
 6.50e-02
 
 1.00e+00
  
 
  
 X
 
  38662916
 
  38663177
 
  PDF   STATS   ENSEMBL   UCSC 
 
  262
 
  8
 
 40964
 
 gene;island
 
 0.23678
 
 0.03855
 
 Not queried
 
  2.8763174
 
 4.99e-02
 
 1.00e+00
  
 
  
 X
 
  38662916
 
  38663062
 
  PDF   STATS   ENSEMBL   UCSC 
 
  147
 
  6
 
 41260
 
 tss
 
 0.25249
 
 0.04662
 
 Not queried
 
  2.8763174
 
 9.31e-02
 
 1.00e+00
  
 
  
 1
 
  16345293
 
  16345491
 
  PDF   STATS   ENSEMBL   UCSC 
 
  199
 
  4
 
 1275
 
 tss
 
 0.26782
 
 0.67698
 
 Not queried
 
  2.8753448
 
 2.86e-02
 
 1.00e+00
  
 
  
 2
 
 118942841
 
 118943184
 
  PDF   STATS   ENSEMBL   UCSC 
 
  344
 
  5
 
 24603
 
 island
 
 0.05412
 
 0.25947
 
 Not queried
 
  2.8743905
 
 7.94e-03
 
 1.00e+00
  
 
  
 12
 
  56109593
 
  56109799
 
  PDF   STATS   ENSEMBL   UCSC 
 
  207
 
  5
 
 9743
 
 tss
 
 0.72552
 
 0.11976
 
 Not queried
 
  2.8736552
 
 3.10e-01
 
 1.00e+00
  
 
  
 6
 
  50692150
 
  50692762
 
  PDF   STATS   ENSEMBL   UCSC 
 
  613
 
  6
 
 34299
 
 gene;island
 
 0.14594
 
 0.55700
 
 Not queried
 
  2.8718224
 
 6.49e-02
 
 1.00e+00
  
 
  
 X
 
 153602509
 
 153602918
 
  PDF   STATS   ENSEMBL   UCSC 
 
  410
 
  4
 
 41155
 
 gene
 
 0.04816
 
 0.29811
 
 Not queried
 
  2.8713454
 
 5.71e-02
 
 1.00e+00
  
 
  
 3
 
 118663470
 
 118663697
 
  PDF   STATS   ENSEMBL   UCSC 
 
  228
 
  4
 
 27861
 
 gene
 
 0.43276
 
 0.88758
 
 Not queried
 
  2.8713234
 
 1.14e-01
 
 1.00e+00
  
 
  
 6
 
 108488751
 
 108489064
 
  PDF   STATS   ENSEMBL   UCSC 
 
  314
 
  4
 
 34373
 
 gene;island
 
 0.42937
 
 0.08144
 
 Not queried
 
  2.8712744
 
 2.86e-02
 
 1.00e+00
  
 
  
 2
 
  87568958
 
  87569447
 
  PDF   STATS   ENSEMBL   UCSC 
 
  490
 
  4
 
 24462
 
 island
 
 0.29709
 
 0.66673
 
 Not queried
 
  2.8696128
 
 1.14e-01
 
 1.00e+00
  
 
  
 22
 
  31317764
 
  31318240
 
  PDF   STATS   ENSEMBL   UCSC 
 
  477
 
  8
 
 27028
 
 tss
 
 0.58726
 
 0.84752
 
 Not queried
 
  2.8676014
 
 2.07e-02
 
 1.00e+00
  
 
  
 5
 
 180527369
 
 180527697
 
  PDF   STATS   ENSEMBL   UCSC 
 
  329
 
  4
 
 33450
 
 island
 
 0.05877
 
 0.41852
 
 Not queried
 
  2.8672656
 
 2.86e-02
 
 1.00e+00
  
 
  
 15
 
  25199028
 
  25199270
 
  PDF   STATS   ENSEMBL   UCSC 
 
  243
 
  4
 
 13228
 
 gene;tss;island
 
 0.76759
 
 0.20804
 
 Not queried
 
  2.8672649
 
 1.14e-01
 
 1.00e+00
  
 
  
 17
 
   7348316
 
   7348399
 
  PDF   STATS   ENSEMBL   UCSC 
 
   84
 
  6
 
 17051
 
 tss
 
 0.18265
 
 0.02925
 
 Not queried
 
  2.8661753
 
 8.66e-03
 
 1.00e+00
  
 
  
 17
 
   7348316
 
   7348490
 
  PDF   STATS   ENSEMBL   UCSC 
 
  175
 
  8
 
 17917
 
 island
 
 0.18265
 
 0.01910
 
 Not queried
 
  2.8661753
 
 2.95e-03
 
 1.00e+00
  
 
  
 14
 
  21769105
 
  21769379
 
  PDF   STATS   ENSEMBL   UCSC 
 
  275
 
  5
 
 11983
 
 gene;island
 
 0.14219
 
 0.61835
 
 Not queried
 
  2.8658758
 
 3.17e-02
 
 1.00e+00
  
 
  
 3
 
  36902435
 
  36902745
 
  PDF   STATS   ENSEMBL   UCSC 
 
  311
 
  4
 
 27673
 
 gene
 
 0.88943
 
 0.54699
 
 Not queried
 
  2.8631734
 
 5.71e-02
 
 1.00e+00
  
 
  
 7
 
  99067116
 
  99067368
 
  PDF   STATS   ENSEMBL   UCSC 
 
  253
 
  5
 
 38669
 
 island
 
 0.23151
 
 0.03836
 
 Not queried
 
  2.8606399
 
 7.94e-03
 
 1.00e+00
  
 
  
 1
 
  27286832
 
  27287541
 
  PDF   STATS   ENSEMBL   UCSC 
 
  710
 
  7
 
 2901
 
 island
 
 0.01821
 
 0.06936
 
 Not queried
 
  2.8594470
 
 1.28e-01
 
 1.00e+00
  
 
  
 7
 
  45927735
 
  45928326
 
  PDF   STATS   ENSEMBL   UCSC 
 
  592
 
  8
 
 38413
 
 island
 
 0.20082
 
 0.69311
 
 Not queried
 
  2.8584933
 
 4.66e-03
 
 1.00e+00
  
 
  
 5
 
 176543790
 
 176544135
 
  PDF   STATS   ENSEMBL   UCSC 
 
  346
 
  5
 
 33377
 
 island
 
 0.55525
 
 0.89006
 
 Not queried
 
  2.8581179
 
 7.94e-03
 
 1.00e+00
  
 
  
 1
 
 220921216
 
 220922217
 
  PDF   STATS   ENSEMBL   UCSC 
 
 1002
 
 14
 
 4206
 
 island
 
 0.07899
 
 0.01698
 
 Not queried
 
  2.8569793
 
 1.18e-03
 
 1.00e+00
  
 
  
 11
 
 108422663
 
 108423325
 
  PDF   STATS   ENSEMBL   UCSC 
 
  663
 
  8
 
 6871
 
 gene
 
 0.41066
 
 0.78887
 
 Not queried
 
  2.8567122
 
 6.99e-03
 
 1.00e+00
  
 
  
 6
 
 167104601
 
 167104926
 
  PDF   STATS   ENSEMBL   UCSC 
 
  326
 
  5
 
 34488
 
 gene
 
 0.20700
 
 0.65403
 
 Not queried
 
  2.8563429
 
 3.10e-01
 
 1.00e+00
  
 
  
 2
 
  71205052
 
  71205685
 
  PDF   STATS   ENSEMBL   UCSC 
 
  634
 
  7
 
 24353
 
 island
 
 0.57256
 
 0.04777
 
 Not queried
 
  2.8549805
 
 5.30e-02
 
 1.00e+00
  
 
  
 1
 
    999599
 
    999888
 
  PDF   STATS   ENSEMBL   UCSC 
 
  290
 
  5
 
 2394
 
 island
 
 0.05527
 
 0.35797
 
 Not queried
 
  2.8549665
 
 2.22e-01
 
 1.00e+00
  
 
  
 X
 
  48776465
 
  48776812
 
  PDF   STATS   ENSEMBL   UCSC 
 
  348
 
  7
 
 41301
 
 tss
 
 0.12728
 
 0.01978
 
 Not queried
 
  2.8534372
 
 2.62e-02
 
 1.00e+00
  
 
  
 7
 
  55224743
 
  55225183
 
  PDF   STATS   ENSEMBL   UCSC 
 
  441
 
  7
 
 36955
 
 gene
 
 0.76632
 
 0.38718
 
 Not queried
 
  2.8513347
 
 6.99e-03
 
 1.00e+00
  
 
  
 2
 
  28114016
 
  28114462
 
  PDF   STATS   ENSEMBL   UCSC 
 
  447
 
  8
 
 22599
 
 gene;tss;island
 
 0.05426
 
 0.35665
 
 Not queried
 
  2.8492850
 
 4.99e-02
 
 1.00e+00
  
 
  
 19
 
   3097565
 
   3097728
 
  PDF   STATS   ENSEMBL   UCSC 
 
  164
 
  4
 
 19669
 
 gene;island
 
 0.65140
 
 0.94154
 
 Not queried
 
  2.8491232
 
 2.86e-02
 
 1.00e+00
  
 
  
 7
 
    814391
 
    814759
 
  PDF   STATS   ENSEMBL   UCSC 
 
  369
 
  5
 
 36660
 
 gene;island
 
 0.61667
 
 0.93002
 
 Not queried
 
  2.8479976
 
 2.22e-01
 
 1.00e+00
  
 
  
 17
 
  72306008
 
  72306215
 
  PDF   STATS   ENSEMBL   UCSC 
 
  208
 
  4
 
 16714
 
 gene
 
 0.14778
 
 0.55386
 
 Not queried
 
  2.8474278
 
 2.86e-02
 
 1.00e+00
  
 
  
 21
 
  46706579
 
  46707012
 
  PDF   STATS   ENSEMBL   UCSC 
 
  434
 
  4
 
 26424
 
 gene;tss;island
 
 0.40656
 
 0.09029
 
 Not queried
 
  2.8473981
 
 4.86e-01
 
 1.00e+00
  
 
  
 17
 
  33734481
 
  33734847
 
  PDF   STATS   ENSEMBL   UCSC 
 
  367
 
  4
 
 18180
 
 island
 
 0.87128
 
 0.40149
 
 Not queried
 
  2.8464802
 
 5.71e-02
 
 1.00e+00
  
 
  
 19
 
  50831762
 
  50832037
 
  PDF   STATS   ENSEMBL   UCSC 
 
  276
 
  5
 
 20136
 
 gene;island
 
 0.05679
 
 0.21471
 
 Not queried
 
  2.8453679
 
 1.51e-01
 
 1.00e+00
  
 
  
 19
 
  12175107
 
  12175540
 
  PDF   STATS   ENSEMBL   UCSC 
 
  434
 
  7
 
 20452
 
 tss
 
 0.04795
 
 0.36617
 
 Not queried
 
  2.8453270
 
 2.59e-01
 
 1.00e+00
  
 
  
 3
 
 119187426
 
 119187686
 
  PDF   STATS   ENSEMBL   UCSC 
 
  261
 
  4
 
 28478
 
 tss;island
 
 0.63805
 
 0.22983
 
 Not queried
 
  2.8448132
 
 2.00e-01
 
 1.00e+00
  
 
  
 X
 
  68348465
 
  68348839
 
  PDF   STATS   ENSEMBL   UCSC 
 
  375
 
  6
 
 41839
 
 island
 
 0.43324
 
 0.85785
 
 Not queried
 
  2.8435357
 
 2.16e-03
 
 1.00e+00
  
 
  
 17
 
  76100911
 
  76101391
 
  PDF   STATS   ENSEMBL   UCSC 
 
  481
 
  5
 
 16777
 
 gene;island
 
 0.62919
 
 0.79580
 
 Not queried
 
  2.8429115
 
 1.51e-01
 
 1.00e+00
  
 
  
 4
 
 111397134
 
 111397220
 
  PDF   STATS   ENSEMBL   UCSC 
 
   87
 
  5
 
 30500
 
 tss
 
 0.40633
 
 0.89301
 
 Not queried
 
  2.8398095
 
 3.17e-02
 
 1.00e+00
  
 
  
 12
 
  46767665
 
  46768016
 
  PDF   STATS   ENSEMBL   UCSC 
 
  352
 
  6
 
 9633
 
 tss;island
 
 0.31130
 
 0.03452
 
 Not queried
 
  2.8393473
 
 8.66e-03
 
 1.00e+00
  
 
  
 15
 
  45406327
 
  45406939
 
  PDF   STATS   ENSEMBL   UCSC 
 
  613
 
 13
 
 13617
 
 tss;island
 
 0.12429
 
 0.56339
 
 Not queried
 
  2.8366654
 
 2.97e-04
 
 1.00e+00
  
 
  
 19
 
  46932069
 
  46932308
 
  PDF   STATS   ENSEMBL   UCSC 
 
  240
 
  4
 
 22137
 
 island
 
 0.26495
 
 0.04342
 
 Not queried
 
  2.8355402
 
 5.71e-02
 
 1.00e+00
  
 
  
 20
 
  20433062
 
  20433328
 
  PDF   STATS   ENSEMBL   UCSC 
 
  267
 
  4
 
 25335
 
 gene;island
 
 0.02994
 
 0.14958
 
 Not queried
 
  2.8354515
 
 1.14e-01
 
 1.00e+00
  
 
  
 14
 
  63568602
 
  63568809
 
  PDF   STATS   ENSEMBL   UCSC 
 
  208
 
  4
 
 12419
 
 tss
 
 0.89675
 
 0.58669
 
 Not queried
 
  2.8343367
 
 2.86e-02
 
 1.00e+00
  
 
  
 10
 
 127584967
 
 127585090
 
  PDF   STATS   ENSEMBL   UCSC 
 
  124
 
  4
 
 5414
 
 tss
 
 0.05914
 
 0.48903
 
 Not queried
 
  2.8342496
 
 1.14e-01
 
 1.00e+00
  
 
  
 1
 
 117753591
 
 117753741
 
  PDF   STATS   ENSEMBL   UCSC 
 
  151
 
  4
 
 1866
 
 tss
 
 0.28531
 
 0.72948
 
 Not queried
 
  2.8338896
 
 2.86e-02
 
 1.00e+00
  
 
  
 22
 
  17680477
 
  17680822
 
  PDF   STATS   ENSEMBL   UCSC 
 
  346
 
  9
 
 26739
 
 gene
 
 0.24197
 
 0.04015
 
 Not queried
 
  2.8326912
 
 2.44e-02
 
 1.00e+00
  
 
  
 22
 
  17680545
 
  17680822
 
  PDF   STATS   ENSEMBL   UCSC 
 
  278
 
  7
 
 26901
 
 tss
 
 0.26968
 
 0.05422
 
 Not queried
 
  2.8326912
 
 1.75e-02
 
 1.00e+00
  
 
  
 6
 
  32005786
 
  32006079
 
  PDF   STATS   ENSEMBL   UCSC 
 
  294
 
  4
 
 34796
 
 tss
 
 0.56494
 
 0.10336
 
 Not queried
 
  2.8325754
 
 5.71e-02
 
 1.00e+00
  
 
  
 3
 
   9811714
 
   9811990
 
  PDF   STATS   ENSEMBL   UCSC 
 
  277
 
  7
 
 28087
 
 tss;island
 
 0.18225
 
 0.02497
 
 Not queried
 
  2.8322013
 
 4.08e-03
 
 1.00e+00
  
 
  
 19
 
  49944488
 
  49944964
 
  PDF   STATS   ENSEMBL   UCSC 
 
  477
 
  8
 
 22231
 
 island
 
 0.04232
 
 0.25452
 
 Not queried
 
  2.8302302
 
 6.22e-04
 
 1.00e+00
  
 
  
 8
 
  22960177
 
  22960427
 
  PDF   STATS   ENSEMBL   UCSC 
 
  251
 
  6
 
 39533
 
 tss
 
 0.25711
 
 0.56908
 
 Not queried
 
  2.8300756
 
 1.32e-01
 
 1.00e+00
  
 
  
 1
 
  38230827
 
  38230887
 
  PDF   STATS   ENSEMBL   UCSC 
 
   61
 
  4
 
 1504
 
 tss
 
 0.09610
 
 0.48629
 
 Not queried
 
  2.8298915
 
 2.86e-02
 
 1.00e+00
  
 
  
 11
 
  75379432
 
  75379624
 
  PDF   STATS   ENSEMBL   UCSC 
 
  193
 
  7
 
 8642
 
 island
 
 0.03995
 
 0.21001
 
 Not queried
 
  2.8298240
 
 1.17e-03
 
 1.00e+00
  
 
  
 14
 
  95234658
 
  95235127
 
  PDF   STATS   ENSEMBL   UCSC 
 
  470
 
  7
 
 12168
 
 gene;island
 
 0.09071
 
 0.01682
 
 Not queried
 
  2.8292097
 
 1.75e-02
 
 1.00e+00
  
 
  
 2
 
 220119179
 
 220119398
 
  PDF   STATS   ENSEMBL   UCSC 
 
  220
 
  4
 
 22994
 
 gene;tss;island
 
 0.03495
 
 0.17929
 
 Not queried
 
  2.8280208
 
 2.86e-02
 
 1.00e+00
  
 
  
 10
 
  95517382
 
  95517562
 
  PDF   STATS   ENSEMBL   UCSC 
 
  181
 
  5
 
 5225
 
 tss
 
 0.60550
 
 0.17776
 
 Not queried
 
  2.8277331
 
 1.59e-02
 
 1.00e+00
  
 
  
 16
 
  66586528
 
  66586745
 
  PDF   STATS   ENSEMBL   UCSC 
 
  218
 
  4
 
 14749
 
 gene
 
 0.21074
 
 0.03403
 
 Not queried
 
  2.8275828
 
 2.86e-02
 
 1.00e+00
  
 
  
 2
 
 177017331
 
 177017797
 
  PDF   STATS   ENSEMBL   UCSC 
 
  467
 
  6
 
 22902
 
 gene;island
 
 0.81117
 
 0.30929
 
 Not queried
 
  2.8247431
 
 2.16e-03
 
 1.00e+00
  
 
  
 5
 
  92938149
 
  92938402
 
  PDF   STATS   ENSEMBL   UCSC 
 
  254
 
  4
 
 32900
 
 island
 
 0.70119
 
 0.21067
 
 Not queried
 
  2.8241453
 
 2.86e-02
 
 1.00e+00
  
 
  
 2
 
 242749925
 
 242750102
 
  PDF   STATS   ENSEMBL   UCSC 
 
  178
 
  5
 
 23956
 
 tss
 
 0.55762
 
 0.91132
 
 Not queried
 
  2.8236161
 
 9.52e-02
 
 1.00e+00
  
 
  
 X
 
  49687028
 
  49687176
 
  PDF   STATS   ENSEMBL   UCSC 
 
  149
 
  8
 
 41318
 
 tss
 
 0.09051
 
 0.01540
 
 Not queried
 
  2.8232908
 
 1.48e-02
 
 1.00e+00
  
 
  
 6
 
  33160250
 
  33160604
 
  PDF   STATS   ENSEMBL   UCSC 
 
  355
 
  9
 
 34825
 
 tss
 
 0.09067
 
 0.38213
 
 Not queried
 
  2.8220399
 
 2.88e-04
 
 1.00e+00
  
 
  
 1
 
 236227410
 
 236227686
 
  PDF   STATS   ENSEMBL   UCSC 
 
  277
 
  6
 
 1093
 
 gene;island
 
 0.24889
 
 0.04262
 
 Not queried
 
  2.8218291
 
 8.66e-03
 
 1.00e+00
  
 
  
 6
 
  72298242
 
  72298704
 
  PDF   STATS   ENSEMBL   UCSC 
 
  463
 
  5
 
 36182
 
 island
 
 0.83102
 
 0.42605
 
 Not queried
 
  2.8203279
 
 7.94e-03
 
 1.00e+00
  
 
  
 X
 
  48769307
 
  48769670
 
  PDF   STATS   ENSEMBL   UCSC 
 
  364
 
  7
 
 41300
 
 tss;island
 
 0.14866
 
 0.57461
 
 Not queried
 
  2.8202572
 
 5.83e-04
 
 1.00e+00
  
 
  
 14
 
  37117706
 
  37117947
 
  PDF   STATS   ENSEMBL   UCSC 
 
  242
 
  4
 
 12761
 
 island
 
 0.01967
 
 0.13218
 
 Not queried
 
  2.8198551
 
 2.86e-02
 
 1.00e+00
  
 
  
 8
 
   6693247
 
   6693540
 
  PDF   STATS   ENSEMBL   UCSC 
 
  294
 
  6
 
 39476
 
 tss
 
 0.36751
 
 0.79013
 
 Not queried
 
  2.8164794
 
 6.49e-02
 
 1.00e+00
  
 
  
 1
 
  46632363
 
  46632871
 
  PDF   STATS   ENSEMBL   UCSC 
 
  509
 
  7
 
 3207
 
 island
 
 0.38397
 
 0.81440
 
 Not queried
 
  2.8154877
 
 2.33e-03
 
 1.00e+00
  
 
  
 7
 
   4832112
 
   4832535
 
  PDF   STATS   ENSEMBL   UCSC 
 
  424
 
  5
 
 38137
 
 island
 
 0.88377
 
 0.53556
 
 Not queried
 
  2.8153209
 
 7.94e-03
 
 1.00e+00
  
 
  
 15
 
  40697419
 
  40697634
 
  PDF   STATS   ENSEMBL   UCSC 
 
  216
 
  4
 
 13560
 
 tss
 
 0.15897
 
 0.56920
 
 Not queried
 
  2.8118858
 
 2.00e-01
 
 1.00e+00
  
 
  
 4
 
 122685352
 
 122685675
 
  PDF   STATS   ENSEMBL   UCSC 
 
  324
 
  6
 
 30066
 
 gene;tss;island
 
 0.46143
 
 0.05760
 
 Not queried
 
  2.8112749
 
 9.31e-02
 
 1.00e+00
  
 
  
 16
 
  88730291
 
  88730494
 
  PDF   STATS   ENSEMBL   UCSC 
 
  204
 
  4
 
 14879
 
 gene;tss;island
 
 0.29995
 
 0.04320
 
 Not queried
 
  2.8100490
 
 4.86e-01
 
 1.00e+00
  
 
  
 14
 
  47120795
 
  47121158
 
  PDF   STATS   ENSEMBL   UCSC 
 
  364
 
  9
 
 12789
 
 island
 
 0.48001
 
 0.85985
 
 Not queried
 
  2.8073586
 
 2.88e-04
 
 1.00e+00
  
 
  
 15
 
  74537819
 
  74538030
 
  PDF   STATS   ENSEMBL   UCSC 
 
  212
 
  4
 
 13392
 
 gene;island
 
 0.41880
 
 0.79177
 
 Not queried
 
  2.8068447
 
 2.86e-02
 
 1.00e+00
  
 
  
 7
 
  56183885
 
  56184217
 
  PDF   STATS   ENSEMBL   UCSC 
 
  333
 
  4
 
 38448
 
 island
 
 0.29098
 
 0.75972
 
 Not queried
 
  2.8061753
 
 2.86e-02
 
 1.00e+00
  
 
  
 10
 
  14051636
 
  14052028
 
  PDF   STATS   ENSEMBL   UCSC 
 
  393
 
  6
 
 4495
 
 gene
 
 0.38745
 
 0.77752
 
 Not queried
 
  2.8050237
 
 2.16e-03
 
 1.00e+00
  
 
  
 5
 
  41870796
 
  41871189
 
  PDF   STATS   ENSEMBL   UCSC 
 
  394
 
  9
 
 31979
 
 tss
 
 0.05395
 
 0.35458
 
 Not queried
 
  2.8047884
 
 6.25e-02
 
 1.00e+00
  
 
  
 7
 
  89783435
 
  89783976
 
  PDF   STATS   ENSEMBL   UCSC 
 
  542
 
 10
 
 38581
 
 island
 
 0.03613
 
 0.23078
 
 Not queried
 
  2.8039012
 
 3.89e-03
 
 1.00e+00
  
 
  
 10
 
  23216637
 
  23216847
 
  PDF   STATS   ENSEMBL   UCSC 
 
  211
 
  6
 
 4969
 
 tss
 
 0.04032
 
 0.32995
 
 Not queried
 
  2.8037442
 
 4.33e-03
 
 1.00e+00
  
 
  
 16
 
  23724774
 
  23724943
 
  PDF   STATS   ENSEMBL   UCSC 
 
  170
 
  6
 
 15736
 
 island
 
 0.76324
 
 0.37646
 
 Not queried
 
  2.8030336
 
 2.16e-03
 
 1.00e+00
  
 
  
 2
 
 202901288
 
 202901470
 
  PDF   STATS   ENSEMBL   UCSC 
 
  183
 
  4
 
 22955
 
 gene;island
 
 0.24029
 
 0.04285
 
 Not queried
 
  2.8013162
 
 2.86e-02
 
 1.00e+00
  
 
  
 22
 
  32767005
 
  32767162
 
  PDF   STATS   ENSEMBL   UCSC 
 
  158
 
  4
 
 27049
 
 tss
 
 0.88401
 
 0.56476
 
 Not queried
 
  2.7982356
 
 2.86e-02
 
 1.00e+00
  
 
  
 8
 
  74005537
 
  74005813
 
  PDF   STATS   ENSEMBL   UCSC 
 
  277
 
  8
 
 39703
 
 tss;island
 
 0.06077
 
 0.34848
 
 Not queried
 
  2.7977375
 
 6.22e-04
 
 1.00e+00
  
 
  
 6
 
  41620951
 
  41621322
 
  PDF   STATS   ENSEMBL   UCSC 
 
  372
 
  6
 
 34266
 
 gene;island
 
 0.27708
 
 0.72727
 
 Not queried
 
  2.7976902
 
 2.16e-03
 
 1.00e+00
  
 
  
 6
 
  62996627
 
  62996702
 
  PDF   STATS   ENSEMBL   UCSC 
 
   76
 
  4
 
 34989
 
 tss;island
 
 0.06769
 
 0.35150
 
 Not queried
 
  2.7975878
 
 2.00e-01
 
 1.00e+00
  
 
  
 6
 
  27100370
 
  27101279
 
  PDF   STATS   ENSEMBL   UCSC 
 
  910
 
 13
 
 34647
 
 tss;island
 
 0.01201
 
 0.06692
 
 Not queried
 
  2.7961976
 
 7.24e-03
 
 1.00e+00
  
 
  
 19
 
  49250180
 
  49250561
 
  PDF   STATS   ENSEMBL   UCSC 
 
  382
 
  5
 
 20890
 
 tss
 
 0.07375
 
 0.02060
 
 Not queried
 
  2.7945302
 
 7.94e-03
 
 1.00e+00
  
 
  
 18
 
  67069959
 
  67070461
 
  PDF   STATS   ENSEMBL   UCSC 
 
  503
 
  6
 
 19090
 
 gene;island
 
 0.11379
 
 0.49385
 
 Not queried
 
  2.7942479
 
 4.11e-02
 
 1.00e+00
  
 
  
 12
 
  54446019
 
  54446576
 
  PDF   STATS   ENSEMBL   UCSC 
 
  558
 
 10
 
 9136
 
 gene;island
 
 0.88256
 
 0.37666
 
 Not queried
 
  2.7942477
 
 2.09e-03
 
 1.00e+00
  
 
  
 16
 
    419800
 
    420230
 
  PDF   STATS   ENSEMBL   UCSC 
 
  431
 
  5
 
 14499
 
 gene;island
 
 0.75333
 
 0.27275
 
 Not queried
 
  2.7926855
 
 1.51e-01
 
 1.00e+00
  
 
  
 12
 
   5936877
 
   5937105
 
  PDF   STATS   ENSEMBL   UCSC 
 
  229
 
  4
 
 8996
 
 gene;island
 
 0.85374
 
 0.46841
 
 Not queried
 
  2.7924415
 
 2.86e-02
 
 1.00e+00
  
 
  
 3
 
  44726891
 
  44727195
 
  PDF   STATS   ENSEMBL   UCSC 
 
  305
 
  5
 
 28996
 
 island
 
 0.08802
 
 0.04892
 
 Not queried
 
  2.7918643
 
 9.52e-02
 
 1.00e+00
  
 
  
 11
 
 133800685
 
 133800913
 
  PDF   STATS   ENSEMBL   UCSC 
 
  229
 
  4
 
 6966
 
 gene;island
 
 0.47766
 
 0.73445
 
 Not queried
 
  2.7916860
 
 2.86e-02
 
 1.00e+00
  
 
  
 19
 
  46094683
 
  46094912
 
  PDF   STATS   ENSEMBL   UCSC 
 
  230
 
  4
 
 20066
 
 gene;island
 
 0.42487
 
 0.70213
 
 Not queried
 
  2.7905940
 
 3.43e-01
 
 1.00e+00
  
 
  
 4
 
   9782891
 
   9783246
 
  PDF   STATS   ENSEMBL   UCSC 
 
  356
 
  8
 
 30252
 
 tss
 
 0.39353
 
 0.89993
 
 Not queried
 
  2.7900717
 
 2.81e-02
 
 1.00e+00
  
 
  
 6
 
  28501816
 
  28501916
 
  PDF   STATS   ENSEMBL   UCSC 
 
  101
 
  5
 
 33572
 
 gene
 
 0.93978
 
 0.62184
 
 Not queried
 
  2.7870514
 
 3.10e-01
 
 1.00e+00
  
 
  
 11
 
 119294283
 
 119294441
 
  PDF   STATS   ENSEMBL   UCSC 
 
  159
 
  5
 
 7735
 
 tss;island
 
 0.83719
 
 0.46623
 
 Not queried
 
  2.7862803
 
 7.94e-03
 
 1.00e+00
  
 
  
 7
 
  45197251
 
  45197727
 
  PDF   STATS   ENSEMBL   UCSC 
 
  477
 
  7
 
 38410
 
 island
 
 0.42210
 
 0.85110
 
 Not queried
 
  2.7860455
 
 1.75e-02
 
 1.00e+00
  
 
  
 20
 
  44098223
 
  44098409
 
  PDF   STATS   ENSEMBL   UCSC 
 
  187
 
  7
 
 26160
 
 island
 
 0.14882
 
 0.59448
 
 Not queried
 
  2.7852564
 
 5.83e-04
 
 1.00e+00
  
 
  
 14
 
  35099471
 
  35099711
 
  PDF   STATS   ENSEMBL   UCSC 
 
  241
 
  5
 
 12319
 
 tss
 
 0.05040
 
 0.11606
 
 Not queried
 
  2.7852467
 
 3.10e-01
 
 1.00e+00
  
 
  
 19
 
   8809057
 
   8809360
 
  PDF   STATS   ENSEMBL   UCSC 
 
  304
 
  4
 
 21455
 
 island
 
 0.49170
 
 0.86939
 
 Not queried
 
  2.7841663
 
 4.86e-01
 
 1.00e+00
  
 
  
 12
 
  56694186
 
  56694667
 
  PDF   STATS   ENSEMBL   UCSC 
 
  482
 
  9
 
 9769
 
 tss
 
 0.02977
 
 0.07638
 
 Not queried
 
  2.7827030
 
 4.00e-02
 
 1.00e+00
  
 
  
 10
 
 102986525
 
 102986973
 
  PDF   STATS   ENSEMBL   UCSC 
 
  449
 
  6
 
 6041
 
 island
 
 0.77078
 
 0.33298
 
 Not queried
 
  2.7823308
 
 8.66e-03
 
 1.00e+00
  
 
  
 15
 
  96952113
 
  96952425
 
  PDF   STATS   ENSEMBL   UCSC 
 
  313
 
  4
 
 14462
 
 island
 
 0.95618
 
 0.75520
 
 Not queried
 
  2.7819261
 
 5.71e-02
 
 1.00e+00
  
 
  
 3
 
 186080023
 
 186080359
 
  PDF   STATS   ENSEMBL   UCSC 
 
  337
 
  7
 
 28729
 
 tss
 
 0.06782
 
 0.34290
 
 Not queried
 
  2.7809939
 
 5.83e-04
 
 1.00e+00
  
 
  
 5
 
 153862183
 
 153862450
 
  PDF   STATS   ENSEMBL   UCSC 
 
  268
 
  4
 
 33269
 
 island
 
 0.83153
 
 0.49772
 
 Not queried
 
  2.7807409
 
 2.86e-02
 
 1.00e+00
  
 
  
 X
 
 117107675
 
 117107923
 
  PDF   STATS   ENSEMBL   UCSC 
 
  249
 
  4
 
 41086
 
 gene
 
 0.30833
 
 0.02316
 
 Not queried
 
  2.7788237
 
 5.71e-02
 
 1.00e+00
  
 
  
 3
 
  10547179
 
  10547228
 
  PDF   STATS   ENSEMBL   UCSC 
 
   50
 
  4
 
 27621
 
 gene
 
 0.19992
 
 0.60576
 
 Not queried
 
  2.7768107
 
 2.86e-02
 
 1.00e+00
  
 
  
 2
 
   4050695
 
   4050845
 
  PDF   STATS   ENSEMBL   UCSC 
 
  151
 
  5
 
 24002
 
 island
 
 0.21593
 
 0.66962
 
 Not queried
 
  2.7764568
 
 7.94e-03
 
 1.00e+00
  
 
  
 5
 
 154091976
 
 154092426
 
  PDF   STATS   ENSEMBL   UCSC 
 
  451
 
  6
 
 32391
 
 tss
 
 0.34112
 
 0.71017
 
 Not queried
 
  2.7764552
 
 2.16e-03
 
 1.00e+00
  
 
  
 7
 
 120968877
 
 120969079
 
  PDF   STATS   ENSEMBL   UCSC 
 
  203
 
  7
 
 37796
 
 tss
 
 0.11651
 
 0.45255
 
 Not queried
 
  2.7763044
 
 5.83e-04
 
 1.00e+00
  
 
  
 5
 
  61601529
 
  61601647
 
  PDF   STATS   ENSEMBL   UCSC 
 
  119
 
  4
 
 32025
 
 tss;island
 
 0.29954
 
 0.05784
 
 Not queried
 
  2.7761772
 
 2.00e-01
 
 1.00e+00
  
 
  
 1
 
  37500195
 
  37500508
 
  PDF   STATS   ENSEMBL   UCSC 
 
  314
 
  5
 
 1496
 
 tss;island
 
 0.12428
 
 0.49202
 
 Not queried
 
  2.7744394
 
 1.59e-02
 
 1.00e+00
  
 
  
 1
 
  24127669
 
  24128066
 
  PDF   STATS   ENSEMBL   UCSC 
 
  398
 
  4
 
 1345
 
 tss;island
 
 0.02092
 
 0.14533
 
 Not queried
 
  2.7743178
 
 5.71e-02
 
 1.00e+00
  
 
  
 5
 
  19988592
 
  19988944
 
  PDF   STATS   ENSEMBL   UCSC 
 
  353
 
  9
 
 31941
 
 tss
 
 0.02962
 
 0.08484
 
 Not queried
 
  2.7737733
 
 4.00e-02
 
 1.00e+00
  
 
  
 19
 
  47551893
 
  47551991
 
  PDF   STATS   ENSEMBL   UCSC 
 
   99
 
  5
 
 20865
 
 tss
 
 0.16099
 
 0.04349
 
 Not queried
 
  2.7737063
 
 9.52e-02
 
 1.00e+00
  
 
  
 16
 
  79623618
 
  79623900
 
  PDF   STATS   ENSEMBL   UCSC 
 
  283
 
  5
 
 16079
 
 island
 
 0.04641
 
 0.24991
 
 Not queried
 
  2.7697613
 
 2.22e-01
 
 1.00e+00
  
 
  
 7
 
   1039875
 
   1040260
 
  PDF   STATS   ENSEMBL   UCSC 
 
  386
 
  4
 
 36669
 
 gene;island
 
 0.39738
 
 0.79243
 
 Not queried
 
  2.7676954
 
 2.86e-02
 
 1.00e+00
  
 
  
 22
 
  29426511
 
  29426832
 
  PDF   STATS   ENSEMBL   UCSC 
 
  322
 
  4
 
 26788
 
 gene;island
 
 0.22854
 
 0.05618
 
 Not queried
 
  2.7662785
 
 2.86e-02
 
 1.00e+00
  
 
  
 6
 
 156718177
 
 156718546
 
  PDF   STATS   ENSEMBL   UCSC 
 
  370
 
  5
 
 36527
 
 island
 
 0.50384
 
 0.87355
 
 Not queried
 
  2.7661981
 
 7.94e-03
 
 1.00e+00
  
 
  
 11
 
  68611260
 
  68611596
 
  PDF   STATS   ENSEMBL   UCSC 
 
  337
 
  4
 
 8557
 
 island
 
 0.19774
 
 0.02272
 
 Not queried
 
  2.7648299
 
 5.71e-02
 
 1.00e+00
  
 
  
 6
 
 134209935
 
 134210257
 
  PDF   STATS   ENSEMBL   UCSC 
 
  323
 
  6
 
 35168
 
 tss
 
 0.33399
 
 0.05527
 
 Not queried
 
  2.7623119
 
 8.66e-03
 
 1.00e+00
  
 
  
 13
 
  33113010
 
  33113343
 
  PDF   STATS   ENSEMBL   UCSC 
 
  334
 
  8
 
 11356
 
 tss
 
 0.00950
 
 0.07178
 
 Not queried
 
  2.7611632
 
 1.04e-02
 
 1.00e+00
  
 
  
 13
 
  28562798
 
  28562902
 
  PDF   STATS   ENSEMBL   UCSC 
 
  105
 
  7
 
 11339
 
 tss
 
 0.37111
 
 0.78960
 
 Not queried
 
  2.7610809
 
 7.28e-02
 
 1.00e+00
  
 
  
 12
 
  54133422
 
  54133730
 
  PDF   STATS   ENSEMBL   UCSC 
 
  309
 
  5
 
 10447
 
 island
 
 0.02185
 
 0.13150
 
 Not queried
 
  2.7610708
 
 7.94e-03
 
 1.00e+00
  
 
  
 1
 
  37499309
 
  37499649
 
  PDF   STATS   ENSEMBL   UCSC 
 
  341
 
  4
 
 430
 
 gene;island
 
 0.14576
 
 0.48953
 
 Not queried
 
  2.7603234
 
 2.86e-02
 
 1.00e+00
  
 
  
 10
 
 124220359
 
 124220874
 
  PDF   STATS   ENSEMBL   UCSC 
 
  516
 
  9
 
 5392
 
 tss
 
 0.17313
 
 0.07461
 
 Not queried
 
  2.7583348
 
 3.87e-01
 
 1.00e+00
  
 
  
 17
 
  78161467
 
  78161572
 
  PDF   STATS   ENSEMBL   UCSC 
 
  106
 
  5
 
 17689
 
 tss
 
 0.46288
 
 0.85356
 
 Not queried
 
  2.7577554
 
 7.94e-03
 
 1.00e+00
  
 
  
 Y
 
  15016579
 
  15016622
 
  PDF   STATS   ENSEMBL   UCSC 
 
   44
 
  4
 
 42168
 
 tss
 
 0.13275
 
 0.51796
 
 Not queried
 
  2.7568391
 
 2.86e-02
 
 1.00e+00
  
 
  
 1
 
  24645885
 
  24646392
 
  PDF   STATS   ENSEMBL   UCSC 
 
  508
 
  9
 
 325
 
 gene
 
 0.03056
 
 0.11459
 
 Not queried
 
  2.7566647
 
 1.85e-03
 
 1.00e+00
  
 
  
 1
 
   1141617
 
   1141991
 
  PDF   STATS   ENSEMBL   UCSC 
 
  375
 
  5
 
 20
 
 gene
 
 0.47915
 
 0.86907
 
 Not queried
 
  2.7546849
 
 1.59e-02
 
 1.00e+00
  
 
  
 17
 
  37331279
 
  37331768
 
  PDF   STATS   ENSEMBL   UCSC 
 
  490
 
  6
 
 16511
 
 gene;island
 
 0.12834
 
 0.63162
 
 Not queried
 
  2.7546342
 
 6.49e-02
 
 1.00e+00
  
 
  
 17
 
  79283062
 
  79283390
 
  PDF   STATS   ENSEMBL   UCSC 
 
  329
 
  8
 
 17703
 
 tss
 
 0.03492
 
 0.18597
 
 Not queried
 
  2.7534115
 
 6.99e-03
 
 1.00e+00
  
 
  
 17
 
  79282893
 
  79283390
 
  PDF   STATS   ENSEMBL   UCSC 
 
  498
 
 10
 
 18880
 
 island
 
 0.04171
 
 0.19837
 
 Not queried
 
  2.7534115
 
 1.05e-03
 
 1.00e+00
  
 
  
 2
 
  11924984
 
  11925375
 
  PDF   STATS   ENSEMBL   UCSC 
 
  392
 
  4
 
 22538
 
 gene;island
 
 0.10690
 
 0.39086
 
 Not queried
 
  2.7521418
 
 3.43e-01
 
 1.00e+00
  
 
  
 11
 
  63258712
 
  63258803
 
  PDF   STATS   ENSEMBL   UCSC 
 
   92
 
  6
 
 7354
 
 tss
 
 0.76639
 
 0.95685
 
 Not queried
 
  2.7515146
 
 1.52e-02
 
 1.00e+00
  
 
  
 11
 
  86383241
 
  86383940
 
  PDF   STATS   ENSEMBL   UCSC 
 
  700
 
 18
 
 7589
 
 tss
 
 0.17821
 
 0.60214
 
 Not queried
 
  2.7513510
 
 1.17e-02
 
 1.00e+00
  
 
  
 3
 
 158390510
 
 158390821
 
  PDF   STATS   ENSEMBL   UCSC 
 
  312
 
  7
 
 28643
 
 tss
 
 0.08304
 
 0.54637
 
 Not queried
 
  2.7498616
 
 2.59e-01
 
 1.00e+00
  
 
  
 X
 
  69353387
 
  69353974
 
  PDF   STATS   ENSEMBL   UCSC 
 
  588
 
  5
 
 41014
 
 gene;island
 
 0.09157
 
 0.02232
 
 Not queried
 
  2.7496371
 
 1.51e-01
 
 1.00e+00
  
 
  
 8
 
  10928985
 
  10929210
 
  PDF   STATS   ENSEMBL   UCSC 
 
  226
 
  4
 
 39140
 
 gene;island
 
 0.28075
 
 0.75210
 
 Not queried
 
  2.7482817
 
 5.71e-02
 
 1.00e+00
  
 
  
 4
 
 174451954
 
 174452159
 
  PDF   STATS   ENSEMBL   UCSC 
 
  206
 
  4
 
 30133
 
 gene;tss;island
 
 0.86681
 
 0.50143
 
 Not queried
 
  2.7482523
 
 2.00e-01
 
 1.00e+00
  
 
  
 14
 
  73706367
 
  73706676
 
  PDF   STATS   ENSEMBL   UCSC 
 
  310
 
  4
 
 12101
 
 gene;island
 
 0.34325
 
 0.08107
 
 Not queried
 
  2.7469041
 
 2.86e-02
 
 1.00e+00
  
 
  
 4
 
  71337658
 
  71337873
 
  PDF   STATS   ENSEMBL   UCSC 
 
  216
 
  4
 
 29971
 
 gene
 
 0.26423
 
 0.57181
 
 Not queried
 
  2.7459263
 
 3.43e-01
 
 1.00e+00
  
 
  
 22
 
  23522307
 
  23522748
 
  PDF   STATS   ENSEMBL   UCSC 
 
  442
 
  9
 
 27307
 
 island
 
 0.43579
 
 0.09564
 
 Not queried
 
  2.7456527
 
 1.90e-01
 
 1.00e+00
  
 
  
 5
 
   1174426
 
   1174666
 
  PDF   STATS   ENSEMBL   UCSC 
 
  241
 
  4
 
 32551
 
 island
 
 0.23050
 
 0.81909
 
 Not queried
 
  2.7450237
 
 2.00e-01
 
 1.00e+00
  
 
  
 19
 
  47925344
 
  47925618
 
  PDF   STATS   ENSEMBL   UCSC 
 
  275
 
  5
 
 22159
 
 island
 
 0.31710
 
 0.06153
 
 Not queried
 
  2.7449539
 
 9.52e-02
 
 1.00e+00
  
 
  
 8
 
  71520850
 
  71520989
 
  PDF   STATS   ENSEMBL   UCSC 
 
  140
 
  4
 
 39696
 
 tss;island
 
 0.23491
 
 0.03900
 
 Not queried
 
  2.7437890
 
 5.71e-02
 
 1.00e+00
  
 
  
 16
 
   2908157
 
   2908245
 
  PDF   STATS   ENSEMBL   UCSC 
 
   89
 
  4
 
 15592
 
 island
 
 0.16249
 
 0.50355
 
 Not queried
 
  2.7434080
 
 5.71e-02
 
 1.00e+00
  
 
  
 22
 
  40766469
 
  40766549
 
  PDF   STATS   ENSEMBL   UCSC 
 
   81
 
  6
 
 27120
 
 tss
 
 0.05124
 
 0.16837
 
 Not queried
 
  2.7429937
 
 4.33e-03
 
 1.00e+00
  
 
  
 16
 
   1069368
 
   1069760
 
  PDF   STATS   ENSEMBL   UCSC 
 
  393
 
  4
 
 15476
 
 island
 
 0.58550
 
 0.92486
 
 Not queried
 
  2.7422693
 
 2.86e-02
 
 1.00e+00
  
 
  
 X
 
 135230594
 
 135230717
 
  PDF   STATS   ENSEMBL   UCSC 
 
  124
 
  4
 
 41546
 
 tss
 
 0.38526
 
 0.10505
 
 Not queried
 
  2.7413396
 
 1.14e-01
 
 1.00e+00
  
 
  
 19
 
  54466297
 
  54466724
 
  PDF   STATS   ENSEMBL   UCSC 
 
  428
 
  5
 
 20174
 
 gene
 
 0.57421
 
 0.23824
 
 Not queried
 
  2.7409282
 
 1.59e-02
 
 1.00e+00
  
 
  
 11
 
  72928926
 
  72929394
 
  PDF   STATS   ENSEMBL   UCSC 
 
  469
 
 12
 
 7524
 
 tss
 
 0.21683
 
 0.02920
 
 Not queried
 
  2.7400765
 
 1.45e-02
 
 1.00e+00
  
 
  
 10
 
  65280766
 
  65280961
 
  PDF   STATS   ENSEMBL   UCSC 
 
  196
 
  5
 
 5089
 
 tss;island
 
 0.18096
 
 0.07636
 
 Not queried
 
  2.7395049
 
 9.52e-02
 
 1.00e+00
  
 
  
 19
 
  51522454
 
  51522921
 
  PDF   STATS   ENSEMBL   UCSC 
 
  468
 
  7
 
 20151
 
 gene;island
 
 0.53419
 
 0.14666
 
 Not queried
 
  2.7383295
 
 6.99e-03
 
 1.00e+00
  
 
  
 19
 
  47551842
 
  47551991
 
  PDF   STATS   ENSEMBL   UCSC 
 
  150
 
  7
 
 22151
 
 island
 
 0.19465
 
 0.03928
 
 Not queried
 
  2.7370949
 
 1.75e-02
 
 1.00e+00
  
 
  
 1
 
  24307153
 
  24307535
 
  PDF   STATS   ENSEMBL   UCSC 
 
  383
 
  5
 
 1350
 
 tss;island
 
 0.96907
 
 0.72054
 
 Not queried
 
  2.7370675
 
 3.17e-02
 
 1.00e+00
  
 
  
 3
 
  40657083
 
  40657497
 
  PDF   STATS   ENSEMBL   UCSC 
 
  415
 
  6
 
 28965
 
 island
 
 0.37873
 
 0.09173
 
 Not queried
 
  2.7360535
 
 6.49e-02
 
 1.00e+00
  
 
  
 X
 
  70521196
 
  70521525
 
  PDF   STATS   ENSEMBL   UCSC 
 
  330
 
  6
 
 41376
 
 tss
 
 0.75848
 
 0.93634
 
 Not queried
 
  2.7353789
 
 2.16e-03
 
 1.00e+00
  
 
  
 1
 
  17026786
 
  17027073
 
  PDF   STATS   ENSEMBL   UCSC 
 
  288
 
  4
 
 260
 
 gene;island
 
 0.14914
 
 0.53741
 
 Not queried
 
  2.7342635
 
 5.71e-02
 
 1.00e+00
  
 
  
 6
 
  30653167
 
  30653799
 
  PDF   STATS   ENSEMBL   UCSC 
 
  633
 
 12
 
 33741
 
 gene;island
 
 0.24017
 
 0.64951
 
 Not queried
 
  2.7337231
 
 2.91e-03
 
 1.00e+00
  
 
  
 10
 
  46168220
 
  46168597
 
  PDF   STATS   ENSEMBL   UCSC 
 
  378
 
  8
 
 5697
 
 island
 
 0.03226
 
 0.19957
 
 Not queried
 
  2.7323474
 
 1.48e-02
 
 1.00e+00
  
 
  
 21
 
  46131601
 
  46131923
 
  PDF   STATS   ENSEMBL   UCSC 
 
  323
 
  5
 
 26546
 
 tss
 
 0.22130
 
 0.65368
 
 Not queried
 
  2.7315256
 
 3.17e-02
 
 1.00e+00
  
 
  
 7
 
 109600399
 
 109600733
 
  PDF   STATS   ENSEMBL   UCSC 
 
  335
 
  4
 
 37772
 
 tss
 
 0.49612
 
 0.86106
 
 Not queried
 
  2.7306197
 
 3.43e-01
 
 1.00e+00
  
 
  
 12
 
 106978920
 
 106979229
 
  PDF   STATS   ENSEMBL   UCSC 
 
  310
 
  4
 
 9255
 
 gene;island
 
 0.29773
 
 0.05770
 
 Not queried
 
  2.7301137
 
 2.86e-02
 
 1.00e+00
  
 
  
 8
 
  57359200
 
  57359414
 
  PDF   STATS   ENSEMBL   UCSC 
 
  215
 
  4
 
 39650
 
 tss;island
 
 0.13464
 
 0.53154
 
 Not queried
 
  2.7297247
 
 2.86e-02
 
 1.00e+00
  
 
  
 21
 
  45232531
 
  45232601
 
  PDF   STATS   ENSEMBL   UCSC 
 
   71
 
  4
 
 26538
 
 tss
 
 0.32161
 
 0.67467
 
 Not queried
 
  2.7259234
 
 2.86e-02
 
 1.00e+00
  
 
  
 8
 
  67782729
 
  67783229
 
  PDF   STATS   ENSEMBL   UCSC 
 
  501
 
  6
 
 39684
 
 tss
 
 0.21495
 
 0.71830
 
 Not queried
 
  2.7234484
 
 1.52e-02
 
 1.00e+00
  
 
  
 3
 
  46759438
 
  46759698
 
  PDF   STATS   ENSEMBL   UCSC 
 
  261
 
  7
 
 28237
 
 tss
 
 0.27916
 
 0.85980
 
 Not queried
 
  2.7234438
 
 5.30e-02
 
 1.00e+00
  
 
  
 8
 
 142401373
 
 142401867
 
  PDF   STATS   ENSEMBL   UCSC 
 
  495
 
  4
 
 40557
 
 island
 
 0.21635
 
 0.72677
 
 Not queried
 
  2.7233206
 
 1.14e-01
 
 1.00e+00
  
 
  
 2
 
 163695776
 
 163696187
 
  PDF   STATS   ENSEMBL   UCSC 
 
  412
 
  6
 
 23652
 
 tss
 
 0.10906
 
 0.45149
 
 Not queried
 
  2.7228877
 
 4.33e-03
 
 1.00e+00
  
 
  
 15
 
  32933661
 
  32933835
 
  PDF   STATS   ENSEMBL   UCSC 
 
  175
 
  4
 
 13524
 
 tss
 
 0.13274
 
 0.49956
 
 Not queried
 
  2.7212443
 
 5.71e-02
 
 1.00e+00
  
 
  
 16
 
    850240
 
    850880
 
  PDF   STATS   ENSEMBL   UCSC 
 
  641
 
  8
 
 15459
 
 island
 
 0.03933
 
 0.33108
 
 Not queried
 
  2.7209449
 
 1.04e-02
 
 1.00e+00
  
 
  
 2
 
 219848258
 
 219848580
 
  PDF   STATS   ENSEMBL   UCSC 
 
  323
 
  5
 
 22985
 
 gene;island
 
 0.88134
 
 0.49765
 
 Not queried
 
  2.7208366
 
 9.52e-02
 
 1.00e+00
  
 
  
 5
 
 140248564
 
 140249080
 
  PDF   STATS   ENSEMBL   UCSC 
 
  517
 
  5
 
 31744
 
 gene;island
 
 0.12155
 
 0.50878
 
 Not queried
 
  2.7191769
 
 9.52e-02
 
 1.00e+00
  
 
  
 20
 
  36151003
 
  36151338
 
  PDF   STATS   ENSEMBL   UCSC 
 
  336
 
  4
 
 25379
 
 gene;island
 
 0.55384
 
 0.82649
 
 Not queried
 
  2.7184005
 
 5.71e-02
 
 1.00e+00
  
 
  
 7
 
 158379132
 
 158379348
 
  PDF   STATS   ENSEMBL   UCSC 
 
  217
 
  4
 
 37315
 
 gene;island
 
 0.61170
 
 0.89876
 
 Not queried
 
  2.7182457
 
 5.71e-02
 
 1.00e+00
  
 
  
 9
 
 130679027
 
 130679246
 
  PDF   STATS   ENSEMBL   UCSC 
 
  220
 
  4
 
 40720
 
 gene;island
 
 0.10496
 
 0.04241
 
 Not queried
 
  2.7176657
 
 3.43e-01
 
 1.00e+00
  
 
  
 12
 
  12223661
 
  12223989
 
  PDF   STATS   ENSEMBL   UCSC 
 
  329
 
  4
 
 9542
 
 tss
 
 0.41508
 
 0.81850
 
 Not queried
 
  2.7176216
 
 1.14e-01
 
 1.00e+00
  
 
  
 X
 
  54556330
 
  54557002
 
  PDF   STATS   ENSEMBL   UCSC 
 
  673
 
 13
 
 41814
 
 island
 
 0.21434
 
 0.03126
 
 Not queried
 
  2.7166368
 
 1.25e-03
 
 1.00e+00
  
 
  
 17
 
  78161467
 
  78161706
 
  PDF   STATS   ENSEMBL   UCSC 
 
  240
 
  6
 
 16824
 
 gene;island
 
 0.47117
 
 0.85436
 
 Not queried
 
  2.7166280
 
 4.33e-03
 
 1.00e+00
  
 
  
 12
 
 132905611
 
 132906010
 
  PDF   STATS   ENSEMBL   UCSC 
 
  400
 
  4
 
 11014
 
 island
 
 0.35113
 
 0.77540
 
 Not queried
 
  2.7152701
 
 2.86e-02
 
 1.00e+00
  
 
  
 7
 
  28450687
 
  28451011
 
  PDF   STATS   ENSEMBL   UCSC 
 
  325
 
  5
 
 36881
 
 gene;tss;island
 
 0.01784
 
 0.04453
 
 Not queried
 
  2.7148601
 
 1.51e-01
 
 1.00e+00
  
 
  
 17
 
  45330862
 
  45331082
 
  PDF   STATS   ENSEMBL   UCSC 
 
  221
 
  5
 
 17407
 
 tss;island
 
 0.54404
 
 0.12396
 
 Not queried
 
  2.7146789
 
 2.22e-01
 
 1.00e+00
  
 
  
 16
 
  69958626
 
  69958964
 
  PDF   STATS   ENSEMBL   UCSC 
 
  339
 
  4
 
 14793
 
 gene
 
 0.59447
 
 0.82015
 
 Not queried
 
  2.7129026
 
 1.14e-01
 
 1.00e+00
  
 
  
 20
 
  13279761
 
  13280201
 
  PDF   STATS   ENSEMBL   UCSC 
 
  441
 
  6
 
 25321
 
 gene;island
 
 0.40188
 
 0.78782
 
 Not queried
 
  2.7112689
 
 2.16e-03
 
 1.00e+00
  
 
  
 12
 
    670520
 
    671027
 
  PDF   STATS   ENSEMBL   UCSC 
 
  508
 
  5
 
 8971
 
 gene
 
 0.24678
 
 0.69033
 
 Not queried
 
  2.7101703
 
 7.94e-03
 
 1.00e+00
  
 
  
 2
 
  19561327
 
  19561709
 
  PDF   STATS   ENSEMBL   UCSC 
 
  383
 
  4
 
 24065
 
 island
 
 0.74129
 
 0.31636
 
 Not queried
 
  2.7097662
 
 2.86e-02
 
 1.00e+00
  
 
  
 1
 
   6304199
 
   6304499
 
  PDF   STATS   ENSEMBL   UCSC 
 
  301
 
  7
 
 2594
 
 island
 
 0.53761
 
 0.09738
 
 Not queried
 
  2.7095243
 
 1.11e-02
 
 1.00e+00
  
 
  
 20
 
  56725695
 
  56725899
 
  PDF   STATS   ENSEMBL   UCSC 
 
  205
 
  8
 
 25808
 
 tss
 
 0.34504
 
 0.77798
 
 Not queried
 
  2.7091751
 
 3.11e-04
 
 1.00e+00
  
 
  
 19
 
   4173254
 
   4173482
 
  PDF   STATS   ENSEMBL   UCSC 
 
  229
 
  4
 
 21329
 
 island
 
 0.01898
 
 0.20709
 
 Not queried
 
  2.7066088
 
 1.14e-01
 
 1.00e+00
  
 
  
 1
 
   3182620
 
   3183165
 
  PDF   STATS   ENSEMBL   UCSC 
 
  546
 
  5
 
 113
 
 gene;island
 
 0.63039
 
 0.87668
 
 Not queried
 
  2.7062523
 
 5.56e-02
 
 1.00e+00
  
 
  
 17
 
  25676201
 
  25676574
 
  PDF   STATS   ENSEMBL   UCSC 
 
  374
 
  4
 
 18101
 
 island
 
 0.14335
 
 0.53619
 
 Not queried
 
  2.7057123
 
 2.00e-01
 
 1.00e+00
  
 
  
 10
 
 131694575
 
 131694856
 
  PDF   STATS   ENSEMBL   UCSC 
 
  282
 
  5
 
 4820
 
 gene;island
 
 0.83842
 
 0.41342
 
 Not queried
 
  2.7055622
 
 2.22e-01
 
 1.00e+00
  
 
  
 13
 
  49549440
 
  49549665
 
  PDF   STATS   ENSEMBL   UCSC 
 
  226
 
  4
 
 11409
 
 tss;island
 
 0.19727
 
 0.01958
 
 Not queried
 
  2.7047839
 
 1.14e-01
 
 1.00e+00
  
 
  
 X
 
  15756372
 
  15756407
 
  PDF   STATS   ENSEMBL   UCSC 
 
   36
 
  4
 
 41202
 
 tss;island
 
 0.02384
 
 0.18953
 
 Not queried
 
  2.7043801
 
 5.71e-02
 
 1.00e+00
  
 
  
 12
 
 106974832
 
 106975246
 
  PDF   STATS   ENSEMBL   UCSC 
 
  415
 
  5
 
 10724
 
 island
 
 0.24463
 
 0.06257
 
 Not queried
 
  2.7035164
 
 2.22e-01
 
 1.00e+00
  
 
  
 7
 
    766100
 
    766323
 
  PDF   STATS   ENSEMBL   UCSC 
 
  224
 
  6
 
 37324
 
 tss
 
 0.21670
 
 0.01767
 
 Not queried
 
  2.7028313
 
 2.40e-01
 
 1.00e+00
  
 
  
 18
 
  54305799
 
  54306437
 
  PDF   STATS   ENSEMBL   UCSC 
 
  639
 
 11
 
 19495
 
 island
 
 0.02468
 
 0.09311
 
 Not queried
 
  2.7026003
 
 4.10e-03
 
 1.00e+00
  
 
  
 8
 
  61591199
 
  61591461
 
  PDF   STATS   ENSEMBL   UCSC 
 
  263
 
  5
 
 40267
 
 island
 
 0.02106
 
 0.04783
 
 Not queried
 
  2.7011256
 
 9.52e-02
 
 1.00e+00
  
 
  
 6
 
 152702330
 
 152702660
 
  PDF   STATS   ENSEMBL   UCSC 
 
  331
 
  6
 
 34451
 
 gene
 
 0.29975
 
 0.69906
 
 Not queried
 
  2.7008681
 
 1.52e-02
 
 1.00e+00
  
 
  
 11
 
  60414615
 
  60415111
 
  PDF   STATS   ENSEMBL   UCSC 
 
  497
 
  5
 
 6603
 
 gene;island
 
 0.12043
 
 0.47262
 
 Not queried
 
  2.6997058
 
 7.94e-03
 
 1.00e+00
  
 
  
 2
 
  27072271
 
  27072564
 
  PDF   STATS   ENSEMBL   UCSC 
 
  294
 
  4
 
 22580
 
 gene;island
 
 0.23049
 
 0.03847
 
 Not queried
 
  2.6995586
 
 2.86e-02
 
 1.00e+00
  
 
  
 17
 
   1811181
 
   1811625
 
  PDF   STATS   ENSEMBL   UCSC 
 
  445
 
  5
 
 17800
 
 island
 
 0.13996
 
 0.30676
 
 Not queried
 
  2.6994496
 
 3.17e-02
 
 1.00e+00
  
 
  
 11
 
  73357019
 
  73357396
 
  PDF   STATS   ENSEMBL   UCSC 
 
  378
 
  8
 
 7529
 
 tss
 
 0.76753
 
 0.34540
 
 Not queried
 
  2.6982030
 
 3.11e-04
 
 1.00e+00
  
 
  
 12
 
   9800259
 
   9800818
 
  PDF   STATS   ENSEMBL   UCSC 
 
  560
 
 11
 
 10211
 
 island
 
 0.07606
 
 0.03185
 
 Not queried
 
  2.6980019
 
 4.10e-03
 
 1.00e+00
  
 
  
 10
 
  50816917
 
  50817306
 
  PDF   STATS   ENSEMBL   UCSC 
 
  390
 
  6
 
 5056
 
 tss;island
 
 0.43224
 
 0.11271
 
 Not queried
 
  2.6974897
 
 4.33e-03
 
 1.00e+00
  
 
  
 10
 
 112256517
 
 112256825
 
  PDF   STATS   ENSEMBL   UCSC 
 
  309
 
  4
 
 5339
 
 tss;island
 
 0.36776
 
 0.06404
 
 Not queried
 
  2.6971445
 
 2.00e-01
 
 1.00e+00
  
 
  
 17
 
  58499679
 
  58499854
 
  PDF   STATS   ENSEMBL   UCSC 
 
  176
 
  6
 
 17519
 
 tss
 
 0.92745
 
 0.70882
 
 Not queried
 
  2.6950112
 
 8.66e-03
 
 1.00e+00
  
 
  
 10
 
  35894012
 
  35894321
 
  PDF   STATS   ENSEMBL   UCSC 
 
  310
 
  7
 
 5011
 
 tss
 
 0.37891
 
 0.88919
 
 Not queried
 
  2.6941344
 
 2.33e-03
 
 1.00e+00
  
 
  
 14
 
 105104840
 
 105105198
 
  PDF   STATS   ENSEMBL   UCSC 
 
  359
 
  4
 
 13180
 
 island
 
 0.41337
 
 0.77179
 
 Not queried
 
  2.6934720
 
 1.14e-01
 
 1.00e+00
  
 
  
 16
 
  14403004
 
  14403138
 
  PDF   STATS   ENSEMBL   UCSC 
 
  135
 
  4
 
 15071
 
 tss
 
 0.73621
 
 0.93736
 
 Not queried
 
  2.6928139
 
 1.14e-01
 
 1.00e+00
  
 
  
 3
 
 113160430
 
 113160821
 
  PDF   STATS   ENSEMBL   UCSC 
 
  392
 
  7
 
 28463
 
 tss
 
 0.67288
 
 0.93617
 
 Not queried
 
  2.6923160
 
 1.75e-02
 
 1.00e+00
  
 
  
 16
 
  57023022
 
  57023398
 
  PDF   STATS   ENSEMBL   UCSC 
 
  377
 
  7
 
 15225
 
 tss
 
 0.21981
 
 0.06187
 
 Not queried
 
  2.6923097
 
 2.59e-01
 
 1.00e+00
  
 
  
 18
 
  44337664
 
  44338147
 
  PDF   STATS   ENSEMBL   UCSC 
 
  484
 
  6
 
 19226
 
 tss;island
 
 0.03268
 
 0.19073
 
 Not queried
 
  2.6917553
 
 2.60e-02
 
 1.00e+00
  
 
  
 2
 
  19553237
 
  19553546
 
  PDF   STATS   ENSEMBL   UCSC 
 
  310
 
  4
 
 22550
 
 gene;island
 
 0.87775
 
 0.59410
 
 Not queried
 
  2.6907105
 
 2.00e-01
 
 1.00e+00
  
 
  
 12
 
  93096622
 
  93096819
 
  PDF   STATS   ENSEMBL   UCSC 
 
  198
 
  4
 
 9888
 
 tss
 
 0.72330
 
 0.94575
 
 Not queried
 
  2.6901170
 
 2.86e-02
 
 1.00e+00
  
 
  
 X
 
  48937688
 
  48937975
 
  PDF   STATS   ENSEMBL   UCSC 
 
  288
 
  5
 
 40991
 
 gene;tss;island
 
 0.09105
 
 0.01938
 
 Not queried
 
  2.6896765
 
 1.59e-02
 
 1.00e+00
  
 
  
 8
 
  37797680
 
  37797828
 
  PDF   STATS   ENSEMBL   UCSC 
 
  149
 
  5
 
 39585
 
 tss
 
 0.31437
 
 0.74203
 
 Not queried
 
  2.6896731
 
 7.94e-03
 
 1.00e+00
  
 
  
 6
 
  29523592
 
  29524117
 
  PDF   STATS   ENSEMBL   UCSC 
 
  526
 
  8
 
 33597
 
 gene;island
 
 0.49533
 
 0.84315
 
 Not queried
 
  2.6883425
 
 3.11e-04
 
 1.00e+00
  
 
  
 19
 
  14196219
 
  14196582
 
  PDF   STATS   ENSEMBL   UCSC 
 
  364
 
  4
 
 21636
 
 island
 
 0.67903
 
 0.19558
 
 Not queried
 
  2.6859975
 
 2.86e-02
 
 1.00e+00
  
 
  
 2
 
 220377593
 
 220377885
 
  PDF   STATS   ENSEMBL   UCSC 
 
  293
 
  4
 
 23870
 
 tss;island
 
 0.19978
 
 0.62183
 
 Not queried
 
  2.6854301
 
 1.14e-01
 
 1.00e+00
  
 
  
 14
 
 105154557
 
 105155327
 
  PDF   STATS   ENSEMBL   UCSC 
 
  771
 
  7
 
 12632
 
 tss;island
 
 0.36023
 
 0.77092
 
 Not queried
 
  2.6828708
 
 2.33e-03
 
 1.00e+00
  
 
  
 19
 
   1155030
 
   1155225
 
  PDF   STATS   ENSEMBL   UCSC 
 
  196
 
  4
 
 19612
 
 gene;island
 
 0.52595
 
 0.86740
 
 Not queried
 
  2.6826582
 
 2.86e-02
 
 1.00e+00
  
 
  
 1
 
  38022359
 
  38022718
 
  PDF   STATS   ENSEMBL   UCSC 
 
  360
 
  8
 
 3061
 
 island
 
 0.41686
 
 0.05772
 
 Not queried
 
  2.6813939
 
 1.30e-01
 
 1.00e+00
  
 
  
 17
 
  58499679
 
  58499911
 
  PDF   STATS   ENSEMBL   UCSC 
 
  233
 
  7
 
 18588
 
 island
 
 0.90576
 
 0.70041
 
 Not queried
 
  2.6813726
 
 2.33e-03
 
 1.00e+00
  
 
  
 12
 
  72233240
 
  72233572
 
  PDF   STATS   ENSEMBL   UCSC 
 
  333
 
 11
 
 10600
 
 island
 
 0.18789
 
 0.06269
 
 Not queried
 
  2.6782463
 
 7.59e-02
 
 1.00e+00
  
 
  
 4
 
  54424476
 
  54424787
 
  PDF   STATS   ENSEMBL   UCSC 
 
  312
 
  4
 
 30349
 
 tss
 
 0.37474
 
 0.76013
 
 Not queried
 
  2.6757753
 
 1.14e-01
 
 1.00e+00
  
 
  
 8
 
  37824306
 
  37824529
 
  PDF   STATS   ENSEMBL   UCSC 
 
  224
 
  4
 
 39586
 
 tss;island
 
 0.52208
 
 0.24369
 
 Not queried
 
  2.6726685
 
 2.00e-01
 
 1.00e+00
  
 
  
 7
 
  48019388
 
  48019713
 
  PDF   STATS   ENSEMBL   UCSC 
 
  326
 
  4
 
 37525
 
 tss
 
 0.66842
 
 0.95016
 
 Not queried
 
  2.6708775
 
 1.14e-01
 
 1.00e+00
  
 
  
 6
 
  32920567
 
  32920880
 
  PDF   STATS   ENSEMBL   UCSC 
 
  314
 
  5
 
 34101
 
 gene
 
 0.76126
 
 0.35003
 
 Not queried
 
  2.6705795
 
 1.51e-01
 
 1.00e+00
  
 
  
 12
 
  68041257
 
  68041428
 
  PDF   STATS   ENSEMBL   UCSC 
 
  172
 
  4
 
 9827
 
 tss;island
 
 0.16836
 
 0.04640
 
 Not queried
 
  2.6699804
 
 1.14e-01
 
 1.00e+00
  
 
  
 X
 
  48379816
 
  48380420
 
  PDF   STATS   ENSEMBL   UCSC 
 
  605
 
  6
 
 41763
 
 island
 
 0.14896
 
 0.50173
 
 Not queried
 
  2.6691136
 
 2.16e-03
 
 1.00e+00
  
 
  
 17
 
  33775860
 
  33776116
 
  PDF   STATS   ENSEMBL   UCSC 
 
  257
 
  7
 
 17238
 
 tss
 
 0.48817
 
 0.82778
 
 Not queried
 
  2.6687845
 
 5.83e-04
 
 1.00e+00
  
 
  
 6
 
 100060917
 
 100061504
 
  PDF   STATS   ENSEMBL   UCSC 
 
  588
 
  6
 
 34364
 
 gene;island
 
 0.07403
 
 0.03170
 
 Not queried
 
  2.6684203
 
 1.32e-01
 
 1.00e+00
  
 
  
 10
 
 112258394
 
 112258680
 
  PDF   STATS   ENSEMBL   UCSC 
 
  287
 
  4
 
 4739
 
 gene;island
 
 0.20073
 
 0.03673
 
 Not queried
 
  2.6682459
 
 1.14e-01
 
 1.00e+00
  
 
  
 6
 
  99290284
 
  99290737
 
  PDF   STATS   ENSEMBL   UCSC 
 
  454
 
  5
 
 36283
 
 island
 
 0.68832
 
 0.21136
 
 Not queried
 
  2.6681207
 
 7.94e-03
 
 1.00e+00
  
 
  
 11
 
  65339691
 
  65339797
 
  PDF   STATS   ENSEMBL   UCSC 
 
  107
 
  5
 
 7413
 
 tss;island
 
 0.04436
 
 0.22475
 
 Not queried
 
  2.6678477
 
 7.94e-03
 
 1.00e+00
  
 
  
 8
 
 125741158
 
 125741346
 
  PDF   STATS   ENSEMBL   UCSC 
 
  189
 
  5
 
 39843
 
 tss;island
 
 0.46779
 
 0.14078
 
 Not queried
 
  2.6678282
 
 5.56e-02
 
 1.00e+00
  
 
  
 1
 
 153283628
 
 153283994
 
  PDF   STATS   ENSEMBL   UCSC 
 
  367
 
  4
 
 1936
 
 tss
 
 0.76095
 
 0.34174
 
 Not queried
 
  2.6636724
 
 2.00e-01
 
 1.00e+00
  
 
  
 12
 
  52545884
 
  52546408
 
  PDF   STATS   ENSEMBL   UCSC 
 
  525
 
  5
 
 10410
 
 island
 
 0.21143
 
 0.65193
 
 Not queried
 
  2.6634709
 
 7.94e-03
 
 1.00e+00
  
 
  
 11
 
 121986961
 
 121987203
 
  PDF   STATS   ENSEMBL   UCSC 
 
  243
 
  7
 
 6928
 
 gene;tss
 
 0.61543
 
 0.81639
 
 Not queried
 
  2.6620551
 
 1.75e-02
 
 1.00e+00
  
 
  
 6
 
  29594830
 
  29595661
 
  PDF   STATS   ENSEMBL   UCSC 
 
  832
 
 15
 
 33615
 
 gene;island
 
 0.33964
 
 0.03911
 
 Not queried
 
  2.6613554
 
 1.96e-03
 
 1.00e+00
  
 
  
 16
 
  66412840
 
  66413098
 
  PDF   STATS   ENSEMBL   UCSC 
 
  259
 
  5
 
 14748
 
 gene
 
 0.17734
 
 0.55438
 
 Not queried
 
  2.6611829
 
 3.17e-02
 
 1.00e+00
  
 
  
 2
 
  71693090
 
  71693793
 
  PDF   STATS   ENSEMBL   UCSC 
 
  704
 
  9
 
 23393
 
 tss
 
 0.30839
 
 0.15488
 
 Not queried
 
  2.6609580
 
 1.06e-02
 
 1.00e+00
  
 
  
 2
 
  27805536
 
  27805844
 
  PDF   STATS   ENSEMBL   UCSC 
 
  309
 
  7
 
 23235
 
 tss
 
 0.87222
 
 0.51144
 
 Not queried
 
  2.6582479
 
 5.83e-04
 
 1.00e+00
  
 
  
 11
 
  71955299
 
  71955599
 
  PDF   STATS   ENSEMBL   UCSC 
 
  301
 
  8
 
 7519
 
 tss
 
 0.21748
 
 0.02672
 
 Not queried
 
  2.6569816
 
 6.22e-04
 
 1.00e+00
  
 
  
 17
 
  76967629
 
  76967825
 
  PDF   STATS   ENSEMBL   UCSC 
 
  197
 
  4
 
 16794
 
 gene
 
 0.08835
 
 0.50581
 
 Not queried
 
  2.6568408
 
 3.43e-01
 
 1.00e+00
  
 
  
 5
 
  38846475
 
  38846743
 
  PDF   STATS   ENSEMBL   UCSC 
 
  269
 
  4
 
 31541
 
 gene;island
 
 0.10737
 
 0.34798
 
 Not queried
 
  2.6564140
 
 2.00e-01
 
 1.00e+00
  
 
  
 7
 
 100964669
 
 100964998
 
  PDF   STATS   ENSEMBL   UCSC 
 
  330
 
  5
 
 37092
 
 gene;island
 
 0.06470
 
 0.12563
 
 Not queried
 
  2.6561892
 
 9.52e-02
 
 1.00e+00
  
 
  
 11
 
    320732
 
    320956
 
  PDF   STATS   ENSEMBL   UCSC 
 
  225
 
  6
 
 7820
 
 island
 
 0.17467
 
 0.52489
 
 Not queried
 
  2.6543940
 
 1.52e-02
 
 1.00e+00
  
 
  
 1
 
   1245014
 
   1245184
 
  PDF   STATS   ENSEMBL   UCSC 
 
  171
 
  4
 
 29
 
 gene;island
 
 0.25502
 
 0.63549
 
 Not queried
 
  2.6535882
 
 1.14e-01
 
 1.00e+00
  
 
  
 22
 
  17565455
 
  17565750
 
  PDF   STATS   ENSEMBL   UCSC 
 
  296
 
  6
 
 26899
 
 tss
 
 0.36062
 
 0.03917
 
 Not queried
 
  2.6522203
 
 1.80e-01
 
 1.00e+00
  
 
  
 5
 
 140571783
 
 140572039
 
  PDF   STATS   ENSEMBL   UCSC 
 
  257
 
  5
 
 33160
 
 island
 
 0.03241
 
 0.27296
 
 Not queried
 
  2.6508133
 
 6.90e-01
 
 1.00e+00
  
 
  
 19
 
  50931222
 
  50931622
 
  PDF   STATS   ENSEMBL   UCSC 
 
  401
 
  6
 
 20139
 
 gene;island
 
 0.39391
 
 0.77983
 
 Not queried
 
  2.6493029
 
 2.16e-03
 
 1.00e+00
  
 
  
 13
 
 100497453
 
 100497789
 
  PDF   STATS   ENSEMBL   UCSC 
 
  337
 
  4
 
 11178
 
 gene
 
 0.77182
 
 0.96306
 
 Not queried
 
  2.6492801
 
 2.86e-02
 
 1.00e+00
  
 
  
 19
 
  40732076
 
  40732316
 
  PDF   STATS   ENSEMBL   UCSC 
 
  241
 
  4
 
 20001
 
 gene;island
 
 0.34549
 
 0.69825
 
 Not queried
 
  2.6487478
 
 2.86e-02
 
 1.00e+00
  
 
  
 6
 
  25882328
 
  25882590
 
  PDF   STATS   ENSEMBL   UCSC 
 
  263
 
  5
 
 35439
 
 island
 
 0.10470
 
 0.32149
 
 Not queried
 
  2.6442896
 
 9.52e-02
 
 1.00e+00
  
 
  
 X
 
  99667001
 
  99667223
 
  PDF   STATS   ENSEMBL   UCSC 
 
  223
 
  4
 
 41900
 
 island
 
 0.07996
 
 0.40065
 
 Not queried
 
  2.6442328
 
 2.86e-02
 
 1.00e+00
  
 
  
 19
 
   1248287
 
   1248507
 
  PDF   STATS   ENSEMBL   UCSC 
 
  221
 
  5
 
 20259
 
 tss
 
 0.42583
 
 0.10172
 
 Not queried
 
  2.6441555
 
 2.22e-01
 
 1.00e+00
  
 
  
 18
 
  78004045
 
  78004474
 
  PDF   STATS   ENSEMBL   UCSC 
 
  430
 
  4
 
 19114
 
 gene;island
 
 0.61724
 
 0.20783
 
 Not queried
 
  2.6423340
 
 3.43e-01
 
 1.00e+00
  
 
  
 10
 
  21799047
 
  21799395
 
  PDF   STATS   ENSEMBL   UCSC 
 
  349
 
  4
 
 5576
 
 island
 
 0.20790
 
 0.06415
 
 Not queried
 
  2.6423147
 
 3.43e-01
 
 1.00e+00
  
 
  
 2
 
 177028606
 
 177028804
 
  PDF   STATS   ENSEMBL   UCSC 
 
  199
 
  5
 
 24873
 
 island
 
 0.83470
 
 0.47027
 
 Not queried
 
  2.6421637
 
 7.94e-03
 
 1.00e+00
  
 
  
 19
 
  34112111
 
  34112376
 
  PDF   STATS   ENSEMBL   UCSC 
 
  266
 
  9
 
 20651
 
 tss;island
 
 0.36838
 
 0.05798
 
 Not queried
 
  2.6412937
 
 1.06e-02
 
 1.00e+00
  
 
  
 19
 
  50316212
 
  50316458
 
  PDF   STATS   ENSEMBL   UCSC 
 
  247
 
  4
 
 20128
 
 gene
 
 0.14075
 
 0.04345
 
 Not queried
 
  2.6409884
 
 2.86e-02
 
 1.00e+00
  
 
  
 6
 
 151646540
 
 151646651
 
  PDF   STATS   ENSEMBL   UCSC 
 
  112
 
  5
 
 35235
 
 tss
 
 0.27881
 
 0.05149
 
 Not queried
 
  2.6391630
 
 7.94e-03
 
 1.00e+00
  
 
  
 14
 
  37666810
 
  37666994
 
  PDF   STATS   ENSEMBL   UCSC 
 
  185
 
  5
 
 12333
 
 tss;island
 
 0.01003
 
 0.09055
 
 Not queried
 
  2.6367353
 
 1.51e-01
 
 1.00e+00
  
 
  
 11
 
   2019079
 
   2019167
 
  PDF   STATS   ENSEMBL   UCSC 
 
   89
 
  5
 
 7034
 
 tss;island
 
 0.06693
 
 0.34400
 
 Not queried
 
  2.6363804
 
 1.59e-02
 
 1.00e+00
  
 
  
 17
 
  78039301
 
  78039528
 
  PDF   STATS   ENSEMBL   UCSC 
 
  228
 
  4
 
 16819
 
 gene;island
 
 0.54473
 
 0.82751
 
 Not queried
 
  2.6349307
 
 2.86e-02
 
 1.00e+00
  
 
  
 19
 
  15838756
 
  15839006
 
  PDF   STATS   ENSEMBL   UCSC 
 
  251
 
  4
 
 21674
 
 island
 
 0.16805
 
 0.54654
 
 Not queried
 
  2.6346569
 
 2.86e-02
 
 1.00e+00
  
 
  
 1
 
   6684860
 
   6685162
 
  PDF   STATS   ENSEMBL   UCSC 
 
  303
 
  7
 
 1211
 
 tss;island
 
 0.03974
 
 0.02685
 
 Not queried
 
  2.6342970
 
 9.73e-02
 
 1.00e+00
  
 
  
 4
 
  54965829
 
  54966187
 
  PDF   STATS   ENSEMBL   UCSC 
 
  359
 
  5
 
 30352
 
 tss
 
 0.08165
 
 0.02898
 
 Not queried
 
  2.6341485
 
 2.22e-01
 
 1.00e+00
  
 
  
 6
 
  43337670
 
  43337791
 
  PDF   STATS   ENSEMBL   UCSC 
 
  122
 
  5
 
 34933
 
 tss;island
 
 0.03865
 
 0.02199
 
 Not queried
 
  2.6333192
 
 6.90e-01
 
 1.00e+00
  
 
  
 12
 
  56075367
 
  56075511
 
  PDF   STATS   ENSEMBL   UCSC 
 
  145
 
  4
 
 9141
 
 gene
 
 0.02870
 
 0.16487
 
 Not queried
 
  2.6331960
 
 5.71e-02
 
 1.00e+00
  
 
  
 6
 
 170605952
 
 170606506
 
  PDF   STATS   ENSEMBL   UCSC 
 
  555
 
  6
 
 36630
 
 island
 
 0.29396
 
 0.07044
 
 Not queried
 
  2.6306017
 
 4.33e-03
 
 1.00e+00
  
 
  
 X
 
 152875453
 
 152875939
 
  PDF   STATS   ENSEMBL   UCSC 
 
  487
 
  4
 
 42114
 
 island
 
 0.01893
 
 0.23784
 
 Not queried
 
  2.6297281
 
 4.86e-01
 
 1.00e+00
  
 
  
 6
 
  33085470
 
  33085800
 
  PDF   STATS   ENSEMBL   UCSC 
 
  331
 
  8
 
 34124
 
 gene;island
 
 0.22189
 
 0.54470
 
 Not queried
 
  2.6295272
 
 6.22e-04
 
 1.00e+00
  
 
  
 3
 
 141516232
 
 141516705
 
  PDF   STATS   ENSEMBL   UCSC 
 
  474
 
  6
 
 27934
 
 gene;island
 
 0.11430
 
 0.02284
 
 Not queried
 
  2.6285809
 
 2.60e-02
 
 1.00e+00
  
 
  
 6
 
  26273370
 
  26273611
 
  PDF   STATS   ENSEMBL   UCSC 
 
  242
 
  5
 
 33546
 
 gene
 
 0.56601
 
 0.88965
 
 Not queried
 
  2.6279678
 
 3.17e-02
 
 1.00e+00
  
 
  
 11
 
 122850490
 
 122850741
 
  PDF   STATS   ENSEMBL   UCSC 
 
  252
 
  4
 
 6929
 
 gene;island
 
 0.08080
 
 0.43238
 
 Not queried
 
  2.6278605
 
 1.14e-01
 
 1.00e+00
  
 
  
 12
 
 133100333
 
 133100714
 
  PDF   STATS   ENSEMBL   UCSC 
 
  382
 
  7
 
 9424
 
 gene;island
 
 0.37293
 
 0.78667
 
 Not queried
 
  2.6275369
 
 6.99e-03
 
 1.00e+00
  
 
  
 11
 
  46366643
 
  46366884
 
  PDF   STATS   ENSEMBL   UCSC 
 
  242
 
  6
 
 7255
 
 tss
 
 0.17852
 
 0.58793
 
 Not queried
 
  2.6272587
 
 8.66e-03
 
 1.00e+00
  
 
  
 11
 
   1891699
 
   1892392
 
  PDF   STATS   ENSEMBL   UCSC 
 
  694
 
 13
 
 6417
 
 gene;island
 
 0.38573
 
 0.73251
 
 Not queried
 
  2.6266716
 
 1.25e-03
 
 1.00e+00
  
 
  
 5
 
 172359526
 
 172359680
 
  PDF   STATS   ENSEMBL   UCSC 
 
  155
 
  4
 
 31838
 
 gene
 
 0.05714
 
 0.39737
 
 Not queried
 
  2.6265977
 
 1.14e-01
 
 1.00e+00
  
 
  
 11
 
  31825605
 
  31825969
 
  PDF   STATS   ENSEMBL   UCSC 
 
  365
 
  5
 
 6542
 
 gene;island
 
 0.39536
 
 0.09246
 
 Not queried
 
  2.6265715
 
 1.59e-02
 
 1.00e+00
  
 
  
 X
 
  99666164
 
  99666486
 
  PDF   STATS   ENSEMBL   UCSC 
 
  323
 
  4
 
 41415
 
 tss;island
 
 0.15456
 
 0.55665
 
 Not queried
 
  2.6253621
 
 2.86e-02
 
 1.00e+00
  
 
  
 16
 
   3243304
 
   3243552
 
  PDF   STATS   ENSEMBL   UCSC 
 
  249
 
  4
 
 15610
 
 island
 
 0.05447
 
 0.25251
 
 Not queried
 
  2.6249058
 
 2.86e-02
 
 1.00e+00
  
 
  
 2
 
  97215789
 
  97216131
 
  PDF   STATS   ENSEMBL   UCSC 
 
  343
 
  4
 
 22745
 
 gene;island
 
 0.16671
 
 0.53502
 
 Not queried
 
  2.6236339
 
 3.43e-01
 
 1.00e+00
  
 
  
 1
 
 248020331
 
 248020436
 
  PDF   STATS   ENSEMBL   UCSC 
 
  106
 
  4
 
 2367
 
 tss
 
 0.02169
 
 0.05460
 
 Not queried
 
  2.6235790
 
 3.43e-01
 
 1.00e+00
  
 
  
 7
 
 158362729
 
 158362966
 
  PDF   STATS   ENSEMBL   UCSC 
 
  238
 
  4
 
 37314
 
 gene;island
 
 0.61639
 
 0.93220
 
 Not queried
 
  2.6232609
 
 5.71e-02
 
 1.00e+00
  
 
  
 17
 
  40346279
 
  40346486
 
  PDF   STATS   ENSEMBL   UCSC 
 
  208
 
  5
 
 16543
 
 gene
 
 0.07344
 
 0.33293
 
 Not queried
 
  2.6231273
 
 5.56e-02
 
 1.00e+00
  
 
  
 2
 
 165698673
 
 165698949
 
  PDF   STATS   ENSEMBL   UCSC 
 
  277
 
  4
 
 23655
 
 tss;island
 
 0.03940
 
 0.16136
 
 Not queried
 
  2.6222570
 
 2.00e-01
 
 1.00e+00
  
 
  
 19
 
  58095424
 
  58095595
 
  PDF   STATS   ENSEMBL   UCSC 
 
  172
 
  7
 
 21083
 
 tss
 
 0.01865
 
 0.08423
 
 Not queried
 
  2.6222103
 
 1.75e-02
 
 1.00e+00
  
 
  
 6
 
 170101455
 
 170101549
 
  PDF   STATS   ENSEMBL   UCSC 
 
   95
 
  4
 
 34509
 
 gene;tss;island
 
 0.10081
 
 0.02841
 
 Not queried
 
  2.6221157
 
 1.14e-01
 
 1.00e+00
  
 
  
 2
 
  71134524
 
  71134936
 
  PDF   STATS   ENSEMBL   UCSC 
 
  413
 
  6
 
 22689
 
 gene;island
 
 0.69157
 
 0.32988
 
 Not queried
 
  2.6220182
 
 2.16e-03
 
 1.00e+00
  
 
  
 5
 
 126409198
 
 126409573
 
  PDF   STATS   ENSEMBL   UCSC 
 
  376
 
  9
 
 32196
 
 tss
 
 0.46267
 
 0.90039
 
 Not queried
 
  2.6216305
 
 5.64e-03
 
 1.00e+00
  
 
  
 5
 
 179499304
 
 179499488
 
  PDF   STATS   ENSEMBL   UCSC 
 
  185
 
  4
 
 32498
 
 tss
 
 0.15702
 
 0.05307
 
 Not queried
 
  2.6203133
 
 6.86e-01
 
 1.00e+00
  
 
  
 17
 
  46667587
 
  46667812
 
  PDF   STATS   ENSEMBL   UCSC 
 
  226
 
  6
 
 17429
 
 tss;island
 
 0.82053
 
 0.42657
 
 Not queried
 
  2.6203078
 
 2.60e-02
 
 1.00e+00
  
 
  
 11
 
  72928926
 
  72929553
 
  PDF   STATS   ENSEMBL   UCSC 
 
  628
 
 13
 
 8616
 
 island
 
 0.12276
 
 0.02930
 
 Not queried
 
  2.6188501
 
 1.91e-02
 
 1.00e+00
  
 
  
 3
 
 157821919
 
 157822072
 
  PDF   STATS   ENSEMBL   UCSC 
 
  154
 
  4
 
 27968
 
 gene;island
 
 0.56315
 
 0.16996
 
 Not queried
 
  2.6183402
 
 2.86e-02
 
 1.00e+00
  
 
  
 19
 
  35981371
 
  35981677
 
  PDF   STATS   ENSEMBL   UCSC 
 
  307
 
  5
 
 20668
 
 tss
 
 0.11393
 
 0.44788
 
 Not queried
 
  2.6177862
 
 7.94e-03
 
 1.00e+00
  
 
  
 7
 
  16505589
 
  16505664
 
  PDF   STATS   ENSEMBL   UCSC 
 
   76
 
  5
 
 37387
 
 tss
 
 0.02978
 
 0.07647
 
 Not queried
 
  2.6166938
 
 3.17e-02
 
 1.00e+00
  
 
  
 1
 
   1475644
 
   1475742
 
  PDF   STATS   ENSEMBL   UCSC 
 
   99
 
  4
 
 2440
 
 island
 
 0.22353
 
 0.62716
 
 Not queried
 
  2.6164602
 
 2.86e-02
 
 1.00e+00
  
 
  
 X
 
  10851838
 
  10852037
 
  PDF   STATS   ENSEMBL   UCSC 
 
  200
 
  4
 
 41181
 
 tss
 
 0.03401
 
 0.13852
 
 Not queried
 
  2.6163037
 
 4.86e-01
 
 1.00e+00
  
 
  
 2
 
 176988203
 
 176988580
 
  PDF   STATS   ENSEMBL   UCSC 
 
  378
 
  5
 
 22900
 
 gene;island
 
 0.02211
 
 0.10006
 
 Not queried
 
  2.6144167
 
 7.94e-03
 
 1.00e+00
  
 
  
 1
 
 221060753
 
 221061254
 
  PDF   STATS   ENSEMBL   UCSC 
 
  502
 
  6
 
 4211
 
 island
 
 0.14249
 
 0.44076
 
 Not queried
 
  2.6128519
 
 2.16e-03
 
 1.00e+00
  
 
  
 7
 
 135413023
 
 135413232
 
  PDF   STATS   ENSEMBL   UCSC 
 
  210
 
  5
 
 37855
 
 tss
 
 0.30948
 
 0.74317
 
 Not queried
 
  2.6127756
 
 7.94e-03
 
 1.00e+00
  
 
  
 5
 
   1386363
 
   1386887
 
  PDF   STATS   ENSEMBL   UCSC 
 
  525
 
  5
 
 32567
 
 island
 
 0.12131
 
 0.36980
 
 Not queried
 
  2.6125823
 
 9.52e-02
 
 1.00e+00
  
 
  
 20
 
  61638420
 
  61638588
 
  PDF   STATS   ENSEMBL   UCSC 
 
  169
 
  7
 
 25848
 
 tss;island
 
 0.12671
 
 0.53513
 
 Not queried
 
  2.6121370
 
 5.83e-04
 
 1.00e+00
  
 
  
 1
 
 209825672
 
 209825747
 
  PDF   STATS   ENSEMBL   UCSC 
 
   76
 
  4
 
 982
 
 gene
 
 0.52348
 
 0.88214
 
 Not queried
 
  2.6114385
 
 5.71e-02
 
 1.00e+00
  
 
  
 10
 
  50339928
 
  50340173
 
  PDF   STATS   ENSEMBL   UCSC 
 
  246
 
  4
 
 4569
 
 gene;island
 
 0.39237
 
 0.81822
 
 Not queried
 
  2.6098249
 
 2.86e-02
 
 1.00e+00
  
 
  
 6
 
  32725075
 
  32725438
 
  PDF   STATS   ENSEMBL   UCSC 
 
  364
 
  5
 
 34065
 
 gene
 
 0.78894
 
 0.44975
 
 Not queried
 
  2.6069445
 
 7.94e-03
 
 1.00e+00
  
 
  
 12
 
  56329615
 
  56329903
 
  PDF   STATS   ENSEMBL   UCSC 
 
  289
 
  4
 
 9145
 
 gene;island
 
 0.81141
 
 0.41811
 
 Not queried
 
  2.6063892
 
 2.86e-02
 
 1.00e+00
  
 
  
 11
 
  75379491
 
  75379624
 
  PDF   STATS   ENSEMBL   UCSC 
 
  134
 
  6
 
 7549
 
 tss
 
 0.04141
 
 0.21559
 
 Not queried
 
  2.6038700
 
 4.33e-03
 
 1.00e+00
  
 
  
 1
 
 116184219
 
 116184423
 
  PDF   STATS   ENSEMBL   UCSC 
 
  205
 
  4
 
 1855
 
 tss;island
 
 0.65358
 
 0.22357
 
 Not queried
 
  2.6031020
 
 2.00e-01
 
 1.00e+00
  
 
  
 6
 
 167763758
 
 167764146
 
  PDF   STATS   ENSEMBL   UCSC 
 
  389
 
  4
 
 36581
 
 island
 
 0.20671
 
 0.53472
 
 Not queried
 
  2.5989230
 
 2.86e-02
 
 1.00e+00
  
 
  
 13
 
 112984478
 
 112984976
 
  PDF   STATS   ENSEMBL   UCSC 
 
  499
 
  5
 
 11889
 
 island
 
 0.50140
 
 0.92124
 
 Not queried
 
  2.5984117
 
 9.52e-02
 
 1.00e+00
  
 
  
 7
 
  27199726
 
  27199956
 
  PDF   STATS   ENSEMBL   UCSC 
 
  231
 
  5
 
 38291
 
 island
 
 0.59461
 
 0.96071
 
 Not queried
 
  2.5977705
 
 7.94e-03
 
 1.00e+00
  
 
  
 X
 
  70151117
 
  70151209
 
  PDF   STATS   ENSEMBL   UCSC 
 
   93
 
  4
 
 41370
 
 tss
 
 0.09005
 
 0.01658
 
 Not queried
 
  2.5964812
 
 2.86e-02
 
 1.00e+00
  
 
  
 7
 
 128809100
 
 128809442
 
  PDF   STATS   ENSEMBL   UCSC 
 
  343
 
  5
 
 38843
 
 island
 
 0.02275
 
 0.25150
 
 Not queried
 
  2.5964660
 
 7.94e-03
 
 1.00e+00
  
 
  
 11
 
  74022742
 
  74023050
 
  PDF   STATS   ENSEMBL   UCSC 
 
  309
 
  7
 
 7535
 
 tss
 
 0.52251
 
 0.15324
 
 Not queried
 
  2.5960966
 
 7.28e-02
 
 1.00e+00
  
 
  
 17
 
  72321767
 
  72322154
 
  PDF   STATS   ENSEMBL   UCSC 
 
  388
 
  5
 
 17590
 
 tss;island
 
 0.71782
 
 0.23147
 
 Not queried
 
  2.5933385
 
 6.90e-01
 
 1.00e+00
  
 
  
 12
 
   7033470
 
   7033628
 
  PDF   STATS   ENSEMBL   UCSC 
 
  159
 
  5
 
 10193
 
 island
 
 0.01721
 
 0.05392
 
 Not queried
 
  2.5920887
 
 2.22e-01
 
 1.00e+00
  
 
  
 3
 
 150690183
 
 150690585
 
  PDF   STATS   ENSEMBL   UCSC 
 
  403
 
  7
 
 27954
 
 gene
 
 0.12562
 
 0.55476
 
 Not queried
 
  2.5916660
 
 2.62e-02
 
 1.00e+00
  
 
  
 X
 
  48334240
 
  48334722
 
  PDF   STATS   ENSEMBL   UCSC 
 
  483
 
 10
 
 41760
 
 island
 
 0.04654
 
 0.01515
 
 Not queried
 
  2.5906874
 
 7.25e-04
 
 1.00e+00
  
 
  
 3
 
  40518669
 
  40518798
 
  PDF   STATS   ENSEMBL   UCSC 
 
  130
 
  4
 
 27686
 
 gene
 
 0.01446
 
 0.08173
 
 Not queried
 
  2.5888646
 
 2.00e-01
 
 1.00e+00
  
 
  
 6
 
  32158153
 
  32158513
 
  PDF   STATS   ENSEMBL   UCSC 
 
  361
 
  9
 
 34806
 
 tss
 
 0.06514
 
 0.27846
 
 Not queried
 
  2.5875694
 
 3.99e-03
 
 1.00e+00
  
 
  
 17
 
  80279310
 
  80279675
 
  PDF   STATS   ENSEMBL   UCSC 
 
  366
 
  5
 
 16909
 
 gene;island
 
 0.08994
 
 0.44891
 
 Not queried
 
  2.5858578
 
 2.22e-01
 
 1.00e+00
  
 
  
 3
 
 114102565
 
 114102863
 
  PDF   STATS   ENSEMBL   UCSC 
 
  299
 
  5
 
 27858
 
 gene;tss
 
 0.10949
 
 0.78366
 
 Not queried
 
  2.5854635
 
 4.21e-01
 
 1.00e+00
  
 
  
 18
 
    904851
 
    905252
 
  PDF   STATS   ENSEMBL   UCSC 
 
  402
 
 12
 
 19123
 
 tss
 
 0.05403
 
 0.23408
 
 Not queried
 
  2.5838774
 
 4.96e-04
 
 1.00e+00
  
 
  
 12
 
  54376019
 
  54376241
 
  PDF   STATS   ENSEMBL   UCSC 
 
  223
 
  4
 
 10458
 
 island
 
 0.86941
 
 0.35590
 
 Not queried
 
  2.5815684
 
 5.71e-02
 
 1.00e+00
  
 
  
 X
 
  48814806
 
  48815125
 
  PDF   STATS   ENSEMBL   UCSC 
 
  320
 
  8
 
 40990
 
 gene;island
 
 0.39768
 
 0.04124
 
 Not queried
 
  2.5787709
 
 2.81e-02
 
 1.00e+00
  
 
  
 11
 
  68748461
 
  68748584
 
  PDF   STATS   ENSEMBL   UCSC 
 
  124
 
  4
 
 7493
 
 tss
 
 0.33436
 
 0.62509
 
 Not queried
 
  2.5766220
 
 5.71e-02
 
 1.00e+00
  
 
  
 15
 
  83317281
 
  83317632
 
  PDF   STATS   ENSEMBL   UCSC 
 
  352
 
  5
 
 13830
 
 tss;island
 
 0.84999
 
 0.43373
 
 Not queried
 
  2.5766086
 
 3.17e-02
 
 1.00e+00
  
 
  
 17
 
  33700745
 
  33700817
 
  PDF   STATS   ENSEMBL   UCSC 
 
   73
 
  6
 
 17234
 
 tss;island
 
 0.01524
 
 0.11487
 
 Not queried
 
  2.5759409
 
 2.60e-02
 
 1.00e+00
  
 
  
 X
 
  25040609
 
  25041258
 
  PDF   STATS   ENSEMBL   UCSC 
 
  650
 
  6
 
 41695
 
 island
 
 0.19412
 
 0.09328
 
 Not queried
 
  2.5752218
 
 2.40e-01
 
 1.00e+00
  
 
  
 14
 
  56045827
 
  56046098
 
  PDF   STATS   ENSEMBL   UCSC 
 
  272
 
  4
 
 12061
 
 gene;tss;island
 
 0.03938
 
 0.01064
 
 Not queried
 
  2.5744792
 
 1.14e-01
 
 1.00e+00
  
 
  
 5
 
  78281734
 
  78282099
 
  PDF   STATS   ENSEMBL   UCSC 
 
  366
 
  6
 
 31602
 
 gene;island
 
 0.44345
 
 0.11735
 
 Not queried
 
  2.5729835
 
 4.11e-02
 
 1.00e+00
  
 
  
 17
 
   4544925
 
   4545157
 
  PDF   STATS   ENSEMBL   UCSC 
 
  233
 
  5
 
 17840
 
 island
 
 0.59325
 
 0.15165
 
 Not queried
 
  2.5717882
 
 1.51e-01
 
 1.00e+00
  
 
  
 13
 
  29394754
 
  29394960
 
  PDF   STATS   ENSEMBL   UCSC 
 
  207
 
  4
 
 11615
 
 island
 
 0.02706
 
 0.15357
 
 Not queried
 
  2.5697975
 
 1.14e-01
 
 1.00e+00
  
 
  
 11
 
  14280742
 
  14281053
 
  PDF   STATS   ENSEMBL   UCSC 
 
  312
 
  4
 
 6494
 
 gene;island
 
 0.25851
 
 0.59398
 
 Not queried
 
  2.5649461
 
 2.86e-02
 
 1.00e+00
  
 
  
 10
 
  91597207
 
  91597593
 
  PDF   STATS   ENSEMBL   UCSC 
 
  387
 
  4
 
 5908
 
 island
 
 0.55982
 
 0.88920
 
 Not queried
 
  2.5637779
 
 2.86e-02
 
 1.00e+00
  
 
  
 3
 
  45635931
 
  45636386
 
  PDF   STATS   ENSEMBL   UCSC 
 
  456
 
  7
 
 29005
 
 island
 
 0.07429
 
 0.32168
 
 Not queried
 
  2.5630678
 
 1.11e-02
 
 1.00e+00
  
 
  
 22
 
  18632404
 
  18633123
 
  PDF   STATS   ENSEMBL   UCSC 
 
  720
 
 10
 
 27243
 
 island
 
 0.02580
 
 0.19296
 
 Not queried
 
  2.5623349
 
 5.24e-02
 
 1.00e+00
  
 
  
 8
 
 144462007
 
 144462235
 
  PDF   STATS   ENSEMBL   UCSC 
 
  229
 
  4
 
 39412
 
 gene;island
 
 0.28101
 
 0.77243
 
 Not queried
 
  2.5617799
 
 2.00e-01
 
 1.00e+00
  
 
  
 2
 
  25390385
 
  25390540
 
  PDF   STATS   ENSEMBL   UCSC 
 
  156
 
  4
 
 22565
 
 gene;island
 
 0.80663
 
 0.41197
 
 Not queried
 
  2.5609396
 
 5.71e-02
 
 1.00e+00
  
 
  
 19
 
  50553682
 
  50553907
 
  PDF   STATS   ENSEMBL   UCSC 
 
  226
 
  5
 
 20935
 
 tss
 
 0.02432
 
 0.07327
 
 Not queried
 
  2.5608248
 
 9.52e-02
 
 1.00e+00
  
 
  
 22
 
  44287876
 
  44288155
 
  PDF   STATS   ENSEMBL   UCSC 
 
  280
 
  6
 
 27164
 
 tss
 
 0.53104
 
 0.21136
 
 Not queried
 
  2.5599415
 
 4.33e-03
 
 1.00e+00
  
 
  
 22
 
  44287772
 
  44288155
 
  PDF   STATS   ENSEMBL   UCSC 
 
  384
 
  8
 
 27518
 
 island
 
 0.47535
 
 0.20073
 
 Not queried
 
  2.5599415
 
 1.86e-03
 
 1.00e+00
  
 
  
 19
 
   1992866
 
   1993265
 
  PDF   STATS   ENSEMBL   UCSC 
 
  400
 
  5
 
 19639
 
 gene;island
 
 0.37905
 
 0.67047
 
 Not queried
 
  2.5596128
 
 7.94e-03
 
 1.00e+00
  
 
  
 11
 
  63258509
 
  63258803
 
  PDF   STATS   ENSEMBL   UCSC 
 
  295
 
  8
 
 8347
 
 island
 
 0.71443
 
 0.95024
 
 Not queried
 
  2.5582204
 
 1.86e-03
 
 1.00e+00
  
 
  
 12
 
  54346784
 
  54347242
 
  PDF   STATS   ENSEMBL   UCSC 
 
  459
 
  5
 
 10454
 
 island
 
 0.02193
 
 0.11659
 
 Not queried
 
  2.5574216
 
 3.17e-02
 
 1.00e+00
  
 
  
 1
 
  44456953
 
  44457185
 
  PDF   STATS   ENSEMBL   UCSC 
 
  233
 
  5
 
 1566
 
 tss
 
 0.01582
 
 0.02605
 
 Not queried
 
  2.5571609
 
 3.10e-01
 
 1.00e+00
  
 
  
 3
 
 195384522
 
 195384752
 
  PDF   STATS   ENSEMBL   UCSC 
 
  231
 
  6
 
 28762
 
 tss;island
 
 0.26191
 
 0.04549
 
 Not queried
 
  2.5569375
 
 3.10e-01
 
 1.00e+00
  
 
  
 1
 
  23696021
 
  23696209
 
  PDF   STATS   ENSEMBL   UCSC 
 
  189
 
  4
 
 311
 
 gene;tss;island
 
 0.01068
 
 0.06923
 
 Not queried
 
  2.5568438
 
 2.86e-02
 
 1.00e+00
  
 
  
 7
 
  45927735
 
  45927947
 
  PDF   STATS   ENSEMBL   UCSC 
 
  213
 
  5
 
 37521
 
 tss
 
 0.22758
 
 0.64353
 
 Not queried
 
  2.5553597
 
 1.51e-01
 
 1.00e+00
  
 
  
 1
 
 100111713
 
 100111949
 
  PDF   STATS   ENSEMBL   UCSC 
 
  237
 
  4
 
 647
 
 gene
 
 0.26149
 
 0.03159
 
 Not queried
 
  2.5542377
 
 2.00e-01
 
 1.00e+00
  
 
  
 4
 
   4864110
 
   4864902
 
  PDF   STATS   ENSEMBL   UCSC 
 
  793
 
 12
 
 29874
 
 gene;island
 
 0.07361
 
 0.57576
 
 Not queried
 
  2.5541116
 
 3.32e-02
 
 1.00e+00
  
 
  
 5
 
  76326040
 
  76326465
 
  PDF   STATS   ENSEMBL   UCSC 
 
  426
 
  6
 
 32834
 
 island
 
 0.88918
 
 0.46221
 
 Not queried
 
  2.5539345
 
 2.16e-03
 
 1.00e+00
  
 
  
 16
 
  23724821
 
  23724943
 
  PDF   STATS   ENSEMBL   UCSC 
 
  123
 
  5
 
 15112
 
 tss
 
 0.75959
 
 0.44472
 
 Not queried
 
  2.5535712
 
 7.94e-03
 
 1.00e+00
  
 
  
 6
 
  32076906
 
  32077103
 
  PDF   STATS   ENSEMBL   UCSC 
 
  198
 
  7
 
 33988
 
 gene
 
 0.11972
 
 0.43744
 
 Not queried
 
  2.5527878
 
 5.83e-04
 
 1.00e+00
  
 
  
 Y
 
  16636984
 
  16637142
 
  PDF   STATS   ENSEMBL   UCSC 
 
  159
 
  4
 
 42161
 
 gene;island
 
 0.06858
 
 0.32944
 
 Not queried
 
  2.5526533
 
 2.00e-01
 
 1.00e+00
  
 
  
 3
 
  46506104
 
  46506374
 
  PDF   STATS   ENSEMBL   UCSC 
 
  271
 
  4
 
 27707
 
 gene
 
 0.64520
 
 0.86676
 
 Not queried
 
  2.5502427
 
 2.86e-02
 
 1.00e+00
  
 
  
 15
 
  72476680
 
  72477069
 
  PDF   STATS   ENSEMBL   UCSC 
 
  390
 
  5
 
 13378
 
 gene;island
 
 0.09520
 
 0.46348
 
 Not queried
 
  2.5501949
 
 3.17e-02
 
 1.00e+00
  
 
  
 6
 
  32119874
 
  32120324
 
  PDF   STATS   ENSEMBL   UCSC 
 
  451
 
  7
 
 34801
 
 tss
 
 0.24073
 
 0.55778
 
 Not queried
 
  2.5496209
 
 6.99e-03
 
 1.00e+00
  
 
  
 17
 
  79495268
 
  79495664
 
  PDF   STATS   ENSEMBL   UCSC 
 
  397
 
  7
 
 18894
 
 island
 
 0.33961
 
 0.74464
 
 Not queried
 
  2.5492354
 
 1.17e-03
 
 1.00e+00
  
 
  
 15
 
  35087506
 
  35087773
 
  PDF   STATS   ENSEMBL   UCSC 
 
  268
 
  5
 
 13252
 
 gene
 
 0.62360
 
 0.84829
 
 Not queried
 
  2.5491490
 
 7.94e-03
 
 1.00e+00
  
 
  
 2
 
  74780172
 
  74780617
 
  PDF   STATS   ENSEMBL   UCSC 
 
  446
 
  6
 
 22713
 
 gene;island
 
 0.63942
 
 0.27504
 
 Not queried
 
  2.5490423
 
 1.52e-02
 
 1.00e+00
  
 
  
 17
 
  50236344
 
  50236765
 
  PDF   STATS   ENSEMBL   UCSC 
 
  422
 
  4
 
 16662
 
 gene;tss;island
 
 0.19128
 
 0.40675
 
 Not queried
 
  2.5481629
 
 3.43e-01
 
 1.00e+00
  
 
  
 3
 
 134369339
 
 134369828
 
  PDF   STATS   ENSEMBL   UCSC 
 
  490
 
  4
 
 27915
 
 gene
 
 0.33691
 
 0.08086
 
 Not queried
 
  2.5472109
 
 4.86e-01
 
 1.00e+00
  
 
  
 19
 
  45975694
 
  45976195
 
  PDF   STATS   ENSEMBL   UCSC 
 
  502
 
  5
 
 20062
 
 gene;island
 
 0.21404
 
 0.58513
 
 Not queried
 
  2.5459284
 
 7.94e-03
 
 1.00e+00
  
 
  
 3
 
 148847598
 
 148847791
 
  PDF   STATS   ENSEMBL   UCSC 
 
  194
 
  4
 
 27949
 
 gene;island
 
 0.02256
 
 0.07990
 
 Not queried
 
  2.5455753
 
 5.71e-02
 
 1.00e+00
  
 
  
 1
 
  26605913
 
  26606110
 
  PDF   STATS   ENSEMBL   UCSC 
 
  198
 
  4
 
 1384
 
 tss;island
 
 0.29933
 
 0.67607
 
 Not queried
 
  2.5447220
 
 2.86e-02
 
 1.00e+00
  
 
  
 4
 
   1983102
 
   1983380
 
  PDF   STATS   ENSEMBL   UCSC 
 
  279
 
  4
 
 29835
 
 gene;island
 
 0.58711
 
 0.93047
 
 Not queried
 
  2.5436910
 
 5.71e-02
 
 1.00e+00
  
 
  
 6
 
  29795350
 
  29795595
 
  PDF   STATS   ENSEMBL   UCSC 
 
  246
 
  9
 
 33632
 
 gene;island
 
 0.35531
 
 0.11712
 
 Not queried
 
  2.5434595
 
 3.15e-02
 
 1.00e+00
  
 
  
 7
 
 155165295
 
 155165891
 
  PDF   STATS   ENSEMBL   UCSC 
 
  597
 
  5
 
 39000
 
 island
 
 0.03280
 
 0.25412
 
 Not queried
 
  2.5427911
 
 9.52e-02
 
 1.00e+00
  
 
  
 11
 
 108422936
 
 108423325
 
  PDF   STATS   ENSEMBL   UCSC 
 
  390
 
  6
 
 7652
 
 tss
 
 0.60180
 
 0.75309
 
 Not queried
 
  2.5426620
 
 4.11e-02
 
 1.00e+00
  
 
  
 1
 
  36771714
 
  36771844
 
  PDF   STATS   ENSEMBL   UCSC 
 
  131
 
  4
 
 1491
 
 tss;island
 
 0.05155
 
 0.29117
 
 Not queried
 
  2.5395361
 
 2.86e-02
 
 1.00e+00
  
 
  
 19
 
  15162515
 
  15162971
 
  PDF   STATS   ENSEMBL   UCSC 
 
  457
 
  4
 
 20517
 
 tss
 
 0.22445
 
 0.44748
 
 Not queried
 
  2.5378335
 
 1.14e-01
 
 1.00e+00
  
 
  
 6
 
 155537901
 
 155538155
 
  PDF   STATS   ENSEMBL   UCSC 
 
  255
 
  7
 
 34455
 
 gene;island
 
 0.15414
 
 0.47430
 
 Not queried
 
  2.5362277
 
 1.17e-03
 
 1.00e+00
  
 
  
 10
 
 135333805
 
 135334122
 
  PDF   STATS   ENSEMBL   UCSC 
 
  318
 
  5
 
 6348
 
 island
 
 0.72841
 
 0.45169
 
 Not queried
 
  2.5359064
 
 9.52e-02
 
 1.00e+00
  
 
  
 1
 
   1935289
 
   1935561
 
  PDF   STATS   ENSEMBL   UCSC 
 
  273
 
  4
 
 1162
 
 tss
 
 0.86119
 
 0.48209
 
 Not queried
 
  2.5347678
 
 2.86e-02
 
 1.00e+00
  
 
  
 1
 
   1935048
 
   1935561
 
  PDF   STATS   ENSEMBL   UCSC 
 
  514
 
  6
 
 2463
 
 island
 
 0.86119
 
 0.43310
 
 Not queried
 
  2.5347678
 
 2.16e-03
 
 1.00e+00
  
 
  
 20
 
   5485245
 
   5485511
 
  PDF   STATS   ENSEMBL   UCSC 
 
  267
 
  7
 
 25544
 
 tss
 
 0.19804
 
 0.07416
 
 Not queried
 
  2.5342695
 
 5.30e-02
 
 1.00e+00
  
 
  
 3
 
 127316944
 
 127317209
 
  PDF   STATS   ENSEMBL   UCSC 
 
  266
 
  6
 
 28521
 
 tss
 
 0.19389
 
 0.03698
 
 Not queried
 
  2.5338691
 
 4.33e-03
 
 1.00e+00
  
 
  
 12
 
  34500585
 
  34500813
 
  PDF   STATS   ENSEMBL   UCSC 
 
  229
 
  4
 
 10296
 
 island
 
 0.07727
 
 0.21200
 
 Not queried
 
  2.5324498
 
 4.86e-01
 
 1.00e+00
  
 
  
 5
 
 140480507
 
 140480967
 
  PDF   STATS   ENSEMBL   UCSC 
 
  461
 
  6
 
 31750
 
 gene;island
 
 0.37920
 
 0.70780
 
 Not queried
 
  2.5318825
 
 2.16e-03
 
 1.00e+00
  
 
  
 11
 
 126872615
 
 126872863
 
  PDF   STATS   ENSEMBL   UCSC 
 
  249
 
  4
 
 8912
 
 island
 
 0.59519
 
 0.14055
 
 Not queried
 
  2.5316608
 
 2.00e-01
 
 1.00e+00
  
 
  
 1
 
 246952048
 
 246952508
 
  PDF   STATS   ENSEMBL   UCSC 
 
  461
 
  6
 
 2360
 
 tss;island
 
 0.59612
 
 0.87916
 
 Not queried
 
  2.5306584
 
 6.49e-02
 
 1.00e+00
  
 
  
 4
 
 140656749
 
 140657110
 
  PDF   STATS   ENSEMBL   UCSC 
 
  362
 
  4
 
 30086
 
 gene;island
 
 0.09267
 
 0.47270
 
 Not queried
 
  2.5296348
 
 2.00e-01
 
 1.00e+00
  
 
  
 1
 
  68299388
 
  68299511
 
  PDF   STATS   ENSEMBL   UCSC 
 
  124
 
  5
 
 1682
 
 tss;island
 
 0.05163
 
 0.20746
 
 Not queried
 
  2.5294073
 
 1.59e-02
 
 1.00e+00
  
 
  
 3
 
 120004186
 
 120004483
 
  PDF   STATS   ENSEMBL   UCSC 
 
  298
 
  4
 
 29345
 
 island
 
 0.23726
 
 0.08853
 
 Not queried
 
  2.5293688
 
 2.00e-01
 
 1.00e+00
  
 
  
 1
 
 150947403
 
 150947589
 
  PDF   STATS   ENSEMBL   UCSC 
 
  187
 
  5
 
 3720
 
 island
 
 0.21176
 
 0.04575
 
 Not queried
 
  2.5291720
 
 3.10e-01
 
 1.00e+00
  
 
  
 2
 
 202097062
 
 202097173
 
  PDF   STATS   ENSEMBL   UCSC 
 
  112
 
  4
 
 23778
 
 tss
 
 0.01157
 
 0.06313
 
 Not queried
 
  2.5275253
 
 2.00e-01
 
 1.00e+00
  
 
  
 X
 
  46432770
 
  46433132
 
  PDF   STATS   ENSEMBL   UCSC 
 
  363
 
  5
 
 41272
 
 tss;island
 
 0.09311
 
 0.02971
 
 Not queried
 
  2.5273936
 
 3.10e-01
 
 1.00e+00
  
 
  
 17
 
  38183370
 
  38183790
 
  PDF   STATS   ENSEMBL   UCSC 
 
  421
 
  5
 
 16522
 
 gene;tss
 
 0.18029
 
 0.66116
 
 Not queried
 
  2.5265304
 
 9.52e-02
 
 1.00e+00
  
 
  
 17
 
  21279561
 
  21279696
 
  PDF   STATS   ENSEMBL   UCSC 
 
  136
 
  5
 
 17170
 
 tss
 
 0.24710
 
 0.06580
 
 Not queried
 
  2.5263870
 
 3.17e-02
 
 1.00e+00
  
 
  
 1
 
  26606846
 
  26606912
 
  PDF   STATS   ENSEMBL   UCSC 
 
   67
 
  4
 
 337
 
 gene;island
 
 0.04874
 
 0.25626
 
 Not queried
 
  2.5261579
 
 2.86e-02
 
 1.00e+00
  
 
  
 17
 
  16119344
 
  16119456
 
  PDF   STATS   ENSEMBL   UCSC 
 
  113
 
  4
 
 17121
 
 tss;island
 
 0.11223
 
 0.02982
 
 Not queried
 
  2.5257806
 
 2.86e-02
 
 1.00e+00
  
 
  
 X
 
 118356756
 
 118357181
 
  PDF   STATS   ENSEMBL   UCSC 
 
  426
 
  5
 
 41980
 
 island
 
 0.96018
 
 0.60308
 
 Not queried
 
  2.5251156
 
 3.10e-01
 
 1.00e+00
  
 
  
 9
 
  93564028
 
  93564603
 
  PDF   STATS   ENSEMBL   UCSC 
 
  576
 
  5
 
 40708
 
 gene;island
 
 0.02771
 
 0.04389
 
 Not queried
 
  2.5235035
 
 5.48e-01
 
 1.00e+00
  
 
  
 1
 
  26487591
 
  26488129
 
  PDF   STATS   ENSEMBL   UCSC 
 
  539
 
  5
 
 336
 
 gene;island
 
 0.33330
 
 0.08003
 
 Not queried
 
  2.5228427
 
 2.22e-01
 
 1.00e+00
  
 
  
 18
 
   2913135
 
   2913254
 
  PDF   STATS   ENSEMBL   UCSC 
 
  120
 
  4
 
 19003
 
 gene;island
 
 0.63912
 
 0.92350
 
 Not queried
 
  2.5228286
 
 2.86e-02
 
 1.00e+00
  
 
  
 X
 
  48397520
 
  48398048
 
  PDF   STATS   ENSEMBL   UCSC 
 
  529
 
 12
 
 41292
 
 tss
 
 0.19150
 
 0.57702
 
 Not queried
 
  2.5221289
 
 1.43e-03
 
 1.00e+00
  
 
  
 10
 
 101287609
 
 101287846
 
  PDF   STATS   ENSEMBL   UCSC 
 
  238
 
  5
 
 5991
 
 island
 
 0.42003
 
 0.08319
 
 Not queried
 
  2.5214765
 
 9.52e-02
 
 1.00e+00
  
 
  
 20
 
  30028062
 
  30028292
 
  PDF   STATS   ENSEMBL   UCSC 
 
  231
 
  6
 
 25618
 
 tss
 
 0.32576
 
 0.59444
 
 Not queried
 
  2.5209452
 
 2.60e-02
 
 1.00e+00
  
 
  
 22
 
  46067363
 
  46067586
 
  PDF   STATS   ENSEMBL   UCSC 
 
  224
 
  4
 
 27182
 
 tss;island
 
 0.52613
 
 0.14146
 
 Not queried
 
  2.5201432
 
 2.00e-01
 
 1.00e+00
  
 
  
 17
 
  72920176
 
  72920385
 
  PDF   STATS   ENSEMBL   UCSC 
 
  210
 
  9
 
 17603
 
 tss;island
 
 0.07928
 
 0.43956
 
 Not queried
 
  2.5197218
 
 2.44e-02
 
 1.00e+00
  
 
  
 14
 
  60977856
 
  60978275
 
  PDF   STATS   ENSEMBL   UCSC 
 
  420
 
  5
 
 12075
 
 gene;island
 
 0.15048
 
 0.02404
 
 Not queried
 
  2.5195909
 
 1.51e-01
 
 1.00e+00
  
 
  
 7
 
 150211761
 
 150211939
 
  PDF   STATS   ENSEMBL   UCSC 
 
  179
 
  5
 
 37917
 
 tss
 
 0.38586
 
 0.81228
 
 Not queried
 
  2.5176660
 
 3.17e-02
 
 1.00e+00
  
 
  
 12
 
 124864528
 
 124864681
 
  PDF   STATS   ENSEMBL   UCSC 
 
  154
 
  4
 
 9366
 
 gene
 
 0.61106
 
 0.90440
 
 Not queried
 
  2.5170714
 
 2.86e-02
 
 1.00e+00
  
 
  
 3
 
  48647215
 
  48647426
 
  PDF   STATS   ENSEMBL   UCSC 
 
  212
 
  5
 
 28260
 
 tss
 
 0.08956
 
 0.02244
 
 Not queried
 
  2.5166561
 
 2.22e-01
 
 1.00e+00
  
 
  
 5
 
 134872610
 
 134872659
 
  PDF   STATS   ENSEMBL   UCSC 
 
   50
 
  4
 
 32247
 
 tss;island
 
 0.91552
 
 0.55534
 
 Not queried
 
  2.5160095
 
 2.86e-02
 
 1.00e+00
  
 
  
 15
 
  77577491
 
  77577655
 
  PDF   STATS   ENSEMBL   UCSC 
 
  165
 
  4
 
 13797
 
 tss
 
 0.74800
 
 0.94791
 
 Not queried
 
  2.5157881
 
 2.00e-01
 
 1.00e+00
  
 
  
 6
 
  29527870
 
  29528119
 
  PDF   STATS   ENSEMBL   UCSC 
 
  250
 
  4
 
 34684
 
 tss
 
 0.57967
 
 0.88774
 
 Not queried
 
  2.5150574
 
 2.86e-02
 
 1.00e+00
  
 
  
 6
 
  43478455
 
  43478829
 
  PDF   STATS   ENSEMBL   UCSC 
 
  375
 
  8
 
 34935
 
 tss;island
 
 0.51589
 
 0.86438
 
 Not queried
 
  2.5134457
 
 2.07e-02
 
 1.00e+00
  
 
  
 1
 
 155247476
 
 155247785
 
  PDF   STATS   ENSEMBL   UCSC 
 
  310
 
  4
 
 797
 
 gene
 
 0.02598
 
 0.09077
 
 Not queried
 
  2.5133333
 
 5.71e-02
 
 1.00e+00
  
 
  
 8
 
  72757787
 
  72758095
 
  PDF   STATS   ENSEMBL   UCSC 
 
  309
 
  4
 
 39700
 
 tss;island
 
 0.06723
 
 0.22663
 
 Not queried
 
  2.5123540
 
 2.00e-01
 
 1.00e+00
  
 
  
 11
 
  46366643
 
  46367100
 
  PDF   STATS   ENSEMBL   UCSC 
 
  458
 
  8
 
 6580
 
 gene;island
 
 0.20168
 
 0.60332
 
 Not queried
 
  2.5119900
 
 6.22e-04
 
 1.00e+00
  
 
  
 6
 
  29794503
 
  29794843
 
  PDF   STATS   ENSEMBL   UCSC 
 
  341
 
  4
 
 35570
 
 island
 
 0.77761
 
 0.38422
 
 Not queried
 
  2.5112582
 
 1.14e-01
 
 1.00e+00
  
 
  
 4
 
   3471816
 
   3472246
 
  PDF   STATS   ENSEMBL   UCSC 
 
  431
 
  4
 
 29861
 
 gene;island
 
 0.68693
 
 0.92626
 
 Not queried
 
  2.5082870
 
 2.86e-02
 
 1.00e+00
  
 
  
 1
 
   6526309
 
   6526583
 
  PDF   STATS   ENSEMBL   UCSC 
 
  275
 
  7
 
 1203
 
 tss
 
 0.31933
 
 0.75345
 
 Not queried
 
  2.5079524
 
 1.28e-01
 
 1.00e+00
  
 
  
 1
 
  13881331
 
  13881656
 
  PDF   STATS   ENSEMBL   UCSC 
 
  326
 
  5
 
 2699
 
 island
 
 0.96367
 
 0.82873
 
 Not queried
 
  2.5068871
 
 9.52e-02
 
 1.00e+00
  
 
  
 X
 
  70288026
 
  70288735
 
  PDF   STATS   ENSEMBL   UCSC 
 
  710
 
 12
 
 41855
 
 island
 
 0.17071
 
 0.02416
 
 Not queried
 
  2.5053026
 
 1.11e-03
 
 1.00e+00
  
 
  
 20
 
  54580070
 
  54580196
 
  PDF   STATS   ENSEMBL   UCSC 
 
  127
 
  4
 
 25793
 
 tss;island
 
 0.09448
 
 0.36321
 
 Not queried
 
  2.5046784
 
 1.14e-01
 
 1.00e+00
  
 
  
 X
 
  75648802
 
  75648992
 
  PDF   STATS   ENSEMBL   UCSC 
 
  191
 
  4
 
 41038
 
 gene;island
 
 0.36253
 
 0.13080
 
 Not queried
 
  2.5033810
 
 2.86e-02
 
 1.00e+00
  
 
  
 17
 
   6616799
 
   6617192
 
  PDF   STATS   ENSEMBL   UCSC 
 
  394
 
  6
 
 17018
 
 tss
 
 0.25153
 
 0.05083
 
 Not queried
 
  2.5030114
 
 1.32e-01
 
 1.00e+00
  
 
  
 17
 
  46806445
 
  46806935
 
  PDF   STATS   ENSEMBL   UCSC 
 
  491
 
  6
 
 17439
 
 tss;island
 
 0.06590
 
 0.28194
 
 Not queried
 
  2.5024509
 
 4.11e-02
 
 1.00e+00
  
 
  
 11
 
  22454152
 
  22454661
 
  PDF   STATS   ENSEMBL   UCSC 
 
  510
 
  6
 
 8113
 
 island
 
 0.32240
 
 0.65678
 
 Not queried
 
  2.5022900
 
 2.16e-03
 
 1.00e+00
  
 
  
 8
 
 144099482
 
 144099719
 
  PDF   STATS   ENSEMBL   UCSC 
 
  238
 
  4
 
 39408
 
 gene
 
 0.05663
 
 0.42055
 
 Not queried
 
  2.5019103
 
 2.00e-01
 
 1.00e+00
  
 
  
 22
 
  48027641
 
  48027921
 
  PDF   STATS   ENSEMBL   UCSC 
 
  281
 
  4
 
 27557
 
 island
 
 0.63995
 
 0.90494
 
 Not queried
 
  2.5009183
 
 2.00e-01
 
 1.00e+00
  
 
  
 3
 
  44596178
 
  44596684
 
  PDF   STATS   ENSEMBL   UCSC 
 
  507
 
  8
 
 28214
 
 tss
 
 0.90228
 
 0.63094
 
 Not queried
 
  2.5007807
 
 1.05e-01
 
 1.00e+00
  
 
  
 6
 
  85473171
 
  85473344
 
  PDF   STATS   ENSEMBL   UCSC 
 
  174
 
  4
 
 34339
 
 gene;island
 
 0.02836
 
 0.14659
 
 Not queried
 
  2.4991433
 
 2.86e-02
 
 1.00e+00
  
 
  
 2
 
  23850319
 
  23850586
 
  PDF   STATS   ENSEMBL   UCSC 
 
  268
 
  4
 
 22558
 
 gene;island
 
 0.61965
 
 0.88600
 
 Not queried
 
  2.4990395
 
 1.14e-01
 
 1.00e+00
  
 
  
 15
 
  53097649
 
  53097840
 
  PDF   STATS   ENSEMBL   UCSC 
 
  192
 
  4
 
 14126
 
 island
 
 0.56687
 
 0.14359
 
 Not queried
 
  2.4983061
 
 2.00e-01
 
 1.00e+00
  
 
  
 6
 
  30954872
 
  30955142
 
  PDF   STATS   ENSEMBL   UCSC 
 
  271
 
  4
 
 33782
 
 gene
 
 0.78384
 
 0.42916
 
 Not queried
 
  2.4981764
 
 2.86e-02
 
 1.00e+00
  
 
  
 3
 
 170303540
 
 170304045
 
  PDF   STATS   ENSEMBL   UCSC 
 
  506
 
  7
 
 29614
 
 island
 
 0.46919
 
 0.15174
 
 Not queried
 
  2.4976727
 
 7.28e-02
 
 1.00e+00
  
 
  
 5
 
 179004535
 
 179004757
 
  PDF   STATS   ENSEMBL   UCSC 
 
  223
 
  4
 
 31875
 
 gene;island
 
 0.79713
 
 0.97856
 
 Not queried
 
  2.4962218
 
 2.00e-01
 
 1.00e+00
  
 
  
 3
 
  48936456
 
  48936584
 
  PDF   STATS   ENSEMBL   UCSC 
 
  129
 
  4
 
 28267
 
 tss
 
 0.03937
 
 0.19524
 
 Not queried
 
  2.4960789
 
 2.00e-01
 
 1.00e+00
  
 
  
 5
 
 114598574
 
 114599159
 
  PDF   STATS   ENSEMBL   UCSC 
 
  586
 
 10
 
 32165
 
 tss
 
 0.03510
 
 0.03632
 
 Not queried
 
  2.4960129
 
 2.47e-01
 
 1.00e+00
  
 
  
 16
 
  67219152
 
  67219218
 
  PDF   STATS   ENSEMBL   UCSC 
 
   67
 
  4
 
 14762
 
 gene;tss;island
 
 0.34531
 
 0.74502
 
 Not queried
 
  2.4937925
 
 1.14e-01
 
 1.00e+00
  
 
  
 3
 
 186334993
 
 186335202
 
  PDF   STATS   ENSEMBL   UCSC 
 
  210
 
  4
 
 28031
 
 gene
 
 0.03126
 
 0.08836
 
 Not queried
 
  2.4927073
 
 1.14e-01
 
 1.00e+00
  
 
  
 X
 
  48694012
 
  48694446
 
  PDF   STATS   ENSEMBL   UCSC 
 
  435
 
  5
 
 41296
 
 tss;island
 
 0.57779
 
 0.05604
 
 Not queried
 
  2.4922063
 
 2.22e-01
 
 1.00e+00
  
 
  
 11
 
 134176598
 
 134176888
 
  PDF   STATS   ENSEMBL   UCSC 
 
  291
 
  5
 
 6976
 
 gene
 
 0.53531
 
 0.73401
 
 Not queried
 
  2.4921712
 
 5.56e-02
 
 1.00e+00
  
 
  
 11
 
    705692
 
    706100
 
  PDF   STATS   ENSEMBL   UCSC 
 
  409
 
  7
 
 7004
 
 tss;island
 
 0.20775
 
 0.59601
 
 Not queried
 
  2.4921542
 
 5.30e-02
 
 1.00e+00
  
 
  
 3
 
 186080015
 
 186080359
 
  PDF   STATS   ENSEMBL   UCSC 
 
  345
 
  8
 
 29705
 
 island
 
 0.06807
 
 0.34839
 
 Not queried
 
  2.4919123
 
 3.11e-04
 
 1.00e+00
  
 
  
 12
 
 132910456
 
 132910801
 
  PDF   STATS   ENSEMBL   UCSC 
 
  346
 
  4
 
 11015
 
 island
 
 0.56082
 
 0.88948
 
 Not queried
 
  2.4904447
 
 5.71e-02
 
 1.00e+00
  
 
  
 14
 
  38091400
 
  38092175
 
  PDF   STATS   ENSEMBL   UCSC 
 
  776
 
  7
 
 12772
 
 island
 
 0.31726
 
 0.62333
 
 Not queried
 
  2.4898245
 
 2.33e-03
 
 1.00e+00
  
 
  
 12
 
 120241760
 
 120242513
 
  PDF   STATS   ENSEMBL   UCSC 
 
  754
 
  9
 
 9317
 
 gene;island
 
 0.39653
 
 0.55085
 
 Not queried
 
  2.4895529
 
 2.58e-01
 
 1.00e+00
  
 
  
 6
 
  27782909
 
  27783331
 
  PDF   STATS   ENSEMBL   UCSC 
 
  423
 
  5
 
 34657
 
 tss;island
 
 0.21893
 
 0.62794
 
 Not queried
 
  2.4859395
 
 5.56e-02
 
 1.00e+00
  
 
  
 7
 
  89748633
 
  89748686
 
  PDF   STATS   ENSEMBL   UCSC 
 
   54
 
  6
 
 37629
 
 tss
 
 0.12516
 
 0.35031
 
 Not queried
 
  2.4853850
 
 2.16e-03
 
 1.00e+00
  
 
  
 1
 
 247694271
 
 247694557
 
  PDF   STATS   ENSEMBL   UCSC 
 
  287
 
  5
 
 1124
 
 gene;tss;island
 
 0.67765
 
 0.95265
 
 Not queried
 
  2.4849705
 
 3.17e-02
 
 1.00e+00
  
 
  
 6
 
  32810551
 
  32810833
 
  PDF   STATS   ENSEMBL   UCSC 
 
  283
 
  5
 
 34085
 
 gene;island
 
 0.91451
 
 0.68226
 
 Not queried
 
  2.4848873
 
 5.56e-02
 
 1.00e+00
  
 
  
 5
 
 134073430
 
 134073579
 
  PDF   STATS   ENSEMBL   UCSC 
 
  150
 
  5
 
 32237
 
 tss;island
 
 0.04936
 
 0.01491
 
 Not queried
 
  2.4847936
 
 5.56e-02
 
 1.00e+00
  
 
  
 10
 
 102731747
 
 102732224
 
  PDF   STATS   ENSEMBL   UCSC 
 
  478
 
  7
 
 5282
 
 tss
 
 0.69153
 
 0.78615
 
 Not queried
 
  2.4838629
 
 1.28e-01
 
 1.00e+00
  
 
  
 6
 
  32115964
 
  32116317
 
  PDF   STATS   ENSEMBL   UCSC 
 
  354
 
 10
 
 35837
 
 island
 
 0.31274
 
 0.68069
 
 Not queried
 
  2.4837009
 
 8.93e-03
 
 1.00e+00
  
 
  
 17
 
  79026049
 
  79026354
 
  PDF   STATS   ENSEMBL   UCSC 
 
  306
 
  5
 
 16856
 
 gene;island
 
 0.62831
 
 0.80027
 
 Not queried
 
  2.4833931
 
 7.94e-03
 
 1.00e+00
  
 
  
 17
 
   3438857
 
   3439372
 
  PDF   STATS   ENSEMBL   UCSC 
 
  516
 
  7
 
 16299
 
 gene;island
 
 0.06999
 
 0.33922
 
 Not queried
 
  2.4831745
 
 7.10e-01
 
 1.00e+00
  
 
  
 18
 
  21852313
 
  21852436
 
  PDF   STATS   ENSEMBL   UCSC 
 
  124
 
  5
 
 19178
 
 tss
 
 0.01600
 
 0.08335
 
 Not queried
 
  2.4831361
 
 7.94e-03
 
 1.00e+00
  
 
  
 11
 
  58673453
 
  58673615
 
  PDF   STATS   ENSEMBL   UCSC 
 
  163
 
  4
 
 8276
 
 island
 
 0.03908
 
 0.19582
 
 Not queried
 
  2.4829733
 
 2.86e-02
 
 1.00e+00
  
 
  
 3
 
 147131069
 
 147131332
 
  PDF   STATS   ENSEMBL   UCSC 
 
  264
 
  4
 
 27948
 
 gene;island
 
 0.73082
 
 0.28735
 
 Not queried
 
  2.4827575
 
 2.86e-02
 
 1.00e+00
  
 
  
 15
 
  83952722
 
  83953068
 
  PDF   STATS   ENSEMBL   UCSC 
 
  347
 
  5
 
 13430
 
 gene;island
 
 0.02655
 
 0.11179
 
 Not queried
 
  2.4812793
 
 2.22e-01
 
 1.00e+00
  
 
  
 12
 
  52652220
 
  52652742
 
  PDF   STATS   ENSEMBL   UCSC 
 
  523
 
  5
 
 10413
 
 island
 
 0.58485
 
 0.92847
 
 Not queried
 
  2.4807632
 
 7.94e-03
 
 1.00e+00
  
 
  
 1
 
   2461820
 
   2461929
 
  PDF   STATS   ENSEMBL   UCSC 
 
  110
 
  6
 
 1172
 
 tss;island
 
 0.27252
 
 0.06492
 
 Not queried
 
  2.4772864
 
 4.33e-03
 
 1.00e+00
  
 
  
 10
 
  18549378
 
  18549536
 
  PDF   STATS   ENSEMBL   UCSC 
 
  159
 
  5
 
 4957
 
 tss
 
 0.30040
 
 0.73956
 
 Not queried
 
  2.4772086
 
 9.52e-02
 
 1.00e+00
  
 
  
 8
 
  70984199
 
  70984559
 
  PDF   STATS   ENSEMBL   UCSC 
 
  361
 
  4
 
 39694
 
 tss;island
 
 0.12126
 
 0.03570
 
 Not queried
 
  2.4754735
 
 2.86e-02
 
 1.00e+00
  
 
  
 2
 
 187558355
 
 187558598
 
  PDF   STATS   ENSEMBL   UCSC 
 
  244
 
  5
 
 23736
 
 tss
 
 0.28529
 
 0.04401
 
 Not queried
 
  2.4748908
 
 3.10e-01
 
 1.00e+00
  
 
  
 2
 
 135476601
 
 135477056
 
  PDF   STATS   ENSEMBL   UCSC 
 
  456
 
  8
 
 23604
 
 tss;island
 
 0.22850
 
 0.04060
 
 Not queried
 
  2.4737124
 
 1.61e-01
 
 1.00e+00
  
 
  
 17
 
  29886672
 
  29886890
 
  PDF   STATS   ENSEMBL   UCSC 
 
  219
 
  4
 
 17215
 
 tss
 
 0.35532
 
 0.71197
 
 Not queried
 
  2.4724104
 
 2.86e-02
 
 1.00e+00
  
 
  
 X
 
  13587506
 
  13587678
 
  PDF   STATS   ENSEMBL   UCSC 
 
  173
 
  4
 
 41190
 
 tss;island
 
 0.39407
 
 0.11925
 
 Not queried
 
  2.4723490
 
 2.00e-01
 
 1.00e+00
  
 
  
 19
 
  38183253
 
  38183456
 
  PDF   STATS   ENSEMBL   UCSC 
 
  204
 
  6
 
 20707
 
 tss
 
 0.25882
 
 0.05133
 
 Not queried
 
  2.4718194
 
 2.16e-03
 
 1.00e+00
  
 
  
 1
 
 228464049
 
 228464394
 
  PDF   STATS   ENSEMBL   UCSC 
 
  346
 
  4
 
 1049
 
 gene;island
 
 0.52786
 
 0.88204
 
 Not queried
 
  2.4707653
 
 1.14e-01
 
 1.00e+00
  
 
  
 11
 
 113953462
 
 113953838
 
  PDF   STATS   ENSEMBL   UCSC 
 
  377
 
  4
 
 6890
 
 gene;island
 
 0.73033
 
 0.30028
 
 Not queried
 
  2.4700102
 
 1.14e-01
 
 1.00e+00
  
 
  
 10
 
 102501755
 
 102502104
 
  PDF   STATS   ENSEMBL   UCSC 
 
  350
 
  4
 
 6014
 
 island
 
 0.30460
 
 0.05519
 
 Not queried
 
  2.4692729
 
 2.86e-02
 
 1.00e+00
  
 
  
 10
 
 119293083
 
 119293550
 
  PDF   STATS   ENSEMBL   UCSC 
 
  468
 
  5
 
 4766
 
 gene;island
 
 0.04352
 
 0.22912
 
 Not queried
 
  2.4690668
 
 9.52e-02
 
 1.00e+00
  
 
  
 1
 
   1141617
 
   1142405
 
  PDF   STATS   ENSEMBL   UCSC 
 
  789
 
  9
 
 2410
 
 island
 
 0.46816
 
 0.84146
 
 Not queried
 
  2.4680211
 
 4.94e-04
 
 1.00e+00
  
 
  
 16
 
  34295778
 
  34296306
 
  PDF   STATS   ENSEMBL   UCSC 
 
  529
 
  4
 
 15858
 
 island
 
 0.23195
 
 0.54499
 
 Not queried
 
  2.4676760
 
 1.14e-01
 
 1.00e+00
  
 
  
 6
 
  27215328
 
  27215397
 
  PDF   STATS   ENSEMBL   UCSC 
 
   70
 
  4
 
 34650
 
 tss
 
 0.50605
 
 0.15647
 
 Not queried
 
  2.4666953
 
 1.14e-01
 
 1.00e+00
  
 
  
 6
 
  27215328
 
  27215675
 
  PDF   STATS   ENSEMBL   UCSC 
 
  348
 
  6
 
 35487
 
 island
 
 0.54792
 
 0.15647
 
 Not queried
 
  2.4666953
 
 2.60e-02
 
 1.00e+00
  
 
  
 6
 
 155537901
 
 155538055
 
  PDF   STATS   ENSEMBL   UCSC 
 
  155
 
  6
 
 35247
 
 tss
 
 0.14551
 
 0.44032
 
 Not queried
 
  2.4650476
 
 4.33e-03
 
 1.00e+00
  
 
  
 12
 
  58003774
 
  58003965
 
  PDF   STATS   ENSEMBL   UCSC 
 
  192
 
  5
 
 9796
 
 tss;island
 
 0.19104
 
 0.69107
 
 Not queried
 
  2.4639497
 
 1.59e-02
 
 1.00e+00
  
 
  
 6
 
  30610729
 
  30610781
 
  PDF   STATS   ENSEMBL   UCSC 
 
   53
 
  4
 
 33727
 
 gene;island
 
 0.77510
 
 0.92604
 
 Not queried
 
  2.4630510
 
 2.86e-02
 
 1.00e+00
  
 
  
 6
 
  29796351
 
  29796770
 
  PDF   STATS   ENSEMBL   UCSC 
 
  420
 
 15
 
 33634
 
 gene;island
 
 0.16317
 
 0.48590
 
 Not queried
 
  2.4630216
 
 1.96e-03
 
 1.00e+00
  
 
  
 6
 
  27841633
 
  27841865
 
  PDF   STATS   ENSEMBL   UCSC 
 
  233
 
  4
 
 34665
 
 tss;island
 
 0.52060
 
 0.87866
 
 Not queried
 
  2.4625795
 
 2.86e-02
 
 1.00e+00
  
 
  
 11
 
  62647979
 
  62648277
 
  PDF   STATS   ENSEMBL   UCSC 
 
  299
 
  6
 
 7352
 
 tss
 
 0.02212
 
 0.17010
 
 Not queried
 
  2.4620099
 
 8.66e-03
 
 1.00e+00
  
 
  
 5
 
 140734543
 
 140735027
 
  PDF   STATS   ENSEMBL   UCSC 
 
  485
 
  6
 
 31763
 
 gene;island
 
 0.31816
 
 0.75040
 
 Not queried
 
  2.4615614
 
 4.11e-02
 
 1.00e+00
  
 
  
 5
 
  87985809
 
  87986067
 
  PDF   STATS   ENSEMBL   UCSC 
 
  259
 
  4
 
 32886
 
 island
 
 0.07196
 
 0.00825
 
 Not queried
 
  2.4564988
 
 2.00e-01
 
 1.00e+00
  
 
  
 11
 
    313120
 
    313624
 
  PDF   STATS   ENSEMBL   UCSC 
 
  505
 
  6
 
 6988
 
 tss;island
 
 0.48387
 
 0.86425
 
 Not queried
 
  2.4562208
 
 2.16e-03
 
 1.00e+00
  
 
  
 6
 
 166722053
 
 166722248
 
  PDF   STATS   ENSEMBL   UCSC 
 
  196
 
  6
 
 35271
 
 tss;island
 
 0.43317
 
 0.09456
 
 Not queried
 
  2.4560381
 
 4.33e-03
 
 1.00e+00
  
 
  
 12
 
 132293329
 
 132293702
 
  PDF   STATS   ENSEMBL   UCSC 
 
  374
 
  5
 
 10986
 
 island
 
 0.68468
 
 0.94191
 
 Not queried
 
  2.4559296
 
 3.17e-02
 
 1.00e+00
  
 
  
 X
 
  37026349
 
  37026473
 
  PDF   STATS   ENSEMBL   UCSC 
 
  125
 
  4
 
 41711
 
 island
 
 0.57138
 
 0.89724
 
 Not queried
 
  2.4557152
 
 2.86e-02
 
 1.00e+00
  
 
  
 19
 
  50249464
 
  50249927
 
  PDF   STATS   ENSEMBL   UCSC 
 
  464
 
  6
 
 20126
 
 gene;island
 
 0.11223
 
 0.55338
 
 Not queried
 
  2.4539774
 
 2.60e-02
 
 1.00e+00
  
 
  
 16
 
  15489614
 
  15489968
 
  PDF   STATS   ENSEMBL   UCSC 
 
  355
 
  4
 
 14644
 
 gene
 
 0.03327
 
 0.26478
 
 Not queried
 
  2.4529712
 
 3.43e-01
 
 1.00e+00
  
 
  
 12
 
   6756377
 
   6756815
 
  PDF   STATS   ENSEMBL   UCSC 
 
  439
 
  6
 
 10176
 
 island
 
 0.10667
 
 0.37829
 
 Not queried
 
  2.4522009
 
 4.11e-02
 
 1.00e+00
  
 
  
 6
 
 100911687
 
 100911746
 
  PDF   STATS   ENSEMBL   UCSC 
 
   60
 
  6
 
 35075
 
 tss
 
 0.25778
 
 0.06611
 
 Not queried
 
  2.4514116
 
 2.16e-03
 
 1.00e+00
  
 
  
 6
 
 100911526
 
 100911746
 
  PDF   STATS   ENSEMBL   UCSC 
 
  221
 
  8
 
 36303
 
 island
 
 0.34942
 
 0.09154
 
 Not queried
 
  2.4514116
 
 1.86e-03
 
 1.00e+00
  
 
  
 10
 
  73156377
 
  73156647
 
  PDF   STATS   ENSEMBL   UCSC 
 
  271
 
  4
 
 5126
 
 tss
 
 0.34493
 
 0.05429
 
 Not queried
 
  2.4508232
 
 3.43e-01
 
 1.00e+00
  
 
  
 2
 
 182545485
 
 182545934
 
  PDF   STATS   ENSEMBL   UCSC 
 
  450
 
  9
 
 23725
 
 tss;island
 
 0.07838
 
 0.35771
 
 Not queried
 
  2.4504676
 
 2.44e-02
 
 1.00e+00
  
 
  
 X
 
  48768927
 
  48769107
 
  PDF   STATS   ENSEMBL   UCSC 
 
  181
 
  7
 
 41299
 
 tss
 
 0.18229
 
 0.59281
 
 Not queried
 
  2.4500122
 
 5.83e-04
 
 1.00e+00
  
 
  
 1
 
 153939684
 
 153939851
 
  PDF   STATS   ENSEMBL   UCSC 
 
  168
 
  5
 
 769
 
 gene;tss;island
 
 0.10817
 
 0.02049
 
 Not queried
 
  2.4497252
 
 7.94e-03
 
 1.00e+00
  
 
  
 19
 
   2717293
 
   2717615
 
  PDF   STATS   ENSEMBL   UCSC 
 
  323
 
  4
 
 19665
 
 gene;island
 
 0.51722
 
 0.83531
 
 Not queried
 
  2.4497125
 
 1.14e-01
 
 1.00e+00
  
 
  
 3
 
 169482714
 
 169483295
 
  PDF   STATS   ENSEMBL   UCSC 
 
  582
 
  9
 
 29603
 
 island
 
 0.32044
 
 0.73358
 
 Not queried
 
  2.4491458
 
 2.22e-01
 
 1.00e+00
  
 
  
 8
 
 103613080
 
 103613429
 
  PDF   STATS   ENSEMBL   UCSC 
 
  350
 
  4
 
 40444
 
 island
 
 0.69671
 
 0.28697
 
 Not queried
 
  2.4477888
 
 2.86e-02
 
 1.00e+00
  
 
  
 1
 
 171810105
 
 171810570
 
  PDF   STATS   ENSEMBL   UCSC 
 
  466
 
  8
 
 2089
 
 tss;island
 
 0.12996
 
 0.02102
 
 Not queried
 
  2.4475824
 
 3.79e-02
 
 1.00e+00
  
 
  
 19
 
  17958339
 
  17958825
 
  PDF   STATS   ENSEMBL   UCSC 
 
  487
 
  6
 
 19889
 
 gene
 
 0.50857
 
 0.12623
 
 Not queried
 
  2.4466090
 
 6.49e-02
 
 1.00e+00
  
 
  
 3
 
  50388115
 
  50388192
 
  PDF   STATS   ENSEMBL   UCSC 
 
   78
 
  5
 
 28313
 
 tss
 
 0.18455
 
 0.04054
 
 Not queried
 
  2.4466025
 
 7.94e-03
 
 1.00e+00
  
 
  
 X
 
  48397520
 
  48398133
 
  PDF   STATS   ENSEMBL   UCSC 
 
  614
 
 13
 
 41764
 
 island
 
 0.19996
 
 0.57772
 
 Not queried
 
  2.4441816
 
 6.28e-04
 
 1.00e+00
  
 
  
 2
 
 223290012
 
 223290515
 
  PDF   STATS   ENSEMBL   UCSC 
 
  504
 
  4
 
 23016
 
 gene;island
 
 0.53752
 
 0.16653
 
 Not queried
 
  2.4401092
 
 2.00e-01
 
 1.00e+00
  
 
  
 10
 
  22725204
 
  22725587
 
  PDF   STATS   ENSEMBL   UCSC 
 
  384
 
  5
 
 5586
 
 island
 
 0.90684
 
 0.64207
 
 Not queried
 
  2.4399811
 
 7.94e-03
 
 1.00e+00
  
 
  
 2
 
 242212422
 
 242212528
 
  PDF   STATS   ENSEMBL   UCSC 
 
  107
 
  5
 
 23096
 
 gene;tss;island
 
 0.13327
 
 0.02844
 
 Not queried
 
  2.4396046
 
 5.48e-01
 
 1.00e+00
  
 
  
 5
 
   1882885
 
   1883011
 
  PDF   STATS   ENSEMBL   UCSC 
 
  127
 
  4
 
 31915
 
 tss
 
 0.01361
 
 0.13235
 
 Not queried
 
  2.4373796
 
 2.00e-01
 
 1.00e+00
  
 
  
 X
 
  47930589
 
  47930653
 
  PDF   STATS   ENSEMBL   UCSC 
 
   65
 
  4
 
 41288
 
 tss;island
 
 0.15301
 
 0.43453
 
 Not queried
 
  2.4358390
 
 2.86e-02
 
 1.00e+00
  
 
  
 1
 
  47696212
 
  47696788
 
  PDF   STATS   ENSEMBL   UCSC 
 
  577
 
  6
 
 1604
 
 tss;island
 
 0.20835
 
 0.02615
 
 Not queried
 
  2.4356038
 
 8.66e-03
 
 1.00e+00
  
 
  
 6
 
  32526263
 
  32526414
 
  PDF   STATS   ENSEMBL   UCSC 
 
  152
 
  5
 
 34054
 
 gene
 
 0.20724
 
 0.58290
 
 Not queried
 
  2.4352573
 
 4.21e-01
 
 1.00e+00
  
 
  
 14
 
 103739856
 
 103740085
 
  PDF   STATS   ENSEMBL   UCSC 
 
  230
 
  4
 
 13156
 
 island
 
 0.64849
 
 0.92099
 
 Not queried
 
  2.4340149
 
 2.86e-02
 
 1.00e+00
  
 
  
 7
 
  29519297
 
  29519450
 
  PDF   STATS   ENSEMBL   UCSC 
 
  154
 
  5
 
 37446
 
 tss
 
 0.39535
 
 0.05908
 
 Not queried
 
  2.4337787
 
 1.51e-01
 
 1.00e+00
  
 
  
 2
 
 175205716
 
 175205852
 
  PDF   STATS   ENSEMBL   UCSC 
 
  137
 
  4
 
 24837
 
 island
 
 0.17727
 
 0.04286
 
 Not queried
 
  2.4336203
 
 1.14e-01
 
 1.00e+00
  
 
  
 17
 
  68128754
 
  68128923
 
  PDF   STATS   ENSEMBL   UCSC 
 
  170
 
  4
 
 16708
 
 gene
 
 0.95642
 
 0.82149
 
 Not queried
 
  2.4335607
 
 1.14e-01
 
 1.00e+00
  
 
  
 4
 
  53617076
 
  53617448
 
  PDF   STATS   ENSEMBL   UCSC 
 
  373
 
  5
 
 30993
 
 island
 
 0.34254
 
 0.06898
 
 Not queried
 
  2.4332257
 
 9.52e-02
 
 1.00e+00
  
 
  
 12
 
 132168981
 
 132169130
 
  PDF   STATS   ENSEMBL   UCSC 
 
  150
 
  4
 
 10983
 
 island
 
 0.30045
 
 0.69719
 
 Not queried
 
  2.4317666
 
 2.86e-02
 
 1.00e+00
  
 
  
 17
 
   1958851
 
   1959132
 
  PDF   STATS   ENSEMBL   UCSC 
 
  282
 
  6
 
 16291
 
 gene;tss;island
 
 0.12527
 
 0.50933
 
 Not queried
 
  2.4310668
 
 2.60e-02
 
 1.00e+00
  
 
  
 15
 
  45315362
 
  45315438
 
  PDF   STATS   ENSEMBL   UCSC 
 
   77
 
  4
 
 13296
 
 gene
 
 0.01477
 
 0.00834
 
 Not queried
 
  2.4300923
 
 6.86e-01
 
 1.00e+00
  
 
  
 17
 
  46802740
 
  46803008
 
  PDF   STATS   ENSEMBL   UCSC 
 
  269
 
  4
 
 16629
 
 gene;island
 
 0.88843
 
 0.52705
 
 Not queried
 
  2.4300716
 
 2.00e-01
 
 1.00e+00
  
 
  
 19
 
  17392161
 
  17392438
 
  PDF   STATS   ENSEMBL   UCSC 
 
  278
 
  7
 
 20548
 
 tss;island
 
 0.67691
 
 0.43216
 
 Not queried
 
  2.4291453
 
 7.28e-02
 
 1.00e+00
  
 
  
 6
 
 155584613
 
 155584968
 
  PDF   STATS   ENSEMBL   UCSC 
 
  356
 
  4
 
 34456
 
 gene;tss
 
 0.81950
 
 0.96476
 
 Not queried
 
  2.4286827
 
 2.86e-02
 
 1.00e+00
  
 
  
 2
 
  27603547
 
  27604131
 
  PDF   STATS   ENSEMBL   UCSC 
 
  585
 
 12
 
 24143
 
 island
 
 0.57713
 
 0.19000
 
 Not queried
 
  2.4284145
 
 3.64e-03
 
 1.00e+00
  
 
  
 11
 
  65640375
 
  65640516
 
  PDF   STATS   ENSEMBL   UCSC 
 
  142
 
  4
 
 7426
 
 tss
 
 0.02749
 
 0.13296
 
 Not queried
 
  2.4280584
 
 5.71e-02
 
 1.00e+00
  
 
  
 6
 
  10883834
 
  10884314
 
  PDF   STATS   ENSEMBL   UCSC 
 
  481
 
  6
 
 35377
 
 island
 
 0.80530
 
 0.42888
 
 Not queried
 
  2.4276109
 
 2.16e-03
 
 1.00e+00
  
 
  
 3
 
  36986513
 
  36987040
 
  PDF   STATS   ENSEMBL   UCSC 
 
  528
 
 10
 
 28936
 
 island
 
 0.38795
 
 0.10256
 
 Not queried
 
  2.4273273
 
 8.92e-02
 
 1.00e+00
  
 
  
 10
 
  95326033
 
  95326409
 
  PDF   STATS   ENSEMBL   UCSC 
 
  377
 
  9
 
 5221
 
 tss;island
 
 0.20239
 
 0.04677
 
 Not queried
 
  2.4272522
 
 2.44e-02
 
 1.00e+00
  
 
  
 2
 
 105275613
 
 105275920
 
  PDF   STATS   ENSEMBL   UCSC 
 
  308
 
  4
 
 24538
 
 island
 
 0.03977
 
 0.18499
 
 Not queried
 
  2.4257292
 
 5.71e-02
 
 1.00e+00
  
 
  
 6
 
 158937125
 
 158937485
 
  PDF   STATS   ENSEMBL   UCSC 
 
  361
 
  4
 
 36534
 
 island
 
 0.46782
 
 0.87171
 
 Not queried
 
  2.4253472
 
 2.86e-02
 
 1.00e+00
  
 
  
 10
 
 134884177
 
 134884645
 
  PDF   STATS   ENSEMBL   UCSC 
 
  469
 
  7
 
 6311
 
 island
 
 0.55740
 
 0.82290
 
 Not queried
 
  2.4242870
 
 1.75e-02
 
 1.00e+00
  
 
  
 1
 
   1142132
 
   1142405
 
  PDF   STATS   ENSEMBL   UCSC 
 
  274
 
  4
 
 1141
 
 tss
 
 0.37994
 
 0.76588
 
 Not queried
 
  2.4240658
 
 2.86e-02
 
 1.00e+00
  
 
  
 10
 
 102590168
 
 102590401
 
  PDF   STATS   ENSEMBL   UCSC 
 
  234
 
  4
 
 6018
 
 island
 
 0.76443
 
 0.39923
 
 Not queried
 
  2.4240504
 
 2.86e-02
 
 1.00e+00
  
 
  
 X
 
  34405983
 
  34406351
 
  PDF   STATS   ENSEMBL   UCSC 
 
  369
 
  4
 
 41708
 
 island
 
 0.57552
 
 0.85003
 
 Not queried
 
  2.4223412
 
 2.86e-02
 
 1.00e+00
  
 
  
 1
 
 229542868
 
 229543254
 
  PDF   STATS   ENSEMBL   UCSC 
 
  387
 
  4
 
 4295
 
 island
 
 0.24480
 
 0.05471
 
 Not queried
 
  2.4219222
 
 5.71e-02
 
 1.00e+00
  
 
  
 13
 
  21893289
 
  21893662
 
  PDF   STATS   ENSEMBL   UCSC 
 
  374
 
  4
 
 11559
 
 island
 
 0.54714
 
 0.88132
 
 Not queried
 
  2.4210263
 
 2.86e-02
 
 1.00e+00
  
 
  
 22
 
  46731652
 
  46731750
 
  PDF   STATS   ENSEMBL   UCSC 
 
   99
 
  4
 
 26871
 
 gene;island
 
 0.00471
 
 0.01348
 
 Not queried
 
  2.4210108
 
 3.43e-01
 
 1.00e+00
  
 
  
 2
 
  27603611
 
  27604131
 
  PDF   STATS   ENSEMBL   UCSC 
 
  521
 
 11
 
 23229
 
 tss
 
 0.58975
 
 0.19293
 
 Not queried
 
  2.4201931
 
 8.33e-03
 
 1.00e+00
  
 
  
 X
 
 102531822
 
 102532056
 
  PDF   STATS   ENSEMBL   UCSC 
 
  235
 
  5
 
 41438
 
 tss
 
 0.30587
 
 0.12728
 
 Not queried
 
  2.4183140
 
 7.94e-03
 
 1.00e+00
  
 
  
 20
 
  31870775
 
  31870935
 
  PDF   STATS   ENSEMBL   UCSC 
 
  161
 
  4
 
 25642
 
 tss
 
 0.32538
 
 0.57719
 
 Not queried
 
  2.4162625
 
 2.00e-01
 
 1.00e+00
  
 
  
 1
 
   6484616
 
   6484980
 
  PDF   STATS   ENSEMBL   UCSC 
 
  365
 
  4
 
 2600
 
 island
 
 0.03587
 
 0.21979
 
 Not queried
 
  2.4161663
 
 3.43e-01
 
 1.00e+00
  
 
  
 1
 
   2398837
 
   2399054
 
  PDF   STATS   ENSEMBL   UCSC 
 
  218
 
  4
 
 2500
 
 island
 
 0.12548
 
 0.39641
 
 Not queried
 
  2.4159799
 
 3.43e-01
 
 1.00e+00
  
 
  
 15
 
  90808551
 
  90808880
 
  PDF   STATS   ENSEMBL   UCSC 
 
  330
 
  7
 
 13871
 
 tss
 
 0.06784
 
 0.03032
 
 Not queried
 
  2.4149141
 
 6.99e-03
 
 1.00e+00
  
 
  
 2
 
 102090791
 
 102091321
 
  PDF   STATS   ENSEMBL   UCSC 
 
  531
 
 11
 
 24533
 
 island
 
 0.24306
 
 0.48398
 
 Not queried
 
  2.4125353
 
 1.92e-02
 
 1.00e+00
  
 
  
 1
 
  19970204
 
  19970337
 
  PDF   STATS   ENSEMBL   UCSC 
 
  134
 
  4
 
 283
 
 gene;tss;island
 
 0.11548
 
 0.38187
 
 Not queried
 
  2.4123038
 
 1.14e-01
 
 1.00e+00
  
 
  
 1
 
 158147446
 
 158147849
 
  PDF   STATS   ENSEMBL   UCSC 
 
  404
 
  4
 
 3866
 
 island
 
 0.01163
 
 0.07164
 
 Not queried
 
  2.4122774
 
 1.14e-01
 
 1.00e+00
  
 
  
 15
 
 100890907
 
 100890996
 
  PDF   STATS   ENSEMBL   UCSC 
 
   90
 
  4
 
 13900
 
 tss;island
 
 0.16036
 
 0.36518
 
 Not queried
 
  2.4114794
 
 2.00e-01
 
 1.00e+00
  
 
  
 7
 
  50132541
 
  50133212
 
  PDF   STATS   ENSEMBL   UCSC 
 
  672
 
 12
 
 38425
 
 island
 
 0.46129
 
 0.84820
 
 Not queried
 
  2.4114782
 
 1.21e-02
 
 1.00e+00
  
 
  
 5
 
 122434853
 
 122435202
 
  PDF   STATS   ENSEMBL   UCSC 
 
  350
 
  4
 
 31675
 
 gene;island
 
 0.03421
 
 0.13935
 
 Not queried
 
  2.4113223
 
 3.43e-01
 
 1.00e+00
  
 
  
 3
 
  48471575
 
  48471901
 
  PDF   STATS   ENSEMBL   UCSC 
 
  327
 
  5
 
 28255
 
 tss;island
 
 0.67018
 
 0.33921
 
 Not queried
 
  2.4108055
 
 5.56e-02
 
 1.00e+00
  
 
  
 6
 
  44310228
 
  44310350
 
  PDF   STATS   ENSEMBL   UCSC 
 
  123
 
  6
 
 34949
 
 tss
 
 0.52642
 
 0.84326
 
 Not queried
 
  2.4106559
 
 2.16e-03
 
 1.00e+00
  
 
  
 4
 
   4866047
 
   4866192
 
  PDF   STATS   ENSEMBL   UCSC 
 
  146
 
  4
 
 30822
 
 island
 
 0.06710
 
 0.28529
 
 Not queried
 
  2.4073966
 
 2.00e-01
 
 1.00e+00
  
 
  
 16
 
  54228169
 
  54228582
 
  PDF   STATS   ENSEMBL   UCSC 
 
  414
 
  5
 
 15897
 
 island
 
 0.57211
 
 0.89491
 
 Not queried
 
  2.4072049
 
 1.59e-02
 
 1.00e+00
  
 
  
 11
 
  68780866
 
  68780984
 
  PDF   STATS   ENSEMBL   UCSC 
 
  119
 
  4
 
 7494
 
 tss;island
 
 0.37169
 
 0.77021
 
 Not queried
 
  2.4063089
 
 2.86e-02
 
 1.00e+00
  
 
  
 13
 
 114786825
 
 114786904
 
  PDF   STATS   ENSEMBL   UCSC 
 
   80
 
  4
 
 11279
 
 gene;island
 
 0.71080
 
 0.92879
 
 Not queried
 
  2.4055554
 
 2.00e-01
 
 1.00e+00
  
 
  
 3
 
 150690183
 
 150690456
 
  PDF   STATS   ENSEMBL   UCSC 
 
  274
 
  6
 
 28621
 
 tss
 
 0.18247
 
 0.56134
 
 Not queried
 
  2.4050193
 
 6.49e-02
 
 1.00e+00
  
 
  
 11
 
  63997306
 
  63997740
 
  PDF   STATS   ENSEMBL   UCSC 
 
  435
 
  7
 
 7366
 
 tss;island
 
 0.03466
 
 0.03775
 
 Not queried
 
  2.4049874
 
 6.20e-01
 
 1.00e+00
  
 
  
 2
 
   2754829
 
   2755386
 
  PDF   STATS   ENSEMBL   UCSC 
 
  558
 
  5
 
 23986
 
 island
 
 0.59600
 
 0.90046
 
 Not queried
 
  2.4044150
 
 3.17e-02
 
 1.00e+00
  
 
  
 X
 
  70521196
 
  70521650
 
  PDF   STATS   ENSEMBL   UCSC 
 
  455
 
  7
 
 41024
 
 gene
 
 0.74717
 
 0.92732
 
 Not queried
 
  2.4039483
 
 5.83e-04
 
 1.00e+00
  
 
  
 X
 
 135862961
 
 135863104
 
  PDF   STATS   ENSEMBL   UCSC 
 
  144
 
  4
 
 41122
 
 gene
 
 0.13582
 
 0.41429
 
 Not queried
 
  2.4035770
 
 2.86e-02
 
 1.00e+00
  
 
  
 X
 
  67718299
 
  67718729
 
  PDF   STATS   ENSEMBL   UCSC 
 
  431
 
  8
 
 41356
 
 tss;island
 
 0.27088
 
 0.06721
 
 Not queried
 
  2.4033197
 
 3.11e-04
 
 1.00e+00
  
 
  
 10
 
  81679675
 
  81679852
 
  PDF   STATS   ENSEMBL   UCSC 
 
  178
 
  4
 
 5167
 
 tss
 
 0.55736
 
 0.89505
 
 Not queried
 
  2.4024783
 
 1.14e-01
 
 1.00e+00
  
 
  
 11
 
  66103386
 
  66103604
 
  PDF   STATS   ENSEMBL   UCSC 
 
  219
 
  4
 
 6713
 
 gene;island
 
 0.25442
 
 0.64317
 
 Not queried
 
  2.4024189
 
 2.86e-02
 
 1.00e+00
  
 
  
 10
 
  97054967
 
  97055439
 
  PDF   STATS   ENSEMBL   UCSC 
 
  473
 
  4
 
 5945
 
 island
 
 0.22170
 
 0.04597
 
 Not queried
 
  2.4018221
 
 5.71e-02
 
 1.00e+00
  
 
  
 4
 
  53727714
 
  53728038
 
  PDF   STATS   ENSEMBL   UCSC 
 
  325
 
  5
 
 30346
 
 tss;island
 
 0.49603
 
 0.33205
 
 Not queried
 
  2.4013849
 
 1.00e+00
 
 1.00e+00
  
 
  
 11
 
  69258203
 
  69258477
 
  PDF   STATS   ENSEMBL   UCSC 
 
  275
 
  4
 
 8565
 
 island
 
 0.12114
 
 0.39331
 
 Not queried
 
  2.4005004
 
 2.86e-02
 
 1.00e+00
  
 
  
 7
 
 100199764
 
 100200009
 
  PDF   STATS   ENSEMBL   UCSC 
 
  246
 
  6
 
 38706
 
 island
 
 0.89798
 
 0.63387
 
 Not queried
 
  2.4002976
 
 2.16e-03
 
 1.00e+00
  
 
  
 17
 
   6616644
 
   6617192
 
  PDF   STATS   ENSEMBL   UCSC 
 
  549
 
  8
 
 17876
 
 island
 
 0.33212
 
 0.08463
 
 Not queried
 
  2.4001037
 
 2.81e-02
 
 1.00e+00
  
 
  
 6
 
  28497278
 
  28497370
 
  PDF   STATS   ENSEMBL   UCSC 
 
   93
 
  4
 
 33571
 
 gene
 
 0.92317
 
 0.66312
 
 Not queried
 
  2.3991480
 
 2.86e-02
 
 1.00e+00
  
 
  
 3
 
 149768455
 
 149768762
 
  PDF   STATS   ENSEMBL   UCSC 
 
  308
 
  5
 
 29543
 
 island
 
 0.81433
 
 0.95798
 
 Not queried
 
  2.3984130
 
 7.94e-03
 
 1.00e+00
  
 
  
 1
 
 226070448
 
 226070808
 
  PDF   STATS   ENSEMBL   UCSC 
 
  361
 
  7
 
 2279
 
 tss
 
 0.12514
 
 0.03047
 
 Not queried
 
  2.3981170
 
 2.62e-02
 
 1.00e+00
  
 
  
 17
 
  47209660
 
  47209827
 
  PDF   STATS   ENSEMBL   UCSC 
 
  168
 
  4
 
 17448
 
 tss;island
 
 0.88183
 
 0.40609
 
 Not queried
 
  2.3979763
 
 2.00e-01
 
 1.00e+00
  
 
  
 2
 
  89065319
 
  89065379
 
  PDF   STATS   ENSEMBL   UCSC 
 
   61
 
  4
 
 23466
 
 tss
 
 0.21685
 
 0.53189
 
 Not queried
 
  2.3975179
 
 2.00e-01
 
 1.00e+00
  
 
  
 1
 
  26737005
 
  26737470
 
  PDF   STATS   ENSEMBL   UCSC 
 
  466
 
  8
 
 2889
 
 island
 
 0.04360
 
 0.20213
 
 Not queried
 
  2.3971391
 
 1.09e-03
 
 1.00e+00
  
 
  
 14
 
  57275166
 
  57275413
 
  PDF   STATS   ENSEMBL   UCSC 
 
  248
 
  4
 
 12064
 
 gene;island
 
 0.29413
 
 0.04513
 
 Not queried
 
  2.3970713
 
 5.71e-02
 
 1.00e+00
  
 
  
 6
 
 168377452
 
 168377744
 
  PDF   STATS   ENSEMBL   UCSC 
 
  293
 
  6
 
 36587
 
 island
 
 0.83671
 
 0.91231
 
 Not queried
 
  2.3964719
 
 1.32e-01
 
 1.00e+00
  
 
  
 12
 
  50297477
 
  50297945
 
  PDF   STATS   ENSEMBL   UCSC 
 
  469
 
  7
 
 10380
 
 island
 
 0.37188
 
 0.63470
 
 Not queried
 
  2.3942413
 
 1.17e-03
 
 1.00e+00
  
 
  
 12
 
    372049
 
    372259
 
  PDF   STATS   ENSEMBL   UCSC 
 
  211
 
  5
 
 9450
 
 tss
 
 0.30903
 
 0.68371
 
 Not queried
 
  2.3932805
 
 5.56e-02
 
 1.00e+00
  
 
  
 2
 
 171570700
 
 171571151
 
  PDF   STATS   ENSEMBL   UCSC 
 
  452
 
  6
 
 23670
 
 tss;island
 
 0.17628
 
 0.03387
 
 Not queried
 
  2.3917727
 
 4.33e-03
 
 1.00e+00
  
 
  
 19
 
  41129881
 
  41130041
 
  PDF   STATS   ENSEMBL   UCSC 
 
  161
 
  4
 
 20013
 
 gene;island
 
 0.01635
 
 0.04554
 
 Not queried
 
  2.3896944
 
 4.86e-01
 
 1.00e+00
  
 
  
 2
 
   3383078
 
   3383502
 
  PDF   STATS   ENSEMBL   UCSC 
 
  425
 
  6
 
 23992
 
 island
 
 0.09000
 
 0.03817
 
 Not queried
 
  2.3896743
 
 3.94e-01
 
 1.00e+00
  
 
  
 8
 
   9008364
 
   9008681
 
  PDF   STATS   ENSEMBL   UCSC 
 
  318
 
  6
 
 39480
 
 tss
 
 0.08829
 
 0.03577
 
 Not queried
 
  2.3884633
 
 4.11e-02
 
 1.00e+00
  
 
  
 19
 
  54466187
 
  54466724
 
  PDF   STATS   ENSEMBL   UCSC 
 
  538
 
  6
 
 22356
 
 island
 
 0.56789
 
 0.23312
 
 Not queried
 
  2.3874372
 
 8.66e-03
 
 1.00e+00
  
 
  
 12
 
  96350519
 
  96350795
 
  PDF   STATS   ENSEMBL   UCSC 
 
  277
 
  5
 
 9230
 
 gene;island
 
 0.92581
 
 0.70489
 
 Not queried
 
  2.3853080
 
 7.94e-03
 
 1.00e+00
  
 
  
 5
 
 142065671
 
 142065819
 
  PDF   STATS   ENSEMBL   UCSC 
 
  149
 
  5
 
 32339
 
 tss
 
 0.47531
 
 0.81882
 
 Not queried
 
  2.3836395
 
 5.56e-02
 
 1.00e+00
  
 
  
 10
 
  35484768
 
  35484972
 
  PDF   STATS   ENSEMBL   UCSC 
 
  205
 
  4
 
 4545
 
 gene
 
 0.04649
 
 0.01407
 
 Not queried
 
  2.3807838
 
 4.86e-01
 
 1.00e+00
  
 
  
 5
 
  37835506
 
  37836392
 
  PDF   STATS   ENSEMBL   UCSC 
 
  887
 
 11
 
 31535
 
 gene;tss;island
 
 0.90825
 
 0.64167
 
 Not queried
 
  2.3805640
 
 3.36e-02
 
 1.00e+00
  
 
  
 7
 
  89783767
 
  89783976
 
  PDF   STATS   ENSEMBL   UCSC 
 
  210
 
  4
 
 37017
 
 gene
 
 0.05311
 
 0.21225
 
 Not queried
 
  2.3790097
 
 2.00e-01
 
 1.00e+00
  
 
  
 12
 
  75603068
 
  75603480
 
  PDF   STATS   ENSEMBL   UCSC 
 
  413
 
  5
 
 9195
 
 gene
 
 0.11642
 
 0.29241
 
 Not queried
 
  2.3789408
 
 1.51e-01
 
 1.00e+00
  
 
  
 12
 
  75603068
 
  75603748
 
  PDF   STATS   ENSEMBL   UCSC 
 
  681
 
  7
 
 10605
 
 island
 
 0.11642
 
 0.29241
 
 Not queried
 
  2.3789408
 
 2.09e-01
 
 1.00e+00
  
 
  
 12
 
 101604027
 
 101604258
 
  PDF   STATS   ENSEMBL   UCSC 
 
  232
 
  4
 
 9918
 
 tss
 
 0.02316
 
 0.10988
 
 Not queried
 
  2.3787055
 
 2.00e-01
 
 1.00e+00
  
 
  
 6
 
  73972704
 
  73973128
 
  PDF   STATS   ENSEMBL   UCSC 
 
  425
 
  9
 
 36186
 
 island
 
 0.18145
 
 0.03920
 
 Not queried
 
  2.3780369
 
 2.44e-02
 
 1.00e+00
  
 
  
 X
 
  34960814
 
  34960973
 
  PDF   STATS   ENSEMBL   UCSC 
 
  160
 
  6
 
 41709
 
 island
 
 0.58331
 
 0.88537
 
 Not queried
 
  2.3776667
 
 2.16e-03
 
 1.00e+00
  
 
  
 5
 
 172660796
 
 172661132
 
  PDF   STATS   ENSEMBL   UCSC 
 
  337
 
  4
 
 31844
 
 gene;island
 
 0.58351
 
 0.83325
 
 Not queried
 
  2.3774432
 
 1.14e-01
 
 1.00e+00
  
 
  
 13
 
 113705742
 
 113706204
 
  PDF   STATS   ENSEMBL   UCSC 
 
  463
 
  4
 
 11244
 
 gene;island
 
 0.71830
 
 0.93099
 
 Not queried
 
  2.3748428
 
 5.71e-02
 
 1.00e+00
  
 
  
 12
 
 132689907
 
 132690782
 
  PDF   STATS   ENSEMBL   UCSC 
 
  876
 
 11
 
 9408
 
 gene;island
 
 0.65594
 
 0.85621
 
 Not queried
 
  2.3744974
 
 1.40e-03
 
 1.00e+00
  
 
  
 1
 
   7023495
 
   7023864
 
  PDF   STATS   ENSEMBL   UCSC 
 
  370
 
  4
 
 183
 
 gene;island
 
 0.20284
 
 0.03109
 
 Not queried
 
  2.3740010
 
 2.00e-01
 
 1.00e+00
  
 
  
 5
 
 141061863
 
 141061943
 
  PDF   STATS   ENSEMBL   UCSC 
 
   81
 
  6
 
 32331
 
 tss
 
 0.03732
 
 0.18017
 
 Not queried
 
  2.3739891
 
 9.31e-02
 
 1.00e+00
  
 
  
 16
 
  76269043
 
  76269409
 
  PDF   STATS   ENSEMBL   UCSC 
 
  367
 
  5
 
 16072
 
 island
 
 0.51351
 
 0.82452
 
 Not queried
 
  2.3733836
 
 7.94e-03
 
 1.00e+00
  
 
  
 1
 
   3459949
 
   3460467
 
  PDF   STATS   ENSEMBL   UCSC 
 
  519
 
  4
 
 128
 
 gene;island
 
 0.31266
 
 0.77636
 
 Not queried
 
  2.3720859
 
 2.00e-01
 
 1.00e+00
  
 
  
 3
 
 180319597
 
 180319907
 
  PDF   STATS   ENSEMBL   UCSC 
 
  311
 
  6
 
 28692
 
 tss
 
 0.14953
 
 0.04812
 
 Not queried
 
  2.3718257
 
 1.80e-01
 
 1.00e+00
  
 
  
 11
 
  65640137
 
  65640516
 
  PDF   STATS   ENSEMBL   UCSC 
 
  380
 
  6
 
 8463
 
 island
 
 0.04869
 
 0.20582
 
 Not queried
 
  2.3696613
 
 2.60e-02
 
 1.00e+00
  
 
  
 11
 
  31823141
 
  31823282
 
  PDF   STATS   ENSEMBL   UCSC 
 
  142
 
  4
 
 6540
 
 gene;island
 
 0.23571
 
 0.58864
 
 Not queried
 
  2.3685076
 
 2.86e-02
 
 1.00e+00
  
 
  
 10
 
  95326801
 
  95327325
 
  PDF   STATS   ENSEMBL   UCSC 
 
  525
 
  6
 
 4668
 
 gene;island
 
 0.16749
 
 0.41797
 
 Not queried
 
  2.3684546
 
 9.31e-02
 
 1.00e+00
  
 
  
 13
 
 112720171
 
 112720565
 
  PDF   STATS   ENSEMBL   UCSC 
 
  395
 
  6
 
 11879
 
 island
 
 0.40287
 
 0.09210
 
 Not queried
 
  2.3683528
 
 6.49e-02
 
 1.00e+00
  
 
  
 5
 
 166405485
 
 166405869
 
  PDF   STATS   ENSEMBL   UCSC 
 
  385
 
  4
 
 33304
 
 island
 
 0.28512
 
 0.04045
 
 Not queried
 
  2.3666099
 
 2.00e-01
 
 1.00e+00
  
 
  
 18
 
  71959244
 
  71959795
 
  PDF   STATS   ENSEMBL   UCSC 
 
  552
 
  8
 
 19287
 
 tss
 
 0.11780
 
 0.01793
 
 Not queried
 
  2.3662970
 
 3.11e-04
 
 1.00e+00
  
 
  
 19
 
  55791810
 
  55792075
 
  PDF   STATS   ENSEMBL   UCSC 
 
  266
 
  5
 
 21038
 
 tss
 
 0.24925
 
 0.06530
 
 Not queried
 
  2.3649822
 
 5.48e-01
 
 1.00e+00
  
 
  
 5
 
 140566303
 
 140566767
 
  PDF   STATS   ENSEMBL   UCSC 
 
  465
 
  5
 
 32306
 
 tss;island
 
 0.06881
 
 0.65320
 
 Not queried
 
  2.3645202
 
 3.10e-01
 
 1.00e+00
  
 
  
 9
 
 139948856
 
 139949108
 
  PDF   STATS   ENSEMBL   UCSC 
 
  253
 
  4
 
 40792
 
 tss;island
 
 0.33063
 
 0.69594
 
 Not queried
 
  2.3642609
 
 3.43e-01
 
 1.00e+00
  
 
  
 5
 
 140501321
 
 140501812
 
  PDF   STATS   ENSEMBL   UCSC 
 
  492
 
 10
 
 33149
 
 island
 
 0.06960
 
 0.58274
 
 Not queried
 
  2.3637028
 
 2.88e-02
 
 1.00e+00
  
 
  
 2
 
    214012
 
    214353
 
  PDF   STATS   ENSEMBL   UCSC 
 
  342
 
  4
 
 23959
 
 island
 
 0.68005
 
 0.88921
 
 Not queried
 
  2.3636344
 
 2.86e-02
 
 1.00e+00
  
 
  
 19
 
  47292038
 
  47292253
 
  PDF   STATS   ENSEMBL   UCSC 
 
  216
 
  4
 
 20863
 
 tss;island
 
 0.32978
 
 0.03104
 
 Not queried
 
  2.3635627
 
 2.00e-01
 
 1.00e+00
  
 
  
 12
 
 124941307
 
 124941423
 
  PDF   STATS   ENSEMBL   UCSC 
 
  117
 
  4
 
 9370
 
 gene;island
 
 0.61861
 
 0.88385
 
 Not queried
 
  2.3627438
 
 2.86e-02
 
 1.00e+00
  
 
  
 6
 
  75911716
 
  75912132
 
  PDF   STATS   ENSEMBL   UCSC 
 
  417
 
  4
 
 34328
 
 gene;island
 
 0.45670
 
 0.11416
 
 Not queried
 
  2.3622565
 
 2.86e-02
 
 1.00e+00
  
 
  
 4
 
   1005876
 
   1006240
 
  PDF   STATS   ENSEMBL   UCSC 
 
  365
 
  8
 
 30189
 
 tss
 
 0.07508
 
 0.04785
 
 Not queried
 
  2.3612276
 
 5.74e-01
 
 1.00e+00
  
 
  
 3
 
 147109426
 
 147109784
 
  PDF   STATS   ENSEMBL   UCSC 
 
  359
 
  4
 
 27943
 
 gene;island
 
 0.17112
 
 0.47719
 
 Not queried
 
  2.3601577
 
 3.43e-01
 
 1.00e+00
  
 
  
 3
 
  44596178
 
  44596770
 
  PDF   STATS   ENSEMBL   UCSC 
 
  593
 
  9
 
 28992
 
 island
 
 0.86903
 
 0.62754
 
 Not queried
 
  2.3601288
 
 2.58e-01
 
 1.00e+00
  
 
  
 1
 
 230468464
 
 230468773
 
  PDF   STATS   ENSEMBL   UCSC 
 
  310
 
  5
 
 1068
 
 gene;island
 
 0.06871
 
 0.36522
 
 Not queried
 
  2.3599362
 
 3.17e-02
 
 1.00e+00
  
 
  
 2
 
 223163573
 
 223163809
 
  PDF   STATS   ENSEMBL   UCSC 
 
  237
 
  5
 
 23011
 
 gene;island
 
 0.17816
 
 0.02686
 
 Not queried
 
  2.3590559
 
 3.17e-02
 
 1.00e+00
  
 
  
 8
 
  30892276
 
  30892490
 
  PDF   STATS   ENSEMBL   UCSC 
 
  215
 
  4
 
 39210
 
 gene;tss
 
 0.45075
 
 0.11580
 
 Not queried
 
  2.3588603
 
 1.14e-01
 
 1.00e+00
  
 
  
 18
 
  56296243
 
  56296607
 
  PDF   STATS   ENSEMBL   UCSC 
 
  365
 
  6
 
 19260
 
 tss
 
 0.55902
 
 0.87804
 
 Not queried
 
  2.3581440
 
 2.60e-02
 
 1.00e+00
  
 
  
 1
 
  32169568
 
  32169868
 
  PDF   STATS   ENSEMBL   UCSC 
 
  301
 
  4
 
 2967
 
 island
 
 0.86191
 
 0.56744
 
 Not queried
 
  2.3577858
 
 2.86e-02
 
 1.00e+00
  
 
  
 12
 
 112204716
 
 112204829
 
  PDF   STATS   ENSEMBL   UCSC 
 
  114
 
  4
 
 9286
 
 gene;island
 
 0.15941
 
 0.02693
 
 Not queried
 
  2.3569385
 
 1.14e-01
 
 1.00e+00
  
 
  
 12
 
  58011764
 
  58011875
 
  PDF   STATS   ENSEMBL   UCSC 
 
  112
 
  4
 
 10540
 
 island
 
 0.33188
 
 0.72105
 
 Not queried
 
  2.3563780
 
 3.43e-01
 
 1.00e+00
  
 
  
 11
 
   1404502
 
   1404750
 
  PDF   STATS   ENSEMBL   UCSC 
 
  249
 
  4
 
 7899
 
 island
 
 0.05509
 
 0.20415
 
 Not queried
 
  2.3555453
 
 2.86e-02
 
 1.00e+00
  
 
  
 11
 
  65425928
 
  65426084
 
  PDF   STATS   ENSEMBL   UCSC 
 
  157
 
  5
 
 6698
 
 gene
 
 0.58713
 
 0.87916
 
 Not queried
 
  2.3549986
 
 7.94e-03
 
 1.00e+00
  
 
  
 5
 
  16508920
 
  16509123
 
  PDF   STATS   ENSEMBL   UCSC 
 
  204
 
  4
 
 31511
 
 gene
 
 0.13520
 
 0.44216
 
 Not queried
 
  2.3538124
 
 2.86e-02
 
 1.00e+00
  
 
  
 5
 
 178487716
 
 178488100
 
  PDF   STATS   ENSEMBL   UCSC 
 
  385
 
  4
 
 31869
 
 gene
 
 0.24941
 
 0.50058
 
 Not queried
 
  2.3526150
 
 4.86e-01
 
 1.00e+00
  
 
  
 17
 
  46824727
 
  46825371
 
  PDF   STATS   ENSEMBL   UCSC 
 
  645
 
  5
 
 18472
 
 island
 
 0.23760
 
 0.61202
 
 Not queried
 
  2.3519161
 
 1.59e-02
 
 1.00e+00
  
 
  
 5
 
 142065539
 
 142065819
 
  PDF   STATS   ENSEMBL   UCSC 
 
  281
 
  6
 
 31786
 
 gene
 
 0.48415
 
 0.79747
 
 Not queried
 
  2.3512043
 
 2.60e-02
 
 1.00e+00
  
 
  
 12
 
 101603835
 
 101603996
 
  PDF   STATS   ENSEMBL   UCSC 
 
  162
 
  4
 
 9242
 
 gene
 
 0.01696
 
 0.06737
 
 Not queried
 
  2.3507743
 
 1.14e-01
 
 1.00e+00
  
 
  
 12
 
 101603835
 
 101604258
 
  PDF   STATS   ENSEMBL   UCSC 
 
  424
 
  8
 
 10690
 
 island
 
 0.01914
 
 0.06737
 
 Not queried
 
  2.3507743
 
 2.07e-02
 
 1.00e+00
  
 
  
 1
 
    936091
 
    936610
 
  PDF   STATS   ENSEMBL   UCSC 
 
  520
 
  5
 
 1134
 
 tss;island
 
 0.03622
 
 0.40415
 
 Not queried
 
  2.3504022
 
 3.17e-02
 
 1.00e+00
  
 
  
 22
 
  18531028
 
  18531591
 
  PDF   STATS   ENSEMBL   UCSC 
 
  564
 
  6
 
 27240
 
 island
 
 0.49552
 
 0.83869
 
 Not queried
 
  2.3502671
 
 8.66e-03
 
 1.00e+00
  
 
  
 4
 
  46126245
 
  46126448
 
  PDF   STATS   ENSEMBL   UCSC 
 
  204
 
  6
 
 30324
 
 tss
 
 0.05423
 
 0.32280
 
 Not queried
 
  2.3498384
 
 6.49e-02
 
 1.00e+00
  
 
  
 X
 
  74743375
 
  74743636
 
  PDF   STATS   ENSEMBL   UCSC 
 
  262
 
  5
 
 41396
 
 tss;island
 
 0.10205
 
 0.02418
 
 Not queried
 
  2.3497420
 
 7.94e-03
 
 1.00e+00
  
 
  
 5
 
  68710808
 
  68710912
 
  PDF   STATS   ENSEMBL   UCSC 
 
  105
 
  6
 
 32051
 
 tss;island
 
 0.03019
 
 0.13718
 
 Not queried
 
  2.3492950
 
 8.66e-03
 
 1.00e+00
  
 
  
 19
 
  58545001
 
  58545333
 
  PDF   STATS   ENSEMBL   UCSC 
 
  333
 
  8
 
 21094
 
 tss;island
 
 0.03064
 
 0.11710
 
 Not queried
 
  2.3491482
 
 6.99e-03
 
 1.00e+00
  
 
  
 8
 
   1496711
 
   1496759
 
  PDF   STATS   ENSEMBL   UCSC 
 
   49
 
  4
 
 39104
 
 gene;island
 
 0.68456
 
 0.90856
 
 Not queried
 
  2.3489337
 
 2.86e-02
 
 1.00e+00
  
 
  
 10
 
 102896376
 
 102896640
 
  PDF   STATS   ENSEMBL   UCSC 
 
  265
 
  4
 
 4711
 
 gene;island
 
 0.93520
 
 0.64312
 
 Not queried
 
  2.3485061
 
 2.86e-02
 
 1.00e+00
  
 
  
 7
 
 157982161
 
 157982589
 
  PDF   STATS   ENSEMBL   UCSC 
 
  429
 
  5
 
 37297
 
 gene;island
 
 0.41888
 
 0.79197
 
 Not queried
 
  2.3481635
 
 2.22e-01
 
 1.00e+00
  
 
  
 10
 
  35894012
 
  35894430
 
  PDF   STATS   ENSEMBL   UCSC 
 
  419
 
  8
 
 5647
 
 island
 
 0.44195
 
 0.86746
 
 Not queried
 
  2.3464194
 
 1.86e-03
 
 1.00e+00
  
 
  
 16
 
  74808678
 
  74808761
 
  PDF   STATS   ENSEMBL   UCSC 
 
   84
 
  5
 
 16058
 
 island
 
 0.01216
 
 0.01658
 
 Not queried
 
  2.3458527
 
 2.22e-01
 
 1.00e+00
  
 
  
 12
 
 128850432
 
 128850696
 
  PDF   STATS   ENSEMBL   UCSC 
 
  265
 
  5
 
 9380
 
 gene;island
 
 0.25633
 
 0.59923
 
 Not queried
 
  2.3452787
 
 7.94e-03
 
 1.00e+00
  
 
  
 6
 
  30690213
 
  30690844
 
  PDF   STATS   ENSEMBL   UCSC 
 
  632
 
 14
 
 33753
 
 gene;island
 
 0.09183
 
 0.41452
 
 Not queried
 
  2.3451503
 
 2.49e-03
 
 1.00e+00
  
 
  
 12
 
  53693054
 
  53693453
 
  PDF   STATS   ENSEMBL   UCSC 
 
  400
 
  8
 
 9713
 
 tss
 
 0.05014
 
 0.25535
 
 Not queried
 
  2.3438402
 
 1.95e-01
 
 1.00e+00
  
 
  
 12
 
  50349173
 
  50349522
 
  PDF   STATS   ENSEMBL   UCSC 
 
  350
 
  4
 
 9101
 
 gene;island
 
 0.86809
 
 0.61145
 
 Not queried
 
  2.3430765
 
 2.00e-01
 
 1.00e+00
  
 
  
 10
 
  27703329
 
  27703547
 
  PDF   STATS   ENSEMBL   UCSC 
 
  219
 
  6
 
 4985
 
 tss
 
 0.37285
 
 0.84329
 
 Not queried
 
  2.3416249
 
 1.52e-02
 
 1.00e+00
  
 
  
 10
 
  27703247
 
  27703547
 
  PDF   STATS   ENSEMBL   UCSC 
 
  301
 
  8
 
 5612
 
 island
 
 0.32199
 
 0.73106
 
 Not queried
 
  2.3416249
 
 6.99e-03
 
 1.00e+00
  
 
  
 2
 
 155554688
 
 155555414
 
  PDF   STATS   ENSEMBL   UCSC 
 
  727
 
  9
 
 24759
 
 island
 
 0.07269
 
 0.50187
 
 Not queried
 
  2.3408938
 
 7.70e-02
 
 1.00e+00
  
 
  
 10
 
  27547536
 
  27547965
 
  PDF   STATS   ENSEMBL   UCSC 
 
  430
 
  6
 
 5610
 
 island
 
 0.15250
 
 0.03707
 
 Not queried
 
  2.3398429
 
 1.80e-01
 
 1.00e+00
  
 
  
 21
 
  15352790
 
  15352983
 
  PDF   STATS   ENSEMBL   UCSC 
 
  194
 
  5
 
 26445
 
 tss
 
 0.19327
 
 0.47338
 
 Not queried
 
  2.3397770
 
 5.56e-02
 
 1.00e+00
  
 
  
 21
 
  15352608
 
  15352983
 
  PDF   STATS   ENSEMBL   UCSC 
 
  376
 
  7
 
 26565
 
 island
 
 0.16295
 
 0.39675
 
 Not queried
 
  2.3397770
 
 1.11e-02
 
 1.00e+00
  
 
  
 7
 
  73256360
 
  73256820
 
  PDF   STATS   ENSEMBL   UCSC 
 
  461
 
  6
 
 36980
 
 gene
 
 0.36246
 
 0.07547
 
 Not queried
 
  2.3392773
 
 2.40e-01
 
 1.00e+00
  
 
  
 7
 
  29519297
 
  29519656
 
  PDF   STATS   ENSEMBL   UCSC 
 
  360
 
  8
 
 36886
 
 gene
 
 0.34363
 
 0.05670
 
 Not queried
 
  2.3390792
 
 2.07e-02
 
 1.00e+00
  
 
  
 10
 
 101290958
 
 101291397
 
  PDF   STATS   ENSEMBL   UCSC 
 
  440
 
  4
 
 5992
 
 island
 
 0.72418
 
 0.40395
 
 Not queried
 
  2.3379505
 
 2.86e-02
 
 1.00e+00
  
 
  
 15
 
  90030016
 
  90030410
 
  PDF   STATS   ENSEMBL   UCSC 
 
  395
 
  5
 
 13440
 
 gene;island
 
 0.24803
 
 0.62503
 
 Not queried
 
  2.3372832
 
 3.10e-01
 
 1.00e+00
  
 
  
 11
 
   1891699
 
   1892142
 
  PDF   STATS   ENSEMBL   UCSC 
 
  444
 
 10
 
 7030
 
 tss
 
 0.49217
 
 0.77104
 
 Not queried
 
  2.3368456
 
 2.32e-02
 
 1.00e+00
  
 
  
 6
 
  27228101
 
  27228330
 
  PDF   STATS   ENSEMBL   UCSC 
 
  230
 
  4
 
 35488
 
 island
 
 0.38608
 
 0.12147
 
 Not queried
 
  2.3347507
 
 1.14e-01
 
 1.00e+00
  
 
  
 14
 
  99880393
 
  99880641
 
  PDF   STATS   ENSEMBL   UCSC 
 
  249
 
  4
 
 12175
 
 gene
 
 0.94431
 
 0.77023
 
 Not queried
 
  2.3346696
 
 1.14e-01
 
 1.00e+00
  
 
  
 8
 
  97169621
 
  97170097
 
  PDF   STATS   ENSEMBL   UCSC 
 
  477
 
  5
 
 39303
 
 gene;island
 
 0.22381
 
 0.61335
 
 Not queried
 
  2.3341886
 
 1.59e-02
 
 1.00e+00
  
 
  
 20
 
  22565881
 
  22566289
 
  PDF   STATS   ENSEMBL   UCSC 
 
  409
 
  6
 
 25594
 
 tss;island
 
 0.07792
 
 0.20382
 
 Not queried
 
  2.3337428
 
 1.52e-02
 
 1.00e+00
  
 
  
 7
 
 114055074
 
 114055210
 
  PDF   STATS   ENSEMBL   UCSC 
 
  137
 
  6
 
 37129
 
 gene
 
 0.01058
 
 0.06058
 
 Not queried
 
  2.3321595
 
 4.11e-02
 
 1.00e+00
  
 
  
 17
 
  61514690
 
  61515010
 
  PDF   STATS   ENSEMBL   UCSC 
 
  321
 
  4
 
 16690
 
 gene;island
 
 0.65023
 
 0.90514
 
 Not queried
 
  2.3308502
 
 2.00e-01
 
 1.00e+00
  
 
  
 6
 
  34499314
 
  34499504
 
  PDF   STATS   ENSEMBL   UCSC 
 
  191
 
  4
 
 34239
 
 gene;island
 
 0.71366
 
 0.93510
 
 Not queried
 
  2.3301640
 
 2.00e-01
 
 1.00e+00
  
 
  
 Y
 
  15016579
 
  15016705
 
  PDF   STATS   ENSEMBL   UCSC 
 
  127
 
  5
 
 42160
 
 gene;island
 
 0.11017
 
 0.48930
 
 Not queried
 
  2.3292507
 
 7.94e-03
 
 1.00e+00
  
 
  
 X
 
  38115344
 
  38115655
 
  PDF   STATS   ENSEMBL   UCSC 
 
  312
 
  4
 
 41719
 
 island
 
 0.40375
 
 0.84128
 
 Not queried
 
  2.3290820
 
 5.71e-02
 
 1.00e+00
  
 
  
 10
 
  75407372
 
  75407991
 
  PDF   STATS   ENSEMBL   UCSC 
 
  620
 
  6
 
 4622
 
 gene;island
 
 0.26253
 
 0.67922
 
 Not queried
 
  2.3281885
 
 2.60e-02
 
 1.00e+00
  
 
  
 2
 
  66659348
 
  66659590
 
  PDF   STATS   ENSEMBL   UCSC 
 
  243
 
  4
 
 24317
 
 island
 
 0.17736
 
 0.04171
 
 Not queried
 
  2.3275964
 
 5.71e-02
 
 1.00e+00
  
 
  
 16
 
  12996046
 
  12996587
 
  PDF   STATS   ENSEMBL   UCSC 
 
  542
 
  6
 
 14642
 
 gene;island
 
 0.01672
 
 0.05437
 
 Not queried
 
  2.3268750
 
 3.10e-01
 
 1.00e+00
  
 
  
 1
 
 205649791
 
 205649956
 
  PDF   STATS   ENSEMBL   UCSC 
 
  166
 
  8
 
 2203
 
 tss;island
 
 0.18049
 
 0.03079
 
 Not queried
 
  2.3254949
 
 3.11e-04
 
 1.00e+00
  
 
  
 5
 
 140501321
 
 140501540
 
  PDF   STATS   ENSEMBL   UCSC 
 
  220
 
  7
 
 32303
 
 tss
 
 0.08670
 
 0.46850
 
 Not queried
 
  2.3252775
 
 2.09e-01
 
 1.00e+00
  
 
  
 12
 
  49487944
 
  49488311
 
  PDF   STATS   ENSEMBL   UCSC 
 
  368
 
  4
 
 9091
 
 gene;island
 
 0.07855
 
 0.21955
 
 Not queried
 
  2.3245854
 
 2.86e-02
 
 1.00e+00
  
 
  
 20
 
  35169260
 
  35169609
 
  PDF   STATS   ENSEMBL   UCSC 
 
  350
 
  5
 
 25677
 
 tss;island
 
 0.43317
 
 0.87463
 
 Not queried
 
  2.3242424
 
 3.17e-02
 
 1.00e+00
  
 
  
 7
 
 157561593
 
 157561925
 
  PDF   STATS   ENSEMBL   UCSC 
 
  333
 
  5
 
 37279
 
 gene;island
 
 0.83898
 
 0.95684
 
 Not queried
 
  2.3240683
 
 1.59e-02
 
 1.00e+00
  
 
  
 16
 
   3096477
 
   3096662
 
  PDF   STATS   ENSEMBL   UCSC 
 
  186
 
  5
 
 15018
 
 tss
 
 0.23891
 
 0.02886
 
 Not queried
 
  2.3238480
 
 1.51e-01
 
 1.00e+00
  
 
  
 X
 
  72782624
 
  72782852
 
  PDF   STATS   ENSEMBL   UCSC 
 
  229
 
  5
 
 41035
 
 gene
 
 0.09375
 
 0.34120
 
 Not queried
 
  2.3237810
 
 7.94e-03
 
 1.00e+00
  
 
  
 3
 
  42543933
 
  42544101
 
  PDF   STATS   ENSEMBL   UCSC 
 
  169
 
  6
 
 28201
 
 tss
 
 0.13055
 
 0.02392
 
 Not queried
 
  2.3226169
 
 8.66e-03
 
 1.00e+00
  
 
  
 6
 
  31733619
 
  31734580
 
  PDF   STATS   ENSEMBL   UCSC 
 
  962
 
 20
 
 33888
 
 gene
 
 0.22033
 
 0.53609
 
 Not queried
 
  2.3225435
 
 5.62e-03
 
 1.00e+00
  
 
  
 13
 
  28498091
 
  28498544
 
  PDF   STATS   ENSEMBL   UCSC 
 
  454
 
  6
 
 11089
 
 gene;island
 
 0.65591
 
 0.25979
 
 Not queried
 
  2.3221176
 
 8.66e-03
 
 1.00e+00
  
 
  
 11
 
 101000436
 
 101000717
 
  PDF   STATS   ENSEMBL   UCSC 
 
  282
 
  6
 
 8731
 
 island
 
 0.02508
 
 0.16148
 
 Not queried
 
  2.3213780
 
 1.32e-01
 
 1.00e+00
  
 
  
 17
 
  58218643
 
  58218965
 
  PDF   STATS   ENSEMBL   UCSC 
 
  323
 
  4
 
 18585
 
 island
 
 0.06222
 
 0.41753
 
 Not queried
 
  2.3211776
 
 5.71e-02
 
 1.00e+00
  
 
  
 15
 
  90808551
 
  90808917
 
  PDF   STATS   ENSEMBL   UCSC 
 
  367
 
  8
 
 14423
 
 island
 
 0.06379
 
 0.03283
 
 Not queried
 
  2.3206538
 
 4.66e-03
 
 1.00e+00
  
 
  
 2
 
 177028606
 
 177028714
 
  PDF   STATS   ENSEMBL   UCSC 
 
  109
 
  4
 
 23704
 
 tss
 
 0.84796
 
 0.48438
 
 Not queried
 
  2.3195877
 
 2.86e-02
 
 1.00e+00
  
 
  
 3
 
  45635931
 
  45636225
 
  PDF   STATS   ENSEMBL   UCSC 
 
  295
 
  6
 
 28226
 
 tss
 
 0.08225
 
 0.28812
 
 Not queried
 
  2.3194467
 
 4.11e-02
 
 1.00e+00
  
 
  
 16
 
  59788951
 
  59789219
 
  PDF   STATS   ENSEMBL   UCSC 
 
  269
 
  4
 
 15951
 
 island
 
 0.64085
 
 0.89846
 
 Not queried
 
  2.3188617
 
 2.86e-02
 
 1.00e+00
  
 
  
 5
 
 140568075
 
 140568535
 
  PDF   STATS   ENSEMBL   UCSC 
 
  461
 
  4
 
 31757
 
 gene;island
 
 0.37680
 
 0.70948
 
 Not queried
 
  2.3175530
 
 2.86e-02
 
 1.00e+00
  
 
  
 5
 
   1876168
 
   1876397
 
  PDF   STATS   ENSEMBL   UCSC 
 
  230
 
  5
 
 32583
 
 island
 
 0.10880
 
 0.34070
 
 Not queried
 
  2.3163372
 
 9.52e-02
 
 1.00e+00
  
 
  
 8
 
   2003810
 
   2004050
 
  PDF   STATS   ENSEMBL   UCSC 
 
  241
 
  5
 
 39124
 
 gene;island
 
 0.58123
 
 0.84292
 
 Not queried
 
  2.3161941
 
 7.94e-03
 
 1.00e+00
  
 
  
 11
 
  65779192
 
  65779847
 
  PDF   STATS   ENSEMBL   UCSC 
 
  656
 
 10
 
 8473
 
 island
 
 0.35053
 
 0.73060
 
 Not queried
 
  2.3159831
 
 2.06e-04
 
 1.00e+00
  
 
  
 X
 
  55743906
 
  55744084
 
  PDF   STATS   ENSEMBL   UCSC 
 
  179
 
  6
 
 41343
 
 tss
 
 0.10504
 
 0.37811
 
 Not queried
 
  2.3153562
 
 4.33e-03
 
 1.00e+00
  
 
  
 11
 
  94771024
 
  94771514
 
  PDF   STATS   ENSEMBL   UCSC 
 
  491
 
  4
 
 8721
 
 island
 
 0.64871
 
 0.87882
 
 Not queried
 
  2.3147743
 
 1.14e-01
 
 1.00e+00
  
 
  
 22
 
  21321253
 
  21321434
 
  PDF   STATS   ENSEMBL   UCSC 
 
  182
 
  4
 
 26944
 
 tss
 
 0.52360
 
 0.14659
 
 Not queried
 
  2.3145961
 
 2.00e-01
 
 1.00e+00
  
 
  
 3
 
 193987426
 
 193987757
 
  PDF   STATS   ENSEMBL   UCSC 
 
  332
 
  4
 
 29741
 
 island
 
 0.57208
 
 0.92530
 
 Not queried
 
  2.3130072
 
 5.71e-02
 
 1.00e+00
  
 
  
 7
 
  95402189
 
  95402528
 
  PDF   STATS   ENSEMBL   UCSC 
 
  340
 
  4
 
 37036
 
 gene;island
 
 0.20245
 
 0.04235
 
 Not queried
 
  2.3129912
 
 2.00e-01
 
 1.00e+00
  
 
  
 19
 
  17322527
 
  17322956
 
  PDF   STATS   ENSEMBL   UCSC 
 
  430
 
  4
 
 19878
 
 gene;island
 
 0.84449
 
 0.95427
 
 Not queried
 
  2.3126331
 
 1.14e-01
 
 1.00e+00
  
 
  
 6
 
  33170692
 
  33170838
 
  PDF   STATS   ENSEMBL   UCSC 
 
  147
 
  6
 
 34159
 
 gene;island
 
 0.83350
 
 0.91065
 
 Not queried
 
  2.3118769
 
 9.31e-02
 
 1.00e+00
  
 
  
 1
 
 167090972
 
 167091161
 
  PDF   STATS   ENSEMBL   UCSC 
 
  190
 
  4
 
 871
 
 gene;island
 
 0.12267
 
 0.02392
 
 Not queried
 
  2.3115696
 
 2.86e-02
 
 1.00e+00
  
 
  
 X
 
  48957963
 
  48958509
 
  PDF   STATS   ENSEMBL   UCSC 
 
  547
 
 12
 
 41783
 
 island
 
 0.12243
 
 0.50297
 
 Not queried
 
  2.3113446
 
 2.01e-04
 
 1.00e+00
  
 
  
 X
 
  64754624
 
  64754995
 
  PDF   STATS   ENSEMBL   UCSC 
 
  372
 
 12
 
 41830
 
 island
 
 0.10025
 
 0.02767
 
 Not queried
 
  2.3106198
 
 2.01e-04
 
 1.00e+00
  
 
  
 1
 
 212688762
 
 212688997
 
  PDF   STATS   ENSEMBL   UCSC 
 
  236
 
  4
 
 4171
 
 island
 
 0.06362
 
 0.23001
 
 Not queried
 
  2.3104793
 
 2.86e-02
 
 1.00e+00
  
 
  
 6
 
  32710933
 
  32711102
 
  PDF   STATS   ENSEMBL   UCSC 
 
  170
 
  4
 
 34062
 
 gene
 
 0.77061
 
 0.47888
 
 Not queried
 
  2.3102393
 
 4.86e-01
 
 1.00e+00
  
 
  
 22
 
  42347906
 
  42348061
 
  PDF   STATS   ENSEMBL   UCSC 
 
  156
 
  4
 
 27142
 
 tss
 
 0.82505
 
 0.47989
 
 Not queried
 
  2.3097840
 
 5.71e-02
 
 1.00e+00
  
 
  
 10
 
  45495677
 
  45496237
 
  PDF   STATS   ENSEMBL   UCSC 
 
  561
 
  9
 
 5040
 
 tss
 
 0.58226
 
 0.07626
 
 Not queried
 
  2.3084844
 
 4.00e-02
 
 1.00e+00
  
 
  
 3
 
  39851641
 
  39851931
 
  PDF   STATS   ENSEMBL   UCSC 
 
  291
 
  4
 
 27684
 
 gene;island
 
 0.19488
 
 0.04159
 
 Not queried
 
  2.3082917
 
 3.43e-01
 
 1.00e+00
  
 
  
 17
 
   1961109
 
   1961510
 
  PDF   STATS   ENSEMBL   UCSC 
 
  402
 
  4
 
 16292
 
 gene;island
 
 0.68805
 
 0.91518
 
 Not queried
 
  2.3072845
 
 2.86e-02
 
 1.00e+00
  
 
  
 X
 
  48958082
 
  48958509
 
  PDF   STATS   ENSEMBL   UCSC 
 
  428
 
 10
 
 41308
 
 tss
 
 0.12243
 
 0.53604
 
 Not queried
 
  2.3057045
 
 7.25e-04
 
 1.00e+00
  
 
  
 5
 
    218073
 
    218200
 
  PDF   STATS   ENSEMBL   UCSC 
 
  128
 
  4
 
 31444
 
 gene;tss;island
 
 0.20286
 
 0.03626
 
 Not queried
 
  2.3052380
 
 4.86e-01
 
 1.00e+00
  
 
  
 8
 
 135490694
 
 135491240
 
  PDF   STATS   ENSEMBL   UCSC 
 
  547
 
  7
 
 39370
 
 gene;island
 
 0.35979
 
 0.77173
 
 Not queried
 
  2.3051657
 
 7.28e-02
 
 1.00e+00
  
 
  
 6
 
  31730260
 
  31730606
 
  PDF   STATS   ENSEMBL   UCSC 
 
  347
 
  4
 
 34773
 
 tss
 
 0.63422
 
 0.89095
 
 Not queried
 
  2.3048732
 
 1.14e-01
 
 1.00e+00
  
 
  
 19
 
  58095424
 
  58095659
 
  PDF   STATS   ENSEMBL   UCSC 
 
  236
 
  8
 
 22438
 
 island
 
 0.02483
 
 0.09818
 
 Not queried
 
  2.3047222
 
 1.04e-02
 
 1.00e+00
  
 
  
 14
 
  95826570
 
  95826997
 
  PDF   STATS   ENSEMBL   UCSC 
 
  428
 
  5
 
 13081
 
 island
 
 0.59440
 
 0.87900
 
 Not queried
 
  2.3044771
 
 7.94e-03
 
 1.00e+00
  
 
  
 4
 
   4405844
 
   4406200
 
  PDF   STATS   ENSEMBL   UCSC 
 
  357
 
  6
 
 29871
 
 gene
 
 0.60550
 
 0.85608
 
 Not queried
 
  2.3027147
 
 2.40e-01
 
 1.00e+00
  
 
  
 X
 
  48687169
 
  48687283
 
  PDF   STATS   ENSEMBL   UCSC 
 
  115
 
  4
 
 41770
 
 island
 
 0.72248
 
 0.95467
 
 Not queried
 
  2.3020477
 
 2.00e-01
 
 1.00e+00
  
 
  
 5
 
  43040885
 
  43041152
 
  PDF   STATS   ENSEMBL   UCSC 
 
  268
 
  4
 
 31984
 
 tss;island
 
 0.08885
 
 0.28041
 
 Not queried
 
  2.3006270
 
 5.71e-02
 
 1.00e+00
  
 
  
 1
 
  43533796
 
  43534324
 
  PDF   STATS   ENSEMBL   UCSC 
 
  529
 
  6
 
 3151
 
 island
 
 0.65155
 
 0.88311
 
 Not queried
 
  2.3000306
 
 4.33e-03
 
 1.00e+00
  
 
  
 15
 
  49338748
 
  49339070
 
  PDF   STATS   ENSEMBL   UCSC 
 
  323
 
  5
 
 13633
 
 tss
 
 0.05111
 
 0.20656
 
 Not queried
 
  2.2986833
 
 4.21e-01
 
 1.00e+00
  
 
  
 7
 
   1588284
 
   1588579
 
  PDF   STATS   ENSEMBL   UCSC 
 
  296
 
  4
 
 36698
 
 gene;island
 
 0.44130
 
 0.80898
 
 Not queried
 
  2.2979507
 
 2.86e-02
 
 1.00e+00
  
 
  
 4
 
  71587640
 
  71587904
 
  PDF   STATS   ENSEMBL   UCSC 
 
  265
 
  4
 
 29972
 
 gene
 
 0.76734
 
 0.51800
 
 Not queried
 
  2.2968016
 
 4.86e-01
 
 1.00e+00
  
 
  
 2
 
   7171869
 
   7172097
 
  PDF   STATS   ENSEMBL   UCSC 
 
  229
 
  4
 
 22522
 
 gene;island
 
 0.49116
 
 0.83898
 
 Not queried
 
  2.2967457
 
 2.00e-01
 
 1.00e+00
  
 
  
 8
 
  11203882
 
  11204132
 
  PDF   STATS   ENSEMBL   UCSC 
 
  251
 
  4
 
 39141
 
 gene;island
 
 0.64864
 
 0.27446
 
 Not queried
 
  2.2959193
 
 2.86e-02
 
 1.00e+00
  
 
  
 10
 
 105239085
 
 105239205
 
  PDF   STATS   ENSEMBL   UCSC 
 
  121
 
  4
 
 5322
 
 tss
 
 0.37911
 
 0.70572
 
 Not queried
 
  2.2951114
 
 2.86e-02
 
 1.00e+00
  
 
  
 19
 
  23299649
 
  23300243
 
  PDF   STATS   ENSEMBL   UCSC 
 
  595
 
  5
 
 21809
 
 island
 
 0.27471
 
 0.06751
 
 Not queried
 
  2.2947446
 
 9.52e-02
 
 1.00e+00
  
 
  
 10
 
   1415977
 
   1416224
 
  PDF   STATS   ENSEMBL   UCSC 
 
  248
 
  4
 
 4448
 
 gene;island
 
 0.27310
 
 0.67515
 
 Not queried
 
  2.2944811
 
 2.86e-02
 
 1.00e+00
  
 
  
 16
 
   2770211
 
   2770429
 
  PDF   STATS   ENSEMBL   UCSC 
 
  219
 
  4
 
 14582
 
 gene;island
 
 0.67877
 
 0.87503
 
 Not queried
 
  2.2940397
 
 2.00e-01
 
 1.00e+00
  
 
  
 15
 
  45406534
 
  45406939
 
  PDF   STATS   ENSEMBL   UCSC 
 
  406
 
  6
 
 13297
 
 gene
 
 0.22967
 
 0.56426
 
 Not queried
 
  2.2924772
 
 2.16e-03
 
 1.00e+00
  
 
  
 6
 
  40567317
 
  40567579
 
  PDF   STATS   ENSEMBL   UCSC 
 
  263
 
  4
 
 36052
 
 island
 
 0.03967
 
 0.32589
 
 Not queried
 
  2.2924336
 
 3.43e-01
 
 1.00e+00
  
 
  
 1
 
 173639044
 
 173639135
 
  PDF   STATS   ENSEMBL   UCSC 
 
   92
 
  4
 
 2093
 
 tss;island
 
 0.29149
 
 0.07286
 
 Not queried
 
  2.2920002
 
 1.14e-01
 
 1.00e+00
  
 
  
 13
 
 103053409
 
 103053761
 
  PDF   STATS   ENSEMBL   UCSC 
 
  353
 
  4
 
 11188
 
 gene;island
 
 0.15695
 
 0.35459
 
 Not queried
 
  2.2919878
 
 1.14e-01
 
 1.00e+00
  
 
  
 6
 
  31590513
 
  31590674
 
  PDF   STATS   ENSEMBL   UCSC 
 
  162
 
  9
 
 34758
 
 tss
 
 0.49484
 
 0.84920
 
 Not queried
 
  2.2916517
 
 4.00e-02
 
 1.00e+00
  
 
  
 17
 
  46804123
 
  46804414
 
  PDF   STATS   ENSEMBL   UCSC 
 
  292
 
  4
 
 16630
 
 gene;island
 
 0.84207
 
 0.55671
 
 Not queried
 
  2.2903442
 
 1.14e-01
 
 1.00e+00
  
 
  
 17
 
  12568499
 
  12568761
 
  PDF   STATS   ENSEMBL   UCSC 
 
  263
 
  4
 
 17107
 
 tss;island
 
 0.01294
 
 0.08486
 
 Not queried
 
  2.2883841
 
 5.71e-02
 
 1.00e+00
  
 
  
 9
 
  36258825
 
  36258971
 
  PDF   STATS   ENSEMBL   UCSC 
 
  147
 
  4
 
 40700
 
 gene;tss;island
 
 0.84993
 
 0.53768
 
 Not queried
 
  2.2878457
 
 2.86e-02
 
 1.00e+00
  
 
  
 19
 
  34263905
 
  34264111
 
  PDF   STATS   ENSEMBL   UCSC 
 
  207
 
  4
 
 19940
 
 gene;island
 
 0.61046
 
 0.83829
 
 Not queried
 
  2.2868906
 
 2.86e-02
 
 1.00e+00
  
 
  
 6
 
  33169581
 
  33169731
 
  PDF   STATS   ENSEMBL   UCSC 
 
  151
 
  4
 
 34157
 
 gene;tss;island
 
 0.48513
 
 0.78851
 
 Not queried
 
  2.2868218
 
 1.14e-01
 
 1.00e+00
  
 
  
 4
 
 174452835
 
 174453287
 
  PDF   STATS   ENSEMBL   UCSC 
 
  453
 
  5
 
 30134
 
 gene;island
 
 0.75815
 
 0.44115
 
 Not queried
 
  2.2855712
 
 7.94e-03
 
 1.00e+00
  
 
  
 16
 
  28075183
 
  28075295
 
  PDF   STATS   ENSEMBL   UCSC 
 
  113
 
  4
 
 15122
 
 tss;island
 
 0.53496
 
 0.18045
 
 Not queried
 
  2.2825057
 
 2.00e-01
 
 1.00e+00
  
 
  
 6
 
  34984836
 
  34984953
 
  PDF   STATS   ENSEMBL   UCSC 
 
  118
 
  4
 
 34243
 
 gene
 
 0.78454
 
 0.91205
 
 Not queried
 
  2.2819563
 
 1.14e-01
 
 1.00e+00
  
 
  
 10
 
   1401818
 
   1402293
 
  PDF   STATS   ENSEMBL   UCSC 
 
  476
 
  5
 
 4447
 
 gene;island
 
 0.56514
 
 0.86329
 
 Not queried
 
  2.2806558
 
 1.51e-01
 
 1.00e+00
  
 
  
 X
 
  34960814
 
  34960893
 
  PDF   STATS   ENSEMBL   UCSC 
 
   80
 
  5
 
 41250
 
 tss
 
 0.62988
 
 0.91450
 
 Not queried
 
  2.2791766
 
 7.94e-03
 
 1.00e+00
  
 
  
 3
 
  10547179
 
  10547584
 
  PDF   STATS   ENSEMBL   UCSC 
 
  406
 
  8
 
 28835
 
 island
 
 0.19992
 
 0.60576
 
 Not queried
 
  2.2786657
 
 1.04e-02
 
 1.00e+00
  
 
  
 19
 
  38182773
 
  38183210
 
  PDF   STATS   ENSEMBL   UCSC 
 
  438
 
  5
 
 19974
 
 gene
 
 0.13469
 
 0.04240
 
 Not queried
 
  2.2766135
 
 7.94e-03
 
 1.00e+00
  
 
  
 6
 
  30135449
 
  30135671
 
  PDF   STATS   ENSEMBL   UCSC 
 
  223
 
  4
 
 33674
 
 gene
 
 0.23924
 
 0.59678
 
 Not queried
 
  2.2759402
 
 2.86e-02
 
 1.00e+00
  
 
  
 16
 
  30023400
 
  30023780
 
  PDF   STATS   ENSEMBL   UCSC 
 
  381
 
  4
 
 15145
 
 tss;island
 
 0.85412
 
 0.55303
 
 Not queried
 
  2.2750917
 
 2.86e-02
 
 1.00e+00
  
 
  
 16
 
  89070647
 
  89070903
 
  PDF   STATS   ENSEMBL   UCSC 
 
  257
 
  4
 
 16199
 
 island
 
 0.55729
 
 0.84316
 
 Not queried
 
  2.2747390
 
 2.86e-02
 
 1.00e+00
  
 
  
 7
 
  50849639
 
  50849931
 
  PDF   STATS   ENSEMBL   UCSC 
 
  293
 
  4
 
 36952
 
 gene;island
 
 0.25460
 
 0.11030
 
 Not queried
 
  2.2742279
 
 3.43e-01
 
 1.00e+00
  
 
  
 2
 
 201980838
 
 201980910
 
  PDF   STATS   ENSEMBL   UCSC 
 
   73
 
  4
 
 22948
 
 gene
 
 0.08860
 
 0.01798
 
 Not queried
 
  2.2738194
 
 2.86e-02
 
 1.00e+00
  
 
  
 15
 
  98196022
 
  98196333
 
  PDF   STATS   ENSEMBL   UCSC 
 
  312
 
  5
 
 14464
 
 island
 
 0.04600
 
 0.31301
 
 Not queried
 
  2.2734794
 
 9.52e-02
 
 1.00e+00
  
 
  
 7
 
 157179773
 
 157180124
 
  PDF   STATS   ENSEMBL   UCSC 
 
  352
 
  6
 
 37257
 
 gene
 
 0.60786
 
 0.88057
 
 Not queried
 
  2.2722320
 
 3.94e-01
 
 1.00e+00
  
 
  
 3
 
  46759335
 
  46759698
 
  PDF   STATS   ENSEMBL   UCSC 
 
  364
 
  8
 
 29016
 
 island
 
 0.53013
 
 0.87586
 
 Not queried
 
  2.2701852
 
 4.99e-02
 
 1.00e+00
  
 
  
 6
 
  32809960
 
  32810258
 
  PDF   STATS   ENSEMBL   UCSC 
 
  299
 
  5
 
 34084
 
 gene;island
 
 0.91004
 
 0.66207
 
 Not queried
 
  2.2701084
 
 9.52e-02
 
 1.00e+00
  
 
  
 X
 
  69509542
 
  69509790
 
  PDF   STATS   ENSEMBL   UCSC 
 
  249
 
  7
 
 41015
 
 gene
 
 0.09616
 
 0.02773
 
 Not queried
 
  2.2699478
 
 2.62e-02
 
 1.00e+00
  
 
  
 19
 
  54177144
 
  54177261
 
  PDF   STATS   ENSEMBL   UCSC 
 
  118
 
  4
 
 20998
 
 tss
 
 0.62871
 
 0.89993
 
 Not queried
 
  2.2687750
 
 2.86e-02
 
 1.00e+00
  
 
  
 6
 
  33158503
 
  33158597
 
  PDF   STATS   ENSEMBL   UCSC 
 
   95
 
  4
 
 34149
 
 gene;island
 
 0.90333
 
 0.57946
 
 Not queried
 
  2.2679958
 
 2.00e-01
 
 1.00e+00
  
 
  
 19
 
  15122135
 
  15122224
 
  PDF   STATS   ENSEMBL   UCSC 
 
   90
 
  4
 
 19862
 
 gene;island
 
 0.12418
 
 0.40644
 
 Not queried
 
  2.2675204
 
 5.71e-02
 
 1.00e+00
  
 
  
 1
 
  89664260
 
  89664582
 
  PDF   STATS   ENSEMBL   UCSC 
 
  323
 
  6
 
 618
 
 gene
 
 0.05962
 
 0.23833
 
 Not queried
 
  2.2670779
 
 2.16e-03
 
 1.00e+00
  
 
  
 3
 
  36986555
 
  36987040
 
  PDF   STATS   ENSEMBL   UCSC 
 
  486
 
  8
 
 28177
 
 tss
 
 0.44102
 
 0.21763
 
 Not queried
 
  2.2669368
 
 1.61e-01
 
 1.00e+00
  
 
  
 10
 
 102508286
 
 102508633
 
  PDF   STATS   ENSEMBL   UCSC 
 
  348
 
  5
 
 4701
 
 gene;island
 
 0.03527
 
 0.10081
 
 Not queried
 
  2.2668566
 
 9.52e-02
 
 1.00e+00
  
 
  
 18
 
  61144150
 
  61144339
 
  PDF   STATS   ENSEMBL   UCSC 
 
  190
 
  4
 
 19088
 
 gene
 
 0.42986
 
 0.79913
 
 Not queried
 
  2.2649292
 
 5.71e-02
 
 1.00e+00
  
 
  
 X
 
  69674800
 
  69675064
 
  PDF   STATS   ENSEMBL   UCSC 
 
  265
 
  4
 
 41018
 
 gene;island
 
 0.06278
 
 0.02370
 
 Not queried
 
  2.2640433
 
 3.43e-01
 
 1.00e+00
  
 
  
 16
 
  89632574
 
  89632749
 
  PDF   STATS   ENSEMBL   UCSC 
 
  176
 
  5
 
 16228
 
 island
 
 0.61335
 
 0.94758
 
 Not queried
 
  2.2637516
 
 3.17e-02
 
 1.00e+00
  
 
  
 19
 
  11353961
 
  11354401
 
  PDF   STATS   ENSEMBL   UCSC 
 
  441
 
  5
 
 19796
 
 gene;island
 
 0.16813
 
 0.12168
 
 Not queried
 
  2.2634783
 
 4.21e-01
 
 1.00e+00
  
 
  
 16
 
  51187388
 
  51187807
 
  PDF   STATS   ENSEMBL   UCSC 
 
  420
 
  5
 
 15888
 
 island
 
 0.12005
 
 0.03091
 
 Not queried
 
  2.2631732
 
 7.94e-03
 
 1.00e+00
  
 
  
 15
 
  75628126
 
  75628370
 
  PDF   STATS   ENSEMBL   UCSC 
 
  245
 
  6
 
 13782
 
 tss
 
 0.05244
 
 0.26017
 
 Not queried
 
  2.2628797
 
 1.32e-01
 
 1.00e+00
  
 
  
 12
 
  54446253
 
  54446576
 
  PDF   STATS   ENSEMBL   UCSC 
 
  324
 
  7
 
 9731
 
 tss
 
 0.78008
 
 0.41123
 
 Not queried
 
  2.2626526
 
 5.30e-02
 
 1.00e+00
  
 
  
 10
 
  32734661
 
  32734958
 
  PDF   STATS   ENSEMBL   UCSC 
 
  298
 
  7
 
 5005
 
 tss
 
 0.06221
 
 0.02296
 
 Not queried
 
  2.2618641
 
 5.30e-02
 
 1.00e+00
  
 
  
 6
 
  28972876
 
  28972975
 
  PDF   STATS   ENSEMBL   UCSC 
 
  100
 
  5
 
 33586
 
 gene
 
 0.85848
 
 0.27809
 
 Not queried
 
  2.2616567
 
 5.56e-02
 
 1.00e+00
  
 
  
 10
 
  23463015
 
  23463377
 
  PDF   STATS   ENSEMBL   UCSC 
 
  363
 
  4
 
 5590
 
 island
 
 0.02473
 
 0.10453
 
 Not queried
 
  2.2603471
 
 2.86e-02
 
 1.00e+00
  
 
  
 16
 
   1128689
 
   1128865
 
  PDF   STATS   ENSEMBL   UCSC 
 
  177
 
  5
 
 14956
 
 tss;island
 
 0.50340
 
 0.82924
 
 Not queried
 
  2.2602159
 
 7.94e-03
 
 1.00e+00
  
 
  
 1
 
 229569608
 
 229570288
 
  PDF   STATS   ENSEMBL   UCSC 
 
  681
 
 14
 
 4297
 
 island
 
 0.15068
 
 0.46250
 
 Not queried
 
  2.2590120
 
 1.73e-03
 
 1.00e+00
  
 
  
 18
 
  77203371
 
  77203667
 
  PDF   STATS   ENSEMBL   UCSC 
 
  297
 
  4
 
 19106
 
 gene;island
 
 0.44896
 
 0.80766
 
 Not queried
 
  2.2570266
 
 2.86e-02
 
 1.00e+00
  
 
  
 17
 
  40337534
 
  40337892
 
  PDF   STATS   ENSEMBL   UCSC 
 
  359
 
  6
 
 17324
 
 tss
 
 0.79698
 
 0.45053
 
 Not queried
 
  2.2565101
 
 2.16e-03
 
 1.00e+00
  
 
  
 4
 
  57547347
 
  57547699
 
  PDF   STATS   ENSEMBL   UCSC 
 
  353
 
  4
 
 29966
 
 gene
 
 0.22367
 
 0.51901
 
 Not queried
 
  2.2557342
 
 2.86e-02
 
 1.00e+00
  
 
  
 4
 
  46391159
 
  46391929
 
  PDF   STATS   ENSEMBL   UCSC 
 
  771
 
 14
 
 29950
 
 gene;island
 
 0.03489
 
 0.08291
 
 Not queried
 
  2.2553467
 
 5.76e-03
 
 1.00e+00
  
 
  
 14
 
  64319089
 
  64319306
 
  PDF   STATS   ENSEMBL   UCSC 
 
  218
 
  4
 
 12424
 
 tss;island
 
 0.38284
 
 0.14012
 
 Not queried
 
  2.2545583
 
 4.86e-01
 
 1.00e+00
  
 
  
 5
 
    523430
 
    523794
 
  PDF   STATS   ENSEMBL   UCSC 
 
  365
 
  4
 
 31457
 
 gene;island
 
 0.33845
 
 0.72036
 
 Not queried
 
  2.2539664
 
 2.86e-02
 
 1.00e+00
  
 
  
 19
 
  52872916
 
  52873125
 
  PDF   STATS   ENSEMBL   UCSC 
 
  210
 
  6
 
 20976
 
 tss
 
 0.04466
 
 0.18560
 
 Not queried
 
  2.2531701
 
 2.60e-02
 
 1.00e+00
  
 
  
 16
 
  64984626
 
  64984977
 
  PDF   STATS   ENSEMBL   UCSC 
 
  352
 
  5
 
 14747
 
 gene;island
 
 0.61315
 
 0.87684
 
 Not queried
 
  2.2526562
 
 9.52e-02
 
 1.00e+00
  
 
  
 20
 
  44098223
 
  44098387
 
  PDF   STATS   ENSEMBL   UCSC 
 
  165
 
  6
 
 25737
 
 tss
 
 0.22702
 
 0.58016
 
 Not queried
 
  2.2525774
 
 2.16e-03
 
 1.00e+00
  
 
  
 11
 
  62647979
 
  62648405
 
  PDF   STATS   ENSEMBL   UCSC 
 
  427
 
  7
 
 6634
 
 gene;island
 
 0.02326
 
 0.14199
 
 Not queried
 
  2.2512852
 
 4.08e-03
 
 1.00e+00
  
 
  
 12
 
 124990897
 
 124991139
 
  PDF   STATS   ENSEMBL   UCSC 
 
  243
 
  4
 
 9373
 
 gene
 
 0.69675
 
 0.91881
 
 Not queried
 
  2.2496996
 
 2.86e-02
 
 1.00e+00
  
 
  
 7
 
 143582146
 
 143582630
 
  PDF   STATS   ENSEMBL   UCSC 
 
  485
 
  4
 
 37193
 
 gene;island
 
 0.06857
 
 0.25214
 
 Not queried
 
  2.2494591
 
 2.86e-02
 
 1.00e+00
  
 
  
 8
 
    599963
 
    600555
 
  PDF   STATS   ENSEMBL   UCSC 
 
  593
 
  6
 
 39939
 
 island
 
 0.62697
 
 0.87572
 
 Not queried
 
  2.2481624
 
 4.33e-03
 
 1.00e+00
  
 
  
 19
 
  49249932
 
  49250561
 
  PDF   STATS   ENSEMBL   UCSC 
 
  630
 
  7
 
 22198
 
 island
 
 0.15454
 
 0.03706
 
 Not queried
 
  2.2477981
 
 1.75e-02
 
 1.00e+00
  
 
  
 17
 
  43047405
 
  43047971
 
  PDF   STATS   ENSEMBL   UCSC 
 
  567
 
  5
 
 18384
 
 island
 
 0.43678
 
 0.06712
 
 Not queried
 
  2.2476160
 
 4.21e-01
 
 1.00e+00
  
 
  
 17
 
  42247565
 
  42248062
 
  PDF   STATS   ENSEMBL   UCSC 
 
  498
 
  6
 
 17370
 
 tss
 
 0.42471
 
 0.75505
 
 Not queried
 
  2.2466834
 
 1.32e-01
 
 1.00e+00
  
 
  
 16
 
  31053788
 
  31054123
 
  PDF   STATS   ENSEMBL   UCSC 
 
  336
 
  4
 
 15831
 
 island
 
 0.39965
 
 0.73369
 
 Not queried
 
  2.2457236
 
 2.86e-02
 
 1.00e+00
  
 
  
 4
 
  80329388
 
  80329633
 
  PDF   STATS   ENSEMBL   UCSC 
 
  246
 
  8
 
 30410
 
 tss
 
 0.55308
 
 0.85975
 
 Not queried
 
  2.2455458
 
 2.81e-02
 
 1.00e+00
  
 
  
 6
 
  33039396
 
  33039804
 
  PDF   STATS   ENSEMBL   UCSC 
 
  409
 
  7
 
 34117
 
 gene
 
 0.89351
 
 0.56630
 
 Not queried
 
  2.2455366
 
 3.79e-02
 
 1.00e+00
  
 
  
 17
 
  37879654
 
  37879858
 
  PDF   STATS   ENSEMBL   UCSC 
 
  205
 
  4
 
 16519
 
 gene;island
 
 0.66219
 
 0.89829
 
 Not queried
 
  2.2454038
 
 2.86e-02
 
 1.00e+00
  
 
  
 22
 
  21321253
 
  21321585
 
  PDF   STATS   ENSEMBL   UCSC 
 
  333
 
  5
 
 26761
 
 gene;island
 
 0.62558
 
 0.17075
 
 Not queried
 
  2.2435559
 
 1.51e-01
 
 1.00e+00
  
 
  
 4
 
   7194841
 
   7195206
 
  PDF   STATS   ENSEMBL   UCSC 
 
  366
 
  5
 
 29890
 
 gene;island
 
 0.11016
 
 0.00867
 
 Not queried
 
  2.2430024
 
 1.51e-01
 
 1.00e+00
  
 
  
 18
 
    712731
 
    712795
 
  PDF   STATS   ENSEMBL   UCSC 
 
   65
 
  4
 
 19122
 
 tss;island
 
 0.01688
 
 0.06855
 
 Not queried
 
  2.2423443
 
 1.14e-01
 
 1.00e+00
  
 
  
 X
 
  51636562
 
  51636681
 
  PDF   STATS   ENSEMBL   UCSC 
 
  120
 
  6
 
 41325
 
 tss
 
 0.10082
 
 0.02292
 
 Not queried
 
  2.2412906
 
 1.32e-01
 
 1.00e+00
  
 
  
 2
 
  66810341
 
  66810606
 
  PDF   STATS   ENSEMBL   UCSC 
 
  266
 
  4
 
 24321
 
 island
 
 0.21780
 
 0.50103
 
 Not queried
 
  2.2410680
 
 2.86e-02
 
 1.00e+00
  
 
  
 7
 
   1277521
 
   1277790
 
  PDF   STATS   ENSEMBL   UCSC 
 
  270
 
  4
 
 38028
 
 island
 
 0.12351
 
 0.31936
 
 Not queried
 
  2.2407508
 
 2.00e-01
 
 1.00e+00
  
 
  
 3
 
  42543933
 
  42544131
 
  PDF   STATS   ENSEMBL   UCSC 
 
  199
 
  7
 
 28970
 
 island
 
 0.14458
 
 0.02720
 
 Not queried
 
  2.2404238
 
 6.99e-03
 
 1.00e+00
  
 
  
 5
 
  76115672
 
  76116169
 
  PDF   STATS   ENSEMBL   UCSC 
 
  498
 
  6
 
 31595
 
 gene;island
 
 0.01159
 
 0.08054
 
 Not queried
 
  2.2401444
 
 8.66e-03
 
 1.00e+00
  
 
  
 10
 
 100993553
 
 100994143
 
  PDF   STATS   ENSEMBL   UCSC 
 
  591
 
 11
 
 4692
 
 gene;island
 
 0.12971
 
 0.03796
 
 Not queried
 
  2.2398822
 
 5.24e-03
 
 1.00e+00
  
 
  
 7
 
    936081
 
    936591
 
  PDF   STATS   ENSEMBL   UCSC 
 
  511
 
  5
 
 37994
 
 island
 
 0.83141
 
 0.94868
 
 Not queried
 
  2.2394445
 
 3.17e-02
 
 1.00e+00
  
 
  
 13
 
  52419332
 
  52419483
 
  PDF   STATS   ENSEMBL   UCSC 
 
  152
 
  5
 
 11426
 
 tss
 
 0.02801
 
 0.13878
 
 Not queried
 
  2.2392322
 
 7.94e-03
 
 1.00e+00
  
 
  
 1
 
 153940090
 
 153940616
 
  PDF   STATS   ENSEMBL   UCSC 
 
  527
 
  7
 
 1954
 
 tss;island
 
 0.15295
 
 0.03474
 
 Not queried
 
  2.2390556
 
 1.11e-02
 
 1.00e+00
  
 
  
 3
 
 181413810
 
 181414237
 
  PDF   STATS   ENSEMBL   UCSC 
 
  428
 
  6
 
 27997
 
 gene;island
 
 0.50439
 
 0.84800
 
 Not queried
 
  2.2375658
 
 4.33e-03
 
 1.00e+00
  
 
  
 8
 
   9008170
 
   9008681
 
  PDF   STATS   ENSEMBL   UCSC 
 
  512
 
  7
 
 39997
 
 island
 
 0.08818
 
 0.04518
 
 Not queried
 
  2.2374209
 
 7.28e-02
 
 1.00e+00
  
 
  
 1
 
 203456691
 
 203457150
 
  PDF   STATS   ENSEMBL   UCSC 
 
  460
 
  6
 
 949
 
 gene;island
 
 0.02077
 
 0.08249
 
 Not queried
 
  2.2371445
 
 4.11e-02
 
 1.00e+00
  
 
  
 2
 
 132056834
 
 132057166
 
  PDF   STATS   ENSEMBL   UCSC 
 
  333
 
  4
 
 22833
 
 gene;island
 
 0.50230
 
 0.86142
 
 Not queried
 
  2.2364168
 
 2.86e-02
 
 1.00e+00
  
 
  
 20
 
  57607335
 
  57607420
 
  PDF   STATS   ENSEMBL   UCSC 
 
   86
 
  4
 
 25442
 
 gene
 
 0.03997
 
 0.17483
 
 Not queried
 
  2.2354418
 
 2.00e-01
 
 1.00e+00
  
 
  
 19
 
  58629828
 
  58630612
 
  PDF   STATS   ENSEMBL   UCSC 
 
  785
 
  9
 
 21097
 
 tss
 
 0.47268
 
 0.70367
 
 Not queried
 
  2.2346241
 
 1.36e-01
 
 1.00e+00
  
 
  
 17
 
  26732120
 
  26732374
 
  PDF   STATS   ENSEMBL   UCSC 
 
  255
 
  5
 
 16460
 
 gene;island
 
 0.63900
 
 0.48040
 
 Not queried
 
  2.2342536
 
 1.59e-02
 
 1.00e+00
  
 
  
 19
 
  13215041
 
  13215387
 
  PDF   STATS   ENSEMBL   UCSC 
 
  347
 
  5
 
 21608
 
 island
 
 0.63476
 
 0.86848
 
 Not queried
 
  2.2342378
 
 2.22e-01
 
 1.00e+00
  
 
  
 11
 
   8954570
 
   8954606
 
  PDF   STATS   ENSEMBL   UCSC 
 
   37
 
  4
 
 7110
 
 tss
 
 0.47881
 
 0.79963
 
 Not queried
 
  2.2324391
 
 2.86e-02
 
 1.00e+00
  
 
  
 17
 
  46507549
 
  46507895
 
  PDF   STATS   ENSEMBL   UCSC 
 
  347
 
  8
 
 18438
 
 island
 
 0.28458
 
 0.62651
 
 Not queried
 
  2.2315312
 
 2.07e-02
 
 1.00e+00
  
 
  
 11
 
 134126284
 
 134126504
 
  PDF   STATS   ENSEMBL   UCSC 
 
  221
 
  4
 
 6973
 
 gene;island
 
 0.75846
 
 0.88802
 
 Not queried
 
  2.2299691
 
 2.00e-01
 
 1.00e+00
  
 
  
 11
 
 128562003
 
 128562408
 
  PDF   STATS   ENSEMBL   UCSC 
 
  406
 
  7
 
 7780
 
 tss;island
 
 0.20413
 
 0.05251
 
 Not queried
 
  2.2285826
 
 2.33e-03
 
 1.00e+00
  
 
  
 2
 
   1595734
 
   1596054
 
  PDF   STATS   ENSEMBL   UCSC 
 
  321
 
  4
 
 23978
 
 island
 
 0.25805
 
 0.60592
 
 Not queried
 
  2.2275038
 
 5.71e-02
 
 1.00e+00
  
 
  
 X
 
  17879602
 
  17879951
 
  PDF   STATS   ENSEMBL   UCSC 
 
  350
 
  5
 
 41211
 
 tss
 
 0.07303
 
 0.06498
 
 Not queried
 
  2.2269406
 
 2.22e-01
 
 1.00e+00
  
 
  
 1
 
  20446641
 
  20446899
 
  PDF   STATS   ENSEMBL   UCSC 
 
  259
 
  4
 
 1308
 
 tss
 
 0.94336
 
 0.75716
 
 Not queried
 
  2.2266587
 
 1.14e-01
 
 1.00e+00
  
 
  
 11
 
  96076205
 
  96076302
 
  PDF   STATS   ENSEMBL   UCSC 
 
   98
 
  4
 
 6854
 
 gene
 
 0.12337
 
 0.13503
 
 Not queried
 
  2.2260865
 
 6.86e-01
 
 1.00e+00
  
 
  
 18
 
  44237002
 
  44237151
 
  PDF   STATS   ENSEMBL   UCSC 
 
  150
 
  6
 
 19225
 
 tss
 
 0.59754
 
 0.87317
 
 Not queried
 
  2.2247808
 
 2.60e-02
 
 1.00e+00
  
 
  
 5
 
 140588139
 
 140588424
 
  PDF   STATS   ENSEMBL   UCSC 
 
  286
 
  5
 
 33162
 
 island
 
 0.07307
 
 0.32109
 
 Not queried
 
  2.2240042
 
 5.56e-02
 
 1.00e+00
  
 
  
 X
 
  30265129
 
  30265251
 
  PDF   STATS   ENSEMBL   UCSC 
 
  123
 
  6
 
 41243
 
 tss
 
 0.76653
 
 0.93398
 
 Not queried
 
  2.2238688
 
 8.66e-03
 
 1.00e+00
  
 
  
 22
 
  49717382
 
  49717821
 
  PDF   STATS   ENSEMBL   UCSC 
 
  440
 
  5
 
 27562
 
 island
 
 0.46099
 
 0.60638
 
 Not queried
 
  2.2238659
 
 5.56e-02
 
 1.00e+00
  
 
  
 5
 
  78365647
 
  78365801
 
  PDF   STATS   ENSEMBL   UCSC 
 
  155
 
  5
 
 31603
 
 gene;tss;island
 
 0.69363
 
 0.93491
 
 Not queried
 
  2.2233523
 
 7.94e-03
 
 1.00e+00
  
 
  
 5
 
 140165938
 
 140166213
 
  PDF   STATS   ENSEMBL   UCSC 
 
  276
 
  4
 
 31736
 
 gene;island
 
 0.55874
 
 0.85529
 
 Not queried
 
  2.2221768
 
 5.71e-02
 
 1.00e+00
  
 
  
 5
 
 131892956
 
 131893381
 
  PDF   STATS   ENSEMBL   UCSC 
 
  426
 
  5
 
 31689
 
 gene;island
 
 0.23557
 
 0.64042
 
 Not queried
 
  2.2203637
 
 3.17e-02
 
 1.00e+00
  
 
  
 15
 
  40697419
 
  40698044
 
  PDF   STATS   ENSEMBL   UCSC 
 
  626
 
  8
 
 14009
 
 island
 
 0.06271
 
 0.11847
 
 Not queried
 
  2.2201470
 
 1.95e-01
 
 1.00e+00
  
 
  
 19
 
  15580310
 
  15580445
 
  PDF   STATS   ENSEMBL   UCSC 
 
  136
 
  4
 
 19872
 
 gene;island
 
 0.48001
 
 0.10240
 
 Not queried
 
  2.2196424
 
 2.00e-01
 
 1.00e+00
  
 
  
 8
 
  11565277
 
  11565725
 
  PDF   STATS   ENSEMBL   UCSC 
 
  449
 
  4
 
 39142
 
 gene;island
 
 0.52202
 
 0.18935
 
 Not queried
 
  2.2186197
 
 2.86e-02
 
 1.00e+00
  
 
  
 17
 
  80291982
 
  80292252
 
  PDF   STATS   ENSEMBL   UCSC 
 
  271
 
  4
 
 17740
 
 tss;island
 
 0.64342
 
 0.89120
 
 Not queried
 
  2.2183066
 
 3.43e-01
 
 1.00e+00
  
 
  
 20
 
  39321346
 
  39321729
 
  PDF   STATS   ENSEMBL   UCSC 
 
  384
 
  4
 
 26124
 
 island
 
 0.25571
 
 0.51906
 
 Not queried
 
  2.2172066
 
 2.86e-02
 
 1.00e+00
  
 
  
 22
 
  19137874
 
  19138267
 
  PDF   STATS   ENSEMBL   UCSC 
 
  394
 
  5
 
 26914
 
 tss;island
 
 0.78066
 
 0.35982
 
 Not queried
 
  2.2161889
 
 3.10e-01
 
 1.00e+00
  
 
  
 14
 
  36983404
 
  36983949
 
  PDF   STATS   ENSEMBL   UCSC 
 
  546
 
  7
 
 12329
 
 tss;island
 
 0.89401
 
 0.51136
 
 Not queried
 
  2.2160875
 
 6.99e-03
 
 1.00e+00
  
 
  
 20
 
  62693943
 
  62694005
 
  PDF   STATS   ENSEMBL   UCSC 
 
   63
 
  6
 
 25478
 
 gene;tss;island
 
 0.52172
 
 0.19354
 
 Not queried
 
  2.2160802
 
 2.60e-02
 
 1.00e+00
  
 
  
 15
 
  63570099
 
  63570580
 
  PDF   STATS   ENSEMBL   UCSC 
 
  482
 
  5
 
 13345
 
 gene;island
 
 0.01880
 
 0.21883
 
 Not queried
 
  2.2144026
 
 1.51e-01
 
 1.00e+00
  
 
  
 10
 
  71562314
 
  71562858
 
  PDF   STATS   ENSEMBL   UCSC 
 
  545
 
  4
 
 4602
 
 gene;island
 
 0.23720
 
 0.06755
 
 Not queried
 
  2.2139171
 
 3.43e-01
 
 1.00e+00
  
 
  
 20
 
  62688573
 
  62688896
 
  PDF   STATS   ENSEMBL   UCSC 
 
  324
 
  4
 
 25477
 
 gene;island
 
 0.52800
 
 0.80970
 
 Not queried
 
  2.2130278
 
 2.86e-02
 
 1.00e+00
  
 
  
 19
 
  35800589
 
  35801014
 
  PDF   STATS   ENSEMBL   UCSC 
 
  426
 
  4
 
 19948
 
 gene;island
 
 0.41175
 
 0.79196
 
 Not queried
 
  2.2113617
 
 2.00e-01
 
 1.00e+00
  
 
  
 3
 
  49203799
 
  49203975
 
  PDF   STATS   ENSEMBL   UCSC 
 
  177
 
  5
 
 28280
 
 tss
 
 0.70863
 
 0.08252
 
 Not queried
 
  2.2108547
 
 2.22e-01
 
 1.00e+00
  
 
  
 6
 
  13014871
 
  13015221
 
  PDF   STATS   ENSEMBL   UCSC 
 
  351
 
  5
 
 33510
 
 gene;island
 
 0.02725
 
 0.10384
 
 Not queried
 
  2.2088245
 
 1.51e-01
 
 1.00e+00
  
 
  
 17
 
  73840822
 
  73840989
 
  PDF   STATS   ENSEMBL   UCSC 
 
  168
 
  4
 
 17630
 
 tss
 
 0.33729
 
 0.62514
 
 Not queried
 
  2.2084917
 
 2.86e-02
 
 1.00e+00
  
 
  
 19
 
    496239
 
    496428
 
  PDF   STATS   ENSEMBL   UCSC 
 
  190
 
  5
 
 20236
 
 tss
 
 0.84973
 
 0.46622
 
 Not queried
 
  2.2075944
 
 9.52e-02
 
 1.00e+00
  
 
  
 11
 
  44972672
 
  44973218
 
  PDF   STATS   ENSEMBL   UCSC 
 
  547
 
 10
 
 7242
 
 tss
 
 0.63934
 
 0.44278
 
 Not queried
 
  2.2074972
 
 2.32e-02
 
 1.00e+00
  
 
  
 5
 
    501863
 
    502074
 
  PDF   STATS   ENSEMBL   UCSC 
 
  212
 
  4
 
 31456
 
 gene;island
 
 0.53641
 
 0.90052
 
 Not queried
 
  2.2070997
 
 5.71e-02
 
 1.00e+00
  
 
  
 12
 
  54357408
 
  54357771
 
  PDF   STATS   ENSEMBL   UCSC 
 
  364
 
  5
 
 9130
 
 gene;island
 
 0.72235
 
 0.23045
 
 Not queried
 
  2.2067234
 
 3.17e-02
 
 1.00e+00
  
 
  
 X
 
  48432562
 
  48433316
 
  PDF   STATS   ENSEMBL   UCSC 
 
  755
 
  9
 
 41765
 
 island
 
 0.20250
 
 0.56956
 
 Not queried
 
  2.2058463
 
 7.77e-03
 
 1.00e+00
  
 
  
 11
 
  93822022
 
  93822294
 
  PDF   STATS   ENSEMBL   UCSC 
 
  273
 
  4
 
 6844
 
 gene;island
 
 0.46343
 
 0.82847
 
 Not queried
 
  2.2052761
 
 2.86e-02
 
 1.00e+00
  
 
  
 16
 
  20084851
 
  20085358
 
  PDF   STATS   ENSEMBL   UCSC 
 
  508
 
  5
 
 15711
 
 island
 
 0.06218
 
 0.04741
 
 Not queried
 
  2.2046748
 
 3.10e-01
 
 1.00e+00
  
 
  
 3
 
  50341009
 
  50341293
 
  PDF   STATS   ENSEMBL   UCSC 
 
  285
 
  4
 
 27759
 
 gene;tss
 
 0.88357
 
 0.61706
 
 Not queried
 
  2.2045691
 
 3.43e-01
 
 1.00e+00
  
 
  
 10
 
  26726877
 
  26727318
 
  PDF   STATS   ENSEMBL   UCSC 
 
  442
 
  8
 
 5601
 
 island
 
 0.18275
 
 0.53520
 
 Not queried
 
  2.2045201
 
 1.30e-01
 
 1.00e+00
  
 
  
 4
 
  89299733
 
  89300321
 
  PDF   STATS   ENSEMBL   UCSC 
 
  589
 
  7
 
 31120
 
 island
 
 0.26394
 
 0.04491
 
 Not queried
 
  2.2043686
 
 3.18e-01
 
 1.00e+00
  
 
  
 X
 
  74493592
 
  74493847
 
  PDF   STATS   ENSEMBL   UCSC 
 
  256
 
  5
 
 41395
 
 tss
 
 0.05568
 
 0.02484
 
 Not queried
 
  2.2037219
 
 9.52e-02
 
 1.00e+00
  
 
  
 16
 
  85699435
 
  85699907
 
  PDF   STATS   ENSEMBL   UCSC 
 
  473
 
  5
 
 14848
 
 gene
 
 0.50075
 
 0.92876
 
 Not queried
 
  2.2037094
 
 9.52e-02
 
 1.00e+00
  
 
  
 12
 
  53267981
 
  53268433
 
  PDF   STATS   ENSEMBL   UCSC 
 
  453
 
  5
 
 10419
 
 island
 
 0.03709
 
 0.20587
 
 Not queried
 
  2.2033001
 
 4.21e-01
 
 1.00e+00
  
 
  
 17
 
  74864041
 
  74864302
 
  PDF   STATS   ENSEMBL   UCSC 
 
  262
 
  4
 
 17657
 
 tss;island
 
 0.41923
 
 0.14832
 
 Not queried
 
  2.2016611
 
 3.43e-01
 
 1.00e+00
  
 
  
 1
 
 113261488
 
 113261894
 
  PDF   STATS   ENSEMBL   UCSC 
 
  407
 
  6
 
 3600
 
 island
 
 0.06736
 
 0.28706
 
 Not queried
 
  2.2013350
 
 6.49e-02
 
 1.00e+00
  
 
  
 17
 
  40337411
 
  40337892
 
  PDF   STATS   ENSEMBL   UCSC 
 
  482
 
  7
 
 18299
 
 island
 
 0.79642
 
 0.45971
 
 Not queried
 
  2.2009728
 
 5.83e-04
 
 1.00e+00
  
 
  
 4
 
  40631571
 
  40632020
 
  PDF   STATS   ENSEMBL   UCSC 
 
  450
 
  6
 
 30944
 
 island
 
 0.00593
 
 0.02814
 
 Not queried
 
  2.2005216
 
 2.60e-02
 
 1.00e+00
  
 
  
 11
 
 101000566
 
 101000717
 
  PDF   STATS   ENSEMBL   UCSC 
 
  152
 
  5
 
 7620
 
 tss
 
 0.03091
 
 0.12783
 
 Not queried
 
  2.2000591
 
 3.10e-01
 
 1.00e+00
  
 
  
 12
 
   6756671
 
   6756815
 
  PDF   STATS   ENSEMBL   UCSC 
 
  145
 
  4
 
 9494
 
 tss
 
 0.10667
 
 0.37279
 
 Not queried
 
  2.1987367
 
 2.00e-01
 
 1.00e+00
  
 
  
 11
 
  65360123
 
  65360509
 
  PDF   STATS   ENSEMBL   UCSC 
 
  387
 
  5
 
 8445
 
 island
 
 0.03937
 
 0.29296
 
 Not queried
 
  2.1980827
 
 1.51e-01
 
 1.00e+00
  
 
  
 2
 
  45155408
 
  45155774
 
  PDF   STATS   ENSEMBL   UCSC 
 
  367
 
  5
 
 24220
 
 island
 
 0.25607
 
 0.08633
 
 Not queried
 
  2.1978112
 
 5.56e-02
 
 1.00e+00
  
 
  
 22
 
  25758621
 
  25758749
 
  PDF   STATS   ENSEMBL   UCSC 
 
  129
 
  4
 
 26988
 
 tss
 
 0.48143
 
 0.78779
 
 Not queried
 
  2.1967497
 
 6.86e-01
 
 1.00e+00
  
 
  
 3
 
 169482895
 
 169483295
 
  PDF   STATS   ENSEMBL   UCSC 
 
  401
 
  8
 
 28664
 
 tss
 
 0.32677
 
 0.76452
 
 Not queried
 
  2.1960267
 
 3.82e-01
 
 1.00e+00
  
 
  
 10
 
 124909711
 
 124910217
 
  PDF   STATS   ENSEMBL   UCSC 
 
  507
 
  4
 
 6199
 
 island
 
 0.55728
 
 0.24503
 
 Not queried
 
  2.1935876
 
 3.43e-01
 
 1.00e+00
  
 
  
 17
 
  19647973
 
  19648414
 
  PDF   STATS   ENSEMBL   UCSC 
 
  442
 
  4
 
 16447
 
 gene;island
 
 0.02990
 
 0.10060
 
 Not queried
 
  2.1934687
 
 3.43e-01
 
 1.00e+00
  
 
  
 3
 
 169487146
 
 169487465
 
  PDF   STATS   ENSEMBL   UCSC 
 
  320
 
  4
 
 27980
 
 gene;island
 
 0.39148
 
 0.78815
 
 Not queried
 
  2.1931957
 
 2.86e-02
 
 1.00e+00
  
 
  
 20
 
  57428282
 
  57428473
 
  PDF   STATS   ENSEMBL   UCSC 
 
  192
 
  6
 
 25434
 
 gene;island
 
 0.89220
 
 0.40937
 
 Not queried
 
  2.1930012
 
 4.11e-02
 
 1.00e+00
  
 
  
 15
 
  73661642
 
  73662002
 
  PDF   STATS   ENSEMBL   UCSC 
 
  361
 
  6
 
 13753
 
 tss
 
 0.19206
 
 0.06028
 
 Not queried
 
  2.1928309
 
 1.32e-01
 
 1.00e+00
  
 
  
 14
 
  37135384
 
  37135671
 
  PDF   STATS   ENSEMBL   UCSC 
 
  288
 
  4
 
 12039
 
 gene;island
 
 0.47730
 
 0.15197
 
 Not queried
 
  2.1925818
 
 2.86e-02
 
 1.00e+00
  
 
  
 20
 
  62795341
 
  62795807
 
  PDF   STATS   ENSEMBL   UCSC 
 
  467
 
  5
 
 25876
 
 tss
 
 0.47673
 
 0.74706
 
 Not queried
 
  2.1924179
 
 9.52e-02
 
 1.00e+00
  
 
  
 1
 
 148000260
 
 148000861
 
  PDF   STATS   ENSEMBL   UCSC 
 
  602
 
  5
 
 3692
 
 island
 
 0.23872
 
 0.05070
 
 Not queried
 
  2.1914911
 
 1.51e-01
 
 1.00e+00
  
 
  
 2
 
  10712018
 
  10712241
 
  PDF   STATS   ENSEMBL   UCSC 
 
  224
 
  4
 
 22531
 
 gene
 
 0.72983
 
 0.91318
 
 Not queried
 
  2.1908579
 
 4.86e-01
 
 1.00e+00
  
 
  
 17
 
  79380400
 
  79380706
 
  PDF   STATS   ENSEMBL   UCSC 
 
  307
 
  5
 
 16874
 
 gene;island
 
 0.58597
 
 0.85737
 
 Not queried
 
  2.1895536
 
 2.22e-01
 
 1.00e+00
  
 
  
 12
 
 132865182
 
 132865474
 
  PDF   STATS   ENSEMBL   UCSC 
 
  293
 
  4
 
 9420
 
 gene;island
 
 0.51589
 
 0.90629
 
 Not queried
 
  2.1877554
 
 1.14e-01
 
 1.00e+00
  
 
  
 8
 
  80523191
 
  80523364
 
  PDF   STATS   ENSEMBL   UCSC 
 
  174
 
  4
 
 39716
 
 tss
 
 0.26789
 
 0.09168
 
 Not queried
 
  2.1876891
 
 1.14e-01
 
 1.00e+00
  
 
  
 8
 
  80523191
 
  80523461
 
  PDF   STATS   ENSEMBL   UCSC 
 
  271
 
  6
 
 40343
 
 island
 
 0.24660
 
 0.08469
 
 Not queried
 
  2.1876891
 
 8.66e-03
 
 1.00e+00
  
 
  
 16
 
   1226067
 
   1226302
 
  PDF   STATS   ENSEMBL   UCSC 
 
  236
 
  4
 
 14529
 
 gene;island
 
 0.53214
 
 0.82594
 
 Not queried
 
  2.1861186
 
 1.14e-01
 
 1.00e+00
  
 
  
 11
 
  67764948
 
  67765204
 
  PDF   STATS   ENSEMBL   UCSC 
 
  257
 
  4
 
 6747
 
 gene;island
 
 0.61688
 
 0.73782
 
 Not queried
 
  2.1861134
 
 6.86e-01
 
 1.00e+00
  
 
  
 11
 
   1036714
 
   1036938
 
  PDF   STATS   ENSEMBL   UCSC 
 
  225
 
  6
 
 7016
 
 tss
 
 0.63765
 
 0.87567
 
 Not queried
 
  2.1859589
 
 2.16e-03
 
 1.00e+00
  
 
  
 10
 
 129535138
 
 129535968
 
  PDF   STATS   ENSEMBL   UCSC 
 
  831
 
 11
 
 6230
 
 island
 
 0.18260
 
 0.47061
 
 Not queried
 
  2.1848877
 
 2.33e-02
 
 1.00e+00
  
 
  
 1
 
 209825672
 
 209825943
 
  PDF   STATS   ENSEMBL   UCSC 
 
  272
 
  7
 
 2226
 
 tss
 
 0.55667
 
 0.80203
 
 Not queried
 
  2.1842153
 
 6.99e-03
 
 1.00e+00
  
 
  
 19
 
  12948848
 
  12949060
 
  PDF   STATS   ENSEMBL   UCSC 
 
  213
 
  4
 
 20480
 
 tss;island
 
 0.19749
 
 0.02716
 
 Not queried
 
  2.1840564
 
 6.86e-01
 
 1.00e+00
  
 
  
 5
 
  76936361
 
  76936793
 
  PDF   STATS   ENSEMBL   UCSC 
 
  433
 
  4
 
 32840
 
 island
 
 0.12908
 
 0.03179
 
 Not queried
 
  2.1836786
 
 5.71e-02
 
 1.00e+00
  
 
  
 17
 
  40346279
 
  40346798
 
  PDF   STATS   ENSEMBL   UCSC 
 
  520
 
  8
 
 17325
 
 tss
 
 0.07050
 
 0.32077
 
 Not queried
 
  2.1827557
 
 4.99e-02
 
 1.00e+00
  
 
  
 22
 
  42062693
 
  42063028
 
  PDF   STATS   ENSEMBL   UCSC 
 
  336
 
  4
 
 27484
 
 island
 
 0.01801
 
 0.07460
 
 Not queried
 
  2.1826776
 
 5.71e-02
 
 1.00e+00
  
 
  
 11
 
  32449163
 
  32450244
 
  PDF   STATS   ENSEMBL   UCSC 
 
 1082
 
  8
 
 6547
 
 gene;island
 
 0.12826
 
 0.51338
 
 Not queried
 
  2.1823128
 
 2.79e-01
 
 1.00e+00
  
 
  
 2
 
 201980504
 
 201980910
 
  PDF   STATS   ENSEMBL   UCSC 
 
  407
 
  8
 
 24968
 
 island
 
 0.31959
 
 0.16124
 
 Not queried
 
  2.1811631
 
 1.95e-01
 
 1.00e+00
  
 
  
 2
 
 129494526
 
 129495163
 
  PDF   STATS   ENSEMBL   UCSC 
 
  638
 
  6
 
 24672
 
 island
 
 0.58585
 
 0.80755
 
 Not queried
 
  2.1802808
 
 9.31e-02
 
 1.00e+00
  
 
  
 2
 
  24397539
 
  24397845
 
  PDF   STATS   ENSEMBL   UCSC 
 
  307
 
  5
 
 23187
 
 tss;island
 
 0.69216
 
 0.94201
 
 Not queried
 
  2.1795766
 
 9.52e-02
 
 1.00e+00
  
 
  
 1
 
  79472532
 
  79472643
 
  PDF   STATS   ENSEMBL   UCSC 
 
  112
 
  4
 
 1708
 
 tss
 
 0.23461
 
 0.06392
 
 Not queried
 
  2.1784545
 
 2.86e-02
 
 1.00e+00
  
 
  
 14
 
  29254530
 
  29255119
 
  PDF   STATS   ENSEMBL   UCSC 
 
  590
 
  6
 
 12026
 
 gene;island
 
 0.21266
 
 0.04692
 
 Not queried
 
  2.1783780
 
 6.49e-02
 
 1.00e+00
  
 
  
 6
 
  29894619
 
  29895260
 
  PDF   STATS   ENSEMBL   UCSC 
 
  642
 
 26
 
 35580
 
 island
 
 0.08022
 
 0.31167
 
 Not queried
 
  2.1782652
 
 2.44e-03
 
 1.00e+00
  
 
  
 X
 
  70288342
 
  70288735
 
  PDF   STATS   ENSEMBL   UCSC 
 
  394
 
  8
 
 41371
 
 tss
 
 0.17891
 
 0.03602
 
 Not queried
 
  2.1779631
 
 1.09e-03
 
 1.00e+00
  
 
  
 1
 
  33351568
 
  33351946
 
  PDF   STATS   ENSEMBL   UCSC 
 
  379
 
  6
 
 1462
 
 tss;island
 
 0.04009
 
 0.18720
 
 Not queried
 
  2.1774674
 
 4.11e-02
 
 1.00e+00
  
 
  
 5
 
   1394296
 
   1394633
 
  PDF   STATS   ENSEMBL   UCSC 
 
  338
 
  5
 
 31482
 
 gene
 
 0.58587
 
 0.86139
 
 Not queried
 
  2.1767639
 
 2.22e-01
 
 1.00e+00
  
 
  
 3
 
  42947481
 
  42947771
 
  PDF   STATS   ENSEMBL   UCSC 
 
  291
 
  4
 
 27694
 
 gene
 
 0.71263
 
 0.91243
 
 Not queried
 
  2.1763259
 
 2.86e-02
 
 1.00e+00
  
 
  
 16
 
  84328104
 
  84328391
 
  PDF   STATS   ENSEMBL   UCSC 
 
  288
 
  6
 
 15362
 
 tss;island
 
 0.29301
 
 0.57490
 
 Not queried
 
  2.1755808
 
 2.60e-02
 
 1.00e+00
  
 
  
 11
 
 116371188
 
 116371487
 
  PDF   STATS   ENSEMBL   UCSC 
 
  300
 
  4
 
 8808
 
 island
 
 0.92918
 
 0.76960
 
 Not queried
 
  2.1754724
 
 2.86e-02
 
 1.00e+00
  
 
  
 12
 
  95009858
 
  95010113
 
  PDF   STATS   ENSEMBL   UCSC 
 
  256
 
  5
 
 9225
 
 gene
 
 0.41958
 
 0.80960
 
 Not queried
 
  2.1741826
 
 1.59e-02
 
 1.00e+00
  
 
  
 8
 
  42623686
 
  42623946
 
  PDF   STATS   ENSEMBL   UCSC 
 
  261
 
  6
 
 39617
 
 tss
 
 0.84220
 
 0.91993
 
 Not queried
 
  2.1727813
 
 1.32e-01
 
 1.00e+00
  
 
  
 17
 
  79259283
 
  79259699
 
  PDF   STATS   ENSEMBL   UCSC 
 
  417
 
  4
 
 16872
 
 gene;island
 
 0.67969
 
 0.89266
 
 Not queried
 
  2.1722604
 
 2.86e-02
 
 1.00e+00
  
 
  
 11
 
  66059064
 
  66059164
 
  PDF   STATS   ENSEMBL   UCSC 
 
  101
 
  4
 
 7442
 
 tss;island
 
 0.14810
 
 0.06115
 
 Not queried
 
  2.1719688
 
 1.14e-01
 
 1.00e+00
  
 
  
 8
 
 143761503
 
 143761811
 
  PDF   STATS   ENSEMBL   UCSC 
 
  309
 
  6
 
 39868
 
 tss
 
 0.38208
 
 0.72665
 
 Not queried
 
  2.1709815
 
 4.11e-02
 
 1.00e+00
  
 
  
 12
 
 113909932
 
 113910034
 
  PDF   STATS   ENSEMBL   UCSC 
 
  103
 
  6
 
 10005
 
 tss;island
 
 0.31995
 
 0.08490
 
 Not queried
 
  2.1708590
 
 2.60e-02
 
 1.00e+00
  
 
  
 19
 
  52872916
 
  52873624
 
  PDF   STATS   ENSEMBL   UCSC 
 
  709
 
 10
 
 22321
 
 island
 
 0.03657
 
 0.16833
 
 Not queried
 
  2.1705117
 
 1.50e-03
 
 1.00e+00
  
 
  
 18
 
  21852118
 
  21852436
 
  PDF   STATS   ENSEMBL   UCSC 
 
  319
 
  6
 
 19045
 
 gene;island
 
 0.01826
 
 0.08311
 
 Not queried
 
  2.1695255
 
 4.11e-02
 
 1.00e+00
  
 
  
 6
 
 134209935
 
 134210307
 
  PDF   STATS   ENSEMBL   UCSC 
 
  373
 
  9
 
 36427
 
 island
 
 0.12941
 
 0.05087
 
 Not queried
 
  2.1695058
 
 1.85e-03
 
 1.00e+00
  
 
  
 6
 
  10521142
 
  10521558
 
  PDF   STATS   ENSEMBL   UCSC 
 
  417
 
  4
 
 34562
 
 tss
 
 0.01500
 
 0.06216
 
 Not queried
 
  2.1689660
 
 2.00e-01
 
 1.00e+00
  
 
  
 14
 
  38065127
 
  38065582
 
  PDF   STATS   ENSEMBL   UCSC 
 
  456
 
  6
 
 12335
 
 tss;island
 
 0.03982
 
 0.17703
 
 Not queried
 
  2.1687903
 
 6.49e-02
 
 1.00e+00
  
 
  
 10
 
 134859687
 
 134860210
 
  PDF   STATS   ENSEMBL   UCSC 
 
  524
 
  7
 
 6310
 
 island
 
 0.58109
 
 0.87324
 
 Not queried
 
  2.1663455
 
 9.73e-02
 
 1.00e+00
  
 
  
 19
 
    496239
 
    496528
 
  PDF   STATS   ENSEMBL   UCSC 
 
  290
 
  6
 
 21126
 
 island
 
 0.86811
 
 0.55396
 
 Not queried
 
  2.1660621
 
 4.11e-02
 
 1.00e+00
  
 
  
 7
 
   1753396
 
   1753659
 
  PDF   STATS   ENSEMBL   UCSC 
 
  264
 
  6
 
 36703
 
 gene;island
 
 0.72256
 
 0.37436
 
 Not queried
 
  2.1656495
 
 2.60e-02
 
 1.00e+00
  
 
  
 17
 
  75451779
 
  75452100
 
  PDF   STATS   ENSEMBL   UCSC 
 
  322
 
  5
 
 16774
 
 gene
 
 0.60470
 
 0.88063
 
 Not queried
 
  2.1654890
 
 7.94e-03
 
 1.00e+00
  
 
  
 6
 
  31324997
 
  31325455
 
  PDF   STATS   ENSEMBL   UCSC 
 
  459
 
 11
 
 34743
 
 tss
 
 0.14153
 
 0.03061
 
 Not queried
 
  2.1649825
 
 1.04e-02
 
 1.00e+00
  
 
  
 22
 
  37099037
 
  37099259
 
  PDF   STATS   ENSEMBL   UCSC 
 
  223
 
  4
 
 27069
 
 tss;island
 
 0.04690
 
 0.08786
 
 Not queried
 
  2.1637815
 
 3.43e-01
 
 1.00e+00
  
 
  
 18
 
  77125738
 
  77125858
 
  PDF   STATS   ENSEMBL   UCSC 
 
  121
 
  4
 
 19102
 
 gene;island
 
 0.73688
 
 0.91542
 
 Not queried
 
  2.1633593
 
 2.86e-02
 
 1.00e+00
  
 
  
 12
 
  57619178
 
  57619401
 
  PDF   STATS   ENSEMBL   UCSC 
 
  224
 
  5
 
 9164
 
 gene;island
 
 0.64128
 
 0.38736
 
 Not queried
 
  2.1633074
 
 1.51e-01
 
 1.00e+00
  
 
  
 X
 
  48858679
 
  48858921
 
  PDF   STATS   ENSEMBL   UCSC 
 
  243
 
  9
 
 41304
 
 tss
 
 0.05618
 
 0.01117
 
 Not queried
 
  2.1624999
 
 3.99e-03
 
 1.00e+00
  
 
  
 4
 
  46391414
 
  46391929
 
  PDF   STATS   ENSEMBL   UCSC 
 
  516
 
 11
 
 30325
 
 tss
 
 0.03441
 
 0.08096
 
 Not queried
 
  2.1622666
 
 1.92e-02
 
 1.00e+00
  
 
  
 19
 
   2428350
 
   2428677
 
  PDF   STATS   ENSEMBL   UCSC 
 
  328
 
  4
 
 19654
 
 gene;tss;island
 
 0.68198
 
 0.90551
 
 Not queried
 
  2.1611734
 
 2.86e-02
 
 1.00e+00
  
 
  
 7
 
  12609907
 
  12610833
 
  PDF   STATS   ENSEMBL   UCSC 
 
  927
 
 15
 
 38203
 
 island
 
 0.04063
 
 0.21967
 
 Not queried
 
  2.1605240
 
 4.94e-03
 
 1.00e+00
  
 
  
 22
 
  25575246
 
  25575559
 
  PDF   STATS   ENSEMBL   UCSC 
 
  314
 
  4
 
 26778
 
 gene;island
 
 0.32074
 
 0.71532
 
 Not queried
 
  2.1596933
 
 2.00e-01
 
 1.00e+00
  
 
  
 3
 
  82857026
 
  82857285
 
  PDF   STATS   ENSEMBL   UCSC 
 
  260
 
  4
 
 29253
 
 island
 
 0.24359
 
 0.67109
 
 Not queried
 
  2.1596357
 
 2.00e-01
 
 1.00e+00
  
 
  
 16
 
  56554066
 
  56554313
 
  PDF   STATS   ENSEMBL   UCSC 
 
  248
 
  5
 
 15214
 
 tss
 
 0.33792
 
 0.06215
 
 Not queried
 
  2.1593695
 
 5.48e-01
 
 1.00e+00
  
 
  
 8
 
   1132712
 
   1133105
 
  PDF   STATS   ENSEMBL   UCSC 
 
  394
 
  5
 
 39953
 
 island
 
 0.86773
 
 0.92962
 
 Not queried
 
  2.1590828
 
 1.51e-01
 
 1.00e+00
  
 
  
 4
 
  55095282
 
  55095529
 
  PDF   STATS   ENSEMBL   UCSC 
 
  248
 
  4
 
 29959
 
 gene
 
 0.18559
 
 0.07300
 
 Not queried
 
  2.1584851
 
 3.43e-01
 
 1.00e+00
  
 
  
 6
 
  30510238
 
  30510459
 
  PDF   STATS   ENSEMBL   UCSC 
 
  222
 
  4
 
 33710
 
 gene
 
 0.27357
 
 0.53186
 
 Not queried
 
  2.1583146
 
 2.00e-01
 
 1.00e+00
  
 
  
 5
 
   1882775
 
   1883011
 
  PDF   STATS   ENSEMBL   UCSC 
 
  237
 
  6
 
 32585
 
 island
 
 0.02183
 
 0.14866
 
 Not queried
 
  2.1563960
 
 9.31e-02
 
 1.00e+00
  
 
  
 8
 
 145106246
 
 145106582
 
  PDF   STATS   ENSEMBL   UCSC 
 
  337
 
  4
 
 39444
 
 gene;island
 
 0.44378
 
 0.80014
 
 Not queried
 
  2.1558887
 
 2.86e-02
 
 1.00e+00
  
 
  
 11
 
 111410935
 
 111411216
 
  PDF   STATS   ENSEMBL   UCSC 
 
  282
 
  7
 
 7666
 
 tss
 
 0.35768
 
 0.04260
 
 Not queried
 
  2.1549053
 
 4.56e-01
 
 1.00e+00
  
 
  
 4
 
 187810446
 
 187810783
 
  PDF   STATS   ENSEMBL   UCSC 
 
  338
 
  5
 
 31433
 
 island
 
 0.74223
 
 0.93161
 
 Not queried
 
  2.1538583
 
 1.59e-02
 
 1.00e+00
  
 
  
 16
 
  56998544
 
  56998712
 
  PDF   STATS   ENSEMBL   UCSC 
 
  169
 
  4
 
 14734
 
 gene
 
 0.05083
 
 0.22490
 
 Not queried
 
  2.1537826
 
 2.00e-01
 
 1.00e+00
  
 
  
 4
 
 153859106
 
 153859560
 
  PDF   STATS   ENSEMBL   UCSC 
 
  455
 
  5
 
 31314
 
 island
 
 0.02269
 
 0.06773
 
 Not queried
 
  2.1512952
 
 2.22e-01
 
 1.00e+00
  
 
  
 17
 
  36734475
 
  36734942
 
  PDF   STATS   ENSEMBL   UCSC 
 
  468
 
  5
 
 16505
 
 gene;island
 
 0.40441
 
 0.15092
 
 Not queried
 
  2.1510680
 
 1.59e-02
 
 1.00e+00
  
 
  
 2
 
 176964404
 
 176964512
 
  PDF   STATS   ENSEMBL   UCSC 
 
  109
 
  5
 
 23695
 
 tss
 
 0.77328
 
 0.38759
 
 Not queried
 
  2.1505575
 
 7.94e-03
 
 1.00e+00
  
 
  
 2
 
 176964404
 
 176964720
 
  PDF   STATS   ENSEMBL   UCSC 
 
  317
 
  7
 
 24855
 
 island
 
 0.68476
 
 0.34850
 
 Not queried
 
  2.1505575
 
 5.83e-04
 
 1.00e+00
  
 
  
 19
 
   1675848
 
   1676136
 
  PDF   STATS   ENSEMBL   UCSC 
 
  289
 
  4
 
 21200
 
 island
 
 0.03652
 
 0.15685
 
 Not queried
 
  2.1489143
 
 5.71e-02
 
 1.00e+00
  
 
  
 2
 
 219857793
 
 219858271
 
  PDF   STATS   ENSEMBL   UCSC 
 
  479
 
  9
 
 25062
 
 island
 
 0.32221
 
 0.04601
 
 Not queried
 
  2.1480820
 
 2.76e-03
 
 1.00e+00
  
 
  
 2
 
  97173713
 
  97174391
 
  PDF   STATS   ENSEMBL   UCSC 
 
  679
 
 12
 
 24487
 
 island
 
 0.07870
 
 0.24660
 
 Not queried
 
  2.1479784
 
 3.32e-02
 
 1.00e+00
  
 
  
 X
 
  48776349
 
  48776812
 
  PDF   STATS   ENSEMBL   UCSC 
 
  464
 
  8
 
 41777
 
 island
 
 0.10576
 
 0.02019
 
 Not queried
 
  2.1465374
 
 1.48e-02
 
 1.00e+00
  
 
  
 7
 
   1067951
 
   1068244
 
  PDF   STATS   ENSEMBL   UCSC 
 
  294
 
  5
 
 36675
 
 gene;island
 
 0.02603
 
 0.13232
 
 Not queried
 
  2.1464231
 
 7.94e-03
 
 1.00e+00
  
 
  
 10
 
  57389676
 
  57390047
 
  PDF   STATS   ENSEMBL   UCSC 
 
  372
 
  4
 
 5735
 
 island
 
 0.54572
 
 0.73537
 
 Not queried
 
  2.1435749
 
 3.43e-01
 
 1.00e+00
  
 
  
 6
 
  13924355
 
  13924708
 
  PDF   STATS   ENSEMBL   UCSC 
 
  354
 
  6
 
 34582
 
 tss;island
 
 0.14897
 
 0.04392
 
 Not queried
 
  2.1432942
 
 3.94e-01
 
 1.00e+00
  
 
  
 7
 
    862427
 
    862831
 
  PDF   STATS   ENSEMBL   UCSC 
 
  405
 
  6
 
 36661
 
 gene;island
 
 0.58200
 
 0.85408
 
 Not queried
 
  2.1415024
 
 1.80e-01
 
 1.00e+00
  
 
  
 17
 
  46670933
 
  46671635
 
  PDF   STATS   ENSEMBL   UCSC 
 
  703
 
 13
 
 16620
 
 gene;island
 
 0.01962
 
 0.05704
 
 Not queried
 
  2.1406984
 
 4.22e-03
 
 1.00e+00
  
 
  
 11
 
 111154742
 
 111155021
 
  PDF   STATS   ENSEMBL   UCSC 
 
  280
 
  6
 
 6877
 
 gene
 
 0.69602
 
 0.89572
 
 Not queried
 
  2.1403433
 
 2.40e-01
 
 1.00e+00
  
 
  
 6
 
  31025933
 
  31026287
 
  PDF   STATS   ENSEMBL   UCSC 
 
  355
 
  6
 
 33786
 
 gene
 
 0.16097
 
 0.43584
 
 Not queried
 
  2.1401050
 
 8.66e-03
 
 1.00e+00
  
 
  
 10
 
   3823758
 
   3824016
 
  PDF   STATS   ENSEMBL   UCSC 
 
  259
 
  5
 
 4462
 
 gene;island
 
 0.67361
 
 0.87639
 
 Not queried
 
  2.1398680
 
 2.22e-01
 
 1.00e+00
  
 
  
 11
 
  69516939
 
  69517471
 
  PDF   STATS   ENSEMBL   UCSC 
 
  533
 
  4
 
 6765
 
 gene;island
 
 0.21265
 
 0.10264
 
 Not queried
 
  2.1395758
 
 4.86e-01
 
 1.00e+00
  
 
  
 11
 
  33680220
 
  33680427
 
  PDF   STATS   ENSEMBL   UCSC 
 
  208
 
  4
 
 6558
 
 gene;island
 
 0.48710
 
 0.82393
 
 Not queried
 
  2.1395060
 
 2.86e-02
 
 1.00e+00
  
 
  
 3
 
  40518435
 
  40518798
 
  PDF   STATS   ENSEMBL   UCSC 
 
  364
 
  6
 
 28962
 
 island
 
 0.05721
 
 0.11746
 
 Not queried
 
  2.1387413
 
 2.40e-01
 
 1.00e+00
  
 
  
 14
 
  71107931
 
  71108199
 
  PDF   STATS   ENSEMBL   UCSC 
 
  269
 
  4
 
 12460
 
 tss;island
 
 0.03151
 
 0.13433
 
 Not queried
 
  2.1379104
 
 5.71e-02
 
 1.00e+00
  
 
  
 12
 
 113592319
 
 113592619
 
  PDF   STATS   ENSEMBL   UCSC 
 
  301
 
  4
 
 9295
 
 gene;island
 
 0.08009
 
 0.29299
 
 Not queried
 
  2.1378057
 
 2.86e-02
 
 1.00e+00
  
 
  
 11
 
  74022348
 
  74023050
 
  PDF   STATS   ENSEMBL   UCSC 
 
  703
 
 11
 
 8626
 
 island
 
 0.25903
 
 0.12362
 
 Not queried
 
  2.1376995
 
 1.93e-01
 
 1.00e+00
  
 
  
 11
 
  43963820
 
  43964150
 
  PDF   STATS   ENSEMBL   UCSC 
 
  331
 
  7
 
 8198
 
 island
 
 0.01406
 
 0.01220
 
 Not queried
 
  2.1375077
 
 3.18e-01
 
 1.00e+00
  
 
  
 2
 
  71680559
 
  71680761
 
  PDF   STATS   ENSEMBL   UCSC 
 
  203
 
  5
 
 24361
 
 island
 
 0.03406
 
 0.14632
 
 Not queried
 
  2.1348918
 
 7.94e-03
 
 1.00e+00
  
 
  
 11
 
  67183148
 
  67183616
 
  PDF   STATS   ENSEMBL   UCSC 
 
  469
 
  5
 
 6735
 
 gene
 
 0.13219
 
 0.40079
 
 Not queried
 
  2.1344800
 
 2.22e-01
 
 1.00e+00
  
 
  
 6
 
  31590513
 
  31590870
 
  PDF   STATS   ENSEMBL   UCSC 
 
  358
 
 10
 
 33847
 
 gene;island
 
 0.58408
 
 0.83327
 
 Not queried
 
  2.1334318
 
 3.55e-02
 
 1.00e+00
  
 
  
 X
 
  67867130
 
  67867448
 
  PDF   STATS   ENSEMBL   UCSC 
 
  319
 
  8
 
 41357
 
 tss
 
 0.68070
 
 0.20895
 
 Not queried
 
  2.1328706
 
 1.05e-01
 
 1.00e+00
  
 
  
 7
 
  45961772
 
  45962236
 
  PDF   STATS   ENSEMBL   UCSC 
 
  465
 
 13
 
 37523
 
 tss;island
 
 0.02801
 
 0.16001
 
 Not queried
 
  2.1322773
 
 2.95e-02
 
 1.00e+00
  
 
  
 19
 
   9473696
 
   9473880
 
  PDF   STATS   ENSEMBL   UCSC 
 
  185
 
  4
 
 19764
 
 gene
 
 0.85052
 
 0.58930
 
 Not queried
 
  2.1315596
 
 2.86e-02
 
 1.00e+00
  
 
  
 10
 
   1256339
 
   1256487
 
  PDF   STATS   ENSEMBL   UCSC 
 
  149
 
  4
 
 5474
 
 island
 
 0.42279
 
 0.76473
 
 Not queried
 
  2.1314863
 
 2.86e-02
 
 1.00e+00
  
 
  
 1
 
  60392140
 
  60392392
 
  PDF   STATS   ENSEMBL   UCSC 
 
  253
 
  4
 
 559
 
 gene
 
 0.19927
 
 0.05900
 
 Not queried
 
  2.1314534
 
 2.86e-02
 
 1.00e+00
  
 
  
 X
 
  17673726
 
  17674041
 
  PDF   STATS   ENSEMBL   UCSC 
 
  316
 
  4
 
 40944
 
 gene;island
 
 0.09540
 
 0.28673
 
 Not queried
 
  2.1310200
 
 2.86e-02
 
 1.00e+00
  
 
  
 13
 
 113698408
 
 113698688
 
  PDF   STATS   ENSEMBL   UCSC 
 
  281
 
  6
 
 11242
 
 gene;island
 
 0.68925
 
 0.88927
 
 Not queried
 
  2.1308201
 
 2.16e-03
 
 1.00e+00
  
 
  
 11
 
  66104018
 
  66104174
 
  PDF   STATS   ENSEMBL   UCSC 
 
  157
 
  4
 
 7444
 
 tss
 
 0.47368
 
 0.79051
 
 Not queried
 
  2.1306389
 
 1.14e-01
 
 1.00e+00
  
 
  
 22
 
  19949873
 
  19950166
 
  PDF   STATS   ENSEMBL   UCSC 
 
  294
 
  7
 
 26754
 
 gene
 
 0.04528
 
 0.28433
 
 Not queried
 
  2.1300865
 
 2.33e-03
 
 1.00e+00
  
 
  
 6
 
  27805693
 
  27806081
 
  PDF   STATS   ENSEMBL   UCSC 
 
  389
 
  5
 
 33557
 
 gene
 
 0.02440
 
 0.13852
 
 Not queried
 
  2.1297685
 
 5.56e-02
 
 1.00e+00
  
 
  
 6
 
  32782617
 
  32783046
 
  PDF   STATS   ENSEMBL   UCSC 
 
  430
 
  5
 
 34068
 
 gene
 
 0.71561
 
 0.46312
 
 Not queried
 
  2.1296478
 
 1.59e-02
 
 1.00e+00
  
 
  
 6
 
  28493019
 
  28493664
 
  PDF   STATS   ENSEMBL   UCSC 
 
  646
 
  9
 
 34679
 
 tss
 
 0.90006
 
 0.65521
 
 Not queried
 
  2.1295780
 
 1.85e-03
 
 1.00e+00
  
 
  
 17
 
  74381133
 
  74381280
 
  PDF   STATS   ENSEMBL   UCSC 
 
  148
 
  4
 
 17644
 
 tss
 
 0.05226
 
 0.13317
 
 Not queried
 
  2.1291159
 
 1.14e-01
 
 1.00e+00
  
 
  
 19
 
   3029503
 
   3029785
 
  PDF   STATS   ENSEMBL   UCSC 
 
  283
 
  4
 
 19667
 
 gene;tss;island
 
 0.59936
 
 0.26006
 
 Not queried
 
  2.1278295
 
 3.43e-01
 
 1.00e+00
  
 
  
 2
 
 201980504
 
 201980867
 
  PDF   STATS   ENSEMBL   UCSC 
 
  364
 
  7
 
 23776
 
 tss
 
 0.48635
 
 0.28557
 
 Not queried
 
  2.1278080
 
 2.59e-01
 
 1.00e+00
  
 
  
 17
 
  41909601
 
  41909716
 
  PDF   STATS   ENSEMBL   UCSC 
 
  116
 
  4
 
 16564
 
 gene;island
 
 0.75395
 
 0.41459
 
 Not queried
 
  2.1271525
 
 2.00e-01
 
 1.00e+00
  
 
  
 17
 
  80373129
 
  80373482
 
  PDF   STATS   ENSEMBL   UCSC 
 
  354
 
  5
 
 16913
 
 gene;island
 
 0.57673
 
 0.88976
 
 Not queried
 
  2.1259070
 
 2.22e-01
 
 1.00e+00
  
 
  
 8
 
 133573739
 
 133574073
 
  PDF   STATS   ENSEMBL   UCSC 
 
  335
 
  5
 
 39855
 
 tss
 
 0.82284
 
 0.51552
 
 Not queried
 
  2.1258981
 
 4.21e-01
 
 1.00e+00
  
 
  
 6
 
  29635110
 
  29635692
 
  PDF   STATS   ENSEMBL   UCSC 
 
  583
 
  8
 
 33623
 
 gene
 
 0.27595
 
 0.11953
 
 Not queried
 
  2.1255020
 
 1.95e-01
 
 1.00e+00
  
 
  
 19
 
  52873173
 
  52873624
 
  PDF   STATS   ENSEMBL   UCSC 
 
  452
 
  4
 
 20157
 
 gene
 
 0.02498
 
 0.13231
 
 Not queried
 
  2.1253946
 
 5.71e-02
 
 1.00e+00
  
 
  
 3
 
 129982835
 
 129983089
 
  PDF   STATS   ENSEMBL   UCSC 
 
  255
 
  4
 
 27904
 
 gene;island
 
 0.34332
 
 0.71023
 
 Not queried
 
  2.1239971
 
 2.86e-02
 
 1.00e+00
  
 
  
 11
 
  67417958
 
  67418113
 
  PDF   STATS   ENSEMBL   UCSC 
 
  156
 
  4
 
 6744
 
 gene
 
 0.45322
 
 0.80418
 
 Not queried
 
  2.1233670
 
 5.71e-02
 
 1.00e+00
  
 
  
 14
 
  37124018
 
  37124434
 
  PDF   STATS   ENSEMBL   UCSC 
 
  417
 
  5
 
 12762
 
 island
 
 0.47932
 
 0.84939
 
 Not queried
 
  2.1221237
 
 1.59e-02
 
 1.00e+00
  
 
  
 11
 
 115372874
 
 115373119
 
  PDF   STATS   ENSEMBL   UCSC 
 
  246
 
  4
 
 6892
 
 gene;island
 
 0.20192
 
 0.03273
 
 Not queried
 
  2.1209453
 
 5.71e-02
 
 1.00e+00
  
 
  
 4
 
   1537622
 
   1537799
 
  PDF   STATS   ENSEMBL   UCSC 
 
  178
 
  4
 
 30741
 
 island
 
 0.67096
 
 0.92718
 
 Not queried
 
  2.1191874
 
 5.71e-02
 
 1.00e+00
  
 
  
 6
 
  29394438
 
  29395425
 
  PDF   STATS   ENSEMBL   UCSC 
 
  988
 
 12
 
 33592
 
 gene
 
 0.50678
 
 0.75441
 
 Not queried
 
  2.1190423
 
 2.42e-02
 
 1.00e+00
  
 
  
 20
 
  30073209
 
  30073576
 
  PDF   STATS   ENSEMBL   UCSC 
 
  368
 
  6
 
 25621
 
 tss;island
 
 0.16373
 
 0.45247
 
 Not queried
 
  2.1188628
 
 4.11e-02
 
 1.00e+00
  
 
  
 8
 
 101224915
 
 101225361
 
  PDF   STATS   ENSEMBL   UCSC 
 
  447
 
  6
 
 39315
 
 gene;island
 
 0.00766
 
 0.03993
 
 Not queried
 
  2.1183866
 
 1.52e-02
 
 1.00e+00
  
 
  
 1
 
  15271830
 
  15272326
 
  PDF   STATS   ENSEMBL   UCSC 
 
  497
 
  6
 
 240
 
 gene;tss
 
 0.40541
 
 0.83824
 
 Not queried
 
  2.1181471
 
 2.60e-02
 
 1.00e+00
  
 
  
 6
 
  32135715
 
  32135803
 
  PDF   STATS   ENSEMBL   UCSC 
 
   89
 
  5
 
 34003
 
 gene;island
 
 0.45457
 
 0.79714
 
 Not queried
 
  2.1180610
 
 7.94e-03
 
 1.00e+00
  
 
  
 2
 
 145277596
 
 145277852
 
  PDF   STATS   ENSEMBL   UCSC 
 
  257
 
  9
 
 22854
 
 gene;island
 
 0.13230
 
 0.02172
 
 Not queried
 
  2.1176852
 
 6.25e-02
 
 1.00e+00
  
 
  
 6
 
 169013150
 
 169013766
 
  PDF   STATS   ENSEMBL   UCSC 
 
  617
 
  6
 
 34498
 
 gene
 
 0.96191
 
 0.87470
 
 Not queried
 
  2.1176339
 
 9.31e-02
 
 1.00e+00
  
 
  
 6
 
 166571782
 
 166572045
 
  PDF   STATS   ENSEMBL   UCSC 
 
  264
 
  5
 
 34478
 
 gene;island
 
 0.94011
 
 0.82917
 
 Not queried
 
  2.1172172
 
 2.22e-01
 
 1.00e+00
  
 
  
 6
 
  31711701
 
  31712195
 
  PDF   STATS   ENSEMBL   UCSC 
 
  495
 
  7
 
 33887
 
 gene;island
 
 0.57988
 
 0.88962
 
 Not queried
 
  2.1171009
 
 2.62e-02
 
 1.00e+00
  
 
  
 X
 
  48754200
 
  48754642
 
  PDF   STATS   ENSEMBL   UCSC 
 
  443
 
  5
 
 40987
 
 gene;tss;island
 
 0.10993
 
 0.02888
 
 Not queried
 
  2.1165431
 
 3.17e-02
 
 1.00e+00
  
 
  
 3
 
 113160071
 
 113160821
 
  PDF   STATS   ENSEMBL   UCSC 
 
  751
 
 10
 
 29321
 
 island
 
 0.71738
 
 0.93257
 
 Not queried
 
  2.1163041
 
 2.88e-03
 
 1.00e+00
  
 
  
 2
 
   3699195
 
   3699353
 
  PDF   STATS   ENSEMBL   UCSC 
 
  159
 
  4
 
 24001
 
 island
 
 0.37881
 
 0.11782
 
 Not queried
 
  2.1159199
 
 2.86e-02
 
 1.00e+00
  
 
  
 5
 
 158531132
 
 158531647
 
  PDF   STATS   ENSEMBL   UCSC 
 
  516
 
  5
 
 33287
 
 island
 
 0.03048
 
 0.18347
 
 Not queried
 
  2.1151458
 
 1.51e-01
 
 1.00e+00
  
 
  
 11
 
 116706372
 
 116706626
 
  PDF   STATS   ENSEMBL   UCSC 
 
  255
 
  4
 
 8814
 
 island
 
 0.32342
 
 0.10255
 
 Not queried
 
  2.1146908
 
 1.14e-01
 
 1.00e+00
  
 
  
 1
 
 149871404
 
 149871658
 
  PDF   STATS   ENSEMBL   UCSC 
 
  255
 
  4
 
 732
 
 gene;island
 
 0.25077
 
 0.56804
 
 Not queried
 
  2.1135015
 
 2.86e-02
 
 1.00e+00
  
 
  
 19
 
   1313407
 
   1313642
 
  PDF   STATS   ENSEMBL   UCSC 
 
  236
 
  4
 
 21177
 
 island
 
 0.43487
 
 0.76752
 
 Not queried
 
  2.1113770
 
 1.14e-01
 
 1.00e+00
  
 
  
 12
 
  54942782
 
  54943102
 
  PDF   STATS   ENSEMBL   UCSC 
 
  321
 
  5
 
 9740
 
 tss
 
 0.57264
 
 0.23994
 
 Not queried
 
  2.1092879
 
 5.56e-02
 
 1.00e+00
  
 
  
 22
 
  19949873
 
  19950040
 
  PDF   STATS   ENSEMBL   UCSC 
 
  168
 
  6
 
 26929
 
 tss
 
 0.04697
 
 0.23148
 
 Not queried
 
  2.1090780
 
 8.66e-03
 
 1.00e+00
  
 
  
 20
 
  42143080
 
  42143502
 
  PDF   STATS   ENSEMBL   UCSC 
 
  423
 
  7
 
 25383
 
 gene
 
 0.00746
 
 0.06478
 
 Not queried
 
  2.1088533
 
 5.30e-02
 
 1.00e+00
  
 
  
 17
 
   6659164
 
   6659562
 
  PDF   STATS   ENSEMBL   UCSC 
 
  399
 
  7
 
 16341
 
 gene
 
 0.37370
 
 0.64329
 
 Not queried
 
  2.1063870
 
 5.83e-04
 
 1.00e+00
  
 
  
 14
 
  51561293
 
  51561618
 
  PDF   STATS   ENSEMBL   UCSC 
 
  326
 
  4
 
 12051
 
 gene;island
 
 0.05342
 
 0.12374
 
 Not queried
 
  2.1062539
 
 5.71e-02
 
 1.00e+00
  
 
  
 14
 
  61788810
 
  61788914
 
  PDF   STATS   ENSEMBL   UCSC 
 
  105
 
  4
 
 12078
 
 gene;island
 
 0.03400
 
 0.20397
 
 Not queried
 
  2.1056527
 
 1.14e-01
 
 1.00e+00
  
 
  
 1
 
 156338639
 
 156338826
 
  PDF   STATS   ENSEMBL   UCSC 
 
  188
 
  4
 
 2011
 
 tss
 
 0.76806
 
 0.51100
 
 Not queried
 
  2.1038503
 
 2.86e-02
 
 1.00e+00
  
 
  
 19
 
   2085286
 
   2085483
 
  PDF   STATS   ENSEMBL   UCSC 
 
  198
 
  4
 
 19643
 
 gene
 
 0.85002
 
 0.96121
 
 Not queried
 
  2.1030086
 
 2.86e-02
 
 1.00e+00
  
 
  
 8
 
 124214953
 
 124215152
 
  PDF   STATS   ENSEMBL   UCSC 
 
  200
 
  4
 
 39355
 
 gene
 
 0.42943
 
 0.64696
 
 Not queried
 
  2.1029845
 
 4.86e-01
 
 1.00e+00
  
 
  
 7
 
  94953956
 
  94954202
 
  PDF   STATS   ENSEMBL   UCSC 
 
  247
 
  4
 
 37660
 
 tss
 
 0.58308
 
 0.91183
 
 Not queried
 
  2.1028190
 
 5.71e-02
 
 1.00e+00
  
 
  
 6
 
  29855110
 
  29855636
 
  PDF   STATS   ENSEMBL   UCSC 
 
  527
 
 16
 
 35577
 
 island
 
 0.66010
 
 0.29621
 
 Not queried
 
  2.1024280
 
 6.53e-04
 
 1.00e+00
  
 
  
 19
 
  40724308
 
  40724388
 
  PDF   STATS   ENSEMBL   UCSC 
 
   81
 
  5
 
 20747
 
 tss;island
 
 0.02452
 
 0.14049
 
 Not queried
 
  2.1023892
 
 5.56e-02
 
 1.00e+00
  
 
  
 19
 
  57352269
 
  57352807
 
  PDF   STATS   ENSEMBL   UCSC 
 
  539
 
 12
 
 20205
 
 gene
 
 0.11638
 
 0.05014
 
 Not queried
 
  2.1008593
 
 1.60e-01
 
 1.00e+00
  
 
  
 19
 
  15530606
 
  15530870
 
  PDF   STATS   ENSEMBL   UCSC 
 
  265
 
  4
 
 20525
 
 tss;island
 
 0.28215
 
 0.51332
 
 Not queried
 
  2.0987470
 
 2.00e-01
 
 1.00e+00
  
 
  
 1
 
 228337181
 
 228337503
 
  PDF   STATS   ENSEMBL   UCSC 
 
  323
 
  5
 
 2296
 
 tss
 
 0.39513
 
 0.60146
 
 Not queried
 
  2.0987398
 
 7.94e-03
 
 1.00e+00
  
 
  
 19
 
  15311886
 
  15312233
 
  PDF   STATS   ENSEMBL   UCSC 
 
  348
 
  5
 
 20520
 
 tss;island
 
 0.15466
 
 0.06644
 
 Not queried
 
  2.0980361
 
 1.51e-01
 
 1.00e+00
  
 
  
 8
 
  25909171
 
  25909599
 
  PDF   STATS   ENSEMBL   UCSC 
 
  429
 
  6
 
 40107
 
 island
 
 0.43021
 
 0.10727
 
 Not queried
 
  2.0958526
 
 8.66e-03
 
 1.00e+00
  
 
  
 12
 
 123636480
 
 123636717
 
  PDF   STATS   ENSEMBL   UCSC 
 
  238
 
  4
 
 10915
 
 island
 
 0.75446
 
 0.94045
 
 Not queried
 
  2.0951530
 
 2.00e-01
 
 1.00e+00
  
 
  
 1
 
 235805403
 
 235805770
 
  PDF   STATS   ENSEMBL   UCSC 
 
  368
 
  4
 
 1088
 
 gene;island
 
 0.43431
 
 0.75094
 
 Not queried
 
  2.0950840
 
 2.86e-02
 
 1.00e+00
  
 
  
 1
 
   6515580
 
   6515748
 
  PDF   STATS   ENSEMBL   UCSC 
 
  169
 
  4
 
 172
 
 gene;island
 
 0.51668
 
 0.84197
 
 Not queried
 
  2.0947384
 
 2.86e-02
 
 1.00e+00
  
 
  
 7
 
 158250129
 
 158250524
 
  PDF   STATS   ENSEMBL   UCSC 
 
  396
 
  4
 
 37309
 
 gene;island
 
 0.70262
 
 0.89599
 
 Not queried
 
  2.0927167
 
 2.86e-02
 
 1.00e+00
  
 
  
 19
 
  17487776
 
  17488116
 
  PDF   STATS   ENSEMBL   UCSC 
 
  341
 
  5
 
 19882
 
 gene
 
 0.77212
 
 0.92530
 
 Not queried
 
  2.0926072
 
 1.59e-02
 
 1.00e+00
  
 
  
 10
 
 120355201
 
 120355427
 
  PDF   STATS   ENSEMBL   UCSC 
 
  227
 
  7
 
 5378
 
 tss
 
 0.20446
 
 0.55113
 
 Not queried
 
  2.0922561
 
 7.28e-02
 
 1.00e+00
  
 
  
 8
 
 144790317
 
 144790410
 
  PDF   STATS   ENSEMBL   UCSC 
 
   94
 
  4
 
 39894
 
 tss
 
 0.68019
 
 0.84893
 
 Not queried
 
  2.0917436
 
 2.86e-02
 
 1.00e+00
  
 
  
 1
 
   3162066
 
   3162173
 
  PDF   STATS   ENSEMBL   UCSC 
 
  108
 
  4
 
 111
 
 gene;island
 
 0.52873
 
 0.88372
 
 Not queried
 
  2.0906232
 
 5.71e-02
 
 1.00e+00
  
 
  
 12
 
  56694168
 
  56694667
 
  PDF   STATS   ENSEMBL   UCSC 
 
  500
 
 10
 
 10506
 
 island
 
 0.02077
 
 0.06639
 
 Not queried
 
  2.0905597
 
 5.24e-02
 
 1.00e+00
  
 
  
 14
 
  21270850
 
  21271355
 
  PDF   STATS   ENSEMBL   UCSC 
 
  506
 
  6
 
 12250
 
 tss
 
 0.83126
 
 0.93758
 
 Not queried
 
  2.0893033
 
 1.52e-02
 
 1.00e+00
  
 
  
 17
 
  46799479
 
  46800028
 
  PDF   STATS   ENSEMBL   UCSC 
 
  550
 
  9
 
 17436
 
 tss;island
 
 0.89281
 
 0.60390
 
 Not queried
 
  2.0887688
 
 1.85e-03
 
 1.00e+00
  
 
  
 11
 
 120080945
 
 120081259
 
  PDF   STATS   ENSEMBL   UCSC 
 
  315
 
  4
 
 7737
 
 tss;island
 
 0.17331
 
 0.04265
 
 Not queried
 
  2.0887487
 
 1.14e-01
 
 1.00e+00
  
 
  
 11
 
 108092087
 
 108092453
 
  PDF   STATS   ENSEMBL   UCSC 
 
  367
 
 10
 
 6866
 
 gene;tss;island
 
 0.04325
 
 0.01879
 
 Not queried
 
  2.0884824
 
 1.90e-01
 
 1.00e+00
  
 
  
 5
 
 140740387
 
 140740607
 
  PDF   STATS   ENSEMBL   UCSC 
 
  221
 
  4
 
 31765
 
 gene;island
 
 0.56006
 
 0.87681
 
 Not queried
 
  2.0883540
 
 2.00e-01
 
 1.00e+00
  
 
  
 4
 
 141264393
 
 141264602
 
  PDF   STATS   ENSEMBL   UCSC 
 
  210
 
  4
 
 30556
 
 tss
 
 0.79424
 
 0.94131
 
 Not queried
 
  2.0877346
 
 2.86e-02
 
 1.00e+00
  
 
  
 14
 
 102680187
 
 102680518
 
  PDF   STATS   ENSEMBL   UCSC 
 
  332
 
  4
 
 12200
 
 gene;island
 
 0.56488
 
 0.82494
 
 Not queried
 
  2.0873657
 
 2.86e-02
 
 1.00e+00
  
 
  
 8
 
  41167660
 
  41168481
 
  PDF   STATS   ENSEMBL   UCSC 
 
  822
 
 12
 
 39604
 
 tss;island
 
 0.78803
 
 0.42533
 
 Not queried
 
  2.0864389
 
 2.74e-04
 
 1.00e+00
  
 
  
 1
 
  41156730
 
  41157266
 
  PDF   STATS   ENSEMBL   UCSC 
 
  537
 
  8
 
 456
 
 gene;tss;island
 
 0.14018
 
 0.02704
 
 Not queried
 
  2.0861698
 
 1.04e-02
 
 1.00e+00
  
 
  
 7
 
  96643454
 
  96643821
 
  PDF   STATS   ENSEMBL   UCSC 
 
  368
 
  5
 
 37669
 
 tss;island
 
 0.07947
 
 0.40436
 
 Not queried
 
  2.0858649
 
 2.22e-01
 
 1.00e+00
  
 
  
 14
 
  75330608
 
  75330798
 
  PDF   STATS   ENSEMBL   UCSC 
 
  191
 
  4
 
 12483
 
 tss
 
 0.76670
 
 0.93318
 
 Not queried
 
  2.0854941
 
 2.86e-02
 
 1.00e+00
  
 
  
 11
 
   3177622
 
   3178322
 
  PDF   STATS   ENSEMBL   UCSC 
 
  701
 
  6
 
 6458
 
 gene;island
 
 0.79037
 
 0.93937
 
 Not queried
 
  2.0843801
 
 8.66e-03
 
 1.00e+00
  
 
  
 10
 
  63213339
 
  63213510
 
  PDF   STATS   ENSEMBL   UCSC 
 
  172
 
  5
 
 5080
 
 tss
 
 0.04116
 
 0.22307
 
 Not queried
 
  2.0843487
 
 9.52e-02
 
 1.00e+00
  
 
  
 17
 
  74381133
 
  74381380
 
  PDF   STATS   ENSEMBL   UCSC 
 
  248
 
  5
 
 16750
 
 gene;island
 
 0.06221
 
 0.18253
 
 Not queried
 
  2.0841537
 
 9.52e-02
 
 1.00e+00
  
 
  
 13
 
  25338113
 
  25338289
 
  PDF   STATS   ENSEMBL   UCSC 
 
  177
 
  6
 
 11319
 
 tss
 
 0.78420
 
 0.95567
 
 Not queried
 
  2.0822015
 
 6.49e-02
 
 1.00e+00
  
 
  
 3
 
   9258385
 
   9258573
 
  PDF   STATS   ENSEMBL   UCSC 
 
  189
 
  5
 
 27609
 
 gene
 
 0.32527
 
 0.90139
 
 Not queried
 
  2.0820885
 
 3.17e-02
 
 1.00e+00
  
 
  
 X
 
  56258900
 
  56259373
 
  PDF   STATS   ENSEMBL   UCSC 
 
  474
 
  6
 
 41003
 
 gene
 
 0.08842
 
 0.02197
 
 Not queried
 
  2.0820171
 
 9.31e-02
 
 1.00e+00
  
 
  
 7
 
   5111488
 
   5111669
 
  PDF   STATS   ENSEMBL   UCSC 
 
  182
 
  8
 
 37355
 
 tss;island
 
 0.05383
 
 0.15506
 
 Not queried
 
  2.0801889
 
 2.07e-02
 
 1.00e+00
  
 
  
 22
 
  39640959
 
  39641154
 
  PDF   STATS   ENSEMBL   UCSC 
 
  196
 
  6
 
 27108
 
 tss
 
 0.05001
 
 0.20745
 
 Not queried
 
  2.0800087
 
 8.66e-03
 
 1.00e+00
  
 
  
 18
 
  71958904
 
  71959795
 
  PDF   STATS   ENSEMBL   UCSC 
 
  892
 
 12
 
 19534
 
 island
 
 0.10561
 
 0.01793
 
 Not queried
 
  2.0796884
 
 4.51e-03
 
 1.00e+00
  
 
  
 15
 
  75470777
 
  75471218
 
  PDF   STATS   ENSEMBL   UCSC 
 
  442
 
  6
 
 14297
 
 island
 
 0.62156
 
 0.29435
 
 Not queried
 
  2.0794780
 
 4.33e-03
 
 1.00e+00
  
 
  
 2
 
  37571677
 
  37571901
 
  PDF   STATS   ENSEMBL   UCSC 
 
  225
 
  6
 
 24185
 
 island
 
 0.03016
 
 0.07086
 
 Not queried
 
  2.0792587
 
 2.60e-02
 
 1.00e+00
  
 
  
 15
 
  95869918
 
  95870475
 
  PDF   STATS   ENSEMBL   UCSC 
 
  558
 
  9
 
 14456
 
 island
 
 0.04171
 
 0.18051
 
 Not queried
 
  2.0785253
 
 2.76e-03
 
 1.00e+00
  
 
  
 7
 
 157366933
 
 157367159
 
  PDF   STATS   ENSEMBL   UCSC 
 
  227
 
  5
 
 37265
 
 gene;island
 
 0.80369
 
 0.94367
 
 Not queried
 
  2.0783919
 
 1.59e-02
 
 1.00e+00
  
 
  
 19
 
  47287778
 
  47288263
 
  PDF   STATS   ENSEMBL   UCSC 
 
  486
 
  7
 
 20078
 
 gene;island
 
 0.28005
 
 0.03852
 
 Not queried
 
  2.0782889
 
 1.75e-02
 
 1.00e+00
  
 
  
 Y
 
  22917782
 
  22917942
 
  PDF   STATS   ENSEMBL   UCSC 
 
  161
 
  5
 
 42174
 
 tss
 
 0.81070
 
 0.54487
 
 Not queried
 
  2.0779321
 
 7.94e-03
 
 1.00e+00
  
 
  
 15
 
  74421739
 
  74421935
 
  PDF   STATS   ENSEMBL   UCSC 
 
  197
 
  7
 
 13387
 
 gene
 
 0.08008
 
 0.02633
 
 Not queried
 
  2.0773142
 
 3.79e-02
 
 1.00e+00
  
 
  
 7
 
   6655711
 
   6655973
 
  PDF   STATS   ENSEMBL   UCSC 
 
  263
 
  4
 
 36824
 
 gene;island
 
 0.38195
 
 0.13045
 
 Not queried
 
  2.0769634
 
 3.43e-01
 
 1.00e+00
  
 
  
 X
 
  34961467
 
  34961658
 
  PDF   STATS   ENSEMBL   UCSC 
 
  192
 
  4
 
 40961
 
 gene;island
 
 0.52421
 
 0.78837
 
 Not queried
 
  2.0768639
 
 2.00e-01
 
 1.00e+00
  
 
  
 8
 
 145162300
 
 145162592
 
  PDF   STATS   ENSEMBL   UCSC 
 
  293
 
  4
 
 39910
 
 tss;island
 
 0.64257
 
 0.88754
 
 Not queried
 
  2.0758414
 
 2.00e-01
 
 1.00e+00
  
 
  
 11
 
 130298544
 
 130298880
 
  PDF   STATS   ENSEMBL   UCSC 
 
  337
 
  8
 
 7797
 
 tss
 
 0.24375
 
 0.04972
 
 Not queried
 
  2.0754116
 
 6.50e-02
 
 1.00e+00
  
 
  
 4
 
  41880280
 
  41880747
 
  PDF   STATS   ENSEMBL   UCSC 
 
  468
 
  6
 
 30956
 
 island
 
 0.85279
 
 0.56694
 
 Not queried
 
  2.0751299
 
 4.33e-03
 
 1.00e+00
  
 
  
 17
 
  14206774
 
  14207036
 
  PDF   STATS   ENSEMBL   UCSC 
 
  263
 
  7
 
 16414
 
 gene;tss;island
 
 0.01389
 
 0.05333
 
 Not queried
 
  2.0741458
 
 1.75e-02
 
 1.00e+00
  
 
  
 8
 
   1051510
 
   1051814
 
  PDF   STATS   ENSEMBL   UCSC 
 
  305
 
  5
 
 39947
 
 island
 
 0.74865
 
 0.89037
 
 Not queried
 
  2.0739155
 
 1.51e-01
 
 1.00e+00
  
 
  
 15
 
  96868857
 
  96869132
 
  PDF   STATS   ENSEMBL   UCSC 
 
  276
 
  7
 
 13889
 
 tss
 
 0.02623
 
 0.13997
 
 Not queried
 
  2.0738314
 
 1.75e-02
 
 1.00e+00
  
 
  
 8
 
 110552297
 
 110552896
 
  PDF   STATS   ENSEMBL   UCSC 
 
  600
 
  6
 
 39335
 
 gene;island
 
 0.12590
 
 0.01550
 
 Not queried
 
  2.0733333
 
 1.32e-01
 
 1.00e+00
  
 
  
 11
 
 130416857
 
 130417168
 
  PDF   STATS   ENSEMBL   UCSC 
 
  312
 
  5
 
 8939
 
 island
 
 0.92275
 
 0.73954
 
 Not queried
 
  2.0726389
 
 3.10e-01
 
 1.00e+00
  
 
  
 12
 
  81110706
 
  81111012
 
  PDF   STATS   ENSEMBL   UCSC 
 
  307
 
  5
 
 10621
 
 island
 
 0.52696
 
 0.76735
 
 Not queried
 
  2.0726046
 
 7.94e-03
 
 1.00e+00
  
 
  
 10
 
  99080594
 
  99081016
 
  PDF   STATS   ENSEMBL   UCSC 
 
  423
 
  4
 
 4679
 
 gene;island
 
 0.27400
 
 0.08659
 
 Not queried
 
  2.0719059
 
 3.43e-01
 
 1.00e+00
  
 
  
 12
 
 132859441
 
 132859951
 
  PDF   STATS   ENSEMBL   UCSC 
 
  511
 
  5
 
 9417
 
 gene;island
 
 0.67001
 
 0.83940
 
 Not queried
 
  2.0717650
 
 9.52e-02
 
 1.00e+00
  
 
  
 X
 
  27825843
 
  27826007
 
  PDF   STATS   ENSEMBL   UCSC 
 
  165
 
  5
 
 41240
 
 tss
 
 0.75923
 
 0.89996
 
 Not queried
 
  2.0715440
 
 5.56e-02
 
 1.00e+00
  
 
  
 5
 
 180646608
 
 180646976
 
  PDF   STATS   ENSEMBL   UCSC 
 
  369
 
  4
 
 33457
 
 island
 
 0.73248
 
 0.39482
 
 Not queried
 
  2.0713995
 
 2.86e-02
 
 1.00e+00
  
 
  
 12
 
  48577720
 
  48578223
 
  PDF   STATS   ENSEMBL   UCSC 
 
  504
 
  4
 
 9086
 
 gene;island
 
 0.03707
 
 0.13868
 
 Not queried
 
  2.0700033
 
 2.00e-01
 
 1.00e+00
  
 
  
 17
 
   7121848
 
   7122261
 
  PDF   STATS   ENSEMBL   UCSC 
 
  414
 
  4
 
 16349
 
 gene;tss;island
 
 0.23995
 
 0.62371
 
 Not queried
 
  2.0696472
 
 5.71e-02
 
 1.00e+00
  
 
  
 6
 
  55039232
 
  55039382
 
  PDF   STATS   ENSEMBL   UCSC 
 
  151
 
  4
 
 34309
 
 gene;island
 
 0.07159
 
 0.26967
 
 Not queried
 
  2.0695007
 
 2.00e-01
 
 1.00e+00
  
 
  
 8
 
  41504078
 
  41504331
 
  PDF   STATS   ENSEMBL   UCSC 
 
  254
 
  4
 
 39225
 
 gene;island
 
 0.01497
 
 0.05728
 
 Not queried
 
  2.0693306
 
 2.86e-02
 
 1.00e+00
  
 
  
 17
 
  21279561
 
  21279874
 
  PDF   STATS   ENSEMBL   UCSC 
 
  314
 
  6
 
 18085
 
 island
 
 0.21345
 
 0.05985
 
 Not queried
 
  2.0691428
 
 1.52e-02
 
 1.00e+00
  
 
  
 4
 
  54965829
 
  54966247
 
  PDF   STATS   ENSEMBL   UCSC 
 
  419
 
  6
 
 30999
 
 island
 
 0.07978
 
 0.05230
 
 Not queried
 
  2.0675948
 
 2.40e-01
 
 1.00e+00
  
 
  
 6
 
  72130209
 
  72130799
 
  PDF   STATS   ENSEMBL   UCSC 
 
  591
 
 12
 
 36181
 
 island
 
 0.02322
 
 0.07069
 
 Not queried
 
  2.0672142
 
 1.00e-02
 
 1.00e+00
  
 
  
 17
 
  74868561
 
  74868725
 
  PDF   STATS   ENSEMBL   UCSC 
 
  165
 
  4
 
 16762
 
 gene;tss;island
 
 0.33492
 
 0.64542
 
 Not queried
 
  2.0671705
 
 2.86e-02
 
 1.00e+00
  
 
  
 16
 
  47007740
 
  47007832
 
  PDF   STATS   ENSEMBL   UCSC 
 
   93
 
  4
 
 15191
 
 tss;island
 
 0.14005
 
 0.02469
 
 Not queried
 
  2.0671023
 
 5.71e-02
 
 1.00e+00
  
 
  
 17
 
  46656543
 
  46656690
 
  PDF   STATS   ENSEMBL   UCSC 
 
  148
 
  4
 
 17427
 
 tss;island
 
 0.14780
 
 0.43123
 
 Not queried
 
  2.0668299
 
 2.00e-01
 
 1.00e+00
  
 
  
 3
 
 126006749
 
 126006903
 
  PDF   STATS   ENSEMBL   UCSC 
 
  155
 
  4
 
 29385
 
 island
 
 0.12654
 
 0.04358
 
 Not queried
 
  2.0664634
 
 4.86e-01
 
 1.00e+00
  
 
  
 17
 
  46827458
 
  46827626
 
  PDF   STATS   ENSEMBL   UCSC 
 
  169
 
  4
 
 18473
 
 island
 
 0.75688
 
 0.36427
 
 Not queried
 
  2.0662443
 
 5.71e-02
 
 1.00e+00
  
 
  
 12
 
 113013048
 
 113013494
 
  PDF   STATS   ENSEMBL   UCSC 
 
  447
 
  5
 
 10794
 
 island
 
 0.53562
 
 0.78704
 
 Not queried
 
  2.0660693
 
 7.94e-03
 
 1.00e+00
  
 
  
 7
 
  55516724
 
  55517132
 
  PDF   STATS   ENSEMBL   UCSC 
 
  409
 
  5
 
 38441
 
 island
 
 0.77221
 
 0.93416
 
 Not queried
 
  2.0654468
 
 7.94e-03
 
 1.00e+00
  
 
  
 19
 
  17877419
 
  17877846
 
  PDF   STATS   ENSEMBL   UCSC 
 
  428
 
  6
 
 19886
 
 gene;island
 
 0.02083
 
 0.13358
 
 Not queried
 
  2.0652201
 
 1.80e-01
 
 1.00e+00
  
 
  
 10
 
 134853084
 
 134853344
 
  PDF   STATS   ENSEMBL   UCSC 
 
  261
 
  4
 
 6309
 
 island
 
 0.56059
 
 0.84588
 
 Not queried
 
  2.0638538
 
 2.00e-01
 
 1.00e+00
  
 
  
 10
 
  79793353
 
  79793501
 
  PDF   STATS   ENSEMBL   UCSC 
 
  149
 
  7
 
 5162
 
 tss
 
 0.02320
 
 0.06280
 
 Not queried
 
  2.0637442
 
 1.65e-01
 
 1.00e+00
  
 
  
 6
 
  29631227
 
  29631447
 
  PDF   STATS   ENSEMBL   UCSC 
 
  221
 
  4
 
 33622
 
 gene
 
 0.53673
 
 0.67756
 
 Not queried
 
  2.0633107
 
 3.43e-01
 
 1.00e+00
  
 
  
 8
 
   9791214
 
   9791557
 
  PDF   STATS   ENSEMBL   UCSC 
 
  344
 
  4
 
 40003
 
 island
 
 0.03902
 
 0.16937
 
 Not queried
 
  2.0621608
 
 2.86e-02
 
 1.00e+00
  
 
  
 3
 
 130368058
 
 130368395
 
  PDF   STATS   ENSEMBL   UCSC 
 
  338
 
  6
 
 27905
 
 gene
 
 0.50772
 
 0.78500
 
 Not queried
 
  2.0621210
 
 1.32e-01
 
 1.00e+00
  
 
  
 3
 
  10547373
 
  10547584
 
  PDF   STATS   ENSEMBL   UCSC 
 
  212
 
  4
 
 28108
 
 tss
 
 0.22541
 
 0.54632
 
 Not queried
 
  2.0619062
 
 3.43e-01
 
 1.00e+00
  
 
  
 12
 
 117319785
 
 117320316
 
  PDF   STATS   ENSEMBL   UCSC 
 
  532
 
  7
 
 10016
 
 tss;island
 
 0.16325
 
 0.03926
 
 Not queried
 
  2.0611043
 
 9.73e-02
 
 1.00e+00
  
 
  
 8
 
  39171866
 
  39172120
 
  PDF   STATS   ENSEMBL   UCSC 
 
  255
 
  7
 
 39599
 
 tss
 
 0.06592
 
 0.25567
 
 Not queried
 
  2.0586940
 
 1.75e-02
 
 1.00e+00
  
 
  
 2
 
 219858132
 
 219858271
 
  PDF   STATS   ENSEMBL   UCSC 
 
  140
 
  6
 
 23849
 
 tss
 
 0.32158
 
 0.07222
 
 Not queried
 
  2.0566108
 
 2.16e-03
 
 1.00e+00
  
 
  
 5
 
 180288429
 
 180288995
 
  PDF   STATS   ENSEMBL   UCSC 
 
  567
 
  5
 
 32504
 
 tss;island
 
 0.24169
 
 0.02355
 
 Not queried
 
  2.0556425
 
 8.41e-01
 
 1.00e+00
  
 
  
 1
 
 167682516
 
 167683013
 
  PDF   STATS   ENSEMBL   UCSC 
 
  498
 
  6
 
 3936
 
 island
 
 0.05915
 
 0.01577
 
 Not queried
 
  2.0548082
 
 8.66e-03
 
 1.00e+00
  
 
  
 3
 
 134083080
 
 134083286
 
  PDF   STATS   ENSEMBL   UCSC 
 
  207
 
  4
 
 27912
 
 gene;island
 
 0.04617
 
 0.17794
 
 Not queried
 
  2.0544845
 
 5.71e-02
 
 1.00e+00
  
 
  
 2
 
  37571677
 
  37571732
 
  PDF   STATS   ENSEMBL   UCSC 
 
   56
 
  5
 
 23268
 
 tss
 
 0.02092
 
 0.06464
 
 Not queried
 
  2.0539932
 
 1.59e-02
 
 1.00e+00
  
 
  
 3
 
  11178593
 
  11178758
 
  PDF   STATS   ENSEMBL   UCSC 
 
  166
 
  5
 
 28112
 
 tss
 
 0.36088
 
 0.62963
 
 Not queried
 
  2.0527737
 
 3.17e-02
 
 1.00e+00
  
 
  
 1
 
  33648648
 
  33648819
 
  PDF   STATS   ENSEMBL   UCSC 
 
  172
 
  4
 
 1467
 
 tss;island
 
 0.44738
 
 0.73981
 
 Not queried
 
  2.0522085
 
 1.14e-01
 
 1.00e+00
  
 
  
 6
 
  32179862
 
  32180305
 
  PDF   STATS   ENSEMBL   UCSC 
 
  444
 
  6
 
 34030
 
 gene
 
 0.82438
 
 0.54202
 
 Not queried
 
  2.0515260
 
 1.32e-01
 
 1.00e+00
  
 
  
 11
 
    537558
 
    537737
 
  PDF   STATS   ENSEMBL   UCSC 
 
  180
 
  4
 
 6360
 
 gene
 
 0.66775
 
 0.24946
 
 Not queried
 
  2.0513894
 
 1.14e-01
 
 1.00e+00
  
 
  
 1
 
  45792557
 
  45792733
 
  PDF   STATS   ENSEMBL   UCSC 
 
  177
 
  5
 
 500
 
 gene
 
 0.62127
 
 0.85807
 
 Not queried
 
  2.0502166
 
 1.59e-02
 
 1.00e+00
  
 
  
 6
 
  30881112
 
  30882384
 
  PDF   STATS   ENSEMBL   UCSC 
 
 1273
 
 39
 
 35678
 
 island
 
 0.06502
 
 0.46325
 
 Not queried
 
  2.0502064
 
 1.01e-03
 
 1.00e+00
  
 
  
 X
 
 139590116
 
 139590479
 
  PDF   STATS   ENSEMBL   UCSC 
 
  364
 
  4
 
 42078
 
 island
 
 0.09268
 
 0.03357
 
 Not queried
 
  2.0490554
 
 4.86e-01
 
 1.00e+00
  
 
  
 2
 
 242927418
 
 242927773
 
  PDF   STATS   ENSEMBL   UCSC 
 
  356
 
  4
 
 25280
 
 island
 
 0.35641
 
 0.69402
 
 Not queried
 
  2.0487110
 
 2.86e-02
 
 1.00e+00
  
 
  
 3
 
  27766009
 
  27766382
 
  PDF   STATS   ENSEMBL   UCSC 
 
  374
 
  6
 
 28910
 
 island
 
 0.49765
 
 0.19825
 
 Not queried
 
  2.0468900
 
 9.31e-02
 
 1.00e+00
  
 
  
 11
 
  69590282
 
  69590580
 
  PDF   STATS   ENSEMBL   UCSC 
 
  299
 
  7
 
 7501
 
 tss
 
 0.09447
 
 0.02927
 
 Not queried
 
  2.0465377
 
 9.73e-02
 
 1.00e+00
  
 
  
 8
 
 142517341
 
 142517553
 
  PDF   STATS   ENSEMBL   UCSC 
 
  213
 
  5
 
 39866
 
 tss
 
 0.46696
 
 0.68113
 
 Not queried
 
  2.0461678
 
 5.56e-02
 
 1.00e+00
  
 
  
 12
 
  40019768
 
  40019919
 
  PDF   STATS   ENSEMBL   UCSC 
 
  152
 
  4
 
 9613
 
 tss
 
 0.63599
 
 0.89483
 
 Not queried
 
  2.0459889
 
 2.86e-02
 
 1.00e+00
  
 
  
 22
 
  50708924
 
  50709221
 
  PDF   STATS   ENSEMBL   UCSC 
 
  298
 
  5
 
 27211
 
 tss;island
 
 0.02030
 
 0.04798
 
 Not queried
 
  2.0450839
 
 7.94e-03
 
 1.00e+00
  
 
  
 11
 
  27743258
 
  27743580
 
  PDF   STATS   ENSEMBL   UCSC 
 
  323
 
  4
 
 6530
 
 gene
 
 0.54421
 
 0.18694
 
 Not queried
 
  2.0442530
 
 3.43e-01
 
 1.00e+00
  
 
  
 1
 
 223888869
 
 223889141
 
  PDF   STATS   ENSEMBL   UCSC 
 
  273
 
  4
 
 2269
 
 tss
 
 0.36176
 
 0.70167
 
 Not queried
 
  2.0440291
 
 2.86e-02
 
 1.00e+00
  
 
  
 17
 
  10560609
 
  10560928
 
  PDF   STATS   ENSEMBL   UCSC 
 
  320
 
  5
 
 17101
 
 tss
 
 0.60541
 
 0.88081
 
 Not queried
 
  2.0426446
 
 7.94e-03
 
 1.00e+00
  
 
  
 17
 
  66508512
 
  66508605
 
  PDF   STATS   ENSEMBL   UCSC 
 
   94
 
  4
 
 16707
 
 gene;island
 
 0.00569
 
 0.02126
 
 Not queried
 
  2.0412343
 
 2.00e-01
 
 1.00e+00
  
 
  
 17
 
   7758397
 
   7758854
 
  PDF   STATS   ENSEMBL   UCSC 
 
  458
 
  7
 
 16382
 
 gene
 
 0.57199
 
 0.84617
 
 Not queried
 
  2.0412168
 
 5.83e-04
 
 1.00e+00
  
 
  
 X
 
  43515213
 
  43515575
 
  PDF   STATS   ENSEMBL   UCSC 
 
  363
 
  7
 
 41730
 
 island
 
 0.15943
 
 0.04716
 
 Not queried
 
  2.0397279
 
 7.28e-02
 
 1.00e+00
  
 
  
 9
 
 139096738
 
 139097029
 
  PDF   STATS   ENSEMBL   UCSC 
 
  292
 
  4
 
 40909
 
 island
 
 0.13621
 
 0.41213
 
 Not queried
 
  2.0383498
 
 3.43e-01
 
 1.00e+00
  
 
  
 12
 
   7341580
 
   7341666
 
  PDF   STATS   ENSEMBL   UCSC 
 
   87
 
  4
 
 9522
 
 tss;island
 
 0.21787
 
 0.03573
 
 Not queried
 
  2.0383132
 
 2.00e-01
 
 1.00e+00
  
 
  
 11
 
  64532839
 
  64533113
 
  PDF   STATS   ENSEMBL   UCSC 
 
  275
 
  4
 
 6676
 
 gene;island
 
 0.80212
 
 0.93980
 
 Not queried
 
  2.0373992
 
 1.14e-01
 
 1.00e+00
  
 
  
 4
 
   3516534
 
   3516758
 
  PDF   STATS   ENSEMBL   UCSC 
 
  225
 
  4
 
 29865
 
 gene;island
 
 0.72943
 
 0.90909
 
 Not queried
 
  2.0368071
 
 1.14e-01
 
 1.00e+00
  
 
  
 1
 
  34632573
 
  34632658
 
  PDF   STATS   ENSEMBL   UCSC 
 
   86
 
  4
 
 1473
 
 tss;island
 
 0.53407
 
 0.22874
 
 Not queried
 
  2.0362067
 
 3.43e-01
 
 1.00e+00
  
 
  
 17
 
  40332731
 
  40333009
 
  PDF   STATS   ENSEMBL   UCSC 
 
  279
 
  4
 
 16542
 
 gene;island
 
 0.05328
 
 0.24997
 
 Not queried
 
  2.0361553
 
 1.14e-01
 
 1.00e+00
  
 
  
 3
 
 148804381
 
 148805294
 
  PDF   STATS   ENSEMBL   UCSC 
 
  914
 
 21
 
 28612
 
 tss
 
 0.94020
 
 0.56346
 
 Not queried
 
  2.0358718
 
 1.46e-02
 
 1.00e+00
  
 
  
 4
 
    995849
 
    996280
 
  PDF   STATS   ENSEMBL   UCSC 
 
  432
 
  5
 
 29811
 
 gene;island
 
 0.73027
 
 0.93271
 
 Not queried
 
  2.0357709
 
 1.59e-02
 
 1.00e+00
  
 
  
 17
 
  47113407
 
  47113596
 
  PDF   STATS   ENSEMBL   UCSC 
 
  190
 
  4
 
 16636
 
 gene;island
 
 0.52601
 
 0.83991
 
 Not queried
 
  2.0352824
 
 1.14e-01
 
 1.00e+00
  
 
  
 8
 
  62200380
 
  62200518
 
  PDF   STATS   ENSEMBL   UCSC 
 
  139
 
  4
 
 39663
 
 tss
 
 0.01809
 
 0.06500
 
 Not queried
 
  2.0340851
 
 2.86e-02
 
 1.00e+00
  
 
  
 7
 
  37955508
 
  37956018
 
  PDF   STATS   ENSEMBL   UCSC 
 
  511
 
  6
 
 36906
 
 gene;island
 
 0.12457
 
 0.39195
 
 Not queried
 
  2.0332043
 
 4.11e-02
 
 1.00e+00
  
 
  
 15
 
  69744390
 
  69745085
 
  PDF   STATS   ENSEMBL   UCSC 
 
  696
 
 12
 
 13736
 
 tss
 
 0.03814
 
 0.01280
 
 Not queried
 
  2.0326342
 
 1.43e-03
 
 1.00e+00
  
 
  
 19
 
  19050971
 
  19051482
 
  PDF   STATS   ENSEMBL   UCSC 
 
  512
 
  8
 
 19909
 
 gene;tss;island
 
 0.27290
 
 0.08326
 
 Not queried
 
  2.0324497
 
 1.04e-02
 
 1.00e+00
  
 
  
 7
 
  77669697
 
  77670191
 
  PDF   STATS   ENSEMBL   UCSC 
 
  495
 
  5
 
 37001
 
 gene;island
 
 0.07148
 
 0.38215
 
 Not queried
 
  2.0321630
 
 5.48e-01
 
 1.00e+00
  
 
  
 12
 
 129337674
 
 129337910
 
  PDF   STATS   ENSEMBL   UCSC 
 
  237
 
  5
 
 10090
 
 tss;island
 
 0.79656
 
 0.48938
 
 Not queried
 
  2.0304244
 
 1.59e-02
 
 1.00e+00
  
 
  
 7
 
  73245439
 
  73245752
 
  PDF   STATS   ENSEMBL   UCSC 
 
  314
 
  4
 
 36979
 
 gene;island
 
 0.54728
 
 0.82555
 
 Not queried
 
  2.0303524
 
 2.86e-02
 
 1.00e+00
  
 
  
 16
 
  56622951
 
  56623215
 
  PDF   STATS   ENSEMBL   UCSC 
 
  265
 
  6
 
 15215
 
 tss
 
 0.50048
 
 0.23544
 
 Not queried
 
  2.0289872
 
 3.10e-01
 
 1.00e+00
  
 
  
 16
 
  72042693
 
  72042925
 
  PDF   STATS   ENSEMBL   UCSC 
 
  233
 
  4
 
 14804
 
 gene
 
 0.01438
 
 0.02784
 
 Not queried
 
  2.0289508
 
 3.43e-01
 
 1.00e+00
  
 
  
 1
 
   6304272
 
   6304499
 
  PDF   STATS   ENSEMBL   UCSC 
 
  228
 
  4
 
 164
 
 gene
 
 0.61521
 
 0.20598
 
 Not queried
 
  2.0271063
 
 1.14e-01
 
 1.00e+00
  
 
  
 7
 
 149411256
 
 149411652
 
  PDF   STATS   ENSEMBL   UCSC 
 
  397
 
  4
 
 37906
 
 tss;island
 
 0.37216
 
 0.13393
 
 Not queried
 
  2.0270356
 
 3.43e-01
 
 1.00e+00
  
 
  
 6
 
  32147440
 
  32147894
 
  PDF   STATS   ENSEMBL   UCSC 
 
  455
 
  8
 
 34012
 
 gene
 
 0.63146
 
 0.87486
 
 Not queried
 
  2.0263951
 
 4.66e-03
 
 1.00e+00
  
 
  
 15
 
  37782885
 
  37783172
 
  PDF   STATS   ENSEMBL   UCSC 
 
  288
 
  6
 
 13988
 
 island
 
 0.38523
 
 0.83156
 
 Not queried
 
  2.0254281
 
 2.16e-03
 
 1.00e+00
  
 
  
 14
 
  38068553
 
  38069206
 
  PDF   STATS   ENSEMBL   UCSC 
 
  654
 
  9
 
 12770
 
 island
 
 0.03982
 
 0.25210
 
 Not queried
 
  2.0249728
 
 1.06e-02
 
 1.00e+00
  
 
  
 3
 
  49449349
 
  49449964
 
  PDF   STATS   ENSEMBL   UCSC 
 
  616
 
 14
 
 28286
 
 tss;island
 
 0.01913
 
 0.05034
 
 Not queried
 
  2.0246448
 
 2.49e-03
 
 1.00e+00
  
 
  
 4
 
   7941764
 
   7942031
 
  PDF   STATS   ENSEMBL   UCSC 
 
  268
 
  8
 
 30247
 
 tss;island
 
 0.10739
 
 0.03424
 
 Not queried
 
  2.0245157
 
 3.28e-01
 
 1.00e+00
  
 
  
 6
 
 108479070
 
 108479584
 
  PDF   STATS   ENSEMBL   UCSC 
 
  515
 
  7
 
 36332
 
 island
 
 0.01349
 
 0.06402
 
 Not queried
 
  2.0240020
 
 2.62e-02
 
 1.00e+00
  
 
  
 12
 
  56843287
 
  56843596
 
  PDF   STATS   ENSEMBL   UCSC 
 
  310
 
  7
 
 9773
 
 tss
 
 0.08834
 
 0.02913
 
 Not queried
 
  2.0238325
 
 3.79e-02
 
 1.00e+00
  
 
  
 19
 
  23941207
 
  23941575
 
  PDF   STATS   ENSEMBL   UCSC 
 
  369
 
  5
 
 19931
 
 gene
 
 0.18208
 
 0.04376
 
 Not queried
 
  2.0236397
 
 7.94e-03
 
 1.00e+00
  
 
  
 17
 
  72772726
 
  72772745
 
  PDF   STATS   ENSEMBL   UCSC 
 
   20
 
  4
 
 16725
 
 gene
 
 0.01120
 
 0.02521
 
 Not queried
 
  2.0233057
 
 4.86e-01
 
 1.00e+00
  
 
  
 1
 
   9129487
 
   9129791
 
  PDF   STATS   ENSEMBL   UCSC 
 
  305
 
  4
 
 198
 
 gene;island
 
 0.77757
 
 0.64820
 
 Not queried
 
  2.0229009
 
 4.86e-01
 
 1.00e+00
  
 
  
 19
 
  55417361
 
  55417500
 
  PDF   STATS   ENSEMBL   UCSC 
 
  140
 
  5
 
 21030
 
 tss
 
 0.54505
 
 0.84329
 
 Not queried
 
  2.0223045
 
 9.52e-02
 
 1.00e+00
  
 
  
 16
 
   3096477
 
   3096711
 
  PDF   STATS   ENSEMBL   UCSC 
 
  235
 
  6
 
 15604
 
 island
 
 0.21683
 
 0.03632
 
 Not queried
 
  2.0220530
 
 9.31e-02
 
 1.00e+00
  
 
  
 19
 
  59066570
 
  59066700
 
  PDF   STATS   ENSEMBL   UCSC 
 
  131
 
  7
 
 21113
 
 tss;island
 
 0.25833
 
 0.63635
 
 Not queried
 
  2.0210842
 
 5.83e-04
 
 1.00e+00
  
 
  
 11
 
   9335544
 
   9335706
 
  PDF   STATS   ENSEMBL   UCSC 
 
  163
 
  4
 
 6485
 
 gene;island
 
 0.01663
 
 0.04044
 
 Not queried
 
  2.0205691
 
 5.71e-02
 
 1.00e+00
  
 
  
 4
 
   7026788
 
   7027124
 
  PDF   STATS   ENSEMBL   UCSC 
 
  337
 
  4
 
 29886
 
 gene;island
 
 0.82404
 
 0.93675
 
 Not queried
 
  2.0188399
 
 2.00e-01
 
 1.00e+00
  
 
  
 21
 
  42880448
 
  42880714
 
  PDF   STATS   ENSEMBL   UCSC 
 
  267
 
  5
 
 26518
 
 tss;island
 
 0.26500
 
 0.14132
 
 Not queried
 
  2.0170918
 
 7.94e-03
 
 1.00e+00
  
 
  
 7
 
 152456579
 
 152456815
 
  PDF   STATS   ENSEMBL   UCSC 
 
  237
 
  5
 
 37943
 
 tss;island
 
 0.02321
 
 0.01698
 
 Not queried
 
  2.0162855
 
 4.21e-01
 
 1.00e+00
  
 
  
 17
 
  41466953
 
  41467212
 
  PDF   STATS   ENSEMBL   UCSC 
 
  260
 
  4
 
 17355
 
 tss
 
 0.01306
 
 0.05795
 
 Not queried
 
  2.0148671
 
 1.14e-01
 
 1.00e+00
  
 
  
 2
 
  37572102
 
  37572423
 
  PDF   STATS   ENSEMBL   UCSC 
 
  322
 
  4
 
 22612
 
 gene;island
 
 0.04122
 
 0.15703
 
 Not queried
 
  2.0143790
 
 1.14e-01
 
 1.00e+00
  
 
  
 2
 
  11796786
 
  11797017
 
  PDF   STATS   ENSEMBL   UCSC 
 
  232
 
  4
 
 24043
 
 island
 
 0.43716
 
 0.75793
 
 Not queried
 
  2.0142552
 
 2.86e-02
 
 1.00e+00
  
 
  
 8
 
 144790187
 
 144790410
 
  PDF   STATS   ENSEMBL   UCSC 
 
  224
 
  5
 
 40616
 
 island
 
 0.69550
 
 0.90693
 
 Not queried
 
  2.0133497
 
 7.94e-03
 
 1.00e+00
  
 
  
 8
 
  99076526
 
  99076734
 
  PDF   STATS   ENSEMBL   UCSC 
 
  209
 
  4
 
 39770
 
 tss
 
 0.43349
 
 0.09346
 
 Not queried
 
  2.0126272
 
 3.43e-01
 
 1.00e+00
  
 
  
 X
 
  55187543
 
  55188047
 
  PDF   STATS   ENSEMBL   UCSC 
 
  505
 
  9
 
 41341
 
 tss
 
 0.19007
 
 0.02851
 
 Not queried
 
  2.0120834
 
 4.00e-02
 
 1.00e+00
  
 
  
 20
 
  61885236
 
  61885291
 
  PDF   STATS   ENSEMBL   UCSC 
 
   56
 
  6
 
 25853
 
 tss
 
 0.00731
 
 0.01221
 
 Not queried
 
  2.0108701
 
 3.94e-01
 
 1.00e+00
  
 
  
 7
 
 102087552
 
 102087746
 
  PDF   STATS   ENSEMBL   UCSC 
 
  195
 
  4
 
 37104
 
 gene
 
 0.62762
 
 0.92454
 
 Not queried
 
  2.0103854
 
 1.14e-01
 
 1.00e+00
  
 
  
 1
 
  14027378
 
  14027581
 
  PDF   STATS   ENSEMBL   UCSC 
 
  204
 
  4
 
 235
 
 gene;island
 
 0.27298
 
 0.04418
 
 Not queried
 
  2.0096306
 
 1.14e-01
 
 1.00e+00
  
 
  
 19
 
  18496984
 
  18497201
 
  PDF   STATS   ENSEMBL   UCSC 
 
  218
 
  4
 
 19899
 
 gene
 
 0.69161
 
 0.82073
 
 Not queried
 
  2.0093348
 
 3.43e-01
 
 1.00e+00
  
 
  
 1
 
 166039444
 
 166039918
 
  PDF   STATS   ENSEMBL   UCSC 
 
  475
 
  5
 
 865
 
 gene
 
 0.20031
 
 0.39340
 
 Not queried
 
  2.0091795
 
 9.52e-02
 
 1.00e+00
  
 
  
 14
 
  77647955
 
  77648040
 
  PDF   STATS   ENSEMBL   UCSC 
 
   86
 
  5
 
 12500
 
 tss
 
 0.05473
 
 0.08149
 
 Not queried
 
  2.0088896
 
 5.56e-02
 
 1.00e+00
  
 
  
 12
 
   6166874
 
   6167147
 
  PDF   STATS   ENSEMBL   UCSC 
 
  274
 
  4
 
 8997
 
 gene;island
 
 0.58630
 
 0.25804
 
 Not queried
 
  2.0085446
 
 2.86e-02
 
 1.00e+00
  
 
  
 13
 
  25874859
 
  25875646
 
  PDF   STATS   ENSEMBL   UCSC 
 
  788
 
 10
 
 11323
 
 tss
 
 0.03092
 
 0.39757
 
 Not queried
 
  2.0083646
 
 1.90e-01
 
 1.00e+00
  
 
  
 11
 
    518649
 
    519271
 
  PDF   STATS   ENSEMBL   UCSC 
 
  623
 
  5
 
 7836
 
 island
 
 0.26875
 
 0.60034
 
 Not queried
 
  2.0081924
 
 4.21e-01
 
 1.00e+00
  
 
  
 17
 
  42385942
 
  42386419
 
  PDF   STATS   ENSEMBL   UCSC 
 
  478
 
  7
 
 16578
 
 gene
 
 0.49570
 
 0.15191
 
 Not queried
 
  2.0080192
 
 1.28e-01
 
 1.00e+00
  
 
  
 15
 
  90455635
 
  90455799
 
  PDF   STATS   ENSEMBL   UCSC 
 
  165
 
  4
 
 13446
 
 gene;island
 
 0.01347
 
 0.03667
 
 Not queried
 
  2.0079406
 
 5.71e-02
 
 1.00e+00
  
 
  
 10
 
 102731747
 
 102732323
 
  PDF   STATS   ENSEMBL   UCSC 
 
  577
 
  8
 
 6021
 
 island
 
 0.65137
 
 0.80292
 
 Not queried
 
  2.0079397
 
 8.30e-02
 
 1.00e+00
  
 
  
 22
 
  27053195
 
  27053470
 
  PDF   STATS   ENSEMBL   UCSC 
 
  276
 
 10
 
 26995
 
 tss
 
 0.08837
 
 0.03023
 
 Not queried
 
  2.0063839
 
 6.84e-03
 
 1.00e+00
  
 
  
 8
 
 144798309
 
 144798477
 
  PDF   STATS   ENSEMBL   UCSC 
 
  169
 
  7
 
 39895
 
 tss
 
 0.01875
 
 0.06145
 
 Not queried
 
  2.0056947
 
 5.30e-02
 
 1.00e+00
  
 
  
 17
 
  20687681
 
  20687912
 
  PDF   STATS   ENSEMBL   UCSC 
 
  232
 
  4
 
 18077
 
 island
 
 0.31823
 
 0.57819
 
 Not queried
 
  2.0028683
 
 2.00e-01
 
 1.00e+00
  
 
  
 X
 
 114423840
 
 114424082
 
  PDF   STATS   ENSEMBL   UCSC 
 
  243
 
  4
 
 41084
 
 gene;island
 
 0.55783
 
 0.87836
 
 Not queried
 
  2.0027089
 
 2.00e-01
 
 1.00e+00
  
 
  
 11
 
   2153991
 
   2154432
 
  PDF   STATS   ENSEMBL   UCSC 
 
  442
 
  6
 
 6427
 
 gene;island
 
 0.23433
 
 0.56969
 
 Not queried
 
  2.0013409
 
 2.60e-02
 
 1.00e+00
  
 
  
 5
 
 141488016
 
 141488153
 
  PDF   STATS   ENSEMBL   UCSC 
 
  138
 
  5
 
 32336
 
 tss;island
 
 0.20461
 
 0.06038
 
 Not queried
 
  2.0013016
 
 5.48e-01
 
 1.00e+00
  
 
  
 18
 
   5196738
 
   5197071
 
  PDF   STATS   ENSEMBL   UCSC 
 
  334
 
  4
 
 19013
 
 gene;island
 
 0.03632
 
 0.05779
 
 Not queried
 
  2.0008407
 
 4.86e-01
 
 1.00e+00
  
 
  
 7
 
 150026554
 
 150026909
 
  PDF   STATS   ENSEMBL   UCSC 
 
  356
 
  4
 
 37912
 
 tss
 
 0.72456
 
 0.75619
 
 Not queried
 
  2.0002074
 
 6.86e-01
 
 1.00e+00
  
 
  
 1
 
 174968089
 
 174968509
 
  PDF   STATS   ENSEMBL   UCSC 
 
  421
 
  9
 
 2098
 
 tss;island
 
 0.07763
 
 0.02559
 
 Not queried
 
  2.0001263
 
 1.42e-02
 
 1.00e+00
  
 
  
 13
 
 108922032
 
 108922504
 
  PDF   STATS   ENSEMBL   UCSC 
 
  473
 
  7
 
 11195
 
 gene
 
 0.22226
 
 0.58397
 
 Not queried
 
  1.9996096
 
 1.28e-01
 
 1.00e+00
  
 
  
 2
 
  74010290
 
  74010621
 
  PDF   STATS   ENSEMBL   UCSC 
 
  332
 
  4
 
 23407
 
 tss;island
 
 0.51303
 
 0.80804
 
 Not queried
 
  1.9991126
 
 2.86e-02
 
 1.00e+00
  
 
  
 11
 
  10472001
 
  10472161
 
  PDF   STATS   ENSEMBL   UCSC 
 
  161
 
  7
 
 7124
 
 tss;island
 
 0.14060
 
 0.05369
 
 Not queried
 
  1.9990248
 
 1.75e-02
 
 1.00e+00
  
 
  
 2
 
  23840537
 
  23840763
 
  PDF   STATS   ENSEMBL   UCSC 
 
  227
 
  4
 
 22557
 
 gene
 
 0.65274
 
 0.89873
 
 Not queried
 
  1.9984463
 
 1.14e-01
 
 1.00e+00
  
 
  
 6
 
  33043072
 
  33043220
 
  PDF   STATS   ENSEMBL   UCSC 
 
  149
 
  4
 
 34824
 
 tss
 
 0.82728
 
 0.63522
 
 Not queried
 
  1.9983122
 
 6.86e-01
 
 1.00e+00
  
 
  
 4
 
   7395388
 
   7395612
 
  PDF   STATS   ENSEMBL   UCSC 
 
  225
 
  4
 
 29891
 
 gene
 
 0.72963
 
 0.89499
 
 Not queried
 
  1.9979669
 
 2.00e-01
 
 1.00e+00
  
 
  
 21
 
  34398085
 
  34398265
 
  PDF   STATS   ENSEMBL   UCSC 
 
  181
 
  8
 
 26601
 
 island
 
 0.01371
 
 0.07794
 
 Not queried
 
  1.9975695
 
 1.86e-03
 
 1.00e+00
  
 
  
 8
 
  63776508
 
  63776762
 
  PDF   STATS   ENSEMBL   UCSC 
 
  255
 
  4
 
 39256
 
 gene
 
 0.46741
 
 0.81984
 
 Not queried
 
  1.9973938
 
 2.00e-01
 
 1.00e+00
  
 
  
 2
 
 144994885
 
 144995206
 
  PDF   STATS   ENSEMBL   UCSC 
 
  322
 
  4
 
 22851
 
 gene
 
 0.82114
 
 0.94898
 
 Not queried
 
  1.9973535
 
 2.86e-02
 
 1.00e+00
  
 
  
 7
 
  31726494
 
  31726704
 
  PDF   STATS   ENSEMBL   UCSC 
 
  211
 
  5
 
 37458
 
 tss
 
 0.92095
 
 0.96451
 
 Not queried
 
  1.9969725
 
 9.52e-02
 
 1.00e+00
  
 
  
 11
 
  94276895
 
  94276960
 
  PDF   STATS   ENSEMBL   UCSC 
 
   66
 
  4
 
 7609
 
 tss
 
 0.18942
 
 0.05438
 
 Not queried
 
  1.9967538
 
 2.86e-02
 
 1.00e+00
  
 
  
 19
 
  17905589
 
  17905835
 
  PDF   STATS   ENSEMBL   UCSC 
 
  247
 
  5
 
 20564
 
 tss;island
 
 0.55068
 
 0.09054
 
 Not queried
 
  1.9959567
 
 3.17e-02
 
 1.00e+00
  
 
  
 12
 
 106533840
 
 106533903
 
  PDF   STATS   ENSEMBL   UCSC 
 
   64
 
  5
 
 9941
 
 tss
 
 0.72111
 
 0.39330
 
 Not queried
 
  1.9959059
 
 3.17e-02
 
 1.00e+00
  
 
  
 7
 
    149629
 
    150084
 
  PDF   STATS   ENSEMBL   UCSC 
 
  456
 
  5
 
 37965
 
 island
 
 0.54094
 
 0.82452
 
 Not queried
 
  1.9955053
 
 5.48e-01
 
 1.00e+00
  
 
  
 15
 
  22646692
 
  22647092
 
  PDF   STATS   ENSEMBL   UCSC 
 
  401
 
  4
 
 13910
 
 island
 
 0.27936
 
 0.60039
 
 Not queried
 
  1.9954818
 
 2.86e-02
 
 1.00e+00
  
 
  
 3
 
 123167507
 
 123167770
 
  PDF   STATS   ENSEMBL   UCSC 
 
  264
 
  4
 
 28502
 
 tss;island
 
 0.23021
 
 0.05209
 
 Not queried
 
  1.9945916
 
 3.43e-01
 
 1.00e+00
  
 
  
 12
 
  54385275
 
  54385625
 
  PDF   STATS   ENSEMBL   UCSC 
 
  351
 
  5
 
 10460
 
 island
 
 0.68784
 
 0.31672
 
 Not queried
 
  1.9942431
 
 7.94e-03
 
 1.00e+00
  
 
  
 18
 
  19997912
 
  19998049
 
  PDF   STATS   ENSEMBL   UCSC 
 
  138
 
  7
 
 19167
 
 tss
 
 0.93075
 
 0.75124
 
 Not queried
 
  1.9941475
 
 5.30e-02
 
 1.00e+00
  
 
  
 17
 
  61615504
 
  61615913
 
  PDF   STATS   ENSEMBL   UCSC 
 
  410
 
  4
 
 16693
 
 gene;island
 
 0.10035
 
 0.30355
 
 Not queried
 
  1.9914601
 
 2.86e-02
 
 1.00e+00
  
 
  
 3
 
 170136159
 
 170136539
 
  PDF   STATS   ENSEMBL   UCSC 
 
  381
 
  9
 
 28671
 
 tss;island
 
 0.09736
 
 0.12113
 
 Not queried
 
  1.9913588
 
 1.61e-01
 
 1.00e+00
  
 
  
 X
 
   9734033
 
   9734312
 
  PDF   STATS   ENSEMBL   UCSC 
 
  280
 
  4
 
 41179
 
 tss
 
 0.76937
 
 0.93448
 
 Not queried
 
  1.9906725
 
 4.86e-01
 
 1.00e+00
  
 
  
 8
 
 144120335
 
 144120648
 
  PDF   STATS   ENSEMBL   UCSC 
 
  314
 
  5
 
 39879
 
 tss
 
 0.58223
 
 0.25967
 
 Not queried
 
  1.9903798
 
 3.17e-02
 
 1.00e+00
  
 
  
 1
 
  19181040
 
  19181459
 
  PDF   STATS   ENSEMBL   UCSC 
 
  420
 
  6
 
 275
 
 gene;island
 
 0.46056
 
 0.72383
 
 Not queried
 
  1.9893714
 
 2.16e-03
 
 1.00e+00
  
 
  
 1
 
  67772896
 
  67773044
 
  PDF   STATS   ENSEMBL   UCSC 
 
  149
 
  5
 
 1680
 
 tss
 
 0.29897
 
 0.07589
 
 Not queried
 
  1.9887422
 
 2.22e-01
 
 1.00e+00
  
 
  
 10
 
 134893401
 
 134894048
 
  PDF   STATS   ENSEMBL   UCSC 
 
  648
 
  8
 
 6312
 
 island
 
 0.56840
 
 0.79816
 
 Not queried
 
  1.9885697
 
 1.05e-01
 
 1.00e+00
  
 
  
 19
 
  51141227
 
  51141413
 
  PDF   STATS   ENSEMBL   UCSC 
 
  187
 
  4
 
 20143
 
 gene;island
 
 0.48268
 
 0.19120
 
 Not queried
 
  1.9885059
 
 2.00e-01
 
 1.00e+00
  
 
  
 16
 
   1384626
 
   1384908
 
  PDF   STATS   ENSEMBL   UCSC 
 
  283
 
  5
 
 15498
 
 island
 
 0.33397
 
 0.11221
 
 Not queried
 
  1.9880676
 
 5.48e-01
 
 1.00e+00
  
 
  
 3
 
  11034279
 
  11034345
 
  PDF   STATS   ENSEMBL   UCSC 
 
   67
 
  4
 
 28111
 
 tss
 
 0.06797
 
 0.38356
 
 Not queried
 
  1.9880040
 
 5.71e-02
 
 1.00e+00
  
 
  
 7
 
  63774078
 
  63774504
 
  PDF   STATS   ENSEMBL   UCSC 
 
  427
 
  5
 
 36960
 
 gene
 
 0.08073
 
 0.02338
 
 Not queried
 
  1.9870302
 
 1.51e-01
 
 1.00e+00
  
 
  
 6
 
  29079939
 
  29080437
 
  PDF   STATS   ENSEMBL   UCSC 
 
  499
 
  5
 
 33587
 
 gene
 
 0.21207
 
 0.56391
 
 Not queried
 
  1.9866165
 
 1.59e-02
 
 1.00e+00
  
 
  
 14
 
 102554826
 
 102554977
 
  PDF   STATS   ENSEMBL   UCSC 
 
  152
 
  4
 
 12198
 
 gene;tss;island
 
 0.51401
 
 0.19835
 
 Not queried
 
  1.9865415
 
 2.86e-02
 
 1.00e+00
  
 
  
 7
 
  28220498
 
  28220645
 
  PDF   STATS   ENSEMBL   UCSC 
 
  148
 
  8
 
 37438
 
 tss;island
 
 0.06718
 
 0.02077
 
 Not queried
 
  1.9864222
 
 2.07e-02
 
 1.00e+00
  
 
  
 17
 
  40489513
 
  40489785
 
  PDF   STATS   ENSEMBL   UCSC 
 
  273
 
  5
 
 16544
 
 gene
 
 0.87118
 
 0.96402
 
 Not queried
 
  1.9863704
 
 5.56e-02
 
 1.00e+00
  
 
  
 22
 
  39640835
 
  39641154
 
  PDF   STATS   ENSEMBL   UCSC 
 
  320
 
  7
 
 27458
 
 island
 
 0.05689
 
 0.21533
 
 Not queried
 
  1.9861295
 
 2.33e-03
 
 1.00e+00
  
 
  
 19
 
  16830613
 
  16830749
 
  PDF   STATS   ENSEMBL   UCSC 
 
  137
 
  4
 
 20539
 
 tss
 
 0.72030
 
 0.91113
 
 Not queried
 
  1.9851389
 
 1.14e-01
 
 1.00e+00
  
 
  
 17
 
  33700180
 
  33700295
 
  PDF   STATS   ENSEMBL   UCSC 
 
  116
 
  4
 
 16484
 
 gene;island
 
 0.04125
 
 0.14508
 
 Not queried
 
  1.9850910
 
 1.14e-01
 
 1.00e+00
  
 
  
 6
 
 117584596
 
 117584815
 
  PDF   STATS   ENSEMBL   UCSC 
 
  220
 
  4
 
 36373
 
 island
 
 0.28111
 
 0.66262
 
 Not queried
 
  1.9842370
 
 2.86e-02
 
 1.00e+00
  
 
  
 16
 
  88569083
 
  88569490
 
  PDF   STATS   ENSEMBL   UCSC 
 
  408
 
  5
 
 14875
 
 gene;island
 
 0.63132
 
 0.79750
 
 Not queried
 
  1.9839968
 
 5.56e-02
 
 1.00e+00
  
 
  
 X
 
 117629034
 
 117629344
 
  PDF   STATS   ENSEMBL   UCSC 
 
  311
 
  5
 
 41481
 
 tss;island
 
 0.26691
 
 0.06216
 
 Not queried
 
  1.9837803
 
 2.22e-01
 
 1.00e+00
  
 
  
 X
 
  54556330
 
  54556538
 
  PDF   STATS   ENSEMBL   UCSC 
 
  209
 
  7
 
 41335
 
 tss
 
 0.09541
 
 0.03030
 
 Not queried
 
  1.9829867
 
 9.73e-02
 
 1.00e+00
  
 
  
 2
 
 176968584
 
 176968920
 
  PDF   STATS   ENSEMBL   UCSC 
 
  337
 
  4
 
 24856
 
 island
 
 0.27907
 
 0.08451
 
 Not queried
 
  1.9813995
 
 2.00e-01
 
 1.00e+00
  
 
  
 1
 
  60392140
 
  60392567
 
  PDF   STATS   ENSEMBL   UCSC 
 
  428
 
  6
 
 3319
 
 island
 
 0.17384
 
 0.04793
 
 Not queried
 
  1.9811833
 
 4.33e-03
 
 1.00e+00
  
 
  
 16
 
   3085571
 
   3085982
 
  PDF   STATS   ENSEMBL   UCSC 
 
  412
 
  4
 
 15017
 
 tss
 
 0.64462
 
 0.50122
 
 Not queried
 
  1.9807218
 
 6.86e-01
 
 1.00e+00
  
 
  
 1
 
  25566331
 
  25566753
 
  PDF   STATS   ENSEMBL   UCSC 
 
  423
 
  5
 
 2862
 
 island
 
 0.02082
 
 0.07743
 
 Not queried
 
  1.9807064
 
 2.22e-01
 
 1.00e+00
  
 
  
 X
 
 105969622
 
 105969840
 
  PDF   STATS   ENSEMBL   UCSC 
 
  219
 
  7
 
 41456
 
 tss
 
 0.01976
 
 0.04789
 
 Not queried
 
  1.9793135
 
 2.62e-02
 
 1.00e+00
  
 
  
 16
 
  66586309
 
  66586415
 
  PDF   STATS   ENSEMBL   UCSC 
 
  107
 
  5
 
 15249
 
 tss
 
 0.11458
 
 0.02559
 
 Not queried
 
  1.9790988
 
 7.94e-03
 
 1.00e+00
  
 
  
 16
 
    433439
 
    433825
 
  PDF   STATS   ENSEMBL   UCSC 
 
  387
 
  5
 
 14501
 
 gene;island
 
 0.02255
 
 0.04813
 
 Not queried
 
  1.9789737
 
 9.52e-02
 
 1.00e+00
  
 
  
 1
 
   2125049
 
   2125243
 
  PDF   STATS   ENSEMBL   UCSC 
 
  195
 
  7
 
 69
 
 gene;tss;island
 
 0.84648
 
 0.58093
 
 Not queried
 
  1.9788522
 
 5.83e-04
 
 1.00e+00
  
 
  
 16
 
  85060822
 
  85061228
 
  PDF   STATS   ENSEMBL   UCSC 
 
  407
 
  5
 
 15365
 
 tss;island
 
 0.04410
 
 0.05776
 
 Not queried
 
  1.9787968
 
 5.48e-01
 
 1.00e+00
  
 
  
 1
 
  19665070
 
  19665240
 
  PDF   STATS   ENSEMBL   UCSC 
 
  171
 
  4
 
 2776
 
 island
 
 0.65706
 
 0.38805
 
 Not queried
 
  1.9765962
 
 5.71e-02
 
 1.00e+00
  
 
  
 19
 
  49685846
 
  49686254
 
  PDF   STATS   ENSEMBL   UCSC 
 
  409
 
  5
 
 20109
 
 gene;island
 
 0.93601
 
 0.72926
 
 Not queried
 
  1.9765768
 
 9.52e-02
 
 1.00e+00
  
 
  
 4
 
 102712010
 
 102712397
 
  PDF   STATS   ENSEMBL   UCSC 
 
  388
 
  4
 
 30025
 
 gene;island
 
 0.18771
 
 0.05804
 
 Not queried
 
  1.9760673
 
 1.14e-01
 
 1.00e+00
  
 
  
 7
 
 100612725
 
 100612891
 
  PDF   STATS   ENSEMBL   UCSC 
 
  167
 
  4
 
 37731
 
 tss
 
 0.31408
 
 0.61765
 
 Not queried
 
  1.9747638
 
 2.86e-02
 
 1.00e+00
  
 
  
 14
 
  29247148
 
  29247705
 
  PDF   STATS   ENSEMBL   UCSC 
 
  558
 
  6
 
 12025
 
 gene;island
 
 0.13173
 
 0.38734
 
 Not queried
 
  1.9747630
 
 9.31e-02
 
 1.00e+00
  
 
  
 19
 
  12997997
 
  12998135
 
  PDF   STATS   ENSEMBL   UCSC 
 
  139
 
  4
 
 21596
 
 island
 
 0.55527
 
 0.24207
 
 Not queried
 
  1.9745259
 
 2.00e-01
 
 1.00e+00
  
 
  
 11
 
   1873757
 
   1874093
 
  PDF   STATS   ENSEMBL   UCSC 
 
  337
 
  5
 
 7029
 
 tss
 
 0.50598
 
 0.85365
 
 Not queried
 
  1.9743440
 
 1.51e-01
 
 1.00e+00
  
 
  
 1
 
 201252200
 
 201252510
 
  PDF   STATS   ENSEMBL   UCSC 
 
  311
 
  6
 
 2161
 
 tss
 
 0.41867
 
 0.76729
 
 Not queried
 
  1.9742233
 
 2.16e-03
 
 1.00e+00
  
 
  
 1
 
 201252200
 
 201252734
 
  PDF   STATS   ENSEMBL   UCSC 
 
  535
 
  8
 
 4061
 
 island
 
 0.42575
 
 0.76729
 
 Not queried
 
  1.9742233
 
 1.86e-03
 
 1.00e+00
  
 
  
 7
 
  44104860
 
  44105166
 
  PDF   STATS   ENSEMBL   UCSC 
 
  307
 
  4
 
 36926
 
 gene
 
 0.15016
 
 0.40278
 
 Not queried
 
  1.9734908
 
 5.71e-02
 
 1.00e+00
  
 
  
 2
 
 219925065
 
 219925330
 
  PDF   STATS   ENSEMBL   UCSC 
 
  266
 
  7
 
 25067
 
 island
 
 0.03174
 
 0.11764
 
 Not queried
 
  1.9727857
 
 7.28e-02
 
 1.00e+00
  
 
  
 18
 
  12253749
 
  12254690
 
  PDF   STATS   ENSEMBL   UCSC 
 
  942
 
 19
 
 19367
 
 island
 
 0.34224
 
 0.64101
 
 Not queried
 
  1.9727101
 
 2.83e-04
 
 1.00e+00
  
 
  
 2
 
  27665017
 
  27665150
 
  PDF   STATS   ENSEMBL   UCSC 
 
  134
 
  6
 
 23232
 
 tss
 
 0.28833
 
 0.64586
 
 Not queried
 
  1.9723942
 
 2.16e-03
 
 1.00e+00
  
 
  
 19
 
    708469
 
    708709
 
  PDF   STATS   ENSEMBL   UCSC 
 
  241
 
  5
 
 20241
 
 tss;island
 
 0.28174
 
 0.11088
 
 Not queried
 
  1.9720900
 
 3.10e-01
 
 1.00e+00
  
 
  
 11
 
  31839521
 
  31839947
 
  PDF   STATS   ENSEMBL   UCSC 
 
  427
 
  7
 
 7204
 
 tss
 
 0.44793
 
 0.09224
 
 Not queried
 
  1.9709403
 
 7.28e-02
 
 1.00e+00
  
 
  
 11
 
  31839314
 
  31839947
 
  PDF   STATS   ENSEMBL   UCSC 
 
  634
 
  9
 
 8146
 
 island
 
 0.22810
 
 0.07325
 
 Not queried
 
  1.9709403
 
 5.03e-02
 
 1.00e+00
  
 
  
 16
 
  31227272
 
  31227515
 
  PDF   STATS   ENSEMBL   UCSC 
 
  244
 
  4
 
 14704
 
 gene;island
 
 0.02986
 
 0.11451
 
 Not queried
 
  1.9706602
 
 1.14e-01
 
 1.00e+00
  
 
  
 2
 
  97193006
 
  97193289
 
  PDF   STATS   ENSEMBL   UCSC 
 
  284
 
  4
 
 24489
 
 island
 
 0.46890
 
 0.19486
 
 Not queried
 
  1.9705061
 
 2.86e-02
 
 1.00e+00
  
 
  
 20
 
  23030343
 
  23030533
 
  PDF   STATS   ENSEMBL   UCSC 
 
  191
 
  5
 
 25597
 
 tss
 
 0.02140
 
 0.08844
 
 Not queried
 
  1.9691737
 
 7.94e-03
 
 1.00e+00
  
 
  
 20
 
  23030102
 
  23030533
 
  PDF   STATS   ENSEMBL   UCSC 
 
  432
 
  7
 
 26008
 
 island
 
 0.02140
 
 0.11655
 
 Not queried
 
  1.9691737
 
 1.17e-03
 
 1.00e+00
  
 
  
 3
 
  54121761
 
  54122146
 
  PDF   STATS   ENSEMBL   UCSC 
 
  386
 
  6
 
 29166
 
 island
 
 0.54103
 
 0.14454
 
 Not queried
 
  1.9691696
 
 2.60e-02
 
 1.00e+00
  
 
  
 X
 
  70150722
 
  70151209
 
  PDF   STATS   ENSEMBL   UCSC 
 
  488
 
  7
 
 41853
 
 island
 
 0.07502
 
 0.02074
 
 Not queried
 
  1.9684376
 
 2.33e-03
 
 1.00e+00
  
 
  
 16
 
   1080388
 
   1080808
 
  PDF   STATS   ENSEMBL   UCSC 
 
  421
 
  4
 
 15478
 
 island
 
 0.57451
 
 0.82243
 
 Not queried
 
  1.9666614
 
 2.86e-02
 
 1.00e+00
  
 
  
 5
 
  45695467
 
  45695922
 
  PDF   STATS   ENSEMBL   UCSC 
 
  456
 
  4
 
 31551
 
 gene;island
 
 0.05346
 
 0.30343
 
 Not queried
 
  1.9663400
 
 5.71e-02
 
 1.00e+00
  
 
  
 17
 
   6796745
 
   6797119
 
  PDF   STATS   ENSEMBL   UCSC 
 
  375
 
  5
 
 16342
 
 gene;island
 
 0.74166
 
 0.42363
 
 Not queried
 
  1.9656713
 
 1.59e-02
 
 1.00e+00
  
 
  
 16
 
    967929
 
    968265
 
  PDF   STATS   ENSEMBL   UCSC 
 
  337
 
  6
 
 14525
 
 gene;island
 
 0.63832
 
 0.89034
 
 Not queried
 
  1.9654574
 
 9.31e-02
 
 1.00e+00
  
 
  
 X
 
 153665624
 
 153665799
 
  PDF   STATS   ENSEMBL   UCSC 
 
  176
 
  4
 
 41160
 
 gene;island
 
 0.00477
 
 0.01324
 
 Not queried
 
  1.9648042
 
 2.86e-02
 
 1.00e+00
  
 
  
 14
 
  60973773
 
  60974039
 
  PDF   STATS   ENSEMBL   UCSC 
 
  267
 
  4
 
 12875
 
 island
 
 0.17533
 
 0.04190
 
 Not queried
 
  1.9621014
 
 2.86e-02
 
 1.00e+00
  
 
  
 19
 
   5822318
 
   5822630
 
  PDF   STATS   ENSEMBL   UCSC 
 
  313
 
  4
 
 20347
 
 tss;island
 
 0.30783
 
 0.69944
 
 Not queried
 
  1.9614997
 
 1.14e-01
 
 1.00e+00
  
 
  
 6
 
  29623646
 
  29623992
 
  PDF   STATS   ENSEMBL   UCSC 
 
  347
 
  4
 
 34687
 
 tss
 
 0.57157
 
 0.83217
 
 Not queried
 
  1.9612542
 
 2.00e-01
 
 1.00e+00
  
 
  
 16
 
  29801602
 
  29801967
 
  PDF   STATS   ENSEMBL   UCSC 
 
  366
 
  7
 
 15137
 
 tss;island
 
 0.02615
 
 0.01691
 
 Not queried
 
  1.9609537
 
 6.20e-01
 
 1.00e+00
  
 
  
 4
 
    987303
 
    987672
 
  PDF   STATS   ENSEMBL   UCSC 
 
  370
 
  5
 
 30187
 
 tss
 
 0.72886
 
 0.84815
 
 Not queried
 
  1.9596671
 
 1.59e-02
 
 1.00e+00
  
 
  
 6
 
  32814921
 
  32815091
 
  PDF   STATS   ENSEMBL   UCSC 
 
  171
 
  6
 
 34088
 
 gene;island
 
 0.70706
 
 0.28341
 
 Not queried
 
  1.9585732
 
 6.49e-02
 
 1.00e+00
  
 
  
 1
 
  46599002
 
  46599128
 
  PDF   STATS   ENSEMBL   UCSC 
 
  127
 
  4
 
 1591
 
 tss;island
 
 0.00500
 
 0.01602
 
 Not queried
 
  1.9584158
 
 4.86e-01
 
 1.00e+00
  
 
  
 1
 
 202858691
 
 202858826
 
  PDF   STATS   ENSEMBL   UCSC 
 
  136
 
  4
 
 2180
 
 tss;island
 
 0.52456
 
 0.88745
 
 Not queried
 
  1.9572164
 
 2.00e-01
 
 1.00e+00
  
 
  
 2
 
 119609429
 
 119609839
 
  PDF   STATS   ENSEMBL   UCSC 
 
  411
 
  6
 
 24612
 
 island
 
 0.69443
 
 0.53167
 
 Not queried
 
  1.9565471
 
 2.16e-03
 
 1.00e+00
  
 
  
 17
 
  72667005
 
  72667394
 
  PDF   STATS   ENSEMBL   UCSC 
 
  390
 
  5
 
 18686
 
 island
 
 0.08222
 
 0.26595
 
 Not queried
 
  1.9552974
 
 1.59e-02
 
 1.00e+00
  
 
  
 X
 
 102318831
 
 102319120
 
  PDF   STATS   ENSEMBL   UCSC 
 
  290
 
  6
 
 41057
 
 gene;island
 
 0.23835
 
 0.09403
 
 Not queried
 
  1.9543561
 
 4.33e-03
 
 1.00e+00
  
 
  
 20
 
  23015899
 
  23015936
 
  PDF   STATS   ENSEMBL   UCSC 
 
   38
 
  5
 
 25596
 
 tss;island
 
 0.53241
 
 0.80482
 
 Not queried
 
  1.9532846
 
 1.59e-02
 
 1.00e+00
  
 
  
 12
 
  14518655
 
  14518974
 
  PDF   STATS   ENSEMBL   UCSC 
 
  320
 
  4
 
 9039
 
 gene
 
 0.07614
 
 0.20898
 
 Not queried
 
  1.9532086
 
 5.71e-02
 
 1.00e+00
  
 
  
 10
 
 131682251
 
 131682509
 
  PDF   STATS   ENSEMBL   UCSC 
 
  259
 
  5
 
 4818
 
 gene
 
 0.70944
 
 0.92314
 
 Not queried
 
  1.9520863
 
 3.17e-02
 
 1.00e+00
  
 
  
 8
 
  20107455
 
  20107758
 
  PDF   STATS   ENSEMBL   UCSC 
 
  304
 
  4
 
 39159
 
 gene;island
 
 0.66733
 
 0.88570
 
 Not queried
 
  1.9513442
 
 2.00e-01
 
 1.00e+00
  
 
  
 10
 
  98480463
 
  98480474
 
  PDF   STATS   ENSEMBL   UCSC 
 
   12
 
  4
 
 5238
 
 tss;island
 
 0.06841
 
 0.02792
 
 Not queried
 
  1.9513341
 
 2.00e-01
 
 1.00e+00
  
 
  
 12
 
  54867429
 
  54867653
 
  PDF   STATS   ENSEMBL   UCSC 
 
  225
 
  4
 
 9739
 
 tss
 
 0.85885
 
 0.61852
 
 Not queried
 
  1.9510982
 
 3.43e-01
 
 1.00e+00
  
 
  
 1
 
  24645802
 
  24646392
 
  PDF   STATS   ENSEMBL   UCSC 
 
  591
 
 13
 
 2852
 
 island
 
 0.02458
 
 0.09434
 
 Not queried
 
  1.9507339
 
 2.97e-04
 
 1.00e+00
  
 
  
 17
 
  38521067
 
  38521482
 
  PDF   STATS   ENSEMBL   UCSC 
 
  416
 
  4
 
 17299
 
 tss;island
 
 0.92156
 
 0.87123
 
 Not queried
 
  1.9506425
 
 2.00e-01
 
 1.00e+00
  
 
  
 11
 
  62457265
 
  62457345
 
  PDF   STATS   ENSEMBL   UCSC 
 
   81
 
  5
 
 7337
 
 tss;island
 
 0.61760
 
 0.88085
 
 Not queried
 
  1.9493505
 
 7.94e-03
 
 1.00e+00
  
 
  
 9
 
   1042605
 
   1042970
 
  PDF   STATS   ENSEMBL   UCSC 
 
  366
 
  4
 
 40796
 
 island
 
 0.13201
 
 0.22750
 
 Not queried
 
  1.9485111
 
 2.00e-01
 
 1.00e+00
  
 
  
 17
 
  54230419
 
  54230793
 
  PDF   STATS   ENSEMBL   UCSC 
 
  375
 
  5
 
 17487
 
 tss
 
 0.15208
 
 0.40894
 
 Not queried
 
  1.9477176
 
 3.17e-02
 
 1.00e+00
  
 
  
 8
 
   1109986
 
   1110263
 
  PDF   STATS   ENSEMBL   UCSC 
 
  278
 
  4
 
 39951
 
 island
 
 0.60887
 
 0.86960
 
 Not queried
 
  1.9463632
 
 5.71e-02
 
 1.00e+00
  
 
  
 16
 
  84681507
 
  84681729
 
  PDF   STATS   ENSEMBL   UCSC 
 
  223
 
  4
 
 15363
 
 tss;island
 
 0.32908
 
 0.08259
 
 Not queried
 
  1.9463555
 
 3.43e-01
 
 1.00e+00
  
 
  
 10
 
 120355139
 
 120355427
 
  PDF   STATS   ENSEMBL   UCSC 
 
  289
 
  8
 
 6169
 
 island
 
 0.22134
 
 0.51548
 
 Not queried
 
  1.9463036
 
 3.79e-02
 
 1.00e+00
  
 
  
 17
 
   6899522
 
   6899888
 
  PDF   STATS   ENSEMBL   UCSC 
 
  367
 
  5
 
 16344
 
 gene
 
 0.57090
 
 0.69158
 
 Not queried
 
  1.9450599
 
 1.00e+00
 
 1.00e+00
  
 
  
 6
 
 169951905
 
 169952299
 
  PDF   STATS   ENSEMBL   UCSC 
 
  395
 
  4
 
 34504
 
 gene
 
 0.87827
 
 0.95475
 
 Not queried
 
  1.9439197
 
 2.86e-02
 
 1.00e+00
  
 
  
 15
 
  89954560
 
  89955171
 
  PDF   STATS   ENSEMBL   UCSC 
 
  612
 
  6
 
 14406
 
 island
 
 0.16737
 
 0.33559
 
 Not queried
 
  1.9428616
 
 3.10e-01
 
 1.00e+00
  
 
  
 10
 
 119292062
 
 119292587
 
  PDF   STATS   ENSEMBL   UCSC 
 
  526
 
  8
 
 4765
 
 gene;island
 
 0.24397
 
 0.56896
 
 Not queried
 
  1.9427143
 
 2.07e-02
 
 1.00e+00
  
 
  
 17
 
   1617291
 
   1617465
 
  PDF   STATS   ENSEMBL   UCSC 
 
  175
 
  5
 
 16960
 
 tss
 
 0.06030
 
 0.14728
 
 Not queried
 
  1.9423213
 
 2.22e-01
 
 1.00e+00
  
 
  
 6
 
 170584106
 
 170584501
 
  PDF   STATS   ENSEMBL   UCSC 
 
  396
 
  4
 
 36625
 
 island
 
 0.92654
 
 0.78651
 
 Not queried
 
  1.9419336
 
 2.86e-02
 
 1.00e+00
  
 
  
 1
 
  34090664
 
  34090941
 
  PDF   STATS   ENSEMBL   UCSC 
 
  278
 
  5
 
 404
 
 gene;island
 
 0.13364
 
 0.32374
 
 Not queried
 
  1.9417376
 
 2.22e-01
 
 1.00e+00
  
 
  
 6
 
  32371488
 
  32371538
 
  PDF   STATS   ENSEMBL   UCSC 
 
   51
 
  4
 
 34047
 
 gene
 
 0.17467
 
 0.43151
 
 Not queried
 
  1.9414746
 
 2.00e-01
 
 1.00e+00
  
 
  
 19
 
  21541756
 
  21541917
 
  PDF   STATS   ENSEMBL   UCSC 
 
  162
 
  5
 
 19924
 
 gene
 
 0.39902
 
 0.12793
 
 Not queried
 
  1.9414141
 
 5.56e-02
 
 1.00e+00
  
 
  
 1
 
  26737005
 
  26737262
 
  PDF   STATS   ENSEMBL   UCSC 
 
  258
 
  5
 
 1388
 
 tss
 
 0.08495
 
 0.17777
 
 Not queried
 
  1.9395352
 
 3.17e-02
 
 1.00e+00
  
 
  
 19
 
  55458886
 
  55459282
 
  PDF   STATS   ENSEMBL   UCSC 
 
  397
 
  6
 
 21031
 
 tss
 
 0.79199
 
 0.92569
 
 Not queried
 
  1.9390777
 
 6.49e-02
 
 1.00e+00
  
 
  
 13
 
  40746370
 
  40746648
 
  PDF   STATS   ENSEMBL   UCSC 
 
  279
 
  4
 
 11656
 
 island
 
 0.32533
 
 0.60832
 
 Not queried
 
  1.9376568
 
 3.43e-01
 
 1.00e+00
  
 
  
 3
 
  49947639
 
  49947934
 
  PDF   STATS   ENSEMBL   UCSC 
 
  296
 
  4
 
 27750
 
 gene;island
 
 0.96277
 
 0.74025
 
 Not queried
 
  1.9374092
 
 1.14e-01
 
 1.00e+00
  
 
  
 5
 
   3326343
 
   3326731
 
  PDF   STATS   ENSEMBL   UCSC 
 
  389
 
  5
 
 32602
 
 island
 
 0.63473
 
 0.87099
 
 Not queried
 
  1.9363789
 
 9.52e-02
 
 1.00e+00
  
 
  
 14
 
  96967798
 
  96968050
 
  PDF   STATS   ENSEMBL   UCSC 
 
  253
 
  4
 
 12567
 
 tss;island
 
 0.04243
 
 0.01027
 
 Not queried
 
  1.9361836
 
 2.00e-01
 
 1.00e+00
  
 
  
 6
 
  32084298
 
  32084658
 
  PDF   STATS   ENSEMBL   UCSC 
 
  361
 
  6
 
 33989
 
 gene
 
 0.71158
 
 0.89119
 
 Not queried
 
  1.9355090
 
 2.60e-02
 
 1.00e+00
  
 
  
 22
 
  19712623
 
  19712750
 
  PDF   STATS   ENSEMBL   UCSC 
 
  128
 
  4
 
 27260
 
 island
 
 0.41419
 
 0.75831
 
 Not queried
 
  1.9354671
 
 3.43e-01
 
 1.00e+00
  
 
  
 8
 
 102504447
 
 102504859
 
  PDF   STATS   ENSEMBL   UCSC 
 
  413
 
  9
 
 40441
 
 island
 
 0.02110
 
 0.13104
 
 Not queried
 
  1.9353525
 
 1.06e-02
 
 1.00e+00
  
 
  
 12
 
  81110749
 
  81111012
 
  PDF   STATS   ENSEMBL   UCSC 
 
  264
 
  4
 
 9201
 
 gene
 
 0.56909
 
 0.77323
 
 Not queried
 
  1.9352414
 
 2.86e-02
 
 1.00e+00
  
 
  
 10
 
 133796164
 
 133796559
 
  PDF   STATS   ENSEMBL   UCSC 
 
  396
 
  6
 
 5426
 
 tss;island
 
 0.67394
 
 0.35091
 
 Not queried
 
  1.9352195
 
 3.94e-01
 
 1.00e+00
  
 
  
 14
 
  24457893
 
  24458014
 
  PDF   STATS   ENSEMBL   UCSC 
 
  122
 
  6
 
 12280
 
 tss
 
 0.03227
 
 0.11988
 
 Not queried
 
  1.9351345
 
 1.80e-01
 
 1.00e+00
  
 
  
 13
 
  36050788
 
  36051073
 
  PDF   STATS   ENSEMBL   UCSC 
 
  286
 
  8
 
 11099
 
 gene;island
 
 0.03085
 
 0.13153
 
 Not queried
 
  1.9343936
 
 1.30e-01
 
 1.00e+00
  
 
  
 17
 
  30669342
 
  30669494
 
  PDF   STATS   ENSEMBL   UCSC 
 
  153
 
  4
 
 17221
 
 tss
 
 0.24416
 
 0.07646
 
 Not queried
 
  1.9333656
 
 4.86e-01
 
 1.00e+00
  
 
  
 13
 
  50702675
 
  50703026
 
  PDF   STATS   ENSEMBL   UCSC 
 
  352
 
  6
 
 11713
 
 island
 
 0.32262
 
 0.15615
 
 Not queried
 
  1.9324681
 
 2.60e-02
 
 1.00e+00
  
 
  
 8
 
  41583018
 
  41583523
 
  PDF   STATS   ENSEMBL   UCSC 
 
  506
 
  5
 
 39229
 
 gene;island
 
 0.44761
 
 0.76805
 
 Not queried
 
  1.9316541
 
 5.56e-02
 
 1.00e+00
  
 
  
 13
 
  21900392
 
  21900591
 
  PDF   STATS   ENSEMBL   UCSC 
 
  200
 
  4
 
 11560
 
 island
 
 0.20943
 
 0.06353
 
 Not queried
 
  1.9313319
 
 1.14e-01
 
 1.00e+00
  
 
  
 4
 
  57522689
 
  57523055
 
  PDF   STATS   ENSEMBL   UCSC 
 
  367
 
  7
 
 30364
 
 tss
 
 0.61849
 
 0.16764
 
 Not queried
 
  1.9312118
 
 7.28e-02
 
 1.00e+00
  
 
  
 11
 
  64058878
 
  64059057
 
  PDF   STATS   ENSEMBL   UCSC 
 
  180
 
  4
 
 6659
 
 gene
 
 0.35318
 
 0.19562
 
 Not queried
 
  1.9310928
 
 3.43e-01
 
 1.00e+00
  
 
  
 11
 
  64058550
 
  64059057
 
  PDF   STATS   ENSEMBL   UCSC 
 
  508
 
  6
 
 8379
 
 island
 
 0.55960
 
 0.27755
 
 Not queried
 
  1.9310928
 
 1.80e-01
 
 1.00e+00
  
 
  
 5
 
 112849156
 
 112849285
 
  PDF   STATS   ENSEMBL   UCSC 
 
  130
 
  4
 
 32161
 
 tss
 
 0.19271
 
 0.02691
 
 Not queried
 
  1.9303854
 
 4.86e-01
 
 1.00e+00
  
 
  
 1
 
  18972919
 
  18973303
 
  PDF   STATS   ENSEMBL   UCSC 
 
  385
 
  5
 
 274
 
 gene;island
 
 0.35787
 
 0.05496
 
 Not queried
 
  1.9299185
 
 3.17e-02
 
 1.00e+00
  
 
  
 8
 
  28748202
 
  28748679
 
  PDF   STATS   ENSEMBL   UCSC 
 
  478
 
  5
 
 39203
 
 gene;tss;island
 
 0.04385
 
 0.12405
 
 Not queried
 
  1.9295313
 
 3.10e-01
 
 1.00e+00
  
 
  
 4
 
  16362340
 
  16362666
 
  PDF   STATS   ENSEMBL   UCSC 
 
  327
 
  5
 
 30892
 
 island
 
 0.64433
 
 0.87343
 
 Not queried
 
  1.9295129
 
 9.52e-02
 
 1.00e+00
  
 
  
 11
 
   7041752
 
   7041987
 
  PDF   STATS   ENSEMBL   UCSC 
 
  236
 
  6
 
 6477
 
 gene
 
 0.04852
 
 0.02087
 
 Not queried
 
  1.9284852
 
 4.11e-02
 
 1.00e+00
  
 
  
 17
 
  80189962
 
  80190136
 
  PDF   STATS   ENSEMBL   UCSC 
 
  175
 
  6
 
 17737
 
 tss
 
 0.27368
 
 0.55145
 
 Not queried
 
  1.9284715
 
 2.16e-03
 
 1.00e+00
  
 
  
 3
 
  49727025
 
  49727474
 
  PDF   STATS   ENSEMBL   UCSC 
 
  450
 
  5
 
 27745
 
 gene
 
 0.02374
 
 0.10392
 
 Not queried
 
  1.9276124
 
 1.51e-01
 
 1.00e+00
  
 
  
 X
 
 147133598
 
 147133830
 
  PDF   STATS   ENSEMBL   UCSC 
 
  233
 
  4
 
 42087
 
 island
 
 0.37411
 
 0.67283
 
 Not queried
 
  1.9270408
 
 2.86e-02
 
 1.00e+00
  
 
  
 8
 
 102504447
 
 102504666
 
  PDF   STATS   ENSEMBL   UCSC 
 
  220
 
  7
 
 39786
 
 tss
 
 0.02110
 
 0.13104
 
 Not queried
 
  1.9270187
 
 7.28e-02
 
 1.00e+00
  
 
  
 8
 
  97345492
 
  97346037
 
  PDF   STATS   ENSEMBL   UCSC 
 
  546
 
  6
 
 39305
 
 gene
 
 0.51341
 
 0.73744
 
 Not queried
 
  1.9269965
 
 2.40e-01
 
 1.00e+00
  
 
  
 12
 
 133656595
 
 133656953
 
  PDF   STATS   ENSEMBL   UCSC 
 
  359
 
  7
 
 10112
 
 tss;island
 
 0.02469
 
 0.02974
 
 Not queried
 
  1.9265318
 
 1.28e-01
 
 1.00e+00
  
 
  
 X
 
  38080219
 
  38080608
 
  PDF   STATS   ENSEMBL   UCSC 
 
  390
 
  7
 
 41256
 
 tss
 
 0.03769
 
 0.04460
 
 Not queried
 
  1.9263307
 
 6.20e-01
 
 1.00e+00
  
 
  
 17
 
  80189962
 
  80190154
 
  PDF   STATS   ENSEMBL   UCSC 
 
  193
 
  7
 
 16907
 
 gene;island
 
 0.27570
 
 0.58624
 
 Not queried
 
  1.9254862
 
 5.83e-04
 
 1.00e+00
  
 
  
 3
 
 141087187
 
 141087363
 
  PDF   STATS   ENSEMBL   UCSC 
 
  177
 
  5
 
 27931
 
 gene
 
 0.57704
 
 0.85231
 
 Not queried
 
  1.9252013
 
 5.56e-02
 
 1.00e+00
  
 
  
 11
 
  68609518
 
  68609747
 
  PDF   STATS   ENSEMBL   UCSC 
 
  230
 
  6
 
 7491
 
 tss;island
 
 0.08997
 
 0.02159
 
 Not queried
 
  1.9246019
 
 2.40e-01
 
 1.00e+00
  
 
  
 6
 
 111279493
 
 111279703
 
  PDF   STATS   ENSEMBL   UCSC 
 
  211
 
  9
 
 35109
 
 tss
 
 0.14355
 
 0.04043
 
 Not queried
 
  1.9244845
 
 1.88e-02
 
 1.00e+00
  
 
  
 20
 
  45313109
 
  45313390
 
  PDF   STATS   ENSEMBL   UCSC 
 
  282
 
  6
 
 25402
 
 gene;tss
 
 0.51339
 
 0.80125
 
 Not queried
 
  1.9239105
 
 2.60e-02
 
 1.00e+00
  
 
  
 4
 
  88343439
 
  88343678
 
  PDF   STATS   ENSEMBL   UCSC 
 
  240
 
  4
 
 30441
 
 tss
 
 0.12825
 
 0.04558
 
 Not queried
 
  1.9232528
 
 4.86e-01
 
 1.00e+00
  
 
  
 17
 
  73055792
 
  73056187
 
  PDF   STATS   ENSEMBL   UCSC 
 
  396
 
  6
 
 16727
 
 gene
 
 0.72301
 
 0.92590
 
 Not queried
 
  1.9219249
 
 9.31e-02
 
 1.00e+00
  
 
  
 10
 
 131989161
 
 131989849
 
  PDF   STATS   ENSEMBL   UCSC 
 
  689
 
  8
 
 6255
 
 island
 
 0.89697
 
 0.63009
 
 Not queried
 
  1.9214554
 
 4.99e-02
 
 1.00e+00
  
 
  
 6
 
 150039400
 
 150039767
 
  PDF   STATS   ENSEMBL   UCSC 
 
  368
 
  8
 
 35222
 
 tss
 
 0.01368
 
 0.11429
 
 Not queried
 
  1.9213814
 
 1.86e-03
 
 1.00e+00
  
 
  
 4
 
    987108
 
    987672
 
  PDF   STATS   ENSEMBL   UCSC 
 
  565
 
  6
 
 29810
 
 gene;island
 
 0.75424
 
 0.88043
 
 Not queried
 
  1.9204697
 
 4.33e-03
 
 1.00e+00
  
 
  
 3
 
 170303540
 
 170303830
 
  PDF   STATS   ENSEMBL   UCSC 
 
  291
 
  4
 
 27987
 
 gene
 
 0.25966
 
 0.09127
 
 Not queried
 
  1.9203782
 
 1.14e-01
 
 1.00e+00
  
 
  
 19
 
  49939549
 
  49940062
 
  PDF   STATS   ENSEMBL   UCSC 
 
  514
 
  6
 
 20113
 
 gene;island
 
 0.72171
 
 0.23482
 
 Not queried
 
  1.9198195
 
 4.11e-02
 
 1.00e+00
  
 
  
 19
 
  57337294
 
  57337648
 
  PDF   STATS   ENSEMBL   UCSC 
 
  355
 
  4
 
 20202
 
 gene;tss
 
 0.86116
 
 0.94385
 
 Not queried
 
  1.9189631
 
 2.86e-02
 
 1.00e+00
  
 
  
 1
 
 156390102
 
 156390240
 
  PDF   STATS   ENSEMBL   UCSC 
 
  139
 
  5
 
 821
 
 gene;island
 
 0.20470
 
 0.50573
 
 Not queried
 
  1.9189416
 
 5.56e-02
 
 1.00e+00
  
 
  
 19
 
  43969650
 
  43970034
 
  PDF   STATS   ENSEMBL   UCSC 
 
  385
 
  6
 
 22048
 
 island
 
 0.14736
 
 0.07838
 
 Not queried
 
  1.9184294
 
 6.49e-02
 
 1.00e+00
  
 
  
 X
 
 130192035
 
 130192160
 
  PDF   STATS   ENSEMBL   UCSC 
 
  126
 
  5
 
 41521
 
 tss
 
 0.24381
 
 0.08078
 
 Not queried
 
  1.9183149
 
 5.56e-02
 
 1.00e+00
  
 
  
 17
 
   7555132
 
   7555411
 
  PDF   STATS   ENSEMBL   UCSC 
 
  280
 
  4
 
 16372
 
 gene;island
 
 0.24101
 
 0.07128
 
 Not queried
 
  1.9172996
 
 2.00e-01
 
 1.00e+00
  
 
  
 17
 
   1665119
 
   1665220
 
  PDF   STATS   ENSEMBL   UCSC 
 
  102
 
  5
 
 16963
 
 tss
 
 0.33131
 
 0.66283
 
 Not queried
 
  1.9171478
 
 7.94e-03
 
 1.00e+00
  
 
  
 1
 
   2776972
 
   2777244
 
  PDF   STATS   ENSEMBL   UCSC 
 
  273
 
  4
 
 2514
 
 island
 
 0.49265
 
 0.74555
 
 Not queried
 
  1.9159032
 
 2.86e-02
 
 1.00e+00
  
 
  
 2
 
  85839322
 
  85839722
 
  PDF   STATS   ENSEMBL   UCSC 
 
  401
 
  5
 
 23448
 
 tss
 
 0.33280
 
 0.11288
 
 Not queried
 
  1.9157156
 
 3.10e-01
 
 1.00e+00
  
 
  
 12
 
  23998921
 
  23999045
 
  PDF   STATS   ENSEMBL   UCSC 
 
  125
 
  4
 
 9057
 
 gene
 
 0.59642
 
 0.86156
 
 Not queried
 
  1.9156495
 
 2.00e-01
 
 1.00e+00
  
 
  
 20
 
  31261784
 
  31261994
 
  PDF   STATS   ENSEMBL   UCSC 
 
  211
 
  4
 
 25636
 
 tss
 
 0.20742
 
 0.49632
 
 Not queried
 
  1.9156406
 
 1.14e-01
 
 1.00e+00
  
 
  
 2
 
 158453600
 
 158454401
 
  PDF   STATS   ENSEMBL   UCSC 
 
  802
 
  9
 
 22866
 
 gene;island
 
 0.03077
 
 0.16180
 
 Not queried
 
  1.9152829
 
 2.44e-02
 
 1.00e+00
  
 
  
 7
 
    645309
 
    645796
 
  PDF   STATS   ENSEMBL   UCSC 
 
  488
 
  6
 
 36653
 
 gene;island
 
 0.88136
 
 0.94743
 
 Not queried
 
  1.9127853
 
 4.11e-02
 
 1.00e+00
  
 
  
 17
 
  35290307
 
  35291580
 
  PDF   STATS   ENSEMBL   UCSC 
 
 1274
 
 12
 
 18202
 
 island
 
 0.54870
 
 0.29256
 
 Not queried
 
  1.9126234
 
 3.64e-03
 
 1.00e+00
  
 
  
 6
 
  74160917
 
  74161222
 
  PDF   STATS   ENSEMBL   UCSC 
 
  306
 
  4
 
 34324
 
 gene;island
 
 0.17926
 
 0.39586
 
 Not queried
 
  1.9125825
 
 2.00e-01
 
 1.00e+00
  
 
  
 7
 
 151330008
 
 151330230
 
  PDF   STATS   ENSEMBL   UCSC 
 
  223
 
  4
 
 37232
 
 gene;tss
 
 0.38233
 
 0.16719
 
 Not queried
 
  1.9118176
 
 2.86e-02
 
 1.00e+00
  
 
  
 5
 
  78407418
 
  78407566
 
  PDF   STATS   ENSEMBL   UCSC 
 
  149
 
  6
 
 32086
 
 tss
 
 0.11702
 
 0.28443
 
 Not queried
 
  1.9116324
 
 1.32e-01
 
 1.00e+00
  
 
  
 1
 
  26394201
 
  26394593
 
  PDF   STATS   ENSEMBL   UCSC 
 
  393
 
  5
 
 1378
 
 tss
 
 0.92953
 
 0.66679
 
 Not queried
 
  1.9109981
 
 9.52e-02
 
 1.00e+00
  
 
  
 1
 
   6341140
 
   6341327
 
  PDF   STATS   ENSEMBL   UCSC 
 
  188
 
  4
 
 166
 
 gene;island
 
 0.76179
 
 0.89144
 
 Not queried
 
  1.9103836
 
 1.14e-01
 
 1.00e+00
  
 
  
 11
 
  69285633
 
  69286352
 
  PDF   STATS   ENSEMBL   UCSC 
 
  720
 
  8
 
 8567
 
 island
 
 0.54408
 
 0.82148
 
 Not queried
 
  1.9097549
 
 1.04e-02
 
 1.00e+00
  
 
  
 5
 
 161494274
 
 161494645
 
  PDF   STATS   ENSEMBL   UCSC 
 
  372
 
  6
 
 32414
 
 tss
 
 0.24908
 
 0.50389
 
 Not queried
 
  1.9095366
 
 2.16e-03
 
 1.00e+00
  
 
  
 12
 
  14996508
 
  14996776
 
  PDF   STATS   ENSEMBL   UCSC 
 
  269
 
  8
 
 9563
 
 tss
 
 0.63979
 
 0.87597
 
 Not queried
 
  1.9086202
 
 6.99e-03
 
 1.00e+00
  
 
  
 7
 
 155174991
 
 155175340
 
  PDF   STATS   ENSEMBL   UCSC 
 
  350
 
  4
 
 39003
 
 island
 
 0.95622
 
 0.86157
 
 Not queried
 
  1.9086097
 
 2.00e-01
 
 1.00e+00
  
 
  
 17
 
  77924180
 
  77924371
 
  PDF   STATS   ENSEMBL   UCSC 
 
  192
 
  4
 
 16811
 
 gene;island
 
 0.39059
 
 0.16480
 
 Not queried
 
  1.9085562
 
 2.00e-01
 
 1.00e+00
  
 
  
 17
 
  46651722
 
  46651952
 
  PDF   STATS   ENSEMBL   UCSC 
 
  231
 
  7
 
 18445
 
 island
 
 0.38312
 
 0.14196
 
 Not queried
 
  1.9084070
 
 7.28e-02
 
 1.00e+00
  
 
  
 10
 
    119786
 
    120151
 
  PDF   STATS   ENSEMBL   UCSC 
 
  366
 
  4
 
 5449
 
 island
 
 0.01911
 
 0.10737
 
 Not queried
 
  1.9083916
 
 1.14e-01
 
 1.00e+00
  
 
  
 10
 
 125853676
 
 125853893
 
  PDF   STATS   ENSEMBL   UCSC 
 
  218
 
  4
 
 6206
 
 island
 
 0.09608
 
 0.20169
 
 Not queried
 
  1.9081029
 
 3.43e-01
 
 1.00e+00
  
 
  
 1
 
  47082037
 
  47082211
 
  PDF   STATS   ENSEMBL   UCSC 
 
  175
 
  4
 
 1599
 
 tss
 
 0.21611
 
 0.06250
 
 Not queried
 
  1.9080699
 
 2.00e-01
 
 1.00e+00
  
 
  
 21
 
  43299789
 
  43300024
 
  PDF   STATS   ENSEMBL   UCSC 
 
  236
 
  4
 
 26520
 
 tss
 
 0.09661
 
 0.03074
 
 Not queried
 
  1.9076341
 
 3.43e-01
 
 1.00e+00
  
 
  
 7
 
  45808644
 
  45808808
 
  PDF   STATS   ENSEMBL   UCSC 
 
  165
 
  5
 
 37520
 
 tss
 
 0.03806
 
 0.06695
 
 Not queried
 
  1.9064715
 
 2.22e-01
 
 1.00e+00
  
 
  
 6
 
  30231639
 
  30232015
 
  PDF   STATS   ENSEMBL   UCSC 
 
  377
 
  5
 
 33692
 
 gene;island
 
 0.29366
 
 0.64839
 
 Not queried
 
  1.9052549
 
 3.17e-02
 
 1.00e+00
  
 
  
 X
 
 129245188
 
 129245245
 
  PDF   STATS   ENSEMBL   UCSC 
 
   58
 
  4
 
 41515
 
 tss;island
 
 0.34628
 
 0.07547
 
 Not queried
 
  1.9042471
 
 1.14e-01
 
 1.00e+00
  
 
  
 12
 
  75601711
 
  75601928
 
  PDF   STATS   ENSEMBL   UCSC 
 
  218
 
  4
 
 9194
 
 gene;island
 
 0.10415
 
 0.33561
 
 Not queried
 
  1.9036234
 
 2.00e-01
 
 1.00e+00
  
 
  
 18
 
  24130267
 
  24130633
 
  PDF   STATS   ENSEMBL   UCSC 
 
  367
 
  4
 
 19048
 
 gene;tss;island
 
 0.22822
 
 0.08642
 
 Not queried
 
  1.9032253
 
 2.00e-01
 
 1.00e+00
  
 
  
 17
 
  78078605
 
  78079086
 
  PDF   STATS   ENSEMBL   UCSC 
 
  482
 
  5
 
 16820
 
 gene;island
 
 0.61216
 
 0.90649
 
 Not queried
 
  1.9029256
 
 1.59e-02
 
 1.00e+00
  
 
  
 3
 
 158390329
 
 158390821
 
  PDF   STATS   ENSEMBL   UCSC 
 
  493
 
 10
 
 27969
 
 gene
 
 0.06823
 
 0.28286
 
 Not queried
 
  1.9026570
 
 2.80e-01
 
 1.00e+00
  
 
  
 2
 
 162283434
 
 162284206
 
  PDF   STATS   ENSEMBL   UCSC 
 
  773
 
  8
 
 24785
 
 island
 
 0.66951
 
 0.90033
 
 Not queried
 
  1.9025597
 
 6.50e-02
 
 1.00e+00
  
 
  
 2
 
 124782117
 
 124782263
 
  PDF   STATS   ENSEMBL   UCSC 
 
  147
 
  4
 
 23568
 
 tss;island
 
 0.05178
 
 0.18672
 
 Not queried
 
  1.9022421
 
 4.86e-01
 
 1.00e+00
  
 
  
 17
 
   7757933
 
   7758291
 
  PDF   STATS   ENSEMBL   UCSC 
 
  359
 
 10
 
 17066
 
 tss
 
 0.55735
 
 0.89390
 
 Not queried
 
  1.9016802
 
 8.93e-03
 
 1.00e+00
  
 
  
 3
 
 111717534
 
 111717969
 
  PDF   STATS   ENSEMBL   UCSC 
 
  436
 
  7
 
 28455
 
 tss
 
 0.04922
 
 0.02380
 
 Not queried
 
  1.9015979
 
 7.28e-02
 
 1.00e+00
  
 
  
 17
 
  66194137
 
  66194797
 
  PDF   STATS   ENSEMBL   UCSC 
 
  661
 
  9
 
 17570
 
 tss
 
 0.07669
 
 0.24742
 
 Not queried
 
  1.9015041
 
 7.70e-02
 
 1.00e+00
  
 
  
 16
 
   3142645
 
   3143250
 
  PDF   STATS   ENSEMBL   UCSC 
 
  606
 
  8
 
 15605
 
 island
 
 0.77878
 
 0.87833
 
 Not queried
 
  1.9009187
 
 1.30e-01
 
 1.00e+00
  
 
  
 6
 
  32407289
 
  32407570
 
  PDF   STATS   ENSEMBL   UCSC 
 
  282
 
  4
 
 34808
 
 tss
 
 0.63825
 
 0.22816
 
 Not queried
 
  1.9007036
 
 4.86e-01
 
 1.00e+00
  
 
  
 X
 
 152759883
 
 152760248
 
  PDF   STATS   ENSEMBL   UCSC 
 
  366
 
  8
 
 41579
 
 tss
 
 0.20379
 
 0.53908
 
 Not queried
 
  1.9005787
 
 1.48e-02
 
 1.00e+00
  
 
  
 19
 
  13134771
 
  13134830
 
  PDF   STATS   ENSEMBL   UCSC 
 
   60
 
  4
 
 19840
 
 gene;island
 
 0.11707
 
 0.27571
 
 Not queried
 
  1.9002242
 
 2.00e-01
 
 1.00e+00
  
 
  
 17
 
  46671131
 
  46671635
 
  PDF   STATS   ENSEMBL   UCSC 
 
  505
 
  9
 
 17431
 
 tss
 
 0.01666
 
 0.05419
 
 Not queried
 
  1.8994521
 
 9.39e-02
 
 1.00e+00
  
 
  
 10
 
   3827595
 
   3827878
 
  PDF   STATS   ENSEMBL   UCSC 
 
  284
 
  9
 
 4903
 
 tss;island
 
 0.01964
 
 0.07357
 
 Not queried
 
  1.8984497
 
 2.88e-04
 
 1.00e+00
  
 
  
 7
 
 141250684
 
 141251045
 
  PDF   STATS   ENSEMBL   UCSC 
 
  362
 
  6
 
 37880
 
 tss
 
 0.11674
 
 0.03477
 
 Not queried
 
  1.8983132
 
 1.32e-01
 
 1.00e+00
  
 
  
 16
 
  21170927
 
  21171239
 
  PDF   STATS   ENSEMBL   UCSC 
 
  313
 
  5
 
 14658
 
 gene;tss;island
 
 0.08808
 
 0.02523
 
 Not queried
 
  1.8976975
 
 3.10e-01
 
 1.00e+00
  
 
  
 1
 
 161059429
 
 161059654
 
  PDF   STATS   ENSEMBL   UCSC 
 
  226
 
  4
 
 2040
 
 tss
 
 0.37066
 
 0.64466
 
 Not queried
 
  1.8966685
 
 2.86e-02
 
 1.00e+00
  
 
  
 7
 
    120577
 
    120801
 
  PDF   STATS   ENSEMBL   UCSC 
 
  225
 
  5
 
 37964
 
 island
 
 0.72452
 
 0.78826
 
 Not queried
 
  1.8952888
 
 3.10e-01
 
 1.00e+00
  
 
  
 17
 
   7165831
 
   7165909
 
  PDF   STATS   ENSEMBL   UCSC 
 
   79
 
  6
 
 17036
 
 tss;island
 
 0.01251
 
 0.04905
 
 Not queried
 
  1.8946904
 
 6.49e-02
 
 1.00e+00
  
 
  
 2
 
  71017846
 
  71018354
 
  PDF   STATS   ENSEMBL   UCSC 
 
  509
 
  5
 
 23383
 
 tss;island
 
 0.09107
 
 0.22974
 
 Not queried
 
  1.8946029
 
 6.90e-01
 
 1.00e+00
  
 
  
 6
 
  33374613
 
  33374667
 
  PDF   STATS   ENSEMBL   UCSC 
 
   55
 
  5
 
 34211
 
 gene;island
 
 0.78280
 
 0.94096
 
 Not queried
 
  1.8945180
 
 3.17e-02
 
 1.00e+00
  
 
  
 16
 
  66599843
 
  66600311
 
  PDF   STATS   ENSEMBL   UCSC 
 
  469
 
  9
 
 15250
 
 tss
 
 0.83775
 
 0.96467
 
 Not queried
 
  1.8943528
 
 3.99e-03
 
 1.00e+00
  
 
  
 16
 
  66599843
 
  66600700
 
  PDF   STATS   ENSEMBL   UCSC 
 
  858
 
 13
 
 15956
 
 island
 
 0.83775
 
 0.96467
 
 Not queried
 
  1.8943528
 
 2.87e-03
 
 1.00e+00
  
 
  
 6
 
  30652647
 
  30652907
 
  PDF   STATS   ENSEMBL   UCSC 
 
  261
 
  5
 
 33740
 
 gene;island
 
 0.33186
 
 0.61607
 
 Not queried
 
  1.8940592
 
 7.94e-03
 
 1.00e+00
  
 
  
 10
 
  56561096
 
  56561213
 
  PDF   STATS   ENSEMBL   UCSC 
 
  118
 
  4
 
 5070
 
 tss
 
 0.04589
 
 0.14218
 
 Not queried
 
  1.8938489
 
 2.86e-02
 
 1.00e+00
  
 
  
 X
 
  51636562
 
  51636726
 
  PDF   STATS   ENSEMBL   UCSC 
 
  165
 
  7
 
 40996
 
 gene;island
 
 0.11935
 
 0.02358
 
 Not queried
 
  1.8928503
 
 9.73e-02
 
 1.00e+00
  
 
  
 X
 
  57022079
 
  57022192
 
  PDF   STATS   ENSEMBL   UCSC 
 
  114
 
  4
 
 41346
 
 tss;island
 
 0.20708
 
 0.40264
 
 Not queried
 
  1.8926759
 
 2.86e-02
 
 1.00e+00
  
 
  
 16
 
  29625133
 
  29625259
 
  PDF   STATS   ENSEMBL   UCSC 
 
  127
 
  5
 
 15135
 
 tss;island
 
 0.09745
 
 0.04028
 
 Not queried
 
  1.8923905
 
 4.21e-01
 
 1.00e+00
  
 
  
 17
 
  72848048
 
  72848669
 
  PDF   STATS   ENSEMBL   UCSC 
 
  622
 
  5
 
 16726
 
 gene;island
 
 0.12185
 
 0.31066
 
 Not queried
 
  1.8917634
 
 3.10e-01
 
 1.00e+00
  
 
  
 4
 
    571371
 
    571737
 
  PDF   STATS   ENSEMBL   UCSC 
 
  367
 
  4
 
 30673
 
 island
 
 0.24344
 
 0.09221
 
 Not queried
 
  1.8917168
 
 5.71e-02
 
 1.00e+00
  
 
  
 6
 
 136172553
 
 136172818
 
  PDF   STATS   ENSEMBL   UCSC 
 
  266
 
  5
 
 35175
 
 tss
 
 0.05485
 
 0.14298
 
 Not queried
 
  1.8916692
 
 2.22e-01
 
 1.00e+00
  
 
  
 20
 
  31331814
 
  31332353
 
  PDF   STATS   ENSEMBL   UCSC 
 
  540
 
  6
 
 25637
 
 tss;island
 
 0.29117
 
 0.08752
 
 Not queried
 
  1.8916254
 
 1.80e-01
 
 1.00e+00
  
 
  
 17
 
  61042718
 
  61043072
 
  PDF   STATS   ENSEMBL   UCSC 
 
  355
 
  4
 
 18605
 
 island
 
 0.41115
 
 0.29119
 
 Not queried
 
  1.8906378
 
 6.86e-01
 
 1.00e+00
  
 
  
 X
 
  55187242
 
  55188047
 
  PDF   STATS   ENSEMBL   UCSC 
 
  806
 
 14
 
 41819
 
 island
 
 0.07562
 
 0.02426
 
 Not queried
 
  1.8904479
 
 1.86e-02
 
 1.00e+00
  
 
  
 11
 
  44972574
 
  44973218
 
  PDF   STATS   ENSEMBL   UCSC 
 
  645
 
 11
 
 8207
 
 island
 
 0.63126
 
 0.40880
 
 Not queried
 
  1.8903964
 
 3.36e-02
 
 1.00e+00
  
 
  
 12
 
  12871752
 
  12871924
 
  PDF   STATS   ENSEMBL   UCSC 
 
  173
 
  4
 
 9034
 
 gene;island
 
 0.03739
 
 0.14791
 
 Not queried
 
  1.8889380
 
 5.71e-02
 
 1.00e+00
  
 
  
 X
 
  43515213
 
  43515377
 
  PDF   STATS   ENSEMBL   UCSC 
 
  165
 
  4
 
 41266
 
 tss
 
 0.17814
 
 0.05037
 
 Not queried
 
  1.8888913
 
 2.00e-01
 
 1.00e+00
  
 
  
 3
 
  49726775
 
  49727474
 
  PDF   STATS   ENSEMBL   UCSC 
 
  700
 
 12
 
 28290
 
 tss;island
 
 0.02333
 
 0.08562
 
 Not queried
 
  1.8883981
 
 1.45e-02
 
 1.00e+00
  
 
  
 12
 
 127212034
 
 127212528
 
  PDF   STATS   ENSEMBL   UCSC 
 
  495
 
  4
 
 10956
 
 island
 
 0.15273
 
 0.47122
 
 Not queried
 
  1.8873011
 
 2.86e-02
 
 1.00e+00
  
 
  
 X
 
  69501934
 
  69502066
 
  PDF   STATS   ENSEMBL   UCSC 
 
  133
 
  6
 
 41366
 
 tss
 
 0.62500
 
 0.83715
 
 Not queried
 
  1.8872347
 
 9.31e-02
 
 1.00e+00
  
 
  
 6
 
  33160067
 
  33160234
 
  PDF   STATS   ENSEMBL   UCSC 
 
  168
 
  6
 
 34151
 
 gene
 
 0.15514
 
 0.38908
 
 Not queried
 
  1.8858807
 
 2.60e-02
 
 1.00e+00
  
 
  
 12
 
 132851783
 
 132852053
 
  PDF   STATS   ENSEMBL   UCSC 
 
  271
 
  4
 
 9415
 
 gene;island
 
 0.38295
 
 0.72109
 
 Not queried
 
  1.8857964
 
 2.86e-02
 
 1.00e+00
  
 
  
 10
 
  97515222
 
  97515398
 
  PDF   STATS   ENSEMBL   UCSC 
 
  177
 
  4
 
 4675
 
 gene;tss
 
 0.19453
 
 0.06424
 
 Not queried
 
  1.8857483
 
 5.71e-02
 
 1.00e+00
  
 
  
 17
 
   3558202
 
   3558426
 
  PDF   STATS   ENSEMBL   UCSC 
 
  225
 
  4
 
 16306
 
 gene
 
 0.83737
 
 0.93726
 
 Not queried
 
  1.8850608
 
 3.43e-01
 
 1.00e+00
  
 
  
 16
 
  56965376
 
  56965658
 
  PDF   STATS   ENSEMBL   UCSC 
 
  283
 
  4
 
 15224
 
 tss
 
 0.25205
 
 0.52558
 
 Not queried
 
  1.8849176
 
 2.00e-01
 
 1.00e+00
  
 
  
 2
 
  24398427
 
  24398859
 
  PDF   STATS   ENSEMBL   UCSC 
 
  433
 
  5
 
 22561
 
 gene;island
 
 0.62745
 
 0.87479
 
 Not queried
 
  1.8848447
 
 9.52e-02
 
 1.00e+00
  
 
  
 3
 
 183993368
 
 183994242
 
  PDF   STATS   ENSEMBL   UCSC 
 
  875
 
 11
 
 28017
 
 gene;island
 
 0.50113
 
 0.76901
 
 Not queried
 
  1.8835116
 
 1.92e-02
 
 1.00e+00
  
 
  
 8
 
  81397601
 
  81397876
 
  PDF   STATS   ENSEMBL   UCSC 
 
  276
 
  5
 
 39718
 
 tss;island
 
 0.01026
 
 0.02188
 
 Not queried
 
  1.8833810
 
 9.52e-02
 
 1.00e+00
  
 
  
 1
 
  45792540
 
  45792733
 
  PDF   STATS   ENSEMBL   UCSC 
 
  194
 
  7
 
 3192
 
 island
 
 0.52352
 
 0.83761
 
 Not queried
 
  1.8820772
 
 6.99e-03
 
 1.00e+00
  
 
  
 5
 
 153852554
 
 153853283
 
  PDF   STATS   ENSEMBL   UCSC 
 
  730
 
  8
 
 33265
 
 island
 
 0.81352
 
 0.51010
 
 Not queried
 
  1.8817918
 
 6.99e-03
 
 1.00e+00
  
 
  
 8
 
  25905478
 
  25905811
 
  PDF   STATS   ENSEMBL   UCSC 
 
  334
 
  4
 
 40106
 
 island
 
 0.07209
 
 0.17252
 
 Not queried
 
  1.8809248
 
 6.86e-01
 
 1.00e+00
  
 
  
 7
 
 136553088
 
 136553327
 
  PDF   STATS   ENSEMBL   UCSC 
 
  240
 
  7
 
 37857
 
 tss;island
 
 0.23269
 
 0.28023
 
 Not queried
 
  1.8809054
 
 3.18e-01
 
 1.00e+00
  
 
  
 12
 
  53183773
 
  53184086
 
  PDF   STATS   ENSEMBL   UCSC 
 
  314
 
  5
 
 9115
 
 gene;island
 
 0.73324
 
 0.90604
 
 Not queried
 
  1.8804743
 
 9.52e-02
 
 1.00e+00
  
 
  
 6
 
  76059450
 
  76059773
 
  PDF   STATS   ENSEMBL   UCSC 
 
  324
 
  6
 
 34331
 
 gene
 
 0.07737
 
 0.02934
 
 Not queried
 
  1.8798050
 
 4.33e-03
 
 1.00e+00
  
 
  
 22
 
  31002757
 
  31003147
 
  PDF   STATS   ENSEMBL   UCSC 
 
  391
 
  8
 
 27024
 
 tss
 
 0.04259
 
 0.15118
 
 Not queried
 
  1.8787087
 
 1.09e-03
 
 1.00e+00
  
 
  
 13
 
 114880761
 
 114881194
 
  PDF   STATS   ENSEMBL   UCSC 
 
  434
 
  6
 
 11288
 
 gene;island
 
 0.61564
 
 0.91299
 
 Not queried
 
  1.8785956
 
 8.66e-03
 
 1.00e+00
  
 
  
 22
 
  31536476
 
  31536784
 
  PDF   STATS   ENSEMBL   UCSC 
 
  309
 
  5
 
 27033
 
 tss
 
 0.39771
 
 0.15234
 
 Not queried
 
  1.8774028
 
 2.22e-01
 
 1.00e+00
  
 
  
 2
 
   1748872
 
   1749076
 
  PDF   STATS   ENSEMBL   UCSC 
 
  205
 
  4
 
 23120
 
 tss;island
 
 0.56957
 
 0.30847
 
 Not queried
 
  1.8774019
 
 2.86e-02
 
 1.00e+00
  
 
  
 X
 
  15755989
 
  15756062
 
  PDF   STATS   ENSEMBL   UCSC 
 
   74
 
  4
 
 41201
 
 tss;island
 
 0.02360
 
 0.06996
 
 Not queried
 
  1.8769502
 
 5.71e-02
 
 1.00e+00
  
 
  
 15
 
  29499226
 
  29499526
 
  PDF   STATS   ENSEMBL   UCSC 
 
  301
 
  5
 
 13241
 
 gene
 
 0.68870
 
 0.91403
 
 Not queried
 
  1.8767004
 
 3.17e-02
 
 1.00e+00
  
 
  
 12
 
 113917395
 
 113917737
 
  PDF   STATS   ENSEMBL   UCSC 
 
  343
 
  5
 
 10807
 
 island
 
 0.03597
 
 0.09153
 
 Not queried
 
  1.8747257
 
 9.52e-02
 
 1.00e+00
  
 
  
 1
 
   3184434
 
   3184704
 
  PDF   STATS   ENSEMBL   UCSC 
 
  271
 
  4
 
 114
 
 gene;island
 
 0.61311
 
 0.86318
 
 Not queried
 
  1.8746586
 
 2.86e-02
 
 1.00e+00
  
 
  
 6
 
  31276088
 
  31276212
 
  PDF   STATS   ENSEMBL   UCSC 
 
  125
 
  5
 
 35701
 
 island
 
 0.03262
 
 0.13569
 
 Not queried
 
  1.8737864
 
 1.59e-02
 
 1.00e+00
  
 
  
 2
 
 242808427
 
 242808907
 
  PDF   STATS   ENSEMBL   UCSC 
 
  481
 
  5
 
 25274
 
 island
 
 0.48093
 
 0.81286
 
 Not queried
 
  1.8735243
 
 7.94e-03
 
 1.00e+00
  
 
  
 1
 
 230777685
 
 230777875
 
  PDF   STATS   ENSEMBL   UCSC 
 
  191
 
  4
 
 2312
 
 tss;island
 
 0.04079
 
 0.02136
 
 Not queried
 
  1.8728211
 
 2.86e-02
 
 1.00e+00
  
 
  
 19
 
  10426722
 
  10426933
 
  PDF   STATS   ENSEMBL   UCSC 
 
  212
 
  4
 
 20409
 
 tss;island
 
 0.13407
 
 0.31237
 
 Not queried
 
  1.8721892
 
 4.86e-01
 
 1.00e+00
  
 
  
 10
 
    729479
 
    729956
 
  PDF   STATS   ENSEMBL   UCSC 
 
  478
 
  6
 
 4438
 
 gene
 
 0.93566
 
 0.81644
 
 Not queried
 
  1.8717924
 
 8.66e-03
 
 1.00e+00
  
 
  
 3
 
  27764473
 
  27764816
 
  PDF   STATS   ENSEMBL   UCSC 
 
  344
 
  4
 
 28154
 
 tss;island
 
 0.45840
 
 0.18801
 
 Not queried
 
  1.8717877
 
 5.71e-02
 
 1.00e+00
  
 
  
 17
 
  44896748
 
  44897024
 
  PDF   STATS   ENSEMBL   UCSC 
 
  277
 
  4
 
 17403
 
 tss;island
 
 0.11554
 
 0.02840
 
 Not queried
 
  1.8713955
 
 2.86e-02
 
 1.00e+00
  
 
  
 4
 
   1595294
 
   1595726
 
  PDF   STATS   ENSEMBL   UCSC 
 
  433
 
  5
 
 30745
 
 island
 
 0.81425
 
 0.96282
 
 Not queried
 
  1.8698062
 
 3.17e-02
 
 1.00e+00
  
 
  
 14
 
  88788343
 
  88788672
 
  PDF   STATS   ENSEMBL   UCSC 
 
  330
 
  6
 
 12137
 
 gene;island
 
 0.16806
 
 0.26250
 
 Not queried
 
  1.8685743
 
 2.40e-01
 
 1.00e+00
  
 
  
 6
 
  32809263
 
  32809657
 
  PDF   STATS   ENSEMBL   UCSC 
 
  395
 
 10
 
 34083
 
 gene;island
 
 0.84434
 
 0.66111
 
 Not queried
 
  1.8683839
 
 5.20e-03
 
 1.00e+00
  
 
  
 3
 
 148804272
 
 148805294
 
  PDF   STATS   ENSEMBL   UCSC 
 
 1023
 
 23
 
 29536
 
 island
 
 0.93849
 
 0.49347
 
 Not queried
 
  1.8678163
 
 3.22e-02
 
 1.00e+00
  
 
  
 6
 
   1515524
 
   1515797
 
  PDF   STATS   ENSEMBL   UCSC 
 
  274
 
  4
 
 35304
 
 island
 
 0.48561
 
 0.79770
 
 Not queried
 
  1.8669636
 
 2.86e-02
 
 1.00e+00
  
 
  
 20
 
   1165065
 
   1165435
 
  PDF   STATS   ENSEMBL   UCSC 
 
  371
 
  5
 
 25893
 
 island
 
 0.79794
 
 0.51992
 
 Not queried
 
  1.8665451
 
 9.52e-02
 
 1.00e+00
  
 
  
 2
 
 240302585
 
 240302855
 
  PDF   STATS   ENSEMBL   UCSC 
 
  271
 
  4
 
 23081
 
 gene
 
 0.61202
 
 0.88583
 
 Not queried
 
  1.8664077
 
 5.71e-02
 
 1.00e+00
  
 
  
 5
 
 122971759
 
 122971951
 
  PDF   STATS   ENSEMBL   UCSC 
 
  193
 
  4
 
 32996
 
 island
 
 0.64553
 
 0.92035
 
 Not queried
 
  1.8659795
 
 1.14e-01
 
 1.00e+00
  
 
  
 19
 
  17487776
 
  17488445
 
  PDF   STATS   ENSEMBL   UCSC 
 
  670
 
  8
 
 21704
 
 island
 
 0.69180
 
 0.90016
 
 Not queried
 
  1.8647053
 
 1.04e-02
 
 1.00e+00
  
 
  
 11
 
   1769152
 
   1769645
 
  PDF   STATS   ENSEMBL   UCSC 
 
  494
 
  9
 
 6412
 
 gene;island
 
 0.71395
 
 0.92663
 
 Not queried
 
  1.8645603
 
 1.85e-03
 
 1.00e+00
  
 
  
 11
 
   1769382
 
   1769645
 
  PDF   STATS   ENSEMBL   UCSC 
 
  264
 
  7
 
 7024
 
 tss
 
 0.71395
 
 0.92663
 
 Not queried
 
  1.8645603
 
 6.99e-03
 
 1.00e+00
  
 
  
 1
 
  51810823
 
  51811185
 
  PDF   STATS   ENSEMBL   UCSC 
 
  363
 
  5
 
 1619
 
 tss
 
 0.32184
 
 0.63338
 
 Not queried
 
  1.8640695
 
 4.21e-01
 
 1.00e+00
  
 
  
 5
 
 172664995
 
 172665149
 
  PDF   STATS   ENSEMBL   UCSC 
 
  155
 
  4
 
 33344
 
 island
 
 0.48385
 
 0.79606
 
 Not queried
 
  1.8636905
 
 2.86e-02
 
 1.00e+00
  
 
  
 X
 
  22290866
 
  22290942
 
  PDF   STATS   ENSEMBL   UCSC 
 
   77
 
  5
 
 41224
 
 tss
 
 0.79643
 
 0.95185
 
 Not queried
 
  1.8629686
 
 7.94e-03
 
 1.00e+00
  
 
  
 X
 
  55187242
 
  55187570
 
  PDF   STATS   ENSEMBL   UCSC 
 
  329
 
  8
 
 41001
 
 gene
 
 0.05755
 
 0.01438
 
 Not queried
 
  1.8624464
 
 1.05e-01
 
 1.00e+00
  
 
  
 13
 
 113655468
 
 113655622
 
  PDF   STATS   ENSEMBL   UCSC 
 
  155
 
  4
 
 11238
 
 gene;island
 
 0.51222
 
 0.79176
 
 Not queried
 
  1.8621928
 
 2.00e-01
 
 1.00e+00
  
 
  
 9
 
   2717146
 
   2717621
 
  PDF   STATS   ENSEMBL   UCSC 
 
  476
 
  5
 
 40797
 
 island
 
 0.80136
 
 0.95176
 
 Not queried
 
  1.8614892
 
 3.17e-02
 
 1.00e+00
  
 
  
 1
 
   2063799
 
   2064216
 
  PDF   STATS   ENSEMBL   UCSC 
 
  418
 
  5
 
 63
 
 gene;island
 
 0.25811
 
 0.56897
 
 Not queried
 
  1.8610345
 
 9.52e-02
 
 1.00e+00
  
 
  
 2
 
 171784945
 
 171785124
 
  PDF   STATS   ENSEMBL   UCSC 
 
  180
 
  4
 
 23672
 
 tss;island
 
 0.75592
 
 0.92667
 
 Not queried
 
  1.8605293
 
 3.43e-01
 
 1.00e+00
  
 
  
 10
 
  94459183
 
  94459665
 
  PDF   STATS   ENSEMBL   UCSC 
 
  483
 
  6
 
 5928
 
 island
 
 0.06858
 
 0.03556
 
 Not queried
 
  1.8602740
 
 9.31e-02
 
 1.00e+00
  
 
  
 16
 
    898803
 
    899108
 
  PDF   STATS   ENSEMBL   UCSC 
 
  306
 
  4
 
 15463
 
 island
 
 0.43605
 
 0.77409
 
 Not queried
 
  1.8600784
 
 2.00e-01
 
 1.00e+00
  
 
  
 11
 
 103908255
 
 103908576
 
  PDF   STATS   ENSEMBL   UCSC 
 
  322
 
  4
 
 6860
 
 gene;island
 
 0.64277
 
 0.83830
 
 Not queried
 
  1.8599434
 
 1.14e-01
 
 1.00e+00
  
 
  
 12
 
  10183167
 
  10183267
 
  PDF   STATS   ENSEMBL   UCSC 
 
  101
 
  6
 
 9534
 
 tss
 
 0.84528
 
 0.94870
 
 Not queried
 
  1.8577019
 
 2.60e-02
 
 1.00e+00
  
 
  
 19
 
   1248287
 
   1248689
 
  PDF   STATS   ENSEMBL   UCSC 
 
  403
 
  6
 
 21169
 
 island
 
 0.26325
 
 0.07483
 
 Not queried
 
  1.8567166
 
 3.10e-01
 
 1.00e+00
  
 
  
 1
 
   3059517
 
   3059820
 
  PDF   STATS   ENSEMBL   UCSC 
 
  304
 
  4
 
 103
 
 gene;island
 
 0.77205
 
 0.92519
 
 Not queried
 
  1.8553906
 
 1.14e-01
 
 1.00e+00
  
 
  
 1
 
 184005360
 
 184005990
 
  PDF   STATS   ENSEMBL   UCSC 
 
  631
 
  5
 
 917
 
 gene;island
 
 0.34637
 
 0.11877
 
 Not queried
 
  1.8546761
 
 2.22e-01
 
 1.00e+00
  
 
  
 3
 
  64670459
 
  64670640
 
  PDF   STATS   ENSEMBL   UCSC 
 
  182
 
  4
 
 27808
 
 gene;island
 
 0.40162
 
 0.16182
 
 Not queried
 
  1.8538817
 
 4.86e-01
 
 1.00e+00
  
 
  
 17
 
  37754160
 
  37754424
 
  PDF   STATS   ENSEMBL   UCSC 
 
  265
 
  4
 
 18244
 
 island
 
 0.14055
 
 0.44284
 
 Not queried
 
  1.8538359
 
 3.43e-01
 
 1.00e+00
  
 
  
 4
 
   1008425
 
   1008738
 
  PDF   STATS   ENSEMBL   UCSC 
 
  314
 
  4
 
 29813
 
 gene;island
 
 0.44055
 
 0.75819
 
 Not queried
 
  1.8518809
 
 5.71e-02
 
 1.00e+00
  
 
  
 13
 
 114760892
 
 114761326
 
  PDF   STATS   ENSEMBL   UCSC 
 
  435
 
  4
 
 11274
 
 gene;island
 
 0.32964
 
 0.74971
 
 Not queried
 
  1.8511052
 
 5.71e-02
 
 1.00e+00
  
 
  
 2
 
 242930039
 
 242930747
 
  PDF   STATS   ENSEMBL   UCSC 
 
  709
 
  7
 
 25281
 
 island
 
 0.66742
 
 0.88194
 
 Not queried
 
  1.8510179
 
 2.62e-02
 
 1.00e+00
  
 
  
 11
 
 130298172
 
 130298880
 
  PDF   STATS   ENSEMBL   UCSC 
 
  709
 
 11
 
 8937
 
 island
 
 0.15684
 
 0.05040
 
 Not queried
 
  1.8505190
 
 1.33e-01
 
 1.00e+00
  
 
  
 19
 
  39989234
 
  39989542
 
  PDF   STATS   ENSEMBL   UCSC 
 
  309
 
  5
 
 20738
 
 tss
 
 0.02656
 
 0.04115
 
 Not queried
 
  1.8498727
 
 2.22e-01
 
 1.00e+00
  
 
  
 19
 
  39989234
 
  39989592
 
  PDF   STATS   ENSEMBL   UCSC 
 
  359
 
  7
 
 21965
 
 island
 
 0.01644
 
 0.03840
 
 Not queried
 
  1.8498727
 
 3.18e-01
 
 1.00e+00
  
 
  
 4
 
 141264393
 
 141264721
 
  PDF   STATS   ENSEMBL   UCSC 
 
  329
 
  5
 
 30089
 
 gene
 
 0.81546
 
 0.94093
 
 Not queried
 
  1.8497789
 
 1.59e-02
 
 1.00e+00
  
 
  
 7
 
  36022276
 
  36022841
 
  PDF   STATS   ENSEMBL   UCSC 
 
  566
 
  5
 
 38349
 
 island
 
 0.64653
 
 0.89256
 
 Not queried
 
  1.8495465
 
 1.59e-02
 
 1.00e+00
  
 
  
 1
 
  31158026
 
  31158299
 
  PDF   STATS   ENSEMBL   UCSC 
 
  274
 
  5
 
 2958
 
 island
 
 0.03952
 
 0.08399
 
 Not queried
 
  1.8494491
 
 4.21e-01
 
 1.00e+00
  
 
  
 3
 
  44063315
 
  44063832
 
  PDF   STATS   ENSEMBL   UCSC 
 
  518
 
  6
 
 28988
 
 island
 
 0.17182
 
 0.06457
 
 Not queried
 
  1.8493886
 
 3.10e-01
 
 1.00e+00
  
 
  
 5
 
  43020161
 
  43020716
 
  PDF   STATS   ENSEMBL   UCSC 
 
  556
 
  7
 
 32703
 
 island
 
 0.02256
 
 0.08442
 
 Not queried
 
  1.8490316
 
 7.28e-02
 
 1.00e+00
  
 
  
 17
 
  38256932
 
  38257323
 
  PDF   STATS   ENSEMBL   UCSC 
 
  392
 
 11
 
 18262
 
 island
 
 0.05626
 
 0.02047
 
 Not queried
 
  1.8457616
 
 1.58e-02
 
 1.00e+00
  
 
  
 12
 
  14927294
 
  14927542
 
  PDF   STATS   ENSEMBL   UCSC 
 
  249
 
  4
 
 9040
 
 gene
 
 0.01377
 
 0.03186
 
 Not queried
 
  1.8447425
 
 2.86e-02
 
 1.00e+00
  
 
  
 7
 
  73241729
 
  73242028
 
  PDF   STATS   ENSEMBL   UCSC 
 
  300
 
  4
 
 38506
 
 island
 
 0.42606
 
 0.70545
 
 Not queried
 
  1.8432397
 
 5.71e-02
 
 1.00e+00
  
 
  
 17
 
  10541401
 
  10541764
 
  PDF   STATS   ENSEMBL   UCSC 
 
  364
 
  4
 
 16406
 
 gene;island
 
 0.46522
 
 0.79982
 
 Not queried
 
  1.8426780
 
 3.43e-01
 
 1.00e+00
  
 
  
 7
 
 154863176
 
 154863381
 
  PDF   STATS   ENSEMBL   UCSC 
 
  206
 
  4
 
 37242
 
 gene;island
 
 0.21130
 
 0.47021
 
 Not queried
 
  1.8426349
 
 2.00e-01
 
 1.00e+00
  
 
  
 6
 
  32825040
 
  32825148
 
  PDF   STATS   ENSEMBL   UCSC 
 
  109
 
  6
 
 34093
 
 gene;island
 
 0.64898
 
 0.42807
 
 Not queried
 
  1.8412955
 
 4.11e-02
 
 1.00e+00
  
 
  
 19
 
  18496756
 
  18496953
 
  PDF   STATS   ENSEMBL   UCSC 
 
  198
 
  4
 
 20582
 
 tss
 
 0.40226
 
 0.70810
 
 Not queried
 
  1.8403999
 
 2.86e-02
 
 1.00e+00
  
 
  
 19
 
  18496756
 
  18497201
 
  PDF   STATS   ENSEMBL   UCSC 
 
  446
 
  8
 
 21743
 
 island
 
 0.49199
 
 0.73314
 
 Not queried
 
  1.8403999
 
 8.30e-02
 
 1.00e+00
  
 
  
 12
 
   2944286
 
   2944493
 
  PDF   STATS   ENSEMBL   UCSC 
 
  208
 
  6
 
 9466
 
 tss
 
 0.45497
 
 0.78275
 
 Not queried
 
  1.8393881
 
 4.11e-02
 
 1.00e+00
  
 
  
 3
 
 183959465
 
 183959853
 
  PDF   STATS   ENSEMBL   UCSC 
 
  389
 
  4
 
 28015
 
 gene;island
 
 0.40304
 
 0.70544
 
 Not queried
 
  1.8389494
 
 2.86e-02
 
 1.00e+00
  
 
  
 12
 
  46319990
 
  46320110
 
  PDF   STATS   ENSEMBL   UCSC 
 
  121
 
  4
 
 9079
 
 gene
 
 0.93892
 
 0.97722
 
 Not queried
 
  1.8388235
 
 2.86e-02
 
 1.00e+00
  
 
  
 10
 
 101282572
 
 101282883
 
  PDF   STATS   ENSEMBL   UCSC 
 
  312
 
  5
 
 5990
 
 island
 
 0.23348
 
 0.04274
 
 Not queried
 
  1.8381317
 
 1.51e-01
 
 1.00e+00
  
 
  
 12
 
  50297777
 
  50297945
 
  PDF   STATS   ENSEMBL   UCSC 
 
  169
 
  4
 
 9672
 
 tss
 
 0.33715
 
 0.62926
 
 Not queried
 
  1.8369906
 
 2.86e-02
 
 1.00e+00
  
 
  
 1
 
  22470218
 
  22470343
 
  PDF   STATS   ENSEMBL   UCSC 
 
  126
 
  4
 
 1327
 
 tss;island
 
 0.34103
 
 0.11028
 
 Not queried
 
  1.8360213
 
 2.00e-01
 
 1.00e+00
  
 
  
 12
 
  22094963
 
  22095477
 
  PDF   STATS   ENSEMBL   UCSC 
 
  515
 
  5
 
 10256
 
 island
 
 0.05735
 
 0.05921
 
 Not queried
 
  1.8342958
 
 5.48e-01
 
 1.00e+00
  
 
  
 15
 
  84047906
 
  84048308
 
  PDF   STATS   ENSEMBL   UCSC 
 
  403
 
  5
 
 14371
 
 island
 
 0.85506
 
 0.59895
 
 Not queried
 
  1.8342534
 
 7.94e-03
 
 1.00e+00
  
 
  
 4
 
  80246713
 
  80247088
 
  PDF   STATS   ENSEMBL   UCSC 
 
  376
 
  4
 
 29987
 
 gene
 
 0.52810
 
 0.75261
 
 Not queried
 
  1.8338982
 
 2.86e-02
 
 1.00e+00
  
 
  
 19
 
  19322527
 
  19322778
 
  PDF   STATS   ENSEMBL   UCSC 
 
  252
 
  6
 
 20603
 
 tss
 
 0.02377
 
 0.06554
 
 Not queried
 
  1.8337394
 
 3.94e-01
 
 1.00e+00
  
 
  
 17
 
  74524852
 
  74525125
 
  PDF   STATS   ENSEMBL   UCSC 
 
  274
 
  4
 
 16756
 
 gene
 
 0.65697
 
 0.90335
 
 Not queried
 
  1.8335810
 
 3.43e-01
 
 1.00e+00
  
 
  
 10
 
  26931731
 
  26931991
 
  PDF   STATS   ENSEMBL   UCSC 
 
  261
 
  8
 
 4978
 
 tss
 
 0.52727
 
 0.81606
 
 Not queried
 
  1.8333702
 
 8.30e-02
 
 1.00e+00
  
 
  
 17
 
  57408935
 
  57409010
 
  PDF   STATS   ENSEMBL   UCSC 
 
   76
 
  5
 
 17512
 
 tss
 
 0.01037
 
 0.01578
 
 Not queried
 
  1.8333589
 
 9.52e-02
 
 1.00e+00
  
 
  
 3
 
  11034279
 
  11034501
 
  PDF   STATS   ENSEMBL   UCSC 
 
  223
 
  5
 
 28837
 
 island
 
 0.06132
 
 0.22320
 
 Not queried
 
  1.8332918
 
 9.52e-02
 
 1.00e+00
  
 
  
 19
 
  48202226
 
  48202596
 
  PDF   STATS   ENSEMBL   UCSC 
 
  371
 
  4
 
 20082
 
 gene;island
 
 0.52776
 
 0.81643
 
 Not queried
 
  1.8327087
 
 2.00e-01
 
 1.00e+00
  
 
  
 1
 
   1293868
 
   1294140
 
  PDF   STATS   ENSEMBL   UCSC 
 
  273
 
  4
 
 2425
 
 island
 
 0.36390
 
 0.69770
 
 Not queried
 
  1.8321791
 
 2.86e-02
 
 1.00e+00
  
 
  
 17
 
  38375119
 
  38375510
 
  PDF   STATS   ENSEMBL   UCSC 
 
  392
 
  7
 
 17294
 
 tss;island
 
 0.02142
 
 0.01226
 
 Not queried
 
  1.8321242
 
 2.09e-01
 
 1.00e+00
  
 
  
 3
 
  42947263
 
  42947771
 
  PDF   STATS   ENSEMBL   UCSC 
 
  509
 
  7
 
 28980
 
 island
 
 0.79867
 
 0.96023
 
 Not queried
 
  1.8314317
 
 1.11e-02
 
 1.00e+00
  
 
  
 11
 
  31846414
 
  31846849
 
  PDF   STATS   ENSEMBL   UCSC 
 
  436
 
  6
 
 8148
 
 island
 
 0.18208
 
 0.09122
 
 Not queried
 
  1.8308580
 
 3.10e-01
 
 1.00e+00
  
 
  
 1
 
 155232925
 
 155233109
 
  PDF   STATS   ENSEMBL   UCSC 
 
  185
 
  4
 
 795
 
 gene;tss;island
 
 0.72435
 
 0.92758
 
 Not queried
 
  1.8307425
 
 1.14e-01
 
 1.00e+00
  
 
  
 1
 
 153508755
 
 153508875
 
  PDF   STATS   ENSEMBL   UCSC 
 
  121
 
  4
 
 1938
 
 tss
 
 0.44514
 
 0.78364
 
 Not queried
 
  1.8293618
 
 2.86e-02
 
 1.00e+00
  
 
  
 4
 
 155662217
 
 155662515
 
  PDF   STATS   ENSEMBL   UCSC 
 
  299
 
  4
 
 31331
 
 island
 
 0.25429
 
 0.03574
 
 Not queried
 
  1.8284227
 
 3.43e-01
 
 1.00e+00
  
 
  
 1
 
   1190026
 
   1190238
 
  PDF   STATS   ENSEMBL   UCSC 
 
  213
 
  4
 
 24
 
 gene;island
 
 0.83740
 
 0.95102
 
 Not queried
 
  1.8270601
 
 4.86e-01
 
 1.00e+00
  
 
  
 10
 
  44788627
 
  44789077
 
  PDF   STATS   ENSEMBL   UCSC 
 
  451
 
  5
 
 5688
 
 island
 
 0.57411
 
 0.83114
 
 Not queried
 
  1.8261284
 
 9.52e-02
 
 1.00e+00
  
 
  
 6
 
  27782909
 
  27783226
 
  PDF   STATS   ENSEMBL   UCSC 
 
  318
 
  4
 
 33556
 
 gene
 
 0.19687
 
 0.59028
 
 Not queried
 
  1.8240630
 
 1.14e-01
 
 1.00e+00
  
 
  
 6
 
  33386967
 
  33387205
 
  PDF   STATS   ENSEMBL   UCSC 
 
  239
 
  6
 
 34844
 
 tss;island
 
 0.66938
 
 0.90471
 
 Not queried
 
  1.8217312
 
 4.11e-02
 
 1.00e+00
  
 
  
 11
 
  10562070
 
  10562473
 
  PDF   STATS   ENSEMBL   UCSC 
 
  404
 
  4
 
 6489
 
 gene;island
 
 0.01496
 
 0.02984
 
 Not queried
 
  1.8215735
 
 2.00e-01
 
 1.00e+00
  
 
  
 17
 
   6735063
 
   6735316
 
  PDF   STATS   ENSEMBL   UCSC 
 
  254
 
  8
 
 17021
 
 tss
 
 0.01875
 
 0.06396
 
 Not queried
 
  1.8206388
 
 4.66e-03
 
 1.00e+00
  
 
  
 7
 
  94536541
 
  94536787
 
  PDF   STATS   ENSEMBL   UCSC 
 
  247
 
  4
 
 37657
 
 tss
 
 0.84506
 
 0.61348
 
 Not queried
 
  1.8201541
 
 2.00e-01
 
 1.00e+00
  
 
  
 7
 
 158045980
 
 158046358
 
  PDF   STATS   ENSEMBL   UCSC 
 
  379
 
  6
 
 37298
 
 gene
 
 0.51641
 
 0.80731
 
 Not queried
 
  1.8197901
 
 9.31e-02
 
 1.00e+00
  
 
  
 7
 
  23530449
 
  23530810
 
  PDF   STATS   ENSEMBL   UCSC 
 
  362
 
  4
 
 36853
 
 gene;island
 
 0.29266
 
 0.64858
 
 Not queried
 
  1.8195021
 
 5.71e-02
 
 1.00e+00
  
 
  
 6
 
  32798417
 
  32798510
 
  PDF   STATS   ENSEMBL   UCSC 
 
   94
 
  4
 
 34075
 
 gene
 
 0.53421
 
 0.23777
 
 Not queried
 
  1.8191934
 
 4.86e-01
 
 1.00e+00
  
 
  
 17
 
  80084554
 
  80084751
 
  PDF   STATS   ENSEMBL   UCSC 
 
  198
 
  4
 
 16906
 
 gene;island
 
 0.50780
 
 0.85138
 
 Not queried
 
  1.8189434
 
 3.43e-01
 
 1.00e+00
  
 
  
 2
 
  85553759
 
  85554135
 
  PDF   STATS   ENSEMBL   UCSC 
 
  377
 
  5
 
 22724
 
 gene;island
 
 0.03647
 
 0.15227
 
 Not queried
 
  1.8187681
 
 9.52e-02
 
 1.00e+00
  
 
  
 14
 
  29234890
 
  29235469
 
  PDF   STATS   ENSEMBL   UCSC 
 
  580
 
 11
 
 12307
 
 tss;island
 
 0.15844
 
 0.05424
 
 Not queried
 
  1.8170272
 
 2.75e-04
 
 1.00e+00
  
 
  
 5
 
  42924215
 
  42924694
 
  PDF   STATS   ENSEMBL   UCSC 
 
  480
 
  4
 
 32698
 
 island
 
 0.59849
 
 0.85513
 
 Not queried
 
  1.8167143
 
 2.00e-01
 
 1.00e+00
  
 
  
 17
 
   7757933
 
   7758102
 
  PDF   STATS   ENSEMBL   UCSC 
 
  170
 
  5
 
 16381
 
 gene
 
 0.62072
 
 0.86521
 
 Not queried
 
  1.8165163
 
 3.10e-01
 
 1.00e+00
  
 
  
 13
 
  47470793
 
  47471090
 
  PDF   STATS   ENSEMBL   UCSC 
 
  298
 
  5
 
 11130
 
 gene
 
 0.87869
 
 0.66106
 
 Not queried
 
  1.8151859
 
 2.22e-01
 
 1.00e+00
  
 
  
 19
 
    863054
 
    863244
 
  PDF   STATS   ENSEMBL   UCSC 
 
  191
 
  4
 
 19600
 
 gene;island
 
 0.60935
 
 0.32380
 
 Not queried
 
  1.8143744
 
 5.71e-02
 
 1.00e+00
  
 
  
 Y
 
  21728952
 
  21729144
 
  PDF   STATS   ENSEMBL   UCSC 
 
  193
 
  6
 
 42171
 
 tss
 
 0.01799
 
 0.11642
 
 Not queried
 
  1.8133815
 
 5.89e-01
 
 1.00e+00
  
 
  
 5
 
 140719008
 
 140719302
 
  PDF   STATS   ENSEMBL   UCSC 
 
  295
 
  4
 
 31760
 
 gene;island
 
 0.69207
 
 0.91039
 
 Not queried
 
  1.8126068
 
 5.71e-02
 
 1.00e+00
  
 
  
 1
 
  16163479
 
  16164122
 
  PDF   STATS   ENSEMBL   UCSC 
 
  644
 
  7
 
 251
 
 gene;island
 
 0.56000
 
 0.82138
 
 Not queried
 
  1.8112601
 
 1.75e-02
 
 1.00e+00
  
 
  
 2
 
 220361916
 
 220362242
 
  PDF   STATS   ENSEMBL   UCSC 
 
  327
 
  6
 
 25088
 
 island
 
 0.41586
 
 0.67391
 
 Not queried
 
  1.8110930
 
 2.16e-03
 
 1.00e+00
  
 
  
 17
 
    146404
 
    146926
 
  PDF   STATS   ENSEMBL   UCSC 
 
  523
 
  5
 
 16250
 
 gene
 
 0.58406
 
 0.83128
 
 Not queried
 
  1.8110246
 
 4.21e-01
 
 1.00e+00
  
 
  
 X
 
 152954816
 
 152954921
 
  PDF   STATS   ENSEMBL   UCSC 
 
  106
 
  6
 
 41585
 
 tss
 
 0.76112
 
 0.58387
 
 Not queried
 
  1.8103547
 
 4.33e-03
 
 1.00e+00
  
 
  
 6
 
  31516015
 
  31516059
 
  PDF   STATS   ENSEMBL   UCSC 
 
   45
 
  4
 
 33836
 
 gene;tss
 
 0.79861
 
 0.53610
 
 Not queried
 
  1.8102020
 
 2.00e-01
 
 1.00e+00
  
 
  
 16
 
  66600294
 
  66600700
 
  PDF   STATS   ENSEMBL   UCSC 
 
  407
 
  6
 
 14751
 
 gene
 
 0.83143
 
 0.95158
 
 Not queried
 
  1.8091373
 
 9.31e-02
 
 1.00e+00
  
 
  
 8
 
  70983567
 
  70983760
 
  PDF   STATS   ENSEMBL   UCSC 
 
  194
 
  4
 
 39693
 
 tss
 
 0.11723
 
 0.04826
 
 Not queried
 
  1.8091120
 
 1.14e-01
 
 1.00e+00
  
 
  
 2
 
 176987161
 
 176987411
 
  PDF   STATS   ENSEMBL   UCSC 
 
  251
 
  5
 
 23699
 
 tss
 
 0.00535
 
 0.01971
 
 Not queried
 
  1.8088162
 
 9.52e-02
 
 1.00e+00
  
 
  
 2
 
 219232590
 
 219232875
 
  PDF   STATS   ENSEMBL   UCSC 
 
  286
 
  4
 
 25046
 
 island
 
 0.49928
 
 0.75700
 
 Not queried
 
  1.8085362
 
 2.86e-02
 
 1.00e+00
  
 
  
 7
 
 136849018
 
 136849317
 
  PDF   STATS   ENSEMBL   UCSC 
 
  300
 
  5
 
 38880
 
 island
 
 0.50388
 
 0.89569
 
 Not queried
 
  1.8085014
 
 9.52e-02
 
 1.00e+00
  
 
  
 1
 
  15256054
 
  15256222
 
  PDF   STATS   ENSEMBL   UCSC 
 
  169
 
  5
 
 239
 
 gene;tss
 
 0.54608
 
 0.82389
 
 Not queried
 
  1.8077759
 
 3.17e-02
 
 1.00e+00
  
 
  
 10
 
  63213104
 
  63213510
 
  PDF   STATS   ENSEMBL   UCSC 
 
  407
 
  7
 
 5749
 
 island
 
 0.04116
 
 0.22307
 
 Not queried
 
  1.8075928
 
 5.30e-02
 
 1.00e+00
  
 
  
 12
 
  91348999
 
  91349273
 
  PDF   STATS   ENSEMBL   UCSC 
 
  275
 
  7
 
 9886
 
 tss;island
 
 0.77283
 
 0.91122
 
 Not queried
 
  1.8074052
 
 9.73e-02
 
 1.00e+00
  
 
  
 5
 
 161494711
 
 161494810
 
  PDF   STATS   ENSEMBL   UCSC 
 
  100
 
  4
 
 31822
 
 gene
 
 0.22073
 
 0.41766
 
 Not queried
 
  1.8073856
 
 2.86e-02
 
 1.00e+00
  
 
  
 4
 
 119948360
 
 119948542
 
  PDF   STATS   ENSEMBL   UCSC 
 
  183
 
  4
 
 30061
 
 gene
 
 0.56808
 
 0.82753
 
 Not queried
 
  1.8066420
 
 2.00e-01
 
 1.00e+00
  
 
  
 2
 
  40739661
 
  40740063
 
  PDF   STATS   ENSEMBL   UCSC 
 
  403
 
  4
 
 23280
 
 tss
 
 0.20379
 
 0.45576
 
 Not queried
 
  1.8063481
 
 1.14e-01
 
 1.00e+00
  
 
  
 5
 
 101632286
 
 101632559
 
  PDF   STATS   ENSEMBL   UCSC 
 
  274
 
  9
 
 32139
 
 tss
 
 0.14716
 
 0.06779
 
 Not queried
 
  1.8059865
 
 6.25e-02
 
 1.00e+00
  
 
  
 14
 
 105041382
 
 105041708
 
  PDF   STATS   ENSEMBL   UCSC 
 
  327
 
  5
 
 13178
 
 island
 
 0.70349
 
 0.87454
 
 Not queried
 
  1.8057549
 
 1.51e-01
 
 1.00e+00
  
 
  
 19
 
  21950196
 
  21950478
 
  PDF   STATS   ENSEMBL   UCSC 
 
  283
 
  6
 
 21804
 
 island
 
 0.39810
 
 0.10549
 
 Not queried
 
  1.8053957
 
 6.49e-02
 
 1.00e+00
  
 
  
 6
 
  31847922
 
  31848099
 
  PDF   STATS   ENSEMBL   UCSC 
 
  178
 
  4
 
 33917
 
 gene;tss
 
 0.64971
 
 0.89993
 
 Not queried
 
  1.8053282
 
 2.86e-02
 
 1.00e+00
  
 
  
 11
 
  22214333
 
  22214683
 
  PDF   STATS   ENSEMBL   UCSC 
 
  351
 
  5
 
 7181
 
 tss
 
 0.05996
 
 0.10986
 
 Not queried
 
  1.8046576
 
 3.17e-02
 
 1.00e+00
  
 
  
 3
 
  53880717
 
  53880872
 
  PDF   STATS   ENSEMBL   UCSC 
 
  156
 
  4
 
 27790
 
 gene;tss;island
 
 0.20992
 
 0.06722
 
 Not queried
 
  1.8042296
 
 2.86e-02
 
 1.00e+00
  
 
  
 13
 
  92051400
 
  92051786
 
  PDF   STATS   ENSEMBL   UCSC 
 
  387
 
  4
 
 11161
 
 gene;island
 
 0.14622
 
 0.28864
 
 Not queried
 
  1.8037797
 
 4.86e-01
 
 1.00e+00
  
 
  
 1
 
  75593804
 
  75594261
 
  PDF   STATS   ENSEMBL   UCSC 
 
  458
 
  6
 
 3378
 
 island
 
 0.85057
 
 0.61325
 
 Not queried
 
  1.8034166
 
 2.60e-02
 
 1.00e+00
  
 
  
 5
 
 171616253
 
 171616574
 
  PDF   STATS   ENSEMBL   UCSC 
 
  322
 
  4
 
 32430
 
 tss;island
 
 0.14522
 
 0.04427
 
 Not queried
 
  1.8028736
 
 5.71e-02
 
 1.00e+00
  
 
  
 3
 
 127316944
 
 127317259
 
  PDF   STATS   ENSEMBL   UCSC 
 
  316
 
  7
 
 29402
 
 island
 
 0.11302
 
 0.03607
 
 Not queried
 
  1.8019754
 
 3.79e-02
 
 1.00e+00
  
 
  
 15
 
  79298209
 
  79298396
 
  PDF   STATS   ENSEMBL   UCSC 
 
  188
 
  4
 
 13416
 
 gene;tss
 
 0.89541
 
 0.73135
 
 Not queried
 
  1.8017475
 
 2.86e-02
 
 1.00e+00
  
 
  
 11
 
  65625941
 
  65626208
 
  PDF   STATS   ENSEMBL   UCSC 
 
  268
 
  7
 
 7423
 
 tss;island
 
 0.00783
 
 0.02116
 
 Not queried
 
  1.8016668
 
 4.56e-01
 
 1.00e+00
  
 
  
 8
 
 114447055
 
 114447871
 
  PDF   STATS   ENSEMBL   UCSC 
 
  817
 
  6
 
 39339
 
 gene;island
 
 0.00653
 
 0.01925
 
 Not queried
 
  1.8015337
 
 6.49e-02
 
 1.00e+00
  
 
  
 20
 
  42544648
 
  42544728
 
  PDF   STATS   ENSEMBL   UCSC 
 
   81
 
  6
 
 25714
 
 tss
 
 0.19976
 
 0.04120
 
 Not queried
 
  1.8013171
 
 1.32e-01
 
 1.00e+00
  
 
  
 22
 
  42475450
 
  42475749
 
  PDF   STATS   ENSEMBL   UCSC 
 
  300
 
  7
 
 27498
 
 island
 
 0.01198
 
 0.03734
 
 Not queried
 
  1.8008690
 
 5.30e-02
 
 1.00e+00
  
 
  
 11
 
  46354005
 
  46354296
 
  PDF   STATS   ENSEMBL   UCSC 
 
  292
 
  4
 
 7253
 
 tss;island
 
 0.25281
 
 0.06710
 
 Not queried
 
  1.8005564
 
 5.71e-02
 
 1.00e+00
  
 
  
 5
 
  87975798
 
  87976294
 
  PDF   STATS   ENSEMBL   UCSC 
 
  497
 
  5
 
 31624
 
 gene;island
 
 0.09482
 
 0.02278
 
 Not queried
 
  1.7995570
 
 2.22e-01
 
 1.00e+00
  
 
  
 1
 
  75593804
 
  75594066
 
  PDF   STATS   ENSEMBL   UCSC 
 
  263
 
  5
 
 1695
 
 tss
 
 0.84160
 
 0.59040
 
 Not queried
 
  1.7991688
 
 5.56e-02
 
 1.00e+00
  
 
  
 5
 
  95768997
 
  95769079
 
  PDF   STATS   ENSEMBL   UCSC 
 
   83
 
  6
 
 32130
 
 tss
 
 0.13400
 
 0.01917
 
 Not queried
 
  1.7989458
 
 1.80e-01
 
 1.00e+00
  
 
  
 8
 
 144798535
 
 144799022
 
  PDF   STATS   ENSEMBL   UCSC 
 
  488
 
  6
 
 39426
 
 gene
 
 0.12354
 
 0.04159
 
 Not queried
 
  1.7971475
 
 3.94e-01
 
 1.00e+00
  
 
  
 2
 
 232791732
 
 232792015
 
  PDF   STATS   ENSEMBL   UCSC 
 
  284
 
  5
 
 23909
 
 tss;island
 
 0.77605
 
 0.46773
 
 Not queried
 
  1.7971251
 
 7.94e-03
 
 1.00e+00
  
 
  
 2
 
  27665017
 
  27665306
 
  PDF   STATS   ENSEMBL   UCSC 
 
  290
 
  7
 
 24147
 
 island
 
 0.33683
 
 0.63831
 
 Not queried
 
  1.7968476
 
 5.83e-04
 
 1.00e+00
  
 
  
 1
 
  20692812
 
  20693027
 
  PDF   STATS   ENSEMBL   UCSC 
 
  216
 
  4
 
 2785
 
 island
 
 0.09655
 
 0.24304
 
 Not queried
 
  1.7964567
 
 2.86e-02
 
 1.00e+00
  
 
  
 19
 
   8274853
 
   8275229
 
  PDF   STATS   ENSEMBL   UCSC 
 
  377
 
  4
 
 19756
 
 gene;island
 
 0.28685
 
 0.11050
 
 Not queried
 
  1.7963737
 
 2.00e-01
 
 1.00e+00
  
 
  
 18
 
  12254365
 
  12254690
 
  PDF   STATS   ENSEMBL   UCSC 
 
  326
 
  9
 
 19027
 
 gene
 
 0.57487
 
 0.81215
 
 Not queried
 
  1.7962168
 
 3.15e-02
 
 1.00e+00
  
 
  
 6
 
  32797476
 
  32797830
 
  PDF   STATS   ENSEMBL   UCSC 
 
  355
 
  5
 
 34074
 
 gene
 
 0.49179
 
 0.71973
 
 Not queried
 
  1.7939979
 
 1.00e+00
 
 1.00e+00
  
 
  
 3
 
 186490656
 
 186490915
 
  PDF   STATS   ENSEMBL   UCSC 
 
  260
 
  5
 
 29708
 
 island
 
 0.37222
 
 0.70058
 
 Not queried
 
  1.7936045
 
 7.94e-03
 
 1.00e+00
  
 
  
 7
 
  44152996
 
  44153500
 
  PDF   STATS   ENSEMBL   UCSC 
 
  505
 
  6
 
 36927
 
 gene;island
 
 0.69841
 
 0.92168
 
 Not queried
 
  1.7923222
 
 6.49e-02
 
 1.00e+00
  
 
  
 10
 
  94455543
 
  94455895
 
  PDF   STATS   ENSEMBL   UCSC 
 
  353
 
  4
 
 5927
 
 island
 
 0.56951
 
 0.79034
 
 Not queried
 
  1.7918164
 
 2.86e-02
 
 1.00e+00
  
 
  
 8
 
   1364427
 
   1364689
 
  PDF   STATS   ENSEMBL   UCSC 
 
  263
 
  5
 
 39959
 
 island
 
 0.83269
 
 0.93345
 
 Not queried
 
  1.7906033
 
 2.22e-01
 
 1.00e+00
  
 
  
 15
 
  79382548
 
  79383167
 
  PDF   STATS   ENSEMBL   UCSC 
 
  620
 
  7
 
 13418
 
 gene;island
 
 0.01747
 
 0.03627
 
 Not queried
 
  1.7901682
 
 1.65e-01
 
 1.00e+00
  
 
  
 11
 
   8832111
 
   8832283
 
  PDF   STATS   ENSEMBL   UCSC 
 
  173
 
  4
 
 6481
 
 gene
 
 0.14729
 
 0.06550
 
 Not queried
 
  1.7900856
 
 2.86e-02
 
 1.00e+00
  
 
  
 16
 
  81254010
 
  81254209
 
  PDF   STATS   ENSEMBL   UCSC 
 
  200
 
  4
 
 15350
 
 tss
 
 0.79701
 
 0.45544
 
 Not queried
 
  1.7890076
 
 5.71e-02
 
 1.00e+00
  
 
  
 6
 
 101846650
 
 101846805
 
  PDF   STATS   ENSEMBL   UCSC 
 
  156
 
  7
 
 35079
 
 tss
 
 0.09792
 
 0.03167
 
 Not queried
 
  1.7888886
 
 9.73e-02
 
 1.00e+00
  
 
  
 8
 
  53852030
 
  53852446
 
  PDF   STATS   ENSEMBL   UCSC 
 
  417
 
  7
 
 39636
 
 tss;island
 
 0.02873
 
 0.08346
 
 Not queried
 
  1.7884824
 
 2.62e-02
 
 1.00e+00
  
 
  
 6
 
 137241923
 
 137242316
 
  PDF   STATS   ENSEMBL   UCSC 
 
  394
 
  5
 
 35181
 
 tss;island
 
 0.23498
 
 0.07598
 
 Not queried
 
  1.7881478
 
 9.52e-02
 
 1.00e+00
  
 
  
 4
 
  90757351
 
  90757814
 
  PDF   STATS   ENSEMBL   UCSC 
 
  464
 
  7
 
 30011
 
 gene;island
 
 0.04168
 
 0.19052
 
 Not queried
 
  1.7878709
 
 1.11e-02
 
 1.00e+00
  
 
  
 X
 
  69665036
 
  69665233
 
  PDF   STATS   ENSEMBL   UCSC 
 
  198
 
  4
 
 41017
 
 gene;island
 
 0.10931
 
 0.33266
 
 Not queried
 
  1.7866741
 
 5.71e-02
 
 1.00e+00
  
 
  
 3
 
  13974286
 
  13974800
 
  PDF   STATS   ENSEMBL   UCSC 
 
  515
 
  4
 
 28866
 
 island
 
 0.96320
 
 0.87359
 
 Not queried
 
  1.7865890
 
 2.00e-01
 
 1.00e+00
  
 
  
 8
 
 142183507
 
 142183860
 
  PDF   STATS   ENSEMBL   UCSC 
 
  354
 
  4
 
 39383
 
 gene;island
 
 0.43779
 
 0.76590
 
 Not queried
 
  1.7861486
 
 2.00e-01
 
 1.00e+00
  
 
  
 3
 
 178865550
 
 178866004
 
  PDF   STATS   ENSEMBL   UCSC 
 
  455
 
  5
 
 28684
 
 tss;island
 
 0.02271
 
 0.06012
 
 Not queried
 
  1.7861250
 
 1.51e-01
 
 1.00e+00
  
 
  
 7
 
  49815117
 
  49815938
 
  PDF   STATS   ENSEMBL   UCSC 
 
  822
 
  7
 
 36946
 
 gene;island
 
 0.58653
 
 0.25561
 
 Not queried
 
  1.7849808
 
 1.28e-01
 
 1.00e+00
  
 
  
 12
 
  66123127
 
  66123496
 
  PDF   STATS   ENSEMBL   UCSC 
 
  370
 
  4
 
 10573
 
 island
 
 0.15719
 
 0.03737
 
 Not queried
 
  1.7848360
 
 2.00e-01
 
 1.00e+00
  
 
  
 8
 
   8859686
 
   8860266
 
  PDF   STATS   ENSEMBL   UCSC 
 
  581
 
  7
 
 39479
 
 tss
 
 0.05894
 
 0.19934
 
 Not queried
 
  1.7843578
 
 3.83e-01
 
 1.00e+00
  
 
  
 7
 
 157368881
 
 157369158
 
  PDF   STATS   ENSEMBL   UCSC 
 
  278
 
  6
 
 37266
 
 gene;island
 
 0.52492
 
 0.89147
 
 Not queried
 
  1.7836790
 
 3.94e-01
 
 1.00e+00
  
 
  
 6
 
  28834254
 
  28834520
 
  PDF   STATS   ENSEMBL   UCSC 
 
  267
 
 11
 
 35537
 
 island
 
 0.32012
 
 0.61235
 
 Not queried
 
  1.7828487
 
 5.53e-04
 
 1.00e+00
  
 
  
 13
 
  50422122
 
  50422495
 
  PDF   STATS   ENSEMBL   UCSC 
 
  374
 
  4
 
 11708
 
 island
 
 0.01684
 
 0.02693
 
 Not queried
 
  1.7819955
 
 8.86e-01
 
 1.00e+00
  
 
  
 16
 
  12183835
 
  12184133
 
  PDF   STATS   ENSEMBL   UCSC 
 
  299
 
  5
 
 14639
 
 gene
 
 0.57953
 
 0.19287
 
 Not queried
 
  1.7819756
 
 3.17e-02
 
 1.00e+00
  
 
  
 1
 
 197744757
 
 197745238
 
  PDF   STATS   ENSEMBL   UCSC 
 
  482
 
  8
 
 2150
 
 tss;island
 
 0.13803
 
 0.08061
 
 Not queried
 
  1.7818600
 
 1.61e-01
 
 1.00e+00
  
 
  
 19
 
  46056723
 
  46057029
 
  PDF   STATS   ENSEMBL   UCSC 
 
  307
 
  4
 
 20065
 
 gene;island
 
 0.73459
 
 0.86422
 
 Not queried
 
  1.7816995
 
 2.86e-02
 
 1.00e+00
  
 
  
 6
 
  33943520
 
  33943929
 
  PDF   STATS   ENSEMBL   UCSC 
 
  410
 
  5
 
 35986
 
 island
 
 0.66591
 
 0.88894
 
 Not queried
 
  1.7816036
 
 1.59e-02
 
 1.00e+00
  
 
  
 13
 
 100624207
 
 100624373
 
  PDF   STATS   ENSEMBL   UCSC 
 
  167
 
  7
 
 11487
 
 tss
 
 0.11964
 
 0.03117
 
 Not queried
 
  1.7812985
 
 1.28e-01
 
 1.00e+00
  
 
  
 13
 
 100623942
 
 100624373
 
  PDF   STATS   ENSEMBL   UCSC 
 
  432
 
  9
 
 11816
 
 island
 
 0.11964
 
 0.02298
 
 Not queried
 
  1.7812985
 
 4.00e-02
 
 1.00e+00
  
 
  
 11
 
  14664598
 
  14665070
 
  PDF   STATS   ENSEMBL   UCSC 
 
  473
 
  7
 
 6498
 
 gene;tss;island
 
 0.28752
 
 0.10405
 
 Not queried
 
  1.7812695
 
 3.18e-01
 
 1.00e+00
  
 
  
 X
 
  17879424
 
  17879951
 
  PDF   STATS   ENSEMBL   UCSC 
 
  528
 
  6
 
 41670
 
 island
 
 0.07282
 
 0.07928
 
 Not queried
 
  1.7800469
 
 5.89e-01
 
 1.00e+00
  
 
  
 12
 
 132923339
 
 132923603
 
  PDF   STATS   ENSEMBL   UCSC 
 
  265
 
  4
 
 11017
 
 island
 
 0.60952
 
 0.83299
 
 Not queried
 
  1.7792274
 
 2.00e-01
 
 1.00e+00
  
 
  
 3
 
  71180032
 
  71180071
 
  PDF   STATS   ENSEMBL   UCSC 
 
   40
 
  4
 
 27816
 
 gene
 
 0.66689
 
 0.77177
 
 Not queried
 
  1.7786762
 
 2.00e-01
 
 1.00e+00
  
 
  
 17
 
  73893198
 
  73893209
 
  PDF   STATS   ENSEMBL   UCSC 
 
   12
 
  4
 
 17632
 
 tss
 
 0.05004
 
 0.14644
 
 Not queried
 
  1.7784689
 
 1.14e-01
 
 1.00e+00
  
 
  
 17
 
   4872326
 
   4872603
 
  PDF   STATS   ENSEMBL   UCSC 
 
  278
 
  4
 
 16325
 
 gene;tss;island
 
 0.76637
 
 0.60068
 
 Not queried
 
  1.7783487
 
 1.14e-01
 
 1.00e+00
  
 
  
 1
 
 156696747
 
 156697041
 
  PDF   STATS   ENSEMBL   UCSC 
 
  295
 
  4
 
 826
 
 gene;island
 
 0.29583
 
 0.51429
 
 Not queried
 
  1.7782638
 
 1.14e-01
 
 1.00e+00
  
 
  
 17
 
   1810459
 
   1810853
 
  PDF   STATS   ENSEMBL   UCSC 
 
  395
 
  5
 
 17799
 
 island
 
 0.58423
 
 0.79278
 
 Not queried
 
  1.7779471
 
 3.17e-02
 
 1.00e+00
  
 
  
 1
 
   6260181
 
   6260373
 
  PDF   STATS   ENSEMBL   UCSC 
 
  193
 
  4
 
 1196
 
 tss;island
 
 0.48522
 
 0.14986
 
 Not queried
 
  1.7777828
 
 3.43e-01
 
 1.00e+00
  
 
  
 1
 
 245753302
 
 245753635
 
  PDF   STATS   ENSEMBL   UCSC 
 
  334
 
  4
 
 1115
 
 gene;island
 
 0.58223
 
 0.82672
 
 Not queried
 
  1.7772866
 
 2.00e-01
 
 1.00e+00
  
 
  
 3
 
 147140754
 
 147140930
 
  PDF   STATS   ENSEMBL   UCSC 
 
  177
 
  4
 
 29532
 
 island
 
 0.08855
 
 0.03780
 
 Not queried
 
  1.7770341
 
 5.71e-02
 
 1.00e+00
  
 
  
 19
 
  58629719
 
  58630612
 
  PDF   STATS   ENSEMBL   UCSC 
 
  894
 
 10
 
 22457
 
 island
 
 0.46031
 
 0.66838
 
 Not queried
 
  1.7760520
 
 7.53e-02
 
 1.00e+00
  
 
  
 1
 
  47082037
 
  47082372
 
  PDF   STATS   ENSEMBL   UCSC 
 
  336
 
  5
 
 511
 
 gene;island
 
 0.18420
 
 0.09447
 
 Not queried
 
  1.7759483
 
 1.51e-01
 
 1.00e+00
  
 
  
 11
 
  20184128
 
  20184779
 
  PDF   STATS   ENSEMBL   UCSC 
 
  652
 
  7
 
 8104
 
 island
 
 0.12325
 
 0.04808
 
 Not queried
 
  1.7745271
 
 7.28e-02
 
 1.00e+00
  
 
  
 15
 
  79298604
 
  79298769
 
  PDF   STATS   ENSEMBL   UCSC 
 
  166
 
  8
 
 13417
 
 gene;tss
 
 0.91532
 
 0.76530
 
 Not queried
 
  1.7744917
 
 6.22e-04
 
 1.00e+00
  
 
  
 14
 
  88792913
 
  88793252
 
  PDF   STATS   ENSEMBL   UCSC 
 
  340
 
  6
 
 12138
 
 gene
 
 0.04774
 
 0.20183
 
 Not queried
 
  1.7744893
 
 2.60e-02
 
 1.00e+00
  
 
  
 4
 
   4250464
 
   4250746
 
  PDF   STATS   ENSEMBL   UCSC 
 
  283
 
  4
 
 30218
 
 tss;island
 
 0.10215
 
 0.02785
 
 Not queried
 
  1.7744212
 
 8.86e-01
 
 1.00e+00
  
 
  
 2
 
 119603383
 
 119603969
 
  PDF   STATS   ENSEMBL   UCSC 
 
  587
 
  5
 
 22804
 
 gene;island
 
 0.38442
 
 0.15438
 
 Not queried
 
  1.7742858
 
 6.90e-01
 
 1.00e+00
  
 
  
 3
 
 164914996
 
 164915196
 
  PDF   STATS   ENSEMBL   UCSC 
 
  201
 
  5
 
 28657
 
 tss
 
 0.13182
 
 0.22455
 
 Not queried
 
  1.7734555
 
 1.51e-01
 
 1.00e+00
  
 
  
 4
 
  38869118
 
  38869779
 
  PDF   STATS   ENSEMBL   UCSC 
 
  662
 
 14
 
 30929
 
 island
 
 0.12378
 
 0.02719
 
 Not queried
 
  1.7734108
 
 4.44e-02
 
 1.00e+00
  
 
  
 17
 
  79412583
 
  79412788
 
  PDF   STATS   ENSEMBL   UCSC 
 
  206
 
  4
 
 16877
 
 gene;island
 
 0.83290
 
 0.94315
 
 Not queried
 
  1.7733162
 
 2.86e-02
 
 1.00e+00
  
 
  
 5
 
  32709071
 
  32709396
 
  PDF   STATS   ENSEMBL   UCSC 
 
  326
 
  4
 
 32657
 
 island
 
 0.79269
 
 0.46239
 
 Not queried
 
  1.7731887
 
 5.71e-02
 
 1.00e+00
  
 
  
 16
 
  67232180
 
  67232557
 
  PDF   STATS   ENSEMBL   UCSC 
 
  378
 
  4
 
 14763
 
 gene;tss;island
 
 0.67488
 
 0.86604
 
 Not queried
 
  1.7730190
 
 5.71e-02
 
 1.00e+00
  
 
  
 17
 
   7486551
 
   7486961
 
  PDF   STATS   ENSEMBL   UCSC 
 
  411
 
  9
 
 17057
 
 tss
 
 0.40301
 
 0.31169
 
 Not queried
 
  1.7728597
 
 2.58e-01
 
 1.00e+00
  
 
  
 12
 
 106533667
 
 106533903
 
  PDF   STATS   ENSEMBL   UCSC 
 
  237
 
  6
 
 10720
 
 island
 
 0.66989
 
 0.38914
 
 Not queried
 
  1.7726795
 
 8.66e-03
 
 1.00e+00
  
 
  
 4
 
   6695203
 
   6695475
 
  PDF   STATS   ENSEMBL   UCSC 
 
  273
 
  6
 
 30236
 
 tss
 
 0.70120
 
 0.88911
 
 Not queried
 
  1.7723759
 
 2.16e-03
 
 1.00e+00
  
 
  
 6
 
  30226874
 
  30227326
 
  PDF   STATS   ENSEMBL   UCSC 
 
  453
 
 10
 
 34702
 
 tss
 
 0.15323
 
 0.04412
 
 Not queried
 
  1.7713104
 
 1.05e-01
 
 1.00e+00
  
 
  
 2
 
  63272214
 
  63272334
 
  PDF   STATS   ENSEMBL   UCSC 
 
  121
 
  4
 
 22665
 
 gene;island
 
 0.36736
 
 0.64386
 
 Not queried
 
  1.7701605
 
 2.86e-02
 
 1.00e+00
  
 
  
 2
 
 132233433
 
 132233607
 
  PDF   STATS   ENSEMBL   UCSC 
 
  175
 
  4
 
 23596
 
 tss
 
 0.96146
 
 0.88058
 
 Not queried
 
  1.7696729
 
 3.43e-01
 
 1.00e+00
  
 
  
 11
 
  47587211
 
  47587672
 
  PDF   STATS   ENSEMBL   UCSC 
 
  462
 
  4
 
 6591
 
 gene;island
 
 0.01508
 
 0.05334
 
 Not queried
 
  1.7691291
 
 1.14e-01
 
 1.00e+00
  
 
  
 12
 
 110888206
 
 110888378
 
  PDF   STATS   ENSEMBL   UCSC 
 
  173
 
  4
 
 9978
 
 tss
 
 0.02088
 
 0.04335
 
 Not queried
 
  1.7681708
 
 4.86e-01
 
 1.00e+00
  
 
  
 X
 
  49087234
 
  49087477
 
  PDF   STATS   ENSEMBL   UCSC 
 
  244
 
  4
 
 40994
 
 gene;island
 
 0.05276
 
 0.02586
 
 Not queried
 
  1.7676534
 
 1.14e-01
 
 1.00e+00
  
 
  
 19
 
  11494980
 
  11495295
 
  PDF   STATS   ENSEMBL   UCSC 
 
  316
 
  4
 
 21530
 
 island
 
 0.33550
 
 0.10042
 
 Not queried
 
  1.7670596
 
 4.86e-01
 
 1.00e+00
  
 
  
 4
 
 144258809
 
 144259119
 
  PDF   STATS   ENSEMBL   UCSC 
 
  311
 
  4
 
 30094
 
 gene;island
 
 0.02772
 
 0.03037
 
 Not queried
 
  1.7666236
 
 6.86e-01
 
 1.00e+00
  
 
  
 15
 
  96868857
 
  96869220
 
  PDF   STATS   ENSEMBL   UCSC 
 
  364
 
  8
 
 14457
 
 island
 
 0.02803
 
 0.21854
 
 Not queried
 
  1.7662180
 
 2.07e-02
 
 1.00e+00
  
 
  
 6
 
  29973221
 
  29973669
 
  PDF   STATS   ENSEMBL   UCSC 
 
  449
 
  5
 
 33642
 
 gene;tss;island
 
 0.77833
 
 0.93528
 
 Not queried
 
  1.7660310
 
 9.52e-02
 
 1.00e+00
  
 
  
 5
 
 112824497
 
 112824920
 
  PDF   STATS   ENSEMBL   UCSC 
 
  424
 
  7
 
 32951
 
 island
 
 0.44245
 
 0.62444
 
 Not queried
 
  1.7659283
 
 3.83e-01
 
 1.00e+00
  
 
  
 5
 
   1813772
 
   1814201
 
  PDF   STATS   ENSEMBL   UCSC 
 
  430
 
  5
 
 31492
 
 gene
 
 0.64523
 
 0.88810
 
 Not queried
 
  1.7657823
 
 1.59e-02
 
 1.00e+00
  
 
  
 19
 
  21579787
 
  21579911
 
  PDF   STATS   ENSEMBL   UCSC 
 
  125
 
  6
 
 20624
 
 tss
 
 0.03751
 
 0.02298
 
 Not queried
 
  1.7656273
 
 1.80e-01
 
 1.00e+00
  
 
  
 11
 
    442018
 
    442380
 
  PDF   STATS   ENSEMBL   UCSC 
 
  363
 
  6
 
 6992
 
 tss
 
 0.52191
 
 0.74533
 
 Not queried
 
  1.7649356
 
 2.16e-03
 
 1.00e+00
  
 
  
 18
 
  72201716
 
  72201785
 
  PDF   STATS   ENSEMBL   UCSC 
 
   70
 
  4
 
 19094
 
 gene
 
 0.75111
 
 0.92419
 
 Not queried
 
  1.7635151
 
 1.14e-01
 
 1.00e+00
  
 
  
 17
 
   7832764
 
   7832943
 
  PDF   STATS   ENSEMBL   UCSC 
 
  180
 
  6
 
 17070
 
 tss
 
 0.08595
 
 0.02647
 
 Not queried
 
  1.7634884
 
 2.60e-02
 
 1.00e+00
  
 
  
 10
 
 134994186
 
 134994455
 
  PDF   STATS   ENSEMBL   UCSC 
 
  270
 
  5
 
 4873
 
 gene;island
 
 0.93225
 
 0.90889
 
 Not queried
 
  1.7633014
 
 6.90e-01
 
 1.00e+00
  
 
  
 6
 
  74072242
 
  74072376
 
  PDF   STATS   ENSEMBL   UCSC 
 
  135
 
  6
 
 35003
 
 tss
 
 0.64418
 
 0.86504
 
 Not queried
 
  1.7630745
 
 1.52e-02
 
 1.00e+00
  
 
  
 12
 
 130645423
 
 130645645
 
  PDF   STATS   ENSEMBL   UCSC 
 
  223
 
  4
 
 10970
 
 island
 
 0.06471
 
 0.02170
 
 Not queried
 
  1.7630213
 
 1.14e-01
 
 1.00e+00
  
 
  
 7
 
  87936225
 
  87936414
 
  PDF   STATS   ENSEMBL   UCSC 
 
  190
 
  5
 
 37626
 
 tss
 
 0.03525
 
 0.11144
 
 Not queried
 
  1.7626711
 
 2.22e-01
 
 1.00e+00
  
 
  
 3
 
 122399230
 
 122400006
 
  PDF   STATS   ENSEMBL   UCSC 
 
  777
 
 10
 
 29360
 
 island
 
 0.31332
 
 0.62739
 
 Not queried
 
  1.7622173
 
 7.53e-02
 
 1.00e+00
  
 
  
 19
 
  49568000
 
  49568214
 
  PDF   STATS   ENSEMBL   UCSC 
 
  215
 
  4
 
 20902
 
 tss
 
 0.06945
 
 0.16191
 
 Not queried
 
  1.7609322
 
 5.71e-02
 
 1.00e+00
  
 
  
 17
 
  48042605
 
  48043063
 
  PDF   STATS   ENSEMBL   UCSC 
 
  459
 
  4
 
 18499
 
 island
 
 0.57636
 
 0.26799
 
 Not queried
 
  1.7609210
 
 2.86e-02
 
 1.00e+00
  
 
  
 1
 
   3230250
 
   3230647
 
  PDF   STATS   ENSEMBL   UCSC 
 
  398
 
  5
 
 118
 
 gene
 
 0.45684
 
 0.75256
 
 Not queried
 
  1.7605632
 
 9.52e-02
 
 1.00e+00
  
 
  
 1
 
 236511281
 
 236511627
 
  PDF   STATS   ENSEMBL   UCSC 
 
  347
 
  4
 
 4347
 
 island
 
 0.58392
 
 0.85729
 
 Not queried
 
  1.7604292
 
 8.86e-01
 
 1.00e+00
  
 
  
 X
 
  48432846
 
  48433316
 
  PDF   STATS   ENSEMBL   UCSC 
 
  471
 
  6
 
 40984
 
 gene
 
 0.21269
 
 0.57303
 
 Not queried
 
  1.7602856
 
 8.66e-03
 
 1.00e+00
  
 
  
 X
 
 133370784
 
 133371052
 
  PDF   STATS   ENSEMBL   UCSC 
 
  269
 
  6
 
 41527
 
 tss
 
 0.31034
 
 0.07082
 
 Not queried
 
  1.7589546
 
 9.31e-02
 
 1.00e+00
  
 
  
 11
 
   1221398
 
   1221812
 
  PDF   STATS   ENSEMBL   UCSC 
 
  415
 
  4
 
 7885
 
 island
 
 0.53706
 
 0.77373
 
 Not queried
 
  1.7586200
 
 2.00e-01
 
 1.00e+00
  
 
  
 21
 
  34638126
 
  34638662
 
  PDF   STATS   ENSEMBL   UCSC 
 
  537
 
  7
 
 26478
 
 tss;island
 
 0.02173
 
 0.05300
 
 Not queried
 
  1.7584906
 
 9.73e-02
 
 1.00e+00
  
 
  
 11
 
  47586666
 
  47586994
 
  PDF   STATS   ENSEMBL   UCSC 
 
  329
 
  4
 
 8245
 
 island
 
 0.26358
 
 0.03758
 
 Not queried
 
  1.7583536
 
 2.00e-01
 
 1.00e+00
  
 
  
 2
 
  97173852
 
  97174391
 
  PDF   STATS   ENSEMBL   UCSC 
 
  540
 
 10
 
 23479
 
 tss
 
 0.05044
 
 0.17838
 
 Not queried
 
  1.7583149
 
 3.55e-02
 
 1.00e+00
  
 
  
 17
 
  48171802
 
  48172203
 
  PDF   STATS   ENSEMBL   UCSC 
 
  402
 
  7
 
 17458
 
 tss;island
 
 0.02532
 
 0.07939
 
 Not queried
 
  1.7576457
 
 7.28e-02
 
 1.00e+00
  
 
  
 1
 
    713921
 
    714177
 
  PDF   STATS   ENSEMBL   UCSC 
 
  257
 
  6
 
 2372
 
 island
 
 0.03709
 
 0.15287
 
 Not queried
 
  1.7576199
 
 4.11e-02
 
 1.00e+00
  
 
  
 17
 
  79010831
 
  79011542
 
  PDF   STATS   ENSEMBL   UCSC 
 
  712
 
  6
 
 16855
 
 gene;island
 
 0.62874
 
 0.94100
 
 Not queried
 
  1.7574283
 
 2.60e-02
 
 1.00e+00
  
 
  
 11
 
    537287
 
    537737
 
  PDF   STATS   ENSEMBL   UCSC 
 
  451
 
 11
 
 7837
 
 island
 
 0.26244
 
 0.03578
 
 Not queried
 
  1.7569473
 
 3.36e-02
 
 1.00e+00
  
 
  
 13
 
 112630296
 
 112630795
 
  PDF   STATS   ENSEMBL   UCSC 
 
  500
 
  7
 
 11875
 
 island
 
 0.82572
 
 0.93603
 
 Not queried
 
  1.7563542
 
 1.28e-01
 
 1.00e+00
  
 
  
 19
 
  48897863
 
  48898160
 
  PDF   STATS   ENSEMBL   UCSC 
 
  298
 
  4
 
 22179
 
 island
 
 0.07439
 
 0.36681
 
 Not queried
 
  1.7551831
 
 1.14e-01
 
 1.00e+00
  
 
  
 11
 
  64703415
 
  64703818
 
  PDF   STATS   ENSEMBL   UCSC 
 
  404
 
  5
 
 7388
 
 tss
 
 0.38197
 
 0.67586
 
 Not queried
 
  1.7542977
 
 5.48e-01
 
 1.00e+00
  
 
  
 6
 
 108436768
 
 108437116
 
  PDF   STATS   ENSEMBL   UCSC 
 
  349
 
  5
 
 36329
 
 island
 
 0.02812
 
 0.08165
 
 Not queried
 
  1.7539872
 
 7.94e-03
 
 1.00e+00
  
 
  
 2
 
 228028601
 
 228028776
 
  PDF   STATS   ENSEMBL   UCSC 
 
  176
 
  4
 
 23020
 
 gene;tss;island
 
 0.03761
 
 0.03335
 
 Not queried
 
  1.7539701
 
 8.86e-01
 
 1.00e+00
  
 
  
 8
 
  23567310
 
  23567941
 
  PDF   STATS   ENSEMBL   UCSC 
 
  632
 
  7
 
 40092
 
 island
 
 0.08131
 
 0.05976
 
 Not queried
 
  1.7535675
 
 3.83e-01
 
 1.00e+00
  
 
  
 3
 
 197183271
 
 197183662
 
  PDF   STATS   ENSEMBL   UCSC 
 
  392
 
  6
 
 29773
 
 island
 
 0.18106
 
 0.45247
 
 Not queried
 
  1.7532051
 
 1.52e-02
 
 1.00e+00
  
 
  
 19
 
  44009019
 
  44009073
 
  PDF   STATS   ENSEMBL   UCSC 
 
   55
 
  5
 
 20793
 
 tss;island
 
 0.45241
 
 0.18037
 
 Not queried
 
  1.7527631
 
 9.52e-02
 
 1.00e+00
  
 
  
 1
 
  47082691
 
  47082869
 
  PDF   STATS   ENSEMBL   UCSC 
 
  179
 
  5
 
 1600
 
 tss;island
 
 0.65430
 
 0.30510
 
 Not queried
 
  1.7519031
 
 1.51e-01
 
 1.00e+00
  
 
  
 7
 
  94536541
 
  94537033
 
  PDF   STATS   ENSEMBL   UCSC 
 
  493
 
  6
 
 38610
 
 island
 
 0.72453
 
 0.35064
 
 Not queried
 
  1.7514498
 
 3.10e-01
 
 1.00e+00
  
 
  
 X
 
  68723670
 
  68723812
 
  PDF   STATS   ENSEMBL   UCSC 
 
  143
 
  4
 
 41361
 
 tss;island
 
 0.07791
 
 0.02425
 
 Not queried
 
  1.7511477
 
 2.86e-02
 
 1.00e+00
  
 
  
 4
 
 106816089
 
 106816564
 
  PDF   STATS   ENSEMBL   UCSC 
 
  476
 
  5
 
 30484
 
 tss;island
 
 0.21926
 
 0.07704
 
 Not queried
 
  1.7503815
 
 2.22e-01
 
 1.00e+00
  
 
  
 5
 
 131892242
 
 131892523
 
  PDF   STATS   ENSEMBL   UCSC 
 
  282
 
  5
 
 32213
 
 tss
 
 0.46355
 
 0.79183
 
 Not queried
 
  1.7502392
 
 1.51e-01
 
 1.00e+00
  
 
  
 17
 
  48585470
 
  48585645
 
  PDF   STATS   ENSEMBL   UCSC 
 
  176
 
  4
 
 17468
 
 tss;island
 
 0.06125
 
 0.02973
 
 Not queried
 
  1.7499454
 
 2.86e-02
 
 1.00e+00
  
 
  
 6
 
  13274151
 
  13274354
 
  PDF   STATS   ENSEMBL   UCSC 
 
  204
 
  5
 
 33511
 
 gene
 
 0.63836
 
 0.84078
 
 Not queried
 
  1.7498660
 
 3.17e-02
 
 1.00e+00
  
 
  
 17
 
  77194008
 
  77194259
 
  PDF   STATS   ENSEMBL   UCSC 
 
  252
 
  4
 
 16802
 
 gene;island
 
 0.63159
 
 0.85171
 
 Not queried
 
  1.7494740
 
 2.86e-02
 
 1.00e+00
  
 
  
 X
 
  68385381
 
  68385467
 
  PDF   STATS   ENSEMBL   UCSC 
 
   87
 
  4
 
 41360
 
 tss
 
 0.08072
 
 0.02004
 
 Not queried
 
  1.7494284
 
 3.43e-01
 
 1.00e+00
  
 
  
 18
 
  76160108
 
  76160443
 
  PDF   STATS   ENSEMBL   UCSC 
 
  336
 
  5
 
 19555
 
 island
 
 0.54030
 
 0.79804
 
 Not queried
 
  1.7492950
 
 1.59e-02
 
 1.00e+00
  
 
  
 8
 
   2242024
 
   2242304
 
  PDF   STATS   ENSEMBL   UCSC 
 
  281
 
  4
 
 39986
 
 island
 
 0.48577
 
 0.75479
 
 Not queried
 
  1.7490087
 
 2.00e-01
 
 1.00e+00
  
 
  
 13
 
 113698931
 
 113699016
 
  PDF   STATS   ENSEMBL   UCSC 
 
   86
 
  7
 
 11243
 
 gene;island
 
 0.67088
 
 0.88203
 
 Not queried
 
  1.7487596
 
 4.08e-03
 
 1.00e+00
  
 
  
 14
 
  24803873
 
  24804342
 
  PDF   STATS   ENSEMBL   UCSC 
 
  470
 
  8
 
 12300
 
 tss
 
 0.03237
 
 0.14257
 
 Not queried
 
  1.7484829
 
 1.09e-03
 
 1.00e+00
  
 
  
 6
 
  32157454
 
  32157596
 
  PDF   STATS   ENSEMBL   UCSC 
 
  143
 
  6
 
 34019
 
 gene
 
 0.23542
 
 0.48648
 
 Not queried
 
  1.7481994
 
 1.80e-01
 
 1.00e+00
  
 
  
 14
 
  32546448
 
  32546633
 
  PDF   STATS   ENSEMBL   UCSC 
 
  186
 
  4
 
 12316
 
 tss;island
 
 0.02330
 
 0.08330
 
 Not queried
 
  1.7465297
 
 1.14e-01
 
 1.00e+00
  
 
  
 1
 
 205745431
 
 205745761
 
  PDF   STATS   ENSEMBL   UCSC 
 
  331
 
  5
 
 2204
 
 tss;island
 
 0.97077
 
 0.88542
 
 Not queried
 
  1.7457579
 
 5.56e-02
 
 1.00e+00
  
 
  
 7
 
 150019955
 
 150020269
 
  PDF   STATS   ENSEMBL   UCSC 
 
  315
 
  9
 
 37910
 
 tss
 
 0.33682
 
 0.15736
 
 Not queried
 
  1.7450054
 
 4.94e-04
 
 1.00e+00
  
 
  
 17
 
  38118571
 
  38118787
 
  PDF   STATS   ENSEMBL   UCSC 
 
  217
 
  4
 
 17285
 
 tss
 
 0.76359
 
 0.89466
 
 Not queried
 
  1.7442304
 
 4.86e-01
 
 1.00e+00
  
 
  
 1
 
  35395541
 
  35395837
 
  PDF   STATS   ENSEMBL   UCSC 
 
  297
 
  4
 
 3024
 
 island
 
 0.08527
 
 0.02567
 
 Not queried
 
  1.7438749
 
 4.86e-01
 
 1.00e+00
  
 
  
 1
 
 207277018
 
 207277581
 
  PDF   STATS   ENSEMBL   UCSC 
 
  564
 
  8
 
 2215
 
 tss
 
 0.68472
 
 0.84385
 
 Not queried
 
  1.7435222
 
 2.79e-01
 
 1.00e+00
  
 
  
 3
 
 136751342
 
 136751795
 
  PDF   STATS   ENSEMBL   UCSC 
 
  454
 
  5
 
 29468
 
 island
 
 0.04408
 
 0.16245
 
 Not queried
 
  1.7430997
 
 9.52e-02
 
 1.00e+00
  
 
  
 X
 
  16888414
 
  16888622
 
  PDF   STATS   ENSEMBL   UCSC 
 
  209
 
  4
 
 41664
 
 island
 
 0.01454
 
 0.03743
 
 Not queried
 
  1.7427307
 
 5.71e-02
 
 1.00e+00
  
 
  
 X
 
  13587976
 
  13588301
 
  PDF   STATS   ENSEMBL   UCSC 
 
  326
 
  4
 
 40939
 
 gene;island
 
 0.03131
 
 0.05188
 
 Not queried
 
  1.7424451
 
 4.86e-01
 
 1.00e+00
  
 
  
 10
 
  12110577
 
  12110759
 
  PDF   STATS   ENSEMBL   UCSC 
 
  183
 
  6
 
 4933
 
 tss
 
 0.01665
 
 0.03564
 
 Not queried
 
  1.7420516
 
 3.10e-01
 
 1.00e+00
  
 
  
 10
 
  81892109
 
  81892161
 
  PDF   STATS   ENSEMBL   UCSC 
 
   53
 
  4
 
 5169
 
 tss;island
 
 0.53282
 
 0.25852
 
 Not queried
 
  1.7418456
 
 2.86e-02
 
 1.00e+00
  
 
  
 12
 
  54329854
 
  54330354
 
  PDF   STATS   ENSEMBL   UCSC 
 
  501
 
  5
 
 10450
 
 island
 
 0.83055
 
 0.59328
 
 Not queried
 
  1.7417190
 
 1.51e-01
 
 1.00e+00
  
 
  
 14
 
  65239327
 
  65239653
 
  PDF   STATS   ENSEMBL   UCSC 
 
  327
 
  4
 
 12087
 
 gene;island
 
 0.57395
 
 0.88451
 
 Not queried
 
  1.7414076
 
 5.71e-02
 
 1.00e+00
  
 
  
 16
 
  31228427
 
  31228555
 
  PDF   STATS   ENSEMBL   UCSC 
 
  129
 
  4
 
 15179
 
 tss
 
 0.14497
 
 0.28756
 
 Not queried
 
  1.7396869
 
 2.86e-02
 
 1.00e+00
  
 
  
 11
 
    598325
 
    598696
 
  PDF   STATS   ENSEMBL   UCSC 
 
  372
 
  4
 
 6364
 
 gene;island
 
 0.70420
 
 0.91248
 
 Not queried
 
  1.7385914
 
 2.86e-02
 
 1.00e+00
  
 
  
 15
 
 100533202
 
 100533336
 
  PDF   STATS   ENSEMBL   UCSC 
 
  135
 
  4
 
 13475
 
 gene;island
 
 0.88860
 
 0.66543
 
 Not queried
 
  1.7382892
 
 2.00e-01
 
 1.00e+00
  
 
  
 11
 
 134147143
 
 134147634
 
  PDF   STATS   ENSEMBL   UCSC 
 
  492
 
  4
 
 6974
 
 gene;island
 
 0.15991
 
 0.03485
 
 Not queried
 
  1.7380120
 
 5.71e-02
 
 1.00e+00
  
 
  
 10
 
  27609163
 
  27609333
 
  PDF   STATS   ENSEMBL   UCSC 
 
  171
 
  4
 
 5611
 
 island
 
 0.39201
 
 0.55679
 
 Not queried
 
  1.7369866
 
 6.86e-01
 
 1.00e+00
  
 
  
 6
 
  31922863
 
  31923212
 
  PDF   STATS   ENSEMBL   UCSC 
 
  350
 
  4
 
 33936
 
 gene;island
 
 0.69860
 
 0.91201
 
 Not queried
 
  1.7363546
 
 5.71e-02
 
 1.00e+00
  
 
  
 19
 
   3062808
 
   3062986
 
  PDF   STATS   ENSEMBL   UCSC 
 
  179
 
  4
 
 20302
 
 tss;island
 
 0.48747
 
 0.16063
 
 Not queried
 
  1.7358760
 
 1.14e-01
 
 1.00e+00
  
 
  
 17
 
   5402883
 
   5403516
 
  PDF   STATS   ENSEMBL   UCSC 
 
  634
 
  6
 
 16332
 
 gene;island
 
 0.41635
 
 0.11760
 
 Not queried
 
  1.7355918
 
 2.40e-01
 
 1.00e+00
  
 
  
 1
 
  35220224
 
  35220710
 
  PDF   STATS   ENSEMBL   UCSC 
 
  487
 
  6
 
 1475
 
 tss
 
 0.81450
 
 0.52442
 
 Not queried
 
  1.7347821
 
 1.52e-02
 
 1.00e+00
  
 
  
 10
 
  11344086
 
  11344395
 
  PDF   STATS   ENSEMBL   UCSC 
 
  310
 
  4
 
 4481
 
 gene
 
 0.96845
 
 0.89516
 
 Not queried
 
  1.7347424
 
 2.86e-02
 
 1.00e+00
  
 
  
 1
 
  85512957
 
  85513315
 
  PDF   STATS   ENSEMBL   UCSC 
 
  359
 
  4
 
 603
 
 gene;island
 
 0.53259
 
 0.26760
 
 Not queried
 
  1.7345241
 
 1.14e-01
 
 1.00e+00
  
 
  
 1
 
  29587088
 
  29587490
 
  PDF   STATS   ENSEMBL   UCSC 
 
  403
 
  5
 
 376
 
 gene;island
 
 0.96602
 
 0.89426
 
 Not queried
 
  1.7334592
 
 5.56e-02
 
 1.00e+00
  
 
  
 17
 
  78388520
 
  78388593
 
  PDF   STATS   ENSEMBL   UCSC 
 
   74
 
  4
 
 16832
 
 gene;tss;island
 
 0.04052
 
 0.11816
 
 Not queried
 
  1.7327401
 
 5.71e-02
 
 1.00e+00
  
 
  
 7
 
  75932840
 
  75932977
 
  PDF   STATS   ENSEMBL   UCSC 
 
  138
 
  6
 
 36990
 
 gene;island
 
 0.02140
 
 0.09785
 
 Not queried
 
  1.7320367
 
 8.66e-03
 
 1.00e+00
  
 
  
 13
 
  33780205
 
  33780362
 
  PDF   STATS   ENSEMBL   UCSC 
 
  158
 
  4
 
 11358
 
 tss
 
 0.80551
 
 0.55617
 
 Not queried
 
  1.7294104
 
 2.86e-02
 
 1.00e+00
  
 
  
 6
 
  33169975
 
  33170452
 
  PDF   STATS   ENSEMBL   UCSC 
 
  478
 
  6
 
 34158
 
 gene;island
 
 0.46920
 
 0.75947
 
 Not queried
 
  1.7287787
 
 6.49e-02
 
 1.00e+00
  
 
  
 7
 
   1315546
 
   1315866
 
  PDF   STATS   ENSEMBL   UCSC 
 
  321
 
  4
 
 38032
 
 island
 
 0.60132
 
 0.92973
 
 Not queried
 
  1.7287515
 
 2.00e-01
 
 1.00e+00
  
 
  
 4
 
   2930073
 
   2930361
 
  PDF   STATS   ENSEMBL   UCSC 
 
  289
 
  4
 
 29850
 
 gene;island
 
 0.89904
 
 0.94246
 
 Not queried
 
  1.7276563
 
 2.00e-01
 
 1.00e+00
  
 
  
 X
 
 108778793
 
 108779030
 
  PDF   STATS   ENSEMBL   UCSC 
 
  238
 
  4
 
 41955
 
 island
 
 0.71408
 
 0.89273
 
 Not queried
 
  1.7275153
 
 2.00e-01
 
 1.00e+00
  
 
  
 2
 
  21266947
 
  21267334
 
  PDF   STATS   ENSEMBL   UCSC 
 
  388
 
  9
 
 23179
 
 tss;island
 
 0.30916
 
 0.58342
 
 Not queried
 
  1.7270848
 
 2.76e-03
 
 1.00e+00
  
 
  
 8
 
 144824625
 
 144824658
 
  PDF   STATS   ENSEMBL   UCSC 
 
   34
 
  4
 
 40621
 
 island
 
 0.73561
 
 0.89884
 
 Not queried
 
  1.7263235
 
 2.86e-02
 
 1.00e+00
  
 
  
 7
 
  45808481
 
  45808808
 
  PDF   STATS   ENSEMBL   UCSC 
 
  328
 
  8
 
 38412
 
 island
 
 0.03652
 
 0.06244
 
 Not queried
 
  1.7259051
 
 8.30e-02
 
 1.00e+00
  
 
  
 10
 
 135122746
 
 135123421
 
  PDF   STATS   ENSEMBL   UCSC 
 
  676
 
 12
 
 5442
 
 tss
 
 0.04225
 
 0.06689
 
 Not queried
 
  1.7256122
 
 1.98e-01
 
 1.00e+00
  
 
  
 10
 
 100991959
 
 100992222
 
  PDF   STATS   ENSEMBL   UCSC 
 
  264
 
  4
 
 4691
 
 gene;island
 
 0.30988
 
 0.10848
 
 Not queried
 
  1.7250149
 
 1.14e-01
 
 1.00e+00
  
 
  
 12
 
 117593259
 
 117593728
 
  PDF   STATS   ENSEMBL   UCSC 
 
  470
 
  7
 
 9310
 
 gene;island
 
 0.55117
 
 0.79206
 
 Not queried
 
  1.7249279
 
 1.65e-01
 
 1.00e+00
  
 
  
 7
 
 121950924
 
 121951055
 
  PDF   STATS   ENSEMBL   UCSC 
 
  132
 
  4
 
 38808
 
 island
 
 0.09864
 
 0.03199
 
 Not queried
 
  1.7248410
 
 5.71e-02
 
 1.00e+00
  
 
  
 13
 
  33780078
 
  33780362
 
  PDF   STATS   ENSEMBL   UCSC 
 
  285
 
  5
 
 11097
 
 gene
 
 0.78745
 
 0.58381
 
 Not queried
 
  1.7245465
 
 1.59e-02
 
 1.00e+00
  
 
  
 1
 
  29213106
 
  29213367
 
  PDF   STATS   ENSEMBL   UCSC 
 
  262
 
  4
 
 1434
 
 tss;island
 
 0.07992
 
 0.04584
 
 Not queried
 
  1.7241010
 
 3.43e-01
 
 1.00e+00
  
 
  
 17
 
  33759929
 
  33759986
 
  PDF   STATS   ENSEMBL   UCSC 
 
   58
 
  4
 
 17236
 
 tss
 
 0.92677
 
 0.97188
 
 Not queried
 
  1.7240124
 
 5.71e-02
 
 1.00e+00
  
 
  
 15
 
  78632670
 
  78632861
 
  PDF   STATS   ENSEMBL   UCSC 
 
  192
 
  5
 
 13409
 
 gene
 
 0.23164
 
 0.49688
 
 Not queried
 
  1.7236598
 
 7.94e-03
 
 1.00e+00
  
 
  
 8
 
  86350278
 
  86350592
 
  PDF   STATS   ENSEMBL   UCSC 
 
  315
 
  6
 
 39730
 
 tss;island
 
 0.15460
 
 0.36232
 
 Not queried
 
  1.7235696
 
 2.60e-02
 
 1.00e+00
  
 
  
 3
 
  35785735
 
  35786030
 
  PDF   STATS   ENSEMBL   UCSC 
 
  296
 
  5
 
 27671
 
 gene
 
 0.57758
 
 0.22165
 
 Not queried
 
  1.7221011
 
 3.10e-01
 
 1.00e+00
  
 
  
 7
 
 157644160
 
 157644665
 
  PDF   STATS   ENSEMBL   UCSC 
 
  506
 
  5
 
 37282
 
 gene;island
 
 0.38756
 
 0.16092
 
 Not queried
 
  1.7211531
 
 9.52e-02
 
 1.00e+00
  
 
  
 1
 
  26503623
 
  26503979
 
  PDF   STATS   ENSEMBL   UCSC 
 
  357
 
  8
 
 1381
 
 tss
 
 0.06364
 
 0.18370
 
 Not queried
 
  1.7211387
 
 6.50e-02
 
 1.00e+00
  
 
  
 14
 
 105953088
 
 105953244
 
  PDF   STATS   ENSEMBL   UCSC 
 
  157
 
  4
 
 12645
 
 tss
 
 0.03199
 
 0.10276
 
 Not queried
 
  1.7206157
 
 2.00e-01
 
 1.00e+00
  
 
  
 14
 
 105953088
 
 105953462
 
  PDF   STATS   ENSEMBL   UCSC 
 
  375
 
  6
 
 13212
 
 island
 
 0.03199
 
 0.11340
 
 Not queried
 
  1.7206157
 
 2.40e-01
 
 1.00e+00
  
 
  
 12
 
  51818350
 
  51818768
 
  PDF   STATS   ENSEMBL   UCSC 
 
  419
 
  7
 
 10401
 
 island
 
 0.11000
 
 0.02150
 
 Not queried
 
  1.7204559
 
 5.30e-02
 
 1.00e+00
  
 
  
 15
 
  89312795
 
  89313222
 
  PDF   STATS   ENSEMBL   UCSC 
 
  428
 
  7
 
 14393
 
 island
 
 0.09094
 
 0.32699
 
 Not queried
 
  1.7200440
 
 1.11e-02
 
 1.00e+00
  
 
  
 15
 
  49338251
 
  49339070
 
  PDF   STATS   ENSEMBL   UCSC 
 
  820
 
  8
 
 14098
 
 island
 
 0.03943
 
 0.05633
 
 Not queried
 
  1.7192486
 
 3.28e-01
 
 1.00e+00
  
 
  
 8
 
 145501817
 
 145502297
 
  PDF   STATS   ENSEMBL   UCSC 
 
  481
 
  5
 
 39450
 
 gene;island
 
 0.72540
 
 0.89377
 
 Not queried
 
  1.7186920
 
 7.94e-03
 
 1.00e+00
  
 
  
 19
 
  38210837
 
  38210932
 
  PDF   STATS   ENSEMBL   UCSC 
 
   96
 
  4
 
 20708
 
 tss
 
 0.04440
 
 0.16420
 
 Not queried
 
  1.7185252
 
 2.00e-01
 
 1.00e+00
  
 
  
 5
 
 110074217
 
 110074725
 
  PDF   STATS   ENSEMBL   UCSC 
 
  509
 
 10
 
 32146
 
 tss
 
 0.07598
 
 0.01868
 
 Not queried
 
  1.7185118
 
 6.84e-03
 
 1.00e+00
  
 
  
 1
 
 113216774
 
 113217043
 
  PDF   STATS   ENSEMBL   UCSC 
 
  270
 
  5
 
 1834
 
 tss
 
 0.06211
 
 0.25522
 
 Not queried
 
  1.7181366
 
 5.56e-02
 
 1.00e+00
  
 
  
 12
 
  52257893
 
  52258264
 
  PDF   STATS   ENSEMBL   UCSC 
 
  372
 
  4
 
 10405
 
 island
 
 0.05056
 
 0.01850
 
 Not queried
 
  1.7177680
 
 2.86e-02
 
 1.00e+00
  
 
  
 X
 
  56258113
 
  56259373
 
  PDF   STATS   ENSEMBL   UCSC 
 
 1261
 
 14
 
 41821
 
 island
 
 0.05722
 
 0.02863
 
 Not queried
 
  1.7164992
 
 3.52e-03
 
 1.00e+00
  
 
  
 1
 
   1549615
 
   1549818
 
  PDF   STATS   ENSEMBL   UCSC 
 
  204
 
  4
 
 1154
 
 tss;island
 
 0.02406
 
 0.12641
 
 Not queried
 
  1.7159173
 
 5.71e-02
 
 1.00e+00
  
 
  
 5
 
  54281437
 
  54281733
 
  PDF   STATS   ENSEMBL   UCSC 
 
  297
 
  4
 
 32003
 
 tss
 
 0.19647
 
 0.43121
 
 Not queried
 
  1.7146248
 
 2.00e-01
 
 1.00e+00
  
 
  
 4
 
   7593370
 
   7593585
 
  PDF   STATS   ENSEMBL   UCSC 
 
  216
 
  4
 
 29894
 
 gene;island
 
 0.80740
 
 0.91963
 
 Not queried
 
  1.7145204
 
 2.00e-01
 
 1.00e+00
  
 
  
 17
 
  66097276
 
  66097529
 
  PDF   STATS   ENSEMBL   UCSC 
 
  254
 
  5
 
 17569
 
 tss;island
 
 0.12116
 
 0.06868
 
 Not queried
 
  1.7142447
 
 5.56e-02
 
 1.00e+00
  
 
  
 6
 
  31237664
 
  31238036
 
  PDF   STATS   ENSEMBL   UCSC 
 
  373
 
  5
 
 33802
 
 gene;island
 
 0.69291
 
 0.68953
 
 Not queried
 
  1.7128316
 
 8.41e-01
 
 1.00e+00
  
 
  
 X
 
 128812233
 
 128812557
 
  PDF   STATS   ENSEMBL   UCSC 
 
  325
 
  5
 
 42018
 
 island
 
 0.56317
 
 0.80400
 
 Not queried
 
  1.7111169
 
 1.59e-02
 
 1.00e+00
  
 
  
 6
 
  31324916
 
  31325455
 
  PDF   STATS   ENSEMBL   UCSC 
 
  540
 
 16
 
 35706
 
 island
 
 0.15277
 
 0.02903
 
 Not queried
 
  1.7108957
 
 1.52e-02
 
 1.00e+00
  
 
  
 2
 
  10567526
 
  10567843
 
  PDF   STATS   ENSEMBL   UCSC 
 
  318
 
  4
 
 24030
 
 island
 
 0.85297
 
 0.96574
 
 Not queried
 
  1.7105722
 
 3.43e-01
 
 1.00e+00
  
 
  
 17
 
  39216457
 
  39216687
 
  PDF   STATS   ENSEMBL   UCSC 
 
  231
 
  4
 
 17304
 
 tss
 
 0.57260
 
 0.87381
 
 Not queried
 
  1.7101568
 
 5.71e-02
 
 1.00e+00
  
 
  
 6
 
 152957995
 
 152958664
 
  PDF   STATS   ENSEMBL   UCSC 
 
  670
 
 14
 
 35242
 
 tss
 
 0.11476
 
 0.05590
 
 Not queried
 
  1.7097872
 
 4.01e-01
 
 1.00e+00
  
 
  
 2
 
 239046684
 
 239047336
 
  PDF   STATS   ENSEMBL   UCSC 
 
  653
 
  8
 
 23932
 
 tss;island
 
 0.45409
 
 0.71173
 
 Not queried
 
  1.7097662
 
 2.07e-02
 
 1.00e+00
  
 
  
 2
 
  98351769
 
  98352002
 
  PDF   STATS   ENSEMBL   UCSC 
 
  234
 
  4
 
 22759
 
 gene;island
 
 0.56908
 
 0.81602
 
 Not queried
 
  1.7096686
 
 2.86e-02
 
 1.00e+00
  
 
  
 17
 
   7832680
 
   7832943
 
  PDF   STATS   ENSEMBL   UCSC 
 
  264
 
  7
 
 17945
 
 island
 
 0.09614
 
 0.03021
 
 Not queried
 
  1.7091903
 
 7.28e-02
 
 1.00e+00
  
 
  
 16
 
  69141250
 
  69141478
 
  PDF   STATS   ENSEMBL   UCSC 
 
  229
 
  7
 
 14787
 
 gene;island
 
 0.10962
 
 0.03527
 
 Not queried
 
  1.7090085
 
 1.11e-02
 
 1.00e+00
  
 
  
 7
 
   1596103
 
   1596260
 
  PDF   STATS   ENSEMBL   UCSC 
 
  158
 
  5
 
 37337
 
 tss
 
 0.65702
 
 0.89842
 
 Not queried
 
  1.7089071
 
 5.56e-02
 
 1.00e+00
  
 
  
 6
 
  30122947
 
  30123226
 
  PDF   STATS   ENSEMBL   UCSC 
 
  280
 
  5
 
 33667
 
 gene
 
 0.21741
 
 0.31255
 
 Not queried
 
  1.7086973
 
 1.51e-01
 
 1.00e+00
  
 
  
 14
 
  31343146
 
  31343427
 
  PDF   STATS   ENSEMBL   UCSC 
 
  282
 
  4
 
 12310
 
 tss;island
 
 0.07037
 
 0.02239
 
 Not queried
 
  1.7086105
 
 5.71e-02
 
 1.00e+00
  
 
  
 1
 
 151812421
 
 151812710
 
  PDF   STATS   ENSEMBL   UCSC 
 
  290
 
  4
 
 754
 
 gene;island
 
 0.16693
 
 0.04870
 
 Not queried
 
  1.7077093
 
 2.86e-02
 
 1.00e+00
  
 
  
 22
 
  51221675
 
  51221773
 
  PDF   STATS   ENSEMBL   UCSC 
 
   99
 
  4
 
 26894
 
 gene;tss;island
 
 0.01507
 
 0.03607
 
 Not queried
 
  1.7069519
 
 3.43e-01
 
 1.00e+00
  
 
  
 6
 
  33136859
 
  33137336
 
  PDF   STATS   ENSEMBL   UCSC 
 
  478
 
  7
 
 34134
 
 gene
 
 0.66933
 
 0.29825
 
 Not queried
 
  1.7057546
 
 1.75e-02
 
 1.00e+00
  
 
  
 6
 
  33383065
 
  33383412
 
  PDF   STATS   ENSEMBL   UCSC 
 
  348
 
  4
 
 34215
 
 gene;island
 
 0.81650
 
 0.92636
 
 Not queried
 
  1.7056431
 
 3.43e-01
 
 1.00e+00
  
 
  
 17
 
  73629082
 
  73629463
 
  PDF   STATS   ENSEMBL   UCSC 
 
  382
 
  6
 
 16736
 
 gene;tss;island
 
 0.37892
 
 0.68529
 
 Not queried
 
  1.7048526
 
 4.11e-02
 
 1.00e+00
  
 
  
 7
 
   2563478
 
   2563909
 
  PDF   STATS   ENSEMBL   UCSC 
 
  432
 
  5
 
 36742
 
 gene;island
 
 0.62786
 
 0.31378
 
 Not queried
 
  1.7048010
 
 7.94e-03
 
 1.00e+00
  
 
  
 5
 
   1298511
 
   1298965
 
  PDF   STATS   ENSEMBL   UCSC 
 
  455
 
  6
 
 32565
 
 island
 
 0.58348
 
 0.76761
 
 Not queried
 
  1.7043719
 
 1.32e-01
 
 1.00e+00
  
 
  
 13
 
  47371412
 
  47371644
 
  PDF   STATS   ENSEMBL   UCSC 
 
  233
 
  8
 
 11400
 
 tss
 
 0.05109
 
 0.11549
 
 Not queried
 
  1.7043528
 
 1.30e-01
 
 1.00e+00
  
 
  
 6
 
  28553927
 
  28555079
 
  PDF   STATS   ENSEMBL   UCSC 
 
 1153
 
 20
 
 33575
 
 gene
 
 0.02775
 
 0.06856
 
 Not queried
 
  1.7043432
 
 1.03e-02
 
 1.00e+00
  
 
  
 17
 
  35078659
 
  35079061
 
  PDF   STATS   ENSEMBL   UCSC 
 
  403
 
  4
 
 18200
 
 island
 
 0.54478
 
 0.79964
 
 Not queried
 
  1.7042055
 
 4.86e-01
 
 1.00e+00
  
 
  
 5
 
 172175604
 
 172175855
 
  PDF   STATS   ENSEMBL   UCSC 
 
  252
 
  4
 
 33331
 
 island
 
 0.47423
 
 0.79001
 
 Not queried
 
  1.7032154
 
 1.14e-01
 
 1.00e+00
  
 
  
 6
 
  31913759
 
  31914305
 
  PDF   STATS   ENSEMBL   UCSC 
 
  547
 
  9
 
 33932
 
 gene
 
 0.69661
 
 0.37580
 
 Not queried
 
  1.7025625
 
 1.23e-03
 
 1.00e+00
  
 
  
 1
 
 226111974
 
 226112534
 
  PDF   STATS   ENSEMBL   UCSC 
 
  561
 
  6
 
 2280
 
 tss
 
 0.05228
 
 0.30579
 
 Not queried
 
  1.7022329
 
 1.80e-01
 
 1.00e+00
  
 
  
 15
 
  85113863
 
  85114479
 
  PDF   STATS   ENSEMBL   UCSC 
 
  617
 
  8
 
 14373
 
 island
 
 0.02556
 
 0.19881
 
 Not queried
 
  1.7017840
 
 1.05e-01
 
 1.00e+00
  
 
  
 4
 
    699224
 
    699435
 
  PDF   STATS   ENSEMBL   UCSC 
 
  212
 
  6
 
 30183
 
 tss;island
 
 0.04400
 
 0.12027
 
 Not queried
 
  1.7013693
 
 1.52e-02
 
 1.00e+00
  
 
  
 1
 
  46951291
 
  46951683
 
  PDF   STATS   ENSEMBL   UCSC 
 
  393
 
  6
 
 3213
 
 island
 
 0.17412
 
 0.04558
 
 Not queried
 
  1.6999153
 
 1.32e-01
 
 1.00e+00
  
 
  
 17
 
  46703854
 
  46704004
 
  PDF   STATS   ENSEMBL   UCSC 
 
  151
 
  6
 
 17435
 
 tss;island
 
 0.05271
 
 0.15411
 
 Not queried
 
  1.6996251
 
 1.32e-01
 
 1.00e+00
  
 
  
 2
 
  51254021
 
  51254170
 
  PDF   STATS   ENSEMBL   UCSC 
 
  150
 
  4
 
 22640
 
 gene;island
 
 0.59827
 
 0.82682
 
 Not queried
 
  1.6991406
 
 2.00e-01
 
 1.00e+00
  
 
  
 2
 
  33359198
 
  33359550
 
  PDF   STATS   ENSEMBL   UCSC 
 
  353
 
  5
 
 23260
 
 tss
 
 0.68991
 
 0.83075
 
 Not queried
 
  1.6974114
 
 5.56e-02
 
 1.00e+00
  
 
  
 8
 
  10872604
 
  10872819
 
  PDF   STATS   ENSEMBL   UCSC 
 
  216
 
  4
 
 39139
 
 gene;island
 
 0.12194
 
 0.03515
 
 Not queried
 
  1.6971625
 
 5.71e-02
 
 1.00e+00
  
 
  
 13
 
 102104976
 
 102105129
 
  PDF   STATS   ENSEMBL   UCSC 
 
  154
 
  5
 
 11185
 
 gene
 
 0.48251
 
 0.74912
 
 Not queried
 
  1.6961263
 
 3.17e-02
 
 1.00e+00
  
 
  
 13
 
 102104573
 
 102105129
 
  PDF   STATS   ENSEMBL   UCSC 
 
  557
 
  9
 
 11827
 
 island
 
 0.48251
 
 0.74912
 
 Not queried
 
  1.6961263
 
 1.85e-03
 
 1.00e+00
  
 
  
 10
 
 121484846
 
 121485184
 
  PDF   STATS   ENSEMBL   UCSC 
 
  339
 
  6
 
 5384
 
 tss;island
 
 0.02025
 
 0.05412
 
 Not queried
 
  1.6955700
 
 4.33e-03
 
 1.00e+00
  
 
  
 10
 
 131665838
 
 131666314
 
  PDF   STATS   ENSEMBL   UCSC 
 
  477
 
  6
 
 4816
 
 gene;island
 
 0.80608
 
 0.94559
 
 Not queried
 
  1.6953735
 
 9.31e-02
 
 1.00e+00
  
 
  
 17
 
   8868541
 
   8868834
 
  PDF   STATS   ENSEMBL   UCSC 
 
  294
 
  4
 
 16399
 
 gene;island
 
 0.23955
 
 0.20248
 
 Not queried
 
  1.6951458
 
 6.86e-01
 
 1.00e+00
  
 
  
 14
 
 103986227
 
 103986467
 
  PDF   STATS   ENSEMBL   UCSC 
 
  241
 
  4
 
 12208
 
 gene;island
 
 0.79480
 
 0.94904
 
 Not queried
 
  1.6950960
 
 5.71e-02
 
 1.00e+00
  
 
  
 15
 
  85114131
 
  85114479
 
  PDF   STATS   ENSEMBL   UCSC 
 
  349
 
  6
 
 13839
 
 tss
 
 0.02028
 
 0.03164
 
 Not queried
 
  1.6941831
 
 3.10e-01
 
 1.00e+00
  
 
  
 10
 
  71087834
 
  71088038
 
  PDF   STATS   ENSEMBL   UCSC 
 
  205
 
  4
 
 4599
 
 gene
 
 0.66866
 
 0.86299
 
 Not queried
 
  1.6938263
 
 2.86e-02
 
 1.00e+00
  
 
  
 2
 
 128422113
 
 128422717
 
  PDF   STATS   ENSEMBL   UCSC 
 
  605
 
 10
 
 22825
 
 gene;island
 
 0.51720
 
 0.80837
 
 Not queried
 
  1.6936144
 
 2.80e-01
 
 1.00e+00
  
 
  
 12
 
  69753119
 
  69753455
 
  PDF   STATS   ENSEMBL   UCSC 
 
  337
 
  5
 
 9835
 
 tss
 
 0.02406
 
 0.02830
 
 Not queried
 
  1.6931192
 
 6.90e-01
 
 1.00e+00
  
 
  
 11
 
   1968266
 
   1968980
 
  PDF   STATS   ENSEMBL   UCSC 
 
  715
 
  8
 
 7918
 
 island
 
 0.03857
 
 0.12590
 
 Not queried
 
  1.6929547
 
 1.48e-02
 
 1.00e+00
  
 
  
 1
 
  27961563
 
  27962037
 
  PDF   STATS   ENSEMBL   UCSC 
 
  475
 
  7
 
 2919
 
 island
 
 0.80453
 
 0.56015
 
 Not queried
 
  1.6924239
 
 1.28e-01
 
 1.00e+00
  
 
  
 13
 
  33859993
 
  33860083
 
  PDF   STATS   ENSEMBL   UCSC 
 
   91
 
  6
 
 11359
 
 tss
 
 0.03569
 
 0.05509
 
 Not queried
 
  1.6923044
 
 2.40e-01
 
 1.00e+00
  
 
  
 10
 
 134928055
 
 134928245
 
  PDF   STATS   ENSEMBL   UCSC 
 
  191
 
  4
 
 4869
 
 gene;island
 
 0.79622
 
 0.92188
 
 Not queried
 
  1.6916866
 
 3.43e-01
 
 1.00e+00
  
 
  
 17
 
   8661887
 
   8662011
 
  PDF   STATS   ENSEMBL   UCSC 
 
  125
 
  5
 
 17088
 
 tss
 
 0.88770
 
 0.71462
 
 Not queried
 
  1.6916245
 
 6.90e-01
 
 1.00e+00
  
 
  
 14
 
  27066404
 
  27066771
 
  PDF   STATS   ENSEMBL   UCSC 
 
  368
 
  4
 
 12023
 
 gene;island
 
 0.01576
 
 0.02443
 
 Not queried
 
  1.6914950
 
 4.86e-01
 
 1.00e+00
  
 
  
 19
 
  35791192
 
  35791409
 
  PDF   STATS   ENSEMBL   UCSC 
 
  218
 
  4
 
 19947
 
 gene;island
 
 0.35174
 
 0.60009
 
 Not queried
 
  1.6907285
 
 3.43e-01
 
 1.00e+00
  
 
  
 13
 
 114914537
 
 114914682
 
  PDF   STATS   ENSEMBL   UCSC 
 
  146
 
  4
 
 11968
 
 island
 
 0.62082
 
 0.84592
 
 Not queried
 
  1.6902196
 
 3.43e-01
 
 1.00e+00
  
 
  
 14
 
  24685281
 
  24685421
 
  PDF   STATS   ENSEMBL   UCSC 
 
  141
 
  4
 
 12293
 
 tss
 
 0.01457
 
 0.03606
 
 Not queried
 
  1.6901799
 
 2.00e-01
 
 1.00e+00
  
 
  
 2
 
 133426129
 
 133426418
 
  PDF   STATS   ENSEMBL   UCSC 
 
  290
 
  4
 
 22841
 
 gene;island
 
 0.03184
 
 0.07861
 
 Not queried
 
  1.6898473
 
 3.43e-01
 
 1.00e+00
  
 
  
 18
 
  74175071
 
  74175208
 
  PDF   STATS   ENSEMBL   UCSC 
 
  138
 
  4
 
 19293
 
 tss
 
 0.91258
 
 0.93877
 
 Not queried
 
  1.6896575
 
 3.43e-01
 
 1.00e+00
  
 
  
 13
 
  61988802
 
  61989203
 
  PDF   STATS   ENSEMBL   UCSC 
 
  402
 
  4
 
 11146
 
 gene
 
 0.02709
 
 0.03970
 
 Not queried
 
  1.6895975
 
 4.86e-01
 
 1.00e+00
  
 
  
 17
 
  50237205
 
  50237844
 
  PDF   STATS   ENSEMBL   UCSC 
 
  640
 
  9
 
 17482
 
 tss;island
 
 0.38541
 
 0.62457
 
 Not queried
 
  1.6891238
 
 7.70e-02
 
 1.00e+00
  
 
  
 1
 
 109655203
 
 109655543
 
  PDF   STATS   ENSEMBL   UCSC 
 
  341
 
  4
 
 662
 
 gene;tss;island
 
 0.04901
 
 0.10725
 
 Not queried
 
  1.6889458
 
 4.86e-01
 
 1.00e+00
  
 
  
 3
 
  98376390
 
  98376574
 
  PDF   STATS   ENSEMBL   UCSC 
 
  185
 
  4
 
 29273
 
 island
 
 0.03341
 
 0.10117
 
 Not queried
 
  1.6885295
 
 2.86e-02
 
 1.00e+00
  
 
  
 5
 
    497397
 
    497639
 
  PDF   STATS   ENSEMBL   UCSC 
 
  243
 
  4
 
 31454
 
 gene;island
 
 0.43932
 
 0.73661
 
 Not queried
 
  1.6883681
 
 2.86e-02
 
 1.00e+00
  
 
  
 16
 
   2907517
 
   2907859
 
  PDF   STATS   ENSEMBL   UCSC 
 
  343
 
  4
 
 14587
 
 gene;island
 
 0.21120
 
 0.45206
 
 Not queried
 
  1.6880436
 
 2.00e-01
 
 1.00e+00
  
 
  
 20
 
  45179226
 
  45179413
 
  PDF   STATS   ENSEMBL   UCSC 
 
  188
 
  5
 
 25754
 
 tss
 
 0.55196
 
 0.83267
 
 Not queried
 
  1.6876593
 
 5.56e-02
 
 1.00e+00
  
 
  
 22
 
  41956810
 
  41956987
 
  PDF   STATS   ENSEMBL   UCSC 
 
  178
 
  4
 
 27133
 
 tss
 
 0.32381
 
 0.58251
 
 Not queried
 
  1.6876392
 
 2.86e-02
 
 1.00e+00
  
 
  
 5
 
  93077290
 
  93077405
 
  PDF   STATS   ENSEMBL   UCSC 
 
  116
 
  5
 
 31635
 
 gene
 
 0.66064
 
 0.79325
 
 Not queried
 
  1.6872213
 
 1.51e-01
 
 1.00e+00
  
 
  
 6
 
 152957910
 
 152958664
 
  PDF   STATS   ENSEMBL   UCSC 
 
  755
 
 15
 
 36518
 
 island
 
 0.07246
 
 0.05526
 
 Not queried
 
  1.6869202
 
 5.12e-01
 
 1.00e+00
  
 
  
 12
 
  14518391
 
  14518974
 
  PDF   STATS   ENSEMBL   UCSC 
 
  584
 
  9
 
 10237
 
 island
 
 0.10071
 
 0.26498
 
 Not queried
 
  1.6865976
 
 1.61e-01
 
 1.00e+00
  
 
  
 6
 
 126071099
 
 126071538
 
  PDF   STATS   ENSEMBL   UCSC 
 
  440
 
  5
 
 34401
 
 gene;island
 
 0.06528
 
 0.03151
 
 Not queried
 
  1.6861993
 
 1.59e-02
 
 1.00e+00
  
 
  
 17
 
  31254544
 
  31254878
 
  PDF   STATS   ENSEMBL   UCSC 
 
  335
 
  8
 
 17224
 
 tss;island
 
 0.26206
 
 0.05604
 
 Not queried
 
  1.6861943
 
 2.81e-02
 
 1.00e+00
  
 
  
 13
 
  53423940
 
  53424127
 
  PDF   STATS   ENSEMBL   UCSC 
 
  188
 
  4
 
 11438
 
 tss;island
 
 0.23823
 
 0.10877
 
 Not queried
 
  1.6860262
 
 2.00e-01
 
 1.00e+00
  
 
  
 13
 
  70682525
 
  70682771
 
  PDF   STATS   ENSEMBL   UCSC 
 
  247
 
  5
 
 11148
 
 gene;island
 
 0.16034
 
 0.38059
 
 Not queried
 
  1.6860234
 
 1.59e-02
 
 1.00e+00
  
 
  
 6
 
  33040535
 
  33040914
 
  PDF   STATS   ENSEMBL   UCSC 
 
  380
 
  5
 
 34118
 
 gene
 
 0.74502
 
 0.47593
 
 Not queried
 
  1.6858536
 
 3.10e-01
 
 1.00e+00
  
 
  
 8
 
  91803657
 
  91803805
 
  PDF   STATS   ENSEMBL   UCSC 
 
  149
 
  5
 
 39743
 
 tss;island
 
 0.06896
 
 0.19241
 
 Not queried
 
  1.6856226
 
 9.52e-02
 
 1.00e+00
  
 
  
 14
 
 105936300
 
 105936715
 
  PDF   STATS   ENSEMBL   UCSC 
 
  416
 
  4
 
 12237
 
 gene;island
 
 0.71582
 
 0.92242
 
 Not queried
 
  1.6844915
 
 2.00e-01
 
 1.00e+00
  
 
  
 19
 
  34918900
 
  34919411
 
  PDF   STATS   ENSEMBL   UCSC 
 
  512
 
  8
 
 21851
 
 island
 
 0.01162
 
 0.02231
 
 Not queried
 
  1.6831380
 
 1.61e-01
 
 1.00e+00
  
 
  
 11
 
  60681193
 
  60681305
 
  PDF   STATS   ENSEMBL   UCSC 
 
  113
 
  6
 
 7304
 
 tss;island
 
 0.00619
 
 0.01960
 
 Not queried
 
  1.6823421
 
 4.11e-02
 
 1.00e+00
  
 
  
 7
 
   2559692
 
   2560104
 
  PDF   STATS   ENSEMBL   UCSC 
 
  413
 
  4
 
 36741
 
 gene;island
 
 0.11149
 
 0.03004
 
 Not queried
 
  1.6818266
 
 4.86e-01
 
 1.00e+00
  
 
  
 6
 
  43252858
 
  43253153
 
  PDF   STATS   ENSEMBL   UCSC 
 
  296
 
  4
 
 34282
 
 gene;island
 
 0.72392
 
 0.88535
 
 Not queried
 
  1.6815988
 
 2.86e-02
 
 1.00e+00
  
 
  
 12
 
 133424221
 
 133424709
 
  PDF   STATS   ENSEMBL   UCSC 
 
  489
 
  7
 
 9441
 
 gene
 
 0.80122
 
 0.85982
 
 Not queried
 
  1.6812534
 
 1.28e-01
 
 1.00e+00
  
 
  
 2
 
    662602
 
    663024
 
  PDF   STATS   ENSEMBL   UCSC 
 
  423
 
  4
 
 23972
 
 island
 
 0.59699
 
 0.79035
 
 Not queried
 
  1.6812362
 
 3.43e-01
 
 1.00e+00
  
 
  
 1
 
 153747551
 
 153747728
 
  PDF   STATS   ENSEMBL   UCSC 
 
  178
 
  6
 
 1949
 
 tss
 
 0.04188
 
 0.13057
 
 Not queried
 
  1.6810273
 
 2.60e-02
 
 1.00e+00
  
 
  
 11
 
  72353049
 
  72353604
 
  PDF   STATS   ENSEMBL   UCSC 
 
  556
 
 10
 
 6795
 
 gene;island
 
 0.16906
 
 0.08271
 
 Not queried
 
  1.6806116
 
 2.88e-02
 
 1.00e+00
  
 
  
 1
 
  26516478
 
  26516623
 
  PDF   STATS   ENSEMBL   UCSC 
 
  146
 
  4
 
 1382
 
 tss
 
 0.39835
 
 0.70687
 
 Not queried
 
  1.6805199
 
 2.86e-02
 
 1.00e+00
  
 
  
 22
 
  46659317
 
  46659595
 
  PDF   STATS   ENSEMBL   UCSC 
 
  279
 
  4
 
 27190
 
 tss;island
 
 0.95545
 
 0.86992
 
 Not queried
 
  1.6799060
 
 2.00e-01
 
 1.00e+00
  
 
  
 6
 
  32909282
 
  32909523
 
  PDF   STATS   ENSEMBL   UCSC 
 
  242
 
  5
 
 34817
 
 tss
 
 0.93240
 
 0.72923
 
 Not queried
 
  1.6796362
 
 2.22e-01
 
 1.00e+00
  
 
  
 1
 
 164545553
 
 164545843
 
  PDF   STATS   ENSEMBL   UCSC 
 
  291
 
  4
 
 860
 
 gene;island
 
 0.69559
 
 0.37196
 
 Not queried
 
  1.6796059
 
 2.86e-02
 
 1.00e+00
  
 
  
 15
 
  47476133
 
  47476482
 
  PDF   STATS   ENSEMBL   UCSC 
 
  350
 
  6
 
 14090
 
 island
 
 0.32161
 
 0.10382
 
 Not queried
 
  1.6795288
 
 1.80e-01
 
 1.00e+00
  
 
  
 8
 
  38326209
 
  38326730
 
  PDF   STATS   ENSEMBL   UCSC 
 
  522
 
  8
 
 40169
 
 island
 
 0.15173
 
 0.03107
 
 Not queried
 
  1.6788529
 
 1.61e-01
 
 1.00e+00
  
 
  
 6
 
  28863395
 
  28863544
 
  PDF   STATS   ENSEMBL   UCSC 
 
  150
 
  4
 
 35538
 
 island
 
 0.01612
 
 0.03562
 
 Not queried
 
  1.6784948
 
 5.71e-02
 
 1.00e+00
  
 
  
 17
 
  46669455
 
  46669674
 
  PDF   STATS   ENSEMBL   UCSC 
 
  220
 
  7
 
 17430
 
 tss
 
 0.86838
 
 0.65297
 
 Not queried
 
  1.6782336
 
 2.62e-02
 
 1.00e+00
  
 
  
 13
 
 100636183
 
 100636299
 
  PDF   STATS   ENSEMBL   UCSC 
 
  117
 
  4
 
 11181
 
 gene;island
 
 0.04788
 
 0.00863
 
 Not queried
 
  1.6777942
 
 1.14e-01
 
 1.00e+00
  
 
  
 7
 
  25989524
 
  25989763
 
  PDF   STATS   ENSEMBL   UCSC 
 
  240
 
  4
 
 38263
 
 island
 
 0.01020
 
 0.04392
 
 Not queried
 
  1.6772681
 
 3.43e-01
 
 1.00e+00
  
 
  
 19
 
   7735239
 
   7735510
 
  PDF   STATS   ENSEMBL   UCSC 
 
  272
 
  4
 
 21425
 
 island
 
 0.11620
 
 0.20474
 
 Not queried
 
  1.6761325
 
 1.14e-01
 
 1.00e+00
  
 
  
 6
 
  42858647
 
  42859023
 
  PDF   STATS   ENSEMBL   UCSC 
 
  377
 
  8
 
 34919
 
 tss
 
 0.01470
 
 0.02697
 
 Not queried
 
  1.6760824
 
 2.79e-01
 
 1.00e+00
  
 
  
 17
 
  54675001
 
  54675231
 
  PDF   STATS   ENSEMBL   UCSC 
 
  231
 
  4
 
 18544
 
 island
 
 0.10528
 
 0.06205
 
 Not queried
 
  1.6755946
 
 2.00e-01
 
 1.00e+00
  
 
  
 12
 
 130821453
 
 130821607
 
  PDF   STATS   ENSEMBL   UCSC 
 
  155
 
  4
 
 10093
 
 tss;island
 
 0.94388
 
 0.81099
 
 Not queried
 
  1.6753291
 
 2.00e-01
 
 1.00e+00
  
 
  
 15
 
  52311035
 
  52311380
 
  PDF   STATS   ENSEMBL   UCSC 
 
  346
 
  4
 
 13648
 
 tss
 
 0.09185
 
 0.04459
 
 Not queried
 
  1.6752240
 
 4.86e-01
 
 1.00e+00
  
 
  
 3
 
 128336483
 
 128337044
 
  PDF   STATS   ENSEMBL   UCSC 
 
  562
 
  8
 
 29413
 
 island
 
 0.06838
 
 0.20911
 
 Not queried
 
  1.6750055
 
 6.99e-03
 
 1.00e+00
  
 
  
 18
 
  74729064
 
  74729251
 
  PDF   STATS   ENSEMBL   UCSC 
 
  188
 
  6
 
 19295
 
 tss
 
 0.81820
 
 0.93965
 
 Not queried
 
  1.6749128
 
 4.11e-02
 
 1.00e+00
  
 
  
 5
 
 172386517
 
 172387101
 
  PDF   STATS   ENSEMBL   UCSC 
 
  585
 
  8
 
 31840
 
 gene
 
 0.05888
 
 0.01613
 
 Not queried
 
  1.6744646
 
 8.30e-02
 
 1.00e+00
  
 
  
 3
 
  45066580
 
  45066971
 
  PDF   STATS   ENSEMBL   UCSC 
 
  392
 
  4
 
 28224
 
 tss
 
 0.71658
 
 0.87039
 
 Not queried
 
  1.6744011
 
 5.71e-02
 
 1.00e+00
  
 
  
 16
 
    474271
 
    474528
 
  PDF   STATS   ENSEMBL   UCSC 
 
  258
 
  4
 
 14936
 
 tss;island
 
 0.93481
 
 0.77527
 
 Not queried
 
  1.6738797
 
 2.00e-01
 
 1.00e+00
  
 
  
 13
 
  51483748
 
  51484248
 
  PDF   STATS   ENSEMBL   UCSC 
 
  501
 
  9
 
 11717
 
 island
 
 0.01780
 
 0.05135
 
 Not queried
 
  1.6737228
 
 9.39e-02
 
 1.00e+00
  
 
  
 6
 
  41470812
 
  41471064
 
  PDF   STATS   ENSEMBL   UCSC 
 
  253
 
  4
 
 36060
 
 island
 
 0.14705
 
 0.07428
 
 Not queried
 
  1.6737114
 
 3.43e-01
 
 1.00e+00
  
 
  
 1
 
   3775078
 
   3775334
 
  PDF   STATS   ENSEMBL   UCSC 
 
  257
 
  4
 
 148
 
 gene;island
 
 0.80816
 
 0.50140
 
 Not queried
 
  1.6726588
 
 3.43e-01
 
 1.00e+00
  
 
  
 11
 
  43701985
 
  43702128
 
  PDF   STATS   ENSEMBL   UCSC 
 
  144
 
  5
 
 7234
 
 tss
 
 0.02225
 
 0.06562
 
 Not queried
 
  1.6723486
 
 9.52e-02
 
 1.00e+00
  
 
  
 20
 
  22566877
 
  22567170
 
  PDF   STATS   ENSEMBL   UCSC 
 
  294
 
  5
 
 25595
 
 tss;island
 
 0.39191
 
 0.68573
 
 Not queried
 
  1.6719416
 
 1.51e-01
 
 1.00e+00
  
 
  
 10
 
  18629497
 
  18629677
 
  PDF   STATS   ENSEMBL   UCSC 
 
  181
 
  5
 
 4509
 
 gene
 
 0.38176
 
 0.08063
 
 Not queried
 
  1.6715270
 
 5.56e-02
 
 1.00e+00
  
 
  
 5
 
   1666974
 
   1667258
 
  PDF   STATS   ENSEMBL   UCSC 
 
  285
 
  4
 
 32579
 
 island
 
 0.21313
 
 0.06265
 
 Not queried
 
  1.6710308
 
 4.86e-01
 
 1.00e+00
  
 
  
 3
 
 120170383
 
 120170646
 
  PDF   STATS   ENSEMBL   UCSC 
 
  264
 
  4
 
 28485
 
 tss;island
 
 0.74197
 
 0.46482
 
 Not queried
 
  1.6705736
 
 2.00e-01
 
 1.00e+00
  
 
  
 10
 
 134944446
 
 134945099
 
  PDF   STATS   ENSEMBL   UCSC 
 
  654
 
  6
 
 4870
 
 gene;island
 
 0.56757
 
 0.80163
 
 Not queried
 
  1.6703307
 
 2.60e-02
 
 1.00e+00
  
 
  
 7
 
  94953653
 
  94954202
 
  PDF   STATS   ENSEMBL   UCSC 
 
  550
 
  9
 
 38613
 
 island
 
 0.61852
 
 0.81183
 
 Not queried
 
  1.6701016
 
 3.15e-02
 
 1.00e+00
  
 
  
 19
 
  13616871
 
  13617094
 
  PDF   STATS   ENSEMBL   UCSC 
 
  224
 
  4
 
 19846
 
 gene;island
 
 0.06174
 
 0.05747
 
 Not queried
 
  1.6700466
 
 6.86e-01
 
 1.00e+00
  
 
  
 5
 
  42949542
 
  42950152
 
  PDF   STATS   ENSEMBL   UCSC 
 
  611
 
  6
 
 32699
 
 island
 
 0.95288
 
 0.88476
 
 Not queried
 
  1.6697514
 
 1.52e-02
 
 1.00e+00
  
 
  
 16
 
  84163803
 
  84163961
 
  PDF   STATS   ENSEMBL   UCSC 
 
  159
 
  4
 
 14832
 
 gene
 
 0.62779
 
 0.83123
 
 Not queried
 
  1.6694601
 
 4.86e-01
 
 1.00e+00
  
 
  
 7
 
  54609587
 
  54609968
 
  PDF   STATS   ENSEMBL   UCSC 
 
  382
 
  8
 
 37536
 
 tss
 
 0.01547
 
 0.05231
 
 Not queried
 
  1.6688574
 
 6.50e-02
 
 1.00e+00
  
 
  
 7
 
 156811252
 
 156811655
 
  PDF   STATS   ENSEMBL   UCSC 
 
  404
 
  6
 
 39031
 
 island
 
 0.12019
 
 0.03898
 
 Not queried
 
  1.6686152
 
 1.32e-01
 
 1.00e+00
  
 
  
 11
 
   2189562
 
   2189856
 
  PDF   STATS   ENSEMBL   UCSC 
 
  295
 
  6
 
 6434
 
 gene;island
 
 0.64841
 
 0.80993
 
 Not queried
 
  1.6677094
 
 6.49e-02
 
 1.00e+00
  
 
  
 2
 
 240868184
 
 240868631
 
  PDF   STATS   ENSEMBL   UCSC 
 
  448
 
  5
 
 25226
 
 island
 
 0.54333
 
 0.86372
 
 Not queried
 
  1.6674575
 
 3.17e-02
 
 1.00e+00
  
 
  
 22
 
  21986976
 
  21987048
 
  PDF   STATS   ENSEMBL   UCSC 
 
   73
 
  5
 
 26955
 
 tss
 
 0.49850
 
 0.67596
 
 Not queried
 
  1.6658015
 
 5.56e-02
 
 1.00e+00
  
 
  
 7
 
   2701419
 
   2701802
 
  PDF   STATS   ENSEMBL   UCSC 
 
  384
 
  5
 
 36749
 
 gene;island
 
 0.92674
 
 0.92920
 
 Not queried
 
  1.6655544
 
 4.21e-01
 
 1.00e+00
  
 
  
 5
 
 172655841
 
 172656166
 
  PDF   STATS   ENSEMBL   UCSC 
 
  326
 
  6
 
 33341
 
 island
 
 0.63037
 
 0.84884
 
 Not queried
 
  1.6652434
 
 8.66e-03
 
 1.00e+00
  
 
  
 12
 
 122356316
 
 122356852
 
  PDF   STATS   ENSEMBL   UCSC 
 
  537
 
  6
 
 10896
 
 island
 
 0.78892
 
 0.88224
 
 Not queried
 
  1.6644926
 
 1.32e-01
 
 1.00e+00
  
 
  
 12
 
  49782966
 
  49783222
 
  PDF   STATS   ENSEMBL   UCSC 
 
  257
 
  5
 
 9094
 
 gene;island
 
 0.46469
 
 0.77572
 
 Not queried
 
  1.6643171
 
 9.52e-02
 
 1.00e+00
  
 
  
 7
 
 150778201
 
 150778340
 
  PDF   STATS   ENSEMBL   UCSC 
 
  140
 
  5
 
 37223
 
 gene
 
 0.10499
 
 0.18599
 
 Not queried
 
  1.6641484
 
 1.51e-01
 
 1.00e+00
  
 
  
 1
 
 198828157
 
 198828249
 
  PDF   STATS   ENSEMBL   UCSC 
 
   93
 
  4
 
 2155
 
 tss
 
 0.95347
 
 0.86300
 
 Not queried
 
  1.6638168
 
 3.43e-01
 
 1.00e+00
  
 
  
 7
 
  27191914
 
  27192656
 
  PDF   STATS   ENSEMBL   UCSC 
 
  743
 
  8
 
 38287
 
 island
 
 0.26971
 
 0.62053
 
 Not queried
 
  1.6636488
 
 1.48e-02
 
 1.00e+00
  
 
  
 12
 
  79198115
 
  79198467
 
  PDF   STATS   ENSEMBL   UCSC 
 
  353
 
  4
 
 10618
 
 island
 
 0.54958
 
 0.75817
 
 Not queried
 
  1.6635422
 
 3.43e-01
 
 1.00e+00
  
 
  
 2
 
 177014467
 
 177015125
 
  PDF   STATS   ENSEMBL   UCSC 
 
  659
 
 13
 
 23702
 
 tss;island
 
 0.82583
 
 0.61181
 
 Not queried
 
  1.6634491
 
 7.23e-02
 
 1.00e+00
  
 
  
 13
 
 102104573
 
 102104848
 
  PDF   STATS   ENSEMBL   UCSC 
 
  276
 
  4
 
 11492
 
 tss
 
 0.48742
 
 0.74106
 
 Not queried
 
  1.6626938
 
 5.71e-02
 
 1.00e+00
  
 
  
 X
 
  30233227
 
  30233603
 
  PDF   STATS   ENSEMBL   UCSC 
 
  377
 
  8
 
 41242
 
 tss
 
 0.82248
 
 0.94188
 
 Not queried
 
  1.6625282
 
 6.99e-03
 
 1.00e+00
  
 
  
 12
 
   8332640
 
   8332802
 
  PDF   STATS   ENSEMBL   UCSC 
 
  163
 
  4
 
 9528
 
 tss
 
 0.03397
 
 0.08107
 
 Not queried
 
  1.6613791
 
 3.43e-01
 
 1.00e+00
  
 
  
 17
 
  65471303
 
  65471507
 
  PDF   STATS   ENSEMBL   UCSC 
 
  205
 
  5
 
 16702
 
 gene
 
 0.94611
 
 0.91908
 
 Not queried
 
  1.6612761
 
 3.10e-01
 
 1.00e+00
  
 
  
 12
 
 133019854
 
 133020030
 
  PDF   STATS   ENSEMBL   UCSC 
 
  177
 
  4
 
 11029
 
 island
 
 0.73459
 
 0.89460
 
 Not queried
 
  1.6605825
 
 1.14e-01
 
 1.00e+00
  
 
  
 2
 
 177027291
 
 177027931
 
  PDF   STATS   ENSEMBL   UCSC 
 
  641
 
  7
 
 24872
 
 island
 
 0.80171
 
 0.50501
 
 Not queried
 
  1.6604367
 
 5.30e-02
 
 1.00e+00
  
 
  
 6
 
  11111894
 
  11112237
 
  PDF   STATS   ENSEMBL   UCSC 
 
  344
 
  5
 
 33505
 
 gene
 
 0.53552
 
 0.82242
 
 Not queried
 
  1.6603803
 
 3.17e-02
 
 1.00e+00
  
 
  
 7
 
   6191780
 
   6192094
 
  PDF   STATS   ENSEMBL   UCSC 
 
  315
 
  6
 
 36822
 
 gene;island
 
 0.79791
 
 0.91170
 
 Not queried
 
  1.6599249
 
 4.11e-02
 
 1.00e+00
  
 
  
 5
 
 139060476
 
 139060922
 
  PDF   STATS   ENSEMBL   UCSC 
 
  447
 
  6
 
 31723
 
 gene;island
 
 0.62958
 
 0.85324
 
 Not queried
 
  1.6593009
 
 2.16e-03
 
 1.00e+00
  
 
  
 7
 
  32996733
 
  32996881
 
  PDF   STATS   ENSEMBL   UCSC 
 
  149
 
  6
 
 37464
 
 tss;island
 
 0.11337
 
 0.03322
 
 Not queried
 
  1.6587234
 
 4.11e-02
 
 1.00e+00
  
 
  
 13
 
 114108433
 
 114108713
 
  PDF   STATS   ENSEMBL   UCSC 
 
  281
 
  6
 
 11524
 
 tss
 
 0.79494
 
 0.90931
 
 Not queried
 
  1.6582197
 
 2.60e-02
 
 1.00e+00
  
 
  
 1
 
 154539974
 
 154540535
 
  PDF   STATS   ENSEMBL   UCSC 
 
  562
 
  8
 
 3783
 
 island
 
 0.44003
 
 0.18350
 
 Not queried
 
  1.6572314
 
 4.42e-01
 
 1.00e+00
  
 
  
 7
 
 150644459
 
 150644866
 
  PDF   STATS   ENSEMBL   UCSC 
 
  408
 
  5
 
 37215
 
 gene;island
 
 0.44487
 
 0.71641
 
 Not queried
 
  1.6564077
 
 3.10e-01
 
 1.00e+00
  
 
  
 10
 
 134411295
 
 134411480
 
  PDF   STATS   ENSEMBL   UCSC 
 
  186
 
  4
 
 4851
 
 gene;island
 
 0.96179
 
 0.91730
 
 Not queried
 
  1.6563779
 
 1.14e-01
 
 1.00e+00
  
 
  
 21
 
  37432620
 
  37432782
 
  PDF   STATS   ENSEMBL   UCSC 
 
  163
 
  4
 
 26492
 
 tss
 
 0.01878
 
 0.02891
 
 Not queried
 
  1.6563337
 
 4.86e-01
 
 1.00e+00
  
 
  
 6
 
 144385771
 
 144386528
 
  PDF   STATS   ENSEMBL   UCSC 
 
  758
 
 20
 
 35206
 
 tss
 
 0.86331
 
 0.51765
 
 Not queried
 
  1.6557780
 
 3.50e-02
 
 1.00e+00
  
 
  
 7
 
  75956931
 
  75957376
 
  PDF   STATS   ENSEMBL   UCSC 
 
  446
 
  7
 
 36991
 
 gene;island
 
 0.86586
 
 0.94673
 
 Not queried
 
  1.6550228
 
 7.28e-02
 
 1.00e+00
  
 
  
 X
 
  75005076
 
  75005131
 
  PDF   STATS   ENSEMBL   UCSC 
 
   56
 
  5
 
 41397
 
 tss
 
 0.09152
 
 0.37602
 
 Not queried
 
  1.6548493
 
 3.17e-02
 
 1.00e+00
  
 
  
 19
 
  52004992
 
  52005188
 
  PDF   STATS   ENSEMBL   UCSC 
 
  197
 
  6
 
 20960
 
 tss
 
 0.24828
 
 0.50251
 
 Not queried
 
  1.6531729
 
 2.60e-02
 
 1.00e+00
  
 
  
 5
 
 167719478
 
 167719742
 
  PDF   STATS   ENSEMBL   UCSC 
 
  265
 
  4
 
 31825
 
 gene;island
 
 0.03042
 
 0.09558
 
 Not queried
 
  1.6526156
 
 2.86e-02
 
 1.00e+00
  
 
  
 8
 
 143333074
 
 143333834
 
  PDF   STATS   ENSEMBL   UCSC 
 
  761
 
  7
 
 39392
 
 gene;island
 
 0.56210
 
 0.81708
 
 Not queried
 
  1.6522252
 
 1.75e-02
 
 1.00e+00
  
 
  
 3
 
 138153127
 
 138153381
 
  PDF   STATS   ENSEMBL   UCSC 
 
  255
 
  8
 
 28580
 
 tss
 
 0.19519
 
 0.07584
 
 Not queried
 
  1.6518362
 
 2.34e-01
 
 1.00e+00
  
 
  
 16
 
  58497230
 
  58497395
 
  PDF   STATS   ENSEMBL   UCSC 
 
  166
 
  4
 
 15241
 
 tss;island
 
 0.33681
 
 0.06219
 
 Not queried
 
  1.6510914
 
 3.43e-01
 
 1.00e+00
  
 
  
 12
 
  48398320
 
  48398730
 
  PDF   STATS   ENSEMBL   UCSC 
 
  411
 
  7
 
 9640
 
 tss;island
 
 0.12749
 
 0.04136
 
 Not queried
 
  1.6500606
 
 3.79e-02
 
 1.00e+00
  
 
  
 19
 
  47778045
 
  47778150
 
  PDF   STATS   ENSEMBL   UCSC 
 
  106
 
  4
 
 22157
 
 island
 
 0.01744
 
 0.08359
 
 Not queried
 
  1.6500245
 
 1.14e-01
 
 1.00e+00
  
 
  
 20
 
  62716332
 
  62716606
 
  PDF   STATS   ENSEMBL   UCSC 
 
  275
 
  4
 
 25481
 
 gene;tss;island
 
 0.79923
 
 0.53472
 
 Not queried
 
  1.6497165
 
 5.71e-02
 
 1.00e+00
  
 
  
 16
 
  66878426
 
  66879016
 
  PDF   STATS   ENSEMBL   UCSC 
 
  591
 
 11
 
 14753
 
 gene;island
 
 0.09547
 
 0.03127
 
 Not queried
 
  1.6495944
 
 2.75e-04
 
 1.00e+00
  
 
  
 16
 
  66878426
 
  66878810
 
  PDF   STATS   ENSEMBL   UCSC 
 
  385
 
  9
 
 15255
 
 tss
 
 0.09002
 
 0.02797
 
 Not queried
 
  1.6495944
 
 2.88e-04
 
 1.00e+00
  
 
  
 1
 
 153600762
 
 153600972
 
  PDF   STATS   ENSEMBL   UCSC 
 
  211
 
  4
 
 761
 
 gene;tss
 
 0.21510
 
 0.47392
 
 Not queried
 
  1.6488188
 
 3.43e-01
 
 1.00e+00
  
 
  
 18
 
  56529784
 
  56529878
 
  PDF   STATS   ENSEMBL   UCSC 
 
   95
 
  5
 
 19262
 
 tss;island
 
 0.12915
 
 0.02393
 
 Not queried
 
  1.6485524
 
 5.56e-02
 
 1.00e+00
  
 
  
 2
 
 213400697
 
 213401333
 
  PDF   STATS   ENSEMBL   UCSC 
 
  637
 
  5
 
 22968
 
 gene;island
 
 0.01893
 
 0.02902
 
 Not queried
 
  1.6481931
 
 5.48e-01
 
 1.00e+00
  
 
  
 19
 
  10602587
 
  10602960
 
  PDF   STATS   ENSEMBL   UCSC 
 
  374
 
  4
 
 19777
 
 gene;island
 
 0.74709
 
 0.88150
 
 Not queried
 
  1.6478554
 
 8.86e-01
 
 1.00e+00
  
 
  
 18
 
  55862577
 
  55862872
 
  PDF   STATS   ENSEMBL   UCSC 
 
  296
 
  7
 
 19087
 
 gene;island
 
 0.66868
 
 0.84658
 
 Not queried
 
  1.6477900
 
 1.11e-02
 
 1.00e+00
  
 
  
 6
 
  29923913
 
  29924475
 
  PDF   STATS   ENSEMBL   UCSC 
 
  563
 
 13
 
 35583
 
 island
 
 0.32372
 
 0.64307
 
 Not queried
 
  1.6466411
 
 1.55e-03
 
 1.00e+00
  
 
  
 11
 
   1463541
 
   1463662
 
  PDF   STATS   ENSEMBL   UCSC 
 
  122
 
  4
 
 6401
 
 gene;island
 
 0.41047
 
 0.69190
 
 Not queried
 
  1.6463082
 
 1.14e-01
 
 1.00e+00
  
 
  
 4
 
  26321136
 
  26321356
 
  PDF   STATS   ENSEMBL   UCSC 
 
  221
 
  8
 
 30288
 
 tss;island
 
 0.04119
 
 0.01498
 
 Not queried
 
  1.6460621
 
 1.09e-03
 
 1.00e+00
  
 
  
 18
 
  51750950
 
  51751391
 
  PDF   STATS   ENSEMBL   UCSC 
 
  442
 
  7
 
 19488
 
 island
 
 0.01555
 
 0.02761
 
 Not queried
 
  1.6457539
 
 2.59e-01
 
 1.00e+00
  
 
  
 8
 
  38126881
 
  38127091
 
  PDF   STATS   ENSEMBL   UCSC 
 
  211
 
  8
 
 39592
 
 tss;island
 
 0.01210
 
 0.03926
 
 Not queried
 
  1.6455694
 
 1.48e-02
 
 1.00e+00
  
 
  
 4
 
   5021203
 
   5021328
 
  PDF   STATS   ENSEMBL   UCSC 
 
  126
 
  5
 
 30225
 
 tss
 
 0.62971
 
 0.42537
 
 Not queried
 
  1.6454946
 
 7.94e-03
 
 1.00e+00
  
 
  
 5
 
 145722132
 
 145722517
 
  PDF   STATS   ENSEMBL   UCSC 
 
  386
 
  5
 
 33220
 
 island
 
 0.30270
 
 0.49337
 
 Not queried
 
  1.6454264
 
 7.94e-03
 
 1.00e+00
  
 
  
 17
 
  22051978
 
  22052191
 
  PDF   STATS   ENSEMBL   UCSC 
 
  214
 
  4
 
 18097
 
 island
 
 0.22300
 
 0.48162
 
 Not queried
 
  1.6453466
 
 5.71e-02
 
 1.00e+00
  
 
  
 17
 
  35732737
 
  35732945
 
  PDF   STATS   ENSEMBL   UCSC 
 
  209
 
  4
 
 16496
 
 gene;tss
 
 0.87643
 
 0.95805
 
 Not queried
 
  1.6451763
 
 2.00e-01
 
 1.00e+00
  
 
  
 12
 
  54422127
 
  54422488
 
  PDF   STATS   ENSEMBL   UCSC 
 
  362
 
  5
 
 9134
 
 gene;island
 
 0.01847
 
 0.03376
 
 Not queried
 
  1.6448237
 
 5.48e-01
 
 1.00e+00
  
 
  
 19
 
  34918900
 
  34919248
 
  PDF   STATS   ENSEMBL   UCSC 
 
  349
 
  7
 
 20656
 
 tss
 
 0.01664
 
 0.02143
 
 Not queried
 
  1.6445317
 
 2.59e-01
 
 1.00e+00
  
 
  
 17
 
  53342593
 
  53342740
 
  PDF   STATS   ENSEMBL   UCSC 
 
  148
 
  4
 
 16665
 
 gene;island
 
 0.01701
 
 0.05427
 
 Not queried
 
  1.6444090
 
 1.14e-01
 
 1.00e+00
  
 
  
 2
 
  27505396
 
  27505593
 
  PDF   STATS   ENSEMBL   UCSC 
 
  198
 
  5
 
 23224
 
 tss
 
 0.56761
 
 0.25219
 
 Not queried
 
  1.6441620
 
 7.94e-03
 
 1.00e+00
  
 
  
 5
 
 172260464
 
 172260815
 
  PDF   STATS   ENSEMBL   UCSC 
 
  352
 
  4
 
 32436
 
 tss;island
 
 0.28482
 
 0.07410
 
 Not queried
 
  1.6439268
 
 4.86e-01
 
 1.00e+00
  
 
  
 6
 
  33384179
 
  33384537
 
  PDF   STATS   ENSEMBL   UCSC 
 
  359
 
  7
 
 34216
 
 gene;island
 
 0.68112
 
 0.91011
 
 Not queried
 
  1.6438883
 
 5.30e-02
 
 1.00e+00
  
 
  
 7
 
   8483249
 
   8483710
 
  PDF   STATS   ENSEMBL   UCSC 
 
  462
 
  4
 
 36830
 
 gene;island
 
 0.02370
 
 0.06640
 
 Not queried
 
  1.6436118
 
 2.00e-01
 
 1.00e+00
  
 
  
 11
 
  58830191
 
  58830610
 
  PDF   STATS   ENSEMBL   UCSC 
 
  420
 
  4
 
 8278
 
 island
 
 0.45531
 
 0.73054
 
 Not queried
 
  1.6431088
 
 2.86e-02
 
 1.00e+00
  
 
  
 3
 
 118892068
 
 118892330
 
  PDF   STATS   ENSEMBL   UCSC 
 
  263
 
  4
 
 28474
 
 tss
 
 0.30237
 
 0.11505
 
 Not queried
 
  1.6421357
 
 2.00e-01
 
 1.00e+00
  
 
  
 12
 
  48152204
 
  48152585
 
  PDF   STATS   ENSEMBL   UCSC 
 
  382
 
  6
 
 9080
 
 gene;tss;island
 
 0.42587
 
 0.18385
 
 Not queried
 
  1.6416951
 
 1.80e-01
 
 1.00e+00
  
 
  
 12
 
   1940380
 
   1940618
 
  PDF   STATS   ENSEMBL   UCSC 
 
  239
 
  4
 
 8976
 
 gene
 
 0.09243
 
 0.23111
 
 Not queried
 
  1.6415873
 
 2.86e-02
 
 1.00e+00
  
 
  
 20
 
  43729808
 
  43730241
 
  PDF   STATS   ENSEMBL   UCSC 
 
  434
 
  9
 
 25729
 
 tss;island
 
 0.33253
 
 0.07822
 
 Not queried
 
  1.6414573
 
 6.25e-02
 
 1.00e+00
  
 
  
 1
 
 236228598
 
 236228837
 
  PDF   STATS   ENSEMBL   UCSC 
 
  240
 
  6
 
 2335
 
 tss;island
 
 0.11111
 
 0.02810
 
 Not queried
 
  1.6411660
 
 2.40e-01
 
 1.00e+00
  
 
  
 19
 
   6534760
 
   6535078
 
  PDF   STATS   ENSEMBL   UCSC 
 
  319
 
  5
 
 19731
 
 gene;island
 
 0.92130
 
 0.84986
 
 Not queried
 
  1.6408627
 
 1.59e-02
 
 1.00e+00
  
 
  
 10
 
 118084109
 
 118084587
 
  PDF   STATS   ENSEMBL   UCSC 
 
  479
 
  6
 
 4760
 
 gene
 
 0.50910
 
 0.74227
 
 Not queried
 
  1.6396524
 
 1.52e-02
 
 1.00e+00
  
 
  
 5
 
  87968528
 
  87968749
 
  PDF   STATS   ENSEMBL   UCSC 
 
  222
 
  4
 
 31620
 
 gene;island
 
 0.14687
 
 0.04107
 
 Not queried
 
  1.6394404
 
 2.00e-01
 
 1.00e+00
  
 
  
 13
 
 114220436
 
 114220724
 
  PDF   STATS   ENSEMBL   UCSC 
 
  289
 
  4
 
 11943
 
 island
 
 0.68403
 
 0.87137
 
 Not queried
 
  1.6389252
 
 5.71e-02
 
 1.00e+00
  
 
  
 11
 
   8832817
 
   8833095
 
  PDF   STATS   ENSEMBL   UCSC 
 
  279
 
  6
 
 6482
 
 gene;tss
 
 0.58346
 
 0.30504
 
 Not queried
 
  1.6388277
 
 2.16e-03
 
 1.00e+00
  
 
  
 1
 
   3331987
 
   3332264
 
  PDF   STATS   ENSEMBL   UCSC 
 
  278
 
  6
 
 124
 
 gene;island
 
 0.58588
 
 0.74273
 
 Not queried
 
  1.6381469
 
 2.16e-03
 
 1.00e+00
  
 
  
 1
 
 155290236
 
 155290614
 
  PDF   STATS   ENSEMBL   UCSC 
 
  379
 
  7
 
 1992
 
 tss
 
 0.10960
 
 0.25611
 
 Not queried
 
  1.6380627
 
 3.18e-01
 
 1.00e+00
  
 
  
 14
 
  89259551
 
  89259748
 
  PDF   STATS   ENSEMBL   UCSC 
 
  198
 
  4
 
 12519
 
 tss;island
 
 0.04002
 
 0.18986
 
 Not queried
 
  1.6379864
 
 3.43e-01
 
 1.00e+00
  
 
  
 2
 
  11809681
 
  11810183
 
  PDF   STATS   ENSEMBL   UCSC 
 
  503
 
  5
 
 22537
 
 gene
 
 0.11813
 
 0.27273
 
 Not queried
 
  1.6378512
 
 4.21e-01
 
 1.00e+00
  
 
  
 6
 
  31549480
 
  31549631
 
  PDF   STATS   ENSEMBL   UCSC 
 
  152
 
  4
 
 33843
 
 gene;island
 
 0.66688
 
 0.42118
 
 Not queried
 
  1.6375782
 
 3.43e-01
 
 1.00e+00
  
 
  
 4
 
  99064459
 
  99064904
 
  PDF   STATS   ENSEMBL   UCSC 
 
  446
 
  7
 
 30460
 
 tss
 
 0.01127
 
 0.03424
 
 Not queried
 
  1.6371633
 
 1.65e-01
 
 1.00e+00
  
 
  
 16
 
  53407421
 
  53407808
 
  PDF   STATS   ENSEMBL   UCSC 
 
  388
 
  7
 
 15892
 
 island
 
 0.34412
 
 0.64560
 
 Not queried
 
  1.6366410
 
 2.33e-03
 
 1.00e+00
  
 
  
 17
 
  29886672
 
  29887049
 
  PDF   STATS   ENSEMBL   UCSC 
 
  378
 
  5
 
 18155
 
 island
 
 0.44708
 
 0.68640
 
 Not queried
 
  1.6364114
 
 7.94e-03
 
 1.00e+00
  
 
  
 15
 
  78632572
 
  78632861
 
  PDF   STATS   ENSEMBL   UCSC 
 
  290
 
  8
 
 14324
 
 island
 
 0.28015
 
 0.50222
 
 Not queried
 
  1.6363656
 
 4.66e-03
 
 1.00e+00
  
 
  
 2
 
  87088796
 
  87088843
 
  PDF   STATS   ENSEMBL   UCSC 
 
   48
 
  4
 
 22736
 
 gene;island
 
 0.57758
 
 0.80659
 
 Not queried
 
  1.6359853
 
 2.86e-02
 
 1.00e+00
  
 
  
 7
 
 127670993
 
 127671195
 
  PDF   STATS   ENSEMBL   UCSC 
 
  203
 
  6
 
 37147
 
 gene;island
 
 0.08400
 
 0.03329
 
 Not queried
 
  1.6352796
 
 4.11e-02
 
 1.00e+00
  
 
  
 5
 
   5139334
 
   5140029
 
  PDF   STATS   ENSEMBL   UCSC 
 
  696
 
 17
 
 31920
 
 tss;island
 
 0.70943
 
 0.41940
 
 Not queried
 
  1.6350685
 
 1.40e-01
 
 1.00e+00
  
 
  
 6
 
  31866508
 
  31866825
 
  PDF   STATS   ENSEMBL   UCSC 
 
  318
 
  4
 
 34786
 
 tss;island
 
 0.25109
 
 0.46706
 
 Not queried
 
  1.6349189
 
 8.86e-01
 
 1.00e+00
  
 
  
 7
 
 100823700
 
 100823790
 
  PDF   STATS   ENSEMBL   UCSC 
 
   91
 
  4
 
 37735
 
 tss;island
 
 0.36607
 
 0.15509
 
 Not queried
 
  1.6347587
 
 1.14e-01
 
 1.00e+00
  
 
  
 11
 
  32459428
 
  32459954
 
  PDF   STATS   ENSEMBL   UCSC 
 
  527
 
  4
 
 6551
 
 gene;island
 
 0.16873
 
 0.08582
 
 Not queried
 
  1.6346394
 
 3.43e-01
 
 1.00e+00
  
 
  
 1
 
   3028813
 
   3029070
 
  PDF   STATS   ENSEMBL   UCSC 
 
  258
 
  6
 
 99
 
 gene;island
 
 0.70999
 
 0.87204
 
 Not queried
 
  1.6343340
 
 2.60e-02
 
 1.00e+00
  
 
  
 11
 
  19367501
 
  19368277
 
  PDF   STATS   ENSEMBL   UCSC 
 
  777
 
  8
 
 8099
 
 island
 
 0.10777
 
 0.27030
 
 Not queried
 
  1.6342034
 
 6.50e-02
 
 1.00e+00
  
 
  
 13
 
 112712424
 
 112712795
 
  PDF   STATS   ENSEMBL   UCSC 
 
  372
 
  4
 
 11878
 
 island
 
 0.42603
 
 0.26657
 
 Not queried
 
  1.6340743
 
 4.86e-01
 
 1.00e+00
  
 
  
 10
 
 134977981
 
 134978147
 
  PDF   STATS   ENSEMBL   UCSC 
 
  167
 
  5
 
 4871
 
 gene;island
 
 0.66303
 
 0.87365
 
 Not queried
 
  1.6340224
 
 9.52e-02
 
 1.00e+00
  
 
  
 17
 
  39019545
 
  39020053
 
  PDF   STATS   ENSEMBL   UCSC 
 
  509
 
  6
 
 16533
 
 gene;island
 
 0.45903
 
 0.70880
 
 Not queried
 
  1.6335788
 
 1.80e-01
 
 1.00e+00
  
 
  
 6
 
  32086755
 
  32087190
 
  PDF   STATS   ENSEMBL   UCSC 
 
  436
 
 12
 
 33991
 
 gene
 
 0.27251
 
 0.46857
 
 Not queried
 
  1.6335019
 
 5.97e-02
 
 1.00e+00
  
 
  
 19
 
  58446312
 
  58446669
 
  PDF   STATS   ENSEMBL   UCSC 
 
  358
 
  4
 
 20217
 
 gene
 
 0.04161
 
 0.11891
 
 Not queried
 
  1.6330978
 
 2.00e-01
 
 1.00e+00
  
 
  
 6
 
  33400477
 
  33400543
 
  PDF   STATS   ENSEMBL   UCSC 
 
   67
 
  4
 
 34226
 
 gene
 
 0.72720
 
 0.89327
 
 Not queried
 
  1.6323640
 
 5.71e-02
 
 1.00e+00
  
 
  
 6
 
  29364418
 
  29365287
 
  PDF   STATS   ENSEMBL   UCSC 
 
  870
 
 11
 
 33591
 
 gene
 
 0.46583
 
 0.79273
 
 Not queried
 
  1.6320140
 
 4.73e-02
 
 1.00e+00
  
 
  
 17
 
  38599271
 
  38599508
 
  PDF   STATS   ENSEMBL   UCSC 
 
  238
 
  4
 
 17301
 
 tss
 
 0.10602
 
 0.05807
 
 Not queried
 
  1.6313830
 
 4.86e-01
 
 1.00e+00
  
 
  
 11
 
 117668035
 
 117668336
 
  PDF   STATS   ENSEMBL   UCSC 
 
  302
 
  8
 
 7700
 
 tss
 
 0.44787
 
 0.23213
 
 Not queried
 
  1.6311420
 
 1.30e-01
 
 1.00e+00
  
 
  
 12
 
 132976722
 
 132977140
 
  PDF   STATS   ENSEMBL   UCSC 
 
  419
 
  4
 
 11021
 
 island
 
 0.42008
 
 0.70950
 
 Not queried
 
  1.6309791
 
 5.71e-02
 
 1.00e+00
  
 
  
 17
 
  38249266
 
  38249729
 
  PDF   STATS   ENSEMBL   UCSC 
 
  464
 
  6
 
 16524
 
 gene;island
 
 0.66051
 
 0.88565
 
 Not queried
 
  1.6305679
 
 1.32e-01
 
 1.00e+00
  
 
  
 18
 
  21452730
 
  21452895
 
  PDF   STATS   ENSEMBL   UCSC 
 
  166
 
  6
 
 19042
 
 gene;tss
 
 0.57022
 
 0.84036
 
 Not queried
 
  1.6304986
 
 2.60e-02
 
 1.00e+00
  
 
  
 22
 
  38071001
 
  38071188
 
  PDF   STATS   ENSEMBL   UCSC 
 
  188
 
  4
 
 27078
 
 tss;island
 
 0.13773
 
 0.32499
 
 Not queried
 
  1.6302885
 
 2.86e-02
 
 1.00e+00
  
 
  
 3
 
 193587490
 
 193587939
 
  PDF   STATS   ENSEMBL   UCSC 
 
  450
 
  4
 
 29734
 
 island
 
 0.58926
 
 0.80841
 
 Not queried
 
  1.6301080
 
 2.00e-01
 
 1.00e+00
  
 
  
 10
 
  77165618
 
  77165905
 
  PDF   STATS   ENSEMBL   UCSC 
 
  288
 
  4
 
 4631
 
 gene;island
 
 0.71300
 
 0.53851
 
 Not queried
 
  1.6299463
 
 3.43e-01
 
 1.00e+00
  
 
  
 1
 
 203242409
 
 203242638
 
  PDF   STATS   ENSEMBL   UCSC 
 
  230
 
  4
 
 4088
 
 island
 
 0.05677
 
 0.20576
 
 Not queried
 
  1.6293476
 
 3.43e-01
 
 1.00e+00
  
 
  
 1
 
  86888585
 
  86888728
 
  PDF   STATS   ENSEMBL   UCSC 
 
  144
 
  4
 
 1724
 
 tss
 
 0.87667
 
 0.66412
 
 Not queried
 
  1.6287418
 
 1.14e-01
 
 1.00e+00
  
 
  
 10
 
  71892593
 
  71892787
 
  PDF   STATS   ENSEMBL   UCSC 
 
  195
 
  7
 
 5793
 
 island
 
 0.07312
 
 0.19906
 
 Not queried
 
  1.6286682
 
 2.62e-02
 
 1.00e+00
  
 
  
 6
 
  74072242
 
  74072447
 
  PDF   STATS   ENSEMBL   UCSC 
 
  206
 
  7
 
 36189
 
 island
 
 0.74862
 
 0.87599
 
 Not queried
 
  1.6284301
 
 5.30e-02
 
 1.00e+00
  
 
  
 16
 
  29757344
 
  29757461
 
  PDF   STATS   ENSEMBL   UCSC 
 
  118
 
  4
 
 15136
 
 tss
 
 0.72582
 
 0.87920
 
 Not queried
 
  1.6283152
 
 2.00e-01
 
 1.00e+00
  
 
  
 17
 
  28563054
 
  28563300
 
  PDF   STATS   ENSEMBL   UCSC 
 
  247
 
  5
 
 17205
 
 tss;island
 
 0.03719
 
 0.10731
 
 Not queried
 
  1.6282830
 
 2.22e-01
 
 1.00e+00
  
 
  
 5
 
 133451237
 
 133451474
 
  PDF   STATS   ENSEMBL   UCSC 
 
  238
 
  7
 
 32228
 
 tss
 
 0.17086
 
 0.03041
 
 Not queried
 
  1.6280495
 
 5.30e-02
 
 1.00e+00
  
 
  
 8
 
  23082721
 
  23082961
 
  PDF   STATS   ENSEMBL   UCSC 
 
  241
 
  8
 
 39535
 
 tss
 
 0.11069
 
 0.04791
 
 Not queried
 
  1.6278157
 
 2.81e-02
 
 1.00e+00
  
 
  
 19
 
  52408259
 
  52408520
 
  PDF   STATS   ENSEMBL   UCSC 
 
  262
 
  8
 
 20964
 
 tss
 
 0.06886
 
 0.02730
 
 Not queried
 
  1.6276882
 
 4.99e-02
 
 1.00e+00
  
 
  
 8
 
 142238691
 
 142239055
 
  PDF   STATS   ENSEMBL   UCSC 
 
  365
 
  6
 
 39864
 
 tss
 
 0.88603
 
 0.94511
 
 Not queried
 
  1.6270185
 
 6.49e-02
 
 1.00e+00
  
 
  
 4
 
  48988015
 
  48988321
 
  PDF   STATS   ENSEMBL   UCSC 
 
  307
 
  7
 
 30985
 
 island
 
 0.30016
 
 0.14263
 
 Not queried
 
  1.6265854
 
 3.79e-02
 
 1.00e+00
  
 
  
 7
 
  94953653
 
  94953877
 
  PDF   STATS   ENSEMBL   UCSC 
 
  225
 
  5
 
 37033
 
 gene
 
 0.61852
 
 0.71747
 
 Not queried
 
  1.6263750
 
 4.21e-01
 
 1.00e+00
  
 
  
 17
 
  59668571
 
  59668616
 
  PDF   STATS   ENSEMBL   UCSC 
 
   46
 
  6
 
 17525
 
 tss
 
 0.63448
 
 0.85894
 
 Not queried
 
  1.6261866
 
 6.49e-02
 
 1.00e+00
  
 
  
 6
 
 160783785
 
 160784044
 
  PDF   STATS   ENSEMBL   UCSC 
 
  260
 
  4
 
 34470
 
 gene;island
 
 0.85808
 
 0.70585
 
 Not queried
 
  1.6260722
 
 2.86e-02
 
 1.00e+00
  
 
  
 1
 
 223254706
 
 223255040
 
  PDF   STATS   ENSEMBL   UCSC 
 
  335
 
  5
 
 4220
 
 island
 
 0.08357
 
 0.02991
 
 Not queried
 
  1.6259311
 
 1.51e-01
 
 1.00e+00
  
 
  
 16
 
  69141250
 
  69141437
 
  PDF   STATS   ENSEMBL   UCSC 
 
  188
 
  5
 
 15295
 
 tss
 
 0.10138
 
 0.03527
 
 Not queried
 
  1.6257353
 
 9.52e-02
 
 1.00e+00
  
 
  
 19
 
  11531294
 
  11531519
 
  PDF   STATS   ENSEMBL   UCSC 
 
  226
 
  5
 
 19804
 
 gene;island
 
 0.03673
 
 0.07334
 
 Not queried
 
  1.6256110
 
 2.22e-01
 
 1.00e+00
  
 
  
 6
 
  30074595
 
  30074764
 
  PDF   STATS   ENSEMBL   UCSC 
 
  170
 
  4
 
 33659
 
 gene;island
 
 0.72782
 
 0.55527
 
 Not queried
 
  1.6252765
 
 3.43e-01
 
 1.00e+00
  
 
  
 7
 
  27207996
 
  27208285
 
  PDF   STATS   ENSEMBL   UCSC 
 
  290
 
  4
 
 38293
 
 island
 
 0.90751
 
 0.72591
 
 Not queried
 
  1.6252248
 
 5.71e-02
 
 1.00e+00
  
 
  
 2
 
  97523237
 
  97523478
 
  PDF   STATS   ENSEMBL   UCSC 
 
  242
 
  4
 
 22751
 
 gene;island
 
 0.02814
 
 0.02552
 
 Not queried
 
  1.6251431
 
 6.86e-01
 
 1.00e+00
  
 
  
 2
 
 132233726
 
 132234134
 
  PDF   STATS   ENSEMBL   UCSC 
 
  409
 
  5
 
 22834
 
 gene
 
 0.96181
 
 0.88415
 
 Not queried
 
  1.6250263
 
 3.17e-02
 
 1.00e+00
  
 
  
 2
 
 132233433
 
 132234134
 
  PDF   STATS   ENSEMBL   UCSC 
 
  702
 
  9
 
 24695
 
 island
 
 0.96181
 
 0.88099
 
 Not queried
 
  1.6250263
 
 7.77e-03
 
 1.00e+00
  
 
  
 6
 
  28192892
 
  28193049
 
  PDF   STATS   ENSEMBL   UCSC 
 
  158
 
  5
 
 34672
 
 tss
 
 0.02296
 
 0.05346
 
 Not queried
 
  1.6248329
 
 3.10e-01
 
 1.00e+00
  
 
  
 21
 
  46131464
 
  46131923
 
  PDF   STATS   ENSEMBL   UCSC 
 
  460
 
  6
 
 26699
 
 island
 
 0.36765
 
 0.63322
 
 Not queried
 
  1.6246602
 
 1.52e-02
 
 1.00e+00
  
 
  
 11
 
   2193041
 
   2193293
 
  PDF   STATS   ENSEMBL   UCSC 
 
  253
 
  4
 
 7043
 
 tss
 
 0.47503
 
 0.82592
 
 Not queried
 
  1.6243857
 
 1.14e-01
 
 1.00e+00
  
 
  
 5
 
  92929319
 
  92929679
 
  PDF   STATS   ENSEMBL   UCSC 
 
  361
 
  5
 
 31633
 
 gene;island
 
 0.77202
 
 0.42354
 
 Not queried
 
  1.6242979
 
 9.52e-02
 
 1.00e+00
  
 
  
 13
 
 114214248
 
 114214740
 
  PDF   STATS   ENSEMBL   UCSC 
 
  493
 
  5
 
 11942
 
 island
 
 0.90237
 
 0.96759
 
 Not queried
 
  1.6242321
 
 5.56e-02
 
 1.00e+00
  
 
  
 14
 
  60974384
 
  60974525
 
  PDF   STATS   ENSEMBL   UCSC 
 
  142
 
  5
 
 12876
 
 island
 
 0.66508
 
 0.40504
 
 Not queried
 
  1.6238349
 
 3.10e-01
 
 1.00e+00
  
 
  
 1
 
   5932351
 
   5932678
 
  PDF   STATS   ENSEMBL   UCSC 
 
  328
 
  5
 
 154
 
 gene
 
 0.85515
 
 0.97393
 
 Not queried
 
  1.6237346
 
 4.21e-01
 
 1.00e+00
  
 
  
 21
 
  34398085
 
  34398226
 
  PDF   STATS   ENSEMBL   UCSC 
 
  142
 
  6
 
 26475
 
 tss
 
 0.01371
 
 0.04953
 
 Not queried
 
  1.6236977
 
 1.52e-02
 
 1.00e+00
  
 
  
 6
 
  32781171
 
  32781384
 
  PDF   STATS   ENSEMBL   UCSC 
 
  214
 
  5
 
 34067
 
 gene
 
 0.83424
 
 0.50996
 
 Not queried
 
  1.6236301
 
 2.22e-01
 
 1.00e+00
  
 
  
 2
 
  85637673
 
  85637851
 
  PDF   STATS   ENSEMBL   UCSC 
 
  179
 
  4
 
 24439
 
 island
 
 0.45597
 
 0.68240
 
 Not queried
 
  1.6235254
 
 2.86e-02
 
 1.00e+00
  
 
  
 1
 
 101360652
 
 101360775
 
  PDF   STATS   ENSEMBL   UCSC 
 
  124
 
  4
 
 1777
 
 tss;island
 
 0.04061
 
 0.01607
 
 Not queried
 
  1.6232570
 
 4.86e-01
 
 1.00e+00
  
 
  
 X
 
 152954816
 
 152954965
 
  PDF   STATS   ENSEMBL   UCSC 
 
  150
 
  7
 
 41144
 
 gene
 
 0.76859
 
 0.58614
 
 Not queried
 
  1.6226940
 
 1.17e-03
 
 1.00e+00
  
 
  
 2
 
 242811830
 
 242811962
 
  PDF   STATS   ENSEMBL   UCSC 
 
  133
 
  4
 
 25275
 
 island
 
 0.73400
 
 0.88735
 
 Not queried
 
  1.6224502
 
 3.43e-01
 
 1.00e+00
  
 
  
 8
 
 144814911
 
 144815275
 
  PDF   STATS   ENSEMBL   UCSC 
 
  365
 
  4
 
 39427
 
 gene;island
 
 0.28904
 
 0.54113
 
 Not queried
 
  1.6205896
 
 2.00e-01
 
 1.00e+00
  
 
  
 11
 
  50257496
 
  50257979
 
  PDF   STATS   ENSEMBL   UCSC 
 
  484
 
  7
 
 8257
 
 island
 
 0.49823
 
 0.71297
 
 Not queried
 
  1.6202671
 
 1.75e-02
 
 1.00e+00
  
 
  
 8
 
  11471395
 
  11471881
 
  PDF   STATS   ENSEMBL   UCSC 
 
  487
 
  5
 
 40018
 
 island
 
 0.60925
 
 0.84758
 
 Not queried
 
  1.6194757
 
 1.59e-02
 
 1.00e+00
  
 
  
 5
 
 148521450
 
 148521623
 
  PDF   STATS   ENSEMBL   UCSC 
 
  174
 
  4
 
 31799
 
 gene;island
 
 0.11348
 
 0.27511
 
 Not queried
 
  1.6194253
 
 2.86e-02
 
 1.00e+00
  
 
  
 17
 
  72931569
 
  72932048
 
  PDF   STATS   ENSEMBL   UCSC 
 
  480
 
 12
 
 18696
 
 island
 
 0.20095
 
 0.45905
 
 Not queried
 
  1.6190519
 
 6.56e-04
 
 1.00e+00
  
 
  
 2
 
 177027440
 
 177027931
 
  PDF   STATS   ENSEMBL   UCSC 
 
  492
 
  6
 
 23703
 
 tss
 
 0.83681
 
 0.51987
 
 Not queried
 
  1.6189886
 
 1.32e-01
 
 1.00e+00
  
 
  
 2
 
  87304015
 
  87304216
 
  PDF   STATS   ENSEMBL   UCSC 
 
  202
 
  4
 
 23459
 
 tss;island
 
 0.02320
 
 0.05820
 
 Not queried
 
  1.6188981
 
 2.86e-02
 
 1.00e+00
  
 
  
 1
 
   7740243
 
   7740286
 
  PDF   STATS   ENSEMBL   UCSC 
 
   44
 
  4
 
 186
 
 gene;island
 
 0.02128
 
 0.07774
 
 Not queried
 
  1.6183216
 
 2.00e-01
 
 1.00e+00
  
 
  
 19
 
  19007311
 
  19007647
 
  PDF   STATS   ENSEMBL   UCSC 
 
  337
 
  5
 
 20594
 
 tss;island
 
 0.87591
 
 0.43218
 
 Not queried
 
  1.6182323
 
 5.56e-02
 
 1.00e+00
  
 
  
 2
 
 220159595
 
 220159976
 
  PDF   STATS   ENSEMBL   UCSC 
 
  382
 
  4
 
 22995
 
 gene;tss;island
 
 0.03256
 
 0.16515
 
 Not queried
 
  1.6175650
 
 1.14e-01
 
 1.00e+00
  
 
  
 8
 
  26047780
 
  26048065
 
  PDF   STATS   ENSEMBL   UCSC 
 
  286
 
  5
 
 40108
 
 island
 
 0.19258
 
 0.36168
 
 Not queried
 
  1.6164406
 
 1.51e-01
 
 1.00e+00
  
 
  
 2
 
  27439738
 
  27439915
 
  PDF   STATS   ENSEMBL   UCSC 
 
  178
 
  4
 
 22587
 
 gene;tss;island
 
 0.85895
 
 0.94569
 
 Not queried
 
  1.6160906
 
 4.86e-01
 
 1.00e+00
  
 
  
 4
 
   1004110
 
   1005405
 
  PDF   STATS   ENSEMBL   UCSC 
 
 1296
 
 28
 
 30188
 
 tss;island
 
 0.03233
 
 0.16594
 
 Not queried
 
  1.6160668
 
 3.91e-03
 
 1.00e+00
  
 
  
 3
 
  52351355
 
  52351963
 
  PDF   STATS   ENSEMBL   UCSC 
 
  609
 
  5
 
 27775
 
 gene
 
 0.01781
 
 0.05266
 
 Not queried
 
  1.6160125
 
 2.22e-01
 
 1.00e+00
  
 
  
 10
 
  77871958
 
  77872458
 
  PDF   STATS   ENSEMBL   UCSC 
 
  501
 
  5
 
 4632
 
 gene;island
 
 0.61922
 
 0.84003
 
 Not queried
 
  1.6159753
 
 9.52e-02
 
 1.00e+00
  
 
  
 10
 
 112289853
 
 112290331
 
  PDF   STATS   ENSEMBL   UCSC 
 
  479
 
  7
 
 6112
 
 island
 
 0.03702
 
 0.09031
 
 Not queried
 
  1.6158582
 
 1.11e-02
 
 1.00e+00
  
 
  
 11
 
  72982929
 
  72983097
 
  PDF   STATS   ENSEMBL   UCSC 
 
  169
 
  4
 
 6802
 
 gene;tss
 
 0.54670
 
 0.83990
 
 Not queried
 
  1.6158216
 
 5.71e-02
 
 1.00e+00
  
 
  
 5
 
   7851280
 
   7851681
 
  PDF   STATS   ENSEMBL   UCSC 
 
  402
 
  5
 
 31924
 
 tss
 
 0.45809
 
 0.16073
 
 Not queried
 
  1.6152964
 
 6.90e-01
 
 1.00e+00
  
 
  
 11
 
  85338771
 
  85338971
 
  PDF   STATS   ENSEMBL   UCSC 
 
  201
 
  4
 
 7581
 
 tss;island
 
 0.03189
 
 0.01180
 
 Not queried
 
  1.6144517
 
 2.00e-01
 
 1.00e+00
  
 
  
 5
 
 140602851
 
 140603034
 
  PDF   STATS   ENSEMBL   UCSC 
 
  184
 
  4
 
 32308
 
 tss;island
 
 0.02472
 
 0.03437
 
 Not queried
 
  1.6144320
 
 6.86e-01
 
 1.00e+00
  
 
  
 3
 
  42947263
 
  42947565
 
  PDF   STATS   ENSEMBL   UCSC 
 
  303
 
  5
 
 28208
 
 tss
 
 0.89356
 
 0.96023
 
 Not queried
 
  1.6141005
 
 5.56e-02
 
 1.00e+00
  
 
  
 4
 
 135865398
 
 135865584
 
  PDF   STATS   ENSEMBL   UCSC 
 
  187
 
  4
 
 31254
 
 island
 
 0.86824
 
 0.92961
 
 Not queried
 
  1.6136782
 
 3.43e-01
 
 1.00e+00
  
 
  
 2
 
 218898491
 
 218898788
 
  PDF   STATS   ENSEMBL   UCSC 
 
  298
 
  4
 
 25040
 
 island
 
 0.65695
 
 0.38695
 
 Not queried
 
  1.6134408
 
 5.71e-02
 
 1.00e+00
  
 
  
 6
 
 138745349
 
 138745739
 
  PDF   STATS   ENSEMBL   UCSC 
 
  391
 
  5
 
 34423
 
 gene;island
 
 0.62649
 
 0.89031
 
 Not queried
 
  1.6132130
 
 2.22e-01
 
 1.00e+00
  
 
  
 1
 
 152733025
 
 152733395
 
  PDF   STATS   ENSEMBL   UCSC 
 
  371
 
  6
 
 757
 
 gene;island
 
 0.24895
 
 0.54743
 
 Not queried
 
  1.6118472
 
 2.16e-03
 
 1.00e+00
  
 
  
 2
 
   9514739
 
   9514994
 
  PDF   STATS   ENSEMBL   UCSC 
 
  256
 
  4
 
 22525
 
 gene;island
 
 0.58726
 
 0.81324
 
 Not queried
 
  1.6112389
 
 2.00e-01
 
 1.00e+00
  
 
  
 19
 
  19322527
 
  19322902
 
  PDF   STATS   ENSEMBL   UCSC 
 
  376
 
  8
 
 21773
 
 island
 
 0.02018
 
 0.05208
 
 Not queried
 
  1.6111760
 
 1.95e-01
 
 1.00e+00
  
 
  
 17
 
    693137
 
    693388
 
  PDF   STATS   ENSEMBL   UCSC 
 
  252
 
  4
 
 16259
 
 gene
 
 0.67559
 
 0.84226
 
 Not queried
 
  1.6111521
 
 5.71e-02
 
 1.00e+00
  
 
  
 7
 
   1054075
 
   1054449
 
  PDF   STATS   ENSEMBL   UCSC 
 
  375
 
  6
 
 36672
 
 gene;island
 
 0.69939
 
 0.92463
 
 Not queried
 
  1.6109610
 
 6.49e-02
 
 1.00e+00
  
 
  
 X
 
  30265129
 
  30265447
 
  PDF   STATS   ENSEMBL   UCSC 
 
  319
 
  8
 
 40954
 
 gene;island
 
 0.82007
 
 0.92055
 
 Not queried
 
  1.6106801
 
 6.99e-03
 
 1.00e+00
  
 
  
 7
 
  56160717
 
  56161020
 
  PDF   STATS   ENSEMBL   UCSC 
 
  304
 
  7
 
 37544
 
 tss
 
 0.25045
 
 0.52937
 
 Not queried
 
  1.6106429
 
 1.75e-02
 
 1.00e+00
  
 
  
 17
 
   2240014
 
   2240362
 
  PDF   STATS   ENSEMBL   UCSC 
 
  349
 
  4
 
 16294
 
 gene;tss;island
 
 0.03335
 
 0.01117
 
 Not queried
 
  1.6105198
 
 3.43e-01
 
 1.00e+00
  
 
  
 16
 
  46603015
 
  46603321
 
  PDF   STATS   ENSEMBL   UCSC 
 
  307
 
  6
 
 15187
 
 tss
 
 0.77050
 
 0.93244
 
 Not queried
 
  1.6097996
 
 9.31e-02
 
 1.00e+00
  
 
  
 14
 
  36993831
 
  36994456
 
  PDF   STATS   ENSEMBL   UCSC 
 
  626
 
  5
 
 12758
 
 island
 
 0.03056
 
 0.09807
 
 Not queried
 
  1.6089756
 
 1.51e-01
 
 1.00e+00
  
 
  
 12
 
   8332808
 
   8333192
 
  PDF   STATS   ENSEMBL   UCSC 
 
  385
 
  5
 
 9024
 
 gene
 
 0.04595
 
 0.18114
 
 Not queried
 
  1.6088801
 
 2.22e-01
 
 1.00e+00
  
 
  
 12
 
   8332640
 
   8333192
 
  PDF   STATS   ENSEMBL   UCSC 
 
  553
 
  9
 
 10207
 
 island
 
 0.03556
 
 0.12003
 
 Not queried
 
  1.6088801
 
 1.13e-01
 
 1.00e+00
  
 
  
 22
 
  51001330
 
  51001387
 
  PDF   STATS   ENSEMBL   UCSC 
 
   58
 
  5
 
 27217
 
 tss
 
 0.00397
 
 0.01630
 
 Not queried
 
  1.6082514
 
 3.10e-01
 
 1.00e+00
  
 
  
 19
 
   7968429
 
   7968594
 
  PDF   STATS   ENSEMBL   UCSC 
 
  166
 
  6
 
 20374
 
 tss;island
 
 0.00624
 
 0.01850
 
 Not queried
 
  1.6081486
 
 1.32e-01
 
 1.00e+00
  
 
  
 12
 
 131512480
 
 131512597
 
  PDF   STATS   ENSEMBL   UCSC 
 
  118
 
  4
 
 9396
 
 gene
 
 0.78096
 
 0.90493
 
 Not queried
 
  1.6073733
 
 3.43e-01
 
 1.00e+00
  
 
  
 1
 
 147782116
 
 147782558
 
  PDF   STATS   ENSEMBL   UCSC 
 
  443
 
  5
 
 3691
 
 island
 
 0.84004
 
 0.70931
 
 Not queried
 
  1.6063609
 
 5.56e-02
 
 1.00e+00
  
 
  
 8
 
 144820683
 
 144820908
 
  PDF   STATS   ENSEMBL   UCSC 
 
  226
 
  4
 
 40620
 
 island
 
 0.53025
 
 0.77865
 
 Not queried
 
  1.6056663
 
 1.14e-01
 
 1.00e+00
  
 
  
 10
 
 135340445
 
 135340850
 
  PDF   STATS   ENSEMBL   UCSC 
 
  406
 
  7
 
 5447
 
 tss
 
 0.94705
 
 0.84129
 
 Not queried
 
  1.6055725
 
 2.62e-02
 
 1.00e+00
  
 
  
 10
 
 135340445
 
 135341025
 
  PDF   STATS   ENSEMBL   UCSC 
 
  581
 
  9
 
 6349
 
 island
 
 0.94213
 
 0.76704
 
 Not queried
 
  1.6055725
 
 1.42e-02
 
 1.00e+00
  
 
  
 X
 
 103810908
 
 103810984
 
  PDF   STATS   ENSEMBL   UCSC 
 
   77
 
  4
 
 41452
 
 tss
 
 0.01732
 
 0.03914
 
 Not queried
 
  1.6052755
 
 2.00e-01
 
 1.00e+00
  
 
  
 5
 
 133304474
 
 133304652
 
  PDF   STATS   ENSEMBL   UCSC 
 
  179
 
  5
 
 32226
 
 tss
 
 0.01050
 
 0.02276
 
 Not queried
 
  1.6047643
 
 3.17e-02
 
 1.00e+00
  
 
  
 1
 
  26372718
 
  26373407
 
  PDF   STATS   ENSEMBL   UCSC 
 
  690
 
  7
 
 1377
 
 tss;island
 
 0.68227
 
 0.34487
 
 Not queried
 
  1.6045014
 
 5.35e-01
 
 1.00e+00
  
 
  
 19
 
  22193293
 
  22193724
 
  PDF   STATS   ENSEMBL   UCSC 
 
  432
 
  5
 
 19928
 
 gene
 
 0.88941
 
 0.72538
 
 Not queried
 
  1.6043288
 
 2.22e-01
 
 1.00e+00
  
 
  
 19
 
  22193293
 
  22193911
 
  PDF   STATS   ENSEMBL   UCSC 
 
  619
 
  7
 
 21806
 
 island
 
 0.88941
 
 0.72538
 
 Not queried
 
  1.6043288
 
 1.65e-01
 
 1.00e+00
  
 
  
 11
 
  15962841
 
  15963167
 
  PDF   STATS   ENSEMBL   UCSC 
 
  327
 
  5
 
 8065
 
 island
 
 0.19455
 
 0.44953
 
 Not queried
 
  1.6043073
 
 3.10e-01
 
 1.00e+00
  
 
  
 1
 
 248903300
 
 248903467
 
  PDF   STATS   ENSEMBL   UCSC 
 
  168
 
  4
 
 2369
 
 tss
 
 0.67637
 
 0.43946
 
 Not queried
 
  1.6023557
 
 2.86e-02
 
 1.00e+00
  
 
  
 3
 
  50540137
 
  50540261
 
  PDF   STATS   ENSEMBL   UCSC 
 
  125
 
  4
 
 27769
 
 gene;island
 
 0.02432
 
 0.08289
 
 Not queried
 
  1.6015500
 
 3.43e-01
 
 1.00e+00
  
 
  
 X
 
  37209004
 
  37209388
 
  PDF   STATS   ENSEMBL   UCSC 
 
  385
 
  4
 
 40962
 
 gene;island
 
 0.02917
 
 0.09203
 
 Not queried
 
  1.6012722
 
 3.43e-01
 
 1.00e+00
  
 
  
 6
 
 168388195
 
 168388388
 
  PDF   STATS   ENSEMBL   UCSC 
 
  194
 
  4
 
 36589
 
 island
 
 0.90716
 
 0.76407
 
 Not queried
 
  1.6005498
 
 2.00e-01
 
 1.00e+00
  
 
  
 7
 
    612458
 
    612876
 
  PDF   STATS   ENSEMBL   UCSC 
 
  419
 
  4
 
 36650
 
 gene;island
 
 0.56382
 
 0.74125
 
 Not queried
 
  1.6003998
 
 4.86e-01
 
 1.00e+00
  
 
  
 18
 
  59560091
 
  59560495
 
  PDF   STATS   ENSEMBL   UCSC 
 
  405
 
  8
 
 19512
 
 island
 
 0.00968
 
 0.03486
 
 Not queried
 
  1.6002015
 
 1.05e-01
 
 1.00e+00
  
 
  
 2
 
 139660149
 
 139660341
 
  PDF   STATS   ENSEMBL   UCSC 
 
  193
 
  4
 
 24727
 
 island
 
 0.71598
 
 0.83329
 
 Not queried
 
  1.6001153
 
 2.00e-01
 
 1.00e+00
  
 
  
 7
 
 157849615
 
 157849794
 
  PDF   STATS   ENSEMBL   UCSC 
 
  180
 
  5
 
 37294
 
 gene
 
 0.48955
 
 0.93100
 
 Not queried
 
  1.6000345
 
 5.56e-02
 
 1.00e+00
  
 
  
 2
 
 219861012
 
 219861150
 
  PDF   STATS   ENSEMBL   UCSC 
 
  139
 
  5
 
 25063
 
 island
 
 0.02203
 
 0.05908
 
 Not queried
 
  1.5998763
 
 3.10e-01
 
 1.00e+00
  
 
  
 20
 
  61885236
 
  61885448
 
  PDF   STATS   ENSEMBL   UCSC 
 
  213
 
  7
 
 25460
 
 gene;island
 
 0.00675
 
 0.01241
 
 Not queried
 
  1.5992340
 
 2.59e-01
 
 1.00e+00
  
 
  
 11
 
  94883242
 
  94883516
 
  PDF   STATS   ENSEMBL   UCSC 
 
  275
 
  4
 
 8722
 
 island
 
 0.18670
 
 0.10568
 
 Not queried
 
  1.5986915
 
 1.14e-01
 
 1.00e+00
  
 
  
 7
 
 134250141
 
 134250223
 
  PDF   STATS   ENSEMBL   UCSC 
 
   83
 
  4
 
 37847
 
 tss
 
 0.78022
 
 0.59435
 
 Not queried
 
  1.5982526
 
 2.00e-01
 
 1.00e+00
  
 
  
 7
 
 142583555
 
 142583836
 
  PDF   STATS   ENSEMBL   UCSC 
 
  282
 
  4
 
 37884
 
 tss
 
 0.04340
 
 0.13478
 
 Not queried
 
  1.5981583
 
 3.43e-01
 
 1.00e+00
  
 
  
 1
 
   2984218
 
   2984869
 
  PDF   STATS   ENSEMBL   UCSC 
 
  652
 
 10
 
 2523
 
 island
 
 0.59984
 
 0.80396
 
 Not queried
 
  1.5974720
 
 8.93e-03
 
 1.00e+00
  
 
  
 7
 
  75831232
 
  75831678
 
  PDF   STATS   ENSEMBL   UCSC 
 
  447
 
  5
 
 36988
 
 gene
 
 0.03890
 
 0.04948
 
 Not queried
 
  1.5971945
 
 4.21e-01
 
 1.00e+00
  
 
  
 3
 
 127634188
 
 127634587
 
  PDF   STATS   ENSEMBL   UCSC 
 
  400
 
  5
 
 29405
 
 island
 
 0.64751
 
 0.90824
 
 Not queried
 
  1.5969342
 
 1.59e-02
 
 1.00e+00
  
 
  
 11
 
  64405764
 
  64406051
 
  PDF   STATS   ENSEMBL   UCSC 
 
  288
 
  4
 
 6673
 
 gene;island
 
 0.46273
 
 0.14141
 
 Not queried
 
  1.5966138
 
 2.00e-01
 
 1.00e+00
  
 
  
 6
 
  32796516
 
  32796739
 
  PDF   STATS   ENSEMBL   UCSC 
 
  224
 
  4
 
 34072
 
 gene
 
 0.71908
 
 0.51268
 
 Not queried
 
  1.5957230
 
 5.71e-02
 
 1.00e+00
  
 
  
 18
 
  12777645
 
  12778029
 
  PDF   STATS   ENSEMBL   UCSC 
 
  385
 
  4
 
 19373
 
 island
 
 0.69899
 
 0.88318
 
 Not queried
 
  1.5952808
 
 2.00e-01
 
 1.00e+00
  
 
  
 X
 
 117250736
 
 117251123
 
  PDF   STATS   ENSEMBL   UCSC 
 
  388
 
  8
 
 41087
 
 gene;island
 
 0.03135
 
 0.05380
 
 Not queried
 
  1.5943562
 
 1.04e-02
 
 1.00e+00
  
 
  
 2
 
 133428422
 
 133428993
 
  PDF   STATS   ENSEMBL   UCSC 
 
  572
 
  7
 
 22842
 
 gene
 
 0.01157
 
 0.11009
 
 Not queried
 
  1.5943454
 
 5.30e-02
 
 1.00e+00
  
 
  
 2
 
 133428502
 
 133429299
 
  PDF   STATS   ENSEMBL   UCSC 
 
  798
 
  9
 
 23602
 
 tss
 
 0.01105
 
 0.06882
 
 Not queried
 
  1.5943454
 
 4.00e-02
 
 1.00e+00
  
 
  
 19
 
  39574483
 
  39574911
 
  PDF   STATS   ENSEMBL   UCSC 
 
  429
 
  9
 
 20729
 
 tss
 
 0.13295
 
 0.26877
 
 Not queried
 
  1.5940875
 
 7.82e-04
 
 1.00e+00
  
 
  
 2
 
 176977220
 
 176977458
 
  PDF   STATS   ENSEMBL   UCSC 
 
  239
 
  4
 
 24859
 
 island
 
 0.80604
 
 0.62117
 
 Not queried
 
  1.5940457
 
 5.71e-02
 
 1.00e+00
  
 
  
 21
 
  45713510
 
  45713719
 
  PDF   STATS   ENSEMBL   UCSC 
 
  210
 
  4
 
 26413
 
 gene;island
 
 0.98010
 
 0.90731
 
 Not queried
 
  1.5931426
 
 4.86e-01
 
 1.00e+00
  
 
  
 7
 
 155249398
 
 155249941
 
  PDF   STATS   ENSEMBL   UCSC 
 
  544
 
  6
 
 37946
 
 tss;island
 
 0.03737
 
 0.03816
 
 Not queried
 
  1.5926676
 
 4.85e-01
 
 1.00e+00
  
 
  
 11
 
  16632311
 
  16632724
 
  PDF   STATS   ENSEMBL   UCSC 
 
  414
 
  4
 
 8070
 
 island
 
 0.31638
 
 0.58541
 
 Not queried
 
  1.5925485
 
 1.14e-01
 
 1.00e+00
  
 
  
 6
 
  32920962
 
  32921233
 
  PDF   STATS   ENSEMBL   UCSC 
 
  272
 
  5
 
 34818
 
 tss
 
 0.32577
 
 0.03247
 
 Not queried
 
  1.5909833
 
 2.22e-01
 
 1.00e+00
  
 
  
 12
 
 132896199
 
 132896939
 
  PDF   STATS   ENSEMBL   UCSC 
 
  741
 
  9
 
 9422
 
 gene;island
 
 0.72182
 
 0.88657
 
 Not queried
 
  1.5908714
 
 4.00e-02
 
 1.00e+00
  
 
  
 X
 
  19533456
 
  19533577
 
  PDF   STATS   ENSEMBL   UCSC 
 
  122
 
  6
 
 41216
 
 tss
 
 0.05483
 
 0.01807
 
 Not queried
 
  1.5908035
 
 6.49e-02
 
 1.00e+00
  
 
  
 12
 
  70083090
 
  70083213
 
  PDF   STATS   ENSEMBL   UCSC 
 
  124
 
  5
 
 9187
 
 gene;tss
 
 0.08946
 
 0.19574
 
 Not queried
 
  1.5897975
 
 1.51e-01
 
 1.00e+00
  
 
  
 20
 
  61924240
 
  61924423
 
  PDF   STATS   ENSEMBL   UCSC 
 
  184
 
  4
 
 25856
 
 tss;island
 
 0.34032
 
 0.65608
 
 Not queried
 
  1.5897951
 
 1.14e-01
 
 1.00e+00
  
 
  
 2
 
  55746547
 
  55746709
 
  PDF   STATS   ENSEMBL   UCSC 
 
  163
 
  5
 
 23325
 
 tss
 
 0.01506
 
 0.02248
 
 Not queried
 
  1.5894491
 
 1.51e-01
 
 1.00e+00
  
 
  
 16
 
   3142911
 
   3143250
 
  PDF   STATS   ENSEMBL   UCSC 
 
  340
 
  6
 
 15020
 
 tss
 
 0.83977
 
 0.90788
 
 Not queried
 
  1.5889751
 
 1.32e-01
 
 1.00e+00
  
 
  
 1
 
 110672769
 
 110673319
 
  PDF   STATS   ENSEMBL   UCSC 
 
  551
 
  6
 
 3566
 
 island
 
 0.66727
 
 0.38855
 
 Not queried
 
  1.5886667
 
 1.52e-02
 
 1.00e+00
  
 
  
 19
 
  16013630
 
  16013895
 
  PDF   STATS   ENSEMBL   UCSC 
 
  266
 
  4
 
 21675
 
 island
 
 0.35259
 
 0.63158
 
 Not queried
 
  1.5886465
 
 1.14e-01
 
 1.00e+00
  
 
  
 11
 
   2828214
 
   2828778
 
  PDF   STATS   ENSEMBL   UCSC 
 
  565
 
  7
 
 6449
 
 gene;island
 
 0.47152
 
 0.76004
 
 Not queried
 
  1.5885718
 
 1.75e-02
 
 1.00e+00
  
 
  
 11
 
  65779526
 
  65779847
 
  PDF   STATS   ENSEMBL   UCSC 
 
  322
 
  4
 
 6703
 
 gene
 
 0.50387
 
 0.77265
 
 Not queried
 
  1.5874724
 
 5.71e-02
 
 1.00e+00
  
 
  
 6
 
  32800412
 
  32800591
 
  PDF   STATS   ENSEMBL   UCSC 
 
  180
 
 11
 
 34076
 
 gene
 
 0.85093
 
 0.56282
 
 Not queried
 
  1.5874103
 
 2.33e-02
 
 1.00e+00
  
 
  
 4
 
  42154840
 
  42154983
 
  PDF   STATS   ENSEMBL   UCSC 
 
  144
 
  7
 
 30961
 
 island
 
 0.06702
 
 0.01547
 
 Not queried
 
  1.5873542
 
 1.65e-01
 
 1.00e+00
  
 
  
 3
 
 127174705
 
 127175109
 
  PDF   STATS   ENSEMBL   UCSC 
 
  405
 
  5
 
 29400
 
 island
 
 0.19521
 
 0.05929
 
 Not queried
 
  1.5870389
 
 1.51e-01
 
 1.00e+00
  
 
  
 22
 
  17517062
 
  17517418
 
  PDF   STATS   ENSEMBL   UCSC 
 
  357
 
  5
 
 26898
 
 tss;island
 
 0.20278
 
 0.35079
 
 Not queried
 
  1.5866168
 
 9.52e-02
 
 1.00e+00
  
 
  
 3
 
 138679347
 
 138679578
 
  PDF   STATS   ENSEMBL   UCSC 
 
  232
 
  4
 
 29488
 
 island
 
 0.88068
 
 0.71289
 
 Not queried
 
  1.5862215
 
 2.86e-02
 
 1.00e+00
  
 
  
 19
 
   7505029
 
   7505344
 
  PDF   STATS   ENSEMBL   UCSC 
 
  316
 
  4
 
 19742
 
 gene;island
 
 0.74287
 
 0.89798
 
 Not queried
 
  1.5861568
 
 5.71e-02
 
 1.00e+00
  
 
  
 10
 
 112431719
 
 112431906
 
  PDF   STATS   ENSEMBL   UCSC 
 
  188
 
  4
 
 4740
 
 gene;island
 
 0.15714
 
 0.04551
 
 Not queried
 
  1.5856072
 
 3.43e-01
 
 1.00e+00
  
 
  
 1
 
  63782980
 
  63783167
 
  PDF   STATS   ENSEMBL   UCSC 
 
  188
 
  4
 
 3332
 
 island
 
 0.01430
 
 0.03063
 
 Not queried
 
  1.5845215
 
 3.43e-01
 
 1.00e+00
  
 
  
 7
 
 155603792
 
 155604091
 
  PDF   STATS   ENSEMBL   UCSC 
 
  300
 
  4
 
 37250
 
 gene;island
 
 0.06276
 
 0.13860
 
 Not queried
 
  1.5841243
 
 6.86e-01
 
 1.00e+00
  
 
  
 5
 
 172672684
 
 172673349
 
  PDF   STATS   ENSEMBL   UCSC 
 
  666
 
  8
 
 33346
 
 island
 
 0.66224
 
 0.88826
 
 Not queried
 
  1.5841092
 
 1.04e-02
 
 1.00e+00
  
 
  
 17
 
  79374181
 
  79374741
 
  PDF   STATS   ENSEMBL   UCSC 
 
  561
 
  6
 
 16873
 
 gene;island
 
 0.36493
 
 0.16516
 
 Not queried
 
  1.5840258
 
 4.85e-01
 
 1.00e+00
  
 
  
 X
 
 134156624
 
 134156809
 
  PDF   STATS   ENSEMBL   UCSC 
 
  186
 
  5
 
 41536
 
 tss
 
 0.03316
 
 0.02326
 
 Not queried
 
  1.5839826
 
 8.41e-01
 
 1.00e+00
  
 
  
 20
 
  31755609
 
  31755943
 
  PDF   STATS   ENSEMBL   UCSC 
 
  335
 
  4
 
 25640
 
 tss
 
 0.85270
 
 0.67267
 
 Not queried
 
  1.5838898
 
 3.43e-01
 
 1.00e+00
  
 
  
 6
 
  31138925
 
  31139359
 
  PDF   STATS   ENSEMBL   UCSC 
 
  435
 
  7
 
 34737
 
 tss
 
 0.34730
 
 0.61451
 
 Not queried
 
  1.5829854
 
 3.83e-01
 
 1.00e+00
  
 
  
 10
 
  47083283
 
  47083504
 
  PDF   STATS   ENSEMBL   UCSC 
 
  222
 
  8
 
 5046
 
 tss
 
 0.40917
 
 0.62622
 
 Not queried
 
  1.5829216
 
 2.95e-03
 
 1.00e+00
  
 
  
 1
 
   3143682
 
   3144209
 
  PDF   STATS   ENSEMBL   UCSC 
 
  528
 
  5
 
 108
 
 gene
 
 0.75468
 
 0.81645
 
 Not queried
 
  1.5828604
 
 9.52e-02
 
 1.00e+00
  
 
  
 10
 
 133938604
 
 133938970
 
  PDF   STATS   ENSEMBL   UCSC 
 
  367
 
  6
 
 4832
 
 gene;island
 
 0.74481
 
 0.89469
 
 Not queried
 
  1.5826385
 
 3.10e-01
 
 1.00e+00
  
 
  
 13
 
  41496623
 
  41496979
 
  PDF   STATS   ENSEMBL   UCSC 
 
  357
 
  4
 
 11379
 
 tss;island
 
 0.60057
 
 0.33541
 
 Not queried
 
  1.5820845
 
 2.86e-02
 
 1.00e+00
  
 
  
 20
 
    865780
 
    866087
 
  PDF   STATS   ENSEMBL   UCSC 
 
  308
 
  5
 
 25287
 
 gene;island
 
 0.04857
 
 0.16100
 
 Not queried
 
  1.5819869
 
 1.51e-01
 
 1.00e+00
  
 
  
 16
 
  67979437
 
  67979792
 
  PDF   STATS   ENSEMBL   UCSC 
 
  356
 
  4
 
 14779
 
 gene
 
 0.80670
 
 0.93528
 
 Not queried
 
  1.5818092
 
 1.14e-01
 
 1.00e+00
  
 
  
 9
 
 131486520
 
 131486918
 
  PDF   STATS   ENSEMBL   UCSC 
 
  399
 
  6
 
 40778
 
 tss
 
 0.01905
 
 0.03397
 
 Not queried
 
  1.5816690
 
 2.40e-01
 
 1.00e+00
  
 
  
 11
 
   2176877
 
   2177018
 
  PDF   STATS   ENSEMBL   UCSC 
 
  142
 
  4
 
 6433
 
 gene;island
 
 0.70843
 
 0.88251
 
 Not queried
 
  1.5810770
 
 2.00e-01
 
 1.00e+00
  
 
  
 7
 
 117513825
 
 117514017
 
  PDF   STATS   ENSEMBL   UCSC 
 
  193
 
  4
 
 37792
 
 tss;island
 
 0.06916
 
 0.03317
 
 Not queried
 
  1.5808661
 
 2.00e-01
 
 1.00e+00
  
 
  
 2
 
  71680559
 
  71680721
 
  PDF   STATS   ENSEMBL   UCSC 
 
  163
 
  4
 
 23392
 
 tss
 
 0.03526
 
 0.10666
 
 Not queried
 
  1.5806814
 
 2.86e-02
 
 1.00e+00
  
 
  
 17
 
   5972281
 
   5972448
 
  PDF   STATS   ENSEMBL   UCSC 
 
  168
 
  4
 
 17868
 
 island
 
 0.02594
 
 0.07394
 
 Not queried
 
  1.5804329
 
 2.00e-01
 
 1.00e+00
  
 
  
 18
 
  59560365
 
  59560495
 
  PDF   STATS   ENSEMBL   UCSC 
 
  131
 
  5
 
 19267
 
 tss
 
 0.00595
 
 0.03394
 
 Not queried
 
  1.5794111
 
 2.22e-01
 
 1.00e+00
  
 
  
 1
 
  59280290
 
  59280619
 
  PDF   STATS   ENSEMBL   UCSC 
 
  330
 
  4
 
 3311
 
 island
 
 0.66555
 
 0.85684
 
 Not queried
 
  1.5793900
 
 2.00e-01
 
 1.00e+00
  
 
  
 5
 
  92914036
 
  92914319
 
  PDF   STATS   ENSEMBL   UCSC 
 
  284
 
  5
 
 31632
 
 gene;island
 
 0.86831
 
 0.58279
 
 Not queried
 
  1.5789169
 
 1.51e-01
 
 1.00e+00
  
 
  
 11
 
   8710368
 
   8710910
 
  PDF   STATS   ENSEMBL   UCSC 
 
  543
 
  5
 
 6480
 
 gene;island
 
 0.03122
 
 0.18232
 
 Not queried
 
  1.5786867
 
 1.51e-01
 
 1.00e+00
  
 
  
 12
 
  49371987
 
  49372180
 
  PDF   STATS   ENSEMBL   UCSC 
 
  194
 
  7
 
 9654
 
 tss
 
 0.06550
 
 0.22484
 
 Not queried
 
  1.5785701
 
 1.28e-01
 
 1.00e+00
  
 
  
 12
 
  49371987
 
  49372474
 
  PDF   STATS   ENSEMBL   UCSC 
 
  488
 
  9
 
 10354
 
 island
 
 0.13167
 
 0.32669
 
 Not queried
 
  1.5785701
 
 1.13e-01
 
 1.00e+00
  
 
  
 17
 
  61613061
 
  61613356
 
  PDF   STATS   ENSEMBL   UCSC 
 
  296
 
  5
 
 16692
 
 gene;island
 
 0.91337
 
 0.77673
 
 Not queried
 
  1.5783952
 
 9.52e-02
 
 1.00e+00
  
 
  
 1
 
   1097027
 
   1097270
 
  PDF   STATS   ENSEMBL   UCSC 
 
  244
 
  4
 
 2400
 
 island
 
 0.56526
 
 0.75550
 
 Not queried
 
  1.5780740
 
 2.86e-02
 
 1.00e+00
  
 
  
 17
 
  26645200
 
  26645523
 
  PDF   STATS   ENSEMBL   UCSC 
 
  324
 
  4
 
 17174
 
 tss;island
 
 0.05885
 
 0.01906
 
 Not queried
 
  1.5780275
 
 2.86e-02
 
 1.00e+00
  
 
  
 11
 
  36422377
 
  36422615
 
  PDF   STATS   ENSEMBL   UCSC 
 
  239
 
  5
 
 6567
 
 gene
 
 0.72037
 
 0.90568
 
 Not queried
 
  1.5775702
 
 9.52e-02
 
 1.00e+00
  
 
  
 7
 
  35494345
 
  35494808
 
  PDF   STATS   ENSEMBL   UCSC 
 
  464
 
  4
 
 38345
 
 island
 
 0.54453
 
 0.78562
 
 Not queried
 
  1.5769777
 
 5.71e-02
 
 1.00e+00
  
 
  
 14
 
  88792913
 
  88793311
 
  PDF   STATS   ENSEMBL   UCSC 
 
  399
 
  8
 
 13028
 
 island
 
 0.07605
 
 0.22816
 
 Not queried
 
  1.5766951
 
 2.81e-02
 
 1.00e+00
  
 
  
 12
 
  16757954
 
  16758267
 
  PDF   STATS   ENSEMBL   UCSC 
 
  314
 
  6
 
 9046
 
 gene
 
 0.23483
 
 0.08319
 
 Not queried
 
  1.5762949
 
 6.49e-02
 
 1.00e+00
  
 
  
 4
 
    828098
 
    828281
 
  PDF   STATS   ENSEMBL   UCSC 
 
  184
 
  4
 
 30690
 
 island
 
 0.70821
 
 0.87845
 
 Not queried
 
  1.5756569
 
 5.71e-02
 
 1.00e+00
  
 
  
 21
 
  45705175
 
  45705742
 
  PDF   STATS   ENSEMBL   UCSC 
 
  568
 
 10
 
 26541
 
 tss;island
 
 0.77832
 
 0.92520
 
 Not queried
 
  1.5755063
 
 1.47e-02
 
 1.00e+00
  
 
  
 7
 
 158235891
 
 158236160
 
  PDF   STATS   ENSEMBL   UCSC 
 
  270
 
  6
 
 37307
 
 gene
 
 0.78021
 
 0.88926
 
 Not queried
 
  1.5754580
 
 3.10e-01
 
 1.00e+00
  
 
  
 11
 
  71724287
 
  71724677
 
  PDF   STATS   ENSEMBL   UCSC 
 
  391
 
  6
 
 6789
 
 gene
 
 0.64993
 
 0.87327
 
 Not queried
 
  1.5746500
 
 6.49e-02
 
 1.00e+00
  
 
  
 5
 
  14405632
 
  14406021
 
  PDF   STATS   ENSEMBL   UCSC 
 
  390
 
  5
 
 31507
 
 gene
 
 0.70299
 
 0.83618
 
 Not queried
 
  1.5746140
 
 3.10e-01
 
 1.00e+00
  
 
  
 22
 
  29875578
 
  29875991
 
  PDF   STATS   ENSEMBL   UCSC 
 
  414
 
  5
 
 27007
 
 tss;island
 
 0.03745
 
 0.24265
 
 Not queried
 
  1.5737763
 
 2.22e-01
 
 1.00e+00
  
 
  
 5
 
 140473857
 
 140474184
 
  PDF   STATS   ENSEMBL   UCSC 
 
  328
 
  6
 
 32302
 
 tss
 
 0.03252
 
 0.10263
 
 Not queried
 
  1.5724309
 
 3.10e-01
 
 1.00e+00
  
 
  
 6
 
 150378738
 
 150379047
 
  PDF   STATS   ENSEMBL   UCSC 
 
  310
 
  5
 
 36504
 
 island
 
 0.23992
 
 0.49910
 
 Not queried
 
  1.5722201
 
 5.56e-02
 
 1.00e+00
  
 
  
 13
 
 111281037
 
 111281479
 
  PDF   STATS   ENSEMBL   UCSC 
 
  443
 
  6
 
 11205
 
 gene;island
 
 0.69596
 
 0.93073
 
 Not queried
 
  1.5717263
 
 2.60e-02
 
 1.00e+00
  
 
  
 17
 
   7452289
 
   7452644
 
  PDF   STATS   ENSEMBL   UCSC 
 
  356
 
  5
 
 17922
 
 island
 
 0.34027
 
 0.19631
 
 Not queried
 
  1.5715398
 
 2.22e-01
 
 1.00e+00
  
 
  
 3
 
  27765232
 
  27765546
 
  PDF   STATS   ENSEMBL   UCSC 
 
  315
 
  5
 
 28909
 
 island
 
 0.26825
 
 0.21156
 
 Not queried
 
  1.5712091
 
 6.90e-01
 
 1.00e+00
  
 
  
 17
 
  46698881
 
  46699155
 
  PDF   STATS   ENSEMBL   UCSC 
 
  275
 
  4
 
 16626
 
 gene;island
 
 0.10499
 
 0.37369
 
 Not queried
 
  1.5706076
 
 5.71e-02
 
 1.00e+00
  
 
  
 7
 
 150065170
 
 150065255
 
  PDF   STATS   ENSEMBL   UCSC 
 
   86
 
  6
 
 37913
 
 tss;island
 
 0.29587
 
 0.10565
 
 Not queried
 
  1.5704748
 
 6.49e-02
 
 1.00e+00
  
 
  
 10
 
 131697669
 
 131697980
 
  PDF   STATS   ENSEMBL   UCSC 
 
  312
 
  4
 
 4821
 
 gene;island
 
 0.88693
 
 0.73047
 
 Not queried
 
  1.5700934
 
 1.14e-01
 
 1.00e+00
  
 
  
 6
 
  30853948
 
  30854233
 
  PDF   STATS   ENSEMBL   UCSC 
 
  286
 
  8
 
 33764
 
 gene;island
 
 0.19983
 
 0.08726
 
 Not queried
 
  1.5697100
 
 1.30e-01
 
 1.00e+00
  
 
  
 2
 
 220196530
 
 220196755
 
  PDF   STATS   ENSEMBL   UCSC 
 
  226
 
  4
 
 22996
 
 gene;island
 
 0.16790
 
 0.44006
 
 Not queried
 
  1.5695645
 
 4.86e-01
 
 1.00e+00
  
 
  
 22
 
  50451054
 
  50451414
 
  PDF   STATS   ENSEMBL   UCSC 
 
  361
 
  5
 
 27568
 
 island
 
 0.40866
 
 0.66550
 
 Not queried
 
  1.5682895
 
 5.56e-02
 
 1.00e+00
  
 
  
 3
 
  13520685
 
  13521744
 
  PDF   STATS   ENSEMBL   UCSC 
 
 1060
 
 25
 
 28122
 
 tss
 
 0.08715
 
 0.03231
 
 Not queried
 
  1.5677998
 
 1.61e-02
 
 1.00e+00
  
 
  
 4
 
  46911291
 
  46911550
 
  PDF   STATS   ENSEMBL   UCSC 
 
  260
 
  5
 
 30327
 
 tss
 
 0.64410
 
 0.84252
 
 Not queried
 
  1.5676911
 
 2.22e-01
 
 1.00e+00
  
 
  
 6
 
  86302808
 
  86302950
 
  PDF   STATS   ENSEMBL   UCSC 
 
  143
 
  4
 
 34340
 
 gene;island
 
 0.01083
 
 0.02983
 
 Not queried
 
  1.5673360
 
 3.43e-01
 
 1.00e+00
  
 
  
 10
 
  71336773
 
  71337104
 
  PDF   STATS   ENSEMBL   UCSC 
 
  332
 
  4
 
 5788
 
 island
 
 0.22090
 
 0.06720
 
 Not queried
 
  1.5671953
 
 4.86e-01
 
 1.00e+00
  
 
  
 11
 
   1474386
 
   1474841
 
  PDF   STATS   ENSEMBL   UCSC 
 
  456
 
  5
 
 6403
 
 gene;island
 
 0.45015
 
 0.76784
 
 Not queried
 
  1.5662754
 
 5.56e-02
 
 1.00e+00
  
 
  
 3
 
  62861071
 
  62861311
 
  PDF   STATS   ENSEMBL   UCSC 
 
  241
 
  6
 
 28385
 
 tss
 
 0.02317
 
 0.09306
 
 Not queried
 
  1.5655864
 
 2.40e-01
 
 1.00e+00
  
 
  
 17
 
  37074902
 
  37075068
 
  PDF   STATS   ENSEMBL   UCSC 
 
  167
 
  4
 
 16510
 
 gene;island
 
 0.67588
 
 0.84191
 
 Not queried
 
  1.5651353
 
 3.43e-01
 
 1.00e+00
  
 
  
 16
 
   1480790
 
   1480992
 
  PDF   STATS   ENSEMBL   UCSC 
 
  203
 
  4
 
 15511
 
 island
 
 0.59308
 
 0.82769
 
 Not queried
 
  1.5648945
 
 3.43e-01
 
 1.00e+00
  
 
  
 17
 
  80185943
 
  80186336
 
  PDF   STATS   ENSEMBL   UCSC 
 
  394
 
  6
 
 17736
 
 tss;island
 
 0.78103
 
 0.50696
 
 Not queried
 
  1.5644330
 
 4.11e-02
 
 1.00e+00
  
 
  
 10
 
 126331180
 
 126331425
 
  PDF   STATS   ENSEMBL   UCSC 
 
  246
 
  4
 
 4793
 
 gene
 
 0.84934
 
 0.93302
 
 Not queried
 
  1.5641765
 
 2.00e-01
 
 1.00e+00
  
 
  
 19
 
   2474993
 
   2475189
 
  PDF   STATS   ENSEMBL   UCSC 
 
  197
 
  4
 
 20291
 
 tss;island
 
 0.04672
 
 0.09118
 
 Not queried
 
  1.5633789
 
 5.71e-02
 
 1.00e+00
  
 
  
 18
 
  55019621
 
  55019711
 
  PDF   STATS   ENSEMBL   UCSC 
 
   91
 
  4
 
 19256
 
 tss
 
 0.15434
 
 0.07835
 
 Not queried
 
  1.5629414
 
 1.14e-01
 
 1.00e+00
  
 
  
 1
 
  27216732
 
  27216796
 
  PDF   STATS   ENSEMBL   UCSC 
 
   65
 
  6
 
 345
 
 gene
 
 0.02023
 
 0.06030
 
 Not queried
 
  1.5628674
 
 1.32e-01
 
 1.00e+00
  
 
  
 11
 
   1154976
 
   1155278
 
  PDF   STATS   ENSEMBL   UCSC 
 
  303
 
  4
 
 7883
 
 island
 
 0.78034
 
 0.90025
 
 Not queried
 
  1.5626721
 
 2.00e-01
 
 1.00e+00
  
 
  
 16
 
   2762569
 
   2762819
 
  PDF   STATS   ENSEMBL   UCSC 
 
  251
 
  4
 
 14580
 
 gene;island
 
 0.63877
 
 0.74082
 
 Not queried
 
  1.5626556
 
 3.43e-01
 
 1.00e+00
  
 
  
 3
 
 156544070
 
 156544233
 
  PDF   STATS   ENSEMBL   UCSC 
 
  164
 
  5
 
 29567
 
 island
 
 0.23353
 
 0.54596
 
 Not queried
 
  1.5624395
 
 7.94e-03
 
 1.00e+00
  
 
  
 12
 
 109162390
 
 109162748
 
  PDF   STATS   ENSEMBL   UCSC 
 
  359
 
  6
 
 10745
 
 island
 
 0.42542
 
 0.18343
 
 Not queried
 
  1.5613691
 
 4.33e-03
 
 1.00e+00
  
 
  
 2
 
  74725040
 
  74725369
 
  PDF   STATS   ENSEMBL   UCSC 
 
  330
 
  4
 
 22706
 
 gene;island
 
 0.12223
 
 0.10328
 
 Not queried
 
  1.5610265
 
 6.86e-01
 
 1.00e+00
  
 
  
 X
 
  43741814
 
  43741945
 
  PDF   STATS   ENSEMBL   UCSC 
 
  132
 
  5
 
 41267
 
 tss;island
 
 0.05694
 
 0.02410
 
 Not queried
 
  1.5601942
 
 5.48e-01
 
 1.00e+00
  
 
  
 18
 
  77230484
 
  77230795
 
  PDF   STATS   ENSEMBL   UCSC 
 
  312
 
  4
 
 19107
 
 gene;island
 
 0.74049
 
 0.89526
 
 Not queried
 
  1.5597842
 
 2.86e-02
 
 1.00e+00
  
 
  
 19
 
  18111623
 
  18111880
 
  PDF   STATS   ENSEMBL   UCSC 
 
  258
 
  5
 
 20571
 
 tss
 
 0.10798
 
 0.04671
 
 Not queried
 
  1.5592222
 
 2.22e-01
 
 1.00e+00
  
 
  
 7
 
 157352978
 
 157353191
 
  PDF   STATS   ENSEMBL   UCSC 
 
  214
 
  5
 
 37263
 
 gene;island
 
 0.88637
 
 0.94305
 
 Not queried
 
  1.5590299
 
 3.10e-01
 
 1.00e+00
  
 
  
 19
 
  13885098
 
  13885429
 
  PDF   STATS   ENSEMBL   UCSC 
 
  332
 
  5
 
 21619
 
 island
 
 0.01990
 
 0.03837
 
 Not queried
 
  1.5582454
 
 4.21e-01
 
 1.00e+00
  
 
  
 15
 
  65068326
 
  65068594
 
  PDF   STATS   ENSEMBL   UCSC 
 
  269
 
  4
 
 13704
 
 tss;island
 
 0.04174
 
 0.02773
 
 Not queried
 
  1.5570241
 
 4.86e-01
 
 1.00e+00
  
 
  
 1
 
   1148973
 
   1149211
 
  PDF   STATS   ENSEMBL   UCSC 
 
  239
 
  4
 
 21
 
 gene;island
 
 0.50013
 
 0.72952
 
 Not queried
 
  1.5563326
 
 2.86e-02
 
 1.00e+00
  
 
  
 17
 
  20946875
 
  20947103
 
  PDF   STATS   ENSEMBL   UCSC 
 
  229
 
  4
 
 17166
 
 tss;island
 
 0.00455
 
 0.01224
 
 Not queried
 
  1.5561535
 
 2.00e-01
 
 1.00e+00
  
 
  
 6
 
  43141823
 
  43142093
 
  PDF   STATS   ENSEMBL   UCSC 
 
  271
 
  4
 
 34280
 
 gene;island
 
 0.39233
 
 0.48923
 
 Not queried
 
  1.5559449
 
 8.86e-01
 
 1.00e+00
  
 
  
 12
 
  51818636
 
  51818768
 
  PDF   STATS   ENSEMBL   UCSC 
 
  133
 
  4
 
 9107
 
 gene
 
 0.03651
 
 0.02251
 
 Not queried
 
  1.5558245
 
 4.86e-01
 
 1.00e+00
  
 
  
 X
 
  37706736
 
  37707161
 
  PDF   STATS   ENSEMBL   UCSC 
 
  426
 
  7
 
 41716
 
 island
 
 0.13258
 
 0.02737
 
 Not queried
 
  1.5553203
 
 1.28e-01
 
 1.00e+00
  
 
  
 5
 
 131346985
 
 131347278
 
  PDF   STATS   ENSEMBL   UCSC 
 
  294
 
  4
 
 31687
 
 gene
 
 0.10407
 
 0.06955
 
 Not queried
 
  1.5547575
 
 8.86e-01
 
 1.00e+00
  
 
  
 7
 
  45613676
 
  45613825
 
  PDF   STATS   ENSEMBL   UCSC 
 
  150
 
  4
 
 37519
 
 tss;island
 
 0.16556
 
 0.04804
 
 Not queried
 
  1.5543740
 
 8.86e-01
 
 1.00e+00
  
 
  
 3
 
  75707805
 
  75708197
 
  PDF   STATS   ENSEMBL   UCSC 
 
  393
 
  4
 
 29246
 
 island
 
 0.45101
 
 0.25516
 
 Not queried
 
  1.5543196
 
 3.43e-01
 
 1.00e+00
  
 
  
 13
 
  88328251
 
  88328471
 
  PDF   STATS   ENSEMBL   UCSC 
 
  221
 
  4
 
 11159
 
 gene;island
 
 0.33452
 
 0.60247
 
 Not queried
 
  1.5540180
 
 2.00e-01
 
 1.00e+00
  
 
  
 10
 
    180188
 
    180411
 
  PDF   STATS   ENSEMBL   UCSC 
 
  224
 
  7
 
 4893
 
 tss
 
 0.01593
 
 0.05383
 
 Not queried
 
  1.5538979
 
 1.65e-01
 
 1.00e+00
  
 
  
 13
 
 103532159
 
 103532435
 
  PDF   STATS   ENSEMBL   UCSC 
 
  277
 
  7
 
 11499
 
 tss
 
 0.54290
 
 0.76720
 
 Not queried
 
  1.5538454
 
 9.73e-02
 
 1.00e+00
  
 
  
 12
 
  50475035
 
  50475372
 
  PDF   STATS   ENSEMBL   UCSC 
 
  338
 
  4
 
 9102
 
 gene;island
 
 0.90620
 
 0.66279
 
 Not queried
 
  1.5535051
 
 1.14e-01
 
 1.00e+00
  
 
  
 7
 
   4848683
 
   4848939
 
  PDF   STATS   ENSEMBL   UCSC 
 
  257
 
  4
 
 36794
 
 gene;island
 
 0.87641
 
 0.93361
 
 Not queried
 
  1.5529434
 
 3.43e-01
 
 1.00e+00
  
 
  
 18
 
   5891948
 
   5892245
 
  PDF   STATS   ENSEMBL   UCSC 
 
  298
 
  4
 
 19342
 
 island
 
 0.67693
 
 0.41619
 
 Not queried
 
  1.5520421
 
 2.86e-02
 
 1.00e+00
  
 
  
 7
 
  80548695
 
  80548752
 
  PDF   STATS   ENSEMBL   UCSC 
 
   58
 
  5
 
 37610
 
 tss;island
 
 0.01554
 
 0.04424
 
 Not queried
 
  1.5518657
 
 4.21e-01
 
 1.00e+00
  
 
  
 5
 
 176937350
 
 176937527
 
  PDF   STATS   ENSEMBL   UCSC 
 
  178
 
  4
 
 32471
 
 tss
 
 0.84449
 
 0.92242
 
 Not queried
 
  1.5516558
 
 1.14e-01
 
 1.00e+00
  
 
  
 6
 
  32126315
 
  32126616
 
  PDF   STATS   ENSEMBL   UCSC 
 
  302
 
  4
 
 34000
 
 gene
 
 0.83323
 
 0.92359
 
 Not queried
 
  1.5515719
 
 2.00e-01
 
 1.00e+00
  
 
  
 6
 
 168197625
 
 168198163
 
  PDF   STATS   ENSEMBL   UCSC 
 
  539
 
  7
 
 35279
 
 tss
 
 0.53069
 
 0.76149
 
 Not queried
 
  1.5515582
 
 1.11e-02
 
 1.00e+00
  
 
  
 12
 
 132939657
 
 132939992
 
  PDF   STATS   ENSEMBL   UCSC 
 
  336
 
  4
 
 11019
 
 island
 
 0.86024
 
 0.94651
 
 Not queried
 
  1.5509405
 
 1.14e-01
 
 1.00e+00
  
 
  
 1
 
 181451906
 
 181452137
 
  PDF   STATS   ENSEMBL   UCSC 
 
  232
 
  6
 
 2125
 
 tss;island
 
 0.04078
 
 0.09543
 
 Not queried
 
  1.5503878
 
 4.33e-03
 
 1.00e+00
  
 
  
 20
 
   1294311
 
   1294376
 
  PDF   STATS   ENSEMBL   UCSC 
 
   66
 
  4
 
 25498
 
 tss
 
 0.17519
 
 0.04332
 
 Not queried
 
  1.5503400
 
 4.86e-01
 
 1.00e+00
  
 
  
 1
 
  24194974
 
  24195659
 
  PDF   STATS   ENSEMBL   UCSC 
 
  686
 
 12
 
 1347
 
 tss;island
 
 0.06355
 
 0.18173
 
 Not queried
 
  1.5498339
 
 4.49e-02
 
 1.00e+00
  
 
  
 6
 
  30540557
 
  30540762
 
  PDF   STATS   ENSEMBL   UCSC 
 
  206
 
  4
 
 33715
 
 gene;island
 
 0.74621
 
 0.89084
 
 Not queried
 
  1.5490763
 
 3.43e-01
 
 1.00e+00
  
 
  
 8
 
  33369905
 
  33370172
 
  PDF   STATS   ENSEMBL   UCSC 
 
  268
 
  5
 
 39213
 
 gene;island
 
 0.02012
 
 0.01177
 
 Not queried
 
  1.5489279
 
 1.51e-01
 
 1.00e+00
  
 
  
 22
 
  46402185
 
  46402486
 
  PDF   STATS   ENSEMBL   UCSC 
 
  302
 
  4
 
 27183
 
 tss
 
 0.07568
 
 0.32368
 
 Not queried
 
  1.5484554
 
 4.86e-01
 
 1.00e+00
  
 
  
 2
 
 130955979
 
 130956271
 
  PDF   STATS   ENSEMBL   UCSC 
 
  293
 
  8
 
 24678
 
 island
 
 0.96948
 
 0.87074
 
 Not queried
 
  1.5481599
 
 1.30e-01
 
 1.00e+00
  
 
  
 1
 
   6240267
 
   6240508
 
  PDF   STATS   ENSEMBL   UCSC 
 
  242
 
  5
 
 1194
 
 tss;island
 
 0.02794
 
 0.09876
 
 Not queried
 
  1.5478154
 
 3.17e-02
 
 1.00e+00
  
 
  
 21
 
  45773189
 
  45773400
 
  PDF   STATS   ENSEMBL   UCSC 
 
  212
 
  4
 
 26543
 
 tss
 
 0.58115
 
 0.81662
 
 Not queried
 
  1.5470777
 
 6.86e-01
 
 1.00e+00
  
 
  
 1
 
   1309161
 
   1309641
 
  PDF   STATS   ENSEMBL   UCSC 
 
  481
 
  4
 
 33
 
 gene;island
 
 0.25564
 
 0.51340
 
 Not queried
 
  1.5470349
 
 2.00e-01
 
 1.00e+00
  
 
  
 15
 
  45926780
 
  45927218
 
  PDF   STATS   ENSEMBL   UCSC 
 
  439
 
  8
 
 13625
 
 tss
 
 0.07224
 
 0.04596
 
 Not queried
 
  1.5469104
 
 6.50e-02
 
 1.00e+00
  
 
  
 22
 
  50451108
 
  50451414
 
  PDF   STATS   ENSEMBL   UCSC 
 
  307
 
  4
 
 27200
 
 tss
 
 0.35610
 
 0.61464
 
 Not queried
 
  1.5469062
 
 2.00e-01
 
 1.00e+00
  
 
  
 3
 
  47620963
 
  47621127
 
  PDF   STATS   ENSEMBL   UCSC 
 
  165
 
  4
 
 28247
 
 tss;island
 
 0.55979
 
 0.37835
 
 Not queried
 
  1.5464441
 
 2.86e-02
 
 1.00e+00
  
 
  
 17
 
   1617102
 
   1617465
 
  PDF   STATS   ENSEMBL   UCSC 
 
  364
 
  6
 
 16282
 
 gene;island
 
 0.06332
 
 0.18089
 
 Not queried
 
  1.5459671
 
 1.80e-01
 
 1.00e+00
  
 
  
 1
 
  25868969
 
  25869196
 
  PDF   STATS   ENSEMBL   UCSC 
 
  228
 
  4
 
 1366
 
 tss;island
 
 0.82786
 
 0.62248
 
 Not queried
 
  1.5459418
 
 4.86e-01
 
 1.00e+00
  
 
  
 20
 
   1165206
 
   1165435
 
  PDF   STATS   ENSEMBL   UCSC 
 
  230
 
  4
 
 25495
 
 tss
 
 0.78586
 
 0.46306
 
 Not queried
 
  1.5458302
 
 2.00e-01
 
 1.00e+00
  
 
  
 17
 
    800084
 
    800512
 
  PDF   STATS   ENSEMBL   UCSC 
 
  429
 
  5
 
 16264
 
 gene
 
 0.89400
 
 0.81934
 
 Not queried
 
  1.5456196
 
 9.52e-02
 
 1.00e+00
  
 
  
 11
 
   1331124
 
   1331497
 
  PDF   STATS   ENSEMBL   UCSC 
 
  374
 
  4
 
 6399
 
 gene;tss;island
 
 0.20994
 
 0.07403
 
 Not queried
 
  1.5454166
 
 2.00e-01
 
 1.00e+00
  
 
  
 5
 
  37838290
 
  37838871
 
  PDF   STATS   ENSEMBL   UCSC 
 
  582
 
  6
 
 31536
 
 gene;island
 
 0.05326
 
 0.12229
 
 Not queried
 
  1.5450791
 
 3.94e-01
 
 1.00e+00
  
 
  
 7
 
 149416674
 
 149416973
 
  PDF   STATS   ENSEMBL   UCSC 
 
  300
 
  5
 
 37203
 
 gene;island
 
 0.78043
 
 0.94994
 
 Not queried
 
  1.5450606
 
 1.51e-01
 
 1.00e+00
  
 
  
 19
 
  50303041
 
  50303389
 
  PDF   STATS   ENSEMBL   UCSC 
 
  349
 
  5
 
 20127
 
 gene;island
 
 0.82492
 
 0.92556
 
 Not queried
 
  1.5447990
 
 3.10e-01
 
 1.00e+00
  
 
  
 6
 
  29723301
 
  29723407
 
  PDF   STATS   ENSEMBL   UCSC 
 
  107
 
  4
 
 35566
 
 island
 
 0.68126
 
 0.47632
 
 Not queried
 
  1.5447165
 
 2.00e-01
 
 1.00e+00
  
 
  
 18
 
  44557635
 
  44557819
 
  PDF   STATS   ENSEMBL   UCSC 
 
  185
 
  5
 
 19072
 
 gene;tss;island
 
 0.64057
 
 0.83868
 
 Not queried
 
  1.5445187
 
 2.22e-01
 
 1.00e+00
  
 
  
 11
 
  46383031
 
  46383209
 
  PDF   STATS   ENSEMBL   UCSC 
 
  179
 
  4
 
 6581
 
 gene
 
 0.48513
 
 0.73982
 
 Not queried
 
  1.5440493
 
 2.86e-02
 
 1.00e+00
  
 
  
 10
 
 131265059
 
 131265435
 
  PDF   STATS   ENSEMBL   UCSC 
 
  377
 
 11
 
 5422
 
 tss
 
 0.04378
 
 0.09774
 
 Not queried
 
  1.5432423
 
 2.81e-02
 
 1.00e+00
  
 
  
 X
 
 103810908
 
 103811025
 
  PDF   STATS   ENSEMBL   UCSC 
 
  118
 
  5
 
 41937
 
 island
 
 0.01011
 
 0.02840
 
 Not queried
 
  1.5426020
 
 1.51e-01
 
 1.00e+00
  
 
  
 7
 
   2078650
 
   2079008
 
  PDF   STATS   ENSEMBL   UCSC 
 
  359
 
  5
 
 36725
 
 gene
 
 0.80407
 
 0.91467
 
 Not queried
 
  1.5424489
 
 5.56e-02
 
 1.00e+00
  
 
  
 8
 
  18666531
 
  18666598
 
  PDF   STATS   ENSEMBL   UCSC 
 
   68
 
  4
 
 39157
 
 gene;tss
 
 0.76047
 
 0.90040
 
 Not queried
 
  1.5421987
 
 2.86e-02
 
 1.00e+00
  
 
  
 3
 
  48342880
 
  48343235
 
  PDF   STATS   ENSEMBL   UCSC 
 
  356
 
  7
 
 28253
 
 tss
 
 0.04477
 
 0.02130
 
 Not queried
 
  1.5411710
 
 5.30e-02
 
 1.00e+00
  
 
  
 13
 
 113759771
 
 113760286
 
  PDF   STATS   ENSEMBL   UCSC 
 
  516
 
  6
 
 11927
 
 island
 
 0.68886
 
 0.87142
 
 Not queried
 
  1.5407508
 
 1.32e-01
 
 1.00e+00
  
 
  
 5
 
   7869257
 
   7869428
 
  PDF   STATS   ENSEMBL   UCSC 
 
  172
 
  5
 
 31502
 
 gene
 
 0.00408
 
 0.01178
 
 Not queried
 
  1.5402659
 
 3.10e-01
 
 1.00e+00
  
 
  
 19
 
  40732518
 
  40732902
 
  PDF   STATS   ENSEMBL   UCSC 
 
  385
 
  9
 
 21983
 
 island
 
 0.47302
 
 0.75926
 
 Not queried
 
  1.5399239
 
 1.85e-03
 
 1.00e+00
  
 
  
 5
 
 158689930
 
 158690029
 
  PDF   STATS   ENSEMBL   UCSC 
 
  100
 
  4
 
 32403
 
 tss
 
 0.01097
 
 0.02698
 
 Not queried
 
  1.5397804
 
 2.00e-01
 
 1.00e+00
  
 
  
 16
 
  84401756
 
  84402160
 
  PDF   STATS   ENSEMBL   UCSC 
 
  405
 
  6
 
 16108
 
 island
 
 0.03015
 
 0.09268
 
 Not queried
 
  1.5396963
 
 3.10e-01
 
 1.00e+00
  
 
  
 12
 
  75905429
 
  75905681
 
  PDF   STATS   ENSEMBL   UCSC 
 
  253
 
  7
 
 9857
 
 tss
 
 0.01607
 
 0.03051
 
 Not queried
 
  1.5394783
 
 9.73e-02
 
 1.00e+00
  
 
  
 3
 
 147124363
 
 147124827
 
  PDF   STATS   ENSEMBL   UCSC 
 
  465
 
  6
 
 28608
 
 tss;island
 
 0.53100
 
 0.39020
 
 Not queried
 
  1.5391371
 
 5.89e-01
 
 1.00e+00
  
 
  
 19
 
  11531294
 
  11531469
 
  PDF   STATS   ENSEMBL   UCSC 
 
  176
 
  4
 
 20436
 
 tss
 
 0.02071
 
 0.04402
 
 Not queried
 
  1.5386918
 
 3.43e-01
 
 1.00e+00
  
 
  
 6
 
  32732585
 
  32732806
 
  PDF   STATS   ENSEMBL   UCSC 
 
  222
 
  4
 
 34809
 
 tss;island
 
 0.73960
 
 0.49877
 
 Not queried
 
  1.5384532
 
 6.86e-01
 
 1.00e+00
  
 
  
 12
 
 113574066
 
 113574368
 
  PDF   STATS   ENSEMBL   UCSC 
 
  303
 
  6
 
 10000
 
 tss
 
 0.43058
 
 0.20455
 
 Not queried
 
  1.5382791
 
 1.32e-01
 
 1.00e+00
  
 
  
 1
 
 228558987
 
 228559321
 
  PDF   STATS   ENSEMBL   UCSC 
 
  335
 
  5
 
 1054
 
 gene;island
 
 0.60893
 
 0.82862
 
 Not queried
 
  1.5382185
 
 7.94e-03
 
 1.00e+00
  
 
  
 11
 
  61355119
 
  61355505
 
  PDF   STATS   ENSEMBL   UCSC 
 
  387
 
  4
 
 8307
 
 island
 
 0.02543
 
 0.07128
 
 Not queried
 
  1.5378996
 
 1.14e-01
 
 1.00e+00
  
 
  
 6
 
  42110740
 
  42111005
 
  PDF   STATS   ENSEMBL   UCSC 
 
  266
 
  5
 
 34914
 
 tss;island
 
 0.53627
 
 0.63831
 
 Not queried
 
  1.5373175
 
 4.21e-01
 
 1.00e+00
  
 
  
 19
 
  16528764
 
  16529096
 
  PDF   STATS   ENSEMBL   UCSC 
 
  333
 
  4
 
 19875
 
 gene;island
 
 0.34024
 
 0.77644
 
 Not queried
 
  1.5366777
 
 1.14e-01
 
 1.00e+00
  
 
  
 17
 
  77216389
 
  77216699
 
  PDF   STATS   ENSEMBL   UCSC 
 
  311
 
  4
 
 16803
 
 gene
 
 0.53032
 
 0.76605
 
 Not queried
 
  1.5366105
 
 5.71e-02
 
 1.00e+00
  
 
  
 22
 
  42949526
 
  42949829
 
  PDF   STATS   ENSEMBL   UCSC 
 
  304
 
  8
 
 27151
 
 tss
 
 0.04861
 
 0.02314
 
 Not queried
 
  1.5358781
 
 1.95e-01
 
 1.00e+00
  
 
  
 3
 
  52008487
 
  52009002
 
  PDF   STATS   ENSEMBL   UCSC 
 
  516
 
 11
 
 28327
 
 tss;island
 
 0.11509
 
 0.25149
 
 Not queried
 
  1.5348898
 
 1.16e-01
 
 1.00e+00
  
 
  
 5
 
 138713750
 
 138714339
 
  PDF   STATS   ENSEMBL   UCSC 
 
  590
 
  7
 
 31718
 
 gene;island
 
 0.56256
 
 0.78992
 
 Not queried
 
  1.5343837
 
 5.83e-04
 
 1.00e+00
  
 
  
 17
 
  41277694
 
  41279022
 
  PDF   STATS   ENSEMBL   UCSC 
 
 1329
 
 28
 
 16560
 
 gene
 
 0.62368
 
 0.38919
 
 Not queried
 
  1.5324919
 
 8.22e-03
 
 1.00e+00
  
 
  
 7
 
 100612725
 
 100612937
 
  PDF   STATS   ENSEMBL   UCSC 
 
  213
 
  5
 
 38722
 
 island
 
 0.34236
 
 0.62597
 
 Not queried
 
  1.5317971
 
 5.56e-02
 
 1.00e+00
  
 
  
 13
 
 111522755
 
 111522985
 
  PDF   STATS   ENSEMBL   UCSC 
 
  231
 
  5
 
 11512
 
 tss
 
 0.67726
 
 0.42200
 
 Not queried
 
  1.5316815
 
 7.94e-03
 
 1.00e+00
  
 
  
 12
 
  11151430
 
  11151622
 
  PDF   STATS   ENSEMBL   UCSC 
 
  193
 
  4
 
 9029
 
 gene;tss
 
 0.75115
 
 0.89248
 
 Not queried
 
  1.5308825
 
 2.86e-02
 
 1.00e+00
  
 
  
 6
 
  33244976
 
  33246488
 
  PDF   STATS   ENSEMBL   UCSC 
 
 1513
 
 45
 
 34181
 
 gene
 
 0.68953
 
 0.45706
 
 Not queried
 
  1.5305513
 
 5.13e-03
 
 1.00e+00
  
 
  
 X
 
  12601534
 
  12601783
 
  PDF   STATS   ENSEMBL   UCSC 
 
  250
 
  4
 
 40938
 
 gene;island
 
 0.84250
 
 0.95389
 
 Not queried
 
  1.5301438
 
 1.14e-01
 
 1.00e+00
  
 
  
 8
 
  99076526
 
  99076837
 
  PDF   STATS   ENSEMBL   UCSC 
 
  312
 
  6
 
 40418
 
 island
 
 0.15597
 
 0.08004
 
 Not queried
 
  1.5300618
 
 2.40e-01
 
 1.00e+00
  
 
  
 11
 
    405846
 
    406019
 
  PDF   STATS   ENSEMBL   UCSC 
 
  174
 
  4
 
 6352
 
 gene;island
 
 0.80728
 
 0.67248
 
 Not queried
 
  1.5295585
 
 1.14e-01
 
 1.00e+00
  
 
  
 7
 
   1407330
 
   1407571
 
  PDF   STATS   ENSEMBL   UCSC 
 
  242
 
  6
 
 38036
 
 island
 
 0.61742
 
 0.84817
 
 Not queried
 
  1.5294663
 
 1.52e-02
 
 1.00e+00
  
 
  
 6
 
   1523751
 
   1524190
 
  PDF   STATS   ENSEMBL   UCSC 
 
  440
 
  5
 
 35305
 
 island
 
 0.85632
 
 0.55137
 
 Not queried
 
  1.5293897
 
 1.51e-01
 
 1.00e+00
  
 
  
 6
 
  33282313
 
  33283328
 
  PDF   STATS   ENSEMBL   UCSC 
 
 1016
 
 34
 
 34199
 
 gene;tss;island
 
 0.14363
 
 0.46921
 
 Not queried
 
  1.5293447
 
 6.80e-03
 
 1.00e+00
  
 
  
 7
 
  73275265
 
  73275436
 
  PDF   STATS   ENSEMBL   UCSC 
 
  172
 
  6
 
 37579
 
 tss
 
 0.83687
 
 0.64973
 
 Not queried
 
  1.5291538
 
 9.31e-02
 
 1.00e+00
  
 
  
 18
 
  77748485
 
  77748761
 
  PDF   STATS   ENSEMBL   UCSC 
 
  277
 
  5
 
 19580
 
 island
 
 0.01578
 
 0.03615
 
 Not queried
 
  1.5290643
 
 9.52e-02
 
 1.00e+00
  
 
  
 13
 
 114927579
 
 114928233
 
  PDF   STATS   ENSEMBL   UCSC 
 
  655
 
  8
 
 11970
 
 island
 
 0.40977
 
 0.64333
 
 Not queried
 
  1.5289640
 
 2.07e-02
 
 1.00e+00
  
 
  
 X
 
  46696351
 
  46696474
 
  PDF   STATS   ENSEMBL   UCSC 
 
  124
 
  4
 
 40972
 
 gene
 
 0.03267
 
 0.02389
 
 Not queried
 
  1.5289216
 
 4.86e-01
 
 1.00e+00
  
 
  
 3
 
  49756301
 
  49757476
 
  PDF   STATS   ENSEMBL   UCSC 
 
 1176
 
 12
 
 27746
 
 gene;island
 
 0.17725
 
 0.36417
 
 Not queried
 
  1.5284970
 
 1.78e-01
 
 1.00e+00
  
 
  
 19
 
  45316493
 
  45316789
 
  PDF   STATS   ENSEMBL   UCSC 
 
  297
 
  4
 
 20049
 
 gene;island
 
 0.97328
 
 0.90048
 
 Not queried
 
  1.5282745
 
 3.43e-01
 
 1.00e+00
  
 
  
 16
 
  89293993
 
  89294245
 
  PDF   STATS   ENSEMBL   UCSC 
 
  253
 
  5
 
 14903
 
 gene
 
 0.97316
 
 0.93890
 
 Not queried
 
  1.5271619
 
 5.56e-02
 
 1.00e+00
  
 
  
 14
 
 101335129
 
 101335386
 
  PDF   STATS   ENSEMBL   UCSC 
 
  258
 
  5
 
 12581
 
 tss
 
 0.48114
 
 0.69143
 
 Not queried
 
  1.5269490
 
 7.94e-03
 
 1.00e+00
  
 
  
 11
 
 117651217
 
 117651424
 
  PDF   STATS   ENSEMBL   UCSC 
 
  208
 
  4
 
 6902
 
 gene;island
 
 0.65093
 
 0.78748
 
 Not queried
 
  1.5269197
 
 5.71e-02
 
 1.00e+00
  
 
  
 2
 
 242844601
 
 242844943
 
  PDF   STATS   ENSEMBL   UCSC 
 
  343
 
  4
 
 25277
 
 island
 
 0.67226
 
 0.85953
 
 Not queried
 
  1.5268637
 
 1.14e-01
 
 1.00e+00
  
 
  
 3
 
  33260732
 
  33261204
 
  PDF   STATS   ENSEMBL   UCSC 
 
  473
 
  7
 
 28170
 
 tss;island
 
 0.30926
 
 0.06613
 
 Not queried
 
  1.5265667
 
 6.20e-01
 
 1.00e+00
  
 
  
 1
 
 166134182
 
 166134699
 
  PDF   STATS   ENSEMBL   UCSC 
 
  518
 
  6
 
 866
 
 gene;island
 
 0.02586
 
 0.07101
 
 Not queried
 
  1.5258265
 
 1.80e-01
 
 1.00e+00
  
 
  
 4
 
  42154912
 
  42154983
 
  PDF   STATS   ENSEMBL   UCSC 
 
   72
 
  6
 
 30319
 
 tss
 
 0.08810
 
 0.03241
 
 Not queried
 
  1.5255040
 
 3.94e-01
 
 1.00e+00
  
 
  
 2
 
 133428422
 
 133429299
 
  PDF   STATS   ENSEMBL   UCSC 
 
  878
 
 10
 
 24708
 
 island
 
 0.01131
 
 0.08896
 
 Not queried
 
  1.5251433
 
 3.55e-02
 
 1.00e+00
  
 
  
 12
 
 132832250
 
 132832440
 
  PDF   STATS   ENSEMBL   UCSC 
 
  191
 
  4
 
 9413
 
 gene;island
 
 0.67394
 
 0.82599
 
 Not queried
 
  1.5251073
 
 2.86e-02
 
 1.00e+00
  
 
  
 4
 
 156298166
 
 156298427
 
  PDF   STATS   ENSEMBL   UCSC 
 
  262
 
  7
 
 30602
 
 tss
 
 0.08218
 
 0.24152
 
 Not queried
 
  1.5251045
 
 1.28e-01
 
 1.00e+00
  
 
  
 1
 
 207494213
 
 207494733
 
  PDF   STATS   ENSEMBL   UCSC 
 
  521
 
  6
 
 2216
 
 tss;island
 
 0.02028
 
 0.01273
 
 Not queried
 
  1.5250029
 
 2.60e-02
 
 1.00e+00
  
 
  
 3
 
 119041204
 
 119041529
 
  PDF   STATS   ENSEMBL   UCSC 
 
  326
 
  4
 
 27865
 
 gene;island
 
 0.08323
 
 0.02764
 
 Not queried
 
  1.5248289
 
 4.86e-01
 
 1.00e+00
  
 
  
 8
 
   1327367
 
   1327621
 
  PDF   STATS   ENSEMBL   UCSC 
 
  255
 
  6
 
 39958
 
 island
 
 0.68994
 
 0.90529
 
 Not queried
 
  1.5246967
 
 4.11e-02
 
 1.00e+00
  
 
  
 16
 
  67428155
 
  67428497
 
  PDF   STATS   ENSEMBL   UCSC 
 
  343
 
  4
 
 15270
 
 tss;island
 
 0.42352
 
 0.66076
 
 Not queried
 
  1.5240103
 
 2.86e-02
 
 1.00e+00
  
 
  
 17
 
  66254884
 
  66255444
 
  PDF   STATS   ENSEMBL   UCSC 
 
  561
 
  8
 
 18656
 
 island
 
 0.02660
 
 0.10243
 
 Not queried
 
  1.5239151
 
 3.79e-02
 
 1.00e+00
  
 
  
 12
 
 107078410
 
 107078680
 
  PDF   STATS   ENSEMBL   UCSC 
 
  271
 
  5
 
 9256
 
 gene
 
 0.82311
 
 0.93191
 
 Not queried
 
  1.5237917
 
 3.10e-01
 
 1.00e+00
  
 
  
 10
 
  86300295
 
  86300605
 
  PDF   STATS   ENSEMBL   UCSC 
 
  311
 
  4
 
 5879
 
 island
 
 0.17245
 
 0.05176
 
 Not queried
 
  1.5232687
 
 2.00e-01
 
 1.00e+00
  
 
  
 11
 
    910094
 
    910475
 
  PDF   STATS   ENSEMBL   UCSC 
 
  382
 
  4
 
 6377
 
 gene;island
 
 0.03185
 
 0.04637
 
 Not queried
 
  1.5231776
 
 4.86e-01
 
 1.00e+00
  
 
  
 7
 
   5527927
 
   5528266
 
  PDF   STATS   ENSEMBL   UCSC 
 
  340
 
  4
 
 36815
 
 gene;island
 
 0.50083
 
 0.84299
 
 Not queried
 
  1.5229127
 
 5.71e-02
 
 1.00e+00
  
 
  
 19
 
  49004523
 
  49004834
 
  PDF   STATS   ENSEMBL   UCSC 
 
  312
 
  4
 
 20092
 
 gene;island
 
 0.11975
 
 0.07747
 
 Not queried
 
  1.5223756
 
 2.00e-01
 
 1.00e+00
  
 
  
 7
 
  44614305
 
  44614519
 
  PDF   STATS   ENSEMBL   UCSC 
 
  215
 
  5
 
 37504
 
 tss;island
 
 0.32965
 
 0.58543
 
 Not queried
 
  1.5218082
 
 3.10e-01
 
 1.00e+00
  
 
  
 2
 
  74153086
 
  74153317
 
  PDF   STATS   ENSEMBL   UCSC 
 
  232
 
  4
 
 23409
 
 tss;island
 
 0.73942
 
 0.88173
 
 Not queried
 
  1.5207102
 
 1.14e-01
 
 1.00e+00
  
 
  
 11
 
  67210033
 
  67210282
 
  PDF   STATS   ENSEMBL   UCSC 
 
  250
 
  5
 
 6739
 
 gene
 
 0.41761
 
 0.66917
 
 Not queried
 
  1.5203068
 
 1.59e-02
 
 1.00e+00
  
 
  
 10
 
  37413610
 
  37413799
 
  PDF   STATS   ENSEMBL   UCSC 
 
  190
 
  4
 
 5013
 
 tss;island
 
 0.54181
 
 0.77471
 
 Not queried
 
  1.5202330
 
 2.86e-02
 
 1.00e+00
  
 
  
 17
 
  72350354
 
  72350710
 
  PDF   STATS   ENSEMBL   UCSC 
 
  357
 
  4
 
 16716
 
 gene;island
 
 0.18101
 
 0.42592
 
 Not queried
 
  1.5198740
 
 2.00e-01
 
 1.00e+00
  
 
  
 5
 
  65222276
 
  65222522
 
  PDF   STATS   ENSEMBL   UCSC 
 
  247
 
  5
 
 32777
 
 island
 
 0.00328
 
 0.03134
 
 Not queried
 
  1.5169115
 
 4.21e-01
 
 1.00e+00
  
 
  
 19
 
  55866087
 
  55866412
 
  PDF   STATS   ENSEMBL   UCSC 
 
  326
 
  9
 
 22383
 
 island
 
 0.03774
 
 0.08280
 
 Not queried
 
  1.5167239
 
 1.61e-01
 
 1.00e+00
  
 
  
 5
 
  93447204
 
  93447700
 
  PDF   STATS   ENSEMBL   UCSC 
 
  497
 
 10
 
 32902
 
 island
 
 0.02459
 
 0.10154
 
 Not queried
 
  1.5165944
 
 1.23e-01
 
 1.00e+00
  
 
  
 X
 
  54466428
 
  54466849
 
  PDF   STATS   ENSEMBL   UCSC 
 
  422
 
  9
 
 41334
 
 tss
 
 0.05076
 
 0.01917
 
 Not queried
 
  1.5161669
 
 1.06e-02
 
 1.00e+00
  
 
  
 17
 
   7909776
 
   7910040
 
  PDF   STATS   ENSEMBL   UCSC 
 
  265
 
  4
 
 16387
 
 gene;island
 
 0.90035
 
 0.76153
 
 Not queried
 
  1.5161624
 
 2.00e-01
 
 1.00e+00
  
 
  
 6
 
  19804711
 
  19805404
 
  PDF   STATS   ENSEMBL   UCSC 
 
  694
 
  7
 
 35419
 
 island
 
 0.01461
 
 0.05984
 
 Not queried
 
  1.5157304
 
 2.59e-01
 
 1.00e+00
  
 
  
 4
 
   3512250
 
   3512607
 
  PDF   STATS   ENSEMBL   UCSC 
 
  358
 
  4
 
 30794
 
 island
 
 0.98041
 
 0.92448
 
 Not queried
 
  1.5156572
 
 2.86e-02
 
 1.00e+00
  
 
  
 13
 
  21140606
 
  21140776
 
  PDF   STATS   ENSEMBL   UCSC 
 
  171
 
  4
 
 11305
 
 tss;island
 
 0.01865
 
 0.00871
 
 Not queried
 
  1.5155083
 
 3.43e-01
 
 1.00e+00
  
 
  
 8
 
   1106401
 
   1106743
 
  PDF   STATS   ENSEMBL   UCSC 
 
  343
 
  5
 
 39950
 
 island
 
 0.89569
 
 0.90187
 
 Not queried
 
  1.5153987
 
 5.48e-01
 
 1.00e+00
  
 
  
 11
 
  44324759
 
  44325029
 
  PDF   STATS   ENSEMBL   UCSC 
 
  271
 
  5
 
 6571
 
 gene;island
 
 0.89146
 
 0.70007
 
 Not queried
 
  1.5153886
 
 1.51e-01
 
 1.00e+00
  
 
  
 10
 
  95848649
 
  95848841
 
  PDF   STATS   ENSEMBL   UCSC 
 
  193
 
  4
 
 4671
 
 gene
 
 0.60929
 
 0.35723
 
 Not queried
 
  1.5147203
 
 2.00e-01
 
 1.00e+00
  
 
  
 8
 
  56791576
 
  56791882
 
  PDF   STATS   ENSEMBL   UCSC 
 
  307
 
  5
 
 39645
 
 tss;island
 
 0.05029
 
 0.00964
 
 Not queried
 
  1.5147003
 
 3.10e-01
 
 1.00e+00
  
 
  
 1
 
  35226707
 
  35227272
 
  PDF   STATS   ENSEMBL   UCSC 
 
  566
 
  6
 
 411
 
 gene;island
 
 0.85364
 
 0.64478
 
 Not queried
 
  1.5140399
 
 4.11e-02
 
 1.00e+00
  
 
  
 15
 
  68113328
 
  68113580
 
  PDF   STATS   ENSEMBL   UCSC 
 
  253
 
  4
 
 14223
 
 island
 
 0.02101
 
 0.04019
 
 Not queried
 
  1.5138577
 
 2.00e-01
 
 1.00e+00
  
 
  
 14
 
  94393497
 
  94393816
 
  PDF   STATS   ENSEMBL   UCSC 
 
  320
 
  5
 
 12165
 
 gene;tss
 
 0.81382
 
 0.92035
 
 Not queried
 
  1.5132254
 
 9.52e-02
 
 1.00e+00
  
 
  
 9
 
  74764263
 
  74764504
 
  PDF   STATS   ENSEMBL   UCSC 
 
  242
 
  4
 
 40828
 
 island
 
 0.14324
 
 0.31828
 
 Not queried
 
  1.5129648
 
 5.71e-02
 
 1.00e+00
  
 
  
 17
 
  47840643
 
  47841033
 
  PDF   STATS   ENSEMBL   UCSC 
 
  391
 
  5
 
 16640
 
 gene;island
 
 0.03247
 
 0.01406
 
 Not queried
 
  1.5126390
 
 8.41e-01
 
 1.00e+00
  
 
  
 19
 
  44455140
 
  44455383
 
  PDF   STATS   ENSEMBL   UCSC 
 
  244
 
  8
 
 20801
 
 tss
 
 0.00689
 
 0.03457
 
 Not queried
 
  1.5124763
 
 1.95e-01
 
 1.00e+00
  
 
  
 6
 
   3849688
 
   3849818
 
  PDF   STATS   ENSEMBL   UCSC 
 
  131
 
  5
 
 33474
 
 gene
 
 0.02970
 
 0.07356
 
 Not queried
 
  1.5123435
 
 2.22e-01
 
 1.00e+00
  
 
  
 12
 
 123319216
 
 123319366
 
  PDF   STATS   ENSEMBL   UCSC 
 
  151
 
  4
 
 10063
 
 tss;island
 
 0.06510
 
 0.02244
 
 Not queried
 
  1.5117587
 
 3.43e-01
 
 1.00e+00
  
 
  
 6
 
 166868856
 
 166869078
 
  PDF   STATS   ENSEMBL   UCSC 
 
  223
 
  4
 
 34479
 
 gene;island
 
 0.96738
 
 0.90917
 
 Not queried
 
  1.5117160
 
 2.86e-02
 
 1.00e+00
  
 
  
 15
 
  23932620
 
  23933079
 
  PDF   STATS   ENSEMBL   UCSC 
 
  460
 
  5
 
 13487
 
 tss;island
 
 0.21595
 
 0.47438
 
 Not queried
 
  1.5110048
 
 4.21e-01
 
 1.00e+00
  
 
  
 2
 
 102091195
 
 102091321
 
  PDF   STATS   ENSEMBL   UCSC 
 
  127
 
  6
 
 23510
 
 tss
 
 0.34811
 
 0.57651
 
 Not queried
 
  1.5103617
 
 4.33e-03
 
 1.00e+00
  
 
  
 X
 
  14048147
 
  14048281
 
  PDF   STATS   ENSEMBL   UCSC 
 
  135
 
  8
 
 41195
 
 tss
 
 0.02309
 
 0.03965
 
 Not queried
 
  1.5102707
 
 1.30e-01
 
 1.00e+00
  
 
  
 8
 
  57233269
 
  57233391
 
  PDF   STATS   ENSEMBL   UCSC 
 
  123
 
  4
 
 39649
 
 tss;island
 
 0.47794
 
 0.78842
 
 Not queried
 
  1.5100925
 
 3.43e-01
 
 1.00e+00
  
 
  
 5
 
 141392652
 
 141392853
 
  PDF   STATS   ENSEMBL   UCSC 
 
  202
 
  6
 
 32335
 
 tss
 
 0.03047
 
 0.08252
 
 Not queried
 
  1.5098485
 
 4.11e-02
 
 1.00e+00
  
 
  
 16
 
  30075788
 
  30076044
 
  PDF   STATS   ENSEMBL   UCSC 
 
  257
 
  5
 
 14687
 
 gene;tss;island
 
 0.07452
 
 0.01965
 
 Not queried
 
  1.5098303
 
 1.51e-01
 
 1.00e+00
  
 
  
 11
 
  89463427
 
  89463689
 
  PDF   STATS   ENSEMBL   UCSC 
 
  263
 
  4
 
 8698
 
 island
 
 0.88017
 
 0.70501
 
 Not queried
 
  1.5094993
 
 3.43e-01
 
 1.00e+00
  
 
  
 16
 
  86588418
 
  86588622
 
  PDF   STATS   ENSEMBL   UCSC 
 
  205
 
  4
 
 14855
 
 gene;tss;island
 
 0.02835
 
 0.04165
 
 Not queried
 
  1.5085679
 
 4.86e-01
 
 1.00e+00
  
 
  
 6
 
  28753834
 
  28754198
 
  PDF   STATS   ENSEMBL   UCSC 
 
  365
 
  5
 
 35531
 
 island
 
 0.58983
 
 0.33576
 
 Not queried
 
  1.5083392
 
 7.94e-03
 
 1.00e+00
  
 
  
 22
 
  27053195
 
  27053934
 
  PDF   STATS   ENSEMBL   UCSC 
 
  740
 
 15
 
 27336
 
 island
 
 0.08329
 
 0.02876
 
 Not queried
 
  1.5083144
 
 4.27e-03
 
 1.00e+00
  
 
  
 10
 
   7670683
 
   7670827
 
  PDF   STATS   ENSEMBL   UCSC 
 
  145
 
  4
 
 4472
 
 gene
 
 0.71842
 
 0.89981
 
 Not queried
 
  1.5083030
 
 2.00e-01
 
 1.00e+00
  
 
  
 8
 
 143359879
 
 143360290
 
  PDF   STATS   ENSEMBL   UCSC 
 
  412
 
  4
 
 39393
 
 gene;island
 
 0.63444
 
 0.81723
 
 Not queried
 
  1.5082559
 
 1.14e-01
 
 1.00e+00
  
 
  
 16
 
   3085468
 
   3085982
 
  PDF   STATS   ENSEMBL   UCSC 
 
  515
 
  6
 
 15603
 
 island
 
 0.75331
 
 0.51759
 
 Not queried
 
  1.5080884
 
 3.94e-01
 
 1.00e+00
  
 
  
 12
 
  57634549
 
  57634855
 
  PDF   STATS   ENSEMBL   UCSC 
 
  307
 
  7
 
 9789
 
 tss
 
 0.04778
 
 0.05778
 
 Not queried
 
  1.5078431
 
 9.73e-02
 
 1.00e+00
  
 
  
 2
 
 118616155
 
 118616576
 
  PDF   STATS   ENSEMBL   UCSC 
 
  422
 
  5
 
 24600
 
 island
 
 0.65514
 
 0.41826
 
 Not queried
 
  1.5075129
 
 6.90e-01
 
 1.00e+00
  
 
  
 10
 
  90712684
 
  90712948
 
  PDF   STATS   ENSEMBL   UCSC 
 
  265
 
  6
 
 4654
 
 gene;tss
 
 0.61657
 
 0.82262
 
 Not queried
 
  1.5068845
 
 8.66e-03
 
 1.00e+00
  
 
  
 4
 
   3671067
 
   3671697
 
  PDF   STATS   ENSEMBL   UCSC 
 
  631
 
  5
 
 30801
 
 island
 
 0.89038
 
 0.94388
 
 Not queried
 
  1.5067833
 
 4.21e-01
 
 1.00e+00
  
 
  
 10
 
  18940606
 
  18940907
 
  PDF   STATS   ENSEMBL   UCSC 
 
  302
 
 10
 
 4959
 
 tss;island
 
 0.05168
 
 0.02214
 
 Not queried
 
  1.5063204
 
 2.32e-02
 
 1.00e+00
  
 
  
 10
 
  31892464
 
  31892769
 
  PDF   STATS   ENSEMBL   UCSC 
 
  306
 
  4
 
 5634
 
 island
 
 0.06681
 
 0.19590
 
 Not queried
 
  1.5061660
 
 2.00e-01
 
 1.00e+00
  
 
  
 16
 
  66599843
 
  66600105
 
  PDF   STATS   ENSEMBL   UCSC 
 
  263
 
  4
 
 14750
 
 gene
 
 0.85681
 
 0.95256
 
 Not queried
 
  1.5061538
 
 2.00e-01
 
 1.00e+00
  
 
  
 1
 
  39281613
 
  39282003
 
  PDF   STATS   ENSEMBL   UCSC 
 
  391
 
  4
 
 3085
 
 island
 
 0.91232
 
 0.77572
 
 Not queried
 
  1.5056486
 
 3.43e-01
 
 1.00e+00
  
 
  
 6
 
  74161911
 
  74162142
 
  PDF   STATS   ENSEMBL   UCSC 
 
  232
 
  7
 
 36194
 
 island
 
 0.68659
 
 0.50973
 
 Not queried
 
  1.5055715
 
 5.83e-04
 
 1.00e+00
  
 
  
 6
 
   5996893
 
   5997375
 
  PDF   STATS   ENSEMBL   UCSC 
 
  483
 
  6
 
 35340
 
 island
 
 0.01355
 
 0.02736
 
 Not queried
 
  1.5055177
 
 1.80e-01
 
 1.00e+00
  
 
  
 19
 
   4851963
 
   4852205
 
  PDF   STATS   ENSEMBL   UCSC 
 
  243
 
  4
 
 19705
 
 gene;island
 
 0.79732
 
 0.87731
 
 Not queried
 
  1.5053088
 
 6.86e-01
 
 1.00e+00
  
 
  
 10
 
  44070149
 
  44070200
 
  PDF   STATS   ENSEMBL   UCSC 
 
   52
 
  4
 
 5035
 
 tss
 
 0.12255
 
 0.05124
 
 Not queried
 
  1.5051402
 
 2.00e-01
 
 1.00e+00
  
 
  
 22
 
  32555255
 
  32555310
 
  PDF   STATS   ENSEMBL   UCSC 
 
   56
 
  4
 
 27045
 
 tss
 
 0.93282
 
 0.73576
 
 Not queried
 
  1.5050097
 
 2.00e-01
 
 1.00e+00
  
 
  
 1
 
 170630397
 
 170630734
 
  PDF   STATS   ENSEMBL   UCSC 
 
  338
 
  5
 
 3949
 
 island
 
 0.24127
 
 0.02754
 
 Not queried
 
  1.5050057
 
 2.22e-01
 
 1.00e+00
  
 
  
 12
 
 132982231
 
 132982483
 
  PDF   STATS   ENSEMBL   UCSC 
 
  253
 
  4
 
 11022
 
 island
 
 0.70476
 
 0.83926
 
 Not queried
 
  1.5049848
 
 1.14e-01
 
 1.00e+00
  
 
  
 2
 
 176993017
 
 176993320
 
  PDF   STATS   ENSEMBL   UCSC 
 
  304
 
  6
 
 23700
 
 tss;island
 
 0.28561
 
 0.10149
 
 Not queried
 
  1.5048598
 
 4.33e-03
 
 1.00e+00
  
 
  
 1
 
  75139470
 
  75139746
 
  PDF   STATS   ENSEMBL   UCSC 
 
  277
 
  6
 
 1693
 
 tss
 
 0.40846
 
 0.70910
 
 Not queried
 
  1.5044370
 
 2.16e-03
 
 1.00e+00
  
 
  
 10
 
 120863603
 
 120863861
 
  PDF   STATS   ENSEMBL   UCSC 
 
  259
 
  5
 
 6172
 
 island
 
 0.01725
 
 0.04594
 
 Not queried
 
  1.5041529
 
 5.56e-02
 
 1.00e+00
  
 
  
 6
 
  32362551
 
  32362744
 
  PDF   STATS   ENSEMBL   UCSC 
 
  194
 
  8
 
 34042
 
 gene
 
 0.72018
 
 0.60131
 
 Not queried
 
  1.5036081
 
 1.05e-01
 
 1.00e+00
  
 
  
 11
 
   1326508
 
   1326879
 
  PDF   STATS   ENSEMBL   UCSC 
 
  372
 
  4
 
 6396
 
 gene;island
 
 0.82564
 
 0.90218
 
 Not queried
 
  1.5035178
 
 5.71e-02
 
 1.00e+00
  
 
  
 10
 
  54074743
 
  54075082
 
  PDF   STATS   ENSEMBL   UCSC 
 
  340
 
  4
 
 4580
 
 gene;island
 
 0.03386
 
 0.08448
 
 Not queried
 
  1.5029746
 
 2.86e-02
 
 1.00e+00
  
 
  
 6
 
  28778091
 
  28778676
 
  PDF   STATS   ENSEMBL   UCSC 
 
  586
 
  9
 
 35532
 
 island
 
 0.01893
 
 0.04019
 
 Not queried
 
  1.5029416
 
 3.99e-03
 
 1.00e+00
  
 
  
 15
 
  52030519
 
  52030746
 
  PDF   STATS   ENSEMBL   UCSC 
 
  228
 
  4
 
 13323
 
 gene;tss;island
 
 0.83675
 
 0.66266
 
 Not queried
 
  1.5024652
 
 5.71e-02
 
 1.00e+00
  
 
  
 11
 
  22214333
 
  22214726
 
  PDF   STATS   ENSEMBL   UCSC 
 
  394
 
  6
 
 8111
 
 island
 
 0.06288
 
 0.10492
 
 Not queried
 
  1.5019591
 
 6.49e-02
 
 1.00e+00
  
 
  
 7
 
 157467883
 
 157468443
 
  PDF   STATS   ENSEMBL   UCSC 
 
  561
 
  7
 
 37276
 
 gene
 
 0.73586
 
 0.90107
 
 Not queried
 
  1.5017382
 
 1.28e-01
 
 1.00e+00
  
 
  
 8
 
  27950625
 
  27950972
 
  PDF   STATS   ENSEMBL   UCSC 
 
  348
 
  5
 
 39197
 
 gene
 
 0.00718
 
 0.03082
 
 Not queried
 
  1.5013806
 
 4.21e-01
 
 1.00e+00
  
 
  
 12
 
  52845930
 
  52846088
 
  PDF   STATS   ENSEMBL   UCSC 
 
  159
 
  4
 
 9703
 
 tss
 
 0.56918
 
 0.80331
 
 Not queried
 
  1.5005590
 
 2.86e-02
 
 1.00e+00
  
 
  
 20
 
    982623
 
    983130
 
  PDF   STATS   ENSEMBL   UCSC 
 
  508
 
  8
 
 25891
 
 island
 
 0.19342
 
 0.39378
 
 Not queried
 
  1.5004556
 
 5.74e-01
 
 1.00e+00
  
 
  
 7
 
  27194614
 
  27195036
 
  PDF   STATS   ENSEMBL   UCSC 
 
  423
 
  5
 
 36873
 
 gene;island
 
 0.54801
 
 0.75202
 
 Not queried
 
  1.5003484
 
 1.51e-01
 
 1.00e+00
  
 
  
 2
 
  73964587
 
  73964834
 
  PDF   STATS   ENSEMBL   UCSC 
 
  248
 
  6
 
 23405
 
 tss
 
 0.13399
 
 0.42365
 
 Not queried
 
  1.5002110
 
 2.40e-01
 
 1.00e+00
  
 
  
 19
 
  13124604
 
  13125281
 
  PDF   STATS   ENSEMBL   UCSC 
 
  678
 
  7
 
 19839
 
 gene;island
 
 0.24263
 
 0.08341
 
 Not queried
 
  1.4997536
 
 1.28e-01
 
 1.00e+00
  
 
  
 X
 
  37404389
 
  37404647
 
  PDF   STATS   ENSEMBL   UCSC 
 
  259
 
  4
 
 41714
 
 island
 
 0.75103
 
 0.89506
 
 Not queried
 
  1.4994343
 
 1.14e-01
 
 1.00e+00
  
 
  
 X
 
  69509542
 
  69510172
 
  PDF   STATS   ENSEMBL   UCSC 
 
  631
 
 12
 
 41367
 
 tss;island
 
 0.08638
 
 0.03004
 
 Not queried
 
  1.4991582
 
 2.32e-03
 
 1.00e+00
  
 
  
 17
 
  77375461
 
  77375842
 
  PDF   STATS   ENSEMBL   UCSC 
 
  382
 
  5
 
 16805
 
 gene
 
 0.55021
 
 0.75687
 
 Not queried
 
  1.4987980
 
 3.17e-02
 
 1.00e+00
  
 
  
 11
 
  74442547
 
  74442900
 
  PDF   STATS   ENSEMBL   UCSC 
 
  354
 
  4
 
 7540
 
 tss;island
 
 0.46462
 
 0.16013
 
 Not queried
 
  1.4987344
 
 4.86e-01
 
 1.00e+00
  
 
  
 3
 
  49203769
 
  49203975
 
  PDF   STATS   ENSEMBL   UCSC 
 
  207
 
  7
 
 29067
 
 island
 
 0.67909
 
 0.07934
 
 Not queried
 
  1.4981343
 
 8.05e-01
 
 1.00e+00
  
 
  
 1
 
  40420503
 
  40420692
 
  PDF   STATS   ENSEMBL   UCSC 
 
  190
 
  9
 
 1528
 
 tss
 
 0.10349
 
 0.03926
 
 Not queried
 
  1.4979991
 
 1.90e-01
 
 1.00e+00
  
 
  
 19
 
   1816188
 
   1816405
 
  PDF   STATS   ENSEMBL   UCSC 
 
  218
 
  8
 
 19627
 
 gene;island
 
 0.85177
 
 0.91640
 
 Not queried
 
  1.4978732
 
 2.81e-02
 
 1.00e+00
  
 
  
 22
 
  41763093
 
  41763370
 
  PDF   STATS   ENSEMBL   UCSC 
 
  278
 
  7
 
 27128
 
 tss
 
 0.39376
 
 0.59252
 
 Not queried
 
  1.4976924
 
 2.62e-02
 
 1.00e+00
  
 
  
 10
 
 101088533
 
 101088828
 
  PDF   STATS   ENSEMBL   UCSC 
 
  296
 
  4
 
 5263
 
 tss
 
 0.26660
 
 0.58511
 
 Not queried
 
  1.4976863
 
 3.43e-01
 
 1.00e+00
  
 
  
 8
 
  38585881
 
  38586183
 
  PDF   STATS   ENSEMBL   UCSC 
 
  303
 
  4
 
 39222
 
 gene
 
 0.13812
 
 0.35075
 
 Not queried
 
  1.4971251
 
 2.00e-01
 
 1.00e+00
  
 
  
 17
 
   7137923
 
   7138078
 
  PDF   STATS   ENSEMBL   UCSC 
 
  156
 
  5
 
 17031
 
 tss
 
 0.02083
 
 0.03932
 
 Not queried
 
  1.4970414
 
 1.51e-01
 
 1.00e+00
  
 
  
 10
 
  79793353
 
  79794011
 
  PDF   STATS   ENSEMBL   UCSC 
 
  659
 
 11
 
 5857
 
 island
 
 0.01944
 
 0.03017
 
 Not queried
 
  1.4963023
 
 1.16e-01
 
 1.00e+00
  
 
  
 5
 
  87969157
 
  87969330
 
  PDF   STATS   ENSEMBL   UCSC 
 
  174
 
  4
 
 31621
 
 gene;tss;island
 
 0.08003
 
 0.02315
 
 Not queried
 
  1.4962771
 
 1.14e-01
 
 1.00e+00
  
 
  
 X
 
 153561128
 
 153561349
 
  PDF   STATS   ENSEMBL   UCSC 
 
  222
 
  4
 
 42135
 
 island
 
 0.93750
 
 0.82855
 
 Not queried
 
  1.4961572
 
 1.14e-01
 
 1.00e+00
  
 
  
 10
 
 102748541
 
 102749041
 
  PDF   STATS   ENSEMBL   UCSC 
 
  501
 
  5
 
 4703
 
 gene;island
 
 0.72465
 
 0.89852
 
 Not queried
 
  1.4959386
 
 3.17e-02
 
 1.00e+00
  
 
  
 X
 
  28605581
 
  28605646
 
  PDF   STATS   ENSEMBL   UCSC 
 
   66
 
  4
 
 41241
 
 tss
 
 0.13562
 
 0.05895
 
 Not queried
 
  1.4954211
 
 4.86e-01
 
 1.00e+00
  
 
  
 18
 
  61034556
 
  61034655
 
  PDF   STATS   ENSEMBL   UCSC 
 
  100
 
  4
 
 19275
 
 tss
 
 0.00532
 
 0.02071
 
 Not queried
 
  1.4950048
 
 2.00e-01
 
 1.00e+00
  
 
  
 19
 
  40732691
 
  40732902
 
  PDF   STATS   ENSEMBL   UCSC 
 
  212
 
  8
 
 20748
 
 tss
 
 0.47656
 
 0.75226
 
 Not queried
 
  1.4945107
 
 6.99e-03
 
 1.00e+00
  
 
  
 16
 
    734504
 
    734844
 
  PDF   STATS   ENSEMBL   UCSC 
 
  341
 
  7
 
 14945
 
 tss
 
 0.07410
 
 0.18486
 
 Not queried
 
  1.4943686
 
 3.83e-01
 
 1.00e+00
  
 
  
 17
 
  46669455
 
  46669781
 
  PDF   STATS   ENSEMBL   UCSC 
 
  327
 
  8
 
 16619
 
 gene;island
 
 0.86298
 
 0.62479
 
 Not queried
 
  1.4935863
 
 6.99e-03
 
 1.00e+00
  
 
  
 7
 
 150715634
 
 150716072
 
  PDF   STATS   ENSEMBL   UCSC 
 
  439
 
  4
 
 37219
 
 gene;island
 
 0.55919
 
 0.73457
 
 Not queried
 
  1.4935145
 
 1.14e-01
 
 1.00e+00
  
 
  
 1
 
   3163341
 
   3163710
 
  PDF   STATS   ENSEMBL   UCSC 
 
  370
 
  4
 
 112
 
 gene;island
 
 0.76133
 
 0.86702
 
 Not queried
 
  1.4933146
 
 3.43e-01
 
 1.00e+00
  
 
  
 8
 
 145625497
 
 145625814
 
  PDF   STATS   ENSEMBL   UCSC 
 
  318
 
  5
 
 39457
 
 gene;island
 
 0.85885
 
 0.93836
 
 Not queried
 
  1.4922240
 
 5.56e-02
 
 1.00e+00
  
 
  
 4
 
  13530094
 
  13530470
 
  PDF   STATS   ENSEMBL   UCSC 
 
  377
 
  4
 
 30879
 
 island
 
 0.91552
 
 0.94511
 
 Not queried
 
  1.4920799
 
 4.86e-01
 
 1.00e+00
  
 
  
 2
 
 136498791
 
 136499146
 
  PDF   STATS   ENSEMBL   UCSC 
 
  356
 
  4
 
 23608
 
 tss;island
 
 0.10606
 
 0.26209
 
 Not queried
 
  1.4918830
 
 8.86e-01
 
 1.00e+00
  
 
  
 17
 
   7137795
 
   7138078
 
  PDF   STATS   ENSEMBL   UCSC 
 
  284
 
  7
 
 17891
 
 island
 
 0.02083
 
 0.03932
 
 Not queried
 
  1.4912646
 
 1.28e-01
 
 1.00e+00
  
 
  
 13
 
 113622539
 
 113622750
 
  PDF   STATS   ENSEMBL   UCSC 
 
  212
 
  7
 
 11517
 
 tss;island
 
 0.67044
 
 0.78131
 
 Not queried
 
  1.4906795
 
 5.30e-02
 
 1.00e+00
  
 
  
 19
 
   1816254
 
   1816405
 
  PDF   STATS   ENSEMBL   UCSC 
 
  152
 
  7
 
 20276
 
 tss
 
 0.86582
 
 0.91797
 
 Not queried
 
  1.4905406
 
 3.79e-02
 
 1.00e+00
  
 
  
 2
 
  10444432
 
  10445016
 
  PDF   STATS   ENSEMBL   UCSC 
 
  585
 
  6
 
 22529
 
 gene;island
 
 0.34234
 
 0.26170
 
 Not queried
 
  1.4892988
 
 2.40e-01
 
 1.00e+00
  
 
  
 X
 
  68047520
 
  68049085
 
  PDF   STATS   ENSEMBL   UCSC 
 
 1566
 
 26
 
 41838
 
 island
 
 0.09770
 
 0.03950
 
 Not queried
 
  1.4892637
 
 1.24e-03
 
 1.00e+00
  
 
  
 17
 
  39728362
 
  39728628
 
  PDF   STATS   ENSEMBL   UCSC 
 
  267
 
  5
 
 17307
 
 tss
 
 0.17510
 
 0.36579
 
 Not queried
 
  1.4892324
 
 7.94e-03
 
 1.00e+00
  
 
  
 12
 
  54366760
 
  54366875
 
  PDF   STATS   ENSEMBL   UCSC 
 
  116
 
  4
 
 9724
 
 tss
 
 0.21030
 
 0.44002
 
 Not queried
 
  1.4880460
 
 3.43e-01
 
 1.00e+00
  
 
  
 6
 
  41168941
 
  41169171
 
  PDF   STATS   ENSEMBL   UCSC 
 
  231
 
  6
 
 34903
 
 tss
 
 0.71544
 
 0.52950
 
 Not queried
 
  1.4879654
 
 3.10e-01
 
 1.00e+00
  
 
  
 8
 
  23423734
 
  23423960
 
  PDF   STATS   ENSEMBL   UCSC 
 
  227
 
  4
 
 39180
 
 gene
 
 0.82321
 
 0.93139
 
 Not queried
 
  1.4873860
 
 5.71e-02
 
 1.00e+00
  
 
  
 11
 
  10772408
 
  10772781
 
  PDF   STATS   ENSEMBL   UCSC 
 
  374
 
  9
 
 7128
 
 tss
 
 0.01187
 
 0.02904
 
 Not queried
 
  1.4867394
 
 1.42e-02
 
 1.00e+00
  
 
  
 11
 
  10772408
 
  10773025
 
  PDF   STATS   ENSEMBL   UCSC 
 
  618
 
 11
 
 8039
 
 island
 
 0.01187
 
 0.03222
 
 Not queried
 
  1.4867394
 
 1.28e-02
 
 1.00e+00
  
 
  
 5
 
 140743780
 
 140744155
 
  PDF   STATS   ENSEMBL   UCSC 
 
  376
 
  6
 
 31766
 
 gene;island
 
 0.70241
 
 0.87744
 
 Not queried
 
  1.4859994
 
 2.16e-03
 
 1.00e+00
  
 
  
 10
 
  93334974
 
  93335196
 
  PDF   STATS   ENSEMBL   UCSC 
 
  223
 
  4
 
 4658
 
 gene
 
 0.79990
 
 0.64396
 
 Not queried
 
  1.4852066
 
 3.43e-01
 
 1.00e+00
  
 
  
 1
 
  50513451
 
  50513661
 
  PDF   STATS   ENSEMBL   UCSC 
 
  211
 
  4
 
 1611
 
 tss
 
 0.02372
 
 0.07321
 
 Not queried
 
  1.4848647
 
 6.86e-01
 
 1.00e+00
  
 
  
 12
 
  56109593
 
  56110162
 
  PDF   STATS   ENSEMBL   UCSC 
 
  570
 
  8
 
 10482
 
 island
 
 0.06742
 
 0.04995
 
 Not queried
 
  1.4848141
 
 4.42e-01
 
 1.00e+00
  
 
  
 10
 
  52834498
 
  52835078
 
  PDF   STATS   ENSEMBL   UCSC 
 
  581
 
  5
 
 4577
 
 gene;island
 
 0.02909
 
 0.07735
 
 Not queried
 
  1.4843912
 
 5.56e-02
 
 1.00e+00
  
 
  
 11
 
 108464380
 
 108464498
 
  PDF   STATS   ENSEMBL   UCSC 
 
  119
 
  4
 
 7653
 
 tss
 
 0.01065
 
 0.01975
 
 Not queried
 
  1.4840507
 
 1.14e-01
 
 1.00e+00
  
 
  
 7
 
  99595095
 
  99595458
 
  PDF   STATS   ENSEMBL   UCSC 
 
  364
 
  5
 
 38678
 
 island
 
 0.30660
 
 0.57695
 
 Not queried
 
  1.4840051
 
 1.51e-01
 
 1.00e+00
  
 
  
 3
 
   9932180
 
   9932217
 
  PDF   STATS   ENSEMBL   UCSC 
 
   38
 
  5
 
 28092
 
 tss
 
 0.04255
 
 0.06170
 
 Not queried
 
  1.4839495
 
 4.21e-01
 
 1.00e+00
  
 
  
 4
 
 155253864
 
 155254602
 
  PDF   STATS   ENSEMBL   UCSC 
 
  739
 
  6
 
 30109
 
 gene;island
 
 0.02544
 
 0.06710
 
 Not queried
 
  1.4838035
 
 1.80e-01
 
 1.00e+00
  
 
  
 12
 
 127765195
 
 127765627
 
  PDF   STATS   ENSEMBL   UCSC 
 
  433
 
  4
 
 10957
 
 island
 
 0.06240
 
 0.27624
 
 Not queried
 
  1.4836714
 
 1.14e-01
 
 1.00e+00
  
 
  
 3
 
 131080235
 
 131080927
 
  PDF   STATS   ENSEMBL   UCSC 
 
  693
 
  9
 
 29436
 
 island
 
 0.55269
 
 0.70881
 
 Not queried
 
  1.4836557
 
 1.06e-02
 
 1.00e+00
  
 
  
 7
 
  26191458
 
  26191726
 
  PDF   STATS   ENSEMBL   UCSC 
 
  269
 
  6
 
 37416
 
 tss;island
 
 0.71484
 
 0.21802
 
 Not queried
 
  1.4832179
 
 3.10e-01
 
 1.00e+00
  
 
  
 11
 
 117667737
 
 117668336
 
  PDF   STATS   ENSEMBL   UCSC 
 
  600
 
 10
 
 8827
 
 island
 
 0.25335
 
 0.08279
 
 Not queried
 
  1.4831379
 
 1.23e-01
 
 1.00e+00
  
 
  
 2
 
 217236783
 
 217237043
 
  PDF   STATS   ENSEMBL   UCSC 
 
  261
 
 10
 
 23829
 
 tss
 
 0.02502
 
 0.05491
 
 Not queried
 
  1.4829346
 
 5.24e-02
 
 1.00e+00
  
 
  
 6
 
  74103959
 
  74104137
 
  PDF   STATS   ENSEMBL   UCSC 
 
  179
 
  4
 
 35005
 
 tss;island
 
 0.65682
 
 0.91000
 
 Not queried
 
  1.4826945
 
 3.43e-01
 
 1.00e+00
  
 
  
 14
 
 102391388
 
 102391830
 
  PDF   STATS   ENSEMBL   UCSC 
 
  443
 
  5
 
 12190
 
 gene;island
 
 0.83123
 
 0.89910
 
 Not queried
 
  1.4825556
 
 1.51e-01
 
 1.00e+00
  
 
  
 6
 
  31623180
 
  31623318
 
  PDF   STATS   ENSEMBL   UCSC 
 
  139
 
  4
 
 34761
 
 tss;island
 
 0.40775
 
 0.64988
 
 Not queried
 
  1.4823954
 
 6.86e-01
 
 1.00e+00
  
 
  
 10
 
    180188
 
    180755
 
  PDF   STATS   ENSEMBL   UCSC 
 
  568
 
 12
 
 5450
 
 island
 
 0.00815
 
 0.02090
 
 Not queried
 
  1.4820871
 
 5.97e-02
 
 1.00e+00
  
 
  
 7
 
  98739496
 
  98739782
 
  PDF   STATS   ENSEMBL   UCSC 
 
  287
 
  4
 
 37060
 
 gene;island
 
 0.05332
 
 0.11475
 
 Not queried
 
  1.4819870
 
 1.14e-01
 
 1.00e+00
  
 
  
 10
 
 115613834
 
 115614022
 
  PDF   STATS   ENSEMBL   UCSC 
 
  189
 
  6
 
 5354
 
 tss;island
 
 0.00634
 
 0.02426
 
 Not queried
 
  1.4815635
 
 4.11e-02
 
 1.00e+00
  
 
  
 2
 
 237086398
 
 237086782
 
  PDF   STATS   ENSEMBL   UCSC 
 
  385
 
  4
 
 25189
 
 island
 
 0.16640
 
 0.42355
 
 Not queried
 
  1.4814118
 
 1.14e-01
 
 1.00e+00
  
 
  
 3
 
  79817125
 
  79817333
 
  PDF   STATS   ENSEMBL   UCSC 
 
  209
 
  4
 
 28405
 
 tss
 
 0.03224
 
 0.05854
 
 Not queried
 
  1.4810462
 
 4.86e-01
 
 1.00e+00
  
 
  
 17
 
  22191704
 
  22191979
 
  PDF   STATS   ENSEMBL   UCSC 
 
  276
 
  4
 
 18098
 
 island
 
 0.72159
 
 0.56653
 
 Not queried
 
  1.4804096
 
 2.00e-01
 
 1.00e+00
  
 
  
 17
 
  46831306
 
  46831742
 
  PDF   STATS   ENSEMBL   UCSC 
 
  437
 
  4
 
 18474
 
 island
 
 0.79327
 
 0.58039
 
 Not queried
 
  1.4802005
 
 2.00e-01
 
 1.00e+00
  
 
  
 17
 
  45894369
 
  45894729
 
  PDF   STATS   ENSEMBL   UCSC 
 
  361
 
  4
 
 16611
 
 gene;island
 
 0.60479
 
 0.80725
 
 Not queried
 
  1.4798009
 
 2.00e-01
 
 1.00e+00
  
 
  
 1
 
 235811811
 
 235812280
 
  PDF   STATS   ENSEMBL   UCSC 
 
  470
 
  6
 
 1089
 
 gene;island
 
 0.50698
 
 0.27198
 
 Not queried
 
  1.4797720
 
 3.94e-01
 
 1.00e+00
  
 
  
 5
 
  93077316
 
  93077405
 
  PDF   STATS   ENSEMBL   UCSC 
 
   90
 
  4
 
 32119
 
 tss
 
 0.52417
 
 0.75441
 
 Not queried
 
  1.4794920
 
 3.43e-01
 
 1.00e+00
  
 
  
 7
 
   2316876
 
   2317124
 
  PDF   STATS   ENSEMBL   UCSC 
 
  249
 
  6
 
 36736
 
 gene;island
 
 0.85175
 
 0.94528
 
 Not queried
 
  1.4794398
 
 9.31e-02
 
 1.00e+00
  
 
  
 5
 
 139944046
 
 139944115
 
  PDF   STATS   ENSEMBL   UCSC 
 
   70
 
  4
 
 31730
 
 gene
 
 0.00676
 
 0.01920
 
 Not queried
 
  1.4793644
 
 4.86e-01
 
 1.00e+00
  
 
  
 5
 
 140473857
 
 140474578
 
  PDF   STATS   ENSEMBL   UCSC 
 
  722
 
  9
 
 33147
 
 island
 
 0.02479
 
 0.08210
 
 Not queried
 
  1.4789970
 
 7.70e-02
 
 1.00e+00
  
 
  
 22
 
  19718172
 
  19718480
 
  PDF   STATS   ENSEMBL   UCSC 
 
  309
 
  4
 
 27261
 
 island
 
 0.02998
 
 0.11792
 
 Not queried
 
  1.4786819
 
 2.00e-01
 
 1.00e+00
  
 
  
 4
 
    737930
 
    738199
 
  PDF   STATS   ENSEMBL   UCSC 
 
  270
 
  4
 
 29790
 
 gene;island
 
 0.67034
 
 0.84838
 
 Not queried
 
  1.4786189
 
 2.00e-01
 
 1.00e+00
  
 
  
 11
 
   6518108
 
   6518485
 
  PDF   STATS   ENSEMBL   UCSC 
 
  378
 
  7
 
 7093
 
 tss
 
 0.77989
 
 0.88882
 
 Not queried
 
  1.4786044
 
 2.59e-01
 
 1.00e+00
  
 
  
 7
 
  75115567
 
  75115859
 
  PDF   STATS   ENSEMBL   UCSC 
 
  293
 
  9
 
 38520
 
 island
 
 0.03495
 
 0.01947
 
 Not queried
 
  1.4780166
 
 3.87e-01
 
 1.00e+00
  
 
  
 4
 
 185926063
 
 185926620
 
  PDF   STATS   ENSEMBL   UCSC 
 
  558
 
  7
 
 31411
 
 island
 
 0.16625
 
 0.06042
 
 Not queried
 
  1.4779832
 
 2.59e-01
 
 1.00e+00
  
 
  
 2
 
 232395269
 
 232395463
 
  PDF   STATS   ENSEMBL   UCSC 
 
  195
 
  6
 
 23904
 
 tss;island
 
 0.03501
 
 0.14462
 
 Not queried
 
  1.4778064
 
 9.31e-02
 
 1.00e+00
  
 
  
 5
 
 178593785
 
 178594649
 
  PDF   STATS   ENSEMBL   UCSC 
 
  865
 
  9
 
 31870
 
 gene;island
 
 0.14410
 
 0.51823
 
 Not queried
 
  1.4775072
 
 4.89e-01
 
 1.00e+00
  
 
  
 20
 
  62715756
 
  62715942
 
  PDF   STATS   ENSEMBL   UCSC 
 
  187
 
  5
 
 25873
 
 tss
 
 0.64301
 
 0.39439
 
 Not queried
 
  1.4773590
 
 4.21e-01
 
 1.00e+00
  
 
  
 11
 
  32421514
 
  32421845
 
  PDF   STATS   ENSEMBL   UCSC 
 
  332
 
  6
 
 6546
 
 gene
 
 0.41148
 
 0.71859
 
 Not queried
 
  1.4770060
 
 4.11e-02
 
 1.00e+00
  
 
  
 6
 
  33037413
 
  33037690
 
  PDF   STATS   ENSEMBL   UCSC 
 
  278
 
  6
 
 34116
 
 gene
 
 0.76172
 
 0.56026
 
 Not queried
 
  1.4767083
 
 1.32e-01
 
 1.00e+00
  
 
  
 X
 
  67653315
 
  67653925
 
  PDF   STATS   ENSEMBL   UCSC 
 
  611
 
  8
 
 41355
 
 tss
 
 0.08138
 
 0.02132
 
 Not queried
 
  1.4764237
 
 1.04e-02
 
 1.00e+00
  
 
  
 5
 
  43037123
 
  43037666
 
  PDF   STATS   ENSEMBL   UCSC 
 
  544
 
  6
 
 32704
 
 island
 
 0.95584
 
 0.85952
 
 Not queried
 
  1.4764235
 
 9.31e-02
 
 1.00e+00
  
 
  
 1
 
  63791922
 
  63792239
 
  PDF   STATS   ENSEMBL   UCSC 
 
  318
 
  6
 
 3336
 
 island
 
 0.03683
 
 0.19314
 
 Not queried
 
  1.4764150
 
 4.11e-02
 
 1.00e+00
  
 
  
 1
 
   4221331
 
   4221616
 
  PDF   STATS   ENSEMBL   UCSC 
 
  286
 
  5
 
 2577
 
 island
 
 0.66450
 
 0.75010
 
 Not queried
 
  1.4761170
 
 2.22e-01
 
 1.00e+00
  
 
  
 19
 
    571107
 
    571416
 
  PDF   STATS   ENSEMBL   UCSC 
 
  310
 
  5
 
 20237
 
 tss;island
 
 0.03831
 
 0.13624
 
 Not queried
 
  1.4759918
 
 5.56e-02
 
 1.00e+00
  
 
  
 6
 
  31657467
 
  31657855
 
  PDF   STATS   ENSEMBL   UCSC 
 
  389
 
  4
 
 33875
 
 gene
 
 0.65566
 
 0.86092
 
 Not queried
 
  1.4758014
 
 4.86e-01
 
 1.00e+00
  
 
  
 6
 
  28550343
 
  28550437
 
  PDF   STATS   ENSEMBL   UCSC 
 
   95
 
  5
 
 33574
 
 gene
 
 0.96037
 
 0.90654
 
 Not queried
 
  1.4756330
 
 9.52e-02
 
 1.00e+00
  
 
  
 6
 
  31930566
 
  31930875
 
  PDF   STATS   ENSEMBL   UCSC 
 
  310
 
  6
 
 33940
 
 gene;island
 
 0.81820
 
 0.91941
 
 Not queried
 
  1.4755927
 
 9.31e-02
 
 1.00e+00
  
 
  
 16
 
    127772
 
    128016
 
  PDF   STATS   ENSEMBL   UCSC 
 
  245
 
  4
 
 14493
 
 gene;tss;island
 
 0.02121
 
 0.07946
 
 Not queried
 
  1.4751460
 
 2.00e-01
 
 1.00e+00
  
 
  
 1
 
 154934627
 
 154934734
 
  PDF   STATS   ENSEMBL   UCSC 
 
  108
 
  4
 
 1967
 
 tss;island
 
 0.03134
 
 0.02388
 
 Not queried
 
  1.4748301
 
 4.86e-01
 
 1.00e+00
  
 
  
 16
 
  88807658
 
  88807933
 
  PDF   STATS   ENSEMBL   UCSC 
 
  276
 
  5
 
 14884
 
 gene;island
 
 0.71263
 
 0.87329
 
 Not queried
 
  1.4746928
 
 1.59e-02
 
 1.00e+00
  
 
  
 12
 
  48396630
 
  48397225
 
  PDF   STATS   ENSEMBL   UCSC 
 
  596
 
  5
 
 9082
 
 gene;island
 
 0.07683
 
 0.25383
 
 Not queried
 
  1.4746632
 
 5.56e-02
 
 1.00e+00
  
 
  
 5
 
 172710685
 
 172711094
 
  PDF   STATS   ENSEMBL   UCSC 
 
  410
 
  6
 
 33347
 
 island
 
 0.33636
 
 0.06344
 
 Not queried
 
  1.4746244
 
 6.49e-02
 
 1.00e+00
  
 
  
 13
 
 113104883
 
 113105341
 
  PDF   STATS   ENSEMBL   UCSC 
 
  459
 
  5
 
 11890
 
 island
 
 0.67066
 
 0.81308
 
 Not queried
 
  1.4744454
 
 4.21e-01
 
 1.00e+00
  
 
  
 6
 
  32018906
 
  32019556
 
  PDF   STATS   ENSEMBL   UCSC 
 
  651
 
  5
 
 33955
 
 gene
 
 0.59577
 
 0.47723
 
 Not queried
 
  1.4739649
 
 2.22e-01
 
 1.00e+00
  
 
  
 15
 
  83240578
 
  83240791
 
  PDF   STATS   ENSEMBL   UCSC 
 
  214
 
  6
 
 13829
 
 tss
 
 0.16050
 
 0.38083
 
 Not queried
 
  1.4731895
 
 9.31e-02
 
 1.00e+00
  
 
  
 19
 
  47217696
 
  47218031
 
  PDF   STATS   ENSEMBL   UCSC 
 
  336
 
  5
 
 20860
 
 tss
 
 0.08970
 
 0.27193
 
 Not queried
 
  1.4729592
 
 9.52e-02
 
 1.00e+00
  
 
  
 14
 
 100752144
 
 100752463
 
  PDF   STATS   ENSEMBL   UCSC 
 
  320
 
  4
 
 13107
 
 island
 
 0.21061
 
 0.40667
 
 Not queried
 
  1.4723724
 
 3.43e-01
 
 1.00e+00
  
 
  
 3
 
 131245815
 
 131246097
 
  PDF   STATS   ENSEMBL   UCSC 
 
  283
 
  4
 
 29439
 
 island
 
 0.44508
 
 0.74515
 
 Not queried
 
  1.4721148
 
 3.43e-01
 
 1.00e+00
  
 
  
 14
 
  37641134
 
  37641726
 
  PDF   STATS   ENSEMBL   UCSC 
 
  593
 
  8
 
 12040
 
 gene;island
 
 0.01816
 
 0.04305
 
 Not queried
 
  1.4718694
 
 1.05e-01
 
 1.00e+00
  
 
  
 17
 
  46795860
 
  46796298
 
  PDF   STATS   ENSEMBL   UCSC 
 
  439
 
  4
 
 18463
 
 island
 
 0.03838
 
 0.13642
 
 Not queried
 
  1.4716451
 
 1.14e-01
 
 1.00e+00
  
 
  
 8
 
 145008909
 
 145009406
 
  PDF   STATS   ENSEMBL   UCSC 
 
  498
 
  5
 
 39434
 
 gene;island
 
 0.80373
 
 0.89447
 
 Not queried
 
  1.4713151
 
 5.56e-02
 
 1.00e+00
  
 
  
 20
 
  26188908
 
  26188997
 
  PDF   STATS   ENSEMBL   UCSC 
 
   90
 
  7
 
 26027
 
 island
 
 0.06960
 
 0.20427
 
 Not queried
 
  1.4709007
 
 9.73e-02
 
 1.00e+00
  
 
  
 16
 
   1133074
 
   1133378
 
  PDF   STATS   ENSEMBL   UCSC 
 
  305
 
  5
 
 15484
 
 island
 
 0.84361
 
 0.93768
 
 Not queried
 
  1.4707953
 
 1.51e-01
 
 1.00e+00
  
 
  
 2
 
 109335627
 
 109335769
 
  PDF   STATS   ENSEMBL   UCSC 
 
  143
 
  5
 
 23527
 
 tss;island
 
 0.01139
 
 0.05736
 
 Not queried
 
  1.4705514
 
 2.22e-01
 
 1.00e+00
  
 
  
 6
 
  37225949
 
  37226214
 
  PDF   STATS   ENSEMBL   UCSC 
 
  266
 
  5
 
 34256
 
 gene;tss;island
 
 0.00808
 
 0.01278
 
 Not queried
 
  1.4702971
 
 9.52e-02
 
 1.00e+00
  
 
  
 2
 
 130956098
 
 130956271
 
  PDF   STATS   ENSEMBL   UCSC 
 
  174
 
  7
 
 23588
 
 tss
 
 0.96692
 
 0.86452
 
 Not queried
 
  1.4693394
 
 2.09e-01
 
 1.00e+00
  
 
  
 1
 
 181454975
 
 181455181
 
  PDF   STATS   ENSEMBL   UCSC 
 
  207
 
  4
 
 906
 
 gene;island
 
 0.03980
 
 0.08227
 
 Not queried
 
  1.4688006
 
 2.86e-02
 
 1.00e+00
  
 
  
 13
 
 112200641
 
 112201036
 
  PDF   STATS   ENSEMBL   UCSC 
 
  396
 
  6
 
 11868
 
 island
 
 0.69463
 
 0.84585
 
 Not queried
 
  1.4686460
 
 2.40e-01
 
 1.00e+00
  
 
  
 5
 
 128300967
 
 128301185
 
  PDF   STATS   ENSEMBL   UCSC 
 
  219
 
  6
 
 32201
 
 tss
 
 0.02201
 
 0.04869
 
 Not queried
 
  1.4681917
 
 4.85e-01
 
 1.00e+00
  
 
  
 3
 
  52279403
 
  52279700
 
  PDF   STATS   ENSEMBL   UCSC 
 
  298
 
  5
 
 28333
 
 tss;island
 
 0.31840
 
 0.28724
 
 Not queried
 
  1.4673104
 
 5.48e-01
 
 1.00e+00
  
 
  
 20
 
  30062970
 
  30063249
 
  PDF   STATS   ENSEMBL   UCSC 
 
  280
 
  8
 
 26031
 
 island
 
 0.05603
 
 0.03085
 
 Not queried
 
  1.4666650
 
 4.99e-02
 
 1.00e+00
  
 
  
 19
 
  18721531
 
  18721840
 
  PDF   STATS   ENSEMBL   UCSC 
 
  310
 
  4
 
 21753
 
 island
 
 0.17423
 
 0.06686
 
 Not queried
 
  1.4662409
 
 6.86e-01
 
 1.00e+00
  
 
  
 4
 
   2470698
 
   2470786
 
  PDF   STATS   ENSEMBL   UCSC 
 
   89
 
  4
 
 30203
 
 tss
 
 0.01010
 
 0.08980
 
 Not queried
 
  1.4662390
 
 1.14e-01
 
 1.00e+00
  
 
  
 4
 
   2470698
 
   2471025
 
  PDF   STATS   ENSEMBL   UCSC 
 
  328
 
  6
 
 30768
 
 island
 
 0.01010
 
 0.05884
 
 Not queried
 
  1.4662390
 
 6.49e-02
 
 1.00e+00
  
 
  
 19
 
  55691730
 
  55692003
 
  PDF   STATS   ENSEMBL   UCSC 
 
  274
 
  7
 
 21036
 
 tss;island
 
 0.02298
 
 0.08796
 
 Not queried
 
  1.4655719
 
 2.09e-01
 
 1.00e+00
  
 
  
 X
 
  69352975
 
  69353185
 
  PDF   STATS   ENSEMBL   UCSC 
 
  211
 
  6
 
 41364
 
 tss;island
 
 0.18620
 
 0.05937
 
 Not queried
 
  1.4644951
 
 6.49e-02
 
 1.00e+00
  
 
  
 8
 
  49293379
 
  49293600
 
  PDF   STATS   ENSEMBL   UCSC 
 
  222
 
  4
 
 40217
 
 island
 
 0.16457
 
 0.07109
 
 Not queried
 
  1.4642687
 
 4.86e-01
 
 1.00e+00
  
 
  
 17
 
  40936498
 
  40936820
 
  PDF   STATS   ENSEMBL   UCSC 
 
  323
 
  5
 
 16553
 
 gene;island
 
 0.91945
 
 0.80125
 
 Not queried
 
  1.4629328
 
 7.94e-03
 
 1.00e+00
  
 
  
 X
 
  51139025
 
  51139247
 
  PDF   STATS   ENSEMBL   UCSC 
 
  223
 
  4
 
 41800
 
 island
 
 0.16569
 
 0.03925
 
 Not queried
 
  1.4627605
 
 1.14e-01
 
 1.00e+00
  
 
  
 19
 
   3207921
 
   3208130
 
  PDF   STATS   ENSEMBL   UCSC 
 
  210
 
  4
 
 19676
 
 gene;island
 
 0.85729
 
 0.94101
 
 Not queried
 
  1.4625940
 
 3.43e-01
 
 1.00e+00
  
 
  
 18
 
  70211774
 
  70211974
 
  PDF   STATS   ENSEMBL   UCSC 
 
  201
 
  6
 
 19284
 
 tss;island
 
 0.18678
 
 0.36116
 
 Not queried
 
  1.4622202
 
 5.89e-01
 
 1.00e+00
  
 
  
 6
 
  31473264
 
  31473581
 
  PDF   STATS   ENSEMBL   UCSC 
 
  318
 
  4
 
 33819
 
 gene
 
 0.90828
 
 0.66132
 
 Not queried
 
  1.4621625
 
 3.43e-01
 
 1.00e+00
  
 
  
 6
 
 101846650
 
 101847058
 
  PDF   STATS   ENSEMBL   UCSC 
 
  409
 
 12
 
 36308
 
 island
 
 0.09973
 
 0.03972
 
 Not queried
 
  1.4620993
 
 3.64e-03
 
 1.00e+00
  
 
  
 17
 
  34058433
 
  34058623
 
  PDF   STATS   ENSEMBL   UCSC 
 
  191
 
  5
 
 17241
 
 tss;island
 
 0.12613
 
 0.05868
 
 Not queried
 
  1.4610202
 
 5.56e-02
 
 1.00e+00
  
 
  
 2
 
  97524767
 
  97525009
 
  PDF   STATS   ENSEMBL   UCSC 
 
  243
 
  4
 
 23487
 
 tss;island
 
 0.56419
 
 0.46934
 
 Not queried
 
  1.4607590
 
 6.86e-01
 
 1.00e+00
  
 
  
 6
 
  30955358
 
  30955425
 
  PDF   STATS   ENSEMBL   UCSC 
 
   68
 
  5
 
 33783
 
 gene
 
 0.88821
 
 0.69389
 
 Not queried
 
  1.4607071
 
 9.52e-02
 
 1.00e+00
  
 
  
 2
 
 128453108
 
 128453484
 
  PDF   STATS   ENSEMBL   UCSC 
 
  377
 
  5
 
 24661
 
 island
 
 0.93265
 
 0.82171
 
 Not queried
 
  1.4605322
 
 1.51e-01
 
 1.00e+00
  
 
  
 22
 
  19710880
 
  19711051
 
  PDF   STATS   ENSEMBL   UCSC 
 
  172
 
  6
 
 26748
 
 gene;tss;island
 
 0.31022
 
 0.57929
 
 Not queried
 
  1.4604456
 
 2.16e-03
 
 1.00e+00
  
 
  
 17
 
  40120250
 
  40120496
 
  PDF   STATS   ENSEMBL   UCSC 
 
  247
 
  4
 
 16538
 
 gene;island
 
 0.85850
 
 0.94829
 
 Not queried
 
  1.4602443
 
 3.43e-01
 
 1.00e+00
  
 
  
 6
 
  74063982
 
  74064594
 
  PDF   STATS   ENSEMBL   UCSC 
 
  613
 
  8
 
 35002
 
 tss;island
 
 0.62920
 
 0.85902
 
 Not queried
 
  1.4599680
 
 3.79e-02
 
 1.00e+00
  
 
  
 2
 
 182549353
 
 182549604
 
  PDF   STATS   ENSEMBL   UCSC 
 
  252
 
  5
 
 24900
 
 island
 
 0.38064
 
 0.61664
 
 Not queried
 
  1.4596556
 
 2.22e-01
 
 1.00e+00
  
 
  
 16
 
   1404834
 
   1405223
 
  PDF   STATS   ENSEMBL   UCSC 
 
  390
 
  4
 
 14537
 
 gene;island
 
 0.81693
 
 0.93094
 
 Not queried
 
  1.4595456
 
 2.00e-01
 
 1.00e+00
  
 
  
 1
 
   3772978
 
   3773316
 
  PDF   STATS   ENSEMBL   UCSC 
 
  339
 
  4
 
 147
 
 gene;tss;island
 
 0.01562
 
 0.00808
 
 Not queried
 
  1.4595315
 
 4.86e-01
 
 1.00e+00
  
 
  
 7
 
   1130967
 
   1131133
 
  PDF   STATS   ENSEMBL   UCSC 
 
  167
 
  4
 
 36687
 
 gene;island
 
 0.73642
 
 0.83751
 
 Not queried
 
  1.4590295
 
 6.86e-01
 
 1.00e+00
  
 
  
 19
 
  49220102
 
  49220485
 
  PDF   STATS   ENSEMBL   UCSC 
 
  384
 
  6
 
 20099
 
 gene;island
 
 0.69093
 
 0.38514
 
 Not queried
 
  1.4589850
 
 2.16e-03
 
 1.00e+00
  
 
  
 2
 
  66809780
 
  66809985
 
  PDF   STATS   ENSEMBL   UCSC 
 
  206
 
  4
 
 24320
 
 island
 
 0.29771
 
 0.49229
 
 Not queried
 
  1.4589306
 
 5.71e-02
 
 1.00e+00
  
 
  
 17
 
   6023675
 
   6024017
 
  PDF   STATS   ENSEMBL   UCSC 
 
  343
 
  4
 
 16335
 
 gene;island
 
 0.63249
 
 0.79106
 
 Not queried
 
  1.4586489
 
 2.86e-02
 
 1.00e+00
  
 
  
 15
 
  72977851
 
  72978371
 
  PDF   STATS   ENSEMBL   UCSC 
 
  521
 
  5
 
 13381
 
 gene;tss;island
 
 0.02801
 
 0.02687
 
 Not queried
 
  1.4586453
 
 4.21e-01
 
 1.00e+00
  
 
  
 1
 
 159825552
 
 159825761
 
  PDF   STATS   ENSEMBL   UCSC 
 
  210
 
  4
 
 836
 
 gene;tss;island
 
 0.82617
 
 0.69825
 
 Not queried
 
  1.4578905
 
 1.14e-01
 
 1.00e+00
  
 
  
 2
 
 169658881
 
 169659047
 
  PDF   STATS   ENSEMBL   UCSC 
 
  167
 
  4
 
 23661
 
 tss
 
 0.72508
 
 0.86393
 
 Not queried
 
  1.4578169
 
 6.86e-01
 
 1.00e+00
  
 
  
 19
 
  54485321
 
  54485509
 
  PDF   STATS   ENSEMBL   UCSC 
 
  189
 
  6
 
 20175
 
 gene;tss;island
 
 0.17492
 
 0.33863
 
 Not queried
 
  1.4573173
 
 3.94e-01
 
 1.00e+00
  
 
  
 1
 
 113216774
 
 113217181
 
  PDF   STATS   ENSEMBL   UCSC 
 
  408
 
  6
 
 3597
 
 island
 
 0.04899
 
 0.16481
 
 Not queried
 
  1.4572587
 
 1.80e-01
 
 1.00e+00
  
 
  
 17
 
  21454528
 
  21455185
 
  PDF   STATS   ENSEMBL   UCSC 
 
  658
 
  7
 
 18092
 
 island
 
 0.02385
 
 0.07588
 
 Not queried
 
  1.4572424
 
 2.59e-01
 
 1.00e+00
  
 
  
 15
 
  78914083
 
  78914439
 
  PDF   STATS   ENSEMBL   UCSC 
 
  357
 
  4
 
 13809
 
 tss;island
 
 0.85091
 
 0.62488
 
 Not queried
 
  1.4570651
 
 1.14e-01
 
 1.00e+00
  
 
  
 14
 
  45553114
 
  45553636
 
  PDF   STATS   ENSEMBL   UCSC 
 
  523
 
  5
 
 12785
 
 island
 
 0.01808
 
 0.03878
 
 Not queried
 
  1.4569725
 
 5.56e-02
 
 1.00e+00
  
 
  
 18
 
  19284796
 
  19285051
 
  PDF   STATS   ENSEMBL   UCSC 
 
  256
 
  9
 
 19166
 
 tss
 
 0.03099
 
 0.10698
 
 Not queried
 
  1.4566583
 
 1.13e-01
 
 1.00e+00
  
 
  
 16
 
   1365584
 
   1365915
 
  PDF   STATS   ENSEMBL   UCSC 
 
  332
 
  4
 
 14533
 
 gene;island
 
 0.73439
 
 0.89039
 
 Not queried
 
  1.4564708
 
 4.86e-01
 
 1.00e+00
  
 
  
 20
 
  62037503
 
  62037758
 
  PDF   STATS   ENSEMBL   UCSC 
 
  256
 
  4
 
 26311
 
 island
 
 0.81749
 
 0.92678
 
 Not queried
 
  1.4560090
 
 1.14e-01
 
 1.00e+00
  
 
  
 12
 
 114847438
 
 114847641
 
  PDF   STATS   ENSEMBL   UCSC 
 
  204
 
  4
 
 10010
 
 tss;island
 
 0.65839
 
 0.34937
 
 Not queried
 
  1.4559226
 
 1.14e-01
 
 1.00e+00
  
 
  
 3
 
  62355884
 
  62356323
 
  PDF   STATS   ENSEMBL   UCSC 
 
  440
 
  4
 
 27803
 
 gene;island
 
 0.15572
 
 0.13585
 
 Not queried
 
  1.4558722
 
 8.86e-01
 
 1.00e+00
  
 
  
 17
 
  13505433
 
  13505799
 
  PDF   STATS   ENSEMBL   UCSC 
 
  367
 
  5
 
 17111
 
 tss;island
 
 0.02710
 
 0.02579
 
 Not queried
 
  1.4557440
 
 5.48e-01
 
 1.00e+00
  
 
  
 17
 
    185102
 
    185263
 
  PDF   STATS   ENSEMBL   UCSC 
 
  162
 
  4
 
 16256
 
 gene;island
 
 0.93567
 
 0.85698
 
 Not queried
 
  1.4556736
 
 3.43e-01
 
 1.00e+00
  
 
  
 17
 
  62084205
 
  62084359
 
  PDF   STATS   ENSEMBL   UCSC 
 
  155
 
  5
 
 16696
 
 gene
 
 0.43233
 
 0.62125
 
 Not queried
 
  1.4536445
 
 2.22e-01
 
 1.00e+00
  
 
  
 15
 
  64387625
 
  64388259
 
  PDF   STATS   ENSEMBL   UCSC 
 
  635
 
  9
 
 14183
 
 island
 
 0.00997
 
 0.02142
 
 Not queried
 
  1.4534917
 
 2.97e-01
 
 1.00e+00
  
 
  
 14
 
  21737751
 
  21738093
 
  PDF   STATS   ENSEMBL   UCSC 
 
  343
 
  6
 
 12254
 
 tss;island
 
 0.00618
 
 0.02059
 
 Not queried
 
  1.4527934
 
 1.52e-02
 
 1.00e+00
  
 
  
 8
 
 122654023
 
 122654328
 
  PDF   STATS   ENSEMBL   UCSC 
 
  306
 
  4
 
 39352
 
 gene;tss;island
 
 0.01526
 
 0.04143
 
 Not queried
 
  1.4524899
 
 3.43e-01
 
 1.00e+00
  
 
  
 16
 
  12997467
 
  12997684
 
  PDF   STATS   ENSEMBL   UCSC 
 
  218
 
  4
 
 14643
 
 gene;island
 
 0.04632
 
 0.14085
 
 Not queried
 
  1.4524885
 
 3.43e-01
 
 1.00e+00
  
 
  
 16
 
  50875140
 
  50875496
 
  PDF   STATS   ENSEMBL   UCSC 
 
  357
 
  4
 
 15883
 
 island
 
 0.25802
 
 0.56406
 
 Not queried
 
  1.4523892
 
 2.00e-01
 
 1.00e+00
  
 
  
 6
 
  32826171
 
  32826281
 
  PDF   STATS   ENSEMBL   UCSC 
 
  111
 
  4
 
 34094
 
 gene;island
 
 0.74542
 
 0.55004
 
 Not queried
 
  1.4521234
 
 4.86e-01
 
 1.00e+00
  
 
  
 15
 
  69323876
 
  69324156
 
  PDF   STATS   ENSEMBL   UCSC 
 
  281
 
  5
 
 13368
 
 gene;island
 
 0.02556
 
 0.06696
 
 Not queried
 
  1.4518642
 
 3.10e-01
 
 1.00e+00
  
 
  
 19
 
  14224992
 
  14225172
 
  PDF   STATS   ENSEMBL   UCSC 
 
  181
 
  5
 
 19855
 
 gene;tss;island
 
 0.56310
 
 0.78905
 
 Not queried
 
  1.4517689
 
 9.52e-02
 
 1.00e+00
  
 
  
 17
 
  53315783
 
  53316308
 
  PDF   STATS   ENSEMBL   UCSC 
 
  526
 
  5
 
 18540
 
 island
 
 0.00488
 
 0.01716
 
 Not queried
 
  1.4517422
 
 4.21e-01
 
 1.00e+00
  
 
  
 2
 
 216177085
 
 216177303
 
  PDF   STATS   ENSEMBL   UCSC 
 
  219
 
  4
 
 22970
 
 gene;island
 
 0.08090
 
 0.03906
 
 Not queried
 
  1.4517317
 
 5.71e-02
 
 1.00e+00
  
 
  
 4
 
   3517779
 
   3518121
 
  PDF   STATS   ENSEMBL   UCSC 
 
  343
 
  4
 
 29866
 
 gene;island
 
 0.84293
 
 0.93912
 
 Not queried
 
  1.4515441
 
 3.43e-01
 
 1.00e+00
  
 
  
 15
 
  41805868
 
  41806234
 
  PDF   STATS   ENSEMBL   UCSC 
 
  367
 
  5
 
 14035
 
 island
 
 0.51567
 
 0.29162
 
 Not queried
 
  1.4513616
 
 1.51e-01
 
 1.00e+00
  
 
  
 15
 
  58357922
 
  58357989
 
  PDF   STATS   ENSEMBL   UCSC 
 
   68
 
  4
 
 13669
 
 tss
 
 0.01209
 
 0.03123
 
 Not queried
 
  1.4513532
 
 2.00e-01
 
 1.00e+00
  
 
  
 15
 
  53086630
 
  53087599
 
  PDF   STATS   ENSEMBL   UCSC 
 
  970
 
  9
 
 14125
 
 island
 
 0.90753
 
 0.76673
 
 Not queried
 
  1.4508228
 
 1.06e-02
 
 1.00e+00
  
 
  
 1
 
 247578859
 
 247579312
 
  PDF   STATS   ENSEMBL   UCSC 
 
  454
 
  4
 
 2364
 
 tss
 
 0.53125
 
 0.75511
 
 Not queried
 
  1.4504166
 
 1.14e-01
 
 1.00e+00
  
 
  
 22
 
  37252282
 
  37252652
 
  PDF   STATS   ENSEMBL   UCSC 
 
  371
 
  4
 
 27417
 
 island
 
 0.08612
 
 0.18275
 
 Not queried
 
  1.4500479
 
 6.86e-01
 
 1.00e+00
  
 
  
 1
 
 206730120
 
 206730453
 
  PDF   STATS   ENSEMBL   UCSC 
 
  334
 
  4
 
 2210
 
 tss
 
 0.08558
 
 0.24781
 
 Not queried
 
  1.4499547
 
 4.86e-01
 
 1.00e+00
  
 
  
 17
 
  73892982
 
  73893209
 
  PDF   STATS   ENSEMBL   UCSC 
 
  228
 
  6
 
 18732
 
 island
 
 0.05004
 
 0.09266
 
 Not queried
 
  1.4498493
 
 9.31e-02
 
 1.00e+00
  
 
  
 1
 
  33937968
 
  33938226
 
  PDF   STATS   ENSEMBL   UCSC 
 
  259
 
  7
 
 1471
 
 tss
 
 0.00948
 
 0.02547
 
 Not queried
 
  1.4497976
 
 1.75e-02
 
 1.00e+00
  
 
  
 8
 
    786658
 
    787298
 
  PDF   STATS   ENSEMBL   UCSC 
 
  641
 
  6
 
 39942
 
 island
 
 0.88397
 
 0.95430
 
 Not queried
 
  1.4497626
 
 1.80e-01
 
 1.00e+00
  
 
  
 10
 
  47083283
 
  47083632
 
  PDF   STATS   ENSEMBL   UCSC 
 
  350
 
  9
 
 5701
 
 island
 
 0.44558
 
 0.64180
 
 Not queried
 
  1.4497529
 
 1.23e-03
 
 1.00e+00
  
 
  
 X
 
  51149400
 
  51149742
 
  PDF   STATS   ENSEMBL   UCSC 
 
  343
 
  5
 
 41324
 
 tss;island
 
 0.76410
 
 0.91653
 
 Not queried
 
  1.4494967
 
 5.48e-01
 
 1.00e+00
  
 
  
 6
 
  30121774
 
  30122593
 
  PDF   STATS   ENSEMBL   UCSC 
 
  820
 
 18
 
 33666
 
 gene
 
 0.35700
 
 0.57667
 
 Not queried
 
  1.4485233
 
 9.86e-04
 
 1.00e+00
  
 
  
 3
 
  13520685
 
  13521915
 
  PDF   STATS   ENSEMBL   UCSC 
 
 1231
 
 27
 
 28859
 
 island
 
 0.07325
 
 0.03231
 
 Not queried
 
  1.4480794
 
 2.37e-02
 
 1.00e+00
  
 
  
 2
 
 219925246
 
 219925330
 
  PDF   STATS   ENSEMBL   UCSC 
 
   85
 
  6
 
 23852
 
 tss
 
 0.02873
 
 0.09665
 
 Not queried
 
  1.4477798
 
 4.11e-02
 
 1.00e+00
  
 
  
 4
 
 185654506
 
 185654737
 
  PDF   STATS   ENSEMBL   UCSC 
 
  232
 
  5
 
 30158
 
 gene;island
 
 0.01558
 
 0.03568
 
 Not queried
 
  1.4475375
 
 2.22e-01
 
 1.00e+00
  
 
  
 1
 
 207669003
 
 207669334
 
  PDF   STATS   ENSEMBL   UCSC 
 
  332
 
  5
 
 2219
 
 tss;island
 
 0.39020
 
 0.35611
 
 Not queried
 
  1.4474403
 
 6.90e-01
 
 1.00e+00
  
 
  
 3
 
  30936239
 
  30936531
 
  PDF   STATS   ENSEMBL   UCSC 
 
  293
 
  8
 
 28159
 
 tss
 
 0.02652
 
 0.08838
 
 Not queried
 
  1.4474279
 
 2.81e-02
 
 1.00e+00
  
 
  
 19
 
  36236496
 
  36236615
 
  PDF   STATS   ENSEMBL   UCSC 
 
  120
 
  4
 
 19956
 
 gene
 
 0.00821
 
 0.00843
 
 Not queried
 
  1.4468988
 
 6.86e-01
 
 1.00e+00
  
 
  
 6
 
  31543169
 
  31543300
 
  PDF   STATS   ENSEMBL   UCSC 
 
  132
 
  4
 
 34755
 
 tss
 
 0.37548
 
 0.61634
 
 Not queried
 
  1.4465768
 
 5.71e-02
 
 1.00e+00
  
 
  
 16
 
  30457273
 
  30457524
 
  PDF   STATS   ENSEMBL   UCSC 
 
  252
 
  9
 
 15154
 
 tss
 
 0.02242
 
 0.03028
 
 Not queried
 
  1.4465252
 
 9.31e-01
 
 1.00e+00
  
 
  
 19
 
  40562144
 
  40562354
 
  PDF   STATS   ENSEMBL   UCSC 
 
  211
 
  5
 
 20744
 
 tss
 
 0.02776
 
 0.07446
 
 Not queried
 
  1.4464152
 
 1.51e-01
 
 1.00e+00
  
 
  
 16
 
  22824877
 
  22825282
 
  PDF   STATS   ENSEMBL   UCSC 
 
  406
 
  5
 
 15102
 
 tss;island
 
 0.04120
 
 0.08208
 
 Not queried
 
  1.4459901
 
 9.52e-02
 
 1.00e+00
  
 
  
 11
 
  62621178
 
  62621406
 
  PDF   STATS   ENSEMBL   UCSC 
 
  229
 
  4
 
 6631
 
 gene;tss;island
 
 0.22696
 
 0.15661
 
 Not queried
 
  1.4459052
 
 3.43e-01
 
 1.00e+00
  
 
  
 1
 
 184006765
 
 184007101
 
  PDF   STATS   ENSEMBL   UCSC 
 
  337
 
  5
 
 4024
 
 island
 
 0.13134
 
 0.05260
 
 Not queried
 
  1.4454085
 
 2.22e-01
 
 1.00e+00
  
 
  
 2
 
 101889574
 
 101889754
 
  PDF   STATS   ENSEMBL   UCSC 
 
  181
 
  5
 
 23508
 
 tss
 
 0.70244
 
 0.86533
 
 Not queried
 
  1.4445996
 
 7.94e-03
 
 1.00e+00
  
 
  
 7
 
  44104860
 
  44105434
 
  PDF   STATS   ENSEMBL   UCSC 
 
  575
 
  6
 
 38390
 
 island
 
 0.23793
 
 0.36151
 
 Not queried
 
  1.4445800
 
 6.49e-02
 
 1.00e+00
  
 
  
 6
 
  29573014
 
  29573377
 
  PDF   STATS   ENSEMBL   UCSC 
 
  364
 
  5
 
 33603
 
 gene
 
 0.68494
 
 0.91528
 
 Not queried
 
  1.4444870
 
 3.17e-02
 
 1.00e+00
  
 
  
 5
 
 141293785
 
 141294040
 
  PDF   STATS   ENSEMBL   UCSC 
 
  256
 
  4
 
 33200
 
 island
 
 0.15691
 
 0.05869
 
 Not queried
 
  1.4443129
 
 1.14e-01
 
 1.00e+00
  
 
  
 10
 
  99257444
 
  99257699
 
  PDF   STATS   ENSEMBL   UCSC 
 
  256
 
  4
 
 4682
 
 gene;tss;island
 
 0.01580
 
 0.04163
 
 Not queried
 
  1.4441117
 
 2.86e-02
 
 1.00e+00
  
 
  
 18
 
  34340585
 
  34340941
 
  PDF   STATS   ENSEMBL   UCSC 
 
  357
 
  4
 
 19059
 
 gene;island
 
 0.79698
 
 0.90858
 
 Not queried
 
  1.4439749
 
 2.00e-01
 
 1.00e+00
  
 
  
 7
 
   5390491
 
   5390564
 
  PDF   STATS   ENSEMBL   UCSC 
 
   74
 
  4
 
 36808
 
 gene;island
 
 0.85203
 
 0.93806
 
 Not queried
 
  1.4437595
 
 2.86e-02
 
 1.00e+00
  
 
  
 5
 
  72746710
 
  72746990
 
  PDF   STATS   ENSEMBL   UCSC 
 
  281
 
  4
 
 32811
 
 island
 
 0.03722
 
 0.06047
 
 Not queried
 
  1.4433928
 
 6.86e-01
 
 1.00e+00
  
 
  
 4
 
 104641033
 
 104641319
 
  PDF   STATS   ENSEMBL   UCSC 
 
  287
 
  6
 
 30479
 
 tss;island
 
 0.09318
 
 0.03873
 
 Not queried
 
  1.4427321
 
 1.52e-02
 
 1.00e+00
  
 
  
 12
 
 133007675
 
 133008035
 
  PDF   STATS   ENSEMBL   UCSC 
 
  361
 
  5
 
 11025
 
 island
 
 0.67626
 
 0.83635
 
 Not queried
 
  1.4420736
 
 4.21e-01
 
 1.00e+00
  
 
  
 17
 
  37843793
 
  37844289
 
  PDF   STATS   ENSEMBL   UCSC 
 
  497
 
  8
 
 16516
 
 gene
 
 0.04468
 
 0.01900
 
 Not queried
 
  1.4419523
 
 4.99e-02
 
 1.00e+00
  
 
  
 13
 
  47371267
 
  47371644
 
  PDF   STATS   ENSEMBL   UCSC 
 
  378
 
 11
 
 11689
 
 island
 
 0.04706
 
 0.09779
 
 Not queried
 
  1.4418483
 
 5.57e-02
 
 1.00e+00
  
 
  
 17
 
  46871708
 
  46871905
 
  PDF   STATS   ENSEMBL   UCSC 
 
  198
 
  5
 
 17440
 
 tss
 
 0.78524
 
 0.57375
 
 Not queried
 
  1.4417907
 
 3.10e-01
 
 1.00e+00
  
 
  
 16
 
  31538556
 
  31538946
 
  PDF   STATS   ENSEMBL   UCSC 
 
  391
 
  4
 
 15183
 
 tss
 
 0.71673
 
 0.68431
 
 Not queried
 
  1.4416960
 
 8.86e-01
 
 1.00e+00
  
 
  
 X
 
 107334429
 
 107334815
 
  PDF   STATS   ENSEMBL   UCSC 
 
  387
 
  5
 
 41073
 
 gene
 
 0.00773
 
 0.03342
 
 Not queried
 
  1.4416577
 
 3.10e-01
 
 1.00e+00
  
 
  
 4
 
  74702421
 
  74702728
 
  PDF   STATS   ENSEMBL   UCSC 
 
  308
 
  5
 
 29976
 
 gene;island
 
 0.11425
 
 0.07065
 
 Not queried
 
  1.4406731
 
 1.51e-01
 
 1.00e+00
  
 
  
 10
 
 102988831
 
 102988889
 
  PDF   STATS   ENSEMBL   UCSC 
 
   59
 
  4
 
 5288
 
 tss;island
 
 0.04166
 
 0.13753
 
 Not queried
 
  1.4405725
 
 4.86e-01
 
 1.00e+00
  
 
  
 19
 
  49375485
 
  49375640
 
  PDF   STATS   ENSEMBL   UCSC 
 
  156
 
  4
 
 20893
 
 tss
 
 0.01833
 
 0.03922
 
 Not queried
 
  1.4399818
 
 4.86e-01
 
 1.00e+00
  
 
  
 1
 
 162838649
 
 162838907
 
  PDF   STATS   ENSEMBL   UCSC 
 
  259
 
  4
 
 2056
 
 tss
 
 0.48754
 
 0.71734
 
 Not queried
 
  1.4395084
 
 1.14e-01
 
 1.00e+00
  
 
  
 6
 
  26247238
 
  26247270
 
  PDF   STATS   ENSEMBL   UCSC 
 
   33
 
  4
 
 34636
 
 tss
 
 0.88934
 
 0.94416
 
 Not queried
 
  1.4392613
 
 3.43e-01
 
 1.00e+00
  
 
  
 2
 
 223160102
 
 223160569
 
  PDF   STATS   ENSEMBL   UCSC 
 
  468
 
  5
 
 23008
 
 gene;island
 
 0.12099
 
 0.32928
 
 Not queried
 
  1.4389886
 
 7.94e-03
 
 1.00e+00
  
 
  
 4
 
  48486087
 
  48486472
 
  PDF   STATS   ENSEMBL   UCSC 
 
  386
 
  4
 
 29954
 
 gene;island
 
 0.45160
 
 0.21723
 
 Not queried
 
  1.4381518
 
 2.86e-02
 
 1.00e+00
  
 
  
 12
 
  89919186
 
  89919388
 
  PDF   STATS   ENSEMBL   UCSC 
 
  203
 
  4
 
 9214
 
 gene;tss;island
 
 0.01469
 
 0.04208
 
 Not queried
 
  1.4380672
 
 6.86e-01
 
 1.00e+00
  
 
  
 19
 
   5250376
 
   5250655
 
  PDF   STATS   ENSEMBL   UCSC 
 
  280
 
  4
 
 19714
 
 gene;island
 
 0.70464
 
 0.86294
 
 Not queried
 
  1.4379711
 
 2.00e-01
 
 1.00e+00
  
 
  
 5
 
 140792511
 
 140792992
 
  PDF   STATS   ENSEMBL   UCSC 
 
  482
 
  8
 
 31775
 
 gene;island
 
 0.01845
 
 0.12484
 
 Not queried
 
  1.4377608
 
 6.50e-02
 
 1.00e+00
  
 
  
 X
 
  48366824
 
  48367230
 
  PDF   STATS   ENSEMBL   UCSC 
 
  407
 
  9
 
 41290
 
 tss;island
 
 0.09070
 
 0.01885
 
 Not queried
 
  1.4374218
 
 3.15e-02
 
 1.00e+00
  
 
  
 19
 
    859330
 
    859680
 
  PDF   STATS   ENSEMBL   UCSC 
 
  351
 
  6
 
 21144
 
 island
 
 0.78725
 
 0.62644
 
 Not queried
 
  1.4368605
 
 2.16e-03
 
 1.00e+00
  
 
  
 16
 
   1451278
 
   1451656
 
  PDF   STATS   ENSEMBL   UCSC 
 
  379
 
  4
 
 14541
 
 gene;island
 
 0.80194
 
 0.88407
 
 Not queried
 
  1.4363950
 
 4.86e-01
 
 1.00e+00
  
 
  
 8
 
    914335
 
    914572
 
  PDF   STATS   ENSEMBL   UCSC 
 
  238
 
  5
 
 39945
 
 island
 
 0.82414
 
 0.95893
 
 Not queried
 
  1.4363019
 
 5.56e-02
 
 1.00e+00
  
 
  
 8
 
 103666796
 
 103667415
 
  PDF   STATS   ENSEMBL   UCSC 
 
  620
 
  5
 
 39324
 
 gene;tss;island
 
 0.02520
 
 0.05453
 
 Not queried
 
  1.4359152
 
 1.51e-01
 
 1.00e+00
  
 
  
 1
 
 156549118
 
 156549431
 
  PDF   STATS   ENSEMBL   UCSC 
 
  314
 
  4
 
 2013
 
 tss
 
 0.61888
 
 0.40737
 
 Not queried
 
  1.4357709
 
 1.14e-01
 
 1.00e+00
  
 
  
 X
 
   2835913
 
   2836300
 
  PDF   STATS   ENSEMBL   UCSC 
 
  388
 
  6
 
 40924
 
 gene;island
 
 0.82366
 
 0.90706
 
 Not queried
 
  1.4348922
 
 1.32e-01
 
 1.00e+00
  
 
  
 18
 
  19284713
 
  19285051
 
  PDF   STATS   ENSEMBL   UCSC 
 
  339
 
 10
 
 19385
 
 island
 
 0.02713
 
 0.09384
 
 Not queried
 
  1.4342215
 
 8.92e-02
 
 1.00e+00
  
 
  
 16
 
  88933068
 
  88933359
 
  PDF   STATS   ENSEMBL   UCSC 
 
  292
 
  4
 
 15387
 
 tss;island
 
 0.46784
 
 0.67263
 
 Not queried
 
  1.4340384
 
 2.00e-01
 
 1.00e+00
  
 
  
 8
 
 145848921
 
 145849607
 
  PDF   STATS   ENSEMBL   UCSC 
 
  687
 
  8
 
 40679
 
 island
 
 0.81355
 
 0.93647
 
 Not queried
 
  1.4339814
 
 2.34e-01
 
 1.00e+00
  
 
  
 13
 
 113759771
 
 113760101
 
  PDF   STATS   ENSEMBL   UCSC 
 
  331
 
  5
 
 11519
 
 tss
 
 0.73379
 
 0.87368
 
 Not queried
 
  1.4337385
 
 2.22e-01
 
 1.00e+00
  
 
  
 13
 
  84455525
 
  84455742
 
  PDF   STATS   ENSEMBL   UCSC 
 
  218
 
  4
 
 11157
 
 gene;island
 
 0.04822
 
 0.13382
 
 Not queried
 
  1.4334314
 
 4.86e-01
 
 1.00e+00
  
 
  
 2
 
  81694296
 
  81694608
 
  PDF   STATS   ENSEMBL   UCSC 
 
  313
 
  5
 
 24428
 
 island
 
 0.94249
 
 0.86369
 
 Not queried
 
  1.4327164
 
 9.52e-02
 
 1.00e+00
  
 
  
 6
 
  32808504
 
  32808918
 
  PDF   STATS   ENSEMBL   UCSC 
 
  415
 
  7
 
 34082
 
 gene;island
 
 0.75633
 
 0.50647
 
 Not queried
 
  1.4326989
 
 1.28e-01
 
 1.00e+00
  
 
  
 12
 
  42681744
 
  42682194
 
  PDF   STATS   ENSEMBL   UCSC 
 
  451
 
  5
 
 10304
 
 island
 
 0.86049
 
 0.90788
 
 Not queried
 
  1.4324418
 
 3.10e-01
 
 1.00e+00
  
 
  
 14
 
  24457893
 
  24458345
 
  PDF   STATS   ENSEMBL   UCSC 
 
  453
 
 10
 
 12695
 
 island
 
 0.07751
 
 0.17684
 
 Not queried
 
  1.4324403
 
 2.80e-01
 
 1.00e+00
  
 
  
 4
 
 186741788
 
 186742139
 
  PDF   STATS   ENSEMBL   UCSC 
 
  352
 
  5
 
 30166
 
 gene
 
 0.73729
 
 0.88006
 
 Not queried
 
  1.4323741
 
 1.59e-02
 
 1.00e+00
  
 
  
 X
 
  69396942
 
  69397273
 
  PDF   STATS   ENSEMBL   UCSC 
 
  332
 
  5
 
 41365
 
 tss
 
 0.25984
 
 0.73113
 
 Not queried
 
  1.4323353
 
 1.51e-01
 
 1.00e+00
  
 
  
 X
 
 130192035
 
 130192365
 
  PDF   STATS   ENSEMBL   UCSC 
 
  331
 
  7
 
 42030
 
 island
 
 0.21880
 
 0.08451
 
 Not queried
 
  1.4322348
 
 2.62e-02
 
 1.00e+00
  
 
  
 7
 
    193229
 
    193732
 
  PDF   STATS   ENSEMBL   UCSC 
 
  504
 
  5
 
 36639
 
 gene;island
 
 0.04116
 
 0.04583
 
 Not queried
 
  1.4319066
 
 3.10e-01
 
 1.00e+00
  
 
  
 11
 
  67188491
 
  67188871
 
  PDF   STATS   ENSEMBL   UCSC 
 
  381
 
  5
 
 6736
 
 gene;island
 
 0.01090
 
 0.01170
 
 Not queried
 
  1.4314936
 
 5.48e-01
 
 1.00e+00
  
 
  
 1
 
  27718858
 
  27719045
 
  PDF   STATS   ENSEMBL   UCSC 
 
  188
 
  4
 
 1408
 
 tss;island
 
 0.01632
 
 0.01032
 
 Not queried
 
  1.4309093
 
 3.43e-01
 
 1.00e+00
  
 
  
 10
 
  96305598
 
  96305691
 
  PDF   STATS   ENSEMBL   UCSC 
 
   94
 
  4
 
 4672
 
 gene
 
 0.01680
 
 0.03120
 
 Not queried
 
  1.4306951
 
 3.43e-01
 
 1.00e+00
  
 
  
 10
 
 120354627
 
 120354870
 
  PDF   STATS   ENSEMBL   UCSC 
 
  244
 
  4
 
 4775
 
 gene;island
 
 0.35675
 
 0.68530
 
 Not queried
 
  1.4305206
 
 2.00e-01
 
 1.00e+00
  
 
  
 14
 
 105616988
 
 105617308
 
  PDF   STATS   ENSEMBL   UCSC 
 
  321
 
  4
 
 12228
 
 gene;island
 
 0.67109
 
 0.84789
 
 Not queried
 
  1.4302527
 
 4.86e-01
 
 1.00e+00
  
 
  
 6
 
  11044877
 
  11044974
 
  PDF   STATS   ENSEMBL   UCSC 
 
   98
 
  4
 
 34569
 
 tss;island
 
 0.01431
 
 0.03383
 
 Not queried
 
  1.4302147
 
 3.43e-01
 
 1.00e+00
  
 
  
 3
 
  55522611
 
  55522835
 
  PDF   STATS   ENSEMBL   UCSC 
 
  225
 
  5
 
 29173
 
 island
 
 0.00784
 
 0.02085
 
 Not queried
 
  1.4301348
 
 4.21e-01
 
 1.00e+00
  
 
  
 2
 
  73053217
 
  73053592
 
  PDF   STATS   ENSEMBL   UCSC 
 
  376
 
  8
 
 23395
 
 tss;island
 
 0.06281
 
 0.02694
 
 Not queried
 
  1.4298336
 
 5.74e-01
 
 1.00e+00
  
 
  
 13
 
  36050844
 
  36051073
 
  PDF   STATS   ENSEMBL   UCSC 
 
  230
 
  7
 
 11362
 
 tss
 
 0.03226
 
 0.14117
 
 Not queried
 
  1.4295819
 
 2.59e-01
 
 1.00e+00
  
 
  
 22
 
  18507401
 
  18507747
 
  PDF   STATS   ENSEMBL   UCSC 
 
  347
 
  5
 
 26905
 
 tss;island
 
 0.02724
 
 0.19864
 
 Not queried
 
  1.4290769
 
 2.22e-01
 
 1.00e+00
  
 
  
 1
 
 244213358
 
 244213618
 
  PDF   STATS   ENSEMBL   UCSC 
 
  261
 
  5
 
 2350
 
 tss;island
 
 0.07411
 
 0.04213
 
 Not queried
 
  1.4278693
 
 3.17e-02
 
 1.00e+00
  
 
  
 7
 
 100484435
 
 100484748
 
  PDF   STATS   ENSEMBL   UCSC 
 
  314
 
  4
 
 37087
 
 gene;island
 
 0.76498
 
 0.89397
 
 Not queried
 
  1.4278433
 
 4.86e-01
 
 1.00e+00
  
 
  
 15
 
  25307169
 
  25307422
 
  PDF   STATS   ENSEMBL   UCSC 
 
  254
 
  4
 
 13493
 
 tss
 
 0.92013
 
 0.97126
 
 Not queried
 
  1.4276025
 
 3.43e-01
 
 1.00e+00
  
 
  
 6
 
  41339246
 
  41340058
 
  PDF   STATS   ENSEMBL   UCSC 
 
  813
 
  7
 
 36057
 
 island
 
 0.04578
 
 0.09848
 
 Not queried
 
  1.4274298
 
 5.30e-02
 
 1.00e+00
  
 
  
 18
 
  35145353
 
  35145650
 
  PDF   STATS   ENSEMBL   UCSC 
 
  298
 
  4
 
 19062
 
 gene;island
 
 0.02860
 
 0.03097
 
 Not queried
 
  1.4273296
 
 4.86e-01
 
 1.00e+00
  
 
  
 4
 
   7436693
 
   7437156
 
  PDF   STATS   ENSEMBL   UCSC 
 
  464
 
  6
 
 29892
 
 gene
 
 0.42404
 
 0.65598
 
 Not queried
 
  1.4272735
 
 1.52e-02
 
 1.00e+00
  
 
  
 4
 
  40751717
 
  40751845
 
  PDF   STATS   ENSEMBL   UCSC 
 
  129
 
  4
 
 30313
 
 tss
 
 0.07757
 
 0.18812
 
 Not queried
 
  1.4272436
 
 4.86e-01
 
 1.00e+00
  
 
  
 4
 
  26492153
 
  26492488
 
  PDF   STATS   ENSEMBL   UCSC 
 
  336
 
  6
 
 30290
 
 tss
 
 0.60177
 
 0.69053
 
 Not queried
 
  1.4270257
 
 3.94e-01
 
 1.00e+00
  
 
  
 20
 
  30060822
 
  30061044
 
  PDF   STATS   ENSEMBL   UCSC 
 
  223
 
  5
 
 25619
 
 tss;island
 
 0.70818
 
 0.50025
 
 Not queried
 
  1.4267433
 
 7.94e-03
 
 1.00e+00
  
 
  
 7
 
 157414294
 
 157414580
 
  PDF   STATS   ENSEMBL   UCSC 
 
  287
 
  4
 
 37270
 
 gene;island
 
 0.89141
 
 0.96828
 
 Not queried
 
  1.4265562
 
 4.86e-01
 
 1.00e+00
  
 
  
 12
 
  54388841
 
  54389264
 
  PDF   STATS   ENSEMBL   UCSC 
 
  424
 
  5
 
 10461
 
 island
 
 0.02932
 
 0.06992
 
 Not queried
 
  1.4263585
 
 1.51e-01
 
 1.00e+00
  
 
  
 4
 
 145568182
 
 145568399
 
  PDF   STATS   ENSEMBL   UCSC 
 
  218
 
  4
 
 30096
 
 gene;island
 
 0.06739
 
 0.20368
 
 Not queried
 
  1.4259399
 
 3.43e-01
 
 1.00e+00
  
 
  
 3
 
 123699145
 
 123699389
 
  PDF   STATS   ENSEMBL   UCSC 
 
  245
 
  4
 
 27878
 
 gene
 
 0.07541
 
 0.18640
 
 Not queried
 
  1.4258934
 
 4.86e-01
 
 1.00e+00
  
 
  
 19
 
  58446312
 
  58446988
 
  PDF   STATS   ENSEMBL   UCSC 
 
  677
 
 12
 
 22450
 
 island
 
 0.03024
 
 0.06135
 
 Not queried
 
  1.4252812
 
 1.73e-02
 
 1.00e+00
  
 
  
 14
 
  47120795
 
  47121002
 
  PDF   STATS   ENSEMBL   UCSC 
 
  208
 
  4
 
 12047
 
 gene
 
 0.67174
 
 0.84560
 
 Not queried
 
  1.4251713
 
 1.14e-01
 
 1.00e+00
  
 
  
 7
 
   4218743
 
   4219174
 
  PDF   STATS   ENSEMBL   UCSC 
 
  432
 
  4
 
 36770
 
 gene
 
 0.75999
 
 0.87313
 
 Not queried
 
  1.4247998
 
 5.71e-02
 
 1.00e+00
  
 
  
 10
 
 102989254
 
 102989376
 
  PDF   STATS   ENSEMBL   UCSC 
 
  123
 
  8
 
 5289
 
 tss;island
 
 0.03783
 
 0.06339
 
 Not queried
 
  1.4238104
 
 1.95e-01
 
 1.00e+00
  
 
  
 19
 
  33571484
 
  33571768
 
  PDF   STATS   ENSEMBL   UCSC 
 
  285
 
  4
 
 20645
 
 tss
 
 0.29024
 
 0.11429
 
 Not queried
 
  1.4234194
 
 3.43e-01
 
 1.00e+00
  
 
  
 1
 
 228403127
 
 228403515
 
  PDF   STATS   ENSEMBL   UCSC 
 
  389
 
  5
 
 1047
 
 gene;island
 
 0.79424
 
 0.91925
 
 Not queried
 
  1.4233289
 
 9.52e-02
 
 1.00e+00
  
 
  
 2
 
  97171137
 
  97171449
 
  PDF   STATS   ENSEMBL   UCSC 
 
  313
 
  5
 
 22744
 
 gene;island
 
 0.52458
 
 0.77508
 
 Not queried
 
  1.4231788
 
 5.56e-02
 
 1.00e+00
  
 
  
 19
 
  44716462
 
  44716642
 
  PDF   STATS   ENSEMBL   UCSC 
 
  181
 
  5
 
 20810
 
 tss
 
 0.01543
 
 0.03443
 
 Not queried
 
  1.4227164
 
 7.94e-03
 
 1.00e+00
  
 
  
 4
 
    949314
 
    949689
 
  PDF   STATS   ENSEMBL   UCSC 
 
  376
 
  4
 
 29805
 
 gene;island
 
 0.85461
 
 0.95628
 
 Not queried
 
  1.4226072
 
 2.00e-01
 
 1.00e+00
  
 
  
 11
 
  65600739
 
  65600888
 
  PDF   STATS   ENSEMBL   UCSC 
 
  150
 
  4
 
 7421
 
 tss;island
 
 0.31783
 
 0.12872
 
 Not queried
 
  1.4221546
 
 2.00e-01
 
 1.00e+00
  
 
  
 18
 
  21017489
 
  21017905
 
  PDF   STATS   ENSEMBL   UCSC 
 
  417
 
  5
 
 19039
 
 gene
 
 0.00835
 
 0.01373
 
 Not queried
 
  1.4220426
 
 4.21e-01
 
 1.00e+00
  
 
  
 14
 
 105964848
 
 105965379
 
  PDF   STATS   ENSEMBL   UCSC 
 
  532
 
  5
 
 12239
 
 gene;island
 
 0.76394
 
 0.92434
 
 Not queried
 
  1.4216048
 
 3.17e-02
 
 1.00e+00
  
 
  
 15
 
  75019070
 
  75019376
 
  PDF   STATS   ENSEMBL   UCSC 
 
  307
 
 10
 
 13772
 
 tss;island
 
 0.81426
 
 0.65842
 
 Not queried
 
  1.4213654
 
 1.43e-01
 
 1.00e+00
  
 
  
 6
 
  29576818
 
  29577348
 
  PDF   STATS   ENSEMBL   UCSC 
 
  531
 
  6
 
 33605
 
 gene
 
 0.72884
 
 0.81977
 
 Not queried
 
  1.4213428
 
 2.40e-01
 
 1.00e+00
  
 
  
 4
 
   2935692
 
   2936284
 
  PDF   STATS   ENSEMBL   UCSC 
 
  593
 
  9
 
 29851
 
 gene;island
 
 0.04493
 
 0.07081
 
 Not queried
 
  1.4212538
 
 1.36e-01
 
 1.00e+00
  
 
  
 8
 
 145162905
 
 145163136
 
  PDF   STATS   ENSEMBL   UCSC 
 
  232
 
  4
 
 39448
 
 gene;island
 
 0.75614
 
 0.92478
 
 Not queried
 
  1.4200394
 
 1.14e-01
 
 1.00e+00
  
 
  
 X
 
  54666063
 
  54666407
 
  PDF   STATS   ENSEMBL   UCSC 
 
  345
 
  4
 
 41815
 
 island
 
 0.13481
 
 0.02773
 
 Not queried
 
  1.4193808
 
 1.14e-01
 
 1.00e+00
  
 
  
 17
 
  79520615
 
  79521089
 
  PDF   STATS   ENSEMBL   UCSC 
 
  475
 
  5
 
 18897
 
 island
 
 0.55868
 
 0.83902
 
 Not queried
 
  1.4182630
 
 2.22e-01
 
 1.00e+00
  
 
  
 X
 
  34150470
 
  34150583
 
  PDF   STATS   ENSEMBL   UCSC 
 
  114
 
  4
 
 41249
 
 tss
 
 0.74881
 
 0.87615
 
 Not queried
 
  1.4181035
 
 2.86e-02
 
 1.00e+00
  
 
  
 1
 
  22140769
 
  22141400
 
  PDF   STATS   ENSEMBL   UCSC 
 
  632
 
  7
 
 300
 
 gene;island
 
 0.23423
 
 0.25952
 
 Not queried
 
  1.4178531
 
 5.35e-01
 
 1.00e+00
  
 
  
 1
 
 111889241
 
 111889533
 
  PDF   STATS   ENSEMBL   UCSC 
 
  293
 
  5
 
 681
 
 gene
 
 0.04068
 
 0.07902
 
 Not queried
 
  1.4176088
 
 5.56e-02
 
 1.00e+00
  
 
  
 22
 
  38902403
 
  38902925
 
  PDF   STATS   ENSEMBL   UCSC 
 
  523
 
  8
 
 27099
 
 tss
 
 0.00946
 
 0.02896
 
 Not queried
 
  1.4175143
 
 1.30e-01
 
 1.00e+00
  
 
  
 22
 
  38902252
 
  38902925
 
  PDF   STATS   ENSEMBL   UCSC 
 
  674
 
 10
 
 27449
 
 island
 
 0.00946
 
 0.02808
 
 Not queried
 
  1.4175143
 
 8.92e-02
 
 1.00e+00
  
 
  
 19
 
  16435203
 
  16435660
 
  PDF   STATS   ENSEMBL   UCSC 
 
  458
 
  5
 
 21681
 
 island
 
 0.02674
 
 0.14748
 
 Not queried
 
  1.4175142
 
 6.90e-01
 
 1.00e+00
  
 
  
 3
 
  46607965
 
  46608179
 
  PDF   STATS   ENSEMBL   UCSC 
 
  215
 
  6
 
 27708
 
 gene;island
 
 0.17890
 
 0.09009
 
 Not queried
 
  1.4174437
 
 1.32e-01
 
 1.00e+00
  
 
  
 8
 
  61590588
 
  61590787
 
  PDF   STATS   ENSEMBL   UCSC 
 
  200
 
  4
 
 39662
 
 tss;island
 
 0.01601
 
 0.06325
 
 Not queried
 
  1.4172662
 
 3.43e-01
 
 1.00e+00
  
 
  
 4
 
 189580331
 
 189580798
 
  PDF   STATS   ENSEMBL   UCSC 
 
  468
 
  5
 
 31438
 
 island
 
 0.75347
 
 0.88658
 
 Not queried
 
  1.4172329
 
 2.22e-01
 
 1.00e+00
  
 
  
 X
 
  73523856
 
  73523965
 
  PDF   STATS   ENSEMBL   UCSC 
 
  110
 
  4
 
 41392
 
 tss
 
 0.77336
 
 0.91533
 
 Not queried
 
  1.4169593
 
 1.14e-01
 
 1.00e+00
  
 
  
 14
 
  37116121
 
  37116408
 
  PDF   STATS   ENSEMBL   UCSC 
 
  288
 
  4
 
 12760
 
 island
 
 0.22969
 
 0.07001
 
 Not queried
 
  1.4164191
 
 3.43e-01
 
 1.00e+00
  
 
  
 11
 
  67206263
 
  67206434
 
  PDF   STATS   ENSEMBL   UCSC 
 
  172
 
  4
 
 6738
 
 gene;tss;island
 
 0.76277
 
 0.90612
 
 Not queried
 
  1.4163825
 
 3.43e-01
 
 1.00e+00
  
 
  
 2
 
 224904626
 
 224904837
 
  PDF   STATS   ENSEMBL   UCSC 
 
  212
 
  5
 
 23886
 
 tss;island
 
 0.13770
 
 0.05613
 
 Not queried
 
  1.4161478
 
 1.51e-01
 
 1.00e+00
  
 
  
 11
 
  64108790
 
  64109158
 
  PDF   STATS   ENSEMBL   UCSC 
 
  369
 
  4
 
 6665
 
 gene;island
 
 0.70825
 
 0.56071
 
 Not queried
 
  1.4159231
 
 6.86e-01
 
 1.00e+00
  
 
  
 18
 
  12656824
 
  12657166
 
  PDF   STATS   ENSEMBL   UCSC 
 
  343
 
  4
 
 19028
 
 gene;tss;island
 
 0.03605
 
 0.03298
 
 Not queried
 
  1.4152000
 
 8.86e-01
 
 1.00e+00
  
 
  
 22
 
  25758455
 
  25758749
 
  PDF   STATS   ENSEMBL   UCSC 
 
  295
 
  5
 
 26780
 
 gene;island
 
 0.15022
 
 0.70438
 
 Not queried
 
  1.4148097
 
 5.48e-01
 
 1.00e+00
  
 
  
 17
 
  40687822
 
  40687943
 
  PDF   STATS   ENSEMBL   UCSC 
 
  122
 
  7
 
 17331
 
 tss
 
 0.04684
 
 0.25767
 
 Not queried
 
  1.4147230
 
 2.59e-01
 
 1.00e+00
  
 
  
 1
 
   3566701
 
   3568245
 
  PDF   STATS   ENSEMBL   UCSC 
 
 1545
 
 34
 
 1179
 
 tss
 
 0.58263
 
 0.40863
 
 Not queried
 
  1.4147130
 
 1.23e-02
 
 1.00e+00
  
 
  
 7
 
  72349822
 
  72349862
 
  PDF   STATS   ENSEMBL   UCSC 
 
   41
 
  4
 
 37565
 
 tss
 
 0.02883
 
 0.01055
 
 Not queried
 
  1.4144093
 
 1.14e-01
 
 1.00e+00
  
 
  
 6
 
  30175792
 
  30176107
 
  PDF   STATS   ENSEMBL   UCSC 
 
  316
 
  5
 
 33688
 
 gene
 
 0.83726
 
 0.91149
 
 Not queried
 
  1.4143135
 
 3.10e-01
 
 1.00e+00
  
 
  
 16
 
  55512806
 
  55513017
 
  PDF   STATS   ENSEMBL   UCSC 
 
  212
 
  5
 
 15208
 
 tss
 
 0.01506
 
 0.02773
 
 Not queried
 
  1.4142113
 
 5.48e-01
 
 1.00e+00
  
 
  
 17
 
  46651822
 
  46651952
 
  PDF   STATS   ENSEMBL   UCSC 
 
  131
 
  5
 
 17425
 
 tss
 
 0.50860
 
 0.27984
 
 Not queried
 
  1.4133384
 
 2.22e-01
 
 1.00e+00
  
 
  
 6
 
  29760856
 
  29761044
 
  PDF   STATS   ENSEMBL   UCSC 
 
  189
 
  6
 
 34691
 
 tss
 
 0.13051
 
 0.26243
 
 Not queried
 
  1.4122905
 
 2.40e-01
 
 1.00e+00
  
 
  
 12
 
 105501526
 
 105501569
 
  PDF   STATS   ENSEMBL   UCSC 
 
   44
 
  4
 
 9250
 
 gene
 
 0.00549
 
 0.01352
 
 Not queried
 
  1.4122379
 
 2.86e-02
 
 1.00e+00
  
 
  
 12
 
 110271439
 
 110271625
 
  PDF   STATS   ENSEMBL   UCSC 
 
  187
 
  4
 
 9971
 
 tss;island
 
 0.04868
 
 0.10333
 
 Not queried
 
  1.4120323
 
 2.00e-01
 
 1.00e+00
  
 
  
 16
 
  56622951
 
  56623549
 
  PDF   STATS   ENSEMBL   UCSC 
 
  599
 
 10
 
 15914
 
 island
 
 0.21636
 
 0.06870
 
 Not queried
 
  1.4116915
 
 4.36e-01
 
 1.00e+00
  
 
  
 1
 
  44456953
 
  44457406
 
  PDF   STATS   ENSEMBL   UCSC 
 
  454
 
  8
 
 3171
 
 island
 
 0.02709
 
 0.04496
 
 Not queried
 
  1.4105737
 
 2.79e-01
 
 1.00e+00
  
 
  
 6
 
  64281439
 
  64281886
 
  PDF   STATS   ENSEMBL   UCSC 
 
  448
 
  8
 
 34990
 
 tss
 
 0.02459
 
 0.01907
 
 Not queried
 
  1.4104693
 
 5.74e-01
 
 1.00e+00
  
 
  
 16
 
   1221825
 
   1222188
 
  PDF   STATS   ENSEMBL   UCSC 
 
  364
 
  4
 
 14528
 
 gene;island
 
 0.70571
 
 0.85786
 
 Not queried
 
  1.4098483
 
 3.43e-01
 
 1.00e+00
  
 
  
 17
 
  80351032
 
  80351457
 
  PDF   STATS   ENSEMBL   UCSC 
 
  426
 
  5
 
 16912
 
 gene;island
 
 0.44913
 
 0.69016
 
 Not queried
 
  1.4096710
 
 9.52e-02
 
 1.00e+00
  
 
  
 16
 
  81348309
 
  81348534
 
  PDF   STATS   ENSEMBL   UCSC 
 
  226
 
  5
 
 15351
 
 tss;island
 
 0.00722
 
 0.01710
 
 Not queried
 
  1.4095086
 
 4.21e-01
 
 1.00e+00
  
 
  
 8
 
 145641991
 
 145642456
 
  PDF   STATS   ENSEMBL   UCSC 
 
  466
 
 10
 
 39918
 
 tss
 
 0.67243
 
 0.85475
 
 Not queried
 
  1.4095036
 
 1.50e-03
 
 1.00e+00
  
 
  
 8
 
  28560467
 
  28560723
 
  PDF   STATS   ENSEMBL   UCSC 
 
  257
 
  5
 
 39199
 
 gene;island
 
 0.30149
 
 0.13347
 
 Not queried
 
  1.4094756
 
 1.51e-01
 
 1.00e+00
  
 
  
 17
 
  53800225
 
  53800554
 
  PDF   STATS   ENSEMBL   UCSC 
 
  330
 
  4
 
 17486
 
 tss
 
 0.04651
 
 0.11638
 
 Not queried
 
  1.4094414
 
 3.43e-01
 
 1.00e+00
  
 
  
 5
 
  63985282
 
  63985489
 
  PDF   STATS   ENSEMBL   UCSC 
 
  208
 
  4
 
 32031
 
 tss;island
 
 0.63241
 
 0.50622
 
 Not queried
 
  1.4088906
 
 4.86e-01
 
 1.00e+00
  
 
  
 11
 
   1096283
 
   1096455
 
  PDF   STATS   ENSEMBL   UCSC 
 
  173
 
  4
 
 6386
 
 gene;island
 
 0.74738
 
 0.88416
 
 Not queried
 
  1.4085167
 
 1.14e-01
 
 1.00e+00
  
 
  
 10
 
 102766322
 
 102766610
 
  PDF   STATS   ENSEMBL   UCSC 
 
  289
 
  4
 
 4705
 
 gene;island
 
 0.74686
 
 0.89770
 
 Not queried
 
  1.4085133
 
 1.14e-01
 
 1.00e+00
  
 
  
 1
 
   3664522
 
   3664680
 
  PDF   STATS   ENSEMBL   UCSC 
 
  159
 
  4
 
 1185
 
 tss;island
 
 0.40335
 
 0.48088
 
 Not queried
 
  1.4084526
 
 6.86e-01
 
 1.00e+00
  
 
  
 3
 
 156544070
 
 156544081
 
  PDF   STATS   ENSEMBL   UCSC 
 
   12
 
  4
 
 28634
 
 tss
 
 0.29215
 
 0.50981
 
 Not queried
 
  1.4081298
 
 2.86e-02
 
 1.00e+00
  
 
  
 15
 
  27111388
 
  27112197
 
  PDF   STATS   ENSEMBL   UCSC 
 
  810
 
 21
 
 13513
 
 tss
 
 0.68389
 
 0.42611
 
 Not queried
 
  1.4081202
 
 1.82e-02
 
 1.00e+00
  
 
  
 1
 
  66258022
 
  66258134
 
  PDF   STATS   ENSEMBL   UCSC 
 
  113
 
  4
 
 1675
 
 tss;island
 
 0.04013
 
 0.10120
 
 Not queried
 
  1.4076656
 
 2.86e-02
 
 1.00e+00
  
 
  
 2
 
 242497960
 
 242498098
 
  PDF   STATS   ENSEMBL   UCSC 
 
  139
 
  4
 
 23953
 
 tss;island
 
 0.18637
 
 0.05883
 
 Not queried
 
  1.4076017
 
 4.86e-01
 
 1.00e+00
  
 
  
 17
 
  19771360
 
  19771813
 
  PDF   STATS   ENSEMBL   UCSC 
 
  454
 
  7
 
 17163
 
 tss;island
 
 0.31615
 
 0.09823
 
 Not queried
 
  1.4074934
 
 3.18e-01
 
 1.00e+00
  
 
  
 2
 
 127414802
 
 127415063
 
  PDF   STATS   ENSEMBL   UCSC 
 
  262
 
  4
 
 22817
 
 gene;island
 
 0.34547
 
 0.15461
 
 Not queried
 
  1.4073746
 
 2.00e-01
 
 1.00e+00
  
 
  
 16
 
  29153044
 
  29153407
 
  PDF   STATS   ENSEMBL   UCSC 
 
  364
 
  5
 
 15767
 
 island
 
 0.43668
 
 0.60692
 
 Not queried
 
  1.4067230
 
 5.56e-02
 
 1.00e+00
  
 
  
 2
 
 129076335
 
 129076394
 
  PDF   STATS   ENSEMBL   UCSC 
 
   60
 
  5
 
 23585
 
 tss;island
 
 0.39514
 
 0.63362
 
 Not queried
 
  1.4064577
 
 5.56e-02
 
 1.00e+00
  
 
  
 5
 
 177581310
 
 177581771
 
  PDF   STATS   ENSEMBL   UCSC 
 
  462
 
  6
 
 32477
 
 tss;island
 
 0.43111
 
 0.67991
 
 Not queried
 
  1.4052356
 
 1.32e-01
 
 1.00e+00
  
 
  
 11
 
   2554198
 
   2554583
 
  PDF   STATS   ENSEMBL   UCSC 
 
  386
 
  6
 
 6444
 
 gene;island
 
 0.27450
 
 0.44230
 
 Not queried
 
  1.4051494
 
 3.94e-01
 
 1.00e+00
  
 
  
 X
 
 154842689
 
 154842856
 
  PDF   STATS   ENSEMBL   UCSC 
 
  168
 
  5
 
 41614
 
 tss
 
 0.05008
 
 0.05370
 
 Not queried
 
  1.4050279
 
 1.00e+00
 
 1.00e+00
  
 
  
 1
 
 153329781
 
 153330257
 
  PDF   STATS   ENSEMBL   UCSC 
 
  477
 
  6
 
 1937
 
 tss
 
 0.66426
 
 0.35543
 
 Not queried
 
  1.4048272
 
 8.66e-03
 
 1.00e+00
  
 
  
 10
 
 118956639
 
 118956998
 
  PDF   STATS   ENSEMBL   UCSC 
 
  360
 
  7
 
 5372
 
 tss
 
 0.81330
 
 0.63496
 
 Not queried
 
  1.4046103
 
 3.79e-02
 
 1.00e+00
  
 
  
 6
 
  30124214
 
  30124299
 
  PDF   STATS   ENSEMBL   UCSC 
 
   86
 
  4
 
 33668
 
 gene
 
 0.61899
 
 0.76132
 
 Not queried
 
  1.4039778
 
 2.00e-01
 
 1.00e+00
  
 
  
 16
 
  58718767
 
  58718971
 
  PDF   STATS   ENSEMBL   UCSC 
 
  205
 
  4
 
 15245
 
 tss
 
 0.64089
 
 0.42357
 
 Not queried
 
  1.4039111
 
 3.43e-01
 
 1.00e+00
  
 
  
 6
 
 142409417
 
 142410235
 
  PDF   STATS   ENSEMBL   UCSC 
 
  819
 
 13
 
 36461
 
 island
 
 0.03230
 
 0.10883
 
 Not queried
 
  1.4038011
 
 1.40e-02
 
 1.00e+00
  
 
  
 13
 
  24734712
 
  24734795
 
  PDF   STATS   ENSEMBL   UCSC 
 
   84
 
  4
 
 11317
 
 tss
 
 0.03471
 
 0.09154
 
 Not queried
 
  1.4032233
 
 1.14e-01
 
 1.00e+00
  
 
  
 11
 
   2151676
 
   2151725
 
  PDF   STATS   ENSEMBL   UCSC 
 
   50
 
  4
 
 6426
 
 gene;island
 
 0.55941
 
 0.85173
 
 Not queried
 
  1.4030605
 
 2.00e-01
 
 1.00e+00
  
 
  
 8
 
 144521374
 
 144521607
 
  PDF   STATS   ENSEMBL   UCSC 
 
  234
 
  7
 
 39414
 
 gene;island
 
 0.85133
 
 0.91565
 
 Not queried
 
  1.4029956
 
 5.30e-02
 
 1.00e+00
  
 
  
 X
 
   9912520
 
   9912880
 
  PDF   STATS   ENSEMBL   UCSC 
 
  361
 
  5
 
 40932
 
 gene;island
 
 0.86385
 
 0.94214
 
 Not queried
 
  1.4028129
 
 2.22e-01
 
 1.00e+00
  
 
  
 3
 
 128444572
 
 128444915
 
  PDF   STATS   ENSEMBL   UCSC 
 
  344
 
  6
 
 28529
 
 tss
 
 0.02198
 
 0.03313
 
 Not queried
 
  1.4021942
 
 5.89e-01
 
 1.00e+00
  
 
  
 5
 
  95768915
 
  95769079
 
  PDF   STATS   ENSEMBL   UCSC 
 
  165
 
  7
 
 32911
 
 island
 
 0.15122
 
 0.02543
 
 Not queried
 
  1.4014086
 
 1.28e-01
 
 1.00e+00
  
 
  
 1
 
   2984245
 
   2984869
 
  PDF   STATS   ENSEMBL   UCSC 
 
  625
 
  9
 
 1175
 
 tss
 
 0.61376
 
 0.81123
 
 Not queried
 
  1.4003995
 
 2.44e-02
 
 1.00e+00
  
 
  
 19
 
   4090224
 
   4090587
 
  PDF   STATS   ENSEMBL   UCSC 
 
  364
 
  4
 
 21328
 
 island
 
 0.71488
 
 0.85581
 
 Not queried
 
  1.4003477
 
 2.86e-02
 
 1.00e+00
  
 
  
 6
 
 168197410
 
 168198163
 
  PDF   STATS   ENSEMBL   UCSC 
 
  754
 
  9
 
 36586
 
 island
 
 0.55468
 
 0.76149
 
 Not queried
 
  1.4003362
 
 1.88e-02
 
 1.00e+00
  
 
  
 8
 
   1094484
 
   1094904
 
  PDF   STATS   ENSEMBL   UCSC 
 
  421
 
  6
 
 39948
 
 island
 
 0.83891
 
 0.91239
 
 Not queried
 
  1.4002161
 
 9.31e-02
 
 1.00e+00
  
 
  
 1
 
  28414998
 
  28415118
 
  PDF   STATS   ENSEMBL   UCSC 
 
  121
 
  5
 
 362
 
 gene
 
 0.01483
 
 0.03493
 
 Not queried
 
  1.4002067
 
 9.52e-02
 
 1.00e+00
  
 
  
 11
 
  19736150
 
  19736333
 
  PDF   STATS   ENSEMBL   UCSC 
 
  184
 
  5
 
 6517
 
 gene;tss;island
 
 0.37189
 
 0.44978
 
 Not queried
 
  1.4001360
 
 1.59e-02
 
 1.00e+00
  
 
  
 11
 
 111383889
 
 111384223
 
  ENSEMBL   UCSC 
 
  335
 
  4
 
 7664
 
 tss;island
 
 0.19829
 
 0.04513
 
 Not queried
 
  1.3995897
 
       NA
 
       NA
  
 
  
 X
 
  50213528
 
  50214061
 
  ENSEMBL   UCSC 
 
  534
 
  8
 
 41796
 
 island
 
 0.13421
 
 0.03430
 
 Not queried
 
  1.3993043
 
       NA
 
       NA
  
 
  
 12
 
  50426531
 
  50427095
 
  ENSEMBL   UCSC 
 
  565
 
  4
 
 10384
 
 island
 
 0.53270
 
 0.33227
 
 Not queried
 
  1.3992573
 
       NA
 
       NA
  
 
  
 2
 
 242878496
 
 242878811
 
  ENSEMBL   UCSC 
 
  316
 
  4
 
 25279
 
 island
 
 0.63005
 
 0.87102
 
 Not queried
 
  1.3991283
 
       NA
 
       NA
  
 
  
 6
 
  26271716
 
  26271827
 
  ENSEMBL   UCSC 
 
  112
 
  4
 
 34638
 
 tss
 
 0.91587
 
 0.96109
 
 Not queried
 
  1.3990178
 
       NA
 
       NA
  
 
  
 X
 
  66765795
 
  66766050
 
  ENSEMBL   UCSC 
 
  256
 
  4
 
 41008
 
 gene;island
 
 0.04421
 
 0.02071
 
 Not queried
 
  1.3987276
 
       NA
 
       NA
  
 
  
 19
 
   3932951
 
   3933195
 
  ENSEMBL   UCSC 
 
  245
 
  5
 
 21317
 
 island
 
 0.08509
 
 0.02617
 
 Not queried
 
  1.3987070
 
       NA
 
       NA
  
 
  
 6
 
  33231268
 
  33231870
 
  ENSEMBL   UCSC 
 
  603
 
  7
 
 34171
 
 gene
 
 0.85926
 
 0.92767
 
 Not queried
 
  1.3985699
 
       NA
 
       NA
  
 
  
 15
 
  42119684
 
  42120091
 
  ENSEMBL   UCSC 
 
  408
 
  6
 
 13583
 
 tss;island
 
 0.05238
 
 0.01753
 
 Not queried
 
  1.3983185
 
       NA
 
       NA
  
 
  
 15
 
  78631878
 
  78632194
 
  ENSEMBL   UCSC 
 
  317
 
  8
 
 13806
 
 tss;island
 
 0.63392
 
 0.48070
 
 Not queried
 
  1.3981218
 
       NA
 
       NA
  
 
  
 5
 
 140792511
 
 140792700
 
  ENSEMBL   UCSC 
 
  190
 
  5
 
 32322
 
 tss
 
 0.02010
 
 0.04257
 
 Not queried
 
  1.3981162
 
       NA
 
       NA
  
 
  
 4
 
   5021084
 
   5021328
 
  ENSEMBL   UCSC 
 
  245
 
  8
 
 30825
 
 island
 
 0.63506
 
 0.41453
 
 Not queried
 
  1.3978612
 
       NA
 
       NA
  
 
  
 6
 
 100583956
 
 100584395
 
  ENSEMBL   UCSC 
 
  440
 
  6
 
 36300
 
 island
 
 0.75991
 
 0.87556
 
 Not queried
 
  1.3974241
 
       NA
 
       NA
  
 
  
 6
 
 108485043
 
 108485182
 
  ENSEMBL   UCSC 
 
  140
 
  4
 
 36333
 
 island
 
 0.29280
 
 0.46274
 
 Not queried
 
  1.3973856
 
       NA
 
       NA
  
 
  
 19
 
   6007842
 
   6008127
 
  ENSEMBL   UCSC 
 
  286
 
  4
 
 19725
 
 gene;island
 
 0.69458
 
 0.82959
 
 Not queried
 
  1.3972796
 
       NA
 
       NA
  
 
  
 X
 
  19533340
 
  19533577
 
  ENSEMBL   UCSC 
 
  238
 
  7
 
 41676
 
 island
 
 0.04096
 
 0.01913
 
 Not queried
 
  1.3971956
 
       NA
 
       NA
  
 
  
 7
 
 150690699
 
 150690969
 
  ENSEMBL   UCSC 
 
  271
 
  4
 
 37217
 
 gene
 
 0.47018
 
 0.69584
 
 Not queried
 
  1.3970420
 
       NA
 
       NA
  
 
  
 10
 
 116164598
 
 116164955
 
  ENSEMBL   UCSC 
 
  358
 
  6
 
 5360
 
 tss;island
 
 0.14734
 
 0.07248
 
 Not queried
 
  1.3967471
 
       NA
 
       NA
  
 
  
 13
 
 111672375
 
 111672726
 
  ENSEMBL   UCSC 
 
  352
 
  4
 
 11864
 
 island
 
 0.91249
 
 0.77348
 
 Not queried
 
  1.3966542
 
       NA
 
       NA
  
 
  
 X
 
  56258113
 
  56258812
 
  ENSEMBL   UCSC 
 
  700
 
  8
 
 41344
 
 tss
 
 0.05722
 
 0.02863
 
 Not queried
 
  1.3965530
 
       NA
 
       NA
  
 
  
 11
 
    811545
 
    811791
 
  ENSEMBL   UCSC 
 
  247
 
  5
 
 6373
 
 gene;island
 
 0.62550
 
 0.80802
 
 Not queried
 
  1.3961333
 
       NA
 
       NA
  
 
  
 10
 
 114610508
 
 114610635
 
  ENSEMBL   UCSC 
 
  128
 
  4
 
 5350
 
 tss
 
 0.61880
 
 0.81029
 
 Not queried
 
  1.3959856
 
       NA
 
       NA
  
 
  
 6
 
  33036464
 
  33036717
 
  ENSEMBL   UCSC 
 
  254
 
  6
 
 34115
 
 gene
 
 0.84883
 
 0.68091
 
 Not queried
 
  1.3958713
 
       NA
 
       NA
  
 
  
 6
 
  32712930
 
  32713044
 
  ENSEMBL   UCSC 
 
  115
 
  4
 
 34064
 
 gene
 
 0.58222
 
 0.30218
 
 Not queried
 
  1.3955884
 
       NA
 
       NA
  
 
  
 17
 
  42015369
 
  42015961
 
  ENSEMBL   UCSC 
 
  593
 
  7
 
 18347
 
 island
 
 0.05688
 
 0.10460
 
 Not queried
 
  1.3955770
 
       NA
 
       NA
  
 
  
 16
 
  51147461
 
  51147902
 
  ENSEMBL   UCSC 
 
  442
 
  6
 
 15884
 
 island
 
 0.18140
 
 0.40827
 
 Not queried
 
  1.3954676
 
       NA
 
       NA
  
 
  
 1
 
  35250229
 
  35250839
 
  ENSEMBL   UCSC 
 
  611
 
  7
 
 412
 
 gene;island
 
 0.71794
 
 0.56221
 
 Not queried
 
  1.3953046
 
       NA
 
       NA
  
 
  
 16
 
  88747591
 
  88747919
 
  ENSEMBL   UCSC 
 
  329
 
  4
 
 14880
 
 gene;island
 
 0.34187
 
 0.64943
 
 Not queried
 
  1.3951976
 
       NA
 
       NA
  
 
  
 11
 
   2922334
 
   2922852
 
  ENSEMBL   UCSC 
 
  519
 
 12
 
 6452
 
 gene;tss;island
 
 0.75528
 
 0.62358
 
 Not queried
 
  1.3950144
 
       NA
 
       NA
  
 
  
 12
 
 133302012
 
 133302266
 
  ENSEMBL   UCSC 
 
  255
 
  6
 
 11044
 
 island
 
 0.88444
 
 0.94677
 
 Not queried
 
  1.3948995
 
       NA
 
       NA
  
 
  
 1
 
  21044685
 
  21044945
 
  ENSEMBL   UCSC 
 
  261
 
  6
 
 1314
 
 tss;island
 
 0.37430
 
 0.25502
 
 Not queried
 
  1.3946788
 
       NA
 
       NA
  
 
  
 X
 
  31090296
 
  31090380
 
  ENSEMBL   UCSC 
 
   85
 
  6
 
 41247
 
 tss
 
 0.78593
 
 0.93556
 
 Not queried
 
  1.3946201
 
       NA
 
       NA
  
 
  
 1
 
 220921216
 
 220921670
 
  ENSEMBL   UCSC 
 
  455
 
  8
 
 2258
 
 tss
 
 0.03631
 
 0.01415
 
 Not queried
 
  1.3946094
 
       NA
 
       NA
  
 
  
 5
 
 153858816
 
 153859049
 
  ENSEMBL   UCSC 
 
  234
 
  5
 
 32390
 
 tss;island
 
 0.02036
 
 0.04302
 
 Not queried
 
  1.3945545
 
       NA
 
       NA
  
 
  
 12
 
  38532352
 
  38532713
 
  ENSEMBL   UCSC 
 
  362
 
  4
 
 10297
 
 island
 
 0.04845
 
 0.09241
 
 Not queried
 
  1.3940556
 
       NA
 
       NA
  
 
  
 12
 
 132863782
 
 132864302
 
  ENSEMBL   UCSC 
 
  521
 
  6
 
 9419
 
 gene;island
 
 0.69368
 
 0.83426
 
 Not queried
 
  1.3922742
 
       NA
 
       NA
  
 
  
 3
 
  48282103
 
  48282590
 
  ENSEMBL   UCSC 
 
  488
 
  9
 
 28252
 
 tss
 
 0.07408
 
 0.03549
 
 Not queried
 
  1.3922322
 
       NA
 
       NA
  
 
  
 17
 
  72931569
 
  72931857
 
  ENSEMBL   UCSC 
 
  289
 
 10
 
 17604
 
 tss
 
 0.20095
 
 0.43305
 
 Not queried
 
  1.3921387
 
       NA
 
       NA
  
 
  
 12
 
  72332386
 
  72332624
 
  ENSEMBL   UCSC 
 
  239
 
  4
 
 9851
 
 tss
 
 0.06756
 
 0.16491
 
 Not queried
 
  1.3918782
 
       NA
 
       NA
  
 
  
 16
 
  89146961
 
  89147306
 
  ENSEMBL   UCSC 
 
  346
 
  4
 
 16201
 
 island
 
 0.43896
 
 0.67756
 
 Not queried
 
  1.3918662
 
       NA
 
       NA
  
 
  
 19
 
  34311123
 
  34311562
 
  ENSEMBL   UCSC 
 
  440
 
  5
 
 21848
 
 island
 
 0.06959
 
 0.15044
 
 Not queried
 
  1.3911696
 
       NA
 
       NA
  
 
  
 14
 
  69951720
 
  69952104
 
  ENSEMBL   UCSC 
 
  385
 
  5
 
 12097
 
 gene;island
 
 0.46662
 
 0.17802
 
 Not queried
 
  1.3910337
 
       NA
 
       NA
  
 
  
 1
 
  28559548
 
  28559620
 
  ENSEMBL   UCSC 
 
   73
 
  4
 
 1421
 
 tss
 
 0.06842
 
 0.04030
 
 Not queried
 
  1.3908645
 
       NA
 
       NA
  
 
  
 8
 
 124219425
 
 124219725
 
  ENSEMBL   UCSC 
 
  301
 
  4
 
 39356
 
 gene;island
 
 0.79874
 
 0.90863
 
 Not queried
 
  1.3908463
 
       NA
 
       NA
  
 
  
 2
 
 220362124
 
 220362242
 
  ENSEMBL   UCSC 
 
  119
 
  4
 
 23869
 
 tss
 
 0.41586
 
 0.59203
 
 Not queried
 
  1.3901075
 
       NA
 
       NA
  
 
  
 15
 
  91497863
 
  91498080
 
  ENSEMBL   UCSC 
 
  218
 
  6
 
 13880
 
 tss
 
 0.02395
 
 0.06688
 
 Not queried
 
  1.3900556
 
       NA
 
       NA
  
 
  
 1
 
  90309205
 
  90309605
 
  ENSEMBL   UCSC 
 
  401
 
  5
 
 621
 
 gene;island
 
 0.85405
 
 0.67216
 
 Not queried
 
  1.3898365
 
       NA
 
       NA
  
 
  
 12
 
  54366760
 
  54367008
 
  ENSEMBL   UCSC 
 
  249
 
  6
 
 10457
 
 island
 
 0.18332
 
 0.25953
 
 Not queried
 
  1.3898005
 
       NA
 
       NA
  
 
  
 5
 
 145724352
 
 145724643
 
  ENSEMBL   UCSC 
 
  292
 
  5
 
 33221
 
 island
 
 0.05227
 
 0.03038
 
 Not queried
 
  1.3897451
 
       NA
 
       NA
  
 
  
 16
 
  88038949
 
  88039257
 
  ENSEMBL   UCSC 
 
  309
 
  4
 
 14866
 
 gene;island
 
 0.79677
 
 0.93780
 
 Not queried
 
  1.3895669
 
       NA
 
       NA
  
 
  
 6
 
  30458519
 
  30458730
 
  ENSEMBL   UCSC 
 
  212
 
  4
 
 33706
 
 gene;island
 
 0.68565
 
 0.92853
 
 Not queried
 
  1.3895649
 
       NA
 
       NA
  
 
  
 17
 
   6947288
 
   6947464
 
  ENSEMBL   UCSC 
 
  177
 
  8
 
 17027
 
 tss
 
 0.09424
 
 0.27385
 
 Not queried
 
  1.3890978
 
       NA
 
       NA
  
 
  
 17
 
   6947032
 
   6947464
 
  ENSEMBL   UCSC 
 
  433
 
 10
 
 17885
 
 island
 
 0.09424
 
 0.27385
 
 Not queried
 
  1.3890978
 
       NA
 
       NA
  
 
  
 8
 
  65489863
 
  65489939
 
  ENSEMBL   UCSC 
 
   77
 
  5
 
 39670
 
 tss;island
 
 0.07222
 
 0.14434
 
 Not queried
 
  1.3890496
 
       NA
 
       NA
  
 
  
 16
 
  67184918
 
  67185194
 
  ENSEMBL   UCSC 
 
  277
 
  6
 
 15261
 
 tss;island
 
 0.05569
 
 0.03109
 
 Not queried
 
  1.3886571
 
       NA
 
       NA
  
 
  
 7
 
 142636371
 
 142636518
 
  ENSEMBL   UCSC 
 
  148
 
  4
 
 37885
 
 tss
 
 0.56649
 
 0.35247
 
 Not queried
 
  1.3882116
 
       NA
 
       NA
  
 
  
 4
 
   6675513
 
   6675790
 
  ENSEMBL   UCSC 
 
  278
 
  9
 
 30235
 
 tss
 
 0.05848
 
 0.03366
 
 Not queried
 
  1.3879258
 
       NA
 
       NA
  
 
  
 10
 
  26726877
 
  26727261
 
  ENSEMBL   UCSC 
 
  385
 
  7
 
 4977
 
 tss
 
 0.22196
 
 0.53268
 
 Not queried
 
  1.3878399
 
       NA
 
       NA
  
 
  
 12
 
  22487658
 
  22487847
 
  ENSEMBL   UCSC 
 
  190
 
  5
 
 9577
 
 tss
 
 0.02246
 
 0.06808
 
 Not queried
 
  1.3874364
 
       NA
 
       NA
  
 
  
 12
 
  22487437
 
  22487847
 
  ENSEMBL   UCSC 
 
  411
 
  7
 
 10258
 
 island
 
 0.02246
 
 0.05790
 
 Not queried
 
  1.3874364
 
       NA
 
       NA
  
 
  
 11
 
  63828569
 
  63828762
 
  ENSEMBL   UCSC 
 
  194
 
  4
 
 6643
 
 gene
 
 0.04946
 
 0.02664
 
 Not queried
 
  1.3870480
 
       NA
 
       NA
  
 
  
 11
 
 113947036
 
 113947359
 
  ENSEMBL   UCSC 
 
  324
 
  4
 
 6889
 
 gene
 
 0.74503
 
 0.53015
 
 Not queried
 
  1.3869265
 
       NA
 
       NA
  
 
  
 12
 
 131417943
 
 131418404
 
  ENSEMBL   UCSC 
 
  462
 
  4
 
 10982
 
 island
 
 0.64346
 
 0.81680
 
 Not queried
 
  1.3868467
 
       NA
 
       NA
  
 
  
 10
 
 115438884
 
 115439008
 
  ENSEMBL   UCSC 
 
  125
 
  5
 
 5352
 
 tss;island
 
 0.01801
 
 0.05437
 
 Not queried
 
  1.3861147
 
       NA
 
       NA
  
 
  
 11
 
  62553869
 
  62554234
 
  ENSEMBL   UCSC 
 
  366
 
  4
 
 6628
 
 gene;tss;island
 
 0.13606
 
 0.25465
 
 Not queried
 
  1.3857149
 
       NA
 
       NA
  
 
  
 8
 
  10261641
 
  10262221
 
  ENSEMBL   UCSC 
 
  581
 
  6
 
 39132
 
 gene;island
 
 0.71647
 
 0.81556
 
 Not queried
 
  1.3856286
 
       NA
 
       NA
  
 
  
 11
 
 108464350
 
 108464498
 
  ENSEMBL   UCSC 
 
  149
 
  5
 
 8761
 
 island
 
 0.00949
 
 0.01826
 
 Not queried
 
  1.3851369
 
       NA
 
       NA
  
 
  
 14
 
  99736387
 
  99736559
 
  ENSEMBL   UCSC 
 
  173
 
  4
 
 12174
 
 gene;island
 
 0.00883
 
 0.01648
 
 Not queried
 
  1.3850328
 
       NA
 
       NA
  
 
  
 19
 
    450940
 
    451320
 
  ENSEMBL   UCSC 
 
  381
 
  4
 
 19592
 
 gene;island
 
 0.90191
 
 0.77108
 
 Not queried
 
  1.3849335
 
       NA
 
       NA
  
 
  
 1
 
  27668325
 
  27668445
 
  ENSEMBL   UCSC 
 
  121
 
  4
 
 1404
 
 tss
 
 0.27140
 
 0.39098
 
 Not queried
 
  1.3848372
 
       NA
 
       NA
  
 
  
 12
 
    534563
 
    534917
 
  ENSEMBL   UCSC 
 
  355
 
  4
 
 8970
 
 gene
 
 0.91151
 
 0.96309
 
 Not queried
 
  1.3845473
 
       NA
 
       NA
  
 
  
 1
 
  47489195
 
  47489543
 
  ENSEMBL   UCSC 
 
  349
 
  5
 
 3221
 
 island
 
 0.03556
 
 0.07659
 
 Not queried
 
  1.3843522
 
       NA
 
       NA
  
 
  
 4
 
 154605087
 
 154605276
 
  ENSEMBL   UCSC 
 
  190
 
  4
 
 30594
 
 tss
 
 0.12890
 
 0.04348
 
 Not queried
 
  1.3840506
 
       NA
 
       NA
  
 
  
 1
 
  28285036
 
  28285385
 
  ENSEMBL   UCSC 
 
  350
 
  4
 
 361
 
 gene;tss;island
 
 0.93512
 
 0.86031
 
 Not queried
 
  1.3839782
 
       NA
 
       NA
  
 
  
 10
 
  99472988
 
  99473224
 
  ENSEMBL   UCSC 
 
  237
 
  5
 
 5253
 
 tss;island
 
 0.62069
 
 0.22018
 
 Not queried
 
  1.3836049
 
       NA
 
       NA
  
 
  
 14
 
  90527891
 
  90527990
 
  ENSEMBL   UCSC 
 
  100
 
  6
 
 12524
 
 tss;island
 
 0.01410
 
 0.03058
 
 Not queried
 
  1.3835840
 
       NA
 
       NA
  
 
  
 10
 
  45495677
 
  45496710
 
  ENSEMBL   UCSC 
 
 1034
 
 16
 
 4562
 
 gene;island
 
 0.10933
 
 0.05367
 
 Not queried
 
  1.3835633
 
       NA
 
       NA
  
 
  
 22
 
  51001200
 
  51001387
 
  ENSEMBL   UCSC 
 
  188
 
  6
 
 27588
 
 island
 
 0.01180
 
 0.01546
 
 Not queried
 
  1.3835160
 
       NA
 
       NA
  
 
  
 4
 
   2935884
 
   2936284
 
  ENSEMBL   UCSC 
 
  401
 
  8
 
 30207
 
 tss
 
 0.04039
 
 0.06958
 
 Not queried
 
  1.3834377
 
       NA
 
       NA
  
 
  
 3
 
  11058836
 
  11059050
 
  ENSEMBL   UCSC 
 
  215
 
  6
 
 27623
 
 gene;island
 
 0.85945
 
 0.64492
 
 Not queried
 
  1.3833566
 
       NA
 
       NA
  
 
  
 1
 
  33937968
 
  33938307
 
  ENSEMBL   UCSC 
 
  340
 
  8
 
 3009
 
 island
 
 0.01165
 
 0.02547
 
 Not queried
 
  1.3832255
 
       NA
 
       NA
  
 
  
 3
 
  44283035
 
  44283355
 
  ENSEMBL   UCSC 
 
  321
 
  9
 
 28212
 
 tss
 
 0.96514
 
 0.87339
 
 Not queried
 
  1.3829600
 
       NA
 
       NA
  
 
  
 22
 
  35947764
 
  35948166
 
  ENSEMBL   UCSC 
 
  403
 
  4
 
 26811
 
 gene;island
 
 0.53919
 
 0.78159
 
 Not queried
 
  1.3822652
 
       NA
 
       NA
  
 
  
 3
 
  49837348
 
  49837689
 
  ENSEMBL   UCSC 
 
  342
 
  4
 
 28294
 
 tss;island
 
 0.61189
 
 0.44933
 
 Not queried
 
  1.3821805
 
       NA
 
       NA
  
 
  
 6
 
  33264834
 
  33265605
 
  ENSEMBL   UCSC 
 
  772
 
 14
 
 34191
 
 gene;island
 
 0.12805
 
 0.04810
 
 Not queried
 
  1.3807163
 
       NA
 
       NA
  
 
  
 21
 
  28339262
 
  28339487
 
  ENSEMBL   UCSC 
 
  226
 
  5
 
 26581
 
 island
 
 0.00876
 
 0.02715
 
 Not queried
 
  1.3806788
 
       NA
 
       NA
  
 
  
 11
 
  67205096
 
  67205291
 
  ENSEMBL   UCSC 
 
  196
 
  5
 
 8527
 
 island
 
 0.67147
 
 0.84178
 
 Not queried
 
  1.3802182
 
       NA
 
       NA
  
 
  
 17
 
  62207504
 
  62207789
 
  ENSEMBL   UCSC 
 
  286
 
  5
 
 17546
 
 tss
 
 0.01346
 
 0.02480
 
 Not queried
 
  1.3797392
 
       NA
 
       NA
  
 
  
 6
 
  30594037
 
  30594605
 
  ENSEMBL   UCSC 
 
  569
 
 10
 
 34712
 
 tss
 
 0.07125
 
 0.27577
 
 Not queried
 
  1.3794450
 
       NA
 
       NA
  
 
  
 6
 
  28058715
 
  28058918
 
  ENSEMBL   UCSC 
 
  204
 
  8
 
 34667
 
 tss
 
 0.76713
 
 0.89753
 
 Not queried
 
  1.3790615
 
       NA
 
       NA
  
 
  
 5
 
 179498943
 
 179499488
 
  ENSEMBL   UCSC 
 
  546
 
  6
 
 33433
 
 island
 
 0.03720
 
 0.03954
 
 Not queried
 
  1.3789684
 
       NA
 
       NA
  
 
  
 15
 
  49255686
 
  49256042
 
  ENSEMBL   UCSC 
 
  357
 
  4
 
 13632
 
 tss
 
 0.01999
 
 0.09904
 
 Not queried
 
  1.3786910
 
       NA
 
       NA
  
 
  
 6
 
  31322577
 
  31322996
 
  ENSEMBL   UCSC 
 
  420
 
  5
 
 33804
 
 gene;island
 
 0.95191
 
 0.75935
 
 Not queried
 
  1.3769811
 
       NA
 
       NA
  
 
  
 5
 
 179763432
 
 179763951
 
  ENSEMBL   UCSC 
 
  520
 
  5
 
 31884
 
 gene
 
 0.62153
 
 0.86318
 
 Not queried
 
  1.3768698
 
       NA
 
       NA
  
 
  
 11
 
  44337982
 
  44338421
 
  ENSEMBL   UCSC 
 
  440
 
  5
 
 8204
 
 island
 
 0.24078
 
 0.09135
 
 Not queried
 
  1.3767788
 
       NA
 
       NA
  
 
  
 2
 
 176962658
 
 176962928
 
  ENSEMBL   UCSC 
 
  271
 
  4
 
 24854
 
 island
 
 0.01381
 
 0.03140
 
 Not queried
 
  1.3765689
 
       NA
 
       NA
  
 
  
 1
 
  99470550
 
  99470967
 
  ENSEMBL   UCSC 
 
  418
 
  7
 
 1769
 
 tss;island
 
 0.02588
 
 0.06145
 
 Not queried
 
  1.3764300
 
       NA
 
       NA
  
 
  
 17
 
  46799479
 
  46799845
 
  ENSEMBL   UCSC 
 
  367
 
  5
 
 16627
 
 gene
 
 0.95243
 
 0.60390
 
 Not queried
 
  1.3763637
 
       NA
 
       NA
  
 
  
 1
 
  12677936
 
  12678355
 
  ENSEMBL   UCSC 
 
  420
 
  7
 
 1256
 
 tss;island
 
 0.00897
 
 0.02411
 
 Not queried
 
  1.3763576
 
       NA
 
       NA
  
 
  
 6
 
  88757302
 
  88757392
 
  ENSEMBL   UCSC 
 
   91
 
  5
 
 35050
 
 tss;island
 
 0.81985
 
 0.90924
 
 Not queried
 
  1.3759837
 
       NA
 
       NA
  
 
  
 1
 
 151042743
 
 151043024
 
  ENSEMBL   UCSC 
 
  282
 
  8
 
 1917
 
 tss
 
 0.02253
 
 0.05523
 
 Not queried
 
  1.3757714
 
       NA
 
       NA
  
 
  
 7
 
  45039598
 
  45039709
 
  ENSEMBL   UCSC 
 
  112
 
  6
 
 37513
 
 tss
 
 0.00804
 
 0.02055
 
 Not queried
 
  1.3755168
 
       NA
 
       NA
  
 
  
 7
 
    609506
 
    609820
 
  ENSEMBL   UCSC 
 
  315
 
  4
 
 36648
 
 gene;island
 
 0.84267
 
 0.92610
 
 Not queried
 
  1.3754530
 
       NA
 
       NA
  
 
  
 13
 
  47126300
 
  47126551
 
  ENSEMBL   UCSC 
 
  252
 
  4
 
 11399
 
 tss;island
 
 0.05275
 
 0.05459
 
 Not queried
 
  1.3751939
 
       NA
 
       NA
  
 
  
 1
 
 151031286
 
 151031323
 
  ENSEMBL   UCSC 
 
   38
 
  4
 
 745
 
 gene;tss;island
 
 0.01824
 
 0.04638
 
 Not queried
 
  1.3749918
 
       NA
 
       NA
  
 
  
 X
 
  47082293
 
  47082510
 
  ENSEMBL   UCSC 
 
  218
 
  4
 
 40976
 
 gene;island
 
 0.82141
 
 0.92137
 
 Not queried
 
  1.3748994
 
       NA
 
       NA
  
 
  
 11
 
  93394605
 
  93394809
 
  ENSEMBL   UCSC 
 
  205
 
  8
 
 7599
 
 tss
 
 0.03547
 
 0.01699
 
 Not queried
 
  1.3742406
 
       NA
 
       NA
  
 
  
 12
 
 130184591
 
 130185016
 
  ENSEMBL   UCSC 
 
  426
 
  4
 
 9386
 
 gene;island
 
 0.92643
 
 0.81701
 
 Not queried
 
  1.3739779
 
       NA
 
       NA
  
 
  
 3
 
 160823231
 
 160823505
 
  ENSEMBL   UCSC 
 
  275
 
  7
 
 28654
 
 tss;island
 
 0.31550
 
 0.11148
 
 Not queried
 
  1.3736384
 
       NA
 
       NA
  
 
  
 1
 
  18691778
 
  18692019
 
  ENSEMBL   UCSC 
 
  242
 
  5
 
 270
 
 gene;island
 
 0.67590
 
 0.76754
 
 Not queried
 
  1.3735720
 
       NA
 
       NA
  
 
  
 6
 
  31475762
 
  31475984
 
  ENSEMBL   UCSC 
 
  223
 
  4
 
 33822
 
 gene
 
 0.83238
 
 0.63405
 
 Not queried
 
  1.3735299
 
       NA
 
       NA
  
 
  
 3
 
  58563609
 
  58563693
 
  ENSEMBL   UCSC 
 
   85
 
  5
 
 28376
 
 tss
 
 0.18640
 
 0.48617
 
 Not queried
 
  1.3732978
 
       NA
 
       NA
  
 
  
 21
 
  43188171
 
  43188367
 
  ENSEMBL   UCSC 
 
  197
 
  4
 
 26519
 
 tss;island
 
 0.01725
 
 0.03922
 
 Not queried
 
  1.3730575
 
       NA
 
       NA
  
 
  
 7
 
 139256124
 
 139256620
 
  ENSEMBL   UCSC 
 
  497
 
  6
 
 37181
 
 gene
 
 0.87949
 
 0.93873
 
 Not queried
 
  1.3730465
 
       NA
 
       NA
  
 
  
 3
 
 136537607
 
 136537856
 
  ENSEMBL   UCSC 
 
  250
 
  8
 
 28571
 
 tss
 
 0.13704
 
 0.05124
 
 Not queried
 
  1.3725685
 
       NA
 
       NA
  
 
  
 6
 
  32363875
 
  32364075
 
  ENSEMBL   UCSC 
 
  201
 
  6
 
 34044
 
 gene
 
 0.85562
 
 0.68201
 
 Not queried
 
  1.3724529
 
       NA
 
       NA
  
 
  
 19
 
  46806907
 
  46807660
 
  ENSEMBL   UCSC 
 
  754
 
  6
 
 20073
 
 gene;island
 
 0.26274
 
 0.07926
 
 Not queried
 
  1.3722595
 
       NA
 
       NA
  
 
  
 4
 
 166795260
 
 166795913
 
  ENSEMBL   UCSC 
 
  654
 
  9
 
 30126
 
 gene;island
 
 0.01532
 
 0.02850
 
 Not queried
 
  1.3720846
 
       NA
 
       NA
  
 
  
 13
 
  91826885
 
  91827042
 
  ENSEMBL   UCSC 
 
  158
 
  4
 
 11783
 
 island
 
 0.64446
 
 0.73801
 
 Not queried
 
  1.3715034
 
       NA
 
       NA
  
 
  
 5
 
 175223225
 
 175223497
 
  ENSEMBL   UCSC 
 
  273
 
  4
 
 32448
 
 tss
 
 0.56781
 
 0.18556
 
 Not queried
 
  1.3710670
 
       NA
 
       NA
  
 
  
 11
 
   8290295
 
   8290476
 
  ENSEMBL   UCSC 
 
  182
 
  5
 
 8011
 
 island
 
 0.06532
 
 0.01351
 
 Not queried
 
  1.3709010
 
       NA
 
       NA
  
 
  
 4
 
  15964913
 
  15965102
 
  ENSEMBL   UCSC 
 
  190
 
  5
 
 30265
 
 tss
 
 0.28647
 
 0.44155
 
 Not queried
 
  1.3704764
 
       NA
 
       NA
  
 
  
 3
 
  71833543
 
  71833907
 
  ENSEMBL   UCSC 
 
  365
 
  5
 
 27818
 
 gene;island
 
 0.06219
 
 0.02485
 
 Not queried
 
  1.3701843
 
       NA
 
       NA
  
 
  
 1
 
  62660188
 
  62660421
 
  ENSEMBL   UCSC 
 
  234
 
  4
 
 1660
 
 tss
 
 0.34304
 
 0.13996
 
 Not queried
 
  1.3701552
 
       NA
 
       NA
  
 
  
 X
 
  50213745
 
  50214061
 
  ENSEMBL   UCSC 
 
  317
 
  5
 
 41320
 
 tss
 
 0.08149
 
 0.02099
 
 Not queried
 
  1.3700411
 
       NA
 
       NA
  
 
  
 15
 
  31175309
 
  31175522
 
  ENSEMBL   UCSC 
 
  214
 
  4
 
 13951
 
 island
 
 0.86019
 
 0.94229
 
 Not queried
 
  1.3698952
 
       NA
 
       NA
  
 
  
 1
 
   1335315
 
   1335706
 
  ENSEMBL   UCSC 
 
  392
 
  5
 
 35
 
 gene;tss;island
 
 0.02353
 
 0.03634
 
 Not queried
 
  1.3698166
 
       NA
 
       NA
  
 
  
 1
 
   3605747
 
   3606082
 
  ENSEMBL   UCSC 
 
  336
 
  5
 
 135
 
 gene;tss;island
 
 0.40914
 
 0.61518
 
 Not queried
 
  1.3692233
 
       NA
 
       NA
  
 
  
 4
 
   3308884
 
   3309069
 
  ENSEMBL   UCSC 
 
  186
 
  4
 
 30786
 
 island
 
 0.95225
 
 0.88477
 
 Not queried
 
  1.3690833
 
       NA
 
       NA
  
 
  
 17
 
  78755379
 
  78755604
 
  ENSEMBL   UCSC 
 
  226
 
  4
 
 16838
 
 gene
 
 0.86641
 
 0.92944
 
 Not queried
 
  1.3688938
 
       NA
 
       NA
  
 
  
 7
 
 158497539
 
 158497565
 
  ENSEMBL   UCSC 
 
   27
 
  5
 
 37958
 
 tss;island
 
 0.07468
 
 0.03772
 
 Not queried
 
  1.3680291
 
       NA
 
       NA
  
 
  
 3
 
 133392794
 
 133393240
 
  ENSEMBL   UCSC 
 
  447
 
  4
 
 29447
 
 island
 
 0.33623
 
 0.19803
 
 Not queried
 
  1.3676812
 
       NA
 
       NA
  
 
  
 5
 
 140787430
 
 140787623
 
  ENSEMBL   UCSC 
 
  194
 
  4
 
 31774
 
 gene;tss;island
 
 0.05401
 
 0.09696
 
 Not queried
 
  1.3672943
 
       NA
 
       NA
  
 
  
 15
 
  44116991
 
  44117354
 
  ENSEMBL   UCSC 
 
  364
 
  6
 
 13607
 
 tss
 
 0.37719
 
 0.08235
 
 Not queried
 
  1.3664594
 
       NA
 
       NA
  
 
  
 17
 
  43506497
 
  43507153
 
  ENSEMBL   UCSC 
 
  657
 
  7
 
 18404
 
 island
 
 0.46959
 
 0.13799
 
 Not queried
 
  1.3664187
 
       NA
 
       NA
  
 
  
 12
 
 132834252
 
 132834467
 
  ENSEMBL   UCSC 
 
  216
 
  5
 
 9414
 
 gene;island
 
 0.86145
 
 0.89797
 
 Not queried
 
  1.3663217
 
       NA
 
       NA
  
 
  
 5
 
  72793969
 
  72794209
 
  ENSEMBL   UCSC 
 
  241
 
  6
 
 32062
 
 tss
 
 0.04364
 
 0.01625
 
 Not queried
 
  1.3661543
 
       NA
 
       NA
  
 
  
 6
 
  33739406
 
  33739653
 
  ENSEMBL   UCSC 
 
  248
 
  4
 
 34233
 
 gene;island
 
 0.10646
 
 0.15432
 
 Not queried
 
  1.3660891
 
       NA
 
       NA
  
 
  
 6
 
  30038712
 
  30039600
 
  ENSEMBL   UCSC 
 
  889
 
 40
 
 33654
 
 gene;island
 
 0.08065
 
 0.15487
 
 Not queried
 
  1.3660730
 
       NA
 
       NA
  
 
  
 15
 
  34630330
 
  34630530
 
  ENSEMBL   UCSC 
 
  201
 
  6
 
 13530
 
 tss;island
 
 0.02622
 
 0.04920
 
 Not queried
 
  1.3658865
 
       NA
 
       NA
  
 
  
 22
 
  26986233
 
  26986419
 
  ENSEMBL   UCSC 
 
  187
 
  5
 
 26994
 
 tss;island
 
 0.01943
 
 0.03328
 
 Not queried
 
  1.3651863
 
       NA
 
       NA
  
 
  
 17
 
  40687822
 
  40688338
 
  ENSEMBL   UCSC 
 
  517
 
 10
 
 18306
 
 island
 
 0.03385
 
 0.13459
 
 Not queried
 
  1.3645101
 
       NA
 
       NA
  
 
  
 12
 
   6657744
 
   6658164
 
  ENSEMBL   UCSC 
 
  421
 
  5
 
 9005
 
 gene;island
 
 0.77372
 
 0.91086
 
 Not queried
 
  1.3641781
 
       NA
 
       NA
  
 
  
 2
 
  85980500
 
  85980874
 
  ENSEMBL   UCSC 
 
  375
 
 10
 
 23450
 
 tss
 
 0.07158
 
 0.03592
 
 Not queried
 
  1.3638592
 
       NA
 
       NA
  
 
  
 16
 
  67197084
 
  67197186
 
  ENSEMBL   UCSC 
 
  103
 
  5
 
 14760
 
 gene;tss;island
 
 0.03347
 
 0.08251
 
 Not queried
 
  1.3638536
 
       NA
 
       NA
  
 
  
 3
 
  46448753
 
  46449100
 
  ENSEMBL   UCSC 
 
  348
 
  5
 
 27706
 
 gene
 
 0.61198
 
 0.79936
 
 Not queried
 
  1.3629872
 
       NA
 
       NA
  
 
  
 21
 
  46912386
 
  46912545
 
  ENSEMBL   UCSC 
 
  160
 
  4
 
 26429
 
 gene;island
 
 0.89256
 
 0.96104
 
 Not queried
 
  1.3627756
 
       NA
 
       NA
  
 
  
 12
 
  53693054
 
  53693490
 
  ENSEMBL   UCSC 
 
  437
 
 10
 
 10434
 
 island
 
 0.04383
 
 0.19522
 
 Not queried
 
  1.3627304
 
       NA
 
       NA
  
 
  
 16
 
  85517170
 
  85517684
 
  ENSEMBL   UCSC 
 
  515
 
  7
 
 16121
 
 island
 
 0.78801
 
 0.89261
 
 Not queried
 
  1.3627198
 
       NA
 
       NA
  
 
  
 1
 
   6639570
 
   6639762
 
  ENSEMBL   UCSC 
 
  193
 
  4
 
 178
 
 gene
 
 0.02431
 
 0.03463
 
 Not queried
 
  1.3625045
 
       NA
 
       NA
  
 
  
 10
 
 124713989
 
 124714384
 
  ENSEMBL   UCSC 
 
  396
 
  8
 
 5394
 
 tss
 
 0.03444
 
 0.06888
 
 Not queried
 
  1.3622998
 
       NA
 
       NA
  
 
  
 3
 
 135684439
 
 135684468
 
  ENSEMBL   UCSC 
 
   30
 
  4
 
 28566
 
 tss;island
 
 0.02492
 
 0.04620
 
 Not queried
 
  1.3622581
 
       NA
 
       NA
  
 
  
 10
 
 134910271
 
 134910540
 
  ENSEMBL   UCSC 
 
  270
 
  4
 
 4867
 
 gene;island
 
 0.68277
 
 0.84577
 
 Not queried
 
  1.3622204
 
       NA
 
       NA
  
 
  
 6
 
 106428957
 
 106429769
 
  ENSEMBL   UCSC 
 
  813
 
  9
 
 36315
 
 island
 
 0.77379
 
 0.90862
 
 Not queried
 
  1.3621285
 
       NA
 
       NA
  
 
  
 10
 
  98802990
 
  98803335
 
  ENSEMBL   UCSC 
 
  346
 
  4
 
 4677
 
 gene;island
 
 0.75292
 
 0.91326
 
 Not queried
 
  1.3619505
 
       NA
 
       NA
  
 
  
 16
 
  67562424
 
  67562672
 
  ENSEMBL   UCSC 
 
  249
 
  4
 
 15274
 
 tss;island
 
 0.25378
 
 0.06751
 
 Not queried
 
  1.3616313
 
       NA
 
       NA
  
 
  
 2
 
 238395764
 
 238395947
 
  ENSEMBL   UCSC 
 
  184
 
  5
 
 25192
 
 island
 
 0.09639
 
 0.03986
 
 Not queried
 
  1.3615889
 
       NA
 
       NA
  
 
  
 19
 
  55314879
 
  55315034
 
  ENSEMBL   UCSC 
 
  156
 
  4
 
 21029
 
 tss
 
 0.45409
 
 0.69988
 
 Not queried
 
  1.3615809
 
       NA
 
       NA
  
 
  
 3
 
  30936070
 
  30936531
 
  ENSEMBL   UCSC 
 
  462
 
  9
 
 28915
 
 island
 
 0.02027
 
 0.08117
 
 Not queried
 
  1.3615802
 
       NA
 
       NA
  
 
  
 8
 
 145625605
 
 145625814
 
  ENSEMBL   UCSC 
 
  210
 
  4
 
 39917
 
 tss
 
 0.86699
 
 0.94158
 
 Not queried
 
  1.3615317
 
       NA
 
       NA
  
 
  
 5
 
 135364827
 
 135365012
 
  ENSEMBL   UCSC 
 
  186
 
  4
 
 31704
 
 gene;island
 
 0.09755
 
 0.02801
 
 Not queried
 
  1.3611813
 
       NA
 
       NA
  
 
  
 17
 
  66194137
 
  66195485
 
  ENSEMBL   UCSC 
 
 1349
 
 14
 
 18652
 
 island
 
 0.11861
 
 0.23765
 
 Not queried
 
  1.3611571
 
       NA
 
       NA
  
 
  
 1
 
    946675
 
    947343
 
  ENSEMBL   UCSC 
 
  669
 
  9
 
 2387
 
 island
 
 0.92853
 
 0.78518
 
 Not queried
 
  1.3610868
 
       NA
 
       NA
  
 
  
 6
 
  33147499
 
  33147659
 
  ENSEMBL   UCSC 
 
  161
 
  4
 
 34143
 
 gene
 
 0.64785
 
 0.41789
 
 Not queried
 
  1.3610053
 
       NA
 
       NA
  
 
  
 18
 
  55019621
 
  55019843
 
  ENSEMBL   UCSC 
 
  223
 
  5
 
 19497
 
 island
 
 0.11159
 
 0.08043
 
 Not queried
 
  1.3609424
 
       NA
 
       NA
  
 
  
 22
 
  36236649
 
  36236760
 
  ENSEMBL   UCSC 
 
  112
 
  4
 
 26813
 
 gene;tss
 
 0.24457
 
 0.06658
 
 Not queried
 
  1.3607947
 
       NA
 
       NA
  
 
  
 11
 
  70385301
 
  70385511
 
  ENSEMBL   UCSC 
 
  211
 
  4
 
 6773
 
 gene
 
 0.71237
 
 0.83387
 
 Not queried
 
  1.3600896
 
       NA
 
       NA
  
 
  
 4
 
 164253207
 
 164253656
 
  ENSEMBL   UCSC 
 
  450
 
  4
 
 30120
 
 gene
 
 0.02333
 
 0.00752
 
 Not queried
 
  1.3600721
 
       NA
 
       NA
  
 
  
 5
 
 134735544
 
 134735701
 
  ENSEMBL   UCSC 
 
  158
 
  7
 
 32244
 
 tss;island
 
 0.05398
 
 0.02208
 
 Not queried
 
  1.3600431
 
       NA
 
       NA
  
 
  
 6
 
  29855890
 
  29856938
 
  ENSEMBL   UCSC 
 
 1049
 
 29
 
 33637
 
 gene;island
 
 0.24612
 
 0.28608
 
 Not queried
 
  1.3600292
 
       NA
 
       NA
  
 
  
 7
 
    856023
 
    856295
 
  ENSEMBL   UCSC 
 
  273
 
  4
 
 37988
 
 island
 
 0.02544
 
 0.07491
 
 Not queried
 
  1.3593485
 
       NA
 
       NA
  
 
  
 3
 
 191048361
 
 191048439
 
  ENSEMBL   UCSC 
 
   79
 
  4
 
 28746
 
 tss
 
 0.01385
 
 0.03608
 
 Not queried
 
  1.3590381
 
       NA
 
       NA
  
 
  
 18
 
  44236847
 
  44237151
 
  ENSEMBL   UCSC 
 
  305
 
  8
 
 19456
 
 island
 
 0.65429
 
 0.87624
 
 Not queried
 
  1.3587769
 
       NA
 
       NA
  
 
  
 5
 
 118965093
 
 118965233
 
  ENSEMBL   UCSC 
 
  141
 
  4
 
 32180
 
 tss
 
 0.85669
 
 0.91645
 
 Not queried
 
  1.3583811
 
       NA
 
       NA
  
 
  
 16
 
  27414272
 
  27414536
 
  ENSEMBL   UCSC 
 
  265
 
  4
 
 14672
 
 gene
 
 0.67095
 
 0.87085
 
 Not queried
 
  1.3574522
 
       NA
 
       NA
  
 
  
 19
 
  20011538
 
  20011770
 
  ENSEMBL   UCSC 
 
  233
 
  4
 
 21795
 
 island
 
 0.05741
 
 0.02864
 
 Not queried
 
  1.3572448
 
       NA
 
       NA
  
 
  
 17
 
  33814163
 
  33814596
 
  ENSEMBL   UCSC 
 
  434
 
  5
 
 16486
 
 gene;island
 
 0.92601
 
 0.85380
 
 Not queried
 
  1.3572173
 
       NA
 
       NA
  
 
  
 15
 
  72978621
 
  72978892
 
  ENSEMBL   UCSC 
 
  272
 
  8
 
 13382
 
 gene;tss;island
 
 0.00730
 
 0.02114
 
 Not queried
 
  1.3569127
 
       NA
 
       NA
  
 
  
 17
 
  29717946
 
  29718306
 
  ENSEMBL   UCSC 
 
  361
 
  5
 
 17214
 
 tss;island
 
 0.32553
 
 0.15856
 
 Not queried
 
  1.3568506
 
       NA
 
       NA
  
 
  
 1
 
 156338639
 
 156339286
 
  ENSEMBL   UCSC 
 
  648
 
  8
 
 3842
 
 island
 
 0.59661
 
 0.32286
 
 Not queried
 
  1.3566945
 
       NA
 
       NA
  
 
  
 3
 
  99536008
 
  99536406
 
  ENSEMBL   UCSC 
 
  399
 
  7
 
 27832
 
 gene;tss;island
 
 0.01627
 
 0.02080
 
 Not queried
 
  1.3564985
 
       NA
 
       NA
  
 
  
 7
 
  37025706
 
  37025890
 
  ENSEMBL   UCSC 
 
  185
 
  4
 
 36904
 
 gene;tss
 
 0.81267
 
 0.92214
 
 Not queried
 
  1.3564819
 
       NA
 
       NA
  
 
  
 7
 
   1512940
 
   1513548
 
  ENSEMBL   UCSC 
 
  609
 
  5
 
 36692
 
 gene;island
 
 0.70944
 
 0.80755
 
 Not queried
 
  1.3554457
 
       NA
 
       NA
  
 
  
 6
 
 143999465
 
 143999715
 
  ENSEMBL   UCSC 
 
  251
 
  4
 
 34432
 
 gene;island
 
 0.01326
 
 0.01906
 
 Not queried
 
  1.3553657
 
       NA
 
       NA
  
 
  
 12
 
   6442873
 
   6443045
 
  ENSEMBL   UCSC 
 
  173
 
  4
 
 9001
 
 gene
 
 0.82138
 
 0.92902
 
 Not queried
 
  1.3551767
 
       NA
 
       NA
  
 
  
 5
 
  92931623
 
  92932120
 
  ENSEMBL   UCSC 
 
  498
 
  5
 
 32899
 
 island
 
 0.90109
 
 0.65766
 
 Not queried
 
  1.3549140
 
       NA
 
       NA
  
 
  
 22
 
  24236284
 
  24236549
 
  ENSEMBL   UCSC 
 
  266
 
  8
 
 26973
 
 tss
 
 0.05255
 
 0.01284
 
 Not queried
 
  1.3548034
 
       NA
 
       NA
  
 
  
 22
 
  24236284
 
  24236617
 
  ENSEMBL   UCSC 
 
  334
 
 10
 
 27314
 
 island
 
 0.05255
 
 0.01284
 
 Not queried
 
  1.3548034
 
       NA
 
       NA
  
 
  
 12
 
 111181439
 
 111181819
 
  ENSEMBL   UCSC 
 
  381
 
  4
 
 9985
 
 tss;island
 
 0.05056
 
 0.05398
 
 Not queried
 
  1.3546305
 
       NA
 
       NA
  
 
  
 X
 
   2825270
 
   2825596
 
  ENSEMBL   UCSC 
 
  327
 
  4
 
 40923
 
 gene;island
 
 0.88113
 
 0.94534
 
 Not queried
 
  1.3544158
 
       NA
 
       NA
  
 
  
 7
 
  81399458
 
  81399747
 
  ENSEMBL   UCSC 
 
  290
 
  4
 
 37611
 
 tss
 
 0.06773
 
 0.13092
 
 Not queried
 
  1.3543815
 
       NA
 
       NA
  
 
  
 7
 
 101768610
 
 101768874
 
  ENSEMBL   UCSC 
 
  265
 
  4
 
 37098
 
 gene
 
 0.34090
 
 0.55575
 
 Not queried
 
  1.3540115
 
       NA
 
       NA
  
 
  
 1
 
   3056542
 
   3056951
 
  ENSEMBL   UCSC 
 
  410
 
  7
 
 101
 
 gene;island
 
 0.63018
 
 0.84620
 
 Not queried
 
  1.3539910
 
       NA
 
       NA
  
 
  
 8
 
 134202337
 
 134202370
 
  ENSEMBL   UCSC 
 
   34
 
  4
 
 39857
 
 tss
 
 0.69807
 
 0.87570
 
 Not queried
 
  1.3533588
 
       NA
 
       NA
  
 
  
 1
 
 205818956
 
 205819251
 
  ENSEMBL   UCSC 
 
  296
 
  4
 
 967
 
 gene
 
 0.97637
 
 0.93175
 
 Not queried
 
  1.3532676
 
       NA
 
       NA
  
 
  
 1
 
 205818956
 
 205819609
 
  ENSEMBL   UCSC 
 
  654
 
  8
 
 4125
 
 island
 
 0.96982
 
 0.93175
 
 Not queried
 
  1.3532676
 
       NA
 
       NA
  
 
  
 19
 
    859330
 
    859554
 
  ENSEMBL   UCSC 
 
  225
 
  5
 
 20245
 
 tss
 
 0.80342
 
 0.66634
 
 Not queried
 
  1.3529134
 
       NA
 
       NA
  
 
  
 3
 
 179169536
 
 179169602
 
  ENSEMBL   UCSC 
 
   67
 
  5
 
 28689
 
 tss;island
 
 0.02663
 
 0.04615
 
 Not queried
 
  1.3524387
 
       NA
 
       NA
  
 
  
 12
 
  15475116
 
  15475342
 
  ENSEMBL   UCSC 
 
  227
 
  6
 
 9566
 
 tss
 
 0.14470
 
 0.05062
 
 Not queried
 
  1.3523169
 
       NA
 
       NA
  
 
  
 12
 
  15475116
 
  15475767
 
  ENSEMBL   UCSC 
 
  652
 
 10
 
 10243
 
 island
 
 0.10877
 
 0.03739
 
 Not queried
 
  1.3523169
 
       NA
 
       NA
  
 
  
 12
 
  54426498
 
  54427884
 
  ENSEMBL   UCSC 
 
 1387
 
 17
 
 9135
 
 gene;island
 
 0.02885
 
 0.15311
 
 Not queried
 
  1.3522216
 
       NA
 
       NA
  
 
  
 19
 
   2041905
 
   2042088
 
  ENSEMBL   UCSC 
 
  184
 
  5
 
 19641
 
 gene;island
 
 0.18151
 
 0.31110
 
 Not queried
 
  1.3519605
 
       NA
 
       NA
  
 
  
 7
 
  45039598
 
  45039799
 
  ENSEMBL   UCSC 
 
  202
 
  7
 
 36933
 
 gene;island
 
 0.00887
 
 0.02233
 
 Not queried
 
  1.3518338
 
       NA
 
       NA
  
 
  
 X
 
  13957159
 
  13957590
 
  ENSEMBL   UCSC 
 
  432
 
  4
 
 41194
 
 tss;island
 
 0.10002
 
 0.21780
 
 Not queried
 
  1.3517237
 
       NA
 
       NA
  
 
  
 1
 
   1683738
 
   1684095
 
  ENSEMBL   UCSC 
 
  358
 
  5
 
 47
 
 gene;island
 
 0.91838
 
 0.76688
 
 Not queried
 
  1.3514026
 
       NA
 
       NA
  
 
  
 1
 
 227750857
 
 227751214
 
  ENSEMBL   UCSC 
 
  358
 
  5
 
 2289
 
 tss
 
 0.92757
 
 0.70368
 
 Not queried
 
  1.3511594
 
       NA
 
       NA
  
 
  
 19
 
   1503042
 
   1503812
 
  ENSEMBL   UCSC 
 
  771
 
  7
 
 21192
 
 island
 
 0.15333
 
 0.44815
 
 Not queried
 
  1.3511290
 
       NA
 
       NA
  
 
  
 1
 
 115397374
 
 115397616
 
  ENSEMBL   UCSC 
 
  243
 
  5
 
 3617
 
 island
 
 0.67575
 
 0.40160
 
 Not queried
 
  1.3510838
 
       NA
 
       NA
  
 
  
 10
 
 134729942
 
 134730258
 
  ENSEMBL   UCSC 
 
  317
 
  5
 
 6298
 
 island
 
 0.57587
 
 0.89420
 
 Not queried
 
  1.3508292
 
       NA
 
       NA
  
 
  
 4
 
  39460910
 
  39461218
 
  ENSEMBL   UCSC 
 
  309
 
  4
 
 29937
 
 gene;tss;island
 
 0.00566
 
 0.01324
 
 Not queried
 
  1.3506227
 
       NA
 
       NA
  
 
  
 22
 
  19776050
 
  19776431
 
  ENSEMBL   UCSC 
 
  382
 
  4
 
 26750
 
 gene;island
 
 0.95561
 
 0.90364
 
 Not queried
 
  1.3497198
 
       NA
 
       NA
  
 
  
 1
 
 117664976
 
 117665186
 
  ENSEMBL   UCSC 
 
  211
 
  4
 
 1865
 
 tss;island
 
 0.02932
 
 0.07443
 
 Not queried
 
  1.3497073
 
       NA
 
       NA
  
 
  
 14
 
  35761165
 
  35761551
 
  ENSEMBL   UCSC 
 
  387
 
  6
 
 12324
 
 tss
 
 0.01136
 
 0.02438
 
 Not queried
 
  1.3496189
 
       NA
 
       NA
  
 
  
 X
 
 153046175
 
 153046451
 
  ENSEMBL   UCSC 
 
  277
 
  4
 
 41586
 
 tss
 
 0.65068
 
 0.86974
 
 Not queried
 
  1.3495756
 
       NA
 
       NA
  
 
  
 19
 
  49016564
 
  49016934
 
  ENSEMBL   UCSC 
 
  371
 
  6
 
 20881
 
 tss;island
 
 0.01967
 
 0.03163
 
 Not queried
 
  1.3491106
 
       NA
 
       NA
  
 
  
 17
 
  38257020
 
  38257323
 
  ENSEMBL   UCSC 
 
  304
 
  9
 
 17290
 
 tss
 
 0.04582
 
 0.02047
 
 Not queried
 
  1.3488512
 
       NA
 
       NA
  
 
  
 11
 
  67432681
 
  67433133
 
  ENSEMBL   UCSC 
 
  453
 
  5
 
 6745
 
 gene;island
 
 0.41221
 
 0.77120
 
 Not queried
 
  1.3487473
 
       NA
 
       NA
  
 
  
 6
 
  30594688
 
  30594955
 
  ENSEMBL   UCSC 
 
  268
 
  8
 
 33726
 
 gene
 
 0.02250
 
 0.08679
 
 Not queried
 
  1.3485217
 
       NA
 
       NA
  
 
  
 8
 
  63997977
 
  63998463
 
  ENSEMBL   UCSC 
 
  487
 
  5
 
 39257
 
 gene
 
 0.04033
 
 0.01291
 
 Not queried
 
  1.3483543
 
       NA
 
       NA
  
 
  
 3
 
 184430016
 
 184430477
 
  ENSEMBL   UCSC 
 
  462
 
  4
 
 28720
 
 tss;island
 
 0.17863
 
 0.05873
 
 Not queried
 
  1.3482229
 
       NA
 
       NA
  
 
  
 15
 
  52043486
 
  52043572
 
  ENSEMBL   UCSC 
 
   87
 
  5
 
 13324
 
 gene
 
 0.01267
 
 0.03410
 
 Not queried
 
  1.3481948
 
       NA
 
       NA
  
 
  
 13
 
  20702922
 
  20703169
 
  ENSEMBL   UCSC 
 
  248
 
  4
 
 11537
 
 island
 
 0.05786
 
 0.25320
 
 Not queried
 
  1.3481707
 
       NA
 
       NA
  
 
  
 14
 
  53620229
 
  53620401
 
  ENSEMBL   UCSC 
 
  173
 
  4
 
 12375
 
 tss;island
 
 0.20024
 
 0.10639
 
 Not queried
 
  1.3479891
 
       NA
 
       NA
  
 
  
 17
 
  37356273
 
  37356554
 
  ENSEMBL   UCSC 
 
  282
 
  4
 
 18238
 
 island
 
 0.01682
 
 0.00666
 
 Not queried
 
  1.3477744
 
       NA
 
       NA
  
 
  
 19
 
  15375283
 
  15375450
 
  ENSEMBL   UCSC 
 
  168
 
  8
 
 19868
 
 gene
 
 0.88511
 
 0.96538
 
 Not queried
 
  1.3477469
 
       NA
 
       NA
  
 
  
 2
 
  26541960
 
  26542188
 
  ENSEMBL   UCSC 
 
  229
 
  4
 
 22573
 
 gene;tss
 
 0.74057
 
 0.53634
 
 Not queried
 
  1.3476609
 
       NA
 
       NA
  
 
  
 15
 
  33360195
 
  33360485
 
  ENSEMBL   UCSC 
 
  291
 
  6
 
 13525
 
 tss
 
 0.42415
 
 0.62233
 
 Not queried
 
  1.3470892
 
       NA
 
       NA
  
 
  
 14
 
  24505537
 
  24505671
 
  ENSEMBL   UCSC 
 
  135
 
  5
 
 12281
 
 tss
 
 0.02469
 
 0.08085
 
 Not queried
 
  1.3470001
 
       NA
 
       NA
  
 
  
 5
 
 133451237
 
 133451625
 
  ENSEMBL   UCSC 
 
  389
 
  8
 
 31693
 
 gene;island
 
 0.21960
 
 0.03475
 
 Not queried
 
  1.3466809
 
       NA
 
       NA
  
 
  
 4
 
   1052372
 
   1052717
 
  ENSEMBL   UCSC 
 
  346
 
  4
 
 30718
 
 island
 
 0.76663
 
 0.87766
 
 Not queried
 
  1.3465609
 
       NA
 
       NA
  
 
  
 10
 
 126303581
 
 126303795
 
  ENSEMBL   UCSC 
 
  215
 
  4
 
 6212
 
 island
 
 0.77802
 
 0.89148
 
 Not queried
 
  1.3465039
 
       NA
 
       NA
  
 
  
 6
 
 100054723
 
 100055173
 
  ENSEMBL   UCSC 
 
  451
 
  5
 
 34362
 
 gene
 
 0.11676
 
 0.21288
 
 Not queried
 
  1.3464809
 
       NA
 
       NA
  
 
  
 6
 
  30910583
 
  30910646
 
  ENSEMBL   UCSC 
 
   64
 
  4
 
 33779
 
 gene
 
 0.02011
 
 0.08436
 
 Not queried
 
  1.3464466
 
       NA
 
       NA
  
 
  
 11
 
  20044428
 
  20044639
 
  ENSEMBL   UCSC 
 
  212
 
  4
 
 6518
 
 gene
 
 0.00876
 
 0.03036
 
 Not queried
 
  1.3464150
 
       NA
 
       NA
  
 
  
 5
 
  40756075
 
  40756512
 
  ENSEMBL   UCSC 
 
  438
 
  8
 
 31976
 
 tss
 
 0.02175
 
 0.05311
 
 Not queried
 
  1.3462381
 
       NA
 
       NA
  
 
  
 17
 
  46507690
 
  46507895
 
  ENSEMBL   UCSC 
 
  206
 
  7
 
 17422
 
 tss
 
 0.40546
 
 0.62937
 
 Not queried
 
  1.3461827
 
       NA
 
       NA
  
 
  
 11
 
  75046147
 
  75046354
 
  ENSEMBL   UCSC 
 
  208
 
  4
 
 6814
 
 gene
 
 0.29238
 
 0.45639
 
 Not queried
 
  1.3450568
 
       NA
 
       NA
  
 
  
 2
 
 217236425
 
 217237043
 
  ENSEMBL   UCSC 
 
  619
 
 14
 
 25034
 
 island
 
 0.02823
 
 0.07149
 
 Not queried
 
  1.3443232
 
       NA
 
       NA
  
 
  
 7
 
   1589295
 
   1589856
 
  ENSEMBL   UCSC 
 
  562
 
  6
 
 36699
 
 gene;island
 
 0.72877
 
 0.90182
 
 Not queried
 
  1.3435753
 
       NA
 
       NA
  
 
  
 1
 
 231471991
 
 231472445
 
  ENSEMBL   UCSC 
 
  455
 
  5
 
 1074
 
 gene;island
 
 0.25753
 
 0.08165
 
 Not queried
 
  1.3433975
 
       NA
 
       NA
  
 
  
 17
 
  66254884
 
  66255265
 
  ENSEMBL   UCSC 
 
  382
 
  6
 
 17573
 
 tss
 
 0.03804
 
 0.07487
 
 Not queried
 
  1.3429710
 
       NA
 
       NA
  
 
  
 2
 
  47382287
 
  47382462
 
  ENSEMBL   UCSC 
 
  176
 
  4
 
 22635
 
 gene
 
 0.78832
 
 0.91855
 
 Not queried
 
  1.3428163
 
       NA
 
       NA
  
 
  
 3
 
  74663507
 
  74663796
 
  ENSEMBL   UCSC 
 
  290
 
  4
 
 29244
 
 island
 
 0.02656
 
 0.04144
 
 Not queried
 
  1.3422422
 
       NA
 
       NA
  
 
  
 14
 
  50328815
 
  50328966
 
  ENSEMBL   UCSC 
 
  152
 
  4
 
 12798
 
 island
 
 0.01723
 
 0.04175
 
 Not queried
 
  1.3416633
 
       NA
 
       NA
  
 
  
 9
 
  99540324
 
  99540614
 
  ENSEMBL   UCSC 
 
  291
 
  4
 
 40853
 
 island
 
 0.01060
 
 0.02201
 
 Not queried
 
  1.3416373
 
       NA
 
       NA
  
 
  
 12
 
   1738035
 
   1738337
 
  ENSEMBL   UCSC 
 
  303
 
  6
 
 9461
 
 tss
 
 0.38285
 
 0.22105
 
 Not queried
 
  1.3415448
 
       NA
 
       NA
  
 
  
 6
 
  26032407
 
  26032585
 
  ENSEMBL   UCSC 
 
  179
 
  4
 
 34617
 
 tss
 
 0.01778
 
 0.05378
 
 Not queried
 
  1.3414124
 
       NA
 
       NA
  
 
  
 4
 
 122854168
 
 122854532
 
  ENSEMBL   UCSC 
 
  365
 
  5
 
 30071
 
 gene;island
 
 0.77007
 
 0.93691
 
 Not queried
 
  1.3413380
 
       NA
 
       NA
  
 
  
 1
 
    983260
 
    983611
 
  ENSEMBL   UCSC 
 
  352
 
  4
 
 10
 
 gene;island
 
 0.92543
 
 0.85350
 
 Not queried
 
  1.3412519
 
       NA
 
       NA
  
 
  
 11
 
 120008025
 
 120008607
 
  ENSEMBL   UCSC 
 
  583
 
  6
 
 6923
 
 gene;island
 
 0.88140
 
 0.72590
 
 Not queried
 
  1.3409833
 
       NA
 
       NA
  
 
  
 10
 
 102440587
 
 102440894
 
  ENSEMBL   UCSC 
 
  308
 
  4
 
 6011
 
 island
 
 0.01430
 
 0.06234
 
 Not queried
 
  1.3408482
 
       NA
 
       NA
  
 
  
 6
 
 169026928
 
 169027218
 
  ENSEMBL   UCSC 
 
  291
 
  5
 
 34500
 
 gene
 
 0.89587
 
 0.77630
 
 Not queried
 
  1.3406347
 
       NA
 
       NA
  
 
  
 7
 
  27209195
 
  27209582
 
  ENSEMBL   UCSC 
 
  388
 
  9
 
 37432
 
 tss
 
 0.03521
 
 0.12436
 
 Not queried
 
  1.3405293
 
       NA
 
       NA
  
 
  
 22
 
  32598479
 
  32598716
 
  ENSEMBL   UCSC 
 
  238
 
  4
 
 26804
 
 gene
 
 0.16557
 
 0.30196
 
 Not queried
 
  1.3402453
 
       NA
 
       NA
  
 
  
 22
 
  30476089
 
  30476345
 
  ENSEMBL   UCSC 
 
  257
 
  9
 
 27013
 
 tss
 
 0.06581
 
 0.15336
 
 Not queried
 
  1.3396398
 
       NA
 
       NA
  
 
  
 19
 
   2302783
 
   2303032
 
  ENSEMBL   UCSC 
 
  250
 
  4
 
 19651
 
 gene;island
 
 0.85447
 
 0.90084
 
 Not queried
 
  1.3392196
 
       NA
 
       NA
  
 
  
 15
 
  89148597
 
  89148733
 
  ENSEMBL   UCSC 
 
  137
 
  4
 
 14391
 
 island
 
 0.02161
 
 0.05828
 
 Not queried
 
  1.3392029
 
       NA
 
       NA
  
 
  
 6
 
  28543508
 
  28543693
 
  ENSEMBL   UCSC 
 
  186
 
  5
 
 33573
 
 gene
 
 0.96475
 
 0.94408
 
 Not queried
 
  1.3388357
 
       NA
 
       NA
  
 
  
 19
 
  54515169
 
  54515341
 
  ENSEMBL   UCSC 
 
  173
 
  4
 
 20176
 
 gene;island
 
 0.52341
 
 0.70473
 
 Not queried
 
  1.3384475
 
       NA
 
       NA
  
 
  
 19
 
   2650727
 
   2650863
 
  ENSEMBL   UCSC 
 
  137
 
  4
 
 19664
 
 gene;island
 
 0.52412
 
 0.71921
 
 Not queried
 
  1.3372651
 
       NA
 
       NA
  
 
  
 6
 
  30124647
 
  30124942
 
  ENSEMBL   UCSC 
 
  296
 
  7
 
 33669
 
 gene
 
 0.59502
 
 0.71222
 
 Not queried
 
  1.3372347
 
       NA
 
       NA
  
 
  
 2
 
  69614451
 
  69614473
 
  ENSEMBL   UCSC 
 
   23
 
  5
 
 23366
 
 tss
 
 0.00547
 
 0.01369
 
 Not queried
 
  1.3367900
 
       NA
 
       NA
  
 
  
 16
 
  12070223
 
  12070416
 
  ENSEMBL   UCSC 
 
  194
 
  4
 
 15066
 
 tss;island
 
 0.01905
 
 0.03732
 
 Not queried
 
  1.3364459
 
       NA
 
       NA
  
 
  
 6
 
 170062623
 
 170063076
 
  ENSEMBL   UCSC 
 
  454
 
  4
 
 34508
 
 gene;island
 
 0.93861
 
 0.85433
 
 Not queried
 
  1.3363638
 
       NA
 
       NA
  
 
  
 2
 
 176987161
 
 176987605
 
  ENSEMBL   UCSC 
 
  445
 
  8
 
 24863
 
 island
 
 0.01154
 
 0.03073
 
 Not queried
 
  1.3363217
 
       NA
 
       NA
  
 
  
 19
 
  19648890
 
  19649144
 
  ENSEMBL   UCSC 
 
  255
 
  8
 
 21786
 
 island
 
 0.22165
 
 0.11091
 
 Not queried
 
  1.3361326
 
       NA
 
       NA
  
 
  
 8
 
   1921165
 
   1921906
 
  ENSEMBL   UCSC 
 
  742
 
  7
 
 39473
 
 tss;island
 
 0.04211
 
 0.06242
 
 Not queried
 
  1.3360112
 
       NA
 
       NA
  
 
  
 1
 
 162467180
 
 162467625
 
  ENSEMBL   UCSC 
 
  446
 
  7
 
 2054
 
 tss
 
 0.36814
 
 0.33031
 
 Not queried
 
  1.3356936
 
       NA
 
       NA
  
 
  
 10
 
  46971222
 
  46971658
 
  ENSEMBL   UCSC 
 
  437
 
  4
 
 5045
 
 tss;island
 
 0.56326
 
 0.87986
 
 Not queried
 
  1.3356014
 
       NA
 
       NA
  
 
  
 3
 
 138153127
 
 138153439
 
  ENSEMBL   UCSC 
 
  313
 
  9
 
 29478
 
 island
 
 0.17177
 
 0.07572
 
 Not queried
 
  1.3350464
 
       NA
 
       NA
  
 
  
 X
 
  14048019
 
  14048281
 
  ENSEMBL   UCSC 
 
  263
 
  9
 
 41652
 
 island
 
 0.02246
 
 0.03540
 
 Not queried
 
  1.3348828
 
       NA
 
       NA
  
 
  
 19
 
  39574483
 
  39575027
 
  ENSEMBL   UCSC 
 
  545
 
 10
 
 21953
 
 island
 
 0.15454
 
 0.26187
 
 Not queried
 
  1.3347885
 
       NA
 
       NA
  
 
  
 4
 
 140201537
 
 140201652
 
  ENSEMBL   UCSC 
 
  116
 
  5
 
 30549
 
 tss
 
 0.30454
 
 0.17969
 
 Not queried
 
  1.3347494
 
       NA
 
       NA
  
 
  
 14
 
  63670711
 
  63671126
 
  ENSEMBL   UCSC 
 
  416
 
  4
 
 12420
 
 tss
 
 0.47612
 
 0.24426
 
 Not queried
 
  1.3342739
 
       NA
 
       NA
  
 
  
 22
 
  20792217
 
  20792535
 
  ENSEMBL   UCSC 
 
  319
 
  8
 
 26938
 
 tss
 
 0.02635
 
 0.02068
 
 Not queried
 
  1.3335939
 
       NA
 
       NA
  
 
  
 19
 
  19932565
 
  19932702
 
  ENSEMBL   UCSC 
 
  138
 
  6
 
 20617
 
 tss
 
 0.55666
 
 0.35918
 
 Not queried
 
  1.3333135
 
       NA
 
       NA
  
 
  
 5
 
 140998629
 
 140998650
 
  ENSEMBL   UCSC 
 
   22
 
  4
 
 32327
 
 tss
 
 0.03083
 
 0.07405
 
 Not queried
 
  1.3332498
 
       NA
 
       NA
  
 
  
 2
 
  11809681
 
  11810683
 
  ENSEMBL   UCSC 
 
 1003
 
 12
 
 24044
 
 island
 
 0.20472
 
 0.33930
 
 Not queried
 
  1.3329555
 
       NA
 
       NA
  
 
  
 10
 
 118030848
 
 118031256
 
  ENSEMBL   UCSC 
 
  409
 
  4
 
 4757
 
 gene;island
 
 0.14328
 
 0.05708
 
 Not queried
 
  1.3324135
 
       NA
 
       NA
  
 
  
 5
 
  60139131
 
  60139708
 
  ENSEMBL   UCSC 
 
  578
 
  5
 
 31569
 
 gene;island
 
 0.01643
 
 0.04036
 
 Not queried
 
  1.3319730
 
       NA
 
       NA
  
 
  
 17
 
  33923795
 
  33924143
 
  ENSEMBL   UCSC 
 
  349
 
  4
 
 16488
 
 gene;island
 
 0.55682
 
 0.77764
 
 Not queried
 
  1.3318566
 
       NA
 
       NA
  
 
  
 11
 
   1092135
 
   1092321
 
  ENSEMBL   UCSC 
 
  187
 
  6
 
 6385
 
 gene;island
 
 0.84639
 
 0.91614
 
 Not queried
 
  1.3317802
 
       NA
 
       NA
  
 
  
 8
 
  65488640
 
  65489065
 
  ENSEMBL   UCSC 
 
  426
 
  5
 
 39259
 
 gene;island
 
 0.08233
 
 0.28287
 
 Not queried
 
  1.3317263
 
       NA
 
       NA
  
 
  
 4
 
  94748617
 
  94748860
 
  ENSEMBL   UCSC 
 
  244
 
  4
 
 30452
 
 tss;island
 
 0.06926
 
 0.02859
 
 Not queried
 
  1.3312395
 
       NA
 
       NA
  
 
  
 11
 
    811545
 
    811669
 
  ENSEMBL   UCSC 
 
  125
 
  4
 
 7010
 
 tss
 
 0.65840
 
 0.80462
 
 Not queried
 
  1.3306078
 
       NA
 
       NA
  
 
  
 6
 
  31860644
 
  31860832
 
  ENSEMBL   UCSC 
 
  189
 
  6
 
 33925
 
 gene;island
 
 0.79865
 
 0.90459
 
 Not queried
 
  1.3300451
 
       NA
 
       NA
  
 
  
 6
 
  32551749
 
  32552547
 
  ENSEMBL   UCSC 
 
  799
 
 14
 
 34056
 
 gene;island
 
 0.20416
 
 0.38412
 
 Not queried
 
  1.3298679
 
       NA
 
       NA
  
 
  
 20
 
  30062970
 
  30063038
 
  ENSEMBL   UCSC 
 
   69
 
  6
 
 25620
 
 tss
 
 0.05519
 
 0.03905
 
 Not queried
 
  1.3297473
 
       NA
 
       NA
  
 
  
 3
 
 113234057
 
 113234093
 
  ENSEMBL   UCSC 
 
   37
 
  4
 
 28464
 
 tss
 
 0.00803
 
 0.02291
 
 Not queried
 
  1.3290796
 
       NA
 
       NA
  
 
  
 2
 
  45168798
 
  45168924
 
  ENSEMBL   UCSC 
 
  127
 
  4
 
 23295
 
 tss;island
 
 0.60905
 
 0.36214
 
 Not queried
 
  1.3286068
 
       NA
 
       NA
  
 
  
 12
 
   9227173
 
   9227320
 
  ENSEMBL   UCSC 
 
  148
 
  5
 
 9026
 
 gene
 
 0.44702
 
 0.66996
 
 Not queried
 
  1.3283221
 
       NA
 
       NA
  
 
  
 6
 
 132272545
 
 132272790
 
  ENSEMBL   UCSC 
 
  246
 
  4
 
 35161
 
 tss
 
 0.01252
 
 0.03053
 
 Not queried
 
  1.3281929
 
       NA
 
       NA
  
 
  
 6
 
 132272342
 
 132272790
 
  ENSEMBL   UCSC 
 
  449
 
  6
 
 36419
 
 island
 
 0.01252
 
 0.03053
 
 Not queried
 
  1.3281929
 
       NA
 
       NA
  
 
  
 7
 
 156332539
 
 156332978
 
  ENSEMBL   UCSC 
 
  440
 
  4
 
 37951
 
 tss
 
 0.47713
 
 0.64112
 
 Not queried
 
  1.3273260
 
       NA
 
       NA
  
 
  
 19
 
  13875014
 
  13875329
 
  ENSEMBL   UCSC 
 
  316
 
  8
 
 20492
 
 tss
 
 0.04881
 
 0.16978
 
 Not queried
 
  1.3265940
 
       NA
 
       NA
  
 
  
 19
 
  13875014
 
  13875430
 
  ENSEMBL   UCSC 
 
  417
 
 10
 
 21618
 
 island
 
 0.03415
 
 0.13892
 
 Not queried
 
  1.3265940
 
       NA
 
       NA
  
 
  
 19
 
  40503048
 
  40503358
 
  ENSEMBL   UCSC 
 
  311
 
  4
 
 19997
 
 gene
 
 0.09844
 
 0.04530
 
 Not queried
 
  1.3263045
 
       NA
 
       NA
  
 
  
 8
 
  74205400
 
  74205666
 
  ENSEMBL   UCSC 
 
  267
 
  4
 
 39273
 
 gene;island
 
 0.03558
 
 0.08392
 
 Not queried
 
  1.3261136
 
       NA
 
       NA
  
 
  
 6
 
  70506422
 
  70506998
 
  ENSEMBL   UCSC 
 
  577
 
  8
 
 34315
 
 gene;island
 
 0.06728
 
 0.02472
 
 Not queried
 
  1.3260857
 
       NA
 
       NA
  
 
  
 17
 
  18684650
 
  18685008
 
  ENSEMBL   UCSC 
 
  359
 
  4
 
 16439
 
 gene
 
 0.03279
 
 0.01499
 
 Not queried
 
  1.3260567
 
       NA
 
       NA
  
 
  
 4
 
 165952731
 
 165953131
 
  ENSEMBL   UCSC 
 
  401
 
  8
 
 30619
 
 tss
 
 0.84557
 
 0.66818
 
 Not queried
 
  1.3260331
 
       NA
 
       NA
  
 
  
 2
 
  54087173
 
  54087552
 
  ENSEMBL   UCSC 
 
  380
 
 10
 
 23314
 
 tss
 
 0.80742
 
 0.92146
 
 Not queried
 
  1.3258909
 
       NA
 
       NA
  
 
  
 4
 
   4866454
 
   4867164
 
  ENSEMBL   UCSC 
 
  711
 
  6
 
 30823
 
 island
 
 0.04688
 
 0.12876
 
 Not queried
 
  1.3252101
 
       NA
 
       NA
  
 
  
 1
 
 180992098
 
 180992117
 
  ENSEMBL   UCSC 
 
   20
 
  4
 
 2123
 
 tss
 
 0.01445
 
 0.03456
 
 Not queried
 
  1.3248531
 
       NA
 
       NA
  
 
  
 5
 
  41925698
 
  41925854
 
  ENSEMBL   UCSC 
 
  157
 
  4
 
 31544
 
 gene;island
 
 0.01247
 
 0.02501
 
 Not queried
 
  1.3247082
 
       NA
 
       NA
  
 
  
 6
 
  28481660
 
  28482191
 
  ENSEMBL   UCSC 
 
  532
 
  6
 
 33570
 
 gene
 
 0.83512
 
 0.66854
 
 Not queried
 
  1.3245481
 
       NA
 
       NA
  
 
  
 4
 
    979022
 
    979389
 
  ENSEMBL   UCSC 
 
  368
 
  6
 
 29807
 
 gene;island
 
 0.44589
 
 0.14000
 
 Not queried
 
  1.3245242
 
       NA
 
       NA
  
 
  
 2
 
  60780415
 
  60780483
 
  ENSEMBL   UCSC 
 
   69
 
  4
 
 22657
 
 gene;island
 
 0.02377
 
 0.22869
 
 Not queried
 
  1.3239692
 
       NA
 
       NA
  
 
  
 8
 
   1785833
 
   1786322
 
  ENSEMBL   UCSC 
 
  490
 
  6
 
 39113
 
 gene
 
 0.68648
 
 0.88593
 
 Not queried
 
  1.3238270
 
       NA
 
       NA
  
 
  
 4
 
 124320678
 
 124321007
 
  ENSEMBL   UCSC 
 
  330
 
  4
 
 30076
 
 gene;island
 
 0.01304
 
 0.03241
 
 Not queried
 
  1.3237962
 
       NA
 
       NA
  
 
  
 16
 
  29822529
 
  29822912
 
  ENSEMBL   UCSC 
 
  384
 
  4
 
 15138
 
 tss;island
 
 0.03086
 
 0.08538
 
 Not queried
 
  1.3236597
 
       NA
 
       NA
  
 
  
 3
 
  62861055
 
  62861311
 
  ENSEMBL   UCSC 
 
  257
 
  7
 
 29209
 
 island
 
 0.02394
 
 0.07906
 
 Not queried
 
  1.3235744
 
       NA
 
       NA
  
 
  
 15
 
  42289618
 
  42289939
 
  ENSEMBL   UCSC 
 
  322
 
  4
 
 13277
 
 gene
 
 0.38417
 
 0.73566
 
 Not queried
 
  1.3232678
 
       NA
 
       NA
  
 
  
 6
 
  29760410
 
  29760832
 
  ENSEMBL   UCSC 
 
  423
 
 23
 
 33631
 
 gene
 
 0.02903
 
 0.07896
 
 Not queried
 
  1.3231273
 
       NA
 
       NA
  
 
  
 6
 
  29760410
 
  29761044
 
  ENSEMBL   UCSC 
 
  635
 
 29
 
 35568
 
 island
 
 0.04830
 
 0.08832
 
 Not queried
 
  1.3231273
 
       NA
 
       NA
  
 
  
 6
 
  15246396
 
  15246613
 
  ENSEMBL   UCSC 
 
  218
 
  4
 
 35402
 
 island
 
 0.01949
 
 0.01286
 
 Not queried
 
  1.3229460
 
       NA
 
       NA
  
 
  
 17
 
   7475727
 
   7476167
 
  ENSEMBL   UCSC 
 
  441
 
 11
 
 17924
 
 island
 
 0.02044
 
 0.04673
 
 Not queried
 
  1.3229031
 
       NA
 
       NA
  
 
  
 5
 
 140430569
 
 140430962
 
  ENSEMBL   UCSC 
 
  394
 
  6
 
 32301
 
 tss
 
 0.14232
 
 0.05984
 
 Not queried
 
  1.3228756
 
       NA
 
       NA
  
 
  
 7
 
   2328746
 
   2329077
 
  ENSEMBL   UCSC 
 
  332
 
  4
 
 36737
 
 gene
 
 0.80985
 
 0.94220
 
 Not queried
 
  1.3223758
 
       NA
 
       NA
  
 
  
 10
 
 102809954
 
 102810157
 
  ENSEMBL   UCSC 
 
  204
 
  5
 
 6030
 
 island
 
 0.03801
 
 0.01691
 
 Not queried
 
  1.3222921
 
       NA
 
       NA
  
 
  
 10
 
  11206772
 
  11206892
 
  ENSEMBL   UCSC 
 
  121
 
  7
 
 4928
 
 tss
 
 0.19684
 
 0.05991
 
 Not queried
 
  1.3215616
 
       NA
 
       NA
  
 
  
 6
 
 110299835
 
 110300308
 
  ENSEMBL   UCSC 
 
  474
 
  8
 
 36348
 
 island
 
 0.05067
 
 0.05333
 
 Not queried
 
  1.3212732
 
       NA
 
       NA
  
 
  
 8
 
  25902400
 
  25902611
 
  ENSEMBL   UCSC 
 
  212
 
  5
 
 39544
 
 tss
 
 0.02033
 
 0.03742
 
 Not queried
 
  1.3212679
 
       NA
 
       NA
  
 
  
 X
 
   9001125
 
   9001351
 
  ENSEMBL   UCSC 
 
  227
 
  4
 
 41176
 
 tss
 
 0.90643
 
 0.95051
 
 Not queried
 
  1.3211163
 
       NA
 
       NA
  
 
  
 8
 
 142139654
 
 142140021
 
  ENSEMBL   UCSC 
 
  368
 
  4
 
 39381
 
 gene;island
 
 0.06114
 
 0.11483
 
 Not queried
 
  1.3210610
 
       NA
 
       NA
  
 
  
 X
 
  48367969
 
  48368144
 
  ENSEMBL   UCSC 
 
  176
 
  6
 
 41291
 
 tss
 
 0.57072
 
 0.42494
 
 Not queried
 
  1.3209647
 
       NA
 
       NA
  
 
  
 7
 
  27143046
 
  27143806
 
  ENSEMBL   UCSC 
 
  761
 
 12
 
 37423
 
 tss;island
 
 0.12858
 
 0.23469
 
 Not queried
 
  1.3205155
 
       NA
 
       NA
  
 
  
 7
 
  27224092
 
  27225897
 
  ENSEMBL   UCSC 
 
 1806
 
 34
 
 37434
 
 tss;island
 
 0.05479
 
 0.13893
 
 Not queried
 
  1.3202152
 
       NA
 
       NA
  
 
  
 6
 
  43211208
 
  43211491
 
  ENSEMBL   UCSC 
 
  284
 
  5
 
 36096
 
 island
 
 0.03099
 
 0.28640
 
 Not queried
 
  1.3197133
 
       NA
 
       NA
  
 
  
 10
 
 102985022
 
 102985514
 
  ENSEMBL   UCSC 
 
  493
 
  4
 
 6040
 
 island
 
 0.50025
 
 0.29236
 
 Not queried
 
  1.3195978
 
       NA
 
       NA
  
 
  
 11
 
  32458656
 
  32459057
 
  ENSEMBL   UCSC 
 
  402
 
  5
 
 6550
 
 gene;island
 
 0.06769
 
 0.14413
 
 Not queried
 
  1.3195886
 
       NA
 
       NA
  
 
  
 8
 
  50823124
 
  50823541
 
  ENSEMBL   UCSC 
 
  418
 
  6
 
 39630
 
 tss;island
 
 0.04933
 
 0.10130
 
 Not queried
 
  1.3195498
 
       NA
 
       NA
  
 
  
 6
 
  33241226
 
  33241770
 
  ENSEMBL   UCSC 
 
  545
 
  5
 
 34180
 
 gene;island
 
 0.84964
 
 0.69371
 
 Not queried
 
  1.3189601
 
       NA
 
       NA
  
 
  
 20
 
   2633258
 
   2633362
 
  ENSEMBL   UCSC 
 
  105
 
  4
 
 25296
 
 gene
 
 0.03091
 
 0.06450
 
 Not queried
 
  1.3185830
 
       NA
 
       NA
  
 
  
 20
 
   2632842
 
   2633362
 
  ENSEMBL   UCSC 
 
  521
 
  8
 
 25508
 
 tss;island
 
 0.03091
 
 0.05668
 
 Not queried
 
  1.3185830
 
       NA
 
       NA
  
 
  
 10
 
 133644553
 
 133644760
 
  ENSEMBL   UCSC 
 
  208
 
  4
 
 6260
 
 island
 
 0.62077
 
 0.77875
 
 Not queried
 
  1.3184683
 
       NA
 
       NA
  
 
  
 5
 
 158533621
 
 158533942
 
  ENSEMBL   UCSC 
 
  322
 
  5
 
 33288
 
 island
 
 0.01514
 
 0.09503
 
 Not queried
 
  1.3183351
 
       NA
 
       NA
  
 
  
 19
 
   1812282
 
   1812854
 
  ENSEMBL   UCSC 
 
  573
 
  6
 
 20275
 
 tss;island
 
 0.02829
 
 0.10260
 
 Not queried
 
  1.3180219
 
       NA
 
       NA
  
 
  
 19
 
  14183619
 
  14183792
 
  ENSEMBL   UCSC 
 
  174
 
  5
 
 20501
 
 tss;island
 
 0.18327
 
 0.12160
 
 Not queried
 
  1.3179789
 
       NA
 
       NA
  
 
  
 12
 
  24737149
 
  24737423
 
  ENSEMBL   UCSC 
 
  275
 
  6
 
 9582
 
 tss
 
 0.78301
 
 0.87810
 
 Not queried
 
  1.3179251
 
       NA
 
       NA
  
 
  
 11
 
  64084966
 
  64085131
 
  ENSEMBL   UCSC 
 
  166
 
  5
 
 7374
 
 tss;island
 
 0.01567
 
 0.02406
 
 Not queried
 
  1.3177833
 
       NA
 
       NA
  
 
  
 17
 
  43318610
 
  43318900
 
  ENSEMBL   UCSC 
 
  291
 
  4
 
 16598
 
 gene;island
 
 0.06383
 
 0.15317
 
 Not queried
 
  1.3172147
 
       NA
 
       NA
  
 
  
 11
 
 124767840
 
 124768015
 
  ENSEMBL   UCSC 
 
  176
 
  5
 
 7765
 
 tss
 
 0.88997
 
 0.64855
 
 Not queried
 
  1.3170547
 
       NA
 
       NA
  
 
  
 6
 
  26031939
 
  26032585
 
  ENSEMBL   UCSC 
 
  647
 
  9
 
 35445
 
 island
 
 0.02269
 
 0.05427
 
 Not queried
 
  1.3164964
 
       NA
 
       NA
  
 
  
 6
 
  33041218
 
  33041343
 
  ENSEMBL   UCSC 
 
  126
 
  5
 
 34119
 
 gene
 
 0.84778
 
 0.52327
 
 Not queried
 
  1.3161300
 
       NA
 
       NA
  
 
  
 8
 
 140716970
 
 140717372
 
  ENSEMBL   UCSC 
 
  403
 
  6
 
 40541
 
 island
 
 0.32546
 
 0.16299
 
 Not queried
 
  1.3156799
 
       NA
 
       NA
  
 
  
 17
 
   3697993
 
   3698239
 
  ENSEMBL   UCSC 
 
  247
 
  4
 
 16310
 
 gene
 
 0.34754
 
 0.60763
 
 Not queried
 
  1.3150737
 
       NA
 
       NA
  
 
  
 1
 
  43389643
 
  43389888
 
  ENSEMBL   UCSC 
 
  246
 
  5
 
 3148
 
 island
 
 0.04395
 
 0.05927
 
 Not queried
 
  1.3150227
 
       NA
 
       NA
  
 
  
 1
 
 205819406
 
 205819609
 
  ENSEMBL   UCSC 
 
  204
 
  4
 
 2206
 
 tss
 
 0.95767
 
 0.92530
 
 Not queried
 
  1.3149195
 
       NA
 
       NA
  
 
  
 10
 
 129924035
 
 129924374
 
  ENSEMBL   UCSC 
 
  340
 
  5
 
 4807
 
 gene
 
 0.00837
 
 0.01364
 
 Not queried
 
  1.3146894
 
       NA
 
       NA
  
 
  
 5
 
  16701194
 
  16701581
 
  ENSEMBL   UCSC 
 
  388
 
  5
 
 31512
 
 gene;island
 
 0.97198
 
 0.93155
 
 Not queried
 
  1.3144673
 
       NA
 
       NA
  
 
  
 1
 
  29557611
 
  29557820
 
  ENSEMBL   UCSC 
 
  210
 
  5
 
 1438
 
 tss
 
 0.01537
 
 0.04446
 
 Not queried
 
  1.3142208
 
       NA
 
       NA
  
 
  
 11
 
  94276895
 
  94277190
 
  ENSEMBL   UCSC 
 
  296
 
  6
 
 8716
 
 island
 
 0.12295
 
 0.04366
 
 Not queried
 
  1.3131640
 
       NA
 
       NA
  
 
  
 19
 
  51411737
 
  51411940
 
  ENSEMBL   UCSC 
 
  204
 
  4
 
 20148
 
 gene;island
 
 0.66663
 
 0.44674
 
 Not queried
 
  1.3129368
 
       NA
 
       NA
  
 
  
 8
 
  38239693
 
  38239917
 
  ENSEMBL   UCSC 
 
  225
 
  4
 
 40167
 
 island
 
 0.02731
 
 0.11192
 
 Not queried
 
  1.3125272
 
       NA
 
       NA
  
 
  
 17
 
  57408935
 
  57409092
 
  ENSEMBL   UCSC 
 
  158
 
  6
 
 18576
 
 island
 
 0.01103
 
 0.01596
 
 Not queried
 
  1.3124051
 
       NA
 
       NA
  
 
  
 17
 
   6358363
 
   6358599
 
  ENSEMBL   UCSC 
 
  237
 
  4
 
 16337
 
 gene;island
 
 0.46676
 
 0.70493
 
 Not queried
 
  1.3123878
 
       NA
 
       NA
  
 
  
 13
 
  99174312
 
  99174609
 
  ENSEMBL   UCSC 
 
  298
 
  6
 
 11174
 
 gene
 
 0.78309
 
 0.89615
 
 Not queried
 
  1.3121322
 
       NA
 
       NA
  
 
  
 5
 
 175108269
 
 175108429
 
  ENSEMBL   UCSC 
 
  161
 
  4
 
 31848
 
 gene;tss
 
 0.68202
 
 0.79672
 
 Not queried
 
  1.3120489
 
       NA
 
       NA
  
 
  
 6
 
 168920172
 
 168920331
 
  ENSEMBL   UCSC 
 
  160
 
  4
 
 34496
 
 gene;island
 
 0.94190
 
 0.86523
 
 Not queried
 
  1.3120284
 
       NA
 
       NA
  
 
  
 13
 
 111358516
 
 111358552
 
  ENSEMBL   UCSC 
 
   37
 
  6
 
 11507
 
 tss;island
 
 0.01481
 
 0.01993
 
 Not queried
 
  1.3113326
 
       NA
 
       NA
  
 
  
 2
 
  73964413
 
  73964834
 
  ENSEMBL   UCSC 
 
  422
 
  9
 
 24379
 
 island
 
 0.12279
 
 0.19270
 
 Not queried
 
  1.3112054
 
       NA
 
       NA
  
 
  
 20
 
    170364
 
    170641
 
  ENSEMBL   UCSC 
 
  278
 
  5
 
 25482
 
 tss
 
 0.74228
 
 0.32891
 
 Not queried
 
  1.3105758
 
       NA
 
       NA
  
 
  
 16
 
  67997858
 
  67998246
 
  ENSEMBL   UCSC 
 
  389
 
  5
 
 14780
 
 gene
 
 0.75767
 
 0.57877
 
 Not queried
 
  1.3102274
 
       NA
 
       NA
  
 
  
 17
 
  80251443
 
  80251697
 
  ENSEMBL   UCSC 
 
  255
 
  4
 
 18949
 
 island
 
 0.61143
 
 0.40698
 
 Not queried
 
  1.3096841
 
       NA
 
       NA
  
 
  
 12
 
   6420020
 
   6420052
 
  ENSEMBL   UCSC 
 
   33
 
  6
 
 8998
 
 gene;tss;island
 
 0.03104
 
 0.06858
 
 Not queried
 
  1.3088755
 
       NA
 
       NA
  
 
  
 1
 
 152010110
 
 152010183
 
  ENSEMBL   UCSC 
 
   74
 
  4
 
 1933
 
 tss;island
 
 0.85512
 
 0.70470
 
 Not queried
 
  1.3085192
 
       NA
 
       NA
  
 
  
 2
 
 211090247
 
 211090487
 
  ENSEMBL   UCSC 
 
  241
 
  6
 
 23816
 
 tss
 
 0.01832
 
 0.04256
 
 Not queried
 
  1.3082485
 
       NA
 
       NA
  
 
  
 1
 
  32410413
 
  32410678
 
  ENSEMBL   UCSC 
 
  266
 
  4
 
 2973
 
 island
 
 0.19108
 
 0.06460
 
 Not queried
 
  1.3081936
 
       NA
 
       NA
  
 
  
 19
 
  10691765
 
  10692029
 
  ENSEMBL   UCSC 
 
  265
 
  6
 
 19780
 
 gene;island
 
 0.20599
 
 0.02731
 
 Not queried
 
  1.3081466
 
       NA
 
       NA
  
 
  
 20
 
  56323934
 
  56324226
 
  ENSEMBL   UCSC 
 
  293
 
  4
 
 26245
 
 island
 
 0.47159
 
 0.73120
 
 Not queried
 
  1.3081110
 
       NA
 
       NA
  
 
  
 19
 
  42636639
 
  42637295
 
  ENSEMBL   UCSC 
 
  657
 
  6
 
 20783
 
 tss
 
 0.59516
 
 0.85661
 
 Not queried
 
  1.3080058
 
       NA
 
       NA
  
 
  
 15
 
  89920824
 
  89921258
 
  ENSEMBL   UCSC 
 
  435
 
 10
 
 13859
 
 tss;island
 
 0.92337
 
 0.79192
 
 Not queried
 
  1.3079515
 
       NA
 
       NA
  
 
  
 11
 
  57508416
 
  57508677
 
  ENSEMBL   UCSC 
 
  262
 
  7
 
 7288
 
 tss
 
 0.04071
 
 0.13817
 
 Not queried
 
  1.3079006
 
       NA
 
       NA
  
 
  
 11
 
  57508416
 
  57509018
 
  ENSEMBL   UCSC 
 
  603
 
  9
 
 8271
 
 island
 
 0.03639
 
 0.07005
 
 Not queried
 
  1.3079006
 
       NA
 
       NA
  
 
  
 19
 
  18699118
 
  18699444
 
  ENSEMBL   UCSC 
 
  327
 
  9
 
 20587
 
 tss
 
 0.04657
 
 0.03205
 
 Not queried
 
  1.3077835
 
       NA
 
       NA
  
 
  
 13
 
  24144483
 
  24144682
 
  ENSEMBL   UCSC 
 
  200
 
  4
 
 11312
 
 tss
 
 0.18859
 
 0.07209
 
 Not queried
 
  1.3072275
 
       NA
 
       NA
  
 
  
 6
 
 126964727
 
 126965162
 
  ENSEMBL   UCSC 
 
  436
 
  5
 
 36405
 
 island
 
 0.46029
 
 0.82684
 
 Not queried
 
  1.3071578
 
       NA
 
       NA
  
 
  
 6
 
  30624395
 
  30624769
 
  ENSEMBL   UCSC 
 
  375
 
  8
 
 33733
 
 gene
 
 0.78315
 
 0.84250
 
 Not queried
 
  1.3071512
 
       NA
 
       NA
  
 
  
 1
 
 221055401
 
 221055964
 
  ENSEMBL   UCSC 
 
  564
 
  6
 
 1018
 
 gene;island
 
 0.70632
 
 0.49838
 
 Not queried
 
  1.3068734
 
       NA
 
       NA
  
 
  
 14
 
  35099088
 
  35099711
 
  ENSEMBL   UCSC 
 
  624
 
  7
 
 12746
 
 island
 
 0.03070
 
 0.09657
 
 Not queried
 
  1.3068286
 
       NA
 
       NA
  
 
  
 2
 
 220491676
 
 220492186
 
  ENSEMBL   UCSC 
 
  511
 
  5
 
 23874
 
 tss;island
 
 0.53476
 
 0.31725
 
 Not queried
 
  1.3066143
 
       NA
 
       NA
  
 
  
 11
 
 118842126
 
 118842413
 
  ENSEMBL   UCSC 
 
  288
 
  7
 
 7720
 
 tss
 
 0.97643
 
 0.94406
 
 Not queried
 
  1.3065237
 
       NA
 
       NA
  
 
  
 3
 
  40350768
 
  40351164
 
  ENSEMBL   UCSC 
 
  397
 
  9
 
 28193
 
 tss
 
 0.05329
 
 0.05264
 
 Not queried
 
  1.3064832
 
       NA
 
       NA
  
 
  
 8
 
    216453
 
    216788
 
  ENSEMBL   UCSC 
 
  336
 
  5
 
 39937
 
 island
 
 0.18985
 
 0.53988
 
 Not queried
 
  1.3064353
 
       NA
 
       NA
  
 
  
 10
 
  90967052
 
  90967233
 
  ENSEMBL   UCSC 
 
  182
 
  7
 
 5901
 
 island
 
 0.01888
 
 0.04213
 
 Not queried
 
  1.3063984
 
       NA
 
       NA
  
 
  
 21
 
  43638893
 
  43639122
 
  ENSEMBL   UCSC 
 
  230
 
  9
 
 26396
 
 gene;tss;island
 
 0.02668
 
 0.04320
 
 Not queried
 
  1.3059021
 
       NA
 
       NA
  
 
  
 2
 
 219827660
 
 219827930
 
  ENSEMBL   UCSC 
 
  271
 
  4
 
 25059
 
 island
 
 0.03268
 
 0.07049
 
 Not queried
 
  1.3058776
 
       NA
 
       NA
  
 
  
 15
 
  41135793
 
  41135900
 
  ENSEMBL   UCSC 
 
  108
 
  4
 
 13569
 
 tss;island
 
 0.02525
 
 0.04702
 
 Not queried
 
  1.3058549
 
       NA
 
       NA
  
 
  
 14
 
  76044591
 
  76044828
 
  ENSEMBL   UCSC 
 
  238
 
  4
 
 12492
 
 tss
 
 0.10752
 
 0.05449
 
 Not queried
 
  1.3056922
 
       NA
 
       NA
  
 
  
 14
 
  76044591
 
  76045025
 
  ENSEMBL   UCSC 
 
  435
 
  6
 
 12989
 
 island
 
 0.13128
 
 0.06921
 
 Not queried
 
  1.3056922
 
       NA
 
       NA
  
 
  
 4
 
 101111617
 
 101111927
 
  ENSEMBL   UCSC 
 
  311
 
  5
 
 30468
 
 tss
 
 0.01115
 
 0.04275
 
 Not queried
 
  1.3055677
 
       NA
 
       NA
  
 
  
 7
 
 128116412
 
 128116732
 
  ENSEMBL   UCSC 
 
  321
 
  6
 
 37817
 
 tss
 
 0.16827
 
 0.04249
 
 Not queried
 
  1.3055203
 
       NA
 
       NA
  
 
  
 1
 
 207627372
 
 207627601
 
  ENSEMBL   UCSC 
 
  230
 
  5
 
 2218
 
 tss;island
 
 0.02034
 
 0.05736
 
 Not queried
 
  1.3053485
 
       NA
 
       NA
  
 
  
 22
 
  37730886
 
  37731229
 
  ENSEMBL   UCSC 
 
  344
 
  4
 
 27420
 
 island
 
 0.02050
 
 0.05768
 
 Not queried
 
  1.3052816
 
       NA
 
       NA
  
 
  
 19
 
  50666238
 
  50666552
 
  ENSEMBL   UCSC 
 
  315
 
  6
 
 22265
 
 island
 
 0.54912
 
 0.75489
 
 Not queried
 
  1.3048957
 
       NA
 
       NA
  
 
  
 19
 
   1151960
 
   1152189
 
  ENSEMBL   UCSC 
 
  230
 
  4
 
 19611
 
 gene;island
 
 0.80798
 
 0.94202
 
 Not queried
 
  1.3046069
 
       NA
 
       NA
  
 
  
 7
 
   2354192
 
   2354484
 
  ENSEMBL   UCSC 
 
  293
 
  8
 
 37343
 
 tss
 
 0.10101
 
 0.24009
 
 Not queried
 
  1.3042423
 
       NA
 
       NA
  
 
  
 7
 
  73894573
 
  73895278
 
  ENSEMBL   UCSC 
 
  706
 
  7
 
 36981
 
 gene;island
 
 0.65494
 
 0.83384
 
 Not queried
 
  1.3039792
 
       NA
 
       NA
  
 
  
 19
 
  55919383
 
  55919836
 
  ENSEMBL   UCSC 
 
  454
 
  6
 
 21043
 
 tss;island
 
 0.12029
 
 0.04351
 
 Not queried
 
  1.3036089
 
       NA
 
       NA
  
 
  
 1
 
 156217801
 
 156218113
 
  ENSEMBL   UCSC 
 
  313
 
  6
 
 2008
 
 tss;island
 
 0.61552
 
 0.77816
 
 Not queried
 
  1.3036015
 
       NA
 
       NA
  
 
  
 4
 
 111397134
 
 111398146
 
  ENSEMBL   UCSC 
 
 1013
 
 13
 
 31190
 
 island
 
 0.76678
 
 0.88085
 
 Not queried
 
  1.3035802
 
       NA
 
       NA
  
 
  
 13
 
 114886700
 
 114886883
 
  ENSEMBL   UCSC 
 
  184
 
  4
 
 11289
 
 gene
 
 0.75862
 
 0.88768
 
 Not queried
 
  1.3032803
 
       NA
 
       NA
  
 
  
 11
 
  69061244
 
  69061473
 
  ENSEMBL   UCSC 
 
  230
 
  4
 
 7497
 
 tss
 
 0.16917
 
 0.40365
 
 Not queried
 
  1.3024271
 
       NA
 
       NA
  
 
  
 17
 
  27188560
 
  27188876
 
  ENSEMBL   UCSC 
 
  317
 
  5
 
 17193
 
 tss
 
 0.58368
 
 0.77240
 
 Not queried
 
  1.3023036
 
       NA
 
       NA
  
 
  
 7
 
   1894025
 
   1894300
 
  ENSEMBL   UCSC 
 
  276
 
  4
 
 36707
 
 gene;island
 
 0.61361
 
 0.79679
 
 Not queried
 
  1.3022086
 
       NA
 
       NA
  
 
  
 11
 
  93466719
 
  93467095
 
  ENSEMBL   UCSC 
 
  377
 
  4
 
 7600
 
 tss
 
 0.85556
 
 0.90420
 
 Not queried
 
  1.3014860
 
       NA
 
       NA
  
 
  
 14
 
  60043704
 
  60043906
 
  ENSEMBL   UCSC 
 
  203
 
  4
 
 12404
 
 tss;island
 
 0.00954
 
 0.06780
 
 Not queried
 
  1.3013855
 
       NA
 
       NA
  
 
  
 20
 
  36147042
 
  36147340
 
  ENSEMBL   UCSC 
 
  299
 
  4
 
 25377
 
 gene;island
 
 0.81869
 
 0.90672
 
 Not queried
 
  1.3012983
 
       NA
 
       NA
  
 
  
 X
 
 152224090
 
 152224686
 
  ENSEMBL   UCSC 
 
  597
 
  6
 
 41574
 
 tss;island
 
 0.44960
 
 0.77099
 
 Not queried
 
  1.3012159
 
       NA
 
       NA
  
 
  
 15
 
  64995494
 
  64995684
 
  ENSEMBL   UCSC 
 
  191
 
  6
 
 13703
 
 tss;island
 
 0.03455
 
 0.07284
 
 Not queried
 
  1.3009334
 
       NA
 
       NA
  
 
  
 2
 
 241185137
 
 241185296
 
  ENSEMBL   UCSC 
 
  160
 
  4
 
 25231
 
 island
 
 0.73671
 
 0.88543
 
 Not queried
 
  1.3006901
 
       NA
 
       NA
  
 
  
 12
 
  56321427
 
  56321889
 
  ENSEMBL   UCSC 
 
  463
 
  9
 
 9748
 
 tss;island
 
 0.09426
 
 0.04053
 
 Not queried
 
  1.3006117
 
       NA
 
       NA
  
 
  
 12
 
 131200394
 
 131200855
 
  ENSEMBL   UCSC 
 
  462
 
  6
 
 10977
 
 island
 
 0.02252
 
 0.05705
 
 Not queried
 
  1.3004602
 
       NA
 
       NA
  
 
  
 7
 
 100464771
 
 100465289
 
  ENSEMBL   UCSC 
 
  519
 
  5
 
 38716
 
 island
 
 0.14000
 
 0.04180
 
 Not queried
 
  1.3003772
 
       NA
 
       NA
  
 
  
 17
 
  21454528
 
  21454896
 
  ENSEMBL   UCSC 
 
  369
 
  4
 
 16455
 
 gene
 
 0.01336
 
 0.02093
 
 Not queried
 
  1.2999917
 
       NA
 
       NA
  
 
  
 15
 
  83776769
 
  83777082
 
  ENSEMBL   UCSC 
 
  314
 
  5
 
 13428
 
 gene;island
 
 0.66768
 
 0.51096
 
 Not queried
 
  1.2999182
 
       NA
 
       NA
  
 
  
 1
 
 229406360
 
 229406711
 
  ENSEMBL   UCSC 
 
  352
 
  6
 
 2306
 
 tss;island
 
 0.63699
 
 0.49989
 
 Not queried
 
  1.2998706
 
       NA
 
       NA
  
 
  
 8
 
 144099482
 
 144099991
 
  ENSEMBL   UCSC 
 
  510
 
  6
 
 39878
 
 tss;island
 
 0.05663
 
 0.12468
 
 Not queried
 
  1.2996086
 
       NA
 
       NA
  
 
  
 19
 
  43423115
 
  43423265
 
  ENSEMBL   UCSC 
 
  151
 
  4
 
 20790
 
 tss
 
 0.63047
 
 0.46261
 
 Not queried
 
  1.2992144
 
       NA
 
       NA
  
 
  
 1
 
  23751369
 
  23751761
 
  ENSEMBL   UCSC 
 
  393
 
  5
 
 1337
 
 tss
 
 0.08153
 
 0.16619
 
 Not queried
 
  1.2988277
 
       NA
 
       NA
  
 
  
 7
 
 100728343
 
 100728731
 
  ENSEMBL   UCSC 
 
  389
 
  6
 
 37732
 
 tss
 
 0.02115
 
 0.05320
 
 Not queried
 
  1.2988092
 
       NA
 
       NA
  
 
  
 19
 
  17958339
 
  17959281
 
  ENSEMBL   UCSC 
 
  943
 
 14
 
 21722
 
 island
 
 0.70347
 
 0.61127
 
 Not queried
 
  1.2986521
 
       NA
 
       NA
  
 
  
 5
 
  63802491
 
  63802993
 
  ENSEMBL   UCSC 
 
  503
 
  5
 
 31571
 
 gene
 
 0.03900
 
 0.02493
 
 Not queried
 
  1.2983990
 
       NA
 
       NA
  
 
  
 1
 
  20512397
 
  20512944
 
  ENSEMBL   UCSC 
 
  548
 
  6
 
 2783
 
 island
 
 0.05292
 
 0.11286
 
 Not queried
 
  1.2979831
 
       NA
 
       NA
  
 
  
 18
 
  57364722
 
  57364872
 
  ENSEMBL   UCSC 
 
  151
 
  4
 
 19265
 
 tss
 
 0.02579
 
 0.02152
 
 Not queried
 
  1.2978997
 
       NA
 
       NA
  
 
  
 8
 
  65490184
 
  65490901
 
  ENSEMBL   UCSC 
 
  718
 
  8
 
 39671
 
 tss;island
 
 0.04627
 
 0.10296
 
 Not queried
 
  1.2971935
 
       NA
 
       NA
  
 
  
 22
 
  43045525
 
  43045736
 
  ENSEMBL   UCSC 
 
  212
 
  4
 
 27154
 
 tss;island
 
 0.06734
 
 0.03772
 
 Not queried
 
  1.2970490
 
       NA
 
       NA
  
 
  
 1
 
 110254662
 
 110254854
 
  ENSEMBL   UCSC 
 
  193
 
  6
 
 1807
 
 tss
 
 0.68008
 
 0.90341
 
 Not queried
 
  1.2969751
 
       NA
 
       NA
  
 
  
 14
 
  61119122
 
  61119419
 
  ENSEMBL   UCSC 
 
  298
 
  4
 
 12881
 
 island
 
 0.05372
 
 0.19215
 
 Not queried
 
  1.2961630
 
       NA
 
       NA
  
 
  
 22
 
  22337153
 
  22337468
 
  ENSEMBL   UCSC 
 
  316
 
  9
 
 26962
 
 tss
 
 0.02727
 
 0.07429
 
 Not queried
 
  1.2958030
 
       NA
 
       NA
  
 
  
 17
 
   6679254
 
   6679541
 
  ENSEMBL   UCSC 
 
  288
 
  9
 
 17020
 
 tss
 
 0.49269
 
 0.24417
 
 Not queried
 
  1.2947932
 
       NA
 
       NA
  
 
  
 7
 
    191704
 
    192015
 
  ENSEMBL   UCSC 
 
  312
 
  4
 
 37321
 
 tss;island
 
 0.18276
 
 0.29829
 
 Not queried
 
  1.2947229
 
       NA
 
       NA
  
 
  
 20
 
  61162000
 
  61162216
 
  ENSEMBL   UCSC 
 
  217
 
  7
 
 25452
 
 gene
 
 0.65408
 
 0.86179
 
 Not queried
 
  1.2942871
 
       NA
 
       NA
  
 
  
 X
 
  73756698
 
  73756802
 
  ENSEMBL   UCSC 
 
  105
 
  4
 
 41877
 
 island
 
 0.11735
 
 0.05980
 
 Not queried
 
  1.2941244
 
       NA
 
       NA
  
 
  
 12
 
  48722474
 
  48722754
 
  ENSEMBL   UCSC 
 
  281
 
  4
 
 9645
 
 tss;island
 
 0.88007
 
 0.66125
 
 Not queried
 
  1.2933070
 
       NA
 
       NA
  
 
  
 12
 
 132862800
 
 132863118
 
  ENSEMBL   UCSC 
 
  319
 
  5
 
 9418
 
 gene;island
 
 0.58981
 
 0.77205
 
 Not queried
 
  1.2929090
 
       NA
 
       NA
  
 
  
 1
 
 153581494
 
 153581793
 
  ENSEMBL   UCSC 
 
  300
 
  5
 
 759
 
 gene
 
 0.69459
 
 0.28151
 
 Not queried
 
  1.2928848
 
       NA
 
       NA
  
 
  
 X
 
  70364353
 
  70364646
 
  ENSEMBL   UCSC 
 
  294
 
  6
 
 41372
 
 tss
 
 0.40082
 
 0.61462
 
 Not queried
 
  1.2928051
 
       NA
 
       NA
  
 
  
 20
 
  26188919
 
  26188997
 
  ENSEMBL   UCSC 
 
   79
 
  6
 
 25616
 
 tss
 
 0.07762
 
 0.16573
 
 Not queried
 
  1.2925368
 
       NA
 
       NA
  
 
  
 X
 
  56589768
 
  56589951
 
  ENSEMBL   UCSC 
 
  184
 
  6
 
 41345
 
 tss
 
 0.04924
 
 0.02186
 
 Not queried
 
  1.2925014
 
       NA
 
       NA
  
 
  
 X
 
  57147697
 
  57147918
 
  ENSEMBL   UCSC 
 
  222
 
  4
 
 41005
 
 gene
 
 0.18576
 
 0.10636
 
 Not queried
 
  1.2924489
 
       NA
 
       NA
  
 
  
 11
 
  17373103
 
  17373302
 
  ENSEMBL   UCSC 
 
  200
 
  6
 
 7154
 
 tss;island
 
 0.03572
 
 0.06643
 
 Not queried
 
  1.2922991
 
       NA
 
       NA
  
 
  
 19
 
  22715300
 
  22715697
 
  ENSEMBL   UCSC 
 
  398
 
  4
 
 21807
 
 island
 
 0.43690
 
 0.65440
 
 Not queried
 
  1.2920982
 
       NA
 
       NA
  
 
  
 10
 
  94452236
 
  94452554
 
  ENSEMBL   UCSC 
 
  319
 
  4
 
 4663
 
 gene;island
 
 0.14597
 
 0.02551
 
 Not queried
 
  1.2914224
 
       NA
 
       NA
  
 
  
 19
 
   7741454
 
   7741838
 
  ENSEMBL   UCSC 
 
  385
 
  5
 
 20368
 
 tss
 
 0.47286
 
 0.79529
 
 Not queried
 
  1.2913873
 
       NA
 
       NA
  
 
  
 5
 
 175200401
 
 175200576
 
  ENSEMBL   UCSC 
 
  176
 
  4
 
 33356
 
 island
 
 0.19720
 
 0.06962
 
 Not queried
 
  1.2912488
 
       NA
 
       NA
  
 
  
 6
 
  99841605
 
  99842025
 
  ENSEMBL   UCSC 
 
  421
 
  4
 
 34358
 
 gene
 
 0.01039
 
 0.03440
 
 Not queried
 
  1.2912240
 
       NA
 
       NA
  
 
  
 1
 
 119526783
 
 119527156
 
  ENSEMBL   UCSC 
 
  374
 
  5
 
 713
 
 gene;island
 
 0.95715
 
 0.90730
 
 Not queried
 
  1.2911675
 
       NA
 
       NA
  
 
  
 14
 
  24629492
 
  24629560
 
  ENSEMBL   UCSC 
 
   69
 
  4
 
 12008
 
 gene;tss
 
 0.90711
 
 0.95589
 
 Not queried
 
  1.2910976
 
       NA
 
       NA
  
 
  
 16
 
  58497714
 
  58497815
 
  ENSEMBL   UCSC 
 
  102
 
  4
 
 14741
 
 gene;tss;island
 
 0.01605
 
 0.03371
 
 Not queried
 
  1.2909939
 
       NA
 
       NA
  
 
  
 20
 
  62168727
 
  62168955
 
  ENSEMBL   UCSC 
 
  229
 
  7
 
 25858
 
 tss
 
 0.58376
 
 0.82197
 
 Not queried
 
  1.2909589
 
       NA
 
       NA
  
 
  
 17
 
  78387621
 
  78387818
 
  ENSEMBL   UCSC 
 
  198
 
  4
 
 16831
 
 gene;tss;island
 
 0.43969
 
 0.60976
 
 Not queried
 
  1.2908129
 
       NA
 
       NA
  
 
  
 6
 
  74162051
 
  74162142
 
  ENSEMBL   UCSC 
 
   92
 
  6
 
 35006
 
 tss
 
 0.67484
 
 0.50440
 
 Not queried
 
  1.2904151
 
       NA
 
       NA
  
 
  
 3
 
  35785735
 
  35785958
 
  ENSEMBL   UCSC 
 
  224
 
  4
 
 28176
 
 tss
 
 0.60612
 
 0.35546
 
 Not queried
 
  1.2903802
 
       NA
 
       NA
  
 
  
 1
 
   1897585
 
   1897959
 
  ENSEMBL   UCSC 
 
  375
 
  4
 
 51
 
 gene;island
 
 0.68607
 
 0.82635
 
 Not queried
 
  1.2899161
 
       NA
 
       NA
  
 
  
 17
 
   8089904
 
   8090248
 
  ENSEMBL   UCSC 
 
  345
 
  4
 
 17961
 
 island
 
 0.00976
 
 0.02385
 
 Not queried
 
  1.2897732
 
       NA
 
       NA
  
 
  
 8
 
  23082233
 
  23082961
 
  ENSEMBL   UCSC 
 
  729
 
 12
 
 40084
 
 island
 
 0.07426
 
 0.04269
 
 Not queried
 
  1.2896651
 
       NA
 
       NA
  
 
  
 8
 
  49231559
 
  49231786
 
  ENSEMBL   UCSC 
 
  228
 
  4
 
 40215
 
 island
 
 0.05059
 
 0.02021
 
 Not queried
 
  1.2893389
 
       NA
 
       NA
  
 
  
 12
 
 119212160
 
 119212319
 
  ENSEMBL   UCSC 
 
  160
 
  4
 
 10843
 
 island
 
 0.14519
 
 0.19299
 
 Not queried
 
  1.2892562
 
       NA
 
       NA
  
 
  
 6
 
  32817662
 
  32818477
 
  ENSEMBL   UCSC 
 
  816
 
 12
 
 34089
 
 gene;island
 
 0.69605
 
 0.54641
 
 Not queried
 
  1.2892245
 
       NA
 
       NA
  
 
  
 3
 
  61235829
 
  61236016
 
  ENSEMBL   UCSC 
 
  188
 
  4
 
 27802
 
 gene;island
 
 0.05264
 
 0.11911
 
 Not queried
 
  1.2889547
 
       NA
 
       NA
  
 
  
 2
 
  54086854
 
  54087552
 
  ENSEMBL   UCSC 
 
  699
 
 14
 
 24257
 
 island
 
 0.78863
 
 0.91735
 
 Not queried
 
  1.2885930
 
       NA
 
       NA
  
 
  
 X
 
 101905837
 
 101906288
 
  ENSEMBL   UCSC 
 
  452
 
  7
 
 41432
 
 tss
 
 0.09200
 
 0.01869
 
 Not queried
 
  1.2884825
 
       NA
 
       NA
  
 
  
 X
 
 101905837
 
 101906537
 
  ENSEMBL   UCSC 
 
  701
 
  9
 
 41918
 
 island
 
 0.03249
 
 0.01869
 
 Not queried
 
  1.2884825
 
       NA
 
       NA
  
 
  
 9
 
 100615084
 
 100615357
 
  ENSEMBL   UCSC 
 
  274
 
  4
 
 40760
 
 tss;island
 
 0.31941
 
 0.53338
 
 Not queried
 
  1.2876202
 
       NA
 
       NA
  
 
  
 20
 
  61162000
 
  61162112
 
  ENSEMBL   UCSC 
 
  113
 
  6
 
 25838
 
 tss
 
 0.61494
 
 0.80221
 
 Not queried
 
  1.2873656
 
       NA
 
       NA
  
 
  
 11
 
  66314211
 
  66314378
 
  ENSEMBL   UCSC 
 
  168
 
  5
 
 7452
 
 tss;island
 
 0.07590
 
 0.07869
 
 Not queried
 
  1.2872843
 
       NA
 
       NA
  
 
  
 4
 
 155661691
 
 155661929
 
  ENSEMBL   UCSC 
 
  239
 
  4
 
 31330
 
 island
 
 0.73480
 
 0.51442
 
 Not queried
 
  1.2871804
 
       NA
 
       NA
  
 
  
 18
 
  12287173
 
  12287561
 
  ENSEMBL   UCSC 
 
  389
 
  4
 
 19368
 
 island
 
 0.80403
 
 0.62088
 
 Not queried
 
  1.2871262
 
       NA
 
       NA
  
 
  
 15
 
  58357874
 
  58357989
 
  ENSEMBL   UCSC 
 
  116
 
  7
 
 14147
 
 island
 
 0.01461
 
 0.02565
 
 Not queried
 
  1.2871022
 
       NA
 
       NA
  
 
  
 7
 
   2802374
 
   2802697
 
  ENSEMBL   UCSC 
 
  324
 
  4
 
 36756
 
 gene;island
 
 0.84010
 
 0.91004
 
 Not queried
 
  1.2867998
 
       NA
 
       NA
  
 
  
 8
 
 142180375
 
 142180720
 
  ENSEMBL   UCSC 
 
  346
 
  4
 
 39382
 
 gene;island
 
 0.90221
 
 0.96102
 
 Not queried
 
  1.2862178
 
       NA
 
       NA
  
 
  
 7
 
   1607146
 
   1607868
 
  ENSEMBL   UCSC 
 
  723
 
  7
 
 36700
 
 gene;island
 
 0.74030
 
 0.60269
 
 Not queried
 
  1.2859138
 
       NA
 
       NA
  
 
  
 5
 
 110074217
 
 110074930
 
  ENSEMBL   UCSC 
 
  714
 
 12
 
 32937
 
 island
 
 0.07037
 
 0.02079
 
 Not queried
 
  1.2844567
 
       NA
 
       NA
  
 
  
 22
 
  21986976
 
  21987190
 
  ENSEMBL   UCSC 
 
  215
 
  7
 
 27293
 
 island
 
 0.61951
 
 0.70446
 
 Not queried
 
  1.2840199
 
       NA
 
       NA
  
 
  
 19
 
  42082102
 
  42082485
 
  ENSEMBL   UCSC 
 
  384
 
  4
 
 20777
 
 tss
 
 0.22681
 
 0.44477
 
 Not queried
 
  1.2838090
 
       NA
 
       NA
  
 
  
 7
 
  43797839
 
  43798207
 
  ENSEMBL   UCSC 
 
  369
 
  7
 
 37493
 
 tss;island
 
 0.05804
 
 0.02468
 
 Not queried
 
  1.2835968
 
       NA
 
       NA
  
 
  
 10
 
  32398778
 
  32399026
 
  ENSEMBL   UCSC 
 
  249
 
  4
 
 5638
 
 island
 
 0.73730
 
 0.88968
 
 Not queried
 
  1.2829532
 
       NA
 
       NA
  
 
  
 7
 
  47694695
 
  47694837
 
  ENSEMBL   UCSC 
 
  143
 
  5
 
 37524
 
 tss
 
 0.75241
 
 0.89968
 
 Not queried
 
  1.2829332
 
       NA
 
       NA
  
 
  
 17
 
  75471193
 
  75471308
 
  ENSEMBL   UCSC 
 
  116
 
  4
 
 17666
 
 tss
 
 0.94884
 
 0.85490
 
 Not queried
 
  1.2826913
 
       NA
 
       NA
  
 
  
 3
 
 129275285
 
 129275655
 
  ENSEMBL   UCSC 
 
  371
 
  4
 
 27900
 
 gene
 
 0.84937
 
 0.96881
 
 Not queried
 
  1.2824180
 
       NA
 
       NA
  
 
  
 19
 
   1256999
 
   1257347
 
  ENSEMBL   UCSC 
 
  349
 
  4
 
 19614
 
 gene;island
 
 0.80489
 
 0.92913
 
 Not queried
 
  1.2820541
 
       NA
 
       NA
  
 
  
 17
 
  46628663
 
  46629018
 
  ENSEMBL   UCSC 
 
  356
 
  4
 
 16616
 
 gene;island
 
 0.53753
 
 0.77240
 
 Not queried
 
  1.2819625
 
       NA
 
       NA
  
 
  
 12
 
  72148251
 
  72148515
 
  ENSEMBL   UCSC 
 
  265
 
  7
 
 9849
 
 tss;island
 
 0.02018
 
 0.03903
 
 Not queried
 
  1.2816503
 
       NA
 
       NA
  
 
  
 12
 
  62586552
 
  62586892
 
  ENSEMBL   UCSC 
 
  341
 
  6
 
 9807
 
 tss
 
 0.03351
 
 0.03017
 
 Not queried
 
  1.2813293
 
       NA
 
       NA
  
 
  
 7
 
 100769903
 
 100770286
 
  ENSEMBL   UCSC 
 
  384
 
  5
 
 37733
 
 tss
 
 0.11317
 
 0.12831
 
 Not queried
 
  1.2810541
 
       NA
 
       NA
  
 
  
 7
 
 143042585
 
 143042798
 
  ENSEMBL   UCSC 
 
  214
 
  4
 
 37190
 
 gene;island
 
 0.03031
 
 0.04651
 
 Not queried
 
  1.2799624
 
       NA
 
       NA
  
 
  
 6
 
  24646254
 
  24646782
 
  ENSEMBL   UCSC 
 
  529
 
  7
 
 34605
 
 tss
 
 0.02750
 
 0.03846
 
 Not queried
 
  1.2798264
 
       NA
 
       NA
  
 
  
 19
 
  43774820
 
  43774982
 
  ENSEMBL   UCSC 
 
  163
 
  4
 
 20791
 
 tss
 
 0.73664
 
 0.48235
 
 Not queried
 
  1.2798025
 
       NA
 
       NA
  
 
  
 7
 
  27197455
 
  27197713
 
  ENSEMBL   UCSC 
 
  259
 
  4
 
 37430
 
 tss
 
 0.89543
 
 0.71261
 
 Not queried
 
  1.2796792
 
       NA
 
       NA
  
 
  
 17
 
   7461260
 
   7461556
 
  ENSEMBL   UCSC 
 
  297
 
  4
 
 17054
 
 tss
 
 0.45810
 
 0.29036
 
 Not queried
 
  1.2796320
 
       NA
 
       NA
  
 
  
 7
 
 157453775
 
 157454207
 
  ENSEMBL   UCSC 
 
  433
 
  8
 
 37274
 
 gene;island
 
 0.81600
 
 0.87151
 
 Not queried
 
  1.2792904
 
       NA
 
       NA
  
 
  
 X
 
  44703151
 
  44703983
 
  ENSEMBL   UCSC 
 
  833
 
  9
 
 41734
 
 island
 
 0.80268
 
 0.91039
 
 Not queried
 
  1.2792477
 
       NA
 
       NA
  
 
  
 5
 
   7851231
 
   7851681
 
  ENSEMBL   UCSC 
 
  451
 
  6
 
 32620
 
 island
 
 0.44410
 
 0.14025
 
 Not queried
 
  1.2792475
 
       NA
 
       NA
  
 
  
 6
 
  32156071
 
  32156527
 
  ENSEMBL   UCSC 
 
  457
 
  7
 
 34018
 
 gene
 
 0.47799
 
 0.73428
 
 Not queried
 
  1.2791276
 
       NA
 
       NA
  
 
  
 14
 
  23821435
 
  23821596
 
  ENSEMBL   UCSC 
 
  162
 
  4
 
 12000
 
 gene;island
 
 0.17617
 
 0.07203
 
 Not queried
 
  1.2786374
 
       NA
 
       NA
  
 
  
 1
 
 173887009
 
 173887360
 
  ENSEMBL   UCSC 
 
  352
 
  5
 
 2097
 
 tss
 
 0.63698
 
 0.83914
 
 Not queried
 
  1.2783625
 
       NA
 
       NA
  
 
  
 4
 
  81951951
 
  81952099
 
  ENSEMBL   UCSC 
 
  149
 
  6
 
 30416
 
 tss;island
 
 0.02132
 
 0.04770
 
 Not queried
 
  1.2781941
 
       NA
 
       NA
  
 
  
 17
 
  73720591
 
  73720840
 
  ENSEMBL   UCSC 
 
  250
 
  4
 
 16740
 
 gene;island
 
 0.82401
 
 0.64226
 
 Not queried
 
  1.2778783
 
       NA
 
       NA
  
 
  
 1
 
  24152579
 
  24152774
 
  ENSEMBL   UCSC 
 
  196
 
  4
 
 1346
 
 tss
 
 0.05276
 
 0.15666
 
 Not queried
 
  1.2778293
 
       NA
 
       NA
  
 
  
 20
 
  61637360
 
  61637856
 
  ENSEMBL   UCSC 
 
  497
 
  4
 
 25457
 
 gene;island
 
 0.46690
 
 0.63675
 
 Not queried
 
  1.2777905
 
       NA
 
       NA
  
 
  
 X
 
  15683192
 
  15683427
 
  ENSEMBL   UCSC 
 
  236
 
  7
 
 41199
 
 tss
 
 0.47157
 
 0.68218
 
 Not queried
 
  1.2774112
 
       NA
 
       NA
  
 
  
 20
 
  47013687
 
  47013919
 
  ENSEMBL   UCSC 
 
  233
 
  4
 
 26186
 
 island
 
 0.58837
 
 0.78594
 
 Not queried
 
  1.2773670
 
       NA
 
       NA
  
 
  
 12
 
   7863853
 
   7864076
 
  ENSEMBL   UCSC 
 
  224
 
  5
 
 9525
 
 tss
 
 0.65770
 
 0.74099
 
 Not queried
 
  1.2768545
 
       NA
 
       NA
  
 
  
 8
 
  27472239
 
  27472321
 
  ENSEMBL   UCSC 
 
   83
 
  4
 
 39195
 
 gene
 
 0.01798
 
 0.04202
 
 Not queried
 
  1.2765825
 
       NA
 
       NA
  
 
  
 4
 
  48988015
 
  48988253
 
  ENSEMBL   UCSC 
 
  239
 
  6
 
 30340
 
 tss
 
 0.30269
 
 0.16523
 
 Not queried
 
  1.2761865
 
       NA
 
       NA
  
 
  
 8
 
  81083339
 
  81083534
 
  ENSEMBL   UCSC 
 
  196
 
  4
 
 39286
 
 gene;island
 
 0.07431
 
 0.09391
 
 Not queried
 
  1.2761729
 
       NA
 
       NA
  
 
  
 6
 
  32820546
 
  32822352
 
  ENSEMBL   UCSC 
 
 1807
 
 58
 
 34815
 
 tss
 
 0.04585
 
 0.11329
 
 Not queried
 
  1.2757553
 
       NA
 
       NA
  
 
  
 1
 
  27113508
 
  27113753
 
  ENSEMBL   UCSC 
 
  246
 
  6
 
 1392
 
 tss;island
 
 0.05884
 
 0.07136
 
 Not queried
 
  1.2757397
 
       NA
 
       NA
  
 
  
 14
 
  23235611
 
  23235738
 
  ENSEMBL   UCSC 
 
  128
 
  5
 
 12672
 
 island
 
 0.02294
 
 0.00961
 
 Not queried
 
  1.2755575
 
       NA
 
       NA
  
 
  
 19
 
  33764284
 
  33764550
 
  ENSEMBL   UCSC 
 
  267
 
  4
 
 21838
 
 island
 
 0.66236
 
 0.82677
 
 Not queried
 
  1.2753990
 
       NA
 
       NA
  
 
  
 11
 
  66084469
 
  66084841
 
  ENSEMBL   UCSC 
 
  373
 
  5
 
 8484
 
 island
 
 0.51407
 
 0.71914
 
 Not queried
 
  1.2752056
 
       NA
 
       NA
  
 
  
 17
 
  42385923
 
  42386419
 
  ENSEMBL   UCSC 
 
  497
 
  8
 
 18363
 
 island
 
 0.49157
 
 0.10629
 
 Not queried
 
  1.2749464
 
       NA
 
       NA
  
 
  
 1
 
   4832352
 
   4832593
 
  ENSEMBL   UCSC 
 
  242
 
  5
 
 152
 
 gene;island
 
 0.87406
 
 0.69298
 
 Not queried
 
  1.2748882
 
       NA
 
       NA
  
 
  
 8
 
   1615950
 
   1616925
 
  ENSEMBL   UCSC 
 
  976
 
 10
 
 39108
 
 gene;island
 
 0.83705
 
 0.92649
 
 Not queried
 
  1.2748517
 
       NA
 
       NA
  
 
  
 12
 
  49504691
 
  49505053
 
  ENSEMBL   UCSC 
 
  363
 
  6
 
 9657
 
 tss
 
 0.24661
 
 0.02333
 
 Not queried
 
  1.2746079
 
       NA
 
       NA
  
 
  
 8
 
 120219902
 
 120220131
 
  ENSEMBL   UCSC 
 
  230
 
  7
 
 39821
 
 tss;island
 
 0.21772
 
 0.48526
 
 Not queried
 
  1.2745436
 
       NA
 
       NA
  
 
  
 5
 
 149535646
 
 149535765
 
  ENSEMBL   UCSC 
 
  120
 
  4
 
 32366
 
 tss
 
 0.78276
 
 0.56398
 
 Not queried
 
  1.2742872
 
       NA
 
       NA
  
 
  
 16
 
   1041053
 
   1041333
 
  ENSEMBL   UCSC 
 
  281
 
  6
 
 15474
 
 island
 
 0.75745
 
 0.88344
 
 Not queried
 
  1.2739187
 
       NA
 
       NA
  
 
  
 22
 
  50682500
 
  50682980
 
  ENSEMBL   UCSC 
 
  481
 
  4
 
 26885
 
 gene;island
 
 0.00623
 
 0.01256
 
 Not queried
 
  1.2732938
 
       NA
 
       NA
  
 
  
 17
 
   9929698
 
   9929867
 
  ENSEMBL   UCSC 
 
  170
 
  4
 
 17100
 
 tss
 
 0.68432
 
 0.52620
 
 Not queried
 
  1.2730594
 
       NA
 
       NA
  
 
  
 20
 
  30618874
 
  30619244
 
  ENSEMBL   UCSC 
 
  371
 
  5
 
 25350
 
 gene;island
 
 0.10001
 
 0.22563
 
 Not queried
 
  1.2729972
 
       NA
 
       NA
  
 
  
 11
 
   2170870
 
   2171184
 
  ENSEMBL   UCSC 
 
  315
 
  5
 
 7039
 
 tss
 
 0.27013
 
 0.55565
 
 Not queried
 
  1.2728392
 
       NA
 
       NA
  
 
  
 8
 
 102092795
 
 102092965
 
  ENSEMBL   UCSC 
 
  171
 
  4
 
 40437
 
 island
 
 0.15722
 
 0.29677
 
 Not queried
 
  1.2727704
 
       NA
 
       NA
  
 
  
 10
 
   5406890
 
   5406971
 
  ENSEMBL   UCSC 
 
   82
 
  6
 
 4906
 
 tss
 
 0.30350
 
 0.48723
 
 Not queried
 
  1.2726900
 
       NA
 
       NA
  
 
  
 12
 
   7280912
 
   7280988
 
  ENSEMBL   UCSC 
 
   77
 
  4
 
 9021
 
 gene
 
 0.92262
 
 0.82990
 
 Not queried
 
  1.2723447
 
       NA
 
       NA
  
 
  
 10
 
  23481385
 
  23481786
 
  ENSEMBL   UCSC 
 
  402
 
  6
 
 5592
 
 island
 
 0.04949
 
 0.07178
 
 Not queried
 
  1.2719537
 
       NA
 
       NA
  
 
  
 3
 
 128205143
 
 128205496
 
  ENSEMBL   UCSC 
 
  354
 
  5
 
 27894
 
 gene;island
 
 0.78794
 
 0.59941
 
 Not queried
 
  1.2718873
 
       NA
 
       NA
  
 
  
 5
 
 150227510
 
 150227845
 
  ENSEMBL   UCSC 
 
  336
 
  4
 
 31807
 
 gene
 
 0.81536
 
 0.68987
 
 Not queried
 
  1.2716163
 
       NA
 
       NA
  
 
  
 19
 
   7701662
 
   7701963
 
  ENSEMBL   UCSC 
 
  302
 
  8
 
 20367
 
 tss;island
 
 0.04176
 
 0.03273
 
 Not queried
 
  1.2713875
 
       NA
 
       NA
  
 
  
 19
 
  47217554
 
  47218031
 
  ENSEMBL   UCSC 
 
  478
 
  6
 
 20076
 
 gene;island
 
 0.10301
 
 0.26871
 
 Not queried
 
  1.2706481
 
       NA
 
       NA
  
 
  
 12
 
  14518391
 
  14518548
 
  ENSEMBL   UCSC 
 
  158
 
  5
 
 9558
 
 tss
 
 0.19332
 
 0.27364
 
 Not queried
 
  1.2705528
 
       NA
 
       NA
  
 
  
 14
 
  69676183
 
  69676460
 
  ENSEMBL   UCSC 
 
  278
 
  6
 
 12450
 
 tss
 
 0.83600
 
 0.92538
 
 Not queried
 
  1.2702487
 
       NA
 
       NA
  
 
  
 13
 
  48669293
 
  48669516
 
  ENSEMBL   UCSC 
 
  224
 
  7
 
 11404
 
 tss
 
 0.12949
 
 0.16921
 
 Not queried
 
  1.2699882
 
       NA
 
       NA
  
 
  
 16
 
  77756058
 
  77756392
 
  ENSEMBL   UCSC 
 
  335
 
  4
 
 15340
 
 tss
 
 0.01846
 
 0.04347
 
 Not queried
 
  1.2696044
 
       NA
 
       NA
  
 
  
 12
 
 133065724
 
 133066069
 
  ENSEMBL   UCSC 
 
  346
 
 16
 
 10104
 
 tss;island
 
 0.09285
 
 0.02434
 
 Not queried
 
  1.2694415
 
       NA
 
       NA
  
 
  
 14
 
 104171695
 
 104172224
 
  ENSEMBL   UCSC 
 
  530
 
  5
 
 12212
 
 gene
 
 0.85041
 
 0.91988
 
 Not queried
 
  1.2690655
 
       NA
 
       NA
  
 
  
 11
 
  65430868
 
  65431114
 
  ENSEMBL   UCSC 
 
  247
 
  4
 
 7417
 
 tss;island
 
 0.03112
 
 0.03814
 
 Not queried
 
  1.2686573
 
       NA
 
       NA
  
 
  
 10
 
  72201484
 
  72201741
 
  ENSEMBL   UCSC 
 
  258
 
  6
 
 5118
 
 tss
 
 0.42538
 
 0.62411
 
 Not queried
 
  1.2686068
 
       NA
 
       NA
  
 
  
 8
 
 143609379
 
 143609637
 
  ENSEMBL   UCSC 
 
  259
 
  4
 
 39402
 
 gene;island
 
 0.59198
 
 0.81189
 
 Not queried
 
  1.2683670
 
       NA
 
       NA
  
 
  
 6
 
  31744339
 
  31744962
 
  ENSEMBL   UCSC 
 
  624
 
 14
 
 33891
 
 gene
 
 0.45366
 
 0.70530
 
 Not queried
 
  1.2682407
 
       NA
 
       NA
  
 
  
 4
 
 113300446
 
 113300821
 
  ENSEMBL   UCSC 
 
  376
 
  5
 
 30051
 
 gene;island
 
 0.03522
 
 0.09819
 
 Not queried
 
  1.2682350
 
       NA
 
       NA
  
 
  
 5
 
  64330880
 
  64330959
 
  ENSEMBL   UCSC 
 
   80
 
  4
 
 32769
 
 island
 
 0.93354
 
 0.84237
 
 Not queried
 
  1.2681180
 
       NA
 
       NA
  
 
  
 2
 
 115920474
 
 115920825
 
  ENSEMBL   UCSC 
 
  352
 
  4
 
 22802
 
 gene;island
 
 0.06981
 
 0.10567
 
 Not queried
 
  1.2676021
 
       NA
 
       NA
  
 
  
 18
 
   9707753
 
   9708098
 
  ENSEMBL   UCSC 
 
  346
 
  5
 
 19145
 
 tss;island
 
 0.12329
 
 0.08589
 
 Not queried
 
  1.2672604
 
       NA
 
       NA
  
 
  
 2
 
  73441162
 
  73441314
 
  ENSEMBL   UCSC 
 
  153
 
  4
 
 23400
 
 tss
 
 0.03461
 
 0.04746
 
 Not queried
 
  1.2671367
 
       NA
 
       NA
  
 
  
 1
 
  86046512
 
  86046961
 
  ENSEMBL   UCSC 
 
  450
 
  5
 
 607
 
 gene
 
 0.01286
 
 0.02921
 
 Not queried
 
  1.2667652
 
       NA
 
       NA
  
 
  
 13
 
  41706543
 
  41707241
 
  ENSEMBL   UCSC 
 
  699
 
 11
 
 11662
 
 island
 
 0.02199
 
 0.02655
 
 Not queried
 
  1.2667453
 
       NA
 
       NA
  
 
  
 2
 
 231917214
 
 231917885
 
  ENSEMBL   UCSC 
 
  672
 
  6
 
 25139
 
 island
 
 0.02768
 
 0.05438
 
 Not queried
 
  1.2666524
 
       NA
 
       NA
  
 
  
 19
 
  11348704
 
  11348863
 
  ENSEMBL   UCSC 
 
  160
 
  4
 
 19795
 
 gene
 
 0.61280
 
 0.83044
 
 Not queried
 
  1.2665846
 
       NA
 
       NA
  
 
  
 13
 
  28493913
 
  28494161
 
  ENSEMBL   UCSC 
 
  249
 
  6
 
 11337
 
 tss
 
 0.03228
 
 0.06959
 
 Not queried
 
  1.2663765
 
       NA
 
       NA
  
 
  
 13
 
  28493913
 
  28494177
 
  ENSEMBL   UCSC 
 
  265
 
  8
 
 11604
 
 island
 
 0.03002
 
 0.06300
 
 Not queried
 
  1.2663765
 
       NA
 
       NA
  
 
  
 6
 
 138428736
 
 138428995
 
  ENSEMBL   UCSC 
 
  260
 
  7
 
 35187
 
 tss
 
 0.00946
 
 0.02379
 
 Not queried
 
  1.2660829
 
       NA
 
       NA
  
 
  
 7
 
 127671017
 
 127671195
 
  ENSEMBL   UCSC 
 
  179
 
  5
 
 37814
 
 tss
 
 0.08262
 
 0.03609
 
 Not queried
 
  1.2660692
 
       NA
 
       NA
  
 
  
 17
 
  59534637
 
  59534998
 
  ENSEMBL   UCSC 
 
  362
 
  5
 
 16685
 
 gene;island
 
 0.48402
 
 0.64954
 
 Not queried
 
  1.2660342
 
       NA
 
       NA
  
 
  
 15
 
  83240550
 
  83240791
 
  ENSEMBL   UCSC 
 
  242
 
  7
 
 13424
 
 gene
 
 0.19026
 
 0.35272
 
 Not queried
 
  1.2657068
 
       NA
 
       NA
  
 
  
 4
 
   7436701
 
   7437156
 
  ENSEMBL   UCSC 
 
  456
 
  5
 
 30245
 
 tss
 
 0.44252
 
 0.65578
 
 Not queried
 
  1.2656635
 
       NA
 
       NA
  
 
  
 1
 
  10707917
 
  10708091
 
  ENSEMBL   UCSC 
 
  175
 
  5
 
 211
 
 gene;island
 
 0.79583
 
 0.90854
 
 Not queried
 
  1.2655971
 
       NA
 
       NA
  
 
  
 1
 
  27216732
 
  27216957
 
  ENSEMBL   UCSC 
 
  226
 
  9
 
 2898
 
 island
 
 0.01967
 
 0.03083
 
 Not queried
 
  1.2655930
 
       NA
 
       NA
  
 
  
 7
 
  63498133
 
  63498367
 
  ENSEMBL   UCSC 
 
  235
 
  5
 
 38457
 
 island
 
 0.82538
 
 0.67766
 
 Not queried
 
  1.2655905
 
       NA
 
       NA
  
 
  
 1
 
  67520192
 
  67520352
 
  ENSEMBL   UCSC 
 
  161
 
  4
 
 1679
 
 tss;island
 
 0.04601
 
 0.01689
 
 Not queried
 
  1.2652520
 
       NA
 
       NA
  
 
  
 17
 
   4648949
 
   4649333
 
  ENSEMBL   UCSC 
 
  385
 
  4
 
 16322
 
 gene
 
 0.06102
 
 0.02635
 
 Not queried
 
  1.2651536
 
       NA
 
       NA
  
 
  
 16
 
  56642024
 
  56642433
 
  ENSEMBL   UCSC 
 
  410
 
  5
 
 15216
 
 tss
 
 0.06748
 
 0.15717
 
 Not queried
 
  1.2650856
 
       NA
 
       NA
  
 
  
 11
 
 121971092
 
 121971247
 
  ENSEMBL   UCSC 
 
  156
 
  4
 
 6927
 
 gene;tss
 
 0.04943
 
 0.03416
 
 Not queried
 
  1.2648895
 
       NA
 
       NA
  
 
  
 22
 
  31317764
 
  31318546
 
  ENSEMBL   UCSC 
 
  783
 
 12
 
 27377
 
 island
 
 0.78809
 
 0.86621
 
 Not queried
 
  1.2648262
 
       NA
 
       NA
  
 
  
 3
 
 140769610
 
 140770013
 
  ENSEMBL   UCSC 
 
  404
 
  4
 
 28593
 
 tss;island
 
 0.13970
 
 0.04107
 
 Not queried
 
  1.2646810
 
       NA
 
       NA
  
 
  
 10
 
  48416891
 
  48416977
 
  ENSEMBL   UCSC 
 
   87
 
  5
 
 5048
 
 tss
 
 0.85262
 
 0.91699
 
 Not queried
 
  1.2645783
 
       NA
 
       NA
  
 
  
 9
 
  74061171
 
  74061514
 
  ENSEMBL   UCSC 
 
  344
 
  4
 
 40827
 
 island
 
 0.01932
 
 0.05441
 
 Not queried
 
  1.2642375
 
       NA
 
       NA
  
 
  
 4
 
  24981801
 
  24982173
 
  ENSEMBL   UCSC 
 
  373
 
  6
 
 30282
 
 tss
 
 0.07039
 
 0.21719
 
 Not queried
 
  1.2640644
 
       NA
 
       NA
  
 
  
 12
 
  49942757
 
  49943340
 
  ENSEMBL   UCSC 
 
  584
 
  5
 
 9095
 
 gene;island
 
 0.59094
 
 0.81798
 
 Not queried
 
  1.2632302
 
       NA
 
       NA
  
 
  
 5
 
    191127
 
    191600
 
  ENSEMBL   UCSC 
 
  474
 
  8
 
 31895
 
 tss
 
 0.55897
 
 0.76336
 
 Not queried
 
  1.2631434
 
       NA
 
       NA
  
 
  
 1
 
 154475068
 
 154475269
 
  ENSEMBL   UCSC 
 
  202
 
  4
 
 777
 
 gene;tss;island
 
 0.33048
 
 0.54120
 
 Not queried
 
  1.2630556
 
       NA
 
       NA
  
 
  
 22
 
  43820888
 
  43821227
 
  ENSEMBL   UCSC 
 
  340
 
  4
 
 26848
 
 gene;island
 
 0.87872
 
 0.92928
 
 Not queried
 
  1.2629261
 
       NA
 
       NA
  
 
  
 6
 
  31804078
 
  31804739
 
  ENSEMBL   UCSC 
 
  662
 
  9
 
 34780
 
 tss
 
 0.14917
 
 0.04201
 
 Not queried
 
  1.2628861
 
       NA
 
       NA
  
 
  
 13
 
 103514655
 
 103514821
 
  ENSEMBL   UCSC 
 
  167
 
  4
 
 11191
 
 gene
 
 0.83185
 
 0.92453
 
 Not queried
 
  1.2627097
 
       NA
 
       NA
  
 
  
 13
 
  31377099
 
  31377406
 
  ENSEMBL   UCSC 
 
  308
 
  5
 
 11621
 
 island
 
 0.04950
 
 0.17222
 
 Not queried
 
  1.2626571
 
       NA
 
       NA
  
 
  
 11
 
    615945
 
    616112
 
  ENSEMBL   UCSC 
 
  168
 
  6
 
 7000
 
 tss;island
 
 0.08881
 
 0.04195
 
 Not queried
 
  1.2626478
 
       NA
 
       NA
  
 
  
 2
 
 223155841
 
 223156136
 
  ENSEMBL   UCSC 
 
  296
 
  4
 
 23007
 
 gene;island
 
 0.04448
 
 0.03542
 
 Not queried
 
  1.2625298
 
       NA
 
       NA
  
 
  
 4
 
  40337262
 
  40337443
 
  ENSEMBL   UCSC 
 
  182
 
  4
 
 30310
 
 tss
 
 0.71418
 
 0.88920
 
 Not queried
 
  1.2623009
 
       NA
 
       NA
  
 
  
 12
 
  54360131
 
  54360511
 
  ENSEMBL   UCSC 
 
  381
 
  4
 
 9131
 
 gene;island
 
 0.29613
 
 0.19721
 
 Not queried
 
  1.2622081
 
       NA
 
       NA
  
 
  
 2
 
 172947846
 
 172948176
 
  ENSEMBL   UCSC 
 
  331
 
  4
 
 24821
 
 island
 
 0.04340
 
 0.09785
 
 Not queried
 
  1.2621996
 
       NA
 
       NA
  
 
  
 1
 
  38157567
 
  38158129
 
  ENSEMBL   UCSC 
 
  563
 
  8
 
 1503
 
 tss
 
 0.01046
 
 0.01972
 
 Not queried
 
  1.2620677
 
       NA
 
       NA
  
 
  
 8
 
   1136127
 
   1136538
 
  ENSEMBL   UCSC 
 
  412
 
  6
 
 39954
 
 island
 
 0.81299
 
 0.94573
 
 Not queried
 
  1.2620671
 
       NA
 
       NA
  
 
  
 3
 
  21792248
 
  21792684
 
  ENSEMBL   UCSC 
 
  437
 
  4
 
 27658
 
 gene
 
 0.03071
 
 0.01461
 
 Not queried
 
  1.2617989
 
       NA
 
       NA
  
 
  
 16
 
  15595854
 
  15596423
 
  ENSEMBL   UCSC 
 
  570
 
  9
 
 14645
 
 gene
 
 0.74229
 
 0.87352
 
 Not queried
 
  1.2616680
 
       NA
 
       NA
  
 
  
 6
 
  32073324
 
  32073700
 
  ENSEMBL   UCSC 
 
  377
 
  5
 
 33986
 
 gene
 
 0.24364
 
 0.59353
 
 Not queried
 
  1.2616217
 
       NA
 
       NA
  
 
  
 6
 
 112041290
 
 112041490
 
  ENSEMBL   UCSC 
 
  201
 
  4
 
 35114
 
 tss
 
 0.81889
 
 0.92444
 
 Not queried
 
  1.2615187
 
       NA
 
       NA
  
 
  
 8
 
  11665843
 
  11666017
 
  ENSEMBL   UCSC 
 
  175
 
  4
 
 39144
 
 gene;island
 
 0.02296
 
 0.05564
 
 Not queried
 
  1.2614639
 
       NA
 
       NA
  
 
  
 1
 
 111888719
 
 111889533
 
  ENSEMBL   UCSC 
 
  815
 
 13
 
 3582
 
 island
 
 0.03550
 
 0.07172
 
 Not queried
 
  1.2612670
 
       NA
 
       NA
  
 
  
 X
 
 153029535
 
 153029722
 
  ENSEMBL   UCSC 
 
  188
 
  4
 
 42118
 
 island
 
 0.05677
 
 0.21932
 
 Not queried
 
  1.2608818
 
       NA
 
       NA
  
 
  
 5
 
  17216222
 
  17216679
 
  ENSEMBL   UCSC 
 
  458
 
  7
 
 31515
 
 gene;island
 
 0.00995
 
 0.01956
 
 Not queried
 
  1.2608024
 
       NA
 
       NA
  
 
  
 8
 
  11536778
 
  11537112
 
  ENSEMBL   UCSC 
 
  335
 
  4
 
 40019
 
 island
 
 0.59154
 
 0.78455
 
 Not queried
 
  1.2605332
 
       NA
 
       NA
  
 
  
 17
 
  44896017
 
  44896223
 
  ENSEMBL   UCSC 
 
  207
 
  8
 
 18413
 
 island
 
 0.07909
 
 0.03243
 
 Not queried
 
  1.2603731
 
       NA
 
       NA
  
 
  
 14
 
 104195181
 
 104195666
 
  ENSEMBL   UCSC 
 
  486
 
  5
 
 12215
 
 gene;island
 
 0.79011
 
 0.92302
 
 Not queried
 
  1.2603400
 
       NA
 
       NA
  
 
  
 3
 
 123679768
 
 123680219
 
  ENSEMBL   UCSC 
 
  452
 
  4
 
 27877
 
 gene;island
 
 0.02323
 
 0.02566
 
 Not queried
 
  1.2601249
 
       NA
 
       NA
  
 
  
 21
 
  37852479
 
  37852822
 
  ENSEMBL   UCSC 
 
  344
 
  4
 
 26495
 
 tss
 
 0.72143
 
 0.84931
 
 Not queried
 
  1.2599626
 
       NA
 
       NA
  
 
  
 12
 
   6933322
 
   6933689
 
  ENSEMBL   UCSC 
 
  368
 
  4
 
 9009
 
 gene;island
 
 0.61743
 
 0.78287
 
 Not queried
 
  1.2595212
 
       NA
 
       NA
  
 
  
 1
 
 249200307
 
 249200781
 
  ENSEMBL   UCSC 
 
  475
 
  5
 
 4415
 
 island
 
 0.04776
 
 0.11322
 
 Not queried
 
  1.2592164
 
       NA
 
       NA
  
 
  
 21
 
  44037277
 
  44037470
 
  ENSEMBL   UCSC 
 
  194
 
  4
 
 26664
 
 island
 
 0.64464
 
 0.83789
 
 Not queried
 
  1.2588042
 
       NA
 
       NA
  
 
  
 13
 
 111972854
 
 111972997
 
  ENSEMBL   UCSC 
 
  144
 
  6
 
 11514
 
 tss
 
 0.69519
 
 0.82673
 
 Not queried
 
  1.2585413
 
       NA
 
       NA
  
 
  
 17
 
  77311577
 
  77311812
 
  ENSEMBL   UCSC 
 
  236
 
  4
 
 16804
 
 gene
 
 0.56606
 
 0.81233
 
 Not queried
 
  1.2581200
 
       NA
 
       NA
  
 
  
 X
 
  56589768
 
  56590537
 
  ENSEMBL   UCSC 
 
  770
 
 13
 
 41822
 
 island
 
 0.03117
 
 0.01894
 
 Not queried
 
  1.2579312
 
       NA
 
       NA
  
 
  
 12
 
  52585786
 
  52585892
 
  ENSEMBL   UCSC 
 
  107
 
  4
 
 9699
 
 tss
 
 0.51900
 
 0.64457
 
 Not queried
 
  1.2578959
 
       NA
 
       NA
  
 
  
 3
 
  46608025
 
  46608179
 
  ENSEMBL   UCSC 
 
  155
 
  5
 
 28234
 
 tss
 
 0.20949
 
 0.09979
 
 Not queried
 
  1.2573431
 
       NA
 
       NA
  
 
  
 11
 
  63753020
 
  63753251
 
  ENSEMBL   UCSC 
 
  232
 
  6
 
 7361
 
 tss
 
 0.44073
 
 0.61576
 
 Not queried
 
  1.2572092
 
       NA
 
       NA
  
 
  
 11
 
  63753020
 
  63753598
 
  ENSEMBL   UCSC 
 
  579
 
 10
 
 8357
 
 island
 
 0.24092
 
 0.44132
 
 Not queried
 
  1.2572092
 
       NA
 
       NA
  
 
  
 14
 
  23589066
 
  23589419
 
  ENSEMBL   UCSC 
 
  354
 
  4
 
 12269
 
 tss
 
 0.62624
 
 0.81700
 
 Not queried
 
  1.2571797
 
       NA
 
       NA
  
 
  
 6
 
  33140660
 
  33141305
 
  ENSEMBL   UCSC 
 
  646
 
  9
 
 34136
 
 gene
 
 0.13376
 
 0.35755
 
 Not queried
 
  1.2565816
 
       NA
 
       NA
  
 
  
 15
 
  78556423
 
  78556534
 
  ENSEMBL   UCSC 
 
  112
 
  4
 
 13803
 
 tss;island
 
 0.73582
 
 0.82185
 
 Not queried
 
  1.2565680
 
       NA
 
       NA
  
 
  
 6
 
 137143497
 
 137143662
 
  ENSEMBL   UCSC 
 
  166
 
  6
 
 35180
 
 tss
 
 0.01218
 
 0.02217
 
 Not queried
 
  1.2565595
 
       NA
 
       NA
  
 
  
 12
 
 130918514
 
 130918994
 
  ENSEMBL   UCSC 
 
  481
 
  5
 
 9388
 
 gene
 
 0.62762
 
 0.91021
 
 Not queried
 
  1.2560933
 
       NA
 
       NA
  
 
  
 13
 
  99100506
 
  99100829
 
  ENSEMBL   UCSC 
 
  324
 
  6
 
 11171
 
 gene
 
 0.67965
 
 0.85259
 
 Not queried
 
  1.2560044
 
       NA
 
       NA
  
 
  
 10
 
 134536030
 
 134536236
 
  ENSEMBL   UCSC 
 
  207
 
  4
 
 4859
 
 gene;island
 
 0.92986
 
 0.84531
 
 Not queried
 
  1.2559972
 
       NA
 
       NA
  
 
  
 X
 
   9863586
 
   9863763
 
  ENSEMBL   UCSC 
 
  178
 
  4
 
 40931
 
 gene;island
 
 0.81569
 
 0.86686
 
 Not queried
 
  1.2556676
 
       NA
 
       NA
  
 
  
 4
 
  87797502
 
  87797676
 
  ENSEMBL   UCSC 
 
  175
 
  4
 
 30005
 
 gene
 
 0.81229
 
 0.90579
 
 Not queried
 
  1.2556276
 
       NA
 
       NA
  
 
  
 14
 
  54955792
 
  54955960
 
  ENSEMBL   UCSC 
 
  169
 
  5
 
 12379
 
 tss;island
 
 0.01033
 
 0.01964
 
 Not queried
 
  1.2550844
 
       NA
 
       NA
  
 
  
 3
 
   9691143
 
   9691379
 
  ENSEMBL   UCSC 
 
  237
 
  4
 
 27612
 
 gene
 
 0.01084
 
 0.02621
 
 Not queried
 
  1.2547037
 
       NA
 
       NA
  
 
  
 1
 
  24645802
 
  24645853
 
  ENSEMBL   UCSC 
 
   52
 
  4
 
 1354
 
 tss
 
 0.02265
 
 0.06516
 
 Not queried
 
  1.2545050
 
       NA
 
       NA
  
 
  
 7
 
   5253741
 
   5254072
 
  ENSEMBL   UCSC 
 
  332
 
  6
 
 36802
 
 gene
 
 0.82828
 
 0.94567
 
 Not queried
 
  1.2544043
 
       NA
 
       NA
  
 
  
 16
 
  57023022
 
  57023494
 
  ENSEMBL   UCSC 
 
  473
 
 10
 
 15925
 
 island
 
 0.10198
 
 0.04727
 
 Not queried
 
  1.2537656
 
       NA
 
       NA
  
 
  
 8
 
  82024462
 
  82024860
 
  ENSEMBL   UCSC 
 
  399
 
  6
 
 39719
 
 tss;island
 
 0.05648
 
 0.03906
 
 Not queried
 
  1.2535437
 
       NA
 
       NA
  
 
  
 10
 
  23384664
 
  23385077
 
  ENSEMBL   UCSC 
 
  414
 
  5
 
 4518
 
 gene;island
 
 0.03588
 
 0.07740
 
 Not queried
 
  1.2532162
 
       NA
 
       NA
  
 
  
 1
 
 207626214
 
 207626535
 
  ENSEMBL   UCSC 
 
  322
 
  4
 
 2217
 
 tss;island
 
 0.36279
 
 0.50852
 
 Not queried
 
  1.2531970
 
       NA
 
       NA
  
 
  
 17
 
  77078091
 
  77079031
 
  ENSEMBL   UCSC 
 
  941
 
  9
 
 16800
 
 gene
 
 0.77164
 
 0.82682
 
 Not queried
 
  1.2531206
 
       NA
 
       NA
  
 
  
 3
 
  96495651
 
  96496135
 
  ENSEMBL   UCSC 
 
  485
 
  5
 
 29266
 
 island
 
 0.83486
 
 0.68824
 
 Not queried
 
  1.2531192
 
       NA
 
       NA
  
 
  
 1
 
    990293
 
    990557
 
  ENSEMBL   UCSC 
 
  265
 
  4
 
 11
 
 gene;island
 
 0.92563
 
 0.83820
 
 Not queried
 
  1.2530635
 
       NA
 
       NA
  
 
  
 6
 
  32974479
 
  32974643
 
  ENSEMBL   UCSC 
 
  165
 
  4
 
 34110
 
 gene;island
 
 0.89542
 
 0.93594
 
 Not queried
 
  1.2527887
 
       NA
 
       NA
  
 
  
 5
 
  65018493
 
  65018638
 
  ENSEMBL   UCSC 
 
  146
 
  4
 
 31576
 
 gene;tss;island
 
 0.01894
 
 0.04404
 
 Not queried
 
  1.2525536
 
       NA
 
       NA
  
 
  
 8
 
  49835988
 
  49836230
 
  ENSEMBL   UCSC 
 
  243
 
  4
 
 40222
 
 island
 
 0.48252
 
 0.71260
 
 Not queried
 
  1.2524849
 
       NA
 
       NA
  
 
  
 17
 
  21281017
 
  21281507
 
  ENSEMBL   UCSC 
 
  491
 
  5
 
 16454
 
 gene;island
 
 0.15739
 
 0.12255
 
 Not queried
 
  1.2521603
 
       NA
 
       NA
  
 
  
 17
 
  34062219
 
  34062573
 
  ENSEMBL   UCSC 
 
  355
 
  5
 
 16489
 
 gene;island
 
 0.89315
 
 0.80272
 
 Not queried
 
  1.2521322
 
       NA
 
       NA
  
 
  
 2
 
 102314465
 
 102314757
 
  ENSEMBL   UCSC 
 
  293
 
  5
 
 24534
 
 island
 
 0.01724
 
 0.03703
 
 Not queried
 
  1.2519102
 
       NA
 
       NA
  
 
  
 19
 
  51051861
 
  51052080
 
  ENSEMBL   UCSC 
 
  220
 
  4
 
 20142
 
 gene;island
 
 0.84591
 
 0.69744
 
 Not queried
 
  1.2516822
 
       NA
 
       NA
  
 
  
 6
 
  30127152
 
  30127389
 
  ENSEMBL   UCSC 
 
  238
 
  4
 
 33671
 
 gene
 
 0.47046
 
 0.71021
 
 Not queried
 
  1.2512926
 
       NA
 
       NA
  
 
  
 14
 
  62229147
 
  62229330
 
  ENSEMBL   UCSC 
 
  184
 
  4
 
 12080
 
 gene
 
 0.02825
 
 0.08220
 
 Not queried
 
  1.2508832
 
       NA
 
       NA
  
 
  
 19
 
  36427426
 
  36427779
 
  ENSEMBL   UCSC 
 
  354
 
  5
 
 20682
 
 tss;island
 
 0.02412
 
 0.07261
 
 Not queried
 
  1.2507959
 
       NA
 
       NA
  
 
  
 18
 
  30050326
 
  30050398
 
  ENSEMBL   UCSC 
 
   73
 
  4
 
 19051
 
 gene;island
 
 0.03414
 
 0.02669
 
 Not queried
 
  1.2506263
 
       NA
 
       NA
  
 
  
 8
 
  79427993
 
  79428325
 
  ENSEMBL   UCSC 
 
  333
 
  4
 
 39714
 
 tss
 
 0.03425
 
 0.05547
 
 Not queried
 
  1.2504695
 
       NA
 
       NA
  
 
  
 1
 
  24017943
 
  24018143
 
  ENSEMBL   UCSC 
 
  201
 
  6
 
 1340
 
 tss;island
 
 0.02585
 
 0.06666
 
 Not queried
 
  1.2494194
 
       NA
 
       NA
  
 
  
 3
 
 131080235
 
 131080697
 
  ENSEMBL   UCSC 
 
  463
 
  7
 
 28550
 
 tss
 
 0.51134
 
 0.66543
 
 Not queried
 
  1.2493485
 
       NA
 
       NA
  
 
  
 3
 
 191048308
 
 191048439
 
  ENSEMBL   UCSC 
 
  132
 
  5
 
 28041
 
 gene;island
 
 0.01993
 
 0.04429
 
 Not queried
 
  1.2484721
 
       NA
 
       NA
  
 
  
 1
 
  71513494
 
  71513789
 
  ENSEMBL   UCSC 
 
  296
 
  6
 
 1690
 
 tss;island
 
 0.02471
 
 0.05195
 
 Not queried
 
  1.2482646
 
       NA
 
       NA
  
 
  
 X
 
  71793001
 
  71793175
 
  ENSEMBL   UCSC 
 
  175
 
  4
 
 41386
 
 tss
 
 0.00233
 
 0.00304
 
 Not queried
 
  1.2480878
 
       NA
 
       NA
  
 
  
 5
 
 141258637
 
 141258964
 
  ENSEMBL   UCSC 
 
  328
 
  5
 
 32332
 
 tss;island
 
 0.01863
 
 0.03636
 
 Not queried
 
  1.2474068
 
       NA
 
       NA
  
 
  
 X
 
  16804211
 
  16804461
 
  ENSEMBL   UCSC 
 
  251
 
  7
 
 41207
 
 tss
 
 0.01211
 
 0.02827
 
 Not queried
 
  1.2474017
 
       NA
 
       NA
  
 
  
 12
 
 133286734
 
 133286970
 
  ENSEMBL   UCSC 
 
  237
 
  4
 
 10107
 
 tss;island
 
 0.02443
 
 0.04598
 
 Not queried
 
  1.2471753
 
       NA
 
       NA
  
 
  
 10
 
 131758331
 
 131758786
 
  ENSEMBL   UCSC 
 
  456
 
  5
 
 4822
 
 gene;island
 
 0.10793
 
 0.05838
 
 Not queried
 
  1.2468613
 
       NA
 
       NA
  
 
  
 15
 
  41056021
 
  41056252
 
  ENSEMBL   UCSC 
 
  232
 
  5
 
 13566
 
 tss
 
 0.00769
 
 0.02161
 
 Not queried
 
  1.2468074
 
       NA
 
       NA
  
 
  
 4
 
 126237325
 
 126237513
 
  ENSEMBL   UCSC 
 
  189
 
  6
 
 30537
 
 tss
 
 0.04747
 
 0.02245
 
 Not queried
 
  1.2466826
 
       NA
 
       NA
  
 
  
 15
 
  40697714
 
  40698044
 
  ENSEMBL   UCSC 
 
  331
 
  4
 
 13263
 
 gene
 
 0.02094
 
 0.04981
 
 Not queried
 
  1.2466265
 
       NA
 
       NA
  
 
  
 14
 
 105676686
 
 105676908
 
  ENSEMBL   UCSC 
 
  223
 
  4
 
 12229
 
 gene
 
 0.77719
 
 0.86839
 
 Not queried
 
  1.2464942
 
       NA
 
       NA
  
 
  
 1
 
  48058877
 
  48059354
 
  ENSEMBL   UCSC 
 
  478
 
  5
 
 3236
 
 island
 
 0.51490
 
 0.75823
 
 Not queried
 
  1.2464324
 
       NA
 
       NA
  
 
  
 1
 
 151042743
 
 151043263
 
  ENSEMBL   UCSC 
 
  521
 
 10
 
 3727
 
 island
 
 0.02401
 
 0.05523
 
 Not queried
 
  1.2463497
 
       NA
 
       NA
  
 
  
 7
 
 150778122
 
 150778340
 
  ENSEMBL   UCSC 
 
  219
 
  7
 
 37925
 
 tss;island
 
 0.05345
 
 0.10750
 
 Not queried
 
  1.2463447
 
       NA
 
       NA
  
 
  
 11
 
  57519282
 
  57519659
 
  ENSEMBL   UCSC 
 
  378
 
  8
 
 7289
 
 tss;island
 
 0.20821
 
 0.40090
 
 Not queried
 
  1.2458692
 
       NA
 
       NA
  
 
  
 5
 
 149546331
 
 149546471
 
  ENSEMBL   UCSC 
 
  141
 
  5
 
 33242
 
 island
 
 0.02890
 
 0.04934
 
 Not queried
 
  1.2458080
 
       NA
 
       NA
  
 
  
 7
 
  27282233
 
  27282444
 
  ENSEMBL   UCSC 
 
  212
 
  6
 
 36880
 
 gene;island
 
 0.02450
 
 0.05398
 
 Not queried
 
  1.2456066
 
       NA
 
       NA
  
 
  
 22
 
  19938916
 
  19939061
 
  ENSEMBL   UCSC 
 
  146
 
  5
 
 26753
 
 gene
 
 0.78232
 
 0.89495
 
 Not queried
 
  1.2451976
 
       NA
 
       NA
  
 
  
 11
 
  71823834
 
  71824165
 
  ENSEMBL   UCSC 
 
  332
 
  8
 
 7517
 
 tss
 
 0.04488
 
 0.01446
 
 Not queried
 
  1.2450635
 
       NA
 
       NA
  
 
  
 1
 
 201476290
 
 201476371
 
  ENSEMBL   UCSC 
 
   82
 
  5
 
 936
 
 gene
 
 0.55209
 
 0.79144
 
 Not queried
 
  1.2449213
 
       NA
 
       NA
  
 
  
 19
 
  18111623
 
  18112042
 
  ENSEMBL   UCSC 
 
  420
 
  7
 
 21728
 
 island
 
 0.10798
 
 0.05121
 
 Not queried
 
  1.2448903
 
       NA
 
       NA
  
 
  
 4
 
  25097558
 
  25098059
 
  ENSEMBL   UCSC 
 
  502
 
  5
 
 30908
 
 island
 
 0.93395
 
 0.87900
 
 Not queried
 
  1.2448557
 
       NA
 
       NA
  
 
  
 7
 
  16460965
 
  16461244
 
  ENSEMBL   UCSC 
 
  280
 
  5
 
 37386
 
 tss
 
 0.08985
 
 0.04975
 
 Not queried
 
  1.2445439
 
       NA
 
       NA
  
 
  
 22
 
  36894057
 
  36894326
 
  ENSEMBL   UCSC 
 
  270
 
  4
 
 26818
 
 gene;island
 
 0.93874
 
 0.83191
 
 Not queried
 
  1.2444047
 
       NA
 
       NA
  
 
  
 16
 
  87812658
 
  87813069
 
  ENSEMBL   UCSC 
 
  412
 
  4
 
 16151
 
 island
 
 0.06661
 
 0.27101
 
 Not queried
 
  1.2443593
 
       NA
 
       NA
  
 
  
 5
 
 141392512
 
 141392853
 
  ENSEMBL   UCSC 
 
  342
 
  8
 
 33203
 
 island
 
 0.03047
 
 0.08252
 
 Not queried
 
  1.2440750
 
       NA
 
       NA
  
 
  
 16
 
    979898
 
    980146
 
  ENSEMBL   UCSC 
 
  249
 
  4
 
 14526
 
 gene;island
 
 0.63492
 
 0.86353
 
 Not queried
 
  1.2440728
 
       NA
 
       NA
  
 
  
 7
 
  50132903
 
  50133212
 
  ENSEMBL   UCSC 
 
  310
 
  8
 
 37528
 
 tss
 
 0.63297
 
 0.88229
 
 Not queried
 
  1.2438632
 
       NA
 
       NA
  
 
  
 1
 
 151735543
 
 151735844
 
  ENSEMBL   UCSC 
 
  302
 
  4
 
 751
 
 gene;island
 
 0.00889
 
 0.03196
 
 Not queried
 
  1.2434551
 
       NA
 
       NA
  
 
  
 11
 
 126870233
 
 126870702
 
  ENSEMBL   UCSC 
 
  470
 
  5
 
 6944
 
 gene
 
 0.04415
 
 0.10920
 
 Not queried
 
  1.2432912
 
       NA
 
       NA
  
 
  
 11
 
 126173516
 
 126173637
 
  ENSEMBL   UCSC 
 
  122
 
  6
 
 7777
 
 tss
 
 0.06381
 
 0.03237
 
 Not queried
 
  1.2432292
 
       NA
 
       NA
  
 
  
 4
 
  17516413
 
  17516819
 
  ENSEMBL   UCSC 
 
  407
 
  7
 
 30894
 
 island
 
 0.91192
 
 0.78462
 
 Not queried
 
  1.2431338
 
       NA
 
       NA
  
 
  
 11
 
  95523445
 
  95523641
 
  ENSEMBL   UCSC 
 
  197
 
  6
 
 7615
 
 tss;island
 
 0.00417
 
 0.01299
 
 Not queried
 
  1.2430541
 
       NA
 
       NA
  
 
  
 11
 
  84634581
 
  84634735
 
  ENSEMBL   UCSC 
 
  155
 
  4
 
 7580
 
 tss
 
 0.84230
 
 0.77576
 
 Not queried
 
  1.2428235
 
       NA
 
       NA
  
 
  
 4
 
    619162
 
    619392
 
  ENSEMBL   UCSC 
 
  231
 
  6
 
 30675
 
 island
 
 0.71292
 
 0.88640
 
 Not queried
 
  1.2427526
 
       NA
 
       NA
  
 
  
 7
 
 149470284
 
 149470343
 
  ENSEMBL   UCSC 
 
   60
 
  4
 
 38940
 
 island
 
 0.03083
 
 0.06642
 
 Not queried
 
  1.2427453
 
       NA
 
       NA
  
 
  
 14
 
  51339072
 
  51339216
 
  ENSEMBL   UCSC 
 
  145
 
  4
 
 12050
 
 gene;island
 
 0.39674
 
 0.60845
 
 Not queried
 
  1.2425889
 
       NA
 
       NA
  
 
  
 14
 
  69256677
 
  69256977
 
  ENSEMBL   UCSC 
 
  301
 
  5
 
 12093
 
 gene;island
 
 0.90794
 
 0.86604
 
 Not queried
 
  1.2425492
 
       NA
 
       NA
  
 
  
 19
 
  54972426
 
  54972723
 
  ENSEMBL   UCSC 
 
  298
 
  4
 
 20185
 
 gene;island
 
 0.90548
 
 0.95888
 
 Not queried
 
  1.2425352
 
       NA
 
       NA
  
 
  
 7
 
 100463416
 
 100463759
 
  ENSEMBL   UCSC 
 
  344
 
  4
 
 37085
 
 gene;island
 
 0.71916
 
 0.87058
 
 Not queried
 
  1.2425062
 
       NA
 
       NA
  
 
  
 6
 
  31474851
 
  31475512
 
  ENSEMBL   UCSC 
 
  662
 
  7
 
 33821
 
 gene
 
 0.86881
 
 0.65472
 
 Not queried
 
  1.2422200
 
       NA
 
       NA
  
 
  
 6
 
  28553927
 
  28555929
 
  ENSEMBL   UCSC 
 
 2003
 
 37
 
 35526
 
 island
 
 0.01846
 
 0.05442
 
 Not queried
 
  1.2418304
 
       NA
 
       NA
  
 
  
 8
 
  21924859
 
  21925182
 
  ENSEMBL   UCSC 
 
  324
 
  4
 
 39163
 
 gene;island
 
 0.83443
 
 0.68652
 
 Not queried
 
  1.2413023
 
       NA
 
       NA
  
 
  
 11
 
 134216065
 
 134216220
 
  ENSEMBL   UCSC 
 
  156
 
  4
 
 6977
 
 gene;island
 
 0.54046
 
 0.80884
 
 Not queried
 
  1.2411731
 
       NA
 
       NA
  
 
  
 10
 
  74386032
 
  74386252
 
  ENSEMBL   UCSC 
 
  221
 
  6
 
 5135
 
 tss;island
 
 0.03023
 
 0.01872
 
 Not queried
 
  1.2409406
 
       NA
 
       NA
  
 
  
 X
 
  67653156
 
  67653925
 
  ENSEMBL   UCSC 
 
  770
 
 10
 
 41834
 
 island
 
 0.06390
 
 0.02411
 
 Not queried
 
  1.2407246
 
       NA
 
       NA
  
 
  
 16
 
  71496259
 
  71496385
 
  ENSEMBL   UCSC 
 
  127
 
  4
 
 15315
 
 tss
 
 0.08581
 
 0.01961
 
 Not queried
 
  1.2401698
 
       NA
 
       NA
  
 
  
 1
 
   2003864
 
   2004346
 
  ENSEMBL   UCSC 
 
  483
 
  5
 
 56
 
 gene;tss;island
 
 0.83148
 
 0.94318
 
 Not queried
 
  1.2400742
 
       NA
 
       NA
  
 
  
 22
 
  45809244
 
  45809454
 
  ENSEMBL   UCSC 
 
  211
 
  6
 
 26862
 
 gene
 
 0.49779
 
 0.25488
 
 Not queried
 
  1.2400634
 
       NA
 
       NA
  
 
  
 1
 
 165324679
 
 165325231
 
  ENSEMBL   UCSC 
 
  553
 
  7
 
 3915
 
 island
 
 0.06695
 
 0.15697
 
 Not queried
 
  1.2400272
 
       NA
 
       NA
  
 
  
 16
 
  81743587
 
  81743972
 
  ENSEMBL   UCSC 
 
  386
 
  7
 
 14825
 
 gene
 
 0.84785
 
 0.91939
 
 Not queried
 
  1.2393896
 
       NA
 
       NA
  
 
  
 1
 
  39324622
 
  39325107
 
  ENSEMBL   UCSC 
 
  486
 
  4
 
 442
 
 gene;island
 
 0.02280
 
 0.03957
 
 Not queried
 
  1.2392454
 
       NA
 
       NA
  
 
  
 6
 
  26272200
 
  26272695
 
  ENSEMBL   UCSC 
 
  496
 
  6
 
 34639
 
 tss;island
 
 0.63020
 
 0.79145
 
 Not queried
 
  1.2390889
 
       NA
 
       NA
  
 
  
 3
 
  13114343
 
  13114803
 
  ENSEMBL   UCSC 
 
  461
 
  6
 
 28858
 
 island
 
 0.46160
 
 0.71621
 
 Not queried
 
  1.2386029
 
       NA
 
       NA
  
 
  
 8
 
    832917
 
    833183
 
  ENSEMBL   UCSC 
 
  267
 
  4
 
 39943
 
 island
 
 0.76545
 
 0.88027
 
 Not queried
 
  1.2382347
 
       NA
 
       NA
  
 
  
 12
 
   6486598
 
   6486709
 
  ENSEMBL   UCSC 
 
  112
 
  4
 
 9485
 
 tss
 
 0.67748
 
 0.83281
 
 Not queried
 
  1.2382318
 
       NA
 
       NA
  
 
  
 1
 
 150521958
 
 150522367
 
  ENSEMBL   UCSC 
 
  410
 
  4
 
 738
 
 gene
 
 0.16680
 
 0.33054
 
 Not queried
 
  1.2382001
 
       NA
 
       NA
  
 
  
 10
 
 103603722
 
 103604019
 
  ENSEMBL   UCSC 
 
  298
 
  5
 
 5295
 
 tss
 
 0.25539
 
 0.06533
 
 Not queried
 
  1.2380918
 
       NA
 
       NA
  
 
  
 14
 
 105293578
 
 105293957
 
  ENSEMBL   UCSC 
 
  380
 
  5
 
 13192
 
 island
 
 0.02787
 
 0.03918
 
 Not queried
 
  1.2379770
 
       NA
 
       NA
  
 
  
 1
 
  38231746
 
  38231980
 
  ENSEMBL   UCSC 
 
  235
 
  4
 
 1505
 
 tss;island
 
 0.06505
 
 0.18312
 
 Not queried
 
  1.2377345
 
       NA
 
       NA
  
 
  
 4
 
  17516413
 
  17516752
 
  ENSEMBL   UCSC 
 
  340
 
  6
 
 30268
 
 tss
 
 0.91248
 
 0.79927
 
 Not queried
 
  1.2367774
 
       NA
 
       NA
  
 
  
 19
 
  18965636
 
  18966003
 
  ENSEMBL   UCSC 
 
  368
 
  4
 
 19907
 
 gene;island
 
 0.86465
 
 0.90393
 
 Not queried
 
  1.2364222
 
       NA
 
       NA
  
 
  
 7
 
 157697057
 
 157697628
 
  ENSEMBL   UCSC 
 
  572
 
  5
 
 37289
 
 gene;island
 
 0.71497
 
 0.85517
 
 Not queried
 
  1.2350862
 
       NA
 
       NA
  
 
  
 5
 
 111495974
 
 111496724
 
  ENSEMBL   UCSC 
 
  751
 
 10
 
 32151
 
 tss;island
 
 0.04747
 
 0.12260
 
 Not queried
 
  1.2349083
 
       NA
 
       NA
  
 
  
 17
 
  48133890
 
  48134226
 
  ENSEMBL   UCSC 
 
  337
 
  4
 
 16644
 
 gene;island
 
 0.01712
 
 0.03065
 
 Not queried
 
  1.2346252
 
       NA
 
       NA
  
 
  
 1
 
  47489282
 
  47489543
 
  ENSEMBL   UCSC 
 
  262
 
  4
 
 513
 
 gene
 
 0.03749
 
 0.10396
 
 Not queried
 
  1.2345665
 
       NA
 
       NA
  
 
  
 19
 
  10172712
 
  10172899
 
  ENSEMBL   UCSC 
 
  188
 
  4
 
 19770
 
 gene;island
 
 0.06937
 
 0.19907
 
 Not queried
 
  1.2342083
 
       NA
 
       NA
  
 
  
 4
 
 170678860
 
 170679082
 
  ENSEMBL   UCSC 
 
  223
 
  5
 
 30129
 
 gene
 
 0.01400
 
 0.03231
 
 Not queried
 
  1.2341081
 
       NA
 
       NA
  
 
  
 17
 
  41477271
 
  41477468
 
  ENSEMBL   UCSC 
 
  198
 
  4
 
 16562
 
 gene;island
 
 0.54166
 
 0.72839
 
 Not queried
 
  1.2340677
 
       NA
 
       NA
  
 
  
 10
 
 103530053
 
 103530255
 
  ENSEMBL   UCSC 
 
  203
 
  4
 
 4715
 
 gene
 
 0.86524
 
 0.77223
 
 Not queried
 
  1.2338452
 
       NA
 
       NA
  
 
  
 20
 
  37076639
 
  37076913
 
  ENSEMBL   UCSC 
 
  275
 
  4
 
 25381
 
 gene;tss;island
 
 0.92333
 
 0.83648
 
 Not queried
 
  1.2338143
 
       NA
 
       NA
  
 
  
 6
 
 168533375
 
 168533689
 
  ENSEMBL   UCSC 
 
  315
 
  4
 
 36597
 
 island
 
 0.38044
 
 0.65739
 
 Not queried
 
  1.2337794
 
       NA
 
       NA
  
 
  
 10
 
 103543078
 
 103543259
 
  ENSEMBL   UCSC 
 
  182
 
  8
 
 6053
 
 island
 
 0.02439
 
 0.04699
 
 Not queried
 
  1.2335855
 
       NA
 
       NA
  
 
  
 2
 
  45233390
 
  45233773
 
  ENSEMBL   UCSC 
 
  384
 
  4
 
 22628
 
 gene;island
 
 0.83742
 
 0.64790
 
 Not queried
 
  1.2334626
 
       NA
 
       NA
  
 
  
 8
 
  72470700
 
  72471153
 
  ENSEMBL   UCSC 
 
  454
 
  4
 
 40316
 
 island
 
 0.05375
 
 0.02123
 
 Not queried
 
  1.2332576
 
       NA
 
       NA
  
 
  
 16
 
    731772
 
    732216
 
  ENSEMBL   UCSC 
 
  445
 
  4
 
 14508
 
 gene;island
 
 0.82344
 
 0.91771
 
 Not queried
 
  1.2329515
 
       NA
 
       NA
  
 
  
 6
 
 100055662
 
 100056127
 
  ENSEMBL   UCSC 
 
  466
 
  5
 
 34363
 
 gene;island
 
 0.23352
 
 0.27803
 
 Not queried
 
  1.2329301
 
       NA
 
       NA
  
 
  
 7
 
 117067616
 
 117067978
 
  ENSEMBL   UCSC 
 
  363
 
  8
 
 37790
 
 tss;island
 
 0.92792
 
 0.84732
 
 Not queried
 
  1.2326782
 
       NA
 
       NA
  
 
  
 X
 
  71497221
 
  71497424
 
  ENSEMBL   UCSC 
 
  204
 
  4
 
 41383
 
 tss
 
 0.01851
 
 0.04463
 
 Not queried
 
  1.2324947
 
       NA
 
       NA
  
 
  
 6
 
  30686747
 
  30686922
 
  ENSEMBL   UCSC 
 
  176
 
  4
 
 34721
 
 tss;island
 
 0.74293
 
 0.69466
 
 Not queried
 
  1.2322304
 
       NA
 
       NA
  
 
  
 1
 
 247712110
 
 247712383
 
  ENSEMBL   UCSC 
 
  274
 
  6
 
 2366
 
 tss
 
 0.78163
 
 0.90064
 
 Not queried
 
  1.2316436
 
       NA
 
       NA
  
 
  
 20
 
   1716270
 
   1716632
 
  ENSEMBL   UCSC 
 
  363
 
  4
 
 25899
 
 island
 
 0.70184
 
 0.82180
 
 Not queried
 
  1.2315041
 
       NA
 
       NA
  
 
  
 3
 
 157217556
 
 157217842
 
  ENSEMBL   UCSC 
 
  287
 
  6
 
 28636
 
 tss
 
 0.04590
 
 0.09253
 
 Not queried
 
  1.2313922
 
       NA
 
       NA
  
 
  
 X
 
  56590097
 
  56590537
 
  ENSEMBL   UCSC 
 
  441
 
  7
 
 41004
 
 gene
 
 0.02970
 
 0.01287
 
 Not queried
 
  1.2311796
 
       NA
 
       NA
  
 
  
 8
 
   8085496
 
   8085544
 
  ENSEMBL   UCSC 
 
   49
 
  4
 
 39477
 
 tss;island
 
 0.02014
 
 0.03807
 
 Not queried
 
  1.2311626
 
       NA
 
       NA
  
 
  
 11
 
 132107419
 
 132107699
 
  ENSEMBL   UCSC 
 
  281
 
  6
 
 6959
 
 gene
 
 0.93716
 
 0.84298
 
 Not queried
 
  1.2310436
 
       NA
 
       NA
  
 
  
 11
 
  34380546
 
  34380839
 
  ENSEMBL   UCSC 
 
  294
 
  4
 
 8181
 
 island
 
 0.02738
 
 0.06709
 
 Not queried
 
  1.2308921
 
       NA
 
       NA
  
 
  
 19
 
   1355910
 
   1356315
 
  ENSEMBL   UCSC 
 
  406
 
  9
 
 20264
 
 tss
 
 0.27814
 
 0.09361
 
 Not queried
 
  1.2303109
 
       NA
 
       NA
  
 
  
 6
 
 137143497
 
 137143711
 
  ENSEMBL   UCSC 
 
  215
 
  7
 
 36441
 
 island
 
 0.01223
 
 0.02631
 
 Not queried
 
  1.2302878
 
       NA
 
       NA
  
 
  
 1
 
  28414998
 
  28415421
 
  ENSEMBL   UCSC 
 
  424
 
 10
 
 2931
 
 island
 
 0.02053
 
 0.03059
 
 Not queried
 
  1.2298329
 
       NA
 
       NA
  
 
  
 19
 
   1438017
 
   1438306
 
  ENSEMBL   UCSC 
 
  290
 
 10
 
 20268
 
 tss
 
 0.07814
 
 0.02645
 
 Not queried
 
  1.2295559
 
       NA
 
       NA
  
 
  
 10
 
 131265059
 
 131265796
 
  ENSEMBL   UCSC 
 
  738
 
 15
 
 6239
 
 island
 
 0.03244
 
 0.08198
 
 Not queried
 
  1.2295341
 
       NA
 
       NA
  
 
  
 14
 
  65569751
 
  65570008
 
  ENSEMBL   UCSC 
 
  258
 
  4
 
 12439
 
 tss;island
 
 0.00760
 
 0.02249
 
 Not queried
 
  1.2293766
 
       NA
 
       NA
  
 
  
 11
 
  63852804
 
  63853037
 
  ENSEMBL   UCSC 
 
  234
 
  5
 
 6644
 
 gene;island
 
 0.85336
 
 0.95808
 
 Not queried
 
  1.2293339
 
       NA
 
       NA
  
 
  
 1
 
  92764174
 
  92764431
 
  ENSEMBL   UCSC 
 
  258
 
  6
 
 1746
 
 tss
 
 0.01189
 
 0.02384
 
 Not queried
 
  1.2289348
 
       NA
 
       NA
  
 
  
 1
 
   6663269
 
   6663664
 
  ENSEMBL   UCSC 
 
  396
 
  5
 
 1209
 
 tss;island
 
 0.02716
 
 0.05154
 
 Not queried
 
  1.2282861
 
       NA
 
       NA
  
 
  
 20
 
  62153067
 
  62153431
 
  ENSEMBL   UCSC 
 
  365
 
  4
 
 25463
 
 gene;island
 
 0.75667
 
 0.58753
 
 Not queried
 
  1.2282413
 
       NA
 
       NA
  
 
  
 5
 
 118324324
 
 118324619
 
  ENSEMBL   UCSC 
 
  296
 
  5
 
 32175
 
 tss;island
 
 0.03585
 
 0.05535
 
 Not queried
 
  1.2282187
 
       NA
 
       NA
  
 
  
 11
 
  71710885
 
  71711087
 
  ENSEMBL   UCSC 
 
  203
 
  4
 
 6788
 
 gene
 
 0.64726
 
 0.81400
 
 Not queried
 
  1.2278802
 
       NA
 
       NA
  
 
  
 10
 
 134756164
 
 134756707
 
  ENSEMBL   UCSC 
 
  544
 
  6
 
 5433
 
 tss;island
 
 0.15213
 
 0.44089
 
 Not queried
 
  1.2278369
 
       NA
 
       NA
  
 
  
 1
 
 165513253
 
 165513361
 
  ENSEMBL   UCSC 
 
  109
 
  5
 
 862
 
 gene;tss
 
 0.54800
 
 0.35182
 
 Not queried
 
  1.2276396
 
       NA
 
       NA
  
 
  
 14
 
  31926776
 
  31926862
 
  ENSEMBL   UCSC 
 
   87
 
  4
 
 12314
 
 tss;island
 
 0.01602
 
 0.03793
 
 Not queried
 
  1.2274837
 
       NA
 
       NA
  
 
  
 4
 
  91156040
 
  91156290
 
  ENSEMBL   UCSC 
 
  251
 
  4
 
 30013
 
 gene
 
 0.48927
 
 0.76307
 
 Not queried
 
  1.2270616
 
       NA
 
       NA
  
 
  
 6
 
  39895360
 
  39895717
 
  ENSEMBL   UCSC 
 
  358
 
  5
 
 34261
 
 gene
 
 0.77167
 
 0.88776
 
 Not queried
 
  1.2267117
 
       NA
 
       NA
  
 
  
 4
 
  37246688
 
  37247022
 
  ENSEMBL   UCSC 
 
  335
 
  4
 
 30922
 
 island
 
 0.00870
 
 0.02335
 
 Not queried
 
  1.2265723
 
       NA
 
       NA
  
 
  
 8
 
 118532625
 
 118532951
 
  ENSEMBL   UCSC 
 
  327
 
  8
 
 39819
 
 tss
 
 0.01025
 
 0.03548
 
 Not queried
 
  1.2263941
 
       NA
 
       NA
  
 
  
 11
 
 134336336
 
 134336476
 
  ENSEMBL   UCSC 
 
  141
 
  4
 
 8964
 
 island
 
 0.32442
 
 0.62444
 
 Not queried
 
  1.2261156
 
       NA
 
       NA
  
 
  
 6
 
  29323191
 
  29323330
 
  ENSEMBL   UCSC 
 
  140
 
  4
 
 33589
 
 gene
 
 0.54593
 
 0.70017
 
 Not queried
 
  1.2258312
 
       NA
 
       NA
  
 
  
 2
 
  54086854
 
  54087066
 
  ENSEMBL   UCSC 
 
  213
 
  4
 
 22643
 
 gene
 
 0.78014
 
 0.89240
 
 Not queried
 
  1.2253756
 
       NA
 
       NA
  
 
  
 6
 
 144329672
 
 144330345
 
  ENSEMBL   UCSC 
 
  674
 
 15
 
 35205
 
 tss
 
 0.26061
 
 0.13102
 
 Not queried
 
  1.2251728
 
       NA
 
       NA
  
 
  
 14
 
  60632234
 
  60632532
 
  ENSEMBL   UCSC 
 
  299
 
  4
 
 12408
 
 tss
 
 0.20668
 
 0.10683
 
 Not queried
 
  1.2249948
 
       NA
 
       NA
  
 
  
 1
 
    848379
 
    848609
 
  ENSEMBL   UCSC 
 
  231
 
  4
 
 2374
 
 island
 
 0.45590
 
 0.62141
 
 Not queried
 
  1.2245050
 
       NA
 
       NA
  
 
  
 19
 
  12792705
 
  12792793
 
  ENSEMBL   UCSC 
 
   89
 
  5
 
 20468
 
 tss;island
 
 0.00467
 
 0.01232
 
 Not queried
 
  1.2244254
 
       NA
 
       NA
  
 
  
 8
 
  49173116
 
  49173425
 
  ENSEMBL   UCSC 
 
  310
 
  4
 
 40214
 
 island
 
 0.91832
 
 0.95567
 
 Not queried
 
  1.2240147
 
       NA
 
       NA
  
 
  
 X
 
  11129115
 
  11129352
 
  ENSEMBL   UCSC 
 
  238
 
  8
 
 41182
 
 tss;island
 
 0.02625
 
 0.05140
 
 Not queried
 
  1.2236614
 
       NA
 
       NA
  
 
  
 12
 
 109728043
 
 109728241
 
  ENSEMBL   UCSC 
 
  199
 
  4
 
 9265
 
 gene;island
 
 0.66938
 
 0.82657
 
 Not queried
 
  1.2231720
 
       NA
 
       NA
  
 
  
 2
 
  44001018
 
  44001158
 
  ENSEMBL   UCSC 
 
  141
 
  5
 
 23290
 
 tss
 
 0.01214
 
 0.03356
 
 Not queried
 
  1.2231662
 
       NA
 
       NA
  
 
  
 6
 
  33171319
 
  33171491
 
  ENSEMBL   UCSC 
 
  173
 
  4
 
 34160
 
 gene;tss;island
 
 0.80726
 
 0.91505
 
 Not queried
 
  1.2231289
 
       NA
 
       NA
  
 
  
 2
 
  73298805
 
  73299297
 
  ENSEMBL   UCSC 
 
  493
 
 10
 
 24371
 
 island
 
 0.02790
 
 0.02569
 
 Not queried
 
  1.2229687
 
       NA
 
       NA
  
 
  
 1
 
  17085860
 
  17086071
 
  ENSEMBL   UCSC 
 
  212
 
  4
 
 261
 
 gene;island
 
 0.48657
 
 0.70228
 
 Not queried
 
  1.2229448
 
       NA
 
       NA
  
 
  
 17
 
  32906394
 
  32906622
 
  ENSEMBL   UCSC 
 
  229
 
  4
 
 17227
 
 tss
 
 0.00473
 
 0.01250
 
 Not queried
 
  1.2226590
 
       NA
 
       NA
  
 
  
 6
 
 101847318
 
 101847706
 
  ENSEMBL   UCSC 
 
  389
 
  6
 
 34368
 
 gene;island
 
 0.11973
 
 0.05853
 
 Not queried
 
  1.2225036
 
       NA
 
       NA
  
 
  
 19
 
  50370992
 
  50371215
 
  ENSEMBL   UCSC 
 
  224
 
  4
 
 20930
 
 tss;island
 
 0.00494
 
 0.01472
 
 Not queried
 
  1.2218973
 
       NA
 
       NA
  
 
  
 22
 
  26908119
 
  26908376
 
  ENSEMBL   UCSC 
 
  258
 
  4
 
 26784
 
 gene
 
 0.01469
 
 0.01031
 
 Not queried
 
  1.2218566
 
       NA
 
       NA
  
 
  
 X
 
  37706936
 
  37707161
 
  ENSEMBL   UCSC 
 
  226
 
  4
 
 41255
 
 tss
 
 0.26712
 
 0.13619
 
 Not queried
 
  1.2217245
 
       NA
 
       NA
  
 
  
 4
 
 159593022
 
 159593173
 
  ENSEMBL   UCSC 
 
  152
 
  5
 
 30117
 
 gene
 
 0.00500
 
 0.01084
 
 Not queried
 
  1.2213102
 
       NA
 
       NA
  
 
  
 1
 
   3147891
 
   3148005
 
  ENSEMBL   UCSC 
 
  115
 
  4
 
 109
 
 gene;island
 
 0.86957
 
 0.91474
 
 Not queried
 
  1.2209357
 
       NA
 
       NA
  
 
  
 5
 
 127871927
 
 127872329
 
  ENSEMBL   UCSC 
 
  403
 
  5
 
 31681
 
 gene;island
 
 0.54723
 
 0.27791
 
 Not queried
 
  1.2207538
 
       NA
 
       NA
  
 
  
 19
 
  19280969
 
  19281559
 
  ENSEMBL   UCSC 
 
  591
 
 11
 
 19912
 
 gene;island
 
 0.02698
 
 0.05178
 
 Not queried
 
  1.2205415
 
       NA
 
       NA
  
 
  
 19
 
  19281140
 
  19281559
 
  ENSEMBL   UCSC 
 
  420
 
  9
 
 20600
 
 tss
 
 0.01228
 
 0.02816
 
 Not queried
 
  1.2205415
 
       NA
 
       NA
  
 
  
 3
 
 126422892
 
 126423087
 
  ENSEMBL   UCSC 
 
  196
 
  7
 
 28519
 
 tss
 
 0.01004
 
 0.01709
 
 Not queried
 
  1.2194810
 
       NA
 
       NA
  
 
  
 4
 
  81256552
 
  81256850
 
  ENSEMBL   UCSC 
 
  299
 
  7
 
 30415
 
 tss
 
 0.03116
 
 0.05870
 
 Not queried
 
  1.2188487
 
       NA
 
       NA
  
 
  
 12
 
  49319263
 
  49319767
 
  ENSEMBL   UCSC 
 
  505
 
 10
 
 9652
 
 tss;island
 
 0.82332
 
 0.92002
 
 Not queried
 
  1.2188123
 
       NA
 
       NA
  
 
  
 1
 
  31845923
 
  31846110
 
  ENSEMBL   UCSC 
 
  188
 
  6
 
 1442
 
 tss
 
 0.05234
 
 0.04655
 
 Not queried
 
  1.2187865
 
       NA
 
       NA
  
 
  
 X
 
  18908079
 
  18908380
 
  ENSEMBL   UCSC 
 
  302
 
  5
 
 41213
 
 tss
 
 0.50805
 
 0.72040
 
 Not queried
 
  1.2186887
 
       NA
 
       NA
  
 
  
 6
 
 168426063
 
 168426413
 
  ENSEMBL   UCSC 
 
  351
 
  5
 
 34492
 
 gene
 
 0.87859
 
 0.94395
 
 Not queried
 
  1.2186774
 
       NA
 
       NA
  
 
  
 2
 
 176971820
 
 176972113
 
  ENSEMBL   UCSC 
 
  294
 
  8
 
 24857
 
 island
 
 0.01908
 
 0.05853
 
 Not queried
 
  1.2184812
 
       NA
 
       NA
  
 
  
 16
 
  67977865
 
  67978216
 
  ENSEMBL   UCSC 
 
  352
 
  5
 
 14778
 
 gene
 
 0.88402
 
 0.77675
 
 Not queried
 
  1.2181978
 
       NA
 
       NA
  
 
  
 2
 
 176046457
 
 176046632
 
  ENSEMBL   UCSC 
 
  176
 
  7
 
 23691
 
 tss;island
 
 0.11522
 
 0.03179
 
 Not queried
 
  1.2179469
 
       NA
 
       NA
  
 
  
 6
 
  26018058
 
  26018185
 
  ENSEMBL   UCSC 
 
  128
 
  5
 
 34613
 
 tss
 
 0.85727
 
 0.92682
 
 Not queried
 
  1.2178316
 
       NA
 
       NA
  
 
  
 6
 
  26017939
 
  26018185
 
  ENSEMBL   UCSC 
 
  247
 
  7
 
 35441
 
 island
 
 0.85727
 
 0.91536
 
 Not queried
 
  1.2178316
 
       NA
 
       NA
  
 
  
 17
 
  39507077
 
  39507261
 
  ENSEMBL   UCSC 
 
  185
 
  4
 
 17306
 
 tss
 
 0.62204
 
 0.36756
 
 Not queried
 
  1.2172630
 
       NA
 
       NA
  
 
  
 6
 
  26027513
 
  26027625
 
  ENSEMBL   UCSC 
 
  113
 
  6
 
 34616
 
 tss
 
 0.00780
 
 0.02429
 
 Not queried
 
  1.2171465
 
       NA
 
       NA
  
 
  
 10
 
 102881114
 
 102881545
 
  ENSEMBL   UCSC 
 
  432
 
  6
 
 4709
 
 gene;island
 
 0.66358
 
 0.50781
 
 Not queried
 
  1.2171335
 
       NA
 
       NA
  
 
  
 6
 
  32085017
 
  32085780
 
  ENSEMBL   UCSC 
 
  764
 
 12
 
 33990
 
 gene
 
 0.65991
 
 0.86491
 
 Not queried
 
  1.2169351
 
       NA
 
       NA
  
 
  
 8
 
   1789169
 
   1789670
 
  ENSEMBL   UCSC 
 
  502
 
  6
 
 39114
 
 gene
 
 0.76590
 
 0.91620
 
 Not queried
 
  1.2168833
 
       NA
 
       NA
  
 
  
 1
 
 243053673
 
 243054071
 
  ENSEMBL   UCSC 
 
  399
 
  4
 
 4370
 
 island
 
 0.07129
 
 0.14058
 
 Not queried
 
  1.2167082
 
       NA
 
       NA
  
 
  
 2
 
  98329691
 
  98330020
 
  ENSEMBL   UCSC 
 
  330
 
  5
 
 23493
 
 tss
 
 0.58025
 
 0.76257
 
 Not queried
 
  1.2162006
 
       NA
 
       NA
  
 
  
 22
 
  30234293
 
  30234508
 
  ENSEMBL   UCSC 
 
  216
 
  4
 
 27012
 
 tss
 
 0.00901
 
 0.01749
 
 Not queried
 
  1.2161515
 
       NA
 
       NA
  
 
  
 10
 
  90967113
 
  90967233
 
  ENSEMBL   UCSC 
 
  121
 
  6
 
 5194
 
 tss
 
 0.01818
 
 0.04121
 
 Not queried
 
  1.2158342
 
       NA
 
       NA
  
 
  
 X
 
 151903039
 
 151903224
 
  ENSEMBL   UCSC 
 
  186
 
  5
 
 41572
 
 tss
 
 0.84608
 
 0.69616
 
 Not queried
 
  1.2155612
 
       NA
 
       NA
  
 
  
 X
 
  55026807
 
  55027018
 
  ENSEMBL   UCSC 
 
  212
 
  4
 
 41000
 
 gene
 
 0.20172
 
 0.38525
 
 Not queried
 
  1.2154829
 
       NA
 
       NA
  
 
  
 5
 
  83017000
 
  83017184
 
  ENSEMBL   UCSC 
 
  185
 
  4
 
 32101
 
 tss;island
 
 0.06521
 
 0.03039
 
 Not queried
 
  1.2153197
 
       NA
 
       NA
  
 
  
 2
 
  73340007
 
  73340489
 
  ENSEMBL   UCSC 
 
  483
 
  5
 
 24372
 
 island
 
 0.00488
 
 0.01216
 
 Not queried
 
  1.2152942
 
       NA
 
       NA
  
 
  
 12
 
  62654212
 
  62654503
 
  ENSEMBL   UCSC 
 
  292
 
  4
 
 9174
 
 gene
 
 0.02544
 
 0.03217
 
 Not queried
 
  1.2151263
 
       NA
 
       NA
  
 
  
 2
 
  12880542
 
  12880961
 
  ENSEMBL   UCSC 
 
  420
 
  4
 
 22541
 
 gene
 
 0.89736
 
 0.95346
 
 Not queried
 
  1.2150466
 
       NA
 
       NA
  
 
  
 18
 
  21242319
 
  21242479
 
  ENSEMBL   UCSC 
 
  161
 
  4
 
 19041
 
 gene;island
 
 0.13225
 
 0.05010
 
 Not queried
 
  1.2150130
 
       NA
 
       NA
  
 
  
 6
 
 168378559
 
 168379113
 
  ENSEMBL   UCSC 
 
  555
 
  8
 
 35280
 
 tss;island
 
 0.86770
 
 0.94172
 
 Not queried
 
  1.2149756
 
       NA
 
       NA
  
 
  
 16
 
  29855904
 
  29856194
 
  ENSEMBL   UCSC 
 
  291
 
  4
 
 14684
 
 gene;island
 
 0.80430
 
 0.91852
 
 Not queried
 
  1.2148360
 
       NA
 
       NA
  
 
  
 5
 
  68787696
 
  68788173
 
  ENSEMBL   UCSC 
 
  478
 
  9
 
 32791
 
 island
 
 0.02367
 
 0.04864
 
 Not queried
 
  1.2146228
 
       NA
 
       NA
  
 
  
 16
 
  88163698
 
  88163879
 
  ENSEMBL   UCSC 
 
  182
 
  4
 
 16160
 
 island
 
 0.93139
 
 0.91234
 
 Not queried
 
  1.2145751
 
       NA
 
       NA
  
 
  
 10
 
  88728073
 
  88728361
 
  ENSEMBL   UCSC 
 
  289
 
  6
 
 5890
 
 island
 
 0.68172
 
 0.84878
 
 Not queried
 
  1.2143930
 
       NA
 
       NA
  
 
  
 1
 
 155317402
 
 155317652
 
  ENSEMBL   UCSC 
 
  251
 
  4
 
 804
 
 gene;tss
 
 0.82335
 
 0.91698
 
 Not queried
 
  1.2142409
 
       NA
 
       NA
  
 
  
 19
 
  51219975
 
  51220537
 
  ENSEMBL   UCSC 
 
  563
 
 10
 
 22285
 
 island
 
 0.63607
 
 0.49399
 
 Not queried
 
  1.2141907
 
       NA
 
       NA
  
 
  
 22
 
  30476089
 
  30476525
 
  ENSEMBL   UCSC 
 
  437
 
 11
 
 27361
 
 island
 
 0.08215
 
 0.20503
 
 Not queried
 
  1.2140874
 
       NA
 
       NA
  
 
  
 15
 
  73661340
 
  73662002
 
  ENSEMBL   UCSC 
 
  663
 
  8
 
 14260
 
 island
 
 0.19206
 
 0.08127
 
 Not queried
 
  1.2137079
 
       NA
 
       NA
  
 
  
 2
 
  26785834
 
  26786046
 
  ENSEMBL   UCSC 
 
  213
 
  4
 
 22578
 
 gene;island
 
 0.68874
 
 0.79295
 
 Not queried
 
  1.2137066
 
       NA
 
       NA
  
 
  
 19
 
  49220223
 
  49220485
 
  ENSEMBL   UCSC 
 
  263
 
  4
 
 20888
 
 tss
 
 0.65703
 
 0.47046
 
 Not queried
 
  1.2134275
 
       NA
 
       NA
  
 
  
 11
 
  17717574
 
  17717818
 
  ENSEMBL   UCSC 
 
  245
 
  4
 
 8081
 
 island
 
 0.89066
 
 0.74626
 
 Not queried
 
  1.2132836
 
       NA
 
       NA
  
 
  
 19
 
  58446745
 
  58446988
 
  ENSEMBL   UCSC 
 
  244
 
  8
 
 21092
 
 tss
 
 0.01908
 
 0.04468
 
 Not queried
 
  1.2130216
 
       NA
 
       NA
  
 
  
 14
 
  73925161
 
  73925225
 
  ENSEMBL   UCSC 
 
   65
 
  4
 
 12102
 
 gene
 
 0.01790
 
 0.03919
 
 Not queried
 
  1.2127320
 
       NA
 
       NA
  
 
  
 12
 
 129338783
 
 129338939
 
  ENSEMBL   UCSC 
 
  157
 
  4
 
 9385
 
 gene;island
 
 0.03898
 
 0.13184
 
 Not queried
 
  1.2126372
 
       NA
 
       NA
  
 
  
 22
 
  31643997
 
  31644282
 
  ENSEMBL   UCSC 
 
  286
 
  4
 
 26800
 
 gene;tss
 
 0.03985
 
 0.08148
 
 Not queried
 
  1.2121598
 
       NA
 
       NA
  
 
  
 16
 
    734426
 
    734844
 
  ENSEMBL   UCSC 
 
  419
 
  8
 
 15444
 
 island
 
 0.05727
 
 0.11756
 
 Not queried
 
  1.2121202
 
       NA
 
       NA
  
 
  
 4
 
  36245396
 
  36245632
 
  ENSEMBL   UCSC 
 
  237
 
  5
 
 29929
 
 gene;island
 
 0.01706
 
 0.03865
 
 Not queried
 
  1.2120699
 
       NA
 
       NA
  
 
  
 14
 
 105147461
 
 105147781
 
  ENSEMBL   UCSC 
 
  321
 
  5
 
 13184
 
 island
 
 0.38390
 
 0.48928
 
 Not queried
 
  1.2117022
 
       NA
 
       NA
  
 
  
 16
 
  69139418
 
  69139629
 
  ENSEMBL   UCSC 
 
  212
 
  6
 
 15294
 
 tss;island
 
 0.02958
 
 0.01132
 
 Not queried
 
  1.2111861
 
       NA
 
       NA
  
 
  
 5
 
 140998609
 
 140998650
 
  ENSEMBL   UCSC 
 
   42
 
  5
 
 33194
 
 island
 
 0.02878
 
 0.07295
 
 Not queried
 
  1.2111378
 
       NA
 
       NA
  
 
  
 5
 
 176829109
 
 176829349
 
  ENSEMBL   UCSC 
 
  241
 
  4
 
 33386
 
 island
 
 0.25102
 
 0.47483
 
 Not queried
 
  1.2108872
 
       NA
 
       NA
  
 
  
 1
 
  38513245
 
  38513641
 
  ENSEMBL   UCSC 
 
  397
 
  4
 
 1513
 
 tss;island
 
 0.23401
 
 0.05553
 
 Not queried
 
  1.2106326
 
       NA
 
       NA
  
 
  
 10
 
  49864382
 
  49864621
 
  ENSEMBL   UCSC 
 
  240
 
  5
 
 5709
 
 island
 
 0.02810
 
 0.07071
 
 Not queried
 
  1.2105108
 
       NA
 
       NA
  
 
  
 19
 
  57828900
 
  57829175
 
  ENSEMBL   UCSC 
 
  276
 
  4
 
 22427
 
 island
 
 0.07891
 
 0.03249
 
 Not queried
 
  1.2104511
 
       NA
 
       NA
  
 
  
 3
 
 186648175
 
 186648392
 
  ENSEMBL   UCSC 
 
  218
 
  4
 
 28734
 
 tss
 
 0.06490
 
 0.04543
 
 Not queried
 
  1.2103577
 
       NA
 
       NA
  
 
  
 17
 
  17465162
 
  17465476
 
  ENSEMBL   UCSC 
 
  315
 
  5
 
 16428
 
 gene;island
 
 0.87720
 
 0.75534
 
 Not queried
 
  1.2103166
 
       NA
 
       NA
  
 
  
 3
 
 150497174
 
 150497588
 
  ENSEMBL   UCSC 
 
  415
 
  4
 
 29550
 
 island
 
 0.50509
 
 0.73181
 
 Not queried
 
  1.2098789
 
       NA
 
       NA
  
 
  
 4
 
   6675513
 
   6676273
 
  ENSEMBL   UCSC 
 
  761
 
 12
 
 30842
 
 island
 
 0.08317
 
 0.03596
 
 Not queried
 
  1.2097411
 
       NA
 
       NA
  
 
  
 8
 
   6479657
 
   6479846
 
  ENSEMBL   UCSC 
 
  190
 
  4
 
 39127
 
 gene
 
 0.80511
 
 0.91794
 
 Not queried
 
  1.2092115
 
       NA
 
       NA
  
 
  
 18
 
    500817
 
    501234
 
  ENSEMBL   UCSC 
 
  418
 
  5
 
 19117
 
 tss;island
 
 0.17434
 
 0.06346
 
 Not queried
 
  1.2092035
 
       NA
 
       NA
  
 
  
 20
 
  17296030
 
  17296597
 
  ENSEMBL   UCSC 
 
  568
 
  5
 
 25326
 
 gene;island
 
 0.35048
 
 0.55468
 
 Not queried
 
  1.2084166
 
       NA
 
       NA
  
 
  
 8
 
  11626984
 
  11627124
 
  ENSEMBL   UCSC 
 
  141
 
  5
 
 39491
 
 tss
 
 0.02205
 
 0.05858
 
 Not queried
 
  1.2083657
 
       NA
 
       NA
  
 
  
 2
 
  74347215
 
  74347776
 
  ENSEMBL   UCSC 
 
  562
 
  5
 
 24386
 
 island
 
 0.45008
 
 0.66833
 
 Not queried
 
  1.2083260
 
       NA
 
       NA
  
 
  
 1
 
 115259226
 
 115259668
 
  ENSEMBL   UCSC 
 
  443
 
  4
 
 699
 
 gene
 
 0.01597
 
 0.05266
 
 Not queried
 
  1.2081235
 
       NA
 
       NA
  
 
  
 6
 
  26598070
 
  26598694
 
  ENSEMBL   UCSC 
 
  625
 
  8
 
 33550
 
 gene;island
 
 0.66868
 
 0.84074
 
 Not queried
 
  1.2080714
 
       NA
 
       NA
  
 
  
 1
 
   1369466
 
   1369948
 
  ENSEMBL   UCSC 
 
  483
 
  6
 
 1149
 
 tss;island
 
 0.63864
 
 0.84614
 
 Not queried
 
  1.2077798
 
       NA
 
       NA
  
 
  
 5
 
  72793969
 
  72794294
 
  ENSEMBL   UCSC 
 
  326
 
  7
 
 32812
 
 island
 
 0.03909
 
 0.02432
 
 Not queried
 
  1.2076314
 
       NA
 
       NA
  
 
  
 6
 
  32976805
 
  32976897
 
  ENSEMBL   UCSC 
 
   93
 
  4
 
 34113
 
 gene;island
 
 0.87166
 
 0.66888
 
 Not queried
 
  1.2075836
 
       NA
 
       NA
  
 
  
 4
 
 155702172
 
 155702411
 
  ENSEMBL   UCSC 
 
  240
 
  7
 
 30600
 
 tss
 
 0.59462
 
 0.81416
 
 Not queried
 
  1.2074921
 
       NA
 
       NA
  
 
  
 4
 
 155702172
 
 155702610
 
  ENSEMBL   UCSC 
 
  439
 
  9
 
 31334
 
 island
 
 0.59462
 
 0.77344
 
 Not queried
 
  1.2074921
 
       NA
 
       NA
  
 
  
 19
 
  10627706
 
  10628183
 
  ENSEMBL   UCSC 
 
  478
 
  4
 
 19778
 
 gene;island
 
 0.04380
 
 0.02237
 
 Not queried
 
  1.2074572
 
       NA
 
       NA
  
 
  
 12
 
  12509705
 
  12509963
 
  ENSEMBL   UCSC 
 
  259
 
  6
 
 9032
 
 gene
 
 0.02055
 
 0.06450
 
 Not queried
 
  1.2073976
 
       NA
 
       NA
  
 
  
 6
 
  84233917
 
  84234463
 
  ENSEMBL   UCSC 
 
  547
 
  8
 
 34338
 
 gene
 
 0.45902
 
 0.66277
 
 Not queried
 
  1.2070441
 
       NA
 
       NA
  
 
  
 15
 
  84115811
 
  84116151
 
  ENSEMBL   UCSC 
 
  341
 
  9
 
 14372
 
 island
 
 0.01343
 
 0.02094
 
 Not queried
 
  1.2070426
 
       NA
 
       NA
  
 
  
 7
 
  12609907
 
  12610196
 
  ENSEMBL   UCSC 
 
  290
 
  9
 
 37380
 
 tss
 
 0.07086
 
 0.20971
 
 Not queried
 
  1.2069957
 
       NA
 
       NA
  
 
  
 X
 
  49091760
 
  49092146
 
  ENSEMBL   UCSC 
 
  387
 
  7
 
 41792
 
 island
 
 0.09087
 
 0.07230
 
 Not queried
 
  1.2068510
 
       NA
 
       NA
  
 
  
 10
 
  88295210
 
  88295591
 
  ENSEMBL   UCSC 
 
  382
 
  4
 
 5886
 
 island
 
 0.67805
 
 0.46252
 
 Not queried
 
  1.2064182
 
       NA
 
       NA
  
 
  
 10
 
  81145959
 
  81146193
 
  ENSEMBL   UCSC 
 
  235
 
  4
 
 4637
 
 gene;island
 
 0.57410
 
 0.82617
 
 Not queried
 
  1.2058468
 
       NA
 
       NA
  
 
  
 12
 
  49488554
 
  49488898
 
  ENSEMBL   UCSC 
 
  345
 
  4
 
 10359
 
 island
 
 0.15044
 
 0.24237
 
 Not queried
 
  1.2058325
 
       NA
 
       NA
  
 
  
 1
 
 166853278
 
 166853812
 
  ENSEMBL   UCSC 
 
  535
 
  6
 
 3928
 
 island
 
 0.08247
 
 0.06191
 
 Not queried
 
  1.2056460
 
       NA
 
       NA
  
 
  
 12
 
   2800755
 
   2801158
 
  ENSEMBL   UCSC 
 
  404
 
  5
 
 8983
 
 gene;island
 
 0.03652
 
 0.03180
 
 Not queried
 
  1.2048872
 
       NA
 
       NA
  
 
  
 13
 
  46961583
 
  46961897
 
  ENSEMBL   UCSC 
 
  315
 
 10
 
 11685
 
 island
 
 0.01190
 
 0.02580
 
 Not queried
 
  1.2042347
 
       NA
 
       NA
  
 
  
 5
 
   2866767
 
   2866877
 
  ENSEMBL   UCSC 
 
  111
 
  4
 
 32600
 
 island
 
 0.47852
 
 0.66072
 
 Not queried
 
  1.2041299
 
       NA
 
       NA
  
 
  
 10
 
 103070221
 
 103070503
 
  ENSEMBL   UCSC 
 
  283
 
  4
 
 6049
 
 island
 
 0.15343
 
 0.34760
 
 Not queried
 
  1.2040386
 
       NA
 
       NA
  
 
  
 6
 
 152127812
 
 152128024
 
  ENSEMBL   UCSC 
 
  213
 
  4
 
 34448
 
 gene;tss;island
 
 0.88957
 
 0.74027
 
 Not queried
 
  1.2040328
 
       NA
 
       NA
  
 
  
 1
 
 231155449
 
 231155983
 
  ENSEMBL   UCSC 
 
  535
 
 12
 
 1072
 
 gene
 
 0.54788
 
 0.76435
 
 Not queried
 
  1.2039600
 
       NA
 
       NA
  
 
  
 19
 
  44259148
 
  44259196
 
  ENSEMBL   UCSC 
 
   49
 
  6
 
 20798
 
 tss
 
 0.01558
 
 0.03907
 
 Not queried
 
  1.2038020
 
       NA
 
       NA
  
 
  
 19
 
  44259116
 
  44259196
 
  ENSEMBL   UCSC 
 
   81
 
  8
 
 22059
 
 island
 
 0.01182
 
 0.01865
 
 Not queried
 
  1.2038020
 
       NA
 
       NA
  
 
  
 2
 
 178973079
 
 178973251
 
  ENSEMBL   UCSC 
 
  173
 
  6
 
 23714
 
 tss;island
 
 0.78570
 
 0.82974
 
 Not queried
 
  1.2037527
 
       NA
 
       NA
  
 
  
 1
 
 111813509
 
 111813852
 
  ENSEMBL   UCSC 
 
  344
 
  4
 
 3581
 
 island
 
 0.91927
 
 0.84793
 
 Not queried
 
  1.2037223
 
       NA
 
       NA
  
 
  
 7
 
 158220708
 
 158221076
 
  ENSEMBL   UCSC 
 
  369
 
  5
 
 37306
 
 gene;island
 
 0.90398
 
 0.95591
 
 Not queried
 
  1.2037092
 
       NA
 
       NA
  
 
  
 7
 
 127841626
 
 127841927
 
  ENSEMBL   UCSC 
 
  302
 
  4
 
 38827
 
 island
 
 0.16957
 
 0.19011
 
 Not queried
 
  1.2031041
 
       NA
 
       NA
  
 
  
 11
 
  61519687
 
  61520258
 
  ENSEMBL   UCSC 
 
  572
 
  6
 
 8309
 
 island
 
 0.12106
 
 0.03465
 
 Not queried
 
  1.2029285
 
       NA
 
       NA
  
 
  
 19
 
  42636625
 
  42637295
 
  ENSEMBL   UCSC 
 
  671
 
  7
 
 22032
 
 island
 
 0.50626
 
 0.84376
 
 Not queried
 
  1.2029048
 
       NA
 
       NA
  
 
  
 6
 
  29716851
 
  29717656
 
  ENSEMBL   UCSC 
 
  806
 
 19
 
 34689
 
 tss
 
 0.03331
 
 0.18044
 
 Not queried
 
  1.2027336
 
       NA
 
       NA
  
 
  
 7
 
   5266987
 
   5267360
 
  ENSEMBL   UCSC 
 
  374
 
  4
 
 36803
 
 gene;island
 
 0.88023
 
 0.94861
 
 Not queried
 
  1.2023805
 
       NA
 
       NA
  
 
  
 6
 
  29716108
 
  29717656
 
  ENSEMBL   UCSC 
 
 1549
 
 40
 
 35563
 
 island
 
 0.02795
 
 0.06586
 
 Not queried
 
  1.2022585
 
       NA
 
       NA
  
 
  
 1
 
  45082704
 
  45083278
 
  ENSEMBL   UCSC 
 
  575
 
  7
 
 490
 
 gene;island
 
 0.03921
 
 0.07033
 
 Not queried
 
  1.2021483
 
       NA
 
       NA
  
 
  
 17
 
  39969264
 
  39969397
 
  ENSEMBL   UCSC 
 
  134
 
  4
 
 16537
 
 gene;tss;island
 
 0.09619
 
 0.03599
 
 Not queried
 
  1.2019506
 
       NA
 
       NA
  
 
  
 6
 
  29716108
 
  29716804
 
  ENSEMBL   UCSC 
 
  697
 
 21
 
 33629
 
 gene
 
 0.02056
 
 0.04635
 
 Not queried
 
  1.2017834
 
       NA
 
       NA
  
 
  
 10
 
 100996579
 
 100997023
 
  ENSEMBL   UCSC 
 
  445
 
  4
 
 5262
 
 tss;island
 
 0.52909
 
 0.35020
 
 Not queried
 
  1.2016930
 
       NA
 
       NA
  
 
  
 20
 
  18038574
 
  18038771
 
  ENSEMBL   UCSC 
 
  198
 
  4
 
 25572
 
 tss
 
 0.02988
 
 0.06826
 
 Not queried
 
  1.2014045
 
       NA
 
       NA
  
 
  
 2
 
 198650009
 
 198650208
 
  ENSEMBL   UCSC 
 
  200
 
  4
 
 22935
 
 gene;island
 
 0.77591
 
 0.89689
 
 Not queried
 
  1.2013310
 
       NA
 
       NA
  
 
  
 11
 
  43701985
 
  43702737
 
  ENSEMBL   UCSC 
 
  753
 
 12
 
 8196
 
 island
 
 0.02081
 
 0.04601
 
 Not queried
 
  1.2011611
 
       NA
 
       NA
  
 
  
 19
 
   1355910
 
   1356444
 
  ENSEMBL   UCSC 
 
  535
 
 10
 
 19617
 
 gene;island
 
 0.36094
 
 0.09378
 
 Not queried
 
  1.2010514
 
       NA
 
       NA
  
 
  
 2
 
 176971820
 
 176972068
 
  ENSEMBL   UCSC 
 
  249
 
  7
 
 23696
 
 tss
 
 0.01657
 
 0.04831
 
 Not queried
 
  1.2009373
 
       NA
 
       NA
  
 
  
 17
 
  37843793
 
  37844579
 
  ENSEMBL   UCSC 
 
  787
 
 14
 
 17280
 
 tss;island
 
 0.03328
 
 0.01729
 
 Not queried
 
  1.2003611
 
       NA
 
       NA
  
 
  
 16
 
  31119484
 
  31119650
 
  ENSEMBL   UCSC 
 
  167
 
  4
 
 15175
 
 tss;island
 
 0.02241
 
 0.01224
 
 Not queried
 
  1.2001026
 
       NA
 
       NA
  
 
  
 6
 
 110299835
 
 110300233
 
  ENSEMBL   UCSC 
 
  399
 
  7
 
 35102
 
 tss
 
 0.04483
 
 0.04351
 
 Not queried
 
  1.1996307
 
       NA
 
       NA
  
 
  
 1
 
  21996199
 
  21996376
 
  ENSEMBL   UCSC 
 
  178
 
  4
 
 1322
 
 tss;island
 
 0.03450
 
 0.07548
 
 Not queried
 
  1.1995615
 
       NA
 
       NA
  
 
  
 12
 
 111473485
 
 111473809
 
  ENSEMBL   UCSC 
 
  325
 
  4
 
 9278
 
 gene;island
 
 0.12754
 
 0.04561
 
 Not queried
 
  1.1993600
 
       NA
 
       NA
  
 
  
 6
 
 166400871
 
 166401018
 
  ENSEMBL   UCSC 
 
  148
 
  4
 
 35268
 
 tss
 
 0.06287
 
 0.03554
 
 Not queried
 
  1.1992212
 
       NA
 
       NA
  
 
  
 1
 
  20880378
 
  20880663
 
  ENSEMBL   UCSC 
 
  286
 
  4
 
 289
 
 gene;island
 
 0.05164
 
 0.11151
 
 Not queried
 
  1.1991853
 
       NA
 
       NA
  
 
  
 6
 
  32942063
 
  32942808
 
  ENSEMBL   UCSC 
 
  746
 
 10
 
 34106
 
 gene;island
 
 0.03975
 
 0.16243
 
 Not queried
 
  1.1987953
 
       NA
 
       NA
  
 
  
 13
 
 112275633
 
 112276127
 
  ENSEMBL   UCSC 
 
  495
 
  5
 
 11870
 
 island
 
 0.90014
 
 0.93075
 
 Not queried
 
  1.1985559
 
       NA
 
       NA
  
 
  
 6
 
  30852307
 
  30853333
 
  ENSEMBL   UCSC 
 
 1027
 
 18
 
 33763
 
 gene;island
 
 0.05987
 
 0.15494
 
 Not queried
 
  1.1983948
 
       NA
 
       NA
  
 
  
 3
 
 178978849
 
 178979013
 
  ENSEMBL   UCSC 
 
  165
 
  5
 
 27993
 
 gene;tss;island
 
 0.34305
 
 0.14650
 
 Not queried
 
  1.1983487
 
       NA
 
       NA
  
 
  
 10
 
  72201417
 
  72201741
 
  ENSEMBL   UCSC 
 
  325
 
  7
 
 5800
 
 island
 
 0.39567
 
 0.57598
 
 Not queried
 
  1.1982116
 
       NA
 
       NA
  
 
  
 6
 
 101846872
 
 101847058
 
  ENSEMBL   UCSC 
 
  187
 
  5
 
 34367
 
 gene
 
 0.13460
 
 0.04903
 
 Not queried
 
  1.1981156
 
       NA
 
       NA
  
 
  
 7
 
  98099806
 
  98100419
 
  ENSEMBL   UCSC 
 
  614
 
  7
 
 38649
 
 island
 
 0.16262
 
 0.29009
 
 Not queried
 
  1.1979960
 
       NA
 
       NA
  
 
  
 17
 
  45973527
 
  45973779
 
  ENSEMBL   UCSC 
 
  253
 
  4
 
 16612
 
 gene
 
 0.01426
 
 0.05274
 
 Not queried
 
  1.1978930
 
       NA
 
       NA
  
 
  
 7
 
   2632080
 
   2632371
 
  ENSEMBL   UCSC 
 
  292
 
  5
 
 36745
 
 gene;island
 
 0.77467
 
 0.88669
 
 Not queried
 
  1.1978060
 
       NA
 
       NA
  
 
  
 10
 
  71892694
 
  71892787
 
  ENSEMBL   UCSC 
 
   94
 
  6
 
 5113
 
 tss
 
 0.07644
 
 0.21548
 
 Not queried
 
  1.1976594
 
       NA
 
       NA
  
 
  
 11
 
  69590090
 
  69590580
 
  ENSEMBL   UCSC 
 
  491
 
  9
 
 8575
 
 island
 
 0.05279
 
 0.02651
 
 Not queried
 
  1.1976173
 
       NA
 
       NA
  
 
  
 X
 
 144898689
 
 144899069
 
  ENSEMBL   UCSC 
 
  381
 
  5
 
 41558
 
 tss;island
 
 0.00684
 
 0.02270
 
 Not queried
 
  1.1973383
 
       NA
 
       NA
  
 
  
 8
 
  63997977
 
  63998778
 
  ENSEMBL   UCSC 
 
  802
 
 11
 
 40272
 
 island
 
 0.03959
 
 0.01538
 
 Not queried
 
  1.1972978
 
       NA
 
       NA
  
 
  
 2
 
 239755455
 
 239755703
 
  ENSEMBL   UCSC 
 
  249
 
  4
 
 23938
 
 tss;island
 
 0.68892
 
 0.83363
 
 Not queried
 
  1.1971528
 
       NA
 
       NA
  
 
  
 10
 
  76859206
 
  76859441
 
  ENSEMBL   UCSC 
 
  236
 
  7
 
 4626
 
 gene
 
 0.55187
 
 0.76562
 
 Not queried
 
  1.1969079
 
       NA
 
       NA
  
 
  
 2
 
 242179181
 
 242179444
 
  ENSEMBL   UCSC 
 
  264
 
  4
 
 23095
 
 gene;island
 
 0.94744
 
 0.89142
 
 Not queried
 
  1.1968430
 
       NA
 
       NA
  
 
  
 1
 
  28585612
 
  28585917
 
  ENSEMBL   UCSC 
 
  306
 
  6
 
 1423
 
 tss
 
 0.01779
 
 0.03581
 
 Not queried
 
  1.1967158
 
       NA
 
       NA
  
 
  
 1
 
 165910516
 
 165910942
 
  ENSEMBL   UCSC 
 
  427
 
  6
 
 3922
 
 island
 
 0.52648
 
 0.73340
 
 Not queried
 
  1.1965991
 
       NA
 
       NA
  
 
  
 20
 
  44538605
 
  44538675
 
  ENSEMBL   UCSC 
 
   71
 
  4
 
 25399
 
 gene;island
 
 0.72552
 
 0.84907
 
 Not queried
 
  1.1963309
 
       NA
 
       NA
  
 
  
 6
 
  30126161
 
  30126535
 
  ENSEMBL   UCSC 
 
  375
 
  4
 
 33670
 
 gene
 
 0.45649
 
 0.71601
 
 Not queried
 
  1.1958786
 
       NA
 
       NA
  
 
  
 19
 
  50553682
 
  50554067
 
  ENSEMBL   UCSC 
 
  386
 
  7
 
 22264
 
 island
 
 0.02432
 
 0.04633
 
 Not queried
 
  1.1957671
 
       NA
 
       NA
  
 
  
 6
 
  30433749
 
  30434552
 
  ENSEMBL   UCSC 
 
  804
 
 21
 
 35621
 
 island
 
 0.03219
 
 0.07610
 
 Not queried
 
  1.1956416
 
       NA
 
       NA
  
 
  
 5
 
 113697330
 
 113697649
 
  ENSEMBL   UCSC 
 
  320
 
  4
 
 32162
 
 tss;island
 
 0.00418
 
 0.00919
 
 Not queried
 
  1.1951486
 
       NA
 
       NA
  
 
  
 5
 
  32710456
 
  32710882
 
  ENSEMBL   UCSC 
 
  427
 
  6
 
 31949
 
 tss;island
 
 0.02816
 
 0.08674
 
 Not queried
 
  1.1950365
 
       NA
 
       NA
  
 
  
 1
 
 200639085
 
 200639502
 
  ENSEMBL   UCSC 
 
  418
 
  6
 
 4054
 
 island
 
 0.00417
 
 0.03040
 
 Not queried
 
  1.1947654
 
       NA
 
       NA
  
 
  
 7
 
 149569715
 
 149570040
 
  ENSEMBL   UCSC 
 
  326
 
  8
 
 37908
 
 tss
 
 0.46107
 
 0.21047
 
 Not queried
 
  1.1941725
 
       NA
 
       NA
  
 
  
 19
 
  17728459
 
  17728637
 
  ENSEMBL   UCSC 
 
  179
 
  4
 
 19883
 
 gene;island
 
 0.64601
 
 0.76517
 
 Not queried
 
  1.1931943
 
       NA
 
       NA
  
 
  
 6
 
  33156779
 
  33157222
 
  ENSEMBL   UCSC 
 
  444
 
 10
 
 34148
 
 gene;island
 
 0.62091
 
 0.39563
 
 Not queried
 
  1.1930414
 
       NA
 
       NA
  
 
  
 7
 
  44163159
 
  44163430
 
  ENSEMBL   UCSC 
 
  272
 
  6
 
 37500
 
 tss
 
 0.01391
 
 0.03268
 
 Not queried
 
  1.1928728
 
       NA
 
       NA
  
 
  
 15
 
  90171643
 
  90171885
 
  ENSEMBL   UCSC 
 
  243
 
  6
 
 13443
 
 gene;island
 
 0.86151
 
 0.76774
 
 Not queried
 
  1.1926211
 
       NA
 
       NA
  
 
  
 X
 
  64627572
 
  64627755
 
  ENSEMBL   UCSC 
 
  184
 
  4
 
 41829
 
 island
 
 0.36490
 
 0.54061
 
 Not queried
 
  1.1925408
 
       NA
 
       NA
  
 
  
 7
 
 157919649
 
 157920174
 
  ENSEMBL   UCSC 
 
  526
 
  4
 
 37295
 
 gene;island
 
 0.90339
 
 0.75114
 
 Not queried
 
  1.1923966
 
       NA
 
       NA
  
 
  
 10
 
 102289529
 
 102289885
 
  ENSEMBL   UCSC 
 
  357
 
  5
 
 6008
 
 island
 
 0.01955
 
 0.05409
 
 Not queried
 
  1.1923262
 
       NA
 
       NA
  
 
  
 11
 
  67775763
 
  67776030
 
  ENSEMBL   UCSC 
 
  268
 
  4
 
 7481
 
 tss
 
 0.75322
 
 0.62119
 
 Not queried
 
  1.1915664
 
       NA
 
       NA
  
 
  
 11
 
   8102311
 
   8102510
 
  ENSEMBL   UCSC 
 
  200
 
  4
 
 6478
 
 gene;tss;island
 
 0.04182
 
 0.05547
 
 Not queried
 
  1.1915571
 
       NA
 
       NA
  
 
  
 3
 
  63263752
 
  63263832
 
  ENSEMBL   UCSC 
 
   81
 
  4
 
 28387
 
 tss;island
 
 0.43646
 
 0.24127
 
 Not queried
 
  1.1914165
 
       NA
 
       NA
  
 
  
 18
 
  31803454
 
  31803836
 
  ENSEMBL   UCSC 
 
  383
 
  6
 
 19195
 
 tss;island
 
 0.08630
 
 0.05374
 
 Not queried
 
  1.1913487
 
       NA
 
       NA
  
 
  
 8
 
  25902284
 
  25902611
 
  ENSEMBL   UCSC 
 
  328
 
  6
 
 40105
 
 island
 
 0.01931
 
 0.02857
 
 Not queried
 
  1.1907489
 
       NA
 
       NA
  
 
  
 X
 
 152711957
 
 152712221
 
  ENSEMBL   UCSC 
 
  265
 
  7
 
 41577
 
 tss
 
 0.70229
 
 0.83495
 
 Not queried
 
  1.1904032
 
       NA
 
       NA
  
 
  
 7
 
   4850075
 
   4850345
 
  ENSEMBL   UCSC 
 
  271
 
  4
 
 36795
 
 gene;island
 
 0.79624
 
 0.89880
 
 Not queried
 
  1.1897294
 
       NA
 
       NA
  
 
  
 6
 
  26204924
 
  26205244
 
  ENSEMBL   UCSC 
 
  321
 
  4
 
 33541
 
 gene
 
 0.05021
 
 0.06963
 
 Not queried
 
  1.1896671
 
       NA
 
       NA
  
 
  
 6
 
 159064992
 
 159065192
 
  ENSEMBL   UCSC 
 
  201
 
  5
 
 34465
 
 gene;island
 
 0.03316
 
 0.01485
 
 Not queried
 
  1.1896082
 
       NA
 
       NA
  
 
  
 14
 
  76452112
 
  76452459
 
  ENSEMBL   UCSC 
 
  348
 
  4
 
 12120
 
 gene
 
 0.00884
 
 0.01908
 
 Not queried
 
  1.1895563
 
       NA
 
       NA
  
 
  
 11
 
 126870233
 
 126870773
 
  ENSEMBL   UCSC 
 
  541
 
  6
 
 8911
 
 island
 
 0.04669
 
 0.10774
 
 Not queried
 
  1.1891008
 
       NA
 
       NA
  
 
  
 3
 
  48646722
 
  48647426
 
  ENSEMBL   UCSC 
 
  705
 
  8
 
 29048
 
 island
 
 0.02892
 
 0.01968
 
 Not queried
 
  1.1890288
 
       NA
 
       NA
  
 
  
 6
 
  29714824
 
  29715162
 
  ENSEMBL   UCSC 
 
  339
 
  5
 
 33628
 
 gene;island
 
 0.77117
 
 0.54030
 
 Not queried
 
  1.1890009
 
       NA
 
       NA
  
 
  
 12
 
  54132086
 
  54132568
 
  ENSEMBL   UCSC 
 
  483
 
  6
 
 10446
 
 island
 
 0.04752
 
 0.11287
 
 Not queried
 
  1.1890002
 
       NA
 
       NA
  
 
  
 17
 
   7153814
 
   7154245
 
  ENSEMBL   UCSC 
 
  432
 
  4
 
 16351
 
 gene;island
 
 0.05105
 
 0.11060
 
 Not queried
 
  1.1882363
 
       NA
 
       NA
  
 
  
 12
 
 131561080
 
 131561312
 
  ENSEMBL   UCSC 
 
  233
 
  4
 
 9397
 
 gene
 
 0.80917
 
 0.60723
 
 Not queried
 
  1.1881550
 
       NA
 
       NA
  
 
  
 3
 
  75834271
 
  75834910
 
  ENSEMBL   UCSC 
 
  640
 
 10
 
 28404
 
 tss
 
 0.65212
 
 0.49818
 
 Not queried
 
  1.1881504
 
       NA
 
       NA
  
 
  
 12
 
  56324837
 
  56325149
 
  ENSEMBL   UCSC 
 
  313
 
  4
 
 9749
 
 tss;island
 
 0.27688
 
 0.14692
 
 Not queried
 
  1.1880932
 
       NA
 
       NA
  
 
  
 14
 
 100658902
 
 100659150
 
  ENSEMBL   UCSC 
 
  249
 
  4
 
 13106
 
 island
 
 0.13556
 
 0.29867
 
 Not queried
 
  1.1880845
 
       NA
 
       NA
  
 
  
 10
 
  11206772
 
  11207015
 
  ENSEMBL   UCSC 
 
  244
 
  8
 
 4480
 
 gene;island
 
 0.21011
 
 0.06828
 
 Not queried
 
  1.1879867
 
       NA
 
       NA
  
 
  
 19
 
  10792615
 
  10793259
 
  ENSEMBL   UCSC 
 
  645
 
  7
 
 19784
 
 gene
 
 0.77773
 
 0.88667
 
 Not queried
 
  1.1878890
 
       NA
 
       NA
  
 
  
 2
 
  72376056
 
  72376239
 
  ENSEMBL   UCSC 
 
  184
 
  4
 
 23394
 
 tss;island
 
 0.03808
 
 0.05654
 
 Not queried
 
  1.1875460
 
       NA
 
       NA
  
 
  
 7
 
  29605624
 
  29606082
 
  ENSEMBL   UCSC 
 
  459
 
  4
 
 36887
 
 gene;island
 
 0.86849
 
 0.74298
 
 Not queried
 
  1.1874314
 
       NA
 
       NA
  
 
  
 21
 
  40759534
 
  40759686
 
  ENSEMBL   UCSC 
 
  153
 
  4
 
 26509
 
 tss
 
 0.04769
 
 0.03030
 
 Not queried
 
  1.1871170
 
       NA
 
       NA
  
 
  
 3
 
  48342648
 
  48343235
 
  ENSEMBL   UCSC 
 
  588
 
 10
 
 29042
 
 island
 
 0.04511
 
 0.02099
 
 Not queried
 
  1.1870259
 
       NA
 
       NA
  
 
  
 20
 
  61151391
 
  61151480
 
  ENSEMBL   UCSC 
 
   90
 
  6
 
 25451
 
 gene;tss;island
 
 0.68232
 
 0.83213
 
 Not queried
 
  1.1868620
 
       NA
 
       NA
  
 
  
 17
 
  32907636
 
  32907731
 
  ENSEMBL   UCSC 
 
   96
 
  4
 
 17228
 
 tss
 
 0.04390
 
 0.10591
 
 Not queried
 
  1.1866219
 
       NA
 
       NA
  
 
  
 11
 
  77850393
 
  77850650
 
  ENSEMBL   UCSC 
 
  258
 
  4
 
 6825
 
 gene
 
 0.00729
 
 0.00685
 
 Not queried
 
  1.1863760
 
       NA
 
       NA
  
 
  
 12
 
 133000028
 
 133000497
 
  ENSEMBL   UCSC 
 
  470
 
  6
 
 11024
 
 island
 
 0.54293
 
 0.75794
 
 Not queried
 
  1.1863287
 
       NA
 
       NA
  
 
  
 4
 
  52904500
 
  52905025
 
  ENSEMBL   UCSC 
 
  526
 
  9
 
 30342
 
 tss
 
 0.05518
 
 0.01861
 
 Not queried
 
  1.1860220
 
       NA
 
       NA
  
 
  
 14
 
  75389066
 
  75389280
 
  ENSEMBL   UCSC 
 
  215
 
  4
 
 12980
 
 island
 
 0.83932
 
 0.70645
 
 Not queried
 
  1.1857589
 
       NA
 
       NA
  
 
  
 4
 
   5570187
 
   5570306
 
  ENSEMBL   UCSC 
 
  120
 
  4
 
 29875
 
 gene;island
 
 0.89346
 
 0.72357
 
 Not queried
 
  1.1854675
 
       NA
 
       NA
  
 
  
 17
 
  34947837
 
  34948150
 
  ENSEMBL   UCSC 
 
  314
 
  7
 
 17248
 
 tss
 
 0.03638
 
 0.06253
 
 Not queried
 
  1.1847960
 
       NA
 
       NA
  
 
  
 6
 
 168389700
 
 168390347
 
  ENSEMBL   UCSC 
 
  648
 
  6
 
 36590
 
 island
 
 0.93223
 
 0.87530
 
 Not queried
 
  1.1845804
 
       NA
 
       NA
  
 
  
 11
 
  63933484
 
  63933778
 
  ENSEMBL   UCSC 
 
  295
 
  4
 
 8363
 
 island
 
 0.02833
 
 0.06346
 
 Not queried
 
  1.1845291
 
       NA
 
       NA
  
 
  
 2
 
 177052486
 
 177052903
 
  ENSEMBL   UCSC 
 
  418
 
  5
 
 23705
 
 tss;island
 
 0.08390
 
 0.17228
 
 Not queried
 
  1.1844445
 
       NA
 
       NA
  
 
  
 17
 
  56405948
 
  56406294
 
  ENSEMBL   UCSC 
 
  347
 
  4
 
 18564
 
 island
 
 0.51045
 
 0.67211
 
 Not queried
 
  1.1844047
 
       NA
 
       NA
  
 
  
 4
 
 186317760
 
 186317907
 
  ENSEMBL   UCSC 
 
  148
 
  4
 
 31420
 
 island
 
 0.01588
 
 0.03115
 
 Not queried
 
  1.1843010
 
       NA
 
       NA
  
 
  
 20
 
  62715661
 
  62715942
 
  ENSEMBL   UCSC 
 
  282
 
  6
 
 25480
 
 gene;island
 
 0.59569
 
 0.39360
 
 Not queried
 
  1.1841085
 
       NA
 
       NA
  
 
  
 11
 
  67203360
 
  67203661
 
  ENSEMBL   UCSC 
 
  302
 
  4
 
 6737
 
 gene;island
 
 0.66219
 
 0.81529
 
 Not queried
 
  1.1840998
 
       NA
 
       NA
  
 
  
 17
 
  46871582
 
  46871905
 
  ENSEMBL   UCSC 
 
  324
 
  6
 
 16632
 
 gene
 
 0.75279
 
 0.54867
 
 Not queried
 
  1.1840252
 
       NA
 
       NA
  
 
  
 12
 
 114843188
 
 114843955
 
  ENSEMBL   UCSC 
 
  768
 
 10
 
 9300
 
 gene;island
 
 0.06361
 
 0.15347
 
 Not queried
 
  1.1837288
 
       NA
 
       NA
  
 
  
 17
 
  38278395
 
  38278610
 
  ENSEMBL   UCSC 
 
  216
 
  4
 
 17291
 
 tss;island
 
 0.03937
 
 0.12473
 
 Not queried
 
  1.1836370
 
       NA
 
       NA
  
 
  
 16
 
   2273123
 
   2273560
 
  ENSEMBL   UCSC 
 
  438
 
  9
 
 14993
 
 tss
 
 0.02207
 
 0.02088
 
 Not queried
 
  1.1833421
 
       NA
 
       NA
  
 
  
 16
 
   2273123
 
   2273898
 
  ENSEMBL   UCSC 
 
  776
 
 11
 
 15565
 
 island
 
 0.02207
 
 0.01383
 
 Not queried
 
  1.1833421
 
       NA
 
       NA
  
 
  
 8
 
 124253706
 
 124253829
 
  ENSEMBL   UCSC 
 
  124
 
  4
 
 39833
 
 tss
 
 0.05850
 
 0.07003
 
 Not queried
 
  1.1829869
 
       NA
 
       NA
  
 
  
 18
 
  77748679
 
  77748761
 
  ENSEMBL   UCSC 
 
   83
 
  4
 
 19305
 
 tss
 
 0.01604
 
 0.04099
 
 Not queried
 
  1.1826963
 
       NA
 
       NA
  
 
  
 6
 
  31804078
 
  31804883
 
  ENSEMBL   UCSC 
 
  806
 
 10
 
 33908
 
 gene;island
 
 0.17081
 
 0.04254
 
 Not queried
 
  1.1826945
 
       NA
 
       NA
  
 
  
 12
 
 121124611
 
 121124913
 
  ENSEMBL   UCSC 
 
  303
 
  6
 
 10040
 
 tss
 
 0.01449
 
 0.02418
 
 Not queried
 
  1.1824697
 
       NA
 
       NA
  
 
  
 12
 
 121124611
 
 121125137
 
  ENSEMBL   UCSC 
 
  527
 
  8
 
 10869
 
 island
 
 0.01486
 
 0.02418
 
 Not queried
 
  1.1824697
 
       NA
 
       NA
  
 
  
 2
 
  20865651
 
  20865847
 
  ENSEMBL   UCSC 
 
  197
 
  4
 
 23176
 
 tss;island
 
 0.17421
 
 0.08970
 
 Not queried
 
  1.1819530
 
       NA
 
       NA
  
 
  
 X
 
 130207219
 
 130207604
 
  ENSEMBL   UCSC 
 
  386
 
  4
 
 41106
 
 gene;island
 
 0.42286
 
 0.29920
 
 Not queried
 
  1.1816603
 
       NA
 
       NA
  
 
  
 6
 
  26501812
 
  26502192
 
  ENSEMBL   UCSC 
 
  381
 
  5
 
 33549
 
 gene;island
 
 0.05579
 
 0.12555
 
 Not queried
 
  1.1814599
 
       NA
 
       NA
  
 
  
 20
 
    327607
 
    327811
 
  ENSEMBL   UCSC 
 
  205
 
  4
 
 25283
 
 gene;island
 
 0.01288
 
 0.02924
 
 Not queried
 
  1.1814584
 
       NA
 
       NA
  
 
  
 X
 
  38080134
 
  38080608
 
  ENSEMBL   UCSC 
 
  475
 
  8
 
 41718
 
 island
 
 0.03098
 
 0.04044
 
 Not queried
 
  1.1813763
 
       NA
 
       NA
  
 
  
 2
 
  45838293
 
  45838581
 
  ENSEMBL   UCSC 
 
  289
 
  7
 
 24233
 
 island
 
 0.03141
 
 0.06351
 
 Not queried
 
  1.1812674
 
       NA
 
       NA
  
 
  
 20
 
  35274570
 
  35274941
 
  ENSEMBL   UCSC 
 
  372
 
  8
 
 25678
 
 tss
 
 0.69486
 
 0.80673
 
 Not queried
 
  1.1810917
 
       NA
 
       NA
  
 
  
 22
 
  45596948
 
  45597035
 
  ENSEMBL   UCSC 
 
   88
 
  7
 
 27176
 
 tss
 
 0.96043
 
 0.89842
 
 Not queried
 
  1.1810903
 
       NA
 
       NA
  
 
  
 20
 
   2781122
 
   2781685
 
  ENSEMBL   UCSC 
 
  564
 
 11
 
 25914
 
 island
 
 0.04527
 
 0.02939
 
 Not queried
 
  1.1810452
 
       NA
 
       NA
  
 
  
 6
 
  31602599
 
  31603535
 
  ENSEMBL   UCSC 
 
  937
 
 11
 
 33858
 
 gene;island
 
 0.70817
 
 0.86624
 
 Not queried
 
  1.1807593
 
       NA
 
       NA
  
 
  
 4
 
 174255082
 
 174255105
 
  ENSEMBL   UCSC 
 
   24
 
  4
 
 30131
 
 gene;tss;island
 
 0.02858
 
 0.06201
 
 Not queried
 
  1.1807492
 
       NA
 
       NA
  
 
  
 6
 
  32370520
 
  32370879
 
  ENSEMBL   UCSC 
 
  360
 
  7
 
 34046
 
 gene
 
 0.30106
 
 0.55652
 
 Not queried
 
  1.1807193
 
       NA
 
       NA
  
 
  
 6
 
 167051518
 
 167051781
 
  ENSEMBL   UCSC 
 
  264
 
  5
 
 34487
 
 gene
 
 0.88146
 
 0.89857
 
 Not queried
 
  1.1805258
 
       NA
 
       NA
  
 
  
 1
 
 115880865
 
 115881259
 
  ENSEMBL   UCSC 
 
  395
 
  7
 
 1854
 
 tss;island
 
 0.12604
 
 0.03981
 
 Not queried
 
  1.1803441
 
       NA
 
       NA
  
 
  
 5
 
  77268331
 
  77268888
 
  ENSEMBL   UCSC 
 
  558
 
  5
 
 32844
 
 island
 
 0.02799
 
 0.04400
 
 Not queried
 
  1.1801148
 
       NA
 
       NA
  
 
  
 10
 
 134996789
 
 134997074
 
  ENSEMBL   UCSC 
 
  286
 
  5
 
 4874
 
 gene;island
 
 0.88605
 
 0.83991
 
 Not queried
 
  1.1800417
 
       NA
 
       NA
  
 
  
 11
 
    843897
 
    844400
 
  ENSEMBL   UCSC 
 
  504
 
  8
 
 7013
 
 tss
 
 0.10692
 
 0.03391
 
 Not queried
 
  1.1800129
 
       NA
 
       NA
  
 
  
 2
 
 230135577
 
 230135929
 
  ENSEMBL   UCSC 
 
  353
 
  5
 
 23022
 
 gene;island
 
 0.15541
 
 0.07513
 
 Not queried
 
  1.1796465
 
       NA
 
       NA
  
 
  
 4
 
 146403866
 
 146403948
 
  ENSEMBL   UCSC 
 
   83
 
  5
 
 30572
 
 tss
 
 0.01128
 
 0.02601
 
 Not queried
 
  1.1794532
 
       NA
 
       NA
  
 
  
 2
 
 208989324
 
 208989469
 
  ENSEMBL   UCSC 
 
  146
 
  4
 
 23807
 
 tss
 
 0.35643
 
 0.59643
 
 Not queried
 
  1.1794517
 
       NA
 
       NA
  
 
  
 2
 
 208989209
 
 208989469
 
  ENSEMBL   UCSC 
 
  261
 
  6
 
 25008
 
 island
 
 0.41609
 
 0.60039
 
 Not queried
 
  1.1794517
 
       NA
 
       NA
  
 
  
 7
 
  18067508
 
  18067537
 
  ENSEMBL   UCSC 
 
   30
 
  5
 
 37392
 
 tss
 
 0.95826
 
 0.93575
 
 Not queried
 
  1.1791689
 
       NA
 
       NA
  
 
  
 10
 
    180465
 
    180755
 
  ENSEMBL   UCSC 
 
  291
 
  5
 
 4417
 
 gene
 
 0.00588
 
 0.01370
 
 Not queried
 
  1.1790089
 
       NA
 
       NA
  
 
  
 X
 
  11777654
 
  11777744
 
  ENSEMBL   UCSC 
 
   91
 
  4
 
 41186
 
 tss
 
 0.02022
 
 0.04613
 
 Not queried
 
  1.1789772
 
       NA
 
       NA
  
 
  
 X
 
  48457128
 
  48457337
 
  ENSEMBL   UCSC 
 
  210
 
  4
 
 40985
 
 gene;island
 
 0.87603
 
 0.94674
 
 Not queried
 
  1.1787540
 
       NA
 
       NA
  
 
  
 1
 
 225616198
 
 225616668
 
  ENSEMBL   UCSC 
 
  471
 
  4
 
 2275
 
 tss;island
 
 0.01631
 
 0.02576
 
 Not queried
 
  1.1787098
 
       NA
 
       NA
  
 
  
 11
 
 120038521
 
 120039029
 
  ENSEMBL   UCSC 
 
  509
 
  5
 
 8865
 
 island
 
 0.90586
 
 0.86684
 
 Not queried
 
  1.1786484
 
       NA
 
       NA
  
 
  
 2
 
 236715747
 
 236716372
 
  ENSEMBL   UCSC 
 
  626
 
  6
 
 23044
 
 gene
 
 0.91967
 
 0.72637
 
 Not queried
 
  1.1786222
 
       NA
 
       NA
  
 
  
 2
 
 168572517
 
 168572814
 
  ENSEMBL   UCSC 
 
  298
 
  4
 
 24796
 
 island
 
 0.35320
 
 0.71305
 
 Not queried
 
  1.1786008
 
       NA
 
       NA
  
 
  
 8
 
  32406613
 
  32406928
 
  ENSEMBL   UCSC 
 
  316
 
  4
 
 39211
 
 gene;island
 
 0.02298
 
 0.02267
 
 Not queried
 
  1.1785892
 
       NA
 
       NA
  
 
  
 6
 
 159290283
 
 159290589
 
  ENSEMBL   UCSC 
 
  307
 
  4
 
 36538
 
 island
 
 0.73906
 
 0.87459
 
 Not queried
 
  1.1784520
 
       NA
 
       NA
  
 
  
 16
 
    595486
 
    596185
 
  ENSEMBL   UCSC 
 
  700
 
  8
 
 14504
 
 gene;island
 
 0.77715
 
 0.83422
 
 Not queried
 
  1.1784446
 
       NA
 
       NA
  
 
  
 19
 
    281291
 
    281467
 
  ENSEMBL   UCSC 
 
  177
 
  4
 
 19586
 
 gene
 
 0.73251
 
 0.57065
 
 Not queried
 
  1.1784357
 
       NA
 
       NA
  
 
  
 16
 
  34407715
 
  34408014
 
  ENSEMBL   UCSC 
 
  300
 
  5
 
 15860
 
 island
 
 0.65505
 
 0.65538
 
 Not queried
 
  1.1783871
 
       NA
 
       NA
  
 
  
 22
 
  38071455
 
  38071607
 
  ENSEMBL   UCSC 
 
  153
 
  5
 
 27079
 
 tss
 
 0.07708
 
 0.15442
 
 Not queried
 
  1.1782536
 
       NA
 
       NA
  
 
  
 22
 
  38071455
 
  38071677
 
  ENSEMBL   UCSC 
 
  223
 
  7
 
 27426
 
 island
 
 0.14225
 
 0.21068
 
 Not queried
 
  1.1782536
 
       NA
 
       NA
  
 
  
 11
 
   1968266
 
   1968453
 
  ENSEMBL   UCSC 
 
  188
 
  5
 
 7031
 
 tss
 
 0.04255
 
 0.09959
 
 Not queried
 
  1.1782098
 
       NA
 
       NA
  
 
  
 6
 
  29590966
 
  29591155
 
  ENSEMBL   UCSC 
 
  190
 
  5
 
 33613
 
 gene
 
 0.82162
 
 0.89528
 
 Not queried
 
  1.1778679
 
       NA
 
       NA
  
 
  
 10
 
  88717364
 
  88717623
 
  ENSEMBL   UCSC 
 
  260
 
  4
 
 5183
 
 tss
 
 0.65794
 
 0.82731
 
 Not queried
 
  1.1777818
 
       NA
 
       NA
  
 
  
 15
 
  91497863
 
  91498497
 
  ENSEMBL   UCSC 
 
  635
 
 10
 
 14436
 
 island
 
 0.02176
 
 0.03877
 
 Not queried
 
  1.1776654
 
       NA
 
       NA
  
 
  
 10
 
  73565440
 
  73565966
 
  ENSEMBL   UCSC 
 
  527
 
  5
 
 4611
 
 gene;island
 
 0.04071
 
 0.44636
 
 Not queried
 
  1.1776599
 
       NA
 
       NA
  
 
  
 13
 
  31736119
 
  31736727
 
  ENSEMBL   UCSC 
 
  609
 
 11
 
 11350
 
 tss
 
 0.01519
 
 0.02560
 
 Not queried
 
  1.1776222
 
       NA
 
       NA
  
 
  
 1
 
  24861604
 
  24861919
 
  ENSEMBL   UCSC 
 
  316
 
  5
 
 327
 
 gene;island
 
 0.93119
 
 0.88935
 
 Not queried
 
  1.1776181
 
       NA
 
       NA
  
 
  
 13
 
  31736105
 
  31736727
 
  ENSEMBL   UCSC 
 
  623
 
 12
 
 11623
 
 island
 
 0.02285
 
 0.02716
 
 Not queried
 
  1.1774401
 
       NA
 
       NA
  
 
  
 6
 
  31129244
 
  31129883
 
  ENSEMBL   UCSC 
 
  640
 
 14
 
 33798
 
 gene;island
 
 0.84600
 
 0.92567
 
 Not queried
 
  1.1773430
 
       NA
 
       NA
  
 
  
 8
 
   1992965
 
   1993125
 
  ENSEMBL   UCSC 
 
  161
 
  4
 
 39474
 
 tss
 
 0.31692
 
 0.66330
 
 Not queried
 
  1.1768145
 
       NA
 
       NA
  
 
  
 16
 
   2563274
 
   2563560
 
  ENSEMBL   UCSC 
 
  287
 
  4
 
 14997
 
 tss;island
 
 0.10334
 
 0.05456
 
 Not queried
 
  1.1767271
 
       NA
 
       NA
  
 
  
 6
 
 166400871
 
 166401051
 
  ENSEMBL   UCSC 
 
  181
 
  5
 
 34476
 
 gene;island
 
 0.05925
 
 0.04479
 
 Not queried
 
  1.1763437
 
       NA
 
       NA
  
 
  
 18
 
  32557882
 
  32558112
 
  ENSEMBL   UCSC 
 
  231
 
  4
 
 19054
 
 gene;tss;island
 
 0.01454
 
 0.04125
 
 Not queried
 
  1.1762594
 
       NA
 
       NA
  
 
  
 6
 
 137817512
 
 137817846
 
  ENSEMBL   UCSC 
 
  335
 
  4
 
 36447
 
 island
 
 0.03629
 
 0.01910
 
 Not queried
 
  1.1761360
 
       NA
 
       NA
  
 
  
 15
 
  35838514
 
  35838733
 
  ENSEMBL   UCSC 
 
  220
 
  6
 
 13537
 
 tss;island
 
 0.01498
 
 0.04127
 
 Not queried
 
  1.1761358
 
       NA
 
       NA
  
 
  
 6
 
  30655870
 
  30656094
 
  ENSEMBL   UCSC 
 
  225
 
  5
 
 33744
 
 gene
 
 0.48465
 
 0.60031
 
 Not queried
 
  1.1760331
 
       NA
 
       NA
  
 
  
 5
 
 134240760
 
 134240791
 
  ENSEMBL   UCSC 
 
   32
 
  6
 
 32241
 
 tss;island
 
 0.01254
 
 0.03546
 
 Not queried
 
  1.1758200
 
       NA
 
       NA
  
 
  
 19
 
  51925128
 
  51925472
 
  ENSEMBL   UCSC 
 
  345
 
  4
 
 22306
 
 island
 
 0.05940
 
 0.11145
 
 Not queried
 
  1.1756770
 
       NA
 
       NA
  
 
  
 2
 
 242051650
 
 242051808
 
  ENSEMBL   UCSC 
 
  159
 
  4
 
 23092
 
 gene
 
 0.91573
 
 0.97173
 
 Not queried
 
  1.1756575
 
       NA
 
       NA
  
 
  
 11
 
  66838934
 
  66839363
 
  ENSEMBL   UCSC 
 
  430
 
  5
 
 6725
 
 gene;island
 
 0.40423
 
 0.19866
 
 Not queried
 
  1.1753695
 
       NA
 
       NA
  
 
  
 3
 
  55518091
 
  55518142
 
  ENSEMBL   UCSC 
 
   52
 
  4
 
 27797
 
 gene;island
 
 0.85896
 
 0.94677
 
 Not queried
 
  1.1753342
 
       NA
 
       NA
  
 
  
 21
 
  46850449
 
  46850840
 
  ENSEMBL   UCSC 
 
  392
 
  5
 
 26426
 
 gene;island
 
 0.60757
 
 0.79879
 
 Not queried
 
  1.1751045
 
       NA
 
       NA
  
 
  
 6
 
  32916946
 
  32917655
 
  ENSEMBL   UCSC 
 
  710
 
 10
 
 34099
 
 gene
 
 0.62977
 
 0.42130
 
 Not queried
 
  1.1749766
 
       NA
 
       NA
  
 
  
 8
 
  29210580
 
  29210800
 
  ENSEMBL   UCSC 
 
  221
 
  4
 
 40137
 
 island
 
 0.12123
 
 0.09138
 
 Not queried
 
  1.1747016
 
       NA
 
       NA
  
 
  
 3
 
 182817190
 
 182817338
 
  ENSEMBL   UCSC 
 
  149
 
  4
 
 28005
 
 gene
 
 0.01763
 
 0.00745
 
 Not queried
 
  1.1745336
 
       NA
 
       NA
  
 
  
 20
 
  30458612
 
  30458745
 
  ENSEMBL   UCSC 
 
  134
 
  5
 
 25347
 
 gene;tss;island
 
 0.30267
 
 0.20687
 
 Not queried
 
  1.1742930
 
       NA
 
       NA
  
 
  
 17
 
  47653018
 
  47653309
 
  ENSEMBL   UCSC 
 
  292
 
  5
 
 18494
 
 island
 
 0.01213
 
 0.02879
 
 Not queried
 
  1.1734367
 
       NA
 
       NA
  
 
  
 7
 
  32982914
 
  32983012
 
  ENSEMBL   UCSC 
 
   99
 
  4
 
 37463
 
 tss;island
 
 0.33451
 
 0.09681
 
 Not queried
 
  1.1733507
 
       NA
 
       NA
  
 
  
 8
 
 144328715
 
 144329027
 
  ENSEMBL   UCSC 
 
  313
 
  7
 
 39881
 
 tss
 
 0.01869
 
 0.04326
 
 Not queried
 
  1.1732774
 
       NA
 
       NA
  
 
  
 5
 
 132155233
 
 132155555
 
  ENSEMBL   UCSC 
 
  323
 
  5
 
 33039
 
 island
 
 0.57279
 
 0.38121
 
 Not queried
 
  1.1731120
 
       NA
 
       NA
  
 
  
 1
 
 111743020
 
 111743271
 
  ENSEMBL   UCSC 
 
  252
 
  4
 
 680
 
 gene
 
 0.94547
 
 0.86817
 
 Not queried
 
  1.1728013
 
       NA
 
       NA
  
 
  
 3
 
 184032711
 
 184033015
 
  ENSEMBL   UCSC 
 
  305
 
  4
 
 28018
 
 gene;island
 
 0.01367
 
 0.02182
 
 Not queried
 
  1.1724611
 
       NA
 
       NA
  
 
  
 11
 
  43963820
 
  43964098
 
  ENSEMBL   UCSC 
 
  279
 
  6
 
 7237
 
 tss
 
 0.03543
 
 0.01895
 
 Not queried
 
  1.1722082
 
       NA
 
       NA
  
 
  
 8
 
 145180403
 
 145180933
 
  ENSEMBL   UCSC 
 
  531
 
  5
 
 40652
 
 island
 
 0.67147
 
 0.84454
 
 Not queried
 
  1.1722058
 
       NA
 
       NA
  
 
  
 4
 
   2366485
 
   2366831
 
  ENSEMBL   UCSC 
 
  347
 
  4
 
 29844
 
 gene;island
 
 0.74955
 
 0.81913
 
 Not queried
 
  1.1721628
 
       NA
 
       NA
  
 
  
 19
 
  53832311
 
  53832670
 
  ENSEMBL   UCSC 
 
  360
 
  5
 
 22339
 
 island
 
 0.81492
 
 0.87689
 
 Not queried
 
  1.1720632
 
       NA
 
       NA
  
 
  
 17
 
   4981403
 
   4981610
 
  ENSEMBL   UCSC 
 
  208
 
  5
 
 17007
 
 tss;island
 
 0.97294
 
 0.90618
 
 Not queried
 
  1.1719588
 
       NA
 
       NA
  
 
  
 17
 
   5019310
 
   5019989
 
  ENSEMBL   UCSC 
 
  680
 
  7
 
 16328
 
 gene;island
 
 0.94090
 
 0.87981
 
 Not queried
 
  1.1716845
 
       NA
 
       NA
  
 
  
 8
 
  12869510
 
  12869722
 
  ENSEMBL   UCSC 
 
  213
 
  5
 
 39147
 
 gene;tss
 
 0.94479
 
 0.89966
 
 Not queried
 
  1.1715283
 
       NA
 
       NA
  
 
  
 7
 
 157260817
 
 157261163
 
  ENSEMBL   UCSC 
 
  347
 
  4
 
 39041
 
 island
 
 0.62008
 
 0.88308
 
 Not queried
 
  1.1709788
 
       NA
 
       NA
  
 
  
 1
 
 231155682
 
 231155983
 
  ENSEMBL   UCSC 
 
  302
 
 10
 
 2315
 
 tss
 
 0.54788
 
 0.76435
 
 Not queried
 
  1.1708887
 
       NA
 
       NA
  
 
  
 5
 
  94620929
 
  94621055
 
  ENSEMBL   UCSC 
 
  127
 
  4
 
 32123
 
 tss;island
 
 0.03611
 
 0.06236
 
 Not queried
 
  1.1707323
 
       NA
 
       NA
  
 
  
 6
 
 134158982
 
 134159412
 
  ENSEMBL   UCSC 
 
  431
 
  5
 
 34413
 
 gene;island
 
 0.01858
 
 0.01438
 
 Not queried
 
  1.1705692
 
       NA
 
       NA
  
 
  
 19
 
  36247867
 
  36248077
 
  ENSEMBL   UCSC 
 
  211
 
  4
 
 20677
 
 tss;island
 
 0.52893
 
 0.71270
 
 Not queried
 
  1.1704448
 
       NA
 
       NA
  
 
  
 6
 
   6320614
 
   6320895
 
  ENSEMBL   UCSC 
 
  282
 
  4
 
 33482
 
 gene
 
 0.10603
 
 0.19813
 
 Not queried
 
  1.1696836
 
       NA
 
       NA
  
 
  
 13
 
 112721712
 
 112721789
 
  ENSEMBL   UCSC 
 
   78
 
  6
 
 11515
 
 tss
 
 0.07151
 
 0.14132
 
 Not queried
 
  1.1694187
 
       NA
 
       NA
  
 
  
 5
 
  57755923
 
  57756026
 
  ENSEMBL   UCSC 
 
  104
 
  4
 
 32017
 
 tss
 
 0.01073
 
 0.01798
 
 Not queried
 
  1.1689717
 
       NA
 
       NA
  
 
  
 19
 
  17958892
 
  17959281
 
  ENSEMBL   UCSC 
 
  390
 
  8
 
 20566
 
 tss
 
 0.78632
 
 0.66157
 
 Not queried
 
  1.1687116
 
       NA
 
       NA
  
 
  
 1
 
  70035080
 
  70035479
 
  ENSEMBL   UCSC 
 
  400
 
  4
 
 3369
 
 island
 
 0.07263
 
 0.15309
 
 Not queried
 
  1.1686026
 
       NA
 
       NA
  
 
  
 2
 
 128990739
 
 128991052
 
  ENSEMBL   UCSC 
 
  314
 
  4
 
 24670
 
 island
 
 0.01852
 
 0.03307
 
 Not queried
 
  1.1685868
 
       NA
 
       NA
  
 
  
 7
 
   2353682
 
   2354484
 
  ENSEMBL   UCSC 
 
  803
 
 12
 
 38089
 
 island
 
 0.06419
 
 0.18702
 
 Not queried
 
  1.1682810
 
       NA
 
       NA
  
 
  
 13
 
  22245333
 
  22245730
 
  ENSEMBL   UCSC 
 
  398
 
  5
 
 11072
 
 gene
 
 0.01449
 
 0.04340
 
 Not queried
 
  1.1678862
 
       NA
 
       NA
  
 
  
 7
 
 127881269
 
 127881440
 
  ENSEMBL   UCSC 
 
  172
 
  5
 
 38828
 
 island
 
 0.97324
 
 0.93124
 
 Not queried
 
  1.1677090
 
       NA
 
       NA
  
 
  
 17
 
  19290503
 
  19290762
 
  ENSEMBL   UCSC 
 
  260
 
  6
 
 17157
 
 tss
 
 0.83038
 
 0.62669
 
 Not queried
 
  1.1676840
 
       NA
 
       NA
  
 
  
 22
 
  19938916
 
  19939028
 
  ENSEMBL   UCSC 
 
  113
 
  4
 
 26928
 
 tss
 
 0.77689
 
 0.88640
 
 Not queried
 
  1.1676544
 
       NA
 
       NA
  
 
  
 12
 
  65218069
 
  65218413
 
  ENSEMBL   UCSC 
 
  345
 
  4
 
 10570
 
 island
 
 0.16659
 
 0.07016
 
 Not queried
 
  1.1676113
 
       NA
 
       NA
  
 
  
 11
 
 125036088
 
 125036420
 
  ENSEMBL   UCSC 
 
  333
 
  4
 
 6937
 
 gene;island
 
 0.10165
 
 0.05127
 
 Not queried
 
  1.1674839
 
       NA
 
       NA
  
 
  
 10
 
  88719848
 
  88720216
 
  ENSEMBL   UCSC 
 
  369
 
  4
 
 4649
 
 gene
 
 0.72428
 
 0.82768
 
 Not queried
 
  1.1674779
 
       NA
 
       NA
  
 
  
 6
 
  97285662
 
  97285948
 
  ENSEMBL   UCSC 
 
  287
 
  5
 
 35064
 
 tss;island
 
 0.05913
 
 0.02759
 
 Not queried
 
  1.1673328
 
       NA
 
       NA
  
 
  
 18
 
  24445134
 
  24445318
 
  ENSEMBL   UCSC 
 
  185
 
  4
 
 19049
 
 gene;island
 
 0.59086
 
 0.77065
 
 Not queried
 
  1.1671526
 
       NA
 
       NA
  
 
  
 2
 
 182850059
 
 182850441
 
  ENSEMBL   UCSC 
 
  383
 
  4
 
 23726
 
 tss
 
 0.60738
 
 0.85839
 
 Not queried
 
  1.1668529
 
       NA
 
       NA
  
 
  
 12
 
 133016214
 
 133016787
 
  ENSEMBL   UCSC 
 
  574
 
  6
 
 11027
 
 island
 
 0.74261
 
 0.89869
 
 Not queried
 
  1.1668153
 
       NA
 
       NA
  
 
  
 19
 
  55173482
 
  55173527
 
  ENSEMBL   UCSC 
 
   46
 
  4
 
 21028
 
 tss
 
 0.47417
 
 0.67941
 
 Not queried
 
  1.1667079
 
       NA
 
       NA
  
 
  
 17
 
   7218821
 
   7219025
 
  ENSEMBL   UCSC 
 
  205
 
  6
 
 17041
 
 tss
 
 0.04298
 
 0.12713
 
 Not queried
 
  1.1657305
 
       NA
 
       NA
  
 
  
 17
 
   7218470
 
   7219025
 
  ENSEMBL   UCSC 
 
  556
 
  8
 
 17903
 
 island
 
 0.04298
 
 0.11488
 
 Not queried
 
  1.1657305
 
       NA
 
       NA
  
 
  
 X
 
 102470047
 
 102470404
 
  ENSEMBL   UCSC 
 
  358
 
  5
 
 41058
 
 gene
 
 0.01555
 
 0.02246
 
 Not queried
 
  1.1654570
 
       NA
 
       NA
  
 
  
 19
 
  38042009
 
  38042154
 
  ENSEMBL   UCSC 
 
  146
 
  5
 
 20705
 
 tss;island
 
 0.65874
 
 0.53649
 
 Not queried
 
  1.1654389
 
       NA
 
       NA
  
 
  
 10
 
 134095646
 
 134096014
 
  ENSEMBL   UCSC 
 
  369
 
  5
 
 4843
 
 gene;island
 
 0.49302
 
 0.67230
 
 Not queried
 
  1.1652049
 
       NA
 
       NA
  
 
  
 22
 
  21336337
 
  21336528
 
  ENSEMBL   UCSC 
 
  192
 
  5
 
 26945
 
 tss
 
 0.03106
 
 0.06490
 
 Not queried
 
  1.1651050
 
       NA
 
       NA
  
 
  
 22
 
  21336337
 
  21336741
 
  ENSEMBL   UCSC 
 
  405
 
  7
 
 27283
 
 island
 
 0.01964
 
 0.06490
 
 Not queried
 
  1.1651050
 
       NA
 
       NA
  
 
  
 11
 
  46957758
 
  46958188
 
  ENSEMBL   UCSC 
 
  431
 
  8
 
 7262
 
 tss
 
 0.02906
 
 0.04876
 
 Not queried
 
  1.1650582
 
       NA
 
       NA
  
 
  
 11
 
    926062
 
    926250
 
  ENSEMBL   UCSC 
 
  189
 
  4
 
 6379
 
 gene;island
 
 0.01833
 
 0.04016
 
 Not queried
 
  1.1650414
 
       NA
 
       NA
  
 
  
 6
 
  31631638
 
  31631801
 
  ENSEMBL   UCSC 
 
  164
 
  7
 
 33868
 
 gene;island
 
 0.72478
 
 0.83004
 
 Not queried
 
  1.1645969
 
       NA
 
       NA
  
 
  
 1
 
  12226913
 
  12226961
 
  ENSEMBL   UCSC 
 
   49
 
  4
 
 1255
 
 tss
 
 0.03777
 
 0.02897
 
 Not queried
 
  1.1645419
 
       NA
 
       NA
  
 
  
 3
 
  11888357
 
  11888606
 
  ENSEMBL   UCSC 
 
  250
 
  7
 
 28114
 
 tss
 
 0.01083
 
 0.02033
 
 Not queried
 
  1.1643881
 
       NA
 
       NA
  
 
  
 16
 
  70323412
 
  70323915
 
  ENSEMBL   UCSC 
 
  504
 
  9
 
 15308
 
 tss
 
 0.01292
 
 0.02089
 
 Not queried
 
  1.1640120
 
       NA
 
       NA
  
 
  
 6
 
  29855401
 
  29855636
 
  ENSEMBL   UCSC 
 
  236
 
  9
 
 33636
 
 gene
 
 0.65590
 
 0.39964
 
 Not queried
 
  1.1639120
 
       NA
 
       NA
  
 
  
 2
 
 242805838
 
 242806119
 
  ENSEMBL   UCSC 
 
  282
 
  5
 
 25273
 
 island
 
 0.91739
 
 0.83213
 
 Not queried
 
  1.1637427
 
       NA
 
       NA
  
 
  
 1
 
  19578443
 
  19578805
 
  ENSEMBL   UCSC 
 
  363
 
  5
 
 279
 
 gene;tss;island
 
 0.00847
 
 0.01962
 
 Not queried
 
  1.1637285
 
       NA
 
       NA
  
 
  
 3
 
  75834108
 
  75834910
 
  ENSEMBL   UCSC 
 
  803
 
 11
 
 29247
 
 island
 
 0.67132
 
 0.50513
 
 Not queried
 
  1.1636385
 
       NA
 
       NA
  
 
  
 4
 
  52904379
 
  52905025
 
  ENSEMBL   UCSC 
 
  647
 
 10
 
 30987
 
 island
 
 0.04991
 
 0.02220
 
 Not queried
 
  1.1635746
 
       NA
 
       NA
  
 
  
 4
 
   8087178
 
   8087448
 
  ENSEMBL   UCSC 
 
  271
 
  4
 
 29901
 
 gene;island
 
 0.72672
 
 0.88220
 
 Not queried
 
  1.1633130
 
       NA
 
       NA
  
 
  
 X
 
 124336658
 
 124337026
 
  ENSEMBL   UCSC 
 
  369
 
  5
 
 42009
 
 island
 
 0.01930
 
 0.05753
 
 Not queried
 
  1.1627303
 
       NA
 
       NA
  
 
  
 11
 
 110583882
 
 110584082
 
  ENSEMBL   UCSC 
 
  201
 
  4
 
 7658
 
 tss;island
 
 0.24109
 
 0.32817
 
 Not queried
 
  1.1627272
 
       NA
 
       NA
  
 
  
 2
 
  89064631
 
  89064965
 
  ENSEMBL   UCSC 
 
  335
 
  4
 
 23465
 
 tss;island
 
 0.68779
 
 0.50746
 
 Not queried
 
  1.1626189
 
       NA
 
       NA
  
 
  
 14
 
 105830509
 
 105830859
 
  ENSEMBL   UCSC 
 
  351
 
  5
 
 12234
 
 gene;island
 
 0.01603
 
 0.05754
 
 Not queried
 
  1.1624499
 
       NA
 
       NA
  
 
  
 15
 
  25318197
 
  25318384
 
  ENSEMBL   UCSC 
 
  188
 
  4
 
 13494
 
 tss
 
 0.89414
 
 0.95007
 
 Not queried
 
  1.1622911
 
       NA
 
       NA
  
 
  
 19
 
   9896597
 
   9896993
 
  ENSEMBL   UCSC 
 
  397
 
  4
 
 21470
 
 island
 
 0.13152
 
 0.06837
 
 Not queried
 
  1.1622805
 
       NA
 
       NA
  
 
  
 7
 
 157216599
 
 157216893
 
  ENSEMBL   UCSC 
 
  295
 
  4
 
 39040
 
 island
 
 0.77958
 
 0.89197
 
 Not queried
 
  1.1622554
 
       NA
 
       NA
  
 
  
 6
 
  33133329
 
  33133675
 
  ENSEMBL   UCSC 
 
  347
 
  8
 
 35901
 
 island
 
 0.34990
 
 0.54927
 
 Not queried
 
  1.1621575
 
       NA
 
       NA
  
 
  
 12
 
 121154526
 
 121154867
 
  ENSEMBL   UCSC 
 
  342
 
  4
 
 9331
 
 gene
 
 0.70880
 
 0.81630
 
 Not queried
 
  1.1620797
 
       NA
 
       NA
  
 
  
 3
 
 122514443
 
 122514814
 
  ENSEMBL   UCSC 
 
  372
 
  4
 
 27875
 
 gene;island
 
 0.10677
 
 0.04416
 
 Not queried
 
  1.1620288
 
       NA
 
       NA
  
 
  
 13
 
 113301636
 
 113301782
 
  ENSEMBL   UCSC 
 
  147
 
  4
 
 11219
 
 gene;island
 
 0.77844
 
 0.84930
 
 Not queried
 
  1.1620155
 
       NA
 
       NA
  
 
  
 11
 
  71149985
 
  71150315
 
  ENSEMBL   UCSC 
 
  331
 
  5
 
 6783
 
 gene;island
 
 0.85686
 
 0.93052
 
 Not queried
 
  1.1617899
 
       NA
 
       NA
  
 
  
 8
 
 110656096
 
 110656442
 
  ENSEMBL   UCSC 
 
  347
 
  5
 
 39336
 
 gene;tss;island
 
 0.06603
 
 0.23246
 
 Not queried
 
  1.1617164
 
       NA
 
       NA
  
 
  
 6
 
  31704375
 
  31704844
 
  ENSEMBL   UCSC 
 
  470
 
  8
 
 34771
 
 tss
 
 0.25112
 
 0.24266
 
 Not queried
 
  1.1614207
 
       NA
 
       NA
  
 
  
 12
 
 118313657
 
 118313990
 
  ENSEMBL   UCSC 
 
  334
 
  5
 
 9313
 
 gene
 
 0.29595
 
 0.23019
 
 Not queried
 
  1.1613985
 
       NA
 
       NA
  
 
  
 11
 
   2466409
 
   2466909
 
  ENSEMBL   UCSC 
 
  501
 
  6
 
 6440
 
 gene;island
 
 0.23095
 
 0.18801
 
 Not queried
 
  1.1611221
 
       NA
 
       NA
  
 
  
 6
 
  27775346
 
  27776039
 
  ENSEMBL   UCSC 
 
  694
 
 10
 
 34654
 
 tss;island
 
 0.02847
 
 0.10705
 
 Not queried
 
  1.1611024
 
       NA
 
       NA
  
 
  
 1
 
 165325030
 
 165325231
 
  ENSEMBL   UCSC 
 
  202
 
  4
 
 2059
 
 tss
 
 0.03503
 
 0.06631
 
 Not queried
 
  1.1603395
 
       NA
 
       NA
  
 
  
 17
 
  28705147
 
  28705464
 
  ENSEMBL   UCSC 
 
  318
 
  4
 
 17207
 
 tss;island
 
 0.31192
 
 0.09679
 
 Not queried
 
  1.1601771
 
       NA
 
       NA
  
 
  
 12
 
 117476704
 
 117477105
 
  ENSEMBL   UCSC 
 
  402
 
  4
 
 10830
 
 island
 
 0.79883
 
 0.90714
 
 Not queried
 
  1.1599952
 
       NA
 
       NA
  
 
  
 16
 
   1440421
 
   1440688
 
  ENSEMBL   UCSC 
 
  268
 
  4
 
 15507
 
 island
 
 0.82515
 
 0.93132
 
 Not queried
 
  1.1597414
 
       NA
 
       NA
  
 
  
 11
 
  27744363
 
  27744816
 
  ENSEMBL   UCSC 
 
  454
 
  6
 
 7196
 
 tss;island
 
 0.03681
 
 0.02990
 
 Not queried
 
  1.1596128
 
       NA
 
       NA
  
 
  
 19
 
  58789751
 
  58790298
 
  ENSEMBL   UCSC 
 
  548
 
  9
 
 21100
 
 tss
 
 0.02272
 
 0.02628
 
 Not queried
 
  1.1594795
 
       NA
 
       NA
  
 
  
 11
 
  70557445
 
  70557881
 
  ENSEMBL   UCSC 
 
  437
 
  6
 
 6776
 
 gene
 
 0.83398
 
 0.92016
 
 Not queried
 
  1.1594691
 
       NA
 
       NA
  
 
  
 14
 
  60557893
 
  60558163
 
  ENSEMBL   UCSC 
 
  271
 
  4
 
 12406
 
 tss;island
 
 0.05594
 
 0.03993
 
 Not queried
 
  1.1589271
 
       NA
 
       NA
  
 
  
 6
 
  99842082
 
  99842368
 
  ENSEMBL   UCSC 
 
  287
 
  7
 
 35069
 
 tss
 
 0.15037
 
 0.13449
 
 Not queried
 
  1.1589100
 
       NA
 
       NA
  
 
  
 3
 
 182970675
 
 182971069
 
  ENSEMBL   UCSC 
 
  395
 
 11
 
 28006
 
 gene;island
 
 0.01761
 
 0.03994
 
 Not queried
 
  1.1588643
 
       NA
 
       NA
  
 
  
 X
 
  67913143
 
  67913327
 
  ENSEMBL   UCSC 
 
  185
 
  6
 
 41010
 
 gene;tss;island
 
 0.15167
 
 0.07162
 
 Not queried
 
  1.1582754
 
       NA
 
       NA
  
 
  
 1
 
   1342230
 
   1342566
 
  ENSEMBL   UCSC 
 
  337
 
  4
 
 36
 
 gene
 
 0.03550
 
 0.01473
 
 Not queried
 
  1.1582272
 
       NA
 
       NA
  
 
  
 1
 
   1342230
 
   1342795
 
  ENSEMBL   UCSC 
 
  566
 
  6
 
 2431
 
 island
 
 0.03550
 
 0.01403
 
 Not queried
 
  1.1582272
 
       NA
 
       NA
  
 
  
 5
 
 172744616
 
 172744941
 
  ENSEMBL   UCSC 
 
  326
 
  5
 
 31846
 
 gene
 
 0.77490
 
 0.89700
 
 Not queried
 
  1.1581992
 
       NA
 
       NA
  
 
  
 16
 
  88698516
 
  88699035
 
  ENSEMBL   UCSC 
 
  520
 
  5
 
 16174
 
 island
 
 0.73260
 
 0.87083
 
 Not queried
 
  1.1581406
 
       NA
 
       NA
  
 
  
 7
 
  83277941
 
  83278138
 
  ENSEMBL   UCSC 
 
  198
 
  5
 
 37005
 
 gene
 
 0.01665
 
 0.02445
 
 Not queried
 
  1.1577855
 
       NA
 
       NA
  
 
  
 4
 
 154605087
 
 154605468
 
  ENSEMBL   UCSC 
 
  382
 
  5
 
 31321
 
 island
 
 0.10234
 
 0.05685
 
 Not queried
 
  1.1574084
 
       NA
 
       NA
  
 
  
 6
 
  31622318
 
  31622439
 
  ENSEMBL   UCSC 
 
  122
 
  6
 
 34760
 
 tss;island
 
 0.87522
 
 0.93390
 
 Not queried
 
  1.1573664
 
       NA
 
       NA
  
 
  
 19
 
  38810162
 
  38810437
 
  ENSEMBL   UCSC 
 
  276
 
  8
 
 20715
 
 tss
 
 0.01900
 
 0.03764
 
 Not queried
 
  1.1572183
 
       NA
 
       NA
  
 
  
 11
 
 123396039
 
 123396505
 
  ENSEMBL   UCSC 
 
  467
 
  6
 
 7756
 
 tss
 
 0.49884
 
 0.34882
 
 Not queried
 
  1.1572096
 
       NA
 
       NA
  
 
  
 17
 
    259755
 
    260058
 
  ENSEMBL   UCSC 
 
  304
 
  9
 
 16946
 
 tss;island
 
 0.01473
 
 0.02125
 
 Not queried
 
  1.1572081
 
       NA
 
       NA
  
 
  
 17
 
  59539251
 
  59539715
 
  ENSEMBL   UCSC 
 
  465
 
  6
 
 16686
 
 gene;island
 
 0.18991
 
 0.33109
 
 Not queried
 
  1.1570086
 
       NA
 
       NA
  
 
  
 19
 
   5913854
 
   5914311
 
  ENSEMBL   UCSC 
 
  458
 
  6
 
 21385
 
 island
 
 0.72283
 
 0.88078
 
 Not queried
 
  1.1569611
 
       NA
 
       NA
  
 
  
 17
 
   7341436
 
   7341641
 
  ENSEMBL   UCSC 
 
  206
 
  4
 
 17050
 
 tss;island
 
 0.10949
 
 0.06823
 
 Not queried
 
  1.1568518
 
       NA
 
       NA
  
 
  
 X
 
  66763359
 
  66763691
 
  ENSEMBL   UCSC 
 
  333
 
  8
 
 41353
 
 tss;island
 
 0.16378
 
 0.08279
 
 Not queried
 
  1.1566956
 
       NA
 
       NA
  
 
  
 1
 
   7022041
 
   7022284
 
  ENSEMBL   UCSC 
 
  244
 
  4
 
 182
 
 gene;island
 
 0.02540
 
 0.07073
 
 Not queried
 
  1.1565073
 
       NA
 
       NA
  
 
  
 X
 
  54835315
 
  54835446
 
  ENSEMBL   UCSC 
 
  132
 
  6
 
 40999
 
 gene;tss;island
 
 0.09103
 
 0.03715
 
 Not queried
 
  1.1562562
 
       NA
 
       NA
  
 
  
 19
 
  34111400
 
  34111733
 
  ENSEMBL   UCSC 
 
  334
 
  5
 
 20650
 
 tss;island
 
 0.85470
 
 0.69650
 
 Not queried
 
  1.1560724
 
       NA
 
       NA
  
 
  
 6
 
 111926749
 
 111927323
 
  ENSEMBL   UCSC 
 
  575
 
  6
 
 34382
 
 gene
 
 0.01421
 
 0.02650
 
 Not queried
 
  1.1559625
 
       NA
 
       NA
  
 
  
 2
 
  74753281
 
  74753358
 
  ENSEMBL   UCSC 
 
   78
 
  4
 
 22710
 
 gene;island
 
 0.89188
 
 0.95354
 
 Not queried
 
  1.1558245
 
       NA
 
       NA
  
 
  
 19
 
  55866246
 
  55866412
 
  ENSEMBL   UCSC 
 
  167
 
  7
 
 21040
 
 tss
 
 0.04513
 
 0.08383
 
 Not queried
 
  1.1556814
 
       NA
 
       NA
  
 
  
 6
 
  28583971
 
  28584464
 
  ENSEMBL   UCSC 
 
  494
 
 18
 
 35528
 
 island
 
 0.30280
 
 0.51710
 
 Not queried
 
  1.1553765
 
       NA
 
       NA
  
 
  
 5
 
  75698309
 
  75698508
 
  ENSEMBL   UCSC 
 
  200
 
  4
 
 32074
 
 tss;island
 
 0.01840
 
 0.02766
 
 Not queried
 
  1.1553452
 
       NA
 
       NA
  
 
  
 10
 
  12085430
 
  12085641
 
  ENSEMBL   UCSC 
 
  212
 
  5
 
 4932
 
 tss;island
 
 0.01382
 
 0.05207
 
 Not queried
 
  1.1552183
 
       NA
 
       NA
  
 
  
 10
 
  30024742
 
  30024894
 
  ENSEMBL   UCSC 
 
  153
 
  5
 
 4992
 
 tss;island
 
 0.01642
 
 0.02260
 
 Not queried
 
  1.1551769
 
       NA
 
       NA
  
 
  
 14
 
  52455805
 
  52456142
 
  ENSEMBL   UCSC 
 
  338
 
  4
 
 12366
 
 tss;island
 
 0.01970
 
 0.04161
 
 Not queried
 
  1.1550082
 
       NA
 
       NA
  
 
  
 19
 
  10735482
 
  10735594
 
  ENSEMBL   UCSC 
 
  113
 
  4
 
 19781
 
 gene;tss;island
 
 0.48114
 
 0.76154
 
 Not queried
 
  1.1550011
 
       NA
 
       NA
  
 
  
 5
 
 139554307
 
 139554569
 
  ENSEMBL   UCSC 
 
  263
 
  8
 
 32279
 
 tss;island
 
 0.09070
 
 0.02825
 
 Not queried
 
  1.1546795
 
       NA
 
       NA
  
 
  
 6
 
  85484287
 
  85484863
 
  ENSEMBL   UCSC 
 
  577
 
  5
 
 36237
 
 island
 
 0.05958
 
 0.06092
 
 Not queried
 
  1.1545963
 
       NA
 
       NA
  
 
  
 5
 
  10563358
 
  10563856
 
  ENSEMBL   UCSC 
 
  499
 
  4
 
 31931
 
 tss;island
 
 0.18290
 
 0.09102
 
 Not queried
 
  1.1544730
 
       NA
 
       NA
  
 
  
 8
 
  87354422
 
  87354819
 
  ENSEMBL   UCSC 
 
  398
 
 11
 
 39734
 
 tss;island
 
 0.02390
 
 0.05091
 
 Not queried
 
  1.1543137
 
       NA
 
       NA
  
 
  
 1
 
 226595334
 
 226595776
 
  ENSEMBL   UCSC 
 
  443
 
  5
 
 1031
 
 gene
 
 0.01947
 
 0.03590
 
 Not queried
 
  1.1542794
 
       NA
 
       NA
  
 
  
 11
 
  66084599
 
  66084841
 
  ENSEMBL   UCSC 
 
  243
 
  4
 
 7443
 
 tss
 
 0.52553
 
 0.71099
 
 Not queried
 
  1.1541437
 
       NA
 
       NA
  
 
  
 15
 
  37391424
 
  37391721
 
  ENSEMBL   UCSC 
 
  298
 
  4
 
 13255
 
 gene;tss;island
 
 0.04672
 
 0.02047
 
 Not queried
 
  1.1531053
 
       NA
 
       NA
  
 
  
 16
 
  88972776
 
  88973351
 
  ENSEMBL   UCSC 
 
  576
 
  5
 
 14891
 
 gene;island
 
 0.69035
 
 0.82632
 
 Not queried
 
  1.1529117
 
       NA
 
       NA
  
 
  
 1
 
 153747551
 
 153747856
 
  ENSEMBL   UCSC 
 
  306
 
  7
 
 3761
 
 island
 
 0.03877
 
 0.08231
 
 Not queried
 
  1.1529037
 
       NA
 
       NA
  
 
  
 11
 
  32112091
 
  32112457
 
  ENSEMBL   UCSC 
 
  367
 
  5
 
 7205
 
 tss
 
 0.03345
 
 0.03424
 
 Not queried
 
  1.1528040
 
       NA
 
       NA
  
 
  
 3
 
 182970675
 
 182971025
 
  ENSEMBL   UCSC 
 
  351
 
 10
 
 28701
 
 tss
 
 0.01939
 
 0.05303
 
 Not queried
 
  1.1526908
 
       NA
 
       NA
  
 
  
 6
 
  30457039
 
  30457252
 
  ENSEMBL   UCSC 
 
  214
 
 10
 
 34708
 
 tss
 
 0.01534
 
 0.03563
 
 Not queried
 
  1.1526719
 
       NA
 
       NA
  
 
  
 8
 
  10755744
 
  10756220
 
  ENSEMBL   UCSC 
 
  477
 
  5
 
 39138
 
 gene
 
 0.92280
 
 0.95696
 
 Not queried
 
  1.1524891
 
       NA
 
       NA
  
 
  
 1
 
  43738485
 
  43738828
 
  ENSEMBL   UCSC 
 
  344
 
  5
 
 473
 
 gene;island
 
 0.84153
 
 0.70495
 
 Not queried
 
  1.1522410
 
       NA
 
       NA
  
 
  
 10
 
 129704949
 
 129705218
 
  ENSEMBL   UCSC 
 
  270
 
  5
 
 5419
 
 tss
 
 0.08310
 
 0.09165
 
 Not queried
 
  1.1520472
 
       NA
 
       NA
  
 
  
 7
 
  75831194
 
  75831678
 
  ENSEMBL   UCSC 
 
  485
 
  8
 
 38531
 
 island
 
 0.01237
 
 0.02524
 
 Not queried
 
  1.1520196
 
       NA
 
       NA
  
 
  
 6
 
  33160847
 
  33161589
 
  ENSEMBL   UCSC 
 
  743
 
 22
 
 34826
 
 tss;island
 
 0.40231
 
 0.64068
 
 Not queried
 
  1.1517478
 
       NA
 
       NA
  
 
  
 6
 
  74019562
 
  74020122
 
  ENSEMBL   UCSC 
 
  561
 
  6
 
 36187
 
 island
 
 0.02970
 
 0.01717
 
 Not queried
 
  1.1515728
 
       NA
 
       NA
  
 
  
 6
 
  35656590
 
  35656906
 
  ENSEMBL   UCSC 
 
  317
 
  4
 
 34245
 
 gene;island
 
 0.01966
 
 0.04534
 
 Not queried
 
  1.1512754
 
       NA
 
       NA
  
 
  
 4
 
   8395941
 
   8396502
 
  ENSEMBL   UCSC 
 
  562
 
  6
 
 29906
 
 gene
 
 0.84122
 
 0.92715
 
 Not queried
 
  1.1507634
 
       NA
 
       NA
  
 
  
 20
 
  48428997
 
  48429246
 
  ENSEMBL   UCSC 
 
  250
 
  8
 
 25771
 
 tss
 
 0.07950
 
 0.04500
 
 Not queried
 
  1.1507055
 
       NA
 
       NA
  
 
  
 7
 
 155255122
 
 155255369
 
  ENSEMBL   UCSC 
 
  248
 
  4
 
 37246
 
 gene;island
 
 0.89513
 
 0.78821
 
 Not queried
 
  1.1506775
 
       NA
 
       NA
  
 
  
 6
 
   3445256
 
   3445330
 
  ENSEMBL   UCSC 
 
   75
 
  4
 
 33473
 
 gene;tss
 
 0.77978
 
 0.88833
 
 Not queried
 
  1.1505756
 
       NA
 
       NA
  
 
  
 3
 
  11034749
 
  11035079
 
  ENSEMBL   UCSC 
 
  331
 
  4
 
 27622
 
 gene;island
 
 0.15024
 
 0.16555
 
 Not queried
 
  1.1503163
 
       NA
 
       NA
  
 
  
 15
 
  34502418
 
  34502580
 
  ENSEMBL   UCSC 
 
  163
 
  7
 
 13528
 
 tss;island
 
 0.03156
 
 0.03963
 
 Not queried
 
  1.1502228
 
       NA
 
       NA
  
 
  
 16
 
    331050
 
    331454
 
  ENSEMBL   UCSC 
 
  405
 
  4
 
 14495
 
 gene;island
 
 0.46881
 
 0.69265
 
 Not queried
 
  1.1498054
 
       NA
 
       NA
  
 
  
 7
 
 155604780
 
 155605094
 
  ENSEMBL   UCSC 
 
  315
 
  7
 
 39015
 
 island
 
 0.04353
 
 0.17309
 
 Not queried
 
  1.1495827
 
       NA
 
       NA
  
 
  
 2
 
  74699973
 
  74700469
 
  ENSEMBL   UCSC 
 
  497
 
  6
 
 23420
 
 tss
 
 0.14358
 
 0.05354
 
 Not queried
 
  1.1493004
 
       NA
 
       NA
  
 
  
 12
 
  48206783
 
  48207063
 
  ENSEMBL   UCSC 
 
  281
 
  4
 
 9081
 
 gene;island
 
 0.22776
 
 0.10862
 
 Not queried
 
  1.1492526
 
       NA
 
       NA
  
 
  
 6
 
  29690766
 
  29691003
 
  ENSEMBL   UCSC 
 
  238
 
  6
 
 34688
 
 tss
 
 0.23271
 
 0.36112
 
 Not queried
 
  1.1491087
 
       NA
 
       NA
  
 
  
 21
 
  45148694
 
  45149141
 
  ENSEMBL   UCSC 
 
  448
 
  5
 
 26406
 
 gene;island
 
 0.10105
 
 0.07328
 
 Not queried
 
  1.1489010
 
       NA
 
       NA
  
 
  
 1
 
    870791
 
    871057
 
  ENSEMBL   UCSC 
 
  267
 
  5
 
 2
 
 gene;island
 
 0.39526
 
 0.58120
 
 Not queried
 
  1.1486364
 
       NA
 
       NA
  
 
  
 6
 
  28048723
 
  28048973
 
  ENSEMBL   UCSC 
 
  251
 
  6
 
 33562
 
 gene
 
 0.01442
 
 0.02150
 
 Not queried
 
  1.1479226
 
       NA
 
       NA
  
 
  
 6
 
  42927940
 
  42928546
 
  ENSEMBL   UCSC 
 
  607
 
 25
 
 36083
 
 island
 
 0.02704
 
 0.05630
 
 Not queried
 
  1.1479130
 
       NA
 
       NA
  
 
  
 10
 
 133953871
 
 133954211
 
  ENSEMBL   UCSC 
 
  341
 
  4
 
 4837
 
 gene;island
 
 0.89270
 
 0.92123
 
 Not queried
 
  1.1475652
 
       NA
 
       NA
  
 
  
 19
 
   2095364
 
   2095643
 
  ENSEMBL   UCSC 
 
  280
 
  4
 
 19644
 
 gene;island
 
 0.32920
 
 0.51641
 
 Not queried
 
  1.1475450
 
       NA
 
       NA
  
 
  
 17
 
  79099711
 
  79100287
 
  ENSEMBL   UCSC 
 
  577
 
 10
 
 16861
 
 gene;tss;island
 
 0.70573
 
 0.89449
 
 Not queried
 
  1.1475233
 
       NA
 
       NA
  
 
  
 2
 
  38304720
 
  38304804
 
  ENSEMBL   UCSC 
 
   85
 
  4
 
 23272
 
 tss;island
 
 0.72890
 
 0.55733
 
 Not queried
 
  1.1472996
 
       NA
 
       NA
  
 
  
 14
 
  77647955
 
  77648283
 
  ENSEMBL   UCSC 
 
  329
 
  8
 
 13002
 
 island
 
 0.04403
 
 0.07893
 
 Not queried
 
  1.1472820
 
       NA
 
       NA
  
 
  
 17
 
  18854560
 
  18854799
 
  ENSEMBL   UCSC 
 
  240
 
  4
 
 17150
 
 tss
 
 0.96133
 
 0.92131
 
 Not queried
 
  1.1468121
 
       NA
 
       NA
  
 
  
 7
 
 141673582
 
 141673903
 
  ENSEMBL   UCSC 
 
  322
 
  4
 
 37883
 
 tss
 
 0.46149
 
 0.45901
 
 Not queried
 
  1.1468012
 
       NA
 
       NA
  
 
  
 2
 
 136876652
 
 136877039
 
  ENSEMBL   UCSC 
 
  388
 
  5
 
 23612
 
 tss;island
 
 0.01426
 
 0.03664
 
 Not queried
 
  1.1466848
 
       NA
 
       NA
  
 
  
 5
 
  80529067
 
  80529135
 
  ENSEMBL   UCSC 
 
   69
 
  4
 
 32094
 
 tss
 
 0.02940
 
 0.08025
 
 Not queried
 
  1.1466296
 
       NA
 
       NA
  
 
  
 5
 
    191127
 
    191806
 
  ENSEMBL   UCSC 
 
  680
 
 11
 
 32513
 
 island
 
 0.56478
 
 0.76891
 
 Not queried
 
  1.1465581
 
       NA
 
       NA
  
 
  
 4
 
 187509603
 
 187510183
 
  ENSEMBL   UCSC 
 
  581
 
  5
 
 30169
 
 gene
 
 0.94592
 
 0.96866
 
 Not queried
 
  1.1462258
 
       NA
 
       NA
  
 
  
 19
 
   3687751
 
   3688176
 
  ENSEMBL   UCSC 
 
  426
 
  5
 
 19686
 
 gene;island
 
 0.67899
 
 0.85540
 
 Not queried
 
  1.1461997
 
       NA
 
       NA
  
 
  
 17
 
  19314299
 
  19314408
 
  ENSEMBL   UCSC 
 
  110
 
  5
 
 17158
 
 tss
 
 0.70016
 
 0.88281
 
 Not queried
 
  1.1458845
 
       NA
 
       NA
  
 
  
 3
 
 186648175
 
 186648544
 
  ENSEMBL   UCSC 
 
  370
 
  5
 
 29712
 
 island
 
 0.04875
 
 0.03516
 
 Not queried
 
  1.1457181
 
       NA
 
       NA
  
 
  
 19
 
  39881965
 
  39882194
 
  ENSEMBL   UCSC 
 
  230
 
  4
 
 19992
 
 gene
 
 0.02207
 
 0.04912
 
 Not queried
 
  1.1454279
 
       NA
 
       NA
  
 
  
 1
 
 156252714
 
 156253152
 
  ENSEMBL   UCSC 
 
  439
 
  6
 
 817
 
 gene
 
 0.02918
 
 0.07134
 
 Not queried
 
  1.1454153
 
       NA
 
       NA
  
 
  
 7
 
  27209131
 
  27209582
 
  ENSEMBL   UCSC 
 
  452
 
 10
 
 38294
 
 island
 
 0.03770
 
 0.13508
 
 Not queried
 
  1.1452197
 
       NA
 
       NA
  
 
  
 22
 
  19554366
 
  19554706
 
  ENSEMBL   UCSC 
 
  341
 
  5
 
 26921
 
 tss
 
 0.74981
 
 0.85401
 
 Not queried
 
  1.1451561
 
       NA
 
       NA
  
 
  
 22
 
  19554053
 
  19554706
 
  ENSEMBL   UCSC 
 
  654
 
  7
 
 27257
 
 island
 
 0.74981
 
 0.85578
 
 Not queried
 
  1.1451561
 
       NA
 
       NA
  
 
  
 11
 
 104035087
 
 104035204
 
  ENSEMBL   UCSC 
 
  118
 
  4
 
 7631
 
 tss
 
 0.03136
 
 0.02307
 
 Not queried
 
  1.1449947
 
       NA
 
       NA
  
 
  
 20
 
  24449668
 
  24449804
 
  ENSEMBL   UCSC 
 
  137
 
  5
 
 25603
 
 tss
 
 0.16485
 
 0.05517
 
 Not queried
 
  1.1449921
 
       NA
 
       NA
  
 
  
 1
 
  43919949
 
  43920264
 
  ENSEMBL   UCSC 
 
  316
 
  8
 
 1560
 
 tss
 
 0.05780
 
 0.03200
 
 Not queried
 
  1.1448861
 
       NA
 
       NA
  
 
  
 16
 
   4421445
 
   4421654
 
  ENSEMBL   UCSC 
 
  210
 
  5
 
 14609
 
 gene;tss;island
 
 0.03892
 
 0.07691
 
 Not queried
 
  1.1447489
 
       NA
 
       NA
  
 
  
 1
 
  20126560
 
  20126875
 
  ENSEMBL   UCSC 
 
  316
 
  5
 
 1305
 
 tss
 
 0.01907
 
 0.04494
 
 Not queried
 
  1.1445143
 
       NA
 
       NA
  
 
  
 6
 
  31601157
 
  31601654
 
  ENSEMBL   UCSC 
 
  498
 
  7
 
 33857
 
 gene;island
 
 0.88538
 
 0.93783
 
 Not queried
 
  1.1444932
 
       NA
 
       NA
  
 
  
 16
 
  82660206
 
  82660505
 
  ENSEMBL   UCSC 
 
  300
 
  9
 
 15355
 
 tss
 
 0.27052
 
 0.29029
 
 Not queried
 
  1.1444111
 
       NA
 
       NA
  
 
  
 15
 
  90294568
 
  90294776
 
  ENSEMBL   UCSC 
 
  209
 
  6
 
 13863
 
 tss
 
 0.23496
 
 0.15079
 
 Not queried
 
  1.1443860
 
       NA
 
       NA
  
 
  
 3
 
  81810621
 
  81811448
 
  ENSEMBL   UCSC 
 
  828
 
 10
 
 29252
 
 island
 
 0.08021
 
 0.03597
 
 Not queried
 
  1.1442698
 
       NA
 
       NA
  
 
  
 11
 
 119993722
 
 119994008
 
  ENSEMBL   UCSC 
 
  287
 
  7
 
 6922
 
 gene
 
 0.89212
 
 0.79015
 
 Not queried
 
  1.1442360
 
       NA
 
       NA
  
 
  
 12
 
  53298678
 
  53298911
 
  ENSEMBL   UCSC 
 
  234
 
  4
 
 10422
 
 island
 
 0.04796
 
 0.09756
 
 Not queried
 
  1.1439228
 
       NA
 
       NA
  
 
  
 16
 
  67997921
 
  67998246
 
  ENSEMBL   UCSC 
 
  326
 
  4
 
 16005
 
 island
 
 0.74637
 
 0.58390
 
 Not queried
 
  1.1438948
 
       NA
 
       NA
  
 
  
 6
 
  32974843
 
  32975360
 
  ENSEMBL   UCSC 
 
  518
 
 13
 
 34111
 
 gene;island
 
 0.53741
 
 0.71411
 
 Not queried
 
  1.1438865
 
       NA
 
       NA
  
 
  
 18
 
  13611370
 
  13611489
 
  ENSEMBL   UCSC 
 
  120
 
  5
 
 19029
 
 gene;tss
 
 0.06295
 
 0.13992
 
 Not queried
 
  1.1437463
 
       NA
 
       NA
  
 
  
 6
 
  99276697
 
  99277015
 
  ENSEMBL   UCSC 
 
  319
 
  4
 
 36279
 
 island
 
 0.06755
 
 0.16816
 
 Not queried
 
  1.1432285
 
       NA
 
       NA
  
 
  
 19
 
   7741602
 
   7741955
 
  ENSEMBL   UCSC 
 
  354
 
  5
 
 21426
 
 island
 
 0.47286
 
 0.73580
 
 Not queried
 
  1.1431487
 
       NA
 
       NA
  
 
  
 1
 
  28969692
 
  28969987
 
  ENSEMBL   UCSC 
 
  296
 
  9
 
 1430
 
 tss
 
 0.02060
 
 0.03830
 
 Not queried
 
  1.1428651
 
       NA
 
       NA
  
 
  
 12
 
  68647289
 
  68647590
 
  ENSEMBL   UCSC 
 
  302
 
  6
 
 9828
 
 tss
 
 0.88965
 
 0.94255
 
 Not queried
 
  1.1426518
 
       NA
 
       NA
  
 
  
 6
 
  32821992
 
  32822352
 
  ENSEMBL   UCSC 
 
  361
 
 15
 
 34091
 
 gene
 
 0.04557
 
 0.09894
 
 Not queried
 
  1.1425339
 
       NA
 
       NA
  
 
  
 22
 
  45596797
 
  45597035
 
  ENSEMBL   UCSC 
 
  239
 
  8
 
 26858
 
 gene;island
 
 0.95965
 
 0.89442
 
 Not queried
 
  1.1425073
 
       NA
 
       NA
  
 
  
 14
 
  50999316
 
  50999710
 
  ENSEMBL   UCSC 
 
  395
 
 10
 
 12360
 
 tss;island
 
 0.04928
 
 0.02617
 
 Not queried
 
  1.1424059
 
       NA
 
       NA
  
 
  
 17
 
   7690159
 
   7690529
 
  ENSEMBL   UCSC 
 
  371
 
  5
 
 16377
 
 gene;island
 
 0.83374
 
 0.86123
 
 Not queried
 
  1.1422505
 
       NA
 
       NA
  
 
  
 9
 
 133589177
 
 133589654
 
  ENSEMBL   UCSC 
 
  478
 
  4
 
 40896
 
 island
 
 0.02498
 
 0.04949
 
 Not queried
 
  1.1421926
 
       NA
 
       NA
  
 
  
 11
 
   1297066
 
   1297558
 
  ENSEMBL   UCSC 
 
  493
 
  5
 
 6390
 
 gene;island
 
 0.82028
 
 0.89605
 
 Not queried
 
  1.1420538
 
       NA
 
       NA
  
 
  
 2
 
  85838641
 
  85839722
 
  ENSEMBL   UCSC 
 
 1082
 
  9
 
 24447
 
 island
 
 0.02916
 
 0.03609
 
 Not queried
 
  1.1419727
 
       NA
 
       NA
  
 
  
 6
 
  32813084
 
  32813715
 
  ENSEMBL   UCSC 
 
  632
 
 11
 
 34087
 
 gene
 
 0.87752
 
 0.70215
 
 Not queried
 
  1.1417955
 
       NA
 
       NA
  
 
  
 17
 
  79045526
 
  79045877
 
  ENSEMBL   UCSC 
 
  352
 
  5
 
 16858
 
 gene;island
 
 0.79381
 
 0.79800
 
 Not queried
 
  1.1414385
 
       NA
 
       NA
  
 
  
 8
 
  27472239
 
  27472513
 
  ENSEMBL   UCSC 
 
  275
 
 11
 
 40119
 
 island
 
 0.03026
 
 0.05886
 
 Not queried
 
  1.1412591
 
       NA
 
       NA
  
 
  
 6
 
  32141584
 
  32142236
 
  ENSEMBL   UCSC 
 
  653
 
 10
 
 34008
 
 gene
 
 0.77583
 
 0.93097
 
 Not queried
 
  1.1409660
 
       NA
 
       NA
  
 
  
 4
 
 129208725
 
 129209112
 
  ENSEMBL   UCSC 
 
  388
 
  6
 
 31248
 
 island
 
 0.05776
 
 0.10232
 
 Not queried
 
  1.1407170
 
       NA
 
       NA
  
 
  
 8
 
  49468684
 
  49468988
 
  ENSEMBL   UCSC 
 
  305
 
  6
 
 40218
 
 island
 
 0.08646
 
 0.04423
 
 Not queried
 
  1.1407110
 
       NA
 
       NA
  
 
  
 19
 
   2273706
 
   2274042
 
  ENSEMBL   UCSC 
 
  337
 
  4
 
 21239
 
 island
 
 0.17802
 
 0.10059
 
 Not queried
 
  1.1403519
 
       NA
 
       NA
  
 
  
 6
 
 150039190
 
 150039767
 
  ENSEMBL   UCSC 
 
  578
 
 10
 
 36495
 
 island
 
 0.01628
 
 0.05360
 
 Not queried
 
  1.1401636
 
       NA
 
       NA
  
 
  
 11
 
  63705693
 
  63705946
 
  ENSEMBL   UCSC 
 
  254
 
  4
 
 7358
 
 tss;island
 
 0.03971
 
 0.02399
 
 Not queried
 
  1.1399616
 
       NA
 
       NA
  
 
  
 7
 
  75624096
 
  75624190
 
  ENSEMBL   UCSC 
 
   95
 
  4
 
 37595
 
 tss
 
 0.02433
 
 0.04965
 
 Not queried
 
  1.1398319
 
       NA
 
       NA
  
 
  
 11
 
  20181877
 
  20182122
 
  ENSEMBL   UCSC 
 
  246
 
  7
 
 7175
 
 tss
 
 0.12041
 
 0.21175
 
 Not queried
 
  1.1395957
 
       NA
 
       NA
  
 
  
 12
 
 106460568
 
 106461007
 
  ENSEMBL   UCSC 
 
  440
 
  5
 
 9251
 
 gene;island
 
 0.92952
 
 0.87228
 
 Not queried
 
  1.1395529
 
       NA
 
       NA
  
 
  
 X
 
  40594903
 
  40595209
 
  ENSEMBL   UCSC 
 
  307
 
  6
 
 41263
 
 tss;island
 
 0.04053
 
 0.01992
 
 Not queried
 
  1.1395252
 
       NA
 
       NA
  
 
  
 18
 
  44526430
 
  44526756
 
  ENSEMBL   UCSC 
 
  327
 
  6
 
 19227
 
 tss
 
 0.00856
 
 0.01445
 
 Not queried
 
  1.1394195
 
       NA
 
       NA
  
 
  
 18
 
  44526430
 
  44527026
 
  ENSEMBL   UCSC 
 
  597
 
  8
 
 19459
 
 island
 
 0.00856
 
 0.01461
 
 Not queried
 
  1.1394195
 
       NA
 
       NA
  
 
  
 2
 
  42795925
 
  42796178
 
  ENSEMBL   UCSC 
 
  254
 
  4
 
 22619
 
 gene;island
 
 0.02951
 
 0.05414
 
 Not queried
 
  1.1393878
 
       NA
 
       NA
  
 
  
 11
 
  30344200
 
  30344599
 
  ENSEMBL   UCSC 
 
  400
 
  8
 
 7199
 
 tss
 
 0.03186
 
 0.02425
 
 Not queried
 
  1.1392144
 
       NA
 
       NA
  
 
  
 19
 
  13049163
 
  13049313
 
  ENSEMBL   UCSC 
 
  151
 
  6
 
 20485
 
 tss
 
 0.01561
 
 0.03418
 
 Not queried
 
  1.1391765
 
       NA
 
       NA
  
 
  
 6
 
  28956226
 
  28956989
 
  ENSEMBL   UCSC 
 
  764
 
 22
 
 35545
 
 island
 
 0.02483
 
 0.05547
 
 Not queried
 
  1.1388731
 
       NA
 
       NA
  
 
  
 1
 
 223538174
 
 223538379
 
  ENSEMBL   UCSC 
 
  206
 
  4
 
 2267
 
 tss;island
 
 0.07154
 
 0.15499
 
 Not queried
 
  1.1386355
 
       NA
 
       NA
  
 
  
 7
 
  27183369
 
  27184737
 
  ENSEMBL   UCSC 
 
 1369
 
 41
 
 37427
 
 tss
 
 0.20106
 
 0.36646
 
 Not queried
 
  1.1386095
 
       NA
 
       NA
  
 
  
 6
 
  36645500
 
  36645886
 
  ENSEMBL   UCSC 
 
  387
 
  5
 
 34876
 
 tss;island
 
 0.87306
 
 0.76446
 
 Not queried
 
  1.1380747
 
       NA
 
       NA
  
 
  
 12
 
 115130855
 
 115131549
 
  ENSEMBL   UCSC 
 
  695
 
  8
 
 10821
 
 island
 
 0.49560
 
 0.32031
 
 Not queried
 
  1.1379893
 
       NA
 
       NA
  
 
  
 10
 
 134121577
 
 134121712
 
  ENSEMBL   UCSC 
 
  136
 
  5
 
 5428
 
 tss
 
 0.02878
 
 0.02369
 
 Not queried
 
  1.1379517
 
       NA
 
       NA
  
 
  
 4
 
 154125208
 
 154125594
 
  ENSEMBL   UCSC 
 
  387
 
  6
 
 30592
 
 tss
 
 0.02823
 
 0.05284
 
 Not queried
 
  1.1379288
 
       NA
 
       NA
  
 
  
 17
 
  49242714
 
  49243027
 
  ENSEMBL   UCSC 
 
  314
 
  6
 
 16659
 
 gene;tss;island
 
 0.62011
 
 0.47465
 
 Not queried
 
  1.1375879
 
       NA
 
       NA
  
 
  
 1
 
 186649153
 
 186649530
 
  ENSEMBL   UCSC 
 
  378
 
  6
 
 922
 
 gene
 
 0.01272
 
 0.01966
 
 Not queried
 
  1.1374320
 
       NA
 
       NA
  
 
  
 19
 
  19648890
 
  19649054
 
  ENSEMBL   UCSC 
 
  165
 
  7
 
 20609
 
 tss
 
 0.28844
 
 0.16234
 
 Not queried
 
  1.1373286
 
       NA
 
       NA
  
 
  
 1
 
  44870946
 
  44871634
 
  ENSEMBL   UCSC 
 
  689
 
  8
 
 3175
 
 island
 
 0.01062
 
 0.03064
 
 Not queried
 
  1.1370440
 
       NA
 
       NA
  
 
  
 8
 
  28928948
 
  28929242
 
  ENSEMBL   UCSC 
 
  295
 
  4
 
 39204
 
 gene;island
 
 0.77122
 
 0.91586
 
 Not queried
 
  1.1369661
 
       NA
 
       NA
  
 
  
 6
 
  17102310
 
  17102678
 
  ENSEMBL   UCSC 
 
  369
 
  6
 
 35409
 
 island
 
 0.03492
 
 0.06349
 
 Not queried
 
  1.1368672
 
       NA
 
       NA
  
 
  
 17
 
   7217969
 
   7218247
 
  ENSEMBL   UCSC 
 
  279
 
  4
 
 16354
 
 gene;island
 
 0.03095
 
 0.05452
 
 Not queried
 
  1.1367181
 
       NA
 
       NA
  
 
  
 2
 
 240001910
 
 240002329
 
  ENSEMBL   UCSC 
 
  420
 
  4
 
 23066
 
 gene;island
 
 0.88827
 
 0.88860
 
 Not queried
 
  1.1363619
 
       NA
 
       NA
  
 
  
 4
 
  54931064
 
  54931210
 
  ENSEMBL   UCSC 
 
  147
 
  4
 
 30351
 
 tss;island
 
 0.00869
 
 0.02036
 
 Not queried
 
  1.1359704
 
       NA
 
       NA
  
 
  
 17
 
   7475727
 
   7476052
 
  ENSEMBL   UCSC 
 
  326
 
  8
 
 17055
 
 tss
 
 0.02368
 
 0.04450
 
 Not queried
 
  1.1357525
 
       NA
 
       NA
  
 
  
 2
 
 176947764
 
 176948105
 
  ENSEMBL   UCSC 
 
  342
 
  4
 
 22897
 
 gene;island
 
 0.01580
 
 0.04765
 
 Not queried
 
  1.1356680
 
       NA
 
       NA
  
 
  
 10
 
  92913472
 
  92913775
 
  ENSEMBL   UCSC 
 
  304
 
  4
 
 5203
 
 tss;island
 
 0.76556
 
 0.54392
 
 Not queried
 
  1.1355839
 
       NA
 
       NA
  
 
  
 2
 
  84743142
 
  84743573
 
  ENSEMBL   UCSC 
 
  432
 
  9
 
 23437
 
 tss
 
 0.01440
 
 0.04711
 
 Not queried
 
  1.1354963
 
       NA
 
       NA
  
 
  
 6
 
  99292665
 
  99293069
 
  ENSEMBL   UCSC 
 
  405
 
  5
 
 36286
 
 island
 
 0.03636
 
 0.02304
 
 Not queried
 
  1.1353225
 
       NA
 
       NA
  
 
  
 1
 
 153651886
 
 153652247
 
  ENSEMBL   UCSC 
 
  362
 
  4
 
 763
 
 gene;island
 
 0.06936
 
 0.03344
 
 Not queried
 
  1.1352859
 
       NA
 
       NA
  
 
  
 6
 
  31600180
 
  31600931
 
  ENSEMBL   UCSC 
 
  752
 
 15
 
 33856
 
 gene;island
 
 0.78902
 
 0.91352
 
 Not queried
 
  1.1351997
 
       NA
 
       NA
  
 
  
 X
 
 113624821
 
 113625152
 
  ENSEMBL   UCSC 
 
  332
 
  4
 
 41965
 
 island
 
 0.91269
 
 0.93766
 
 Not queried
 
  1.1350493
 
       NA
 
       NA
  
 
  
 12
 
  56473229
 
  56473759
 
  ENSEMBL   UCSC 
 
  531
 
  6
 
 9756
 
 tss;island
 
 0.02442
 
 0.05796
 
 Not queried
 
  1.1350048
 
       NA
 
       NA
  
 
  
 6
 
  44253910
 
  44254291
 
  ENSEMBL   UCSC 
 
  382
 
  6
 
 34290
 
 gene;island
 
 0.52348
 
 0.74652
 
 Not queried
 
  1.1348781
 
       NA
 
       NA
  
 
  
 17
 
  27312855
 
  27313254
 
  ENSEMBL   UCSC 
 
  400
 
  6
 
 16468
 
 gene;island
 
 0.30689
 
 0.11288
 
 Not queried
 
  1.1348303
 
       NA
 
       NA
  
 
  
 6
 
  28129313
 
  28129498
 
  ENSEMBL   UCSC 
 
  186
 
  7
 
 34670
 
 tss
 
 0.09393
 
 0.06177
 
 Not queried
 
  1.1345055
 
       NA
 
       NA
  
 
  
 19
 
  13976610
 
  13976956
 
  ENSEMBL   UCSC 
 
  347
 
  5
 
 21624
 
 island
 
 0.03726
 
 0.11465
 
 Not queried
 
  1.1344963
 
       NA
 
       NA
  
 
  
 4
 
  55095148
 
  55095529
 
  ENSEMBL   UCSC 
 
  382
 
  8
 
 31003
 
 island
 
 0.17910
 
 0.08695
 
 Not queried
 
  1.1342452
 
       NA
 
       NA
  
 
  
 21
 
  40984760
 
  40984886
 
  ENSEMBL   UCSC 
 
  127
 
  5
 
 26511
 
 tss
 
 0.03911
 
 0.05500
 
 Not queried
 
  1.1342362
 
       NA
 
       NA
  
 
  
 6
 
  30165961
 
  30166173
 
  ENSEMBL   UCSC 
 
  213
 
  6
 
 33683
 
 gene
 
 0.87209
 
 0.93896
 
 Not queried
 
  1.1341115
 
       NA
 
       NA
  
 
  
 7
 
  93519220
 
  93519621
 
  ENSEMBL   UCSC 
 
  402
 
  4
 
 37027
 
 gene;island
 
 0.05826
 
 0.12172
 
 Not queried
 
  1.1340897
 
       NA
 
       NA
  
 
  
 11
 
  57424284
 
  57424534
 
  ENSEMBL   UCSC 
 
  251
 
  4
 
 7284
 
 tss;island
 
 0.01910
 
 0.04066
 
 Not queried
 
  1.1339659
 
       NA
 
       NA
  
 
  
 6
 
 170591749
 
 170592108
 
  ENSEMBL   UCSC 
 
  360
 
  6
 
 34516
 
 gene;island
 
 0.92457
 
 0.86968
 
 Not queried
 
  1.1339497
 
       NA
 
       NA
  
 
  
 4
 
 146403866
 
 146404134
 
  ENSEMBL   UCSC 
 
  269
 
  8
 
 30099
 
 gene;island
 
 0.01053
 
 0.02561
 
 Not queried
 
  1.1333874
 
       NA
 
       NA
  
 
  
 11
 
 133938676
 
 133938810
 
  ENSEMBL   UCSC 
 
  135
 
  4
 
 7805
 
 tss
 
 0.02439
 
 0.04677
 
 Not queried
 
  1.1331214
 
       NA
 
       NA
  
 
  
 11
 
 133938676
 
 133939064
 
  ENSEMBL   UCSC 
 
  389
 
  6
 
 8950
 
 island
 
 0.02439
 
 0.03994
 
 Not queried
 
  1.1331214
 
       NA
 
       NA
  
 
  
 2
 
 233390712
 
 233390969
 
  ENSEMBL   UCSC 
 
  258
 
  5
 
 25160
 
 island
 
 0.83085
 
 0.71035
 
 Not queried
 
  1.1329764
 
       NA
 
       NA
  
 
  
 11
 
   1483099
 
   1483390
 
  ENSEMBL   UCSC 
 
  292
 
  4
 
 7904
 
 island
 
 0.85435
 
 0.93730
 
 Not queried
 
  1.1329496
 
       NA
 
       NA
  
 
  
 11
 
  63753376
 
  63753598
 
  ENSEMBL   UCSC 
 
  223
 
  4
 
 6639
 
 gene
 
 0.11181
 
 0.17698
 
 Not queried
 
  1.1328946
 
       NA
 
       NA
  
 
  
 6
 
  32803444
 
  32803500
 
  ENSEMBL   UCSC 
 
   57
 
  5
 
 34079
 
 gene;island
 
 0.64540
 
 0.53085
 
 Not queried
 
  1.1328610
 
       NA
 
       NA
  
 
  
 1
 
 156721823
 
 156722410
 
  ENSEMBL   UCSC 
 
  588
 
 12
 
 2019
 
 tss;island
 
 0.06078
 
 0.02250
 
 Not queried
 
  1.1327035
 
       NA
 
       NA
  
 
  
 19
 
   1650224
 
   1650452
 
  ENSEMBL   UCSC 
 
  229
 
  4
 
 19625
 
 gene;island
 
 0.66913
 
 0.83939
 
 Not queried
 
  1.1326545
 
       NA
 
       NA
  
 
  
 12
 
 102036140
 
 102036563
 
  ENSEMBL   UCSC 
 
  424
 
  5
 
 9244
 
 gene;island
 
 0.02150
 
 0.03034
 
 Not queried
 
  1.1323483
 
       NA
 
       NA
  
 
  
 X
 
  99891813
 
  99892067
 
  ENSEMBL   UCSC 
 
  255
 
  6
 
 41416
 
 tss
 
 0.06127
 
 0.01301
 
 Not queried
 
  1.1315369
 
       NA
 
       NA
  
 
  
 12
 
 133384565
 
 133384913
 
  ENSEMBL   UCSC 
 
  349
 
  4
 
 9438
 
 gene
 
 0.74874
 
 0.81717
 
 Not queried
 
  1.1314228
 
       NA
 
       NA
  
 
  
 3
 
  32726203
 
  32726639
 
  ENSEMBL   UCSC 
 
  437
 
  8
 
 28165
 
 tss
 
 0.01473
 
 0.03427
 
 Not queried
 
  1.1313463
 
       NA
 
       NA
  
 
  
 3
 
  32726203
 
  32726786
 
  ENSEMBL   UCSC 
 
  584
 
 10
 
 28924
 
 island
 
 0.01318
 
 0.02698
 
 Not queried
 
  1.1313463
 
       NA
 
       NA
  
 
  
 14
 
  78432211
 
  78432599
 
  ENSEMBL   UCSC 
 
  389
 
  4
 
 13015
 
 island
 
 0.27367
 
 0.40742
 
 Not queried
 
  1.1311132
 
       NA
 
       NA
  
 
  
 6
 
  31645968
 
  31646377
 
  ENSEMBL   UCSC 
 
  410
 
  7
 
 33874
 
 gene;island
 
 0.43855
 
 0.62827
 
 Not queried
 
  1.1311102
 
       NA
 
       NA
  
 
  
 3
 
  52526235
 
  52526520
 
  ENSEMBL   UCSC 
 
  286
 
  4
 
 27779
 
 gene;island
 
 0.77649
 
 0.87679
 
 Not queried
 
  1.1306808
 
       NA
 
       NA
  
 
  
 6
 
  30878399
 
  30879000
 
  ENSEMBL   UCSC 
 
  602
 
 10
 
 33770
 
 gene;island
 
 0.82375
 
 0.90303
 
 Not queried
 
  1.1305399
 
       NA
 
       NA
  
 
  
 11
 
  18230754
 
  18231021
 
  ENSEMBL   UCSC 
 
  268
 
  5
 
 6508
 
 gene
 
 0.94430
 
 0.88564
 
 Not queried
 
  1.1303872
 
       NA
 
       NA
  
 
  
 7
 
 155604999
 
 155605094
 
  ENSEMBL   UCSC 
 
   96
 
  5
 
 37950
 
 tss
 
 0.04353
 
 0.09908
 
 Not queried
 
  1.1303693
 
       NA
 
       NA
  
 
  
 X
 
  53123426
 
  53123624
 
  ENSEMBL   UCSC 
 
  199
 
  4
 
 41805
 
 island
 
 0.08228
 
 0.03779
 
 Not queried
 
  1.1301247
 
       NA
 
       NA
  
 
  
 14
 
  20881581
 
  20881764
 
  ENSEMBL   UCSC 
 
  184
 
  5
 
 12241
 
 tss
 
 0.01669
 
 0.02523
 
 Not queried
 
  1.1297077
 
       NA
 
       NA
  
 
  
 12
 
   7260546
 
   7260888
 
  ENSEMBL   UCSC 
 
  343
 
  8
 
 9019
 
 gene;tss
 
 0.59988
 
 0.37028
 
 Not queried
 
  1.1295459
 
       NA
 
       NA
  
 
  
 17
 
  37894258
 
  37894413
 
  ENSEMBL   UCSC 
 
  156
 
  4
 
 16520
 
 gene
 
 0.02079
 
 0.03184
 
 Not queried
 
  1.1294356
 
       NA
 
       NA
  
 
  
 10
 
  88125351
 
  88125521
 
  ENSEMBL   UCSC 
 
  171
 
  4
 
 4646
 
 gene;island
 
 0.23730
 
 0.34876
 
 Not queried
 
  1.1293545
 
       NA
 
       NA
  
 
  
 6
 
  31938744
 
  31939322
 
  ENSEMBL   UCSC 
 
  579
 
 19
 
 33944
 
 gene;tss;island
 
 0.71199
 
 0.78561
 
 Not queried
 
  1.1293034
 
       NA
 
       NA
  
 
  
 11
 
   9385487
 
   9385980
 
  ENSEMBL   UCSC 
 
  494
 
  4
 
 8024
 
 island
 
 0.04438
 
 0.13623
 
 Not queried
 
  1.1292744
 
       NA
 
       NA
  
 
  
 12
 
 133351029
 
 133351421
 
  ENSEMBL   UCSC 
 
  393
 
  5
 
 9436
 
 gene;island
 
 0.83487
 
 0.88975
 
 Not queried
 
  1.1289915
 
       NA
 
       NA
  
 
  
 22
 
  17083412
 
  17083727
 
  ENSEMBL   UCSC 
 
  316
 
  4
 
 26738
 
 gene;island
 
 0.01075
 
 0.02395
 
 Not queried
 
  1.1288419
 
       NA
 
       NA
  
 
  
 11
 
  62369394
 
  62369870
 
  ENSEMBL   UCSC 
 
  477
 
  7
 
 7327
 
 tss;island
 
 0.02083
 
 0.04473
 
 Not queried
 
  1.1287405
 
       NA
 
       NA
  
 
  
 17
 
   7342585
 
   7342894
 
  ENSEMBL   UCSC 
 
  310
 
  4
 
 17916
 
 island
 
 0.39413
 
 0.23111
 
 Not queried
 
  1.1286842
 
       NA
 
       NA
  
 
  
 8
 
 144503255
 
 144503586
 
  ENSEMBL   UCSC 
 
  332
 
  4
 
 40596
 
 island
 
 0.56353
 
 0.81638
 
 Not queried
 
  1.1285236
 
       NA
 
       NA
  
 
  
 3
 
 124931627
 
 124931746
 
  ENSEMBL   UCSC 
 
  120
 
  4
 
 28507
 
 tss
 
 0.13842
 
 0.05657
 
 Not queried
 
  1.1284542
 
       NA
 
       NA
  
 
  
 11
 
  67276127
 
  67276453
 
  ENSEMBL   UCSC 
 
  327
 
  9
 
 7475
 
 tss
 
 0.01495
 
 0.02569
 
 Not queried
 
  1.1284197
 
       NA
 
       NA
  
 
  
 3
 
  37284287
 
  37284680
 
  ENSEMBL   UCSC 
 
  394
 
  9
 
 28180
 
 tss;island
 
 0.03569
 
 0.02419
 
 Not queried
 
  1.1283474
 
       NA
 
       NA
  
 
  
 17
 
  17991157
 
  17991318
 
  ENSEMBL   UCSC 
 
  162
 
  4
 
 18044
 
 island
 
 0.03678
 
 0.07043
 
 Not queried
 
  1.1279869
 
       NA
 
       NA
  
 
  
 1
 
 227058426
 
 227059109
 
  ENSEMBL   UCSC 
 
  684
 
  6
 
 1035
 
 gene;island
 
 0.04902
 
 0.06257
 
 Not queried
 
  1.1277598
 
       NA
 
       NA
  
 
  
 11
 
  67806337
 
  67806668
 
  ENSEMBL   UCSC 
 
  332
 
  8
 
 8542
 
 island
 
 0.02854
 
 0.06683
 
 Not queried
 
  1.1276689
 
       NA
 
       NA
  
 
  
 1
 
  33502512
 
  33502810
 
  ENSEMBL   UCSC 
 
  299
 
  8
 
 1465
 
 tss
 
 0.01638
 
 0.03616
 
 Not queried
 
  1.1276594
 
       NA
 
       NA
  
 
  
 1
 
  79472282
 
  79472452
 
  ENSEMBL   UCSC 
 
  171
 
  5
 
 595
 
 gene
 
 0.03335
 
 0.11008
 
 Not queried
 
  1.1275651
 
       NA
 
       NA
  
 
  
 19
 
  39935857
 
  39936212
 
  ENSEMBL   UCSC 
 
  356
 
  9
 
 20737
 
 tss
 
 0.01695
 
 0.02748
 
 Not queried
 
  1.1274394
 
       NA
 
       NA
  
 
  
 16
 
  84178939
 
  84179331
 
  ENSEMBL   UCSC 
 
  393
 
  6
 
 14833
 
 gene
 
 0.01803
 
 0.03873
 
 Not queried
 
  1.1273512
 
       NA
 
       NA
  
 
  
 5
 
 148931071
 
 148931367
 
  ENSEMBL   UCSC 
 
  297
 
  8
 
 32360
 
 tss;island
 
 0.00796
 
 0.01361
 
 Not queried
 
  1.1269977
 
       NA
 
       NA
  
 
  
 11
 
  65123257
 
  65123609
 
  ENSEMBL   UCSC 
 
  353
 
  4
 
 6688
 
 gene;island
 
 0.40638
 
 0.61438
 
 Not queried
 
  1.1268885
 
       NA
 
       NA
  
 
  
 5
 
  31532447
 
  31532653
 
  ENSEMBL   UCSC 
 
  207
 
  4
 
 31520
 
 gene
 
 0.01105
 
 0.02363
 
 Not queried
 
  1.1267440
 
       NA
 
       NA
  
 
  
 11
 
  18230491
 
  18231021
 
  ENSEMBL   UCSC 
 
  531
 
 10
 
 8086
 
 island
 
 0.93838
 
 0.89931
 
 Not queried
 
  1.1266683
 
       NA
 
       NA
  
 
  
 4
 
  37245916
 
  37246280
 
  ENSEMBL   UCSC 
 
  365
 
  4
 
 30293
 
 tss;island
 
 0.04350
 
 0.09786
 
 Not queried
 
  1.1265387
 
       NA
 
       NA
  
 
  
 11
 
  65153757
 
  65153960
 
  ENSEMBL   UCSC 
 
  204
 
  9
 
 7406
 
 tss
 
 0.03101
 
 0.04783
 
 Not queried
 
  1.1259920
 
       NA
 
       NA
  
 
  
 11
 
  66049756
 
  66050123
 
  ENSEMBL   UCSC 
 
  368
 
  4
 
 6710
 
 gene;island
 
 0.40013
 
 0.19054
 
 Not queried
 
  1.1258422
 
       NA
 
       NA
  
 
  
 7
 
  23637017
 
  23637253
 
  ENSEMBL   UCSC 
 
  237
 
  5
 
 36855
 
 gene
 
 0.01731
 
 0.03183
 
 Not queried
 
  1.1256755
 
       NA
 
       NA
  
 
  
 11
 
  64107374
 
  64107672
 
  ENSEMBL   UCSC 
 
  299
 
  4
 
 7376
 
 tss
 
 0.77740
 
 0.61028
 
 Not queried
 
  1.1254906
 
       NA
 
       NA
  
 
  
 10
 
 101088533
 
 101089149
 
  ENSEMBL   UCSC 
 
  617
 
  7
 
 5988
 
 island
 
 0.26124
 
 0.56485
 
 Not queried
 
  1.1254900
 
       NA
 
       NA
  
 
  
 3
 
  28390637
 
  28390998
 
  ENSEMBL   UCSC 
 
  362
 
  7
 
 28156
 
 tss;island
 
 0.02238
 
 0.03827
 
 Not queried
 
  1.1254453
 
       NA
 
       NA
  
 
  
 2
 
  24162256
 
  24162667
 
  ENSEMBL   UCSC 
 
  412
 
  5
 
 23181
 
 tss;island
 
 0.07405
 
 0.05512
 
 Not queried
 
  1.1252458
 
       NA
 
       NA
  
 
  
 4
 
   1044486
 
   1044797
 
  ENSEMBL   UCSC 
 
  312
 
  4
 
 30715
 
 island
 
 0.78910
 
 0.91876
 
 Not queried
 
  1.1250225
 
       NA
 
       NA
  
 
  
 16
 
  67312928
 
  67313043
 
  ENSEMBL   UCSC 
 
  116
 
  7
 
 14767
 
 gene;tss;island
 
 0.03548
 
 0.06941
 
 Not queried
 
  1.1249585
 
       NA
 
       NA
  
 
  
 10
 
 128994297
 
 128994702
 
  ENSEMBL   UCSC 
 
  406
 
  7
 
 4803
 
 gene;island
 
 0.01479
 
 0.03415
 
 Not queried
 
  1.1247836
 
       NA
 
       NA
  
 
  
 17
 
  79317768
 
  79318146
 
  ENSEMBL   UCSC 
 
  379
 
  4
 
 18881
 
 island
 
 0.04902
 
 0.01628
 
 Not queried
 
  1.1247168
 
       NA
 
       NA
  
 
  
 8
 
  65498340
 
  65498812
 
  ENSEMBL   UCSC 
 
  473
 
  6
 
 40283
 
 island
 
 0.53405
 
 0.62168
 
 Not queried
 
  1.1246746
 
       NA
 
       NA
  
 
  
 8
 
  27695476
 
  27695870
 
  ENSEMBL   UCSC 
 
  395
 
  8
 
 39554
 
 tss
 
 0.05384
 
 0.15412
 
 Not queried
 
  1.1243380
 
       NA
 
       NA
  
 
  
 4
 
   8345155
 
   8345588
 
  ENSEMBL   UCSC 
 
  434
 
  4
 
 30869
 
 island
 
 0.85843
 
 0.92108
 
 Not queried
 
  1.1243159
 
       NA
 
       NA
  
 
  
 6
 
  31431312
 
  31431573
 
  ENSEMBL   UCSC 
 
  262
 
  4
 
 33814
 
 gene
 
 0.63900
 
 0.54424
 
 Not queried
 
  1.1242965
 
       NA
 
       NA
  
 
  
 1
 
  28969554
 
  28969987
 
  ENSEMBL   UCSC 
 
  434
 
 11
 
 2943
 
 island
 
 0.02060
 
 0.03830
 
 Not queried
 
  1.1241646
 
       NA
 
       NA
  
 
  
 5
 
 101631990
 
 101632559
 
  ENSEMBL   UCSC 
 
  570
 
 12
 
 32923
 
 island
 
 0.08398
 
 0.06259
 
 Not queried
 
  1.1241021
 
       NA
 
       NA
  
 
  
 13
 
 100637113
 
 100637258
 
  ENSEMBL   UCSC 
 
  146
 
  4
 
 11182
 
 gene;island
 
 0.06192
 
 0.02050
 
 Not queried
 
  1.1240414
 
       NA
 
       NA
  
 
  
 2
 
  97426921
 
  97427102
 
  ENSEMBL   UCSC 
 
  182
 
  4
 
 22749
 
 gene;island
 
 0.11927
 
 0.21623
 
 Not queried
 
  1.1239544
 
       NA
 
       NA
  
 
  
 16
 
  74641227
 
  74641419
 
  ENSEMBL   UCSC 
 
  193
 
  4
 
 15324
 
 tss;island
 
 0.03336
 
 0.02746
 
 Not queried
 
  1.1237120
 
       NA
 
       NA
  
 
  
 12
 
  52679967
 
  52680245
 
  ENSEMBL   UCSC 
 
  279
 
  4
 
 9111
 
 gene
 
 0.92957
 
 0.74592
 
 Not queried
 
  1.1236110
 
       NA
 
       NA
  
 
  
 7
 
  98552702
 
  98552952
 
  ENSEMBL   UCSC 
 
  251
 
  4
 
 37053
 
 gene;island
 
 0.78158
 
 0.89702
 
 Not queried
 
  1.1235232
 
       NA
 
       NA
  
 
  
 18
 
  72999596
 
  73000004
 
  ENSEMBL   UCSC 
 
  409
 
  5
 
 19096
 
 gene;island
 
 0.87922
 
 0.91239
 
 Not queried
 
  1.1231400
 
       NA
 
       NA
  
 
  
 13
 
  99405102
 
  99405269
 
  ENSEMBL   UCSC 
 
  168
 
  5
 
 11482
 
 tss;island
 
 0.47584
 
 0.42822
 
 Not queried
 
  1.1229733
 
       NA
 
       NA
  
 
  
 19
 
  48964810
 
  48965006
 
  ENSEMBL   UCSC 
 
  197
 
  4
 
 20090
 
 gene;island
 
 0.85109
 
 0.90071
 
 Not queried
 
  1.1229681
 
       NA
 
       NA
  
 
  
 11
 
  18230491
 
  18230799
 
  ENSEMBL   UCSC 
 
  309
 
  7
 
 7161
 
 tss
 
 0.93247
 
 0.91298
 
 Not queried
 
  1.1229495
 
       NA
 
       NA
  
 
  
 11
 
  75141722
 
  75142012
 
  ENSEMBL   UCSC 
 
  291
 
  7
 
 7546
 
 tss
 
 0.06489
 
 0.17006
 
 Not queried
 
  1.1227782
 
       NA
 
       NA
  
 
  
 10
 
 133793442
 
 133793734
 
  ENSEMBL   UCSC 
 
  293
 
  5
 
 4830
 
 gene;island
 
 0.06966
 
 0.11047
 
 Not queried
 
  1.1227753
 
       NA
 
       NA
  
 
  
 6
 
  40345376
 
  40345751
 
  ENSEMBL   UCSC 
 
  376
 
  4
 
 34897
 
 tss
 
 0.86405
 
 0.94466
 
 Not queried
 
  1.1224542
 
       NA
 
       NA
  
 
  
 6
 
 169261176
 
 169261475
 
  ENSEMBL   UCSC 
 
  300
 
  5
 
 36607
 
 island
 
 0.83555
 
 0.88071
 
 Not queried
 
  1.1223676
 
       NA
 
       NA
  
 
  
 1
 
   6052593
 
   6052770
 
  ENSEMBL   UCSC 
 
  178
 
  4
 
 1192
 
 tss
 
 0.01693
 
 0.02836
 
 Not queried
 
  1.1222846
 
       NA
 
       NA
  
 
  
 15
 
  25436114
 
  25436246
 
  ENSEMBL   UCSC 
 
  133
 
  4
 
 13503
 
 tss
 
 0.63880
 
 0.73594
 
 Not queried
 
  1.1222401
 
       NA
 
       NA
  
 
  
 5
 
  95159365
 
  95159639
 
  ENSEMBL   UCSC 
 
  275
 
  4
 
 32128
 
 tss
 
 0.61967
 
 0.40676
 
 Not queried
 
  1.1222046
 
       NA
 
       NA
  
 
  
 19
 
   6481819
 
   6482169
 
  ENSEMBL   UCSC 
 
  351
 
  8
 
 20355
 
 tss
 
 0.36845
 
 0.51980
 
 Not queried
 
  1.1219996
 
       NA
 
       NA
  
 
  
 2
 
  11679845
 
  11680144
 
  ENSEMBL   UCSC 
 
  300
 
  7
 
 22534
 
 gene
 
 0.79203
 
 0.93278
 
 Not queried
 
  1.1219446
 
       NA
 
       NA
  
 
  
 2
 
  11679845
 
  11680057
 
  ENSEMBL   UCSC 
 
  213
 
  5
 
 23153
 
 tss
 
 0.79203
 
 0.93278
 
 Not queried
 
  1.1219446
 
       NA
 
       NA
  
 
  
 16
 
  46655349
 
  46655473
 
  ENSEMBL   UCSC 
 
  125
 
  4
 
 15188
 
 tss
 
 0.00549
 
 0.01175
 
 Not queried
 
  1.1217817
 
       NA
 
       NA
  
 
  
 11
 
  55135046
 
  55135337
 
  ENSEMBL   UCSC 
 
  292
 
  4
 
 7276
 
 tss
 
 0.51429
 
 0.46940
 
 Not queried
 
  1.1217679
 
       NA
 
       NA
  
 
  
 6
 
  30159925
 
  30160141
 
  ENSEMBL   UCSC 
 
  217
 
  6
 
 33681
 
 gene
 
 0.86490
 
 0.88869
 
 Not queried
 
  1.1217492
 
       NA
 
       NA
  
 
  
 2
 
  45182153
 
  45182417
 
  ENSEMBL   UCSC 
 
  265
 
  4
 
 24228
 
 island
 
 0.38839
 
 0.16369
 
 Not queried
 
  1.1214479
 
       NA
 
       NA
  
 
  
 14
 
  75179846
 
  75180043
 
  ENSEMBL   UCSC 
 
  198
 
  6
 
 12481
 
 tss;island
 
 0.01237
 
 0.02995
 
 Not queried
 
  1.1211219
 
       NA
 
       NA
  
 
  
 20
 
  53092781
 
  53093152
 
  ENSEMBL   UCSC 
 
  372
 
  4
 
 25421
 
 gene;island
 
 0.03909
 
 0.10973
 
 Not queried
 
  1.1206412
 
       NA
 
       NA
  
 
  
 6
 
  33133329
 
  33133752
 
  ENSEMBL   UCSC 
 
  424
 
  9
 
 34130
 
 gene
 
 0.34833
 
 0.54173
 
 Not queried
 
  1.1204852
 
       NA
 
       NA
  
 
  
 1
 
  55317098
 
  55317306
 
  ENSEMBL   UCSC 
 
  209
 
  4
 
 549
 
 gene
 
 0.88854
 
 0.82284
 
 Not queried
 
  1.1203970
 
       NA
 
       NA
  
 
  
 5
 
  71014736
 
  71014918
 
  ENSEMBL   UCSC 
 
  183
 
  7
 
 32056
 
 tss;island
 
 0.23716
 
 0.56110
 
 Not queried
 
  1.1202898
 
       NA
 
       NA
  
 
  
 11
 
  66488919
 
  66489022
 
  ENSEMBL   UCSC 
 
  104
 
  4
 
 7458
 
 tss
 
 0.74950
 
 0.91065
 
 Not queried
 
  1.1201753
 
       NA
 
       NA
  
 
  
 8
 
  65292217
 
  65292498
 
  ENSEMBL   UCSC 
 
  282
 
  4
 
 40278
 
 island
 
 0.64778
 
 0.74288
 
 Not queried
 
  1.1198993
 
       NA
 
       NA
  
 
  
 1
 
 151881785
 
 151882406
 
  ENSEMBL   UCSC 
 
  622
 
  7
 
 3745
 
 island
 
 0.06919
 
 0.02796
 
 Not queried
 
  1.1198505
 
       NA
 
       NA
  
 
  
 7
 
  20816184
 
  20816695
 
  ENSEMBL   UCSC 
 
  512
 
  5
 
 38226
 
 island
 
 0.32381
 
 0.57872
 
 Not queried
 
  1.1197081
 
       NA
 
       NA
  
 
  
 7
 
  99698799
 
  99699161
 
  ENSEMBL   UCSC 
 
  363
 
  5
 
 37073
 
 gene;tss;island
 
 0.01728
 
 0.02537
 
 Not queried
 
  1.1194800
 
       NA
 
       NA
  
 
  
 6
 
   6588693
 
   6588931
 
  ENSEMBL   UCSC 
 
  239
 
  4
 
 34545
 
 tss
 
 0.67284
 
 0.72629
 
 Not queried
 
  1.1191088
 
       NA
 
       NA
  
 
  
 11
 
 115024362
 
 115024626
 
  ENSEMBL   UCSC 
 
  265
 
  4
 
 8802
 
 island
 
 0.88052
 
 0.80388
 
 Not queried
 
  1.1190361
 
       NA
 
       NA
  
 
  
 1
 
 240776162
 
 240776316
 
  ENSEMBL   UCSC 
 
  155
 
  4
 
 2344
 
 tss
 
 0.55982
 
 0.37989
 
 Not queried
 
  1.1189996
 
       NA
 
       NA
  
 
  
 X
 
  55026360
 
  55027018
 
  ENSEMBL   UCSC 
 
  659
 
  8
 
 41818
 
 island
 
 0.20172
 
 0.36964
 
 Not queried
 
  1.1188862
 
       NA
 
       NA
  
 
  
 1
 
 155290236
 
 155290961
 
  ENSEMBL   UCSC 
 
  726
 
  9
 
 801
 
 gene;island
 
 0.08756
 
 0.21898
 
 Not queried
 
  1.1188335
 
       NA
 
       NA
  
 
  
 6
 
 170571686
 
 170572016
 
  ENSEMBL   UCSC 
 
  331
 
  8
 
 35290
 
 tss
 
 0.92511
 
 0.84652
 
 Not queried
 
  1.1184676
 
       NA
 
       NA
  
 
  
 3
 
  10332460
 
  10332750
 
  ENSEMBL   UCSC 
 
  291
 
  4
 
 27616
 
 gene;tss
 
 0.76660
 
 0.88373
 
 Not queried
 
  1.1182360
 
       NA
 
       NA
  
 
  
 4
 
   4859935
 
   4860190
 
  ENSEMBL   UCSC 
 
  256
 
 10
 
 30222
 
 tss
 
 0.27300
 
 0.13242
 
 Not queried
 
  1.1182247
 
       NA
 
       NA
  
 
  
 12
 
  15942522
 
  15942969
 
  ENSEMBL   UCSC 
 
  448
 
  9
 
 9567
 
 tss;island
 
 0.04961
 
 0.01876
 
 Not queried
 
  1.1179142
 
       NA
 
       NA
  
 
  
 7
 
 158109339
 
 158109694
 
  ENSEMBL   UCSC 
 
  356
 
  4
 
 37303
 
 gene;island
 
 0.97353
 
 0.92274
 
 Not queried
 
  1.1168370
 
       NA
 
       NA
  
 
  
 2
 
 132558939
 
 132559613
 
  ENSEMBL   UCSC 
 
  675
 
  7
 
 24700
 
 island
 
 0.12560
 
 0.23150
 
 Not queried
 
  1.1166932
 
       NA
 
       NA
  
 
  
 1
 
 111888719
 
 111889159
 
  ENSEMBL   UCSC 
 
  441
 
  8
 
 1820
 
 tss
 
 0.03336
 
 0.06175
 
 Not queried
 
  1.1165832
 
       NA
 
       NA
  
 
  
 19
 
  21106002
 
  21106053
 
  ENSEMBL   UCSC 
 
   52
 
  6
 
 20621
 
 tss
 
 0.31510
 
 0.18236
 
 Not queried
 
  1.1162805
 
       NA
 
       NA
  
 
  
 22
 
  46481603
 
  46481822
 
  ENSEMBL   UCSC 
 
  220
 
  7
 
 27186
 
 tss
 
 0.73241
 
 0.86826
 
 Not queried
 
  1.1161348
 
       NA
 
       NA
  
 
  
 6
 
  33360495
 
  33360836
 
  ENSEMBL   UCSC 
 
  342
 
  5
 
 34207
 
 gene;island
 
 0.15018
 
 0.12676
 
 Not queried
 
  1.1160142
 
       NA
 
       NA
  
 
  
 17
 
   6939138
 
   6939304
 
  ENSEMBL   UCSC 
 
  167
 
  6
 
 17026
 
 tss;island
 
 0.03627
 
 0.01464
 
 Not queried
 
  1.1159389
 
       NA
 
       NA
  
 
  
 11
 
  65188784
 
  65189075
 
  ENSEMBL   UCSC 
 
  292
 
  4
 
 7407
 
 tss;island
 
 0.69816
 
 0.79430
 
 Not queried
 
  1.1158483
 
       NA
 
       NA
  
 
  
 19
 
  44331239
 
  44331454
 
  ENSEMBL   UCSC 
 
  216
 
  6
 
 20800
 
 tss
 
 0.01270
 
 0.03392
 
 Not queried
 
  1.1155980
 
       NA
 
       NA
  
 
  
 1
 
  12251718
 
  12251934
 
  ENSEMBL   UCSC 
 
  217
 
  4
 
 231
 
 gene;island
 
 0.69326
 
 0.84194
 
 Not queried
 
  1.1154356
 
       NA
 
       NA
  
 
  
 2
 
  80549579
 
  80550119
 
  ENSEMBL   UCSC 
 
  541
 
  5
 
 22720
 
 gene;island
 
 0.24565
 
 0.41368
 
 Not queried
 
  1.1154234
 
       NA
 
       NA
  
 
  
 1
 
  20959826
 
  20959926
 
  ENSEMBL   UCSC 
 
  101
 
  5
 
 1312
 
 tss
 
 0.01696
 
 0.01871
 
 Not queried
 
  1.1152286
 
       NA
 
       NA
  
 
  
 X
 
  73523856
 
  73524064
 
  ENSEMBL   UCSC 
 
  209
 
  7
 
 41875
 
 island
 
 0.88046
 
 0.92577
 
 Not queried
 
  1.1149185
 
       NA
 
       NA
  
 
  
 19
 
   5568093
 
   5568218
 
  ENSEMBL   UCSC 
 
  126
 
  5
 
 20341
 
 tss;island
 
 0.13452
 
 0.30179
 
 Not queried
 
  1.1145527
 
       NA
 
       NA
  
 
  
 20
 
  49548413
 
  49548661
 
  ENSEMBL   UCSC 
 
  249
 
  4
 
 25784
 
 tss;island
 
 0.85724
 
 0.71174
 
 Not queried
 
  1.1144990
 
       NA
 
       NA
  
 
  
 6
 
  31864071
 
  31864236
 
  ENSEMBL   UCSC 
 
  166
 
  4
 
 33927
 
 gene;island
 
 0.87742
 
 0.92813
 
 Not queried
 
  1.1143163
 
       NA
 
       NA
  
 
  
 8
 
  12989237
 
  12989686
 
  ENSEMBL   UCSC 
 
  450
 
  4
 
 39148
 
 gene;island
 
 0.03052
 
 0.06199
 
 Not queried
 
  1.1141957
 
       NA
 
       NA
  
 
  
 13
 
  38443950
 
  38444227
 
  ENSEMBL   UCSC 
 
  278
 
  6
 
 11373
 
 tss
 
 0.02661
 
 0.04836
 
 Not queried
 
  1.1140823
 
       NA
 
       NA
  
 
  
 14
 
  23451889
 
  23452111
 
  ENSEMBL   UCSC 
 
  223
 
  5
 
 12263
 
 tss
 
 0.01536
 
 0.02047
 
 Not queried
 
  1.1137934
 
       NA
 
       NA
  
 
  
 2
 
 183731169
 
 183732062
 
  ENSEMBL   UCSC 
 
  894
 
 10
 
 24902
 
 island
 
 0.04027
 
 0.06267
 
 Not queried
 
  1.1135808
 
       NA
 
       NA
  
 
  
 11
 
  64107374
 
  64107720
 
  ENSEMBL   UCSC 
 
  347
 
  5
 
 8387
 
 island
 
 0.82322
 
 0.68707
 
 Not queried
 
  1.1134873
 
       NA
 
       NA
  
 
  
 2
 
  43454447
 
  43455103
 
  ENSEMBL   UCSC 
 
  657
 
 21
 
 22621
 
 gene
 
 0.10049
 
 0.04877
 
 Not queried
 
  1.1134472
 
       NA
 
       NA
  
 
  
 14
 
  75179859
 
  75180043
 
  ENSEMBL   UCSC 
 
  185
 
  5
 
 12113
 
 gene
 
 0.01383
 
 0.03045
 
 Not queried
 
  1.1134101
 
       NA
 
       NA
  
 
  
 1
 
  44870976
 
  44871634
 
  ENSEMBL   UCSC 
 
  659
 
  7
 
 488
 
 gene
 
 0.01201
 
 0.03076
 
 Not queried
 
  1.1131902
 
       NA
 
       NA
  
 
  
 19
 
  30164820
 
  30165308
 
  ENSEMBL   UCSC 
 
  489
 
  4
 
 19933
 
 gene;island
 
 0.85209
 
 0.91500
 
 Not queried
 
  1.1131752
 
       NA
 
       NA
  
 
  
 19
 
  49646093
 
  49646246
 
  ENSEMBL   UCSC 
 
  154
 
  5
 
 20107
 
 gene;island
 
 0.04246
 
 0.08746
 
 Not queried
 
  1.1121660
 
       NA
 
       NA
  
 
  
 2
 
 237993901
 
 237994083
 
  ENSEMBL   UCSC 
 
  183
 
  4
 
 25191
 
 island
 
 0.02906
 
 0.01249
 
 Not queried
 
  1.1120507
 
       NA
 
       NA
  
 
  
 3
 
 152879629
 
 152879841
 
  ENSEMBL   UCSC 
 
  213
 
  4
 
 28623
 
 tss;island
 
 0.03778
 
 0.12088
 
 Not queried
 
  1.1119318
 
       NA
 
       NA
  
 
  
 19
 
   3730505
 
   3730768
 
  ENSEMBL   UCSC 
 
  264
 
  4
 
 19687
 
 gene;island
 
 0.95014
 
 0.84454
 
 Not queried
 
  1.1115394
 
       NA
 
       NA
  
 
  
 7
 
  97841334
 
  97841513
 
  ENSEMBL   UCSC 
 
  180
 
  5
 
 37678
 
 tss
 
 0.89924
 
 0.71386
 
 Not queried
 
  1.1114932
 
       NA
 
       NA
  
 
  
 16
 
  71264282
 
  71264545
 
  ENSEMBL   UCSC 
 
  264
 
  4
 
 14799
 
 gene
 
 0.12113
 
 0.03856
 
 Not queried
 
  1.1114440
 
       NA
 
       NA
  
 
  
 X
 
 130964695
 
 130964931
 
  ENSEMBL   UCSC 
 
  237
 
  5
 
 41523
 
 tss
 
 0.01630
 
 0.04100
 
 Not queried
 
  1.1114351
 
       NA
 
       NA
  
 
  
 1
 
 103574469
 
 103574619
 
  ENSEMBL   UCSC 
 
  151
 
  4
 
 1782
 
 tss
 
 0.07876
 
 0.03456
 
 Not queried
 
  1.1114330
 
       NA
 
       NA
  
 
  
 2
 
 183731563
 
 183732062
 
  ENSEMBL   UCSC 
 
  500
 
  7
 
 23728
 
 tss
 
 0.03982
 
 0.04349
 
 Not queried
 
  1.1112188
 
       NA
 
       NA
  
 
  
 4
 
 122854330
 
 122854532
 
  ENSEMBL   UCSC 
 
  203
 
  4
 
 30529
 
 tss
 
 0.82987
 
 0.91944
 
 Not queried
 
  1.1112038
 
       NA
 
       NA
  
 
  
 3
 
  13860477
 
  13860812
 
  ENSEMBL   UCSC 
 
  336
 
  4
 
 27642
 
 gene;island
 
 0.87960
 
 0.74883
 
 Not queried
 
  1.1110257
 
       NA
 
       NA
  
 
  
 5
 
  40755959
 
  40756512
 
  ENSEMBL   UCSC 
 
  554
 
  9
 
 32689
 
 island
 
 0.02399
 
 0.04269
 
 Not queried
 
  1.1110128
 
       NA
 
       NA
  
 
  
 14
 
 101908561
 
 101909021
 
  ENSEMBL   UCSC 
 
  461
 
  6
 
 13127
 
 island
 
 0.91605
 
 0.83589
 
 Not queried
 
  1.1108318
 
       NA
 
       NA
  
 
  
 15
 
  41056021
 
  41056369
 
  ENSEMBL   UCSC 
 
  349
 
  7
 
 14017
 
 island
 
 0.02546
 
 0.03355
 
 Not queried
 
  1.1107983
 
       NA
 
       NA
  
 
  
 2
 
 240031454
 
 240031700
 
  ENSEMBL   UCSC 
 
  247
 
  4
 
 23068
 
 gene;island
 
 0.92659
 
 0.85360
 
 Not queried
 
  1.1105700
 
       NA
 
       NA
  
 
  
 14
 
  74705942
 
  74706336
 
  ENSEMBL   UCSC 
 
  395
 
  8
 
 12968
 
 island
 
 0.54641
 
 0.34208
 
 Not queried
 
  1.1103828
 
       NA
 
       NA
  
 
  
 18
 
  37421319
 
  37421988
 
  ENSEMBL   UCSC 
 
  670
 
  5
 
 19445
 
 island
 
 0.05625
 
 0.02920
 
 Not queried
 
  1.1102940
 
       NA
 
       NA
  
 
  
 10
 
 134121413
 
 134121712
 
  ENSEMBL   UCSC 
 
  300
 
  7
 
 6280
 
 island
 
 0.02878
 
 0.02369
 
 Not queried
 
  1.1102823
 
       NA
 
       NA
  
 
  
 11
 
 115630531
 
 115631762
 
  ENSEMBL   UCSC 
 
 1232
 
 14
 
 8807
 
 island
 
 0.26043
 
 0.10056
 
 Not queried
 
  1.1101540
 
       NA
 
       NA
  
 
  
 1
 
 234743527
 
 234743830
 
  ENSEMBL   UCSC 
 
  304
 
  4
 
 1084
 
 gene;island
 
 0.06693
 
 0.13686
 
 Not queried
 
  1.1100909
 
       NA
 
       NA
  
 
  
 18
 
  55254078
 
  55254238
 
  ENSEMBL   UCSC 
 
  161
 
  6
 
 19257
 
 tss
 
 0.01276
 
 0.03515
 
 Not queried
 
  1.1100320
 
       NA
 
       NA
  
 
  
 16
 
  67282824
 
  67283238
 
  ENSEMBL   UCSC 
 
  415
 
  5
 
 15982
 
 island
 
 0.08947
 
 0.04354
 
 Not queried
 
  1.1099739
 
       NA
 
       NA
  
 
  
 X
 
 118284520
 
 118285064
 
  ENSEMBL   UCSC 
 
  545
 
 11
 
 41979
 
 island
 
 0.84992
 
 0.70735
 
 Not queried
 
  1.1099234
 
       NA
 
       NA
  
 
  
 7
 
   1498866
 
   1499391
 
  ENSEMBL   UCSC 
 
  526
 
 11
 
 38042
 
 island
 
 0.02131
 
 0.05255
 
 Not queried
 
  1.1091743
 
       NA
 
       NA
  
 
  
 1
 
  84543369
 
  84543558
 
  ENSEMBL   UCSC 
 
  190
 
  5
 
 1709
 
 tss;island
 
 0.01029
 
 0.03062
 
 Not queried
 
  1.1089171
 
       NA
 
       NA
  
 
  
 17
 
   6926003
 
   6926414
 
  ENSEMBL   UCSC 
 
  412
 
  5
 
 17883
 
 island
 
 0.54385
 
 0.75874
 
 Not queried
 
  1.1082599
 
       NA
 
       NA
  
 
  
 17
 
   2415219
 
   2415399
 
  ENSEMBL   UCSC 
 
  181
 
  6
 
 16974
 
 tss
 
 0.00778
 
 0.02176
 
 Not queried
 
  1.1079854
 
       NA
 
       NA
  
 
  
 2
 
 209271661
 
 209272057
 
  ENSEMBL   UCSC 
 
  397
 
  5
 
 22963
 
 gene
 
 0.03778
 
 0.08889
 
 Not queried
 
  1.1077508
 
       NA
 
       NA
  
 
  
 17
 
   8647944
 
   8648193
 
  ENSEMBL   UCSC 
 
  250
 
  5
 
 17974
 
 island
 
 0.92335
 
 0.74647
 
 Not queried
 
  1.1074192
 
       NA
 
       NA
  
 
  
 2
 
 176981919
 
 176982342
 
  ENSEMBL   UCSC 
 
  424
 
  4
 
 22899
 
 gene;island
 
 0.57637
 
 0.71981
 
 Not queried
 
  1.1071446
 
       NA
 
       NA
  
 
  
 5
 
  72526137
 
  72526496
 
  ENSEMBL   UCSC 
 
  360
 
  4
 
 32803
 
 island
 
 0.73348
 
 0.61458
 
 Not queried
 
  1.1065975
 
       NA
 
       NA
  
 
  
 6
 
 168122607
 
 168122963
 
  ENSEMBL   UCSC 
 
  357
 
  6
 
 36585
 
 island
 
 0.90377
 
 0.93435
 
 Not queried
 
  1.1062714
 
       NA
 
       NA
  
 
  
 19
 
  49141115
 
  49141420
 
  ENSEMBL   UCSC 
 
  306
 
  5
 
 20885
 
 tss;island
 
 0.02964
 
 0.04797
 
 Not queried
 
  1.1062224
 
       NA
 
       NA
  
 
  
 3
 
 183146663
 
 183146882
 
  ENSEMBL   UCSC 
 
  220
 
  4
 
 28703
 
 tss;island
 
 0.05409
 
 0.03098
 
 Not queried
 
  1.1060959
 
       NA
 
       NA
  
 
  
 16
 
  56965376
 
  56965804
 
  ENSEMBL   UCSC 
 
  429
 
  6
 
 15924
 
 island
 
 0.14655
 
 0.28736
 
 Not queried
 
  1.1059664
 
       NA
 
       NA
  
 
  
 2
 
  85980500
 
  85981021
 
  ENSEMBL   UCSC 
 
  522
 
 12
 
 24449
 
 island
 
 0.05027
 
 0.03139
 
 Not queried
 
  1.1058850
 
       NA
 
       NA
  
 
  
 1
 
  38461540
 
  38461896
 
  ENSEMBL   UCSC 
 
  357
 
  4
 
 3075
 
 island
 
 0.71051
 
 0.82874
 
 Not queried
 
  1.1058176
 
       NA
 
       NA
  
 
  
 7
 
  45953027
 
  45953207
 
  ENSEMBL   UCSC 
 
  181
 
  4
 
 36938
 
 gene
 
 0.81455
 
 0.85273
 
 Not queried
 
  1.1057737
 
       NA
 
       NA
  
 
  
 10
 
  21783344
 
  21783930
 
  ENSEMBL   UCSC 
 
  587
 
  6
 
 5571
 
 island
 
 0.05523
 
 0.03778
 
 Not queried
 
  1.1057662
 
       NA
 
       NA
  
 
  
 3
 
 157824148
 
 157824283
 
  ENSEMBL   UCSC 
 
  136
 
  5
 
 28639
 
 tss;island
 
 0.02396
 
 0.04347
 
 Not queried
 
  1.1056641
 
       NA
 
       NA
  
 
  
 22
 
  42949526
 
  42949946
 
  ENSEMBL   UCSC 
 
  421
 
  9
 
 27502
 
 island
 
 0.05436
 
 0.02788
 
 Not queried
 
  1.1047708
 
       NA
 
       NA
  
 
  
 8
 
    644521
 
    644878
 
  ENSEMBL   UCSC 
 
  358
 
  5
 
 39101
 
 gene
 
 0.94298
 
 0.96493
 
 Not queried
 
  1.1046733
 
       NA
 
       NA
  
 
  
 5
 
  87974369
 
  87974547
 
  ENSEMBL   UCSC 
 
  179
 
  7
 
 31623
 
 gene;tss;island
 
 0.04020
 
 0.02764
 
 Not queried
 
  1.1044715
 
       NA
 
       NA
  
 
  
 11
 
  20181725
 
  20182122
 
  ENSEMBL   UCSC 
 
  398
 
  8
 
 8103
 
 island
 
 0.11696
 
 0.15602
 
 Not queried
 
  1.1042330
 
       NA
 
       NA
  
 
  
 19
 
   2246724
 
   2246968
 
  ENSEMBL   UCSC 
 
  245
 
  4
 
 19646
 
 gene;island
 
 0.80562
 
 0.91389
 
 Not queried
 
  1.1041252
 
       NA
 
       NA
  
 
  
 17
 
  48072565
 
  48072833
 
  ENSEMBL   UCSC 
 
  269
 
  4
 
 18503
 
 island
 
 0.03600
 
 0.05345
 
 Not queried
 
  1.1041165
 
       NA
 
       NA
  
 
  
 3
 
 141496874
 
 141496998
 
  ENSEMBL   UCSC 
 
  125
 
  5
 
 28596
 
 tss
 
 0.74283
 
 0.87321
 
 Not queried
 
  1.1041105
 
       NA
 
       NA
  
 
  
 15
 
  32907173
 
  32907450
 
  ENSEMBL   UCSC 
 
  278
 
  4
 
 13523
 
 tss;island
 
 0.00836
 
 0.02377
 
 Not queried
 
  1.1040775
 
       NA
 
       NA
  
 
  
 1
 
   4715467
 
   4715615
 
  ENSEMBL   UCSC 
 
  149
 
  4
 
 151
 
 gene;island
 
 0.02726
 
 0.04892
 
 Not queried
 
  1.1040368
 
       NA
 
       NA
  
 
  
 7
 
 142490932
 
 142491168
 
  ENSEMBL   UCSC 
 
  237
 
  5
 
 38908
 
 island
 
 0.03433
 
 0.06317
 
 Not queried
 
  1.1040130
 
       NA
 
       NA
  
 
  
 11
 
   6342214
 
   6342510
 
  ENSEMBL   UCSC 
 
  297
 
  4
 
 7087
 
 tss;island
 
 0.06496
 
 0.03124
 
 Not queried
 
  1.1037436
 
       NA
 
       NA
  
 
  
 8
 
  48872801
 
  48872876
 
  ENSEMBL   UCSC 
 
   76
 
  4
 
 39625
 
 tss;island
 
 0.02214
 
 0.04248
 
 Not queried
 
  1.1035856
 
       NA
 
       NA
  
 
  
 1
 
  41832369
 
  41832649
 
  ENSEMBL   UCSC 
 
  281
 
  4
 
 3132
 
 island
 
 0.34778
 
 0.26641
 
 Not queried
 
  1.1035372
 
       NA
 
       NA
  
 
  
 X
 
 138285233
 
 138285540
 
  ENSEMBL   UCSC 
 
  308
 
  4
 
 41126
 
 gene;island
 
 0.08228
 
 0.03513
 
 Not queried
 
  1.1030682
 
       NA
 
       NA
  
 
  
 6
 
  73330106
 
  73330358
 
  ENSEMBL   UCSC 
 
  253
 
  5
 
 34999
 
 tss
 
 0.04085
 
 0.09382
 
 Not queried
 
  1.1029382
 
       NA
 
       NA
  
 
  
 18
 
  46447634
 
  46447961
 
  ENSEMBL   UCSC 
 
  328
 
  5
 
 19076
 
 gene;island
 
 0.76802
 
 0.87670
 
 Not queried
 
  1.1027839
 
       NA
 
       NA
  
 
  
 13
 
 100150711
 
 100151066
 
  ENSEMBL   UCSC 
 
  356
 
  4
 
 11809
 
 island
 
 0.02973
 
 0.05799
 
 Not queried
 
  1.1026100
 
       NA
 
       NA
  
 
  
 7
 
 154862021
 
 154862157
 
  ENSEMBL   UCSC 
 
  137
 
  4
 
 37945
 
 tss;island
 
 0.07513
 
 0.12986
 
 Not queried
 
  1.1025801
 
       NA
 
       NA
  
 
  
 11
 
  45928841
 
  45928988
 
  ENSEMBL   UCSC 
 
  148
 
  6
 
 7250
 
 tss
 
 0.60063
 
 0.75483
 
 Not queried
 
  1.1019950
 
       NA
 
       NA
  
 
  
 17
 
  32690255
 
  32690732
 
  ENSEMBL   UCSC 
 
  478
 
  5
 
 17226
 
 tss
 
 0.56100
 
 0.32195
 
 Not queried
 
  1.1018835
 
       NA
 
       NA
  
 
  
 X
 
  24711474
 
  24711994
 
  ENSEMBL   UCSC 
 
  521
 
  5
 
 41234
 
 tss
 
 0.04599
 
 0.02197
 
 Not queried
 
  1.1017811
 
       NA
 
       NA
  
 
  
 2
 
 233390712
 
 233390859
 
  ENSEMBL   UCSC 
 
  148
 
  4
 
 23912
 
 tss
 
 0.82735
 
 0.70576
 
 Not queried
 
  1.1016885
 
       NA
 
       NA
  
 
  
 19
 
  51143207
 
  51143348
 
  ENSEMBL   UCSC 
 
  142
 
  4
 
 20946
 
 tss;island
 
 0.27931
 
 0.10917
 
 Not queried
 
  1.1016707
 
       NA
 
       NA
  
 
  
 8
 
 142437128
 
 142437236
 
  ENSEMBL   UCSC 
 
  109
 
  4
 
 39390
 
 gene;island
 
 0.74034
 
 0.90952
 
 Not queried
 
  1.1013038
 
       NA
 
       NA
  
 
  
 1
 
  26232706
 
  26233035
 
  ENSEMBL   UCSC 
 
  330
 
  7
 
 334
 
 gene;tss;island
 
 0.01538
 
 0.00966
 
 Not queried
 
  1.1012854
 
       NA
 
       NA
  
 
  
 X
 
 105969622
 
 105970061
 
  ENSEMBL   UCSC 
 
  440
 
 10
 
 41070
 
 gene;island
 
 0.02618
 
 0.05067
 
 Not queried
 
  1.1011921
 
       NA
 
       NA
  
 
  
 11
 
    818752
 
    818917
 
  ENSEMBL   UCSC 
 
  166
 
  5
 
 7862
 
 island
 
 0.64428
 
 0.69268
 
 Not queried
 
  1.1008818
 
       NA
 
       NA
  
 
  
 6
 
  43484627
 
  43485109
 
  ENSEMBL   UCSC 
 
  483
 
 10
 
 34936
 
 tss;island
 
 0.04785
 
 0.07686
 
 Not queried
 
  1.1004795
 
       NA
 
       NA
  
 
  
 22
 
  46263463
 
  46263834
 
  ENSEMBL   UCSC 
 
  372
 
  4
 
 27536
 
 island
 
 0.53243
 
 0.77875
 
 Not queried
 
  1.0992256
 
       NA
 
       NA
  
 
  
 6
 
  30582174
 
  30582416
 
  ENSEMBL   UCSC 
 
  243
 
  5
 
 33722
 
 gene;island
 
 0.52892
 
 0.71177
 
 Not queried
 
  1.0991984
 
       NA
 
       NA
  
 
  
 1
 
  27994728
 
  27994978
 
  ENSEMBL   UCSC 
 
  251
 
  4
 
 356
 
 gene;island
 
 0.64234
 
 0.37801
 
 Not queried
 
  1.0991846
 
       NA
 
       NA
  
 
  
 2
 
 170440636
 
 170440819
 
  ENSEMBL   UCSC 
 
  184
 
  6
 
 23665
 
 tss
 
 0.10530
 
 0.02437
 
 Not queried
 
  1.0991141
 
       NA
 
       NA
  
 
  
 3
 
  71633826
 
  71634044
 
  ENSEMBL   UCSC 
 
  219
 
  4
 
 28399
 
 tss;island
 
 0.00663
 
 0.01552
 
 Not queried
 
  1.0990044
 
       NA
 
       NA
  
 
  
 4
 
   3371876
 
   3372272
 
  ENSEMBL   UCSC 
 
  397
 
  5
 
 29859
 
 gene;island
 
 0.90290
 
 0.75834
 
 Not queried
 
  1.0989352
 
       NA
 
       NA
  
 
  
 10
 
 135020656
 
 135021100
 
  ENSEMBL   UCSC 
 
  445
 
  5
 
 4875
 
 gene;island
 
 0.83588
 
 0.89804
 
 Not queried
 
  1.0988112
 
       NA
 
       NA
  
 
  
 6
 
  33244709
 
  33246488
 
  ENSEMBL   UCSC 
 
 1780
 
 52
 
 35933
 
 island
 
 0.65757
 
 0.29266
 
 Not queried
 
  1.0987282
 
       NA
 
       NA
  
 
  
 1
 
 221051758
 
 221052143
 
  ENSEMBL   UCSC 
 
  386
 
  4
 
 2259
 
 tss;island
 
 0.08213
 
 0.04812
 
 Not queried
 
  1.0986961
 
       NA
 
       NA
  
 
  
 1
 
  87793364
 
  87793607
 
  ENSEMBL   UCSC 
 
  244
 
  4
 
 1730
 
 tss;island
 
 0.31165
 
 0.18237
 
 Not queried
 
  1.0986378
 
       NA
 
       NA
  
 
  
 16
 
  86912525
 
  86912782
 
  ENSEMBL   UCSC 
 
  258
 
  4
 
 16143
 
 island
 
 0.85994
 
 0.73586
 
 Not queried
 
  1.0986364
 
       NA
 
       NA
  
 
  
 10
 
  88022641
 
  88023243
 
  ENSEMBL   UCSC 
 
  603
 
  7
 
 4644
 
 gene;island
 
 0.83974
 
 0.96282
 
 Not queried
 
  1.0985909
 
       NA
 
       NA
  
 
  
 X
 
  35937617
 
  35937794
 
  ENSEMBL   UCSC 
 
  178
 
  7
 
 41252
 
 tss
 
 0.01056
 
 0.05878
 
 Not queried
 
  1.0984990
 
       NA
 
       NA
  
 
  
 4
 
  53522821
 
  53522945
 
  ENSEMBL   UCSC 
 
  125
 
  4
 
 29957
 
 gene;tss;island
 
 0.90977
 
 0.94922
 
 Not queried
 
  1.0984364
 
       NA
 
       NA
  
 
  
 6
 
 170597326
 
 170597588
 
  ENSEMBL   UCSC 
 
  263
 
  4
 
 34517
 
 gene;island
 
 0.79725
 
 0.62240
 
 Not queried
 
  1.0979689
 
       NA
 
       NA
  
 
  
 1
 
 220102277
 
 220102517
 
  ENSEMBL   UCSC 
 
  241
 
  5
 
 2254
 
 tss;island
 
 0.62886
 
 0.50632
 
 Not queried
 
  1.0978411
 
       NA
 
       NA
  
 
  
 6
 
  32301073
 
  32301513
 
  ENSEMBL   UCSC 
 
  441
 
  4
 
 34041
 
 gene
 
 0.87413
 
 0.71062
 
 Not queried
 
  1.0975844
 
       NA
 
       NA
  
 
  
 20
 
  31407338
 
  31407559
 
  ENSEMBL   UCSC 
 
  222
 
  6
 
 25638
 
 tss;island
 
 0.14395
 
 0.22502
 
 Not queried
 
  1.0974330
 
       NA
 
       NA
  
 
  
 11
 
  75237526
 
  75237935
 
  ENSEMBL   UCSC 
 
  410
 
  5
 
 7548
 
 tss;island
 
 0.56503
 
 0.22494
 
 Not queried
 
  1.0973735
 
       NA
 
       NA
  
 
  
 11
 
 124735961
 
 124736135
 
  ENSEMBL   UCSC 
 
  175
 
  4
 
 6934
 
 gene;island
 
 0.04725
 
 0.06843
 
 Not queried
 
  1.0973198
 
       NA
 
       NA
  
 
  
 8
 
 143763326
 
 143763565
 
  ENSEMBL   UCSC 
 
  240
 
  4
 
 39403
 
 gene
 
 0.84129
 
 0.77156
 
 Not queried
 
  1.0972387
 
       NA
 
       NA
  
 
  
 13
 
 113663206
 
 113663538
 
  ENSEMBL   UCSC 
 
  333
 
  4
 
 11240
 
 gene;island
 
 0.86118
 
 0.92485
 
 Not queried
 
  1.0971905
 
       NA
 
       NA
  
 
  
 12
 
 123756720
 
 123757070
 
  ENSEMBL   UCSC 
 
  351
 
  7
 
 10071
 
 tss;island
 
 0.03418
 
 0.01817
 
 Not queried
 
  1.0971026
 
       NA
 
       NA
  
 
  
 1
 
 201617847
 
 201618284
 
  ENSEMBL   UCSC 
 
  438
 
  6
 
 937
 
 gene;island
 
 0.04152
 
 0.02599
 
 Not queried
 
  1.0969153
 
       NA
 
       NA
  
 
  
 6
 
 130031504
 
 130031784
 
  ENSEMBL   UCSC 
 
  281
 
  6
 
 35155
 
 tss
 
 0.00903
 
 0.02013
 
 Not queried
 
  1.0968613
 
       NA
 
       NA
  
 
  
 17
 
  42835688
 
  42836320
 
  ENSEMBL   UCSC 
 
  633
 
  5
 
 17382
 
 tss;island
 
 0.09250
 
 0.09011
 
 Not queried
 
  1.0966240
 
       NA
 
       NA
  
 
  
 X
 
  24665296
 
  24665669
 
  ENSEMBL   UCSC 
 
  374
 
  8
 
 40951
 
 gene
 
 0.04827
 
 0.02733
 
 Not queried
 
  1.0964816
 
       NA
 
       NA
  
 
  
 7
 
   4998415
 
   4998714
 
  ENSEMBL   UCSC 
 
  300
 
  4
 
 36801
 
 gene;island
 
 0.13709
 
 0.07518
 
 Not queried
 
  1.0963844
 
       NA
 
       NA
  
 
  
 17
 
  79869748
 
  79870056
 
  ENSEMBL   UCSC 
 
  309
 
  6
 
 17723
 
 tss;island
 
 0.72920
 
 0.80577
 
 Not queried
 
  1.0963704
 
       NA
 
       NA
  
 
  
 11
 
   1219189
 
   1219644
 
  ENSEMBL   UCSC 
 
  456
 
  5
 
 7884
 
 island
 
 0.52853
 
 0.72909
 
 Not queried
 
  1.0963314
 
       NA
 
       NA
  
 
  
 10
 
 134538827
 
 134539084
 
  ENSEMBL   UCSC 
 
  258
 
  4
 
 4860
 
 gene;island
 
 0.75269
 
 0.88399
 
 Not queried
 
  1.0962939
 
       NA
 
       NA
  
 
  
 3
 
  44283035
 
  44283837
 
  ENSEMBL   UCSC 
 
  803
 
 12
 
 28989
 
 island
 
 0.93605
 
 0.86721
 
 Not queried
 
  1.0961150
 
       NA
 
       NA
  
 
  
 14
 
  21493964
 
  21494269
 
  ENSEMBL   UCSC 
 
  306
 
  6
 
 12252
 
 tss;island
 
 0.09467
 
 0.04167
 
 Not queried
 
  1.0959869
 
       NA
 
       NA
  
 
  
 6
 
  31770844
 
  31771439
 
  ENSEMBL   UCSC 
 
  596
 
  6
 
 33898
 
 gene;island
 
 0.79615
 
 0.90034
 
 Not queried
 
  1.0959683
 
       NA
 
       NA
  
 
  
 13
 
 100153203
 
 100153694
 
  ENSEMBL   UCSC 
 
  492
 
  9
 
 11485
 
 tss
 
 0.00709
 
 0.01199
 
 Not queried
 
  1.0958228
 
       NA
 
       NA
  
 
  
 10
 
  45470023
 
  45470562
 
  ENSEMBL   UCSC 
 
  540
 
  6
 
 4561
 
 gene;island
 
 0.08536
 
 0.03624
 
 Not queried
 
  1.0955188
 
       NA
 
       NA
  
 
  
 17
 
  37821447
 
  37821661
 
  ENSEMBL   UCSC 
 
  215
 
  5
 
 18250
 
 island
 
 0.44057
 
 0.56894
 
 Not queried
 
  1.0954554
 
       NA
 
       NA
  
 
  
 16
 
  68481813
 
  68482277
 
  ENSEMBL   UCSC 
 
  465
 
  5
 
 14786
 
 gene;island
 
 0.13827
 
 0.50445
 
 Not queried
 
  1.0954463
 
       NA
 
       NA
  
 
  
 14
 
  96152464
 
  96152706
 
  ENSEMBL   UCSC 
 
  243
 
  5
 
 12561
 
 tss
 
 0.49068
 
 0.72521
 
 Not queried
 
  1.0950930
 
       NA
 
       NA
  
 
  
 17
 
  32905966
 
  32906622
 
  ENSEMBL   UCSC 
 
  657
 
  7
 
 18168
 
 island
 
 0.00779
 
 0.01654
 
 Not queried
 
  1.0950190
 
       NA
 
       NA
  
 
  
 15
 
  40452931
 
  40453319
 
  ENSEMBL   UCSC 
 
  389
 
 11
 
 14003
 
 island
 
 0.01658
 
 0.03097
 
 Not queried
 
  1.0949814
 
       NA
 
       NA
  
 
  
 4
 
   5053085
 
   5053596
 
  ENSEMBL   UCSC 
 
  512
 
  7
 
 30827
 
 island
 
 0.06084
 
 0.03296
 
 Not queried
 
  1.0948632
 
       NA
 
       NA
  
 
  
 X
 
  68047520
 
  68048818
 
  ENSEMBL   UCSC 
 
 1299
 
 24
 
 41359
 
 tss
 
 0.08544
 
 0.04432
 
 Not queried
 
  1.0947391
 
       NA
 
       NA
  
 
  
 17
 
   8021572
 
   8022155
 
  ENSEMBL   UCSC 
 
  584
 
  8
 
 16389
 
 gene
 
 0.02048
 
 0.03275
 
 Not queried
 
  1.0946593
 
       NA
 
       NA
  
 
  
 6
 
  31996931
 
  31997067
 
  ENSEMBL   UCSC 
 
  137
 
  4
 
 33948
 
 gene
 
 0.76679
 
 0.61315
 
 Not queried
 
  1.0943228
 
       NA
 
       NA
  
 
  
 X
 
 119738402
 
 119738603
 
  ENSEMBL   UCSC 
 
  202
 
  4
 
 41101
 
 gene
 
 0.00860
 
 0.01765
 
 Not queried
 
  1.0939029
 
       NA
 
       NA
  
 
  
 19
 
  56158714
 
  56158885
 
  ENSEMBL   UCSC 
 
  172
 
  4
 
 21051
 
 tss;island
 
 0.38392
 
 0.45765
 
 Not queried
 
  1.0938821
 
       NA
 
       NA
  
 
  
 19
 
  17357097
 
  17357641
 
  ENSEMBL   UCSC 
 
  545
 
  7
 
 20546
 
 tss;island
 
 0.04258
 
 0.04559
 
 Not queried
 
  1.0936701
 
       NA
 
       NA
  
 
  
 1
 
 154965419
 
 154965572
 
  ENSEMBL   UCSC 
 
  154
 
  4
 
 785
 
 gene;tss
 
 0.65857
 
 0.82634
 
 Not queried
 
  1.0932774
 
       NA
 
       NA
  
 
  
 15
 
  74494781
 
  74494909
 
  ENSEMBL   UCSC 
 
  129
 
  5
 
 13391
 
 gene
 
 0.10268
 
 0.04242
 
 Not queried
 
  1.0932261
 
       NA
 
       NA
  
 
  
 16
 
   3507460
 
   3507875
 
  ENSEMBL   UCSC 
 
  416
 
  7
 
 15026
 
 tss
 
 0.03597
 
 0.10482
 
 Not queried
 
  1.0930749
 
       NA
 
       NA
  
 
  
 11
 
 117747822
 
 117748236
 
  ENSEMBL   UCSC 
 
  415
 
  9
 
 7701
 
 tss
 
 0.38398
 
 0.08412
 
 Not queried
 
  1.0930650
 
       NA
 
       NA
  
 
  
 19
 
  45417526
 
  45417814
 
  ENSEMBL   UCSC 
 
  289
 
  5
 
 20820
 
 tss
 
 0.52738
 
 0.48815
 
 Not queried
 
  1.0926030
 
       NA
 
       NA
  
 
  
 7
 
  87104724
 
  87105416
 
  ENSEMBL   UCSC 
 
  693
 
  8
 
 38571
 
 island
 
 0.05372
 
 0.03314
 
 Not queried
 
  1.0923432
 
       NA
 
       NA
  
 
  
 6
 
  31927895
 
  31928257
 
  ENSEMBL   UCSC 
 
  363
 
  5
 
 34791
 
 tss
 
 0.67614
 
 0.89359
 
 Not queried
 
  1.0919232
 
       NA
 
       NA
  
 
  
 14
 
  91580129
 
  91580613
 
  ENSEMBL   UCSC 
 
  485
 
  9
 
 12530
 
 tss
 
 0.05172
 
 0.02630
 
 Not queried
 
  1.0915581
 
       NA
 
       NA
  
 
  
 19
 
  49109809
 
  49109961
 
  ENSEMBL   UCSC 
 
  153
 
  4
 
 20094
 
 gene;tss;island
 
 0.73270
 
 0.86904
 
 Not queried
 
  1.0915223
 
       NA
 
       NA
  
 
  
 3
 
 157217187
 
 157217842
 
  ENSEMBL   UCSC 
 
  656
 
 11
 
 27966
 
 gene
 
 0.03346
 
 0.08616
 
 Not queried
 
  1.0913438
 
       NA
 
       NA
  
 
  
 10
 
 134188633
 
 134188873
 
  ENSEMBL   UCSC 
 
  241
 
  5
 
 4846
 
 gene
 
 0.90848
 
 0.93863
 
 Not queried
 
  1.0912918
 
       NA
 
       NA
  
 
  
 5
 
  72415687
 
  72416275
 
  ENSEMBL   UCSC 
 
  589
 
  9
 
 32061
 
 tss
 
 0.51622
 
 0.27194
 
 Not queried
 
  1.0911436
 
       NA
 
       NA
  
 
  
 22
 
  22020380
 
  22020749
 
  ENSEMBL   UCSC 
 
  370
 
  4
 
 26764
 
 gene
 
 0.01862
 
 0.01610
 
 Not queried
 
  1.0909700
 
       NA
 
       NA
  
 
  
 1
 
  36042986
 
  36043084
 
  ENSEMBL   UCSC 
 
   99
 
  4
 
 421
 
 gene;island
 
 0.58300
 
 0.74572
 
 Not queried
 
  1.0907474
 
       NA
 
       NA
  
 
  
 1
 
  55504649
 
  55505189
 
  ENSEMBL   UCSC 
 
  541
 
 10
 
 1648
 
 tss
 
 0.54408
 
 0.72406
 
 Not queried
 
  1.0905803
 
       NA
 
       NA
  
 
  
 3
 
 154146398
 
 154146871
 
  ENSEMBL   UCSC 
 
  474
 
  5
 
 27960
 
 gene;island
 
 0.06998
 
 0.03691
 
 Not queried
 
  1.0904930
 
       NA
 
       NA
  
 
  
 20
 
   3140367
 
   3140493
 
  ENSEMBL   UCSC 
 
  127
 
  4
 
 25305
 
 gene
 
 0.01250
 
 0.02633
 
 Not queried
 
  1.0903249
 
       NA
 
       NA
  
 
  
 10
 
 134698483
 
 134698937
 
  ENSEMBL   UCSC 
 
  455
 
  4
 
 6297
 
 island
 
 0.63154
 
 0.81552
 
 Not queried
 
  1.0902869
 
       NA
 
       NA
  
 
  
 4
 
   8207058
 
   8207325
 
  ENSEMBL   UCSC 
 
  268
 
  4
 
 29904
 
 gene
 
 0.80702
 
 0.89015
 
 Not queried
 
  1.0900669
 
       NA
 
       NA
  
 
  
 1
 
  32714023
 
  32714308
 
  ENSEMBL   UCSC 
 
  286
 
  5
 
 391
 
 gene;island
 
 0.19606
 
 0.03634
 
 Not queried
 
  1.0897788
 
       NA
 
       NA
  
 
  
 17
 
  29719023
 
  29719431
 
  ENSEMBL   UCSC 
 
  409
 
  5
 
 16474
 
 gene;island
 
 0.50662
 
 0.22952
 
 Not queried
 
  1.0897015
 
       NA
 
       NA
  
 
  
 2
 
  37035539
 
  37035865
 
  ENSEMBL   UCSC 
 
  327
 
  5
 
 22608
 
 gene;island
 
 0.89728
 
 0.85219
 
 Not queried
 
  1.0895623
 
       NA
 
       NA
  
 
  
 22
 
  24115096
 
  24115568
 
  ENSEMBL   UCSC 
 
  473
 
  4
 
 26769
 
 gene
 
 0.08372
 
 0.11866
 
 Not queried
 
  1.0894420
 
       NA
 
       NA
  
 
  
 13
 
  38443634
 
  38444227
 
  ENSEMBL   UCSC 
 
  594
 
  9
 
 11649
 
 island
 
 0.02315
 
 0.04611
 
 Not queried
 
  1.0891784
 
       NA
 
       NA
  
 
  
 10
 
  48416781
 
  48416977
 
  ENSEMBL   UCSC 
 
  197
 
  7
 
 5705
 
 island
 
 0.82142
 
 0.90389
 
 Not queried
 
  1.0891595
 
       NA
 
       NA
  
 
  
 3
 
  88198982
 
  88199395
 
  ENSEMBL   UCSC 
 
  414
 
  5
 
 27825
 
 gene
 
 0.01140
 
 0.02368
 
 Not queried
 
  1.0889756
 
       NA
 
       NA
  
 
  
 6
 
 170101828
 
 170102495
 
  ENSEMBL   UCSC 
 
  668
 
  8
 
 35287
 
 tss;island
 
 0.01768
 
 0.01288
 
 Not queried
 
  1.0888296
 
       NA
 
       NA
  
 
  
 7
 
  77428232
 
  77428337
 
  ENSEMBL   UCSC 
 
  106
 
  4
 
 36998
 
 gene
 
 0.00752
 
 0.00408
 
 Not queried
 
  1.0887897
 
       NA
 
       NA
  
 
  
 19
 
  38810162
 
  38810538
 
  ENSEMBL   UCSC 
 
  377
 
  9
 
 21936
 
 island
 
 0.01681
 
 0.03255
 
 Not queried
 
  1.0886274
 
       NA
 
       NA
  
 
  
 6
 
  31549853
 
  31550241
 
  ENSEMBL   UCSC 
 
  389
 
  4
 
 35728
 
 island
 
 0.70623
 
 0.54140
 
 Not queried
 
  1.0877224
 
       NA
 
       NA
  
 
  
 7
 
 112120730
 
 112121036
 
  ENSEMBL   UCSC 
 
  307
 
  7
 
 37777
 
 tss
 
 0.85155
 
 0.93136
 
 Not queried
 
  1.0870547
 
       NA
 
       NA
  
 
  
 2
 
 137748366
 
 137748460
 
  ENSEMBL   UCSC 
 
   95
 
  4
 
 23613
 
 tss
 
 0.64990
 
 0.73998
 
 Not queried
 
  1.0870448
 
       NA
 
       NA
  
 
  
 6
 
  88182297
 
  88182594
 
  ENSEMBL   UCSC 
 
  298
 
  7
 
 35046
 
 tss;island
 
 0.01593
 
 0.03129
 
 Not queried
 
  1.0869682
 
       NA
 
       NA
  
 
  
 16
 
  67062687
 
  67062970
 
  ENSEMBL   UCSC 
 
  284
 
  7
 
 15259
 
 tss;island
 
 0.03726
 
 0.06740
 
 Not queried
 
  1.0867448
 
       NA
 
       NA
  
 
  
 11
 
   1431735
 
   1432205
 
  ENSEMBL   UCSC 
 
  471
 
  4
 
 6400
 
 gene;island
 
 0.67429
 
 0.81765
 
 Not queried
 
  1.0865178
 
       NA
 
       NA
  
 
  
 1
 
  28907478
 
  28907903
 
  ENSEMBL   UCSC 
 
  426
 
  6
 
 368
 
 gene;tss;island
 
 0.01429
 
 0.02542
 
 Not queried
 
  1.0863862
 
       NA
 
       NA
  
 
  
 X
 
 133370784
 
 133371084
 
  ENSEMBL   UCSC 
 
  301
 
  7
 
 42037
 
 island
 
 0.23702
 
 0.06106
 
 Not queried
 
  1.0863822
 
       NA
 
       NA
  
 
  
 13
 
 100258524
 
 100258954
 
  ENSEMBL   UCSC 
 
  431
 
  6
 
 11811
 
 island
 
 0.02762
 
 0.02401
 
 Not queried
 
  1.0862891
 
       NA
 
       NA
  
 
  
 10
 
  44500693
 
  44501318
 
  ENSEMBL   UCSC 
 
  626
 
  6
 
 5686
 
 island
 
 0.90448
 
 0.77148
 
 Not queried
 
  1.0862318
 
       NA
 
       NA
  
 
  
 6
 
  33422380
 
  33423398
 
  ENSEMBL   UCSC 
 
 1019
 
 29
 
 34229
 
 gene
 
 0.03075
 
 0.04363
 
 Not queried
 
  1.0861906
 
       NA
 
       NA
  
 
  
 14
 
  58862146
 
  58862503
 
  ENSEMBL   UCSC 
 
  358
 
  6
 
 12397
 
 tss;island
 
 0.07131
 
 0.04589
 
 Not queried
 
  1.0860789
 
       NA
 
       NA
  
 
  
 14
 
  24505537
 
  24505898
 
  ENSEMBL   UCSC 
 
  362
 
  7
 
 12696
 
 island
 
 0.03714
 
 0.08085
 
 Not queried
 
  1.0860636
 
       NA
 
       NA
  
 
  
 15
 
  28341500
 
  28341980
 
  ENSEMBL   UCSC 
 
  481
 
  5
 
 13236
 
 gene;island
 
 0.02645
 
 0.02375
 
 Not queried
 
  1.0860135
 
       NA
 
       NA
  
 
  
 8
 
 142138543
 
 142138643
 
  ENSEMBL   UCSC 
 
  101
 
  5
 
 39863
 
 tss;island
 
 0.09413
 
 0.14062
 
 Not queried
 
  1.0857203
 
       NA
 
       NA
  
 
  
 14
 
  23291749
 
  23292277
 
  ENSEMBL   UCSC 
 
  529
 
  5
 
 12673
 
 island
 
 0.05558
 
 0.11104
 
 Not queried
 
  1.0856385
 
       NA
 
       NA
  
 
  
 17
 
  40333360
 
  40333549
 
  ENSEMBL   UCSC 
 
  190
 
  8
 
 17323
 
 tss;island
 
 0.03697
 
 0.10804
 
 Not queried
 
  1.0856382
 
       NA
 
       NA
  
 
  
 1
 
 246887303
 
 246887464
 
  ENSEMBL   UCSC 
 
  162
 
  4
 
 4395
 
 island
 
 0.08704
 
 0.05654
 
 Not queried
 
  1.0856165
 
       NA
 
       NA
  
 
  
 1
 
  34631324
 
  34631613
 
  ENSEMBL   UCSC 
 
  290
 
  4
 
 1472
 
 tss;island
 
 0.16727
 
 0.28741
 
 Not queried
 
  1.0851875
 
       NA
 
       NA
  
 
  
 1
 
  36184717
 
  36184863
 
  ENSEMBL   UCSC 
 
  147
 
  4
 
 3037
 
 island
 
 0.17186
 
 0.29450
 
 Not queried
 
  1.0851504
 
       NA
 
       NA
  
 
  
 17
 
  38935846
 
  38936092
 
  ENSEMBL   UCSC 
 
  247
 
  4
 
 16532
 
 gene;island
 
 0.11889
 
 0.22224
 
 Not queried
 
  1.0850407
 
       NA
 
       NA
  
 
  
 16
 
   1662131
 
   1662444
 
  ENSEMBL   UCSC 
 
  314
 
  7
 
 14967
 
 tss
 
 0.01358
 
 0.02727
 
 Not queried
 
  1.0850227
 
       NA
 
       NA
  
 
  
 20
 
  44650068
 
  44650270
 
  ENSEMBL   UCSC 
 
  203
 
  6
 
 25747
 
 tss
 
 0.06445
 
 0.04458
 
 Not queried
 
  1.0846799
 
       NA
 
       NA
  
 
  
 16
 
  56642024
 
  56642500
 
  ENSEMBL   UCSC 
 
  477
 
  6
 
 15915
 
 island
 
 0.05331
 
 0.09716
 
 Not queried
 
  1.0846756
 
       NA
 
       NA
  
 
  
 1
 
 241587493
 
 241587783
 
  ENSEMBL   UCSC 
 
  291
 
  4
 
 4364
 
 island
 
 0.23717
 
 0.10962
 
 Not queried
 
  1.0845364
 
       NA
 
       NA
  
 
  
 20
 
  50159346
 
  50159653
 
  ENSEMBL   UCSC 
 
  308
 
  4
 
 25786
 
 tss
 
 0.07721
 
 0.03786
 
 Not queried
 
  1.0840702
 
       NA
 
       NA
  
 
  
 2
 
  26569718
 
  26570144
 
  ENSEMBL   UCSC 
 
  427
 
  8
 
 23204
 
 tss
 
 0.01039
 
 0.02251
 
 Not queried
 
  1.0838636
 
       NA
 
       NA
  
 
  
 10
 
  15761192
 
  15761657
 
  ENSEMBL   UCSC 
 
  466
 
  4
 
 4501
 
 gene
 
 0.04569
 
 0.09082
 
 Not queried
 
  1.0838380
 
       NA
 
       NA
  
 
  
 X
 
 152711731
 
 152712221
 
  ENSEMBL   UCSC 
 
  491
 
  9
 
 42110
 
 island
 
 0.77648
 
 0.83495
 
 Not queried
 
  1.0836217
 
       NA
 
       NA
  
 
  
 17
 
  35303285
 
  35303488
 
  ENSEMBL   UCSC 
 
  204
 
  4
 
 18206
 
 island
 
 0.23843
 
 0.09828
 
 Not queried
 
  1.0835540
 
       NA
 
       NA
  
 
  
 3
 
  50243021
 
  50243323
 
  ENSEMBL   UCSC 
 
  303
 
  4
 
 27753
 
 gene;island
 
 0.01991
 
 0.06192
 
 Not queried
 
  1.0827026
 
       NA
 
       NA
  
 
  
 1
 
   7913667
 
   7914143
 
  ENSEMBL   UCSC 
 
  477
 
  5
 
 1215
 
 tss
 
 0.62199
 
 0.85433
 
 Not queried
 
  1.0826071
 
       NA
 
       NA
  
 
  
 6
 
 160220921
 
 160221261
 
  ENSEMBL   UCSC 
 
  341
 
  8
 
 35260
 
 tss
 
 0.95068
 
 0.91931
 
 Not queried
 
  1.0825377
 
       NA
 
       NA
  
 
  
 8
 
 103251433
 
 103251664
 
  ENSEMBL   UCSC 
 
  232
 
  5
 
 39787
 
 tss;island
 
 0.04237
 
 0.06064
 
 Not queried
 
  1.0822863
 
       NA
 
       NA
  
 
  
 11
 
  70508022
 
  70508659
 
  ENSEMBL   UCSC 
 
  638
 
 10
 
 7508
 
 tss
 
 0.06366
 
 0.11368
 
 Not queried
 
  1.0822127
 
       NA
 
       NA
  
 
  
 4
 
 154265415
 
 154265635
 
  ENSEMBL   UCSC 
 
  221
 
  5
 
 30593
 
 tss;island
 
 0.01247
 
 0.02718
 
 Not queried
 
  1.0821568
 
       NA
 
       NA
  
 
  
 5
 
 159827164
 
 159827503
 
  ENSEMBL   UCSC 
 
  340
 
  4
 
 32409
 
 tss
 
 0.02510
 
 0.05451
 
 Not queried
 
  1.0820595
 
       NA
 
       NA
  
 
  
 1
 
 182573596
 
 182573989
 
  ENSEMBL   UCSC 
 
  394
 
  7
 
 2128
 
 tss
 
 0.16222
 
 0.07829
 
 Not queried
 
  1.0820517
 
       NA
 
       NA
  
 
  
 1
 
 110754087
 
 110754436
 
  ENSEMBL   UCSC 
 
  350
 
  4
 
 673
 
 gene
 
 0.34344
 
 0.20553
 
 Not queried
 
  1.0819535
 
       NA
 
       NA
  
 
  
 X
 
   8769404
 
   8769701
 
  ENSEMBL   UCSC 
 
  298
 
 10
 
 41175
 
 tss
 
 0.83478
 
 0.90000
 
 Not queried
 
  1.0818457
 
       NA
 
       NA
  
 
  
 X
 
   8769269
 
   8769701
 
  ENSEMBL   UCSC 
 
  433
 
 12
 
 41625
 
 island
 
 0.88071
 
 0.90295
 
 Not queried
 
  1.0818457
 
       NA
 
       NA
  
 
  
 19
 
   1241377
 
   1241627
 
  ENSEMBL   UCSC 
 
  251
 
  5
 
 20258
 
 tss
 
 0.01833
 
 0.03206
 
 Not queried
 
  1.0817810
 
       NA
 
       NA
  
 
  
 17
 
  53800041
 
  53800554
 
  ENSEMBL   UCSC 
 
  514
 
  5
 
 16666
 
 gene
 
 0.05348
 
 0.12596
 
 Not queried
 
  1.0816122
 
       NA
 
       NA
  
 
  
 10
 
  23460245
 
  23460646
 
  ENSEMBL   UCSC 
 
  402
 
  4
 
 5589
 
 island
 
 0.23843
 
 0.43203
 
 Not queried
 
  1.0813646
 
       NA
 
       NA
  
 
  
 22
 
  38713874
 
  38714466
 
  ENSEMBL   UCSC 
 
  593
 
 10
 
 27095
 
 tss;island
 
 0.06298
 
 0.09479
 
 Not queried
 
  1.0806904
 
       NA
 
       NA
  
 
  
 16
 
  10970801
 
  10971250
 
  ENSEMBL   UCSC 
 
  450
 
  5
 
 15665
 
 island
 
 0.39308
 
 0.51640
 
 Not queried
 
  1.0805510
 
       NA
 
       NA
  
 
  
 17
 
  72983622
 
  72983826
 
  ENSEMBL   UCSC 
 
  205
 
  5
 
 18699
 
 island
 
 0.01554
 
 0.02859
 
 Not queried
 
  1.0805110
 
       NA
 
       NA
  
 
  
 6
 
 139012860
 
 139013146
 
  ENSEMBL   UCSC 
 
  287
 
  4
 
 36453
 
 island
 
 0.02525
 
 0.04667
 
 Not queried
 
  1.0804358
 
       NA
 
       NA
  
 
  
 1
 
  38157567
 
  38158195
 
  ENSEMBL   UCSC 
 
  629
 
  9
 
 3066
 
 island
 
 0.01093
 
 0.02112
 
 Not queried
 
  1.0802887
 
       NA
 
       NA
  
 
  
 18
 
   8367314
 
   8367691
 
  ENSEMBL   UCSC 
 
  378
 
  5
 
 19020
 
 gene;tss;island
 
 0.02831
 
 0.04933
 
 Not queried
 
  1.0802065
 
       NA
 
       NA
  
 
  
 17
 
  76836740
 
  76836835
 
  ENSEMBL   UCSC 
 
   96
 
  4
 
 16792
 
 gene
 
 0.02213
 
 0.02889
 
 Not queried
 
  1.0798424
 
       NA
 
       NA
  
 
  
 6
 
  33561181
 
  33561449
 
  ENSEMBL   UCSC 
 
  269
 
  7
 
 34846
 
 tss
 
 0.29301
 
 0.46696
 
 Not queried
 
  1.0797534
 
       NA
 
       NA
  
 
  
 6
 
  33560953
 
  33561449
 
  ENSEMBL   UCSC 
 
  497
 
  9
 
 35980
 
 island
 
 0.29301
 
 0.49977
 
 Not queried
 
  1.0797534
 
       NA
 
       NA
  
 
  
 3
 
 195943444
 
 195943713
 
  ENSEMBL   UCSC 
 
  270
 
  4
 
 28056
 
 gene
 
 0.69062
 
 0.76039
 
 Not queried
 
  1.0797404
 
       NA
 
       NA
  
 
  
 14
 
  93582398
 
  93582779
 
  ENSEMBL   UCSC 
 
  382
 
  7
 
 12542
 
 tss;island
 
 0.04781
 
 0.03874
 
 Not queried
 
  1.0796850
 
       NA
 
       NA
  
 
  
 20
 
  32274191
 
  32274504
 
  ENSEMBL   UCSC 
 
  314
 
  5
 
 26061
 
 island
 
 0.01750
 
 0.01494
 
 Not queried
 
  1.0796660
 
       NA
 
       NA
  
 
  
 1
 
  34175169
 
  34175343
 
  ENSEMBL   UCSC 
 
  175
 
  4
 
 406
 
 gene
 
 0.62598
 
 0.52580
 
 Not queried
 
  1.0795942
 
       NA
 
       NA
  
 
  
 1
 
 180991969
 
 180992117
 
  ENSEMBL   UCSC 
 
  149
 
  5
 
 4000
 
 island
 
 0.01077
 
 0.02697
 
 Not queried
 
  1.0794422
 
       NA
 
       NA
  
 
  
 10
 
   1256339
 
   1256651
 
  ENSEMBL   UCSC 
 
  313
 
  6
 
 4446
 
 gene
 
 0.46758
 
 0.76817
 
 Not queried
 
  1.0793209
 
       NA
 
       NA
  
 
  
 1
 
 203595048
 
 203595283
 
  ENSEMBL   UCSC 
 
  236
 
  4
 
 2190
 
 tss;island
 
 0.68870
 
 0.79961
 
 Not queried
 
  1.0792801
 
       NA
 
       NA
  
 
  
 1
 
  31191959
 
  31192337
 
  ENSEMBL   UCSC 
 
  379
 
  7
 
 378
 
 gene;island
 
 0.03206
 
 0.06197
 
 Not queried
 
  1.0786491
 
       NA
 
       NA
  
 
  
 13
 
 114111864
 
 114112218
 
  ENSEMBL   UCSC 
 
  355
 
  6
 
 11259
 
 gene
 
 0.83058
 
 0.93502
 
 Not queried
 
  1.0784693
 
       NA
 
       NA
  
 
  
 15
 
  25437871
 
  25438443
 
  ENSEMBL   UCSC 
 
  573
 
  6
 
 13504
 
 tss
 
 0.63472
 
 0.80049
 
 Not queried
 
  1.0780754
 
       NA
 
       NA
  
 
  
 16
 
  88851914
 
  88851969
 
  ENSEMBL   UCSC 
 
   56
 
  4
 
 15384
 
 tss;island
 
 0.01777
 
 0.04213
 
 Not queried
 
  1.0778206
 
       NA
 
       NA
  
 
  
 6
 
 170764589
 
 170764764
 
  ENSEMBL   UCSC 
 
  176
 
  4
 
 36633
 
 island
 
 0.91001
 
 0.82857
 
 Not queried
 
  1.0776779
 
       NA
 
       NA
  
 
  
 5
 
  72415687
 
  72416469
 
  ENSEMBL   UCSC 
 
  783
 
 10
 
 32802
 
 island
 
 0.47156
 
 0.26731
 
 Not queried
 
  1.0775871
 
       NA
 
       NA
  
 
  
 16
 
  67918484
 
  67918754
 
  ENSEMBL   UCSC 
 
  271
 
  5
 
 15284
 
 tss
 
 0.40031
 
 0.58723
 
 Not queried
 
  1.0774304
 
       NA
 
       NA
  
 
  
 6
 
  33378912
 
  33379330
 
  ENSEMBL   UCSC 
 
  419
 
 10
 
 34213
 
 gene;island
 
 0.04863
 
 0.02400
 
 Not queried
 
  1.0773795
 
       NA
 
       NA
  
 
  
 4
 
  40817953
 
  40818214
 
  ENSEMBL   UCSC 
 
  262
 
  4
 
 29944
 
 gene
 
 0.91694
 
 0.95133
 
 Not queried
 
  1.0764949
 
       NA
 
       NA
  
 
  
 7
 
   1370343
 
   1370727
 
  ENSEMBL   UCSC 
 
  385
 
  4
 
 38035
 
 island
 
 0.80356
 
 0.87945
 
 Not queried
 
  1.0761095
 
       NA
 
       NA
  
 
  
 14
 
  78227565
 
  78227771
 
  ENSEMBL   UCSC 
 
  207
 
  8
 
 12130
 
 gene;tss;island
 
 0.00991
 
 0.01930
 
 Not queried
 
  1.0761068
 
       NA
 
       NA
  
 
  
 15
 
  83477608
 
  83477965
 
  ENSEMBL   UCSC 
 
  358
 
  5
 
 13832
 
 tss;island
 
 0.08485
 
 0.07898
 
 Not queried
 
  1.0761061
 
       NA
 
       NA
  
 
  
 7
 
 112758734
 
 112758954
 
  ENSEMBL   UCSC 
 
  221
 
  6
 
 37781
 
 tss
 
 0.02255
 
 0.01689
 
 Not queried
 
  1.0760957
 
       NA
 
       NA
  
 
  
 5
 
  32585604
 
  32585895
 
  ENSEMBL   UCSC 
 
  292
 
  4
 
 31522
 
 gene
 
 0.01203
 
 0.00478
 
 Not queried
 
  1.0759019
 
       NA
 
       NA
  
 
  
 5
 
  32585221
 
  32585895
 
  ENSEMBL   UCSC 
 
  675
 
 10
 
 32656
 
 island
 
 0.01359
 
 0.01734
 
 Not queried
 
  1.0759019
 
       NA
 
       NA
  
 
  
 14
 
  56232650
 
  56233178
 
  ENSEMBL   UCSC 
 
  529
 
  7
 
 12843
 
 island
 
 0.83027
 
 0.91604
 
 Not queried
 
  1.0758710
 
       NA
 
       NA
  
 
  
 8
 
  52321814
 
  52322341
 
  ENSEMBL   UCSC 
 
  528
 
  7
 
 39240
 
 gene;island
 
 0.86361
 
 0.93447
 
 Not queried
 
  1.0756503
 
       NA
 
       NA
  
 
  
 2
 
  95787883
 
  95788279
 
  ENSEMBL   UCSC 
 
  397
 
  7
 
 23469
 
 tss;island
 
 0.01028
 
 0.01680
 
 Not queried
 
  1.0752493
 
       NA
 
       NA
  
 
  
 2
 
 234763269
 
 234763532
 
  ENSEMBL   UCSC 
 
  264
 
  9
 
 23922
 
 tss
 
 0.02664
 
 0.03943
 
 Not queried
 
  1.0752044
 
       NA
 
       NA
  
 
  
 20
 
  23066999
 
  23067159
 
  ENSEMBL   UCSC 
 
  161
 
  6
 
 25598
 
 tss
 
 0.56653
 
 0.74893
 
 Not queried
 
  1.0751630
 
       NA
 
       NA
  
 
  
 1
 
  26699448
 
  26699617
 
  ENSEMBL   UCSC 
 
  170
 
  4
 
 1386
 
 tss
 
 0.70290
 
 0.60068
 
 Not queried
 
  1.0751180
 
       NA
 
       NA
  
 
  
 4
 
   4859772
 
   4860190
 
  ENSEMBL   UCSC 
 
  419
 
 11
 
 30817
 
 island
 
 0.24409
 
 0.17370
 
 Not queried
 
  1.0750420
 
       NA
 
       NA
  
 
  
 19
 
  57901022
 
  57901154
 
  ENSEMBL   UCSC 
 
  133
 
  4
 
 21076
 
 tss
 
 0.02380
 
 0.06385
 
 Not queried
 
  1.0747848
 
       NA
 
       NA
  
 
  
 22
 
  24114690
 
  24115568
 
  ENSEMBL   UCSC 
 
  879
 
 11
 
 27311
 
 island
 
 0.06518
 
 0.07904
 
 Not queried
 
  1.0744338
 
       NA
 
       NA
  
 
  
 19
 
  18632949
 
  18633063
 
  ENSEMBL   UCSC 
 
  115
 
  5
 
 20584
 
 tss
 
 0.00787
 
 0.01644
 
 Not queried
 
  1.0743300
 
       NA
 
       NA
  
 
  
 15
 
  34610829
 
  34611069
 
  ENSEMBL   UCSC 
 
  241
 
  4
 
 13248
 
 gene
 
 0.00263
 
 0.00923
 
 Not queried
 
  1.0743194
 
       NA
 
       NA
  
 
  
 6
 
  87647370
 
  87647644
 
  ENSEMBL   UCSC 
 
  275
 
  5
 
 34343
 
 gene;island
 
 0.02890
 
 0.08392
 
 Not queried
 
  1.0743179
 
       NA
 
       NA
  
 
  
 21
 
  47918542
 
  47918727
 
  ENSEMBL   UCSC 
 
  186
 
  4
 
 26439
 
 gene;island
 
 0.93134
 
 0.94942
 
 Not queried
 
  1.0741543
 
       NA
 
       NA
  
 
  
 12
 
 110888114
 
 110888378
 
  ENSEMBL   UCSC 
 
  265
 
  6
 
 10768
 
 island
 
 0.03969
 
 0.05802
 
 Not queried
 
  1.0741170
 
       NA
 
       NA
  
 
  
 12
 
  54392734
 
  54393725
 
  ENSEMBL   UCSC 
 
  992
 
 16
 
 9726
 
 tss;island
 
 0.02052
 
 0.11507
 
 Not queried
 
  1.0740199
 
       NA
 
       NA
  
 
  
 3
 
  44666362
 
  44666508
 
  ENSEMBL   UCSC 
 
  147
 
  5
 
 28216
 
 tss
 
 0.05999
 
 0.02942
 
 Not queried
 
  1.0738956
 
       NA
 
       NA
  
 
  
 15
 
  83951663
 
  83952019
 
  ENSEMBL   UCSC 
 
  357
 
  4
 
 13429
 
 gene;island
 
 0.15701
 
 0.08758
 
 Not queried
 
  1.0736371
 
       NA
 
       NA
  
 
  
 16
 
  49732224
 
  49732623
 
  ENSEMBL   UCSC 
 
  400
 
  4
 
 14715
 
 gene;island
 
 0.91436
 
 0.73679
 
 Not queried
 
  1.0736216
 
       NA
 
       NA
  
 
  
 16
 
  89202817
 
  89203153
 
  ENSEMBL   UCSC 
 
  337
 
  4
 
 14902
 
 gene
 
 0.82881
 
 0.90232
 
 Not queried
 
  1.0731404
 
       NA
 
       NA
  
 
  
 5
 
 154026930
 
 154027256
 
  ENSEMBL   UCSC 
 
  327
 
  4
 
 33271
 
 island
 
 0.75823
 
 0.90620
 
 Not queried
 
  1.0730649
 
       NA
 
       NA
  
 
  
 5
 
  33936171
 
  33936752
 
  ENSEMBL   UCSC 
 
  582
 
 13
 
 32661
 
 island
 
 0.02366
 
 0.05264
 
 Not queried
 
  1.0729445
 
       NA
 
       NA
  
 
  
 2
 
 220436894
 
 220436948
 
  ENSEMBL   UCSC 
 
   55
 
  4
 
 23872
 
 tss;island
 
 0.43774
 
 0.62398
 
 Not queried
 
  1.0729394
 
       NA
 
       NA
  
 
  
 6
 
  15517209
 
  15517467
 
  ENSEMBL   UCSC 
 
  259
 
  4
 
 33518
 
 gene;island
 
 0.85562
 
 0.93735
 
 Not queried
 
  1.0727103
 
       NA
 
       NA
  
 
  
 11
 
 118842126
 
 118842650
 
  ENSEMBL   UCSC 
 
  525
 
 10
 
 8840
 
 island
 
 0.95711
 
 0.91296
 
 Not queried
 
  1.0726294
 
       NA
 
       NA
  
 
  
 19
 
  10230499
 
  10230727
 
  ENSEMBL   UCSC 
 
  229
 
  4
 
 21485
 
 island
 
 0.03480
 
 0.06607
 
 Not queried
 
  1.0725142
 
       NA
 
       NA
  
 
  
 4
 
  25235369
 
  25235616
 
  ENSEMBL   UCSC 
 
  248
 
  7
 
 30285
 
 tss
 
 0.03436
 
 0.02872
 
 Not queried
 
  1.0723842
 
       NA
 
       NA
  
 
  
 22
 
  46450059
 
  46450114
 
  ENSEMBL   UCSC 
 
   56
 
  4
 
 27185
 
 tss
 
 0.76951
 
 0.88656
 
 Not queried
 
  1.0722800
 
       NA
 
       NA
  
 
  
 13
 
  88323266
 
  88323607
 
  ENSEMBL   UCSC 
 
  342
 
  5
 
 11778
 
 island
 
 0.02966
 
 0.03740
 
 Not queried
 
  1.0720789
 
       NA
 
       NA
  
 
  
 7
 
 142494148
 
 142495098
 
  ENSEMBL   UCSC 
 
  951
 
 15
 
 38909
 
 island
 
 0.11035
 
 0.22327
 
 Not queried
 
  1.0715736
 
       NA
 
       NA
  
 
  
 7
 
 150147988
 
 150148073
 
  ENSEMBL   UCSC 
 
   86
 
  4
 
 37211
 
 gene
 
 0.55004
 
 0.69438
 
 Not queried
 
  1.0715722
 
       NA
 
       NA
  
 
  
 1
 
 174992598
 
 174992861
 
  ENSEMBL   UCSC 
 
  264
 
  6
 
 2100
 
 tss
 
 0.06401
 
 0.02386
 
 Not queried
 
  1.0715100
 
       NA
 
       NA
  
 
  
 13
 
  37572869
 
  37573100
 
  ENSEMBL   UCSC 
 
  232
 
  4
 
 11108
 
 gene;island
 
 0.01492
 
 0.02254
 
 Not queried
 
  1.0714921
 
       NA
 
       NA
  
 
  
 20
 
  53091600
 
  53092259
 
  ENSEMBL   UCSC 
 
  660
 
  6
 
 25792
 
 tss
 
 0.09998
 
 0.05393
 
 Not queried
 
  1.0713501
 
       NA
 
       NA
  
 
  
 10
 
 102497298
 
 102497666
 
  ENSEMBL   UCSC 
 
  369
 
  5
 
 6013
 
 island
 
 0.15738
 
 0.03462
 
 Not queried
 
  1.0710793
 
       NA
 
       NA
  
 
  
 16
 
  30457166
 
  30457524
 
  ENSEMBL   UCSC 
 
  359
 
 10
 
 15799
 
 island
 
 0.02056
 
 0.02652
 
 Not queried
 
  1.0709590
 
       NA
 
       NA
  
 
  
 21
 
  34774882
 
  34775045
 
  ENSEMBL   UCSC 
 
  164
 
  4
 
 26479
 
 tss;island
 
 0.71463
 
 0.58238
 
 Not queried
 
  1.0708936
 
       NA
 
       NA
  
 
  
 4
 
  41361623
 
  41362397
 
  ENSEMBL   UCSC 
 
  775
 
  9
 
 30316
 
 tss;island
 
 0.29281
 
 0.09219
 
 Not queried
 
  1.0707582
 
       NA
 
       NA
  
 
  
 1
 
 228473704
 
 228473980
 
  ENSEMBL   UCSC 
 
  277
 
  5
 
 1051
 
 gene;island
 
 0.76552
 
 0.78668
 
 Not queried
 
  1.0707501
 
       NA
 
       NA
  
 
  
 3
 
   9291334
 
   9291499
 
  ENSEMBL   UCSC 
 
  166
 
  4
 
 28081
 
 tss
 
 0.02204
 
 0.05041
 
 Not queried
 
  1.0705096
 
       NA
 
       NA
  
 
  
 20
 
   3024107
 
   3024368
 
  ENSEMBL   UCSC 
 
  262
 
  4
 
 25917
 
 island
 
 0.66330
 
 0.76614
 
 Not queried
 
  1.0704433
 
       NA
 
       NA
  
 
  
 10
 
 115998843
 
 115999009
 
  ENSEMBL   UCSC 
 
  167
 
  4
 
 5359
 
 tss
 
 0.03103
 
 0.09395
 
 Not queried
 
  1.0703651
 
       NA
 
       NA
  
 
  
 6
 
  28557047
 
  28558113
 
  ENSEMBL   UCSC 
 
 1067
 
 25
 
 35527
 
 island
 
 0.05439
 
 0.19913
 
 Not queried
 
  1.0702329
 
       NA
 
       NA
  
 
  
 16
 
  57496324
 
  57496545
 
  ENSEMBL   UCSC 
 
  222
 
  5
 
 15232
 
 tss
 
 0.03627
 
 0.06215
 
 Not queried
 
  1.0700769
 
       NA
 
       NA
  
 
  
 4
 
 142054660
 
 142054878
 
  ENSEMBL   UCSC 
 
  219
 
  8
 
 30561
 
 tss;island
 
 0.01375
 
 0.02052
 
 Not queried
 
  1.0698113
 
       NA
 
       NA
  
 
  
 2
 
  84517738
 
  84517782
 
  ENSEMBL   UCSC 
 
   45
 
  5
 
 23436
 
 tss
 
 0.95871
 
 0.93069
 
 Not queried
 
  1.0696005
 
       NA
 
       NA
  
 
  
 17
 
   1657448
 
   1657676
 
  ENSEMBL   UCSC 
 
  229
 
  4
 
 16286
 
 gene;island
 
 0.85896
 
 0.76831
 
 Not queried
 
  1.0694658
 
       NA
 
       NA
  
 
  
 19
 
   1021257
 
   1021368
 
  ENSEMBL   UCSC 
 
  112
 
  4
 
 20251
 
 tss;island
 
 0.01110
 
 0.01483
 
 Not queried
 
  1.0689038
 
       NA
 
       NA
  
 
  
 19
 
   3880535
 
   3880670
 
  ENSEMBL   UCSC 
 
  136
 
  4
 
 21316
 
 island
 
 0.01870
 
 0.03897
 
 Not queried
 
  1.0685906
 
       NA
 
       NA
  
 
  
 19
 
  57018848
 
  57019069
 
  ENSEMBL   UCSC 
 
  222
 
  6
 
 21063
 
 tss;island
 
 0.01489
 
 0.02560
 
 Not queried
 
  1.0684009
 
       NA
 
       NA
  
 
  
 18
 
  76829138
 
  76829335
 
  ENSEMBL   UCSC 
 
  198
 
  4
 
 19299
 
 tss;island
 
 0.01923
 
 0.03263
 
 Not queried
 
  1.0683058
 
       NA
 
       NA
  
 
  
 1
 
  14219312
 
  14219825
 
  ENSEMBL   UCSC 
 
  514
 
  4
 
 2706
 
 island
 
 0.07423
 
 0.02672
 
 Not queried
 
  1.0680711
 
       NA
 
       NA
  
 
  
 7
 
 123484898
 
 123485052
 
  ENSEMBL   UCSC 
 
  155
 
  4
 
 37806
 
 tss
 
 0.88083
 
 0.72146
 
 Not queried
 
  1.0680644
 
       NA
 
       NA
  
 
  
 20
 
  56803778
 
  56804327
 
  ENSEMBL   UCSC 
 
  550
 
  4
 
 26247
 
 island
 
 0.01654
 
 0.02898
 
 Not queried
 
  1.0680235
 
       NA
 
       NA
  
 
  
 14
 
 100625027
 
 100625253
 
  ENSEMBL   UCSC 
 
  227
 
  4
 
 12178
 
 gene;island
 
 0.46945
 
 0.28089
 
 Not queried
 
  1.0680076
 
       NA
 
       NA
  
 
  
 7
 
   8301971
 
   8302301
 
  ENSEMBL   UCSC 
 
  331
 
  8
 
 37373
 
 tss;island
 
 0.14352
 
 0.08360
 
 Not queried
 
  1.0678454
 
       NA
 
       NA
  
 
  
 18
 
    908130
 
    908505
 
  ENSEMBL   UCSC 
 
  376
 
  4
 
 19001
 
 gene;island
 
 0.04304
 
 0.20179
 
 Not queried
 
  1.0677706
 
       NA
 
       NA
  
 
  
 3
 
 122640625
 
 122641316
 
  ENSEMBL   UCSC 
 
  692
 
  7
 
 27876
 
 gene;island
 
 0.21924
 
 0.26526
 
 Not queried
 
  1.0677605
 
       NA
 
       NA
  
 
  
 22
 
  22288352
 
  22288559
 
  ENSEMBL   UCSC 
 
  208
 
  4
 
 26765
 
 gene;island
 
 0.75246
 
 0.85222
 
 Not queried
 
  1.0677586
 
       NA
 
       NA
  
 
  
 17
 
  77776428
 
  77776716
 
  ENSEMBL   UCSC 
 
  289
 
  4
 
 18809
 
 island
 
 0.13175
 
 0.02663
 
 Not queried
 
  1.0673540
 
       NA
 
       NA
  
 
  
 21
 
  47813364
 
  47813491
 
  ENSEMBL   UCSC 
 
  128
 
  4
 
 26435
 
 gene;island
 
 0.73480
 
 0.86372
 
 Not queried
 
  1.0673218
 
       NA
 
       NA
  
 
  
 11
 
 134582289
 
 134582508
 
  ENSEMBL   UCSC 
 
  220
 
  4
 
 8965
 
 island
 
 0.65838
 
 0.84377
 
 Not queried
 
  1.0670437
 
       NA
 
       NA
  
 
  
 17
 
  75471193
 
  75471491
 
  ENSEMBL   UCSC 
 
  299
 
  6
 
 16775
 
 gene
 
 0.88378
 
 0.80212
 
 Not queried
 
  1.0669746
 
       NA
 
       NA
  
 
  
 1
 
  20811132
 
  20811408
 
  ENSEMBL   UCSC 
 
  277
 
  4
 
 287
 
 gene;island
 
 0.04668
 
 0.07589
 
 Not queried
 
  1.0667714
 
       NA
 
       NA
  
 
  
 12
 
  32831969
 
  32832045
 
  ENSEMBL   UCSC 
 
   77
 
  4
 
 9608
 
 tss;island
 
 0.06659
 
 0.20187
 
 Not queried
 
  1.0667074
 
       NA
 
       NA
  
 
  
 12
 
  56615532
 
  56615727
 
  ENSEMBL   UCSC 
 
  196
 
  5
 
 9150
 
 gene
 
 0.01967
 
 0.04561
 
 Not queried
 
  1.0662673
 
       NA
 
       NA
  
 
  
 19
 
  55770037
 
  55770147
 
  ENSEMBL   UCSC 
 
  111
 
  4
 
 22380
 
 island
 
 0.04333
 
 0.08782
 
 Not queried
 
  1.0660409
 
       NA
 
       NA
  
 
  
 16
 
    126848
 
    127088
 
  ENSEMBL   UCSC 
 
  241
 
  5
 
 14927
 
 tss;island
 
 0.56403
 
 0.79019
 
 Not queried
 
  1.0659607
 
       NA
 
       NA
  
 
  
 2
 
 135809940
 
 135810355
 
  ENSEMBL   UCSC 
 
  416
 
  4
 
 22844
 
 gene
 
 0.02175
 
 0.03341
 
 Not queried
 
  1.0655211
 
       NA
 
       NA
  
 
  
 3
 
  13921803
 
  13921889
 
  ENSEMBL   UCSC 
 
   87
 
  4
 
 28125
 
 tss;island
 
 0.03116
 
 0.05880
 
 Not queried
 
  1.0654844
 
       NA
 
       NA
  
 
  
 12
 
  12878752
 
  12878810
 
  ENSEMBL   UCSC 
 
   59
 
  4
 
 9551
 
 tss
 
 0.01169
 
 0.01918
 
 Not queried
 
  1.0653772
 
       NA
 
       NA
  
 
  
 15
 
  55611214
 
  55611607
 
  ENSEMBL   UCSC 
 
  394
 
  6
 
 13329
 
 gene
 
 0.06126
 
 0.02580
 
 Not queried
 
  1.0650700
 
       NA
 
       NA
  
 
  
 17
 
   1090123
 
   1090557
 
  ENSEMBL   UCSC 
 
  435
 
  7
 
 16275
 
 gene
 
 0.46164
 
 0.28656
 
 Not queried
 
  1.0645616
 
       NA
 
       NA
  
 
  
 10
 
  75118622
 
  75118678
 
  ENSEMBL   UCSC 
 
   57
 
  6
 
 5141
 
 tss
 
 0.00892
 
 0.01622
 
 Not queried
 
  1.0642857
 
       NA
 
       NA
  
 
  
 17
 
  48473757
 
  48474099
 
  ENSEMBL   UCSC 
 
  343
 
  4
 
 16651
 
 gene;island
 
 0.03479
 
 0.07295
 
 Not queried
 
  1.0642832
 
       NA
 
       NA
  
 
  
 19
 
  10077007
 
  10077324
 
  ENSEMBL   UCSC 
 
  318
 
  4
 
 19769
 
 gene;island
 
 0.21415
 
 0.40655
 
 Not queried
 
  1.0642391
 
       NA
 
       NA
  
 
  
 7
 
  16438022
 
  16438465
 
  ENSEMBL   UCSC 
 
  444
 
  6
 
 36840
 
 gene
 
 0.91421
 
 0.87135
 
 Not queried
 
  1.0642038
 
       NA
 
       NA
  
 
  
 10
 
 125732604
 
 125732842
 
  ENSEMBL   UCSC 
 
  239
 
  4
 
 6203
 
 island
 
 0.61189
 
 0.77837
 
 Not queried
 
  1.0638663
 
       NA
 
       NA
  
 
  
 19
 
  37997675
 
  37997831
 
  ENSEMBL   UCSC 
 
  157
 
  8
 
 20704
 
 tss
 
 0.00643
 
 0.00972
 
 Not queried
 
  1.0636136
 
       NA
 
       NA
  
 
  
 11
 
  66643314
 
  66643572
 
  ENSEMBL   UCSC 
 
  259
 
  5
 
 6724
 
 gene
 
 0.91278
 
 0.89339
 
 Not queried
 
  1.0632629
 
       NA
 
       NA
  
 
  
 20
 
  62369445
 
  62369605
 
  ENSEMBL   UCSC 
 
  161
 
  4
 
 25472
 
 gene;island
 
 0.78654
 
 0.64067
 
 Not queried
 
  1.0631414
 
       NA
 
       NA
  
 
  
 1
 
 212004169
 
 212004468
 
  ENSEMBL   UCSC 
 
  300
 
  6
 
 2237
 
 tss
 
 0.02414
 
 0.04441
 
 Not queried
 
  1.0627992
 
       NA
 
       NA
  
 
  
 6
 
  32154416
 
  32154833
 
  ENSEMBL   UCSC 
 
  418
 
  7
 
 34016
 
 gene
 
 0.63864
 
 0.81015
 
 Not queried
 
  1.0625276
 
       NA
 
       NA
  
 
  
 3
 
  14444035
 
  14444067
 
  ENSEMBL   UCSC 
 
   33
 
  4
 
 28128
 
 tss
 
 0.08661
 
 0.12096
 
 Not queried
 
  1.0620323
 
       NA
 
       NA
  
 
  
 X
 
  46937571
 
  46937829
 
  ENSEMBL   UCSC 
 
  259
 
  4
 
 41742
 
 island
 
 0.76859
 
 0.86884
 
 Not queried
 
  1.0618682
 
       NA
 
       NA
  
 
  
 12
 
  94071150
 
  94071446
 
  ENSEMBL   UCSC 
 
  297
 
  4
 
 9221
 
 gene
 
 0.00850
 
 0.00819
 
 Not queried
 
  1.0618631
 
       NA
 
       NA
  
 
  
 19
 
   7504512
 
   7504569
 
  ENSEMBL   UCSC 
 
   58
 
  4
 
 20361
 
 tss
 
 0.89765
 
 0.93451
 
 Not queried
 
  1.0618168
 
       NA
 
       NA
  
 
  
 1
 
  86046322
 
  86046961
 
  ENSEMBL   UCSC 
 
  640
 
  8
 
 3419
 
 island
 
 0.01970
 
 0.03320
 
 Not queried
 
  1.0614454
 
       NA
 
       NA
  
 
  
 1
 
 116710227
 
 116710606
 
  ENSEMBL   UCSC 
 
  380
 
  4
 
 3627
 
 island
 
 0.18821
 
 0.30151
 
 Not queried
 
  1.0614090
 
       NA
 
       NA
  
 
  
 13
 
  37574747
 
  37575268
 
  ENSEMBL   UCSC 
 
  522
 
  6
 
 11109
 
 gene
 
 0.00941
 
 0.02288
 
 Not queried
 
  1.0606613
 
       NA
 
       NA
  
 
  
 13
 
  37574527
 
  37575268
 
  ENSEMBL   UCSC 
 
  742
 
 12
 
 11647
 
 island
 
 0.00842
 
 0.01676
 
 Not queried
 
  1.0606613
 
       NA
 
       NA
  
 
  
 10
 
  61469599
 
  61469980
 
  ENSEMBL   UCSC 
 
  382
 
  9
 
 5745
 
 island
 
 0.09511
 
 0.05086
 
 Not queried
 
  1.0605612
 
       NA
 
       NA
  
 
  
 2
 
 241721922
 
 241722281
 
  ENSEMBL   UCSC 
 
  360
 
  4
 
 23088
 
 gene;island
 
 0.73493
 
 0.85533
 
 Not queried
 
  1.0605001
 
       NA
 
       NA
  
 
  
 2
 
 158454083
 
 158454401
 
  ENSEMBL   UCSC 
 
  319
 
  5
 
 23639
 
 tss
 
 0.03077
 
 0.14497
 
 Not queried
 
  1.0600144
 
       NA
 
       NA
  
 
  
 2
 
 239112427
 
 239112731
 
  ENSEMBL   UCSC 
 
  305
 
  5
 
 23933
 
 tss
 
 0.04401
 
 0.05423
 
 Not queried
 
  1.0598450
 
       NA
 
       NA
  
 
  
 7
 
   4806755
 
   4807062
 
  ENSEMBL   UCSC 
 
  308
 
  4
 
 36788
 
 gene;island
 
 0.83057
 
 0.90718
 
 Not queried
 
  1.0597296
 
       NA
 
       NA
  
 
  
 5
 
   5287980
 
   5288296
 
  ENSEMBL   UCSC 
 
  317
 
  6
 
 31496
 
 gene
 
 0.90541
 
 0.96332
 
 Not queried
 
  1.0596922
 
       NA
 
       NA
  
 
  
 17
 
  60885856
 
  60886079
 
  ENSEMBL   UCSC 
 
  224
 
  4
 
 17529
 
 tss
 
 0.42202
 
 0.59358
 
 Not queried
 
  1.0596808
 
       NA
 
       NA
  
 
  
 11
 
  76381044
 
  76381236
 
  ENSEMBL   UCSC 
 
  193
 
  6
 
 7555
 
 tss
 
 0.27069
 
 0.43610
 
 Not queried
 
  1.0595870
 
       NA
 
       NA
  
 
  
 6
 
  25930894
 
  25931205
 
  ENSEMBL   UCSC 
 
  312
 
  5
 
 34612
 
 tss
 
 0.95005
 
 0.86264
 
 Not queried
 
  1.0595353
 
       NA
 
       NA
  
 
  
 19
 
  41860004
 
  41860095
 
  ENSEMBL   UCSC 
 
   92
 
  5
 
 20770
 
 tss;island
 
 0.04012
 
 0.02526
 
 Not queried
 
  1.0594049
 
       NA
 
       NA
  
 
  
 5
 
  59189375
 
  59189934
 
  ENSEMBL   UCSC 
 
  560
 
 10
 
 31568
 
 gene;island
 
 0.05220
 
 0.10659
 
 Not queried
 
  1.0593012
 
       NA
 
       NA
  
 
  
 2
 
 175351869
 
 175351956
 
  ENSEMBL   UCSC 
 
   88
 
  6
 
 23687
 
 tss
 
 0.05034
 
 0.06277
 
 Not queried
 
  1.0592317
 
       NA
 
       NA
  
 
  
 2
 
  73871782
 
  73871942
 
  ENSEMBL   UCSC 
 
  161
 
  5
 
 23404
 
 tss
 
 0.48604
 
 0.55752
 
 Not queried
 
  1.0591689
 
       NA
 
       NA
  
 
  
 17
 
  32909042
 
  32909375
 
  ENSEMBL   UCSC 
 
  334
 
  4
 
 16480
 
 gene;island
 
 0.45625
 
 0.21592
 
 Not queried
 
  1.0591608
 
       NA
 
       NA
  
 
  
 4
 
  48271072
 
  48271149
 
  ENSEMBL   UCSC 
 
   78
 
  4
 
 29953
 
 gene;island
 
 0.04104
 
 0.02480
 
 Not queried
 
  1.0590134
 
       NA
 
       NA
  
 
  
 7
 
  73867797
 
  73868174
 
  ENSEMBL   UCSC 
 
  378
 
 10
 
 37584
 
 tss;island
 
 0.02144
 
 0.02113
 
 Not queried
 
  1.0588506
 
       NA
 
       NA
  
 
  
 17
 
  72357992
 
  72358370
 
  ENSEMBL   UCSC 
 
  379
 
  5
 
 17591
 
 tss;island
 
 0.45372
 
 0.64822
 
 Not queried
 
  1.0588390
 
       NA
 
       NA
  
 
  
 1
 
  53527412
 
  53527693
 
  ENSEMBL   UCSC 
 
  282
 
  6
 
 1632
 
 tss;island
 
 0.34729
 
 0.16848
 
 Not queried
 
  1.0588114
 
       NA
 
       NA
  
 
  
 X
 
  47479048
 
  47479190
 
  ENSEMBL   UCSC 
 
  143
 
  5
 
 40979
 
 gene;island
 
 0.03371
 
 0.05047
 
 Not queried
 
  1.0586951
 
       NA
 
       NA
  
 
  
 11
 
   9113152
 
   9113458
 
  ENSEMBL   UCSC 
 
  307
 
  9
 
 7112
 
 tss
 
 0.04309
 
 0.06846
 
 Not queried
 
  1.0585560
 
       NA
 
       NA
  
 
  
 11
 
   3817692
 
   3817849
 
  ENSEMBL   UCSC 
 
  158
 
  4
 
 6462
 
 gene;tss;island
 
 0.03182
 
 0.06288
 
 Not queried
 
  1.0584333
 
       NA
 
       NA
  
 
  
 17
 
  72427538
 
  72427637
 
  ENSEMBL   UCSC 
 
  100
 
  5
 
 17593
 
 tss
 
 0.02781
 
 0.06346
 
 Not queried
 
  1.0584136
 
       NA
 
       NA
  
 
  
 1
 
 197882003
 
 197882469
 
  ENSEMBL   UCSC 
 
  467
 
  4
 
 927
 
 gene;island
 
 0.37199
 
 0.18026
 
 Not queried
 
  1.0583941
 
       NA
 
       NA
  
 
  
 6
 
  80714048
 
  80714315
 
  ENSEMBL   UCSC 
 
  268
 
  7
 
 35020
 
 tss
 
 0.01794
 
 0.02346
 
 Not queried
 
  1.0583759
 
       NA
 
       NA
  
 
  
 1
 
   3077798
 
   3078206
 
  ENSEMBL   UCSC 
 
  409
 
  4
 
 104
 
 gene;island
 
 0.71756
 
 0.84214
 
 Not queried
 
  1.0580891
 
       NA
 
       NA
  
 
  
 11
 
 111895234
 
 111895577
 
  ENSEMBL   UCSC 
 
  344
 
  7
 
 8785
 
 island
 
 0.04790
 
 0.02531
 
 Not queried
 
  1.0577408
 
       NA
 
       NA
  
 
  
 1
 
  47655861
 
  47656140
 
  ENSEMBL   UCSC 
 
  280
 
  4
 
 1603
 
 tss
 
 0.63539
 
 0.47456
 
 Not queried
 
  1.0571677
 
       NA
 
       NA
  
 
  
 6
 
  41343210
 
  41343830
 
  ENSEMBL   UCSC 
 
  621
 
  6
 
 36058
 
 island
 
 0.38901
 
 0.14914
 
 Not queried
 
  1.0567235
 
       NA
 
       NA
  
 
  
 16
 
   1198408
 
   1198900
 
  ENSEMBL   UCSC 
 
  493
 
  5
 
 15487
 
 island
 
 0.68587
 
 0.87449
 
 Not queried
 
  1.0567107
 
       NA
 
       NA
  
 
  
 14
 
 101509144
 
 101509391
 
  ENSEMBL   UCSC 
 
  248
 
  4
 
 12598
 
 tss
 
 0.87770
 
 0.77518
 
 Not queried
 
  1.0566320
 
       NA
 
       NA
  
 
  
 6
 
  27799308
 
  27799458
 
  ENSEMBL   UCSC 
 
  151
 
  6
 
 34659
 
 tss
 
 0.01637
 
 0.02434
 
 Not queried
 
  1.0565992
 
       NA
 
       NA
  
 
  
 1
 
 215256254
 
 215256978
 
  ENSEMBL   UCSC 
 
  725
 
  9
 
 1006
 
 gene;island
 
 0.04013
 
 0.04237
 
 Not queried
 
  1.0565710
 
       NA
 
       NA
  
 
  
 13
 
  46961656
 
  46961897
 
  ENSEMBL   UCSC 
 
  242
 
  9
 
 11398
 
 tss
 
 0.01386
 
 0.02035
 
 Not queried
 
  1.0565666
 
       NA
 
       NA
  
 
  
 17
 
  78172177
 
  78172613
 
  ENSEMBL   UCSC 
 
  437
 
  5
 
 16825
 
 gene;island
 
 0.74351
 
 0.82530
 
 Not queried
 
  1.0565079
 
       NA
 
       NA
  
 
  
 21
 
  43299412
 
  43299503
 
  ENSEMBL   UCSC 
 
   92
 
  5
 
 26392
 
 gene
 
 0.01314
 
 0.02351
 
 Not queried
 
  1.0563172
 
       NA
 
       NA
  
 
  
 16
 
  30661534
 
  30662237
 
  ENSEMBL   UCSC 
 
  704
 
 10
 
 15159
 
 tss
 
 0.06336
 
 0.08981
 
 Not queried
 
  1.0562792
 
       NA
 
       NA
  
 
  
 7
 
   2670978
 
   2671564
 
  ENSEMBL   UCSC 
 
  587
 
  7
 
 37349
 
 tss;island
 
 0.03885
 
 0.18081
 
 Not queried
 
  1.0561380
 
       NA
 
       NA
  
 
  
 X
 
  55515154
 
  55515614
 
  ENSEMBL   UCSC 
 
  461
 
  4
 
 41002
 
 gene
 
 0.05036
 
 0.07935
 
 Not queried
 
  1.0559997
 
       NA
 
       NA
  
 
  
 16
 
  23652837
 
  23653174
 
  ENSEMBL   UCSC 
 
  338
 
  7
 
 14665
 
 gene
 
 0.04813
 
 0.01781
 
 Not queried
 
  1.0558657
 
       NA
 
       NA
  
 
  
 1
 
   3123338
 
   3123854
 
  ENSEMBL   UCSC 
 
  517
 
  4
 
 107
 
 gene
 
 0.65716
 
 0.83021
 
 Not queried
 
  1.0557755
 
       NA
 
       NA
  
 
  
 4
 
  99064322
 
  99064904
 
  ENSEMBL   UCSC 
 
  583
 
  8
 
 31139
 
 island
 
 0.01959
 
 0.02761
 
 Not queried
 
  1.0557409
 
       NA
 
       NA
  
 
  
 4
 
 185655352
 
 185655703
 
  ENSEMBL   UCSC 
 
  352
 
  7
 
 30648
 
 tss
 
 0.01414
 
 0.03185
 
 Not queried
 
  1.0556269
 
       NA
 
       NA
  
 
  
 16
 
   1661904
 
   1662444
 
  ENSEMBL   UCSC 
 
  541
 
  9
 
 15522
 
 island
 
 0.01358
 
 0.02369
 
 Not queried
 
  1.0552504
 
       NA
 
       NA
  
 
  
 1
 
 228346014
 
 228346347
 
  ENSEMBL   UCSC 
 
  334
 
  4
 
 1045
 
 gene;island
 
 0.85055
 
 0.91709
 
 Not queried
 
  1.0552112
 
       NA
 
       NA
  
 
  
 12
 
   6560689
 
   6560851
 
  ENSEMBL   UCSC 
 
  163
 
  5
 
 9003
 
 gene
 
 0.58500
 
 0.71522
 
 Not queried
 
  1.0551251
 
       NA
 
       NA
  
 
  
 12
 
   6560689
 
   6561007
 
  ENSEMBL   UCSC 
 
  319
 
  7
 
 9486
 
 tss
 
 0.58500
 
 0.71522
 
 Not queried
 
  1.0551251
 
       NA
 
       NA
  
 
  
 6
 
 170189761
 
 170190129
 
  ENSEMBL   UCSC 
 
  369
 
  9
 
 34514
 
 gene;tss;island
 
 0.03031
 
 0.07704
 
 Not queried
 
  1.0550035
 
       NA
 
       NA
  
 
  
 5
 
  68462622
 
  68462783
 
  ENSEMBL   UCSC 
 
  162
 
  5
 
 32046
 
 tss;island
 
 0.04538
 
 0.05805
 
 Not queried
 
  1.0545746
 
       NA
 
       NA
  
 
  
 7
 
 150434452
 
 150434640
 
  ENSEMBL   UCSC 
 
  189
 
  5
 
 37213
 
 gene
 
 0.33147
 
 0.62001
 
 Not queried
 
  1.0541025
 
       NA
 
       NA
  
 
  
 19
 
  51670251
 
  51670552
 
  ENSEMBL   UCSC 
 
  302
 
  5
 
 20958
 
 tss
 
 0.56902
 
 0.47193
 
 Not queried
 
  1.0540744
 
       NA
 
       NA
  
 
  
 2
 
 183942982
 
 183943203
 
  ENSEMBL   UCSC 
 
  222
 
  4
 
 23731
 
 tss
 
 0.10015
 
 0.10056
 
 Not queried
 
  1.0539392
 
       NA
 
       NA
  
 
  
 7
 
 149195083
 
 149195257
 
  ENSEMBL   UCSC 
 
  175
 
  5
 
 37904
 
 tss;island
 
 0.06046
 
 0.03253
 
 Not queried
 
  1.0536375
 
       NA
 
       NA
  
 
  
 1
 
 119543057
 
 119543336
 
  ENSEMBL   UCSC 
 
  280
 
  4
 
 3649
 
 island
 
 0.47214
 
 0.64852
 
 Not queried
 
  1.0534682
 
       NA
 
       NA
  
 
  
 6
 
  31740787
 
  31741184
 
  ENSEMBL   UCSC 
 
  398
 
  9
 
 33889
 
 gene
 
 0.37278
 
 0.61062
 
 Not queried
 
  1.0532611
 
       NA
 
       NA
  
 
  
 1
 
 113162105
 
 113162646
 
  ENSEMBL   UCSC 
 
  542
 
  8
 
 691
 
 gene
 
 0.01552
 
 0.03787
 
 Not queried
 
  1.0531161
 
       NA
 
       NA
  
 
  
 10
 
   8555579
 
   8556087
 
  ENSEMBL   UCSC 
 
  509
 
  5
 
 5524
 
 island
 
 0.40970
 
 0.59018
 
 Not queried
 
  1.0530955
 
       NA
 
       NA
  
 
  
 X
 
  69509958
 
  69510172
 
  ENSEMBL   UCSC 
 
  215
 
  4
 
 41016
 
 gene
 
 0.09301
 
 0.04096
 
 Not queried
 
  1.0530797
 
       NA
 
       NA
  
 
  
 19
 
   1885442
 
   1885691
 
  ENSEMBL   UCSC 
 
  250
 
  4
 
 21214
 
 island
 
 0.06491
 
 0.16036
 
 Not queried
 
  1.0528722
 
       NA
 
       NA
  
 
  
 3
 
 187387555
 
 187388148
 
  ENSEMBL   UCSC 
 
  594
 
  8
 
 28034
 
 gene
 
 0.03412
 
 0.14949
 
 Not queried
 
  1.0528651
 
       NA
 
       NA
  
 
  
 3
 
 187387555
 
 187388281
 
  ENSEMBL   UCSC 
 
  727
 
 10
 
 29715
 
 island
 
 0.03412
 
 0.16881
 
 Not queried
 
  1.0528651
 
       NA
 
       NA
  
 
  
 8
 
 135724771
 
 135725096
 
  ENSEMBL   UCSC 
 
  326
 
  4
 
 39371
 
 gene;island
 
 0.00993
 
 0.03867
 
 Not queried
 
  1.0527264
 
       NA
 
       NA
  
 
  
 2
 
  54483431
 
  54483817
 
  ENSEMBL   UCSC 
 
  387
 
  5
 
 23316
 
 tss
 
 0.90164
 
 0.88121
 
 Not queried
 
  1.0524874
 
       NA
 
       NA
  
 
  
 15
 
  41228521
 
  41228736
 
  ENSEMBL   UCSC 
 
  216
 
  5
 
 13271
 
 gene;island
 
 0.86684
 
 0.73714
 
 Not queried
 
  1.0523515
 
       NA
 
       NA
  
 
  
 2
 
 234749818
 
 234750116
 
  ENSEMBL   UCSC 
 
  299
 
  5
 
 23041
 
 gene
 
 0.86539
 
 0.93744
 
 Not queried
 
  1.0522049
 
       NA
 
       NA
  
 
  
 2
 
 107459661
 
 107459860
 
  ENSEMBL   UCSC 
 
  200
 
  4
 
 22779
 
 gene;island
 
 0.78333
 
 0.81865
 
 Not queried
 
  1.0520794
 
       NA
 
       NA
  
 
  
 16
 
    875881
 
    876216
 
  ENSEMBL   UCSC 
 
  336
 
  4
 
 15461
 
 island
 
 0.91991
 
 0.95562
 
 Not queried
 
  1.0520622
 
       NA
 
       NA
  
 
  
 1
 
  35259746
 
  35260256
 
  ENSEMBL   UCSC 
 
  511
 
  5
 
 413
 
 gene;island
 
 0.73645
 
 0.62169
 
 Not queried
 
  1.0517958
 
       NA
 
       NA
  
 
  
 11
 
  84634409
 
  84634735
 
  ENSEMBL   UCSC 
 
  327
 
  5
 
 6832
 
 gene
 
 0.78051
 
 0.63172
 
 Not queried
 
  1.0517558
 
       NA
 
       NA
  
 
  
 18
 
  47013750
 
  47014260
 
  ENSEMBL   UCSC 
 
  511
 
  6
 
 19236
 
 tss;island
 
 0.08058
 
 0.20373
 
 Not queried
 
  1.0515016
 
       NA
 
       NA
  
 
  
 13
 
  49001104
 
  49001351
 
  ENSEMBL   UCSC 
 
  248
 
  4
 
 11134
 
 gene;tss
 
 0.73390
 
 0.87661
 
 Not queried
 
  1.0513872
 
       NA
 
       NA
  
 
  
 6
 
  31323253
 
  31324024
 
  ENSEMBL   UCSC 
 
  772
 
 11
 
 33805
 
 gene;island
 
 0.77988
 
 0.61203
 
 Not queried
 
  1.0513764
 
       NA
 
       NA
  
 
  
 19
 
   3595702
 
   3595996
 
  ENSEMBL   UCSC 
 
  295
 
  4
 
 19681
 
 gene;island
 
 0.83762
 
 0.71493
 
 Not queried
 
  1.0507581
 
       NA
 
       NA
  
 
  
 10
 
 128076910
 
 128077080
 
  ENSEMBL   UCSC 
 
  171
 
  4
 
 4800
 
 gene;island
 
 0.06909
 
 0.04318
 
 Not queried
 
  1.0505137
 
       NA
 
       NA
  
 
  
 6
 
   1608396
 
   1608667
 
  ENSEMBL   UCSC 
 
  272
 
  4
 
 35309
 
 island
 
 0.03332
 
 0.06553
 
 Not queried
 
  1.0504004
 
       NA
 
       NA
  
 
  
 4
 
 156875082
 
 156875336
 
  ENSEMBL   UCSC 
 
  255
 
  6
 
 30604
 
 tss;island
 
 0.03249
 
 0.07249
 
 Not queried
 
  1.0501489
 
       NA
 
       NA
  
 
  
 7
 
  27197455
 
  27198429
 
  ENSEMBL   UCSC 
 
  975
 
 10
 
 38290
 
 island
 
 0.91518
 
 0.86280
 
 Not queried
 
  1.0501031
 
       NA
 
       NA
  
 
  
 11
 
   2417651
 
   2418157
 
  ENSEMBL   UCSC 
 
  507
 
  5
 
 6439
 
 gene;island
 
 0.87190
 
 0.93374
 
 Not queried
 
  1.0499971
 
       NA
 
       NA
  
 
  
 11
 
  94964236
 
  94964516
 
  ENSEMBL   UCSC 
 
  281
 
  7
 
 8723
 
 island
 
 0.02058
 
 0.02910
 
 Not queried
 
  1.0499235
 
       NA
 
       NA
  
 
  
 8
 
   1712007
 
   1712330
 
  ENSEMBL   UCSC 
 
  324
 
  4
 
 39111
 
 gene;island
 
 0.02086
 
 0.03655
 
 Not queried
 
  1.0497133
 
       NA
 
       NA
  
 
  
 17
 
   2414945
 
   2415399
 
  ENSEMBL   UCSC 
 
  455
 
  8
 
 17814
 
 island
 
 0.00981
 
 0.02176
 
 Not queried
 
  1.0496724
 
       NA
 
       NA
  
 
  
 3
 
 128444572
 
 128445120
 
  ENSEMBL   UCSC 
 
  549
 
  8
 
 29415
 
 island
 
 0.02355
 
 0.03313
 
 Not queried
 
  1.0496596
 
       NA
 
       NA
  
 
  
 1
 
   2429996
 
   2430249
 
  ENSEMBL   UCSC 
 
  254
 
  4
 
 87
 
 gene;island
 
 0.55596
 
 0.69515
 
 Not queried
 
  1.0492208
 
       NA
 
       NA
  
 
  
 2
 
   3321998
 
   3322515
 
  ENSEMBL   UCSC 
 
  518
 
  7
 
 22509
 
 gene;island
 
 0.76102
 
 0.82108
 
 Not queried
 
  1.0488290
 
       NA
 
       NA
  
 
  
 1
 
   2238720
 
   2239065
 
  ENSEMBL   UCSC 
 
  346
 
  4
 
 73
 
 gene;island
 
 0.85336
 
 0.87253
 
 Not queried
 
  1.0486543
 
       NA
 
       NA
  
 
  
 6
 
 166582159
 
 166582585
 
  ENSEMBL   UCSC 
 
  427
 
  9
 
 35270
 
 tss;island
 
 0.09843
 
 0.03233
 
 Not queried
 
  1.0484192
 
       NA
 
       NA
  
 
  
 14
 
  96670628
 
  96671104
 
  ENSEMBL   UCSC 
 
  477
 
 10
 
 12564
 
 tss
 
 0.32342
 
 0.43874
 
 Not queried
 
  1.0481175
 
       NA
 
       NA
  
 
  
 1
 
   8878173
 
   8878227
 
  ENSEMBL   UCSC 
 
   55
 
  4
 
 1220
 
 tss;island
 
 0.01473
 
 0.02676
 
 Not queried
 
  1.0480948
 
       NA
 
       NA
  
 
  
 12
 
 131443231
 
 131443486
 
  ENSEMBL   UCSC 
 
  256
 
  6
 
 9395
 
 gene
 
 0.68621
 
 0.85364
 
 Not queried
 
  1.0478307
 
       NA
 
       NA
  
 
  
 19
 
  36120042
 
  36120543
 
  ENSEMBL   UCSC 
 
  502
 
  4
 
 19952
 
 gene
 
 0.03041
 
 0.05313
 
 Not queried
 
  1.0477225
 
       NA
 
       NA
  
 
  
 16
 
  53468112
 
  53468205
 
  ENSEMBL   UCSC 
 
   94
 
  4
 
 15204
 
 tss
 
 0.05634
 
 0.03033
 
 Not queried
 
  1.0475816
 
       NA
 
       NA
  
 
  
 5
 
 172571388
 
 172571438
 
  ENSEMBL   UCSC 
 
   51
 
  4
 
 32441
 
 tss
 
 0.05111
 
 0.03142
 
 Not queried
 
  1.0474150
 
       NA
 
       NA
  
 
  
 7
 
 107643925
 
 107644024
 
  ENSEMBL   UCSC 
 
  100
 
  6
 
 37768
 
 tss;island
 
 0.00710
 
 0.00577
 
 Not queried
 
  1.0473931
 
       NA
 
       NA
  
 
  
 15
 
  40452931
 
  40453199
 
  ENSEMBL   UCSC 
 
  269
 
  9
 
 13554
 
 tss
 
 0.01658
 
 0.03097
 
 Not queried
 
  1.0471965
 
       NA
 
       NA
  
 
  
 14
 
  23503928
 
  23504423
 
  ENSEMBL   UCSC 
 
  496
 
  6
 
 11993
 
 gene
 
 0.01116
 
 0.02447
 
 Not queried
 
  1.0468122
 
       NA
 
       NA
  
 
  
 1
 
  39956641
 
  39956807
 
  ENSEMBL   UCSC 
 
  167
 
  4
 
 1519
 
 tss;island
 
 0.06288
 
 0.12250
 
 Not queried
 
  1.0467728
 
       NA
 
       NA
  
 
  
 17
 
  37764220
 
  37764293
 
  ENSEMBL   UCSC 
 
   74
 
  4
 
 17277
 
 tss
 
 0.23270
 
 0.36387
 
 Not queried
 
  1.0467428
 
       NA
 
       NA
  
 
  
 10
 
  43800133
 
  43800478
 
  ENSEMBL   UCSC 
 
  346
 
  6
 
 5674
 
 island
 
 0.43985
 
 0.28081
 
 Not queried
 
  1.0466009
 
       NA
 
       NA
  
 
  
 X
 
  55026360
 
  55026774
 
  ENSEMBL   UCSC 
 
  415
 
  4
 
 41338
 
 tss
 
 0.21114
 
 0.28072
 
 Not queried
 
  1.0465778
 
       NA
 
       NA
  
 
  
 13
 
  41363245
 
  41363591
 
  ENSEMBL   UCSC 
 
  347
 
  4
 
 11658
 
 island
 
 0.07174
 
 0.03485
 
 Not queried
 
  1.0463142
 
       NA
 
       NA
  
 
  
 3
 
  50396971
 
  50397174
 
  ENSEMBL   UCSC 
 
  204
 
  6
 
 28315
 
 tss;island
 
 0.05112
 
 0.02994
 
 Not queried
 
  1.0462964
 
       NA
 
       NA
  
 
  
 6
 
 144385609
 
 144386528
 
  ENSEMBL   UCSC 
 
  920
 
 22
 
 36472
 
 island
 
 0.77102
 
 0.46748
 
 Not queried
 
  1.0461810
 
       NA
 
       NA
  
 
  
 7
 
 133811808
 
 133812072
 
  ENSEMBL   UCSC 
 
  265
 
  8
 
 37844
 
 tss
 
 0.01371
 
 0.02416
 
 Not queried
 
  1.0460069
 
       NA
 
       NA
  
 
  
 7
 
    569642
 
    570041
 
  ENSEMBL   UCSC 
 
  400
 
  5
 
 37972
 
 island
 
 0.79399
 
 0.91056
 
 Not queried
 
  1.0459407
 
       NA
 
       NA
  
 
  
 11
 
  30344200
 
  30344725
 
  ENSEMBL   UCSC 
 
  526
 
  9
 
 8132
 
 island
 
 0.02812
 
 0.02581
 
 Not queried
 
  1.0458916
 
       NA
 
       NA
  
 
  
 7
 
  27183133
 
  27184853
 
  ENSEMBL   UCSC 
 
 1721
 
 47
 
 38283
 
 island
 
 0.13823
 
 0.31296
 
 Not queried
 
  1.0457383
 
       NA
 
       NA
  
 
  
 22
 
  38453296
 
  38453489
 
  ENSEMBL   UCSC 
 
  194
 
  4
 
 26827
 
 gene
 
 0.02588
 
 0.05314
 
 Not queried
 
  1.0456270
 
       NA
 
       NA
  
 
  
 7
 
  30492035
 
  30492171
 
  ENSEMBL   UCSC 
 
  137
 
  4
 
 36891
 
 gene
 
 0.74445
 
 0.85625
 
 Not queried
 
  1.0455333
 
       NA
 
       NA
  
 
  
 2
 
 177021702
 
 177022285
 
  ENSEMBL   UCSC 
 
  584
 
  7
 
 24871
 
 island
 
 0.02852
 
 0.03653
 
 Not queried
 
  1.0451893
 
       NA
 
       NA
  
 
  
 17
 
  33446966
 
  33447236
 
  ENSEMBL   UCSC 
 
  271
 
  9
 
 17232
 
 tss;island
 
 0.02225
 
 0.05618
 
 Not queried
 
  1.0446018
 
       NA
 
       NA
  
 
  
 8
 
 142192468
 
 142192718
 
  ENSEMBL   UCSC 
 
  251
 
  5
 
 39386
 
 gene
 
 0.80511
 
 0.87815
 
 Not queried
 
  1.0445404
 
       NA
 
       NA
  
 
  
 15
 
  41061984
 
  41062113
 
  ENSEMBL   UCSC 
 
  130
 
  4
 
 13567
 
 tss
 
 0.82569
 
 0.91009
 
 Not queried
 
  1.0444733
 
       NA
 
       NA
  
 
  
 17
 
  46114615
 
  46114933
 
  ENSEMBL   UCSC 
 
  319
 
  6
 
 17417
 
 tss
 
 0.06114
 
 0.17663
 
 Not queried
 
  1.0444224
 
       NA
 
       NA
  
 
  
 11
 
   1974132
 
   1974332
 
  ENSEMBL   UCSC 
 
  201
 
  4
 
 6422
 
 gene;island
 
 0.82157
 
 0.89693
 
 Not queried
 
  1.0444040
 
       NA
 
       NA
  
 
  
 1
 
  41328133
 
  41328420
 
  ENSEMBL   UCSC 
 
  288
 
  8
 
 1539
 
 tss;island
 
 0.04065
 
 0.05846
 
 Not queried
 
  1.0441466
 
       NA
 
       NA
  
 
  
 19
 
  39322242
 
  39322471
 
  ENSEMBL   UCSC 
 
  230
 
  4
 
 19985
 
 gene
 
 0.00837
 
 0.01847
 
 Not queried
 
  1.0439813
 
       NA
 
       NA
  
 
  
 2
 
 132348567
 
 132348901
 
  ENSEMBL   UCSC 
 
  335
 
  4
 
 24697
 
 island
 
 0.66103
 
 0.83361
 
 Not queried
 
  1.0434142
 
       NA
 
       NA
  
 
  
 16
 
    832942
 
    833250
 
  ENSEMBL   UCSC 
 
  309
 
  7
 
 14952
 
 tss
 
 0.48479
 
 0.70320
 
 Not queried
 
  1.0433568
 
       NA
 
       NA
  
 
  
 15
 
  38857079
 
  38857474
 
  ENSEMBL   UCSC 
 
  396
 
  7
 
 13546
 
 tss;island
 
 0.03636
 
 0.02341
 
 Not queried
 
  1.0433275
 
       NA
 
       NA
  
 
  
 10
 
  12171666
 
  12171805
 
  ENSEMBL   UCSC 
 
  140
 
  4
 
 4484
 
 gene;island
 
 0.01577
 
 0.03080
 
 Not queried
 
  1.0433018
 
       NA
 
       NA
  
 
  
 1
 
 112532802
 
 112533260
 
  ENSEMBL   UCSC 
 
  459
 
  7
 
 1829
 
 tss;island
 
 0.05455
 
 0.07893
 
 Not queried
 
  1.0432906
 
       NA
 
       NA
  
 
  
 1
 
 166845374
 
 166845621
 
  ENSEMBL   UCSC 
 
  248
 
  4
 
 868
 
 gene
 
 0.01731
 
 0.02633
 
 Not queried
 
  1.0432484
 
       NA
 
       NA
  
 
  
 5
 
   1100096
 
   1100544
 
  ENSEMBL   UCSC 
 
  449
 
  4
 
 31470
 
 gene;island
 
 0.88808
 
 0.92527
 
 Not queried
 
  1.0431293
 
       NA
 
       NA
  
 
  
 10
 
  75757809
 
  75757817
 
  ENSEMBL   UCSC 
 
    9
 
  4
 
 5150
 
 tss
 
 0.01317
 
 0.01997
 
 Not queried
 
  1.0430692
 
       NA
 
       NA
  
 
  
 12
 
 132900274
 
 132901088
 
  ENSEMBL   UCSC 
 
  815
 
  7
 
 9423
 
 gene;island
 
 0.83453
 
 0.89370
 
 Not queried
 
  1.0429562
 
       NA
 
       NA
  
 
  
 15
 
  45996340
 
  45996787
 
  ENSEMBL   UCSC 
 
  448
 
  5
 
 14089
 
 island
 
 0.03123
 
 0.00835
 
 Not queried
 
  1.0428812
 
       NA
 
       NA
  
 
  
 7
 
 129845338
 
 129845597
 
  ENSEMBL   UCSC 
 
  260
 
 10
 
 37832
 
 tss
 
 0.01328
 
 0.02439
 
 Not queried
 
  1.0428004
 
       NA
 
       NA
  
 
  
 6
 
  31476231
 
  31476403
 
  ENSEMBL   UCSC 
 
  173
 
  4
 
 33823
 
 gene
 
 0.63849
 
 0.50784
 
 Not queried
 
  1.0427408
 
       NA
 
       NA
  
 
  
 16
 
   3225355
 
   3225686
 
  ENSEMBL   UCSC 
 
  332
 
  5
 
 15608
 
 island
 
 0.01714
 
 0.04395
 
 Not queried
 
  1.0427284
 
       NA
 
       NA
  
 
  
 16
 
  89163343
 
  89163800
 
  ENSEMBL   UCSC 
 
  458
 
  5
 
 14896
 
 gene;island
 
 0.86348
 
 0.88679
 
 Not queried
 
  1.0424097
 
       NA
 
       NA
  
 
  
 8
 
  80942618
 
  80942820
 
  ENSEMBL   UCSC 
 
  203
 
  5
 
 39717
 
 tss;island
 
 0.00889
 
 0.01400
 
 Not queried
 
  1.0421805
 
       NA
 
       NA
  
 
  
 17
 
  43974804
 
  43975063
 
  ENSEMBL   UCSC 
 
  260
 
  5
 
 16606
 
 gene;island
 
 0.16740
 
 0.24402
 
 Not queried
 
  1.0420366
 
       NA
 
       NA
  
 
  
 X
 
  24665458
 
  24665669
 
  ENSEMBL   UCSC 
 
  212
 
  7
 
 41232
 
 tss
 
 0.04804
 
 0.02941
 
 Not queried
 
  1.0420191
 
       NA
 
       NA
  
 
  
 5
 
  43602595
 
  43602919
 
  ENSEMBL   UCSC 
 
  325
 
  5
 
 31991
 
 tss;island
 
 0.00771
 
 0.01678
 
 Not queried
 
  1.0420039
 
       NA
 
       NA
  
 
  
 10
 
 118897836
 
 118898082
 
  ENSEMBL   UCSC 
 
  247
 
  6
 
 5371
 
 tss;island
 
 0.01072
 
 0.02098
 
 Not queried
 
  1.0417517
 
       NA
 
       NA
  
 
  
 11
 
    537287
 
    537496
 
  ENSEMBL   UCSC 
 
  210
 
  7
 
 6995
 
 tss
 
 0.07051
 
 0.03439
 
 Not queried
 
  1.0417215
 
       NA
 
       NA
  
 
  
 1
 
 159174001
 
 159174162
 
  ENSEMBL   UCSC 
 
  162
 
  4
 
 2025
 
 tss
 
 0.20807
 
 0.34882
 
 Not queried
 
  1.0416559
 
       NA
 
       NA
  
 
  
 2
 
 132558939
 
 132559214
 
  ENSEMBL   UCSC 
 
  276
 
  4
 
 22835
 
 gene
 
 0.22936
 
 0.37631
 
 Not queried
 
  1.0415356
 
       NA
 
       NA
  
 
  
 1
 
  87169752
 
  87170127
 
  ENSEMBL   UCSC 
 
  376
 
  7
 
 1725
 
 tss;island
 
 0.13062
 
 0.02239
 
 Not queried
 
  1.0414887
 
       NA
 
       NA
  
 
  
 6
 
  30078954
 
  30079662
 
  ENSEMBL   UCSC 
 
  709
 
 21
 
 33661
 
 gene
 
 0.52228
 
 0.30650
 
 Not queried
 
  1.0414191
 
       NA
 
       NA
  
 
  
 1
 
 228509380
 
 228509762
 
  ENSEMBL   UCSC 
 
  383
 
  5
 
 1053
 
 gene;island
 
 0.58772
 
 0.77821
 
 Not queried
 
  1.0411385
 
       NA
 
       NA
  
 
  
 X
 
  30671630
 
  30671787
 
  ENSEMBL   UCSC 
 
  158
 
  4
 
 40956
 
 gene;island
 
 0.11241
 
 0.20709
 
 Not queried
 
  1.0408491
 
       NA
 
       NA
  
 
  
 6
 
 118241243
 
 118241659
 
  ENSEMBL   UCSC 
 
  417
 
  5
 
 34391
 
 gene;island
 
 0.04310
 
 0.04413
 
 Not queried
 
  1.0406715
 
       NA
 
       NA
  
 
  
 5
 
  10250999
 
  10251252
 
  ENSEMBL   UCSC 
 
  254
 
  4
 
 31503
 
 gene;tss;island
 
 0.01988
 
 0.03938
 
 Not queried
 
  1.0406670
 
       NA
 
       NA
  
 
  
 X
 
  40506749
 
  40507041
 
  ENSEMBL   UCSC 
 
  293
 
  8
 
 41725
 
 island
 
 0.03471
 
 0.02014
 
 Not queried
 
  1.0406597
 
       NA
 
       NA
  
 
  
 17
 
   7402445
 
   7402673
 
  ENSEMBL   UCSC 
 
  229
 
  6
 
 16363
 
 gene
 
 0.94900
 
 0.94188
 
 Not queried
 
  1.0405097
 
       NA
 
       NA
  
 
  
 6
 
  31869982
 
  31870990
 
  ENSEMBL   UCSC 
 
 1009
 
 21
 
 34787
 
 tss;island
 
 0.08521
 
 0.06530
 
 Not queried
 
  1.0404966
 
       NA
 
       NA
  
 
  
 7
 
  39989474
 
  39989867
 
  ENSEMBL   UCSC 
 
  394
 
  6
 
 37483
 
 tss
 
 0.00410
 
 0.00936
 
 Not queried
 
  1.0402277
 
       NA
 
       NA
  
 
  
 2
 
  95522774
 
  95522902
 
  ENSEMBL   UCSC 
 
  129
 
  4
 
 24471
 
 island
 
 0.27731
 
 0.40218
 
 Not queried
 
  1.0398391
 
       NA
 
       NA
  
 
  
 4
 
    619162
 
    619314
 
  ENSEMBL   UCSC 
 
  153
 
  4
 
 30179
 
 tss
 
 0.71983
 
 0.87586
 
 Not queried
 
  1.0398359
 
       NA
 
       NA
  
 
  
 19
 
  58789751
 
  58790439
 
  ENSEMBL   UCSC 
 
  689
 
 12
 
 22464
 
 island
 
 0.02130
 
 0.03014
 
 Not queried
 
  1.0397786
 
       NA
 
       NA
  
 
  
 2
 
 214016774
 
 214017273
 
  ENSEMBL   UCSC 
 
  500
 
  4
 
 23820
 
 tss;island
 
 0.13758
 
 0.07514
 
 Not queried
 
  1.0397691
 
       NA
 
       NA
  
 
  
 10
 
  38146040
 
  38146444
 
  ENSEMBL   UCSC 
 
  405
 
  6
 
 4546
 
 gene
 
 0.03911
 
 0.04074
 
 Not queried
 
  1.0396812
 
       NA
 
       NA
  
 
  
 16
 
  87916134
 
  87916394
 
  ENSEMBL   UCSC 
 
  261
 
  4
 
 16153
 
 island
 
 0.89227
 
 0.78020
 
 Not queried
 
  1.0394730
 
       NA
 
       NA
  
 
  
 16
 
  83841324
 
  83841530
 
  ENSEMBL   UCSC 
 
  207
 
  5
 
 15356
 
 tss
 
 0.04275
 
 0.05974
 
 Not queried
 
  1.0394159
 
       NA
 
       NA
  
 
  
 X
 
  34150405
 
  34150583
 
  ENSEMBL   UCSC 
 
  179
 
  5
 
 41706
 
 island
 
 0.81591
 
 0.87074
 
 Not queried
 
  1.0389595
 
       NA
 
       NA
  
 
  
 6
 
  32053600
 
  32053748
 
  ENSEMBL   UCSC 
 
  149
 
  5
 
 33977
 
 gene;island
 
 0.62075
 
 0.41613
 
 Not queried
 
  1.0388311
 
       NA
 
       NA
  
 
  
 13
 
 114876061
 
 114876521
 
  ENSEMBL   UCSC 
 
  461
 
  4
 
 11287
 
 gene;island
 
 0.83102
 
 0.92017
 
 Not queried
 
  1.0388233
 
       NA
 
       NA
  
 
  
 1
 
  23857884
 
  23858035
 
  ENSEMBL   UCSC 
 
  152
 
  4
 
 1339
 
 tss;island
 
 0.02443
 
 0.03478
 
 Not queried
 
  1.0381236
 
       NA
 
       NA
  
 
  
 15
 
  90727783
 
  90728140
 
  ENSEMBL   UCSC 
 
  358
 
  7
 
 13868
 
 tss
 
 0.02228
 
 0.10525
 
 Not queried
 
  1.0379068
 
       NA
 
       NA
  
 
  
 22
 
  17178585
 
  17178751
 
  ENSEMBL   UCSC 
 
  167
 
  4
 
 27226
 
 island
 
 0.89072
 
 0.82751
 
 Not queried
 
  1.0377580
 
       NA
 
       NA
  
 
  
 11
 
  18433500
 
  18433745
 
  ENSEMBL   UCSC 
 
  246
 
  6
 
 7164
 
 tss
 
 0.57326
 
 0.72132
 
 Not queried
 
  1.0376955
 
       NA
 
       NA
  
 
  
 12
 
  25348252
 
  25348358
 
  ENSEMBL   UCSC 
 
  107
 
  5
 
 9060
 
 gene
 
 0.01842
 
 0.04742
 
 Not queried
 
  1.0373771
 
       NA
 
       NA
  
 
  
 8
 
  16884197
 
  16884576
 
  ENSEMBL   UCSC 
 
  380
 
  6
 
 39500
 
 tss;island
 
 0.00675
 
 0.01483
 
 Not queried
 
  1.0373738
 
       NA
 
       NA
  
 
  
 7
 
 157667762
 
 157668141
 
  ENSEMBL   UCSC 
 
  380
 
  5
 
 37284
 
 gene;island
 
 0.90162
 
 0.91227
 
 Not queried
 
  1.0371012
 
       NA
 
       NA
  
 
  
 6
 
  29580340
 
  29580367
 
  ENSEMBL   UCSC 
 
   28
 
  4
 
 33608
 
 gene
 
 0.94893
 
 0.97302
 
 Not queried
 
  1.0369737
 
       NA
 
       NA
  
 
  
 6
 
  33234201
 
  33234620
 
  ENSEMBL   UCSC 
 
  420
 
  8
 
 34173
 
 gene
 
 0.87520
 
 0.91434
 
 Not queried
 
  1.0369396
 
       NA
 
       NA
  
 
  
 2
 
 215275757
 
 215276325
 
  ENSEMBL   UCSC 
 
  569
 
  6
 
 23822
 
 tss
 
 0.02219
 
 0.04442
 
 Not queried
 
  1.0368565
 
       NA
 
       NA
  
 
  
 7
 
    194719
 
    194861
 
  ENSEMBL   UCSC 
 
  143
 
  4
 
 36640
 
 gene;island
 
 0.40309
 
 0.62100
 
 Not queried
 
  1.0365960
 
       NA
 
       NA
  
 
  
 16
 
  86588932
 
  86589283
 
  ENSEMBL   UCSC 
 
  352
 
 10
 
 14856
 
 gene;tss;island
 
 0.01795
 
 0.03109
 
 Not queried
 
  1.0363894
 
       NA
 
       NA
  
 
  
 6
 
  39760416
 
  39760833
 
  ENSEMBL   UCSC 
 
  418
 
  5
 
 36050
 
 island
 
 0.39500
 
 0.32916
 
 Not queried
 
  1.0362942
 
       NA
 
       NA
  
 
  
 15
 
  27111388
 
  27112430
 
  ENSEMBL   UCSC 
 
 1043
 
 24
 
 13937
 
 island
 
 0.64708
 
 0.42163
 
 Not queried
 
  1.0360291
 
       NA
 
       NA
  
 
  
 8
 
  56361901
 
  56362167
 
  ENSEMBL   UCSC 
 
  267
 
  4
 
 39245
 
 gene;island
 
 0.83651
 
 0.94250
 
 Not queried
 
  1.0346003
 
       NA
 
       NA
  
 
  
 6
 
  42145851
 
  42146143
 
  ENSEMBL   UCSC 
 
  293
 
  4
 
 34273
 
 gene;island
 
 0.07751
 
 0.03174
 
 Not queried
 
  1.0343836
 
       NA
 
       NA
  
 
  
 1
 
 202172778
 
 202172912
 
  ENSEMBL   UCSC 
 
  135
 
  4
 
 941
 
 gene
 
 0.70452
 
 0.59249
 
 Not queried
 
  1.0341747
 
       NA
 
       NA
  
 
  
 5
 
  39075035
 
  39075257
 
  ENSEMBL   UCSC 
 
  223
 
  5
 
 31973
 
 tss;island
 
 0.01149
 
 0.02860
 
 Not queried
 
  1.0341292
 
       NA
 
       NA
  
 
  
 7
 
  54609587
 
  54610320
 
  ENSEMBL   UCSC 
 
  734
 
 12
 
 38434
 
 island
 
 0.02039
 
 0.03093
 
 Not queried
 
  1.0340361
 
       NA
 
       NA
  
 
  
 14
 
 100150602
 
 100150722
 
  ENSEMBL   UCSC 
 
  121
 
  5
 
 12572
 
 tss
 
 0.03633
 
 0.03682
 
 Not queried
 
  1.0335383
 
       NA
 
       NA
  
 
  
 5
 
 140514811
 
 140515207
 
  ENSEMBL   UCSC 
 
  397
 
  5
 
 31751
 
 gene
 
 0.01005
 
 0.02005
 
 Not queried
 
  1.0335261
 
       NA
 
       NA
  
 
  
 X
 
 122696079
 
 122696417
 
  ENSEMBL   UCSC 
 
  339
 
  7
 
 41503
 
 tss
 
 0.87665
 
 0.77638
 
 Not queried
 
  1.0335053
 
       NA
 
       NA
  
 
  
 8
 
 110986685
 
 110987019
 
  ENSEMBL   UCSC 
 
  335
 
  5
 
 40477
 
 island
 
 0.01690
 
 0.05934
 
 Not queried
 
  1.0333364
 
       NA
 
       NA
  
 
  
 6
 
  33259866
 
  33260284
 
  ENSEMBL   UCSC 
 
  419
 
  8
 
 34188
 
 gene;island
 
 0.77536
 
 0.87514
 
 Not queried
 
  1.0331894
 
       NA
 
       NA
  
 
  
 19
 
  39971040
 
  39971347
 
  ENSEMBL   UCSC 
 
  308
 
  4
 
 21964
 
 island
 
 0.01962
 
 0.04342
 
 Not queried
 
  1.0329730
 
       NA
 
       NA
  
 
  
 1
 
   8378213
 
   8378538
 
  ENSEMBL   UCSC 
 
  326
 
  4
 
 2631
 
 island
 
 0.08193
 
 0.15724
 
 Not queried
 
  1.0325034
 
       NA
 
       NA
  
 
  
 16
 
   4466639
 
   4466888
 
  ENSEMBL   UCSC 
 
  250
 
  4
 
 15036
 
 tss
 
 0.01826
 
 0.02599
 
 Not queried
 
  1.0323213
 
       NA
 
       NA
  
 
  
 6
 
  29589415
 
  29590143
 
  ENSEMBL   UCSC 
 
  729
 
 14
 
 33612
 
 gene
 
 0.84739
 
 0.90983
 
 Not queried
 
  1.0318027
 
       NA
 
       NA
  
 
  
 1
 
   2100169
 
   2100435
 
  ENSEMBL   UCSC 
 
  267
 
  4
 
 66
 
 gene
 
 0.93315
 
 0.87626
 
 Not queried
 
  1.0317673
 
       NA
 
       NA
  
 
  
 19
 
  49558849
 
  49559064
 
  ENSEMBL   UCSC 
 
  216
 
  4
 
 22215
 
 island
 
 0.70607
 
 0.49963
 
 Not queried
 
  1.0314711
 
       NA
 
       NA
  
 
  
 X
 
  44703298
 
  44703983
 
  ENSEMBL   UCSC 
 
  686
 
  6
 
 40967
 
 gene
 
 0.82887
 
 0.90922
 
 Not queried
 
  1.0313949
 
       NA
 
       NA
  
 
  
 6
 
  41703314
 
  41703613
 
  ENSEMBL   UCSC 
 
  300
 
  4
 
 34267
 
 gene;tss;island
 
 0.03895
 
 0.05832
 
 Not queried
 
  1.0313544
 
       NA
 
       NA
  
 
  
 17
 
  73521354
 
  73521755
 
  ENSEMBL   UCSC 
 
  402
 
  4
 
 17622
 
 tss;island
 
 0.02330
 
 0.04664
 
 Not queried
 
  1.0310872
 
       NA
 
       NA
  
 
  
 1
 
 179544476
 
 179544765
 
  ENSEMBL   UCSC 
 
  290
 
  4
 
 902
 
 gene;island
 
 0.49088
 
 0.38865
 
 Not queried
 
  1.0309489
 
       NA
 
       NA
  
 
  
 6
 
 132931714
 
 132931926
 
  ENSEMBL   UCSC 
 
  213
 
  4
 
 35164
 
 tss
 
 0.75775
 
 0.84897
 
 Not queried
 
  1.0307312
 
       NA
 
       NA
  
 
  
 19
 
  51165207
 
  51165845
 
  ENSEMBL   UCSC 
 
  639
 
  6
 
 20144
 
 gene;island
 
 0.47992
 
 0.62870
 
 Not queried
 
  1.0306247
 
       NA
 
       NA
  
 
  
 X
 
  17754811
 
  17755305
 
  ENSEMBL   UCSC 
 
  495
 
  6
 
 41210
 
 tss;island
 
 0.23262
 
 0.11416
 
 Not queried
 
  1.0305777
 
       NA
 
       NA
  
 
  
 2
 
  51259693
 
  51260003
 
  ENSEMBL   UCSC 
 
  311
 
  6
 
 23311
 
 tss
 
 0.07507
 
 0.07608
 
 Not queried
 
  1.0305608
 
       NA
 
       NA
  
 
  
 4
 
   2795105
 
   2795501
 
  ENSEMBL   UCSC 
 
  397
 
  6
 
 29848
 
 gene;island
 
 0.12662
 
 0.23886
 
 Not queried
 
  1.0304907
 
       NA
 
       NA
  
 
  
 6
 
  32819858
 
  32821742
 
  ENSEMBL   UCSC 
 
 1885
 
 54
 
 34090
 
 gene
 
 0.06243
 
 0.28679
 
 Not queried
 
  1.0304665
 
       NA
 
       NA
  
 
  
 6
 
  32819858
 
  32822352
 
  ENSEMBL   UCSC 
 
 2495
 
 70
 
 35876
 
 island
 
 0.05634
 
 0.23534
 
 Not queried
 
  1.0304665
 
       NA
 
       NA
  
 
  
 18
 
  20513667
 
  20513759
 
  ENSEMBL   UCSC 
 
   93
 
  6
 
 19169
 
 tss
 
 0.01874
 
 0.04746
 
 Not queried
 
  1.0302206
 
       NA
 
       NA
  
 
  
 10
 
  61469748
 
  61469980
 
  ENSEMBL   UCSC 
 
  233
 
  8
 
 5077
 
 tss
 
 0.08286
 
 0.04942
 
 Not queried
 
  1.0298785
 
       NA
 
       NA
  
 
  
 15
 
  88799707
 
  88799999
 
  ENSEMBL   UCSC 
 
  293
 
  4
 
 13849
 
 tss
 
 0.01616
 
 0.02978
 
 Not queried
 
  1.0297198
 
       NA
 
       NA
  
 
  
 7
 
 117824011
 
 117824075
 
  ENSEMBL   UCSC 
 
   65
 
  4
 
 37793
 
 tss
 
 0.04206
 
 0.02903
 
 Not queried
 
  1.0294732
 
       NA
 
       NA
  
 
  
 4
 
 126237325
 
 126237597
 
  ENSEMBL   UCSC 
 
  273
 
  7
 
 31242
 
 island
 
 0.04564
 
 0.02787
 
 Not queried
 
  1.0293805
 
       NA
 
       NA
  
 
  
 8
 
  99956467
 
  99956585
 
  ENSEMBL   UCSC 
 
  119
 
  5
 
 39773
 
 tss
 
 0.02478
 
 0.03639
 
 Not queried
 
  1.0293081
 
       NA
 
       NA
  
 
  
 19
 
   6737385
 
   6738068
 
  ENSEMBL   UCSC 
 
  684
 
 15
 
 20357
 
 tss;island
 
 0.02754
 
 0.03348
 
 Not queried
 
  1.0287145
 
       NA
 
       NA
  
 
  
 6
 
 160679391
 
 160679811
 
  ENSEMBL   UCSC 
 
  421
 
  4
 
 34469
 
 gene
 
 0.92581
 
 0.85240
 
 Not queried
 
  1.0286306
 
       NA
 
       NA
  
 
  
 19
 
  57631118
 
  57631478
 
  ENSEMBL   UCSC 
 
  361
 
  9
 
 21068
 
 tss;island
 
 0.86210
 
 0.91925
 
 Not queried
 
  1.0284890
 
       NA
 
       NA
  
 
  
 15
 
  83680325
 
  83680570
 
  ENSEMBL   UCSC 
 
  246
 
  7
 
 14363
 
 island
 
 0.01189
 
 0.02491
 
 Not queried
 
  1.0284565
 
       NA
 
       NA
  
 
  
 14
 
  52118110
 
  52118390
 
  ENSEMBL   UCSC 
 
  281
 
  4
 
 12052
 
 gene;tss;island
 
 0.04052
 
 0.03175
 
 Not queried
 
  1.0283455
 
       NA
 
       NA
  
 
  
 2
 
 169658881
 
 169659182
 
  ENSEMBL   UCSC 
 
  302
 
  6
 
 22876
 
 gene
 
 0.85268
 
 0.86393
 
 Not queried
 
  1.0279864
 
       NA
 
       NA
  
 
  
 19
 
  35773231
 
  35773479
 
  ENSEMBL   UCSC 
 
  249
 
  5
 
 21866
 
 island
 
 0.66279
 
 0.74004
 
 Not queried
 
  1.0279228
 
       NA
 
       NA
  
 
  
 20
 
  55101001
 
  55101089
 
  ENSEMBL   UCSC 
 
   89
 
  4
 
 25798
 
 tss
 
 0.77707
 
 0.88093
 
 Not queried
 
  1.0278639
 
       NA
 
       NA
  
 
  
 8
 
  95487014
 
  95487732
 
  ENSEMBL   UCSC 
 
  719
 
 12
 
 40392
 
 island
 
 0.01454
 
 0.02171
 
 Not queried
 
  1.0278636
 
       NA
 
       NA
  
 
  
 3
 
  67704890
 
  67705285
 
  ENSEMBL   UCSC 
 
  396
 
  7
 
 29224
 
 island
 
 0.04069
 
 0.06710
 
 Not queried
 
  1.0274781
 
       NA
 
       NA
  
 
  
 20
 
  54824316
 
  54824646
 
  ENSEMBL   UCSC 
 
  331
 
  4
 
 25422
 
 gene;island
 
 0.16405
 
 0.37266
 
 Not queried
 
  1.0273661
 
       NA
 
       NA
  
 
  
 10
 
 102469807
 
 102470340
 
  ENSEMBL   UCSC 
 
  534
 
  5
 
 6012
 
 island
 
 0.03785
 
 0.07422
 
 Not queried
 
  1.0271590
 
       NA
 
       NA
  
 
  
 5
 
 138211071
 
 138211184
 
  ENSEMBL   UCSC 
 
  114
 
  5
 
 32267
 
 tss
 
 0.80848
 
 0.89586
 
 Not queried
 
  1.0270507
 
       NA
 
       NA
  
 
  
 12
 
 129028586
 
 129028733
 
  ENSEMBL   UCSC 
 
  148
 
  4
 
 9382
 
 gene
 
 0.81086
 
 0.64034
 
 Not queried
 
  1.0270218
 
       NA
 
       NA
  
 
  
 1
 
 232941468
 
 232941775
 
  ENSEMBL   UCSC 
 
  308
 
  4
 
 1078
 
 gene;island
 
 0.11823
 
 0.04168
 
 Not queried
 
  1.0266975
 
       NA
 
       NA
  
 
  
 19
 
   5455176
 
   5455386
 
  ENSEMBL   UCSC 
 
  211
 
  4
 
 20340
 
 tss
 
 0.88270
 
 0.78912
 
 Not queried
 
  1.0266413
 
       NA
 
       NA
  
 
  
 3
 
 183543564
 
 183544049
 
  ENSEMBL   UCSC 
 
  486
 
  6
 
 28705
 
 tss;island
 
 0.06642
 
 0.17901
 
 Not queried
 
  1.0264844
 
       NA
 
       NA
  
 
  
 4
 
  72897565
 
  72897868
 
  ENSEMBL   UCSC 
 
  304
 
  5
 
 29974
 
 gene;island
 
 0.02008
 
 0.04005
 
 Not queried
 
  1.0261811
 
       NA
 
       NA
  
 
  
 10
 
 106093685
 
 106093830
 
  ENSEMBL   UCSC 
 
  146
 
  6
 
 5331
 
 tss
 
 0.67840
 
 0.81718
 
 Not queried
 
  1.0261003
 
       NA
 
       NA
  
 
  
 1
 
  21877524
 
  21877781
 
  ENSEMBL   UCSC 
 
  258
 
  5
 
 297
 
 gene;tss
 
 0.86155
 
 0.91727
 
 Not queried
 
  1.0257451
 
       NA
 
       NA
  
 
  
 18
 
  55253948
 
  55254238
 
  ENSEMBL   UCSC 
 
  291
 
  7
 
 19499
 
 island
 
 0.01371
 
 0.03600
 
 Not queried
 
  1.0255415
 
       NA
 
       NA
  
 
  
 1
 
  34629098
 
  34629559
 
  ENSEMBL   UCSC 
 
  462
 
  5
 
 407
 
 gene;island
 
 0.09359
 
 0.24325
 
 Not queried
 
  1.0253856
 
       NA
 
       NA
  
 
  
 5
 
  17216259
 
  17216679
 
  ENSEMBL   UCSC 
 
  421
 
  6
 
 31940
 
 tss
 
 0.01068
 
 0.01802
 
 Not queried
 
  1.0253072
 
       NA
 
       NA
  
 
  
 20
 
   9494838
 
   9495199
 
  ENSEMBL   UCSC 
 
  362
 
  5
 
 25553
 
 tss
 
 0.04440
 
 0.02875
 
 Not queried
 
  1.0250168
 
       NA
 
       NA
  
 
  
 17
 
  46713842
 
  46714169
 
  ENSEMBL   UCSC 
 
  328
 
  4
 
 18460
 
 island
 
 0.14815
 
 0.08693
 
 Not queried
 
  1.0249555
 
       NA
 
       NA
  
 
  
 15
 
  73611079
 
  73611614
 
  ENSEMBL   UCSC 
 
  536
 
  6
 
 14259
 
 island
 
 0.45281
 
 0.34596
 
 Not queried
 
  1.0249495
 
       NA
 
       NA
  
 
  
 3
 
 113465485
 
 113465925
 
  ENSEMBL   UCSC 
 
  441
 
  7
 
 28466
 
 tss;island
 
 0.01138
 
 0.03511
 
 Not queried
 
  1.0249392
 
       NA
 
       NA
  
 
  
 17
 
   6756633
 
   6756891
 
  ENSEMBL   UCSC 
 
  259
 
  5
 
 17022
 
 tss
 
 0.77389
 
 0.75735
 
 Not queried
 
  1.0249066
 
       NA
 
       NA
  
 
  
 19
 
  35739448
 
  35739762
 
  ENSEMBL   UCSC 
 
  315
 
  5
 
 21865
 
 island
 
 0.01182
 
 0.01308
 
 Not queried
 
  1.0241923
 
       NA
 
       NA
  
 
  
 5
 
 179225479
 
 179225558
 
  ENSEMBL   UCSC 
 
   80
 
  5
 
 31877
 
 gene;tss;island
 
 0.72506
 
 0.80035
 
 Not queried
 
  1.0241738
 
       NA
 
       NA
  
 
  
 16
 
  29196472
 
  29196699
 
  ENSEMBL   UCSC 
 
  228
 
  4
 
 15768
 
 island
 
 0.75388
 
 0.87061
 
 Not queried
 
  1.0240136
 
       NA
 
       NA
  
 
  
 2
 
 223162666
 
 223162800
 
  ENSEMBL   UCSC 
 
  135
 
  4
 
 23877
 
 tss
 
 0.37145
 
 0.24660
 
 Not queried
 
  1.0239294
 
       NA
 
       NA
  
 
  
 12
 
 130526797
 
 130527212
 
  ENSEMBL   UCSC 
 
  416
 
  9
 
 10968
 
 island
 
 0.04377
 
 0.08515
 
 Not queried
 
  1.0238928
 
       NA
 
       NA
  
 
  
 16
 
  14724253
 
  14724468
 
  ENSEMBL   UCSC 
 
  216
 
  4
 
 15072
 
 tss;island
 
 0.01892
 
 0.05665
 
 Not queried
 
  1.0237288
 
       NA
 
       NA
  
 
  
 7
 
  39772772
 
  39773156
 
  ENSEMBL   UCSC 
 
  385
 
  9
 
 37482
 
 tss
 
 0.02060
 
 0.04519
 
 Not queried
 
  1.0236739
 
       NA
 
       NA
  
 
  
 10
 
  46090241
 
  46090807
 
  ENSEMBL   UCSC 
 
  567
 
  8
 
 5696
 
 island
 
 0.01442
 
 0.03497
 
 Not queried
 
  1.0236587
 
       NA
 
       NA
  
 
  
 6
 
  32016070
 
  32016803
 
  ENSEMBL   UCSC 
 
  734
 
 23
 
 33953
 
 gene
 
 0.35520
 
 0.48606
 
 Not queried
 
  1.0236561
 
       NA
 
       NA
  
 
  
 13
 
  50265738
 
  50265991
 
  ENSEMBL   UCSC 
 
  254
 
  7
 
 11415
 
 tss;island
 
 0.01007
 
 0.01990
 
 Not queried
 
  1.0234835
 
       NA
 
       NA
  
 
  
 20
 
  57765815
 
  57766071
 
  ENSEMBL   UCSC 
 
  257
 
  4
 
 25820
 
 tss;island
 
 0.71976
 
 0.90746
 
 Not queried
 
  1.0229800
 
       NA
 
       NA
  
 
  
 6
 
  30070738
 
  30071612
 
  ENSEMBL   UCSC 
 
  875
 
 27
 
 33657
 
 gene;island
 
 0.02340
 
 0.04086
 
 Not queried
 
  1.0229794
 
       NA
 
       NA
  
 
  
 19
 
  46390101
 
  46390327
 
  ENSEMBL   UCSC 
 
  227
 
  4
 
 20848
 
 tss;island
 
 0.02668
 
 0.03169
 
 Not queried
 
  1.0229731
 
       NA
 
       NA
  
 
  
 6
 
  26235224
 
  26235699
 
  ENSEMBL   UCSC 
 
  476
 
  8
 
 34634
 
 tss
 
 0.02382
 
 0.03301
 
 Not queried
 
  1.0228478
 
       NA
 
       NA
  
 
  
 11
 
  72353580
 
  72353604
 
  ENSEMBL   UCSC 
 
   25
 
  5
 
 7521
 
 tss
 
 0.10550
 
 0.08049
 
 Not queried
 
  1.0228458
 
       NA
 
       NA
  
 
  
 6
 
  32155079
 
  32155141
 
  ENSEMBL   UCSC 
 
   63
 
  4
 
 34017
 
 gene
 
 0.90515
 
 0.95109
 
 Not queried
 
  1.0226514
 
       NA
 
       NA
  
 
  
 2
 
 120281354
 
 120281999
 
  ENSEMBL   UCSC 
 
  646
 
  6
 
 22810
 
 gene
 
 0.01930
 
 0.03457
 
 Not queried
 
  1.0226448
 
       NA
 
       NA
  
 
  
 6
 
  31650735
 
  31651362
 
  ENSEMBL   UCSC 
 
  628
 
 21
 
 35760
 
 island
 
 0.17065
 
 0.03942
 
 Not queried
 
  1.0225080
 
       NA
 
       NA
  
 
  
 17
 
   6555093
 
   6555202
 
  ENSEMBL   UCSC 
 
  110
 
  4
 
 16340
 
 gene
 
 0.02614
 
 0.05590
 
 Not queried
 
  1.0224209
 
       NA
 
       NA
  
 
  
 19
 
   6712321
 
   6712593
 
  ENSEMBL   UCSC 
 
  273
 
  7
 
 19732
 
 gene;island
 
 0.71112
 
 0.86708
 
 Not queried
 
  1.0222404
 
       NA
 
       NA
  
 
  
 2
 
  84743142
 
  84743743
 
  ENSEMBL   UCSC 
 
  602
 
 12
 
 24431
 
 island
 
 0.01313
 
 0.04537
 
 Not queried
 
  1.0222177
 
       NA
 
       NA
  
 
  
 7
 
 100464958
 
 100465289
 
  ENSEMBL   UCSC 
 
  332
 
  4
 
 37086
 
 gene
 
 0.10606
 
 0.04422
 
 Not queried
 
  1.0220478
 
       NA
 
       NA
  
 
  
 9
 
  15422589
 
  15423182
 
  ENSEMBL   UCSC 
 
  594
 
  5
 
 40803
 
 island
 
 0.02006
 
 0.02983
 
 Not queried
 
  1.0217407
 
       NA
 
       NA
  
 
  
 8
 
 121137537
 
 121137879
 
  ENSEMBL   UCSC 
 
  343
 
  4
 
 39347
 
 gene;island
 
 0.03713
 
 0.07399
 
 Not queried
 
  1.0216180
 
       NA
 
       NA
  
 
  
 19
 
  58661689
 
  58661962
 
  ENSEMBL   UCSC 
 
  274
 
  4
 
 20220
 
 gene;island
 
 0.01478
 
 0.02920
 
 Not queried
 
  1.0215377
 
       NA
 
       NA
  
 
  
 19
 
   3869086
 
   3869599
 
  ENSEMBL   UCSC 
 
  514
 
  6
 
 20316
 
 tss;island
 
 0.16022
 
 0.18890
 
 Not queried
 
  1.0212595
 
       NA
 
       NA
  
 
  
 2
 
 219646316
 
 219646831
 
  ENSEMBL   UCSC 
 
  516
 
  5
 
 25056
 
 island
 
 0.04000
 
 0.02012
 
 Not queried
 
  1.0210591
 
       NA
 
       NA
  
 
  
 20
 
  18038510
 
  18038771
 
  ENSEMBL   UCSC 
 
  262
 
  5
 
 25981
 
 island
 
 0.02648
 
 0.05419
 
 Not queried
 
  1.0209956
 
       NA
 
       NA
  
 
  
 14
 
  37132291
 
  37132774
 
  ENSEMBL   UCSC 
 
  484
 
  5
 
 12038
 
 gene;island
 
 0.09771
 
 0.15737
 
 Not queried
 
  1.0207196
 
       NA
 
       NA
  
 
  
 10
 
  26222654
 
  26223310
 
  ENSEMBL   UCSC 
 
  657
 
  6
 
 5598
 
 island
 
 0.08649
 
 0.03796
 
 Not queried
 
  1.0203447
 
       NA
 
       NA
  
 
  
 10
 
    661009
 
    661730
 
  ENSEMBL   UCSC 
 
  722
 
  8
 
 4433
 
 gene
 
 0.71684
 
 0.89884
 
 Not queried
 
  1.0202956
 
       NA
 
       NA
  
 
  
 6
 
  32365491
 
  32366096
 
  ENSEMBL   UCSC 
 
  606
 
  7
 
 34045
 
 gene
 
 0.80435
 
 0.63095
 
 Not queried
 
  1.0201053
 
       NA
 
       NA
  
 
  
 15
 
  74315179
 
  74315710
 
  ENSEMBL   UCSC 
 
  532
 
  5
 
 13384
 
 gene;island
 
 0.92732
 
 0.85840
 
 Not queried
 
  1.0199334
 
       NA
 
       NA
  
 
  
 3
 
  50382952
 
  50383134
 
  ENSEMBL   UCSC 
 
  183
 
  4
 
 27765
 
 gene
 
 0.72904
 
 0.59708
 
 Not queried
 
  1.0198917
 
       NA
 
       NA
  
 
  
 19
 
  46800054
 
  46800602
 
  ENSEMBL   UCSC 
 
  549
 
  8
 
 20855
 
 tss;island
 
 0.04820
 
 0.04282
 
 Not queried
 
  1.0196636
 
       NA
 
       NA
  
 
  
 2
 
  12857398
 
  12857811
 
  ENSEMBL   UCSC 
 
  414
 
  4
 
 22539
 
 gene;island
 
 0.01054
 
 0.01921
 
 Not queried
 
  1.0193768
 
       NA
 
       NA
  
 
  
 7
 
  27225058
 
  27225897
 
  ENSEMBL   UCSC 
 
  840
 
 23
 
 36879
 
 gene
 
 0.10385
 
 0.19925
 
 Not queried
 
  1.0191291
 
       NA
 
       NA
  
 
  
 15
 
  85174433
 
  85174687
 
  ENSEMBL   UCSC 
 
  255
 
  6
 
 13841
 
 tss
 
 0.01072
 
 0.01853
 
 Not queried
 
  1.0188283
 
       NA
 
       NA
  
 
  
 15
 
  85174433
 
  85174859
 
  ENSEMBL   UCSC 
 
  427
 
  8
 
 14375
 
 island
 
 0.01072
 
 0.01691
 
 Not queried
 
  1.0188283
 
       NA
 
       NA
  
 
  
 20
 
  62205597
 
  62205981
 
  ENSEMBL   UCSC 
 
  385
 
  7
 
 25859
 
 tss
 
 0.02165
 
 0.06305
 
 Not queried
 
  1.0187913
 
       NA
 
       NA
  
 
  
 2
 
  42329446
 
  42329760
 
  ENSEMBL   UCSC 
 
  315
 
  5
 
 24201
 
 island
 
 0.35946
 
 0.44598
 
 Not queried
 
  1.0187450
 
       NA
 
       NA
  
 
  
 21
 
  34100294
 
  34100315
 
  ENSEMBL   UCSC 
 
   22
 
  5
 
 26359
 
 gene
 
 0.00940
 
 0.01660
 
 Not queried
 
  1.0187281
 
       NA
 
       NA
  
 
  
 21
 
  34100294
 
  34100595
 
  ENSEMBL   UCSC 
 
  302
 
  7
 
 26472
 
 tss;island
 
 0.01007
 
 0.01886
 
 Not queried
 
  1.0187281
 
       NA
 
       NA
  
 
  
 3
 
 157155088
 
 157155476
 
  ENSEMBL   UCSC 
 
  389
 
  5
 
 27965
 
 gene;island
 
 0.03582
 
 0.05664
 
 Not queried
 
  1.0187041
 
       NA
 
       NA
  
 
  
 11
 
  71823750
 
  71824165
 
  ENSEMBL   UCSC 
 
  416
 
  9
 
 8605
 
 island
 
 0.02620
 
 0.01428
 
 Not queried
 
  1.0186825
 
       NA
 
       NA
  
 
  
 13
 
 112721712
 
 112721950
 
  ENSEMBL   UCSC 
 
  239
 
  7
 
 11880
 
 island
 
 0.06978
 
 0.12283
 
 Not queried
 
  1.0182858
 
       NA
 
       NA
  
 
  
 10
 
  43904326
 
  43904553
 
  ENSEMBL   UCSC 
 
  228
 
  7
 
 4559
 
 gene;tss;island
 
 0.03042
 
 0.05276
 
 Not queried
 
  1.0182754
 
       NA
 
       NA
  
 
  
 15
 
  84115811
 
  84116018
 
  ENSEMBL   UCSC 
 
  208
 
  6
 
 13838
 
 tss
 
 0.01405
 
 0.02880
 
 Not queried
 
  1.0182685
 
       NA
 
       NA
  
 
  
 13
 
  41706942
 
  41707241
 
  ENSEMBL   UCSC 
 
  300
 
  8
 
 11381
 
 tss
 
 0.02782
 
 0.05471
 
 Not queried
 
  1.0181530
 
       NA
 
       NA
  
 
  
 8
 
 142238452
 
 142239055
 
  ENSEMBL   UCSC 
 
  604
 
  8
 
 40555
 
 island
 
 0.88603
 
 0.93059
 
 Not queried
 
  1.0180074
 
       NA
 
       NA
  
 
  
 3
 
 189348936
 
 189349129
 
  ENSEMBL   UCSC 
 
  194
 
  4
 
 28739
 
 tss
 
 0.72899
 
 0.81628
 
 Not queried
 
  1.0176950
 
       NA
 
       NA
  
 
  
 19
 
  49106849
 
  49107139
 
  ENSEMBL   UCSC 
 
  291
 
  4
 
 20093
 
 gene;island
 
 0.85471
 
 0.91800
 
 Not queried
 
  1.0176543
 
       NA
 
       NA
  
 
  
 5
 
   1253833
 
   1254048
 
  ENSEMBL   UCSC 
 
  216
 
  4
 
 31475
 
 gene;island
 
 0.78946
 
 0.90127
 
 Not queried
 
  1.0176222
 
       NA
 
       NA
  
 
  
 16
 
  69975149
 
  69975590
 
  ENSEMBL   UCSC 
 
  442
 
  4
 
 14796
 
 gene
 
 0.77444
 
 0.87797
 
 Not queried
 
  1.0176036
 
       NA
 
       NA
  
 
  
 7
 
 140396347
 
 140396458
 
  ENSEMBL   UCSC 
 
  112
 
  6
 
 37876
 
 tss
 
 0.01494
 
 0.02176
 
 Not queried
 
  1.0173362
 
       NA
 
       NA
  
 
  
 5
 
  36607333
 
  36607417
 
  ENSEMBL   UCSC 
 
   85
 
  4
 
 31531
 
 gene;tss
 
 0.03331
 
 0.01671
 
 Not queried
 
  1.0172814
 
       NA
 
       NA
  
 
  
 7
 
  98972104
 
  98972197
 
  ENSEMBL   UCSC 
 
   94
 
  6
 
 37687
 
 tss;island
 
 0.07162
 
 0.02604
 
 Not queried
 
  1.0171719
 
       NA
 
       NA
  
 
  
 6
 
  32795705
 
  32796216
 
  ENSEMBL   UCSC 
 
  512
 
  4
 
 34071
 
 gene
 
 0.63001
 
 0.47921
 
 Not queried
 
  1.0170857
 
       NA
 
       NA
  
 
  
 17
 
  80350140
 
  80350405
 
  ENSEMBL   UCSC 
 
  266
 
  5
 
 16911
 
 gene
 
 0.85556
 
 0.91521
 
 Not queried
 
  1.0169322
 
       NA
 
       NA
  
 
  
 6
 
  31130221
 
  31130548
 
  ENSEMBL   UCSC 
 
  328
 
 12
 
 33799
 
 gene;island
 
 0.58436
 
 0.87192
 
 Not queried
 
  1.0169053
 
       NA
 
       NA
  
 
  
 19
 
  10220752
 
  10221146
 
  ENSEMBL   UCSC 
 
  395
 
  5
 
 19772
 
 gene;tss;island
 
 0.81715
 
 0.90043
 
 Not queried
 
  1.0168389
 
       NA
 
       NA
  
 
  
 8
 
  26724744
 
  26725033
 
  ENSEMBL   UCSC 
 
  290
 
  5
 
 40112
 
 island
 
 0.01589
 
 0.04350
 
 Not queried
 
  1.0167829
 
       NA
 
       NA
  
 
  
 11
 
  59578351
 
  59578540
 
  ENSEMBL   UCSC 
 
  190
 
  5
 
 7298
 
 tss
 
 0.09590
 
 0.04121
 
 Not queried
 
  1.0167584
 
       NA
 
       NA
  
 
  
 1
 
 153030514
 
 153030751
 
  ENSEMBL   UCSC 
 
  238
 
  4
 
 1935
 
 tss
 
 0.23109
 
 0.28437
 
 Not queried
 
  1.0167338
 
       NA
 
       NA
  
 
  
 6
 
  32181764
 
  32182326
 
  ENSEMBL   UCSC 
 
  563
 
  6
 
 34031
 
 gene
 
 0.60185
 
 0.42465
 
 Not queried
 
  1.0165376
 
       NA
 
       NA
  
 
  
 19
 
  44529223
 
  44529477
 
  ENSEMBL   UCSC 
 
  255
 
  7
 
 20804
 
 tss
 
 0.01700
 
 0.00925
 
 Not queried
 
  1.0164042
 
       NA
 
       NA
  
 
  
 3
 
 137905836
 
 137906122
 
  ENSEMBL   UCSC 
 
  287
 
  7
 
 28577
 
 tss
 
 0.01332
 
 0.02327
 
 Not queried
 
  1.0163165
 
       NA
 
       NA
  
 
  
 15
 
  75628126
 
  75628553
 
  ENSEMBL   UCSC 
 
  428
 
  8
 
 14299
 
 island
 
 0.03534
 
 0.16375
 
 Not queried
 
  1.0157617
 
       NA
 
       NA
  
 
  
 12
 
 130526916
 
 130527212
 
  ENSEMBL   UCSC 
 
  297
 
  8
 
 10091
 
 tss
 
 0.05408
 
 0.11364
 
 Not queried
 
  1.0157575
 
       NA
 
       NA
  
 
  
 2
 
  11774142
 
  11774474
 
  ENSEMBL   UCSC 
 
  333
 
  5
 
 22536
 
 gene;island
 
 0.87887
 
 0.87126
 
 Not queried
 
  1.0155313
 
       NA
 
       NA
  
 
  
 22
 
  49376354
 
  49376628
 
  ENSEMBL   UCSC 
 
  275
 
  4
 
 27560
 
 island
 
 0.90439
 
 0.81739
 
 Not queried
 
  1.0155004
 
       NA
 
       NA
  
 
  
 13
 
  78271874
 
  78271894
 
  ENSEMBL   UCSC 
 
   21
 
  4
 
 11457
 
 tss;island
 
 0.02621
 
 0.03411
 
 Not queried
 
  1.0154360
 
       NA
 
       NA
  
 
  
 1
 
  33722125
 
  33722135
 
  ENSEMBL   UCSC 
 
   11
 
  4
 
 1469
 
 tss;island
 
 0.00979
 
 0.03419
 
 Not queried
 
  1.0152701
 
       NA
 
       NA
  
 
  
 1
 
   6321181
 
   6321737
 
  ENSEMBL   UCSC 
 
  557
 
  7
 
 1200
 
 tss;island
 
 0.03035
 
 0.08025
 
 Not queried
 
  1.0151000
 
       NA
 
       NA
  
 
  
 9
 
 116861288
 
 116861532
 
  ENSEMBL   UCSC 
 
  245
 
  4
 
 40867
 
 island
 
 0.03069
 
 0.09569
 
 Not queried
 
  1.0148747
 
       NA
 
       NA
  
 
  
 4
 
 140477613
 
 140477727
 
  ENSEMBL   UCSC 
 
  115
 
  5
 
 30554
 
 tss
 
 0.01335
 
 0.02771
 
 Not queried
 
  1.0148336
 
       NA
 
       NA
  
 
  
 10
 
  89263900
 
  89264128
 
  ENSEMBL   UCSC 
 
  229
 
  6
 
 5185
 
 tss;island
 
 0.51013
 
 0.30901
 
 Not queried
 
  1.0146846
 
       NA
 
       NA
  
 
  
 7
 
   1199903
 
   1200143
 
  ENSEMBL   UCSC 
 
  241
 
  5
 
 37332
 
 tss
 
 0.01363
 
 0.02185
 
 Not queried
 
  1.0144101
 
       NA
 
       NA
  
 
  
 10
 
 135122580
 
 135123421
 
  ENSEMBL   UCSC 
 
  842
 
 13
 
 4884
 
 gene;island
 
 0.04562
 
 0.06561
 
 Not queried
 
  1.0141670
 
       NA
 
       NA
  
 
  
 7
 
  30951064
 
  30951432
 
  ENSEMBL   UCSC 
 
  369
 
  8
 
 37457
 
 tss
 
 0.54816
 
 0.72473
 
 Not queried
 
  1.0138994
 
       NA
 
       NA
  
 
  
 4
 
    812387
 
    812684
 
  ENSEMBL   UCSC 
 
  298
 
  4
 
 29795
 
 gene;island
 
 0.72805
 
 0.82197
 
 Not queried
 
  1.0137165
 
       NA
 
       NA
  
 
  
 17
 
  27224533
 
  27225046
 
  ENSEMBL   UCSC 
 
  514
 
  9
 
 16467
 
 gene;island
 
 0.02889
 
 0.05312
 
 Not queried
 
  1.0136938
 
       NA
 
       NA
  
 
  
 17
 
  27224801
 
  27225046
 
  ENSEMBL   UCSC 
 
  246
 
  7
 
 17194
 
 tss
 
 0.06293
 
 0.05340
 
 Not queried
 
  1.0136938
 
       NA
 
       NA
  
 
  
 X
 
  49056661
 
  49057013
 
  ENSEMBL   UCSC 
 
  353
 
  9
 
 41314
 
 tss
 
 0.04881
 
 0.02260
 
 Not queried
 
  1.0135706
 
       NA
 
       NA
  
 
  
 6
 
   7911240
 
   7911480
 
  ENSEMBL   UCSC 
 
  241
 
  4
 
 34552
 
 tss;island
 
 0.06158
 
 0.02982
 
 Not queried
 
  1.0134423
 
       NA
 
       NA
  
 
  
 16
 
    539845
 
    540266
 
  ENSEMBL   UCSC 
 
  422
 
  5
 
 14503
 
 gene
 
 0.83087
 
 0.87686
 
 Not queried
 
  1.0133853
 
       NA
 
       NA
  
 
  
 1
 
  91176405
 
  91176810
 
  ENSEMBL   UCSC 
 
  406
 
  5
 
 3449
 
 island
 
 0.06683
 
 0.02961
 
 Not queried
 
  1.0132060
 
       NA
 
       NA
  
 
  
 X
 
 101410339
 
 101410928
 
  ENSEMBL   UCSC 
 
  590
 
  7
 
 41054
 
 gene
 
 0.01520
 
 0.04241
 
 Not queried
 
  1.0128846
 
       NA
 
       NA
  
 
  
 8
 
   1830682
 
   1830965
 
  ENSEMBL   UCSC 
 
  284
 
  5
 
 39119
 
 gene;island
 
 0.77112
 
 0.88403
 
 Not queried
 
  1.0126849
 
       NA
 
       NA
  
 
  
 13
 
  70682019
 
  70682324
 
  ENSEMBL   UCSC 
 
  306
 
  4
 
 11147
 
 gene;island
 
 0.04615
 
 0.15647
 
 Not queried
 
  1.0126263
 
       NA
 
       NA
  
 
  
 16
 
  70557093
 
  70557485
 
  ENSEMBL   UCSC 
 
  393
 
  6
 
 15310
 
 tss;island
 
 0.00786
 
 0.02779
 
 Not queried
 
  1.0123115
 
       NA
 
       NA
  
 
  
 19
 
  12035674
 
  12035877
 
  ENSEMBL   UCSC 
 
  204
 
  5
 
 20449
 
 tss
 
 0.01162
 
 0.04412
 
 Not queried
 
  1.0122928
 
       NA
 
       NA
  
 
  
 3
 
 197401399
 
 197401639
 
  ENSEMBL   UCSC 
 
  241
 
  4
 
 28061
 
 gene
 
 0.70217
 
 0.88378
 
 Not queried
 
  1.0122005
 
       NA
 
       NA
  
 
  
 16
 
  19179231
 
  19179512
 
  ENSEMBL   UCSC 
 
  282
 
  6
 
 15083
 
 tss;island
 
 0.02429
 
 0.04678
 
 Not queried
 
  1.0119401
 
       NA
 
       NA
  
 
  
 19
 
  59084942
 
  59085608
 
  ENSEMBL   UCSC 
 
  667
 
 14
 
 21116
 
 tss
 
 0.03437
 
 0.11054
 
 Not queried
 
  1.0118840
 
       NA
 
       NA
  
 
  
 5
 
 110062384
 
 110062473
 
  ENSEMBL   UCSC 
 
   90
 
  4
 
 32145
 
 tss
 
 0.01247
 
 0.03833
 
 Not queried
 
  1.0116739
 
       NA
 
       NA
  
 
  
 1
 
 207669922
 
 207670205
 
  ENSEMBL   UCSC 
 
  284
 
  4
 
 975
 
 gene;island
 
 0.10670
 
 0.21764
 
 Not queried
 
  1.0115563
 
       NA
 
       NA
  
 
  
 8
 
  95962352
 
  95962463
 
  ENSEMBL   UCSC 
 
  112
 
  5
 
 39757
 
 tss;island
 
 0.03121
 
 0.05887
 
 Not queried
 
  1.0113293
 
       NA
 
       NA
  
 
  
 2
 
 239973913
 
 239974232
 
  ENSEMBL   UCSC 
 
  320
 
  5
 
 23063
 
 gene;island
 
 0.91864
 
 0.90177
 
 Not queried
 
  1.0113234
 
       NA
 
       NA
  
 
  
 11
 
  62623202
 
  62623623
 
  ENSEMBL   UCSC 
 
  422
 
 12
 
 7351
 
 tss;island
 
 0.04284
 
 0.02040
 
 Not queried
 
  1.0110507
 
       NA
 
       NA
  
 
  
 1
 
 165667906
 
 165668349
 
  ENSEMBL   UCSC 
 
  444
 
  6
 
 2064
 
 tss
 
 0.06635
 
 0.02295
 
 Not queried
 
  1.0109577
 
       NA
 
       NA
  
 
  
 10
 
  42970618
 
  42970923
 
  ENSEMBL   UCSC 
 
  306
 
  6
 
 5023
 
 tss
 
 0.83575
 
 0.69388
 
 Not queried
 
  1.0109202
 
       NA
 
       NA
  
 
  
 17
 
  79430641
 
  79431121
 
  ENSEMBL   UCSC 
 
  481
 
  5
 
 16879
 
 gene;island
 
 0.77840
 
 0.87720
 
 Not queried
 
  1.0108844
 
       NA
 
       NA
  
 
  
 3
 
  27754391
 
  27754675
 
  ENSEMBL   UCSC 
 
  285
 
  5
 
 28904
 
 island
 
 0.02025
 
 0.03869
 
 Not queried
 
  1.0107672
 
       NA
 
       NA
  
 
  
 6
 
  33995939
 
  33996203
 
  ENSEMBL   UCSC 
 
  265
 
  5
 
 34235
 
 gene;island
 
 0.95245
 
 0.89920
 
 Not queried
 
  1.0107240
 
       NA
 
       NA
  
 
  
 15
 
  90792609
 
  90793056
 
  ENSEMBL   UCSC 
 
  448
 
  7
 
 14422
 
 island
 
 0.03962
 
 0.04559
 
 Not queried
 
  1.0105870
 
       NA
 
       NA
  
 
  
 10
 
 134751598
 
 134751800
 
  ENSEMBL   UCSC 
 
  203
 
  4
 
 4865
 
 gene
 
 0.67658
 
 0.83371
 
 Not queried
 
  1.0105268
 
       NA
 
       NA
  
 
  
 15
 
  74421523
 
  74421584
 
  ENSEMBL   UCSC 
 
   62
 
  4
 
 13386
 
 gene
 
 0.06218
 
 0.02708
 
 Not queried
 
  1.0101098
 
       NA
 
       NA
  
 
  
 15
 
  74421523
 
  74421935
 
  ENSEMBL   UCSC 
 
  413
 
 12
 
 13761
 
 tss;island
 
 0.07228
 
 0.02789
 
 Not queried
 
  1.0101098
 
       NA
 
       NA
  
 
  
 22
 
  45705512
 
  45705707
 
  ENSEMBL   UCSC 
 
  196
 
  6
 
 27180
 
 tss
 
 0.03139
 
 0.03830
 
 Not queried
 
  1.0100330
 
       NA
 
       NA
  
 
  
 2
 
     47150
 
     47452
 
  ENSEMBL   UCSC 
 
  303
 
  4
 
 23114
 
 tss;island
 
 0.20732
 
 0.07982
 
 Not queried
 
  1.0099798
 
       NA
 
       NA
  
 
  
 16
 
    604802
 
    605347
 
  ENSEMBL   UCSC 
 
  546
 
  6
 
 15432
 
 island
 
 0.81620
 
 0.92070
 
 Not queried
 
  1.0099565
 
       NA
 
       NA
  
 
  
 14
 
  24685205
 
  24685421
 
  ENSEMBL   UCSC 
 
  217
 
  6
 
 12709
 
 island
 
 0.01457
 
 0.03606
 
 Not queried
 
  1.0097191
 
       NA
 
       NA
  
 
  
 16
 
  46655096
 
  46655473
 
  ENSEMBL   UCSC 
 
  378
 
  6
 
 15862
 
 island
 
 0.00745
 
 0.01271
 
 Not queried
 
  1.0093719
 
       NA
 
       NA
  
 
  
 6
 
  33171765
 
  33172208
 
  ENSEMBL   UCSC 
 
  444
 
  5
 
 34161
 
 gene
 
 0.22574
 
 0.39801
 
 Not queried
 
  1.0091886
 
       NA
 
       NA
  
 
  
 11
 
  67806337
 
  67806437
 
  ENSEMBL   UCSC 
 
  101
 
  6
 
 7484
 
 tss
 
 0.03246
 
 0.06356
 
 Not queried
 
  1.0091441
 
       NA
 
       NA
  
 
  
 22
 
  26137738
 
  26137997
 
  ENSEMBL   UCSC 
 
  260
 
  5
 
 26990
 
 tss
 
 0.60763
 
 0.79215
 
 Not queried
 
  1.0088998
 
       NA
 
       NA
  
 
  
 8
 
   1906312
 
   1906498
 
  ENSEMBL   UCSC 
 
  187
 
  4
 
 39123
 
 gene
 
 0.84631
 
 0.93111
 
 Not queried
 
  1.0087679
 
       NA
 
       NA
  
 
  
 18
 
  44676656
 
  44676764
 
  ENSEMBL   UCSC 
 
  109
 
  4
 
 19074
 
 gene
 
 0.00664
 
 0.01310
 
 Not queried
 
  1.0087156
 
       NA
 
       NA
  
 
  
 18
 
  44676656
 
  44677236
 
  ENSEMBL   UCSC 
 
  581
 
 10
 
 19462
 
 island
 
 0.00880
 
 0.01634
 
 Not queried
 
  1.0087156
 
       NA
 
       NA
  
 
  
 X
 
 101410814
 
 101411275
 
  ENSEMBL   UCSC 
 
  462
 
  8
 
 41429
 
 tss
 
 0.02257
 
 0.03694
 
 Not queried
 
  1.0084411
 
       NA
 
       NA
  
 
  
 3
 
  58291814
 
  58292201
 
  ENSEMBL   UCSC 
 
  388
 
  8
 
 29192
 
 island
 
 0.02141
 
 0.04226
 
 Not queried
 
  1.0082022
 
       NA
 
       NA
  
 
  
 1
 
 221068730
 
 221068935
 
  ENSEMBL   UCSC 
 
  206
 
  4
 
 4213
 
 island
 
 0.86469
 
 0.93328
 
 Not queried
 
  1.0080804
 
       NA
 
       NA
  
 
  
 8
 
  11324048
 
  11324448
 
  ENSEMBL   UCSC 
 
  401
 
  5
 
 40016
 
 island
 
 0.01475
 
 0.05663
 
 Not queried
 
  1.0080393
 
       NA
 
       NA
  
 
  
 2
 
  33359198
 
  33359688
 
  ENSEMBL   UCSC 
 
  491
 
  6
 
 22604
 
 gene
 
 0.72713
 
 0.85629
 
 Not queried
 
  1.0079285
 
       NA
 
       NA
  
 
  
 11
 
 125772925
 
 125773242
 
  ENSEMBL   UCSC 
 
  318
 
  6
 
 7772
 
 tss;island
 
 0.01290
 
 0.02151
 
 Not queried
 
  1.0075527
 
       NA
 
       NA
  
 
  
 1
 
  48938017
 
  48938264
 
  ENSEMBL   UCSC 
 
  248
 
  5
 
 1608
 
 tss;island
 
 0.02055
 
 0.04246
 
 Not queried
 
  1.0074501
 
       NA
 
       NA
  
 
  
 19
 
   4483111
 
   4483577
 
  ENSEMBL   UCSC 
 
  467
 
  5
 
 19699
 
 gene;island
 
 0.90940
 
 0.95079
 
 Not queried
 
  1.0074480
 
       NA
 
       NA
  
 
  
 20
 
  62461159
 
  62461539
 
  ENSEMBL   UCSC 
 
  381
 
  4
 
 26332
 
 island
 
 0.02186
 
 0.03884
 
 Not queried
 
  1.0074412
 
       NA
 
       NA
  
 
  
 17
 
  56609534
 
  56609687
 
  ENSEMBL   UCSC 
 
  154
 
  5
 
 17505
 
 tss;island
 
 0.07905
 
 0.03375
 
 Not queried
 
  1.0072210
 
       NA
 
       NA
  
 
  
 1
 
 211667297
 
 211667751
 
  ENSEMBL   UCSC 
 
  455
 
  5
 
 2235
 
 tss
 
 0.43703
 
 0.86054
 
 Not queried
 
  1.0070184
 
       NA
 
       NA
  
 
  
 19
 
  17448797
 
  17449202
 
  ENSEMBL   UCSC 
 
  406
 
  4
 
 19881
 
 gene;island
 
 0.15614
 
 0.07573
 
 Not queried
 
  1.0069517
 
       NA
 
       NA
  
 
  
 2
 
 128284465
 
 128284730
 
  ENSEMBL   UCSC 
 
  266
 
  4
 
 23575
 
 tss;island
 
 0.02602
 
 0.01849
 
 Not queried
 
  1.0063168
 
       NA
 
       NA
  
 
  
 11
 
  66024375
 
  66025139
 
  ENSEMBL   UCSC 
 
  765
 
 15
 
 7438
 
 tss
 
 0.02864
 
 0.02526
 
 Not queried
 
  1.0061435
 
       NA
 
       NA
  
 
  
 8
 
 107782513
 
 107782734
 
  ENSEMBL   UCSC 
 
  222
 
  5
 
 39800
 
 tss
 
 0.53358
 
 0.74250
 
 Not queried
 
  1.0057552
 
       NA
 
       NA
  
 
  
 11
 
   1243947
 
   1244229
 
  ENSEMBL   UCSC 
 
  283
 
  4
 
 7019
 
 tss
 
 0.68271
 
 0.75385
 
 Not queried
 
  1.0054321
 
       NA
 
       NA
  
 
  
 19
 
  50173392
 
  50173685
 
  ENSEMBL   UCSC 
 
  294
 
  4
 
 20123
 
 gene;island
 
 0.62628
 
 0.75672
 
 Not queried
 
  1.0051395
 
       NA
 
       NA
  
 
  
 1
 
 201951076
 
 201951606
 
  ENSEMBL   UCSC 
 
  531
 
  6
 
 2168
 
 tss;island
 
 0.12298
 
 0.04220
 
 Not queried
 
  1.0050743
 
       NA
 
       NA
  
 
  
 11
 
  16759900
 
  16760091
 
  ENSEMBL   UCSC 
 
  192
 
  9
 
 7150
 
 tss
 
 0.02119
 
 0.03182
 
 Not queried
 
  1.0050276
 
       NA
 
       NA
  
 
  
 3
 
 160475035
 
 160475128
 
  ENSEMBL   UCSC 
 
   94
 
  4
 
 27974
 
 gene;island
 
 0.78069
 
 0.64396
 
 Not queried
 
  1.0049785
 
       NA
 
       NA
  
 
  
 4
 
 187984412
 
 187984718
 
  ENSEMBL   UCSC 
 
  307
 
  4
 
 31434
 
 island
 
 0.72361
 
 0.82217
 
 Not queried
 
  1.0047743
 
       NA
 
       NA
  
 
  
 2
 
  19562750
 
  19563449
 
  ENSEMBL   UCSC 
 
  700
 
 10
 
 24066
 
 island
 
 0.04137
 
 0.07018
 
 Not queried
 
  1.0046874
 
       NA
 
       NA
  
 
  
 15
 
  90777190
 
  90777350
 
  ENSEMBL   UCSC 
 
  161
 
  4
 
 13869
 
 tss;island
 
 0.05999
 
 0.11689
 
 Not queried
 
  1.0043234
 
       NA
 
       NA
  
 
  
 3
 
  50403130
 
  50403312
 
  ENSEMBL   UCSC 
 
  183
 
  4
 
 27767
 
 gene;island
 
 0.81938
 
 0.59070
 
 Not queried
 
  1.0040019
 
       NA
 
       NA
  
 
  
 16
 
    751312
 
    751603
 
  ENSEMBL   UCSC 
 
  292
 
  4
 
 14511
 
 gene;island
 
 0.29206
 
 0.39664
 
 Not queried
 
  1.0037384
 
       NA
 
       NA
  
 
  
 12
 
 121175417
 
 121175819
 
  ENSEMBL   UCSC 
 
  403
 
  4
 
 9333
 
 gene;island
 
 0.68830
 
 0.86887
 
 Not queried
 
  1.0035755
 
       NA
 
       NA
  
 
  
 19
 
  10397429
 
  10397780
 
  ENSEMBL   UCSC 
 
  352
 
  6
 
 21490
 
 island
 
 0.23643
 
 0.13897
 
 Not queried
 
  1.0031767
 
       NA
 
       NA
  
 
  
 4
 
  74809912
 
  74810310
 
  ENSEMBL   UCSC 
 
  399
 
  4
 
 31046
 
 island
 
 0.07507
 
 0.16983
 
 Not queried
 
  1.0029707
 
       NA
 
       NA
  
 
  
 1
 
   2391121
 
   2391347
 
  ENSEMBL   UCSC 
 
  227
 
  4
 
 2499
 
 island
 
 0.71224
 
 0.81486
 
 Not queried
 
  1.0028373
 
       NA
 
       NA
  
 
  
 9
 
 136039125
 
 136039706
 
  ENSEMBL   UCSC 
 
  582
 
  6
 
 40901
 
 island
 
 0.17143
 
 0.06437
 
 Not queried
 
  1.0027391
 
       NA
 
       NA
  
 
  
 1
 
  40943161
 
  40943386
 
  ENSEMBL   UCSC 
 
  226
 
  4
 
 3118
 
 island
 
 0.01222
 
 0.02023
 
 Not queried
 
  1.0026370
 
       NA
 
       NA
  
 
  
 17
 
  37355961
 
  37356047
 
  ENSEMBL   UCSC 
 
   87
 
  4
 
 17272
 
 tss;island
 
 0.13597
 
 0.03600
 
 Not queried
 
  1.0025764
 
       NA
 
       NA
  
 
  
 2
 
  74710011
 
  74710227
 
  ENSEMBL   UCSC 
 
  217
 
  4
 
 22704
 
 gene;island
 
 0.03302
 
 0.01676
 
 Not queried
 
  1.0025619
 
       NA
 
       NA
  
 
  
 12
 
 110283333
 
 110283596
 
  ENSEMBL   UCSC 
 
  264
 
  4
 
 10760
 
 island
 
 0.01546
 
 0.03555
 
 Not queried
 
  1.0025015
 
       NA
 
       NA
  
 
  
 9
 
 131486216
 
 131486918
 
  ENSEMBL   UCSC 
 
  703
 
  8
 
 40886
 
 island
 
 0.02995
 
 0.03898
 
 Not queried
 
  1.0024900
 
       NA
 
       NA
  
 
  
 11
 
    695873
 
    695896
 
  ENSEMBL   UCSC 
 
   24
 
  4
 
 6369
 
 gene;tss;island
 
 0.01801
 
 0.06064
 
 Not queried
 
  1.0023274
 
       NA
 
       NA
  
 
  
 14
 
 105190158
 
 105190481
 
  ENSEMBL   UCSC 
 
  324
 
  5
 
 12633
 
 tss
 
 0.02802
 
 0.04263
 
 Not queried
 
  1.0022513
 
       NA
 
       NA
  
 
  
 16
 
  70488291
 
  70488580
 
  ENSEMBL   UCSC 
 
  290
 
  5
 
 16036
 
 island
 
 0.01004
 
 0.02360
 
 Not queried
 
  1.0019666
 
       NA
 
       NA
  
 
  
 20
 
   2820742
 
   2821322
 
  ENSEMBL   UCSC 
 
  581
 
  8
 
 25301
 
 gene
 
 0.05689
 
 0.09511
 
 Not queried
 
  1.0018778
 
       NA
 
       NA
  
 
  
 13
 
 114202537
 
 114202861
 
  ENSEMBL   UCSC 
 
  325
 
  4
 
 11264
 
 gene;island
 
 0.85506
 
 0.92969
 
 Not queried
 
  1.0016804
 
       NA
 
       NA
  
 
  
 6
 
  27799295
 
  27799458
 
  ENSEMBL   UCSC 
 
  164
 
  7
 
 35505
 
 island
 
 0.01669
 
 0.02683
 
 Not queried
 
  1.0014542
 
       NA
 
       NA
  
 
  
 21
 
  38353114
 
  38353318
 
  ENSEMBL   UCSC 
 
  205
 
  4
 
 26379
 
 gene;island
 
 0.06015
 
 0.02627
 
 Not queried
 
  1.0013836
 
       NA
 
       NA
  
 
  
 7
 
   4822807
 
   4823097
 
  ENSEMBL   UCSC 
 
  291
 
  4
 
 36790
 
 gene;island
 
 0.78217
 
 0.83860
 
 Not queried
 
  1.0013750
 
       NA
 
       NA
  
 
  
 2
 
 160761262
 
 160761622
 
  ENSEMBL   UCSC 
 
  361
 
  6
 
 23645
 
 tss
 
 0.03281
 
 0.02305
 
 Not queried
 
  1.0013174
 
       NA
 
       NA
  
 
  
 17
 
   1880884
 
   1881333
 
  ENSEMBL   UCSC 
 
  450
 
  6
 
 16289
 
 gene;island
 
 0.78122
 
 0.86875
 
 Not queried
 
  1.0011770
 
       NA
 
       NA
  
 
  
 7
 
 143318009
 
 143318047
 
  ENSEMBL   UCSC 
 
   39
 
  4
 
 37891
 
 tss;island
 
 0.05297
 
 0.10121
 
 Not queried
 
  1.0011743
 
       NA
 
       NA
  
 
  
 3
 
  42201314
 
  42201649
 
  ENSEMBL   UCSC 
 
  336
 
  5
 
 27688
 
 gene;tss
 
 0.09519
 
 0.04322
 
 Not queried
 
  1.0010290
 
       NA
 
       NA
  
 
  
 19
 
  50643420
 
  50643731
 
  ENSEMBL   UCSC 
 
  312
 
  5
 
 20936
 
 tss
 
 0.81584
 
 0.69193
 
 Not queried
 
  1.0009476
 
       NA
 
       NA
  
 
  
 4
 
  38869118
 
  38869650
 
  ENSEMBL   UCSC 
 
  533
 
 12
 
 30299
 
 tss
 
 0.06719
 
 0.02719
 
 Not queried
 
  1.0007125
 
       NA
 
       NA
  
 
  
 1
 
 159824866
 
 159825173
 
  ENSEMBL   UCSC 
 
  308
 
  5
 
 835
 
 gene;island
 
 0.26938
 
 0.11608
 
 Not queried
 
  1.0006474
 
       NA
 
       NA
  
 
  
 19
 
  37701476
 
  37701738
 
  ENSEMBL   UCSC 
 
  263
 
  7
 
 20699
 
 tss
 
 0.02992
 
 0.01410
 
 Not queried
 
  1.0005014
 
       NA
 
       NA
  
 
  
 6
 
 134274008
 
 134274298
 
  ENSEMBL   UCSC 
 
  291
 
  5
 
 35169
 
 tss
 
 0.01943
 
 0.02886
 
 Not queried
 
  1.0004529
 
       NA
 
       NA
  
 
  
 5
 
 139944046
 
 139944509
 
  ENSEMBL   UCSC 
 
  464
 
 10
 
 32284
 
 tss;island
 
 0.01416
 
 0.02002
 
 Not queried
 
  1.0003816
 
       NA
 
       NA
  
 
  
 16
 
   2009084
 
   2009345
 
  ENSEMBL   UCSC 
 
  262
 
  4
 
 14980
 
 tss;island
 
 0.02762
 
 0.05694
 
 Not queried
 
  1.0002395
 
       NA
 
       NA
  
 
  
 7
 
   1423622
 
   1424047
 
  ENSEMBL   UCSC 
 
  426
 
  6
 
 38037
 
 island
 
 0.66328
 
 0.85706
 
 Not queried
 
  1.0002376
 
       NA
 
       NA
  
 
  
 3
 
 183852978
 
 183853494
 
  ENSEMBL   UCSC 
 
  517
 
  6
 
 28010
 
 gene
 
 0.02327
 
 0.03784
 
 Not queried
 
  1.0001605
 
       NA
 
       NA
  
 
  
 6
 
  31589593
 
  31589994
 
  ENSEMBL   UCSC 
 
  402
 
  5
 
 33846
 
 gene;tss;island
 
 0.02640
 
 0.05193
 
 Not queried
 
  0.9999976
 
       NA
 
       NA
  
 
  
 11
 
  18727552
 
  18727949
 
  ENSEMBL   UCSC 
 
  398
 
  4
 
 6514
 
 gene;island
 
 0.42089
 
 0.59153
 
 Not queried
 
  0.9997010
 
       NA
 
       NA
  
 
  
 5
 
 148810061
 
 148810203
 
  ENSEMBL   UCSC 
 
  143
 
  6
 
 31800
 
 gene;tss
 
 0.67839
 
 0.80279
 
 Not queried
 
  0.9994432
 
       NA
 
       NA
  
 
  
 12
 
   6716557
 
   6716597
 
  ENSEMBL   UCSC 
 
   41
 
  4
 
 9493
 
 tss;island
 
 0.01745
 
 0.04611
 
 Not queried
 
  0.9994018
 
       NA
 
       NA
  
 
  
 1
 
   2428864
 
   2429005
 
  ENSEMBL   UCSC 
 
  142
 
  4
 
 86
 
 gene
 
 0.67028
 
 0.79941
 
 Not queried
 
  0.9992849
 
       NA
 
       NA
  
 
  
 2
 
  36582038
 
  36582467
 
  ENSEMBL   UCSC 
 
  430
 
  5
 
 23263
 
 tss;island
 
 0.03873
 
 0.10037
 
 Not queried
 
  0.9989937
 
       NA
 
       NA
  
 
  
 2
 
 134785392
 
 134785775
 
  ENSEMBL   UCSC 
 
  384
 
  5
 
 24710
 
 island
 
 0.72950
 
 0.86904
 
 Not queried
 
  0.9989852
 
       NA
 
       NA
  
 
  
 7
 
  16460779
 
  16461244
 
  ENSEMBL   UCSC 
 
  466
 
  6
 
 38210
 
 island
 
 0.11434
 
 0.05456
 
 Not queried
 
  0.9989024
 
       NA
 
       NA
  
 
  
 21
 
  39644216
 
  39644354
 
  ENSEMBL   UCSC 
 
  139
 
  4
 
 26385
 
 gene;tss
 
 0.52392
 
 0.40624
 
 Not queried
 
  0.9986886
 
       NA
 
       NA
  
 
  
 1
 
  15943511
 
  15943714
 
  ENSEMBL   UCSC 
 
  204
 
  4
 
 1271
 
 tss;island
 
 0.02667
 
 0.02305
 
 Not queried
 
  0.9986157
 
       NA
 
       NA
  
 
  
 22
 
  35695066
 
  35695301
 
  ENSEMBL   UCSC 
 
  236
 
  4
 
 27056
 
 tss;island
 
 0.64325
 
 0.77448
 
 Not queried
 
  0.9984634
 
       NA
 
       NA
  
 
  
 15
 
  79164541
 
  79164980
 
  ENSEMBL   UCSC 
 
  440
 
  4
 
 13811
 
 tss;island
 
 0.04623
 
 0.03387
 
 Not queried
 
  0.9984499
 
       NA
 
       NA
  
 
  
 2
 
  74710460
 
  74710667
 
  ENSEMBL   UCSC 
 
  208
 
  7
 
 22705
 
 gene;tss;island
 
 0.01790
 
 0.03601
 
 Not queried
 
  0.9983833
 
       NA
 
       NA
  
 
  
 10
 
  70319998
 
  70320073
 
  ENSEMBL   UCSC 
 
   76
 
  5
 
 5097
 
 tss
 
 0.01634
 
 0.02654
 
 Not queried
 
  0.9981415
 
       NA
 
       NA
  
 
  
 17
 
   7486551
 
   7487593
 
  ENSEMBL   UCSC 
 
 1043
 
 14
 
 17926
 
 island
 
 0.19379
 
 0.10253
 
 Not queried
 
  0.9980269
 
       NA
 
       NA
  
 
  
 6
 
  41715166
 
  41715322
 
  ENSEMBL   UCSC 
 
  157
 
  4
 
 34907
 
 tss
 
 0.89106
 
 0.78850
 
 Not queried
 
  0.9976953
 
       NA
 
       NA
  
 
  
 11
 
  10715175
 
  10715445
 
  ENSEMBL   UCSC 
 
  271
 
  7
 
 6492
 
 gene
 
 0.66689
 
 0.81357
 
 Not queried
 
  0.9976876
 
       NA
 
       NA
  
 
  
 11
 
  10715175
 
  10715767
 
  ENSEMBL   UCSC 
 
  593
 
 11
 
 7127
 
 tss
 
 0.70405
 
 0.81357
 
 Not queried
 
  0.9976876
 
       NA
 
       NA
  
 
  
 16
 
  67918484
 
  67918965
 
  ENSEMBL   UCSC 
 
  482
 
  8
 
 16000
 
 island
 
 0.40929
 
 0.59328
 
 Not queried
 
  0.9973370
 
       NA
 
       NA
  
 
  
 17
 
   9674817
 
   9675212
 
  ENSEMBL   UCSC 
 
  396
 
  5
 
 16402
 
 gene;island
 
 0.84010
 
 0.72466
 
 Not queried
 
  0.9973271
 
       NA
 
       NA
  
 
  
 11
 
    368351
 
    369192
 
  ENSEMBL   UCSC 
 
  842
 
 24
 
 6989
 
 tss
 
 0.34376
 
 0.13152
 
 Not queried
 
  0.9970538
 
       NA
 
       NA
  
 
  
 10
 
  43634212
 
  43634491
 
  ENSEMBL   UCSC 
 
  280
 
  5
 
 4554
 
 gene;island
 
 0.01383
 
 0.02423
 
 Not queried
 
  0.9970380
 
       NA
 
       NA
  
 
  
 16
 
  57416050
 
  57416337
 
  ENSEMBL   UCSC 
 
  288
 
  5
 
 14735
 
 gene;island
 
 0.78108
 
 0.78309
 
 Not queried
 
  0.9967999
 
       NA
 
       NA
  
 
  
 17
 
  43972806
 
  43973522
 
  ENSEMBL   UCSC 
 
  717
 
  8
 
 16605
 
 gene;tss;island
 
 0.01955
 
 0.03728
 
 Not queried
 
  0.9967921
 
       NA
 
       NA
  
 
  
 10
 
 129924035
 
 129924700
 
  ENSEMBL   UCSC 
 
  666
 
  8
 
 6232
 
 island
 
 0.01009
 
 0.02159
 
 Not queried
 
  0.9967541
 
       NA
 
       NA
  
 
  
 19
 
  15281200
 
  15281607
 
  ENSEMBL   UCSC 
 
  408
 
  5
 
 19864
 
 gene;island
 
 0.89600
 
 0.79317
 
 Not queried
 
  0.9966417
 
       NA
 
       NA
  
 
  
 20
 
  33865797
 
  33866020
 
  ENSEMBL   UCSC 
 
  224
 
  4
 
 26081
 
 island
 
 0.01692
 
 0.02430
 
 Not queried
 
  0.9962288
 
       NA
 
       NA
  
 
  
 5
 
 125800764
 
 125800916
 
  ENSEMBL   UCSC 
 
  153
 
  4
 
 31677
 
 gene
 
 0.68396
 
 0.77989
 
 Not queried
 
  0.9962182
 
       NA
 
       NA
  
 
  
 6
 
  35265616
 
  35265759
 
  ENSEMBL   UCSC 
 
  144
 
  4
 
 34244
 
 gene
 
 0.04144
 
 0.08588
 
 Not queried
 
  0.9961911
 
       NA
 
       NA
  
 
  
 21
 
  47064079
 
  47064462
 
  ENSEMBL   UCSC 
 
  384
 
  4
 
 26722
 
 island
 
 0.13294
 
 0.05797
 
 Not queried
 
  0.9961635
 
       NA
 
       NA
  
 
  
 1
 
  36348216
 
  36348629
 
  ENSEMBL   UCSC 
 
  414
 
  6
 
 1486
 
 tss;island
 
 0.54804
 
 0.45874
 
 Not queried
 
  0.9960814
 
       NA
 
       NA
  
 
  
 7
 
    600452
 
    600658
 
  ENSEMBL   UCSC 
 
  207
 
  4
 
 36647
 
 gene;island
 
 0.80506
 
 0.86682
 
 Not queried
 
  0.9958983
 
       NA
 
       NA
  
 
  
 18
 
  23713407
 
  23713741
 
  ENSEMBL   UCSC 
 
  335
 
  6
 
 19182
 
 tss
 
 0.92080
 
 0.95861
 
 Not queried
 
  0.9955924
 
       NA
 
       NA
  
 
  
 5
 
   1409773
 
   1410090
 
  ENSEMBL   UCSC 
 
  318
 
  4
 
 31483
 
 gene
 
 0.35801
 
 0.51013
 
 Not queried
 
  0.9955173
 
       NA
 
       NA
  
 
  
 14
 
  74227020
 
  74227201
 
  ENSEMBL   UCSC 
 
  182
 
  6
 
 12471
 
 tss
 
 0.01410
 
 0.02788
 
 Not queried
 
  0.9954959
 
       NA
 
       NA
  
 
  
 11
 
 118306957
 
 118307152
 
  ENSEMBL   UCSC 
 
  196
 
  6
 
 7711
 
 tss;island
 
 0.01443
 
 0.02222
 
 Not queried
 
  0.9954332
 
       NA
 
       NA
  
 
  
 12
 
 133179887
 
 133180238
 
  ENSEMBL   UCSC 
 
  352
 
  4
 
 11037
 
 island
 
 0.66478
 
 0.79908
 
 Not queried
 
  0.9954052
 
       NA
 
       NA
  
 
  
 15
 
  74466704
 
  74467158
 
  ENSEMBL   UCSC 
 
  455
 
  6
 
 13389
 
 gene;island
 
 0.87511
 
 0.93005
 
 Not queried
 
  0.9953870
 
       NA
 
       NA
  
 
  
 16
 
    832683
 
    833250
 
  ENSEMBL   UCSC 
 
  568
 
 10
 
 15455
 
 island
 
 0.52073
 
 0.70751
 
 Not queried
 
  0.9953839
 
       NA
 
       NA
  
 
  
 1
 
  15850456
 
  15850746
 
  ENSEMBL   UCSC 
 
  291
 
  5
 
 245
 
 gene
 
 0.02191
 
 0.01787
 
 Not queried
 
  0.9953308
 
       NA
 
       NA
  
 
  
 5
 
 168174689
 
 168174935
 
  ENSEMBL   UCSC 
 
  247
 
  4
 
 31826
 
 gene
 
 0.62254
 
 0.68667
 
 Not queried
 
  0.9953158
 
       NA
 
       NA
  
 
  
 10
 
 106093659
 
 106093830
 
  ENSEMBL   UCSC 
 
  172
 
  7
 
 6100
 
 island
 
 0.79492
 
 0.91742
 
 Not queried
 
  0.9950644
 
       NA
 
       NA
  
 
  
 10
 
  70166985
 
  70167018
 
  ENSEMBL   UCSC 
 
   34
 
  4
 
 4594
 
 gene
 
 0.01672
 
 0.00996
 
 Not queried
 
  0.9950611
 
       NA
 
       NA
  
 
  
 18
 
  61637105
 
  61637441
 
  ENSEMBL   UCSC 
 
  337
 
  4
 
 19523
 
 island
 
 0.04215
 
 0.08376
 
 Not queried
 
  0.9949838
 
       NA
 
       NA
  
 
  
 10
 
 111767584
 
 111767685
 
  ENSEMBL   UCSC 
 
  102
 
  5
 
 5336
 
 tss
 
 0.04253
 
 0.04791
 
 Not queried
 
  0.9949838
 
       NA
 
       NA
  
 
  
 16
 
   1464758
 
   1465116
 
  ENSEMBL   UCSC 
 
  359
 
  6
 
 14963
 
 tss
 
 0.11744
 
 0.21010
 
 Not queried
 
  0.9948001
 
       NA
 
       NA
  
 
  
 6
 
  88875236
 
  88875651
 
  ENSEMBL   UCSC 
 
  416
 
  5
 
 34348
 
 gene
 
 0.02056
 
 0.03151
 
 Not queried
 
  0.9946884
 
       NA
 
       NA
  
 
  
 10
 
  44069847
 
  44070200
 
  ENSEMBL   UCSC 
 
  354
 
  7
 
 5682
 
 island
 
 0.05899
 
 0.04441
 
 Not queried
 
  0.9946802
 
       NA
 
       NA
  
 
  
 4
 
 154713235
 
 154713496
 
  ENSEMBL   UCSC 
 
  262
 
  4
 
 31324
 
 island
 
 0.05503
 
 0.08588
 
 Not queried
 
  0.9946567
 
       NA
 
       NA
  
 
  
 8
 
  67090752
 
  67090928
 
  ENSEMBL   UCSC 
 
  177
 
  5
 
 39676
 
 tss
 
 0.61824
 
 0.47706
 
 Not queried
 
  0.9945578
 
       NA
 
       NA
  
 
  
 6
 
  30709428
 
  30709924
 
  ENSEMBL   UCSC 
 
  497
 
  5
 
 33759
 
 gene;island
 
 0.30682
 
 0.07996
 
 Not queried
 
  0.9941726
 
       NA
 
       NA
  
 
  
 6
 
 106441441
 
 106441618
 
  ENSEMBL   UCSC 
 
  178
 
  5
 
 36318
 
 island
 
 0.05414
 
 0.03350
 
 Not queried
 
  0.9940834
 
       NA
 
       NA
  
 
  
 8
 
  49647907
 
  49648363
 
  ENSEMBL   UCSC 
 
  457
 
  5
 
 39628
 
 tss
 
 0.10902
 
 0.13476
 
 Not queried
 
  0.9937674
 
       NA
 
       NA
  
 
  
 6
 
 100916916
 
 100917427
 
  ENSEMBL   UCSC 
 
  512
 
  7
 
 36306
 
 island
 
 0.23688
 
 0.07800
 
 Not queried
 
  0.9936115
 
       NA
 
       NA
  
 
  
 18
 
  30352975
 
  30353283
 
  ENSEMBL   UCSC 
 
  309
 
  7
 
 19193
 
 tss
 
 0.08307
 
 0.12172
 
 Not queried
 
  0.9935043
 
       NA
 
       NA
  
 
  
 5
 
 180650776
 
 180651052
 
  ENSEMBL   UCSC 
 
  277
 
  4
 
 31892
 
 gene;island
 
 0.04613
 
 0.06896
 
 Not queried
 
  0.9933442
 
       NA
 
       NA
  
 
  
 17
 
  74463439
 
  74463767
 
  ENSEMBL   UCSC 
 
  329
 
  6
 
 16752
 
 gene
 
 0.54571
 
 0.66406
 
 Not queried
 
  0.9932600
 
       NA
 
       NA
  
 
  
 17
 
  74463439
 
  74463585
 
  ENSEMBL   UCSC 
 
  147
 
  4
 
 17646
 
 tss
 
 0.46614
 
 0.63005
 
 Not queried
 
  0.9932600
 
       NA
 
       NA
  
 
  
 15
 
  49255364
 
  49256042
 
  ENSEMBL   UCSC 
 
  679
 
  8
 
 14097
 
 island
 
 0.03462
 
 0.05735
 
 Not queried
 
  0.9931696
 
       NA
 
       NA
  
 
  
 6
 
  29598285
 
  29598417
 
  ENSEMBL   UCSC 
 
  133
 
  5
 
 33617
 
 gene;island
 
 0.73488
 
 0.56023
 
 Not queried
 
  0.9931405
 
       NA
 
       NA
  
 
  
 14
 
  20903410
 
  20903611
 
  ENSEMBL   UCSC 
 
  202
 
  4
 
 11976
 
 gene;island
 
 0.84595
 
 0.92338
 
 Not queried
 
  0.9928085
 
       NA
 
       NA
  
 
  
 12
 
 115134148
 
 115136308
 
  ENSEMBL   UCSC 
 
 2161
 
 54
 
 10822
 
 island
 
 0.13597
 
 0.05761
 
 Not queried
 
  0.9926001
 
       NA
 
       NA
  
 
  
 1
 
  39492095
 
  39492570
 
  ENSEMBL   UCSC 
 
  476
 
  6
 
 444
 
 gene
 
 0.01614
 
 0.02510
 
 Not queried
 
  0.9925118
 
       NA
 
       NA
  
 
  
 17
 
  62339926
 
  62340143
 
  ENSEMBL   UCSC 
 
  218
 
  4
 
 16697
 
 gene;island
 
 0.01937
 
 0.02437
 
 Not queried
 
  0.9923052
 
       NA
 
       NA
  
 
  
 6
 
  17600158
 
  17600386
 
  ENSEMBL   UCSC 
 
  229
 
  6
 
 34591
 
 tss
 
 0.04293
 
 0.04897
 
 Not queried
 
  0.9921198
 
       NA
 
       NA
  
 
  
 20
 
   2844659
 
   2844713
 
  ENSEMBL   UCSC 
 
   55
 
  5
 
 25515
 
 tss
 
 0.74050
 
 0.86594
 
 Not queried
 
  0.9919901
 
       NA
 
       NA
  
 
  
 6
 
  31539973
 
  31540169
 
  ENSEMBL   UCSC 
 
  197
 
 11
 
 33837
 
 gene
 
 0.92280
 
 0.85517
 
 Not queried
 
  0.9918526
 
       NA
 
       NA
  
 
  
 6
 
  13814875
 
  13815054
 
  ENSEMBL   UCSC 
 
  180
 
  5
 
 34581
 
 tss;island
 
 0.01719
 
 0.01895
 
 Not queried
 
  0.9918413
 
       NA
 
       NA
  
 
  
 16
 
  88459356
 
  88459714
 
  ENSEMBL   UCSC 
 
  359
 
  5
 
 16164
 
 island
 
 0.45277
 
 0.62739
 
 Not queried
 
  0.9918266
 
       NA
 
       NA
  
 
  
 1
 
 246670726
 
 246670940
 
  ENSEMBL   UCSC 
 
  215
 
  7
 
 2358
 
 tss;island
 
 0.01848
 
 0.03609
 
 Not queried
 
  0.9918061
 
       NA
 
       NA
  
 
  
 11
 
    299545
 
    299668
 
  ENSEMBL   UCSC 
 
  124
 
  5
 
 6987
 
 tss
 
 0.69751
 
 0.87228
 
 Not queried
 
  0.9914997
 
       NA
 
       NA
  
 
  
 17
 
  27055507
 
  27055725
 
  ENSEMBL   UCSC 
 
  219
 
  5
 
 17189
 
 tss;island
 
 0.01480
 
 0.01642
 
 Not queried
 
  0.9913668
 
       NA
 
       NA
  
 
  
 X
 
  11683977
 
  11684119
 
  ENSEMBL   UCSC 
 
  143
 
  4
 
 41183
 
 tss;island
 
 0.01857
 
 0.02642
 
 Not queried
 
  0.9913477
 
       NA
 
       NA
  
 
  
 16
 
  11438938
 
  11439288
 
  ENSEMBL   UCSC 
 
  351
 
  5
 
 15062
 
 tss;island
 
 0.00896
 
 0.01410
 
 Not queried
 
  0.9911428
 
       NA
 
       NA
  
 
  
 19
 
  44455140
 
  44455605
 
  ENSEMBL   UCSC 
 
  466
 
 10
 
 22063
 
 island
 
 0.02104
 
 0.03457
 
 Not queried
 
  0.9911227
 
       NA
 
       NA
  
 
  
 4
 
 147867081
 
 147867360
 
  ENSEMBL   UCSC 
 
  280
 
  6
 
 30577
 
 tss;island
 
 0.03912
 
 0.06633
 
 Not queried
 
  0.9908811
 
       NA
 
       NA
  
 
  
 8
 
 145662110
 
 145662334
 
  ENSEMBL   UCSC 
 
  225
 
  5
 
 39461
 
 gene;island
 
 0.73224
 
 0.85689
 
 Not queried
 
  0.9908202
 
       NA
 
       NA
  
 
  
 19
 
  44079778
 
  44079985
 
  ENSEMBL   UCSC 
 
  208
 
  7
 
 20795
 
 tss
 
 0.01479
 
 0.02897
 
 Not queried
 
  0.9905971
 
       NA
 
       NA
  
 
  
 5
 
 137549033
 
 137549144
 
  ENSEMBL   UCSC 
 
  112
 
  6
 
 32257
 
 tss
 
 0.00760
 
 0.01696
 
 Not queried
 
  0.9900805
 
       NA
 
       NA
  
 
  
 15
 
  41185800
 
  41186038
 
  ENSEMBL   UCSC 
 
  239
 
  4
 
 13571
 
 tss;island
 
 0.07828
 
 0.18281
 
 Not queried
 
  0.9898795
 
       NA
 
       NA
  
 
  
 1
 
 247275735
 
 247276096
 
  ENSEMBL   UCSC 
 
  362
 
  5
 
 2363
 
 tss
 
 0.14856
 
 0.13195
 
 Not queried
 
  0.9897683
 
       NA
 
       NA
  
 
  
 X
 
 100074813
 
 100075588
 
  ENSEMBL   UCSC 
 
  776
 
 11
 
 41904
 
 island
 
 0.01053
 
 0.02385
 
 Not queried
 
  0.9896785
 
       NA
 
       NA
  
 
  
 X
 
  19140934
 
  19141251
 
  ENSEMBL   UCSC 
 
  318
 
  4
 
 41215
 
 tss;island
 
 0.05682
 
 0.07617
 
 Not queried
 
  0.9894446
 
       NA
 
       NA
  
 
  
 6
 
 170456806
 
 170457111
 
  ENSEMBL   UCSC 
 
  306
 
  5
 
 36617
 
 island
 
 0.90054
 
 0.88093
 
 Not queried
 
  0.9893357
 
       NA
 
       NA
  
 
  
 8
 
  98636709
 
  98636900
 
  ENSEMBL   UCSC 
 
  192
 
  4
 
 40412
 
 island
 
 0.73349
 
 0.85978
 
 Not queried
 
  0.9890327
 
       NA
 
       NA
  
 
  
 18
 
  31020418
 
  31021065
 
  ENSEMBL   UCSC 
 
  648
 
  8
 
 19194
 
 tss;island
 
 0.08782
 
 0.05201
 
 Not queried
 
  0.9889026
 
       NA
 
       NA
  
 
  
 6
 
  73972704
 
  73972852
 
  ENSEMBL   UCSC 
 
  149
 
  4
 
 34321
 
 gene
 
 0.04208
 
 0.03349
 
 Not queried
 
  0.9888062
 
       NA
 
       NA
  
 
  
 11
 
   2481857
 
   2482594
 
  ENSEMBL   UCSC 
 
  738
 
 15
 
 7056
 
 tss
 
 0.71127
 
 0.79050
 
 Not queried
 
  0.9884008
 
       NA
 
       NA
  
 
  
 14
 
  75745358
 
  75745470
 
  ENSEMBL   UCSC 
 
  113
 
  4
 
 12490
 
 tss
 
 0.01058
 
 0.02321
 
 Not queried
 
  0.9883514
 
       NA
 
       NA
  
 
  
 8
 
  10191983
 
  10192642
 
  ENSEMBL   UCSC 
 
  660
 
  8
 
 39131
 
 gene;island
 
 0.04655
 
 0.02381
 
 Not queried
 
  0.9878622
 
       NA
 
       NA
  
 
  
 2
 
  98350651
 
  98350847
 
  ENSEMBL   UCSC 
 
  197
 
  7
 
 23494
 
 tss
 
 0.96051
 
 0.90395
 
 Not queried
 
  0.9877898
 
       NA
 
       NA
  
 
  
 22
 
  37678548
 
  37678791
 
  ENSEMBL   UCSC 
 
  244
 
  4
 
 26820
 
 gene
 
 0.46720
 
 0.67686
 
 Not queried
 
  0.9877821
 
       NA
 
       NA
  
 
  
 7
 
   1250038
 
   1251125
 
  ENSEMBL   UCSC 
 
 1088
 
 17
 
 38025
 
 island
 
 0.67614
 
 0.82008
 
 Not queried
 
  0.9877682
 
       NA
 
       NA
  
 
  
 19
 
  55013549
 
  55013954
 
  ENSEMBL   UCSC 
 
  406
 
  6
 
 21027
 
 tss
 
 0.33999
 
 0.34961
 
 Not queried
 
  0.9875385
 
       NA
 
       NA
  
 
  
 5
 
   1416793
 
   1417003
 
  ENSEMBL   UCSC 
 
  211
 
  4
 
 31484
 
 gene;island
 
 0.86636
 
 0.91577
 
 Not queried
 
  0.9873877
 
       NA
 
       NA
  
 
  
 4
 
 165118829
 
 165118938
 
  ENSEMBL   UCSC 
 
  110
 
  4
 
 30123
 
 gene
 
 0.39941
 
 0.39636
 
 Not queried
 
  0.9873568
 
       NA
 
       NA
  
 
  
 21
 
  46424715
 
  46424976
 
  ENSEMBL   UCSC 
 
  262
 
  6
 
 26550
 
 tss;island
 
 0.61235
 
 0.80450
 
 Not queried
 
  0.9873418
 
       NA
 
       NA
  
 
  
 5
 
 171710856
 
 171710973
 
  ENSEMBL   UCSC 
 
  118
 
  4
 
 32431
 
 tss;island
 
 0.05652
 
 0.08831
 
 Not queried
 
  0.9872521
 
       NA
 
       NA
  
 
  
 8
 
 145641882
 
 145642456
 
  ENSEMBL   UCSC 
 
  575
 
 11
 
 40663
 
 island
 
 0.67422
 
 0.85333
 
 Not queried
 
  0.9870465
 
       NA
 
       NA
  
 
  
 15
 
  73735098
 
  73735476
 
  ENSEMBL   UCSC 
 
  379
 
  8
 
 13754
 
 tss
 
 0.88694
 
 0.77890
 
 Not queried
 
  0.9868948
 
       NA
 
       NA
  
 
  
 21
 
  35445589
 
  35446012
 
  ENSEMBL   UCSC 
 
  424
 
  6
 
 26617
 
 island
 
 0.01525
 
 0.02591
 
 Not queried
 
  0.9866988
 
       NA
 
       NA
  
 
  
 11
 
  31848347
 
  31848828
 
  ENSEMBL   UCSC 
 
  482
 
  5
 
 8149
 
 island
 
 0.12442
 
 0.05406
 
 Not queried
 
  0.9859297
 
       NA
 
       NA
  
 
  
 5
 
  80256371
 
  80256738
 
  ENSEMBL   UCSC 
 
  368
 
  5
 
 32862
 
 island
 
 0.01699
 
 0.03528
 
 Not queried
 
  0.9854472
 
       NA
 
       NA
  
 
  
 X
 
  48901069
 
  48901405
 
  ENSEMBL   UCSC 
 
  337
 
  8
 
 41305
 
 tss;island
 
 0.03895
 
 0.02819
 
 Not queried
 
  0.9849925
 
       NA
 
       NA
  
 
  
 7
 
  39453532
 
  39453855
 
  ENSEMBL   UCSC 
 
  324
 
  5
 
 36910
 
 gene;island
 
 0.03664
 
 0.07168
 
 Not queried
 
  0.9845302
 
       NA
 
       NA
  
 
  
 16
 
  56659336
 
  56659552
 
  ENSEMBL   UCSC 
 
  217
 
  4
 
 15217
 
 tss
 
 0.03446
 
 0.02220
 
 Not queried
 
  0.9841536
 
       NA
 
       NA
  
 
  
 12
 
 117319246
 
 117319577
 
  ENSEMBL   UCSC 
 
  332
 
  4
 
 10015
 
 tss
 
 0.04093
 
 0.02933
 
 Not queried
 
  0.9841080
 
       NA
 
       NA
  
 
  
 18
 
  20513667
 
  20514031
 
  ENSEMBL   UCSC 
 
  365
 
  8
 
 19037
 
 gene;island
 
 0.02894
 
 0.04754
 
 Not queried
 
  0.9840831
 
       NA
 
       NA
  
 
  
 3
 
 124103560
 
 124103696
 
  ENSEMBL   UCSC 
 
  137
 
  4
 
 27880
 
 gene
 
 0.70294
 
 0.53466
 
 Not queried
 
  0.9840727
 
       NA
 
       NA
  
 
  
 16
 
  68119049
 
  68119261
 
  ENSEMBL   UCSC 
 
  213
 
  8
 
 15287
 
 tss
 
 0.01734
 
 0.02254
 
 Not queried
 
  0.9839232
 
       NA
 
       NA
  
 
  
 18
 
  44676959
 
  44677236
 
  ENSEMBL   UCSC 
 
  278
 
  6
 
 19230
 
 tss
 
 0.01024
 
 0.01800
 
 Not queried
 
  0.9838491
 
       NA
 
       NA
  
 
  
 17
 
  79608890
 
  79609346
 
  ENSEMBL   UCSC 
 
  457
 
  7
 
 17710
 
 tss
 
 0.71358
 
 0.83769
 
 Not queried
 
  0.9834595
 
       NA
 
       NA
  
 
  
 16
 
   1967781
 
   1968232
 
  ENSEMBL   UCSC 
 
  452
 
  4
 
 15538
 
 island
 
 0.04213
 
 0.06906
 
 Not queried
 
  0.9832982
 
       NA
 
       NA
  
 
  
 1
 
 153700021
 
 153700146
 
  ENSEMBL   UCSC 
 
  126
 
  4
 
 1947
 
 tss;island
 
 0.02658
 
 0.04214
 
 Not queried
 
  0.9832308
 
       NA
 
       NA
  
 
  
 3
 
  72788183
 
  72788482
 
  ENSEMBL   UCSC 
 
  300
 
  4
 
 29239
 
 island
 
 0.02818
 
 0.06762
 
 Not queried
 
  0.9831771
 
       NA
 
       NA
  
 
  
 17
 
  21356245
 
  21356372
 
  ENSEMBL   UCSC 
 
  128
 
  4
 
 18088
 
 island
 
 0.34487
 
 0.49438
 
 Not queried
 
  0.9829986
 
       NA
 
       NA
  
 
  
 6
 
  32795153
 
  32795393
 
  ENSEMBL   UCSC 
 
  241
 
  5
 
 34070
 
 gene
 
 0.20530
 
 0.27736
 
 Not queried
 
  0.9829806
 
       NA
 
       NA
  
 
  
 9
 
 131670239
 
 131670714
 
  ENSEMBL   UCSC 
 
  476
 
  4
 
 40722
 
 gene;island
 
 0.94218
 
 0.89237
 
 Not queried
 
  0.9824753
 
       NA
 
       NA
  
 
  
 6
 
 168841257
 
 168841653
 
  ENSEMBL   UCSC 
 
  397
 
  6
 
 35285
 
 tss;island
 
 0.29708
 
 0.16329
 
 Not queried
 
  0.9823673
 
       NA
 
       NA
  
 
  
 X
 
  49777565
 
  49777974
 
  ENSEMBL   UCSC 
 
  410
 
  4
 
 40995
 
 gene;tss
 
 0.60327
 
 0.76694
 
 Not queried
 
  0.9821410
 
       NA
 
       NA
  
 
  
 2
 
  48757477
 
  48757722
 
  ENSEMBL   UCSC 
 
  246
 
  4
 
 24250
 
 island
 
 0.01542
 
 0.02808
 
 Not queried
 
  0.9820754
 
       NA
 
       NA
  
 
  
 2
 
  31806755
 
  31806898
 
  ENSEMBL   UCSC 
 
  144
 
  4
 
 23252
 
 tss;island
 
 0.91502
 
 0.83045
 
 Not queried
 
  0.9819849
 
       NA
 
       NA
  
 
  
 2
 
 220427157
 
 220427342
 
  ENSEMBL   UCSC 
 
  186
 
  4
 
 23003
 
 gene;island
 
 0.44119
 
 0.62476
 
 Not queried
 
  0.9819236
 
       NA
 
       NA
  
 
  
 6
 
 139094390
 
 139094629
 
  ENSEMBL   UCSC 
 
  240
 
  8
 
 35190
 
 tss
 
 0.01203
 
 0.02470
 
 Not queried
 
  0.9817350
 
       NA
 
       NA
  
 
  
 1
 
  21978612
 
  21978863
 
  ENSEMBL   UCSC 
 
  252
 
  4
 
 299
 
 gene;tss;island
 
 0.14615
 
 0.07264
 
 Not queried
 
  0.9817043
 
       NA
 
       NA
  
 
  
 16
 
  29912904
 
  29913223
 
  ENSEMBL   UCSC 
 
  320
 
  4
 
 14685
 
 gene;island
 
 0.52650
 
 0.60504
 
 Not queried
 
  0.9816932
 
       NA
 
       NA
  
 
  
 12
 
  54321302
 
  54321653
 
  ENSEMBL   UCSC 
 
  352
 
  4
 
 10448
 
 island
 
 0.20335
 
 0.26742
 
 Not queried
 
  0.9814499
 
       NA
 
       NA
  
 
  
 1
 
  21766650
 
  21766849
 
  ENSEMBL   UCSC 
 
  200
 
  4
 
 295
 
 gene
 
 0.01456
 
 0.01768
 
 Not queried
 
  0.9812527
 
       NA
 
       NA
  
 
  
 17
 
  44896147
 
  44896223
 
  ENSEMBL   UCSC 
 
   77
 
  6
 
 17402
 
 tss
 
 0.07295
 
 0.03639
 
 Not queried
 
  0.9812116
 
       NA
 
       NA
  
 
  
 12
 
  52701963
 
  52702318
 
  ENSEMBL   UCSC 
 
  356
 
  4
 
 9112
 
 gene;island
 
 0.05000
 
 0.02881
 
 Not queried
 
  0.9811824
 
       NA
 
       NA
  
 
  
 5
 
  68787696
 
  68788117
 
  ENSEMBL   UCSC 
 
  422
 
  8
 
 32052
 
 tss
 
 0.02876
 
 0.04908
 
 Not queried
 
  0.9811562
 
       NA
 
       NA
  
 
  
 2
 
 233470847
 
 233471248
 
  ENSEMBL   UCSC 
 
  402
 
  5
 
 23030
 
 gene
 
 0.03388
 
 0.05074
 
 Not queried
 
  0.9810089
 
       NA
 
       NA
  
 
  
 17
 
  79868964
 
  79869136
 
  ENSEMBL   UCSC 
 
  173
 
  4
 
 16894
 
 gene;island
 
 0.01868
 
 0.02696
 
 Not queried
 
  0.9809884
 
       NA
 
       NA
  
 
  
 17
 
  66287480
 
  66287507
 
  ENSEMBL   UCSC 
 
   28
 
  5
 
 17574
 
 tss
 
 0.03431
 
 0.08728
 
 Not queried
 
  0.9809874
 
       NA
 
       NA
  
 
  
 10
 
  30638275
 
  30638503
 
  ENSEMBL   UCSC 
 
  229
 
  5
 
 4994
 
 tss
 
 0.01069
 
 0.00842
 
 Not queried
 
  0.9809103
 
       NA
 
       NA
  
 
  
 17
 
  17480829
 
  17481059
 
  ENSEMBL   UCSC 
 
  231
 
  5
 
 16429
 
 gene;tss
 
 0.87616
 
 0.91706
 
 Not queried
 
  0.9806953
 
       NA
 
       NA
  
 
  
 5
 
 140186484
 
 140186983
 
  ENSEMBL   UCSC 
 
  500
 
  6
 
 31738
 
 gene;island
 
 0.04951
 
 0.14588
 
 Not queried
 
  0.9805553
 
       NA
 
       NA
  
 
  
 6
 
  10882238
 
  10882336
 
  ENSEMBL   UCSC 
 
   99
 
  5
 
 34566
 
 tss
 
 0.01716
 
 0.03330
 
 Not queried
 
  0.9804054
 
       NA
 
       NA
  
 
  
 21
 
  38445169
 
  38445384
 
  ENSEMBL   UCSC 
 
  216
 
  4
 
 26380
 
 gene;tss;island
 
 0.02526
 
 0.03230
 
 Not queried
 
  0.9803393
 
       NA
 
       NA
  
 
  
 10
 
 103347979
 
 103348258
 
  ENSEMBL   UCSC 
 
  280
 
  8
 
 5291
 
 tss;island
 
 0.01987
 
 0.03253
 
 Not queried
 
  0.9803127
 
       NA
 
       NA
  
 
  
 1
 
   2716877
 
   2717193
 
  ENSEMBL   UCSC 
 
  317
 
  4
 
 2512
 
 island
 
 0.54758
 
 0.78602
 
 Not queried
 
  0.9801315
 
       NA
 
       NA
  
 
  
 2
 
  31456964
 
  31457061
 
  ENSEMBL   UCSC 
 
   98
 
  5
 
 22601
 
 gene
 
 0.01001
 
 0.01815
 
 Not queried
 
  0.9799482
 
       NA
 
       NA
  
 
  
 17
 
   1553202
 
   1553661
 
  ENSEMBL   UCSC 
 
  460
 
  9
 
 17789
 
 island
 
 0.60723
 
 0.75955
 
 Not queried
 
  0.9799392
 
       NA
 
       NA
  
 
  
 12
 
  49740639
 
  49740996
 
  ENSEMBL   UCSC 
 
  358
 
 10
 
 9662
 
 tss
 
 0.02196
 
 0.03773
 
 Not queried
 
  0.9794873
 
       NA
 
       NA
  
 
  
 12
 
  49740639
 
  49741245
 
  ENSEMBL   UCSC 
 
  607
 
 12
 
 10366
 
 island
 
 0.02583
 
 0.03773
 
 Not queried
 
  0.9794873
 
       NA
 
       NA
  
 
  
 1
 
  43770523
 
  43770899
 
  ENSEMBL   UCSC 
 
  377
 
  5
 
 474
 
 gene;island
 
 0.92268
 
 0.83805
 
 Not queried
 
  0.9794715
 
       NA
 
       NA
  
 
  
 5
 
 177412586
 
 177412718
 
  ENSEMBL   UCSC 
 
  133
 
  4
 
 33398
 
 island
 
 0.73826
 
 0.86176
 
 Not queried
 
  0.9793305
 
       NA
 
       NA
  
 
  
 7
 
   1950147
 
   1950478
 
  ENSEMBL   UCSC 
 
  332
 
  4
 
 36710
 
 gene;island
 
 0.72768
 
 0.81195
 
 Not queried
 
  0.9791725
 
       NA
 
       NA
  
 
  
 4
 
  77723057
 
  77723415
 
  ENSEMBL   UCSC 
 
  359
 
  4
 
 31067
 
 island
 
 0.58587
 
 0.71627
 
 Not queried
 
  0.9790555
 
       NA
 
       NA
  
 
  
 8
 
  85095275
 
  85095697
 
  ENSEMBL   UCSC 
 
  423
 
  5
 
 39724
 
 tss;island
 
 0.04709
 
 0.08876
 
 Not queried
 
  0.9788936
 
       NA
 
       NA
  
 
  
 6
 
  30920763
 
  30921007
 
  ENSEMBL   UCSC 
 
  245
 
  7
 
 33780
 
 gene;island
 
 0.91896
 
 0.83347
 
 Not queried
 
  0.9786762
 
       NA
 
       NA
  
 
  
 1
 
  11919018
 
  11919699
 
  ENSEMBL   UCSC 
 
  682
 
  9
 
 1249
 
 tss
 
 0.03806
 
 0.08312
 
 Not queried
 
  0.9778753
 
       NA
 
       NA
  
 
  
 1
 
  11918807
 
  11919699
 
  ENSEMBL   UCSC 
 
  893
 
 11
 
 2686
 
 island
 
 0.03954
 
 0.10411
 
 Not queried
 
  0.9778753
 
       NA
 
       NA
  
 
  
 11
 
  62358814
 
  62359087
 
  ENSEMBL   UCSC 
 
  274
 
  4
 
 6619
 
 gene
 
 0.01210
 
 0.02659
 
 Not queried
 
  0.9778317
 
       NA
 
       NA
  
 
  
 5
 
   1551883
 
   1552259
 
  ENSEMBL   UCSC 
 
  377
 
  4
 
 32573
 
 island
 
 0.74582
 
 0.88669
 
 Not queried
 
  0.9775604
 
       NA
 
       NA
  
 
  
 18
 
  71958904
 
  71959170
 
  ENSEMBL   UCSC 
 
  267
 
  4
 
 19093
 
 gene
 
 0.04906
 
 0.01619
 
 Not queried
 
  0.9773996
 
       NA
 
       NA
  
 
  
 19
 
  36631058
 
  36631318
 
  ENSEMBL   UCSC 
 
  261
 
  4
 
 19965
 
 gene
 
 0.01103
 
 0.02268
 
 Not queried
 
  0.9773166
 
       NA
 
       NA
  
 
  
 17
 
  41739130
 
  41739326
 
  ENSEMBL   UCSC 
 
  197
 
  4
 
 17359
 
 tss
 
 0.46822
 
 0.63398
 
 Not queried
 
  0.9772910
 
       NA
 
       NA
  
 
  
 2
 
  45838438
 
  45838581
 
  ENSEMBL   UCSC 
 
  144
 
  4
 
 23298
 
 tss
 
 0.03007
 
 0.03941
 
 Not queried
 
  0.9772416
 
       NA
 
       NA
  
 
  
 10
 
  88126273
 
  88126306
 
  ENSEMBL   UCSC 
 
   34
 
  6
 
 5180
 
 tss
 
 0.03184
 
 0.08172
 
 Not queried
 
  0.9770436
 
       NA
 
       NA
  
 
  
 19
 
  14542609
 
  14543048
 
  ENSEMBL   UCSC 
 
  440
 
  4
 
 21644
 
 island
 
 0.02927
 
 0.04255
 
 Not queried
 
  0.9770325
 
       NA
 
       NA
  
 
  
 1
 
 147012684
 
 147012802
 
  ENSEMBL   UCSC 
 
  119
 
  4
 
 1890
 
 tss
 
 0.11082
 
 0.05383
 
 Not queried
 
  0.9770062
 
       NA
 
       NA
  
 
  
 20
 
   2844659
 
   2844843
 
  ENSEMBL   UCSC 
 
  185
 
  6
 
 25303
 
 gene
 
 0.70557
 
 0.85069
 
 Not queried
 
  0.9769719
 
       NA
 
       NA
  
 
  
 6
 
 134213992
 
 134214307
 
  ENSEMBL   UCSC 
 
  316
 
  4
 
 34415
 
 gene;island
 
 0.03343
 
 0.04816
 
 Not queried
 
  0.9767232
 
       NA
 
       NA
  
 
  
 1
 
   2316354
 
   2316653
 
  ENSEMBL   UCSC 
 
  300
 
  4
 
 79
 
 gene;island
 
 0.68815
 
 0.84700
 
 Not queried
 
  0.9765185
 
       NA
 
       NA
  
 
  
 10
 
 112678594
 
 112678801
 
  ENSEMBL   UCSC 
 
  208
 
  5
 
 4743
 
 gene;tss;island
 
 0.01139
 
 0.01875
 
 Not queried
 
  0.9764974
 
       NA
 
       NA
  
 
  
 16
 
  50099438
 
  50099780
 
  ENSEMBL   UCSC 
 
  343
 
  4
 
 15197
 
 tss
 
 0.10649
 
 0.04265
 
 Not queried
 
  0.9764420
 
       NA
 
       NA
  
 
  
 11
 
  67418140
 
  67418405
 
  ENSEMBL   UCSC 
 
  266
 
  9
 
 7479
 
 tss
 
 0.64796
 
 0.73388
 
 Not queried
 
  0.9760064
 
       NA
 
       NA
  
 
  
 19
 
  14142047
 
  14142353
 
  ENSEMBL   UCSC 
 
  307
 
  4
 
 21631
 
 island
 
 0.40147
 
 0.30745
 
 Not queried
 
  0.9759835
 
       NA
 
       NA
  
 
  
 12
 
 109729478
 
 109729809
 
  ENSEMBL   UCSC 
 
  332
 
  4
 
 9266
 
 gene;island
 
 0.48915
 
 0.63566
 
 Not queried
 
  0.9759107
 
       NA
 
       NA
  
 
  
 1
 
  51433840
 
  51434014
 
  ENSEMBL   UCSC 
 
  175
 
  4
 
 1615
 
 tss;island
 
 0.00891
 
 0.02267
 
 Not queried
 
  0.9758759
 
       NA
 
       NA
  
 
  
 14
 
  50863043
 
  50863307
 
  ENSEMBL   UCSC 
 
  265
 
  4
 
 12359
 
 tss;island
 
 0.01795
 
 0.03675
 
 Not queried
 
  0.9758040
 
       NA
 
       NA
  
 
  
 1
 
 160336475
 
 160336712
 
  ENSEMBL   UCSC 
 
  238
 
  5
 
 2035
 
 tss
 
 0.48495
 
 0.75266
 
 Not queried
 
  0.9757651
 
       NA
 
       NA
  
 
  
 4
 
 154170410
 
 154170822
 
  ENSEMBL   UCSC 
 
  413
 
  6
 
 30106
 
 gene;island
 
 0.01075
 
 0.01718
 
 Not queried
 
  0.9757017
 
       NA
 
       NA
  
 
  
 13
 
  45694523
 
  45694609
 
  ENSEMBL   UCSC 
 
   87
 
  7
 
 11391
 
 tss
 
 0.01732
 
 0.02732
 
 Not queried
 
  0.9755659
 
       NA
 
       NA
  
 
  
 15
 
  44116616
 
  44117354
 
  ENSEMBL   UCSC 
 
  739
 
 10
 
 14066
 
 island
 
 0.03990
 
 0.03574
 
 Not queried
 
  0.9755557
 
       NA
 
       NA
  
 
  
 2
 
  74730507
 
  74730948
 
  ENSEMBL   UCSC 
 
  442
 
  9
 
 23423
 
 tss
 
 0.05102
 
 0.09198
 
 Not queried
 
  0.9750420
 
       NA
 
       NA
  
 
  
 17
 
  37617470
 
  37617707
 
  ENSEMBL   UCSC 
 
  238
 
  7
 
 17276
 
 tss;island
 
 0.01150
 
 0.02374
 
 Not queried
 
  0.9750012
 
       NA
 
       NA
  
 
  
 10
 
 115614308
 
 115614712
 
  ENSEMBL   UCSC 
 
  405
 
  8
 
 5355
 
 tss;island
 
 0.01851
 
 0.04343
 
 Not queried
 
  0.9749609
 
       NA
 
       NA
  
 
  
 6
 
   5995766
 
   5996657
 
  ENSEMBL   UCSC 
 
  892
 
  8
 
 35339
 
 island
 
 0.05332
 
 0.12152
 
 Not queried
 
  0.9749393
 
       NA
 
       NA
  
 
  
 2
 
 236840170
 
 236840562
 
  ENSEMBL   UCSC 
 
  393
 
  5
 
 23048
 
 gene
 
 0.92182
 
 0.88582
 
 Not queried
 
  0.9749166
 
       NA
 
       NA
  
 
  
 1
 
  27817084
 
  27817404
 
  ENSEMBL   UCSC 
 
  321
 
  4
 
 1410
 
 tss;island
 
 0.19368
 
 0.35941
 
 Not queried
 
  0.9748385
 
       NA
 
       NA
  
 
  
 17
 
  66287369
 
  66287507
 
  ENSEMBL   UCSC 
 
  139
 
  6
 
 16706
 
 gene;island
 
 0.05247
 
 0.09851
 
 Not queried
 
  0.9748069
 
       NA
 
       NA
  
 
  
 1
 
 100816943
 
 100817041
 
  ENSEMBL   UCSC 
 
   99
 
  4
 
 1776
 
 tss;island
 
 0.00762
 
 0.01657
 
 Not queried
 
  0.9747787
 
       NA
 
       NA
  
 
  
 4
 
 156680207
 
 156680518
 
  ENSEMBL   UCSC 
 
  312
 
  4
 
 30113
 
 gene
 
 0.02827
 
 0.05731
 
 Not queried
 
  0.9745465
 
       NA
 
       NA
  
 
  
 7
 
  87849017
 
  87849201
 
  ENSEMBL   UCSC 
 
  185
 
  4
 
 37014
 
 gene;island
 
 0.02099
 
 0.04024
 
 Not queried
 
  0.9744963
 
       NA
 
       NA
  
 
  
 10
 
  71333037
 
  71333216
 
  ENSEMBL   UCSC 
 
  180
 
  6
 
 5787
 
 island
 
 0.20933
 
 0.10198
 
 Not queried
 
  0.9744557
 
       NA
 
       NA
  
 
  
 22
 
  42475450
 
  42475697
 
  ENSEMBL   UCSC 
 
  248
 
  6
 
 27146
 
 tss
 
 0.01599
 
 0.03537
 
 Not queried
 
  0.9740164
 
       NA
 
       NA
  
 
  
 12
 
   2903984
 
   2904101
 
  ENSEMBL   UCSC 
 
  118
 
  7
 
 9464
 
 tss
 
 0.00798
 
 0.01734
 
 Not queried
 
  0.9736310
 
       NA
 
       NA
  
 
  
 11
 
  64115989
 
  64116279
 
  ENSEMBL   UCSC 
 
  291
 
  4
 
 6667
 
 gene;island
 
 0.77609
 
 0.83836
 
 Not queried
 
  0.9735166
 
       NA
 
       NA
  
 
  
 7
 
   2025220
 
   2025903
 
  ENSEMBL   UCSC 
 
  684
 
  6
 
 36718
 
 gene;island
 
 0.91180
 
 0.91546
 
 Not queried
 
  0.9734700
 
       NA
 
       NA
  
 
  
 2
 
  88427581
 
  88427827
 
  ENSEMBL   UCSC 
 
  247
 
  4
 
 23461
 
 tss
 
 0.43394
 
 0.62297
 
 Not queried
 
  0.9733506
 
       NA
 
       NA
  
 
  
 17
 
  47091978
 
  47092272
 
  ENSEMBL   UCSC 
 
  295
 
  5
 
 16635
 
 gene;island
 
 0.73275
 
 0.88470
 
 Not queried
 
  0.9730057
 
       NA
 
       NA
  
 
  
 10
 
  28035631
 
  28035894
 
  ENSEMBL   UCSC 
 
  264
 
  7
 
 4988
 
 tss;island
 
 0.39950
 
 0.16509
 
 Not queried
 
  0.9729963
 
       NA
 
       NA
  
 
  
 7
 
 134464242
 
 134464411
 
  ENSEMBL   UCSC 
 
  170
 
  4
 
 37168
 
 gene
 
 0.04553
 
 0.06514
 
 Not queried
 
  0.9729741
 
       NA
 
       NA
  
 
  
 15
 
  91643376
 
  91643742
 
  ENSEMBL   UCSC 
 
  367
 
  5
 
 13454
 
 gene;island
 
 0.02375
 
 0.03440
 
 Not queried
 
  0.9728220
 
       NA
 
       NA
  
 
  
 4
 
  17578564
 
  17579002
 
  ENSEMBL   UCSC 
 
  439
 
  9
 
 30895
 
 island
 
 0.02894
 
 0.05526
 
 Not queried
 
  0.9728133
 
       NA
 
       NA
  
 
  
 17
 
  80889047
 
  80889350
 
  ENSEMBL   UCSC 
 
  304
 
  4
 
 16933
 
 gene;island
 
 0.83107
 
 0.93847
 
 Not queried
 
  0.9727115
 
       NA
 
       NA
  
 
  
 3
 
 126422892
 
 126423272
 
  ENSEMBL   UCSC 
 
  381
 
  9
 
 29394
 
 island
 
 0.01004
 
 0.01709
 
 Not queried
 
  0.9723957
 
       NA
 
       NA
  
 
  
 17
 
  73726093
 
  73726430
 
  ENSEMBL   UCSC 
 
  338
 
  6
 
 16741
 
 gene;island
 
 0.82996
 
 0.76722
 
 Not queried
 
  0.9723504
 
       NA
 
       NA
  
 
  
 1
 
   1473141
 
   1473443
 
  ENSEMBL   UCSC 
 
  303
 
  6
 
 39
 
 gene;island
 
 0.90410
 
 0.83261
 
 Not queried
 
  0.9722554
 
       NA
 
       NA
  
 
  
 4
 
  83294709
 
  83294886
 
  ENSEMBL   UCSC 
 
  178
 
  4
 
 29993
 
 gene;island
 
 0.09931
 
 0.17300
 
 Not queried
 
  0.9722263
 
       NA
 
       NA
  
 
  
 11
 
  17036042
 
  17036644
 
  ENSEMBL   UCSC 
 
  603
 
  7
 
 7151
 
 tss
 
 0.01796
 
 0.03463
 
 Not queried
 
  0.9722017
 
       NA
 
       NA
  
 
  
 8
 
  67454546
 
  67454892
 
  ENSEMBL   UCSC 
 
  347
 
  5
 
 40291
 
 island
 
 0.13766
 
 0.04747
 
 Not queried
 
  0.9721730
 
       NA
 
       NA
  
 
  
 19
 
  10220342
 
  10220494
 
  ENSEMBL   UCSC 
 
  153
 
  4
 
 19771
 
 gene;island
 
 0.87688
 
 0.93388
 
 Not queried
 
  0.9719020
 
       NA
 
       NA
  
 
  
 13
 
 111642443
 
 111642660
 
  ENSEMBL   UCSC 
 
  218
 
  4
 
 11863
 
 island
 
 0.63976
 
 0.87872
 
 Not queried
 
  0.9715272
 
       NA
 
       NA
  
 
  
 8
 
 143869138
 
 143869473
 
  ENSEMBL   UCSC 
 
  336
 
  5
 
 39876
 
 tss
 
 0.84655
 
 0.70993
 
 Not queried
 
  0.9710270
 
       NA
 
       NA
  
 
  
 16
 
  30661534
 
  30662270
 
  ENSEMBL   UCSC 
 
  737
 
 11
 
 15810
 
 island
 
 0.04748
 
 0.08901
 
 Not queried
 
  0.9709852
 
       NA
 
       NA
  
 
  
 17
 
    406249
 
    406501
 
  ENSEMBL   UCSC 
 
  253
 
  4
 
 17763
 
 island
 
 0.96282
 
 0.86187
 
 Not queried
 
  0.9708417
 
       NA
 
       NA
  
 
  
 2
 
  48844728
 
  48844916
 
  ENSEMBL   UCSC 
 
  189
 
  5
 
 23309
 
 tss
 
 0.85816
 
 0.92932
 
 Not queried
 
  0.9707015
 
       NA
 
       NA
  
 
  
 6
 
 167136206
 
 167136432
 
  ENSEMBL   UCSC 
 
  227
 
  4
 
 34489
 
 gene
 
 0.92732
 
 0.84649
 
 Not queried
 
  0.9705202
 
       NA
 
       NA
  
 
  
 8
 
 101661918
 
 101662246
 
  ENSEMBL   UCSC 
 
  329
 
  7
 
 39780
 
 tss
 
 0.03123
 
 0.04687
 
 Not queried
 
  0.9704401
 
       NA
 
       NA
  
 
  
 8
 
 101661767
 
 101662246
 
  ENSEMBL   UCSC 
 
  480
 
  9
 
 40433
 
 island
 
 0.03123
 
 0.04511
 
 Not queried
 
  0.9704401
 
       NA
 
       NA
  
 
  
 11
 
  98891492
 
  98891820
 
  ENSEMBL   UCSC 
 
  329
 
  9
 
 7618
 
 tss;island
 
 0.01675
 
 0.04908
 
 Not queried
 
  0.9703788
 
       NA
 
       NA
  
 
  
 6
 
  71665936
 
  71666282
 
  ENSEMBL   UCSC 
 
  347
 
  4
 
 34320
 
 gene;island
 
 0.02599
 
 0.05094
 
 Not queried
 
  0.9702057
 
       NA
 
       NA
  
 
  
 6
 
  28555118
 
  28555929
 
  ENSEMBL   UCSC 
 
  812
 
 17
 
 34680
 
 tss
 
 0.01351
 
 0.04573
 
 Not queried
 
  0.9700682
 
       NA
 
       NA
  
 
  
 X
 
 118370278
 
 118370461
 
  ENSEMBL   UCSC 
 
  184
 
  4
 
 41088
 
 gene
 
 0.02006
 
 0.01284
 
 Not queried
 
  0.9698218
 
       NA
 
       NA
  
 
  
 10
 
 127511674
 
 127512288
 
  ENSEMBL   UCSC 
 
  615
 
 11
 
 5413
 
 tss;island
 
 0.01960
 
 0.02128
 
 Not queried
 
  0.9696308
 
       NA
 
       NA
  
 
  
 17
 
  26733294
 
  26733583
 
  ENSEMBL   UCSC 
 
  290
 
  6
 
 17178
 
 tss;island
 
 0.02943
 
 0.05316
 
 Not queried
 
  0.9695467
 
       NA
 
       NA
  
 
  
 19
 
   8028518
 
   8028819
 
  ENSEMBL   UCSC 
 
  302
 
  4
 
 19754
 
 gene;island
 
 0.91033
 
 0.93032
 
 Not queried
 
  0.9695262
 
       NA
 
       NA
  
 
  
 19
 
  50887127
 
  50887530
 
  ENSEMBL   UCSC 
 
  404
 
  4
 
 20940
 
 tss;island
 
 0.02281
 
 0.04367
 
 Not queried
 
  0.9688527
 
       NA
 
       NA
  
 
  
 6
 
  28477957
 
  28478579
 
  ENSEMBL   UCSC 
 
  623
 
  8
 
 33568
 
 gene
 
 0.85736
 
 0.66763
 
 Not queried
 
  0.9687308
 
       NA
 
       NA
  
 
  
 10
 
  12110577
 
  12111049
 
  ENSEMBL   UCSC 
 
  473
 
  9
 
 5533
 
 island
 
 0.02152
 
 0.04846
 
 Not queried
 
  0.9686011
 
       NA
 
       NA
  
 
  
 5
 
 137878937
 
 137879011
 
  ENSEMBL   UCSC 
 
   75
 
  4
 
 32264
 
 tss
 
 0.01780
 
 0.03149
 
 Not queried
 
  0.9684452
 
       NA
 
       NA
  
 
  
 5
 
 158689930
 
 158690246
 
  ENSEMBL   UCSC 
 
  317
 
  6
 
 33292
 
 island
 
 0.00643
 
 0.02067
 
 Not queried
 
  0.9683956
 
       NA
 
       NA
  
 
  
 2
 
  26569594
 
  26570144
 
  ENSEMBL   UCSC 
 
  551
 
  9
 
 22575
 
 gene;island
 
 0.01136
 
 0.02595
 
 Not queried
 
  0.9683917
 
       NA
 
       NA
  
 
  
 4
 
  47034181
 
  47034428
 
  ENSEMBL   UCSC 
 
  248
 
  4
 
 29951
 
 gene;island
 
 0.05262
 
 0.08482
 
 Not queried
 
  0.9683673
 
       NA
 
       NA
  
 
  
 13
 
  88324432
 
  88324711
 
  ENSEMBL   UCSC 
 
  280
 
  4
 
 11466
 
 tss
 
 0.02554
 
 0.03226
 
 Not queried
 
  0.9683196
 
       NA
 
       NA
  
 
  
 5
 
  71616216
 
  71616505
 
  ENSEMBL   UCSC 
 
  290
 
  6
 
 31585
 
 gene
 
 0.01698
 
 0.03323
 
 Not queried
 
  0.9683047
 
       NA
 
       NA
  
 
  
 3
 
  49907414
 
  49907692
 
  ENSEMBL   UCSC 
 
  279
 
  6
 
 28296
 
 tss
 
 0.24981
 
 0.07398
 
 Not queried
 
  0.9681785
 
       NA
 
       NA
  
 
  
 1
 
  15478848
 
  15479474
 
  ENSEMBL   UCSC 
 
  627
 
 10
 
 1265
 
 tss;island
 
 0.01099
 
 0.02146
 
 Not queried
 
  0.9679818
 
       NA
 
       NA
  
 
  
 6
 
  13574034
 
  13574371
 
  ENSEMBL   UCSC 
 
  338
 
  5
 
 34579
 
 tss;island
 
 0.04298
 
 0.03634
 
 Not queried
 
  0.9678545
 
       NA
 
       NA
  
 
  
 15
 
  83621517
 
  83621779
 
  ENSEMBL   UCSC 
 
  263
 
  5
 
 13833
 
 tss;island
 
 0.03611
 
 0.02109
 
 Not queried
 
  0.9678030
 
       NA
 
       NA
  
 
  
 17
 
  48502923
 
  48503406
 
  ENSEMBL   UCSC 
 
  484
 
  5
 
 17466
 
 tss;island
 
 0.40167
 
 0.08924
 
 Not queried
 
  0.9677073
 
       NA
 
       NA
  
 
  
 1
 
 155715037
 
 155715510
 
  ENSEMBL   UCSC 
 
  474
 
  8
 
 1999
 
 tss;island
 
 0.09947
 
 0.05295
 
 Not queried
 
  0.9676971
 
       NA
 
       NA
  
 
  
 X
 
 118284586
 
 118285064
 
  ENSEMBL   UCSC 
 
  479
 
 10
 
 41485
 
 tss
 
 0.84678
 
 0.70245
 
 Not queried
 
  0.9673193
 
       NA
 
       NA
  
 
  
 6
 
  26027410
 
  26027625
 
  ENSEMBL   UCSC 
 
  216
 
  7
 
 35444
 
 island
 
 0.00782
 
 0.02551
 
 Not queried
 
  0.9673171
 
       NA
 
       NA
  
 
  
 14
 
  92788914
 
  92789193
 
  ENSEMBL   UCSC 
 
  280
 
  4
 
 12538
 
 tss;island
 
 0.31586
 
 0.14775
 
 Not queried
 
  0.9672597
 
       NA
 
       NA
  
 
  
 1
 
 112938339
 
 112938731
 
  ENSEMBL   UCSC 
 
  393
 
  6
 
 1830
 
 tss;island
 
 0.01937
 
 0.02416
 
 Not queried
 
  0.9662311
 
       NA
 
       NA
  
 
  
 10
 
   1095069
 
   1095235
 
  ENSEMBL   UCSC 
 
  167
 
  5
 
 4896
 
 tss
 
 0.00778
 
 0.01837
 
 Not queried
 
  0.9656674
 
       NA
 
       NA
  
 
  
 10
 
   1094976
 
   1095235
 
  ENSEMBL   UCSC 
 
  260
 
  7
 
 5467
 
 island
 
 0.00867
 
 0.02035
 
 Not queried
 
  0.9656674
 
       NA
 
       NA
  
 
  
 4
 
  77507102
 
  77507411
 
  ENSEMBL   UCSC 
 
  310
 
  5
 
 29983
 
 gene;island
 
 0.01766
 
 0.03897
 
 Not queried
 
  0.9655790
 
       NA
 
       NA
  
 
  
 11
 
  75141627
 
  75142012
 
  ENSEMBL   UCSC 
 
  386
 
  8
 
 8639
 
 island
 
 0.06426
 
 0.13920
 
 Not queried
 
  0.9654457
 
       NA
 
       NA
  
 
  
 17
 
  80818992
 
  80819418
 
  ENSEMBL   UCSC 
 
  427
 
  7
 
 16928
 
 gene;island
 
 0.91744
 
 0.93802
 
 Not queried
 
  0.9651981
 
       NA
 
       NA
  
 
  
 6
 
  31850465
 
  31850515
 
  ENSEMBL   UCSC 
 
   51
 
  4
 
 33918
 
 gene
 
 0.95198
 
 0.97799
 
 Not queried
 
  0.9650097
 
       NA
 
       NA
  
 
  
 16
 
    783597
 
    783916
 
  ENSEMBL   UCSC 
 
  320
 
  4
 
 14514
 
 gene;island
 
 0.68694
 
 0.76183
 
 Not queried
 
  0.9648312
 
       NA
 
       NA
  
 
  
 10
 
 123734501
 
 123734712
 
  ENSEMBL   UCSC 
 
  212
 
  4
 
 4781
 
 gene
 
 0.01546
 
 0.03176
 
 Not queried
 
  0.9648155
 
       NA
 
       NA
  
 
  
 11
 
   1947601
 
   1947949
 
  ENSEMBL   UCSC 
 
  349
 
  4
 
 6420
 
 gene;island
 
 0.77263
 
 0.85857
 
 Not queried
 
  0.9644705
 
       NA
 
       NA
  
 
  
 X
 
  11775676
 
  11775785
 
  ENSEMBL   UCSC 
 
  110
 
  4
 
 41184
 
 tss;island
 
 0.16674
 
 0.06161
 
 Not queried
 
  0.9643974
 
       NA
 
       NA
  
 
  
 14
 
  97263458
 
  97263643
 
  ENSEMBL   UCSC 
 
  186
 
  4
 
 12569
 
 tss
 
 0.02917
 
 0.04518
 
 Not queried
 
  0.9643146
 
       NA
 
       NA
  
 
  
 10
 
 134404873
 
 134405126
 
  ENSEMBL   UCSC 
 
  254
 
  4
 
 4850
 
 gene
 
 0.86863
 
 0.94403
 
 Not queried
 
  0.9643138
 
       NA
 
       NA
  
 
  
 20
 
   3644097
 
   3644300
 
  ENSEMBL   UCSC 
 
  204
 
  4
 
 25527
 
 tss
 
 0.57615
 
 0.43805
 
 Not queried
 
  0.9641606
 
       NA
 
       NA
  
 
  
 22
 
  20748269
 
  20748445
 
  ENSEMBL   UCSC 
 
  177
 
  7
 
 26937
 
 tss;island
 
 0.01763
 
 0.03383
 
 Not queried
 
  0.9640176
 
       NA
 
       NA
  
 
  
 12
 
  25538177
 
  25538380
 
  ENSEMBL   UCSC 
 
  204
 
  4
 
 10266
 
 island
 
 0.03575
 
 0.01947
 
 Not queried
 
  0.9640171
 
       NA
 
       NA
  
 
  
 14
 
  96750481
 
  96750614
 
  ENSEMBL   UCSC 
 
  134
 
  4
 
 12172
 
 gene
 
 0.95395
 
 0.98144
 
 Not queried
 
  0.9639678
 
       NA
 
       NA
  
 
  
 7
 
  39989474
 
  39990042
 
  ENSEMBL   UCSC 
 
  569
 
  7
 
 38365
 
 island
 
 0.00441
 
 0.01086
 
 Not queried
 
  0.9638754
 
       NA
 
       NA
  
 
  
 17
 
   7146254
 
   7146757
 
  ENSEMBL   UCSC 
 
  504
 
  7
 
 17034
 
 tss;island
 
 0.06134
 
 0.10448
 
 Not queried
 
  0.9638296
 
       NA
 
       NA
  
 
  
 19
 
  39897131
 
  39897500
 
  ENSEMBL   UCSC 
 
  370
 
  8
 
 21960
 
 island
 
 0.02781
 
 0.04779
 
 Not queried
 
  0.9633612
 
       NA
 
       NA
  
 
  
 5
 
 135188170
 
 135188477
 
  ENSEMBL   UCSC 
 
  308
 
  4
 
 31702
 
 gene;island
 
 0.86257
 
 0.77976
 
 Not queried
 
  0.9631926
 
       NA
 
       NA
  
 
  
 11
 
   7041194
 
   7041570
 
  ENSEMBL   UCSC 
 
  377
 
  8
 
 6476
 
 gene
 
 0.06174
 
 0.02530
 
 Not queried
 
  0.9630800
 
       NA
 
       NA
  
 
  
 10
 
 114043123
 
 114043435
 
  ENSEMBL   UCSC 
 
  313
 
  5
 
 5347
 
 tss
 
 0.64194
 
 0.51741
 
 Not queried
 
  0.9627047
 
       NA
 
       NA
  
 
  
 1
 
 155162752
 
 155163240
 
  ENSEMBL   UCSC 
 
  489
 
  6
 
 1981
 
 tss;island
 
 0.20868
 
 0.27299
 
 Not queried
 
  0.9625229
 
       NA
 
       NA
  
 
  
 19
 
  42437245
 
  42437425
 
  ENSEMBL   UCSC 
 
  181
 
  4
 
 22025
 
 island
 
 0.78110
 
 0.63961
 
 Not queried
 
  0.9624980
 
       NA
 
       NA
  
 
  
 7
 
 152131894
 
 152132218
 
  ENSEMBL   UCSC 
 
  325
 
  4
 
 37237
 
 gene
 
 0.01896
 
 0.04722
 
 Not queried
 
  0.9624147
 
       NA
 
       NA
  
 
  
 6
 
  29580896
 
  29581228
 
  ENSEMBL   UCSC 
 
  333
 
  7
 
 33609
 
 gene
 
 0.90328
 
 0.93043
 
 Not queried
 
  0.9623807
 
       NA
 
       NA
  
 
  
 16
 
  71523259
 
  71523560
 
  ENSEMBL   UCSC 
 
  302
 
  8
 
 15316
 
 tss
 
 0.71196
 
 0.82776
 
 Not queried
 
  0.9621812
 
       NA
 
       NA
  
 
  
 11
 
   6440690
 
   6440968
 
  ENSEMBL   UCSC 
 
  279
 
 10
 
 7090
 
 tss;island
 
 0.01879
 
 0.03057
 
 Not queried
 
  0.9621783
 
       NA
 
       NA
  
 
  
 4
 
  84256184
 
  84256398
 
  ENSEMBL   UCSC 
 
  215
 
  9
 
 30427
 
 tss;island
 
 0.02484
 
 0.02891
 
 Not queried
 
  0.9621584
 
       NA
 
       NA
  
 
  
 10
 
  50323706
 
  50323832
 
  ENSEMBL   UCSC 
 
  127
 
  5
 
 5053
 
 tss;island
 
 0.53973
 
 0.36636
 
 Not queried
 
  0.9621340
 
       NA
 
       NA
  
 
  
 6
 
  31617322
 
  31617606
 
  ENSEMBL   UCSC 
 
  285
 
  7
 
 33861
 
 gene;island
 
 0.93512
 
 0.95243
 
 Not queried
 
  0.9620461
 
       NA
 
       NA
  
 
  
 1
 
  70033219
 
  70033902
 
  ENSEMBL   UCSC 
 
  684
 
  8
 
 3368
 
 island
 
 0.02362
 
 0.01975
 
 Not queried
 
  0.9618894
 
       NA
 
       NA
  
 
  
 17
 
  38501397
 
  38501773
 
  ENSEMBL   UCSC 
 
  377
 
  4
 
 16529
 
 gene;island
 
 0.03448
 
 0.07595
 
 Not queried
 
  0.9618872
 
       NA
 
       NA
  
 
  
 7
 
 157567895
 
 157568392
 
  ENSEMBL   UCSC 
 
  498
 
  6
 
 37280
 
 gene;island
 
 0.82564
 
 0.87894
 
 Not queried
 
  0.9617065
 
       NA
 
       NA
  
 
  
 2
 
 160761085
 
 160761622
 
  ENSEMBL   UCSC 
 
  538
 
  8
 
 24774
 
 island
 
 0.03850
 
 0.02305
 
 Not queried
 
  0.9614813
 
       NA
 
       NA
  
 
  
 6
 
  31973051
 
  31973345
 
  ENSEMBL   UCSC 
 
  295
 
  5
 
 34795
 
 tss
 
 0.31972
 
 0.27153
 
 Not queried
 
  0.9614138
 
       NA
 
       NA
  
 
  
 17
 
  79829883
 
  79830196
 
  ENSEMBL   UCSC 
 
  314
 
  4
 
 17719
 
 tss;island
 
 0.06522
 
 0.17699
 
 Not queried
 
  0.9613451
 
       NA
 
       NA
  
 
  
 X
 
 118986976
 
 118987328
 
  ENSEMBL   UCSC 
 
  353
 
 10
 
 41493
 
 tss
 
 0.02387
 
 0.03380
 
 Not queried
 
  0.9611984
 
       NA
 
       NA
  
 
  
 16
 
  85676292
 
  85676526
 
  ENSEMBL   UCSC 
 
  235
 
  4
 
 14847
 
 gene;island
 
 0.81353
 
 0.87516
 
 Not queried
 
  0.9609073
 
       NA
 
       NA
  
 
  
 8
 
   1642380
 
   1642625
 
  ENSEMBL   UCSC 
 
  246
 
  4
 
 39109
 
 gene;island
 
 0.79893
 
 0.87835
 
 Not queried
 
  0.9607499
 
       NA
 
       NA
  
 
  
 11
 
   1506936
 
   1507347
 
  ENSEMBL   UCSC 
 
  412
 
  6
 
 6405
 
 gene
 
 0.81554
 
 0.85697
 
 Not queried
 
  0.9606307
 
       NA
 
       NA
  
 
  
 10
 
 103824819
 
 103825131
 
  ENSEMBL   UCSC 
 
  313
 
  5
 
 5297
 
 tss
 
 0.01332
 
 0.02481
 
 Not queried
 
  0.9605979
 
       NA
 
       NA
  
 
  
 20
 
  34204988
 
  34205182
 
  ENSEMBL   UCSC 
 
  195
 
  4
 
 25369
 
 gene;island
 
 0.03425
 
 0.02120
 
 Not queried
 
  0.9605523
 
       NA
 
       NA
  
 
  
 1
 
   1046086
 
   1046324
 
  ENSEMBL   UCSC 
 
  239
 
  4
 
 15
 
 gene;island
 
 0.92120
 
 0.92901
 
 Not queried
 
  0.9604468
 
       NA
 
       NA
  
 
  
 4
 
 159093841
 
 159094034
 
  ENSEMBL   UCSC 
 
  194
 
  4
 
 30115
 
 gene
 
 0.04960
 
 0.13356
 
 Not queried
 
  0.9601712
 
       NA
 
       NA
  
 
  
 6
 
  30520935
 
  30521320
 
  ENSEMBL   UCSC 
 
  386
 
  9
 
 33712
 
 gene;island
 
 0.80966
 
 0.85348
 
 Not queried
 
  0.9601700
 
       NA
 
       NA
  
 
  
 9
 
  90112515
 
  90112711
 
  ENSEMBL   UCSC 
 
  197
 
  4
 
 40754
 
 tss;island
 
 0.00774
 
 0.01095
 
 Not queried
 
  0.9601232
 
       NA
 
       NA
  
 
  
 8
 
  63998640
 
  63998778
 
  ENSEMBL   UCSC 
 
  139
 
  6
 
 39667
 
 tss
 
 0.02980
 
 0.01999
 
 Not queried
 
  0.9600435
 
       NA
 
       NA
  
 
  
 2
 
 237072673
 
 237072998
 
  ENSEMBL   UCSC 
 
  326
 
  4
 
 25185
 
 island
 
 0.89387
 
 0.75759
 
 Not queried
 
  0.9599497
 
       NA
 
       NA
  
 
  
 6
 
  33228341
 
  33228498
 
  ENSEMBL   UCSC 
 
  158
 
  4
 
 34170
 
 gene
 
 0.84866
 
 0.94042
 
 Not queried
 
  0.9598443
 
       NA
 
       NA
  
 
  
 1
 
 156818592
 
 156819191
 
  ENSEMBL   UCSC 
 
  600
 
  6
 
 830
 
 gene;island
 
 0.39106
 
 0.51778
 
 Not queried
 
  0.9595173
 
       NA
 
       NA
  
 
  
 1
 
 145826877
 
 145827062
 
  ENSEMBL   UCSC 
 
  186
 
  4
 
 726
 
 gene;island
 
 0.01601
 
 0.05597
 
 Not queried
 
  0.9593807
 
       NA
 
       NA
  
 
  
 20
 
  35580195
 
  35580555
 
  ENSEMBL   UCSC 
 
  361
 
  9
 
 25683
 
 tss
 
 0.04964
 
 0.03815
 
 Not queried
 
  0.9593753
 
       NA
 
       NA
  
 
  
 10
 
  91174099
 
  91174309
 
  ENSEMBL   UCSC 
 
  211
 
  7
 
 5197
 
 tss;island
 
 0.00905
 
 0.01375
 
 Not queried
 
  0.9592094
 
       NA
 
       NA
  
 
  
 8
 
  27472374
 
  27472513
 
  ENSEMBL   UCSC 
 
  140
 
  7
 
 39552
 
 tss
 
 0.05173
 
 0.06505
 
 Not queried
 
  0.9590981
 
       NA
 
       NA
  
 
  
 1
 
  50893469
 
  50893984
 
  ENSEMBL   UCSC 
 
  516
 
  5
 
 3247
 
 island
 
 0.12875
 
 0.12020
 
 Not queried
 
  0.9590886
 
       NA
 
       NA
  
 
  
 3
 
  46448496
 
  46448963
 
  ENSEMBL   UCSC 
 
  468
 
  8
 
 28232
 
 tss
 
 0.66806
 
 0.76834
 
 Not queried
 
  0.9589971
 
       NA
 
       NA
  
 
  
 3
 
 179041226
 
 179041590
 
  ENSEMBL   UCSC 
 
  365
 
  9
 
 29634
 
 island
 
 0.04502
 
 0.05693
 
 Not queried
 
  0.9589448
 
       NA
 
       NA
  
 
  
 2
 
  28614438
 
  28614671
 
  ENSEMBL   UCSC 
 
  234
 
  4
 
 23242
 
 tss;island
 
 0.02463
 
 0.03686
 
 Not queried
 
  0.9588062
 
       NA
 
       NA
  
 
  
 3
 
 128151940
 
 128152268
 
  ENSEMBL   UCSC 
 
  329
 
  4
 
 29410
 
 island
 
 0.04885
 
 0.02511
 
 Not queried
 
  0.9587348
 
       NA
 
       NA
  
 
  
 8
 
 145159308
 
 145159375
 
  ENSEMBL   UCSC 
 
   68
 
  4
 
 39447
 
 gene
 
 0.01060
 
 0.01869
 
 Not queried
 
  0.9586041
 
       NA
 
       NA
  
 
  
 1
 
 155035008
 
 155035530
 
  ENSEMBL   UCSC 
 
  523
 
  5
 
 1974
 
 tss;island
 
 0.03071
 
 0.07630
 
 Not queried
 
  0.9584710
 
       NA
 
       NA
  
 
  
 1
 
  43250691
 
  43251253
 
  ENSEMBL   UCSC 
 
  563
 
  6
 
 3145
 
 island
 
 0.72650
 
 0.87005
 
 Not queried
 
  0.9583592
 
       NA
 
       NA
  
 
  
 6
 
  31021134
 
  31021422
 
  ENSEMBL   UCSC 
 
  289
 
  4
 
 34733
 
 tss
 
 0.79499
 
 0.69014
 
 Not queried
 
  0.9583134
 
       NA
 
       NA
  
 
  
 X
 
 142721811
 
 142722174
 
  ENSEMBL   UCSC 
 
  364
 
  5
 
 41130
 
 gene;island
 
 0.01392
 
 0.03362
 
 Not queried
 
  0.9582784
 
       NA
 
       NA
  
 
  
 15
 
  55611086
 
  55611607
 
  ENSEMBL   UCSC 
 
  522
 
  8
 
 14129
 
 island
 
 0.04889
 
 0.03203
 
 Not queried
 
  0.9582497
 
       NA
 
       NA
  
 
  
 6
 
  46655306
 
  46655543
 
  ENSEMBL   UCSC 
 
  238
 
  7
 
 34957
 
 tss;island
 
 0.75066
 
 0.79582
 
 Not queried
 
  0.9582133
 
       NA
 
       NA
  
 
  
 16
 
  86542659
 
  86543092
 
  ENSEMBL   UCSC 
 
  434
 
 10
 
 15370
 
 tss;island
 
 0.04746
 
 0.06826
 
 Not queried
 
  0.9580034
 
       NA
 
       NA
  
 
  
 17
 
   1801637
 
   1802074
 
  ENSEMBL   UCSC 
 
  438
 
  4
 
 16288
 
 gene;island
 
 0.93460
 
 0.87659
 
 Not queried
 
  0.9579198
 
       NA
 
       NA
  
 
  
 5
 
  89825494
 
  89825908
 
  ENSEMBL   UCSC 
 
  415
 
  7
 
 32114
 
 tss
 
 0.03739
 
 0.02680
 
 Not queried
 
  0.9578690
 
       NA
 
       NA
  
 
  
 13
 
  25745946
 
  25746644
 
  ENSEMBL   UCSC 
 
  699
 
 11
 
 11322
 
 tss
 
 0.53388
 
 0.37100
 
 Not queried
 
  0.9574672
 
       NA
 
       NA
  
 
  
 20
 
    656446
 
    656743
 
  ENSEMBL   UCSC 
 
  298
 
  4
 
 25285
 
 gene
 
 0.03291
 
 0.06945
 
 Not queried
 
  0.9573387
 
       NA
 
       NA
  
 
  
 3
 
  15642818
 
  15643321
 
  ENSEMBL   UCSC 
 
  504
 
 13
 
 28133
 
 tss;island
 
 0.02347
 
 0.02659
 
 Not queried
 
  0.9570672
 
       NA
 
       NA
  
 
  
 3
 
  15140786
 
  15140889
 
  ENSEMBL   UCSC 
 
  104
 
  4
 
 28130
 
 tss
 
 0.01664
 
 0.02525
 
 Not queried
 
  0.9568247
 
       NA
 
       NA
  
 
  
 X
 
  69281860
 
  69282293
 
  ENSEMBL   UCSC 
 
  434
 
  8
 
 41363
 
 tss
 
 0.83192
 
 0.88406
 
 Not queried
 
  0.9567074
 
       NA
 
       NA
  
 
  
 14
 
 102414421
 
 102414739
 
  ENSEMBL   UCSC 
 
  319
 
  4
 
 13136
 
 island
 
 0.02444
 
 0.03962
 
 Not queried
 
  0.9566013
 
       NA
 
       NA
  
 
  
 1
 
 156626749
 
 156627099
 
  ENSEMBL   UCSC 
 
  351
 
  4
 
 824
 
 gene;island
 
 0.22661
 
 0.33025
 
 Not queried
 
  0.9565867
 
       NA
 
       NA
  
 
  
 19
 
  18942465
 
  18942619
 
  ENSEMBL   UCSC 
 
  155
 
  5
 
 20593
 
 tss
 
 0.02512
 
 0.04306
 
 Not queried
 
  0.9564984
 
       NA
 
       NA
  
 
  
 19
 
  58220657
 
  58220837
 
  ENSEMBL   UCSC 
 
  181
 
  6
 
 21087
 
 tss
 
 0.03054
 
 0.01951
 
 Not queried
 
  0.9564048
 
       NA
 
       NA
  
 
  
 17
 
  79109729
 
  79110023
 
  ENSEMBL   UCSC 
 
  295
 
  5
 
 16864
 
 gene;island
 
 0.73695
 
 0.88040
 
 Not queried
 
  0.9563946
 
       NA
 
       NA
  
 
  
 12
 
  15475490
 
  15475767
 
  ENSEMBL   UCSC 
 
  278
 
  4
 
 9044
 
 gene
 
 0.05713
 
 0.03316
 
 Not queried
 
  0.9563196
 
       NA
 
       NA
  
 
  
 16
 
   8995607
 
   8995984
 
  ENSEMBL   UCSC 
 
  378
 
  6
 
 14625
 
 gene
 
 0.82673
 
 0.91967
 
 Not queried
 
  0.9560925
 
       NA
 
       NA
  
 
  
 18
 
  49865711
 
  49866548
 
  ENSEMBL   UCSC 
 
  838
 
 18
 
 19484
 
 island
 
 0.04168
 
 0.13975
 
 Not queried
 
  0.9560505
 
       NA
 
       NA
  
 
  
 10
 
 123734501
 
 123734889
 
  ENSEMBL   UCSC 
 
  389
 
  5
 
 6186
 
 island
 
 0.01693
 
 0.03959
 
 Not queried
 
  0.9558943
 
       NA
 
       NA
  
 
  
 7
 
   2271673
 
   2271814
 
  ENSEMBL   UCSC 
 
  142
 
  4
 
 36733
 
 gene;island
 
 0.04688
 
 0.01590
 
 Not queried
 
  0.9555727
 
       NA
 
       NA
  
 
  
 6
 
 161188022
 
 161188822
 
  ENSEMBL   UCSC 
 
  801
 
  8
 
 36553
 
 island
 
 0.07058
 
 0.11949
 
 Not queried
 
  0.9555707
 
       NA
 
       NA
  
 
  
 11
 
    597231
 
    597651
 
  ENSEMBL   UCSC 
 
  421
 
  5
 
 6363
 
 gene;island
 
 0.83889
 
 0.90989
 
 Not queried
 
  0.9555512
 
       NA
 
       NA
  
 
  
 20
 
  48530309
 
  48530654
 
  ENSEMBL   UCSC 
 
  346
 
  4
 
 25411
 
 gene;tss;island
 
 0.65490
 
 0.78220
 
 Not queried
 
  0.9554498
 
       NA
 
       NA
  
 
  
 1
 
   2222253
 
   2222674
 
  ENSEMBL   UCSC 
 
  422
 
  5
 
 72
 
 gene;island
 
 0.67202
 
 0.85897
 
 Not queried
 
  0.9554000
 
       NA
 
       NA
  
 
  
 5
 
  57755773
 
  57756026
 
  ENSEMBL   UCSC 
 
  254
 
  8
 
 32752
 
 island
 
 0.01434
 
 0.02638
 
 Not queried
 
  0.9553559
 
       NA
 
       NA
  
 
  
 6
 
  31545836
 
  31546085
 
  ENSEMBL   UCSC 
 
  250
 
  4
 
 33841
 
 gene;island
 
 0.48041
 
 0.74805
 
 Not queried
 
  0.9553300
 
       NA
 
       NA
  
 
  
 17
 
  21495693
 
  21495791
 
  ENSEMBL   UCSC 
 
   99
 
  4
 
 18093
 
 island
 
 0.91084
 
 0.85315
 
 Not queried
 
  0.9552081
 
       NA
 
       NA
  
 
  
 12
 
 124773870
 
 124774078
 
  ENSEMBL   UCSC 
 
  209
 
  4
 
 9361
 
 gene
 
 0.78644
 
 0.87543
 
 Not queried
 
  0.9551814
 
       NA
 
       NA
  
 
  
 6
 
 168936486
 
 168936684
 
  ENSEMBL   UCSC 
 
  199
 
  4
 
 34497
 
 gene;island
 
 0.94598
 
 0.88595
 
 Not queried
 
  0.9551712
 
       NA
 
       NA
  
 
  
 19
 
   2251588
 
   2252069
 
  ENSEMBL   UCSC 
 
  482
 
  5
 
 19648
 
 gene;island
 
 0.85457
 
 0.93079
 
 Not queried
 
  0.9551689
 
       NA
 
       NA
  
 
  
 11
 
  66335975
 
  66336293
 
  ENSEMBL   UCSC 
 
  319
 
  7
 
 8496
 
 island
 
 0.77384
 
 0.91633
 
 Not queried
 
  0.9548665
 
       NA
 
       NA
  
 
  
 7
 
  37960731
 
  37960974
 
  ENSEMBL   UCSC 
 
  244
 
  4
 
 36907
 
 gene;island
 
 0.05045
 
 0.02595
 
 Not queried
 
  0.9547212
 
       NA
 
       NA
  
 
  
 17
 
  79480921
 
  79481108
 
  ENSEMBL   UCSC 
 
  188
 
  4
 
 17706
 
 tss;island
 
 0.02070
 
 0.01013
 
 Not queried
 
  0.9545606
 
       NA
 
       NA
  
 
  
 15
 
  30114295
 
  30114912
 
  ENSEMBL   UCSC 
 
  618
 
  7
 
 13949
 
 island
 
 0.00643
 
 0.01381
 
 Not queried
 
  0.9545440
 
       NA
 
       NA
  
 
  
 19
 
   1241377
 
   1241808
 
  ENSEMBL   UCSC 
 
  432
 
  6
 
 21168
 
 island
 
 0.01896
 
 0.03211
 
 Not queried
 
  0.9543978
 
       NA
 
       NA
  
 
  
 4
 
  44728642
 
  44728857
 
  ENSEMBL   UCSC 
 
  216
 
  7
 
 30323
 
 tss
 
 0.01441
 
 0.02988
 
 Not queried
 
  0.9542606
 
       NA
 
       NA
  
 
  
 7
 
   5463140
 
   5463911
 
  ENSEMBL   UCSC 
 
  772
 
 10
 
 38160
 
 island
 
 0.06365
 
 0.07031
 
 Not queried
 
  0.9541992
 
       NA
 
       NA
  
 
  
 1
 
  47902085
 
  47902371
 
  ENSEMBL   UCSC 
 
  287
 
  5
 
 517
 
 gene;island
 
 0.02722
 
 0.06000
 
 Not queried
 
  0.9540310
 
       NA
 
       NA
  
 
  
 6
 
 168615184
 
 168615500
 
  ENSEMBL   UCSC 
 
  317
 
  6
 
 36599
 
 island
 
 0.85554
 
 0.77776
 
 Not queried
 
  0.9537276
 
       NA
 
       NA
  
 
  
 X
 
  15692787
 
  15693037
 
  ENSEMBL   UCSC 
 
  251
 
  7
 
 41200
 
 tss;island
 
 0.03843
 
 0.04993
 
 Not queried
 
  0.9534814
 
       NA
 
       NA
  
 
  
 11
 
  70666452
 
  70666748
 
  ENSEMBL   UCSC 
 
  297
 
  4
 
 6778
 
 gene;island
 
 0.86162
 
 0.76422
 
 Not queried
 
  0.9533697
 
       NA
 
       NA
  
 
  
 10
 
  75531971
 
  75532169
 
  ENSEMBL   UCSC 
 
  199
 
  6
 
 5837
 
 island
 
 0.07024
 
 0.05081
 
 Not queried
 
  0.9533686
 
       NA
 
       NA
  
 
  
 6
 
  32025416
 
  32025657
 
  ENSEMBL   UCSC 
 
  242
 
  4
 
 33959
 
 gene
 
 0.76071
 
 0.62556
 
 Not queried
 
  0.9532812
 
       NA
 
       NA
  
 
  
 2
 
 176936377
 
 176936581
 
  ENSEMBL   UCSC 
 
  205
 
  4
 
 24847
 
 island
 
 0.70472
 
 0.81705
 
 Not queried
 
  0.9529916
 
       NA
 
       NA
  
 
  
 X
 
  48367969
 
  48368195
 
  ENSEMBL   UCSC 
 
  227
 
  7
 
 40983
 
 gene;island
 
 0.49018
 
 0.38530
 
 Not queried
 
  0.9526402
 
       NA
 
       NA
  
 
  
 4
 
  13486103
 
  13486582
 
  ENSEMBL   UCSC 
 
  480
 
  8
 
 30256
 
 tss;island
 
 0.01152
 
 0.02208
 
 Not queried
 
  0.9525895
 
       NA
 
       NA
  
 
  
 2
 
  95537044
 
  95537475
 
  ENSEMBL   UCSC 
 
  432
 
  8
 
 24472
 
 island
 
 0.44626
 
 0.60130
 
 Not queried
 
  0.9525690
 
       NA
 
       NA
  
 
  
 11
 
  44087367
 
  44087808
 
  ENSEMBL   UCSC 
 
  442
 
  9
 
 7238
 
 tss
 
 0.05104
 
 0.02353
 
 Not queried
 
  0.9524643
 
       NA
 
       NA
  
 
  
 16
 
  22825621
 
  22825843
 
  ENSEMBL   UCSC 
 
  223
 
  4
 
 15103
 
 tss;island
 
 0.09742
 
 0.22622
 
 Not queried
 
  0.9523964
 
       NA
 
       NA
  
 
  
 21
 
  46340776
 
  46341067
 
  ENSEMBL   UCSC 
 
  292
 
  7
 
 26419
 
 gene
 
 0.73067
 
 0.82549
 
 Not queried
 
  0.9523909
 
       NA
 
       NA
  
 
  
 16
 
   2059813
 
   2060056
 
  ENSEMBL   UCSC 
 
  244
 
  5
 
 14984
 
 tss
 
 0.00855
 
 0.00750
 
 Not queried
 
  0.9523556
 
       NA
 
       NA
  
 
  
 1
 
  52520593
 
  52520979
 
  ENSEMBL   UCSC 
 
  387
 
  5
 
 529
 
 gene;tss;island
 
 0.02515
 
 0.01065
 
 Not queried
 
  0.9522937
 
       NA
 
       NA
  
 
  
 1
 
  64669336
 
  64669472
 
  ENSEMBL   UCSC 
 
  137
 
  6
 
 1669
 
 tss
 
 0.96510
 
 0.95169
 
 Not queried
 
  0.9522579
 
       NA
 
       NA
  
 
  
 6
 
 116937920
 
 116938100
 
  ENSEMBL   UCSC 
 
  181
 
  4
 
 34387
 
 gene
 
 0.04288
 
 0.12704
 
 Not queried
 
  0.9521357
 
       NA
 
       NA
  
 
  
 20
 
  37353032
 
  37353158
 
  ENSEMBL   UCSC 
 
  127
 
  7
 
 26119
 
 island
 
 0.01946
 
 0.04021
 
 Not queried
 
  0.9518287
 
       NA
 
       NA
  
 
  
 17
 
    202588
 
    202759
 
  ENSEMBL   UCSC 
 
  172
 
  4
 
 16945
 
 tss;island
 
 0.67434
 
 0.77424
 
 Not queried
 
  0.9516350
 
       NA
 
       NA
  
 
  
 11
 
    299390
 
    299668
 
  ENSEMBL   UCSC 
 
  279
 
  8
 
 7816
 
 island
 
 0.76844
 
 0.88134
 
 Not queried
 
  0.9515373
 
       NA
 
       NA
  
 
  
 8
 
 143580770
 
 143581070
 
  ENSEMBL   UCSC 
 
  301
 
  4
 
 39400
 
 gene;island
 
 0.58733
 
 0.83749
 
 Not queried
 
  0.9513350
 
       NA
 
       NA
  
 
  
 1
 
 167599527
 
 167599830
 
  ENSEMBL   UCSC 
 
  304
 
  4
 
 872
 
 gene
 
 0.04039
 
 0.05178
 
 Not queried
 
  0.9512750
 
       NA
 
       NA
  
 
  
 13
 
 112322853
 
 112323195
 
  ENSEMBL   UCSC 
 
  343
 
  4
 
 11872
 
 island
 
 0.84087
 
 0.92079
 
 Not queried
 
  0.9512450
 
       NA
 
       NA
  
 
  
 1
 
 166958221
 
 166958583
 
  ENSEMBL   UCSC 
 
  363
 
 11
 
 3931
 
 island
 
 0.83803
 
 0.91044
 
 Not queried
 
  0.9508758
 
       NA
 
       NA
  
 
  
 6
 
  33756678
 
  33756905
 
  ENSEMBL   UCSC 
 
  228
 
  4
 
 34234
 
 gene
 
 0.05221
 
 0.08589
 
 Not queried
 
  0.9507530
 
       NA
 
       NA
  
 
  
 5
 
 140344290
 
 140344745
 
  ENSEMBL   UCSC 
 
  456
 
  4
 
 31748
 
 gene;tss;island
 
 0.50667
 
 0.66508
 
 Not queried
 
  0.9507474
 
       NA
 
       NA
  
 
  
 12
 
  14133129
 
  14133426
 
  ENSEMBL   UCSC 
 
  298
 
  6
 
 9557
 
 tss
 
 0.05370
 
 0.16219
 
 Not queried
 
  0.9507453
 
       NA
 
       NA
  
 
  
 14
 
  24769112
 
  24769404
 
  ENSEMBL   UCSC 
 
  293
 
  7
 
 12015
 
 gene
 
 0.01888
 
 0.02874
 
 Not queried
 
  0.9507150
 
       NA
 
       NA
  
 
  
 7
 
 150754040
 
 150754283
 
  ENSEMBL   UCSC 
 
  244
 
  4
 
 37221
 
 gene;island
 
 0.13197
 
 0.25904
 
 Not queried
 
  0.9506166
 
       NA
 
       NA
  
 
  
 2
 
 173420778
 
 173421107
 
  ENSEMBL   UCSC 
 
  330
 
  4
 
 22888
 
 gene
 
 0.02355
 
 0.03847
 
 Not queried
 
  0.9504011
 
       NA
 
       NA
  
 
  
 X
 
  48455705
 
  48455861
 
  ENSEMBL   UCSC 
 
  157
 
  4
 
 41293
 
 tss;island
 
 0.05745
 
 0.03149
 
 Not queried
 
  0.9502493
 
       NA
 
       NA
  
 
  
 1
 
 228297092
 
 228297275
 
  ENSEMBL   UCSC 
 
  184
 
  5
 
 2294
 
 tss
 
 0.01629
 
 0.03100
 
 Not queried
 
  0.9500760
 
       NA
 
       NA
  
 
  
 19
 
   3762581
 
   3762740
 
  ENSEMBL   UCSC 
 
  160
 
  6
 
 20315
 
 tss;island
 
 0.00581
 
 0.02378
 
 Not queried
 
  0.9499302
 
       NA
 
       NA
  
 
  
 7
 
  65235546
 
  65235879
 
  ENSEMBL   UCSC 
 
  334
 
  6
 
 38466
 
 island
 
 0.04210
 
 0.08248
 
 Not queried
 
  0.9498490
 
       NA
 
       NA
  
 
  
 X
 
  99891595
 
  99892067
 
  ENSEMBL   UCSC 
 
  473
 
  9
 
 41901
 
 island
 
 0.01190
 
 0.00952
 
 Not queried
 
  0.9497560
 
       NA
 
       NA
  
 
  
 11
 
  65266482
 
  65266561
 
  ENSEMBL   UCSC 
 
   80
 
  4
 
 6690
 
 gene;island
 
 0.00724
 
 0.02467
 
 Not queried
 
  0.9493994
 
       NA
 
       NA
  
 
  
 5
 
   2642995
 
   2643299
 
  ENSEMBL   UCSC 
 
  305
 
  4
 
 32593
 
 island
 
 0.86557
 
 0.77796
 
 Not queried
 
  0.9493985
 
       NA
 
       NA
  
 
  
 12
 
 121881891
 
 121882415
 
  ENSEMBL   UCSC 
 
  525
 
  5
 
 9341
 
 gene;island
 
 0.72446
 
 0.87620
 
 Not queried
 
  0.9493166
 
       NA
 
       NA
  
 
  
 2
 
 120281354
 
 120282327
 
  ENSEMBL   UCSC 
 
  974
 
 11
 
 24619
 
 island
 
 0.07446
 
 0.31876
 
 Not queried
 
  0.9492493
 
       NA
 
       NA
  
 
  
 16
 
  68676451
 
  68676806
 
  ENSEMBL   UCSC 
 
  356
 
  5
 
 16016
 
 island
 
 0.03859
 
 0.01991
 
 Not queried
 
  0.9491576
 
       NA
 
       NA
  
 
  
 20
 
   2517085
 
   2517231
 
  ENSEMBL   UCSC 
 
  147
 
  4
 
 25507
 
 tss
 
 0.79786
 
 0.65909
 
 Not queried
 
  0.9491093
 
       NA
 
       NA
  
 
  
 X
 
  48334240
 
  48334539
 
  ENSEMBL   UCSC 
 
  300
 
  7
 
 41289
 
 tss
 
 0.03525
 
 0.01539
 
 Not queried
 
  0.9490930
 
       NA
 
       NA
  
 
  
 1
 
 151736090
 
 151736255
 
  ENSEMBL   UCSC 
 
  166
 
  5
 
 752
 
 gene;tss;island
 
 0.01872
 
 0.02929
 
 Not queried
 
  0.9486806
 
       NA
 
       NA
  
 
  
 5
 
 110848437
 
 110848688
 
  ENSEMBL   UCSC 
 
  252
 
  4
 
 32149
 
 tss;island
 
 0.02610
 
 0.02212
 
 Not queried
 
  0.9486656
 
       NA
 
       NA
  
 
  
 6
 
  33047944
 
  33048919
 
  ENSEMBL   UCSC 
 
  976
 
 26
 
 34121
 
 gene;island
 
 0.04643
 
 0.08218
 
 Not queried
 
  0.9486592
 
       NA
 
       NA
  
 
  
 6
 
  31914952
 
  31915198
 
  ENSEMBL   UCSC 
 
  247
 
  8
 
 33933
 
 gene
 
 0.52498
 
 0.68077
 
 Not queried
 
  0.9485285
 
       NA
 
       NA
  
 
  
 20
 
   1974738
 
   1974924
 
  ENSEMBL   UCSC 
 
  187
 
  4
 
 25501
 
 tss
 
 0.14169
 
 0.23377
 
 Not queried
 
  0.9484977
 
       NA
 
       NA
  
 
  
 13
 
  20797356
 
  20797693
 
  ENSEMBL   UCSC 
 
  338
 
  5
 
 11067
 
 gene
 
 0.69257
 
 0.81086
 
 Not queried
 
  0.9483845
 
       NA
 
       NA
  
 
  
 4
 
  71553882
 
  71554125
 
  ENSEMBL   UCSC 
 
  244
 
  8
 
 30374
 
 tss
 
 0.00874
 
 0.02209
 
 Not queried
 
  0.9482858
 
       NA
 
       NA
  
 
  
 9
 
 130186444
 
 130186736
 
  ENSEMBL   UCSC 
 
  293
 
  4
 
 40878
 
 island
 
 0.01929
 
 0.03090
 
 Not queried
 
  0.9481952
 
       NA
 
       NA
  
 
  
 12
 
  46121515
 
  46121883
 
  ENSEMBL   UCSC 
 
  369
 
  5
 
 10318
 
 island
 
 0.01112
 
 0.01405
 
 Not queried
 
  0.9478867
 
       NA
 
       NA
  
 
  
 7
 
 122527351
 
 122527522
 
  ENSEMBL   UCSC 
 
  172
 
  5
 
 37803
 
 tss;island
 
 0.02548
 
 0.04801
 
 Not queried
 
  0.9476870
 
       NA
 
       NA
  
 
  
 1
 
   6507693
 
   6508035
 
  ENSEMBL   UCSC 
 
  343
 
  5
 
 171
 
 gene;island
 
 0.40437
 
 0.57239
 
 Not queried
 
  0.9471633
 
       NA
 
       NA
  
 
  
 1
 
  94884039
 
  94884189
 
  ENSEMBL   UCSC 
 
  151
 
  4
 
 641
 
 gene;island
 
 0.01148
 
 0.02067
 
 Not queried
 
  0.9471573
 
       NA
 
       NA
  
 
  
 1
 
   3319264
 
   3319509
 
  ENSEMBL   UCSC 
 
  246
 
  4
 
 122
 
 gene;island
 
 0.67840
 
 0.76765
 
 Not queried
 
  0.9471486
 
       NA
 
       NA
  
 
  
 1
 
 226111643
 
 226112534
 
  ENSEMBL   UCSC 
 
  892
 
  8
 
 4238
 
 island
 
 0.05082
 
 0.07889
 
 Not queried
 
  0.9471111
 
       NA
 
       NA
  
 
  
 11
 
  65743851
 
  65744159
 
  ENSEMBL   UCSC 
 
  309
 
  5
 
 6701
 
 gene;island
 
 0.84527
 
 0.93112
 
 Not queried
 
  0.9470249
 
       NA
 
       NA
  
 
  
 6
 
 169224886
 
 169225041
 
  ENSEMBL   UCSC 
 
  156
 
  4
 
 36605
 
 island
 
 0.90212
 
 0.85924
 
 Not queried
 
  0.9468246
 
       NA
 
       NA
  
 
  
 6
 
  32812933
 
  32813715
 
  ENSEMBL   UCSC 
 
  783
 
 14
 
 34814
 
 tss;island
 
 0.89203
 
 0.71157
 
 Not queried
 
  0.9467601
 
       NA
 
       NA
  
 
  
 6
 
  30043727
 
  30044259
 
  ENSEMBL   UCSC 
 
  533
 
 10
 
 34699
 
 tss
 
 0.52680
 
 0.27357
 
 Not queried
 
  0.9467082
 
       NA
 
       NA
  
 
  
 11
 
 118016247
 
 118016368
 
  ENSEMBL   UCSC 
 
  122
 
  5
 
 6904
 
 gene;tss;island
 
 0.01631
 
 0.03097
 
 Not queried
 
  0.9465866
 
       NA
 
       NA
  
 
  
 5
 
  74807535
 
  74808051
 
  ENSEMBL   UCSC 
 
  517
 
 11
 
 31591
 
 gene;tss;island
 
 0.00838
 
 0.01867
 
 Not queried
 
  0.9465628
 
       NA
 
       NA
  
 
  
 16
 
  78133117
 
  78133395
 
  ENSEMBL   UCSC 
 
  279
 
  4
 
 15342
 
 tss;island
 
 0.01649
 
 0.00834
 
 Not queried
 
  0.9464317
 
       NA
 
       NA
  
 
  
 20
 
  43743835
 
  43744110
 
  ENSEMBL   UCSC 
 
  276
 
  5
 
 25730
 
 tss
 
 0.39983
 
 0.60126
 
 Not queried
 
  0.9462331
 
       NA
 
       NA
  
 
  
 6
 
  32632106
 
  32632338
 
  ENSEMBL   UCSC 
 
  233
 
  6
 
 34059
 
 gene;island
 
 0.63374
 
 0.74110
 
 Not queried
 
  0.9461851
 
       NA
 
       NA
  
 
  
 X
 
  21857475
 
  21857632
 
  ENSEMBL   UCSC 
 
  158
 
  6
 
 41222
 
 tss
 
 0.01760
 
 0.02855
 
 Not queried
 
  0.9461816
 
       NA
 
       NA
  
 
  
 2
 
 155554688
 
 155555053
 
  ENSEMBL   UCSC 
 
  366
 
  6
 
 23633
 
 tss
 
 0.10194
 
 0.24082
 
 Not queried
 
  0.9461644
 
       NA
 
       NA
  
 
  
 17
 
  46970175
 
  46970705
 
  ENSEMBL   UCSC 
 
  531
 
  6
 
 16634
 
 gene
 
 0.01514
 
 0.02042
 
 Not queried
 
  0.9460999
 
       NA
 
       NA
  
 
  
 19
 
  18717742
 
  18717783
 
  ENSEMBL   UCSC 
 
   42
 
  4
 
 20588
 
 tss
 
 0.04575
 
 0.10812
 
 Not queried
 
  0.9460013
 
       NA
 
       NA
  
 
  
 11
 
  72492171
 
  72492538
 
  ENSEMBL   UCSC 
 
  368
 
  4
 
 6800
 
 gene;island
 
 0.05102
 
 0.08399
 
 Not queried
 
  0.9459700
 
       NA
 
       NA
  
 
  
 3
 
 179754483
 
 179754761
 
  ENSEMBL   UCSC 
 
  279
 
  8
 
 29639
 
 island
 
 0.03714
 
 0.05998
 
 Not queried
 
  0.9456942
 
       NA
 
       NA
  
 
  
 15
 
  66161798
 
  66162152
 
  ENSEMBL   UCSC 
 
  355
 
  4
 
 13355
 
 gene
 
 0.01511
 
 0.02836
 
 Not queried
 
  0.9456831
 
       NA
 
       NA
  
 
  
 7
 
 157461081
 
 157461368
 
  ENSEMBL   UCSC 
 
  288
 
  5
 
 37275
 
 gene
 
 0.90469
 
 0.94048
 
 Not queried
 
  0.9456248
 
       NA
 
       NA
  
 
  
 6
 
  25652531
 
  25652815
 
  ENSEMBL   UCSC 
 
  285
 
  4
 
 33531
 
 gene
 
 0.02979
 
 0.07004
 
 Not queried
 
  0.9456018
 
       NA
 
       NA
  
 
  
 5
 
 176757391
 
 176757703
 
  ENSEMBL   UCSC 
 
  313
 
  4
 
 33382
 
 island
 
 0.73554
 
 0.84562
 
 Not queried
 
  0.9455414
 
       NA
 
       NA
  
 
  
 22
 
  22337034
 
  22337468
 
  ENSEMBL   UCSC 
 
  435
 
 11
 
 27301
 
 island
 
 0.02727
 
 0.06974
 
 Not queried
 
  0.9454449
 
       NA
 
       NA
  
 
  
 16
 
  70557093
 
  70557429
 
  ENSEMBL   UCSC 
 
  337
 
  5
 
 14798
 
 gene
 
 0.00820
 
 0.01716
 
 Not queried
 
  0.9451977
 
       NA
 
       NA
  
 
  
 18
 
   9102417
 
   9102542
 
  ENSEMBL   UCSC 
 
  126
 
  7
 
 19141
 
 tss;island
 
 0.02600
 
 0.02590
 
 Not queried
 
  0.9451927
 
       NA
 
       NA
  
 
  
 13
 
  21635722
 
  21635991
 
  ENSEMBL   UCSC 
 
  270
 
  8
 
 11307
 
 tss
 
 0.01716
 
 0.03167
 
 Not queried
 
  0.9451065
 
       NA
 
       NA
  
 
  
 3
 
 122399230
 
 122399601
 
  ENSEMBL   UCSC 
 
  372
 
  7
 
 28497
 
 tss
 
 0.43028
 
 0.61142
 
 Not queried
 
  0.9450389
 
       NA
 
       NA
  
 
  
 7
 
 150778724
 
 150779390
 
  ENSEMBL   UCSC 
 
  667
 
 14
 
 37224
 
 gene;tss;island
 
 0.53785
 
 0.81797
 
 Not queried
 
  0.9450024
 
       NA
 
       NA
  
 
  
 1
 
  27240319
 
  27240514
 
  ENSEMBL   UCSC 
 
  196
 
  4
 
 346
 
 gene
 
 0.90250
 
 0.78979
 
 Not queried
 
  0.9449825
 
       NA
 
       NA
  
 
  
 18
 
  63417605
 
  63417783
 
  ENSEMBL   UCSC 
 
  179
 
  4
 
 19089
 
 gene;tss;island
 
 0.02951
 
 0.01965
 
 Not queried
 
  0.9446846
 
       NA
 
       NA
  
 
  
 Y
 
   2802103
 
   2802332
 
  ENSEMBL   UCSC 
 
  230
 
  4
 
 42164
 
 tss;island
 
 0.24570
 
 0.25236
 
 Not queried
 
  0.9446560
 
       NA
 
       NA
  
 
  
 19
 
  51535231
 
  51535405
 
  ENSEMBL   UCSC 
 
  175
 
  4
 
 20153
 
 gene;island
 
 0.75229
 
 0.55219
 
 Not queried
 
  0.9446436
 
       NA
 
       NA
  
 
  
 21
 
  46875142
 
  46875433
 
  ENSEMBL   UCSC 
 
  292
 
  5
 
 26427
 
 gene;island
 
 0.82504
 
 0.87068
 
 Not queried
 
  0.9444956
 
       NA
 
       NA
  
 
  
 13
 
  20138761
 
  20139220
 
  ENSEMBL   UCSC 
 
  460
 
  5
 
 11531
 
 island
 
 0.27803
 
 0.08384
 
 Not queried
 
  0.9443986
 
       NA
 
       NA
  
 
  
 5
 
 172261146
 
 172261515
 
  ENSEMBL   UCSC 
 
  370
 
  5
 
 33335
 
 island
 
 0.03498
 
 0.03181
 
 Not queried
 
  0.9440874
 
       NA
 
       NA
  
 
  
 1
 
  27693187
 
  27693474
 
  ENSEMBL   UCSC 
 
  288
 
  7
 
 2911
 
 island
 
 0.01502
 
 0.03608
 
 Not queried
 
  0.9437433
 
       NA
 
       NA
  
 
  
 3
 
  42304966
 
  42305237
 
  ENSEMBL   UCSC 
 
  272
 
  5
 
 27689
 
 gene;island
 
 0.34664
 
 0.52937
 
 Not queried
 
  0.9437328
 
       NA
 
       NA
  
 
  
 7
 
   1708566
 
   1708892
 
  ENSEMBL   UCSC 
 
  327
 
  4
 
 38058
 
 island
 
 0.17597
 
 0.21896
 
 Not queried
 
  0.9434632
 
       NA
 
       NA
  
 
  
 7
 
 103630810
 
 103631129
 
  ENSEMBL   UCSC 
 
  320
 
  4
 
 37749
 
 tss;island
 
 0.11303
 
 0.23182
 
 Not queried
 
  0.9434171
 
       NA
 
       NA
  
 
  
 10
 
 118083701
 
 118083921
 
  ENSEMBL   UCSC 
 
  221
 
  5
 
 5367
 
 tss
 
 0.88939
 
 0.94748
 
 Not queried
 
  0.9427783
 
       NA
 
       NA
  
 
  
 11
 
  75140541
 
  75140865
 
  ENSEMBL   UCSC 
 
  325
 
  4
 
 6818
 
 gene;island
 
 0.87987
 
 0.72799
 
 Not queried
 
  0.9427665
 
       NA
 
       NA
  
 
  
 1
 
  28879563
 
  28879976
 
  ENSEMBL   UCSC 
 
  414
 
  4
 
 366
 
 gene
 
 0.02550
 
 0.06277
 
 Not queried
 
  0.9427643
 
       NA
 
       NA
  
 
  
 1
 
 156721823
 
 156722206
 
  ENSEMBL   UCSC 
 
  384
 
  9
 
 828
 
 gene
 
 0.02519
 
 0.02024
 
 Not queried
 
  0.9427464
 
       NA
 
       NA
  
 
  
 12
 
  50101354
 
  50101564
 
  ENSEMBL   UCSC 
 
  211
 
  6
 
 9668
 
 tss;island
 
 0.01771
 
 0.03153
 
 Not queried
 
  0.9425328
 
       NA
 
       NA
  
 
  
 7
 
  93550986
 
  93551014
 
  ENSEMBL   UCSC 
 
   29
 
  4
 
 37651
 
 tss
 
 0.05940
 
 0.04598
 
 Not queried
 
  0.9425118
 
       NA
 
       NA
  
 
  
 2
 
 173940203
 
 173940561
 
  ENSEMBL   UCSC 
 
  359
 
  6
 
 23683
 
 tss
 
 0.07592
 
 0.05298
 
 Not queried
 
  0.9423218
 
       NA
 
       NA
  
 
  
 2
 
 173940203
 
 173940639
 
  ENSEMBL   UCSC 
 
  437
 
  8
 
 24831
 
 island
 
 0.04459
 
 0.04482
 
 Not queried
 
  0.9423218
 
       NA
 
       NA
  
 
  
 6
 
  37322624
 
  37322835
 
  ENSEMBL   UCSC 
 
  212
 
  5
 
 34257
 
 gene;island
 
 0.04041
 
 0.10552
 
 Not queried
 
  0.9421385
 
       NA
 
       NA
  
 
  
 1
 
   8938701
 
   8938754
 
  ENSEMBL   UCSC 
 
   54
 
  4
 
 197
 
 gene
 
 0.01730
 
 0.01083
 
 Not queried
 
  0.9419685
 
       NA
 
       NA
  
 
  
 1
 
 200639189
 
 200639502
 
  ENSEMBL   UCSC 
 
  314
 
  5
 
 2157
 
 tss
 
 0.00632
 
 0.04060
 
 Not queried
 
  0.9419205
 
       NA
 
       NA
  
 
  
 17
 
  37793028
 
  37793326
 
  ENSEMBL   UCSC 
 
  299
 
  8
 
 17278
 
 tss
 
 0.02754
 
 0.04732
 
 Not queried
 
  0.9418015
 
       NA
 
       NA
  
 
  
 1
 
  24151609
 
  24151885
 
  ENSEMBL   UCSC 
 
  277
 
  4
 
 321
 
 gene
 
 0.01986
 
 0.01377
 
 Not queried
 
  0.9416810
 
       NA
 
       NA
  
 
  
 4
 
  39046023
 
  39046192
 
  ENSEMBL   UCSC 
 
  170
 
  4
 
 30300
 
 tss;island
 
 0.02812
 
 0.04356
 
 Not queried
 
  0.9414567
 
       NA
 
       NA
  
 
  
 10
 
  46090406
 
  46090807
 
  ENSEMBL   UCSC 
 
  402
 
  7
 
 5043
 
 tss
 
 0.01266
 
 0.03420
 
 Not queried
 
  0.9413548
 
       NA
 
       NA
  
 
  
 8
 
 143915776
 
 143916377
 
  ENSEMBL   UCSC 
 
  602
 
 10
 
 40581
 
 island
 
 0.87242
 
 0.78064
 
 Not queried
 
  0.9410974
 
       NA
 
       NA
  
 
  
 16
 
  30845651
 
  30846150
 
  ENSEMBL   UCSC 
 
  500
 
  5
 
 15818
 
 island
 
 0.92687
 
 0.86921
 
 Not queried
 
  0.9410796
 
       NA
 
       NA
  
 
  
 11
 
  67350153
 
  67350663
 
  ENSEMBL   UCSC 
 
  511
 
  7
 
 7476
 
 tss;island
 
 0.59074
 
 0.43599
 
 Not queried
 
  0.9409270
 
       NA
 
       NA
  
 
  
 2
 
  56150255
 
  56150675
 
  ENSEMBL   UCSC 
 
  421
 
  6
 
 22654
 
 gene;island
 
 0.05126
 
 0.06990
 
 Not queried
 
  0.9409220
 
       NA
 
       NA
  
 
  
 2
 
  56150475
 
  56150675
 
  ENSEMBL   UCSC 
 
  201
 
  4
 
 23327
 
 tss
 
 0.04015
 
 0.06990
 
 Not queried
 
  0.9409220
 
       NA
 
       NA
  
 
  
 1
 
  19638330
 
  19638460
 
  ENSEMBL   UCSC 
 
  131
 
  4
 
 280
 
 gene;tss;island
 
 0.06655
 
 0.19680
 
 Not queried
 
  0.9406932
 
       NA
 
       NA
  
 
  
 1
 
  85666673
 
  85666970
 
  ENSEMBL   UCSC 
 
  298
 
  6
 
 3414
 
 island
 
 0.01833
 
 0.03795
 
 Not queried
 
  0.9405151
 
       NA
 
       NA
  
 
  
 12
 
    678853
 
    679287
 
  ENSEMBL   UCSC 
 
  435
 
  4
 
 8972
 
 gene;island
 
 0.03688
 
 0.02432
 
 Not queried
 
  0.9404991
 
       NA
 
       NA
  
 
  
 6
 
 170494301
 
 170494475
 
  ENSEMBL   UCSC 
 
  175
 
  4
 
 36619
 
 island
 
 0.90680
 
 0.85065
 
 Not queried
 
  0.9404196
 
       NA
 
       NA
  
 
  
 2
 
 238289816
 
 238289897
 
  ENSEMBL   UCSC 
 
   82
 
  4
 
 23054
 
 gene
 
 0.87402
 
 0.81923
 
 Not queried
 
  0.9403109
 
       NA
 
       NA
  
 
  
 19
 
  11471954
 
  11472397
 
  ENSEMBL   UCSC 
 
  444
 
  4
 
 19801
 
 gene;island
 
 0.08744
 
 0.04098
 
 Not queried
 
  0.9402852
 
       NA
 
       NA
  
 
  
 11
 
   1858467
 
   1858737
 
  ENSEMBL   UCSC 
 
  271
 
  4
 
 6415
 
 gene
 
 0.50804
 
 0.66189
 
 Not queried
 
  0.9401802
 
       NA
 
       NA
  
 
  
 7
 
  91763737
 
  91764007
 
  ENSEMBL   UCSC 
 
  271
 
  7
 
 37019
 
 gene
 
 0.01236
 
 0.02345
 
 Not queried
 
  0.9400051
 
       NA
 
       NA
  
 
  
 7
 
  91763737
 
  91764605
 
  ENSEMBL   UCSC 
 
  869
 
 17
 
 38590
 
 island
 
 0.01236
 
 0.02471
 
 Not queried
 
  0.9400051
 
       NA
 
       NA
  
 
  
 7
 
   1499152
 
   1499391
 
  ENSEMBL   UCSC 
 
  240
 
  8
 
 37334
 
 tss
 
 0.02334
 
 0.06349
 
 Not queried
 
  0.9399390
 
       NA
 
       NA
  
 
  
 10
 
   8101307
 
   8101566
 
  ENSEMBL   UCSC 
 
  260
 
  4
 
 4477
 
 gene;island
 
 0.59948
 
 0.85906
 
 Not queried
 
  0.9398351
 
       NA
 
       NA
  
 
  
 6
 
 108454859
 
 108455338
 
  ENSEMBL   UCSC 
 
  480
 
  5
 
 36331
 
 island
 
 0.02566
 
 0.02945
 
 Not queried
 
  0.9398139
 
       NA
 
       NA
  
 
  
 17
 
   8791471
 
   8791860
 
  ENSEMBL   UCSC 
 
  390
 
  4
 
 16398
 
 gene;island
 
 0.83174
 
 0.69458
 
 Not queried
 
  0.9397286
 
       NA
 
       NA
  
 
  
 19
 
  13056423
 
  13056485
 
  ENSEMBL   UCSC 
 
   63
 
  4
 
 20486
 
 tss;island
 
 0.02697
 
 0.06642
 
 Not queried
 
  0.9396814
 
       NA
 
       NA
  
 
  
 6
 
 166418799
 
 166419138
 
  ENSEMBL   UCSC 
 
  340
 
  4
 
 36562
 
 island
 
 0.63233
 
 0.45142
 
 Not queried
 
  0.9396704
 
       NA
 
       NA
  
 
  
 X
 
 100548103
 
 100548242
 
  ENSEMBL   UCSC 
 
  140
 
  5
 
 41421
 
 tss
 
 0.86198
 
 0.94737
 
 Not queried
 
  0.9396649
 
       NA
 
       NA
  
 
  
 X
 
 100547965
 
 100548242
 
  ENSEMBL   UCSC 
 
  278
 
  7
 
 41910
 
 island
 
 0.85713
 
 0.92463
 
 Not queried
 
  0.9396649
 
       NA
 
       NA
  
 
  
 12
 
 107380930
 
 107381105
 
  ENSEMBL   UCSC 
 
  176
 
  6
 
 9948
 
 tss
 
 0.02625
 
 0.05047
 
 Not queried
 
  0.9391038
 
       NA
 
       NA
  
 
  
 1
 
  38470540
 
  38470665
 
  ENSEMBL   UCSC 
 
  126
 
  4
 
 440
 
 gene;island
 
 0.02224
 
 0.03886
 
 Not queried
 
  0.9389365
 
       NA
 
       NA
  
 
  
 X
 
  69281860
 
  69282357
 
  ENSEMBL   UCSC 
 
  498
 
  9
 
 41845
 
 island
 
 0.84028
 
 0.88523
 
 Not queried
 
  0.9388029
 
       NA
 
       NA
  
 
  
 14
 
  75894136
 
  75894395
 
  ENSEMBL   UCSC 
 
  260
 
  8
 
 12491
 
 tss;island
 
 0.13688
 
 0.03446
 
 Not queried
 
  0.9381217
 
       NA
 
       NA
  
 
  
 11
 
  63706246
 
  63706312
 
  ENSEMBL   UCSC 
 
   67
 
  4
 
 7359
 
 tss
 
 0.02666
 
 0.04982
 
 Not queried
 
  0.9380984
 
       NA
 
       NA
  
 
  
 11
 
  44087367
 
  44087867
 
  ENSEMBL   UCSC 
 
  501
 
 10
 
 8199
 
 island
 
 0.05037
 
 0.01988
 
 Not queried
 
  0.9379931
 
       NA
 
       NA
  
 
  
 21
 
  43430222
 
  43430494
 
  ENSEMBL   UCSC 
 
  273
 
  4
 
 26393
 
 gene
 
 0.02062
 
 0.03039
 
 Not queried
 
  0.9378291
 
       NA
 
       NA
  
 
  
 21
 
  43430222
 
  43430507
 
  ENSEMBL   UCSC 
 
  286
 
  6
 
 26657
 
 island
 
 0.01984
 
 0.02497
 
 Not queried
 
  0.9378291
 
       NA
 
       NA
  
 
  
 19
 
   3942158
 
   3942330
 
  ENSEMBL   UCSC 
 
  173
 
  4
 
 19690
 
 gene;island
 
 0.77492
 
 0.85430
 
 Not queried
 
  0.9377747
 
       NA
 
       NA
  
 
  
 1
 
 151226935
 
 151227126
 
  ENSEMBL   UCSC 
 
  192
 
  4
 
 1921
 
 tss
 
 0.04403
 
 0.06714
 
 Not queried
 
  0.9377489
 
       NA
 
       NA
  
 
  
 1
 
  23751134
 
  23751761
 
  ENSEMBL   UCSC 
 
  628
 
  9
 
 2829
 
 island
 
 0.11292
 
 0.16256
 
 Not queried
 
  0.9377402
 
       NA
 
       NA
  
 
  
 10
 
  95242111
 
  95242332
 
  ENSEMBL   UCSC 
 
  222
 
  7
 
 5219
 
 tss
 
 0.01416
 
 0.04421
 
 Not queried
 
  0.9376738
 
       NA
 
       NA
  
 
  
 1
 
 219347252
 
 219347458
 
  ENSEMBL   UCSC 
 
  207
 
  5
 
 1010
 
 gene
 
 0.02603
 
 0.03016
 
 Not queried
 
  0.9375294
 
       NA
 
       NA
  
 
  
 10
 
 103824819
 
 103825210
 
  ENSEMBL   UCSC 
 
  392
 
  6
 
 6058
 
 island
 
 0.01476
 
 0.02281
 
 Not queried
 
  0.9372460
 
       NA
 
       NA
  
 
  
 3
 
  49757239
 
  49757476
 
  ENSEMBL   UCSC 
 
  238
 
  5
 
 28291
 
 tss
 
 0.26017
 
 0.86941
 
 Not queried
 
  0.9369828
 
       NA
 
       NA
  
 
  
 3
 
 156393520
 
 156393772
 
  ENSEMBL   UCSC 
 
  253
 
  5
 
 27964
 
 gene;tss;island
 
 0.01866
 
 0.03513
 
 Not queried
 
  0.9368631
 
       NA
 
       NA
  
 
  
 17
 
  56595363
 
  56595606
 
  ENSEMBL   UCSC 
 
  244
 
  5
 
 17504
 
 tss
 
 0.01932
 
 0.00851
 
 Not queried
 
  0.9367959
 
       NA
 
       NA
  
 
  
 2
 
  55509105
 
  55509334
 
  ENSEMBL   UCSC 
 
  230
 
  5
 
 23324
 
 tss
 
 0.28320
 
 0.43063
 
 Not queried
 
  0.9367939
 
       NA
 
       NA
  
 
  
 17
 
    171006
 
    171434
 
  ENSEMBL   UCSC 
 
  429
 
  5
 
 16254
 
 gene;island
 
 0.91120
 
 0.84279
 
 Not queried
 
  0.9367417
 
       NA
 
       NA
  
 
  
 20
 
  62209427
 
  62209642
 
  ENSEMBL   UCSC 
 
  216
 
  4
 
 26320
 
 island
 
 0.87896
 
 0.90334
 
 Not queried
 
  0.9366599
 
       NA
 
       NA
  
 
  
 10
 
   7709639
 
   7709962
 
  ENSEMBL   UCSC 
 
  324
 
  4
 
 4923
 
 tss
 
 0.11433
 
 0.07360
 
 Not queried
 
  0.9364793
 
       NA
 
       NA
  
 
  
 7
 
 139763542
 
 139763773
 
  ENSEMBL   UCSC 
 
  232
 
  4
 
 37871
 
 tss;island
 
 0.85317
 
 0.73678
 
 Not queried
 
  0.9362754
 
       NA
 
       NA
  
 
  
 1
 
    805338
 
    805554
 
  ENSEMBL   UCSC 
 
  217
 
  4
 
 1
 
 gene;island
 
 0.33323
 
 0.41018
 
 Not queried
 
  0.9361296
 
       NA
 
       NA
  
 
  
 12
 
 133306722
 
 133306893
 
  ENSEMBL   UCSC 
 
  172
 
  4
 
 9431
 
 gene;island
 
 0.96226
 
 0.97987
 
 Not queried
 
  0.9361253
 
       NA
 
       NA
  
 
  
 15
 
  34875117
 
  34875325
 
  ENSEMBL   UCSC 
 
  209
 
  4
 
 13250
 
 gene;island
 
 0.01473
 
 0.02253
 
 Not queried
 
  0.9358235
 
       NA
 
       NA
  
 
  
 16
 
  89634447
 
  89634715
 
  ENSEMBL   UCSC 
 
  269
 
  4
 
 16229
 
 island
 
 0.81899
 
 0.88199
 
 Not queried
 
  0.9357549
 
       NA
 
       NA
  
 
  
 11
 
 131779469
 
 131780499
 
  ENSEMBL   UCSC 
 
 1031
 
 18
 
 6958
 
 gene;tss;island
 
 0.58056
 
 0.41874
 
 Not queried
 
  0.9356999
 
       NA
 
       NA
  
 
  
 2
 
  85107190
 
  85107488
 
  ENSEMBL   UCSC 
 
  299
 
  5
 
 22722
 
 gene;island
 
 0.11186
 
 0.02491
 
 Not queried
 
  0.9355682
 
       NA
 
       NA
  
 
  
 1
 
  53925109
 
  53925368
 
  ENSEMBL   UCSC 
 
  260
 
  4
 
 540
 
 gene
 
 0.93143
 
 0.85315
 
 Not queried
 
  0.9352873
 
       NA
 
       NA
  
 
  
 1
 
  53924998
 
  53925368
 
  ENSEMBL   UCSC 
 
  371
 
  6
 
 3278
 
 island
 
 0.94479
 
 0.88927
 
 Not queried
 
  0.9352873
 
       NA
 
       NA
  
 
  
 4
 
    652527
 
    652907
 
  ENSEMBL   UCSC 
 
  381
 
  6
 
 29787
 
 gene;island
 
 0.89194
 
 0.93410
 
 Not queried
 
  0.9352758
 
       NA
 
       NA
  
 
  
 6
 
  88875236
 
  88875844
 
  ENSEMBL   UCSC 
 
  609
 
  8
 
 36253
 
 island
 
 0.01597
 
 0.02939
 
 Not queried
 
  0.9352268
 
       NA
 
       NA
  
 
  
 15
 
  74907203
 
  74907362
 
  ENSEMBL   UCSC 
 
  160
 
  5
 
 13396
 
 gene;island
 
 0.01767
 
 0.03394
 
 Not queried
 
  0.9349461
 
       NA
 
       NA
  
 
  
 5
 
 139927007
 
 139927242
 
  ENSEMBL   UCSC 
 
  236
 
  8
 
 32283
 
 tss
 
 0.00904
 
 0.01929
 
 Not queried
 
  0.9348548
 
       NA
 
       NA
  
 
  
 2
 
 176994142
 
 176994448
 
  ENSEMBL   UCSC 
 
  307
 
  5
 
 23701
 
 tss
 
 0.00888
 
 0.02209
 
 Not queried
 
  0.9347843
 
       NA
 
       NA
  
 
  
 3
 
 194090477
 
 194090730
 
  ENSEMBL   UCSC 
 
  254
 
  5
 
 28756
 
 tss
 
 0.30592
 
 0.39141
 
 Not queried
 
  0.9347560
 
       NA
 
       NA
  
 
  
 6
 
  33254385
 
  33254611
 
  ENSEMBL   UCSC 
 
  227
 
  6
 
 34184
 
 gene;island
 
 0.94123
 
 0.94838
 
 Not queried
 
  0.9347138
 
       NA
 
       NA
  
 
  
 15
 
  91642909
 
  91643135
 
  ENSEMBL   UCSC 
 
  227
 
  9
 
 13882
 
 tss;island
 
 0.03832
 
 0.05096
 
 Not queried
 
  0.9346448
 
       NA
 
       NA
  
 
  
 5
 
 151304409
 
 151304559
 
  ENSEMBL   UCSC 
 
  151
 
  6
 
 32385
 
 tss
 
 0.15196
 
 0.25029
 
 Not queried
 
  0.9345491
 
       NA
 
       NA
  
 
  
 17
 
  26972196
 
  26972480
 
  ENSEMBL   UCSC 
 
  285
 
  5
 
 17182
 
 tss
 
 0.01231
 
 0.03929
 
 Not queried
 
  0.9343468
 
       NA
 
       NA
  
 
  
 12
 
   6419409
 
   6419750
 
  ENSEMBL   UCSC 
 
  342
 
  6
 
 9481
 
 tss;island
 
 0.11095
 
 0.07324
 
 Not queried
 
  0.9342760
 
       NA
 
       NA
  
 
  
 8
 
  99306716
 
  99306953
 
  ENSEMBL   UCSC 
 
  238
 
  6
 
 39772
 
 tss;island
 
 0.02285
 
 0.03851
 
 Not queried
 
  0.9341679
 
       NA
 
       NA
  
 
  
 4
 
   1205914
 
   1206150
 
  ENSEMBL   UCSC 
 
  237
 
  4
 
 29818
 
 gene;island
 
 0.84899
 
 0.92511
 
 Not queried
 
  0.9340069
 
       NA
 
       NA
  
 
  
 3
 
  52813652
 
  52813920
 
  ENSEMBL   UCSC 
 
  269
 
  6
 
 27784
 
 gene;tss
 
 0.48974
 
 0.65882
 
 Not queried
 
  0.9339405
 
       NA
 
       NA
  
 
  
 1
 
  27998703
 
  27998988
 
  ENSEMBL   UCSC 
 
  286
 
  6
 
 2922
 
 island
 
 0.03402
 
 0.04742
 
 Not queried
 
  0.9333171
 
       NA
 
       NA
  
 
  
 11
 
  85358760
 
  85359022
 
  ENSEMBL   UCSC 
 
  263
 
  8
 
 7583
 
 tss
 
 0.01658
 
 0.01927
 
 Not queried
 
  0.9332055
 
       NA
 
       NA
  
 
  
 7
 
  91763850
 
  91764605
 
  ENSEMBL   UCSC 
 
  756
 
 16
 
 37638
 
 tss
 
 0.01469
 
 0.02525
 
 Not queried
 
  0.9331993
 
       NA
 
       NA
  
 
  
 11
 
 129939817
 
 129939958
 
  ENSEMBL   UCSC 
 
  142
 
  4
 
 6952
 
 gene
 
 0.02423
 
 0.03775
 
 Not queried
 
  0.9331336
 
       NA
 
       NA
  
 
  
 12
 
  71552188
 
  71552569
 
  ENSEMBL   UCSC 
 
  382
 
  4
 
 9845
 
 tss
 
 0.85121
 
 0.90476
 
 Not queried
 
  0.9328719
 
       NA
 
       NA
  
 
  
 7
 
    610887
 
    611487
 
  ENSEMBL   UCSC 
 
  601
 
  5
 
 36649
 
 gene;island
 
 0.90812
 
 0.93985
 
 Not queried
 
  0.9328605
 
       NA
 
       NA
  
 
  
 18
 
  29523271
 
  29523426
 
  ENSEMBL   UCSC 
 
  156
 
  6
 
 19188
 
 tss
 
 0.02005
 
 0.04153
 
 Not queried
 
  0.9327599
 
       NA
 
       NA
  
 
  
 4
 
 142141972
 
 142142045
 
  ENSEMBL   UCSC 
 
   74
 
  5
 
 30562
 
 tss
 
 0.01365
 
 0.03118
 
 Not queried
 
  0.9326100
 
       NA
 
       NA
  
 
  
 19
 
   7852051
 
   7852238
 
  ENSEMBL   UCSC 
 
  188
 
  6
 
 20371
 
 tss;island
 
 0.70198
 
 0.50174
 
 Not queried
 
  0.9318682
 
       NA
 
       NA
  
 
  
 7
 
  21985589
 
  21985935
 
  ENSEMBL   UCSC 
 
  347
 
  8
 
 37399
 
 tss;island
 
 0.02320
 
 0.02042
 
 Not queried
 
  0.9318649
 
       NA
 
       NA
  
 
  
 19
 
   4045025
 
   4045108
 
  ENSEMBL   UCSC 
 
   84
 
  4
 
 21326
 
 island
 
 0.85297
 
 0.92279
 
 Not queried
 
  0.9316865
 
       NA
 
       NA
  
 
  
 12
 
   7052614
 
   7053170
 
  ENSEMBL   UCSC 
 
  557
 
  9
 
 9511
 
 tss
 
 0.02930
 
 0.04331
 
 Not queried
 
  0.9314561
 
       NA
 
       NA
  
 
  
 15
 
  71184279
 
  71184618
 
  ENSEMBL   UCSC 
 
  340
 
  5
 
 13372
 
 gene
 
 0.03266
 
 0.04561
 
 Not queried
 
  0.9313670
 
       NA
 
       NA
  
 
  
 16
 
  70323367
 
  70323915
 
  ENSEMBL   UCSC 
 
  549
 
 10
 
 16034
 
 island
 
 0.01334
 
 0.01859
 
 Not queried
 
  0.9312675
 
       NA
 
       NA
  
 
  
 6
 
  35772606
 
  35772999
 
  ENSEMBL   UCSC 
 
  394
 
  8
 
 34868
 
 tss;island
 
 0.01316
 
 0.02837
 
 Not queried
 
  0.9311368
 
       NA
 
       NA
  
 
  
 5
 
   3535836
 
   3536279
 
  ENSEMBL   UCSC 
 
  444
 
  5
 
 32603
 
 island
 
 0.88284
 
 0.81013
 
 Not queried
 
  0.9310855
 
       NA
 
       NA
  
 
  
 7
 
 111202666
 
 111202755
 
  ENSEMBL   UCSC 
 
   90
 
  4
 
 37774
 
 tss;island
 
 0.03487
 
 0.05398
 
 Not queried
 
  0.9310350
 
       NA
 
       NA
  
 
  
 14
 
  74705942
 
  74706113
 
  ENSEMBL   UCSC 
 
  172
 
  6
 
 12477
 
 tss
 
 0.62892
 
 0.41446
 
 Not queried
 
  0.9309011
 
       NA
 
       NA
  
 
  
 7
 
  96651915
 
  96652481
 
  ENSEMBL   UCSC 
 
  567
 
 10
 
 37043
 
 gene;island
 
 0.57268
 
 0.45743
 
 Not queried
 
  0.9304705
 
       NA
 
       NA
  
 
  
 19
 
  57149436
 
  57149813
 
  ENSEMBL   UCSC 
 
  378
 
  5
 
 22414
 
 island
 
 0.01911
 
 0.04410
 
 Not queried
 
  0.9303850
 
       NA
 
       NA
  
 
  
 11
 
  16628281
 
  16628944
 
  ENSEMBL   UCSC 
 
  664
 
  6
 
 8068
 
 island
 
 0.05125
 
 0.11969
 
 Not queried
 
  0.9303756
 
       NA
 
       NA
  
 
  
 6
 
  35309867
 
  35310274
 
  ENSEMBL   UCSC 
 
  408
 
  7
 
 34865
 
 tss;island
 
 0.08404
 
 0.09660
 
 Not queried
 
  0.9298987
 
       NA
 
       NA
  
 
  
 17
 
   7258337
 
   7258501
 
  ENSEMBL   UCSC 
 
  165
 
  4
 
 17907
 
 island
 
 0.61293
 
 0.44669
 
 Not queried
 
  0.9297945
 
       NA
 
       NA
  
 
  
 7
 
 102920641
 
 102921054
 
  ENSEMBL   UCSC 
 
  414
 
  9
 
 38743
 
 island
 
 0.17885
 
 0.29324
 
 Not queried
 
  0.9297679
 
       NA
 
       NA
  
 
  
 19
 
   8601136
 
   8601423
 
  ENSEMBL   UCSC 
 
  288
 
  4
 
 19760
 
 gene;island
 
 0.39446
 
 0.53416
 
 Not queried
 
  0.9295506
 
       NA
 
       NA
  
 
  
 19
 
  46580305
 
  46580807
 
  ENSEMBL   UCSC 
 
  503
 
  4
 
 22134
 
 island
 
 0.02429
 
 0.04440
 
 Not queried
 
  0.9294972
 
       NA
 
       NA
  
 
  
 7
 
  12443880
 
  12444290
 
  ENSEMBL   UCSC 
 
  411
 
  7
 
 37379
 
 tss
 
 0.03471
 
 0.01480
 
 Not queried
 
  0.9294526
 
       NA
 
       NA
  
 
  
 16
 
  89033692
 
  89034292
 
  ENSEMBL   UCSC 
 
  601
 
  7
 
 14895
 
 gene;island
 
 0.71812
 
 0.84184
 
 Not queried
 
  0.9294106
 
       NA
 
       NA
  
 
  
 15
 
  48470205
 
  48470516
 
  ENSEMBL   UCSC 
 
  312
 
  4
 
 13307
 
 gene
 
 0.03053
 
 0.05626
 
 Not queried
 
  0.9291030
 
       NA
 
       NA
  
 
  
 6
 
 127840328
 
 127840755
 
  ENSEMBL   UCSC 
 
  428
 
 10
 
 36411
 
 island
 
 0.04553
 
 0.03077
 
 Not queried
 
  0.9290859
 
       NA
 
       NA
  
 
  
 20
 
  57485765
 
  57486076
 
  ENSEMBL   UCSC 
 
  312
 
  9
 
 25439
 
 gene
 
 0.96658
 
 0.93825
 
 Not queried
 
  0.9289037
 
       NA
 
       NA
  
 
  
 1
 
   1974661
 
   1974941
 
  ENSEMBL   UCSC 
 
  281
 
  5
 
 2465
 
 island
 
 0.76522
 
 0.78676
 
 Not queried
 
  0.9287837
 
       NA
 
       NA
  
 
  
 5
 
 176944032
 
 176944204
 
  ENSEMBL   UCSC 
 
  173
 
  7
 
 32472
 
 tss
 
 0.04250
 
 0.06152
 
 Not queried
 
  0.9287517
 
       NA
 
       NA
  
 
  
 15
 
  44085795
 
  44086040
 
  ENSEMBL   UCSC 
 
  246
 
  4
 
 13290
 
 gene;island
 
 0.63231
 
 0.79661
 
 Not queried
 
  0.9286826
 
       NA
 
       NA
  
 
  
 20
 
  62680986
 
  62681428
 
  ENSEMBL   UCSC 
 
  443
 
  9
 
 25870
 
 tss
 
 0.04219
 
 0.09610
 
 Not queried
 
  0.9286060
 
       NA
 
       NA
  
 
  
 3
 
  72897627
 
  72897842
 
  ENSEMBL   UCSC 
 
  216
 
  7
 
 28402
 
 tss
 
 0.01217
 
 0.01538
 
 Not queried
 
  0.9282515
 
       NA
 
       NA
  
 
  
 3
 
  72897540
 
  72897842
 
  ENSEMBL   UCSC 
 
  303
 
  9
 
 29240
 
 island
 
 0.01393
 
 0.01538
 
 Not queried
 
  0.9282515
 
       NA
 
       NA
  
 
  
 4
 
  17710804
 
  17711202
 
  ENSEMBL   UCSC 
 
  399
 
  4
 
 29918
 
 gene;island
 
 0.81268
 
 0.89764
 
 Not queried
 
  0.9280165
 
       NA
 
       NA
  
 
  
 19
 
  32836297
 
  32836510
 
  ENSEMBL   UCSC 
 
  214
 
  8
 
 20639
 
 tss
 
 0.02793
 
 0.06031
 
 Not queried
 
  0.9278758
 
       NA
 
       NA
  
 
  
 20
 
   6104215
 
   6104274
 
  ENSEMBL   UCSC 
 
   60
 
  4
 
 25550
 
 tss
 
 0.34125
 
 0.21313
 
 Not queried
 
  0.9278685
 
       NA
 
       NA
  
 
  
 14
 
  74486154
 
  74486355
 
  ENSEMBL   UCSC 
 
  202
 
  5
 
 12109
 
 gene;tss;island
 
 0.03671
 
 0.06120
 
 Not queried
 
  0.9278528
 
       NA
 
       NA
  
 
  
 2
 
 227663533
 
 227663911
 
  ENSEMBL   UCSC 
 
  379
 
  6
 
 23889
 
 tss;island
 
 0.02379
 
 0.02560
 
 Not queried
 
  0.9278005
 
       NA
 
       NA
  
 
  
 20
 
  61809557
 
  61809841
 
  ENSEMBL   UCSC 
 
  285
 
  7
 
 25850
 
 tss
 
 0.03777
 
 0.02989
 
 Not queried
 
  0.9277945
 
       NA
 
       NA
  
 
  
 2
 
 233877969
 
 233878150
 
  ENSEMBL   UCSC 
 
  182
 
  4
 
 23917
 
 tss
 
 0.82094
 
 0.73241
 
 Not queried
 
  0.9277318
 
       NA
 
       NA
  
 
  
 8
 
   1049167
 
   1049477
 
  ENSEMBL   UCSC 
 
  311
 
  5
 
 39946
 
 island
 
 0.84780
 
 0.89564
 
 Not queried
 
  0.9276717
 
       NA
 
       NA
  
 
  
 2
 
  74730266
 
  74730948
 
  ENSEMBL   UCSC 
 
  683
 
 11
 
 22708
 
 gene;island
 
 0.03879
 
 0.08812
 
 Not queried
 
  0.9275349
 
       NA
 
       NA
  
 
  
 14
 
  60097124
 
  60097536
 
  ENSEMBL   UCSC 
 
  413
 
  5
 
 12071
 
 gene;island
 
 0.01621
 
 0.02770
 
 Not queried
 
  0.9275041
 
       NA
 
       NA
  
 
  
 7
 
 158279694
 
 158279891
 
  ENSEMBL   UCSC 
 
  198
 
  4
 
 37312
 
 gene;island
 
 0.92532
 
 0.84735
 
 Not queried
 
  0.9274981
 
       NA
 
       NA
  
 
  
 19
 
  36035831
 
  36036175
 
  ENSEMBL   UCSC 
 
  345
 
  5
 
 19950
 
 gene
 
 0.05249
 
 0.03783
 
 Not queried
 
  0.9274449
 
       NA
 
       NA
  
 
  
 6
 
  32297615
 
  32297861
 
  ENSEMBL   UCSC 
 
  247
 
  4
 
 34039
 
 gene
 
 0.86014
 
 0.76564
 
 Not queried
 
  0.9274290
 
       NA
 
       NA
  
 
  
 3
 
 185216311
 
 185216613
 
  ENSEMBL   UCSC 
 
  303
 
  4
 
 28026
 
 gene;island
 
 0.03497
 
 0.04387
 
 Not queried
 
  0.9274289
 
       NA
 
       NA
  
 
  
 16
 
  58426207
 
  58426274
 
  ENSEMBL   UCSC 
 
   68
 
  5
 
 15240
 
 tss
 
 0.02590
 
 0.04232
 
 Not queried
 
  0.9272800
 
       NA
 
       NA
  
 
  
 1
 
  11986612
 
  11986842
 
  ENSEMBL   UCSC 
 
  231
 
  5
 
 1250
 
 tss;island
 
 0.04162
 
 0.05155
 
 Not queried
 
  0.9271824
 
       NA
 
       NA
  
 
  
 11
 
   1975236
 
   1975560
 
  ENSEMBL   UCSC 
 
  325
 
  4
 
 6423
 
 gene;island
 
 0.74797
 
 0.84634
 
 Not queried
 
  0.9270810
 
       NA
 
       NA
  
 
  
 5
 
 180230113
 
 180230146
 
  ENSEMBL   UCSC 
 
   34
 
  4
 
 32501
 
 tss
 
 0.19089
 
 0.11232
 
 Not queried
 
  0.9269363
 
       NA
 
       NA
  
 
  
 X
 
   3235329
 
   3235730
 
  ENSEMBL   UCSC 
 
  402
 
  4
 
 40925
 
 gene;island
 
 0.81443
 
 0.90926
 
 Not queried
 
  0.9267373
 
       NA
 
       NA
  
 
  
 6
 
  31634602
 
  31634890
 
  ENSEMBL   UCSC 
 
  289
 
  6
 
 33871
 
 gene;island
 
 0.38614
 
 0.69730
 
 Not queried
 
  0.9267136
 
       NA
 
       NA
  
 
  
 4
 
 186435443
 
 186435667
 
  ENSEMBL   UCSC 
 
  225
 
  6
 
 30163
 
 gene
 
 0.86399
 
 0.95239
 
 Not queried
 
  0.9266881
 
       NA
 
       NA
  
 
  
 7
 
  95401692
 
  95401856
 
  ENSEMBL   UCSC 
 
  165
 
  5
 
 38619
 
 island
 
 0.03834
 
 0.06685
 
 Not queried
 
  0.9262865
 
       NA
 
       NA
  
 
  
 1
 
  55504649
 
  55505532
 
  ENSEMBL   UCSC 
 
  884
 
 13
 
 3299
 
 island
 
 0.44671
 
 0.69405
 
 Not queried
 
  0.9260004
 
       NA
 
       NA
  
 
  
 3
 
  98452812
 
  98453086
 
  ENSEMBL   UCSC 
 
  275
 
  4
 
 27831
 
 gene;island
 
 0.17634
 
 0.07175
 
 Not queried
 
  0.9259855
 
       NA
 
       NA
  
 
  
 17
 
  73501021
 
  73501230
 
  ENSEMBL   UCSC 
 
  210
 
  5
 
 16731
 
 gene;island
 
 0.64495
 
 0.79888
 
 Not queried
 
  0.9259176
 
       NA
 
       NA
  
 
  
 3
 
 197475984
 
 197476591
 
  ENSEMBL   UCSC 
 
  608
 
 12
 
 28064
 
 gene;tss;island
 
 0.02593
 
 0.04591
 
 Not queried
 
  0.9258885
 
       NA
 
       NA
  
 
  
 6
 
  80714048
 
  80714474
 
  ENSEMBL   UCSC 
 
  427
 
 10
 
 36217
 
 island
 
 0.01814
 
 0.03005
 
 Not queried
 
  0.9258592
 
       NA
 
       NA
  
 
  
 22
 
  24891129
 
  24891452
 
  ENSEMBL   UCSC 
 
  324
 
  8
 
 26979
 
 tss;island
 
 0.92273
 
 0.95948
 
 Not queried
 
  0.9255975
 
       NA
 
       NA
  
 
  
 1
 
  33115703
 
  33116289
 
  ENSEMBL   UCSC 
 
  587
 
  5
 
 1459
 
 tss;island
 
 0.01626
 
 0.02109
 
 Not queried
 
  0.9255552
 
       NA
 
       NA
  
 
  
 1
 
  22777672
 
  22777839
 
  ENSEMBL   UCSC 
 
  168
 
  4
 
 1328
 
 tss;island
 
 0.96334
 
 0.91132
 
 Not queried
 
  0.9255388
 
       NA
 
       NA
  
 
  
 6
 
  29618205
 
  29618382
 
  ENSEMBL   UCSC 
 
  178
 
  4
 
 35560
 
 island
 
 0.10543
 
 0.17972
 
 Not queried
 
  0.9255022
 
       NA
 
       NA
  
 
  
 5
 
   1645268
 
   1645640
 
  ENSEMBL   UCSC 
 
  373
 
  5
 
 32578
 
 island
 
 0.30055
 
 0.18449
 
 Not queried
 
  0.9254711
 
       NA
 
       NA
  
 
  
 X
 
 103499636
 
 103500137
 
  ENSEMBL   UCSC 
 
  502
 
 10
 
 41450
 
 tss
 
 0.01905
 
 0.03626
 
 Not queried
 
  0.9252321
 
       NA
 
       NA
  
 
  
 10
 
 126106425
 
 126106614
 
  ENSEMBL   UCSC 
 
  190
 
  4
 
 4790
 
 gene;island
 
 0.02319
 
 0.01098
 
 Not queried
 
  0.9249598
 
       NA
 
       NA
  
 
  
 4
 
  47839876
 
  47840162
 
  ENSEMBL   UCSC 
 
  287
 
  6
 
 30973
 
 island
 
 0.03761
 
 0.07804
 
 Not queried
 
  0.9248609
 
       NA
 
       NA
  
 
  
 12
 
 132690643
 
 132690782
 
  ENSEMBL   UCSC 
 
  140
 
  5
 
 10103
 
 tss
 
 0.79122
 
 0.85621
 
 Not queried
 
  0.9248510
 
       NA
 
       NA
  
 
  
 16
 
  84599875
 
  84600468
 
  ENSEMBL   UCSC 
 
  594
 
  6
 
 14839
 
 gene
 
 0.83122
 
 0.88528
 
 Not queried
 
  0.9247798
 
       NA
 
       NA
  
 
  
 13
 
  35515865
 
  35516223
 
  ENSEMBL   UCSC 
 
  359
 
  4
 
 11361
 
 tss
 
 0.04620
 
 0.02473
 
 Not queried
 
  0.9247586
 
       NA
 
       NA
  
 
  
 14
 
  65453398
 
  65453466
 
  ENSEMBL   UCSC 
 
   69
 
  5
 
 12437
 
 tss
 
 0.01452
 
 0.02673
 
 Not queried
 
  0.9246660
 
       NA
 
       NA
  
 
  
 5
 
 154317806
 
 154318104
 
  ENSEMBL   UCSC 
 
  299
 
  9
 
 32393
 
 tss
 
 0.03211
 
 0.05503
 
 Not queried
 
  0.9245957
 
       NA
 
       NA
  
 
  
 5
 
 154317555
 
 154318104
 
  ENSEMBL   UCSC 
 
  550
 
 11
 
 33274
 
 island
 
 0.03211
 
 0.05503
 
 Not queried
 
  0.9245957
 
       NA
 
       NA
  
 
  
 6
 
  31794278
 
  31794519
 
  ENSEMBL   UCSC 
 
  242
 
  4
 
 34776
 
 tss;island
 
 0.66260
 
 0.32106
 
 Not queried
 
  0.9244996
 
       NA
 
       NA
  
 
  
 16
 
  15595854
 
  15596082
 
  ENSEMBL   UCSC 
 
  229
 
  6
 
 15076
 
 tss
 
 0.78650
 
 0.86569
 
 Not queried
 
  0.9241347
 
       NA
 
       NA
  
 
  
 6
 
  28249142
 
  28249271
 
  ENSEMBL   UCSC 
 
  130
 
  7
 
 34675
 
 tss
 
 0.03216
 
 0.04989
 
 Not queried
 
  0.9239428
 
       NA
 
       NA
  
 
  
 2
 
 176944273
 
 176944601
 
  ENSEMBL   UCSC 
 
  329
 
  4
 
 24848
 
 island
 
 0.02590
 
 0.04192
 
 Not queried
 
  0.9238738
 
       NA
 
       NA
  
 
  
 21
 
  43989949
 
  43990142
 
  ENSEMBL   UCSC 
 
  194
 
  4
 
 26397
 
 gene;island
 
 0.67222
 
 0.88291
 
 Not queried
 
  0.9237373
 
       NA
 
       NA
  
 
  
 10
 
  57387511
 
  57387715
 
  ENSEMBL   UCSC 
 
  205
 
  4
 
 5734
 
 island
 
 0.01264
 
 0.03605
 
 Not queried
 
  0.9235805
 
       NA
 
       NA
  
 
  
 6
 
  32807230
 
  32807551
 
  ENSEMBL   UCSC 
 
  322
 
 10
 
 34812
 
 tss;island
 
 0.91710
 
 0.81104
 
 Not queried
 
  0.9234895
 
       NA
 
       NA
  
 
  
 2
 
    466712
 
    467135
 
  ENSEMBL   UCSC 
 
  424
 
  8
 
 23964
 
 island
 
 0.88986
 
 0.93878
 
 Not queried
 
  0.9234329
 
       NA
 
       NA
  
 
  
 1
 
 102462445
 
 102462682
 
  ENSEMBL   UCSC 
 
  238
 
  5
 
 655
 
 gene
 
 0.02692
 
 0.05038
 
 Not queried
 
  0.9234113
 
       NA
 
       NA
  
 
  
 1
 
 112531802
 
 112532085
 
  ENSEMBL   UCSC 
 
  284
 
  7
 
 1828
 
 tss
 
 0.02602
 
 0.05752
 
 Not queried
 
  0.9230810
 
       NA
 
       NA
  
 
  
 1
 
 112531263
 
 112532085
 
  ENSEMBL   UCSC 
 
  823
 
 11
 
 3590
 
 island
 
 0.10152
 
 0.13713
 
 Not queried
 
  0.9230810
 
       NA
 
       NA
  
 
  
 10
 
  71333152
 
  71333216
 
  ENSEMBL   UCSC 
 
   65
 
  5
 
 5109
 
 tss
 
 0.23568
 
 0.10489
 
 Not queried
 
  0.9227721
 
       NA
 
       NA
  
 
  
 17
 
  39743164
 
  39743427
 
  ENSEMBL   UCSC 
 
  264
 
  4
 
 17308
 
 tss
 
 0.74773
 
 0.68317
 
 Not queried
 
  0.9227677
 
       NA
 
       NA
  
 
  
 16
 
   1464660
 
   1465116
 
  ENSEMBL   UCSC 
 
  457
 
  7
 
 15510
 
 island
 
 0.10173
 
 0.17673
 
 Not queried
 
  0.9225362
 
       NA
 
       NA
  
 
  
 17
 
  73150870
 
  73151149
 
  ENSEMBL   UCSC 
 
  280
 
  5
 
 17612
 
 tss
 
 0.02285
 
 0.02638
 
 Not queried
 
  0.9224269
 
       NA
 
       NA
  
 
  
 14
 
 105511982
 
 105512306
 
  ENSEMBL   UCSC 
 
  325
 
  5
 
 13199
 
 island
 
 0.01528
 
 0.03297
 
 Not queried
 
  0.9222899
 
       NA
 
       NA
  
 
  
 1
 
  17751272
 
  17752023
 
  ENSEMBL   UCSC 
 
  752
 
  9
 
 266
 
 gene
 
 0.87314
 
 0.89862
 
 Not queried
 
  0.9219911
 
       NA
 
       NA
  
 
  
 17
 
  40265577
 
  40265827
 
  ENSEMBL   UCSC 
 
  251
 
  5
 
 16541
 
 gene;tss;island
 
 0.92947
 
 0.95178
 
 Not queried
 
  0.9219188
 
       NA
 
       NA
  
 
  
 13
 
  21635665
 
  21635991
 
  ENSEMBL   UCSC 
 
  327
 
  9
 
 11554
 
 island
 
 0.01860
 
 0.03157
 
 Not queried
 
  0.9215733
 
       NA
 
       NA
  
 
  
 6
 
 170400942
 
 170401305
 
  ENSEMBL   UCSC 
 
  364
 
  4
 
 36616
 
 island
 
 0.52888
 
 0.65238
 
 Not queried
 
  0.9213838
 
       NA
 
       NA
  
 
  
 17
 
  76989451
 
  76989965
 
  ENSEMBL   UCSC 
 
  515
 
  6
 
 16795
 
 gene;island
 
 0.88023
 
 0.94664
 
 Not queried
 
  0.9213386
 
       NA
 
       NA
  
 
  
 6
 
  83073619
 
  83073954
 
  ENSEMBL   UCSC 
 
  336
 
  7
 
 35026
 
 tss
 
 0.02225
 
 0.05104
 
 Not queried
 
  0.9212160
 
       NA
 
       NA
  
 
  
 6
 
  33395936
 
  33396407
 
  ENSEMBL   UCSC 
 
  472
 
 15
 
 34223
 
 gene;island
 
 0.28854
 
 0.17688
 
 Not queried
 
  0.9211759
 
       NA
 
       NA
  
 
  
 16
 
    928778
 
    929101
 
  ENSEMBL   UCSC 
 
  324
 
  4
 
 14520
 
 gene;island
 
 0.74830
 
 0.90735
 
 Not queried
 
  0.9211340
 
       NA
 
       NA
  
 
  
 15
 
  64648470
 
  64648652
 
  ENSEMBL   UCSC 
 
  183
 
  7
 
 13701
 
 tss
 
 0.01121
 
 0.01926
 
 Not queried
 
  0.9210105
 
       NA
 
       NA
  
 
  
 11
 
   2011216
 
   2011346
 
  ENSEMBL   UCSC 
 
  131
 
  6
 
 7032
 
 tss;island
 
 0.78694
 
 0.82642
 
 Not queried
 
  0.9208373
 
       NA
 
       NA
  
 
  
 7
 
  95064397
 
  95064474
 
  ENSEMBL   UCSC 
 
   78
 
  5
 
 37662
 
 tss
 
 0.02351
 
 0.07183
 
 Not queried
 
  0.9207759
 
       NA
 
       NA
  
 
  
 10
 
    466232
 
    466395
 
  ENSEMBL   UCSC 
 
  164
 
  4
 
 4425
 
 gene;island
 
 0.94102
 
 0.87269
 
 Not queried
 
  0.9207693
 
       NA
 
       NA
  
 
  
 1
 
  26324332
 
  26324909
 
  ENSEMBL   UCSC 
 
  578
 
  9
 
 2878
 
 island
 
 0.00881
 
 0.01169
 
 Not queried
 
  0.9207547
 
       NA
 
       NA
  
 
  
 2
 
 232063073
 
 232063399
 
  ENSEMBL   UCSC 
 
  327
 
  8
 
 25141
 
 island
 
 0.01642
 
 0.02663
 
 Not queried
 
  0.9207382
 
       NA
 
       NA
  
 
  
 7
 
 158800869
 
 158800986
 
  ENSEMBL   UCSC 
 
  118
 
  4
 
 37961
 
 tss;island
 
 0.86271
 
 0.92788
 
 Not queried
 
  0.9207303
 
       NA
 
       NA
  
 
  
 11
 
  76380921
 
  76381236
 
  ENSEMBL   UCSC 
 
  316
 
  8
 
 6819
 
 gene;island
 
 0.30834
 
 0.43610
 
 Not queried
 
  0.9207166
 
       NA
 
       NA
  
 
  
 8
 
   2046752
 
   2047010
 
  ENSEMBL   UCSC 
 
  259
 
  4
 
 39125
 
 gene;island
 
 0.85634
 
 0.90221
 
 Not queried
 
  0.9206497
 
       NA
 
       NA
  
 
  
 1
 
 153606037
 
 153606480
 
  ENSEMBL   UCSC 
 
  444
 
  6
 
 1942
 
 tss
 
 0.03728
 
 0.03998
 
 Not queried
 
  0.9203162
 
       NA
 
       NA
  
 
  
 6
 
 143832933
 
 143833251
 
  ENSEMBL   UCSC 
 
  319
 
  7
 
 35202
 
 tss
 
 0.09553
 
 0.05286
 
 Not queried
 
  0.9201921
 
       NA
 
       NA
  
 
  
 1
 
 182573453
 
 182573989
 
  ENSEMBL   UCSC 
 
  537
 
 10
 
 4010
 
 island
 
 0.09865
 
 0.05785
 
 Not queried
 
  0.9200617
 
       NA
 
       NA
  
 
  
 12
 
  48099724
 
  48099889
 
  ENSEMBL   UCSC 
 
  166
 
  4
 
 10326
 
 island
 
 0.02799
 
 0.03596
 
 Not queried
 
  0.9194913
 
       NA
 
       NA
  
 
  
 2
 
 237080267
 
 237080535
 
  ENSEMBL   UCSC 
 
  269
 
  4
 
 25188
 
 island
 
 0.07336
 
 0.03826
 
 Not queried
 
  0.9193505
 
       NA
 
       NA
  
 
  
 17
 
  37764118
 
  37764293
 
  ENSEMBL   UCSC 
 
  176
 
  7
 
 18246
 
 island
 
 0.23441
 
 0.38899
 
 Not queried
 
  0.9192858
 
       NA
 
       NA
  
 
  
 13
 
  43148684
 
  43149281
 
  ENSEMBL   UCSC 
 
  598
 
  7
 
 11119
 
 gene;island
 
 0.03006
 
 0.05747
 
 Not queried
 
  0.9192761
 
       NA
 
       NA
  
 
  
 19
 
  51523550
 
  51523933
 
  ENSEMBL   UCSC 
 
  384
 
  5
 
 20954
 
 tss;island
 
 0.58601
 
 0.37219
 
 Not queried
 
  0.9192725
 
       NA
 
       NA
  
 
  
 8
 
 143535437
 
 143535891
 
  ENSEMBL   UCSC 
 
  455
 
  5
 
 40569
 
 island
 
 0.81543
 
 0.89254
 
 Not queried
 
  0.9192141
 
       NA
 
       NA
  
 
  
 6
 
 122793073
 
 122793338
 
  ENSEMBL   UCSC 
 
  266
 
  5
 
 34397
 
 gene
 
 0.00704
 
 0.00713
 
 Not queried
 
  0.9192020
 
       NA
 
       NA
  
 
  
 11
 
  67275985
 
  67276453
 
  ENSEMBL   UCSC 
 
  469
 
 10
 
 8532
 
 island
 
 0.01541
 
 0.02618
 
 Not queried
 
  0.9190770
 
       NA
 
       NA
  
 
  
 17
 
  77070793
 
  77070844
 
  ENSEMBL   UCSC 
 
   52
 
  4
 
 17680
 
 tss
 
 0.11149
 
 0.18921
 
 Not queried
 
  0.9189112
 
       NA
 
       NA
  
 
  
 3
 
 176915592
 
 176915853
 
  ENSEMBL   UCSC 
 
  262
 
  4
 
 28682
 
 tss;island
 
 0.02778
 
 0.03423
 
 Not queried
 
  0.9188058
 
       NA
 
       NA
  
 
  
 20
 
  37434158
 
  37434262
 
  ENSEMBL   UCSC 
 
  105
 
  6
 
 25701
 
 tss
 
 0.02617
 
 0.02815
 
 Not queried
 
  0.9187846
 
       NA
 
       NA
  
 
  
 17
 
  47547353
 
  47547767
 
  ENSEMBL   UCSC 
 
  415
 
  5
 
 18489
 
 island
 
 0.75619
 
 0.62921
 
 Not queried
 
  0.9186533
 
       NA
 
       NA
  
 
  
 6
 
  32150404
 
  32150739
 
  ENSEMBL   UCSC 
 
  336
 
  6
 
 34014
 
 gene
 
 0.69670
 
 0.83171
 
 Not queried
 
  0.9186344
 
       NA
 
       NA
  
 
  
 6
 
 111136481
 
 111136640
 
  ENSEMBL   UCSC 
 
  160
 
  4
 
 35107
 
 tss
 
 0.01915
 
 0.01204
 
 Not queried
 
  0.9186292
 
       NA
 
       NA
  
 
  
 13
 
  20710941
 
  20711179
 
  ENSEMBL   UCSC 
 
  239
 
  4
 
 11538
 
 island
 
 0.01331
 
 0.02003
 
 Not queried
 
  0.9185567
 
       NA
 
       NA
  
 
  
 7
 
  19157634
 
  19158747
 
  ENSEMBL   UCSC 
 
 1114
 
 22
 
 37395
 
 tss;island
 
 0.10024
 
 0.11730
 
 Not queried
 
  0.9183723
 
       NA
 
       NA
  
 
  
 5
 
  76011127
 
  76011428
 
  ENSEMBL   UCSC 
 
  302
 
  5
 
 32076
 
 tss;island
 
 0.04212
 
 0.03274
 
 Not queried
 
  0.9181931
 
       NA
 
       NA
  
 
  
 19
 
  58400494
 
  58400574
 
  ENSEMBL   UCSC 
 
   81
 
  4
 
 21090
 
 tss
 
 0.03413
 
 0.01695
 
 Not queried
 
  0.9181600
 
       NA
 
       NA
  
 
  
 14
 
 105044984
 
 105045347
 
  ENSEMBL   UCSC 
 
  364
 
  4
 
 12630
 
 tss;island
 
 0.59072
 
 0.72992
 
 Not queried
 
  0.9179234
 
       NA
 
       NA
  
 
  
 7
 
  18126902
 
  18127112
 
  ENSEMBL   UCSC 
 
  211
 
  4
 
 38216
 
 island
 
 0.04864
 
 0.10070
 
 Not queried
 
  0.9177999
 
       NA
 
       NA
  
 
  
 6
 
  31124408
 
  31124589
 
  ENSEMBL   UCSC 
 
  182
 
  5
 
 33794
 
 gene;island
 
 0.87281
 
 0.91891
 
 Not queried
 
  0.9177815
 
       NA
 
       NA
  
 
  
 X
 
 100183936
 
 100184041
 
  ENSEMBL   UCSC 
 
  106
 
  4
 
 41419
 
 tss
 
 0.01698
 
 0.02725
 
 Not queried
 
  0.9168827
 
       NA
 
       NA
  
 
  
 X
 
 100183764
 
 100184041
 
  ENSEMBL   UCSC 
 
  278
 
  6
 
 41905
 
 island
 
 0.01950
 
 0.02725
 
 Not queried
 
  0.9168827
 
       NA
 
       NA
  
 
  
 6
 
  28249142
 
  28249704
 
  ENSEMBL   UCSC 
 
  563
 
 10
 
 35524
 
 island
 
 0.02708
 
 0.03802
 
 Not queried
 
  0.9167930
 
       NA
 
       NA
  
 
  
 17
 
  77133977
 
  77134303
 
  ENSEMBL   UCSC 
 
  327
 
  4
 
 16801
 
 gene;island
 
 0.67048
 
 0.85707
 
 Not queried
 
  0.9167145
 
       NA
 
       NA
  
 
  
 6
 
  28828946
 
  28830310
 
  ENSEMBL   UCSC 
 
 1365
 
 34
 
 35535
 
 island
 
 0.87494
 
 0.70398
 
 Not queried
 
  0.9166955
 
       NA
 
       NA
  
 
  
 22
 
  50608033
 
  50608598
 
  ENSEMBL   UCSC 
 
  566
 
  6
 
 27206
 
 tss;island
 
 0.05694
 
 0.03772
 
 Not queried
 
  0.9166890
 
       NA
 
       NA
  
 
  
 6
 
  31702632
 
  31702915
 
  ENSEMBL   UCSC 
 
  284
 
  5
 
 33885
 
 gene
 
 0.05662
 
 0.09759
 
 Not queried
 
  0.9164295
 
       NA
 
       NA
  
 
  
 X
 
  70316045
 
  70316444
 
  ENSEMBL   UCSC 
 
  400
 
  4
 
 41020
 
 gene;island
 
 0.09930
 
 0.04086
 
 Not queried
 
  0.9163085
 
       NA
 
       NA
  
 
  
 16
 
  27280150
 
  27280434
 
  ENSEMBL   UCSC 
 
  285
 
  8
 
 15119
 
 tss
 
 0.01732
 
 0.02465
 
 Not queried
 
  0.9160503
 
       NA
 
       NA
  
 
  
 19
 
  16178091
 
  16178253
 
  ENSEMBL   UCSC 
 
  163
 
  6
 
 20530
 
 tss
 
 0.90052
 
 0.92149
 
 Not queried
 
  0.9159532
 
       NA
 
       NA
  
 
  
 14
 
 100150602
 
 100150802
 
  ENSEMBL   UCSC 
 
  201
 
  6
 
 13100
 
 island
 
 0.04390
 
 0.06151
 
 Not queried
 
  0.9158984
 
       NA
 
       NA
  
 
  
 7
 
  25897148
 
  25897907
 
  ENSEMBL   UCSC 
 
  760
 
  8
 
 38260
 
 island
 
 0.03175
 
 0.06831
 
 Not queried
 
  0.9156397
 
       NA
 
       NA
  
 
  
 16
 
   2198777
 
   2199120
 
  ENSEMBL   UCSC 
 
  344
 
  4
 
 14572
 
 gene;island
 
 0.08131
 
 0.04591
 
 Not queried
 
  0.9154684
 
       NA
 
       NA
  
 
  
 22
 
  24998974
 
  24999253
 
  ENSEMBL   UCSC 
 
  280
 
  6
 
 26774
 
 gene
 
 0.30894
 
 0.45194
 
 Not queried
 
  0.9153722
 
       NA
 
       NA
  
 
  
 11
 
  20620680
 
  20621109
 
  ENSEMBL   UCSC 
 
  430
 
  7
 
 8109
 
 island
 
 0.69890
 
 0.48175
 
 Not queried
 
  0.9153584
 
       NA
 
       NA
  
 
  
 10
 
  95462161
 
  95462325
 
  ENSEMBL   UCSC 
 
  165
 
  5
 
 4669
 
 gene
 
 0.01759
 
 0.03266
 
 Not queried
 
  0.9150683
 
       NA
 
       NA
  
 
  
 16
 
  31475839
 
  31476161
 
  ENSEMBL   UCSC 
 
  323
 
  5
 
 14708
 
 gene;island
 
 0.76721
 
 0.86372
 
 Not queried
 
  0.9150610
 
       NA
 
       NA
  
 
  
 6
 
 105404769
 
 105405068
 
  ENSEMBL   UCSC 
 
  300
 
  4
 
 36312
 
 island
 
 0.03280
 
 0.01395
 
 Not queried
 
  0.9147525
 
       NA
 
       NA
  
 
  
 X
 
 117250943
 
 117251123
 
  ENSEMBL   UCSC 
 
  181
 
  6
 
 41479
 
 tss
 
 0.03135
 
 0.04782
 
 Not queried
 
  0.9147148
 
       NA
 
       NA
  
 
  
 6
 
  43603252
 
  43603546
 
  ENSEMBL   UCSC 
 
  295
 
  5
 
 34939
 
 tss
 
 0.05693
 
 0.06909
 
 Not queried
 
  0.9146935
 
       NA
 
       NA
  
 
  
 X
 
  31089692
 
  31090380
 
  ENSEMBL   UCSC 
 
  689
 
 10
 
 41702
 
 island
 
 0.80158
 
 0.90632
 
 Not queried
 
  0.9144720
 
       NA
 
       NA
  
 
  
 Y
 
  16634027
 
  16634382
 
  ENSEMBL   UCSC 
 
  356
 
  5
 
 42169
 
 tss;island
 
 0.43550
 
 0.23003
 
 Not queried
 
  0.9144636
 
       NA
 
       NA
  
 
  
 19
 
   3870806
 
   3871035
 
  ENSEMBL   UCSC 
 
  230
 
  4
 
 21315
 
 island
 
 0.32670
 
 0.52148
 
 Not queried
 
  0.9144614
 
       NA
 
       NA
  
 
  
 7
 
  42275813
 
  42276004
 
  ENSEMBL   UCSC 
 
  192
 
  4
 
 36914
 
 gene;island
 
 0.03960
 
 0.01308
 
 Not queried
 
  0.9143546
 
       NA
 
       NA
  
 
  
 7
 
  96654425
 
  96654572
 
  ENSEMBL   UCSC 
 
  148
 
  4
 
 37671
 
 tss;island
 
 0.32805
 
 0.20575
 
 Not queried
 
  0.9142490
 
       NA
 
       NA
  
 
  
 3
 
 179754521
 
 179754761
 
  ENSEMBL   UCSC 
 
  241
 
  7
 
 28691
 
 tss
 
 0.03791
 
 0.06801
 
 Not queried
 
  0.9142212
 
       NA
 
       NA
  
 
  
 1
 
  34098656
 
  34099118
 
  ENSEMBL   UCSC 
 
  463
 
  4
 
 405
 
 gene;island
 
 0.01663
 
 0.03128
 
 Not queried
 
  0.9141639
 
       NA
 
       NA
  
 
  
 10
 
  30637833
 
  30638134
 
  ENSEMBL   UCSC 
 
  302
 
  4
 
 4536
 
 gene
 
 0.01164
 
 0.02675
 
 Not queried
 
  0.9141185
 
       NA
 
       NA
  
 
  
 12
 
 117468061
 
 117468529
 
  ENSEMBL   UCSC 
 
  469
 
  5
 
 9308
 
 gene
 
 0.82910
 
 0.93101
 
 Not queried
 
  0.9140679
 
       NA
 
       NA
  
 
  
 4
 
 154073371
 
 154074259
 
  ENSEMBL   UCSC 
 
  889
 
 14
 
 30591
 
 tss
 
 0.01138
 
 0.03679
 
 Not queried
 
  0.9139805
 
       NA
 
       NA
  
 
  
 10
 
  28032282
 
  28032855
 
  ENSEMBL   UCSC 
 
  574
 
  7
 
 4530
 
 gene;island
 
 0.01270
 
 0.02037
 
 Not queried
 
  0.9139581
 
       NA
 
       NA
  
 
  
 16
 
    875257
 
    875626
 
  ENSEMBL   UCSC 
 
  370
 
  4
 
 15460
 
 island
 
 0.84185
 
 0.90323
 
 Not queried
 
  0.9137690
 
       NA
 
       NA
  
 
  
 15
 
  40213270
 
  40213409
 
  ENSEMBL   UCSC 
 
  140
 
  6
 
 13550
 
 tss;island
 
 0.02040
 
 0.02861
 
 Not queried
 
  0.9136472
 
       NA
 
       NA
  
 
  
 6
 
  29815787
 
  29815902
 
  ENSEMBL   UCSC 
 
  116
 
  4
 
 35575
 
 island
 
 0.02696
 
 0.05420
 
 Not queried
 
  0.9136015
 
       NA
 
       NA
  
 
  
 1
 
 169863438
 
 169863532
 
  ENSEMBL   UCSC 
 
   95
 
  4
 
 2084
 
 tss;island
 
 0.02471
 
 0.03904
 
 Not queried
 
  0.9135475
 
       NA
 
       NA
  
 
  
 1
 
  24513865
 
  24514013
 
  ENSEMBL   UCSC 
 
  149
 
  4
 
 1352
 
 tss
 
 0.02364
 
 0.04010
 
 Not queried
 
  0.9135371
 
       NA
 
       NA
  
 
  
 1
 
  24513473
 
  24514013
 
  ENSEMBL   UCSC 
 
  541
 
  8
 
 2850
 
 island
 
 0.02364
 
 0.03839
 
 Not queried
 
  0.9135371
 
       NA
 
       NA
  
 
  
 8
 
 144328715
 
 144329279
 
  ENSEMBL   UCSC 
 
  565
 
 10
 
 40584
 
 island
 
 0.01562
 
 0.03618
 
 Not queried
 
  0.9134888
 
       NA
 
       NA
  
 
  
 12
 
   1738035
 
   1738446
 
  ENSEMBL   UCSC 
 
  412
 
  7
 
 8974
 
 gene;island
 
 0.28020
 
 0.22393
 
 Not queried
 
  0.9134354
 
       NA
 
       NA
  
 
  
 2
 
 158114069
 
 158114300
 
  ENSEMBL   UCSC 
 
  232
 
  4
 
 23638
 
 tss
 
 0.59488
 
 0.73727
 
 Not queried
 
  0.9133196
 
       NA
 
       NA
  
 
  
 1
 
  32930375
 
  32930544
 
  ENSEMBL   UCSC 
 
  170
 
  6
 
 1458
 
 tss;island
 
 0.01434
 
 0.01968
 
 Not queried
 
  0.9132787
 
       NA
 
       NA
  
 
  
 1
 
  28099796
 
  28100114
 
  ENSEMBL   UCSC 
 
  319
 
  5
 
 358
 
 gene;island
 
 0.02562
 
 0.04800
 
 Not queried
 
  0.9132742
 
       NA
 
       NA
  
 
  
 2
 
  26468086
 
  26468406
 
  ENSEMBL   UCSC 
 
  321
 
  5
 
 22572
 
 gene;tss;island
 
 0.01253
 
 0.02334
 
 Not queried
 
  0.9131714
 
       NA
 
       NA
  
 
  
 6
 
   2636241
 
   2636435
 
  ENSEMBL   UCSC 
 
  195
 
  4
 
 34524
 
 tss;island
 
 0.92206
 
 0.86804
 
 Not queried
 
  0.9130938
 
       NA
 
       NA
  
 
  
 12
 
 111855825
 
 111855929
 
  ENSEMBL   UCSC 
 
  105
 
  5
 
 9283
 
 gene;island
 
 0.83152
 
 0.85029
 
 Not queried
 
  0.9130746
 
       NA
 
       NA
  
 
  
 7
 
    211714
 
    212112
 
  ENSEMBL   UCSC 
 
  399
 
  4
 
 36642
 
 gene
 
 0.79813
 
 0.83025
 
 Not queried
 
  0.9128548
 
       NA
 
       NA
  
 
  
 6
 
 125623414
 
 125623573
 
  ENSEMBL   UCSC 
 
  160
 
  5
 
 35146
 
 tss;island
 
 0.88659
 
 0.92233
 
 Not queried
 
  0.9127692
 
       NA
 
       NA
  
 
  
 2
 
  62932705
 
  62933278
 
  ENSEMBL   UCSC 
 
  574
 
  8
 
 22664
 
 gene;tss;island
 
 0.03055
 
 0.06387
 
 Not queried
 
  0.9127196
 
       NA
 
       NA
  
 
  
 6
 
  32634245
 
  32634362
 
  ENSEMBL   UCSC 
 
  118
 
  5
 
 34061
 
 gene
 
 0.58974
 
 0.43080
 
 Not queried
 
  0.9126513
 
       NA
 
       NA
  
 
  
 12
 
  56727551
 
  56727976
 
  ENSEMBL   UCSC 
 
  426
 
 12
 
 9771
 
 tss
 
 0.02126
 
 0.03544
 
 Not queried
 
  0.9122765
 
       NA
 
       NA
  
 
  
 5
 
  89825117
 
  89825908
 
  ENSEMBL   UCSC 
 
  792
 
  9
 
 32891
 
 island
 
 0.01838
 
 0.02361
 
 Not queried
 
  0.9122388
 
       NA
 
       NA
  
 
  
 1
 
 205719204
 
 205719341
 
  ENSEMBL   UCSC 
 
  138
 
  4
 
 966
 
 gene
 
 0.01165
 
 0.03822
 
 Not queried
 
  0.9122320
 
       NA
 
       NA
  
 
  
 1
 
 146649635
 
 146649851
 
  ENSEMBL   UCSC 
 
  217
 
  4
 
 727
 
 gene;island
 
 0.94163
 
 0.89159
 
 Not queried
 
  0.9121063
 
       NA
 
       NA
  
 
  
 2
 
  68546899
 
  68547349
 
  ENSEMBL   UCSC 
 
  451
 
  7
 
 24325
 
 island
 
 0.05030
 
 0.09097
 
 Not queried
 
  0.9120931
 
       NA
 
       NA
  
 
  
 12
 
 111806472
 
 111806796
 
  ENSEMBL   UCSC 
 
  325
 
  4
 
 9282
 
 gene;island
 
 0.01244
 
 0.01973
 
 Not queried
 
  0.9120638
 
       NA
 
       NA
  
 
  
 14
 
 102695264
 
 102695875
 
  ENSEMBL   UCSC 
 
  612
 
  7
 
 12202
 
 gene;island
 
 0.87604
 
 0.92157
 
 Not queried
 
  0.9119043
 
       NA
 
       NA
  
 
  
 3
 
 181429233
 
 181429704
 
  ENSEMBL   UCSC 
 
  472
 
  9
 
 27999
 
 gene;tss;island
 
 0.01624
 
 0.02315
 
 Not queried
 
  0.9112753
 
       NA
 
       NA
  
 
  
 15
 
  79576060
 
  79576298
 
  ENSEMBL   UCSC 
 
  239
 
  4
 
 13419
 
 gene;island
 
 0.03453
 
 0.05344
 
 Not queried
 
  0.9112362
 
       NA
 
       NA
  
 
  
 19
 
  13049163
 
  13049634
 
  ENSEMBL   UCSC 
 
  472
 
  9
 
 21601
 
 island
 
 0.01847
 
 0.03119
 
 Not queried
 
  0.9112029
 
       NA
 
       NA
  
 
  
 6
 
  31698492
 
  31698899
 
  ENSEMBL   UCSC 
 
  408
 
 10
 
 33883
 
 gene;tss;island
 
 0.78412
 
 0.89440
 
 Not queried
 
  0.9111807
 
       NA
 
       NA
  
 
  
 8
 
  89339404
 
  89339819
 
  ENSEMBL   UCSC 
 
  416
 
  5
 
 40376
 
 island
 
 0.02028
 
 0.06262
 
 Not queried
 
  0.9111156
 
       NA
 
       NA
  
 
  
 19
 
  16629806
 
  16630285
 
  ENSEMBL   UCSC 
 
  480
 
  6
 
 19876
 
 gene;island
 
 0.86715
 
 0.91376
 
 Not queried
 
  0.9110724
 
       NA
 
       NA
  
 
  
 21
 
  45246060
 
  45246441
 
  ENSEMBL   UCSC 
 
  382
 
  6
 
 26684
 
 island
 
 0.73244
 
 0.82071
 
 Not queried
 
  0.9109467
 
       NA
 
       NA
  
 
  
 11
 
    798350
 
    798458
 
  ENSEMBL   UCSC 
 
  109
 
  4
 
 7859
 
 island
 
 0.03896
 
 0.03507
 
 Not queried
 
  0.9109415
 
       NA
 
       NA
  
 
  
 14
 
  90722653
 
  90722978
 
  ENSEMBL   UCSC 
 
  326
 
  6
 
 13043
 
 island
 
 0.04468
 
 0.05653
 
 Not queried
 
  0.9108772
 
       NA
 
       NA
  
 
  
 12
 
  14132940
 
  14133426
 
  ENSEMBL   UCSC 
 
  487
 
  7
 
 10236
 
 island
 
 0.06101
 
 0.21005
 
 Not queried
 
  0.9108124
 
       NA
 
       NA
  
 
  
 1
 
  31885655
 
  31885890
 
  ENSEMBL   UCSC 
 
  236
 
  8
 
 1444
 
 tss;island
 
 0.06646
 
 0.15860
 
 Not queried
 
  0.9107162
 
       NA
 
       NA
  
 
  
 1
 
 168194826
 
 168195108
 
  ENSEMBL   UCSC 
 
  283
 
  9
 
 2076
 
 tss;island
 
 0.01825
 
 0.03558
 
 Not queried
 
  0.9104314
 
       NA
 
       NA
  
 
  
 17
 
  25620944
 
  25621539
 
  ENSEMBL   UCSC 
 
  596
 
 10
 
 18100
 
 island
 
 0.02069
 
 0.03528
 
 Not queried
 
  0.9103556
 
       NA
 
       NA
  
 
  
 7
 
  96632294
 
  96632841
 
  ENSEMBL   UCSC 
 
  548
 
  5
 
 37038
 
 gene;island
 
 0.08860
 
 0.05019
 
 Not queried
 
  0.9102940
 
       NA
 
       NA
  
 
  
 7
 
   6198496
 
   6198846
 
  ENSEMBL   UCSC 
 
  351
 
  4
 
 36823
 
 gene;island
 
 0.71823
 
 0.88882
 
 Not queried
 
  0.9102591
 
       NA
 
       NA
  
 
  
 14
 
  24837072
 
  24837184
 
  ENSEMBL   UCSC 
 
  113
 
  4
 
 12020
 
 gene;tss;island
 
 0.02000
 
 0.02729
 
 Not queried
 
  0.9100566
 
       NA
 
       NA
  
 
  
 13
 
  39358777
 
  39358893
 
  ENSEMBL   UCSC 
 
  117
 
  4
 
 11112
 
 gene
 
 0.91352
 
 0.80869
 
 Not queried
 
  0.9099671
 
       NA
 
       NA
  
 
  
 8
 
 139926236
 
 139926686
 
  ENSEMBL   UCSC 
 
  451
 
  5
 
 39858
 
 tss
 
 0.04931
 
 0.08880
 
 Not queried
 
  0.9099163
 
       NA
 
       NA
  
 
  
 20
 
  57582581
 
  57583709
 
  ENSEMBL   UCSC 
 
 1129
 
 22
 
 25817
 
 tss;island
 
 0.91361
 
 0.80290
 
 Not queried
 
  0.9095843
 
       NA
 
       NA
  
 
  
 10
 
 130012874
 
 130013154
 
  ENSEMBL   UCSC 
 
  281
 
  6
 
 6235
 
 island
 
 0.92129
 
 0.93191
 
 Not queried
 
  0.9095387
 
       NA
 
       NA
  
 
  
 6
 
 169629684
 
 169630013
 
  ENSEMBL   UCSC 
 
  330
 
  7
 
 34501
 
 gene;island
 
 0.80538
 
 0.88563
 
 Not queried
 
  0.9093519
 
       NA
 
       NA
  
 
  
 19
 
  19971578
 
  19972043
 
  ENSEMBL   UCSC 
 
  466
 
  4
 
 21794
 
 island
 
 0.62509
 
 0.53777
 
 Not queried
 
  0.9092901
 
       NA
 
       NA
  
 
  
 11
 
  70507825
 
  70508659
 
  ENSEMBL   UCSC 
 
  835
 
 11
 
 6775
 
 gene;island
 
 0.06394
 
 0.09929
 
 Not queried
 
  0.9092507
 
       NA
 
       NA
  
 
  
 11
 
 117747689
 
 117748236
 
  ENSEMBL   UCSC 
 
  548
 
 10
 
 8828
 
 island
 
 0.37866
 
 0.07681
 
 Not queried
 
  0.9092378
 
       NA
 
       NA
  
 
  
 2
 
 240168836
 
 240169280
 
  ENSEMBL   UCSC 
 
  445
 
  6
 
 23074
 
 gene
 
 0.82078
 
 0.86846
 
 Not queried
 
  0.9090817
 
       NA
 
       NA
  
 
  
 1
 
  60539362
 
  60539675
 
  ENSEMBL   UCSC 
 
  314
 
  6
 
 3320
 
 island
 
 0.02253
 
 0.03870
 
 Not queried
 
  0.9090493
 
       NA
 
       NA
  
 
  
 22
 
  20067313
 
  20067491
 
  ENSEMBL   UCSC 
 
  179
 
  4
 
 26932
 
 tss;island
 
 0.01986
 
 0.03212
 
 Not queried
 
  0.9089909
 
       NA
 
       NA
  
 
  
 13
 
  99174341
 
  99174609
 
  ENSEMBL   UCSC 
 
  269
 
  5
 
 11481
 
 tss
 
 0.85701
 
 0.89562
 
 Not queried
 
  0.9087516
 
       NA
 
       NA
  
 
  
 7
 
 158061615
 
 158061820
 
  ENSEMBL   UCSC 
 
  206
 
  4
 
 37300
 
 gene;island
 
 0.87189
 
 0.78816
 
 Not queried
 
  0.9087221
 
       NA
 
       NA
  
 
  
 5
 
  74349644
 
  74350214
 
  ENSEMBL   UCSC 
 
  571
 
  5
 
 32819
 
 island
 
 0.16827
 
 0.09030
 
 Not queried
 
  0.9082804
 
       NA
 
       NA
  
 
  
 19
 
  18385409
 
  18385724
 
  ENSEMBL   UCSC 
 
  316
 
  4
 
 20579
 
 tss
 
 0.83088
 
 0.87259
 
 Not queried
 
  0.9082419
 
       NA
 
       NA
  
 
  
 19
 
    409357
 
    409679
 
  ENSEMBL   UCSC 
 
  323
 
  4
 
 20234
 
 tss;island
 
 0.03609
 
 0.06453
 
 Not queried
 
  0.9082384
 
       NA
 
       NA
  
 
  
 2
 
 223169352
 
 223169609
 
  ENSEMBL   UCSC 
 
  258
 
  4
 
 23015
 
 gene;island
 
 0.34526
 
 0.17283
 
 Not queried
 
  0.9082217
 
       NA
 
       NA
  
 
  
 21
 
  40759534
 
  40759694
 
  ENSEMBL   UCSC 
 
  161
 
  5
 
 26387
 
 gene;island
 
 0.03966
 
 0.02122
 
 Not queried
 
  0.9081879
 
       NA
 
       NA
  
 
  
 1
 
 101361710
 
 101362002
 
  ENSEMBL   UCSC 
 
  293
 
  4
 
 652
 
 gene;island
 
 0.00653
 
 0.01248
 
 Not queried
 
  0.9081732
 
       NA
 
       NA
  
 
  
 4
 
 140477492
 
 140477727
 
  ENSEMBL   UCSC 
 
  236
 
  6
 
 31265
 
 island
 
 0.01177
 
 0.02372
 
 Not queried
 
  0.9081693
 
       NA
 
       NA
  
 
  
 19
 
  56347722
 
  56347927
 
  ENSEMBL   UCSC 
 
  206
 
  5
 
 20196
 
 gene;tss
 
 0.84250
 
 0.74030
 
 Not queried
 
  0.9080874
 
       NA
 
       NA
  
 
  
 1
 
 109505929
 
 109506060
 
  ENSEMBL   UCSC 
 
  132
 
  4
 
 660
 
 gene
 
 0.02100
 
 0.03256
 
 Not queried
 
  0.9080086
 
       NA
 
       NA
  
 
  
 1
 
   6479628
 
   6479813
 
  ENSEMBL   UCSC 
 
  186
 
  4
 
 170
 
 gene;island
 
 0.04108
 
 0.06047
 
 Not queried
 
  0.9079717
 
       NA
 
       NA
  
 
  
 2
 
 120980838
 
 120981005
 
  ENSEMBL   UCSC 
 
  168
 
  4
 
 24624
 
 island
 
 0.01685
 
 0.03810
 
 Not queried
 
  0.9079168
 
       NA
 
       NA
  
 
  
 18
 
   2654819
 
   2655213
 
  ENSEMBL   UCSC 
 
  395
 
  6
 
 19125
 
 tss;island
 
 0.01489
 
 0.01921
 
 Not queried
 
  0.9077667
 
       NA
 
       NA
  
 
  
 10
 
  23983496
 
  23983538
 
  ENSEMBL   UCSC 
 
   43
 
  4
 
 4972
 
 tss
 
 0.05956
 
 0.03331
 
 Not queried
 
  0.9077286
 
       NA
 
       NA
  
 
  
 12
 
  49182837
 
  49183212
 
  ENSEMBL   UCSC 
 
  376
 
  7
 
 9648
 
 tss;island
 
 0.03938
 
 0.05138
 
 Not queried
 
  0.9077244
 
       NA
 
       NA
  
 
  
 8
 
  73163524
 
  73164024
 
  ENSEMBL   UCSC 
 
  501
 
  5
 
 40321
 
 island
 
 0.03220
 
 0.04333
 
 Not queried
 
  0.9076358
 
       NA
 
       NA
  
 
  
 19
 
  46996347
 
  46996868
 
  ENSEMBL   UCSC 
 
  522
 
  4
 
 20074
 
 gene;island
 
 0.72888
 
 0.66406
 
 Not queried
 
  0.9074661
 
       NA
 
       NA
  
 
  
 13
 
  22178312
 
  22178600
 
  ENSEMBL   UCSC 
 
  289
 
  5
 
 11310
 
 tss;island
 
 0.01474
 
 0.03112
 
 Not queried
 
  0.9072633
 
       NA
 
       NA
  
 
  
 X
 
 135849691
 
 135850058
 
  ENSEMBL   UCSC 
 
  368
 
  4
 
 41121
 
 gene;island
 
 0.06075
 
 0.04121
 
 Not queried
 
  0.9071652
 
       NA
 
       NA
  
 
  
 1
 
 201476290
 
 201476960
 
  ENSEMBL   UCSC 
 
  671
 
 11
 
 4064
 
 island
 
 0.68244
 
 0.74498
 
 Not queried
 
  0.9069132
 
       NA
 
       NA
  
 
  
 3
 
 197025853
 
 197026407
 
  ENSEMBL   UCSC 
 
  555
 
  8
 
 28777
 
 tss;island
 
 0.06134
 
 0.02183
 
 Not queried
 
  0.9067903
 
       NA
 
       NA
  
 
  
 11
 
  66444632
 
  66444806
 
  ENSEMBL   UCSC 
 
  175
 
  4
 
 6717
 
 gene;island
 
 0.02045
 
 0.02300
 
 Not queried
 
  0.9067853
 
       NA
 
       NA
  
 
  
 3
 
 138647715
 
 138648052
 
  ENSEMBL   UCSC 
 
  338
 
  4
 
 29483
 
 island
 
 0.95975
 
 0.90423
 
 Not queried
 
  0.9067468
 
       NA
 
       NA
  
 
  
 7
 
   2314707
 
   2314947
 
  ENSEMBL   UCSC 
 
  241
 
  4
 
 36735
 
 gene;island
 
 0.79439
 
 0.89368
 
 Not queried
 
  0.9067263
 
       NA
 
       NA
  
 
  
 2
 
 171785479
 
 171785547
 
  ENSEMBL   UCSC 
 
   69
 
  4
 
 23673
 
 tss;island
 
 0.02116
 
 0.03451
 
 Not queried
 
  0.9066879
 
       NA
 
       NA
  
 
  
 19
 
  55574619
 
  55574733
 
  ENSEMBL   UCSC 
 
  115
 
  6
 
 21033
 
 tss
 
 0.02458
 
 0.03558
 
 Not queried
 
  0.9064401
 
       NA
 
       NA
  
 
  
 2
 
  42396143
 
  42396458
 
  ENSEMBL   UCSC 
 
  316
 
  8
 
 23281
 
 tss;island
 
 0.02952
 
 0.07625
 
 Not queried
 
  0.9063771
 
       NA
 
       NA
  
 
  
 9
 
 139743586
 
 139743802
 
  ENSEMBL   UCSC 
 
  217
 
  4
 
 40730
 
 gene;island
 
 0.01737
 
 0.02857
 
 Not queried
 
  0.9062034
 
       NA
 
       NA
  
 
  
 7
 
   2026965
 
   2027283
 
  ENSEMBL   UCSC 
 
  319
 
  4
 
 36719
 
 gene;island
 
 0.77372
 
 0.83724
 
 Not queried
 
  0.9059552
 
       NA
 
       NA
  
 
  
 18
 
  32957329
 
  32957683
 
  ENSEMBL   UCSC 
 
  355
 
  8
 
 19204
 
 tss;island
 
 0.05839
 
 0.06568
 
 Not queried
 
  0.9058874
 
       NA
 
       NA
  
 
  
 3
 
 153838788
 
 153839142
 
  ENSEMBL   UCSC 
 
  355
 
  7
 
 28624
 
 tss
 
 0.01325
 
 0.02297
 
 Not queried
 
  0.9058645
 
       NA
 
       NA
  
 
  
 8
 
  77618058
 
  77618401
 
  ENSEMBL   UCSC 
 
  344
 
  6
 
 39281
 
 gene
 
 0.61248
 
 0.76734
 
 Not queried
 
  0.9053572
 
       NA
 
       NA
  
 
  
 17
 
  35289351
 
  35289875
 
  ENSEMBL   UCSC 
 
  525
 
  5
 
 18201
 
 island
 
 0.51603
 
 0.45923
 
 Not queried
 
  0.9052943
 
       NA
 
       NA
  
 
  
 8
 
 144990141
 
 144990644
 
  ENSEMBL   UCSC 
 
  504
 
  4
 
 39433
 
 gene;island
 
 0.77758
 
 0.89750
 
 Not queried
 
  0.9052691
 
       NA
 
       NA
  
 
  
 6
 
  42927940
 
  42928497
 
  ENSEMBL   UCSC 
 
  558
 
 22
 
 34921
 
 tss
 
 0.02574
 
 0.04496
 
 Not queried
 
  0.9052292
 
       NA
 
       NA
  
 
  
 2
 
 203499669
 
 203499802
 
  ENSEMBL   UCSC 
 
  134
 
  4
 
 23788
 
 tss;island
 
 0.03542
 
 0.05359
 
 Not queried
 
  0.9052248
 
       NA
 
       NA
  
 
  
 2
 
   3327027
 
   3327336
 
  ENSEMBL   UCSC 
 
  310
 
  4
 
 22510
 
 gene
 
 0.79567
 
 0.89626
 
 Not queried
 
  0.9051615
 
       NA
 
       NA
  
 
  
 3
 
 138763670
 
 138764193
 
  ENSEMBL   UCSC 
 
  524
 
  8
 
 29491
 
 island
 
 0.95146
 
 0.89922
 
 Not queried
 
  0.9051578
 
       NA
 
       NA
  
 
  
 6
 
 163149167
 
 163149453
 
  ENSEMBL   UCSC 
 
  287
 
  4
 
 34475
 
 gene;tss;island
 
 0.17641
 
 0.06781
 
 Not queried
 
  0.9051485
 
       NA
 
       NA
  
 
  
 8
 
  11561283
 
  11561620
 
  ENSEMBL   UCSC 
 
  338
 
  9
 
 39490
 
 tss
 
 0.19088
 
 0.08707
 
 Not queried
 
  0.9050554
 
       NA
 
       NA
  
 
  
 15
 
  74422275
 
  74423077
 
  ENSEMBL   UCSC 
 
  803
 
 14
 
 13388
 
 gene;tss;island
 
 0.03411
 
 0.08302
 
 Not queried
 
  0.9049960
 
       NA
 
       NA
  
 
  
 4
 
   7512016
 
   7512498
 
  ENSEMBL   UCSC 
 
  483
 
  5
 
 29893
 
 gene;island
 
 0.90153
 
 0.90689
 
 Not queried
 
  0.9048427
 
       NA
 
       NA
  
 
  
 11
 
  65101065
 
  65101167
 
  ENSEMBL   UCSC 
 
  103
 
  4
 
 7403
 
 tss;island
 
 0.04185
 
 0.06399
 
 Not queried
 
  0.9047315
 
       NA
 
       NA
  
 
  
 15
 
  45722346
 
  45722694
 
  ENSEMBL   UCSC 
 
  349
 
  6
 
 13622
 
 tss
 
 0.04171
 
 0.08159
 
 Not queried
 
  0.9046074
 
       NA
 
       NA
  
 
  
 6
 
  32822857
 
  32823116
 
  ENSEMBL   UCSC 
 
  260
 
  4
 
 34816
 
 tss
 
 0.35837
 
 0.55822
 
 Not queried
 
  0.9045087
 
       NA
 
       NA
  
 
  
 10
 
  79470767
 
  79471309
 
  ENSEMBL   UCSC 
 
  543
 
  5
 
 5854
 
 island
 
 0.64178
 
 0.48906
 
 Not queried
 
  0.9043914
 
       NA
 
       NA
  
 
  
 2
 
 185462269
 
 185462928
 
  ENSEMBL   UCSC 
 
  660
 
  8
 
 23733
 
 tss;island
 
 0.04628
 
 0.10246
 
 Not queried
 
  0.9041854
 
       NA
 
       NA
  
 
  
 7
 
   2044499
 
   2044787
 
  ENSEMBL   UCSC 
 
  289
 
  4
 
 36721
 
 gene;island
 
 0.65084
 
 0.78303
 
 Not queried
 
  0.9039747
 
       NA
 
       NA
  
 
  
 X
 
  57147697
 
  57148257
 
  ENSEMBL   UCSC 
 
  561
 
  7
 
 41824
 
 island
 
 0.12092
 
 0.04381
 
 Not queried
 
  0.9039735
 
       NA
 
       NA
  
 
  
 11
 
  31530778
 
  31531135
 
  ENSEMBL   UCSC 
 
  358
 
  4
 
 6536
 
 gene
 
 0.01759
 
 0.02235
 
 Not queried
 
  0.9039343
 
       NA
 
       NA
  
 
  
 8
 
   1814745
 
   1815153
 
  ENSEMBL   UCSC 
 
  409
 
  6
 
 39117
 
 gene
 
 0.92183
 
 0.95543
 
 Not queried
 
  0.9039211
 
       NA
 
       NA
  
 
  
 4
 
 111558988
 
 111559134
 
  ENSEMBL   UCSC 
 
  147
 
  4
 
 30502
 
 tss;island
 
 0.18150
 
 0.11446
 
 Not queried
 
  0.9035881
 
       NA
 
       NA
  
 
  
 5
 
 137878905
 
 137879011
 
  ENSEMBL   UCSC 
 
  107
 
  5
 
 33099
 
 island
 
 0.01863
 
 0.03740
 
 Not queried
 
  0.9035746
 
       NA
 
       NA
  
 
  
 8
 
  27695307
 
  27695870
 
  ENSEMBL   UCSC 
 
  564
 
  9
 
 40122
 
 island
 
 0.05302
 
 0.15402
 
 Not queried
 
  0.9035421
 
       NA
 
       NA
  
 
  
 7
 
  30635712
 
  30635889
 
  ENSEMBL   UCSC 
 
  178
 
  4
 
 36893
 
 gene;island
 
 0.01246
 
 0.02125
 
 Not queried
 
  0.9035152
 
       NA
 
       NA
  
 
  
 21
 
  46378243
 
  46378625
 
  ENSEMBL   UCSC 
 
  383
 
  5
 
 26422
 
 gene;island
 
 0.82306
 
 0.90730
 
 Not queried
 
  0.9033027
 
       NA
 
       NA
  
 
  
 4
 
 122722220
 
 122722461
 
  ENSEMBL   UCSC 
 
  242
 
  5
 
 30526
 
 tss
 
 0.01544
 
 0.02850
 
 Not queried
 
  0.9031884
 
       NA
 
       NA
  
 
  
 7
 
  95115026
 
  95115246
 
  ENSEMBL   UCSC 
 
  221
 
  5
 
 37664
 
 tss
 
 0.83639
 
 0.90529
 
 Not queried
 
  0.9029346
 
       NA
 
       NA
  
 
  
 11
 
 125366238
 
 125366590
 
  ENSEMBL   UCSC 
 
  353
 
  9
 
 7769
 
 tss;island
 
 0.16306
 
 0.11123
 
 Not queried
 
  0.9027750
 
       NA
 
       NA
  
 
  
 22
 
  20790837
 
  20791300
 
  ENSEMBL   UCSC 
 
  464
 
  5
 
 26758
 
 gene;island
 
 0.02197
 
 0.03400
 
 Not queried
 
  0.9025483
 
       NA
 
       NA
  
 
  
 6
 
  31023592
 
  31023770
 
  ENSEMBL   UCSC 
 
  179
 
  4
 
 33785
 
 gene
 
 0.89433
 
 0.74484
 
 Not queried
 
  0.9025234
 
       NA
 
       NA
  
 
  
 6
 
  39759941
 
  39760092
 
  ENSEMBL   UCSC 
 
  152
 
  4
 
 34894
 
 tss;island
 
 0.05062
 
 0.02847
 
 Not queried
 
  0.9025103
 
       NA
 
       NA
  
 
  
 14
 
  70346151
 
  70346477
 
  ENSEMBL   UCSC 
 
  327
 
  5
 
 12099
 
 gene
 
 0.06415
 
 0.04293
 
 Not queried
 
  0.9024736
 
       NA
 
       NA
  
 
  
 12
 
   6797663
 
   6798118
 
  ENSEMBL   UCSC 
 
  456
 
  6
 
 9006
 
 gene;island
 
 0.06167
 
 0.04263
 
 Not queried
 
  0.9021767
 
       NA
 
       NA
  
 
  
 6
 
   6004686
 
   6004897
 
  ENSEMBL   UCSC 
 
  212
 
  4
 
 33481
 
 gene;island
 
 0.01822
 
 0.04755
 
 Not queried
 
  0.9020529
 
       NA
 
       NA
  
 
  
 16
 
  10674326
 
  10674663
 
  ENSEMBL   UCSC 
 
  338
 
  6
 
 15662
 
 island
 
 0.01424
 
 0.02602
 
 Not queried
 
  0.9020096
 
       NA
 
       NA
  
 
  
 8
 
   1771973
 
   1772393
 
  ENSEMBL   UCSC 
 
  421
 
  6
 
 39975
 
 island
 
 0.02692
 
 0.04131
 
 Not queried
 
  0.9019352
 
       NA
 
       NA
  
 
  
 7
 
   4118583
 
   4118868
 
  ENSEMBL   UCSC 
 
  286
 
  5
 
 36764
 
 gene
 
 0.82781
 
 0.91305
 
 Not queried
 
  0.9018917
 
       NA
 
       NA
  
 
  
 1
 
  47779824
 
  47780210
 
  ENSEMBL   UCSC 
 
  387
 
  9
 
 1605
 
 tss
 
 0.02120
 
 0.03724
 
 Not queried
 
  0.9015889
 
       NA
 
       NA
  
 
  
 10
 
 104628846
 
 104629179
 
  ENSEMBL   UCSC 
 
  334
 
  8
 
 5315
 
 tss;island
 
 0.02176
 
 0.03530
 
 Not queried
 
  0.9012945
 
       NA
 
       NA
  
 
  
 7
 
 157669880
 
 157670171
 
  ENSEMBL   UCSC 
 
  292
 
  4
 
 37285
 
 gene;island
 
 0.40779
 
 0.50722
 
 Not queried
 
  0.9012799
 
       NA
 
       NA
  
 
  
 3
 
 194393231
 
 194393536
 
  ENSEMBL   UCSC 
 
  306
 
  6
 
 28758
 
 tss
 
 0.01821
 
 0.03507
 
 Not queried
 
  0.9011029
 
       NA
 
       NA
  
 
  
 15
 
  76552574
 
  76552771
 
  ENSEMBL   UCSC 
 
  198
 
  4
 
 13793
 
 tss
 
 0.88502
 
 0.74502
 
 Not queried
 
  0.9010089
 
       NA
 
       NA
  
 
  
 12
 
 106480546
 
 106480771
 
  ENSEMBL   UCSC 
 
  226
 
  4
 
 9252
 
 gene
 
 0.90388
 
 0.84878
 
 Not queried
 
  0.9008100
 
       NA
 
       NA
  
 
  
 2
 
 223162666
 
 223163326
 
  ENSEMBL   UCSC 
 
  661
 
  9
 
 23010
 
 gene;island
 
 0.17669
 
 0.10310
 
 Not queried
 
  0.9007113
 
       NA
 
       NA
  
 
  
 X
 
  27481481
 
  27481558
 
  ENSEMBL   UCSC 
 
   78
 
  4
 
 41238
 
 tss
 
 0.77584
 
 0.90459
 
 Not queried
 
  0.9006971
 
       NA
 
       NA
  
 
  
 2
 
 131675994
 
 131676293
 
  ENSEMBL   UCSC 
 
  300
 
  4
 
 22832
 
 gene;island
 
 0.93230
 
 0.83511
 
 Not queried
 
  0.9005997
 
       NA
 
       NA
  
 
  
 7
 
  63667212
 
  63667572
 
  ENSEMBL   UCSC 
 
  361
 
  6
 
 37549
 
 tss
 
 0.76918
 
 0.62000
 
 Not queried
 
  0.9005663
 
       NA
 
       NA
  
 
  
 20
 
  62886664
 
  62887006
 
  ENSEMBL   UCSC 
 
  343
 
  7
 
 25877
 
 tss;island
 
 0.00873
 
 0.01943
 
 Not queried
 
  0.9005271
 
       NA
 
       NA
  
 
  
 4
 
 183061830
 
 183062004
 
  ENSEMBL   UCSC 
 
  175
 
  5
 
 30141
 
 gene;island
 
 0.86270
 
 0.77861
 
 Not queried
 
  0.9003775
 
       NA
 
       NA
  
 
  
 10
 
 118609728
 
 118609965
 
  ENSEMBL   UCSC 
 
  238
 
  4
 
 6150
 
 island
 
 0.51568
 
 0.43928
 
 Not queried
 
  0.9003722
 
       NA
 
       NA
  
 
  
 7
 
  44579245
 
  44579475
 
  ENSEMBL   UCSC 
 
  231
 
  5
 
 36930
 
 gene
 
 0.65353
 
 0.81852
 
 Not queried
 
  0.9001990
 
       NA
 
       NA
  
 
  
 11
 
 120894635
 
 120894801
 
  ENSEMBL   UCSC 
 
  167
 
  4
 
 7743
 
 tss
 
 0.01559
 
 0.02658
 
 Not queried
 
  0.9001044
 
       NA
 
       NA
  
 
  
 19
 
  30866192
 
  30866365
 
  ENSEMBL   UCSC 
 
  174
 
  4
 
 19935
 
 gene;island
 
 0.05976
 
 0.02238
 
 Not queried
 
  0.8999533
 
       NA
 
       NA
  
 
  
 13
 
  25254440
 
  25254512
 
  ENSEMBL   UCSC 
 
   73
 
  4
 
 11318
 
 tss;island
 
 0.02832
 
 0.06016
 
 Not queried
 
  0.8997977
 
       NA
 
       NA
  
 
  
 11
 
  43602845
 
  43602920
 
  ENSEMBL   UCSC 
 
   76
 
  6
 
 7233
 
 tss
 
 0.01443
 
 0.02311
 
 Not queried
 
  0.8997434
 
       NA
 
       NA
  
 
  
 3
 
  55522611
 
  55522716
 
  ENSEMBL   UCSC 
 
  106
 
  4
 
 28360
 
 tss
 
 0.01874
 
 0.02162
 
 Not queried
 
  0.8997397
 
       NA
 
       NA
  
 
  
 17
 
  60885491
 
  60886079
 
  ENSEMBL   UCSC 
 
  589
 
  7
 
 18604
 
 island
 
 0.04100
 
 0.13400
 
 Not queried
 
  0.8997311
 
       NA
 
       NA
  
 
  
 2
 
 131851172
 
 131851464
 
  ENSEMBL   UCSC 
 
  293
 
  6
 
 23594
 
 tss;island
 
 0.02488
 
 0.04654
 
 Not queried
 
  0.8996865
 
       NA
 
       NA
  
 
  
 17
 
  72427538
 
  72427693
 
  ENSEMBL   UCSC 
 
  156
 
  6
 
 18683
 
 island
 
 0.03494
 
 0.06591
 
 Not queried
 
  0.8995240
 
       NA
 
       NA
  
 
  
 12
 
  45269318
 
  45269790
 
  ENSEMBL   UCSC 
 
  473
 
  7
 
 9075
 
 gene;island
 
 0.06197
 
 0.05141
 
 Not queried
 
  0.8994863
 
       NA
 
       NA
  
 
  
 12
 
 118453982
 
 118454420
 
  ENSEMBL   UCSC 
 
  439
 
  9
 
 10021
 
 tss
 
 0.04569
 
 0.05071
 
 Not queried
 
  0.8991753
 
       NA
 
       NA
  
 
  
 X
 
 135382438
 
 135382675
 
  ENSEMBL   UCSC 
 
  238
 
  5
 
 41548
 
 tss;island
 
 0.91739
 
 0.78108
 
 Not queried
 
  0.8991135
 
       NA
 
       NA
  
 
  
 12
 
 120763487
 
 120763869
 
  ENSEMBL   UCSC 
 
  383
 
  5
 
 9324
 
 gene
 
 0.04806
 
 0.08673
 
 Not queried
 
  0.8991116
 
       NA
 
       NA
  
 
  
 11
 
  47664250
 
  47664508
 
  ENSEMBL   UCSC 
 
  259
 
  7
 
 7270
 
 tss;island
 
 0.03040
 
 0.03365
 
 Not queried
 
  0.8985956
 
       NA
 
       NA
  
 
  
 5
 
  68485086
 
  68485488
 
  ENSEMBL   UCSC 
 
  403
 
  7
 
 32785
 
 island
 
 0.01574
 
 0.02032
 
 Not queried
 
  0.8985547
 
       NA
 
       NA
  
 
  
 4
 
 184319589
 
 184320187
 
  ENSEMBL   UCSC 
 
  599
 
  7
 
 31397
 
 island
 
 0.19009
 
 0.30436
 
 Not queried
 
  0.8985055
 
       NA
 
       NA
  
 
  
 16
 
  11038165
 
  11038455
 
  ENSEMBL   UCSC 
 
  291
 
  5
 
 15668
 
 island
 
 0.03002
 
 0.04171
 
 Not queried
 
  0.8984906
 
       NA
 
       NA
  
 
  
 15
 
  43425285
 
  43425698
 
  ENSEMBL   UCSC 
 
  414
 
  7
 
 13595
 
 tss
 
 0.00591
 
 0.01369
 
 Not queried
 
  0.8984546
 
       NA
 
       NA
  
 
  
 16
 
  48277879
 
  48278202
 
  ENSEMBL   UCSC 
 
  324
 
  8
 
 15193
 
 tss
 
 0.04039
 
 0.04337
 
 Not queried
 
  0.8983540
 
       NA
 
       NA
  
 
  
 1
 
 119542159
 
 119542629
 
  ENSEMBL   UCSC 
 
  471
 
  6
 
 3648
 
 island
 
 0.82173
 
 0.71595
 
 Not queried
 
  0.8982443
 
       NA
 
       NA
  
 
  
 7
 
   1198522
 
   1198977
 
  ENSEMBL   UCSC 
 
  456
 
  5
 
 36690
 
 gene;island
 
 0.18918
 
 0.24450
 
 Not queried
 
  0.8982357
 
       NA
 
       NA
  
 
  
 11
 
  60524073
 
  60524323
 
  ENSEMBL   UCSC 
 
  251
 
  4
 
 7300
 
 tss
 
 0.18030
 
 0.33394
 
 Not queried
 
  0.8982233
 
       NA
 
       NA
  
 
  
 10
 
  13933996
 
  13934438
 
  ENSEMBL   UCSC 
 
  443
 
  5
 
 4492
 
 gene;island
 
 0.02260
 
 0.04131
 
 Not queried
 
  0.8981088
 
       NA
 
       NA
  
 
  
 22
 
  46449752
 
  46450114
 
  ENSEMBL   UCSC 
 
  363
 
  7
 
 26865
 
 gene;island
 
 0.77111
 
 0.86858
 
 Not queried
 
  0.8980263
 
       NA
 
       NA
  
 
  
 18
 
  43652255
 
  43652594
 
  ENSEMBL   UCSC 
 
  340
 
  8
 
 19218
 
 tss
 
 0.01605
 
 0.02481
 
 Not queried
 
  0.8979902
 
       NA
 
       NA
  
 
  
 15
 
  64648051
 
  64648652
 
  ENSEMBL   UCSC 
 
  602
 
 10
 
 14186
 
 island
 
 0.01803
 
 0.02352
 
 Not queried
 
  0.8979128
 
       NA
 
       NA
  
 
  
 1
 
   7844735
 
   7845237
 
  ENSEMBL   UCSC 
 
  503
 
  5
 
 2622
 
 island
 
 0.77069
 
 0.86230
 
 Not queried
 
  0.8978820
 
       NA
 
       NA
  
 
  
 19
 
  51531297
 
  51531484
 
  ENSEMBL   UCSC 
 
  188
 
  4
 
 20956
 
 tss;island
 
 0.57106
 
 0.48629
 
 Not queried
 
  0.8978747
 
       NA
 
       NA
  
 
  
 3
 
 127056837
 
 127057149
 
  ENSEMBL   UCSC 
 
  313
 
  5
 
 29399
 
 island
 
 0.82355
 
 0.80328
 
 Not queried
 
  0.8978298
 
       NA
 
       NA
  
 
  
 17
 
   9862752
 
   9863081
 
  ENSEMBL   UCSC 
 
  330
 
  9
 
 16403
 
 gene
 
 0.16930
 
 0.08655
 
 Not queried
 
  0.8977415
 
       NA
 
       NA
  
 
  
 19
 
   2588241
 
   2588629
 
  ENSEMBL   UCSC 
 
  389
 
  5
 
 19660
 
 gene;island
 
 0.74318
 
 0.86306
 
 Not queried
 
  0.8976121
 
       NA
 
       NA
  
 
  
 17
 
  25620944
 
  25621101
 
  ENSEMBL   UCSC 
 
  158
 
  7
 
 17171
 
 tss
 
 0.02201
 
 0.03273
 
 Not queried
 
  0.8976068
 
       NA
 
       NA
  
 
  
 1
 
   2334785
 
   2334974
 
  ENSEMBL   UCSC 
 
  190
 
  4
 
 82
 
 gene;island
 
 0.91428
 
 0.96601
 
 Not queried
 
  0.8974913
 
       NA
 
       NA
  
 
  
 12
 
  28122232
 
  28122626
 
  ENSEMBL   UCSC 
 
  395
 
  4
 
 9063
 
 gene;island
 
 0.10477
 
 0.05416
 
 Not queried
 
  0.8971341
 
       NA
 
       NA
  
 
  
 1
 
   6284844
 
   6285107
 
  ENSEMBL   UCSC 
 
  264
 
  4
 
 163
 
 gene
 
 0.86299
 
 0.90152
 
 Not queried
 
  0.8971241
 
       NA
 
       NA
  
 
  
 11
 
   2481857
 
   2482929
 
  ENSEMBL   UCSC 
 
 1073
 
 17
 
 6442
 
 gene
 
 0.74187
 
 0.81327
 
 Not queried
 
  0.8970937
 
       NA
 
       NA
  
 
  
 7
 
  99156634
 
  99157059
 
  ENSEMBL   UCSC 
 
  426
 
  4
 
 37068
 
 gene;island
 
 0.02602
 
 0.04359
 
 Not queried
 
  0.8970167
 
       NA
 
       NA
  
 
  
 7
 
  22894795
 
  22894898
 
  ENSEMBL   UCSC 
 
  104
 
  4
 
 37402
 
 tss;island
 
 0.01924
 
 0.02029
 
 Not queried
 
  0.8969492
 
       NA
 
       NA
  
 
  
 12
 
 108733253
 
 108733370
 
  ENSEMBL   UCSC 
 
  118
 
  6
 
 9955
 
 tss
 
 0.58639
 
 0.74516
 
 Not queried
 
  0.8968928
 
       NA
 
       NA
  
 
  
 13
 
  37004536
 
  37005582
 
  ENSEMBL   UCSC 
 
 1047
 
 27
 
 11368
 
 tss;island
 
 0.11879
 
 0.07229
 
 Not queried
 
  0.8966588
 
       NA
 
       NA
  
 
  
 13
 
  24844846
 
  24844938
 
  ENSEMBL   UCSC 
 
   93
 
  6
 
 11078
 
 gene
 
 0.45142
 
 0.29276
 
 Not queried
 
  0.8966048
 
       NA
 
       NA
  
 
  
 3
 
 125239063
 
 125239413
 
  ENSEMBL   UCSC 
 
  351
 
  8
 
 28509
 
 tss
 
 0.02404
 
 0.03564
 
 Not queried
 
  0.8964521
 
       NA
 
       NA
  
 
  
 3
 
 125239027
 
 125239413
 
  ENSEMBL   UCSC 
 
  387
 
 10
 
 29379
 
 island
 
 0.02404
 
 0.03564
 
 Not queried
 
  0.8964521
 
       NA
 
       NA
  
 
  
 X
 
 107975753
 
 107976214
 
  ENSEMBL   UCSC 
 
  462
 
  6
 
 41076
 
 gene;island
 
 0.02205
 
 0.04503
 
 Not queried
 
  0.8963389
 
       NA
 
       NA
  
 
  
 5
 
  80529067
 
  80529340
 
  ENSEMBL   UCSC 
 
  274
 
  6
 
 31609
 
 gene
 
 0.02940
 
 0.06736
 
 Not queried
 
  0.8961479
 
       NA
 
       NA
  
 
  
 6
 
  83073619
 
  83073964
 
  ENSEMBL   UCSC 
 
  346
 
  8
 
 34336
 
 gene;island
 
 0.03798
 
 0.07254
 
 Not queried
 
  0.8961029
 
       NA
 
       NA
  
 
  
 6
 
  30080189
 
  30080782
 
  ENSEMBL   UCSC 
 
  594
 
 10
 
 33662
 
 gene
 
 0.40344
 
 0.55673
 
 Not queried
 
  0.8958948
 
       NA
 
       NA
  
 
  
 4
 
 146402752
 
 146402992
 
  ENSEMBL   UCSC 
 
  241
 
  4
 
 30571
 
 tss;island
 
 0.01914
 
 0.02471
 
 Not queried
 
  0.8958667
 
       NA
 
       NA
  
 
  
 13
 
 113633379
 
 113633600
 
  ENSEMBL   UCSC 
 
  222
 
  4
 
 11518
 
 tss
 
 0.60099
 
 0.73947
 
 Not queried
 
  0.8957013
 
       NA
 
       NA
  
 
  
 2
 
 131594925
 
 131595321
 
  ENSEMBL   UCSC 
 
  397
 
  4
 
 24686
 
 island
 
 0.11091
 
 0.04786
 
 Not queried
 
  0.8955785
 
       NA
 
       NA
  
 
  
 13
 
 101327124
 
 101327190
 
  ENSEMBL   UCSC 
 
   67
 
  6
 
 11490
 
 tss;island
 
 0.01585
 
 0.02059
 
 Not queried
 
  0.8955264
 
       NA
 
       NA
  
 
  
 4
 
  95128661
 
  95129012
 
  ENSEMBL   UCSC 
 
  352
 
 11
 
 30454
 
 tss
 
 0.01621
 
 0.01151
 
 Not queried
 
  0.8951868
 
       NA
 
       NA
  
 
  
 11
 
  60282712
 
  60282864
 
  ENSEMBL   UCSC 
 
  153
 
  6
 
 7299
 
 tss
 
 0.93767
 
 0.86645
 
 Not queried
 
  0.8950239
 
       NA
 
       NA
  
 
  
 16
 
  58059105
 
  58059154
 
  ENSEMBL   UCSC 
 
   50
 
  5
 
 15238
 
 tss
 
 0.02555
 
 0.04648
 
 Not queried
 
  0.8947649
 
       NA
 
       NA
  
 
  
 15
 
  40674878
 
  40674910
 
  ENSEMBL   UCSC 
 
   33
 
  4
 
 13559
 
 tss
 
 0.02429
 
 0.02169
 
 Not queried
 
  0.8945980
 
       NA
 
       NA
  
 
  
 18
 
  49865711
 
  49866531
 
  ENSEMBL   UCSC 
 
  821
 
 17
 
 19248
 
 tss
 
 0.04897
 
 0.13576
 
 Not queried
 
  0.8945578
 
       NA
 
       NA
  
 
  
 12
 
  19593453
 
  19593684
 
  ENSEMBL   UCSC 
 
  232
 
  4
 
 9050
 
 gene;island
 
 0.11600
 
 0.18786
 
 Not queried
 
  0.8944947
 
       NA
 
       NA
  
 
  
 17
 
  72772340
 
  72772745
 
  ENSEMBL   UCSC 
 
  406
 
 10
 
 17598
 
 tss;island
 
 0.01376
 
 0.01742
 
 Not queried
 
  0.8944553
 
       NA
 
       NA
  
 
  
 21
 
  44588552
 
  44589128
 
  ENSEMBL   UCSC 
 
  577
 
  9
 
 26533
 
 tss
 
 0.69157
 
 0.77203
 
 Not queried
 
  0.8942711
 
       NA
 
       NA
  
 
  
 6
 
 143832855
 
 143833251
 
  ENSEMBL   UCSC 
 
  397
 
  8
 
 36467
 
 island
 
 0.08634
 
 0.04385
 
 Not queried
 
  0.8935254
 
       NA
 
       NA
  
 
  
 13
 
  45992551
 
  45992673
 
  ENSEMBL   UCSC 
 
  123
 
  6
 
 11393
 
 tss
 
 0.02263
 
 0.02136
 
 Not queried
 
  0.8931106
 
       NA
 
       NA
  
 
  
 6
 
 168402227
 
 168402503
 
  ENSEMBL   UCSC 
 
  277
 
  4
 
 36592
 
 island
 
 0.89318
 
 0.81866
 
 Not queried
 
  0.8931036
 
       NA
 
       NA
  
 
  
 1
 
  32801964
 
  32802312
 
  ENSEMBL   UCSC 
 
  349
 
  8
 
 1455
 
 tss;island
 
 0.13088
 
 0.07629
 
 Not queried
 
  0.8931033
 
       NA
 
       NA
  
 
  
 19
 
    847943
 
    848071
 
  ENSEMBL   UCSC 
 
  129
 
  4
 
 19598
 
 gene;island
 
 0.48286
 
 0.61941
 
 Not queried
 
  0.8930059
 
       NA
 
       NA
  
 
  
 1
 
  32083737
 
  32083990
 
  ENSEMBL   UCSC 
 
  254
 
  4
 
 381
 
 gene;island
 
 0.04854
 
 0.06743
 
 Not queried
 
  0.8929432
 
       NA
 
       NA
  
 
  
 16
 
  83986635
 
  83986941
 
  ENSEMBL   UCSC 
 
  307
 
  6
 
 14830
 
 gene
 
 0.02907
 
 0.07257
 
 Not queried
 
  0.8927712
 
       NA
 
       NA
  
 
  
 10
 
   1779730
 
   1780263
 
  ENSEMBL   UCSC 
 
  534
 
 10
 
 4900
 
 tss
 
 0.01858
 
 0.03987
 
 Not queried
 
  0.8927272
 
       NA
 
       NA
  
 
  
 6
 
 169242769
 
 169243075
 
  ENSEMBL   UCSC 
 
  307
 
  4
 
 36606
 
 island
 
 0.91108
 
 0.78876
 
 Not queried
 
  0.8926480
 
       NA
 
       NA
  
 
  
 1
 
  29557230
 
  29557820
 
  ENSEMBL   UCSC 
 
  591
 
  9
 
 2955
 
 island
 
 0.01537
 
 0.02995
 
 Not queried
 
  0.8925848
 
       NA
 
       NA
  
 
  
 4
 
 169239550
 
 169239900
 
  ENSEMBL   UCSC 
 
  351
 
  6
 
 30127
 
 gene
 
 0.03149
 
 0.04173
 
 Not queried
 
  0.8924548
 
       NA
 
       NA
  
 
  
 22
 
  17082581
 
  17082787
 
  ENSEMBL   UCSC 
 
  207
 
  4
 
 26896
 
 tss;island
 
 0.08205
 
 0.04523
 
 Not queried
 
  0.8923929
 
       NA
 
       NA
  
 
  
 16
 
  27279496
 
  27279887
 
  ENSEMBL   UCSC 
 
  392
 
  4
 
 14671
 
 gene;island
 
 0.17454
 
 0.29218
 
 Not queried
 
  0.8923668
 
       NA
 
       NA
  
 
  
 17
 
  21101571
 
  21101960
 
  ENSEMBL   UCSC 
 
  390
 
  4
 
 16450
 
 gene
 
 0.93950
 
 0.88154
 
 Not queried
 
  0.8922241
 
       NA
 
       NA
  
 
  
 6
 
  31690580
 
  31691050
 
  ENSEMBL   UCSC 
 
  471
 
  8
 
 34768
 
 tss
 
 0.74750
 
 0.76228
 
 Not queried
 
  0.8920437
 
       NA
 
       NA
  
 
  
 3
 
  42003553
 
  42003982
 
  ENSEMBL   UCSC 
 
  430
 
  9
 
 28967
 
 island
 
 0.01579
 
 0.02908
 
 Not queried
 
  0.8920389
 
       NA
 
       NA
  
 
  
 11
 
 120530774
 
 120530973
 
  ENSEMBL   UCSC 
 
  200
 
  5
 
 7742
 
 tss
 
 0.57320
 
 0.55909
 
 Not queried
 
  0.8919162
 
       NA
 
       NA
  
 
  
 16
 
  83986635
 
  83986795
 
  ENSEMBL   UCSC 
 
  161
 
  5
 
 15357
 
 tss
 
 0.01994
 
 0.03309
 
 Not queried
 
  0.8915987
 
       NA
 
       NA
  
 
  
 3
 
  52516316
 
  52516676
 
  ENSEMBL   UCSC 
 
  361
 
  6
 
 27778
 
 gene
 
 0.86516
 
 0.90531
 
 Not queried
 
  0.8914526
 
       NA
 
       NA
  
 
  
 4
 
  84377120
 
  84377400
 
  ENSEMBL   UCSC 
 
  281
 
  4
 
 30000
 
 gene
 
 0.01739
 
 0.02613
 
 Not queried
 
  0.8913817
 
       NA
 
       NA
  
 
  
 15
 
  99434633
 
  99434912
 
  ENSEMBL   UCSC 
 
  280
 
  5
 
 13469
 
 gene;island
 
 0.91052
 
 0.86268
 
 Not queried
 
  0.8913516
 
       NA
 
       NA
  
 
  
 8
 
  42751637
 
  42751762
 
  ENSEMBL   UCSC 
 
  126
 
  4
 
 39236
 
 gene;tss;island
 
 0.01912
 
 0.02597
 
 Not queried
 
  0.8913500
 
       NA
 
       NA
  
 
  
 22
 
  51038379
 
  51038723
 
  ENSEMBL   UCSC 
 
  345
 
  4
 
 27220
 
 tss;island
 
 0.07858
 
 0.12937
 
 Not queried
 
  0.8913194
 
       NA
 
       NA
  
 
  
 22
 
  39715126
 
  39715225
 
  ENSEMBL   UCSC 
 
  100
 
  4
 
 27112
 
 tss
 
 0.03284
 
 0.08088
 
 Not queried
 
  0.8911050
 
       NA
 
       NA
  
 
  
 X
 
  72667095
 
  72667379
 
  ENSEMBL   UCSC 
 
  285
 
  4
 
 41034
 
 gene
 
 0.65275
 
 0.48948
 
 Not queried
 
  0.8910754
 
       NA
 
       NA
  
 
  
 4
 
  81187601
 
  81187906
 
  ENSEMBL   UCSC 
 
  306
 
  5
 
 31083
 
 island
 
 0.04169
 
 0.03756
 
 Not queried
 
  0.8909367
 
       NA
 
       NA
  
 
  
 6
 
  75794800
 
  75795067
 
  ENSEMBL   UCSC 
 
  268
 
  4
 
 34327
 
 gene;island
 
 0.02652
 
 0.06022
 
 Not queried
 
  0.8908863
 
       NA
 
       NA
  
 
  
 16
 
   1557366
 
   1557733
 
  ENSEMBL   UCSC 
 
  368
 
  4
 
 14544
 
 gene;island
 
 0.90015
 
 0.90924
 
 Not queried
 
  0.8907817
 
       NA
 
       NA
  
 
  
 15
 
  96876248
 
  96876486
 
  ENSEMBL   UCSC 
 
  239
 
  5
 
 13465
 
 gene;tss;island
 
 0.01660
 
 0.03074
 
 Not queried
 
  0.8906537
 
       NA
 
       NA
  
 
  
 2
 
  98962900
 
  98963134
 
  ENSEMBL   UCSC 
 
  235
 
  4
 
 22763
 
 gene;island
 
 0.02966
 
 0.04085
 
 Not queried
 
  0.8906196
 
       NA
 
       NA
  
 
  
 20
 
  48428997
 
  48429251
 
  ENSEMBL   UCSC 
 
  255
 
  9
 
 26197
 
 island
 
 0.04739
 
 0.04755
 
 Not queried
 
  0.8901448
 
       NA
 
       NA
  
 
  
 7
 
   5632251
 
   5632424
 
  ENSEMBL   UCSC 
 
  174
 
  7
 
 37360
 
 tss
 
 0.02022
 
 0.02641
 
 Not queried
 
  0.8901017
 
       NA
 
       NA
  
 
  
 7
 
 100027352
 
 100027501
 
  ENSEMBL   UCSC 
 
  150
 
  5
 
 37716
 
 tss;island
 
 0.03510
 
 0.05811
 
 Not queried
 
  0.8899738
 
       NA
 
       NA
  
 
  
 6
 
  24646213
 
  24646782
 
  ENSEMBL   UCSC 
 
  570
 
  8
 
 35430
 
 island
 
 0.01915
 
 0.03281
 
 Not queried
 
  0.8899479
 
       NA
 
       NA
  
 
  
 6
 
  33286540
 
  33286885
 
  ENSEMBL   UCSC 
 
  346
 
  5
 
 34202
 
 gene;tss;island
 
 0.79033
 
 0.83991
 
 Not queried
 
  0.8898294
 
       NA
 
       NA
  
 
  
 17
 
  18908220
 
  18908324
 
  ENSEMBL   UCSC 
 
  105
 
  4
 
 16441
 
 gene;tss;island
 
 0.05680
 
 0.02801
 
 Not queried
 
  0.8898063
 
       NA
 
       NA
  
 
  
 1
 
 211306986
 
 211307408
 
  ENSEMBL   UCSC 
 
  423
 
  5
 
 990
 
 gene;island
 
 0.00495
 
 0.01568
 
 Not queried
 
  0.8897612
 
       NA
 
       NA
  
 
  
 5
 
  93905257
 
  93905482
 
  ENSEMBL   UCSC 
 
  226
 
  6
 
 31636
 
 gene;tss
 
 0.57497
 
 0.72451
 
 Not queried
 
  0.8896007
 
       NA
 
       NA
  
 
  
 17
 
  62971706
 
  62971911
 
  ENSEMBL   UCSC 
 
  206
 
  7
 
 17557
 
 tss
 
 0.01708
 
 0.02811
 
 Not queried
 
  0.8896002
 
       NA
 
       NA
  
 
  
 16
 
  23652268
 
  23653174
 
  ENSEMBL   UCSC 
 
  907
 
 17
 
 15110
 
 tss;island
 
 0.06586
 
 0.02932
 
 Not queried
 
  0.8894641
 
       NA
 
       NA
  
 
  
 22
 
  45073031
 
  45073165
 
  ENSEMBL   UCSC 
 
  135
 
  5
 
 26852
 
 gene
 
 0.82930
 
 0.88676
 
 Not queried
 
  0.8894470
 
       NA
 
       NA
  
 
  
 6
 
 127840517
 
 127840755
 
  ENSEMBL   UCSC 
 
  239
 
  8
 
 35153
 
 tss
 
 0.04553
 
 0.03077
 
 Not queried
 
  0.8894349
 
       NA
 
       NA
  
 
  
 22
 
  27053559
 
  27053934
 
  ENSEMBL   UCSC 
 
  376
 
  5
 
 26785
 
 gene
 
 0.06423
 
 0.02814
 
 Not queried
 
  0.8894116
 
       NA
 
       NA
  
 
  
 4
 
  38665594
 
  38665613
 
  ENSEMBL   UCSC 
 
   20
 
  5
 
 29931
 
 gene;tss;island
 
 0.04429
 
 0.08987
 
 Not queried
 
  0.8893675
 
       NA
 
       NA
  
 
  
 6
 
  33372767
 
  33372941
 
  ENSEMBL   UCSC 
 
  175
 
  5
 
 34209
 
 gene
 
 0.85312
 
 0.91495
 
 Not queried
 
  0.8891999
 
       NA
 
       NA
  
 
  
 10
 
   1779667
 
   1780263
 
  ENSEMBL   UCSC 
 
  597
 
 11
 
 5482
 
 island
 
 0.01380
 
 0.02965
 
 Not queried
 
  0.8891716
 
       NA
 
       NA
  
 
  
 7
 
   1748302
 
   1748766
 
  ENSEMBL   UCSC 
 
  465
 
  9
 
 37340
 
 tss
 
 0.88038
 
 0.75638
 
 Not queried
 
  0.8891391
 
       NA
 
       NA
  
 
  
 5
 
 150080676
 
 150080927
 
  ENSEMBL   UCSC 
 
  252
 
  9
 
 32373
 
 tss
 
 0.00766
 
 0.01902
 
 Not queried
 
  0.8890347
 
       NA
 
       NA
  
 
  
 12
 
  50361294
 
  50361679
 
  ENSEMBL   UCSC 
 
  386
 
  5
 
 10382
 
 island
 
 0.03812
 
 0.07712
 
 Not queried
 
  0.8888461
 
       NA
 
       NA
  
 
  
 X
 
  62780753
 
  62780853
 
  ENSEMBL   UCSC 
 
  101
 
  4
 
 41006
 
 gene
 
 0.02012
 
 0.02585
 
 Not queried
 
  0.8887726
 
       NA
 
       NA
  
 
  
 21
 
  41238940
 
  41239323
 
  ENSEMBL   UCSC 
 
  384
 
  4
 
 26512
 
 tss
 
 0.05237
 
 0.05391
 
 Not queried
 
  0.8886615
 
       NA
 
       NA
  
 
  
 17
 
   3500274
 
   3500417
 
  ENSEMBL   UCSC 
 
  144
 
  6
 
 16302
 
 gene;tss
 
 0.81687
 
 0.88194
 
 Not queried
 
  0.8886462
 
       NA
 
       NA
  
 
  
 11
 
  73086934
 
  73087233
 
  ENSEMBL   UCSC 
 
  300
 
  6
 
 7527
 
 tss;island
 
 0.12670
 
 0.27910
 
 Not queried
 
  0.8886284
 
       NA
 
       NA
  
 
  
 6
 
  32051857
 
  32052736
 
  ENSEMBL   UCSC 
 
  880
 
 16
 
 33976
 
 gene;island
 
 0.75825
 
 0.64280
 
 Not queried
 
  0.8883839
 
       NA
 
       NA
  
 
  
 X
 
 150067249
 
 150067397
 
  ENSEMBL   UCSC 
 
  149
 
  5
 
 41567
 
 tss
 
 0.01791
 
 0.02885
 
 Not queried
 
  0.8882145
 
       NA
 
       NA
  
 
  
 11
 
  65153757
 
  65154083
 
  ENSEMBL   UCSC 
 
  327
 
 10
 
 8430
 
 island
 
 0.04214
 
 0.04917
 
 Not queried
 
  0.8881977
 
       NA
 
       NA
  
 
  
 12
 
 125020047
 
 125020479
 
  ENSEMBL   UCSC 
 
  433
 
  8
 
 10944
 
 island
 
 0.84108
 
 0.93853
 
 Not queried
 
  0.8881957
 
       NA
 
       NA
  
 
  
 11
 
 117947459
 
 117947657
 
  ENSEMBL   UCSC 
 
  199
 
  5
 
 7703
 
 tss
 
 0.54341
 
 0.71335
 
 Not queried
 
  0.8880829
 
       NA
 
       NA
  
 
  
 6
 
  17600158
 
  17600760
 
  ENSEMBL   UCSC 
 
  603
 
  9
 
 35412
 
 island
 
 0.01886
 
 0.02760
 
 Not queried
 
  0.8880733
 
       NA
 
       NA
  
 
  
 5
 
 179224563
 
 179225039
 
  ENSEMBL   UCSC 
 
  477
 
  5
 
 33428
 
 island
 
 0.89387
 
 0.94070
 
 Not queried
 
  0.8880224
 
       NA
 
       NA
  
 
  
 18
 
  56338508
 
  56338584
 
  ENSEMBL   UCSC 
 
   77
 
  4
 
 19261
 
 tss
 
 0.01999
 
 0.02701
 
 Not queried
 
  0.8879682
 
       NA
 
       NA
  
 
  
 18
 
  56338508
 
  56338661
 
  ENSEMBL   UCSC 
 
  154
 
  6
 
 19504
 
 island
 
 0.01487
 
 0.02017
 
 Not queried
 
  0.8879682
 
       NA
 
       NA
  
 
  
 1
 
  46016123
 
  46016485
 
  ENSEMBL   UCSC 
 
  363
 
  7
 
 1584
 
 tss
 
 0.02610
 
 0.03925
 
 Not queried
 
  0.8879271
 
       NA
 
       NA
  
 
  
 3
 
  46718941
 
  46719086
 
  ENSEMBL   UCSC 
 
  146
 
  4
 
 27710
 
 gene;tss
 
 0.81575
 
 0.90979
 
 Not queried
 
  0.8876612
 
       NA
 
       NA
  
 
  
 20
 
  61885926
 
  61886313
 
  ENSEMBL   UCSC 
 
  388
 
  6
 
 25854
 
 tss
 
 0.05087
 
 0.03549
 
 Not queried
 
  0.8875172
 
       NA
 
       NA
  
 
  
 10
 
  75255789
 
  75256221
 
  ENSEMBL   UCSC 
 
  433
 
  8
 
 5143
 
 tss
 
 0.01163
 
 0.02083
 
 Not queried
 
  0.8874819
 
       NA
 
       NA
  
 
  
 14
 
  68141658
 
  68142077
 
  ENSEMBL   UCSC 
 
  420
 
  9
 
 12447
 
 tss;island
 
 0.01237
 
 0.03262
 
 Not queried
 
  0.8873298
 
       NA
 
       NA
  
 
  
 3
 
 147136904
 
 147137503
 
  ENSEMBL   UCSC 
 
  600
 
  7
 
 29531
 
 island
 
 0.04025
 
 0.02796
 
 Not queried
 
  0.8872273
 
       NA
 
       NA
  
 
  
 3
 
  53194945
 
  53195186
 
  ENSEMBL   UCSC 
 
  242
 
  7
 
 28354
 
 tss
 
 0.02317
 
 0.02212
 
 Not queried
 
  0.8871706
 
       NA
 
       NA
  
 
  
 7
 
 102985332
 
 102985587
 
  ENSEMBL   UCSC 
 
  256
 
  4
 
 37747
 
 tss
 
 0.15712
 
 0.04931
 
 Not queried
 
  0.8871160
 
       NA
 
       NA
  
 
  
 19
 
  14640367
 
  14640546
 
  ENSEMBL   UCSC 
 
  180
 
  9
 
 19860
 
 gene
 
 0.01762
 
 0.01653
 
 Not queried
 
  0.8870047
 
       NA
 
       NA
  
 
  
 6
 
  75912410
 
  75912711
 
  ENSEMBL   UCSC 
 
  302
 
  4
 
 34329
 
 gene;island
 
 0.58804
 
 0.35699
 
 Not queried
 
  0.8869575
 
       NA
 
       NA
  
 
  
 X
 
 101380291
 
 101380608
 
  ENSEMBL   UCSC 
 
  318
 
  8
 
 41428
 
 tss
 
 0.02919
 
 0.03867
 
 Not queried
 
  0.8869478
 
       NA
 
       NA
  
 
  
 5
 
 140764302
 
 140764748
 
  ENSEMBL   UCSC 
 
  447
 
  4
 
 31769
 
 gene;island
 
 0.43017
 
 0.61588
 
 Not queried
 
  0.8868083
 
       NA
 
       NA
  
 
  
 2
 
  46612724
 
  46613024
 
  ENSEMBL   UCSC 
 
  301
 
  4
 
 22629
 
 gene
 
 0.87956
 
 0.75628
 
 Not queried
 
  0.8867703
 
       NA
 
       NA
  
 
  
 18
 
  44259787
 
  44260091
 
  ENSEMBL   UCSC 
 
  305
 
  4
 
 19071
 
 gene;island
 
 0.93964
 
 0.89044
 
 Not queried
 
  0.8867390
 
       NA
 
       NA
  
 
  
 13
 
 103249000
 
 103249272
 
  ENSEMBL   UCSC 
 
  273
 
  7
 
 11494
 
 tss
 
 0.01684
 
 0.01123
 
 Not queried
 
  0.8867077
 
       NA
 
       NA
  
 
  
 13
 
 103249000
 
 103249370
 
  ENSEMBL   UCSC 
 
  371
 
  9
 
 11832
 
 island
 
 0.01684
 
 0.01353
 
 Not queried
 
  0.8867077
 
       NA
 
       NA
  
 
  
 22
 
  37823517
 
  37823656
 
  ENSEMBL   UCSC 
 
  140
 
  4
 
 27074
 
 tss;island
 
 0.16762
 
 0.34605
 
 Not queried
 
  0.8866107
 
       NA
 
       NA
  
 
  
 2
 
 157256809
 
 157257110
 
  ENSEMBL   UCSC 
 
  302
 
  4
 
 24763
 
 island
 
 0.01101
 
 0.02427
 
 Not queried
 
  0.8865557
 
       NA
 
       NA
  
 
  
 8
 
  48873190
 
  48873533
 
  ENSEMBL   UCSC 
 
  344
 
  4
 
 39626
 
 tss;island
 
 0.01012
 
 0.02420
 
 Not queried
 
  0.8865510
 
       NA
 
       NA
  
 
  
 4
 
 144434253
 
 144434637
 
  ENSEMBL   UCSC 
 
  385
 
 10
 
 31281
 
 island
 
 0.00992
 
 0.01475
 
 Not queried
 
  0.8862840
 
       NA
 
       NA
  
 
  
 13
 
 115080241
 
 115080469
 
  ENSEMBL   UCSC 
 
  229
 
  4
 
 11292
 
 gene;island
 
 0.01779
 
 0.00964
 
 Not queried
 
  0.8862128
 
       NA
 
       NA
  
 
  
 13
 
  50018044
 
  50018171
 
  ENSEMBL   UCSC 
 
  128
 
  4
 
 11137
 
 gene
 
 0.01763
 
 0.02952
 
 Not queried
 
  0.8860473
 
       NA
 
       NA
  
 
  
 16
 
  68544012
 
  68544404
 
  ENSEMBL   UCSC 
 
  393
 
  4
 
 16014
 
 island
 
 0.11781
 
 0.22750
 
 Not queried
 
  0.8860176
 
       NA
 
       NA
  
 
  
 15
 
  53075421
 
  53075877
 
  ENSEMBL   UCSC 
 
  457
 
  6
 
 13328
 
 gene;island
 
 0.03388
 
 0.04515
 
 Not queried
 
  0.8859866
 
       NA
 
       NA
  
 
  
 15
 
  43477606
 
  43478176
 
  ENSEMBL   UCSC 
 
  571
 
 11
 
 13284
 
 gene
 
 0.01760
 
 0.02668
 
 Not queried
 
  0.8859795
 
       NA
 
       NA
  
 
  
 7
 
   5463284
 
   5463911
 
  ENSEMBL   UCSC 
 
  628
 
  9
 
 37357
 
 tss
 
 0.09869
 
 0.09347
 
 Not queried
 
  0.8859356
 
       NA
 
       NA
  
 
  
 12
 
   6797846
 
   6798118
 
  ENSEMBL   UCSC 
 
  273
 
  5
 
 9496
 
 tss
 
 0.05948
 
 0.03483
 
 Not queried
 
  0.8859098
 
       NA
 
       NA
  
 
  
 11
 
 110300429
 
 110300584
 
  ENSEMBL   UCSC 
 
  156
 
  4
 
 7656
 
 tss
 
 0.07630
 
 0.02784
 
 Not queried
 
  0.8858516
 
       NA
 
       NA
  
 
  
 X
 
 104465196
 
 104465526
 
  ENSEMBL   UCSC 
 
  331
 
  5
 
 41067
 
 gene
 
 0.89552
 
 0.93633
 
 Not queried
 
  0.8858476
 
       NA
 
       NA
  
 
  
 4
 
  25090198
 
  25090665
 
  ENSEMBL   UCSC 
 
  468
 
  6
 
 30907
 
 island
 
 0.94003
 
 0.90855
 
 Not queried
 
  0.8858019
 
       NA
 
       NA
  
 
  
 19
 
  36210688
 
  36210913
 
  ENSEMBL   UCSC 
 
  226
 
  4
 
 19954
 
 gene;island
 
 0.73067
 
 0.79641
 
 Not queried
 
  0.8856748
 
       NA
 
       NA
  
 
  
 13
 
 113633379
 
 113634042
 
  ENSEMBL   UCSC 
 
  664
 
  8
 
 11234
 
 gene;island
 
 0.60099
 
 0.76277
 
 Not queried
 
  0.8853165
 
       NA
 
       NA
  
 
  
 7
 
  36700744
 
  36701016
 
  ENSEMBL   UCSC 
 
  273
 
  4
 
 36903
 
 gene
 
 0.83899
 
 0.92173
 
 Not queried
 
  0.8852371
 
       NA
 
       NA
  
 
  
 2
 
 220420805
 
 220420917
 
  ENSEMBL   UCSC 
 
  113
 
  4
 
 23002
 
 gene;island
 
 0.83919
 
 0.69085
 
 Not queried
 
  0.8851909
 
       NA
 
       NA
  
 
  
 19
 
  18761132
 
  18761564
 
  ENSEMBL   UCSC 
 
  433
 
  5
 
 19904
 
 gene;island
 
 0.70282
 
 0.90825
 
 Not queried
 
  0.8851317
 
       NA
 
       NA
  
 
  
 16
 
  48277879
 
  48278340
 
  ENSEMBL   UCSC 
 
  462
 
 11
 
 15870
 
 island
 
 0.01463
 
 0.02425
 
 Not queried
 
  0.8849983
 
       NA
 
       NA
  
 
  
 3
 
 183353051
 
 183353393
 
  ENSEMBL   UCSC 
 
  343
 
  5
 
 28704
 
 tss
 
 0.01096
 
 0.02006
 
 Not queried
 
  0.8849025
 
       NA
 
       NA
  
 
  
 3
 
 183353051
 
 183353451
 
  ENSEMBL   UCSC 
 
  401
 
  7
 
 29663
 
 island
 
 0.01096
 
 0.02006
 
 Not queried
 
  0.8849025
 
       NA
 
       NA
  
 
  
 20
 
  62205530
 
  62205981
 
  ENSEMBL   UCSC 
 
  452
 
  9
 
 26319
 
 island
 
 0.02398
 
 0.05907
 
 Not queried
 
  0.8848277
 
       NA
 
       NA
  
 
  
 15
 
  89903506
 
  89903704
 
  ENSEMBL   UCSC 
 
  199
 
  4
 
 14400
 
 island
 
 0.33998
 
 0.16674
 
 Not queried
 
  0.8848250
 
       NA
 
       NA
  
 
  
 16
 
  56677634
 
  56677855
 
  ENSEMBL   UCSC 
 
  222
 
  4
 
 14733
 
 gene
 
 0.16813
 
 0.23167
 
 Not queried
 
  0.8845034
 
       NA
 
       NA
  
 
  
 3
 
 139108945
 
 139109058
 
  ENSEMBL   UCSC 
 
  114
 
  4
 
 28589
 
 tss;island
 
 0.02149
 
 0.03024
 
 Not queried
 
  0.8844653
 
       NA
 
       NA
  
 
  
 1
 
  35544807
 
  35544887
 
  ENSEMBL   UCSC 
 
   81
 
  4
 
 1478
 
 tss
 
 0.02306
 
 0.04253
 
 Not queried
 
  0.8844378
 
       NA
 
       NA
  
 
  
 X
 
 149930750
 
 149930926
 
  ENSEMBL   UCSC 
 
  177
 
  4
 
 41136
 
 gene;island
 
 0.87254
 
 0.85671
 
 Not queried
 
  0.8843350
 
       NA
 
       NA
  
 
  
 10
 
   1595543
 
   1596108
 
  ENSEMBL   UCSC 
 
  566
 
  9
 
 4456
 
 gene;island
 
 0.78075
 
 0.86610
 
 Not queried
 
  0.8843018
 
       NA
 
       NA
  
 
  
 6
 
  27279917
 
  27280722
 
  ENSEMBL   UCSC 
 
  806
 
 12
 
 35491
 
 island
 
 0.02175
 
 0.06424
 
 Not queried
 
  0.8842612
 
       NA
 
       NA
  
 
  
 11
 
   1006985
 
   1007447
 
  ENSEMBL   UCSC 
 
  463
 
  7
 
 6381
 
 gene;island
 
 0.80956
 
 0.91010
 
 Not queried
 
  0.8841666
 
       NA
 
       NA
  
 
  
 19
 
  55628995
 
  55629363
 
  ENSEMBL   UCSC 
 
  369
 
  7
 
 21034
 
 tss;island
 
 0.03211
 
 0.06718
 
 Not queried
 
  0.8837801
 
       NA
 
       NA
  
 
  
 13
 
  21348120
 
  21348215
 
  ENSEMBL   UCSC 
 
   96
 
  5
 
 11306
 
 tss
 
 0.02677
 
 0.04097
 
 Not queried
 
  0.8836945
 
       NA
 
       NA
  
 
  
 15
 
  43477396
 
  43478095
 
  ENSEMBL   UCSC 
 
  700
 
 12
 
 13596
 
 tss
 
 0.02332
 
 0.03351
 
 Not queried
 
  0.8836832
 
       NA
 
       NA
  
 
  
 12
 
  57824134
 
  57824322
 
  ENSEMBL   UCSC 
 
  189
 
  4
 
 10531
 
 island
 
 0.02159
 
 0.03979
 
 Not queried
 
  0.8836815
 
       NA
 
       NA
  
 
  
 16
 
    699218
 
    699357
 
  ENSEMBL   UCSC 
 
  140
 
  5
 
 14941
 
 tss
 
 0.85693
 
 0.93623
 
 Not queried
 
  0.8833766
 
       NA
 
       NA
  
 
  
 20
 
  30697241
 
  30697291
 
  ENSEMBL   UCSC 
 
   51
 
  5
 
 25633
 
 tss
 
 0.02067
 
 0.02322
 
 Not queried
 
  0.8833135
 
       NA
 
       NA
  
 
  
 15
 
  75748063
 
  75748191
 
  ENSEMBL   UCSC 
 
  129
 
  5
 
 14303
 
 island
 
 0.02400
 
 0.04338
 
 Not queried
 
  0.8831059
 
       NA
 
       NA
  
 
  
 2
 
   3622594
 
   3622752
 
  ENSEMBL   UCSC 
 
  159
 
  6
 
 23124
 
 tss;island
 
 0.02177
 
 0.03741
 
 Not queried
 
  0.8830828
 
       NA
 
       NA
  
 
  
 3
 
  51704960
 
  51705150
 
  ENSEMBL   UCSC 
 
  191
 
  7
 
 28323
 
 tss;island
 
 0.02748
 
 0.06267
 
 Not queried
 
  0.8829655
 
       NA
 
       NA
  
 
  
 17
 
   1395864
 
   1396123
 
  ENSEMBL   UCSC 
 
  260
 
  5
 
 16954
 
 tss;island
 
 0.77115
 
 0.60728
 
 Not queried
 
  0.8828890
 
       NA
 
       NA
  
 
  
 13
 
 113260640
 
 113260912
 
  ENSEMBL   UCSC 
 
  273
 
  4
 
 11897
 
 island
 
 0.02645
 
 0.03389
 
 Not queried
 
  0.8828081
 
       NA
 
       NA
  
 
  
 11
 
   2170831
 
   2171184
 
  ENSEMBL   UCSC 
 
  354
 
  6
 
 6431
 
 gene
 
 0.46813
 
 0.59683
 
 Not queried
 
  0.8827191
 
       NA
 
       NA
  
 
  
 7
 
   1071539
 
   1071921
 
  ENSEMBL   UCSC 
 
  383
 
  5
 
 36676
 
 gene;island
 
 0.79283
 
 0.87413
 
 Not queried
 
  0.8826387
 
       NA
 
       NA
  
 
  
 17
 
  72368048
 
  72368401
 
  ENSEMBL   UCSC 
 
  354
 
  5
 
 16719
 
 gene;island
 
 0.61994
 
 0.75550
 
 Not queried
 
  0.8826211
 
       NA
 
       NA
  
 
  
 19
 
  17931932
 
  17932244
 
  ENSEMBL   UCSC 
 
  313
 
  5
 
 19887
 
 gene
 
 0.87920
 
 0.80451
 
 Not queried
 
  0.8826185
 
       NA
 
       NA
  
 
  
 16
 
  23847556
 
  23848003
 
  ENSEMBL   UCSC 
 
  448
 
  5
 
 14666
 
 gene;island
 
 0.08597
 
 0.10290
 
 Not queried
 
  0.8825562
 
       NA
 
       NA
  
 
  
 1
 
 208083913
 
 208084640
 
  ENSEMBL   UCSC 
 
  728
 
  9
 
 979
 
 gene;island
 
 0.19747
 
 0.12333
 
 Not queried
 
  0.8820673
 
       NA
 
       NA
  
 
  
 2
 
 232265670
 
 232266036
 
  ENSEMBL   UCSC 
 
  367
 
  4
 
 25143
 
 island
 
 0.55003
 
 0.59726
 
 Not queried
 
  0.8819292
 
       NA
 
       NA
  
 
  
 2
 
 233470647
 
 233471248
 
  ENSEMBL   UCSC 
 
  602
 
 10
 
 25162
 
 island
 
 0.02549
 
 0.04143
 
 Not queried
 
  0.8818743
 
       NA
 
       NA
  
 
  
 13
 
  95248247
 
  95248457
 
  ENSEMBL   UCSC 
 
  211
 
  5
 
 11163
 
 gene
 
 0.00897
 
 0.01640
 
 Not queried
 
  0.8818684
 
       NA
 
       NA
  
 
  
 13
 
  95248247
 
  95248888
 
  ENSEMBL   UCSC 
 
  642
 
 11
 
 11790
 
 island
 
 0.01144
 
 0.02018
 
 Not queried
 
  0.8818684
 
       NA
 
       NA
  
 
  
 20
 
  33759605
 
  33760017
 
  ENSEMBL   UCSC 
 
  413
 
  7
 
 26080
 
 island
 
 0.17160
 
 0.10121
 
 Not queried
 
  0.8817766
 
       NA
 
       NA
  
 
  
 10
 
 101380128
 
 101380456
 
  ENSEMBL   UCSC 
 
  329
 
  8
 
 5994
 
 island
 
 0.02993
 
 0.04679
 
 Not queried
 
  0.8814808
 
       NA
 
       NA
  
 
  
 6
 
  17282284
 
  17282859
 
  ENSEMBL   UCSC 
 
  576
 
 10
 
 33523
 
 gene;tss;island
 
 0.02496
 
 0.05147
 
 Not queried
 
  0.8814573
 
       NA
 
       NA
  
 
  
 15
 
 100882213
 
 100882647
 
  ENSEMBL   UCSC 
 
  435
 
  8
 
 13898
 
 tss
 
 0.03180
 
 0.07497
 
 Not queried
 
  0.8814543
 
       NA
 
       NA
  
 
  
 15
 
  43477396
 
  43478176
 
  ENSEMBL   UCSC 
 
  781
 
 13
 
 14054
 
 island
 
 0.02238
 
 0.02668
 
 Not queried
 
  0.8813870
 
       NA
 
       NA
  
 
  
 1
 
  29450569
 
  29450836
 
  ENSEMBL   UCSC 
 
  268
 
  6
 
 1436
 
 tss;island
 
 0.01479
 
 0.02449
 
 Not queried
 
  0.8812516
 
       NA
 
       NA
  
 
  
 3
 
  59035729
 
  59036158
 
  ENSEMBL   UCSC 
 
  430
 
  5
 
 28377
 
 tss
 
 0.05769
 
 0.09822
 
 Not queried
 
  0.8812281
 
       NA
 
       NA
  
 
  
 16
 
  66400320
 
  66400411
 
  ENSEMBL   UCSC 
 
   92
 
  4
 
 15248
 
 tss
 
 0.71292
 
 0.81327
 
 Not queried
 
  0.8808569
 
       NA
 
       NA
  
 
  
 1
 
 202311100
 
 202311278
 
  ENSEMBL   UCSC 
 
  179
 
  6
 
 2174
 
 tss
 
 0.00958
 
 0.01694
 
 Not queried
 
  0.8805565
 
       NA
 
       NA
  
 
  
 12
 
 132606563
 
 132606805
 
  ENSEMBL   UCSC 
 
  243
 
  6
 
 9407
 
 gene;island
 
 0.92894
 
 0.96691
 
 Not queried
 
  0.8804687
 
       NA
 
       NA
  
 
  
 6
 
  30646707
 
  30647148
 
  ENSEMBL   UCSC 
 
  442
 
 13
 
 33737
 
 gene
 
 0.09842
 
 0.15463
 
 Not queried
 
  0.8804593
 
       NA
 
       NA
  
 
  
 14
 
  95236539
 
  95236615
 
  ENSEMBL   UCSC 
 
   77
 
  4
 
 12557
 
 tss;island
 
 0.01891
 
 0.01046
 
 Not queried
 
  0.8804207
 
       NA
 
       NA
  
 
  
 17
 
  72732698
 
  72733290
 
  ENSEMBL   UCSC 
 
  593
 
 13
 
 17596
 
 tss
 
 0.04504
 
 0.04534
 
 Not queried
 
  0.8802872
 
       NA
 
       NA
  
 
  
 5
 
 159845898
 
 159846119
 
  ENSEMBL   UCSC 
 
  222
 
  4
 
 31819
 
 gene
 
 0.03064
 
 0.03465
 
 Not queried
 
  0.8800466
 
       NA
 
       NA
  
 
  
 2
 
 223161771
 
 223162128
 
  ENSEMBL   UCSC 
 
  358
 
  4
 
 23009
 
 gene;tss;island
 
 0.51512
 
 0.62272
 
 Not queried
 
  0.8798721
 
       NA
 
       NA
  
 
  
 6
 
  30295772
 
  30296049
 
  ENSEMBL   UCSC 
 
  278
 
  4
 
 33697
 
 gene;tss;island
 
 0.83304
 
 0.91105
 
 Not queried
 
  0.8797498
 
       NA
 
       NA
  
 
  
 10
 
 119293800
 
 119294332
 
  ENSEMBL   UCSC 
 
  533
 
  5
 
 4767
 
 gene;island
 
 0.18460
 
 0.27580
 
 Not queried
 
  0.8797380
 
       NA
 
       NA
  
 
  
 10
 
  43133935
 
  43134302
 
  ENSEMBL   UCSC 
 
  368
 
  8
 
 5666
 
 island
 
 0.02503
 
 0.05108
 
 Not queried
 
  0.8797105
 
       NA
 
       NA
  
 
  
 6
 
  53213948
 
  53214258
 
  ENSEMBL   UCSC 
 
  311
 
  8
 
 34975
 
 tss
 
 0.09652
 
 0.06645
 
 Not queried
 
  0.8796090
 
       NA
 
       NA
  
 
  
 3
 
 179280643
 
 179280746
 
  ENSEMBL   UCSC 
 
  104
 
  5
 
 29637
 
 island
 
 0.03397
 
 0.06445
 
 Not queried
 
  0.8792804
 
       NA
 
       NA
  
 
  
 17
 
  46800992
 
  46801314
 
  ENSEMBL   UCSC 
 
  323
 
  4
 
 16628
 
 gene;tss;island
 
 0.04209
 
 0.06998
 
 Not queried
 
  0.8792592
 
       NA
 
       NA
  
 
  
 2
 
  75426726
 
  75427699
 
  ENSEMBL   UCSC 
 
  974
 
 12
 
 23430
 
 tss;island
 
 0.01931
 
 0.05470
 
 Not queried
 
  0.8791277
 
       NA
 
       NA
  
 
  
 22
 
  42353589
 
  42353933
 
  ENSEMBL   UCSC 
 
  345
 
  4
 
 26844
 
 gene;island
 
 0.15065
 
 0.37259
 
 Not queried
 
  0.8789701
 
       NA
 
       NA
  
 
  
 19
 
  51529846
 
  51529999
 
  ENSEMBL   UCSC 
 
  154
 
  4
 
 20152
 
 gene
 
 0.69181
 
 0.45206
 
 Not queried
 
  0.8787555
 
       NA
 
       NA
  
 
  
 8
 
 124428540
 
 124428910
 
  ENSEMBL   UCSC 
 
  371
 
  6
 
 39836
 
 tss;island
 
 0.04283
 
 0.06161
 
 Not queried
 
  0.8786916
 
       NA
 
       NA
  
 
  
 4
 
  96470887
 
  96471143
 
  ENSEMBL   UCSC 
 
  257
 
  4
 
 30458
 
 tss;island
 
 0.01166
 
 0.02368
 
 Not queried
 
  0.8786185
 
       NA
 
       NA
  
 
  
 20
 
  30225325
 
  30226046
 
  ENSEMBL   UCSC 
 
  722
 
  9
 
 26037
 
 island
 
 0.79790
 
 0.89118
 
 Not queried
 
  0.8784505
 
       NA
 
       NA
  
 
  
 8
 
  39441968
 
  39442089
 
  ENSEMBL   UCSC 
 
  122
 
  5
 
 39600
 
 tss
 
 0.96757
 
 0.93728
 
 Not queried
 
  0.8780615
 
       NA
 
       NA
  
 
  
 12
 
   8123380
 
   8123627
 
  ENSEMBL   UCSC 
 
  248
 
  4
 
 10204
 
 island
 
 0.01024
 
 0.01670
 
 Not queried
 
  0.8779678
 
       NA
 
       NA
  
 
  
 X
 
 133371383
 
 133371717
 
  ENSEMBL   UCSC 
 
  335
 
  4
 
 41110
 
 gene;island
 
 0.02055
 
 0.02164
 
 Not queried
 
  0.8779172
 
       NA
 
       NA
  
 
  
 3
 
  85008587
 
  85008991
 
  ENSEMBL   UCSC 
 
  405
 
  5
 
 27823
 
 gene;island
 
 0.02264
 
 0.04084
 
 Not queried
 
  0.8778958
 
       NA
 
       NA
  
 
  
 7
 
  27142427
 
  27142810
 
  ENSEMBL   UCSC 
 
  384
 
 11
 
 37422
 
 tss
 
 0.03233
 
 0.06116
 
 Not queried
 
  0.8778855
 
       NA
 
       NA
  
 
  
 17
 
  46655164
 
  46655736
 
  ENSEMBL   UCSC 
 
  573
 
 11
 
 16618
 
 gene
 
 0.02144
 
 0.04124
 
 Not queried
 
  0.8778836
 
       NA
 
       NA
  
 
  
 5
 
  86563744
 
  86564119
 
  ENSEMBL   UCSC 
 
  376
 
  6
 
 32103
 
 tss;island
 
 0.03895
 
 0.06961
 
 Not queried
 
  0.8778705
 
       NA
 
       NA
  
 
  
 19
 
    827715
 
    827821
 
  ENSEMBL   UCSC 
 
  107
 
  4
 
 20243
 
 tss
 
 0.76553
 
 0.66037
 
 Not queried
 
  0.8777733
 
       NA
 
       NA
  
 
  
 4
 
    780856
 
    781272
 
  ENSEMBL   UCSC 
 
  417
 
  4
 
 29794
 
 gene;island
 
 0.78266
 
 0.87269
 
 Not queried
 
  0.8776486
 
       NA
 
       NA
  
 
  
 15
 
  45926780
 
  45927255
 
  ENSEMBL   UCSC 
 
  476
 
  9
 
 14088
 
 island
 
 0.07043
 
 0.04301
 
 Not queried
 
  0.8774365
 
       NA
 
       NA
  
 
  
 8
 
  49647579
 
  49648363
 
  ENSEMBL   UCSC 
 
  785
 
 11
 
 40219
 
 island
 
 0.01991
 
 0.07144
 
 Not queried
 
  0.8773126
 
       NA
 
       NA
  
 
  
 11
 
  62572592
 
  62572907
 
  ENSEMBL   UCSC 
 
  316
 
  4
 
 6630
 
 gene
 
 0.01388
 
 0.02471
 
 Not queried
 
  0.8772131
 
       NA
 
       NA
  
 
  
 19
 
  59010761
 
  59011010
 
  ENSEMBL   UCSC 
 
  250
 
  4
 
 20225
 
 gene;island
 
 0.02277
 
 0.04122
 
 Not queried
 
  0.8771198
 
       NA
 
       NA
  
 
  
 19
 
  54619057
 
  54619166
 
  ENSEMBL   UCSC 
 
  110
 
  7
 
 20178
 
 gene;tss;island
 
 0.01501
 
 0.02004
 
 Not queried
 
  0.8770563
 
       NA
 
       NA
  
 
  
 12
 
  75699252
 
  75699625
 
  ENSEMBL   UCSC 
 
  374
 
  6
 
 9196
 
 gene;island
 
 0.68476
 
 0.74701
 
 Not queried
 
  0.8770225
 
       NA
 
       NA
  
 
  
 6
 
  29999615
 
  29999908
 
  ENSEMBL   UCSC 
 
  294
 
  6
 
 33647
 
 gene
 
 0.74805
 
 0.87803
 
 Not queried
 
  0.8766896
 
       NA
 
       NA
  
 
  
 11
 
 123172362
 
 123172864
 
  ENSEMBL   UCSC 
 
  503
 
  5
 
 8883
 
 island
 
 0.02369
 
 0.02446
 
 Not queried
 
  0.8766488
 
       NA
 
       NA
  
 
  
 2
 
 160654866
 
 160655081
 
  ENSEMBL   UCSC 
 
  216
 
  4
 
 23644
 
 tss;island
 
 0.32260
 
 0.21242
 
 Not queried
 
  0.8765324
 
       NA
 
       NA
  
 
  
 1
 
  35324574
 
  35324786
 
  ENSEMBL   UCSC 
 
  213
 
  5
 
 415
 
 gene;island
 
 0.02305
 
 0.04153
 
 Not queried
 
  0.8765297
 
       NA
 
       NA
  
 
  
 16
 
  77756058
 
  77756761
 
  ENSEMBL   UCSC 
 
  704
 
  8
 
 16076
 
 island
 
 0.02404
 
 0.03480
 
 Not queried
 
  0.8762759
 
       NA
 
       NA
  
 
  
 20
 
  57041294
 
  57041622
 
  ENSEMBL   UCSC 
 
  329
 
  4
 
 25430
 
 gene;island
 
 0.79555
 
 0.87897
 
 Not queried
 
  0.8762237
 
       NA
 
       NA
  
 
  
 17
 
   6347533
 
   6347791
 
  ENSEMBL   UCSC 
 
  259
 
  5
 
 17015
 
 tss
 
 0.03933
 
 0.02400
 
 Not queried
 
  0.8760076
 
       NA
 
       NA
  
 
  
 17
 
   6347533
 
   6348200
 
  ENSEMBL   UCSC 
 
  668
 
 11
 
 17871
 
 island
 
 0.03933
 
 0.02400
 
 Not queried
 
  0.8760076
 
       NA
 
       NA
  
 
  
 1
 
 162792092
 
 162792613
 
  ENSEMBL   UCSC 
 
  522
 
  6
 
 3909
 
 island
 
 0.02325
 
 0.03978
 
 Not queried
 
  0.8759976
 
       NA
 
       NA
  
 
  
 16
 
   3507460
 
   3508002
 
  ENSEMBL   UCSC 
 
  543
 
  8
 
 14600
 
 gene;island
 
 0.02888
 
 0.08162
 
 Not queried
 
  0.8755113
 
       NA
 
       NA
  
 
  
 17
 
  15689920
 
  15690181
 
  ENSEMBL   UCSC 
 
  262
 
  4
 
 18011
 
 island
 
 0.81936
 
 0.66813
 
 Not queried
 
  0.8754869
 
       NA
 
       NA
  
 
  
 1
 
  52521668
 
  52521894
 
  ENSEMBL   UCSC 
 
  227
 
  4
 
 1625
 
 tss;island
 
 0.00717
 
 0.01266
 
 Not queried
 
  0.8752845
 
       NA
 
       NA
  
 
  
 10
 
 102027447
 
 102027787
 
  ENSEMBL   UCSC 
 
  341
 
  8
 
 5272
 
 tss
 
 0.01875
 
 0.03368
 
 Not queried
 
  0.8752395
 
       NA
 
       NA
  
 
  
 10
 
 102027002
 
 102027787
 
  ENSEMBL   UCSC 
 
  786
 
 12
 
 6002
 
 island
 
 0.01574
 
 0.02747
 
 Not queried
 
  0.8752395
 
       NA
 
       NA
  
 
  
 19
 
  37997675
 
  37998171
 
  ENSEMBL   UCSC 
 
  497
 
 11
 
 21922
 
 island
 
 0.00768
 
 0.01633
 
 Not queried
 
  0.8751648
 
       NA
 
       NA
  
 
  
 1
 
   3738334
 
   3738610
 
  ENSEMBL   UCSC 
 
  277
 
  5
 
 146
 
 gene
 
 0.77226
 
 0.85812
 
 Not queried
 
  0.8751425
 
       NA
 
       NA
  
 
  
 8
 
    179176
 
    179287
 
  ENSEMBL   UCSC 
 
  112
 
  4
 
 39100
 
 gene;island
 
 0.92591
 
 0.85443
 
 Not queried
 
  0.8750844
 
       NA
 
       NA
  
 
  
 11
 
 111944731
 
 111944894
 
  ENSEMBL   UCSC 
 
  164
 
  5
 
 6885
 
 gene
 
 0.00984
 
 0.02044
 
 Not queried
 
  0.8750096
 
       NA
 
       NA
  
 
  
 14
 
  91580380
 
  91580613
 
  ENSEMBL   UCSC 
 
  234
 
  6
 
 12148
 
 gene
 
 0.03914
 
 0.01678
 
 Not queried
 
  0.8749893
 
       NA
 
       NA
  
 
  
 1
 
  45965343
 
  45965625
 
  ENSEMBL   UCSC 
 
  283
 
  4
 
 502
 
 gene
 
 0.03455
 
 0.04209
 
 Not queried
 
  0.8748982
 
       NA
 
       NA
  
 
  
 3
 
 184209105
 
 184209412
 
  ENSEMBL   UCSC 
 
  308
 
  4
 
 29684
 
 island
 
 0.69340
 
 0.55027
 
 Not queried
 
  0.8747517
 
       NA
 
       NA
  
 
  
 5
 
   4866318
 
   4866602
 
  ENSEMBL   UCSC 
 
  285
 
  4
 
 32610
 
 island
 
 0.81752
 
 0.90834
 
 Not queried
 
  0.8747238
 
       NA
 
       NA
  
 
  
 10
 
  46222474
 
  46222657
 
  ENSEMBL   UCSC 
 
  184
 
  4
 
 5698
 
 island
 
 0.01616
 
 0.02115
 
 Not queried
 
  0.8745638
 
       NA
 
       NA
  
 
  
 6
 
  56112392
 
  56112696
 
  ENSEMBL   UCSC 
 
  305
 
  8
 
 34980
 
 tss
 
 0.05309
 
 0.02743
 
 Not queried
 
  0.8743009
 
       NA
 
       NA
  
 
  
 4
 
  54424280
 
  54424787
 
  ENSEMBL   UCSC 
 
  508
 
  5
 
 29958
 
 gene
 
 0.61641
 
 0.77372
 
 Not queried
 
  0.8741394
 
       NA
 
       NA
  
 
  
 12
 
  53472792
 
  53473136
 
  ENSEMBL   UCSC 
 
  345
 
  4
 
 9120
 
 gene
 
 0.09553
 
 0.10360
 
 Not queried
 
  0.8740155
 
       NA
 
       NA
  
 
  
 20
 
  62738197
 
  62738358
 
  ENSEMBL   UCSC 
 
  162
 
  5
 
 25875
 
 tss
 
 0.66657
 
 0.78978
 
 Not queried
 
  0.8737992
 
       NA
 
       NA
  
 
  
 17
 
  76129984
 
  76130496
 
  ENSEMBL   UCSC 
 
  513
 
  4
 
 16780
 
 gene;island
 
 0.68835
 
 0.69577
 
 Not queried
 
  0.8736545
 
       NA
 
       NA
  
 
  
 1
 
 116370764
 
 116371475
 
  ENSEMBL   UCSC 
 
  712
 
  7
 
 3621
 
 island
 
 0.02234
 
 0.03525
 
 Not queried
 
  0.8734420
 
       NA
 
       NA
  
 
  
 8
 
   1441386
 
   1441539
 
  ENSEMBL   UCSC 
 
  154
 
  4
 
 39962
 
 island
 
 0.85104
 
 0.90689
 
 Not queried
 
  0.8733614
 
       NA
 
       NA
  
 
  
 2
 
 170220063
 
 170220388
 
  ENSEMBL   UCSC 
 
  326
 
  4
 
 23663
 
 tss;island
 
 0.07087
 
 0.11853
 
 Not queried
 
  0.8732660
 
       NA
 
       NA
  
 
  
 20
 
  55100881
 
  55101089
 
  ENSEMBL   UCSC 
 
  209
 
  5
 
 25426
 
 gene
 
 0.70802
 
 0.86744
 
 Not queried
 
  0.8732648
 
       NA
 
       NA
  
 
  
 14
 
  20923200
 
  20923486
 
  ENSEMBL   UCSC 
 
  287
 
  4
 
 12244
 
 tss;island
 
 0.01916
 
 0.02900
 
 Not queried
 
  0.8730142
 
       NA
 
       NA
  
 
  
 1
 
 214170114
 
 214170376
 
  ENSEMBL   UCSC 
 
  263
 
  5
 
 1002
 
 gene
 
 0.86614
 
 0.92457
 
 Not queried
 
  0.8728329
 
       NA
 
       NA
  
 
  
 12
 
  74564847
 
  74565116
 
  ENSEMBL   UCSC 
 
  270
 
  4
 
 10602
 
 island
 
 0.62191
 
 0.76590
 
 Not queried
 
  0.8726707
 
       NA
 
       NA
  
 
  
 X
 
  47441573
 
  47441779
 
  ENSEMBL   UCSC 
 
  207
 
  5
 
 40978
 
 gene
 
 0.04081
 
 0.03303
 
 Not queried
 
  0.8725226
 
       NA
 
       NA
  
 
  
 4
 
  81256552
 
  81257269
 
  ENSEMBL   UCSC 
 
  718
 
 12
 
 31084
 
 island
 
 0.02198
 
 0.02218
 
 Not queried
 
  0.8724098
 
       NA
 
       NA
  
 
  
 7
 
 158380884
 
 158381220
 
  ENSEMBL   UCSC 
 
  337
 
  4
 
 37957
 
 tss;island
 
 0.18346
 
 0.09102
 
 Not queried
 
  0.8723594
 
       NA
 
       NA
  
 
  
 6
 
  24126060
 
  24126330
 
  ENSEMBL   UCSC 
 
  271
 
  8
 
 34601
 
 tss
 
 0.07881
 
 0.10265
 
 Not queried
 
  0.8723588
 
       NA
 
       NA
  
 
  
 5
 
  33936171
 
  33936462
 
  ENSEMBL   UCSC 
 
  292
 
 10
 
 31951
 
 tss
 
 0.02096
 
 0.03837
 
 Not queried
 
  0.8723403
 
       NA
 
       NA
  
 
  
 11
 
  27527925
 
  27528142
 
  ENSEMBL   UCSC 
 
  218
 
  4
 
 6524
 
 gene;tss;island
 
 0.02916
 
 0.07336
 
 Not queried
 
  0.8722125
 
       NA
 
       NA
  
 
  
 3
 
 137484220
 
 137484586
 
  ENSEMBL   UCSC 
 
  367
 
  4
 
 29470
 
 island
 
 0.31431
 
 0.14694
 
 Not queried
 
  0.8720510
 
       NA
 
       NA
  
 
  
 20
 
  58515340
 
  58515583
 
  ENSEMBL   UCSC 
 
  244
 
  8
 
 25823
 
 tss;island
 
 0.01355
 
 0.02282
 
 Not queried
 
  0.8720052
 
       NA
 
       NA
  
 
  
 16
 
  10674326
 
  10674468
 
  ENSEMBL   UCSC 
 
  143
 
  5
 
 14630
 
 gene
 
 0.01228
 
 0.02531
 
 Not queried
 
  0.8718495
 
       NA
 
       NA
  
 
  
 6
 
 150070697
 
 150070787
 
  ENSEMBL   UCSC 
 
   91
 
  5
 
 35224
 
 tss;island
 
 0.11645
 
 0.04246
 
 Not queried
 
  0.8718481
 
       NA
 
       NA
  
 
  
 22
 
  44568699
 
  44568812
 
  ENSEMBL   UCSC 
 
  114
 
  6
 
 27168
 
 tss
 
 0.85548
 
 0.76653
 
 Not queried
 
  0.8711128
 
       NA
 
       NA
  
 
  
 8
 
  21988724
 
  21988809
 
  ENSEMBL   UCSC 
 
   86
 
  4
 
 39517
 
 tss;island
 
 0.22453
 
 0.33977
 
 Not queried
 
  0.8710504
 
       NA
 
       NA
  
 
  
 10
 
 134971594
 
 134972063
 
  ENSEMBL   UCSC 
 
  470
 
  5
 
 6319
 
 island
 
 0.68492
 
 0.81320
 
 Not queried
 
  0.8710246
 
       NA
 
       NA
  
 
  
 17
 
  48350468
 
  48350615
 
  ENSEMBL   UCSC 
 
  148
 
  4
 
 16649
 
 gene;tss;island
 
 0.06483
 
 0.04045
 
 Not queried
 
  0.8709666
 
       NA
 
       NA
  
 
  
 12
 
  48298622
 
  48298992
 
  ENSEMBL   UCSC 
 
  371
 
  6
 
 10331
 
 island
 
 0.01469
 
 0.02726
 
 Not queried
 
  0.8708457
 
       NA
 
       NA
  
 
  
 1
 
 231297516
 
 231297737
 
  ENSEMBL   UCSC 
 
  222
 
  4
 
 2317
 
 tss;island
 
 0.03583
 
 0.02656
 
 Not queried
 
  0.8706913
 
       NA
 
       NA
  
 
  
 3
 
   2141614
 
   2141937
 
  ENSEMBL   UCSC 
 
  324
 
  4
 
 28069
 
 tss;island
 
 0.15979
 
 0.28647
 
 Not queried
 
  0.8705777
 
       NA
 
       NA
  
 
  
 12
 
  54763081
 
  54763433
 
  ENSEMBL   UCSC 
 
  353
 
  4
 
 9140
 
 gene;island
 
 0.20050
 
 0.15240
 
 Not queried
 
  0.8705633
 
       NA
 
       NA
  
 
  
 7
 
   1524917
 
   1525238
 
  ENSEMBL   UCSC 
 
  322
 
  4
 
 36694
 
 gene;island
 
 0.67418
 
 0.79434
 
 Not queried
 
  0.8705490
 
       NA
 
       NA
  
 
  
 8
 
  88886301
 
  88886432
 
  ENSEMBL   UCSC 
 
  132
 
  6
 
 39737
 
 tss
 
 0.92913
 
 0.95440
 
 Not queried
 
  0.8704514
 
       NA
 
       NA
  
 
  
 10
 
 131671265
 
 131671830
 
  ENSEMBL   UCSC 
 
  566
 
  7
 
 4817
 
 gene
 
 0.79184
 
 0.88028
 
 Not queried
 
  0.8701569
 
       NA
 
       NA
  
 
  
 2
 
  70418186
 
  70418309
 
  ENSEMBL   UCSC 
 
  124
 
  5
 
 23376
 
 tss
 
 0.02301
 
 0.04607
 
 Not queried
 
  0.8700357
 
       NA
 
       NA
  
 
  
 4
 
 154073371
 
 154074387
 
  ENSEMBL   UCSC 
 
 1017
 
 15
 
 31316
 
 island
 
 0.01168
 
 0.02442
 
 Not queried
 
  0.8700212
 
       NA
 
       NA
  
 
  
 6
 
  29342522
 
  29342824
 
  ENSEMBL   UCSC 
 
  303
 
  8
 
 33590
 
 gene
 
 0.78636
 
 0.60015
 
 Not queried
 
  0.8699028
 
       NA
 
       NA
  
 
  
 1
 
 205537752
 
 205537877
 
  ENSEMBL   UCSC 
 
  126
 
  4
 
 2202
 
 tss;island
 
 0.37036
 
 0.26509
 
 Not queried
 
  0.8698934
 
       NA
 
       NA
  
 
  
 14
 
  45603667
 
  45603864
 
  ENSEMBL   UCSC 
 
  198
 
  6
 
 12344
 
 tss
 
 0.01137
 
 0.02899
 
 Not queried
 
  0.8696197
 
       NA
 
       NA
  
 
  
 6
 
 163148718
 
 163148903
 
  ENSEMBL   UCSC 
 
  186
 
  5
 
 34474
 
 gene;tss;island
 
 0.02354
 
 0.02691
 
 Not queried
 
  0.8695931
 
       NA
 
       NA
  
 
  
 7
 
  96338924
 
  96339613
 
  ENSEMBL   UCSC 
 
  690
 
 11
 
 38622
 
 island
 
 0.04295
 
 0.04520
 
 Not queried
 
  0.8694980
 
       NA
 
       NA
  
 
  
 17
 
  16472670
 
  16472866
 
  ENSEMBL   UCSC 
 
  197
 
  7
 
 17127
 
 tss;island
 
 0.03689
 
 0.08224
 
 Not queried
 
  0.8692603
 
       NA
 
       NA
  
 
  
 13
 
 113652069
 
 113652368
 
  ENSEMBL   UCSC 
 
  300
 
  5
 
 11237
 
 gene;island
 
 0.79176
 
 0.87412
 
 Not queried
 
  0.8690728
 
       NA
 
       NA
  
 
  
 5
 
 176237221
 
 176237381
 
  ENSEMBL   UCSC 
 
  161
 
  4
 
 32458
 
 tss;island
 
 0.01058
 
 0.02858
 
 Not queried
 
  0.8689809
 
       NA
 
       NA
  
 
  
 1
 
  40506702
 
  40507006
 
  ENSEMBL   UCSC 
 
  305
 
  5
 
 449
 
 gene;island
 
 0.02078
 
 0.01484
 
 Not queried
 
  0.8688823
 
       NA
 
       NA
  
 
  
 4
 
   1642054
 
   1642257
 
  ENSEMBL   UCSC 
 
  204
 
  4
 
 29828
 
 gene;island
 
 0.77578
 
 0.77687
 
 Not queried
 
  0.8688565
 
       NA
 
       NA
  
 
  
 6
 
  30617565
 
  30617896
 
  ENSEMBL   UCSC 
 
  332
 
  4
 
 33730
 
 gene;island
 
 0.89510
 
 0.92948
 
 Not queried
 
  0.8688482
 
       NA
 
       NA
  
 
  
 11
 
  72465788
 
  72466113
 
  ENSEMBL   UCSC 
 
  326
 
  4
 
 6799
 
 gene;island
 
 0.91452
 
 0.84646
 
 Not queried
 
  0.8687230
 
       NA
 
       NA
  
 
  
 20
 
  54933521
 
  54933931
 
  ENSEMBL   UCSC 
 
  411
 
  9
 
 25794
 
 tss
 
 0.04928
 
 0.07035
 
 Not queried
 
  0.8686660
 
       NA
 
       NA
  
 
  
 19
 
  47290532
 
  47290716
 
  ENSEMBL   UCSC 
 
  185
 
  5
 
 20079
 
 gene;island
 
 0.05439
 
 0.02902
 
 Not queried
 
  0.8685939
 
       NA
 
       NA
  
 
  
 8
 
  29940777
 
  29940921
 
  ENSEMBL   UCSC 
 
  145
 
  5
 
 39565
 
 tss
 
 0.02658
 
 0.01474
 
 Not queried
 
  0.8685389
 
       NA
 
       NA
  
 
  
 17
 
  48858534
 
  48858873
 
  ENSEMBL   UCSC 
 
  340
 
  5
 
 18527
 
 island
 
 0.89682
 
 0.85547
 
 Not queried
 
  0.8685060
 
       NA
 
       NA
  
 
  
 19
 
    807147
 
    807355
 
  ENSEMBL   UCSC 
 
  209
 
  4
 
 19597
 
 gene;island
 
 0.82836
 
 0.89569
 
 Not queried
 
  0.8683639
 
       NA
 
       NA
  
 
  
 19
 
  38746538
 
  38746749
 
  ENSEMBL   UCSC 
 
  212
 
  4
 
 19977
 
 gene;island
 
 0.15190
 
 0.04102
 
 Not queried
 
  0.8682994
 
       NA
 
       NA
  
 
  
 2
 
 236826207
 
 236826485
 
  ENSEMBL   UCSC 
 
  279
 
  5
 
 23047
 
 gene
 
 0.84220
 
 0.74104
 
 Not queried
 
  0.8678067
 
       NA
 
       NA
  
 
  
 3
 
 160117310
 
 160117717
 
  ENSEMBL   UCSC 
 
  408
 
  6
 
 28649
 
 tss;island
 
 0.01332
 
 0.03819
 
 Not queried
 
  0.8677861
 
       NA
 
       NA
  
 
  
 8
 
 144965352
 
 144965577
 
  ENSEMBL   UCSC 
 
  226
 
  4
 
 40631
 
 island
 
 0.77278
 
 0.80462
 
 Not queried
 
  0.8676142
 
       NA
 
       NA
  
 
  
 6
 
 142409939
 
 142410235
 
  ENSEMBL   UCSC 
 
  297
 
  8
 
 35196
 
 tss
 
 0.03294
 
 0.05746
 
 Not queried
 
  0.8675985
 
       NA
 
       NA
  
 
  
 11
 
   2162341
 
   2162930
 
  ENSEMBL   UCSC 
 
  590
 
 17
 
 7038
 
 tss
 
 0.35350
 
 0.47608
 
 Not queried
 
  0.8675932
 
       NA
 
       NA
  
 
  
 22
 
  20004356
 
  20004382
 
  ENSEMBL   UCSC 
 
   27
 
  6
 
 26930
 
 tss;island
 
 0.01655
 
 0.02800
 
 Not queried
 
  0.8675474
 
       NA
 
       NA
  
 
  
 17
 
  42856469
 
  42856823
 
  ENSEMBL   UCSC 
 
  355
 
  4
 
 16584
 
 gene;island
 
 0.03482
 
 0.04181
 
 Not queried
 
  0.8674072
 
       NA
 
       NA
  
 
  
 1
 
  27480543
 
  27480849
 
  ENSEMBL   UCSC 
 
  307
 
  4
 
 349
 
 gene;island
 
 0.68653
 
 0.81328
 
 Not queried
 
  0.8673823
 
       NA
 
       NA
  
 
  
 6
 
  32549283
 
  32549631
 
  ENSEMBL   UCSC 
 
  349
 
  4
 
 34055
 
 gene;island
 
 0.58254
 
 0.46643
 
 Not queried
 
  0.8673602
 
       NA
 
       NA
  
 
  
 1
 
  40562564
 
  40562842
 
  ENSEMBL   UCSC 
 
  279
 
  4
 
 450
 
 gene;island
 
 0.02430
 
 0.04504
 
 Not queried
 
  0.8671057
 
       NA
 
       NA
  
 
  
 16
 
  75032477
 
  75032853
 
  ENSEMBL   UCSC 
 
  377
 
  6
 
 15327
 
 tss;island
 
 0.05646
 
 0.03291
 
 Not queried
 
  0.8669444
 
       NA
 
       NA
  
 
  
 7
 
 157956042
 
 157956161
 
  ENSEMBL   UCSC 
 
  120
 
  4
 
 37296
 
 gene;island
 
 0.75624
 
 0.81352
 
 Not queried
 
  0.8668366
 
       NA
 
       NA
  
 
  
 5
 
 180622072
 
 180622643
 
  ENSEMBL   UCSC 
 
  572
 
  5
 
 31891
 
 gene;island
 
 0.80927
 
 0.69940
 
 Not queried
 
  0.8667933
 
       NA
 
       NA
  
 
  
 6
 
  72595944
 
  72596566
 
  ENSEMBL   UCSC 
 
  623
 
  8
 
 34998
 
 tss
 
 0.02726
 
 0.02890
 
 Not queried
 
  0.8667676
 
       NA
 
       NA
  
 
  
 6
 
  10422139
 
  10422636
 
  ENSEMBL   UCSC 
 
  498
 
  5
 
 35370
 
 island
 
 0.96481
 
 0.83697
 
 Not queried
 
  0.8667213
 
       NA
 
       NA
  
 
  
 2
 
  63274857
 
  63275178
 
  ENSEMBL   UCSC 
 
  322
 
  4
 
 23346
 
 tss
 
 0.02320
 
 0.05715
 
 Not queried
 
  0.8666527
 
       NA
 
       NA
  
 
  
 5
 
 137514240
 
 137514385
 
  ENSEMBL   UCSC 
 
  146
 
  6
 
 32255
 
 tss;island
 
 0.02037
 
 0.03651
 
 Not queried
 
  0.8666494
 
       NA
 
       NA
  
 
  
 14
 
  35761165
 
  35761922
 
  ENSEMBL   UCSC 
 
  758
 
 10
 
 12751
 
 island
 
 0.01283
 
 0.02279
 
 Not queried
 
  0.8666191
 
       NA
 
       NA
  
 
  
 10
 
 103599617
 
 103599757
 
  ENSEMBL   UCSC 
 
  141
 
  5
 
 5294
 
 tss
 
 0.02323
 
 0.04506
 
 Not queried
 
  0.8665884
 
       NA
 
       NA
  
 
  
 3
 
 160822268
 
 160822911
 
  ENSEMBL   UCSC 
 
  644
 
  8
 
 27975
 
 gene;island
 
 0.02994
 
 0.04976
 
 Not queried
 
  0.8664444
 
       NA
 
       NA
  
 
  
 3
 
 160822711
 
 160822911
 
  ENSEMBL   UCSC 
 
  201
 
  4
 
 28653
 
 tss
 
 0.02994
 
 0.04976
 
 Not queried
 
  0.8664444
 
       NA
 
       NA
  
 
  
 2
 
  85109273
 
  85109418
 
  ENSEMBL   UCSC 
 
  146
 
  4
 
 23439
 
 tss;island
 
 0.01898
 
 0.01468
 
 Not queried
 
  0.8663372
 
       NA
 
       NA
  
 
  
 14
 
  33403060
 
  33403226
 
  ENSEMBL   UCSC 
 
  167
 
  4
 
 12743
 
 island
 
 0.02803
 
 0.06506
 
 Not queried
 
  0.8663053
 
       NA
 
       NA
  
 
  
 4
 
  57844912
 
  57845134
 
  ENSEMBL   UCSC 
 
  223
 
  7
 
 30368
 
 tss;island
 
 0.03796
 
 0.03323
 
 Not queried
 
  0.8662703
 
       NA
 
       NA
  
 
  
 4
 
 101111147
 
 101111927
 
  ENSEMBL   UCSC 
 
  781
 
  9
 
 31149
 
 island
 
 0.01115
 
 0.04106
 
 Not queried
 
  0.8661674
 
       NA
 
       NA
  
 
  
 1
 
  37939724
 
  37940113
 
  ENSEMBL   UCSC 
 
  390
 
  8
 
 1497
 
 tss
 
 0.01546
 
 0.02900
 
 Not queried
 
  0.8660318
 
       NA
 
       NA
  
 
  
 1
 
  53558066
 
  53558596
 
  ENSEMBL   UCSC 
 
  531
 
  6
 
 535
 
 gene;island
 
 0.69606
 
 0.80291
 
 Not queried
 
  0.8657912
 
       NA
 
       NA
  
 
  
 6
 
  33390907
 
  33391341
 
  ENSEMBL   UCSC 
 
  435
 
  9
 
 34219
 
 gene;island
 
 0.74531
 
 0.79974
 
 Not queried
 
  0.8656965
 
       NA
 
       NA
  
 
  
 11
 
  67141628
 
  67142030
 
  ENSEMBL   UCSC 
 
  403
 
  8
 
 6731
 
 gene;tss;island
 
 0.07119
 
 0.05793
 
 Not queried
 
  0.8656435
 
       NA
 
       NA
  
 
  
 22
 
  23484241
 
  23484600
 
  ENSEMBL   UCSC 
 
  360
 
  9
 
 26965
 
 tss
 
 0.02003
 
 0.03915
 
 Not queried
 
  0.8655674
 
       NA
 
       NA
  
 
  
 3
 
 185911519
 
 185911885
 
  ENSEMBL   UCSC 
 
  367
 
  4
 
 28029
 
 gene;island
 
 0.02399
 
 0.06621
 
 Not queried
 
  0.8654075
 
       NA
 
       NA
  
 
  
 15
 
  99789622
 
  99789855
 
  ENSEMBL   UCSC 
 
  234
 
  5
 
 14475
 
 island
 
 0.03212
 
 0.01789
 
 Not queried
 
  0.8651970
 
       NA
 
       NA
  
 
  
 19
 
   6502101
 
   6502304
 
  ENSEMBL   UCSC 
 
  204
 
  4
 
 19730
 
 gene
 
 0.03883
 
 0.06573
 
 Not queried
 
  0.8650458
 
       NA
 
       NA
  
 
  
 1
 
 101491408
 
 101491652
 
  ENSEMBL   UCSC 
 
  245
 
  7
 
 1779
 
 tss
 
 0.01518
 
 0.02080
 
 Not queried
 
  0.8650423
 
       NA
 
       NA
  
 
  
 12
 
 111358446
 
 111358639
 
  ENSEMBL   UCSC 
 
  194
 
  7
 
 9987
 
 tss
 
 0.73561
 
 0.84905
 
 Not queried
 
  0.8649774
 
       NA
 
       NA
  
 
  
 20
 
  62601556
 
  62601743
 
  ENSEMBL   UCSC 
 
  188
 
  4
 
 25868
 
 tss;island
 
 0.10791
 
 0.06479
 
 Not queried
 
  0.8649747
 
       NA
 
       NA
  
 
  
 1
 
  45243311
 
  45243518
 
  ENSEMBL   UCSC 
 
  208
 
  4
 
 496
 
 gene;tss;island
 
 0.82150
 
 0.65056
 
 Not queried
 
  0.8649253
 
       NA
 
       NA
  
 
  
 3
 
  10206254
 
  10206537
 
  ENSEMBL   UCSC 
 
  284
 
  7
 
 28102
 
 tss
 
 0.02961
 
 0.04785
 
 Not queried
 
  0.8648691
 
       NA
 
       NA
  
 
  
 10
 
 133850441
 
 133850760
 
  ENSEMBL   UCSC 
 
  320
 
  5
 
 6265
 
 island
 
 0.05853
 
 0.03477
 
 Not queried
 
  0.8648626
 
       NA
 
       NA
  
 
  
 1
 
 234614967
 
 234615370
 
  ENSEMBL   UCSC 
 
  404
 
  6
 
 2325
 
 tss
 
 0.03494
 
 0.05455
 
 Not queried
 
  0.8647618
 
       NA
 
       NA
  
 
  
 1
 
 234614614
 
 234615370
 
  ENSEMBL   UCSC 
 
  757
 
 10
 
 4325
 
 island
 
 0.01482
 
 0.02475
 
 Not queried
 
  0.8647618
 
       NA
 
       NA
  
 
  
 3
 
 155524173
 
 155524344
 
  ENSEMBL   UCSC 
 
  172
 
  5
 
 28629
 
 tss;island
 
 0.02611
 
 0.02677
 
 Not queried
 
  0.8643521
 
       NA
 
       NA
  
 
  
 16
 
  22926347
 
  22926625
 
  ENSEMBL   UCSC 
 
  279
 
  4
 
 14661
 
 gene;island
 
 0.55503
 
 0.73862
 
 Not queried
 
  0.8638858
 
       NA
 
       NA
  
 
  
 3
 
 138725153
 
 138725425
 
  ENSEMBL   UCSC 
 
  273
 
  7
 
 28584
 
 tss;island
 
 0.96086
 
 0.91110
 
 Not queried
 
  0.8637113
 
       NA
 
       NA
  
 
  
 6
 
  30457039
 
  30458237
 
  ENSEMBL   UCSC 
 
 1199
 
 41
 
 35623
 
 island
 
 0.03150
 
 0.05028
 
 Not queried
 
  0.8635343
 
       NA
 
       NA
  
 
  
 10
 
  75531971
 
  75531995
 
  ENSEMBL   UCSC 
 
   25
 
  5
 
 5146
 
 tss
 
 0.08344
 
 0.07436
 
 Not queried
 
  0.8633942
 
       NA
 
       NA
  
 
  
 1
 
  42384310
 
  42384474
 
  ENSEMBL   UCSC 
 
  165
 
  4
 
 462
 
 gene
 
 0.01766
 
 0.05267
 
 Not queried
 
  0.8633324
 
       NA
 
       NA
  
 
  
 18
 
  21017489
 
  21017974
 
  ENSEMBL   UCSC 
 
  486
 
  6
 
 19393
 
 island
 
 0.01140
 
 0.01869
 
 Not queried
 
  0.8632999
 
       NA
 
       NA
  
 
  
 4
 
  71570323
 
  71570509
 
  ENSEMBL   UCSC 
 
  187
 
  4
 
 30375
 
 tss;island
 
 0.04488
 
 0.06677
 
 Not queried
 
  0.8631471
 
       NA
 
       NA
  
 
  
 1
 
 145382340
 
 145382734
 
  ENSEMBL   UCSC 
 
  395
 
  4
 
 3666
 
 island
 
 0.05536
 
 0.20557
 
 Not queried
 
  0.8630845
 
       NA
 
       NA
  
 
  
 3
 
  12330263
 
  12330532
 
  ENSEMBL   UCSC 
 
  270
 
  4
 
 27630
 
 gene;island
 
 0.03180
 
 0.05444
 
 Not queried
 
  0.8630338
 
       NA
 
       NA
  
 
  
 6
 
  32165877
 
  32165917
 
  ENSEMBL   UCSC 
 
   41
 
  4
 
 34022
 
 gene;island
 
 0.79536
 
 0.85893
 
 Not queried
 
  0.8629858
 
       NA
 
       NA
  
 
  
 16
 
  11267859
 
  11268374
 
  ENSEMBL   UCSC 
 
  516
 
  5
 
 14633
 
 gene;island
 
 0.94401
 
 0.90262
 
 Not queried
 
  0.8629748
 
       NA
 
       NA
  
 
  
 2
 
  73114863
 
  73115011
 
  ENSEMBL   UCSC 
 
  149
 
  4
 
 22696
 
 gene;island
 
 0.01061
 
 0.02313
 
 Not queried
 
  0.8628560
 
       NA
 
       NA
  
 
  
 6
 
  32178603
 
  32178775
 
  ENSEMBL   UCSC 
 
  173
 
  4
 
 34029
 
 gene
 
 0.71694
 
 0.59620
 
 Not queried
 
  0.8627110
 
       NA
 
       NA
  
 
  
 7
 
  17338213
 
  17338260
 
  ENSEMBL   UCSC 
 
   48
 
  4
 
 37391
 
 tss
 
 0.06099
 
 0.14039
 
 Not queried
 
  0.8626961
 
       NA
 
       NA
  
 
  
 2
 
  26986235
 
  26986550
 
  ENSEMBL   UCSC 
 
  316
 
  5
 
 23207
 
 tss;island
 
 0.93762
 
 0.87424
 
 Not queried
 
  0.8625303
 
       NA
 
       NA
  
 
  
 5
 
  82770250
 
  82770619
 
  ENSEMBL   UCSC 
 
  370
 
  4
 
 31613
 
 gene;island
 
 0.01862
 
 0.02964
 
 Not queried
 
  0.8625233
 
       NA
 
       NA
  
 
  
 17
 
  46810540
 
  46810934
 
  ENSEMBL   UCSC 
 
  395
 
  6
 
 18471
 
 island
 
 0.02553
 
 0.05379
 
 Not queried
 
  0.8623716
 
       NA
 
       NA
  
 
  
 7
 
 149569715
 
 149570367
 
  ENSEMBL   UCSC 
 
  653
 
 13
 
 37205
 
 gene;island
 
 0.15784
 
 0.07777
 
 Not queried
 
  0.8623146
 
       NA
 
       NA
  
 
  
 6
 
  39760416
 
  39760776
 
  ENSEMBL   UCSC 
 
  361
 
  4
 
 34895
 
 tss
 
 0.37115
 
 0.31351
 
 Not queried
 
  0.8622664
 
       NA
 
       NA
  
 
  
 5
 
 159826822
 
 159827503
 
  ENSEMBL   UCSC 
 
  682
 
  7
 
 33296
 
 island
 
 0.02505
 
 0.02760
 
 Not queried
 
  0.8622423
 
       NA
 
       NA
  
 
  
 10
 
 124220359
 
 124221066
 
  ENSEMBL   UCSC 
 
  708
 
 12
 
 6189
 
 island
 
 0.12393
 
 0.05865
 
 Not queried
 
  0.8621965
 
       NA
 
       NA
  
 
  
 3
 
 128997295
 
 128997702
 
  ENSEMBL   UCSC 
 
  408
 
  8
 
 28538
 
 tss;island
 
 0.04815
 
 0.10661
 
 Not queried
 
  0.8621279
 
       NA
 
       NA
  
 
  
 21
 
  44515263
 
  44515676
 
  ENSEMBL   UCSC 
 
  414
 
  4
 
 26403
 
 gene;island
 
 0.85596
 
 0.94420
 
 Not queried
 
  0.8619833
 
       NA
 
       NA
  
 
  
 19
 
   9609335
 
   9609704
 
  ENSEMBL   UCSC 
 
  370
 
  8
 
 20389
 
 tss
 
 0.78835
 
 0.86238
 
 Not queried
 
  0.8619552
 
       NA
 
       NA
  
 
  
 19
 
   9609152
 
   9609704
 
  ENSEMBL   UCSC 
 
  553
 
 10
 
 21463
 
 island
 
 0.77299
 
 0.80515
 
 Not queried
 
  0.8619552
 
       NA
 
       NA
  
 
  
 8
 
  87526527
 
  87526650
 
  ENSEMBL   UCSC 
 
  124
 
  5
 
 39736
 
 tss
 
 0.03802
 
 0.04268
 
 Not queried
 
  0.8619418
 
       NA
 
       NA
  
 
  
 15
 
  49462118
 
  49462660
 
  ENSEMBL   UCSC 
 
  543
 
  9
 
 13313
 
 gene
 
 0.03589
 
 0.02869
 
 Not queried
 
  0.8618638
 
       NA
 
       NA
  
 
  
 15
 
  49462118
 
  49462405
 
  ENSEMBL   UCSC 
 
  288
 
  7
 
 13635
 
 tss
 
 0.03589
 
 0.02869
 
 Not queried
 
  0.8618638
 
       NA
 
       NA
  
 
  
 13
 
  37633916
 
  37634133
 
  ENSEMBL   UCSC 
 
  218
 
  6
 
 11372
 
 tss
 
 0.03994
 
 0.01233
 
 Not queried
 
  0.8615977
 
       NA
 
       NA
  
 
  
 10
 
  45454796
 
  45455141
 
  ENSEMBL   UCSC 
 
  346
 
  8
 
 5039
 
 tss
 
 0.01596
 
 0.02571
 
 Not queried
 
  0.8614789
 
       NA
 
       NA
  
 
  
 19
 
   3464765
 
   3465071
 
  ENSEMBL   UCSC 
 
  307
 
  5
 
 21292
 
 island
 
 0.74149
 
 0.87666
 
 Not queried
 
  0.8613985
 
       NA
 
       NA
  
 
  
 1
 
 167599371
 
 167599830
 
  ENSEMBL   UCSC 
 
  460
 
  5
 
 3935
 
 island
 
 0.02157
 
 0.03850
 
 Not queried
 
  0.8613302
 
       NA
 
       NA
  
 
  
 11
 
  10589810
 
  10590282
 
  ENSEMBL   UCSC 
 
  473
 
  4
 
 6490
 
 gene
 
 0.88170
 
 0.94372
 
 Not queried
 
  0.8613189
 
       NA
 
       NA
  
 
  
 6
 
 168613133
 
 168613387
 
  ENSEMBL   UCSC 
 
  255
 
  4
 
 36598
 
 island
 
 0.63779
 
 0.81057
 
 Not queried
 
  0.8613081
 
       NA
 
       NA
  
 
  
 1
 
  27481531
 
  27481857
 
  ENSEMBL   UCSC 
 
  327
 
  6
 
 1401
 
 tss;island
 
 0.02802
 
 0.07356
 
 Not queried
 
  0.8612827
 
       NA
 
       NA
  
 
  
 9
 
 108210605
 
 108210846
 
  ENSEMBL   UCSC 
 
  242
 
  4
 
 40713
 
 gene;island
 
 0.01832
 
 0.03004
 
 Not queried
 
  0.8609460
 
       NA
 
       NA
  
 
  
 8
 
 143915776
 
 143916206
 
  ENSEMBL   UCSC 
 
  431
 
  7
 
 39877
 
 tss
 
 0.86589
 
 0.80403
 
 Not queried
 
  0.8609431
 
       NA
 
       NA
  
 
  
 19
 
   4531638
 
   4531961
 
  ENSEMBL   UCSC 
 
  324
 
  4
 
 19700
 
 gene;island
 
 0.68355
 
 0.55234
 
 Not queried
 
  0.8608949
 
       NA
 
       NA
  
 
  
 10
 
  76859248
 
  76859441
 
  ENSEMBL   UCSC 
 
  194
 
  6
 
 5154
 
 tss
 
 0.66849
 
 0.81134
 
 Not queried
 
  0.8608460
 
       NA
 
       NA
  
 
  
 12
 
 111180856
 
 111181027
 
  ENSEMBL   UCSC 
 
  172
 
  6
 
 9984
 
 tss
 
 0.01542
 
 0.01369
 
 Not queried
 
  0.8608073
 
       NA
 
       NA
  
 
  
 19
 
  40895469
 
  40895711
 
  ENSEMBL   UCSC 
 
  243
 
  4
 
 20005
 
 gene;island
 
 0.92479
 
 0.87252
 
 Not queried
 
  0.8605751
 
       NA
 
       NA
  
 
  
 X
 
  68385233
 
  68385467
 
  ENSEMBL   UCSC 
 
  235
 
  5
 
 41840
 
 island
 
 0.04857
 
 0.02211
 
 Not queried
 
  0.8601395
 
       NA
 
       NA
  
 
  
 1
 
  19639298
 
  19639494
 
  ENSEMBL   UCSC 
 
  197
 
  4
 
 281
 
 gene;tss;island
 
 0.14599
 
 0.11204
 
 Not queried
 
  0.8598589
 
       NA
 
       NA
  
 
  
 7
 
 150745278
 
 150745709
 
  ENSEMBL   UCSC 
 
  432
 
  7
 
 38958
 
 island
 
 0.83598
 
 0.72862
 
 Not queried
 
  0.8594497
 
       NA
 
       NA
  
 
  
 2
 
 233562332
 
 233562546
 
  ENSEMBL   UCSC 
 
  215
 
  4
 
 23032
 
 gene;island
 
 0.01983
 
 0.03657
 
 Not queried
 
  0.8594295
 
       NA
 
       NA
  
 
  
 2
 
  17935206
 
  17935227
 
  ENSEMBL   UCSC 
 
   22
 
  4
 
 22546
 
 gene;tss;island
 
 0.00980
 
 0.02073
 
 Not queried
 
  0.8593082
 
       NA
 
       NA
  
 
  
 6
 
  30876795
 
  30876992
 
  ENSEMBL   UCSC 
 
  198
 
  4
 
 33769
 
 gene;island
 
 0.34882
 
 0.44552
 
 Not queried
 
  0.8592159
 
       NA
 
       NA
  
 
  
 12
 
 133414255
 
 133414571
 
  ENSEMBL   UCSC 
 
  317
 
  5
 
 11052
 
 island
 
 0.80272
 
 0.86055
 
 Not queried
 
  0.8591503
 
       NA
 
       NA
  
 
  
 7
 
  25893749
 
  25894005
 
  ENSEMBL   UCSC 
 
  257
 
  4
 
 38259
 
 island
 
 0.28625
 
 0.18977
 
 Not queried
 
  0.8590510
 
       NA
 
       NA
  
 
  
 8
 
 102217569
 
 102217924
 
  ENSEMBL   UCSC 
 
  356
 
  7
 
 39320
 
 gene
 
 0.03589
 
 0.11254
 
 Not queried
 
  0.8589365
 
       NA
 
       NA
  
 
  
 2
 
  25016078
 
  25016199
 
  ENSEMBL   UCSC 
 
  122
 
  5
 
 22562
 
 gene
 
 0.01446
 
 0.02593
 
 Not queried
 
  0.8588061
 
       NA
 
       NA
  
 
  
 17
 
   7405230
 
   7405314
 
  ENSEMBL   UCSC 
 
   85
 
  4
 
 16364
 
 gene
 
 0.92912
 
 0.95860
 
 Not queried
 
  0.8587948
 
       NA
 
       NA
  
 
  
 3
 
 138665654
 
 138665957
 
  ENSEMBL   UCSC 
 
  304
 
  4
 
 27924
 
 gene
 
 0.05453
 
 0.11314
 
 Not queried
 
  0.8587678
 
       NA
 
       NA
  
 
  
 17
 
    291017
 
    291251
 
  ENSEMBL   UCSC 
 
  235
 
  4
 
 16257
 
 gene;island
 
 0.87649
 
 0.80116
 
 Not queried
 
  0.8587496
 
       NA
 
       NA
  
 
  
 12
 
 126018024
 
 126018364
 
  ENSEMBL   UCSC 
 
  341
 
  5
 
 9378
 
 gene;island
 
 0.01315
 
 0.03880
 
 Not queried
 
  0.8587360
 
       NA
 
       NA
  
 
  
 7
 
   2419958
 
   2420339
 
  ENSEMBL   UCSC 
 
  382
 
  4
 
 36739
 
 gene;island
 
 0.85418
 
 0.90394
 
 Not queried
 
  0.8586968
 
       NA
 
       NA
  
 
  
 X
 
  16804211
 
  16804636
 
  ENSEMBL   UCSC 
 
  426
 
  8
 
 41663
 
 island
 
 0.01479
 
 0.02917
 
 Not queried
 
  0.8586328
 
       NA
 
       NA
  
 
  
 7
 
  65215780
 
  65215969
 
  ENSEMBL   UCSC 
 
  190
 
  6
 
 37554
 
 tss;island
 
 0.02658
 
 0.03550
 
 Not queried
 
  0.8586029
 
       NA
 
       NA
  
 
  
 11
 
  70842116
 
  70842453
 
  ENSEMBL   UCSC 
 
  338
 
  4
 
 6780
 
 gene
 
 0.91686
 
 0.87623
 
 Not queried
 
  0.8583256
 
       NA
 
       NA
  
 
  
 12
 
  96429111
 
  96429508
 
  ENSEMBL   UCSC 
 
  398
 
  4
 
 10669
 
 island
 
 0.03569
 
 0.05190
 
 Not queried
 
  0.8581644
 
       NA
 
       NA
  
 
  
 11
 
  27743258
 
  27743664
 
  ENSEMBL   UCSC 
 
  407
 
  8
 
 7195
 
 tss;island
 
 0.09353
 
 0.07079
 
 Not queried
 
  0.8580893
 
       NA
 
       NA
  
 
  
 10
 
  90751198
 
  90751269
 
  ENSEMBL   UCSC 
 
   72
 
  5
 
 5193
 
 tss
 
 0.05236
 
 0.11111
 
 Not queried
 
  0.8579899
 
       NA
 
       NA
  
 
  
 11
 
 125439445
 
 125439882
 
  ENSEMBL   UCSC 
 
  438
 
  4
 
 6939
 
 gene
 
 0.03751
 
 0.02050
 
 Not queried
 
  0.8579774
 
       NA
 
       NA
  
 
  
 17
 
  30814514
 
  30814814
 
  ENSEMBL   UCSC 
 
  301
 
  5
 
 16477
 
 gene;island
 
 0.06227
 
 0.03560
 
 Not queried
 
  0.8579443
 
       NA
 
       NA
  
 
  
 10
 
 134672412
 
 134672917
 
  ENSEMBL   UCSC 
 
  506
 
  8
 
 6295
 
 island
 
 0.88120
 
 0.75427
 
 Not queried
 
  0.8578473
 
       NA
 
       NA
  
 
  
 19
 
  13135514
 
  13135808
 
  ENSEMBL   UCSC 
 
  295
 
  4
 
 19841
 
 gene;island
 
 0.25193
 
 0.31029
 
 Not queried
 
  0.8577869
 
       NA
 
       NA
  
 
  
 22
 
  30793027
 
  30793192
 
  ENSEMBL   UCSC 
 
  166
 
  4
 
 26796
 
 gene
 
 0.01992
 
 0.02450
 
 Not queried
 
  0.8577066
 
       NA
 
       NA
  
 
  
 11
 
  70672740
 
  70672986
 
  ENSEMBL   UCSC 
 
  247
 
  7
 
 6779
 
 gene;island
 
 0.87280
 
 0.79744
 
 Not queried
 
  0.8575531
 
       NA
 
       NA
  
 
  
 11
 
  63871228
 
  63871343
 
  ENSEMBL   UCSC 
 
  116
 
  5
 
 6645
 
 gene;tss
 
 0.57495
 
 0.71004
 
 Not queried
 
  0.8575341
 
       NA
 
       NA
  
 
  
 1
 
 223317364
 
 223317548
 
  ENSEMBL   UCSC 
 
  185
 
  4
 
 2266
 
 tss;island
 
 0.65410
 
 0.52380
 
 Not queried
 
  0.8575062
 
       NA
 
       NA
  
 
  
 12
 
  54694795
 
  54695036
 
  ENSEMBL   UCSC 
 
  242
 
  5
 
 9736
 
 tss
 
 0.02343
 
 0.03910
 
 Not queried
 
  0.8574399
 
       NA
 
       NA
  
 
  
 3
 
  15642818
 
  15643125
 
  ENSEMBL   UCSC 
 
  308
 
  8
 
 27651
 
 gene
 
 0.02391
 
 0.03179
 
 Not queried
 
  0.8573115
 
       NA
 
       NA
  
 
  
 10
 
  95256104
 
  95256359
 
  ENSEMBL   UCSC 
 
  256
 
  7
 
 5220
 
 tss
 
 0.02068
 
 0.03239
 
 Not queried
 
  0.8571238
 
       NA
 
       NA
  
 
  
 10
 
 131761150
 
 131761386
 
  ENSEMBL   UCSC 
 
  237
 
  6
 
 4823
 
 gene;island
 
 0.08234
 
 0.04507
 
 Not queried
 
  0.8569802
 
       NA
 
       NA
  
 
  
 11
 
  93394605
 
  93394910
 
  ENSEMBL   UCSC 
 
  306
 
 10
 
 8705
 
 island
 
 0.02449
 
 0.01688
 
 Not queried
 
  0.8569778
 
       NA
 
       NA
  
 
  
 6
 
  31539973
 
  31540051
 
  ENSEMBL   UCSC 
 
   79
 
  7
 
 34754
 
 tss
 
 0.93994
 
 0.88911
 
 Not queried
 
  0.8567419
 
       NA
 
       NA
  
 
  
 11
 
  19734490
 
  19735068
 
  ENSEMBL   UCSC 
 
  579
 
  8
 
 6516
 
 gene;island
 
 0.03614
 
 0.04444
 
 Not queried
 
  0.8566299
 
       NA
 
       NA
  
 
  
 16
 
  21311968
 
  21312276
 
  ENSEMBL   UCSC 
 
  309
 
  4
 
 14660
 
 gene;island
 
 0.01257
 
 0.03464
 
 Not queried
 
  0.8562510
 
       NA
 
       NA
  
 
  
 10
 
  98740656
 
  98741020
 
  ENSEMBL   UCSC 
 
  365
 
  8
 
 5239
 
 tss
 
 0.86623
 
 0.93246
 
 Not queried
 
  0.8562269
 
       NA
 
       NA
  
 
  
 3
 
  59035660
 
  59036158
 
  ENSEMBL   UCSC 
 
  499
 
  6
 
 29196
 
 island
 
 0.04968
 
 0.06757
 
 Not queried
 
  0.8561928
 
       NA
 
       NA
  
 
  
 1
 
 173836636
 
 173837071
 
  ENSEMBL   UCSC 
 
  436
 
  8
 
 888
 
 gene
 
 0.01830
 
 0.03175
 
 Not queried
 
  0.8561552
 
       NA
 
       NA
  
 
  
 2
 
  74699836
 
  74700469
 
  ENSEMBL   UCSC 
 
  634
 
  8
 
 22703
 
 gene;island
 
 0.05200
 
 0.02578
 
 Not queried
 
  0.8560612
 
       NA
 
       NA
  
 
  
 17
 
  80045966
 
  80046302
 
  ENSEMBL   UCSC 
 
  337
 
  4
 
 16904
 
 gene;island
 
 0.81523
 
 0.84309
 
 Not queried
 
  0.8560398
 
       NA
 
       NA
  
 
  
 19
 
  38085706
 
  38085810
 
  ENSEMBL   UCSC 
 
  105
 
  6
 
 19973
 
 gene;tss;island
 
 0.00919
 
 0.01879
 
 Not queried
 
  0.8560327
 
       NA
 
       NA
  
 
  
 19
 
  17982599
 
  17982930
 
  ENSEMBL   UCSC 
 
  332
 
  9
 
 21724
 
 island
 
 0.38608
 
 0.24471
 
 Not queried
 
  0.8558588
 
       NA
 
       NA
  
 
  
 15
 
  73735098
 
  73735694
 
  ENSEMBL   UCSC 
 
  597
 
 12
 
 14261
 
 island
 
 0.91031
 
 0.83152
 
 Not queried
 
  0.8557061
 
       NA
 
       NA
  
 
  
 7
 
    631862
 
    632031
 
  ENSEMBL   UCSC 
 
  170
 
  4
 
 36651
 
 gene;island
 
 0.82654
 
 0.90236
 
 Not queried
 
  0.8556978
 
       NA
 
       NA
  
 
  
 17
 
  21416258
 
  21416410
 
  ENSEMBL   UCSC 
 
  153
 
  4
 
 18091
 
 island
 
 0.12830
 
 0.07054
 
 Not queried
 
  0.8556353
 
       NA
 
       NA
  
 
  
 6
 
  33255172
 
  33255784
 
  ENSEMBL   UCSC 
 
  613
 
 16
 
 34185
 
 gene;island
 
 0.82462
 
 0.90802
 
 Not queried
 
  0.8556110
 
       NA
 
       NA
  
 
  
 13
 
  26625273
 
  26625396
 
  ENSEMBL   UCSC 
 
  124
 
  6
 
 11324
 
 tss
 
 0.05538
 
 0.08046
 
 Not queried
 
  0.8553152
 
       NA
 
       NA
  
 
  
 2
 
  63275868
 
  63276268
 
  ENSEMBL   UCSC 
 
  401
 
  6
 
 23347
 
 tss;island
 
 0.02553
 
 0.04715
 
 Not queried
 
  0.8552646
 
       NA
 
       NA
  
 
  
 19
 
   7746796
 
   7747220
 
  ENSEMBL   UCSC 
 
  425
 
  4
 
 19750
 
 gene;island
 
 0.83721
 
 0.74571
 
 Not queried
 
  0.8552434
 
       NA
 
       NA
  
 
  
 7
 
 130129946
 
 130131931
 
  ENSEMBL   UCSC 
 
 1986
 
 47
 
 37161
 
 gene;tss;island
 
 0.01908
 
 0.06221
 
 Not queried
 
  0.8551724
 
       NA
 
       NA
  
 
  
 19
 
  53758233
 
  53758309
 
  ENSEMBL   UCSC 
 
   77
 
  4
 
 20990
 
 tss
 
 0.01752
 
 0.05192
 
 Not queried
 
  0.8550186
 
       NA
 
       NA
  
 
  
 11
 
 111895234
 
 111895485
 
  ENSEMBL   UCSC 
 
  252
 
  6
 
 7674
 
 tss
 
 0.04760
 
 0.02827
 
 Not queried
 
  0.8550171
 
       NA
 
       NA
  
 
  
 13
 
  31479935
 
  31480335
 
  ENSEMBL   UCSC 
 
  401
 
  7
 
 11622
 
 island
 
 0.60171
 
 0.69514
 
 Not queried
 
  0.8547442
 
       NA
 
       NA
  
 
  
 13
 
 113464041
 
 113464532
 
  ENSEMBL   UCSC 
 
  492
 
  6
 
 11227
 
 gene;island
 
 0.93778
 
 0.95037
 
 Not queried
 
  0.8547279
 
       NA
 
       NA
  
 
  
 20
 
  25129126
 
  25129400
 
  ENSEMBL   UCSC 
 
  275
 
  4
 
 25340
 
 gene
 
 0.76123
 
 0.83934
 
 Not queried
 
  0.8546929
 
       NA
 
       NA
  
 
  
 12
 
   4382980
 
   4383281
 
  ENSEMBL   UCSC 
 
  302
 
  5
 
 8987
 
 gene
 
 0.01407
 
 0.02324
 
 Not queried
 
  0.8544436
 
       NA
 
       NA
  
 
  
 1
 
 153606037
 
 153606943
 
  ENSEMBL   UCSC 
 
  907
 
 13
 
 762
 
 gene;island
 
 0.01739
 
 0.02946
 
 Not queried
 
  0.8543353
 
       NA
 
       NA
  
 
  
 6
 
  43612346
 
  43612764
 
  ENSEMBL   UCSC 
 
  419
 
  6
 
 34940
 
 tss
 
 0.15781
 
 0.11036
 
 Not queried
 
  0.8542561
 
       NA
 
       NA
  
 
  
 12
 
  54402697
 
  54402909
 
  ENSEMBL   UCSC 
 
  213
 
  7
 
 10465
 
 island
 
 0.01175
 
 0.02842
 
 Not queried
 
  0.8542298
 
       NA
 
       NA
  
 
  
 10
 
 130339134
 
 130339927
 
  ENSEMBL   UCSC 
 
  794
 
 12
 
 6237
 
 island
 
 0.04137
 
 0.06914
 
 Not queried
 
  0.8541511
 
       NA
 
       NA
  
 
  
 20
 
  33759605
 
  33759753
 
  ENSEMBL   UCSC 
 
  149
 
  4
 
 25663
 
 tss
 
 0.21997
 
 0.12862
 
 Not queried
 
  0.8539995
 
       NA
 
       NA
  
 
  
 1
 
  46598380
 
  46598507
 
  ENSEMBL   UCSC 
 
  128
 
  5
 
 1589
 
 tss
 
 0.02277
 
 0.04041
 
 Not queried
 
  0.8538584
 
       NA
 
       NA
  
 
  
 5
 
  75013131
 
  75013238
 
  ENSEMBL   UCSC 
 
  108
 
  4
 
 31592
 
 gene
 
 0.02750
 
 0.04772
 
 Not queried
 
  0.8537693
 
       NA
 
       NA
  
 
  
 19
 
   4769146
 
   4769240
 
  ENSEMBL   UCSC 
 
   95
 
  4
 
 19703
 
 gene
 
 0.29373
 
 0.46484
 
 Not queried
 
  0.8537510
 
       NA
 
       NA
  
 
  
 3
 
 124931535
 
 124931746
 
  ENSEMBL   UCSC 
 
  212
 
  5
 
 29376
 
 island
 
 0.15713
 
 0.05916
 
 Not queried
 
  0.8534926
 
       NA
 
       NA
  
 
  
 12
 
  51632284
 
  51632444
 
  ENSEMBL   UCSC 
 
  161
 
  7
 
 9689
 
 tss;island
 
 0.01039
 
 0.03028
 
 Not queried
 
  0.8532727
 
       NA
 
       NA
  
 
  
 1
 
  40137913
 
  40138052
 
  ENSEMBL   UCSC 
 
  140
 
  4
 
 1522
 
 tss;island
 
 0.21330
 
 0.10807
 
 Not queried
 
  0.8531719
 
       NA
 
       NA
  
 
  
 2
 
  99347854
 
  99348232
 
  ENSEMBL   UCSC 
 
  379
 
  5
 
 23500
 
 tss;island
 
 0.05505
 
 0.03784
 
 Not queried
 
  0.8528047
 
       NA
 
       NA
  
 
  
 7
 
   2272667
 
   2272828
 
  ENSEMBL   UCSC 
 
  162
 
  4
 
 37341
 
 tss
 
 0.00931
 
 0.02070
 
 Not queried
 
  0.8524307
 
       NA
 
       NA
  
 
  
 7
 
   2272392
 
   2272828
 
  ENSEMBL   UCSC 
 
  437
 
  6
 
 38085
 
 island
 
 0.00931
 
 0.01392
 
 Not queried
 
  0.8524307
 
       NA
 
       NA
  
 
  
 19
 
  22816896
 
  22817096
 
  ENSEMBL   UCSC 
 
  201
 
  6
 
 20628
 
 tss
 
 0.02793
 
 0.07043
 
 Not queried
 
  0.8522931
 
       NA
 
       NA
  
 
  
 19
 
  22816896
 
  22817496
 
  ENSEMBL   UCSC 
 
  601
 
 10
 
 21808
 
 island
 
 0.02793
 
 0.07043
 
 Not queried
 
  0.8522931
 
       NA
 
       NA
  
 
  
 1
 
 115300693
 
 115300877
 
  ENSEMBL   UCSC 
 
  185
 
  5
 
 1849
 
 tss
 
 0.01600
 
 0.03102
 
 Not queried
 
  0.8521094
 
       NA
 
       NA
  
 
  
 11
 
  70458782
 
  70459053
 
  ENSEMBL   UCSC 
 
  272
 
  4
 
 6774
 
 gene
 
 0.64570
 
 0.75022
 
 Not queried
 
  0.8519176
 
       NA
 
       NA
  
 
  
 1
 
 116380316
 
 116380575
 
  ENSEMBL   UCSC 
 
  260
 
  4
 
 702
 
 gene;island
 
 0.04374
 
 0.15487
 
 Not queried
 
  0.8517982
 
       NA
 
       NA
  
 
  
 2
 
  73114144
 
  73114426
 
  ENSEMBL   UCSC 
 
  283
 
  9
 
 23396
 
 tss
 
 0.03187
 
 0.06600
 
 Not queried
 
  0.8517908
 
       NA
 
       NA
  
 
  
 12
 
  95467511
 
  95467612
 
  ENSEMBL   UCSC 
 
  102
 
  5
 
 9896
 
 tss
 
 0.02579
 
 0.03920
 
 Not queried
 
  0.8517463
 
       NA
 
       NA
  
 
  
 8
 
 101117949
 
 101118083
 
  ENSEMBL   UCSC 
 
  135
 
  5
 
 39313
 
 gene;island
 
 0.80965
 
 0.93616
 
 Not queried
 
  0.8514974
 
       NA
 
       NA
  
 
  
 18
 
  77507539
 
  77507733
 
  ENSEMBL   UCSC 
 
  195
 
  4
 
 19109
 
 gene;island
 
 0.84189
 
 0.86988
 
 Not queried
 
  0.8514747
 
       NA
 
       NA
  
 
  
 18
 
  57364491
 
  57364872
 
  ENSEMBL   UCSC 
 
  382
 
  6
 
 19510
 
 island
 
 0.03430
 
 0.03352
 
 Not queried
 
  0.8513248
 
       NA
 
       NA
  
 
  
 14
 
  89021229
 
  89021477
 
  ENSEMBL   UCSC 
 
  249
 
  6
 
 12516
 
 tss
 
 0.02996
 
 0.05783
 
 Not queried
 
  0.8511358
 
       NA
 
       NA
  
 
  
 8
 
  27347861
 
  27348453
 
  ENSEMBL   UCSC 
 
  593
 
  5
 
 39550
 
 tss
 
 0.27873
 
 0.18078
 
 Not queried
 
  0.8510202
 
       NA
 
       NA
  
 
  
 19
 
  14606622
 
  14606902
 
  ENSEMBL   UCSC 
 
  281
 
  4
 
 19859
 
 gene
 
 0.02234
 
 0.03348
 
 Not queried
 
  0.8508767
 
       NA
 
       NA
  
 
  
 7
 
 151826130
 
 151826540
 
  ENSEMBL   UCSC 
 
  411
 
  4
 
 38984
 
 island
 
 0.95905
 
 0.93623
 
 Not queried
 
  0.8507973
 
       NA
 
       NA
  
 
  
 14
 
 102026086
 
 102026555
 
  ENSEMBL   UCSC 
 
  470
 
 11
 
 13129
 
 island
 
 0.09869
 
 0.05008
 
 Not queried
 
  0.8506509
 
       NA
 
       NA
  
 
  
 3
 
  39230030
 
  39230320
 
  ENSEMBL   UCSC 
 
  291
 
  4
 
 27682
 
 gene
 
 0.47644
 
 0.62114
 
 Not queried
 
  0.8503053
 
       NA
 
       NA
  
 
  
 3
 
  20226964
 
  20227091
 
  ENSEMBL   UCSC 
 
  128
 
  4
 
 27655
 
 gene;island
 
 0.01272
 
 0.01876
 
 Not queried
 
  0.8502918
 
       NA
 
       NA
  
 
  
 14
 
  45431486
 
  45431885
 
  ENSEMBL   UCSC 
 
  400
 
  5
 
 12044
 
 gene
 
 0.01413
 
 0.00892
 
 Not queried
 
  0.8502733
 
       NA
 
       NA
  
 
  
 5
 
  59189649
 
  59189934
 
  ENSEMBL   UCSC 
 
  286
 
  8
 
 32020
 
 tss
 
 0.06160
 
 0.10183
 
 Not queried
 
  0.8501795
 
       NA
 
       NA
  
 
  
 19
 
  12551931
 
  12552133
 
  ENSEMBL   UCSC 
 
  203
 
  4
 
 20460
 
 tss
 
 0.02640
 
 0.04694
 
 Not queried
 
  0.8501442
 
       NA
 
       NA
  
 
  
 22
 
  20236751
 
  20236856
 
  ENSEMBL   UCSC 
 
  106
 
  4
 
 26757
 
 gene;tss
 
 0.77554
 
 0.85199
 
 Not queried
 
  0.8500723
 
       NA
 
       NA
  
 
  
 1
 
 100731266
 
 100731633
 
  ENSEMBL   UCSC 
 
  368
 
  6
 
 1775
 
 tss
 
 0.02840
 
 0.03938
 
 Not queried
 
  0.8500444
 
       NA
 
       NA
  
 
  
 6
 
  30457271
 
  30458237
 
  ENSEMBL   UCSC 
 
  967
 
 31
 
 33705
 
 gene
 
 0.03728
 
 0.06532
 
 Not queried
 
  0.8500389
 
       NA
 
       NA
  
 
  
 17
 
  34842576
 
  34843101
 
  ENSEMBL   UCSC 
 
  526
 
  6
 
 16493
 
 gene
 
 0.03020
 
 0.05263
 
 Not queried
 
  0.8500245
 
       NA
 
       NA
  
 
  
 17
 
   1956958
 
   1958478
 
  ENSEMBL   UCSC 
 
 1521
 
 45
 
 16968
 
 tss;island
 
 0.04248
 
 0.07134
 
 Not queried
 
  0.8499241
 
       NA
 
       NA
  
 
  
 22
 
  31318349
 
  31318546
 
  ENSEMBL   UCSC 
 
  198
 
  4
 
 26798
 
 gene
 
 0.91627
 
 0.92256
 
 Not queried
 
  0.8498386
 
       NA
 
       NA
  
 
  
 17
 
   7307419
 
   7307572
 
  ENSEMBL   UCSC 
 
  154
 
  7
 
 17046
 
 tss
 
 0.00774
 
 0.01266
 
 Not queried
 
  0.8498361
 
       NA
 
       NA
  
 
  
 20
 
  57607335
 
  57607775
 
  ENSEMBL   UCSC 
 
  441
 
 12
 
 26262
 
 island
 
 0.02619
 
 0.03687
 
 Not queried
 
  0.8494989
 
       NA
 
       NA
  
 
  
 6
 
  26597000
 
  26597531
 
  ENSEMBL   UCSC 
 
  532
 
  5
 
 35478
 
 island
 
 0.03441
 
 0.05174
 
 Not queried
 
  0.8491106
 
       NA
 
       NA
  
 
  
 17
 
  18163716
 
  18163865
 
  ENSEMBL   UCSC 
 
  150
 
  7
 
 17143
 
 tss;island
 
 0.03241
 
 0.03274
 
 Not queried
 
  0.8491091
 
       NA
 
       NA
  
 
  
 3
 
 113233880
 
 113234093
 
  ENSEMBL   UCSC 
 
  214
 
  6
 
 29322
 
 island
 
 0.01623
 
 0.02806
 
 Not queried
 
  0.8488563
 
       NA
 
       NA
  
 
  
 5
 
    218988
 
    219334
 
  ENSEMBL   UCSC 
 
  347
 
  4
 
 31445
 
 gene;tss;island
 
 0.02072
 
 0.03233
 
 Not queried
 
  0.8487308
 
       NA
 
       NA
  
 
  
 5
 
   3854465
 
   3854732
 
  ENSEMBL   UCSC 
 
  268
 
  5
 
 32609
 
 island
 
 0.88696
 
 0.81335
 
 Not queried
 
  0.8485315
 
       NA
 
       NA
  
 
  
 15
 
  43425285
 
  43425949
 
  ENSEMBL   UCSC 
 
  665
 
  9
 
 14053
 
 island
 
 0.01022
 
 0.01824
 
 Not queried
 
  0.8482138
 
       NA
 
       NA
  
 
  
 14
 
 105911631
 
 105912068
 
  ENSEMBL   UCSC 
 
  438
 
  5
 
 12236
 
 gene;island
 
 0.83893
 
 0.91723
 
 Not queried
 
  0.8482085
 
       NA
 
       NA
  
 
  
 15
 
  45315202
 
  45315313
 
  ENSEMBL   UCSC 
 
  112
 
  4
 
 13616
 
 tss
 
 0.01180
 
 0.01687
 
 Not queried
 
  0.8481865
 
       NA
 
       NA
  
 
  
 6
 
  29570507
 
  29570781
 
  ENSEMBL   UCSC 
 
  275
 
  5
 
 33600
 
 gene
 
 0.69689
 
 0.78657
 
 Not queried
 
  0.8481524
 
       NA
 
       NA
  
 
  
 11
 
 111807963
 
 111808394
 
  ENSEMBL   UCSC 
 
  432
 
  4
 
 6883
 
 gene
 
 0.01869
 
 0.02413
 
 Not queried
 
  0.8480224
 
       NA
 
       NA
  
 
  
 2
 
 239228826
 
 239229182
 
  ENSEMBL   UCSC 
 
  357
 
  4
 
 23936
 
 tss
 
 0.02093
 
 0.01590
 
 Not queried
 
  0.8478848
 
       NA
 
       NA
  
 
  
 16
 
   1227293
 
   1227475
 
  ENSEMBL   UCSC 
 
  183
 
  4
 
 14530
 
 gene;island
 
 0.72609
 
 0.84284
 
 Not queried
 
  0.8478420
 
       NA
 
       NA
  
 
  
 15
 
  68346336
 
  68346524
 
  ENSEMBL   UCSC 
 
  189
 
  6
 
 13730
 
 tss
 
 0.01628
 
 0.02757
 
 Not queried
 
  0.8478118
 
       NA
 
       NA
  
 
  
 15
 
  65689058
 
  65689433
 
  ENSEMBL   UCSC 
 
  376
 
  6
 
 13351
 
 gene;island
 
 0.85139
 
 0.75219
 
 Not queried
 
  0.8477152
 
       NA
 
       NA
  
 
  
 6
 
  33421468
 
  33423398
 
  ENSEMBL   UCSC 
 
 1931
 
 49
 
 35977
 
 island
 
 0.02480
 
 0.03789
 
 Not queried
 
  0.8476885
 
       NA
 
       NA
  
 
  
 4
 
 144434253
 
 144434579
 
  ENSEMBL   UCSC 
 
  327
 
  9
 
 30565
 
 tss
 
 0.01000
 
 0.01487
 
 Not queried
 
  0.8474529
 
       NA
 
       NA
  
 
  
 12
 
  54426498
 
  54426772
 
  ENSEMBL   UCSC 
 
  275
 
  8
 
 9730
 
 tss
 
 0.04194
 
 0.07526
 
 Not queried
 
  0.8473706
 
       NA
 
       NA
  
 
  
 19
 
  43918868
 
  43919039
 
  ENSEMBL   UCSC 
 
  172
 
  7
 
 20037
 
 gene;island
 
 0.88047
 
 0.85778
 
 Not queried
 
  0.8473051
 
       NA
 
       NA
  
 
  
 1
 
 114355064
 
 114355299
 
  ENSEMBL   UCSC 
 
  236
 
  6
 
 3608
 
 island
 
 0.01622
 
 0.02110
 
 Not queried
 
  0.8473015
 
       NA
 
       NA
  
 
  
 16
 
  86337662
 
  86337823
 
  ENSEMBL   UCSC 
 
  162
 
  5
 
 16133
 
 island
 
 0.89229
 
 0.80942
 
 Not queried
 
  0.8471864
 
       NA
 
       NA
  
 
  
 1
 
 155006911
 
 155007323
 
  ENSEMBL   UCSC 
 
  413
 
  5
 
 786
 
 gene;tss
 
 0.74532
 
 0.57991
 
 Not queried
 
  0.8471682
 
       NA
 
       NA
  
 
  
 17
 
    900031
 
    900325
 
  ENSEMBL   UCSC 
 
  295
 
 10
 
 16951
 
 tss
 
 0.01434
 
 0.02423
 
 Not queried
 
  0.8471405
 
       NA
 
       NA
  
 
  
 1
 
   4192648
 
   4193192
 
  ENSEMBL   UCSC 
 
  545
 
  5
 
 2576
 
 island
 
 0.81722
 
 0.77256
 
 Not queried
 
  0.8470515
 
       NA
 
       NA
  
 
  
 1
 
  95698827
 
  95699097
 
  ENSEMBL   UCSC 
 
  271
 
  5
 
 1767
 
 tss;island
 
 0.04696
 
 0.06204
 
 Not queried
 
  0.8468552
 
       NA
 
       NA
  
 
  
 12
 
   3982588
 
   3982976
 
  ENSEMBL   UCSC 
 
  389
 
  5
 
 10141
 
 island
 
 0.01407
 
 0.01718
 
 Not queried
 
  0.8468016
 
       NA
 
       NA
  
 
  
 11
 
 111473054
 
 111473380
 
  ENSEMBL   UCSC 
 
  327
 
  4
 
 8779
 
 island
 
 0.01094
 
 0.01132
 
 Not queried
 
  0.8467618
 
       NA
 
       NA
  
 
  
 1
 
 166958221
 
 166958439
 
  ENSEMBL   UCSC 
 
  219
 
  9
 
 2069
 
 tss
 
 0.81431
 
 0.90551
 
 Not queried
 
  0.8466401
 
       NA
 
       NA
  
 
  
 12
 
  49393027
 
  49393408
 
  ENSEMBL   UCSC 
 
  382
 
  8
 
 10355
 
 island
 
 0.03838
 
 0.06044
 
 Not queried
 
  0.8464634
 
       NA
 
       NA
  
 
  
 7
 
  72972107
 
  72972316
 
  ENSEMBL   UCSC 
 
  210
 
  6
 
 37570
 
 tss;island
 
 0.04490
 
 0.04692
 
 Not queried
 
  0.8460439
 
       NA
 
       NA
  
 
  
 14
 
  94392718
 
  94392932
 
  ENSEMBL   UCSC 
 
  215
 
  4
 
 12164
 
 gene;tss
 
 0.91176
 
 0.84869
 
 Not queried
 
  0.8459747
 
       NA
 
       NA
  
 
  
 17
 
  72919318
 
  72919704
 
  ENSEMBL   UCSC 
 
  387
 
  8
 
 17602
 
 tss;island
 
 0.04696
 
 0.07344
 
 Not queried
 
  0.8456993
 
       NA
 
       NA
  
 
  
 3
 
  57199422
 
  57199533
 
  ENSEMBL   UCSC 
 
  112
 
  6
 
 28364
 
 tss
 
 0.01146
 
 0.02939
 
 Not queried
 
  0.8455658
 
       NA
 
       NA
  
 
  
 1
 
  28879147
 
  28879482
 
  ENSEMBL   UCSC 
 
  336
 
  6
 
 1426
 
 tss
 
 0.04247
 
 0.05921
 
 Not queried
 
  0.8455285
 
       NA
 
       NA
  
 
  
 1
 
  28879147
 
  28879976
 
  ENSEMBL   UCSC 
 
  830
 
 10
 
 2939
 
 island
 
 0.03369
 
 0.05921
 
 Not queried
 
  0.8455285
 
       NA
 
       NA
  
 
  
 1
 
  40997253
 
  40997551
 
  ENSEMBL   UCSC 
 
  299
 
  5
 
 455
 
 gene
 
 0.03641
 
 0.03927
 
 Not queried
 
  0.8454432
 
       NA
 
       NA
  
 
  
 22
 
  21400059
 
  21400184
 
  ENSEMBL   UCSC 
 
  126
 
  5
 
 26952
 
 tss;island
 
 0.02831
 
 0.05530
 
 Not queried
 
  0.8454069
 
       NA
 
       NA
  
 
  
 11
 
  92702373
 
  92702663
 
  ENSEMBL   UCSC 
 
  291
 
  7
 
 7596
 
 tss;island
 
 0.69179
 
 0.58191
 
 Not queried
 
  0.8453438
 
       NA
 
       NA
  
 
  
 9
 
  35096058
 
  35096453
 
  ENSEMBL   UCSC 
 
  396
 
  6
 
 40698
 
 gene;island
 
 0.03244
 
 0.01755
 
 Not queried
 
  0.8450032
 
       NA
 
       NA
  
 
  
 4
 
  47033126
 
  47033274
 
  ENSEMBL   UCSC 
 
  149
 
  5
 
 30329
 
 tss
 
 0.20794
 
 0.09924
 
 Not queried
 
  0.8447812
 
       NA
 
       NA
  
 
  
 1
 
 201924833
 
 201925070
 
  ENSEMBL   UCSC 
 
  238
 
  4
 
 940
 
 gene;island
 
 0.02365
 
 0.03376
 
 Not queried
 
  0.8446727
 
       NA
 
       NA
  
 
  
 2
 
  84686330
 
  84686570
 
  ENSEMBL   UCSC 
 
  241
 
  4
 
 22721
 
 gene
 
 0.01793
 
 0.01690
 
 Not queried
 
  0.8446207
 
       NA
 
       NA
  
 
  
 12
 
   7060187
 
   7060569
 
  ENSEMBL   UCSC 
 
  383
 
  8
 
 9015
 
 gene
 
 0.74530
 
 0.86129
 
 Not queried
 
  0.8446098
 
       NA
 
       NA
  
 
  
 12
 
   7060187
 
   7060386
 
  ENSEMBL   UCSC 
 
  200
 
  6
 
 9513
 
 tss
 
 0.74530
 
 0.86129
 
 Not queried
 
  0.8446098
 
       NA
 
       NA
  
 
  
 17
 
  72580901
 
  72581014
 
  ENSEMBL   UCSC 
 
  114
 
  4
 
 17595
 
 tss
 
 0.67168
 
 0.50489
 
 Not queried
 
  0.8446008
 
       NA
 
       NA
  
 
  
 8
 
  22223852
 
  22224272
 
  ENSEMBL   UCSC 
 
  421
 
  6
 
 39522
 
 tss;island
 
 0.65015
 
 0.50755
 
 Not queried
 
  0.8445680
 
       NA
 
       NA
  
 
  
 1
 
 155099774
 
 155100190
 
  ENSEMBL   UCSC 
 
  417
 
  7
 
 1976
 
 tss
 
 0.01131
 
 0.02488
 
 Not queried
 
  0.8443170
 
       NA
 
       NA
  
 
  
 4
 
  48908469
 
  48909110
 
  ENSEMBL   UCSC 
 
  642
 
  9
 
 30984
 
 island
 
 0.91771
 
 0.82632
 
 Not queried
 
  0.8440302
 
       NA
 
       NA
  
 
  
 11
 
   2871400
 
   2871992
 
  ENSEMBL   UCSC 
 
  593
 
  9
 
 7952
 
 island
 
 0.76174
 
 0.85069
 
 Not queried
 
  0.8437304
 
       NA
 
       NA
  
 
  
 1
 
   3623319
 
   3623605
 
  ENSEMBL   UCSC 
 
  287
 
  4
 
 140
 
 gene;island
 
 0.88480
 
 0.79517
 
 Not queried
 
  0.8435709
 
       NA
 
       NA
  
 
  
 10
 
  45454796
 
  45455500
 
  ENSEMBL   UCSC 
 
  705
 
 12
 
 5690
 
 island
 
 0.01851
 
 0.03197
 
 Not queried
 
  0.8435545
 
       NA
 
       NA
  
 
  
 1
 
 111743020
 
 111743537
 
  ENSEMBL   UCSC 
 
  518
 
  7
 
 3579
 
 island
 
 0.90786
 
 0.82713
 
 Not queried
 
  0.8435126
 
       NA
 
       NA
  
 
  
 19
 
  11998686
 
  11999148
 
  ENSEMBL   UCSC 
 
  463
 
  7
 
 19814
 
 gene
 
 0.01864
 
 0.03101
 
 Not queried
 
  0.8434949
 
       NA
 
       NA
  
 
  
 19
 
  11517079
 
  11517436
 
  ENSEMBL   UCSC 
 
  358
 
  5
 
 19802
 
 gene;island
 
 0.38083
 
 0.49084
 
 Not queried
 
  0.8434842
 
       NA
 
       NA
  
 
  
 10
 
  75457622
 
  75457686
 
  ENSEMBL   UCSC 
 
   65
 
  4
 
 5144
 
 tss
 
 0.90871
 
 0.87495
 
 Not queried
 
  0.8434414
 
       NA
 
       NA
  
 
  
 3
 
  11314015
 
  11314335
 
  ENSEMBL   UCSC 
 
  321
 
  4
 
 27625
 
 gene
 
 0.01551
 
 0.02847
 
 Not queried
 
  0.8434352
 
       NA
 
       NA
  
 
  
 10
 
  45455245
 
  45455500
 
  ENSEMBL   UCSC 
 
  256
 
  4
 
 4560
 
 gene
 
 0.02554
 
 0.04703
 
 Not queried
 
  0.8433807
 
       NA
 
       NA
  
 
  
 15
 
  85143675
 
  85144228
 
  ENSEMBL   UCSC 
 
  554
 
 10
 
 13840
 
 tss
 
 0.02046
 
 0.03596
 
 Not queried
 
  0.8432355
 
       NA
 
       NA
  
 
  
 21
 
  34442160
 
  34442377
 
  ENSEMBL   UCSC 
 
  218
 
  7
 
 26476
 
 tss
 
 0.03759
 
 0.02491
 
 Not queried
 
  0.8432035
 
       NA
 
       NA
  
 
  
 19
 
  45927563
 
  45927696
 
  ENSEMBL   UCSC 
 
  134
 
  4
 
 20835
 
 tss;island
 
 0.03659
 
 0.01796
 
 Not queried
 
  0.8429541
 
       NA
 
       NA
  
 
  
 3
 
 184530243
 
 184530595
 
  ENSEMBL   UCSC 
 
  353
 
  5
 
 28024
 
 gene;island
 
 0.01445
 
 0.03343
 
 Not queried
 
  0.8428781
 
       NA
 
       NA
  
 
  
 7
 
 157345452
 
 157345827
 
  ENSEMBL   UCSC 
 
  376
 
  6
 
 37262
 
 gene;island
 
 0.78473
 
 0.82296
 
 Not queried
 
  0.8426517
 
       NA
 
       NA
  
 
  
 2
 
  98852704
 
  98852938
 
  ENSEMBL   UCSC 
 
  235
 
  5
 
 22762
 
 gene
 
 0.94219
 
 0.90399
 
 Not queried
 
  0.8425467
 
       NA
 
       NA
  
 
  
 19
 
   1753444
 
   1753640
 
  ENSEMBL   UCSC 
 
  197
 
  6
 
 20274
 
 tss;island
 
 0.08773
 
 0.02884
 
 Not queried
 
  0.8424173
 
       NA
 
       NA
  
 
  
 2
 
 219849133
 
 219849307
 
  ENSEMBL   UCSC 
 
  175
 
  4
 
 22986
 
 gene;island
 
 0.31178
 
 0.16555
 
 Not queried
 
  0.8421308
 
       NA
 
       NA
  
 
  
 17
 
  46655164
 
  46656093
 
  ENSEMBL   UCSC 
 
  930
 
 18
 
 18446
 
 island
 
 0.02099
 
 0.03726
 
 Not queried
 
  0.8420948
 
       NA
 
       NA
  
 
  
 2
 
  98280624
 
  98280854
 
  ENSEMBL   UCSC 
 
  231
 
  9
 
 23492
 
 tss
 
 0.01265
 
 0.02526
 
 Not queried
 
  0.8420114
 
       NA
 
       NA
  
 
  
 5
 
 180230022
 
 180230146
 
  ENSEMBL   UCSC 
 
  125
 
  5
 
 31887
 
 gene;island
 
 0.14906
 
 0.07992
 
 Not queried
 
  0.8418959
 
       NA
 
       NA
  
 
  
 7
 
  35577028
 
  35577269
 
  ENSEMBL   UCSC 
 
  242
 
  6
 
 38346
 
 island
 
 0.02941
 
 0.01589
 
 Not queried
 
  0.8417318
 
       NA
 
       NA
  
 
  
 X
 
  38420326
 
  38420581
 
  ENSEMBL   UCSC 
 
  256
 
  5
 
 41258
 
 tss
 
 0.13805
 
 0.09983
 
 Not queried
 
  0.8415859
 
       NA
 
       NA
  
 
  
 X
 
  38420326
 
  38420783
 
  ENSEMBL   UCSC 
 
  458
 
  7
 
 41721
 
 island
 
 0.13320
 
 0.06371
 
 Not queried
 
  0.8415859
 
       NA
 
       NA
  
 
  
 13
 
 100626995
 
 100627366
 
  ENSEMBL   UCSC 
 
  372
 
  4
 
 11817
 
 island
 
 0.30682
 
 0.47282
 
 Not queried
 
  0.8415454
 
       NA
 
       NA
  
 
  
 16
 
  66914113
 
  66914412
 
  ENSEMBL   UCSC 
 
  300
 
  4
 
 15256
 
 tss
 
 0.02493
 
 0.04901
 
 Not queried
 
  0.8413539
 
       NA
 
       NA
  
 
  
 19
 
  13906139
 
  13906241
 
  ENSEMBL   UCSC 
 
  103
 
  5
 
 20493
 
 tss;island
 
 0.01915
 
 0.03380
 
 Not queried
 
  0.8412564
 
       NA
 
       NA
  
 
  
 8
 
 141646205
 
 141646502
 
  ENSEMBL   UCSC 
 
  298
 
  6
 
 39862
 
 tss;island
 
 0.02603
 
 0.05158
 
 Not queried
 
  0.8412430
 
       NA
 
       NA
  
 
  
 1
 
 155099774
 
 155100511
 
  ENSEMBL   UCSC 
 
  738
 
 11
 
 3797
 
 island
 
 0.02858
 
 0.03123
 
 Not queried
 
  0.8411162
 
       NA
 
       NA
  
 
  
 5
 
   1093580
 
   1093797
 
  ENSEMBL   UCSC 
 
  218
 
  4
 
 31469
 
 gene;island
 
 0.79271
 
 0.85089
 
 Not queried
 
  0.8411142
 
       NA
 
       NA
  
 
  
 7
 
  97841334
 
  97841591
 
  ENSEMBL   UCSC 
 
  258
 
  6
 
 38645
 
 island
 
 0.89878
 
 0.74578
 
 Not queried
 
  0.8410919
 
       NA
 
       NA
  
 
  
 19
 
  50321295
 
  50321467
 
  ENSEMBL   UCSC 
 
  173
 
  8
 
 20928
 
 tss
 
 0.01136
 
 0.01504
 
 Not queried
 
  0.8410699
 
       NA
 
       NA
  
 
  
 11
 
  17229487
 
  17229731
 
  ENSEMBL   UCSC 
 
  245
 
  5
 
 8074
 
 island
 
 0.00574
 
 0.00821
 
 Not queried
 
  0.8405474
 
       NA
 
       NA
  
 
  
 2
 
 233470647
 
 233470724
 
  ENSEMBL   UCSC 
 
   78
 
  5
 
 23914
 
 tss
 
 0.02296
 
 0.03934
 
 Not queried
 
  0.8405365
 
       NA
 
       NA
  
 
  
 1
 
   3239916
 
   3240227
 
  ENSEMBL   UCSC 
 
  312
 
  4
 
 119
 
 gene;island
 
 0.66068
 
 0.79618
 
 Not queried
 
  0.8405099
 
       NA
 
       NA
  
 
  
 19
 
   3825141
 
   3825630
 
  ENSEMBL   UCSC 
 
  490
 
  5
 
 19689
 
 gene;island
 
 0.74883
 
 0.84342
 
 Not queried
 
  0.8404567
 
       NA
 
       NA
  
 
  
 10
 
 109674854
 
 109675284
 
  ENSEMBL   UCSC 
 
  431
 
  4
 
 6103
 
 island
 
 0.04426
 
 0.02481
 
 Not queried
 
  0.8403647
 
       NA
 
       NA
  
 
  
 6
 
 137815539
 
 137815725
 
  ENSEMBL   UCSC 
 
  187
 
  6
 
 35185
 
 tss;island
 
 0.05024
 
 0.02281
 
 Not queried
 
  0.8403429
 
       NA
 
       NA
  
 
  
 19
 
  17861850
 
  17862210
 
  ENSEMBL   UCSC 
 
  361
 
  6
 
 20563
 
 tss
 
 0.78637
 
 0.87133
 
 Not queried
 
  0.8401864
 
       NA
 
       NA
  
 
  
 7
 
   1544019
 
   1544333
 
  ENSEMBL   UCSC 
 
  315
 
  4
 
 37335
 
 tss
 
 0.01890
 
 0.02734
 
 Not queried
 
  0.8401584
 
       NA
 
       NA
  
 
  
 6
 
  30094947
 
  30095802
 
  ENSEMBL   UCSC 
 
  856
 
 30
 
 35602
 
 island
 
 0.05209
 
 0.10385
 
 Not queried
 
  0.8401233
 
       NA
 
       NA
  
 
  
 16
 
   2261069
 
   2261237
 
  ENSEMBL   UCSC 
 
  169
 
  7
 
 14990
 
 tss
 
 0.89840
 
 0.86200
 
 Not queried
 
  0.8401225
 
       NA
 
       NA
  
 
  
 19
 
   3557658
 
   3557873
 
  ENSEMBL   UCSC 
 
  216
 
  5
 
 20309
 
 tss
 
 0.01267
 
 0.02294
 
 Not queried
 
  0.8401193
 
       NA
 
       NA
  
 
  
 19
 
   3557418
 
   3557873
 
  ENSEMBL   UCSC 
 
  456
 
  7
 
 21297
 
 island
 
 0.01714
 
 0.03924
 
 Not queried
 
  0.8401193
 
       NA
 
       NA
  
 
  
 6
 
  99278991
 
  99279108
 
  ENSEMBL   UCSC 
 
  118
 
  4
 
 36280
 
 island
 
 0.10861
 
 0.17666
 
 Not queried
 
  0.8400879
 
       NA
 
       NA
  
 
  
 5
 
 108745819
 
 108746499
 
  ENSEMBL   UCSC 
 
  681
 
  7
 
 32143
 
 tss;island
 
 0.01098
 
 0.00994
 
 Not queried
 
  0.8400536
 
       NA
 
       NA
  
 
  
 10
 
   6243791
 
   6244010
 
  ENSEMBL   UCSC 
 
  220
 
  4
 
 4466
 
 gene;tss;island
 
 0.02811
 
 0.03306
 
 Not queried
 
  0.8400237
 
       NA
 
       NA
  
 
  
 1
 
  16464377
 
  16464509
 
  ENSEMBL   UCSC 
 
  133
 
  4
 
 258
 
 gene;island
 
 0.92166
 
 0.86010
 
 Not queried
 
  0.8399676
 
       NA
 
       NA
  
 
  
 3
 
  52811297
 
  52811637
 
  ENSEMBL   UCSC 
 
  341
 
  4
 
 28348
 
 tss
 
 0.83759
 
 0.77368
 
 Not queried
 
  0.8399298
 
       NA
 
       NA
  
 
  
 7
 
    588936
 
    589354
 
  ENSEMBL   UCSC 
 
  419
 
  4
 
 36645
 
 gene;island
 
 0.70844
 
 0.86698
 
 Not queried
 
  0.8397415
 
       NA
 
       NA
  
 
  
 11
 
   2017362
 
   2018247
 
  ENSEMBL   UCSC 
 
  886
 
 13
 
 6425
 
 gene;island
 
 0.83313
 
 0.71006
 
 Not queried
 
  0.8397385
 
       NA
 
       NA
  
 
  
 20
 
  54933521
 
  54934110
 
  ENSEMBL   UCSC 
 
  590
 
 10
 
 26232
 
 island
 
 0.04855
 
 0.06687
 
 Not queried
 
  0.8396413
 
       NA
 
       NA
  
 
  
 19
 
  19643011
 
  19643269
 
  ENSEMBL   UCSC 
 
  259
 
  4
 
 19919
 
 gene;island
 
 0.08956
 
 0.18773
 
 Not queried
 
  0.8396202
 
       NA
 
       NA
  
 
  
 16
 
   1755945
 
   1756201
 
  ENSEMBL   UCSC 
 
  257
 
  4
 
 14971
 
 tss
 
 0.02532
 
 0.04441
 
 Not queried
 
  0.8396098
 
       NA
 
       NA
  
 
  
 21
 
  18985610
 
  18985761
 
  ENSEMBL   UCSC 
 
  152
 
  4
 
 26451
 
 tss;island
 
 0.13764
 
 0.07252
 
 Not queried
 
  0.8394576
 
       NA
 
       NA
  
 
  
 17
 
  81052622
 
  81052999
 
  ENSEMBL   UCSC 
 
  378
 
  8
 
 18997
 
 island
 
 0.95139
 
 0.96047
 
 Not queried
 
  0.8393400
 
       NA
 
       NA
  
 
  
 1
 
  15375064
 
  15375511
 
  ENSEMBL   UCSC 
 
  448
 
  5
 
 241
 
 gene;island
 
 0.91572
 
 0.86797
 
 Not queried
 
  0.8393189
 
       NA
 
       NA
  
 
  
 15
 
 101084980
 
 101085111
 
  ENSEMBL   UCSC 
 
  132
 
  4
 
 13901
 
 tss;island
 
 0.95005
 
 0.86870
 
 Not queried
 
  0.8391311
 
       NA
 
       NA
  
 
  
 9
 
  72873345
 
  72873728
 
  ENSEMBL   UCSC 
 
  384
 
  4
 
 40750
 
 tss;island
 
 0.04895
 
 0.02420
 
 Not queried
 
  0.8391268
 
       NA
 
       NA
  
 
  
 14
 
  35873967
 
  35874353
 
  ENSEMBL   UCSC 
 
  387
 
  7
 
 12325
 
 tss;island
 
 0.00937
 
 0.02213
 
 Not queried
 
  0.8391117
 
       NA
 
       NA
  
 
  
 15
 
  79296377
 
  79296585
 
  ENSEMBL   UCSC 
 
  209
 
  4
 
 13415
 
 gene
 
 0.86199
 
 0.92973
 
 Not queried
 
  0.8388819
 
       NA
 
       NA
  
 
  
 19
 
  39138026
 
  39138551
 
  ENSEMBL   UCSC 
 
  526
 
 10
 
 21945
 
 island
 
 0.05189
 
 0.04062
 
 Not queried
 
  0.8387992
 
       NA
 
       NA
  
 
  
 20
 
  30326920
 
  30327157
 
  ENSEMBL   UCSC 
 
  238
 
  4
 
 25343
 
 gene
 
 0.00899
 
 0.01476
 
 Not queried
 
  0.8387348
 
       NA
 
       NA
  
 
  
 19
 
  16394366
 
  16394774
 
  ENSEMBL   UCSC 
 
  409
 
  5
 
 21680
 
 island
 
 0.68500
 
 0.75547
 
 Not queried
 
  0.8386548
 
       NA
 
       NA
  
 
  
 7
 
 155326713
 
 155326803
 
  ENSEMBL   UCSC 
 
   91
 
  4
 
 37948
 
 tss;island
 
 0.22459
 
 0.09818
 
 Not queried
 
  0.8385774
 
       NA
 
       NA
  
 
  
 19
 
  40889848
 
  40890114
 
  ENSEMBL   UCSC 
 
  267
 
  4
 
 20004
 
 gene;island
 
 0.87024
 
 0.78741
 
 Not queried
 
  0.8385467
 
       NA
 
       NA
  
 
  
 14
 
 100203942
 
 100204258
 
  ENSEMBL   UCSC 
 
  317
 
  6
 
 13101
 
 island
 
 0.67879
 
 0.77940
 
 Not queried
 
  0.8385283
 
       NA
 
       NA
  
 
  
 16
 
  66584061
 
  66584358
 
  ENSEMBL   UCSC 
 
  298
 
  4
 
 15954
 
 island
 
 0.03116
 
 0.01495
 
 Not queried
 
  0.8385196
 
       NA
 
       NA
  
 
  
 5
 
  40679636
 
  40680039
 
  ENSEMBL   UCSC 
 
  404
 
 10
 
 32688
 
 island
 
 0.01837
 
 0.02914
 
 Not queried
 
  0.8384195
 
       NA
 
       NA
  
 
  
 19
 
  53635967
 
  53636398
 
  ENSEMBL   UCSC 
 
  432
 
  8
 
 22333
 
 island
 
 0.01631
 
 0.02679
 
 Not queried
 
  0.8383816
 
       NA
 
       NA
  
 
  
 17
 
  72355995
 
  72356254
 
  ENSEMBL   UCSC 
 
  260
 
  4
 
 16718
 
 gene;island
 
 0.57524
 
 0.66127
 
 Not queried
 
  0.8383061
 
       NA
 
       NA
  
 
  
 1
 
  35324702
 
  35324786
 
  ENSEMBL   UCSC 
 
   85
 
  4
 
 1476
 
 tss
 
 0.01898
 
 0.03471
 
 Not queried
 
  0.8381280
 
       NA
 
       NA
  
 
  
 3
 
  32147645
 
  32148040
 
  ENSEMBL   UCSC 
 
  396
 
  7
 
 28162
 
 tss
 
 0.02912
 
 0.06009
 
 Not queried
 
  0.8381170
 
       NA
 
       NA
  
 
  
 5
 
 169930841
 
 169930938
 
  ENSEMBL   UCSC 
 
   98
 
  6
 
 32425
 
 tss
 
 0.33024
 
 0.30429
 
 Not queried
 
  0.8380805
 
       NA
 
       NA
  
 
  
 10
 
 124913438
 
 124913724
 
  ENSEMBL   UCSC 
 
  287
 
  7
 
 5400
 
 tss
 
 0.02562
 
 0.02569
 
 Not queried
 
  0.8380339
 
       NA
 
       NA
  
 
  
 2
 
 114048829
 
 114049041
 
  ENSEMBL   UCSC 
 
  213
 
  5
 
 22798
 
 gene;island
 
 0.09077
 
 0.11026
 
 Not queried
 
  0.8379431
 
       NA
 
       NA
  
 
  
 2
 
 233321600
 
 233321900
 
  ENSEMBL   UCSC 
 
  301
 
  4
 
 23025
 
 gene;island
 
 0.81806
 
 0.77552
 
 Not queried
 
  0.8375604
 
       NA
 
       NA
  
 
  
 5
 
 154320467
 
 154320616
 
  ENSEMBL   UCSC 
 
  150
 
  5
 
 32394
 
 tss
 
 0.03738
 
 0.03821
 
 Not queried
 
  0.8375363
 
       NA
 
       NA
  
 
  
 5
 
 154320467
 
 154320678
 
  ENSEMBL   UCSC 
 
  212
 
  7
 
 33275
 
 island
 
 0.03823
 
 0.04402
 
 Not queried
 
  0.8375363
 
       NA
 
       NA
  
 
  
 10
 
  88126089
 
  88126306
 
  ENSEMBL   UCSC 
 
  218
 
  8
 
 5883
 
 island
 
 0.03471
 
 0.05146
 
 Not queried
 
  0.8371842
 
       NA
 
       NA
  
 
  
 10
 
 104154298
 
 104154605
 
  ENSEMBL   UCSC 
 
  308
 
  4
 
 4719
 
 gene
 
 0.01673
 
 0.03025
 
 Not queried
 
  0.8369615
 
       NA
 
       NA
  
 
  
 13
 
  31479935
 
  31480304
 
  ENSEMBL   UCSC 
 
  370
 
  6
 
 11348
 
 tss
 
 0.66289
 
 0.69227
 
 Not queried
 
  0.8369528
 
       NA
 
       NA
  
 
  
 6
 
  15663024
 
  15663538
 
  ENSEMBL   UCSC 
 
  515
 
 11
 
 35404
 
 island
 
 0.01904
 
 0.02060
 
 Not queried
 
  0.8369174
 
       NA
 
       NA
  
 
  
 11
 
    695461
 
    695585
 
  ENSEMBL   UCSC 
 
  125
 
  5
 
 6368
 
 gene;tss;island
 
 0.01228
 
 0.01810
 
 Not queried
 
  0.8368847
 
       NA
 
       NA
  
 
  
 12
 
    322514
 
    322983
 
  ENSEMBL   UCSC 
 
  470
 
 10
 
 8967
 
 gene;tss
 
 0.50363
 
 0.54774
 
 Not queried
 
  0.8368419
 
       NA
 
       NA
  
 
  
 19
 
  47617042
 
  47617301
 
  ENSEMBL   UCSC 
 
  260
 
  7
 
 20866
 
 tss;island
 
 0.03617
 
 0.02845
 
 Not queried
 
  0.8366672
 
       NA
 
       NA
  
 
  
 7
 
 151553471
 
 151553868
 
  ENSEMBL   UCSC 
 
  398
 
  6
 
 37234
 
 gene
 
 0.56851
 
 0.70045
 
 Not queried
 
  0.8364769
 
       NA
 
       NA
  
 
  
 2
 
 154335014
 
 154335250
 
  ENSEMBL   UCSC 
 
  237
 
  4
 
 22862
 
 gene
 
 0.03322
 
 0.05402
 
 Not queried
 
  0.8360398
 
       NA
 
       NA
  
 
  
 5
 
 140514711
 
 140515207
 
  ENSEMBL   UCSC 
 
  497
 
  6
 
 33150
 
 island
 
 0.01013
 
 0.02363
 
 Not queried
 
  0.8358961
 
       NA
 
       NA
  
 
  
 3
 
 100428005
 
 100428172
 
  ENSEMBL   UCSC 
 
  168
 
  7
 
 29286
 
 island
 
 0.00997
 
 0.01743
 
 Not queried
 
  0.8358864
 
       NA
 
       NA
  
 
  
 15
 
  50647096
 
  50647267
 
  ENSEMBL   UCSC 
 
  172
 
  6
 
 13639
 
 tss
 
 0.01652
 
 0.03626
 
 Not queried
 
  0.8358713
 
       NA
 
       NA
  
 
  
 6
 
  32044104
 
  32044496
 
  ENSEMBL   UCSC 
 
  393
 
  8
 
 33971
 
 gene;island
 
 0.76620
 
 0.63329
 
 Not queried
 
  0.8357712
 
       NA
 
       NA
  
 
  
 18
 
  43683922
 
  43684181
 
  ENSEMBL   UCSC 
 
  260
 
  8
 
 19067
 
 gene
 
 0.08588
 
 0.02514
 
 Not queried
 
  0.8356967
 
       NA
 
       NA
  
 
  
 17
 
  27717641
 
  27718019
 
  ENSEMBL   UCSC 
 
  379
 
  9
 
 18135
 
 island
 
 0.01434
 
 0.02046
 
 Not queried
 
  0.8356017
 
       NA
 
       NA
  
 
  
 20
 
   3643863
 
   3644300
 
  ENSEMBL   UCSC 
 
  438
 
  6
 
 25932
 
 island
 
 0.61401
 
 0.47080
 
 Not queried
 
  0.8355259
 
       NA
 
       NA
  
 
  
 7
 
 157561010
 
 157561161
 
  ENSEMBL   UCSC 
 
  152
 
  4
 
 37278
 
 gene;island
 
 0.77321
 
 0.85665
 
 Not queried
 
  0.8355229
 
       NA
 
       NA
  
 
  
 6
 
  35995246
 
  35995431
 
  ENSEMBL   UCSC 
 
  186
 
  9
 
 34870
 
 tss
 
 0.01627
 
 0.03183
 
 Not queried
 
  0.8354969
 
       NA
 
       NA
  
 
  
 3
 
 158518888
 
 158519176
 
  ENSEMBL   UCSC 
 
  289
 
  4
 
 28644
 
 tss;island
 
 0.10822
 
 0.03535
 
 Not queried
 
  0.8352445
 
       NA
 
       NA
  
 
  
 19
 
  45926999
 
  45927056
 
  ENSEMBL   UCSC 
 
   58
 
  5
 
 20061
 
 gene
 
 0.02584
 
 0.03985
 
 Not queried
 
  0.8351939
 
       NA
 
       NA
  
 
  
 2
 
 223166909
 
 223167617
 
  ENSEMBL   UCSC 
 
  709
 
  8
 
 23013
 
 gene;island
 
 0.34777
 
 0.23068
 
 Not queried
 
  0.8351553
 
       NA
 
       NA
  
 
  
 6
 
  24774877
 
  24775011
 
  ENSEMBL   UCSC 
 
  135
 
  4
 
 34607
 
 tss;island
 
 0.01397
 
 0.01322
 
 Not queried
 
  0.8349741
 
       NA
 
       NA
  
 
  
 8
 
  65281397
 
  65281689
 
  ENSEMBL   UCSC 
 
  293
 
  5
 
 40274
 
 island
 
 0.04656
 
 0.01831
 
 Not queried
 
  0.8349532
 
       NA
 
       NA
  
 
  
 19
 
  51071294
 
  51071328
 
  ENSEMBL   UCSC 
 
   35
 
  5
 
 22280
 
 island
 
 0.04376
 
 0.09968
 
 Not queried
 
  0.8348393
 
       NA
 
       NA
  
 
  
 8
 
  42397159
 
  42397328
 
  ENSEMBL   UCSC 
 
  170
 
  6
 
 39234
 
 gene;tss;island
 
 0.01522
 
 0.02482
 
 Not queried
 
  0.8347015
 
       NA
 
       NA
  
 
  
 16
 
  28961797
 
  28962284
 
  ENSEMBL   UCSC 
 
  488
 
  8
 
 15131
 
 tss;island
 
 0.02081
 
 0.08135
 
 Not queried
 
  0.8346787
 
       NA
 
       NA
  
 
  
 11
 
  62559514
 
  62559882
 
  ENSEMBL   UCSC 
 
  369
 
  8
 
 7346
 
 tss
 
 0.04607
 
 0.09618
 
 Not queried
 
  0.8345841
 
       NA
 
       NA
  
 
  
 18
 
  72201504
 
  72201665
 
  ENSEMBL   UCSC 
 
  162
 
  5
 
 19290
 
 tss
 
 0.74103
 
 0.86056
 
 Not queried
 
  0.8343682
 
       NA
 
       NA
  
 
  
 6
 
 134210598
 
 134211217
 
  ENSEMBL   UCSC 
 
  620
 
  6
 
 34414
 
 gene;island
 
 0.08232
 
 0.04402
 
 Not queried
 
  0.8341775
 
       NA
 
       NA
  
 
  
 6
 
 110501610
 
 110501899
 
  ENSEMBL   UCSC 
 
  290
 
  5
 
 35104
 
 tss;island
 
 0.01391
 
 0.01890
 
 Not queried
 
  0.8341464
 
       NA
 
       NA
  
 
  
 2
 
 128283239
 
 128283815
 
  ENSEMBL   UCSC 
 
  577
 
  5
 
 22822
 
 gene;island
 
 0.02276
 
 0.03856
 
 Not queried
 
  0.8340007
 
       NA
 
       NA
  
 
  
 14
 
  93260171
 
  93260381
 
  ENSEMBL   UCSC 
 
  211
 
  4
 
 12541
 
 tss;island
 
 0.01886
 
 0.03345
 
 Not queried
 
  0.8339000
 
       NA
 
       NA
  
 
  
 17
 
  19648846
 
  19649293
 
  ENSEMBL   UCSC 
 
  448
 
 10
 
 17161
 
 tss
 
 0.79882
 
 0.63079
 
 Not queried
 
  0.8338714
 
       NA
 
       NA
  
 
  
 6
 
  31508106
 
  31508318
 
  ENSEMBL   UCSC 
 
  213
 
  6
 
 33831
 
 gene;island
 
 0.59446
 
 0.77348
 
 Not queried
 
  0.8337356
 
       NA
 
       NA
  
 
  
 19
 
   3369702
 
   3369820
 
  ENSEMBL   UCSC 
 
  119
 
  4
 
 19679
 
 gene;island
 
 0.28512
 
 0.37806
 
 Not queried
 
  0.8334698
 
       NA
 
       NA
  
 
  
 6
 
  31937731
 
  31938533
 
  ENSEMBL   UCSC 
 
  803
 
 13
 
 33943
 
 gene
 
 0.87504
 
 0.93150
 
 Not queried
 
  0.8334266
 
       NA
 
       NA
  
 
  
 1
 
  25291905
 
  25292412
 
  ENSEMBL   UCSC 
 
  508
 
 11
 
 1362
 
 tss
 
 0.87411
 
 0.85905
 
 Not queried
 
  0.8333361
 
       NA
 
       NA
  
 
  
 1
 
 156339011
 
 156339286
 
  ENSEMBL   UCSC 
 
  276
 
  4
 
 820
 
 gene
 
 0.15971
 
 0.14092
 
 Not queried
 
  0.8332959
 
       NA
 
       NA
  
 
  
 14
 
 104569519
 
 104569653
 
  ENSEMBL   UCSC 
 
  135
 
  4
 
 12217
 
 gene;island
 
 0.60521
 
 0.70433
 
 Not queried
 
  0.8331943
 
       NA
 
       NA
  
 
  
 19
 
  17861850
 
  17862385
 
  ENSEMBL   UCSC 
 
  536
 
  7
 
 19885
 
 gene;island
 
 0.78269
 
 0.87019
 
 Not queried
 
  0.8331619
 
       NA
 
       NA
  
 
  
 10
 
 100995599
 
 100995821
 
  ENSEMBL   UCSC 
 
  223
 
  4
 
 5986
 
 island
 
 0.03956
 
 0.07504
 
 Not queried
 
  0.8331234
 
       NA
 
       NA
  
 
  
 11
 
  86662968
 
  86663418
 
  ENSEMBL   UCSC 
 
  451
 
  6
 
 6836
 
 gene;island
 
 0.88483
 
 0.93190
 
 Not queried
 
  0.8331158
 
       NA
 
       NA
  
 
  
 1
 
  36689760
 
  36689900
 
  ENSEMBL   UCSC 
 
  141
 
  5
 
 1490
 
 tss
 
 0.01651
 
 0.03421
 
 Not queried
 
  0.8329702
 
       NA
 
       NA
  
 
  
 17
 
  40713676
 
  40714003
 
  ENSEMBL   UCSC 
 
  328
 
  8
 
 17332
 
 tss
 
 0.03923
 
 0.06054
 
 Not queried
 
  0.8329701
 
       NA
 
       NA
  
 
  
 17
 
  68165632
 
  68165759
 
  ENSEMBL   UCSC 
 
  128
 
  4
 
 18662
 
 island
 
 0.03843
 
 0.05585
 
 Not queried
 
  0.8329465
 
       NA
 
       NA
  
 
  
 15
 
  79043428
 
  79043637
 
  ENSEMBL   UCSC 
 
  210
 
  4
 
 14332
 
 island
 
 0.06603
 
 0.16850
 
 Not queried
 
  0.8327785
 
       NA
 
       NA
  
 
  
 1
 
  17446027
 
  17446162
 
  ENSEMBL   UCSC 
 
  136
 
  5
 
 1288
 
 tss
 
 0.02787
 
 0.05751
 
 Not queried
 
  0.8327221
 
       NA
 
       NA
  
 
  
 6
 
  30899145
 
  30899649
 
  ENSEMBL   UCSC 
 
  505
 
  7
 
 33778
 
 gene
 
 0.51338
 
 0.57829
 
 Not queried
 
  0.8324357
 
       NA
 
       NA
  
 
  
 X
 
  13397207
 
  13397532
 
  ENSEMBL   UCSC 
 
  326
 
  5
 
 41645
 
 island
 
 0.96617
 
 0.92785
 
 Not queried
 
  0.8323712
 
       NA
 
       NA
  
 
  
 13
 
  43566401
 
  43566642
 
  ENSEMBL   UCSC 
 
  242
 
  9
 
 11386
 
 tss
 
 0.03568
 
 0.06180
 
 Not queried
 
  0.8323480
 
       NA
 
       NA
  
 
  
 X
 
  66787995
 
  66788395
 
  ENSEMBL   UCSC 
 
  401
 
  6
 
 41009
 
 gene;tss
 
 0.75896
 
 0.89518
 
 Not queried
 
  0.8322992
 
       NA
 
       NA
  
 
  
 11
 
 120894635
 
 120894841
 
  ENSEMBL   UCSC 
 
  207
 
  5
 
 8873
 
 island
 
 0.01883
 
 0.03210
 
 Not queried
 
  0.8319959
 
       NA
 
       NA
  
 
  
 15
 
  41694502
 
  41694790
 
  ENSEMBL   UCSC 
 
  289
 
  4
 
 14032
 
 island
 
 0.01385
 
 0.02423
 
 Not queried
 
  0.8319817
 
       NA
 
       NA
  
 
  
 9
 
 130828969
 
 130829320
 
  ENSEMBL   UCSC 
 
  352
 
  4
 
 40721
 
 gene;island
 
 0.79726
 
 0.68204
 
 Not queried
 
  0.8319165
 
       NA
 
       NA
  
 
  
 7
 
  73134065
 
  73134111
 
  ENSEMBL   UCSC 
 
   47
 
  5
 
 37575
 
 tss
 
 0.02904
 
 0.10587
 
 Not queried
 
  0.8315801
 
       NA
 
       NA
  
 
  
 1
 
  28573736
 
  28573940
 
  ENSEMBL   UCSC 
 
  205
 
  4
 
 2934
 
 island
 
 0.01548
 
 0.02686
 
 Not queried
 
  0.8313215
 
       NA
 
       NA
  
 
  
 6
 
  33095664
 
  33096501
 
  ENSEMBL   UCSC 
 
  838
 
  9
 
 34127
 
 gene
 
 0.85994
 
 0.75347
 
 Not queried
 
  0.8312825
 
       NA
 
       NA
  
 
  
 13
 
  37393069
 
  37393246
 
  ENSEMBL   UCSC 
 
  178
 
  6
 
 11370
 
 tss
 
 0.03239
 
 0.02065
 
 Not queried
 
  0.8311973
 
       NA
 
       NA
  
 
  
 12
 
 121890163
 
 121890503
 
  ENSEMBL   UCSC 
 
  341
 
  4
 
 9342
 
 gene;island
 
 0.78865
 
 0.86913
 
 Not queried
 
  0.8311445
 
       NA
 
       NA
  
 
  
 2
 
 130940427
 
 130940706
 
  ENSEMBL   UCSC 
 
  280
 
  5
 
 22829
 
 gene;tss;island
 
 0.16668
 
 0.11926
 
 Not queried
 
  0.8309989
 
       NA
 
       NA
  
 
  
 11
 
  64546243
 
  64546534
 
  ENSEMBL   UCSC 
 
  292
 
  8
 
 7382
 
 tss
 
 0.00499
 
 0.00972
 
 Not queried
 
  0.8309795
 
       NA
 
       NA
  
 
  
 7
 
 158669801
 
 158669978
 
  ENSEMBL   UCSC 
 
  178
 
  5
 
 37316
 
 gene;island
 
 0.89284
 
 0.95434
 
 Not queried
 
  0.8309554
 
       NA
 
       NA
  
 
  
 4
 
  40751717
 
  40752151
 
  ENSEMBL   UCSC 
 
  435
 
  6
 
 30946
 
 island
 
 0.05147
 
 0.12241
 
 Not queried
 
  0.8309519
 
       NA
 
       NA
  
 
  
 10
 
  86088163
 
  86088467
 
  ENSEMBL   UCSC 
 
  305
 
  8
 
 5878
 
 island
 
 0.01782
 
 0.03701
 
 Not queried
 
  0.8308583
 
       NA
 
       NA
  
 
  
 6
 
 114178192
 
 114178507
 
  ENSEMBL   UCSC 
 
  316
 
  9
 
 35121
 
 tss;island
 
 0.01149
 
 0.01639
 
 Not queried
 
  0.8307892
 
       NA
 
       NA
  
 
  
 10
 
   3500383
 
   3500727
 
  ENSEMBL   UCSC 
 
  345
 
  4
 
 5494
 
 island
 
 0.76450
 
 0.64121
 
 Not queried
 
  0.8307828
 
       NA
 
       NA
  
 
  
 1
 
  28157569
 
  28157761
 
  ENSEMBL   UCSC 
 
  193
 
  4
 
 359
 
 gene;island
 
 0.02933
 
 0.05302
 
 Not queried
 
  0.8305882
 
       NA
 
       NA
  
 
  
 17
 
  72428287
 
  72428390
 
  ENSEMBL   UCSC 
 
  104
 
  4
 
 16720
 
 gene;island
 
 0.04572
 
 0.06790
 
 Not queried
 
  0.8305500
 
       NA
 
       NA
  
 
  
 17
 
  17124521
 
  17124969
 
  ENSEMBL   UCSC 
 
  449
 
  6
 
 16426
 
 gene;island
 
 0.87792
 
 0.92140
 
 Not queried
 
  0.8305129
 
       NA
 
       NA
  
 
  
 12
 
  50344211
 
  50344475
 
  ENSEMBL   UCSC 
 
  265
 
  5
 
 9673
 
 tss
 
 0.70219
 
 0.62910
 
 Not queried
 
  0.8304643
 
       NA
 
       NA
  
 
  
 2
 
 105461096
 
 105461392
 
  ENSEMBL   UCSC 
 
  297
 
  4
 
 24539
 
 island
 
 0.01973
 
 0.03896
 
 Not queried
 
  0.8302967
 
       NA
 
       NA
  
 
  
 X
 
 115594157
 
 115594242
 
  ENSEMBL   UCSC 
 
   86
 
  7
 
 41478
 
 tss
 
 0.96147
 
 0.93997
 
 Not queried
 
  0.8301155
 
       NA
 
       NA
  
 
  
 19
 
   1238119
 
   1238538
 
  ENSEMBL   UCSC 
 
  420
 
  6
 
 20257
 
 tss;island
 
 0.19204
 
 0.04021
 
 Not queried
 
  0.8301070
 
       NA
 
       NA
  
 
  
 10
 
  30637833
 
  30638503
 
  ENSEMBL   UCSC 
 
  671
 
  9
 
 5625
 
 island
 
 0.01111
 
 0.02194
 
 Not queried
 
  0.8300129
 
       NA
 
       NA
  
 
  
 2
 
 211090117
 
 211090487
 
  ENSEMBL   UCSC 
 
  371
 
  8
 
 25020
 
 island
 
 0.01253
 
 0.03902
 
 Not queried
 
  0.8299486
 
       NA
 
       NA
  
 
  
 1
 
  46216399
 
  46216737
 
  ENSEMBL   UCSC 
 
  339
 
  6
 
 3202
 
 island
 
 0.06138
 
 0.02639
 
 Not queried
 
  0.8299419
 
       NA
 
       NA
  
 
  
 19
 
  44716462
 
  44716952
 
  ENSEMBL   UCSC 
 
  491
 
  8
 
 22070
 
 island
 
 0.01569
 
 0.03822
 
 Not queried
 
  0.8298338
 
       NA
 
       NA
  
 
  
 19
 
  45866874
 
  45867461
 
  ENSEMBL   UCSC 
 
  588
 
  6
 
 20056
 
 gene;island
 
 0.84181
 
 0.89258
 
 Not queried
 
  0.8298287
 
       NA
 
       NA
  
 
  
 11
 
 115530932
 
 115531299
 
  ENSEMBL   UCSC 
 
  368
 
  5
 
 8806
 
 island
 
 0.05536
 
 0.03292
 
 Not queried
 
  0.8297704
 
       NA
 
       NA
  
 
  
 7
 
  79081666
 
  79081807
 
  ENSEMBL   UCSC 
 
  142
 
  4
 
 37002
 
 gene;island
 
 0.07519
 
 0.03477
 
 Not queried
 
  0.8296482
 
       NA
 
       NA
  
 
  
 4
 
 164253767
 
 164254230
 
  ENSEMBL   UCSC 
 
  464
 
  7
 
 30612
 
 tss
 
 0.02930
 
 0.02841
 
 Not queried
 
  0.8295154
 
       NA
 
       NA
  
 
  
 7
 
  30029231
 
  30029442
 
  ENSEMBL   UCSC 
 
  212
 
  4
 
 36888
 
 gene;island
 
 0.01635
 
 0.02656
 
 Not queried
 
  0.8292166
 
       NA
 
       NA
  
 
  
 6
 
  29588894
 
  29589056
 
  ENSEMBL   UCSC 
 
  163
 
  4
 
 33611
 
 gene
 
 0.88443
 
 0.91088
 
 Not queried
 
  0.8291826
 
       NA
 
       NA
  
 
  
 10
 
  75504193
 
  75504431
 
  ENSEMBL   UCSC 
 
  239
 
  4
 
 4623
 
 gene
 
 0.01677
 
 0.02440
 
 Not queried
 
  0.8291391
 
       NA
 
       NA
  
 
  
 15
 
  35046610
 
  35046765
 
  ENSEMBL   UCSC 
 
  156
 
  4
 
 13972
 
 island
 
 0.18702
 
 0.33446
 
 Not queried
 
  0.8290558
 
       NA
 
       NA
  
 
  
 22
 
  22007095
 
  22007286
 
  ENSEMBL   UCSC 
 
  192
 
  4
 
 26957
 
 tss;island
 
 0.11709
 
 0.03444
 
 Not queried
 
  0.8289992
 
       NA
 
       NA
  
 
  
 19
 
   8373282
 
   8373608
 
  ENSEMBL   UCSC 
 
  327
 
  7
 
 20379
 
 tss
 
 0.01544
 
 0.03575
 
 Not queried
 
  0.8289200
 
       NA
 
       NA
  
 
  
 1
 
  36689760
 
  36690116
 
  ENSEMBL   UCSC 
 
  357
 
  7
 
 3046
 
 island
 
 0.01351
 
 0.01827
 
 Not queried
 
  0.8286784
 
       NA
 
       NA
  
 
  
 6
 
  29697700
 
  29697949
 
  ENSEMBL   UCSC 
 
  250
 
  4
 
 33625
 
 gene
 
 0.69059
 
 0.68958
 
 Not queried
 
  0.8285574
 
       NA
 
       NA
  
 
  
 6
 
  36646473
 
  36646551
 
  ENSEMBL   UCSC 
 
   79
 
  5
 
 34252
 
 gene
 
 0.06214
 
 0.10713
 
 Not queried
 
  0.8285489
 
       NA
 
       NA
  
 
  
 10
 
   1228893
 
   1229080
 
  ENSEMBL   UCSC 
 
  188
 
  4
 
 4444
 
 gene;island
 
 0.82400
 
 0.87724
 
 Not queried
 
  0.8284766
 
       NA
 
       NA
  
 
  
 4
 
 134070039
 
 134070441
 
  ENSEMBL   UCSC 
 
  403
 
  8
 
 30546
 
 tss
 
 0.01168
 
 0.02233
 
 Not queried
 
  0.8283799
 
       NA
 
       NA
  
 
  
 20
 
   2082199
 
   2082453
 
  ENSEMBL   UCSC 
 
  255
 
  9
 
 25502
 
 tss
 
 0.02158
 
 0.03589
 
 Not queried
 
  0.8283053
 
       NA
 
       NA
  
 
  
 17
 
  72442928
 
  72443179
 
  ENSEMBL   UCSC 
 
  252
 
  5
 
 16722
 
 gene;island
 
 0.81867
 
 0.71039
 
 Not queried
 
  0.8281907
 
       NA
 
       NA
  
 
  
 20
 
  62737818
 
  62738358
 
  ENSEMBL   UCSC 
 
  541
 
  8
 
 26345
 
 island
 
 0.55949
 
 0.68724
 
 Not queried
 
  0.8281830
 
       NA
 
       NA
  
 
  
 19
 
  17420039
 
  17420304
 
  ENSEMBL   UCSC 
 
  266
 
  7
 
 20551
 
 tss
 
 0.02013
 
 0.03514
 
 Not queried
 
  0.8280538
 
       NA
 
       NA
  
 
  
 2
 
 127783082
 
 127783370
 
  ENSEMBL   UCSC 
 
  289
 
  4
 
 24645
 
 island
 
 0.01712
 
 0.02664
 
 Not queried
 
  0.8279510
 
       NA
 
       NA
  
 
  
 1
 
  16553267
 
  16553549
 
  ENSEMBL   UCSC 
 
  283
 
  5
 
 2735
 
 island
 
 0.23959
 
 0.33413
 
 Not queried
 
  0.8279311
 
       NA
 
       NA
  
 
  
 13
 
  48669116
 
  48669516
 
  ENSEMBL   UCSC 
 
  401
 
  8
 
 11692
 
 island
 
 0.08485
 
 0.16380
 
 Not queried
 
  0.8278786
 
       NA
 
       NA
  
 
  
 8
 
   9763552
 
   9764073
 
  ENSEMBL   UCSC 
 
  522
 
  5
 
 40002
 
 island
 
 0.00621
 
 0.03558
 
 Not queried
 
  0.8278191
 
       NA
 
       NA
  
 
  
 12
 
  72666976
 
  72667707
 
  ENSEMBL   UCSC 
 
  732
 
 10
 
 9193
 
 gene;island
 
 0.10231
 
 0.23183
 
 Not queried
 
  0.8277749
 
       NA
 
       NA
  
 
  
 19
 
  19373566
 
  19373755
 
  ENSEMBL   UCSC 
 
  190
 
  5
 
 21774
 
 island
 
 0.58939
 
 0.42359
 
 Not queried
 
  0.8277539
 
       NA
 
       NA
  
 
  
 1
 
 190447232
 
 190447640
 
  ENSEMBL   UCSC 
 
  409
 
  5
 
 2143
 
 tss
 
 0.03189
 
 0.03341
 
 Not queried
 
  0.8275985
 
       NA
 
       NA
  
 
  
 3
 
 111805225
 
 111805408
 
  ENSEMBL   UCSC 
 
  184
 
  6
 
 27852
 
 gene
 
 0.02298
 
 0.04231
 
 Not queried
 
  0.8273907
 
       NA
 
       NA
  
 
  
 1
 
  29508658
 
  29508888
 
  ENSEMBL   UCSC 
 
  231
 
  6
 
 1437
 
 tss
 
 0.02243
 
 0.04604
 
 Not queried
 
  0.8273494
 
       NA
 
       NA
  
 
  
 16
 
  83841324
 
  83841917
 
  ENSEMBL   UCSC 
 
  594
 
  8
 
 16097
 
 island
 
 0.02082
 
 0.03757
 
 Not queried
 
  0.8273409
 
       NA
 
       NA
  
 
  
 17
 
  56065813
 
  56066027
 
  ENSEMBL   UCSC 
 
  215
 
  4
 
 17496
 
 tss;island
 
 0.02659
 
 0.05024
 
 Not queried
 
  0.8272738
 
       NA
 
       NA
  
 
  
 17
 
  33701321
 
  33701529
 
  ENSEMBL   UCSC 
 
  209
 
  4
 
 17235
 
 tss;island
 
 0.09197
 
 0.20204
 
 Not queried
 
  0.8271933
 
       NA
 
       NA
  
 
  
 20
 
  61810348
 
  61810902
 
  ENSEMBL   UCSC 
 
  555
 
  5
 
 26303
 
 island
 
 0.03420
 
 0.04260
 
 Not queried
 
  0.8271914
 
       NA
 
       NA
  
 
  
 6
 
  79943342
 
  79943592
 
  ENSEMBL   UCSC 
 
  251
 
  4
 
 34333
 
 gene;island
 
 0.01981
 
 0.00920
 
 Not queried
 
  0.8270534
 
       NA
 
       NA
  
 
  
 2
 
 133175192
 
 133175397
 
  ENSEMBL   UCSC 
 
  206
 
  4
 
 22838
 
 gene;island
 
 0.91318
 
 0.81785
 
 Not queried
 
  0.8268955
 
       NA
 
       NA
  
 
  
 19
 
  36024217
 
  36024439
 
  ENSEMBL   UCSC 
 
  223
 
  8
 
 21870
 
 island
 
 0.90117
 
 0.80392
 
 Not queried
 
  0.8268481
 
       NA
 
       NA
  
 
  
 6
 
  32632565
 
  32633163
 
  ENSEMBL   UCSC 
 
  599
 
 21
 
 34060
 
 gene;island
 
 0.51545
 
 0.40606
 
 Not queried
 
  0.8268471
 
       NA
 
       NA
  
 
  
 17
 
  35870381
 
  35870705
 
  ENSEMBL   UCSC 
 
  325
 
  5
 
 16498
 
 gene;island
 
 0.86609
 
 0.91626
 
 Not queried
 
  0.8268094
 
       NA
 
       NA
  
 
  
 1
 
 100731266
 
 100731936
 
  ENSEMBL   UCSC 
 
  671
 
  9
 
 3517
 
 island
 
 0.02751
 
 0.03692
 
 Not queried
 
  0.8267263
 
       NA
 
       NA
  
 
  
 1
 
  85931072
 
  85931301
 
  ENSEMBL   UCSC 
 
  230
 
  7
 
 605
 
 gene;tss;island
 
 0.02684
 
 0.03957
 
 Not queried
 
  0.8264858
 
       NA
 
       NA
  
 
  
 11
 
 118042453
 
 118042630
 
  ENSEMBL   UCSC 
 
  178
 
  4
 
 6906
 
 gene
 
 0.01982
 
 0.03791
 
 Not queried
 
  0.8264206
 
       NA
 
       NA
  
 
  
 12
 
  21654197
 
  21654546
 
  ENSEMBL   UCSC 
 
  350
 
  6
 
 9052
 
 gene
 
 0.01335
 
 0.02200
 
 Not queried
 
  0.8263981
 
       NA
 
       NA
  
 
  
 2
 
  20424975
 
  20425000
 
  ENSEMBL   UCSC 
 
   26
 
  4
 
 23172
 
 tss
 
 0.01794
 
 0.02781
 
 Not queried
 
  0.8262309
 
       NA
 
       NA
  
 
  
 15
 
  78631512
 
  78631652
 
  ENSEMBL   UCSC 
 
  141
 
  4
 
 13805
 
 tss;island
 
 0.88106
 
 0.86391
 
 Not queried
 
  0.8261784
 
       NA
 
       NA
  
 
  
 17
 
  46184579
 
  46185283
 
  ENSEMBL   UCSC 
 
  705
 
 11
 
 18437
 
 island
 
 0.00746
 
 0.01889
 
 Not queried
 
  0.8261668
 
       NA
 
       NA
  
 
  
 15
 
  65809563
 
  65809771
 
  ENSEMBL   UCSC 
 
  209
 
  4
 
 13352
 
 gene;island
 
 0.04308
 
 0.04731
 
 Not queried
 
  0.8260561
 
       NA
 
       NA
  
 
  
 7
 
  42971956
 
  42972593
 
  ENSEMBL   UCSC 
 
  638
 
  9
 
 36916
 
 gene
 
 0.03303
 
 0.04734
 
 Not queried
 
  0.8259995
 
       NA
 
       NA
  
 
  
 17
 
  65850328
 
  65850546
 
  ENSEMBL   UCSC 
 
  219
 
  4
 
 16703
 
 gene
 
 0.97043
 
 0.97847
 
 Not queried
 
  0.8259849
 
       NA
 
       NA
  
 
  
 11
 
    790142
 
    790293
 
  ENSEMBL   UCSC 
 
  152
 
  6
 
 7007
 
 tss;island
 
 0.44057
 
 0.55428
 
 Not queried
 
  0.8258297
 
       NA
 
       NA
  
 
  
 19
 
  38893816
 
  38894173
 
  ENSEMBL   UCSC 
 
  358
 
  4
 
 19982
 
 gene
 
 0.02590
 
 0.04408
 
 Not queried
 
  0.8256568
 
       NA
 
       NA
  
 
  
 15
 
  25425326
 
  25425615
 
  ENSEMBL   UCSC 
 
  290
 
  4
 
 13498
 
 tss
 
 0.56320
 
 0.64868
 
 Not queried
 
  0.8254296
 
       NA
 
       NA
  
 
  
 3
 
  57261306
 
  57261572
 
  ENSEMBL   UCSC 
 
  267
 
  5
 
 28365
 
 tss;island
 
 0.01038
 
 0.02079
 
 Not queried
 
  0.8252522
 
       NA
 
       NA
  
 
  
 2
 
  46925970
 
  46926272
 
  ENSEMBL   UCSC 
 
  303
 
  5
 
 23301
 
 tss
 
 0.01770
 
 0.02316
 
 Not queried
 
  0.8251479
 
       NA
 
       NA
  
 
  
 19
 
   1799935
 
   1800331
 
  ENSEMBL   UCSC 
 
  397
 
  5
 
 19626
 
 gene;island
 
 0.76115
 
 0.90288
 
 Not queried
 
  0.8248715
 
       NA
 
       NA
  
 
  
 6
 
  31628639
 
  31629199
 
  ENSEMBL   UCSC 
 
  561
 
 10
 
 34762
 
 tss
 
 0.02493
 
 0.03991
 
 Not queried
 
  0.8248340
 
       NA
 
       NA
  
 
  
 5
 
    500202
 
    500565
 
  ENSEMBL   UCSC 
 
  364
 
  5
 
 31455
 
 gene;island
 
 0.88083
 
 0.78285
 
 Not queried
 
  0.8246769
 
       NA
 
       NA
  
 
  
 X
 
 152066574
 
 152066845
 
  ENSEMBL   UCSC 
 
  272
 
  6
 
 42104
 
 island
 
 0.01067
 
 0.02629
 
 Not queried
 
  0.8245659
 
       NA
 
       NA
  
 
  
 1
 
  25228582
 
  25229192
 
  ENSEMBL   UCSC 
 
  611
 
  9
 
 328
 
 gene;island
 
 0.92177
 
 0.89349
 
 Not queried
 
  0.8245485
 
       NA
 
       NA
  
 
  
 19
 
   2607726
 
   2607903
 
  ENSEMBL   UCSC 
 
  178
 
  4
 
 19661
 
 gene;island
 
 0.56501
 
 0.74546
 
 Not queried
 
  0.8245443
 
       NA
 
       NA
  
 
  
 14
 
  35452110
 
  35452602
 
  ENSEMBL   UCSC 
 
  493
 
  6
 
 12032
 
 gene
 
 0.02527
 
 0.03681
 
 Not queried
 
  0.8244295
 
       NA
 
       NA
  
 
  
 11
 
  61659326
 
  61659518
 
  ENSEMBL   UCSC 
 
  193
 
  4
 
 7321
 
 tss;island
 
 0.06584
 
 0.05004
 
 Not queried
 
  0.8244186
 
       NA
 
       NA
  
 
  
 1
 
  26880767
 
  26881009
 
  ENSEMBL   UCSC 
 
  243
 
  4
 
 342
 
 gene;tss
 
 0.61494
 
 0.69000
 
 Not queried
 
  0.8243157
 
       NA
 
       NA
  
 
  
 1
 
   1998716
 
   1999183
 
  ENSEMBL   UCSC 
 
  468
 
  4
 
 55
 
 gene
 
 0.88365
 
 0.93801
 
 Not queried
 
  0.8242717
 
       NA
 
       NA
  
 
  
 X
 
 151806225
 
 151806923
 
  ENSEMBL   UCSC 
 
  699
 
 10
 
 42102
 
 island
 
 0.01951
 
 0.03599
 
 Not queried
 
  0.8242321
 
       NA
 
       NA
  
 
  
 15
 
  50647051
 
  50647267
 
  ENSEMBL   UCSC 
 
  217
 
  7
 
 13316
 
 gene;island
 
 0.01907
 
 0.02666
 
 Not queried
 
  0.8241168
 
       NA
 
       NA
  
 
  
 17
 
  73761301
 
  73761710
 
  ENSEMBL   UCSC 
 
  410
 
  8
 
 17627
 
 tss
 
 0.04522
 
 0.04643
 
 Not queried
 
  0.8240383
 
       NA
 
       NA
  
 
  
 8
 
  98656054
 
  98656329
 
  ENSEMBL   UCSC 
 
  276
 
  7
 
 39766
 
 tss;island
 
 0.01695
 
 0.03689
 
 Not queried
 
  0.8240041
 
       NA
 
       NA
  
 
  
 20
 
  44746392
 
  44746902
 
  ENSEMBL   UCSC 
 
  511
 
  9
 
 25750
 
 tss
 
 0.03529
 
 0.06481
 
 Not queried
 
  0.8239427
 
       NA
 
       NA
  
 
  
 12
 
  57636980
 
  57637690
 
  ENSEMBL   UCSC 
 
  711
 
  7
 
 10530
 
 island
 
 0.02785
 
 0.03594
 
 Not queried
 
  0.8237831
 
       NA
 
       NA
  
 
  
 X
 
  12992684
 
  12993214
 
  ENSEMBL   UCSC 
 
  531
 
  8
 
 41188
 
 tss;island
 
 0.02953
 
 0.08093
 
 Not queried
 
  0.8237301
 
       NA
 
       NA
  
 
  
 17
 
  57642628
 
  57642873
 
  ENSEMBL   UCSC 
 
  246
 
  8
 
 17513
 
 tss
 
 0.17378
 
 0.05997
 
 Not queried
 
  0.8236962
 
       NA
 
       NA
  
 
  
 17
 
   2952096
 
   2952533
 
  ENSEMBL   UCSC 
 
  438
 
  6
 
 17818
 
 island
 
 0.01323
 
 0.02770
 
 Not queried
 
  0.8234933
 
       NA
 
       NA
  
 
  
 5
 
 118690955
 
 118691126
 
  ENSEMBL   UCSC 
 
  172
 
  5
 
 31667
 
 gene;tss;island
 
 0.05095
 
 0.05268
 
 Not queried
 
  0.8234433
 
       NA
 
       NA
  
 
  
 4
 
   3250845
 
   3251386
 
  ENSEMBL   UCSC 
 
  542
 
  5
 
 29856
 
 gene
 
 0.89978
 
 0.89606
 
 Not queried
 
  0.8231010
 
       NA
 
       NA
  
 
  
 16
 
  15149995
 
  15150255
 
  ENSEMBL   UCSC 
 
  261
 
  5
 
 15074
 
 tss;island
 
 0.03348
 
 0.04612
 
 Not queried
 
  0.8230990
 
       NA
 
       NA
  
 
  
 1
 
  41445556
 
  41445888
 
  ENSEMBL   UCSC 
 
  333
 
  4
 
 458
 
 gene;island
 
 0.01871
 
 0.02061
 
 Not queried
 
  0.8229858
 
       NA
 
       NA
  
 
  
 1
 
  27693782
 
  27693942
 
  ENSEMBL   UCSC 
 
  161
 
  4
 
 1406
 
 tss;island
 
 0.41354
 
 0.32342
 
 Not queried
 
  0.8227353
 
       NA
 
       NA
  
 
  
 20
 
    524484
 
    524791
 
  ENSEMBL   UCSC 
 
  308
 
  8
 
 25488
 
 tss
 
 0.00911
 
 0.01426
 
 Not queried
 
  0.8225043
 
       NA
 
       NA
  
 
  
 3
 
  52445096
 
  52445239
 
  ENSEMBL   UCSC 
 
  144
 
  4
 
 27777
 
 gene;tss;island
 
 0.01036
 
 0.02225
 
 Not queried
 
  0.8224797
 
       NA
 
       NA
  
 
  
 16
 
   9857475
 
   9857925
 
  ENSEMBL   UCSC 
 
  451
 
  4
 
 14627
 
 gene
 
 0.73390
 
 0.85945
 
 Not queried
 
  0.8224594
 
       NA
 
       NA
  
 
  
 3
 
  51428372
 
  51428651
 
  ENSEMBL   UCSC 
 
  280
 
  7
 
 28321
 
 tss;island
 
 0.02977
 
 0.05291
 
 Not queried
 
  0.8223285
 
       NA
 
       NA
  
 
  
 19
 
  10812111
 
  10812419
 
  ENSEMBL   UCSC 
 
  309
 
  4
 
 19785
 
 gene
 
 0.01890
 
 0.02624
 
 Not queried
 
  0.8223044
 
       NA
 
       NA
  
 
  
 17
 
  33468875
 
  33469488
 
  ENSEMBL   UCSC 
 
  614
 
  7
 
 18175
 
 island
 
 0.01301
 
 0.01644
 
 Not queried
 
  0.8222571
 
       NA
 
       NA
  
 
  
 4
 
 174443242
 
 174443567
 
  ENSEMBL   UCSC 
 
  326
 
  4
 
 31373
 
 island
 
 0.05281
 
 0.03955
 
 Not queried
 
  0.8221746
 
       NA
 
       NA
  
 
  
 1
 
  92001033
 
  92001429
 
  ENSEMBL   UCSC 
 
  397
 
  4
 
 3467
 
 island
 
 0.93888
 
 0.96243
 
 Not queried
 
  0.8221100
 
       NA
 
       NA
  
 
  
 1
 
 224180288
 
 224180516
 
  ENSEMBL   UCSC 
 
  229
 
  4
 
 4226
 
 island
 
 0.29865
 
 0.37168
 
 Not queried
 
  0.8220323
 
       NA
 
       NA
  
 
  
 11
 
 101917847
 
 101918171
 
  ENSEMBL   UCSC 
 
  325
 
  7
 
 7623
 
 tss
 
 0.03583
 
 0.03902
 
 Not queried
 
  0.8219123
 
       NA
 
       NA
  
 
  
 6
 
  56911139
 
  56911540
 
  ENSEMBL   UCSC 
 
  402
 
  7
 
 36161
 
 island
 
 0.01251
 
 0.01574
 
 Not queried
 
  0.8218865
 
       NA
 
       NA
  
 
  
 3
 
  49907297
 
  49907692
 
  ENSEMBL   UCSC 
 
  396
 
  7
 
 29089
 
 island
 
 0.15956
 
 0.09699
 
 Not queried
 
  0.8218622
 
       NA
 
       NA
  
 
  
 5
 
  94955575
 
  94955893
 
  ENSEMBL   UCSC 
 
  319
 
  6
 
 32125
 
 tss
 
 0.03815
 
 0.02979
 
 Not queried
 
  0.8218177
 
       NA
 
       NA
  
 
  
 1
 
 220263509
 
 220263698
 
  ENSEMBL   UCSC 
 
  190
 
  4
 
 2255
 
 tss;island
 
 0.01531
 
 0.02506
 
 Not queried
 
  0.8214464
 
       NA
 
       NA
  
 
  
 4
 
 184908254
 
 184909018
 
  ENSEMBL   UCSC 
 
  765
 
  9
 
 30149
 
 gene
 
 0.05577
 
 0.13466
 
 Not queried
 
  0.8212767
 
       NA
 
       NA
  
 
  
 12
 
  93861141
 
  93861583
 
  ENSEMBL   UCSC 
 
  443
 
  7
 
 10647
 
 island
 
 0.01283
 
 0.02153
 
 Not queried
 
  0.8210675
 
       NA
 
       NA
  
 
  
 12
 
  89746101
 
  89746771
 
  ENSEMBL   UCSC 
 
  671
 
 10
 
 10637
 
 island
 
 0.01996
 
 0.02874
 
 Not queried
 
  0.8209899
 
       NA
 
       NA
  
 
  
 17
 
  34947837
 
  34948351
 
  ENSEMBL   UCSC 
 
  515
 
  9
 
 18197
 
 island
 
 0.03629
 
 0.04340
 
 Not queried
 
  0.8209792
 
       NA
 
       NA
  
 
  
 3
 
 196295218
 
 196295526
 
  ENSEMBL   UCSC 
 
  309
 
  7
 
 28772
 
 tss;island
 
 0.01668
 
 0.02920
 
 Not queried
 
  0.8207936
 
       NA
 
       NA
  
 
  
 4
 
  55095148
 
  55095261
 
  ENSEMBL   UCSC 
 
  114
 
  4
 
 30354
 
 tss
 
 0.17910
 
 0.13562
 
 Not queried
 
  0.8207208
 
       NA
 
       NA
  
 
  
 1
 
  88928168
 
  88928839
 
  ENSEMBL   UCSC 
 
  672
 
  6
 
 3436
 
 island
 
 0.14009
 
 0.05308
 
 Not queried
 
  0.8204768
 
       NA
 
       NA
  
 
  
 1
 
  86622395
 
  86622737
 
  ENSEMBL   UCSC 
 
  343
 
  6
 
 1722
 
 tss;island
 
 0.03168
 
 0.02509
 
 Not queried
 
  0.8203369
 
       NA
 
       NA
  
 
  
 19
 
  10828487
 
  10829071
 
  ENSEMBL   UCSC 
 
  585
 
 12
 
 20422
 
 tss
 
 0.01021
 
 0.02227
 
 Not queried
 
  0.8201580
 
       NA
 
       NA
  
 
  
 2
 
  20424804
 
  20425000
 
  ENSEMBL   UCSC 
 
  197
 
  5
 
 22552
 
 gene;island
 
 0.02336
 
 0.02798
 
 Not queried
 
  0.8200755
 
       NA
 
       NA
  
 
  
 20
 
  37353032
 
  37353096
 
  ENSEMBL   UCSC 
 
   65
 
  4
 
 25699
 
 tss
 
 0.02256
 
 0.03659
 
 Not queried
 
  0.8200104
 
       NA
 
       NA
  
 
  
 2
 
 242127690
 
 242127852
 
  ENSEMBL   UCSC 
 
  163
 
  4
 
 23948
 
 tss
 
 0.72315
 
 0.86168
 
 Not queried
 
  0.8199188
 
       NA
 
       NA
  
 
  
 17
 
  61988667
 
  61988811
 
  ENSEMBL   UCSC 
 
  145
 
  4
 
 17543
 
 tss
 
 0.43458
 
 0.31739
 
 Not queried
 
  0.8199078
 
       NA
 
       NA
  
 
  
 8
 
   1495025
 
   1495332
 
  ENSEMBL   UCSC 
 
  308
 
  5
 
 39103
 
 gene;island
 
 0.91395
 
 0.86986
 
 Not queried
 
  0.8197491
 
       NA
 
       NA
  
 
  
 6
 
  33053576
 
  33054001
 
  ENSEMBL   UCSC 
 
  426
 
  6
 
 34122
 
 gene
 
 0.84116
 
 0.69915
 
 Not queried
 
  0.8196312
 
       NA
 
       NA
  
 
  
 10
 
  42970987
 
  42971251
 
  ENSEMBL   UCSC 
 
  265
 
  4
 
 4551
 
 gene
 
 0.16347
 
 0.18501
 
 Not queried
 
  0.8195477
 
       NA
 
       NA
  
 
  
 5
 
 175223225
 
 175223646
 
  ENSEMBL   UCSC 
 
  422
 
  6
 
 33357
 
 island
 
 0.34280
 
 0.14873
 
 Not queried
 
  0.8195036
 
       NA
 
       NA
  
 
  
 2
 
 120125096
 
 120125452
 
  ENSEMBL   UCSC 
 
  357
 
  6
 
 22808
 
 gene;tss;island
 
 0.03986
 
 0.03615
 
 Not queried
 
  0.8193777
 
       NA
 
       NA
  
 
  
 19
 
  41869545
 
  41869851
 
  ENSEMBL   UCSC 
 
  307
 
  6
 
 20771
 
 tss
 
 0.01384
 
 0.02185
 
 Not queried
 
  0.8192841
 
       NA
 
       NA
  
 
  
 6
 
 157472027
 
 157472164
 
  ENSEMBL   UCSC 
 
  138
 
  4
 
 34457
 
 gene
 
 0.91812
 
 0.96345
 
 Not queried
 
  0.8192217
 
       NA
 
       NA
  
 
  
 13
 
 111290667
 
 111290952
 
  ENSEMBL   UCSC 
 
  286
 
  5
 
 11206
 
 gene;island
 
 0.86536
 
 0.94570
 
 Not queried
 
  0.8191067
 
       NA
 
       NA
  
 
  
 7
 
 157199417
 
 157199595
 
  ENSEMBL   UCSC 
 
  179
 
  4
 
 37258
 
 gene;island
 
 0.97166
 
 0.93920
 
 Not queried
 
  0.8190948
 
       NA
 
       NA
  
 
  
 2
 
 219906245
 
 219906468
 
  ENSEMBL   UCSC 
 
  224
 
  7
 
 23851
 
 tss
 
 0.01104
 
 0.01580
 
 Not queried
 
  0.8190553
 
       NA
 
       NA
  
 
  
 3
 
 195622522
 
 195622598
 
  ENSEMBL   UCSC 
 
   77
 
  4
 
 28053
 
 gene;tss;island
 
 0.04195
 
 0.03662
 
 Not queried
 
  0.8189706
 
       NA
 
       NA
  
 
  
 14
 
  36989287
 
  36989588
 
  ENSEMBL   UCSC 
 
  302
 
  6
 
 12330
 
 tss;island
 
 0.01778
 
 0.04149
 
 Not queried
 
  0.8189559
 
       NA
 
       NA
  
 
  
 22
 
  25159879
 
  25160098
 
  ENSEMBL   UCSC 
 
  220
 
  4
 
 26776
 
 gene;tss;island
 
 0.89195
 
 0.93759
 
 Not queried
 
  0.8188486
 
       NA
 
       NA
  
 
  
 5
 
  56206110
 
  56206289
 
  ENSEMBL   UCSC 
 
  180
 
  4
 
 31564
 
 gene;island
 
 0.01928
 
 0.04699
 
 Not queried
 
  0.8188197
 
       NA
 
       NA
  
 
  
 11
 
  31391061
 
  31391307
 
  ENSEMBL   UCSC 
 
  247
 
  8
 
 6534
 
 gene
 
 0.01623
 
 0.02745
 
 Not queried
 
  0.8185861
 
       NA
 
       NA
  
 
  
 2
 
 201390524
 
 201390889
 
  ENSEMBL   UCSC 
 
  366
 
  4
 
 24963
 
 island
 
 0.01831
 
 0.01767
 
 Not queried
 
  0.8185848
 
       NA
 
       NA
  
 
  
 11
 
   6640720
 
   6640996
 
  ENSEMBL   UCSC 
 
  277
 
  4
 
 7096
 
 tss
 
 0.01601
 
 0.02831
 
 Not queried
 
  0.8184802
 
       NA
 
       NA
  
 
  
 19
 
  35085512
 
  35085677
 
  ENSEMBL   UCSC 
 
  166
 
  4
 
 20657
 
 tss
 
 0.86038
 
 0.78035
 
 Not queried
 
  0.8183693
 
       NA
 
       NA
  
 
  
 3
 
  53164576
 
  53164697
 
  ENSEMBL   UCSC 
 
  122
 
  6
 
 28353
 
 tss
 
 0.01069
 
 0.01763
 
 Not queried
 
  0.8181700
 
       NA
 
       NA
  
 
  
 3
 
  53164314
 
  53164697
 
  ENSEMBL   UCSC 
 
  384
 
 10
 
 29154
 
 island
 
 0.01069
 
 0.01908
 
 Not queried
 
  0.8181700
 
       NA
 
       NA
  
 
  
 20
 
  57465439
 
  57466137
 
  ENSEMBL   UCSC 
 
  699
 
 22
 
 25437
 
 gene;tss;island
 
 0.01980
 
 0.02360
 
 Not queried
 
  0.8181613
 
       NA
 
       NA
  
 
  
 7
 
 155573684
 
 155573868
 
  ENSEMBL   UCSC 
 
  185
 
  4
 
 37248
 
 gene;island
 
 0.86590
 
 0.89041
 
 Not queried
 
  0.8180867
 
       NA
 
       NA
  
 
  
 19
 
  18717638
 
  18717783
 
  ENSEMBL   UCSC 
 
  146
 
  6
 
 21752
 
 island
 
 0.03806
 
 0.06011
 
 Not queried
 
  0.8178853
 
       NA
 
       NA
  
 
  
 19
 
  51568260
 
  51568523
 
  ENSEMBL   UCSC 
 
  264
 
  4
 
 22302
 
 island
 
 0.02846
 
 0.04491
 
 Not queried
 
  0.8178642
 
       NA
 
       NA
  
 
  
 13
 
  45992445
 
  45992673
 
  ENSEMBL   UCSC 
 
  229
 
  7
 
 11682
 
 island
 
 0.01935
 
 0.02277
 
 Not queried
 
  0.8178319
 
       NA
 
       NA
  
 
  
 17
 
  40718810
 
  40719026
 
  ENSEMBL   UCSC 
 
  217
 
  8
 
 17333
 
 tss;island
 
 0.03229
 
 0.07083
 
 Not queried
 
  0.8177680
 
       NA
 
       NA
  
 
  
 7
 
  39772772
 
  39773291
 
  ENSEMBL   UCSC 
 
  520
 
 11
 
 38364
 
 island
 
 0.02815
 
 0.04519
 
 Not queried
 
  0.8177503
 
       NA
 
       NA
  
 
  
 18
 
  74691097
 
  74691423
 
  ENSEMBL   UCSC 
 
  327
 
  4
 
 19098
 
 gene;island
 
 0.90860
 
 0.94116
 
 Not queried
 
  0.8177396
 
       NA
 
       NA
  
 
  
 11
 
    612588
 
    612837
 
  ENSEMBL   UCSC 
 
  250
 
  4
 
 6365
 
 gene;island
 
 0.83340
 
 0.92455
 
 Not queried
 
  0.8175008
 
       NA
 
       NA
  
 
  
 1
 
 173445917
 
 173446516
 
  ENSEMBL   UCSC 
 
  600
 
 10
 
 3957
 
 island
 
 0.03524
 
 0.04721
 
 Not queried
 
  0.8174804
 
       NA
 
       NA
  
 
  
 18
 
  67955861
 
  67955960
 
  ENSEMBL   UCSC 
 
  100
 
  4
 
 19283
 
 tss;island
 
 0.04045
 
 0.03173
 
 Not queried
 
  0.8174549
 
       NA
 
       NA
  
 
  
 3
 
 107646471
 
 107647202
 
  ENSEMBL   UCSC 
 
  732
 
  7
 
 27845
 
 gene;island
 
 0.02351
 
 0.01874
 
 Not queried
 
  0.8173675
 
       NA
 
       NA
  
 
  
 11
 
    842517
 
    842977
 
  ENSEMBL   UCSC 
 
  461
 
 11
 
 7012
 
 tss;island
 
 0.01663
 
 0.02761
 
 Not queried
 
  0.8173091
 
       NA
 
       NA
  
 
  
 20
 
  32899771
 
  32899839
 
  ENSEMBL   UCSC 
 
   69
 
  5
 
 25651
 
 tss
 
 0.03459
 
 0.03515
 
 Not queried
 
  0.8172736
 
       NA
 
       NA
  
 
  
 1
 
  42384310
 
  42384647
 
  ENSEMBL   UCSC 
 
  338
 
  6
 
 3136
 
 island
 
 0.02953
 
 0.05073
 
 Not queried
 
  0.8171992
 
       NA
 
       NA
  
 
  
 12
 
 131356526
 
 131356676
 
  ENSEMBL   UCSC 
 
  151
 
  5
 
 10981
 
 island
 
 0.02473
 
 0.03905
 
 Not queried
 
  0.8171905
 
       NA
 
       NA
  
 
  
 5
 
 131892242
 
 131892723
 
  ENSEMBL   UCSC 
 
  482
 
  7
 
 33031
 
 island
 
 0.46355
 
 0.78940
 
 Not queried
 
  0.8171406
 
       NA
 
       NA
  
 
  
 6
 
  31927895
 
  31928395
 
  ENSEMBL   UCSC 
 
  501
 
  6
 
 33938
 
 gene;island
 
 0.77617
 
 0.90032
 
 Not queried
 
  0.8169986
 
       NA
 
       NA
  
 
  
 14
 
  21852428
 
  21852744
 
  ENSEMBL   UCSC 
 
  317
 
  6
 
 12255
 
 tss;island
 
 0.02419
 
 0.03340
 
 Not queried
 
  0.8168155
 
       NA
 
       NA
  
 
  
 1
 
   6269260
 
   6269635
 
  ENSEMBL   UCSC 
 
  376
 
  4
 
 162
 
 gene;island
 
 0.03642
 
 0.07952
 
 Not queried
 
  0.8168086
 
       NA
 
       NA
  
 
  
 14
 
  78226708
 
  78227346
 
  ENSEMBL   UCSC 
 
  639
 
  6
 
 12129
 
 gene;island
 
 0.01410
 
 0.02257
 
 Not queried
 
  0.8165503
 
       NA
 
       NA
  
 
  
 19
 
  18632784
 
  18633063
 
  ENSEMBL   UCSC 
 
  280
 
  6
 
 21746
 
 island
 
 0.00781
 
 0.01502
 
 Not queried
 
  0.8165288
 
       NA
 
       NA
  
 
  
 6
 
 100050480
 
 100051116
 
  ENSEMBL   UCSC 
 
  637
 
  8
 
 36294
 
 island
 
 0.02497
 
 0.02957
 
 Not queried
 
  0.8163878
 
       NA
 
       NA
  
 
  
 5
 
  68530331
 
  68530605
 
  ENSEMBL   UCSC 
 
  275
 
  7
 
 32049
 
 tss
 
 0.01896
 
 0.01932
 
 Not queried
 
  0.8163159
 
       NA
 
       NA
  
 
  
 1
 
   3213431
 
   3213790
 
  ENSEMBL   UCSC 
 
  360
 
  4
 
 117
 
 gene
 
 0.63403
 
 0.80706
 
 Not queried
 
  0.8163096
 
       NA
 
       NA
  
 
  
 6
 
  12290322
 
  12290473
 
  ENSEMBL   UCSC 
 
  152
 
  6
 
 34575
 
 tss
 
 0.01289
 
 0.02258
 
 Not queried
 
  0.8162115
 
       NA
 
       NA
  
 
  
 15
 
  63889093
 
  63889711
 
  ENSEMBL   UCSC 
 
  619
 
  6
 
 14181
 
 island
 
 0.59897
 
 0.72230
 
 Not queried
 
  0.8161363
 
       NA
 
       NA
  
 
  
 12
 
  95467350
 
  95467612
 
  ENSEMBL   UCSC 
 
  263
 
  6
 
 10657
 
 island
 
 0.02449
 
 0.03646
 
 Not queried
 
  0.8161357
 
       NA
 
       NA
  
 
  
 17
 
  73727435
 
  73728061
 
  ENSEMBL   UCSC 
 
  627
 
  6
 
 16742
 
 gene;island
 
 0.75983
 
 0.87501
 
 Not queried
 
  0.8159737
 
       NA
 
       NA
  
 
  
 X
 
 151806225
 
 151806628
 
  ENSEMBL   UCSC 
 
  404
 
  7
 
 41571
 
 tss
 
 0.01860
 
 0.03019
 
 Not queried
 
  0.8159497
 
       NA
 
       NA
  
 
  
 14
 
  67982146
 
  67982198
 
  ENSEMBL   UCSC 
 
   53
 
  6
 
 12443
 
 tss;island
 
 0.01712
 
 0.02030
 
 Not queried
 
  0.8157964
 
       NA
 
       NA
  
 
  
 X
 
  75647859
 
  75648222
 
  ENSEMBL   UCSC 
 
  364
 
  5
 
 41881
 
 island
 
 0.05015
 
 0.03500
 
 Not queried
 
  0.8157694
 
       NA
 
       NA
  
 
  
 2
 
 219255879
 
 219256187
 
  ENSEMBL   UCSC 
 
  309
 
  4
 
 22979
 
 gene;island
 
 0.90223
 
 0.79924
 
 Not queried
 
  0.8157410
 
       NA
 
       NA
  
 
  
 19
 
   1510494
 
   1510692
 
  ENSEMBL   UCSC 
 
  199
 
  4
 
 19622
 
 gene;island
 
 0.85350
 
 0.75213
 
 Not queried
 
  0.8157232
 
       NA
 
       NA
  
 
  
 17
 
  16487631
 
  16487821
 
  ENSEMBL   UCSC 
 
  191
 
  4
 
 18023
 
 island
 
 0.01660
 
 0.02545
 
 Not queried
 
  0.8156996
 
       NA
 
       NA
  
 
  
 14
 
 100118672
 
 100119095
 
  ENSEMBL   UCSC 
 
  424
 
  5
 
 12177
 
 gene;island
 
 0.68449
 
 0.80242
 
 Not queried
 
  0.8154692
 
       NA
 
       NA
  
 
  
 10
 
  82116203
 
  82116596
 
  ENSEMBL   UCSC 
 
  394
 
 10
 
 4640
 
 gene;tss;island
 
 0.05604
 
 0.04201
 
 Not queried
 
  0.8152605
 
       NA
 
       NA
  
 
  
 2
 
 204400159
 
 204400358
 
  ENSEMBL   UCSC 
 
  200
 
  6
 
 23793
 
 tss
 
 0.01157
 
 0.02487
 
 Not queried
 
  0.8152463
 
       NA
 
       NA
  
 
  
 1
 
 118166013
 
 118166402
 
  ENSEMBL   UCSC 
 
  390
 
  4
 
 710
 
 gene
 
 0.88222
 
 0.84726
 
 Not queried
 
  0.8152318
 
       NA
 
       NA
  
 
  
 19
 
  51017855
 
  51018014
 
  ENSEMBL   UCSC 
 
  160
 
  4
 
 20944
 
 tss;island
 
 0.72509
 
 0.60703
 
 Not queried
 
  0.8152120
 
       NA
 
       NA
  
 
  
 6
 
 168106055
 
 168106326
 
  ENSEMBL   UCSC 
 
  272
 
  4
 
 36583
 
 island
 
 0.92770
 
 0.84849
 
 Not queried
 
  0.8149754
 
       NA
 
       NA
  
 
  
 13
 
 112547341
 
 112547653
 
  ENSEMBL   UCSC 
 
  313
 
  4
 
 11873
 
 island
 
 0.03690
 
 0.02436
 
 Not queried
 
  0.8146330
 
       NA
 
       NA
  
 
  
 4
 
   6114498
 
   6114699
 
  ENSEMBL   UCSC 
 
  202
 
  4
 
 29881
 
 gene;island
 
 0.78871
 
 0.87088
 
 Not queried
 
  0.8145951
 
       NA
 
       NA
  
 
  
 17
 
  30676722
 
  30677138
 
  ENSEMBL   UCSC 
 
  417
 
  9
 
 17222
 
 tss
 
 0.03043
 
 0.02830
 
 Not queried
 
  0.8145678
 
       NA
 
       NA
  
 
  
 3
 
 111392915
 
 111393453
 
  ENSEMBL   UCSC 
 
  539
 
  9
 
 28453
 
 tss
 
 0.03260
 
 0.02175
 
 Not queried
 
  0.8144022
 
       NA
 
       NA
  
 
  
 3
 
 114866835
 
 114867274
 
  ENSEMBL   UCSC 
 
  440
 
  5
 
 28471
 
 tss;island
 
 0.02849
 
 0.07370
 
 Not queried
 
  0.8143646
 
       NA
 
       NA
  
 
  
 7
 
  54610022
 
  54610320
 
  ENSEMBL   UCSC 
 
  299
 
  4
 
 36954
 
 gene
 
 0.02713
 
 0.03093
 
 Not queried
 
  0.8143622
 
       NA
 
       NA
  
 
  
 16
 
  56659336
 
  56659724
 
  ENSEMBL   UCSC 
 
  389
 
  7
 
 15916
 
 island
 
 0.03480
 
 0.02290
 
 Not queried
 
  0.8142191
 
       NA
 
       NA
  
 
  
 19
 
  57349679
 
  57350503
 
  ENSEMBL   UCSC 
 
  825
 
  8
 
 20203
 
 gene;island
 
 0.02739
 
 0.03889
 
 Not queried
 
  0.8141487
 
       NA
 
       NA
  
 
  
 5
 
    476497
 
    476842
 
  ENSEMBL   UCSC 
 
  346
 
  4
 
 31451
 
 gene;island
 
 0.83071
 
 0.77964
 
 Not queried
 
  0.8140559
 
       NA
 
       NA
  
 
  
 19
 
  55549722
 
  55549842
 
  ENSEMBL   UCSC 
 
  121
 
  6
 
 21032
 
 tss
 
 0.82382
 
 0.86775
 
 Not queried
 
  0.8140393
 
       NA
 
       NA
  
 
  
 16
 
  27280098
 
  27280434
 
  ENSEMBL   UCSC 
 
  337
 
  9
 
 15750
 
 island
 
 0.01670
 
 0.02141
 
 Not queried
 
  0.8139518
 
       NA
 
       NA
  
 
  
 17
 
  42219409
 
  42219681
 
  ENSEMBL   UCSC 
 
  273
 
  4
 
 16571
 
 gene
 
 0.00675
 
 0.01348
 
 Not queried
 
  0.8139476
 
       NA
 
       NA
  
 
  
 7
 
  16685473
 
  16685943
 
  ENSEMBL   UCSC 
 
  471
 
 10
 
 37389
 
 tss;island
 
 0.02446
 
 0.04373
 
 Not queried
 
  0.8138229
 
       NA
 
       NA
  
 
  
 10
 
  18940291
 
  18940404
 
  ENSEMBL   UCSC 
 
  114
 
  4
 
 4511
 
 gene;island
 
 0.00666
 
 0.01105
 
 Not queried
 
  0.8137052
 
       NA
 
       NA
  
 
  
 10
 
  35379341
 
  35379687
 
  ENSEMBL   UCSC 
 
  347
 
  7
 
 5008
 
 tss
 
 0.00868
 
 0.02594
 
 Not queried
 
  0.8136800
 
       NA
 
       NA
  
 
  
 2
 
  44314198
 
  44314662
 
  ENSEMBL   UCSC 
 
  465
 
  5
 
 24216
 
 island
 
 0.06870
 
 0.02606
 
 Not queried
 
  0.8136167
 
       NA
 
       NA
  
 
  
 20
 
  23346232
 
  23346733
 
  ENSEMBL   UCSC 
 
  502
 
  4
 
 25337
 
 gene;island
 
 0.65877
 
 0.84051
 
 Not queried
 
  0.8135363
 
       NA
 
       NA
  
 
  
 X
 
  53111116
 
  53111427
 
  ENSEMBL   UCSC 
 
  312
 
  6
 
 41327
 
 tss
 
 0.01625
 
 0.02979
 
 Not queried
 
  0.8134814
 
       NA
 
       NA
  
 
  
 8
 
  65492528
 
  65492936
 
  ENSEMBL   UCSC 
 
  409
 
 10
 
 40282
 
 island
 
 0.02571
 
 0.03449
 
 Not queried
 
  0.8133377
 
       NA
 
       NA
  
 
  
 12
 
  54446944
 
  54447596
 
  ENSEMBL   UCSC 
 
  653
 
 11
 
 9137
 
 gene;tss;island
 
 0.26647
 
 0.15937
 
 Not queried
 
  0.8133069
 
       NA
 
       NA
  
 
  
 13
 
  80915447
 
  80915525
 
  ENSEMBL   UCSC 
 
   79
 
  4
 
 11464
 
 tss;island
 
 0.02396
 
 0.03941
 
 Not queried
 
  0.8132522
 
       NA
 
       NA
  
 
  
 15
 
  29562049
 
  29562285
 
  ENSEMBL   UCSC 
 
  237
 
  7
 
 13242
 
 gene;tss;island
 
 0.03526
 
 0.02038
 
 Not queried
 
  0.8129542
 
       NA
 
       NA
  
 
  
 3
 
 197517855
 
 197518017
 
  ENSEMBL   UCSC 
 
  163
 
  8
 
 28783
 
 tss
 
 0.04025
 
 0.03694
 
 Not queried
 
  0.8129080
 
       NA
 
       NA
  
 
  
 20
 
   6103629
 
   6104274
 
  ENSEMBL   UCSC 
 
  646
 
  7
 
 25952
 
 island
 
 0.20751
 
 0.11276
 
 Not queried
 
  0.8128964
 
       NA
 
       NA
  
 
  
 16
 
   3550730
 
   3550932
 
  ENSEMBL   UCSC 
 
  203
 
  7
 
 15028
 
 tss
 
 0.01834
 
 0.03195
 
 Not queried
 
  0.8127517
 
       NA
 
       NA
  
 
  
 5
 
 134366831
 
 134367099
 
  ENSEMBL   UCSC 
 
  269
 
  4
 
 31700
 
 gene;island
 
 0.69495
 
 0.80829
 
 Not queried
 
  0.8126475
 
       NA
 
       NA
  
 
  
 4
 
 148401661
 
 148401958
 
  ENSEMBL   UCSC 
 
  298
 
  5
 
 30578
 
 tss
 
 0.79636
 
 0.72847
 
 Not queried
 
  0.8123918
 
       NA
 
       NA
  
 
  
 12
 
 113495386
 
 113495839
 
  ENSEMBL   UCSC 
 
  454
 
  5
 
 10796
 
 island
 
 0.57475
 
 0.50225
 
 Not queried
 
  0.8122406
 
       NA
 
       NA
  
 
  
 17
 
  40950545
 
  40950901
 
  ENSEMBL   UCSC 
 
  357
 
  7
 
 17342
 
 tss;island
 
 0.01742
 
 0.02900
 
 Not queried
 
  0.8121839
 
       NA
 
       NA
  
 
  
 17
 
   7307207
 
   7307572
 
  ENSEMBL   UCSC 
 
  366
 
  9
 
 17911
 
 island
 
 0.00896
 
 0.01387
 
 Not queried
 
  0.8121569
 
       NA
 
       NA
  
 
  
 1
 
  60539559
 
  60539675
 
  ENSEMBL   UCSC 
 
  117
 
  4
 
 1657
 
 tss
 
 0.02196
 
 0.03622
 
 Not queried
 
  0.8121189
 
       NA
 
       NA
  
 
  
 6
 
  31868724
 
  31869588
 
  ENSEMBL   UCSC 
 
  865
 
 26
 
 33930
 
 gene;island
 
 0.46044
 
 0.61953
 
 Not queried
 
  0.8120444
 
       NA
 
       NA
  
 
  
 1
 
 226411715
 
 226411954
 
  ENSEMBL   UCSC 
 
  240
 
  4
 
 1029
 
 gene;island
 
 0.02620
 
 0.04048
 
 Not queried
 
  0.8118929
 
       NA
 
       NA
  
 
  
 12
 
  15114703
 
  15114904
 
  ENSEMBL   UCSC 
 
  202
 
  4
 
 9564
 
 tss
 
 0.93590
 
 0.89141
 
 Not queried
 
  0.8117682
 
       NA
 
       NA
  
 
  
 6
 
  29705815
 
  29706048
 
  ENSEMBL   UCSC 
 
  234
 
  4
 
 33626
 
 gene
 
 0.10921
 
 0.04755
 
 Not queried
 
  0.8117148
 
       NA
 
       NA
  
 
  
 16
 
  56228114
 
  56228901
 
  ENSEMBL   UCSC 
 
  788
 
 12
 
 14726
 
 gene;island
 
 0.05205
 
 0.06297
 
 Not queried
 
  0.8115606
 
       NA
 
       NA
  
 
  
 3
 
  68981852
 
  68982098
 
  ENSEMBL   UCSC 
 
  247
 
  9
 
 28394
 
 tss;island
 
 0.01920
 
 0.02978
 
 Not queried
 
  0.8114336
 
       NA
 
       NA
  
 
  
 1
 
  46598275
 
  46598507
 
  ENSEMBL   UCSC 
 
  233
 
  8
 
 506
 
 gene;island
 
 0.01970
 
 0.03267
 
 Not queried
 
  0.8113569
 
       NA
 
       NA
  
 
  
 20
 
  61427684
 
  61427693
 
  ENSEMBL   UCSC 
 
   10
 
  4
 
 25841
 
 tss;island
 
 0.03814
 
 0.05733
 
 Not queried
 
  0.8112582
 
       NA
 
       NA
  
 
  
 14
 
 101350645
 
 101351026
 
  ENSEMBL   UCSC 
 
  382
 
  7
 
 12186
 
 gene
 
 0.93418
 
 0.92148
 
 Not queried
 
  0.8112009
 
       NA
 
       NA
  
 
  
 6
 
  30460244
 
  30460798
 
  ENSEMBL   UCSC 
 
  555
 
  7
 
 33708
 
 gene;island
 
 0.87205
 
 0.86686
 
 Not queried
 
  0.8111547
 
       NA
 
       NA
  
 
  
 4
 
 106395307
 
 106395643
 
  ENSEMBL   UCSC 
 
  337
 
  8
 
 30482
 
 tss
 
 0.02281
 
 0.03426
 
 Not queried
 
  0.8111370
 
       NA
 
       NA
  
 
  
 19
 
  39694477
 
  39694831
 
  ENSEMBL   UCSC 
 
  355
 
  4
 
 19991
 
 gene
 
 0.82466
 
 0.90603
 
 Not queried
 
  0.8110722
 
       NA
 
       NA
  
 
  
 14
 
 105190158
 
 105190590
 
  ENSEMBL   UCSC 
 
  433
 
  6
 
 13187
 
 island
 
 0.01896
 
 0.03941
 
 Not queried
 
  0.8109309
 
       NA
 
       NA
  
 
  
 1
 
   2436883
 
   2437200
 
  ENSEMBL   UCSC 
 
  318
 
  4
 
 2503
 
 island
 
 0.67042
 
 0.79193
 
 Not queried
 
  0.8108998
 
       NA
 
       NA
  
 
  
 1
 
 151693838
 
 151694320
 
  ENSEMBL   UCSC 
 
  483
 
  6
 
 3739
 
 island
 
 0.03806
 
 0.02533
 
 Not queried
 
  0.8108358
 
       NA
 
       NA
  
 
  
 1
 
  35222918
 
  35223247
 
  ENSEMBL   UCSC 
 
  330
 
  5
 
 410
 
 gene
 
 0.74745
 
 0.63935
 
 Not queried
 
  0.8108261
 
       NA
 
       NA
  
 
  
 X
 
  24331868
 
  24332162
 
  ENSEMBL   UCSC 
 
  295
 
  4
 
 41230
 
 tss;island
 
 0.86100
 
 0.89661
 
 Not queried
 
  0.8108255
 
       NA
 
       NA
  
 
  
 12
 
  69080752
 
  69081084
 
  ENSEMBL   UCSC 
 
  333
 
  4
 
 9184
 
 gene
 
 0.01052
 
 0.01424
 
 Not queried
 
  0.8107748
 
       NA
 
       NA
  
 
  
 11
 
  18656078
 
  18656167
 
  ENSEMBL   UCSC 
 
   90
 
  6
 
 7168
 
 tss
 
 0.00724
 
 0.01966
 
 Not queried
 
  0.8107305
 
       NA
 
       NA
  
 
  
 8
 
  80942046
 
  80942378
 
  ENSEMBL   UCSC 
 
  333
 
  4
 
 39285
 
 gene;island
 
 0.03943
 
 0.03457
 
 Not queried
 
  0.8101962
 
       NA
 
       NA
  
 
  
 17
 
  12692246
 
  12692692
 
  ENSEMBL   UCSC 
 
  447
 
  6
 
 17109
 
 tss;island
 
 0.04463
 
 0.07624
 
 Not queried
 
  0.8101853
 
       NA
 
       NA
  
 
  
 17
 
  43129629
 
  43129957
 
  ENSEMBL   UCSC 
 
  329
 
  4
 
 16590
 
 gene;tss;island
 
 0.22435
 
 0.32082
 
 Not queried
 
  0.8100330
 
       NA
 
       NA
  
 
  
 1
 
  54204112
 
  54204517
 
  ENSEMBL   UCSC 
 
  406
 
  5
 
 3280
 
 island
 
 0.04374
 
 0.04636
 
 Not queried
 
  0.8098333
 
       NA
 
       NA
  
 
  
 6
 
  26031939
 
  26032281
 
  ENSEMBL   UCSC 
 
  343
 
  5
 
 33533
 
 gene
 
 0.05762
 
 0.09680
 
 Not queried
 
  0.8096815
 
       NA
 
       NA
  
 
  
 2
 
  26987182
 
  26987250
 
  ENSEMBL   UCSC 
 
   69
 
  5
 
 22579
 
 gene
 
 0.01040
 
 0.01391
 
 Not queried
 
  0.8094709
 
       NA
 
       NA
  
 
  
 16
 
  51184786
 
  51185147
 
  ENSEMBL   UCSC 
 
  362
 
  9
 
 14719
 
 gene
 
 0.02438
 
 0.04557
 
 Not queried
 
  0.8091970
 
       NA
 
       NA
  
 
  
 1
 
  15541182
 
  15541446
 
  ENSEMBL   UCSC 
 
  265
 
  5
 
 244
 
 gene;island
 
 0.73993
 
 0.79502
 
 Not queried
 
  0.8091492
 
       NA
 
       NA
  
 
  
 8
 
   1955069
 
   1955323
 
  ENSEMBL   UCSC 
 
  255
 
  4
 
 39981
 
 island
 
 0.84044
 
 0.84496
 
 Not queried
 
  0.8090506
 
       NA
 
       NA
  
 
  
 X
 
  26210302
 
  26210529
 
  ENSEMBL   UCSC 
 
  228
 
  4
 
 41236
 
 tss
 
 0.82230
 
 0.78980
 
 Not queried
 
  0.8089715
 
       NA
 
       NA
  
 
  
 5
 
  93447407
 
  93447700
 
  ENSEMBL   UCSC 
 
  294
 
  7
 
 32120
 
 tss
 
 0.05679
 
 0.13904
 
 Not queried
 
  0.8089132
 
       NA
 
       NA
  
 
  
 3
 
  57199318
 
  57199533
 
  ENSEMBL   UCSC 
 
  216
 
  7
 
 29179
 
 island
 
 0.01194
 
 0.03576
 
 Not queried
 
  0.8088413
 
       NA
 
       NA
  
 
  
 1
 
  95538546
 
  95538837
 
  ENSEMBL   UCSC 
 
  292
 
  7
 
 1765
 
 tss
 
 0.01302
 
 0.01951
 
 Not queried
 
  0.8088324
 
       NA
 
       NA
  
 
  
 6
 
  70992907
 
  70993107
 
  ENSEMBL   UCSC 
 
  201
 
  4
 
 34318
 
 gene;island
 
 0.15035
 
 0.08599
 
 Not queried
 
  0.8087985
 
       NA
 
       NA
  
 
  
 19
 
   1140909
 
   1141150
 
  ENSEMBL   UCSC 
 
  242
 
  4
 
 19610
 
 gene;island
 
 0.95963
 
 0.91332
 
 Not queried
 
  0.8087632
 
       NA
 
       NA
  
 
  
 10
 
  77542302
 
  77542488
 
  ENSEMBL   UCSC 
 
  187
 
  7
 
 5160
 
 tss
 
 0.86107
 
 0.95362
 
 Not queried
 
  0.8086498
 
       NA
 
       NA
  
 
  
 15
 
  90727783
 
  90728157
 
  ENSEMBL   UCSC 
 
  375
 
  8
 
 14420
 
 island
 
 0.01919
 
 0.08233
 
 Not queried
 
  0.8086177
 
       NA
 
       NA
  
 
  
 15
 
  89346629
 
  89347176
 
  ENSEMBL   UCSC 
 
  548
 
  4
 
 14394
 
 island
 
 0.04538
 
 0.03007
 
 Not queried
 
  0.8085443
 
       NA
 
       NA
  
 
  
 15
 
  85197162
 
  85197409
 
  ENSEMBL   UCSC 
 
  248
 
  4
 
 13433
 
 gene;island
 
 0.01202
 
 0.02166
 
 Not queried
 
  0.8085063
 
       NA
 
       NA
  
 
  
 2
 
 210636350
 
 210636844
 
  ENSEMBL   UCSC 
 
  495
 
  8
 
 25014
 
 island
 
 0.02968
 
 0.05400
 
 Not queried
 
  0.8083329
 
       NA
 
       NA
  
 
  
 12
 
   6881590
 
   6881629
 
  ENSEMBL   UCSC 
 
   40
 
  5
 
 9502
 
 tss;island
 
 0.86519
 
 0.94542
 
 Not queried
 
  0.8082012
 
       NA
 
       NA
  
 
  
 1
 
  17445660
 
  17446162
 
  ENSEMBL   UCSC 
 
  503
 
  8
 
 2752
 
 island
 
 0.02380
 
 0.04071
 
 Not queried
 
  0.8081890
 
       NA
 
       NA
  
 
  
 4
 
 186125334
 
 186125504
 
  ENSEMBL   UCSC 
 
  171
 
  6
 
 30652
 
 tss;island
 
 0.01571
 
 0.03454
 
 Not queried
 
  0.8080511
 
       NA
 
       NA
  
 
  
 16
 
   1110844
 
   1111220
 
  ENSEMBL   UCSC 
 
  377
 
  4
 
 15481
 
 island
 
 0.76982
 
 0.83658
 
 Not queried
 
  0.8079936
 
       NA
 
       NA
  
 
  
 9
 
  36572645
 
  36572901
 
  ENSEMBL   UCSC 
 
  257
 
  4
 
 40748
 
 tss
 
 0.01853
 
 0.02720
 
 Not queried
 
  0.8078959
 
       NA
 
       NA
  
 
  
 6
 
  16128926
 
  16129570
 
  ENSEMBL   UCSC 
 
  645
 
 12
 
 35405
 
 island
 
 0.01683
 
 0.03951
 
 Not queried
 
  0.8078517
 
       NA
 
       NA
  
 
  
 13
 
  99852409
 
  99852629
 
  ENSEMBL   UCSC 
 
  221
 
  9
 
 11484
 
 tss;island
 
 0.01700
 
 0.02479
 
 Not queried
 
  0.8077613
 
       NA
 
       NA
  
 
  
 1
 
 231176985
 
 231177201
 
  ENSEMBL   UCSC 
 
  217
 
  4
 
 2316
 
 tss;island
 
 0.30787
 
 0.45875
 
 Not queried
 
  0.8077285
 
       NA
 
       NA
  
 
  
 15
 
  41061984
 
  41062273
 
  ENSEMBL   UCSC 
 
  290
 
  6
 
 13267
 
 gene
 
 0.80708
 
 0.89844
 
 Not queried
 
  0.8076910
 
       NA
 
       NA
  
 
  
 22
 
  45704902
 
  45705042
 
  ENSEMBL   UCSC 
 
  141
 
  6
 
 27179
 
 tss;island
 
 0.45964
 
 0.38317
 
 Not queried
 
  0.8076141
 
       NA
 
       NA
  
 
  
 14
 
 102028994
 
 102029375
 
  ENSEMBL   UCSC 
 
  382
 
  4
 
 12187
 
 gene;island
 
 0.38748
 
 0.55550
 
 Not queried
 
  0.8075498
 
       NA
 
       NA
  
 
  
 4
 
 155471567
 
 155471827
 
  ENSEMBL   UCSC 
 
  261
 
  8
 
 30597
 
 tss
 
 0.01371
 
 0.02204
 
 Not queried
 
  0.8075261
 
       NA
 
       NA
  
 
  
 10
 
  25305002
 
  25305418
 
  ENSEMBL   UCSC 
 
  417
 
 11
 
 4973
 
 tss;island
 
 0.00968
 
 0.02148
 
 Not queried
 
  0.8075131
 
       NA
 
       NA
  
 
  
 11
 
  49914985
 
  49915371
 
  ENSEMBL   UCSC 
 
  387
 
  4
 
 8255
 
 island
 
 0.50975
 
 0.69962
 
 Not queried
 
  0.8074253
 
       NA
 
       NA
  
 
  
 11
 
  58874608
 
  58874645
 
  ENSEMBL   UCSC 
 
   38
 
  5
 
 7292
 
 tss
 
 0.05585
 
 0.06271
 
 Not queried
 
  0.8074144
 
       NA
 
       NA
  
 
  
 3
 
 181437145
 
 181437712
 
  ENSEMBL   UCSC 
 
  568
 
  5
 
 28000
 
 gene;island
 
 0.73397
 
 0.63413
 
 Not queried
 
  0.8073687
 
       NA
 
       NA
  
 
  
 13
 
  25874859
 
  25875890
 
  ENSEMBL   UCSC 
 
 1032
 
 13
 
 11583
 
 island
 
 0.03501
 
 0.06164
 
 Not queried
 
  0.8071412
 
       NA
 
       NA
  
 
  
 11
 
  86013123
 
  86013510
 
  ENSEMBL   UCSC 
 
  388
 
  8
 
 7587
 
 tss
 
 0.01850
 
 0.00907
 
 Not queried
 
  0.8071315
 
       NA
 
       NA
  
 
  
 6
 
  29600462
 
  29600642
 
  ENSEMBL   UCSC 
 
  181
 
  4
 
 33620
 
 gene;island
 
 0.10362
 
 0.14326
 
 Not queried
 
  0.8070558
 
       NA
 
       NA
  
 
  
 11
 
  64879061
 
  64879336
 
  ENSEMBL   UCSC 
 
  276
 
  8
 
 7393
 
 tss
 
 0.04743
 
 0.07626
 
 Not queried
 
  0.8069932
 
       NA
 
       NA
  
 
  
 13
 
  37574527
 
  37574825
 
  ENSEMBL   UCSC 
 
  299
 
  8
 
 11371
 
 tss
 
 0.00842
 
 0.01129
 
 Not queried
 
  0.8069840
 
       NA
 
       NA
  
 
  
 20
 
  62680970
 
  62681428
 
  ENSEMBL   UCSC 
 
  459
 
 11
 
 26339
 
 island
 
 0.04219
 
 0.09610
 
 Not queried
 
  0.8069180
 
       NA
 
       NA
  
 
  
 11
 
   1862020
 
   1862477
 
  ENSEMBL   UCSC 
 
  458
 
  4
 
 6416
 
 gene
 
 0.76341
 
 0.88427
 
 Not queried
 
  0.8067920
 
       NA
 
       NA
  
 
  
 3
 
  12856650
 
  12856862
 
  ENSEMBL   UCSC 
 
  213
 
  5
 
 27633
 
 gene;island
 
 0.87624
 
 0.89952
 
 Not queried
 
  0.8067592
 
       NA
 
       NA
  
 
  
 6
 
  83072747
 
  83072781
 
  ENSEMBL   UCSC 
 
   35
 
  4
 
 35024
 
 tss;island
 
 0.09699
 
 0.14727
 
 Not queried
 
  0.8067038
 
       NA
 
       NA
  
 
  
 17
 
  76850052
 
  76850277
 
  ENSEMBL   UCSC 
 
  226
 
  5
 
 16793
 
 gene;island
 
 0.92243
 
 0.93834
 
 Not queried
 
  0.8066956
 
       NA
 
       NA
  
 
  
 6
 
 167263025
 
 167263454
 
  ENSEMBL   UCSC 
 
  430
 
  6
 
 34490
 
 gene
 
 0.91540
 
 0.84224
 
 Not queried
 
  0.8065561
 
       NA
 
       NA
  
 
  
 13
 
  25745768
 
  25746644
 
  ENSEMBL   UCSC 
 
  877
 
 13
 
 11581
 
 island
 
 0.13489
 
 0.17160
 
 Not queried
 
  0.8065130
 
       NA
 
       NA
  
 
  
 17
 
   7905636
 
   7905928
 
  ENSEMBL   UCSC 
 
  293
 
  4
 
 17072
 
 tss
 
 0.68610
 
 0.44530
 
 Not queried
 
  0.8062173
 
       NA
 
       NA
  
 
  
 1
 
  36859272
 
  36859592
 
  ENSEMBL   UCSC 
 
  321
 
  4
 
 428
 
 gene;island
 
 0.93282
 
 0.88849
 
 Not queried
 
  0.8061363
 
       NA
 
       NA
  
 
  
 21
 
  38071673
 
  38071911
 
  ENSEMBL   UCSC 
 
  239
 
  4
 
 26498
 
 tss
 
 0.03119
 
 0.06017
 
 Not queried
 
  0.8060302
 
       NA
 
       NA
  
 
  
 19
 
  39897131
 
  39897474
 
  ENSEMBL   UCSC 
 
  344
 
  7
 
 20734
 
 tss
 
 0.03094
 
 0.05072
 
 Not queried
 
  0.8056694
 
       NA
 
       NA
  
 
  
 2
 
  46925970
 
  46926433
 
  ENSEMBL   UCSC 
 
  464
 
  6
 
 24239
 
 island
 
 0.01856
 
 0.03161
 
 Not queried
 
  0.8056362
 
       NA
 
       NA
  
 
  
 16
 
  67472363
 
  67472507
 
  ENSEMBL   UCSC 
 
  145
 
  4
 
 14769
 
 gene
 
 0.83465
 
 0.96874
 
 Not queried
 
  0.8052946
 
       NA
 
       NA
  
 
  
 2
 
 216301341
 
 216301766
 
  ENSEMBL   UCSC 
 
  426
 
  5
 
 23825
 
 tss;island
 
 0.05507
 
 0.03228
 
 Not queried
 
  0.8050024
 
       NA
 
       NA
  
 
  
 5
 
  44809616
 
  44809978
 
  ENSEMBL   UCSC 
 
  363
 
  4
 
 31550
 
 gene;island
 
 0.01917
 
 0.01152
 
 Not queried
 
  0.8049575
 
       NA
 
       NA
  
 
  
 17
 
  19280802
 
  19281001
 
  ENSEMBL   UCSC 
 
  200
 
  4
 
 17155
 
 tss;island
 
 0.07982
 
 0.05733
 
 Not queried
 
  0.8048277
 
       NA
 
       NA
  
 
  
 17
 
  46184579
 
  46184917
 
  ENSEMBL   UCSC 
 
  339
 
  9
 
 17421
 
 tss
 
 0.01305
 
 0.02808
 
 Not queried
 
  0.8048085
 
       NA
 
       NA
  
 
  
 11
 
  65246411
 
  65246590
 
  ENSEMBL   UCSC 
 
  180
 
  4
 
 8436
 
 island
 
 0.89264
 
 0.86138
 
 Not queried
 
  0.8047665
 
       NA
 
       NA
  
 
  
 16
 
  11761803
 
  11762212
 
  ENSEMBL   UCSC 
 
  410
 
  7
 
 15063
 
 tss;island
 
 0.03605
 
 0.06277
 
 Not queried
 
  0.8047410
 
       NA
 
       NA
  
 
  
 1
 
 115632236
 
 115632727
 
  ENSEMBL   UCSC 
 
  492
 
  8
 
 1853
 
 tss
 
 0.10793
 
 0.09794
 
 Not queried
 
  0.8047047
 
       NA
 
       NA
  
 
  
 16
 
   4674642
 
   4674718
 
  ENSEMBL   UCSC 
 
   77
 
  5
 
 15042
 
 tss;island
 
 0.01225
 
 0.02120
 
 Not queried
 
  0.8044467
 
       NA
 
       NA
  
 
  
 16
 
  89225495
 
  89225922
 
  ENSEMBL   UCSC 
 
  428
 
  6
 
 16208
 
 island
 
 0.74933
 
 0.77557
 
 Not queried
 
  0.8044311
 
       NA
 
       NA
  
 
  
 15
 
  65133392
 
  65133765
 
  ENSEMBL   UCSC 
 
  374
 
  5
 
 13706
 
 tss;island
 
 0.02057
 
 0.02844
 
 Not queried
 
  0.8042315
 
       NA
 
       NA
  
 
  
 5
 
 180018465
 
 180018735
 
  ENSEMBL   UCSC 
 
  271
 
  7
 
 33440
 
 island
 
 0.79540
 
 0.84194
 
 Not queried
 
  0.8042282
 
       NA
 
       NA
  
 
  
 X
 
  27764690
 
  27764899
 
  ENSEMBL   UCSC 
 
  210
 
  4
 
 41239
 
 tss
 
 0.94134
 
 0.92471
 
 Not queried
 
  0.8040929
 
       NA
 
       NA
  
 
  
 11
 
  62783361
 
  62783573
 
  ENSEMBL   UCSC 
 
  213
 
  7
 
 7353
 
 tss
 
 0.67267
 
 0.57734
 
 Not queried
 
  0.8040928
 
       NA
 
       NA
  
 
  
 1
 
  41978691
 
  41979124
 
  ENSEMBL   UCSC 
 
  434
 
  5
 
 460
 
 gene;island
 
 0.59589
 
 0.70979
 
 Not queried
 
  0.8040483
 
       NA
 
       NA
  
 
  
 12
 
 131568441
 
 131568764
 
  ENSEMBL   UCSC 
 
  324
 
  5
 
 9398
 
 gene
 
 0.96479
 
 0.91094
 
 Not queried
 
  0.8040450
 
       NA
 
       NA
  
 
  
 11
 
  43602845
 
  43602965
 
  ENSEMBL   UCSC 
 
  121
 
  7
 
 8195
 
 island
 
 0.01241
 
 0.02147
 
 Not queried
 
  0.8039999
 
       NA
 
       NA
  
 
  
 2
 
  74681787
 
  74682200
 
  ENSEMBL   UCSC 
 
  414
 
 11
 
 24395
 
 island
 
 0.01315
 
 0.01299
 
 Not queried
 
  0.8039112
 
       NA
 
       NA
  
 
  
 12
 
   4382878
 
   4383281
 
  ENSEMBL   UCSC 
 
  404
 
  6
 
 10145
 
 island
 
 0.01761
 
 0.02393
 
 Not queried
 
  0.8037652
 
       NA
 
       NA
  
 
  
 5
 
  65222276
 
  65222365
 
  ENSEMBL   UCSC 
 
   90
 
  4
 
 32039
 
 tss
 
 0.01882
 
 0.03392
 
 Not queried
 
  0.8037567
 
       NA
 
       NA
  
 
  
 19
 
   1580400
 
   1580629
 
  ENSEMBL   UCSC 
 
  230
 
  4
 
 19623
 
 gene;island
 
 0.81430
 
 0.85293
 
 Not queried
 
  0.8037509
 
       NA
 
       NA
  
 
  
 16
 
   6068831
 
   6069047
 
  ENSEMBL   UCSC 
 
  217
 
  4
 
 15049
 
 tss
 
 0.17328
 
 0.27823
 
 Not queried
 
  0.8035915
 
       NA
 
       NA
  
 
  
 3
 
 178984841
 
 178984973
 
  ENSEMBL   UCSC 
 
  133
 
  4
 
 28687
 
 tss
 
 0.57194
 
 0.69374
 
 Not queried
 
  0.8034882
 
       NA
 
       NA
  
 
  
 19
 
  14543874
 
  14544044
 
  ENSEMBL   UCSC 
 
  171
 
  6
 
 20508
 
 tss;island
 
 0.06814
 
 0.09897
 
 Not queried
 
  0.8034658
 
       NA
 
       NA
  
 
  
 16
 
    960911
 
    961353
 
  ENSEMBL   UCSC 
 
  443
 
  4
 
 14522
 
 gene;island
 
 0.92516
 
 0.96427
 
 Not queried
 
  0.8033036
 
       NA
 
       NA
  
 
  
 4
 
  83483204
 
  83483487
 
  ENSEMBL   UCSC 
 
  284
 
  6
 
 30420
 
 tss;island
 
 0.03699
 
 0.02579
 
 Not queried
 
  0.8032846
 
       NA
 
       NA
  
 
  
 1
 
 204182957
 
 204183116
 
  ENSEMBL   UCSC 
 
  160
 
  4
 
 955
 
 gene
 
 0.18462
 
 0.32153
 
 Not queried
 
  0.8032829
 
       NA
 
       NA
  
 
  
 2
 
 238969253
 
 238969609
 
  ENSEMBL   UCSC 
 
  357
 
  8
 
 23931
 
 tss;island
 
 0.03262
 
 0.05537
 
 Not queried
 
  0.8032228
 
       NA
 
       NA
  
 
  
 7
 
  75958866
 
  75959203
 
  ENSEMBL   UCSC 
 
  338
 
  6
 
 36992
 
 gene;island
 
 0.86455
 
 0.90223
 
 Not queried
 
  0.8030062
 
       NA
 
       NA
  
 
  
 1
 
  47133596
 
  47133953
 
  ENSEMBL   UCSC 
 
  358
 
  4
 
 512
 
 gene;island
 
 0.01958
 
 0.03342
 
 Not queried
 
  0.8029612
 
       NA
 
       NA
  
 
  
 6
 
  30707966
 
  30708009
 
  ENSEMBL   UCSC 
 
   44
 
  4
 
 33757
 
 gene;island
 
 0.95488
 
 0.97434
 
 Not queried
 
  0.8029075
 
       NA
 
       NA
  
 
  
 20
 
  33577997
 
  33578094
 
  ENSEMBL   UCSC 
 
   98
 
  4
 
 25660
 
 tss
 
 0.77792
 
 0.87605
 
 Not queried
 
  0.8028910
 
       NA
 
       NA
  
 
  
 11
 
 115374969
 
 115375226
 
  ENSEMBL   UCSC 
 
  258
 
  4
 
 6893
 
 gene
 
 0.02015
 
 0.04661
 
 Not queried
 
  0.8027000
 
       NA
 
       NA
  
 
  
 2
 
 160568689
 
 160568836
 
  ENSEMBL   UCSC 
 
  148
 
  5
 
 23643
 
 tss;island
 
 0.01680
 
 0.02990
 
 Not queried
 
  0.8025760
 
       NA
 
       NA
  
 
  
 21
 
  36261029
 
  36261071
 
  ENSEMBL   UCSC 
 
   43
 
  4
 
 26372
 
 gene;tss;island
 
 0.01578
 
 0.03471
 
 Not queried
 
  0.8024663
 
       NA
 
       NA
  
 
  
 17
 
    928723
 
    929075
 
  ENSEMBL   UCSC 
 
  353
 
  5
 
 16269
 
 gene;island
 
 0.79189
 
 0.91407
 
 Not queried
 
  0.8022598
 
       NA
 
       NA
  
 
  
 5
 
 139283051
 
 139283351
 
  ENSEMBL   UCSC 
 
  301
 
  5
 
 31725
 
 gene;island
 
 0.22088
 
 0.17671
 
 Not queried
 
  0.8021766
 
       NA
 
       NA
  
 
  
 X
 
  38186830
 
  38187196
 
  ENSEMBL   UCSC 
 
  367
 
  8
 
 41257
 
 tss
 
 0.04806
 
 0.06478
 
 Not queried
 
  0.8020818
 
       NA
 
       NA
  
 
  
 14
 
 104314434
 
 104314734
 
  ENSEMBL   UCSC 
 
  301
 
  6
 
 12626
 
 tss;island
 
 0.01623
 
 0.02134
 
 Not queried
 
  0.8020611
 
       NA
 
       NA
  
 
  
 13
 
 114926584
 
 114927035
 
  ENSEMBL   UCSC 
 
  452
 
  4
 
 11969
 
 island
 
 0.82606
 
 0.93546
 
 Not queried
 
  0.8019544
 
       NA
 
       NA
  
 
  
 16
 
   1598866
 
   1599150
 
  ENSEMBL   UCSC 
 
  285
 
  4
 
 14547
 
 gene;island
 
 0.76370
 
 0.83508
 
 Not queried
 
  0.8019397
 
       NA
 
       NA
  
 
  
 7
 
 129425330
 
 129425932
 
  ENSEMBL   UCSC 
 
  603
 
  6
 
 38850
 
 island
 
 0.10872
 
 0.06213
 
 Not queried
 
  0.8019144
 
       NA
 
       NA
  
 
  
 17
 
  48546258
 
  48546620
 
  ENSEMBL   UCSC 
 
  363
 
  8
 
 17467
 
 tss
 
 0.85464
 
 0.79192
 
 Not queried
 
  0.8018665
 
       NA
 
       NA
  
 
  
 17
 
  27230118
 
  27230533
 
  ENSEMBL   UCSC 
 
  416
 
  9
 
 17195
 
 tss;island
 
 0.03739
 
 0.08611
 
 Not queried
 
  0.8018325
 
       NA
 
       NA
  
 
  
 19
 
  29455596
 
  29455913
 
  ENSEMBL   UCSC 
 
  318
 
  4
 
 20632
 
 tss
 
 0.72025
 
 0.86548
 
 Not queried
 
  0.8018112
 
       NA
 
       NA
  
 
  
 4
 
 157996959
 
 157997526
 
  ENSEMBL   UCSC 
 
  568
 
 12
 
 30605
 
 tss
 
 0.03248
 
 0.04065
 
 Not queried
 
  0.8017675
 
       NA
 
       NA
  
 
  
 8
 
  28573503
 
  28573832
 
  ENSEMBL   UCSC 
 
  330
 
  4
 
 39200
 
 gene;island
 
 0.94421
 
 0.95626
 
 Not queried
 
  0.8017363
 
       NA
 
       NA
  
 
  
 17
 
  42462958
 
  42463277
 
  ENSEMBL   UCSC 
 
  320
 
  4
 
 16583
 
 gene;island
 
 0.02089
 
 0.03106
 
 Not queried
 
  0.8016505
 
       NA
 
       NA
  
 
  
 15
 
  49103180
 
  49103565
 
  ENSEMBL   UCSC 
 
  386
 
  7
 
 14095
 
 island
 
 0.00996
 
 0.01718
 
 Not queried
 
  0.8016067
 
       NA
 
       NA
  
 
  
 7
 
  62514477
 
  62514926
 
  ENSEMBL   UCSC 
 
  450
 
  5
 
 38456
 
 island
 
 0.33401
 
 0.43143
 
 Not queried
 
  0.8015698
 
       NA
 
       NA
  
 
  
 7
 
 100318080
 
 100318321
 
  ENSEMBL   UCSC 
 
  242
 
  8
 
 37726
 
 tss;island
 
 0.02472
 
 0.04411
 
 Not queried
 
  0.8015520
 
       NA
 
       NA
  
 
  
 13
 
  47472050
 
  47472429
 
  ENSEMBL   UCSC 
 
  380
 
 12
 
 11401
 
 tss
 
 0.91405
 
 0.83199
 
 Not queried
 
  0.8014871
 
       NA
 
       NA
  
 
  
 10
 
 135113191
 
 135113459
 
  ENSEMBL   UCSC 
 
  269
 
  5
 
 4881
 
 gene
 
 0.89627
 
 0.95553
 
 Not queried
 
  0.8014581
 
       NA
 
       NA
  
 
  
 8
 
   9762716
 
   9763263
 
  ENSEMBL   UCSC 
 
  548
 
  6
 
 40001
 
 island
 
 0.02565
 
 0.04388
 
 Not queried
 
  0.8014519
 
       NA
 
       NA
  
 
  
 7
 
   2884021
 
   2884448
 
  ENSEMBL   UCSC 
 
  428
 
 11
 
 37350
 
 tss;island
 
 0.01856
 
 0.02796
 
 Not queried
 
  0.8013103
 
       NA
 
       NA
  
 
  
 13
 
  95248547
 
  95248888
 
  ENSEMBL   UCSC 
 
  342
 
  6
 
 11470
 
 tss
 
 0.09136
 
 0.16475
 
 Not queried
 
  0.8012311
 
       NA
 
       NA
  
 
  
 11
 
   1274018
 
   1274269
 
  ENSEMBL   UCSC 
 
  252
 
  4
 
 6388
 
 gene;island
 
 0.65836
 
 0.75186
 
 Not queried
 
  0.8011440
 
       NA
 
       NA
  
 
  
 12
 
 113415883
 
 113416268
 
  ENSEMBL   UCSC 
 
  386
 
  5
 
 9999
 
 tss
 
 0.70567
 
 0.80685
 
 Not queried
 
  0.8009978
 
       NA
 
       NA
  
 
  
 19
 
   8643540
 
   8643798
 
  ENSEMBL   UCSC 
 
  259
 
  4
 
 20385
 
 tss
 
 0.13804
 
 0.05484
 
 Not queried
 
  0.8009060
 
       NA
 
       NA
  
 
  
 6
 
 167040675
 
 167040954
 
  ENSEMBL   UCSC 
 
  280
 
  4
 
 34486
 
 gene;island
 
 0.15444
 
 0.08180
 
 Not queried
 
  0.8009011
 
       NA
 
       NA
  
 
  
 1
 
  38260988
 
  38261192
 
  ENSEMBL   UCSC 
 
  205
 
  4
 
 435
 
 gene;island
 
 0.15320
 
 0.06047
 
 Not queried
 
  0.8007365
 
       NA
 
       NA
  
 
  
 4
 
   1807118
 
   1808029
 
  ENSEMBL   UCSC 
 
  912
 
  8
 
 29832
 
 gene;island
 
 0.85796
 
 0.89539
 
 Not queried
 
  0.8005519
 
       NA
 
       NA
  
 
  
 16
 
  31214126
 
  31214607
 
  ENSEMBL   UCSC 
 
  482
 
 10
 
 15841
 
 island
 
 0.40369
 
 0.27886
 
 Not queried
 
  0.8004142
 
       NA
 
       NA
  
 
  
 19
 
  50666238
 
  50666484
 
  ENSEMBL   UCSC 
 
  247
 
  4
 
 20134
 
 gene
 
 0.63278
 
 0.71962
 
 Not queried
 
  0.8004078
 
       NA
 
       NA
  
 
  
 21
 
  37432493
 
  37432782
 
  ENSEMBL   UCSC 
 
  290
 
  6
 
 26373
 
 gene;island
 
 0.02147
 
 0.02891
 
 Not queried
 
  0.8003791
 
       NA
 
       NA
  
 
  
 17
 
  46894191
 
  46894465
 
  ENSEMBL   UCSC 
 
  275
 
  4
 
 16633
 
 gene
 
 0.03117
 
 0.04914
 
 Not queried
 
  0.8003621
 
       NA
 
       NA
  
 
  
 2
 
 240230452
 
 240230892
 
  ENSEMBL   UCSC 
 
  441
 
  5
 
 23077
 
 gene
 
 0.90090
 
 0.91561
 
 Not queried
 
  0.8003083
 
       NA
 
       NA
  
 
  
 4
 
 150999899
 
 151000014
 
  ENSEMBL   UCSC 
 
  116
 
  6
 
 30583
 
 tss
 
 0.01262
 
 0.01847
 
 Not queried
 
  0.8002794
 
       NA
 
       NA
  
 
  
 3
 
  50382952
 
  50383485
 
  ENSEMBL   UCSC 
 
  534
 
 11
 
 29111
 
 island
 
 0.69325
 
 0.55309
 
 Not queried
 
  0.8001137
 
       NA
 
       NA
  
 
  
 7
 
   4030274
 
   4030736
 
  ENSEMBL   UCSC 
 
  463
 
  5
 
 36762
 
 gene
 
 0.82767
 
 0.86566
 
 Not queried
 
  0.7998723
 
       NA
 
       NA
  
 
  
 3
 
  32147645
 
  32148175
 
  ENSEMBL   UCSC 
 
  531
 
  8
 
 28918
 
 island
 
 0.02815
 
 0.04733
 
 Not queried
 
  0.7997386
 
       NA
 
       NA
  
 
  
 19
 
  17420039
 
  17420434
 
  ENSEMBL   UCSC 
 
  396
 
  9
 
 21700
 
 island
 
 0.02013
 
 0.03514
 
 Not queried
 
  0.7997347
 
       NA
 
       NA
  
 
  
 1
 
 108508067
 
 108508207
 
  ENSEMBL   UCSC 
 
  141
 
  4
 
 1785
 
 tss;island
 
 0.02876
 
 0.01542
 
 Not queried
 
  0.7996822
 
       NA
 
       NA
  
 
  
 8
 
 143509263
 
 143509556
 
  ENSEMBL   UCSC 
 
  294
 
  4
 
 40567
 
 island
 
 0.67467
 
 0.79741
 
 Not queried
 
  0.7996466
 
       NA
 
       NA
  
 
  
 6
 
  31645405
 
  31645677
 
  ENSEMBL   UCSC 
 
  273
 
  4
 
 33873
 
 gene;island
 
 0.53554
 
 0.74962
 
 Not queried
 
  0.7996433
 
       NA
 
       NA
  
 
  
 2
 
 109402912
 
 109403139
 
  ENSEMBL   UCSC 
 
  228
 
  8
 
 23528
 
 tss
 
 0.01546
 
 0.02210
 
 Not queried
 
  0.7996299
 
       NA
 
       NA
  
 
  
 1
 
 159869927
 
 159870326
 
  ENSEMBL   UCSC 
 
  400
 
  7
 
 2028
 
 tss;island
 
 0.04266
 
 0.06416
 
 Not queried
 
  0.7996254
 
       NA
 
       NA
  
 
  
 6
 
  31474043
 
  31474506
 
  ENSEMBL   UCSC 
 
  464
 
 11
 
 33820
 
 gene
 
 0.84689
 
 0.77397
 
 Not queried
 
  0.7994686
 
       NA
 
       NA
  
 
  
 6
 
 139094390
 
 139095140
 
  ENSEMBL   UCSC 
 
  751
 
 13
 
 36454
 
 island
 
 0.01370
 
 0.02231
 
 Not queried
 
  0.7994443
 
       NA
 
       NA
  
 
  
 11
 
  74660429
 
  74660536
 
  ENSEMBL   UCSC 
 
  108
 
  4
 
 6812
 
 gene
 
 0.03443
 
 0.04619
 
 Not queried
 
  0.7993579
 
       NA
 
       NA
  
 
  
 8
 
  23261731
 
  23262159
 
  ENSEMBL   UCSC 
 
  429
 
  6
 
 39536
 
 tss
 
 0.11111
 
 0.05447
 
 Not queried
 
  0.7992583
 
       NA
 
       NA
  
 
  
 3
 
  52488037
 
  52488229
 
  ENSEMBL   UCSC 
 
  193
 
  4
 
 28339
 
 tss;island
 
 0.59524
 
 0.67445
 
 Not queried
 
  0.7992579
 
       NA
 
       NA
  
 
  
 19
 
  45926999
 
  45927335
 
  ENSEMBL   UCSC 
 
  337
 
 10
 
 20834
 
 tss;island
 
 0.01900
 
 0.02634
 
 Not queried
 
  0.7992260
 
       NA
 
       NA
  
 
  
 3
 
  50383167
 
  50383485
 
  ENSEMBL   UCSC 
 
  319
 
  7
 
 28312
 
 tss
 
 0.65325
 
 0.54122
 
 Not queried
 
  0.7992212
 
       NA
 
       NA
  
 
  
 10
 
 131356942
 
 131357256
 
  ENSEMBL   UCSC 
 
  315
 
  6
 
 4812
 
 gene
 
 0.81507
 
 0.89424
 
 Not queried
 
  0.7991952
 
       NA
 
       NA
  
 
  
 14
 
  57263993
 
  57264287
 
  ENSEMBL   UCSC 
 
  295
 
  4
 
 12847
 
 island
 
 0.93893
 
 0.91479
 
 Not queried
 
  0.7990686
 
       NA
 
       NA
  
 
  
 12
 
 133312061
 
 133312361
 
  ENSEMBL   UCSC 
 
  301
 
  5
 
 9432
 
 gene;island
 
 0.85132
 
 0.88200
 
 Not queried
 
  0.7990066
 
       NA
 
       NA
  
 
  
 8
 
 104383715
 
 104383730
 
  ENSEMBL   UCSC 
 
   16
 
  4
 
 39794
 
 tss
 
 0.00646
 
 0.00490
 
 Not queried
 
  0.7989278
 
       NA
 
       NA
  
 
  
 18
 
   9136381
 
   9136711
 
  ENSEMBL   UCSC 
 
  331
 
  6
 
 19142
 
 tss
 
 0.00985
 
 0.02097
 
 Not queried
 
  0.7988674
 
       NA
 
       NA
  
 
  
 17
 
  21415244
 
  21415527
 
  ENSEMBL   UCSC 
 
  284
 
  4
 
 18090
 
 island
 
 0.02399
 
 0.29925
 
 Not queried
 
  0.7988419
 
       NA
 
       NA
  
 
  
 8
 
  60032607
 
  60032983
 
  ENSEMBL   UCSC 
 
  377
 
  5
 
 39659
 
 tss;island
 
 0.07134
 
 0.03092
 
 Not queried
 
  0.7984366
 
       NA
 
       NA
  
 
  
 12
 
    298151
 
    298484
 
  ENSEMBL   UCSC 
 
  334
 
  4
 
 10115
 
 island
 
 0.11478
 
 0.22412
 
 Not queried
 
  0.7983409
 
       NA
 
       NA
  
 
  
 14
 
  39901757
 
  39902004
 
  ENSEMBL   UCSC 
 
  248
 
  6
 
 12340
 
 tss;island
 
 0.00797
 
 0.01559
 
 Not queried
 
  0.7983257
 
       NA
 
       NA
  
 
  
 6
 
  52535550
 
  52535846
 
  ENSEMBL   UCSC 
 
  297
 
  8
 
 34971
 
 tss
 
 0.06094
 
 0.09140
 
 Not queried
 
  0.7983050
 
       NA
 
       NA
  
 
  
 7
 
  79083408
 
  79084196
 
  ENSEMBL   UCSC 
 
  789
 
 16
 
 37609
 
 tss;island
 
 0.08540
 
 0.06151
 
 Not queried
 
  0.7982419
 
       NA
 
       NA
  
 
  
 8
 
  88886051
 
  88886432
 
  ENSEMBL   UCSC 
 
  382
 
  9
 
 40375
 
 island
 
 0.92129
 
 0.95542
 
 Not queried
 
  0.7982144
 
       NA
 
       NA
  
 
  
 11
 
 123524633
 
 123525237
 
  ENSEMBL   UCSC 
 
  605
 
  5
 
 6931
 
 gene;island
 
 0.03249
 
 0.08703
 
 Not queried
 
  0.7979493
 
       NA
 
       NA
  
 
  
 5
 
 122424399
 
 122424706
 
  ENSEMBL   UCSC 
 
  308
 
  4
 
 32189
 
 tss;island
 
 0.01471
 
 0.02097
 
 Not queried
 
  0.7978128
 
       NA
 
       NA
  
 
  
 13
 
 113410887
 
 113411200
 
  ENSEMBL   UCSC 
 
  314
 
  6
 
 11221
 
 gene;island
 
 0.92492
 
 0.89500
 
 Not queried
 
  0.7977376
 
       NA
 
       NA
  
 
  
 7
 
  45144602
 
  45144871
 
  ENSEMBL   UCSC 
 
  270
 
  4
 
 36937
 
 gene;tss
 
 0.72618
 
 0.81405
 
 Not queried
 
  0.7976758
 
       NA
 
       NA
  
 
  
 16
 
  53468112
 
  53468350
 
  ENSEMBL   UCSC 
 
  239
 
  5
 
 15893
 
 island
 
 0.03514
 
 0.02761
 
 Not queried
 
  0.7973318
 
       NA
 
       NA
  
 
  
 8
 
  53322451
 
  53322619
 
  ENSEMBL   UCSC 
 
  169
 
  4
 
 39633
 
 tss
 
 0.60827
 
 0.72750
 
 Not queried
 
  0.7972864
 
       NA
 
       NA
  
 
  
 1
 
 111746873
 
 111747324
 
  ENSEMBL   UCSC 
 
  452
 
  8
 
 3580
 
 island
 
 0.84694
 
 0.88886
 
 Not queried
 
  0.7971837
 
       NA
 
       NA
  
 
  
 X
 
  20160007
 
  20160252
 
  ENSEMBL   UCSC 
 
  246
 
  7
 
 41219
 
 tss
 
 0.01315
 
 0.02774
 
 Not queried
 
  0.7968881
 
       NA
 
       NA
  
 
  
 12
 
 125002940
 
 125003558
 
  ENSEMBL   UCSC 
 
  619
 
  7
 
 9375
 
 gene;island
 
 0.84093
 
 0.85132
 
 Not queried
 
  0.7968759
 
       NA
 
       NA
  
 
  
 5
 
 177697400
 
 177697669
 
  ENSEMBL   UCSC 
 
  270
 
  4
 
 31867
 
 gene
 
 0.87968
 
 0.86570
 
 Not queried
 
  0.7964752
 
       NA
 
       NA
  
 
  
 5
 
   1641593
 
   1641876
 
  ENSEMBL   UCSC 
 
  284
 
  6
 
 32577
 
 island
 
 0.09867
 
 0.12614
 
 Not queried
 
  0.7964474
 
       NA
 
       NA
  
 
  
 3
 
 197807842
 
 197808169
 
  ENSEMBL   UCSC 
 
  328
 
  5
 
 28786
 
 tss;island
 
 0.04253
 
 0.04079
 
 Not queried
 
  0.7963360
 
       NA
 
       NA
  
 
  
 19
 
  12476508
 
  12476577
 
  ENSEMBL   UCSC 
 
   70
 
  4
 
 20458
 
 tss
 
 0.00857
 
 0.01982
 
 Not queried
 
  0.7963350
 
       NA
 
       NA
  
 
  
 19
 
  39881208
 
  39882194
 
  ENSEMBL   UCSC 
 
  987
 
  9
 
 20733
 
 tss;island
 
 0.02120
 
 0.04136
 
 Not queried
 
  0.7962186
 
       NA
 
       NA
  
 
  
 20
 
  44746392
 
  44747006
 
  ENSEMBL   UCSC 
 
  615
 
 11
 
 26175
 
 island
 
 0.04401
 
 0.06481
 
 Not queried
 
  0.7962109
 
       NA
 
       NA
  
 
  
 1
 
   6265071
 
   6265417
 
  ENSEMBL   UCSC 
 
  347
 
  4
 
 1197
 
 tss;island
 
 0.02730
 
 0.01657
 
 Not queried
 
  0.7960445
 
       NA
 
       NA
  
 
  
 19
 
  54024182
 
  54024435
 
  ENSEMBL   UCSC 
 
  254
 
  4
 
 20166
 
 gene
 
 0.02233
 
 0.01299
 
 Not queried
 
  0.7960399
 
       NA
 
       NA
  
 
  
 3
 
  25705791
 
  25705861
 
  ENSEMBL   UCSC 
 
   71
 
  4
 
 28149
 
 tss
 
 0.05824
 
 0.09781
 
 Not queried
 
  0.7959892
 
       NA
 
       NA
  
 
  
 17
 
  37821447
 
  37821536
 
  ENSEMBL   UCSC 
 
   90
 
  4
 
 17279
 
 tss
 
 0.46036
 
 0.55783
 
 Not queried
 
  0.7957345
 
       NA
 
       NA
  
 
  
 8
 
  82754017
 
  82754497
 
  ENSEMBL   UCSC 
 
  481
 
  7
 
 39288
 
 gene;island
 
 0.03585
 
 0.04450
 
 Not queried
 
  0.7956496
 
       NA
 
       NA
  
 
  
 22
 
  36682843
 
  36682870
 
  ENSEMBL   UCSC 
 
   28
 
  4
 
 26816
 
 gene
 
 0.45440
 
 0.58953
 
 Not queried
 
  0.7956149
 
       NA
 
       NA
  
 
  
 3
 
 101443535
 
 101443992
 
  ENSEMBL   UCSC 
 
  458
 
  5
 
 27839
 
 gene
 
 0.01285
 
 0.02356
 
 Not queried
 
  0.7955134
 
       NA
 
       NA
  
 
  
 1
 
 173445917
 
 173446460
 
  ENSEMBL   UCSC 
 
  544
 
  9
 
 2092
 
 tss
 
 0.04129
 
 0.05586
 
 Not queried
 
  0.7953807
 
       NA
 
       NA
  
 
  
 15
 
  30917918
 
  30918065
 
  ENSEMBL   UCSC 
 
  148
 
  4
 
 13519
 
 tss;island
 
 0.00683
 
 0.01119
 
 Not queried
 
  0.7953465
 
       NA
 
       NA
  
 
  
 16
 
  11680075
 
  11680400
 
  ENSEMBL   UCSC 
 
  326
 
  4
 
 14635
 
 gene;island
 
 0.06595
 
 0.03580
 
 Not queried
 
  0.7953204
 
       NA
 
       NA
  
 
  
 X
 
 118370063
 
 118370187
 
  ENSEMBL   UCSC 
 
  125
 
  5
 
 41486
 
 tss
 
 0.02162
 
 0.03988
 
 Not queried
 
  0.7953122
 
       NA
 
       NA
  
 
  
 22
 
  45125218
 
  45125666
 
  ENSEMBL   UCSC 
 
  449
 
  4
 
 26854
 
 gene;island
 
 0.82131
 
 0.82435
 
 Not queried
 
  0.7951766
 
       NA
 
       NA
  
 
  
 17
 
  73663407
 
  73663551
 
  ENSEMBL   UCSC 
 
  145
 
  4
 
 16739
 
 gene
 
 0.03012
 
 0.02739
 
 Not queried
 
  0.7949700
 
       NA
 
       NA
  
 
  
 17
 
   3704471
 
   3704494
 
  ENSEMBL   UCSC 
 
   24
 
  4
 
 16311
 
 gene
 
 0.93051
 
 0.88331
 
 Not queried
 
  0.7947934
 
       NA
 
       NA
  
 
  
 19
 
   7827560
 
   7827894
 
  ENSEMBL   UCSC 
 
  335
 
  4
 
 20370
 
 tss
 
 0.72532
 
 0.60239
 
 Not queried
 
  0.7947918
 
       NA
 
       NA
  
 
  
 14
 
  59932101
 
  59932321
 
  ENSEMBL   UCSC 
 
  221
 
  9
 
 12402
 
 tss
 
 0.09088
 
 0.05344
 
 Not queried
 
  0.7946787
 
       NA
 
       NA
  
 
  
 19
 
   2740086
 
   2740392
 
  ENSEMBL   UCSC 
 
  307
 
  9
 
 20293
 
 tss
 
 0.02122
 
 0.03643
 
 Not queried
 
  0.7946408
 
       NA
 
       NA
  
 
  
 2
 
  73114144
 
  73114610
 
  ENSEMBL   UCSC 
 
  467
 
 10
 
 24368
 
 island
 
 0.03210
 
 0.05993
 
 Not queried
 
  0.7943258
 
       NA
 
       NA
  
 
  
 5
 
 139781174
 
 139781390
 
  ENSEMBL   UCSC 
 
  217
 
  8
 
 32282
 
 tss;island
 
 0.01835
 
 0.03089
 
 Not queried
 
  0.7943095
 
       NA
 
       NA
  
 
  
 10
 
 123355268
 
 123355748
 
  ENSEMBL   UCSC 
 
  481
 
  4
 
 4779
 
 gene;island
 
 0.09623
 
 0.04706
 
 Not queried
 
  0.7941740
 
       NA
 
       NA
  
 
  
 17
 
  48942734
 
  48942959
 
  ENSEMBL   UCSC 
 
  226
 
  4
 
 18528
 
 island
 
 0.03024
 
 0.04139
 
 Not queried
 
  0.7940747
 
       NA
 
       NA
  
 
  
 5
 
    893015
 
    893076
 
  ENSEMBL   UCSC 
 
   62
 
  6
 
 31461
 
 gene;tss;island
 
 0.01460
 
 0.02618
 
 Not queried
 
  0.7940521
 
       NA
 
       NA
  
 
  
 14
 
  57283942
 
  57284319
 
  ENSEMBL   UCSC 
 
  378
 
  4
 
 12065
 
 gene;island
 
 0.89558
 
 0.77222
 
 Not queried
 
  0.7940348
 
       NA
 
       NA
  
 
  
 3
 
 105587981
 
 105588247
 
  ENSEMBL   UCSC 
 
  267
 
  8
 
 28441
 
 tss
 
 0.01402
 
 0.02159
 
 Not queried
 
  0.7939529
 
       NA
 
       NA
  
 
  
 6
 
  30074078
 
  30074219
 
  ENSEMBL   UCSC 
 
  142
 
  6
 
 33658
 
 gene;island
 
 0.53204
 
 0.49515
 
 Not queried
 
  0.7937507
 
       NA
 
       NA
  
 
  
 10
 
  81838959
 
  81839181
 
  ENSEMBL   UCSC 
 
  223
 
  8
 
 5168
 
 tss
 
 0.02510
 
 0.04544
 
 Not queried
 
  0.7936519
 
       NA
 
       NA
  
 
  
 16
 
   8768488
 
   8768700
 
  ENSEMBL   UCSC 
 
  213
 
  4
 
 14621
 
 gene
 
 0.01353
 
 0.04363
 
 Not queried
 
  0.7936240
 
       NA
 
       NA
  
 
  
 17
 
  78120972
 
  78121542
 
  ENSEMBL   UCSC 
 
  571
 
  8
 
 17687
 
 tss
 
 0.01218
 
 0.02483
 
 Not queried
 
  0.7936155
 
       NA
 
       NA
  
 
  
 2
 
 242659698
 
 242660065
 
  ENSEMBL   UCSC 
 
  368
 
  4
 
 23103
 
 gene;island
 
 0.90950
 
 0.83892
 
 Not queried
 
  0.7933543
 
       NA
 
       NA
  
 
  
 11
 
   8615356
 
   8616172
 
  ENSEMBL   UCSC 
 
  817
 
 16
 
 8012
 
 island
 
 0.04589
 
 0.02061
 
 Not queried
 
  0.7931136
 
       NA
 
       NA
  
 
  
 2
 
 236866987
 
 236867278
 
  ENSEMBL   UCSC 
 
  292
 
  4
 
 23049
 
 gene;island
 
 0.86280
 
 0.89281
 
 Not queried
 
  0.7930612
 
       NA
 
       NA
  
 
  
 10
 
  27793255
 
  27793713
 
  ENSEMBL   UCSC 
 
  459
 
  4
 
 4528
 
 gene
 
 0.09567
 
 0.16046
 
 Not queried
 
  0.7929544
 
       NA
 
       NA
  
 
  
 12
 
 132929654
 
 132930230
 
  ENSEMBL   UCSC 
 
  577
 
  6
 
 11018
 
 island
 
 0.72710
 
 0.78000
 
 Not queried
 
  0.7929533
 
       NA
 
       NA
  
 
  
 2
 
 114514412
 
 114514736
 
  ENSEMBL   UCSC 
 
  325
 
  7
 
 23547
 
 tss
 
 0.02411
 
 0.02552
 
 Not queried
 
  0.7929132
 
       NA
 
       NA
  
 
  
 6
 
 100911949
 
 100912168
 
  ENSEMBL   UCSC 
 
  220
 
  4
 
 35076
 
 tss;island
 
 0.05638
 
 0.03177
 
 Not queried
 
  0.7928747
 
       NA
 
       NA
  
 
  
 19
 
  18942465
 
  18942818
 
  ENSEMBL   UCSC 
 
  354
 
  6
 
 21760
 
 island
 
 0.02037
 
 0.03250
 
 Not queried
 
  0.7926816
 
       NA
 
       NA
  
 
  
 1
 
  36396823
 
  36397042
 
  ENSEMBL   UCSC 
 
  220
 
  4
 
 423
 
 gene;island
 
 0.03935
 
 0.07352
 
 Not queried
 
  0.7926039
 
       NA
 
       NA
  
 
  
 17
 
   1381764
 
   1382042
 
  ENSEMBL   UCSC 
 
  279
 
  4
 
 16277
 
 gene;island
 
 0.89159
 
 0.79980
 
 Not queried
 
  0.7922595
 
       NA
 
       NA
  
 
  
 2
 
 219267198
 
 219267634
 
  ENSEMBL   UCSC 
 
  437
 
  5
 
 22981
 
 gene;island
 
 0.78392
 
 0.85710
 
 Not queried
 
  0.7921686
 
       NA
 
       NA
  
 
  
 19
 
  14063031
 
  14063234
 
  ENSEMBL   UCSC 
 
  204
 
  7
 
 20497
 
 tss
 
 0.00983
 
 0.01602
 
 Not queried
 
  0.7921327
 
       NA
 
       NA
  
 
  
 10
 
  14879979
 
  14880066
 
  ENSEMBL   UCSC 
 
   88
 
  5
 
 4942
 
 tss;island
 
 0.01472
 
 0.02370
 
 Not queried
 
  0.7920505
 
       NA
 
       NA
  
 
  
 22
 
  19746984
 
  19747140
 
  ENSEMBL   UCSC 
 
  157
 
  4
 
 26749
 
 gene;island
 
 0.94516
 
 0.90115
 
 Not queried
 
  0.7920278
 
       NA
 
       NA
  
 
  
 8
 
  12990898
 
  12991196
 
  ENSEMBL   UCSC 
 
  299
 
  7
 
 39497
 
 tss
 
 0.01869
 
 0.02792
 
 Not queried
 
  0.7917984
 
       NA
 
       NA
  
 
  
 19
 
  49403890
 
  49404253
 
  ENSEMBL   UCSC 
 
  364
 
  4
 
 22206
 
 island
 
 0.13468
 
 0.10117
 
 Not queried
 
  0.7917820
 
       NA
 
       NA
  
 
  
 6
 
  41040518
 
  41040849
 
  ENSEMBL   UCSC 
 
  332
 
  7
 
 34901
 
 tss;island
 
 0.02089
 
 0.02421
 
 Not queried
 
  0.7916806
 
       NA
 
       NA
  
 
  
 3
 
 121796580
 
 121796767
 
  ENSEMBL   UCSC 
 
  188
 
  4
 
 27871
 
 gene
 
 0.84362
 
 0.89675
 
 Not queried
 
  0.7916611
 
       NA
 
       NA
  
 
  
 20
 
  43439387
 
  43439688
 
  ENSEMBL   UCSC 
 
  302
 
  5
 
 25725
 
 tss;island
 
 0.02193
 
 0.01749
 
 Not queried
 
  0.7915365
 
       NA
 
       NA
  
 
  
 20
 
   5451661
 
   5451815
 
  ENSEMBL   UCSC 
 
  155
 
  6
 
 25543
 
 tss
 
 0.51986
 
 0.74150
 
 Not queried
 
  0.7915273
 
       NA
 
       NA
  
 
  
 13
 
  95655083
 
  95655579
 
  ENSEMBL   UCSC 
 
  497
 
  5
 
 11795
 
 island
 
 0.56604
 
 0.40964
 
 Not queried
 
  0.7913538
 
       NA
 
       NA
  
 
  
 5
 
   1225074
 
   1225397
 
  ENSEMBL   UCSC 
 
  324
 
  7
 
 31909
 
 tss
 
 0.89477
 
 0.89951
 
 Not queried
 
  0.7913296
 
       NA
 
       NA
  
 
  
 5
 
   1225074
 
   1225719
 
  ENSEMBL   UCSC 
 
  646
 
  9
 
 32556
 
 island
 
 0.88726
 
 0.87211
 
 Not queried
 
  0.7913296
 
       NA
 
       NA
  
 
  
 19
 
  34895039
 
  34895283
 
  ENSEMBL   UCSC 
 
  245
 
  7
 
 20655
 
 tss
 
 0.01034
 
 0.01794
 
 Not queried
 
  0.7912785
 
       NA
 
       NA
  
 
  
 1
 
   3566304
 
   3568245
 
  ENSEMBL   UCSC 
 
 1942
 
 38
 
 2556
 
 island
 
 0.50244
 
 0.36438
 
 Not queried
 
  0.7911864
 
       NA
 
       NA
  
 
  
 11
 
  47608047
 
  47608487
 
  ENSEMBL   UCSC 
 
  441
 
  4
 
 8248
 
 island
 
 0.53809
 
 0.64720
 
 Not queried
 
  0.7910894
 
       NA
 
       NA
  
 
  
 6
 
 144416767
 
 144416990
 
  ENSEMBL   UCSC 
 
  224
 
  6
 
 35207
 
 tss
 
 0.01175
 
 0.01822
 
 Not queried
 
  0.7910484
 
       NA
 
       NA
  
 
  
 6
 
 144416565
 
 144416990
 
  ENSEMBL   UCSC 
 
  426
 
  8
 
 36473
 
 island
 
 0.01175
 
 0.01822
 
 Not queried
 
  0.7910484
 
       NA
 
       NA
  
 
  
 16
 
  14379220
 
  14379541
 
  ENSEMBL   UCSC 
 
  322
 
  5
 
 15688
 
 island
 
 0.01179
 
 0.02107
 
 Not queried
 
  0.7910078
 
       NA
 
       NA
  
 
  
 X
 
 124453915
 
 124454138
 
  ENSEMBL   UCSC 
 
  224
 
  4
 
 42011
 
 island
 
 0.93133
 
 0.89643
 
 Not queried
 
  0.7909351
 
       NA
 
       NA
  
 
  
 7
 
  27260102
 
  27260585
 
  ENSEMBL   UCSC 
 
  484
 
  6
 
 38299
 
 island
 
 0.80592
 
 0.68955
 
 Not queried
 
  0.7909225
 
       NA
 
       NA
  
 
  
 10
 
 135097477
 
 135097800
 
  ENSEMBL   UCSC 
 
  324
 
  4
 
 4879
 
 gene;island
 
 0.82097
 
 0.89558
 
 Not queried
 
  0.7908068
 
       NA
 
       NA
  
 
  
 14
 
 103367306
 
 103367632
 
  ENSEMBL   UCSC 
 
  327
 
  4
 
 12206
 
 gene
 
 0.82709
 
 0.87339
 
 Not queried
 
  0.7907529
 
       NA
 
       NA
  
 
  
 17
 
  45266785
 
  45267114
 
  ENSEMBL   UCSC 
 
  330
 
  8
 
 17406
 
 tss
 
 0.01081
 
 0.02618
 
 Not queried
 
  0.7906309
 
       NA
 
       NA
  
 
  
 16
 
   4401344
 
   4401729
 
  ENSEMBL   UCSC 
 
  386
 
  5
 
 15034
 
 tss;island
 
 0.04478
 
 0.13443
 
 Not queried
 
  0.7906227
 
       NA
 
       NA
  
 
  
 6
 
  18264719
 
  18264854
 
  ENSEMBL   UCSC 
 
  136
 
  4
 
 35416
 
 island
 
 0.02833
 
 0.03128
 
 Not queried
 
  0.7905554
 
       NA
 
       NA
  
 
  
 3
 
 137487770
 
 137488094
 
  ENSEMBL   UCSC 
 
  325
 
  4
 
 29471
 
 island
 
 0.14456
 
 0.08909
 
 Not queried
 
  0.7905434
 
       NA
 
       NA
  
 
  
 16
 
   1251787
 
   1252140
 
  ENSEMBL   UCSC 
 
  354
 
  4
 
 14531
 
 gene;island
 
 0.86895
 
 0.93140
 
 Not queried
 
  0.7905333
 
       NA
 
       NA
  
 
  
 14
 
  68086581
 
  68086880
 
  ENSEMBL   UCSC 
 
  300
 
  4
 
 12091
 
 gene
 
 0.02367
 
 0.05083
 
 Not queried
 
  0.7905250
 
       NA
 
       NA
  
 
  
 5
 
 137514240
 
 137514312
 
  ENSEMBL   UCSC 
 
   73
 
  5
 
 31708
 
 gene
 
 0.02192
 
 0.03684
 
 Not queried
 
  0.7904778
 
       NA
 
       NA
  
 
  
 6
 
  28891819
 
  28893092
 
  ENSEMBL   UCSC 
 
 1274
 
 23
 
 34681
 
 tss
 
 0.03877
 
 0.02973
 
 Not queried
 
  0.7903956
 
       NA
 
       NA
  
 
  
 19
 
   7913785
 
   7913967
 
  ENSEMBL   UCSC 
 
  183
 
  4
 
 19752
 
 gene;island
 
 0.88725
 
 0.75194
 
 Not queried
 
  0.7903104
 
       NA
 
       NA
  
 
  
 15
 
  35384648
 
  35384864
 
  ENSEMBL   UCSC 
 
  217
 
  5
 
 13976
 
 island
 
 0.89831
 
 0.93883
 
 Not queried
 
  0.7902037
 
       NA
 
       NA
  
 
  
 10
 
 124713766
 
 124714384
 
  ENSEMBL   UCSC 
 
  619
 
 10
 
 6193
 
 island
 
 0.03444
 
 0.06634
 
 Not queried
 
  0.7901827
 
       NA
 
       NA
  
 
  
 6
 
 137366125
 
 137366545
 
  ENSEMBL   UCSC 
 
  421
 
  8
 
 36444
 
 island
 
 0.93874
 
 0.85783
 
 Not queried
 
  0.7901005
 
       NA
 
       NA
  
 
  
 1
 
  67772896
 
  67773175
 
  ENSEMBL   UCSC 
 
  280
 
  8
 
 3358
 
 island
 
 0.06369
 
 0.04478
 
 Not queried
 
  0.7900316
 
       NA
 
       NA
  
 
  
 13
 
  46425866
 
  46426263
 
  ENSEMBL   UCSC 
 
  398
 
  9
 
 11396
 
 tss
 
 0.63686
 
 0.75199
 
 Not queried
 
  0.7898809
 
       NA
 
       NA
  
 
  
 17
 
   5372292
 
   5372445
 
  ENSEMBL   UCSC 
 
  154
 
  4
 
 17013
 
 tss;island
 
 0.02126
 
 0.03676
 
 Not queried
 
  0.7897154
 
       NA
 
       NA
  
 
  
 10
 
  44198369
 
  44198617
 
  ENSEMBL   UCSC 
 
  249
 
  4
 
 5685
 
 island
 
 0.84294
 
 0.77522
 
 Not queried
 
  0.7897114
 
       NA
 
       NA
  
 
  
 15
 
  75018495
 
  75018852
 
  ENSEMBL   UCSC 
 
  358
 
 12
 
 13771
 
 tss;island
 
 0.02696
 
 0.02544
 
 Not queried
 
  0.7896618
 
       NA
 
       NA
  
 
  
 3
 
   3842527
 
   3842717
 
  ENSEMBL   UCSC 
 
  191
 
  4
 
 27597
 
 gene;island
 
 0.04106
 
 0.05017
 
 Not queried
 
  0.7896344
 
       NA
 
       NA
  
 
  
 7
 
 124405366
 
 124405760
 
  ENSEMBL   UCSC 
 
  395
 
  6
 
 38817
 
 island
 
 0.01092
 
 0.03885
 
 Not queried
 
  0.7896086
 
       NA
 
       NA
  
 
  
 7
 
 140396347
 
 140396638
 
  ENSEMBL   UCSC 
 
  292
 
  7
 
 37184
 
 gene;island
 
 0.01486
 
 0.02105
 
 Not queried
 
  0.7895270
 
       NA
 
       NA
  
 
  
 22
 
  43830900
 
  43831161
 
  ENSEMBL   UCSC 
 
  262
 
  4
 
 26849
 
 gene;island
 
 0.74956
 
 0.87198
 
 Not queried
 
  0.7895060
 
       NA
 
       NA
  
 
  
 14
 
  97263458
 
  97263801
 
  ENSEMBL   UCSC 
 
  344
 
  7
 
 13092
 
 island
 
 0.03014
 
 0.03384
 
 Not queried
 
  0.7894264
 
       NA
 
       NA
  
 
  
 19
 
  45754942
 
  45755260
 
  ENSEMBL   UCSC 
 
  319
 
  4
 
 20054
 
 gene
 
 0.05437
 
 0.04773
 
 Not queried
 
  0.7893525
 
       NA
 
       NA
  
 
  
 18
 
  76123613
 
  76123832
 
  ENSEMBL   UCSC 
 
  220
 
  4
 
 19554
 
 island
 
 0.70142
 
 0.82740
 
 Not queried
 
  0.7892415
 
       NA
 
       NA
  
 
  
 19
 
  46105466
 
  46105602
 
  ENSEMBL   UCSC 
 
  137
 
  5
 
 20840
 
 tss
 
 0.05025
 
 0.05342
 
 Not queried
 
  0.7890776
 
       NA
 
       NA
  
 
  
 2
 
  20189711
 
  20190349
 
  ENSEMBL   UCSC 
 
  639
 
 11
 
 24068
 
 island
 
 0.02589
 
 0.02461
 
 Not queried
 
  0.7890304
 
       NA
 
       NA
  
 
  
 22
 
  37595162
 
  37595533
 
  ENSEMBL   UCSC 
 
  372
 
  4
 
 27419
 
 island
 
 0.01715
 
 0.02274
 
 Not queried
 
  0.7890193
 
       NA
 
       NA
  
 
  
 7
 
 130132161
 
 130132453
 
  ENSEMBL   UCSC 
 
  293
 
 13
 
 37162
 
 gene;tss;island
 
 0.14953
 
 0.10291
 
 Not queried
 
  0.7889437
 
       NA
 
       NA
  
 
  
 6
 
  30860866
 
  30860960
 
  ENSEMBL   UCSC 
 
   95
 
  4
 
 33766
 
 gene
 
 0.57411
 
 0.69900
 
 Not queried
 
  0.7884756
 
       NA
 
       NA
  
 
  
 19
 
  45754802
 
  45755260
 
  ENSEMBL   UCSC 
 
  459
 
  5
 
 22092
 
 island
 
 0.05334
 
 0.05405
 
 Not queried
 
  0.7884380
 
       NA
 
       NA
  
 
  
 2
 
 109402912
 
 109403692
 
  ENSEMBL   UCSC 
 
  781
 
 13
 
 24564
 
 island
 
 0.01895
 
 0.02371
 
 Not queried
 
  0.7883311
 
       NA
 
       NA
  
 
  
 2
 
 197036400
 
 197036780
 
  ENSEMBL   UCSC 
 
  381
 
  9
 
 23755
 
 tss
 
 0.01088
 
 0.02542
 
 Not queried
 
  0.7882011
 
       NA
 
       NA
  
 
  
 1
 
 151882143
 
 151882406
 
  ENSEMBL   UCSC 
 
  264
 
  5
 
 1930
 
 tss
 
 0.07929
 
 0.02796
 
 Not queried
 
  0.7881751
 
       NA
 
       NA
  
 
  
 5
 
 114598137
 
 114599159
 
  ENSEMBL   UCSC 
 
 1023
 
 14
 
 32958
 
 island
 
 0.04049
 
 0.03632
 
 Not queried
 
  0.7880822
 
       NA
 
       NA
  
 
  
 7
 
 150652948
 
 150653195
 
  ENSEMBL   UCSC 
 
  248
 
  5
 
 37920
 
 tss
 
 0.74697
 
 0.68225
 
 Not queried
 
  0.7879487
 
       NA
 
       NA
  
 
  
 17
 
  62207299
 
  62207789
 
  ENSEMBL   UCSC 
 
  491
 
  7
 
 18621
 
 island
 
 0.01346
 
 0.02480
 
 Not queried
 
  0.7878316
 
       NA
 
       NA
  
 
  
 14
 
  24809297
 
  24809759
 
  ENSEMBL   UCSC 
 
  463
 
  4
 
 12301
 
 tss
 
 0.23659
 
 0.15207
 
 Not queried
 
  0.7877708
 
       NA
 
       NA
  
 
  
 3
 
 184290594
 
 184290794
 
  ENSEMBL   UCSC 
 
  201
 
  4
 
 28022
 
 gene;island
 
 0.90345
 
 0.86670
 
 Not queried
 
  0.7876178
 
       NA
 
       NA
  
 
  
 3
 
 184279336
 
 184279528
 
  ENSEMBL   UCSC 
 
  193
 
  6
 
 28719
 
 tss
 
 0.01530
 
 0.03039
 
 Not queried
 
  0.7875840
 
       NA
 
       NA
  
 
  
 3
 
  46792023
 
  46792462
 
  ENSEMBL   UCSC 
 
  440
 
  5
 
 29017
 
 island
 
 0.91627
 
 0.85210
 
 Not queried
 
  0.7874988
 
       NA
 
       NA
  
 
  
 10
 
  21784250
 
  21784432
 
  ENSEMBL   UCSC 
 
  183
 
  4
 
 4513
 
 gene;island
 
 0.01621
 
 0.02549
 
 Not queried
 
  0.7874789
 
       NA
 
       NA
  
 
  
 2
 
 197036243
 
 197036780
 
  ENSEMBL   UCSC 
 
  538
 
 11
 
 24935
 
 island
 
 0.01345
 
 0.02344
 
 Not queried
 
  0.7872670
 
       NA
 
       NA
  
 
  
 5
 
  56204834
 
  56205094
 
  ENSEMBL   UCSC 
 
  261
 
  7
 
 32014
 
 tss
 
 0.01327
 
 0.01737
 
 Not queried
 
  0.7868025
 
       NA
 
       NA
  
 
  
 19
 
  42721870
 
  42722189
 
  ENSEMBL   UCSC 
 
  320
 
  9
 
 20784
 
 tss
 
 0.03285
 
 0.05972
 
 Not queried
 
  0.7867414
 
       NA
 
       NA
  
 
  
 16
 
   4743284
 
   4743703
 
  ENSEMBL   UCSC 
 
  420
 
  5
 
 15043
 
 tss
 
 0.02299
 
 0.02921
 
 Not queried
 
  0.7867069
 
       NA
 
       NA
  
 
  
 6
 
  10838625
 
  10838914
 
  ENSEMBL   UCSC 
 
  290
 
  5
 
 35374
 
 island
 
 0.04135
 
 0.02877
 
 Not queried
 
  0.7866998
 
       NA
 
       NA
  
 
  
 8
 
 144798309
 
 144799022
 
  ENSEMBL   UCSC 
 
  714
 
 13
 
 40617
 
 island
 
 0.03331
 
 0.04357
 
 Not queried
 
  0.7866726
 
       NA
 
       NA
  
 
  
 5
 
  71615866
 
  71616505
 
  ENSEMBL   UCSC 
 
  640
 
 13
 
 32057
 
 tss;island
 
 0.01504
 
 0.03219
 
 Not queried
 
  0.7865773
 
       NA
 
       NA
  
 
  
 7
 
  43288635
 
  43288889
 
  ENSEMBL   UCSC 
 
  255
 
  4
 
 36918
 
 gene;island
 
 0.72285
 
 0.79659
 
 Not queried
 
  0.7864313
 
       NA
 
       NA
  
 
  
 2
 
 208575839
 
 208576199
 
  ENSEMBL   UCSC 
 
  361
 
  8
 
 23805
 
 tss
 
 0.01176
 
 0.02367
 
 Not queried
 
  0.7863024
 
       NA
 
       NA
  
 
  
 6
 
 126661274
 
 126661628
 
  ENSEMBL   UCSC 
 
  355
 
  5
 
 34403
 
 gene
 
 0.04219
 
 0.06836
 
 Not queried
 
  0.7862576
 
       NA
 
       NA
  
 
  
 3
 
  20081367
 
  20081585
 
  ENSEMBL   UCSC 
 
  219
 
  5
 
 28887
 
 island
 
 0.01398
 
 0.02459
 
 Not queried
 
  0.7861125
 
       NA
 
       NA
  
 
  
 11
 
  68519060
 
  68519252
 
  ENSEMBL   UCSC 
 
  193
 
  6
 
 7490
 
 tss
 
 0.05997
 
 0.06567
 
 Not queried
 
  0.7858664
 
       NA
 
       NA
  
 
  
 7
 
  73184526
 
  73184671
 
  ENSEMBL   UCSC 
 
  146
 
  7
 
 38504
 
 island
 
 0.01165
 
 0.02675
 
 Not queried
 
  0.7858553
 
       NA
 
       NA
  
 
  
 19
 
  41903010
 
  41903236
 
  ENSEMBL   UCSC 
 
  227
 
  5
 
 20024
 
 gene
 
 0.03205
 
 0.05643
 
 Not queried
 
  0.7857106
 
       NA
 
       NA
  
 
  
 11
 
  69468789
 
  69469175
 
  ENSEMBL   UCSC 
 
  387
 
  6
 
 6764
 
 gene
 
 0.89433
 
 0.91410
 
 Not queried
 
  0.7856206
 
       NA
 
       NA
  
 
  
 10
 
  64892616
 
  64892916
 
  ENSEMBL   UCSC 
 
  301
 
 10
 
 5088
 
 tss
 
 0.03735
 
 0.06260
 
 Not queried
 
  0.7854692
 
       NA
 
       NA
  
 
  
 12
 
 123464511
 
 123464823
 
  ENSEMBL   UCSC 
 
  313
 
  5
 
 10069
 
 tss
 
 0.07538
 
 0.04657
 
 Not queried
 
  0.7853569
 
       NA
 
       NA
  
 
  
 1
 
   8395307
 
   8395557
 
  ENSEMBL   UCSC 
 
  251
 
  5
 
 194
 
 gene;island
 
 0.89896
 
 0.83772
 
 Not queried
 
  0.7853496
 
       NA
 
       NA
  
 
  
 7
 
  27127448
 
  27127752
 
  ENSEMBL   UCSC 
 
  305
 
  4
 
 38270
 
 island
 
 0.81407
 
 0.72208
 
 Not queried
 
  0.7853428
 
       NA
 
       NA
  
 
  
 11
 
  64971926
 
  64972403
 
  ENSEMBL   UCSC 
 
  478
 
  4
 
 6685
 
 gene;island
 
 0.33653
 
 0.46426
 
 Not queried
 
  0.7850835
 
       NA
 
       NA
  
 
  
 12
 
   7055452
 
   7055683
 
  ENSEMBL   UCSC 
 
  232
 
  5
 
 9512
 
 tss
 
 0.05392
 
 0.08942
 
 Not queried
 
  0.7849675
 
       NA
 
       NA
  
 
  
 12
 
   7055452
 
   7056008
 
  ENSEMBL   UCSC 
 
  557
 
  7
 
 10195
 
 island
 
 0.05392
 
 0.07452
 
 Not queried
 
  0.7849675
 
       NA
 
       NA
  
 
  
 11
 
 118442837
 
 118443028
 
  ENSEMBL   UCSC 
 
  192
 
  6
 
 7714
 
 tss;island
 
 0.03604
 
 0.02315
 
 Not queried
 
  0.7848198
 
       NA
 
       NA
  
 
  
 19
 
  40023151
 
  40023459
 
  ENSEMBL   UCSC 
 
  309
 
  4
 
 19995
 
 gene
 
 0.01404
 
 0.02931
 
 Not queried
 
  0.7847901
 
       NA
 
       NA
  
 
  
 7
 
  27213893
 
  27214383
 
  ENSEMBL   UCSC 
 
  491
 
 10
 
 36876
 
 gene;island
 
 0.08812
 
 0.27579
 
 Not queried
 
  0.7847239
 
       NA
 
       NA
  
 
  
 15
 
  42066489
 
  42066583
 
  ENSEMBL   UCSC 
 
   95
 
  4
 
 13582
 
 tss;island
 
 0.01477
 
 0.01437
 
 Not queried
 
  0.7846933
 
       NA
 
       NA
  
 
  
 1
 
  27320200
 
  27320653
 
  ENSEMBL   UCSC 
 
  454
 
  6
 
 348
 
 gene
 
 0.02072
 
 0.03233
 
 Not queried
 
  0.7845739
 
       NA
 
       NA
  
 
  
 1
 
  27319836
 
  27320653
 
  ENSEMBL   UCSC 
 
  818
 
 12
 
 2902
 
 island
 
 0.01747
 
 0.02927
 
 Not queried
 
  0.7845739
 
       NA
 
       NA
  
 
  
 10
 
 129704949
 
 129705415
 
  ENSEMBL   UCSC 
 
  467
 
  6
 
 6231
 
 island
 
 0.06327
 
 0.08920
 
 Not queried
 
  0.7844412
 
       NA
 
       NA
  
 
  
 2
 
 154335014
 
 154335386
 
  ENSEMBL   UCSC 
 
  373
 
  9
 
 24754
 
 island
 
 0.02447
 
 0.03149
 
 Not queried
 
  0.7844217
 
       NA
 
       NA
  
 
  
 19
 
  48248531
 
  48248730
 
  ENSEMBL   UCSC 
 
  200
 
  6
 
 20873
 
 tss
 
 0.02552
 
 0.04192
 
 Not queried
 
  0.7844164
 
       NA
 
       NA
  
 
  
 10
 
 128994432
 
 128994702
 
  ENSEMBL   UCSC 
 
  271
 
  6
 
 5416
 
 tss
 
 0.01747
 
 0.03124
 
 Not queried
 
  0.7844033
 
       NA
 
       NA
  
 
  
 17
 
  21117669
 
  21117780
 
  ENSEMBL   UCSC 
 
  112
 
  4
 
 16451
 
 gene
 
 0.01841
 
 0.03816
 
 Not queried
 
  0.7843716
 
       NA
 
       NA
  
 
  
 1
 
  87170575
 
  87170951
 
  ENSEMBL   UCSC 
 
  377
 
  4
 
 608
 
 gene;island
 
 0.01926
 
 0.04101
 
 Not queried
 
  0.7842989
 
       NA
 
       NA
  
 
  
 17
 
  33905661
 
  33905888
 
  ENSEMBL   UCSC 
 
  228
 
  9
 
 17239
 
 tss
 
 0.02036
 
 0.02707
 
 Not queried
 
  0.7842012
 
       NA
 
       NA
  
 
  
 17
 
  40713676
 
  40714406
 
  ENSEMBL   UCSC 
 
  731
 
 11
 
 18307
 
 island
 
 0.02982
 
 0.05361
 
 Not queried
 
  0.7840049
 
       NA
 
       NA
  
 
  
 21
 
  47743775
 
  47744347
 
  ENSEMBL   UCSC 
 
  573
 
  9
 
 26560
 
 tss;island
 
 0.02453
 
 0.03383
 
 Not queried
 
  0.7839931
 
       NA
 
       NA
  
 
  
 16
 
   2021690
 
   2022090
 
  ENSEMBL   UCSC 
 
  401
 
  9
 
 15543
 
 island
 
 0.02643
 
 0.02628
 
 Not queried
 
  0.7838593
 
       NA
 
       NA
  
 
  
 1
 
  38099874
 
  38100467
 
  ENSEMBL   UCSC 
 
  594
 
  6
 
 434
 
 gene;island
 
 0.03779
 
 0.06313
 
 Not queried
 
  0.7838531
 
       NA
 
       NA
  
 
  
 17
 
  51899837
 
  51900115
 
  ENSEMBL   UCSC 
 
  279
 
  6
 
 17483
 
 tss
 
 0.84008
 
 0.73753
 
 Not queried
 
  0.7837760
 
       NA
 
       NA
  
 
  
 6
 
  32130375
 
  32130758
 
  ENSEMBL   UCSC 
 
  384
 
  7
 
 34001
 
 gene
 
 0.90926
 
 0.95342
 
 Not queried
 
  0.7837570
 
       NA
 
       NA
  
 
  
 6
 
  32130562
 
  32130758
 
  ENSEMBL   UCSC 
 
  197
 
  5
 
 35843
 
 island
 
 0.90926
 
 0.95342
 
 Not queried
 
  0.7837570
 
       NA
 
       NA
  
 
  
 8
 
  72274546
 
  72274615
 
  ENSEMBL   UCSC 
 
   70
 
  4
 
 39698
 
 tss
 
 0.01241
 
 0.02987
 
 Not queried
 
  0.7837203
 
       NA
 
       NA
  
 
  
 6
 
  31648514
 
  31649728
 
  ENSEMBL   UCSC 
 
 1215
 
 34
 
 35759
 
 island
 
 0.04996
 
 0.03364
 
 Not queried
 
  0.7836147
 
       NA
 
       NA
  
 
  
 11
 
  67236227
 
  67236722
 
  ENSEMBL   UCSC 
 
  496
 
  5
 
 6742
 
 gene
 
 0.01381
 
 0.04582
 
 Not queried
 
  0.7835979
 
       NA
 
       NA
  
 
  
 22
 
  47081634
 
  47082044
 
  ENSEMBL   UCSC 
 
  411
 
  4
 
 26874
 
 gene;island
 
 0.84486
 
 0.91480
 
 Not queried
 
  0.7835926
 
       NA
 
       NA
  
 
  
 6
 
 142468191
 
 142468362
 
  ENSEMBL   UCSC 
 
  172
 
  8
 
 35197
 
 tss
 
 0.01086
 
 0.01745
 
 Not queried
 
  0.7835273
 
       NA
 
       NA
  
 
  
 6
 
  32031395
 
  32031820
 
  ENSEMBL   UCSC 
 
  426
 
  4
 
 33965
 
 gene
 
 0.71432
 
 0.77833
 
 Not queried
 
  0.7833095
 
       NA
 
       NA
  
 
  
 3
 
   9745369
 
   9745610
 
  ENSEMBL   UCSC 
 
  242
 
  4
 
 28816
 
 island
 
 0.13421
 
 0.25593
 
 Not queried
 
  0.7833031
 
       NA
 
       NA
  
 
  
 12
 
  56367680
 
  56367828
 
  ENSEMBL   UCSC 
 
  149
 
  5
 
 9753
 
 tss
 
 0.02492
 
 0.04213
 
 Not queried
 
  0.7831873
 
       NA
 
       NA
  
 
  
 7
 
   4757669
 
   4757991
 
  ENSEMBL   UCSC 
 
  323
 
  4
 
 36781
 
 gene;island
 
 0.73479
 
 0.81557
 
 Not queried
 
  0.7830823
 
       NA
 
       NA
  
 
  
 15
 
  34393959
 
  34394376
 
  ENSEMBL   UCSC 
 
  418
 
  8
 
 13527
 
 tss;island
 
 0.01522
 
 0.01932
 
 Not queried
 
  0.7830512
 
       NA
 
       NA
  
 
  
 7
 
 107205042
 
 107205392
 
  ENSEMBL   UCSC 
 
  351
 
  5
 
 37117
 
 gene;tss;island
 
 0.44764
 
 0.05965
 
 Not queried
 
  0.7830181
 
       NA
 
       NA
  
 
  
 X
 
  49091760
 
  49091907
 
  ENSEMBL   UCSC 
 
  148
 
  4
 
 41315
 
 tss
 
 0.07437
 
 0.04528
 
 Not queried
 
  0.7829172
 
       NA
 
       NA
  
 
  
 1
 
   2410885
 
   2411643
 
  ENSEMBL   UCSC 
 
  759
 
  9
 
 85
 
 gene;island
 
 0.58609
 
 0.74694
 
 Not queried
 
  0.7828617
 
       NA
 
       NA
  
 
  
 10
 
 102295463
 
 102295624
 
  ENSEMBL   UCSC 
 
  162
 
  6
 
 5278
 
 tss
 
 0.01526
 
 0.03111
 
 Not queried
 
  0.7828462
 
       NA
 
       NA
  
 
  
 11
 
  17743610
 
  17743946
 
  ENSEMBL   UCSC 
 
  337
 
  5
 
 8083
 
 island
 
 0.01784
 
 0.04963
 
 Not queried
 
  0.7826929
 
       NA
 
       NA
  
 
  
 X
 
 105065721
 
 105066049
 
  ENSEMBL   UCSC 
 
  329
 
  4
 
 41453
 
 tss;island
 
 0.02062
 
 0.03406
 
 Not queried
 
  0.7826588
 
       NA
 
       NA
  
 
  
 3
 
  10182854
 
  10183274
 
  ENSEMBL   UCSC 
 
  421
 
  9
 
 28101
 
 tss
 
 0.31900
 
 0.38625
 
 Not queried
 
  0.7825886
 
       NA
 
       NA
  
 
  
 16
 
  56228442
 
  56228901
 
  ENSEMBL   UCSC 
 
  460
 
  9
 
 15212
 
 tss
 
 0.05231
 
 0.06238
 
 Not queried
 
  0.7824447
 
       NA
 
       NA
  
 
  
 21
 
  34442160
 
  34442674
 
  ENSEMBL   UCSC 
 
  515
 
 10
 
 26602
 
 island
 
 0.03720
 
 0.02288
 
 Not queried
 
  0.7823106
 
       NA
 
       NA
  
 
  
 19
 
  31640642
 
  31640711
 
  ENSEMBL   UCSC 
 
   70
 
  5
 
 20637
 
 tss
 
 0.95998
 
 0.81016
 
 Not queried
 
  0.7822701
 
       NA
 
       NA
  
 
  
 2
 
  45179312
 
  45180267
 
  ENSEMBL   UCSC 
 
  956
 
  9
 
 24227
 
 island
 
 0.06301
 
 0.04064
 
 Not queried
 
  0.7822679
 
       NA
 
       NA
  
 
  
 10
 
  75504089
 
  75504431
 
  ENSEMBL   UCSC 
 
  343
 
  5
 
 5836
 
 island
 
 0.01915
 
 0.03248
 
 Not queried
 
  0.7819481
 
       NA
 
       NA
  
 
  
 3
 
 172167445
 
 172167810
 
  ENSEMBL   UCSC 
 
  366
 
  5
 
 29623
 
 island
 
 0.02614
 
 0.11189
 
 Not queried
 
  0.7817670
 
       NA
 
       NA
  
 
  
 X
 
  16964556
 
  16964808
 
  ENSEMBL   UCSC 
 
  253
 
  7
 
 41208
 
 tss
 
 0.03017
 
 0.03183
 
 Not queried
 
  0.7817447
 
       NA
 
       NA
  
 
  
 X
 
  16964556
 
  16964965
 
  ENSEMBL   UCSC 
 
  410
 
  9
 
 41665
 
 island
 
 0.03017
 
 0.03183
 
 Not queried
 
  0.7817447
 
       NA
 
       NA
  
 
  
 19
 
  34112683
 
  34113010
 
  ENSEMBL   UCSC 
 
  328
 
  4
 
 21844
 
 island
 
 0.03329
 
 0.05591
 
 Not queried
 
  0.7815580
 
       NA
 
       NA
  
 
  
 11
 
  31824973
 
  31825226
 
  ENSEMBL   UCSC 
 
  254
 
  4
 
 6541
 
 gene;island
 
 0.19797
 
 0.29988
 
 Not queried
 
  0.7815494
 
       NA
 
       NA
  
 
  
 7
 
  23636744
 
  23636910
 
  ENSEMBL   UCSC 
 
  167
 
  5
 
 37409
 
 tss
 
 0.02489
 
 0.02519
 
 Not queried
 
  0.7815060
 
       NA
 
       NA
  
 
  
 12
 
 132855060
 
 132855381
 
  ENSEMBL   UCSC 
 
  322
 
  4
 
 9416
 
 gene;island
 
 0.72122
 
 0.80813
 
 Not queried
 
  0.7813948
 
       NA
 
       NA
  
 
  
 7
 
   5647658
 
   5647920
 
  ENSEMBL   UCSC 
 
  263
 
  4
 
 38170
 
 island
 
 0.77904
 
 0.90478
 
 Not queried
 
  0.7812349
 
       NA
 
       NA
  
 
  
 17
 
  73761019
 
  73761710
 
  ENSEMBL   UCSC 
 
  692
 
 10
 
 18726
 
 island
 
 0.02579
 
 0.02095
 
 Not queried
 
  0.7811402
 
       NA
 
       NA
  
 
  
 5
 
  61708168
 
  61708440
 
  ENSEMBL   UCSC 
 
  273
 
  8
 
 32027
 
 tss;island
 
 0.01269
 
 0.01997
 
 Not queried
 
  0.7811136
 
       NA
 
       NA
  
 
  
 11
 
  94964273
 
  94964516
 
  ENSEMBL   UCSC 
 
  244
 
  6
 
 7612
 
 tss
 
 0.01549
 
 0.02639
 
 Not queried
 
  0.7810629
 
       NA
 
       NA
  
 
  
 3
 
  97483466
 
  97483591
 
  ENSEMBL   UCSC 
 
  126
 
  6
 
 28415
 
 tss
 
 0.01131
 
 0.01825
 
 Not queried
 
  0.7810624
 
       NA
 
       NA
  
 
  
 12
 
 132972609
 
 132972940
 
  ENSEMBL   UCSC 
 
  332
 
  4
 
 11020
 
 island
 
 0.89374
 
 0.91830
 
 Not queried
 
  0.7810502
 
       NA
 
       NA
  
 
  
 11
 
    788406
 
    788714
 
  ENSEMBL   UCSC 
 
  309
 
  4
 
 6371
 
 gene;island
 
 0.60409
 
 0.71543
 
 Not queried
 
  0.7809889
 
       NA
 
       NA
  
 
  
 17
 
  58156134
 
  58156411
 
  ENSEMBL   UCSC 
 
  278
 
  5
 
 18582
 
 island
 
 0.01162
 
 0.02299
 
 Not queried
 
  0.7809599
 
       NA
 
       NA
  
 
  
 19
 
  54410030
 
  54410305
 
  ENSEMBL   UCSC 
 
  276
 
  4
 
 20173
 
 gene;island
 
 0.01646
 
 0.04640
 
 Not queried
 
  0.7808059
 
       NA
 
       NA
  
 
  
 11
 
  57232331
 
  57232742
 
  ENSEMBL   UCSC 
 
  412
 
  5
 
 6595
 
 gene;island
 
 0.65450
 
 0.52441
 
 Not queried
 
  0.7807502
 
       NA
 
       NA
  
 
  
 1
 
 206786000
 
 206786222
 
  ENSEMBL   UCSC 
 
  223
 
  6
 
 2211
 
 tss;island
 
 0.04384
 
 0.06247
 
 Not queried
 
  0.7805893
 
       NA
 
       NA
  
 
  
 10
 
  51572113
 
  51572718
 
  ENSEMBL   UCSC 
 
  606
 
 10
 
 4573
 
 gene;island
 
 0.04009
 
 0.06162
 
 Not queried
 
  0.7803920
 
       NA
 
       NA
  
 
  
 10
 
  51572113
 
  51572323
 
  ENSEMBL   UCSC 
 
  211
 
  6
 
 5062
 
 tss
 
 0.04009
 
 0.05277
 
 Not queried
 
  0.7803920
 
       NA
 
       NA
  
 
  
 12
 
 118814149
 
 118814623
 
  ENSEMBL   UCSC 
 
  475
 
  6
 
 10842
 
 island
 
 0.03489
 
 0.06886
 
 Not queried
 
  0.7803457
 
       NA
 
       NA
  
 
  
 15
 
  63334355
 
  63334761
 
  ENSEMBL   UCSC 
 
  407
 
  8
 
 13689
 
 tss
 
 0.03632
 
 0.04092
 
 Not queried
 
  0.7803398
 
       NA
 
       NA
  
 
  
 15
 
  63334355
 
  63335016
 
  ENSEMBL   UCSC 
 
  662
 
 10
 
 14173
 
 island
 
 0.02535
 
 0.03075
 
 Not queried
 
  0.7803398
 
       NA
 
       NA
  
 
  
 6
 
 142468191
 
 142468656
 
  ENSEMBL   UCSC 
 
  466
 
 11
 
 36462
 
 island
 
 0.01305
 
 0.01573
 
 Not queried
 
  0.7802283
 
       NA
 
       NA
  
 
  
 20
 
  24449668
 
  24449916
 
  ENSEMBL   UCSC 
 
  249
 
  7
 
 26014
 
 island
 
 0.10523
 
 0.05517
 
 Not queried
 
  0.7801853
 
       NA
 
       NA
  
 
  
 7
 
 140714412
 
 140714747
 
  ENSEMBL   UCSC 
 
  336
 
  9
 
 37186
 
 gene
 
 0.00846
 
 0.01733
 
 Not queried
 
  0.7801653
 
       NA
 
       NA
  
 
  
 17
 
  48474864
 
  48475044
 
  ENSEMBL   UCSC 
 
  181
 
  5
 
 17465
 
 tss
 
 0.00816
 
 0.01976
 
 Not queried
 
  0.7800652
 
       NA
 
       NA
  
 
  
 X
 
  51074836
 
  51075046
 
  ENSEMBL   UCSC 
 
  211
 
  4
 
 41323
 
 tss
 
 0.35823
 
 0.62718
 
 Not queried
 
  0.7800056
 
       NA
 
       NA
  
 
  
 15
 
  81666512
 
  81666847
 
  ENSEMBL   UCSC 
 
  336
 
  4
 
 13826
 
 tss
 
 0.77051
 
 0.59374
 
 Not queried
 
  0.7798126
 
       NA
 
       NA
  
 
  
 6
 
  32802924
 
  32803210
 
  ENSEMBL   UCSC 
 
  287
 
  8
 
 34078
 
 gene;island
 
 0.60051
 
 0.51616
 
 Not queried
 
  0.7796545
 
       NA
 
       NA
  
 
  
 1
 
 241802961
 
 241803367
 
  ENSEMBL   UCSC 
 
  407
 
  4
 
 1106
 
 gene;island
 
 0.02716
 
 0.05113
 
 Not queried
 
  0.7795359
 
       NA
 
       NA
  
 
  
 16
 
  23521605
 
  23521983
 
  ENSEMBL   UCSC 
 
  379
 
  5
 
 15731
 
 island
 
 0.01421
 
 0.01453
 
 Not queried
 
  0.7795068
 
       NA
 
       NA
  
 
  
 6
 
  31382027
 
  31382250
 
  ENSEMBL   UCSC 
 
  224
 
  6
 
 33812
 
 gene
 
 0.79234
 
 0.62513
 
 Not queried
 
  0.7795057
 
       NA
 
       NA
  
 
  
 11
 
  65337543
 
  65338038
 
  ENSEMBL   UCSC 
 
  496
 
  6
 
 8442
 
 island
 
 0.01184
 
 0.01850
 
 Not queried
 
  0.7794461
 
       NA
 
       NA
  
 
  
 17
 
  16256937
 
  16257223
 
  ENSEMBL   UCSC 
 
  287
 
  9
 
 17123
 
 tss;island
 
 0.03109
 
 0.02615
 
 Not queried
 
  0.7793867
 
       NA
 
       NA
  
 
  
 11
 
  58874608
 
  58874733
 
  ENSEMBL   UCSC 
 
  126
 
  7
 
 8280
 
 island
 
 0.05585
 
 0.07367
 
 Not queried
 
  0.7793383
 
       NA
 
       NA
  
 
  
 1
 
  11561497
 
  11562134
 
  ENSEMBL   UCSC 
 
  638
 
  6
 
 220
 
 gene;island
 
 0.88206
 
 0.92632
 
 Not queried
 
  0.7792237
 
       NA
 
       NA
  
 
  
 6
 
  32076079
 
  32076592
 
  ENSEMBL   UCSC 
 
  514
 
  9
 
 33987
 
 gene
 
 0.12832
 
 0.34202
 
 Not queried
 
  0.7792110
 
       NA
 
       NA
  
 
  
 17
 
  40761563
 
  40761818
 
  ENSEMBL   UCSC 
 
  256
 
 10
 
 17336
 
 tss
 
 0.01225
 
 0.01695
 
 Not queried
 
  0.7792011
 
       NA
 
       NA
  
 
  
 10
 
 132873657
 
 132873903
 
  ENSEMBL   UCSC 
 
  247
 
  5
 
 6256
 
 island
 
 0.89525
 
 0.87214
 
 Not queried
 
  0.7790764
 
       NA
 
       NA
  
 
  
 5
 
 145758586
 
 145758881
 
  ENSEMBL   UCSC 
 
  296
 
  5
 
 33222
 
 island
 
 0.70731
 
 0.60467
 
 Not queried
 
  0.7790625
 
       NA
 
       NA
  
 
  
 14
 
 102430244
 
 102430709
 
  ENSEMBL   UCSC 
 
  466
 
  5
 
 12611
 
 tss
 
 0.04912
 
 0.05397
 
 Not queried
 
  0.7790269
 
       NA
 
       NA
  
 
  
 19
 
  51330265
 
  51330469
 
  ENSEMBL   UCSC 
 
  205
 
  4
 
 20147
 
 gene;island
 
 0.80717
 
 0.68307
 
 Not queried
 
  0.7789779
 
       NA
 
       NA
  
 
  
 22
 
  43505892
 
  43506651
 
  ENSEMBL   UCSC 
 
  760
 
 24
 
 27158
 
 tss
 
 0.02015
 
 0.03487
 
 Not queried
 
  0.7789640
 
       NA
 
       NA
  
 
  
 22
 
  30868060
 
  30868226
 
  ENSEMBL   UCSC 
 
  167
 
  4
 
 27021
 
 tss
 
 0.54054
 
 0.37357
 
 Not queried
 
  0.7789589
 
       NA
 
       NA
  
 
  
 11
 
   2925439
 
   2926601
 
  ENSEMBL   UCSC 
 
 1163
 
 19
 
 6454
 
 gene;tss;island
 
 0.53794
 
 0.70098
 
 Not queried
 
  0.7787921
 
       NA
 
       NA
  
 
  
 1
 
  95392925
 
  95393138
 
  ENSEMBL   UCSC 
 
  214
 
  4
 
 1764
 
 tss;island
 
 0.10699
 
 0.06304
 
 Not queried
 
  0.7787510
 
       NA
 
       NA
  
 
  
 11
 
 109293945
 
 109294233
 
  ENSEMBL   UCSC 
 
  289
 
  6
 
 6874
 
 gene;island
 
 0.08251
 
 0.04915
 
 Not queried
 
  0.7787084
 
       NA
 
       NA
  
 
  
 12
 
 110211321
 
 110211413
 
  ENSEMBL   UCSC 
 
   93
 
  5
 
 9970
 
 tss
 
 0.47817
 
 0.57792
 
 Not queried
 
  0.7783968
 
       NA
 
       NA
  
 
  
 6
 
  37616410
 
  37616803
 
  ENSEMBL   UCSC 
 
  394
 
  5
 
 34258
 
 gene;island
 
 0.02947
 
 0.07485
 
 Not queried
 
  0.7783955
 
       NA
 
       NA
  
 
  
 10
 
 133747754
 
 133748048
 
  ENSEMBL   UCSC 
 
  295
 
  8
 
 6261
 
 island
 
 0.96424
 
 0.93975
 
 Not queried
 
  0.7783802
 
       NA
 
       NA
  
 
  
 20
 
  62317412
 
  62317768
 
  ENSEMBL   UCSC 
 
  357
 
  4
 
 25467
 
 gene;island
 
 0.76662
 
 0.88618
 
 Not queried
 
  0.7783595
 
       NA
 
       NA
  
 
  
 19
 
  54618544
 
  54618756
 
  ENSEMBL   UCSC 
 
  213
 
  4
 
 20177
 
 gene;tss;island
 
 0.03153
 
 0.04452
 
 Not queried
 
  0.7783173
 
       NA
 
       NA
  
 
  
 1
 
  18806517
 
  18806982
 
  ENSEMBL   UCSC 
 
  466
 
  4
 
 1291
 
 tss;island
 
 0.84464
 
 0.90006
 
 Not queried
 
  0.7783001
 
       NA
 
       NA
  
 
  
 19
 
  48896922
 
  48897279
 
  ENSEMBL   UCSC 
 
  358
 
  6
 
 20878
 
 tss;island
 
 0.02115
 
 0.03130
 
 Not queried
 
  0.7782258
 
       NA
 
       NA
  
 
  
 11
 
  76091674
 
  76092056
 
  ENSEMBL   UCSC 
 
  383
 
  5
 
 8647
 
 island
 
 0.02920
 
 0.04905
 
 Not queried
 
  0.7782223
 
       NA
 
       NA
  
 
  
 1
 
  46806025
 
  46806375
 
  ENSEMBL   UCSC 
 
  351
 
  9
 
 1596
 
 tss
 
 0.01716
 
 0.03049
 
 Not queried
 
  0.7781619
 
       NA
 
       NA
  
 
  
 1
 
  46806025
 
  46806563
 
  ENSEMBL   UCSC 
 
  539
 
 11
 
 3211
 
 island
 
 0.01716
 
 0.02707
 
 Not queried
 
  0.7781619
 
       NA
 
       NA
  
 
  
 20
 
  62184207
 
  62184397
 
  ENSEMBL   UCSC 
 
  191
 
  4
 
 26316
 
 island
 
 0.24379
 
 0.45002
 
 Not queried
 
  0.7780906
 
       NA
 
       NA
  
 
  
 2
 
 171673110
 
 171673272
 
  ENSEMBL   UCSC 
 
  163
 
  4
 
 24812
 
 island
 
 0.04283
 
 0.06247
 
 Not queried
 
  0.7778838
 
       NA
 
       NA
  
 
  
 11
 
  57102965
 
  57103334
 
  ENSEMBL   UCSC 
 
  370
 
  4
 
 6593
 
 gene
 
 0.00801
 
 0.01329
 
 Not queried
 
  0.7778007
 
       NA
 
       NA
  
 
  
 10
 
 120925308
 
 120925632
 
  ENSEMBL   UCSC 
 
  325
 
  7
 
 5381
 
 tss
 
 0.01492
 
 0.01405
 
 Not queried
 
  0.7777327
 
       NA
 
       NA
  
 
  
 4
 
   8430380
 
   8430496
 
  ENSEMBL   UCSC 
 
  117
 
  5
 
 29908
 
 gene;island
 
 0.02718
 
 0.02683
 
 Not queried
 
  0.7776960
 
       NA
 
       NA
  
 
  
 8
 
  99058293
 
  99058672
 
  ENSEMBL   UCSC 
 
  380
 
  4
 
 39769
 
 tss;island
 
 0.03038
 
 0.05024
 
 Not queried
 
  0.7775788
 
       NA
 
       NA
  
 
  
 17
 
  20811860
 
  20812310
 
  ENSEMBL   UCSC 
 
  451
 
  5
 
 18080
 
 island
 
 0.05401
 
 0.07486
 
 Not queried
 
  0.7773299
 
       NA
 
       NA
  
 
  
 5
 
 177581052
 
 177581072
 
  ENSEMBL   UCSC 
 
   21
 
  4
 
 32476
 
 tss
 
 0.02290
 
 0.03388
 
 Not queried
 
  0.7773192
 
       NA
 
       NA
  
 
  
 22
 
  41843111
 
  41843233
 
  ENSEMBL   UCSC 
 
  123
 
  6
 
 27130
 
 tss;island
 
 0.01069
 
 0.01777
 
 Not queried
 
  0.7771827
 
       NA
 
       NA
  
 
  
 9
 
 131038152
 
 131038602
 
  ENSEMBL   UCSC 
 
  451
 
  5
 
 40777
 
 tss;island
 
 0.03782
 
 0.06148
 
 Not queried
 
  0.7770990
 
       NA
 
       NA
  
 
  
 1
 
 110577591
 
 110577941
 
  ENSEMBL   UCSC 
 
  351
 
  5
 
 672
 
 gene;island
 
 0.02587
 
 0.05759
 
 Not queried
 
  0.7768241
 
       NA
 
       NA
  
 
  
 2
 
 220252704
 
 220252832
 
  ENSEMBL   UCSC 
 
  129
 
  4
 
 23867
 
 tss
 
 0.10925
 
 0.11592
 
 Not queried
 
  0.7765192
 
       NA
 
       NA
  
 
  
 15
 
  40649930
 
  40650336
 
  ENSEMBL   UCSC 
 
  407
 
  8
 
 13558
 
 tss;island
 
 0.06854
 
 0.04113
 
 Not queried
 
  0.7764139
 
       NA
 
       NA
  
 
  
 8
 
  27449716
 
  27450062
 
  ENSEMBL   UCSC 
 
  347
 
  6
 
 40117
 
 island
 
 0.01322
 
 0.03147
 
 Not queried
 
  0.7763014
 
       NA
 
       NA
  
 
  
 6
 
  10413233
 
  10413523
 
  ENSEMBL   UCSC 
 
  291
 
  4
 
 33494
 
 gene;tss;island
 
 0.06826
 
 0.08524
 
 Not queried
 
  0.7762879
 
       NA
 
       NA
  
 
  
 2
 
 109558966
 
 109559185
 
  ENSEMBL   UCSC 
 
  220
 
  4
 
 22784
 
 gene;island
 
 0.92904
 
 0.89370
 
 Not queried
 
  0.7761802
 
       NA
 
       NA
  
 
  
 11
 
 134152000
 
 134152202
 
  ENSEMBL   UCSC 
 
  203
 
  4
 
 6975
 
 gene
 
 0.79365
 
 0.83344
 
 Not queried
 
  0.7761058
 
       NA
 
       NA
  
 
  
 1
 
  47900197
 
  47900320
 
  ENSEMBL   UCSC 
 
  124
 
  4
 
 1607
 
 tss;island
 
 0.26730
 
 0.19192
 
 Not queried
 
  0.7759265
 
       NA
 
       NA
  
 
  
 6
 
  43197245
 
  43197675
 
  ENSEMBL   UCSC 
 
  431
 
  8
 
 34931
 
 tss
 
 0.04874
 
 0.03428
 
 Not queried
 
  0.7758269
 
       NA
 
       NA
  
 
  
 5
 
  75013131
 
  75013682
 
  ENSEMBL   UCSC 
 
  552
 
 11
 
 32823
 
 island
 
 0.02610
 
 0.05047
 
 Not queried
 
  0.7757489
 
       NA
 
       NA
  
 
  
 11
 
   2162183
 
   2162930
 
  ENSEMBL   UCSC 
 
  748
 
 19
 
 6430
 
 gene;island
 
 0.35350
 
 0.47586
 
 Not queried
 
  0.7757292
 
       NA
 
       NA
  
 
  
 19
 
  37825307
 
  37825446
 
  ENSEMBL   UCSC 
 
  140
 
  6
 
 20700
 
 tss;island
 
 0.97568
 
 0.95957
 
 Not queried
 
  0.7755799
 
       NA
 
       NA
  
 
  
 1
 
  26758808
 
  26758996
 
  ENSEMBL   UCSC 
 
  189
 
  5
 
 340
 
 gene
 
 0.02367
 
 0.03012
 
 Not queried
 
  0.7753955
 
       NA
 
       NA
  
 
  
 X
 
 107682766
 
 107682966
 
  ENSEMBL   UCSC 
 
  201
 
  5
 
 41465
 
 tss
 
 0.01750
 
 0.02176
 
 Not queried
 
  0.7751869
 
       NA
 
       NA
  
 
  
 1
 
  93913424
 
  93913524
 
  ENSEMBL   UCSC 
 
  101
 
  4
 
 1755
 
 tss;island
 
 0.02122
 
 0.04778
 
 Not queried
 
  0.7750127
 
       NA
 
       NA
  
 
  
 19
 
   5913854
 
   5914158
 
  ENSEMBL   UCSC 
 
  305
 
  5
 
 20350
 
 tss
 
 0.75766
 
 0.89453
 
 Not queried
 
  0.7749428
 
       NA
 
       NA
  
 
  
 7
 
 157412628
 
 157413057
 
  ENSEMBL   UCSC 
 
  430
 
  5
 
 37269
 
 gene;island
 
 0.84468
 
 0.89216
 
 Not queried
 
  0.7748758
 
       NA
 
       NA
  
 
  
 20
 
   4764077
 
   4764312
 
  ENSEMBL   UCSC 
 
  236
 
  5
 
 25311
 
 gene
 
 0.92575
 
 0.95522
 
 Not queried
 
  0.7748282
 
       NA
 
       NA
  
 
  
 17
 
  41832753
 
  41833324
 
  ENSEMBL   UCSC 
 
  572
 
  6
 
 16563
 
 gene;island
 
 0.92984
 
 0.88191
 
 Not queried
 
  0.7748130
 
       NA
 
       NA
  
 
  
 14
 
  21457976
 
  21458155
 
  ENSEMBL   UCSC 
 
  180
 
  4
 
 11980
 
 gene
 
 0.01238
 
 0.02102
 
 Not queried
 
  0.7747434
 
       NA
 
       NA
  
 
  
 17
 
  79799358
 
  79799549
 
  ENSEMBL   UCSC 
 
  192
 
  5
 
 18909
 
 island
 
 0.68920
 
 0.82638
 
 Not queried
 
  0.7747306
 
       NA
 
       NA
  
 
  
 17
 
  73937173
 
  73937416
 
  ENSEMBL   UCSC 
 
  244
 
  6
 
 17633
 
 tss
 
 0.01632
 
 0.02637
 
 Not queried
 
  0.7746854
 
       NA
 
       NA
  
 
  
 8
 
   8859686
 
   8860691
 
  ENSEMBL   UCSC 
 
 1006
 
 11
 
 39996
 
 island
 
 0.04413
 
 0.04921
 
 Not queried
 
  0.7744025
 
       NA
 
       NA
  
 
  
 17
 
  40715222
 
  40715281
 
  ENSEMBL   UCSC 
 
   60
 
  5
 
 16545
 
 gene;island
 
 0.73337
 
 0.57426
 
 Not queried
 
  0.7742327
 
       NA
 
       NA
  
 
  
 19
 
  58011309
 
  58011582
 
  ENSEMBL   UCSC 
 
  274
 
  4
 
 20209
 
 gene
 
 0.01570
 
 0.02480
 
 Not queried
 
  0.7741101
 
       NA
 
       NA
  
 
  
 14
 
 101350645
 
 101351197
 
  ENSEMBL   UCSC 
 
  553
 
  8
 
 12586
 
 tss;island
 
 0.91674
 
 0.91330
 
 Not queried
 
  0.7739059
 
       NA
 
       NA
  
 
  
 1
 
 113161597
 
 113162646
 
  ENSEMBL   UCSC 
 
 1050
 
 15
 
 1833
 
 tss;island
 
 0.01497
 
 0.03269
 
 Not queried
 
  0.7737678
 
       NA
 
       NA
  
 
  
 7
 
 129931572
 
 129932033
 
  ENSEMBL   UCSC 
 
  462
 
  4
 
 37833
 
 tss
 
 0.95626
 
 0.92981
 
 Not queried
 
  0.7737599
 
       NA
 
       NA
  
 
  
 2
 
  23913322
 
  23913568
 
  ENSEMBL   UCSC 
 
  247
 
  4
 
 22559
 
 gene;island
 
 0.84075
 
 0.83227
 
 Not queried
 
  0.7737243
 
       NA
 
       NA
  
 
  
 11
 
  63912583
 
  63912769
 
  ENSEMBL   UCSC 
 
  187
 
  4
 
 6648
 
 gene
 
 0.91383
 
 0.95871
 
 Not queried
 
  0.7736879
 
       NA
 
       NA
  
 
  
 11
 
  64512829
 
  64513289
 
  ENSEMBL   UCSC 
 
  461
 
  6
 
 7381
 
 tss;island
 
 0.07905
 
 0.05071
 
 Not queried
 
  0.7736422
 
       NA
 
       NA
  
 
  
 7
 
  92158172
 
  92158437
 
  ENSEMBL   UCSC 
 
  266
 
  4
 
 37022
 
 gene
 
 0.01249
 
 0.01101
 
 Not queried
 
  0.7736357
 
       NA
 
       NA
  
 
  
 11
 
  60282712
 
  60282972
 
  ENSEMBL   UCSC 
 
  261
 
  9
 
 8288
 
 island
 
 0.93994
 
 0.87572
 
 Not queried
 
  0.7734499
 
       NA
 
       NA
  
 
  
 3
 
 159706823
 
 159707145
 
  ENSEMBL   UCSC 
 
  323
 
  4
 
 27970
 
 gene;island
 
 0.13305
 
 0.07604
 
 Not queried
 
  0.7733111
 
       NA
 
       NA
  
 
  
 11
 
  31845148
 
  31845639
 
  ENSEMBL   UCSC 
 
  492
 
  5
 
 8147
 
 island
 
 0.12568
 
 0.06907
 
 Not queried
 
  0.7732965
 
       NA
 
       NA
  
 
  
 20
 
  51589616
 
  51589985
 
  ENSEMBL   UCSC 
 
  370
 
  4
 
 25419
 
 gene;island
 
 0.07028
 
 0.04399
 
 Not queried
 
  0.7732622
 
       NA
 
       NA
  
 
  
 22
 
  23484001
 
  23484600
 
  ENSEMBL   UCSC 
 
  600
 
 11
 
 27305
 
 island
 
 0.02100
 
 0.03915
 
 Not queried
 
  0.7732550
 
       NA
 
       NA
  
 
  
 13
 
  43566114
 
  43566642
 
  ENSEMBL   UCSC 
 
  529
 
 12
 
 11669
 
 island
 
 0.04493
 
 0.06733
 
 Not queried
 
  0.7732407
 
       NA
 
       NA
  
 
  
 16
 
  11350045
 
  11350371
 
  ENSEMBL   UCSC 
 
  327
 
  9
 
 15060
 
 tss;island
 
 0.05747
 
 0.07725
 
 Not queried
 
  0.7731315
 
       NA
 
       NA
  
 
  
 7
 
  19748289
 
  19748896
 
  ENSEMBL   UCSC 
 
  608
 
 11
 
 38224
 
 island
 
 0.01852
 
 0.03031
 
 Not queried
 
  0.7731276
 
       NA
 
       NA
  
 
  
 11
 
  50227079
 
  50227265
 
  ENSEMBL   UCSC 
 
  187
 
  4
 
 8256
 
 island
 
 0.75658
 
 0.61348
 
 Not queried
 
  0.7727397
 
       NA
 
       NA
  
 
  
 7
 
  87229552
 
  87230344
 
  ENSEMBL   UCSC 
 
  793
 
  9
 
 37009
 
 gene;island
 
 0.03235
 
 0.05640
 
 Not queried
 
  0.7726840
 
       NA
 
       NA
  
 
  
 6
 
  32187918
 
  32188404
 
  ENSEMBL   UCSC 
 
  487
 
  5
 
 34036
 
 gene
 
 0.41814
 
 0.20802
 
 Not queried
 
  0.7726294
 
       NA
 
       NA
  
 
  
 15
 
  89951787
 
  89952299
 
  ENSEMBL   UCSC 
 
  513
 
  6
 
 14405
 
 island
 
 0.64003
 
 0.76136
 
 Not queried
 
  0.7725345
 
       NA
 
       NA
  
 
  
 11
 
  65409760
 
  65410126
 
  ENSEMBL   UCSC 
 
  367
 
  4
 
 6697
 
 gene;island
 
 0.67324
 
 0.55650
 
 Not queried
 
  0.7724790
 
       NA
 
       NA
  
 
  
 12
 
 115122028
 
 115122304
 
  ENSEMBL   UCSC 
 
  277
 
  6
 
 10011
 
 tss;island
 
 0.01789
 
 0.02612
 
 Not queried
 
  0.7724632
 
       NA
 
       NA
  
 
  
 10
 
  88149210
 
  88149632
 
  ENSEMBL   UCSC 
 
  423
 
  4
 
 5884
 
 island
 
 0.01621
 
 0.03724
 
 Not queried
 
  0.7723813
 
       NA
 
       NA
  
 
  
 X
 
 114468681
 
 114468879
 
  ENSEMBL   UCSC 
 
  199
 
  4
 
 41476
 
 tss
 
 0.04748
 
 0.03249
 
 Not queried
 
  0.7722716
 
       NA
 
       NA
  
 
  
 8
 
 105379368
 
 105379726
 
  ENSEMBL   UCSC 
 
  359
 
  5
 
 40458
 
 island
 
 0.76905
 
 0.86881
 
 Not queried
 
  0.7721184
 
       NA
 
       NA
  
 
  
 5
 
  73937141
 
  73937330
 
  ENSEMBL   UCSC 
 
  190
 
  5
 
 32815
 
 island
 
 0.01235
 
 0.00926
 
 Not queried
 
  0.7721162
 
       NA
 
       NA
  
 
  
 6
 
  31779308
 
  31779728
 
  ENSEMBL   UCSC 
 
  421
 
 11
 
 33901
 
 gene;island
 
 0.67501
 
 0.68669
 
 Not queried
 
  0.7720365
 
       NA
 
       NA
  
 
  
 7
 
  65540778
 
  65541137
 
  ENSEMBL   UCSC 
 
  360
 
  5
 
 36966
 
 gene
 
 0.03424
 
 0.05707
 
 Not queried
 
  0.7717728
 
       NA
 
       NA
  
 
  
 5
 
   1463488
 
   1463850
 
  ENSEMBL   UCSC 
 
  363
 
  4
 
 31486
 
 gene;island
 
 0.92972
 
 0.86693
 
 Not queried
 
  0.7716494
 
       NA
 
       NA
  
 
  
 8
 
  41504884
 
  41505042
 
  ENSEMBL   UCSC 
 
  159
 
  5
 
 39607
 
 tss
 
 0.75123
 
 0.64427
 
 Not queried
 
  0.7716217
 
       NA
 
       NA
  
 
  
 15
 
  89631044
 
  89631334
 
  ENSEMBL   UCSC 
 
  291
 
  5
 
 13855
 
 tss;island
 
 0.02146
 
 0.04729
 
 Not queried
 
  0.7714513
 
       NA
 
       NA
  
 
  
 14
 
  75643373
 
  75643727
 
  ENSEMBL   UCSC 
 
  355
 
  7
 
 12489
 
 tss
 
 0.01028
 
 0.00918
 
 Not queried
 
  0.7712883
 
       NA
 
       NA
  
 
  
 14
 
  75642944
 
  75643727
 
  ENSEMBL   UCSC 
 
  784
 
 11
 
 12985
 
 island
 
 0.01028
 
 0.01771
 
 Not queried
 
  0.7712883
 
       NA
 
       NA
  
 
  
 12
 
  57569768
 
  57569940
 
  ENSEMBL   UCSC 
 
  173
 
  4
 
 9158
 
 gene;island
 
 0.53471
 
 0.70589
 
 Not queried
 
  0.7712528
 
       NA
 
       NA
  
 
  
 17
 
  48545805
 
  48546620
 
  ENSEMBL   UCSC 
 
  816
 
 12
 
 16652
 
 gene;island
 
 0.88462
 
 0.81390
 
 Not queried
 
  0.7711716
 
       NA
 
       NA
  
 
  
 6
 
  32363084
 
  32363325
 
  ENSEMBL   UCSC 
 
  242
 
  9
 
 34043
 
 gene
 
 0.76278
 
 0.65329
 
 Not queried
 
  0.7710729
 
       NA
 
       NA
  
 
  
 19
 
  44079527
 
  44079985
 
  ENSEMBL   UCSC 
 
  459
 
  9
 
 22055
 
 island
 
 0.01479
 
 0.02697
 
 Not queried
 
  0.7709095
 
       NA
 
       NA
  
 
  
 22
 
  24114690
 
  24115027
 
  ENSEMBL   UCSC 
 
  338
 
  7
 
 26970
 
 tss
 
 0.06138
 
 0.07904
 
 Not queried
 
  0.7708319
 
       NA
 
       NA
  
 
  
 3
 
 185655949
 
 185656289
 
  ENSEMBL   UCSC 
 
  341
 
  4
 
 28726
 
 tss
 
 0.71806
 
 0.59526
 
 Not queried
 
  0.7707536
 
       NA
 
       NA
  
 
  
 7
 
   2565944
 
   2566263
 
  ENSEMBL   UCSC 
 
  320
 
  5
 
 36743
 
 gene;island
 
 0.91510
 
 0.93328
 
 Not queried
 
  0.7706567
 
       NA
 
       NA
  
 
  
 7
 
  44163058
 
  44163430
 
  ENSEMBL   UCSC 
 
  373
 
  8
 
 38393
 
 island
 
 0.01535
 
 0.02937
 
 Not queried
 
  0.7706035
 
       NA
 
       NA
  
 
  
 17
 
  46719577
 
  46720050
 
  ENSEMBL   UCSC 
 
  474
 
  5
 
 18461
 
 island
 
 0.45128
 
 0.54819
 
 Not queried
 
  0.7704839
 
       NA
 
       NA
  
 
  
 11
 
   7041194
 
   7041987
 
  ENSEMBL   UCSC 
 
  794
 
 18
 
 7099
 
 tss;island
 
 0.05305
 
 0.02371
 
 Not queried
 
  0.7704632
 
       NA
 
       NA
  
 
  
 11
 
  46638831
 
  46639039
 
  ENSEMBL   UCSC 
 
  209
 
  6
 
 6584
 
 gene
 
 0.01207
 
 0.01640
 
 Not queried
 
  0.7704369
 
       NA
 
       NA
  
 
  
 18
 
  74728834
 
  74729251
 
  ENSEMBL   UCSC 
 
  418
 
 12
 
 19100
 
 gene
 
 0.87473
 
 0.88573
 
 Not queried
 
  0.7704138
 
       NA
 
       NA
  
 
  
 16
 
   4466593
 
   4466888
 
  ENSEMBL   UCSC 
 
  296
 
  5
 
 15636
 
 island
 
 0.02694
 
 0.02782
 
 Not queried
 
  0.7704050
 
       NA
 
       NA
  
 
  
 11
 
    746801
 
    747301
 
  ENSEMBL   UCSC 
 
  501
 
  6
 
 7005
 
 tss
 
 0.07429
 
 0.03172
 
 Not queried
 
  0.7703277
 
       NA
 
       NA
  
 
  
 13
 
 114114198
 
 114114583
 
  ENSEMBL   UCSC 
 
  386
 
  5
 
 11260
 
 gene
 
 0.81576
 
 0.87436
 
 Not queried
 
  0.7702571
 
       NA
 
       NA
  
 
  
 2
 
 119600348
 
 119600627
 
  ENSEMBL   UCSC 
 
  280
 
  5
 
 22803
 
 gene;island
 
 0.22011
 
 0.12077
 
 Not queried
 
  0.7702561
 
       NA
 
       NA
  
 
  
 10
 
  73078578
 
  73079101
 
  ENSEMBL   UCSC 
 
  524
 
  7
 
 5808
 
 island
 
 0.07489
 
 0.12364
 
 Not queried
 
  0.7700659
 
       NA
 
       NA
  
 
  
 8
 
  23539627
 
  23539955
 
  ENSEMBL   UCSC 
 
  329
 
  4
 
 39181
 
 gene;island
 
 0.05026
 
 0.03228
 
 Not queried
 
  0.7700012
 
       NA
 
       NA
  
 
  
 11
 
  36615733
 
  36616048
 
  ENSEMBL   UCSC 
 
  316
 
  6
 
 7230
 
 tss
 
 0.02673
 
 0.04977
 
 Not queried
 
  0.7699483
 
       NA
 
       NA
  
 
  
 8
 
  12990213
 
  12991196
 
  ENSEMBL   UCSC 
 
  984
 
 12
 
 39149
 
 gene;island
 
 0.01750
 
 0.02247
 
 Not queried
 
  0.7699290
 
       NA
 
       NA
  
 
  
 1
 
  85156187
 
  85156797
 
  ENSEMBL   UCSC 
 
  611
 
  9
 
 1713
 
 tss;island
 
 0.02235
 
 0.03061
 
 Not queried
 
  0.7699176
 
       NA
 
       NA
  
 
  
 7
 
  96339249
 
  96339613
 
  ENSEMBL   UCSC 
 
  365
 
  8
 
 37667
 
 tss
 
 0.07382
 
 0.08191
 
 Not queried
 
  0.7698502
 
       NA
 
       NA
  
 
  
 11
 
  65479281
 
  65479678
 
  ENSEMBL   UCSC 
 
  398
 
  9
 
 8454
 
 island
 
 0.01723
 
 0.02175
 
 Not queried
 
  0.7695043
 
       NA
 
       NA
  
 
  
 9
 
 101017931
 
 101018113
 
  ENSEMBL   UCSC 
 
  183
 
  4
 
 40856
 
 island
 
 0.01176
 
 0.02627
 
 Not queried
 
  0.7694894
 
       NA
 
       NA
  
 
  
 9
 
  27573650
 
  27573986
 
  ENSEMBL   UCSC 
 
  337
 
  4
 
 40740
 
 tss;island
 
 0.01291
 
 0.01782
 
 Not queried
 
  0.7692732
 
       NA
 
       NA
  
 
  
 4
 
 140201065
 
 140201652
 
  ENSEMBL   UCSC 
 
  588
 
  9
 
 31259
 
 island
 
 0.27185
 
 0.08323
 
 Not queried
 
  0.7692015
 
       NA
 
       NA
  
 
  
 1
 
  36235406
 
  36235538
 
  ENSEMBL   UCSC 
 
  133
 
  5
 
 422
 
 gene
 
 0.02206
 
 0.02695
 
 Not queried
 
  0.7690490
 
       NA
 
       NA
  
 
  
 10
 
  75910788
 
  75911181
 
  ENSEMBL   UCSC 
 
  394
 
  6
 
 5151
 
 tss;island
 
 0.01556
 
 0.02994
 
 Not queried
 
  0.7690427
 
       NA
 
       NA
  
 
  
 13
 
 114065669
 
 114066074
 
  ENSEMBL   UCSC 
 
  406
 
  4
 
 11934
 
 island
 
 0.82176
 
 0.89974
 
 Not queried
 
  0.7689965
 
       NA
 
       NA
  
 
  
 14
 
 101520369
 
 101520662
 
  ENSEMBL   UCSC 
 
  294
 
  4
 
 12600
 
 tss
 
 0.85588
 
 0.77229
 
 Not queried
 
  0.7689352
 
       NA
 
       NA
  
 
  
 2
 
   7005942
 
   7006004
 
  ENSEMBL   UCSC 
 
   63
 
  4
 
 23128
 
 tss
 
 0.07561
 
 0.12442
 
 Not queried
 
  0.7689196
 
       NA
 
       NA
  
 
  
 4
 
 146682377
 
 146682755
 
  ENSEMBL   UCSC 
 
  379
 
  4
 
 30101
 
 gene;island
 
 0.90228
 
 0.93610
 
 Not queried
 
  0.7688955
 
       NA
 
       NA
  
 
  
 1
 
  32645788
 
  32646132
 
  ENSEMBL   UCSC 
 
  345
 
  5
 
 386
 
 gene;island
 
 0.03348
 
 0.07236
 
 Not queried
 
  0.7688250
 
       NA
 
       NA
  
 
  
 4
 
  78783825
 
  78784263
 
  ENSEMBL   UCSC 
 
  439
 
  4
 
 29984
 
 gene
 
 0.00950
 
 0.01544
 
 Not queried
 
  0.7684568
 
       NA
 
       NA
  
 
  
 11
 
  94227180
 
  94227583
 
  ENSEMBL   UCSC 
 
  404
 
  6
 
 6847
 
 gene
 
 0.01561
 
 0.02659
 
 Not queried
 
  0.7684399
 
       NA
 
       NA
  
 
  
 1
 
 101491252
 
 101491652
 
  ENSEMBL   UCSC 
 
  401
 
 10
 
 3523
 
 island
 
 0.01611
 
 0.02383
 
 Not queried
 
  0.7683957
 
       NA
 
       NA
  
 
  
 2
 
 139259466
 
 139259770
 
  ENSEMBL   UCSC 
 
  305
 
  4
 
 22850
 
 gene;island
 
 0.01687
 
 0.02304
 
 Not queried
 
  0.7682501
 
       NA
 
       NA
  
 
  
 13
 
  99853132
 
  99853209
 
  ENSEMBL   UCSC 
 
   78
 
  4
 
 11176
 
 gene;island
 
 0.01498
 
 0.02498
 
 Not queried
 
  0.7682348
 
       NA
 
       NA
  
 
  
 12
 
  31744043
 
  31744436
 
  ENSEMBL   UCSC 
 
  394
 
  4
 
 9602
 
 tss
 
 0.04778
 
 0.03877
 
 Not queried
 
  0.7682319
 
       NA
 
       NA
  
 
  
 2
 
 119605210
 
 119605441
 
  ENSEMBL   UCSC 
 
  232
 
  4
 
 22805
 
 gene;island
 
 0.01217
 
 0.02068
 
 Not queried
 
  0.7680390
 
       NA
 
       NA
  
 
  
 4
 
 146100881
 
 146101151
 
  ENSEMBL   UCSC 
 
  271
 
  4
 
 30570
 
 tss
 
 0.01853
 
 0.02195
 
 Not queried
 
  0.7679755
 
       NA
 
       NA
  
 
  
 5
 
  40835490
 
  40835760
 
  ENSEMBL   UCSC 
 
  271
 
  7
 
 31977
 
 tss
 
 0.02676
 
 0.04494
 
 Not queried
 
  0.7679474
 
       NA
 
       NA
  
 
  
 15
 
  96873850
 
  96874052
 
  ENSEMBL   UCSC 
 
  203
 
  9
 
 13463
 
 gene;tss;island
 
 0.01427
 
 0.02460
 
 Not queried
 
  0.7679216
 
       NA
 
       NA
  
 
  
 1
 
   9258678
 
   9258836
 
  ENSEMBL   UCSC 
 
  159
 
  4
 
 2642
 
 island
 
 0.03811
 
 0.06549
 
 Not queried
 
  0.7678485
 
       NA
 
       NA
  
 
  
 2
 
  55921045
 
  55921125
 
  ENSEMBL   UCSC 
 
   81
 
  6
 
 23326
 
 tss
 
 0.00710
 
 0.01600
 
 Not queried
 
  0.7678363
 
       NA
 
       NA
  
 
  
 6
 
  27101633
 
  27101941
 
  ENSEMBL   UCSC 
 
  309
 
  4
 
 34648
 
 tss;island
 
 0.02585
 
 0.02517
 
 Not queried
 
  0.7678258
 
       NA
 
       NA
  
 
  
 5
 
  56468858
 
  56469322
 
  ENSEMBL   UCSC 
 
  465
 
  4
 
 32015
 
 tss;island
 
 0.03526
 
 0.03581
 
 Not queried
 
  0.7678118
 
       NA
 
       NA
  
 
  
 X
 
 107979988
 
 107980358
 
  ENSEMBL   UCSC 
 
  371
 
  5
 
 41467
 
 tss;island
 
 0.05546
 
 0.07587
 
 Not queried
 
  0.7677264
 
       NA
 
       NA
  
 
  
 7
 
 100728343
 
 100728792
 
  ENSEMBL   UCSC 
 
  450
 
  7
 
 38723
 
 island
 
 0.02032
 
 0.04923
 
 Not queried
 
  0.7676181
 
       NA
 
       NA
  
 
  
 1
 
  48459813
 
  48460182
 
  ENSEMBL   UCSC 
 
  370
 
  5
 
 3238
 
 island
 
 0.84024
 
 0.73157
 
 Not queried
 
  0.7675199
 
       NA
 
       NA
  
 
  
 19
 
  50269978
 
  50270115
 
  ENSEMBL   UCSC 
 
  138
 
  6
 
 20926
 
 tss;island
 
 0.03177
 
 0.05259
 
 Not queried
 
  0.7674904
 
       NA
 
       NA
  
 
  
 2
 
  37311651
 
  37311892
 
  ENSEMBL   UCSC 
 
  242
 
  4
 
 22609
 
 gene
 
 0.01159
 
 0.01568
 
 Not queried
 
  0.7674879
 
       NA
 
       NA
  
 
  
 19
 
  53696623
 
  53696886
 
  ENSEMBL   UCSC 
 
  264
 
  6
 
 20989
 
 tss
 
 0.03090
 
 0.04751
 
 Not queried
 
  0.7673838
 
       NA
 
       NA
  
 
  
 6
 
  33771799
 
  33772017
 
  ENSEMBL   UCSC 
 
  219
 
  4
 
 34850
 
 tss
 
 0.88372
 
 0.90577
 
 Not queried
 
  0.7672930
 
       NA
 
       NA
  
 
  
 11
 
  65150159
 
  65150416
 
  ENSEMBL   UCSC 
 
  258
 
  4
 
 7405
 
 tss
 
 0.70779
 
 0.74123
 
 Not queried
 
  0.7672630
 
       NA
 
       NA
  
 
  
 1
 
  94147461
 
  94147831
 
  ENSEMBL   UCSC 
 
  371
 
  4
 
 1756
 
 tss;island
 
 0.02955
 
 0.02087
 
 Not queried
 
  0.7672617
 
       NA
 
       NA
  
 
  
 15
 
  64387625
 
  64388147
 
  ENSEMBL   UCSC 
 
  523
 
  7
 
 13698
 
 tss
 
 0.02249
 
 0.03280
 
 Not queried
 
  0.7672390
 
       NA
 
       NA
  
 
  
 8
 
 145654007
 
 145654090
 
  ENSEMBL   UCSC 
 
   84
 
  5
 
 39919
 
 tss
 
 0.01481
 
 0.01992
 
 Not queried
 
  0.7670481
 
       NA
 
       NA
  
 
  
 22
 
  38863905
 
  38864244
 
  ENSEMBL   UCSC 
 
  340
 
  5
 
 27448
 
 island
 
 0.02318
 
 0.03881
 
 Not queried
 
  0.7669662
 
       NA
 
       NA
  
 
  
 11
 
  61322742
 
  61323413
 
  ENSEMBL   UCSC 
 
  672
 
  6
 
 6612
 
 gene;island
 
 0.09819
 
 0.10706
 
 Not queried
 
  0.7667309
 
       NA
 
       NA
  
 
  
 21
 
  45078342
 
  45078437
 
  ENSEMBL   UCSC 
 
   96
 
  4
 
 26404
 
 gene;tss;island
 
 0.97115
 
 0.94193
 
 Not queried
 
  0.7665550
 
       NA
 
       NA
  
 
  
 7
 
 108095719
 
 108096041
 
  ENSEMBL   UCSC 
 
  323
 
  5
 
 37122
 
 gene;island
 
 0.02191
 
 0.02351
 
 Not queried
 
  0.7664453
 
       NA
 
       NA
  
 
  
 7
 
  28452066
 
  28452131
 
  ENSEMBL   UCSC 
 
   66
 
  4
 
 37442
 
 tss
 
 0.31644
 
 0.16192
 
 Not queried
 
  0.7663906
 
       NA
 
       NA
  
 
  
 17
 
   6347867
 
   6348200
 
  ENSEMBL   UCSC 
 
  334
 
  6
 
 16336
 
 gene
 
 0.04609
 
 0.02639
 
 Not queried
 
  0.7662353
 
       NA
 
       NA
  
 
  
 1
 
 111218079
 
 111218287
 
  ENSEMBL   UCSC 
 
  209
 
  4
 
 1816
 
 tss;island
 
 0.16269
 
 0.09906
 
 Not queried
 
  0.7662193
 
       NA
 
       NA
  
 
  
 17
 
    900031
 
    900491
 
  ENSEMBL   UCSC 
 
  461
 
 11
 
 17771
 
 island
 
 0.01283
 
 0.02414
 
 Not queried
 
  0.7661290
 
       NA
 
       NA
  
 
  
 5
 
   1948875
 
   1949277
 
  ENSEMBL   UCSC 
 
  403
 
  5
 
 32587
 
 island
 
 0.66403
 
 0.76536
 
 Not queried
 
  0.7661037
 
       NA
 
       NA
  
 
  
 5
 
 169724590
 
 169724803
 
  ENSEMBL   UCSC 
 
  214
 
  5
 
 31830
 
 gene
 
 0.95341
 
 0.80457
 
 Not queried
 
  0.7660673
 
       NA
 
       NA
  
 
  
 11
 
  73309256
 
  73309466
 
  ENSEMBL   UCSC 
 
  211
 
  8
 
 7528
 
 tss;island
 
 0.01255
 
 0.02482
 
 Not queried
 
  0.7659934
 
       NA
 
       NA
  
 
  
 22
 
  18121127
 
  18121424
 
  ENSEMBL   UCSC 
 
  298
 
  7
 
 26904
 
 tss;island
 
 0.02698
 
 0.03000
 
 Not queried
 
  0.7659870
 
       NA
 
       NA
  
 
  
 2
 
  91847976
 
  91848380
 
  ENSEMBL   UCSC 
 
  405
 
  9
 
 23467
 
 tss
 
 0.07482
 
 0.26058
 
 Not queried
 
  0.7659469
 
       NA
 
       NA
  
 
  
 11
 
    843897
 
    844686
 
  ENSEMBL   UCSC 
 
  790
 
 11
 
 6374
 
 gene;island
 
 0.06163
 
 0.04357
 
 Not queried
 
  0.7658342
 
       NA
 
       NA
  
 
  
 18
 
    596703
 
    596943
 
  ENSEMBL   UCSC 
 
  241
 
  5
 
 19119
 
 tss
 
 0.03976
 
 0.04956
 
 Not queried
 
  0.7658305
 
       NA
 
       NA
  
 
  
 17
 
  62971592
 
  62971911
 
  ENSEMBL   UCSC 
 
  320
 
  8
 
 18634
 
 island
 
 0.01844
 
 0.02924
 
 Not queried
 
  0.7657031
 
       NA
 
       NA
  
 
  
 17
 
  80349836
 
  80350405
 
  ENSEMBL   UCSC 
 
  570
 
  8
 
 18956
 
 island
 
 0.86772
 
 0.89709
 
 Not queried
 
  0.7656662
 
       NA
 
       NA
  
 
  
 10
 
  21785561
 
  21785767
 
  ENSEMBL   UCSC 
 
  207
 
  7
 
 4514
 
 gene;island
 
 0.01922
 
 0.01795
 
 Not queried
 
  0.7655956
 
       NA
 
       NA
  
 
  
 13
 
 112320825
 
 112321270
 
  ENSEMBL   UCSC 
 
  446
 
  4
 
 11871
 
 island
 
 0.91421
 
 0.94647
 
 Not queried
 
  0.7655211
 
       NA
 
       NA
  
 
  
 7
 
 158622328
 
 158622612
 
  ENSEMBL   UCSC 
 
  285
 
  6
 
 37959
 
 tss;island
 
 0.01663
 
 0.02875
 
 Not queried
 
  0.7655086
 
       NA
 
       NA
  
 
  
 8
 
  72756812
 
  72757004
 
  ENSEMBL   UCSC 
 
  193
 
  7
 
 39699
 
 tss
 
 0.02467
 
 0.03578
 
 Not queried
 
  0.7654128
 
       NA
 
       NA
  
 
  
 2
 
  11750735
 
  11751071
 
  ENSEMBL   UCSC 
 
  337
 
  4
 
 22535
 
 gene;island
 
 0.84836
 
 0.92676
 
 Not queried
 
  0.7653621
 
       NA
 
       NA
  
 
  
 15
 
  30114814
 
  30114912
 
  ENSEMBL   UCSC 
 
   99
 
  4
 
 13518
 
 tss
 
 0.00659
 
 0.01418
 
 Not queried
 
  0.7653558
 
       NA
 
       NA
  
 
  
 8
 
 101118377
 
 101118548
 
  ENSEMBL   UCSC 
 
  172
 
  4
 
 39775
 
 tss;island
 
 0.81011
 
 0.87204
 
 Not queried
 
  0.7653470
 
       NA
 
       NA
  
 
  
 4
 
   1554380
 
   1554677
 
  ENSEMBL   UCSC 
 
  298
 
  5
 
 30742
 
 island
 
 0.77767
 
 0.87609
 
 Not queried
 
  0.7652203
 
       NA
 
       NA
  
 
  
 1
 
 205181033
 
 205181261
 
  ENSEMBL   UCSC 
 
  229
 
  4
 
 2198
 
 tss;island
 
 0.05201
 
 0.03268
 
 Not queried
 
  0.7650568
 
       NA
 
       NA
  
 
  
 2
 
 113808452
 
 113808755
 
  ENSEMBL   UCSC 
 
  304
 
  4
 
 22794
 
 gene;island
 
 0.79501
 
 0.61229
 
 Not queried
 
  0.7650098
 
       NA
 
       NA
  
 
  
 5
 
  42951935
 
  42952369
 
  ENSEMBL   UCSC 
 
  435
 
  4
 
 32700
 
 island
 
 0.95072
 
 0.87529
 
 Not queried
 
  0.7649871
 
       NA
 
       NA
  
 
  
 6
 
  31648514
 
  31649638
 
  ENSEMBL   UCSC 
 
 1125
 
 33
 
 34764
 
 tss
 
 0.04592
 
 0.03063
 
 Not queried
 
  0.7649140
 
       NA
 
       NA
  
 
  
 17
 
  45056626
 
  45056976
 
  ENSEMBL   UCSC 
 
  351
 
  8
 
 17405
 
 tss;island
 
 0.03950
 
 0.02161
 
 Not queried
 
  0.7648650
 
       NA
 
       NA
  
 
  
 19
 
  45663415
 
  45663527
 
  ENSEMBL   UCSC 
 
  113
 
  4
 
 20826
 
 tss
 
 0.74333
 
 0.65777
 
 Not queried
 
  0.7648253
 
       NA
 
       NA
  
 
  
 10
 
  70748213
 
  70748486
 
  ENSEMBL   UCSC 
 
  274
 
  9
 
 5103
 
 tss
 
 0.01722
 
 0.01833
 
 Not queried
 
  0.7647877
 
       NA
 
       NA
  
 
  
 7
 
  94538165
 
  94538408
 
  ENSEMBL   UCSC 
 
  244
 
  6
 
 37032
 
 gene;tss;island
 
 0.14910
 
 0.06733
 
 Not queried
 
  0.7646299
 
       NA
 
       NA
  
 
  
 X
 
 119378521
 
 119378908
 
  ENSEMBL   UCSC 
 
  388
 
  4
 
 41096
 
 gene;island
 
 0.01221
 
 0.02062
 
 Not queried
 
  0.7645968
 
       NA
 
       NA
  
 
  
 22
 
  41572924
 
  41573176
 
  ENSEMBL   UCSC 
 
  253
 
  6
 
 26839
 
 gene
 
 0.95861
 
 0.95354
 
 Not queried
 
  0.7645764
 
       NA
 
       NA
  
 
  
 12
 
   5603131
 
   5603261
 
  ENSEMBL   UCSC 
 
  131
 
  6
 
 8994
 
 gene;tss;island
 
 0.71933
 
 0.84904
 
 Not queried
 
  0.7645541
 
       NA
 
       NA
  
 
  
 13
 
 112212075
 
 112212266
 
  ENSEMBL   UCSC 
 
  192
 
  4
 
 11869
 
 island
 
 0.75094
 
 0.80314
 
 Not queried
 
  0.7644740
 
       NA
 
       NA
  
 
  
 X
 
  62780937
 
  62781086
 
  ENSEMBL   UCSC 
 
  150
 
  4
 
 41347
 
 tss
 
 0.02264
 
 0.01357
 
 Not queried
 
  0.7644341
 
       NA
 
       NA
  
 
  
 1
 
  10719929
 
  10720466
 
  ENSEMBL   UCSC 
 
  538
 
  5
 
 213
 
 gene;island
 
 0.87023
 
 0.88595
 
 Not queried
 
  0.7643074
 
       NA
 
       NA
  
 
  
 1
 
  15480555
 
  15480853
 
  ENSEMBL   UCSC 
 
  299
 
  4
 
 243
 
 gene;island
 
 0.03150
 
 0.05015
 
 Not queried
 
  0.7641978
 
       NA
 
       NA
  
 
  
 8
 
 145743334
 
 145743412
 
  ENSEMBL   UCSC 
 
   79
 
  4
 
 39925
 
 tss;island
 
 0.01654
 
 0.02467
 
 Not queried
 
  0.7641920
 
       NA
 
       NA
  
 
  
 17
 
  16120515
 
  16120890
 
  ENSEMBL   UCSC 
 
  376
 
  4
 
 16421
 
 gene
 
 0.00770
 
 0.01299
 
 Not queried
 
  0.7640869
 
       NA
 
       NA
  
 
  
 2
 
 179278332
 
 179278855
 
  ENSEMBL   UCSC 
 
  524
 
  6
 
 22908
 
 gene;island
 
 0.01642
 
 0.03365
 
 Not queried
 
  0.7640837
 
       NA
 
       NA
  
 
  
 10
 
  85899064
 
  85899175
 
  ENSEMBL   UCSC 
 
  112
 
  6
 
 5174
 
 tss
 
 0.01916
 
 0.02605
 
 Not queried
 
  0.7640364
 
       NA
 
       NA
  
 
  
 17
 
  16341984
 
  16342235
 
  ENSEMBL   UCSC 
 
  252
 
  8
 
 17125
 
 tss;island
 
 0.01154
 
 0.01942
 
 Not queried
 
  0.7640178
 
       NA
 
       NA
  
 
  
 2
 
 136289456
 
 136289752
 
  ENSEMBL   UCSC 
 
  297
 
  4
 
 22846
 
 gene;tss;island
 
 0.01854
 
 0.02857
 
 Not queried
 
  0.7639276
 
       NA
 
       NA
  
 
  
 3
 
  62360162
 
  62360464
 
  ENSEMBL   UCSC 
 
  303
 
  4
 
 28384
 
 tss;island
 
 0.23388
 
 0.13756
 
 Not queried
 
  0.7639264
 
       NA
 
       NA
  
 
  
 1
 
  16369395
 
  16369631
 
  ENSEMBL   UCSC 
 
  237
 
  4
 
 1277
 
 tss
 
 0.51356
 
 0.63465
 
 Not queried
 
  0.7638688
 
       NA
 
       NA
  
 
  
 16
 
   4384967
 
   4385435
 
  ENSEMBL   UCSC 
 
  469
 
  8
 
 14608
 
 gene
 
 0.71085
 
 0.85489
 
 Not queried
 
  0.7637534
 
       NA
 
       NA
  
 
  
 7
 
 157709849
 
 157710215
 
  ENSEMBL   UCSC 
 
  367
 
  4
 
 37291
 
 gene
 
 0.89282
 
 0.94718
 
 Not queried
 
  0.7637179
 
       NA
 
       NA
  
 
  
 1
 
  50489654
 
  50489954
 
  ENSEMBL   UCSC 
 
  301
 
  8
 
 1610
 
 tss
 
 0.01963
 
 0.03147
 
 Not queried
 
  0.7636952
 
       NA
 
       NA
  
 
  
 17
 
  41003236
 
  41003399
 
  ENSEMBL   UCSC 
 
  164
 
  4
 
 16554
 
 gene
 
 0.61808
 
 0.68775
 
 Not queried
 
  0.7636814
 
       NA
 
       NA
  
 
  
 5
 
 128300967
 
 128301488
 
  ENSEMBL   UCSC 
 
  522
 
 10
 
 33010
 
 island
 
 0.02284
 
 0.03672
 
 Not queried
 
  0.7636036
 
       NA
 
       NA
  
 
  
 15
 
  99193929
 
  99194172
 
  ENSEMBL   UCSC 
 
  244
 
  4
 
 13466
 
 gene;island
 
 0.01527
 
 0.03017
 
 Not queried
 
  0.7634039
 
       NA
 
       NA
  
 
  
 12
 
  98987143
 
  98987358
 
  ENSEMBL   UCSC 
 
  216
 
  6
 
 9910
 
 tss
 
 0.01903
 
 0.02295
 
 Not queried
 
  0.7633911
 
       NA
 
       NA
  
 
  
 1
 
  41827960
 
  41828276
 
  ENSEMBL   UCSC 
 
  317
 
  4
 
 3131
 
 island
 
 0.26660
 
 0.10033
 
 Not queried
 
  0.7633765
 
       NA
 
       NA
  
 
  
 10
 
   3145966
 
   3146205
 
  ENSEMBL   UCSC 
 
  240
 
  4
 
 4458
 
 gene;island
 
 0.90332
 
 0.88304
 
 Not queried
 
  0.7631754
 
       NA
 
       NA
  
 
  
 1
 
  36023025
 
  36023927
 
  ENSEMBL   UCSC 
 
  903
 
 12
 
 1481
 
 tss;island
 
 0.03014
 
 0.02663
 
 Not queried
 
  0.7631502
 
       NA
 
       NA
  
 
  
 17
 
  73775869
 
  73776120
 
  ENSEMBL   UCSC 
 
  252
 
  9
 
 17628
 
 tss
 
 0.01296
 
 0.02463
 
 Not queried
 
  0.7631389
 
       NA
 
       NA
  
 
  
 11
 
  43380251
 
  43380484
 
  ENSEMBL   UCSC 
 
  234
 
  7
 
 7232
 
 tss
 
 0.02348
 
 0.02097
 
 Not queried
 
  0.7630196
 
       NA
 
       NA
  
 
  
 4
 
   3027700
 
   3027983
 
  ENSEMBL   UCSC 
 
  284
 
  5
 
 29855
 
 gene
 
 0.93388
 
 0.91545
 
 Not queried
 
  0.7629140
 
       NA
 
       NA
  
 
  
 22
 
  43539525
 
  43539658
 
  ENSEMBL   UCSC 
 
  134
 
  6
 
 27159
 
 tss;island
 
 0.04384
 
 0.05199
 
 Not queried
 
  0.7629110
 
       NA
 
       NA
  
 
  
 11
 
  69456412
 
  69457121
 
  ENSEMBL   UCSC 
 
  710
 
  6
 
 6762
 
 gene;island
 
 0.02274
 
 0.02138
 
 Not queried
 
  0.7627944
 
       NA
 
       NA
  
 
  
 5
 
 115152413
 
 115152494
 
  ENSEMBL   UCSC 
 
   82
 
  6
 
 32171
 
 tss
 
 0.01773
 
 0.03470
 
 Not queried
 
  0.7625961
 
       NA
 
       NA
  
 
  
 5
 
 115152326
 
 115152494
 
  ENSEMBL   UCSC 
 
  169
 
  8
 
 32965
 
 island
 
 0.02300
 
 0.04317
 
 Not queried
 
  0.7625961
 
       NA
 
       NA
  
 
  
 14
 
  64931743
 
  64932123
 
  ENSEMBL   UCSC 
 
  381
 
  7
 
 12427
 
 tss;island
 
 0.01775
 
 0.02554
 
 Not queried
 
  0.7625815
 
       NA
 
       NA
  
 
  
 14
 
  24658124
 
  24658292
 
  ENSEMBL   UCSC 
 
  169
 
  7
 
 12291
 
 tss
 
 0.01679
 
 0.01732
 
 Not queried
 
  0.7624678
 
       NA
 
       NA
  
 
  
 15
 
  90118856
 
  90119427
 
  ENSEMBL   UCSC 
 
  572
 
  6
 
 13442
 
 gene
 
 0.01682
 
 0.03122
 
 Not queried
 
  0.7624058
 
       NA
 
       NA
  
 
  
 3
 
 193720874
 
 193721215
 
  ENSEMBL   UCSC 
 
  342
 
  4
 
 29735
 
 island
 
 0.02823
 
 0.04126
 
 Not queried
 
  0.7623146
 
       NA
 
       NA
  
 
  
 7
 
  45115588
 
  45115954
 
  ENSEMBL   UCSC 
 
  367
 
  4
 
 36935
 
 gene
 
 0.73941
 
 0.78815
 
 Not queried
 
  0.7623135
 
       NA
 
       NA
  
 
  
 2
 
  27341795
 
  27341901
 
  ENSEMBL   UCSC 
 
  107
 
  4
 
 22583
 
 gene;tss;island
 
 0.04411
 
 0.08985
 
 Not queried
 
  0.7622805
 
       NA
 
       NA
  
 
  
 8
 
  75227148
 
  75227355
 
  ENSEMBL   UCSC 
 
  208
 
  4
 
 39275
 
 gene;island
 
 0.79281
 
 0.91441
 
 Not queried
 
  0.7622803
 
       NA
 
       NA
  
 
  
 13
 
 113489734
 
 113490012
 
  ENSEMBL   UCSC 
 
  279
 
  4
 
 11229
 
 gene;island
 
 0.84571
 
 0.86689
 
 Not queried
 
  0.7621384
 
       NA
 
       NA
  
 
  
 X
 
 135730236
 
 135730306
 
  ENSEMBL   UCSC 
 
   71
 
  4
 
 41551
 
 tss
 
 0.89528
 
 0.93413
 
 Not queried
 
  0.7620725
 
       NA
 
       NA
  
 
  
 3
 
 125690986
 
 125691192
 
  ENSEMBL   UCSC 
 
  207
 
  4
 
 27885
 
 gene;island
 
 0.07942
 
 0.07921
 
 Not queried
 
  0.7620471
 
       NA
 
       NA
  
 
  
 6
 
  32022898
 
  32023650
 
  ENSEMBL   UCSC 
 
  753
 
 13
 
 33958
 
 gene
 
 0.83112
 
 0.72089
 
 Not queried
 
  0.7619052
 
       NA
 
       NA
  
 
  
 19
 
   2578938
 
   2579285
 
  ENSEMBL   UCSC 
 
  348
 
  4
 
 19659
 
 gene;island
 
 0.04076
 
 0.07359
 
 Not queried
 
  0.7619045
 
       NA
 
       NA
  
 
  
 1
 
 156889826
 
 156890244
 
  ENSEMBL   UCSC 
 
  419
 
  5
 
 2022
 
 tss;island
 
 0.03665
 
 0.11633
 
 Not queried
 
  0.7617082
 
       NA
 
       NA
  
 
  
 2
 
 118572045
 
 118572392
 
  ENSEMBL   UCSC 
 
  348
 
  9
 
 24599
 
 island
 
 0.01061
 
 0.01998
 
 Not queried
 
  0.7616155
 
       NA
 
       NA
  
 
  
 7
 
   1460673
 
   1460944
 
  ENSEMBL   UCSC 
 
  272
 
  4
 
 38040
 
 island
 
 0.83430
 
 0.91151
 
 Not queried
 
  0.7616001
 
       NA
 
       NA
  
 
  
 8
 
  11561283
 
  11561728
 
  ENSEMBL   UCSC 
 
  446
 
 11
 
 40023
 
 island
 
 0.16200
 
 0.09828
 
 Not queried
 
  0.7614376
 
       NA
 
       NA
  
 
  
 6
 
 167032331
 
 167032502
 
  ENSEMBL   UCSC 
 
  172
 
  4
 
 34485
 
 gene;island
 
 0.94054
 
 0.89355
 
 Not queried
 
  0.7613755
 
       NA
 
       NA
  
 
  
 1
 
  39457099
 
  39457270
 
  ENSEMBL   UCSC 
 
  172
 
  6
 
 443
 
 gene;island
 
 0.02950
 
 0.05049
 
 Not queried
 
  0.7612725
 
       NA
 
       NA
  
 
  
 15
 
  59397042
 
  59397300
 
  ENSEMBL   UCSC 
 
  259
 
  6
 
 13676
 
 tss;island
 
 0.02863
 
 0.03062
 
 Not queried
 
  0.7611464
 
       NA
 
       NA
  
 
  
 1
 
 160312929
 
 160313057
 
  ENSEMBL   UCSC 
 
  129
 
  5
 
 2034
 
 tss
 
 0.01641
 
 0.01827
 
 Not queried
 
  0.7611186
 
       NA
 
       NA
  
 
  
 17
 
   9694619
 
   9695137
 
  ENSEMBL   UCSC 
 
  519
 
  6
 
 17097
 
 tss
 
 0.86744
 
 0.71413
 
 Not queried
 
  0.7610375
 
       NA
 
       NA
  
 
  
 1
 
 109419416
 
 109419606
 
  ENSEMBL   UCSC 
 
  191
 
  4
 
 3539
 
 island
 
 0.02205
 
 0.04781
 
 Not queried
 
  0.7610085
 
       NA
 
       NA
  
 
  
 22
 
  43570062
 
  43570418
 
  ENSEMBL   UCSC 
 
  357
 
  4
 
 26847
 
 gene;island
 
 0.90696
 
 0.86351
 
 Not queried
 
  0.7608628
 
       NA
 
       NA
  
 
  
 2
 
  20251043
 
  20251361
 
  ENSEMBL   UCSC 
 
  319
 
  5
 
 22551
 
 gene;island
 
 0.03961
 
 0.03799
 
 Not queried
 
  0.7607822
 
       NA
 
       NA
  
 
  
 19
 
  36024217
 
  36024282
 
  ENSEMBL   UCSC 
 
   66
 
  5
 
 20670
 
 tss
 
 0.96913
 
 0.94416
 
 Not queried
 
  0.7607174
 
       NA
 
       NA
  
 
  
 12
 
 117348446
 
 117348663
 
  ENSEMBL   UCSC 
 
  218
 
  5
 
 10017
 
 tss
 
 0.03257
 
 0.04064
 
 Not queried
 
  0.7606699
 
       NA
 
       NA
  
 
  
 7
 
   1882997
 
   1883420
 
  ENSEMBL   UCSC 
 
  424
 
  6
 
 36705
 
 gene;island
 
 0.89536
 
 0.91851
 
 Not queried
 
  0.7606662
 
       NA
 
       NA
  
 
  
 17
 
  61959271
 
  61959568
 
  ENSEMBL   UCSC 
 
  298
 
  5
 
 17542
 
 tss
 
 0.62861
 
 0.49975
 
 Not queried
 
  0.7606467
 
       NA
 
       NA
  
 
  
 4
 
    903032
 
    903575
 
  ENSEMBL   UCSC 
 
  544
 
  6
 
 29802
 
 gene;island
 
 0.86291
 
 0.93501
 
 Not queried
 
  0.7606457
 
       NA
 
       NA
  
 
  
 15
 
  66789796
 
  66790095
 
  ENSEMBL   UCSC 
 
  300
 
  5
 
 13359
 
 gene
 
 0.02471
 
 0.03570
 
 Not queried
 
  0.7605130
 
       NA
 
       NA
  
 
  
 15
 
  66789796
 
  66790410
 
  ENSEMBL   UCSC 
 
  615
 
  9
 
 14216
 
 island
 
 0.02922
 
 0.05552
 
 Not queried
 
  0.7605130
 
       NA
 
       NA
  
 
  
 7
 
   5938193
 
   5938293
 
  ENSEMBL   UCSC 
 
  101
 
  6
 
 37363
 
 tss;island
 
 0.01635
 
 0.01339
 
 Not queried
 
  0.7604505
 
       NA
 
       NA
  
 
  
 8
 
  71581111
 
  71581445
 
  ENSEMBL   UCSC 
 
  335
 
  4
 
 39270
 
 gene
 
 0.01536
 
 0.02573
 
 Not queried
 
  0.7604155
 
       NA
 
       NA
  
 
  
 17
 
  26926457
 
  26926868
 
  ENSEMBL   UCSC 
 
  412
 
  6
 
 17181
 
 tss;island
 
 0.03958
 
 0.08137
 
 Not queried
 
  0.7602238
 
       NA
 
       NA
  
 
  
 13
 
  22244956
 
  22245730
 
  ENSEMBL   UCSC 
 
  775
 
 13
 
 11564
 
 island
 
 0.01529
 
 0.03482
 
 Not queried
 
  0.7601949
 
       NA
 
       NA
  
 
  
 11
 
   1501640
 
   1502195
 
  ENSEMBL   UCSC 
 
  556
 
  5
 
 6404
 
 gene
 
 0.82721
 
 0.88285
 
 Not queried
 
  0.7601838
 
       NA
 
       NA
  
 
  
 15
 
  26327718
 
  26328092
 
  ENSEMBL   UCSC 
 
  375
 
  4
 
 13934
 
 island
 
 0.03994
 
 0.02877
 
 Not queried
 
  0.7601211
 
       NA
 
       NA
  
 
  
 8
 
  17433926
 
  17434541
 
  ENSEMBL   UCSC 
 
  616
 
  9
 
 39504
 
 tss;island
 
 0.02050
 
 0.02403
 
 Not queried
 
  0.7601200
 
       NA
 
       NA
  
 
  
 3
 
 125635174
 
 125635399
 
  ENSEMBL   UCSC 
 
  226
 
  5
 
 28511
 
 tss;island
 
 0.03256
 
 0.05385
 
 Not queried
 
  0.7601128
 
       NA
 
       NA
  
 
  
 15
 
  43594466
 
  43594820
 
  ENSEMBL   UCSC 
 
  355
 
  5
 
 13597
 
 tss
 
 0.94176
 
 0.93507
 
 Not queried
 
  0.7598988
 
       NA
 
       NA
  
 
  
 2
 
 109403293
 
 109403692
 
  ENSEMBL   UCSC 
 
  400
 
  5
 
 22783
 
 gene
 
 0.02547
 
 0.02881
 
 Not queried
 
  0.7598915
 
       NA
 
       NA
  
 
  
 12
 
  89744471
 
  89744877
 
  ENSEMBL   UCSC 
 
  407
 
  8
 
 9213
 
 gene;island
 
 0.02519
 
 0.02995
 
 Not queried
 
  0.7597615
 
       NA
 
       NA
  
 
  
 20
 
  50769044
 
  50769317
 
  ENSEMBL   UCSC 
 
  274
 
  4
 
 25418
 
 gene;island
 
 0.84455
 
 0.85677
 
 Not queried
 
  0.7596951
 
       NA
 
       NA
  
 
  
 7
 
  96642510
 
  96642794
 
  ENSEMBL   UCSC 
 
  285
 
  4
 
 37041
 
 gene;island
 
 0.11037
 
 0.20551
 
 Not queried
 
  0.7594740
 
       NA
 
       NA
  
 
  
 14
 
  38080048
 
  38080446
 
  ENSEMBL   UCSC 
 
  399
 
  4
 
 12771
 
 island
 
 0.41114
 
 0.53799
 
 Not queried
 
  0.7592397
 
       NA
 
       NA
  
 
  
 2
 
 170655263
 
 170655474
 
  ENSEMBL   UCSC 
 
  212
 
  7
 
 24804
 
 island
 
 0.02412
 
 0.03724
 
 Not queried
 
  0.7592325
 
       NA
 
       NA
  
 
  
 17
 
   7835172
 
   7835277
 
  ENSEMBL   UCSC 
 
  106
 
  5
 
 16386
 
 gene
 
 0.01155
 
 0.01378
 
 Not queried
 
  0.7591148
 
       NA
 
       NA
  
 
  
 1
 
  29557230
 
  29557446
 
  ENSEMBL   UCSC 
 
  217
 
  4
 
 374
 
 gene
 
 0.01571
 
 0.02599
 
 Not queried
 
  0.7589156
 
       NA
 
       NA
  
 
  
 19
 
  12035674
 
  12036118
 
  ENSEMBL   UCSC 
 
  445
 
  8
 
 21551
 
 island
 
 0.01722
 
 0.06221
 
 Not queried
 
  0.7589139
 
       NA
 
       NA
  
 
  
 1
 
  17380506
 
  17380646
 
  ENSEMBL   UCSC 
 
  141
 
  5
 
 264
 
 gene
 
 0.01292
 
 0.00911
 
 Not queried
 
  0.7587450
 
       NA
 
       NA
  
 
  
 16
 
  50613289
 
  50613700
 
  ENSEMBL   UCSC 
 
  412
 
  4
 
 14716
 
 gene;island
 
 0.94862
 
 0.91589
 
 Not queried
 
  0.7587349
 
       NA
 
       NA
  
 
  
 17
 
  70116185
 
  70116434
 
  ENSEMBL   UCSC 
 
  250
 
  4
 
 17579
 
 tss;island
 
 0.04261
 
 0.07013
 
 Not queried
 
  0.7586129
 
       NA
 
       NA
  
 
  
 3
 
 184045017
 
 184045221
 
  ENSEMBL   UCSC 
 
  205
 
  4
 
 28019
 
 gene;island
 
 0.93182
 
 0.93457
 
 Not queried
 
  0.7585234
 
       NA
 
       NA
  
 
  
 4
 
   1340807
 
   1341064
 
  ENSEMBL   UCSC 
 
  258
 
 13
 
 30196
 
 tss;island
 
 0.02342
 
 0.02670
 
 Not queried
 
  0.7584727
 
       NA
 
       NA
  
 
  
 1
 
 244998656
 
 244998909
 
  ENSEMBL   UCSC 
 
  254
 
  6
 
 1111
 
 gene
 
 0.02146
 
 0.03834
 
 Not queried
 
  0.7584617
 
       NA
 
       NA
  
 
  
 1
 
 244998532
 
 244998909
 
  ENSEMBL   UCSC 
 
  378
 
  8
 
 4384
 
 island
 
 0.02203
 
 0.03834
 
 Not queried
 
  0.7584617
 
       NA
 
       NA
  
 
  
 3
 
   9004664
 
   9005113
 
  ENSEMBL   UCSC 
 
  450
 
  4
 
 27606
 
 gene
 
 0.01439
 
 0.02286
 
 Not queried
 
  0.7584253
 
       NA
 
       NA
  
 
  
 11
 
  77122839
 
  77123047
 
  ENSEMBL   UCSC 
 
  209
 
  4
 
 6822
 
 gene;island
 
 0.05273
 
 0.03412
 
 Not queried
 
  0.7584080
 
       NA
 
       NA
  
 
  
 4
 
 157996959
 
 157997880
 
  ENSEMBL   UCSC 
 
  922
 
 15
 
 31340
 
 island
 
 0.03367
 
 0.05156
 
 Not queried
 
  0.7583237
 
       NA
 
       NA
  
 
  
 10
 
 102419209
 
 102419617
 
  ENSEMBL   UCSC 
 
  409
 
  6
 
 6010
 
 island
 
 0.59605
 
 0.71362
 
 Not queried
 
  0.7582926
 
       NA
 
       NA
  
 
  
 17
 
  18167551
 
  18167836
 
  ENSEMBL   UCSC 
 
  286
 
  4
 
 16438
 
 gene;island
 
 0.87367
 
 0.78307
 
 Not queried
 
  0.7582877
 
       NA
 
       NA
  
 
  
 15
 
  90893182
 
  90893535
 
  ENSEMBL   UCSC 
 
  354
 
  4
 
 13872
 
 tss;island
 
 0.90852
 
 0.83382
 
 Not queried
 
  0.7581730
 
       NA
 
       NA
  
 
  
 12
 
 116996773
 
 116997095
 
  ENSEMBL   UCSC 
 
  323
 
  8
 
 10013
 
 tss
 
 0.76858
 
 0.85162
 
 Not queried
 
  0.7580497
 
       NA
 
       NA
  
 
  
 8
 
 145823277
 
 145823653
 
  ENSEMBL   UCSC 
 
  377
 
  5
 
 39466
 
 gene;island
 
 0.76195
 
 0.88017
 
 Not queried
 
  0.7579793
 
       NA
 
       NA
  
 
  
 6
 
  31832173
 
  31833452
 
  ENSEMBL   UCSC 
 
 1280
 
 25
 
 33913
 
 gene;island
 
 0.67038
 
 0.85893
 
 Not queried
 
  0.7578389
 
       NA
 
       NA
  
 
  
 15
 
  42873451
 
  42873882
 
  ENSEMBL   UCSC 
 
  432
 
  5
 
 13283
 
 gene;island
 
 0.02925
 
 0.02140
 
 Not queried
 
  0.7577263
 
       NA
 
       NA
  
 
  
 7
 
   4275634
 
   4275962
 
  ENSEMBL   UCSC 
 
  329
 
  4
 
 36774
 
 gene
 
 0.78870
 
 0.89323
 
 Not queried
 
  0.7576906
 
       NA
 
       NA
  
 
  
 11
 
   2182457
 
   2182795
 
  ENSEMBL   UCSC 
 
  339
 
  8
 
 7041
 
 tss
 
 0.59843
 
 0.74504
 
 Not queried
 
  0.7576188
 
       NA
 
       NA
  
 
  
 19
 
  47290586
 
  47290716
 
  ENSEMBL   UCSC 
 
  131
 
  4
 
 20862
 
 tss
 
 0.04908
 
 0.03192
 
 Not queried
 
  0.7574435
 
       NA
 
       NA
  
 
  
 17
 
  16120399
 
  16120890
 
  ENSEMBL   UCSC 
 
  492
 
  9
 
 18015
 
 island
 
 0.02027
 
 0.01668
 
 Not queried
 
  0.7574394
 
       NA
 
       NA
  
 
  
 14
 
  93260599
 
  93260913
 
  ENSEMBL   UCSC 
 
  315
 
  4
 
 13063
 
 island
 
 0.03026
 
 0.02090
 
 Not queried
 
  0.7573760
 
       NA
 
       NA
  
 
  
 X
 
 109662113
 
 109662303
 
  ENSEMBL   UCSC 
 
  191
 
  6
 
 41079
 
 gene
 
 0.06774
 
 0.02542
 
 Not queried
 
  0.7573273
 
       NA
 
       NA
  
 
  
 16
 
  57278643
 
  57279026
 
  ENSEMBL   UCSC 
 
  384
 
  8
 
 15228
 
 tss;island
 
 0.02344
 
 0.02136
 
 Not queried
 
  0.7571093
 
       NA
 
       NA
  
 
  
 4
 
  78783538
 
  78784263
 
  ENSEMBL   UCSC 
 
  726
 
  8
 
 31071
 
 island
 
 0.00950
 
 0.01544
 
 Not queried
 
  0.7570212
 
       NA
 
       NA
  
 
  
 6
 
  31238751
 
  31239411
 
  ENSEMBL   UCSC 
 
  661
 
 19
 
 33803
 
 gene;island
 
 0.28511
 
 0.19010
 
 Not queried
 
  0.7569905
 
       NA
 
       NA
  
 
  
 1
 
 205424972
 
 205425241
 
  ENSEMBL   UCSC 
 
  270
 
  6
 
 4114
 
 island
 
 0.02222
 
 0.04896
 
 Not queried
 
  0.7569679
 
       NA
 
       NA
  
 
  
 1
 
 113249942
 
 113250120
 
  ENSEMBL   UCSC 
 
  179
 
  6
 
 1835
 
 tss;island
 
 0.04021
 
 0.06189
 
 Not queried
 
  0.7569567
 
       NA
 
       NA
  
 
  
 11
 
  45868770
 
  45869049
 
  ENSEMBL   UCSC 
 
  280
 
  9
 
 6578
 
 gene;island
 
 0.02492
 
 0.02877
 
 Not queried
 
  0.7569009
 
       NA
 
       NA
  
 
  
 2
 
  89065319
 
  89065605
 
  ENSEMBL   UCSC 
 
  287
 
  7
 
 24469
 
 island
 
 0.11255
 
 0.48148
 
 Not queried
 
  0.7567577
 
       NA
 
       NA
  
 
  
 11
 
  74109510
 
  74110223
 
  ENSEMBL   UCSC 
 
  714
 
 10
 
 7536
 
 tss;island
 
 0.01350
 
 0.02347
 
 Not queried
 
  0.7567507
 
       NA
 
       NA
  
 
  
 11
 
  61522741
 
  61522807
 
  ENSEMBL   UCSC 
 
   67
 
  5
 
 6614
 
 gene;tss;island
 
 0.86384
 
 0.84412
 
 Not queried
 
  0.7566463
 
       NA
 
       NA
  
 
  
 4
 
 129209001
 
 129209112
 
  ENSEMBL   UCSC 
 
  112
 
  4
 
 30542
 
 tss
 
 0.07624
 
 0.10784
 
 Not queried
 
  0.7563531
 
       NA
 
       NA
  
 
  
 19
 
  17137685
 
  17137714
 
  ENSEMBL   UCSC 
 
   30
 
  4
 
 20542
 
 tss
 
 0.04264
 
 0.05992
 
 Not queried
 
  0.7563009
 
       NA
 
       NA
  
 
  
 3
 
 179041226
 
 179041494
 
  ENSEMBL   UCSC 
 
  269
 
  8
 
 28688
 
 tss
 
 0.04596
 
 0.07092
 
 Not queried
 
  0.7562898
 
       NA
 
       NA
  
 
  
 18
 
  47825004
 
  47825363
 
  ENSEMBL   UCSC 
 
  360
 
  5
 
 19476
 
 island
 
 0.63032
 
 0.72785
 
 Not queried
 
  0.7560625
 
       NA
 
       NA
  
 
  
 11
 
  32112091
 
  32112810
 
  ENSEMBL   UCSC 
 
  720
 
  8
 
 8152
 
 island
 
 0.11276
 
 0.06677
 
 Not queried
 
  0.7560147
 
       NA
 
       NA
  
 
  
 1
 
 206223453
 
 206223719
 
  ENSEMBL   UCSC 
 
  267
 
  5
 
 2207
 
 tss;island
 
 0.08848
 
 0.14085
 
 Not queried
 
  0.7559941
 
       NA
 
       NA
  
 
  
 1
 
  40626599
 
  40626967
 
  ENSEMBL   UCSC 
 
  369
 
  6
 
 1530
 
 tss
 
 0.02790
 
 0.02345
 
 Not queried
 
  0.7558359
 
       NA
 
       NA
  
 
  
 6
 
  36410747
 
  36411216
 
  ENSEMBL   UCSC 
 
  470
 
  6
 
 34874
 
 tss
 
 0.01432
 
 0.02436
 
 Not queried
 
  0.7558116
 
       NA
 
       NA
  
 
  
 8
 
  95653174
 
  95653325
 
  ENSEMBL   UCSC 
 
  152
 
  4
 
 39754
 
 tss
 
 0.04642
 
 0.08221
 
 Not queried
 
  0.7556880
 
       NA
 
       NA
  
 
  
 5
 
  40835193
 
  40835760
 
  ENSEMBL   UCSC 
 
  568
 
 11
 
 32690
 
 island
 
 0.02319
 
 0.03129
 
 Not queried
 
  0.7556475
 
       NA
 
       NA
  
 
  
 19
 
  12624466
 
  12624832
 
  ENSEMBL   UCSC 
 
  367
 
  8
 
 21568
 
 island
 
 0.88394
 
 0.93711
 
 Not queried
 
  0.7554652
 
       NA
 
       NA
  
 
  
 12
 
   7342464
 
   7343020
 
  ENSEMBL   UCSC 
 
  557
 
  9
 
 9023
 
 gene;island
 
 0.02710
 
 0.03143
 
 Not queried
 
  0.7554602
 
       NA
 
       NA
  
 
  
 12
 
 121570677
 
 121571068
 
  ENSEMBL   UCSC 
 
  392
 
  4
 
 9336
 
 gene
 
 0.53629
 
 0.41687
 
 Not queried
 
  0.7554024
 
       NA
 
       NA
  
 
  
 12
 
  12715438
 
  12715811
 
  ENSEMBL   UCSC 
 
  374
 
  5
 
 9546
 
 tss;island
 
 0.01617
 
 0.02699
 
 Not queried
 
  0.7552911
 
       NA
 
       NA
  
 
  
 1
 
 226829496
 
 226829776
 
  ENSEMBL   UCSC 
 
  281
 
  5
 
 1033
 
 gene;island
 
 0.87617
 
 0.82123
 
 Not queried
 
  0.7552570
 
       NA
 
       NA
  
 
  
 3
 
 178969440
 
 178969733
 
  ENSEMBL   UCSC 
 
  294
 
  5
 
 28685
 
 tss
 
 0.91299
 
 0.86126
 
 Not queried
 
  0.7551070
 
       NA
 
       NA
  
 
  
 17
 
  37607610
 
  37607663
 
  ENSEMBL   UCSC 
 
   54
 
  4
 
 17275
 
 tss
 
 0.01138
 
 0.01562
 
 Not queried
 
  0.7549018
 
       NA
 
       NA
  
 
  
 17
 
  48450457
 
  48450912
 
  ENSEMBL   UCSC 
 
  456
 
 12
 
 16650
 
 gene;tss;island
 
 0.04254
 
 0.02285
 
 Not queried
 
  0.7547856
 
       NA
 
       NA
  
 
  
 10
 
 118547556
 
 118547891
 
  ENSEMBL   UCSC 
 
  336
 
  5
 
 6148
 
 island
 
 0.04422
 
 0.06822
 
 Not queried
 
  0.7547189
 
       NA
 
       NA
  
 
  
 1
 
 153650884
 
 153651147
 
  ENSEMBL   UCSC 
 
  264
 
  7
 
 1946
 
 tss
 
 0.04829
 
 0.06088
 
 Not queried
 
  0.7547076
 
       NA
 
       NA
  
 
  
 7
 
  76009240
 
  76009472
 
  ENSEMBL   UCSC 
 
  233
 
  4
 
 38539
 
 island
 
 0.02112
 
 0.03513
 
 Not queried
 
  0.7546485
 
       NA
 
       NA
  
 
  
 1
 
  92764174
 
  92764833
 
  ENSEMBL   UCSC 
 
  660
 
 12
 
 633
 
 gene;island
 
 0.01189
 
 0.02384
 
 Not queried
 
  0.7544907
 
       NA
 
       NA
  
 
  
 7
 
  94138743
 
  94138947
 
  ENSEMBL   UCSC 
 
  205
 
  4
 
 37654
 
 tss;island
 
 0.10463
 
 0.05520
 
 Not queried
 
  0.7543843
 
       NA
 
       NA
  
 
  
 1
 
 156646293
 
 156646746
 
  ENSEMBL   UCSC 
 
  454
 
  5
 
 825
 
 gene;island
 
 0.03978
 
 0.06531
 
 Not queried
 
  0.7543639
 
       NA
 
       NA
  
 
  
 X
 
  47489285
 
  47489663
 
  ENSEMBL   UCSC 
 
  379
 
  4
 
 40980
 
 gene
 
 0.55686
 
 0.68237
 
 Not queried
 
  0.7543497
 
       NA
 
       NA
  
 
  
 3
 
 112013079
 
 112013357
 
  ENSEMBL   UCSC 
 
  279
 
  4
 
 28458
 
 tss
 
 0.75255
 
 0.88018
 
 Not queried
 
  0.7542571
 
       NA
 
       NA
  
 
  
 6
 
 166139905
 
 166140706
 
  ENSEMBL   UCSC 
 
  802
 
  8
 
 36559
 
 island
 
 0.82144
 
 0.92589
 
 Not queried
 
  0.7542093
 
       NA
 
       NA
  
 
  
 1
 
 244080781
 
 244081189
 
  ENSEMBL   UCSC 
 
  409
 
  6
 
 4377
 
 island
 
 0.01750
 
 0.03359
 
 Not queried
 
  0.7541259
 
       NA
 
       NA
  
 
  
 6
 
  74363449
 
  74363913
 
  ENSEMBL   UCSC 
 
  465
 
  9
 
 36199
 
 island
 
 0.02295
 
 0.03509
 
 Not queried
 
  0.7539593
 
       NA
 
       NA
  
 
  
 4
 
  37688031
 
  37688612
 
  ENSEMBL   UCSC 
 
  582
 
  8
 
 30295
 
 tss;island
 
 0.03449
 
 0.02583
 
 Not queried
 
  0.7538993
 
       NA
 
       NA
  
 
  
 11
 
  36615733
 
  36616365
 
  ENSEMBL   UCSC 
 
  633
 
  9
 
 6568
 
 gene
 
 0.02553
 
 0.03828
 
 Not queried
 
  0.7538875
 
       NA
 
       NA
  
 
  
 20
 
   3220565
 
   3220985
 
  ENSEMBL   UCSC 
 
  421
 
  5
 
 25929
 
 island
 
 0.04463
 
 0.05206
 
 Not queried
 
  0.7538704
 
       NA
 
       NA
  
 
  
 1
 
 179846986
 
 179847329
 
  ENSEMBL   UCSC 
 
  344
 
  7
 
 2117
 
 tss
 
 0.02924
 
 0.02518
 
 Not queried
 
  0.7538321
 
       NA
 
       NA
  
 
  
 1
 
  23884703
 
  23885086
 
  ENSEMBL   UCSC 
 
  384
 
  4
 
 315
 
 gene;island
 
 0.04075
 
 0.05318
 
 Not queried
 
  0.7538201
 
       NA
 
       NA
  
 
  
 14
 
  96730482
 
  96730780
 
  ENSEMBL   UCSC 
 
  299
 
  4
 
 12171
 
 gene
 
 0.75928
 
 0.85306
 
 Not queried
 
  0.7538031
 
       NA
 
       NA
  
 
  
 12
 
  58087331
 
  58087721
 
  ENSEMBL   UCSC 
 
  391
 
  8
 
 9799
 
 tss
 
 0.05077
 
 0.04288
 
 Not queried
 
  0.7537738
 
       NA
 
       NA
  
 
  
 1
 
 203055422
 
 203055662
 
  ENSEMBL   UCSC 
 
  241
 
  4
 
 2185
 
 tss
 
 0.65831
 
 0.75577
 
 Not queried
 
  0.7537303
 
       NA
 
       NA
  
 
  
 12
 
  40499811
 
  40500139
 
  ENSEMBL   UCSC 
 
  329
 
  4
 
 9614
 
 tss
 
 0.04168
 
 0.01866
 
 Not queried
 
  0.7537176
 
       NA
 
       NA
  
 
  
 2
 
 153032369
 
 153032884
 
  ENSEMBL   UCSC 
 
  516
 
 11
 
 24750
 
 island
 
 0.01774
 
 0.02946
 
 Not queried
 
  0.7536165
 
       NA
 
       NA
  
 
  
 5
 
 149493008
 
 149493258
 
  ENSEMBL   UCSC 
 
  251
 
  6
 
 32365
 
 tss
 
 0.51088
 
 0.63541
 
 Not queried
 
  0.7535834
 
       NA
 
       NA
  
 
  
 19
 
  54982500
 
  54982896
 
  ENSEMBL   UCSC 
 
  397
 
  4
 
 20186
 
 gene;island
 
 0.08754
 
 0.14850
 
 Not queried
 
  0.7535577
 
       NA
 
       NA
  
 
  
 10
 
  92631135
 
  92631706
 
  ENSEMBL   UCSC 
 
  572
 
 12
 
 5202
 
 tss
 
 0.01039
 
 0.03559
 
 Not queried
 
  0.7532528
 
       NA
 
       NA
  
 
  
 14
 
  24682679
 
  24682916
 
  ENSEMBL   UCSC 
 
  238
 
  4
 
 12010
 
 gene;island
 
 0.04159
 
 0.03555
 
 Not queried
 
  0.7531738
 
       NA
 
       NA
  
 
  
 5
 
 148960831
 
 148961011
 
  ENSEMBL   UCSC 
 
  181
 
  7
 
 32361
 
 tss;island
 
 0.04936
 
 0.02078
 
 Not queried
 
  0.7530197
 
       NA
 
       NA
  
 
  
 2
 
 210636350
 
 210636560
 
  ENSEMBL   UCSC 
 
  211
 
  5
 
 23813
 
 tss
 
 0.03372
 
 0.07098
 
 Not queried
 
  0.7529339
 
       NA
 
       NA
  
 
  
 5
 
 179233589
 
 179233875
 
  ENSEMBL   UCSC 
 
  287
 
  4
 
 31879
 
 gene;tss;island
 
 0.02371
 
 0.04142
 
 Not queried
 
  0.7524937
 
       NA
 
       NA
  
 
  
 21
 
  35747390
 
  35747661
 
  ENSEMBL   UCSC 
 
  272
 
  6
 
 26487
 
 tss
 
 0.03458
 
 0.05291
 
 Not queried
 
  0.7524230
 
       NA
 
       NA
  
 
  
 6
 
  30035104
 
  30035241
 
  ENSEMBL   UCSC 
 
  138
 
  4
 
 33651
 
 gene;island
 
 0.05058
 
 0.07141
 
 Not queried
 
  0.7522739
 
       NA
 
       NA
  
 
  
 16
 
  27413459
 
  27413904
 
  ENSEMBL   UCSC 
 
  446
 
  5
 
 15120
 
 tss
 
 0.45616
 
 0.29173
 
 Not queried
 
  0.7519688
 
       NA
 
       NA
  
 
  
 19
 
  11372779
 
  11373095
 
  ENSEMBL   UCSC 
 
  317
 
  4
 
 19797
 
 gene;island
 
 0.01088
 
 0.01980
 
 Not queried
 
  0.7519510
 
       NA
 
       NA
  
 
  
 1
 
  36915704
 
  36916039
 
  ENSEMBL   UCSC 
 
  336
 
  6
 
 429
 
 gene
 
 0.20277
 
 0.12515
 
 Not queried
 
  0.7516982
 
       NA
 
       NA
  
 
  
 8
 
 124553450
 
 124553689
 
  ENSEMBL   UCSC 
 
  240
 
  7
 
 39837
 
 tss;island
 
 0.02453
 
 0.01851
 
 Not queried
 
  0.7516532
 
       NA
 
       NA
  
 
  
 14
 
  38064490
 
  38064532
 
  ENSEMBL   UCSC 
 
   43
 
  4
 
 12334
 
 tss
 
 0.01268
 
 0.02215
 
 Not queried
 
  0.7514447
 
       NA
 
       NA
  
 
  
 1
 
  84972303
 
  84972490
 
  ENSEMBL   UCSC 
 
  188
 
  5
 
 601
 
 gene;tss;island
 
 0.02065
 
 0.04019
 
 Not queried
 
  0.7512192
 
       NA
 
       NA
  
 
  
 X
 
 100074813
 
 100075247
 
  ENSEMBL   UCSC 
 
  435
 
  8
 
 41418
 
 tss
 
 0.01696
 
 0.02421
 
 Not queried
 
  0.7511982
 
       NA
 
       NA
  
 
  
 19
 
  42348327
 
  42349127
 
  ENSEMBL   UCSC 
 
  801
 
 13
 
 20778
 
 tss;island
 
 0.67777
 
 0.79171
 
 Not queried
 
  0.7511819
 
       NA
 
       NA
  
 
  
 3
 
 169781404
 
 169781717
 
  ENSEMBL   UCSC 
 
  314
 
  5
 
 27984
 
 gene
 
 0.84656
 
 0.95865
 
 Not queried
 
  0.7506896
 
       NA
 
       NA
  
 
  
 22
 
  50528564
 
  50528752
 
  ENSEMBL   UCSC 
 
  189
 
  5
 
 26882
 
 gene;island
 
 0.96282
 
 0.92697
 
 Not queried
 
  0.7506876
 
       NA
 
       NA
  
 
  
 18
 
  30352782
 
  30353283
 
  ENSEMBL   UCSC 
 
  502
 
  8
 
 19420
 
 island
 
 0.08751
 
 0.15072
 
 Not queried
 
  0.7504354
 
       NA
 
       NA
  
 
  
 2
 
 196522755
 
 196523030
 
  ENSEMBL   UCSC 
 
  276
 
  4
 
 22929
 
 gene;island
 
 0.01765
 
 0.02634
 
 Not queried
 
  0.7503514
 
       NA
 
       NA
  
 
  
 10
 
 103599520
 
 103599757
 
  ENSEMBL   UCSC 
 
  238
 
  6
 
 4716
 
 gene;island
 
 0.02499
 
 0.04033
 
 Not queried
 
  0.7503323
 
       NA
 
       NA
  
 
  
 3
 
 183894727
 
 183894778
 
  ENSEMBL   UCSC 
 
   52
 
  4
 
 28012
 
 gene;island
 
 0.21874
 
 0.33913
 
 Not queried
 
  0.7502751
 
       NA
 
       NA
  
 
  
 1
 
  37939724
 
  37940281
 
  ENSEMBL   UCSC 
 
  558
 
 10
 
 3058
 
 island
 
 0.01546
 
 0.02900
 
 Not queried
 
  0.7502315
 
       NA
 
       NA
  
 
  
 X
 
  34165065
 
  34165329
 
  ENSEMBL   UCSC 
 
  265
 
  4
 
 41707
 
 island
 
 0.92116
 
 0.86757
 
 Not queried
 
  0.7501515
 
       NA
 
       NA
  
 
  
 1
 
  92545580
 
  92545768
 
  ENSEMBL   UCSC 
 
  189
 
  4
 
 1744
 
 tss
 
 0.16014
 
 0.06870
 
 Not queried
 
  0.7501134
 
       NA
 
       NA
  
 
  
 20
 
  62168463
 
  62168955
 
  ENSEMBL   UCSC 
 
  493
 
 10
 
 26315
 
 island
 
 0.68015
 
 0.82482
 
 Not queried
 
  0.7500292
 
       NA
 
       NA
  
 
  
 15
 
  56757108
 
  56757325
 
  ENSEMBL   UCSC 
 
  218
 
  6
 
 13332
 
 gene
 
 0.01113
 
 0.01700
 
 Not queried
 
  0.7499952
 
       NA
 
       NA
  
 
  
 14
 
  76127164
 
  76127409
 
  ENSEMBL   UCSC 
 
  246
 
  6
 
 12118
 
 gene;tss;island
 
 0.03813
 
 0.04279
 
 Not queried
 
  0.7499605
 
       NA
 
       NA
  
 
  
 5
 
 140018644
 
 140018944
 
  ENSEMBL   UCSC 
 
  301
 
  9
 
 32285
 
 tss
 
 0.02233
 
 0.03140
 
 Not queried
 
  0.7498962
 
       NA
 
       NA
  
 
  
 5
 
 140018644
 
 140019523
 
  ENSEMBL   UCSC 
 
  880
 
 15
 
 33129
 
 island
 
 0.02612
 
 0.03653
 
 Not queried
 
  0.7498962
 
       NA
 
       NA
  
 
  
 4
 
  15376072
 
  15376170
 
  ENSEMBL   UCSC 
 
   99
 
  6
 
 30261
 
 tss
 
 0.91379
 
 0.96046
 
 Not queried
 
  0.7497958
 
       NA
 
       NA
  
 
  
 2
 
  62422689
 
  62423149
 
  ENSEMBL   UCSC 
 
  461
 
  8
 
 23344
 
 tss
 
 0.04355
 
 0.06661
 
 Not queried
 
  0.7497430
 
       NA
 
       NA
  
 
  
 19
 
    375746
 
    376152
 
  ENSEMBL   UCSC 
 
  407
 
  6
 
 21121
 
 island
 
 0.83728
 
 0.71454
 
 Not queried
 
  0.7497147
 
       NA
 
       NA
  
 
  
 13
 
  45563328
 
  45563598
 
  ENSEMBL   UCSC 
 
  271
 
  5
 
 11125
 
 gene
 
 0.05014
 
 0.23810
 
 Not queried
 
  0.7497108
 
       NA
 
       NA
  
 
  
 4
 
 155547985
 
 155548273
 
  ENSEMBL   UCSC 
 
  289
 
  5
 
 31329
 
 island
 
 0.01652
 
 0.04061
 
 Not queried
 
  0.7495907
 
       NA
 
       NA
  
 
  
 12
 
  69753119
 
  69753742
 
  ENSEMBL   UCSC 
 
  624
 
  7
 
 10587
 
 island
 
 0.03118
 
 0.05133
 
 Not queried
 
  0.7495137
 
       NA
 
       NA
  
 
  
 4
 
  54969963
 
  54970254
 
  ENSEMBL   UCSC 
 
  292
 
  4
 
 31000
 
 island
 
 0.10548
 
 0.04164
 
 Not queried
 
  0.7493683
 
       NA
 
       NA
  
 
  
 1
 
 208417738
 
 208418015
 
  ENSEMBL   UCSC 
 
  278
 
  5
 
 2223
 
 tss;island
 
 0.06233
 
 0.04344
 
 Not queried
 
  0.7493074
 
       NA
 
       NA
  
 
  
 2
 
  74741647
 
  74741941
 
  ENSEMBL   UCSC 
 
  295
 
  4
 
 22709
 
 gene
 
 0.24204
 
 0.14161
 
 Not queried
 
  0.7492447
 
       NA
 
       NA
  
 
  
 2
 
 220379635
 
 220379968
 
  ENSEMBL   UCSC 
 
  334
 
  4
 
 23000
 
 gene;island
 
 0.85817
 
 0.77973
 
 Not queried
 
  0.7492255
 
       NA
 
       NA
  
 
  
 11
 
 101917847
 
 101918710
 
  ENSEMBL   UCSC 
 
  864
 
 11
 
 8734
 
 island
 
 0.03583
 
 0.03902
 
 Not queried
 
  0.7491227
 
       NA
 
       NA
  
 
  
 19
 
  50353971
 
  50354304
 
  ENSEMBL   UCSC 
 
  334
 
  5
 
 20929
 
 tss;island
 
 0.02606
 
 0.02739
 
 Not queried
 
  0.7490330
 
       NA
 
       NA
  
 
  
 16
 
  89173705
 
  89174012
 
  ENSEMBL   UCSC 
 
  308
 
  4
 
 14899
 
 gene;island
 
 0.97000
 
 0.94580
 
 Not queried
 
  0.7489692
 
       NA
 
       NA
  
 
  
 16
 
  28996270
 
  28996362
 
  ENSEMBL   UCSC 
 
   93
 
  4
 
 15134
 
 tss
 
 0.77242
 
 0.84634
 
 Not queried
 
  0.7489667
 
       NA
 
       NA
  
 
  
 7
 
  56131505
 
  56131904
 
  ENSEMBL   UCSC 
 
  400
 
  7
 
 37543
 
 tss
 
 0.04805
 
 0.08181
 
 Not queried
 
  0.7487186
 
       NA
 
       NA
  
 
  
 3
 
  53857698
 
  53857989
 
  ENSEMBL   UCSC 
 
  292
 
  5
 
 27789
 
 gene;island
 
 0.91753
 
 0.85394
 
 Not queried
 
  0.7487159
 
       NA
 
       NA
  
 
  
 1
 
  15127680
 
  15127907
 
  ENSEMBL   UCSC 
 
  228
 
  4
 
 237
 
 gene;island
 
 0.91182
 
 0.83287
 
 Not queried
 
  0.7485908
 
       NA
 
       NA
  
 
  
 X
 
 114468596
 
 114468879
 
  ENSEMBL   UCSC 
 
  284
 
  5
 
 41970
 
 island
 
 0.04388
 
 0.03231
 
 Not queried
 
  0.7485601
 
       NA
 
       NA
  
 
  
 3
 
 127309512
 
 127309564
 
  ENSEMBL   UCSC 
 
   53
 
  4
 
 27890
 
 gene
 
 0.00846
 
 0.00733
 
 Not queried
 
  0.7483956
 
       NA
 
       NA
  
 
  
 12
 
  56521594
 
  56522042
 
  ENSEMBL   UCSC 
 
  449
 
  9
 
 9760
 
 tss
 
 0.01941
 
 0.02921
 
 Not queried
 
  0.7481463
 
       NA
 
       NA
  
 
  
 12
 
  56521594
 
  56522453
 
  ENSEMBL   UCSC 
 
  860
 
 13
 
 10498
 
 island
 
 0.01941
 
 0.02921
 
 Not queried
 
  0.7481463
 
       NA
 
       NA
  
 
  
 6
 
  31964193
 
  31964391
 
  ENSEMBL   UCSC 
 
  199
 
  5
 
 33947
 
 gene
 
 0.74721
 
 0.56517
 
 Not queried
 
  0.7480889
 
       NA
 
       NA
  
 
  
 1
 
  17292908
 
  17293256
 
  ENSEMBL   UCSC 
 
  349
 
  4
 
 263
 
 gene;island
 
 0.62208
 
 0.73141
 
 Not queried
 
  0.7480016
 
       NA
 
       NA
  
 
  
 22
 
  43505892
 
  43506952
 
  ENSEMBL   UCSC 
 
 1061
 
 27
 
 27509
 
 island
 
 0.02092
 
 0.03626
 
 Not queried
 
  0.7479131
 
       NA
 
       NA
  
 
  
 2
 
  33824170
 
  33824342
 
  ENSEMBL   UCSC 
 
  173
 
  4
 
 22606
 
 gene
 
 0.02206
 
 0.02924
 
 Not queried
 
  0.7478987
 
       NA
 
       NA
  
 
  
 2
 
 175193377
 
 175193551
 
  ENSEMBL   UCSC 
 
  175
 
  4
 
 24834
 
 island
 
 0.03706
 
 0.02627
 
 Not queried
 
  0.7477779
 
       NA
 
       NA
  
 
  
 19
 
  44905782
 
  44905980
 
  ENSEMBL   UCSC 
 
  199
 
  7
 
 20812
 
 tss
 
 0.02214
 
 0.06828
 
 Not queried
 
  0.7473521
 
       NA
 
       NA
  
 
  
 17
 
  73936726
 
  73937416
 
  ENSEMBL   UCSC 
 
  691
 
 10
 
 18733
 
 island
 
 0.01873
 
 0.02857
 
 Not queried
 
  0.7471920
 
       NA
 
       NA
  
 
  
 11
 
   1097602
 
   1098080
 
  ENSEMBL   UCSC 
 
  479
 
  4
 
 6387
 
 gene;island
 
 0.53324
 
 0.61507
 
 Not queried
 
  0.7471839
 
       NA
 
       NA
  
 
  
 17
 
  36831188
 
  36831499
 
  ENSEMBL   UCSC 
 
  312
 
  8
 
 17260
 
 tss
 
 0.00940
 
 0.01236
 
 Not queried
 
  0.7471638
 
       NA
 
       NA
  
 
  
 5
 
   1257034
 
   1257221
 
  ENSEMBL   UCSC 
 
  188
 
  5
 
 31476
 
 gene;island
 
 0.76977
 
 0.88807
 
 Not queried
 
  0.7470853
 
       NA
 
       NA
  
 
  
 3
 
 170302890
 
 170303286
 
  ENSEMBL   UCSC 
 
  397
 
  5
 
 27986
 
 gene;island
 
 0.12510
 
 0.10698
 
 Not queried
 
  0.7467589
 
       NA
 
       NA
  
 
  
 15
 
  35047023
 
  35047411
 
  ENSEMBL   UCSC 
 
  389
 
  6
 
 13535
 
 tss;island
 
 0.02076
 
 0.04022
 
 Not queried
 
  0.7466925
 
       NA
 
       NA
  
 
  
 15
 
  90792609
 
  90792717
 
  ENSEMBL   UCSC 
 
  109
 
  4
 
 13870
 
 tss
 
 0.02491
 
 0.03143
 
 Not queried
 
  0.7466357
 
       NA
 
       NA
  
 
  
 17
 
  71640181
 
  71640369
 
  ENSEMBL   UCSC 
 
  189
 
  7
 
 18671
 
 island
 
 0.04967
 
 0.02997
 
 Not queried
 
  0.7465687
 
       NA
 
       NA
  
 
  
 6
 
  91006592
 
  91006647
 
  ENSEMBL   UCSC 
 
   56
 
  6
 
 35059
 
 tss;island
 
 0.00999
 
 0.01792
 
 Not queried
 
  0.7465518
 
       NA
 
       NA
  
 
  
 6
 
  32062160
 
  32062554
 
  ENSEMBL   UCSC 
 
  395
 
  5
 
 33983
 
 gene;island
 
 0.66697
 
 0.55730
 
 Not queried
 
  0.7462315
 
       NA
 
       NA
  
 
  
 X
 
  21857475
 
  21857796
 
  ENSEMBL   UCSC 
 
  322
 
  9
 
 41682
 
 island
 
 0.00929
 
 0.02490
 
 Not queried
 
  0.7461259
 
       NA
 
       NA
  
 
  
 16
 
  23689753
 
  23690079
 
  ENSEMBL   UCSC 
 
  327
 
  5
 
 15111
 
 tss;island
 
 0.04597
 
 0.09817
 
 Not queried
 
  0.7460882
 
       NA
 
       NA
  
 
  
 7
 
 152063901
 
 152064079
 
  ENSEMBL   UCSC 
 
  179
 
  4
 
 37236
 
 gene;island
 
 0.96867
 
 0.94743
 
 Not queried
 
  0.7459746
 
       NA
 
       NA
  
 
  
 11
 
  10830634
 
  10830706
 
  ENSEMBL   UCSC 
 
   73
 
  4
 
 7129
 
 tss
 
 0.01719
 
 0.02078
 
 Not queried
 
  0.7459490
 
       NA
 
       NA
  
 
  
 1
 
 202311052
 
 202311278
 
  ENSEMBL   UCSC 
 
  227
 
  7
 
 4074
 
 island
 
 0.01011
 
 0.02023
 
 Not queried
 
  0.7459352
 
       NA
 
       NA
  
 
  
 3
 
  14220276
 
  14220525
 
  ENSEMBL   UCSC 
 
  250
 
  8
 
 27647
 
 gene
 
 0.01916
 
 0.02695
 
 Not queried
 
  0.7459320
 
       NA
 
       NA
  
 
  
 6
 
  31828980
 
  31830035
 
  ENSEMBL   UCSC 
 
 1056
 
 20
 
 33910
 
 gene;island
 
 0.73233
 
 0.80190
 
 Not queried
 
  0.7456364
 
       NA
 
       NA
  
 
  
 11
 
  73019337
 
  73019558
 
  ENSEMBL   UCSC 
 
  222
 
  7
 
 7526
 
 tss
 
 0.01532
 
 0.02112
 
 Not queried
 
  0.7456305
 
       NA
 
       NA
  
 
  
 20
 
  55966417
 
  55966451
 
  ENSEMBL   UCSC 
 
   35
 
  5
 
 25804
 
 tss
 
 0.01963
 
 0.03248
 
 Not queried
 
  0.7456179
 
       NA
 
       NA
  
 
  
 4
 
  37961862
 
  37962205
 
  ENSEMBL   UCSC 
 
  344
 
  4
 
 29930
 
 gene
 
 0.91707
 
 0.91298
 
 Not queried
 
  0.7455845
 
       NA
 
       NA
  
 
  
 5
 
 139493486
 
 139493680
 
  ENSEMBL   UCSC 
 
  195
 
  9
 
 32278
 
 tss;island
 
 0.03088
 
 0.03667
 
 Not queried
 
  0.7455638
 
       NA
 
       NA
  
 
  
 19
 
  41815869
 
  41816349
 
  ENSEMBL   UCSC 
 
  481
 
  8
 
 22015
 
 island
 
 0.01441
 
 0.02180
 
 Not queried
 
  0.7455503
 
       NA
 
       NA
  
 
  
 11
 
   1595783
 
   1596165
 
  ENSEMBL   UCSC 
 
  383
 
  5
 
 6407
 
 gene;island
 
 0.02905
 
 0.02830
 
 Not queried
 
  0.7455151
 
       NA
 
       NA
  
 
  
 1
 
 201708500
 
 201708932
 
  ENSEMBL   UCSC 
 
  433
 
  8
 
 938
 
 gene;tss;island
 
 0.07130
 
 0.03278
 
 Not queried
 
  0.7454975
 
       NA
 
       NA
  
 
  
 1
 
  28585612
 
  28586105
 
  ENSEMBL   UCSC 
 
  494
 
  7
 
 2935
 
 island
 
 0.02454
 
 0.03037
 
 Not queried
 
  0.7452634
 
       NA
 
       NA
  
 
  
 19
 
   5838729
 
   5838999
 
  ENSEMBL   UCSC 
 
  271
 
  7
 
 19722
 
 gene
 
 0.89201
 
 0.78884
 
 Not queried
 
  0.7451712
 
       NA
 
       NA
  
 
  
 1
 
  53904065
 
  53904319
 
  ENSEMBL   UCSC 
 
  255
 
  4
 
 539
 
 gene;island
 
 0.91353
 
 0.86566
 
 Not queried
 
  0.7451487
 
       NA
 
       NA
  
 
  
 6
 
  24126060
 
  24126497
 
  ENSEMBL   UCSC 
 
  438
 
  9
 
 35424
 
 island
 
 0.08029
 
 0.06948
 
 Not queried
 
  0.7450515
 
       NA
 
       NA
  
 
  
 11
 
   1847227
 
   1847357
 
  ENSEMBL   UCSC 
 
  131
 
  4
 
 7913
 
 island
 
 0.72808
 
 0.85988
 
 Not queried
 
  0.7450465
 
       NA
 
       NA
  
 
  
 19
 
  49699879
 
  49700021
 
  ENSEMBL   UCSC 
 
  143
 
  4
 
 20110
 
 gene;island
 
 0.75406
 
 0.60378
 
 Not queried
 
  0.7449990
 
       NA
 
       NA
  
 
  
 1
 
  71546313
 
  71546685
 
  ENSEMBL   UCSC 
 
  373
 
  4
 
 581
 
 gene
 
 0.02552
 
 0.04203
 
 Not queried
 
  0.7448490
 
       NA
 
       NA
  
 
  
 8
 
  72469430
 
  72469560
 
  ENSEMBL   UCSC 
 
  131
 
  4
 
 40315
 
 island
 
 0.02863
 
 0.02343
 
 Not queried
 
  0.7447362
 
       NA
 
       NA
  
 
  
 16
 
  31128800
 
  31128929
 
  ENSEMBL   UCSC 
 
  130
 
  4
 
 15176
 
 tss;island
 
 0.02897
 
 0.04152
 
 Not queried
 
  0.7447318
 
       NA
 
       NA
  
 
  
 7
 
   5601811
 
   5602219
 
  ENSEMBL   UCSC 
 
  409
 
  4
 
 38167
 
 island
 
 0.03399
 
 0.02155
 
 Not queried
 
  0.7446819
 
       NA
 
       NA
  
 
  
 19
 
    375746
 
    375995
 
  ENSEMBL   UCSC 
 
  250
 
  4
 
 19589
 
 gene
 
 0.76383
 
 0.65915
 
 Not queried
 
  0.7446488
 
       NA
 
       NA
  
 
  
 17
 
  73127913
 
  73128192
 
  ENSEMBL   UCSC 
 
  280
 
  9
 
 17611
 
 tss
 
 0.03344
 
 0.04791
 
 Not queried
 
  0.7445127
 
       NA
 
       NA
  
 
  
 3
 
  44519063
 
  44519333
 
  ENSEMBL   UCSC 
 
  271
 
  4
 
 28991
 
 island
 
 0.17204
 
 0.05321
 
 Not queried
 
  0.7444251
 
       NA
 
       NA
  
 
  
 2
 
  20866107
 
  20866500
 
  ENSEMBL   UCSC 
 
  394
 
  9
 
 24077
 
 island
 
 0.01934
 
 0.02605
 
 Not queried
 
  0.7443352
 
       NA
 
       NA
  
 
  
 22
 
  46508451
 
  46508604
 
  ENSEMBL   UCSC 
 
  154
 
  6
 
 26866
 
 gene;tss
 
 0.88489
 
 0.90094
 
 Not queried
 
  0.7443105
 
       NA
 
       NA
  
 
  
 8
 
 145981074
 
 145981224
 
  ENSEMBL   UCSC 
 
  151
 
  8
 
 39927
 
 tss;island
 
 0.01829
 
 0.03243
 
 Not queried
 
  0.7443057
 
       NA
 
       NA
  
 
  
 8
 
 145064008
 
 145064193
 
  ENSEMBL   UCSC 
 
  186
 
  5
 
 39905
 
 tss;island
 
 0.02456
 
 0.03516
 
 Not queried
 
  0.7442564
 
       NA
 
       NA
  
 
  
 4
 
  55093834
 
  55094011
 
  ENSEMBL   UCSC 
 
  178
 
  4
 
 30353
 
 tss
 
 0.01464
 
 0.02743
 
 Not queried
 
  0.7442085
 
       NA
 
       NA
  
 
  
 4
 
  55093612
 
  55094011
 
  ENSEMBL   UCSC 
 
  400
 
  6
 
 31002
 
 island
 
 0.02080
 
 0.02877
 
 Not queried
 
  0.7442085
 
       NA
 
       NA
  
 
  
 18
 
  47866494
 
  47866831
 
  ENSEMBL   UCSC 
 
  338
 
  5
 
 19477
 
 island
 
 0.90527
 
 0.80502
 
 Not queried
 
  0.7440537
 
       NA
 
       NA
  
 
  
 5
 
 176943966
 
 176944204
 
  ENSEMBL   UCSC 
 
  239
 
  8
 
 33392
 
 island
 
 0.05242
 
 0.06234
 
 Not queried
 
  0.7440227
 
       NA
 
       NA
  
 
  
 11
 
   2464822
 
   2465491
 
  ENSEMBL   UCSC 
 
  670
 
 15
 
 7053
 
 tss;island
 
 0.41397
 
 0.52565
 
 Not queried
 
  0.7437699
 
       NA
 
       NA
  
 
  
 15
 
  43802735
 
  43802888
 
  ENSEMBL   UCSC 
 
  154
 
  7
 
 13601
 
 tss
 
 0.02044
 
 0.04001
 
 Not queried
 
  0.7437452
 
       NA
 
       NA
  
 
  
 1
 
  54871856
 
  54872113
 
  ENSEMBL   UCSC 
 
  258
 
  5
 
 3288
 
 island
 
 0.06444
 
 0.10341
 
 Not queried
 
  0.7437447
 
       NA
 
       NA
  
 
  
 16
 
  89752847
 
  89753195
 
  ENSEMBL   UCSC 
 
  349
 
  5
 
 16232
 
 island
 
 0.07730
 
 0.04764
 
 Not queried
 
  0.7437104
 
       NA
 
       NA
  
 
  
 17
 
  48911983
 
  48912556
 
  ENSEMBL   UCSC 
 
  574
 
 12
 
 17475
 
 tss
 
 0.80035
 
 0.87962
 
 Not queried
 
  0.7436580
 
       NA
 
       NA
  
 
  
 21
 
  45209501
 
  45210009
 
  ENSEMBL   UCSC 
 
  509
 
  6
 
 26407
 
 gene;island
 
 0.02030
 
 0.02904
 
 Not queried
 
  0.7436254
 
       NA
 
       NA
  
 
  
 19
 
   5784679
 
   5784947
 
  ENSEMBL   UCSC 
 
  269
 
  6
 
 20346
 
 tss;island
 
 0.88665
 
 0.91937
 
 Not queried
 
  0.7435299
 
       NA
 
       NA
  
 
  
 17
 
  42147905
 
  42148070
 
  ENSEMBL   UCSC 
 
  166
 
  7
 
 17367
 
 tss
 
 0.01409
 
 0.02068
 
 Not queried
 
  0.7435192
 
       NA
 
       NA
  
 
  
 17
 
  59940709
 
  59941132
 
  ENSEMBL   UCSC 
 
  424
 
  8
 
 18599
 
 island
 
 0.00566
 
 0.01581
 
 Not queried
 
  0.7434516
 
       NA
 
       NA
  
 
  
 16
 
  31724470
 
  31724534
 
  ENSEMBL   UCSC 
 
   65
 
  4
 
 15185
 
 tss
 
 0.01912
 
 0.03121
 
 Not queried
 
  0.7433920
 
       NA
 
       NA
  
 
  
 15
 
  25483498
 
  25483711
 
  ENSEMBL   UCSC 
 
  214
 
  4
 
 13505
 
 tss
 
 0.85322
 
 0.90979
 
 Not queried
 
  0.7432355
 
       NA
 
       NA
  
 
  
 12
 
 117537264
 
 117537710
 
  ENSEMBL   UCSC 
 
  447
 
  6
 
 10018
 
 tss;island
 
 0.05127
 
 0.04663
 
 Not queried
 
  0.7431227
 
       NA
 
       NA
  
 
  
 19
 
   8372814
 
   8373608
 
  ENSEMBL   UCSC 
 
  795
 
 10
 
 21443
 
 island
 
 0.01484
 
 0.02632
 
 Not queried
 
  0.7430825
 
       NA
 
       NA
  
 
  
 1
 
 110949893
 
 110950539
 
  ENSEMBL   UCSC 
 
  647
 
  6
 
 676
 
 gene
 
 0.02711
 
 0.03950
 
 Not queried
 
  0.7429406
 
       NA
 
       NA
  
 
  
 11
 
  10830481
 
  10830706
 
  ENSEMBL   UCSC 
 
  226
 
  5
 
 8040
 
 island
 
 0.02021
 
 0.02194
 
 Not queried
 
  0.7429276
 
       NA
 
       NA
  
 
  
 7
 
  39605536
 
  39606071
 
  ENSEMBL   UCSC 
 
  536
 
 11
 
 38361
 
 island
 
 0.01371
 
 0.02484
 
 Not queried
 
  0.7428299
 
       NA
 
       NA
  
 
  
 7
 
 121943990
 
 121944500
 
  ENSEMBL   UCSC 
 
  511
 
  5
 
 37142
 
 gene
 
 0.08800
 
 0.01636
 
 Not queried
 
  0.7420082
 
       NA
 
       NA
  
 
  
 11
 
  46414401
 
  46414626
 
  ENSEMBL   UCSC 
 
  226
 
  4
 
 8229
 
 island
 
 0.00918
 
 0.01490
 
 Not queried
 
  0.7418835
 
       NA
 
       NA
  
 
  
 2
 
 170655263
 
 170655321
 
  ENSEMBL   UCSC 
 
   59
 
  4
 
 23667
 
 tss
 
 0.02323
 
 0.03114
 
 Not queried
 
  0.7418281
 
       NA
 
       NA
  
 
  
 1
 
  57890317
 
  57890636
 
  ENSEMBL   UCSC 
 
  320
 
  4
 
 554
 
 gene;island
 
 0.02929
 
 0.02601
 
 Not queried
 
  0.7417940
 
       NA
 
       NA
  
 
  
 19
 
  19611834
 
  19612071
 
  ENSEMBL   UCSC 
 
  238
 
  4
 
 19917
 
 gene;island
 
 0.71771
 
 0.82481
 
 Not queried
 
  0.7417871
 
       NA
 
       NA
  
 
  
 2
 
  79740185
 
  79740322
 
  ENSEMBL   UCSC 
 
  138
 
  4
 
 22717
 
 gene;island
 
 0.01190
 
 0.02190
 
 Not queried
 
  0.7416856
 
       NA
 
       NA
  
 
  
 7
 
  72349822
 
  72350033
 
  ENSEMBL   UCSC 
 
  212
 
  6
 
 38485
 
 island
 
 0.02800
 
 0.01699
 
 Not queried
 
  0.7414658
 
       NA
 
       NA
  
 
  
 12
 
  53895069
 
  53895312
 
  ENSEMBL   UCSC 
 
  244
 
  7
 
 9719
 
 tss
 
 0.02118
 
 0.03342
 
 Not queried
 
  0.7414391
 
       NA
 
       NA
  
 
  
 6
 
  28226885
 
  28227093
 
  ENSEMBL   UCSC 
 
  209
 
  7
 
 34674
 
 tss
 
 0.87371
 
 0.91625
 
 Not queried
 
  0.7413771
 
       NA
 
       NA
  
 
  
 6
 
  28226885
 
  28227482
 
  ENSEMBL   UCSC 
 
  598
 
 11
 
 35522
 
 island
 
 0.87371
 
 0.92228
 
 Not queried
 
  0.7413771
 
       NA
 
       NA
  
 
  
 7
 
 156741514
 
 156741808
 
  ENSEMBL   UCSC 
 
  295
 
  5
 
 37954
 
 tss;island
 
 0.87202
 
 0.58788
 
 Not queried
 
  0.7413542
 
       NA
 
       NA
  
 
  
 11
 
  31819148
 
  31819464
 
  ENSEMBL   UCSC 
 
  317
 
  6
 
 6537
 
 gene;island
 
 0.06564
 
 0.13607
 
 Not queried
 
  0.7413290
 
       NA
 
       NA
  
 
  
 2
 
 121411889
 
 121412432
 
  ENSEMBL   UCSC 
 
  544
 
  5
 
 24630
 
 island
 
 0.88548
 
 0.84789
 
 Not queried
 
  0.7412184
 
       NA
 
       NA
  
 
  
 11
 
  64022763
 
  64023183
 
  ENSEMBL   UCSC 
 
  421
 
  7
 
 6652
 
 gene;island
 
 0.82857
 
 0.84662
 
 Not queried
 
  0.7411229
 
       NA
 
       NA
  
 
  
 1
 
 156736588
 
 156737250
 
  ENSEMBL   UCSC 
 
  663
 
  9
 
 2020
 
 tss;island
 
 0.01801
 
 0.02945
 
 Not queried
 
  0.7410518
 
       NA
 
       NA
  
 
  
 5
 
 176827671
 
 176827833
 
  ENSEMBL   UCSC 
 
  163
 
  4
 
 32467
 
 tss;island
 
 0.70653
 
 0.84002
 
 Not queried
 
  0.7410231
 
       NA
 
       NA
  
 
  
 19
 
  18118666
 
  18119069
 
  ENSEMBL   UCSC 
 
  404
 
  6
 
 19891
 
 gene;island
 
 0.01230
 
 0.03070
 
 Not queried
 
  0.7409861
 
       NA
 
       NA
  
 
  
 19
 
  18118666
 
  18118880
 
  ENSEMBL   UCSC 
 
  215
 
  4
 
 20572
 
 tss
 
 0.02353
 
 0.03842
 
 Not queried
 
  0.7409861
 
       NA
 
       NA
  
 
  
 4
 
  73434487
 
  73434856
 
  ENSEMBL   UCSC 
 
  370
 
  5
 
 31040
 
 island
 
 0.02530
 
 0.04145
 
 Not queried
 
  0.7409531
 
       NA
 
       NA
  
 
  
 11
 
  64081430
 
  64081631
 
  ENSEMBL   UCSC 
 
  202
 
  5
 
 6662
 
 gene;island
 
 0.88289
 
 0.92504
 
 Not queried
 
  0.7408831
 
       NA
 
       NA
  
 
  
 4
 
 144435546
 
 144435908
 
  ENSEMBL   UCSC 
 
  363
 
  4
 
 30095
 
 gene;island
 
 0.00751
 
 0.01475
 
 Not queried
 
  0.7408445
 
       NA
 
       NA
  
 
  
 3
 
 183165321
 
 183165594
 
  ENSEMBL   UCSC 
 
  274
 
  6
 
 29661
 
 island
 
 0.02331
 
 0.02817
 
 Not queried
 
  0.7407168
 
       NA
 
       NA
  
 
  
 6
 
  52441723
 
  52441892
 
  ENSEMBL   UCSC 
 
  170
 
  4
 
 36142
 
 island
 
 0.05871
 
 0.10963
 
 Not queried
 
  0.7406709
 
       NA
 
       NA
  
 
  
 10
 
 104155285
 
 104155650
 
  ENSEMBL   UCSC 
 
  366
 
  6
 
 4720
 
 gene;island
 
 0.02652
 
 0.04951
 
 Not queried
 
  0.7406406
 
       NA
 
       NA
  
 
  
 1
 
 156862113
 
 156862299
 
  ENSEMBL   UCSC 
 
  187
 
  4
 
 2021
 
 tss;island
 
 0.01306
 
 0.02198
 
 Not queried
 
  0.7404323
 
       NA
 
       NA
  
 
  
 8
 
  99956467
 
  99956874
 
  ENSEMBL   UCSC 
 
  408
 
  7
 
 40421
 
 island
 
 0.01954
 
 0.02593
 
 Not queried
 
  0.7403934
 
       NA
 
       NA
  
 
  
 2
 
 197457277
 
 197457430
 
  ENSEMBL   UCSC 
 
  154
 
  6
 
 24936
 
 island
 
 0.03152
 
 0.04074
 
 Not queried
 
  0.7402367
 
       NA
 
       NA
  
 
  
 7
 
 127983655
 
 127983900
 
  ENSEMBL   UCSC 
 
  246
 
  4
 
 37149
 
 gene
 
 0.01317
 
 0.02013
 
 Not queried
 
  0.7401563
 
       NA
 
       NA
  
 
  
 16
 
  11350740
 
  11351330
 
  ENSEMBL   UCSC 
 
  591
 
 10
 
 15061
 
 tss;island
 
 0.03260
 
 0.02424
 
 Not queried
 
  0.7400848
 
       NA
 
       NA
  
 
  
 13
 
 113429178
 
 113429562
 
  ENSEMBL   UCSC 
 
  385
 
  5
 
 11225
 
 gene;island
 
 0.96446
 
 0.95651
 
 Not queried
 
  0.7399688
 
       NA
 
       NA
  
 
  
 17
 
  77478569
 
  77478927
 
  ENSEMBL   UCSC 
 
  359
 
  5
 
 17681
 
 tss
 
 0.58384
 
 0.70488
 
 Not queried
 
  0.7399205
 
       NA
 
       NA
  
 
  
 2
 
  71357459
 
  71357524
 
  ENSEMBL   UCSC 
 
   66
 
  5
 
 22692
 
 gene
 
 0.01737
 
 0.03265
 
 Not queried
 
  0.7396065
 
       NA
 
       NA
  
 
  
 19
 
  11456854
 
  11456965
 
  ENSEMBL   UCSC 
 
  112
 
  5
 
 19799
 
 gene
 
 0.05597
 
 0.09006
 
 Not queried
 
  0.7394684
 
       NA
 
       NA
  
 
  
 X
 
  89176658
 
  89176915
 
  ENSEMBL   UCSC 
 
  258
 
  4
 
 41412
 
 tss
 
 0.88970
 
 0.89835
 
 Not queried
 
  0.7393749
 
       NA
 
       NA
  
 
  
 5
 
 138211043
 
 138211184
 
  ENSEMBL   UCSC 
 
  142
 
  6
 
 31714
 
 gene
 
 0.86643
 
 0.90787
 
 Not queried
 
  0.7391691
 
       NA
 
       NA
  
 
  
 2
 
  68592345
 
  68592394
 
  ENSEMBL   UCSC 
 
   50
 
  4
 
 22677
 
 gene
 
 0.61975
 
 0.52863
 
 Not queried
 
  0.7390835
 
       NA
 
       NA
  
 
  
 1
 
  47690795
 
  47691301
 
  ENSEMBL   UCSC 
 
  507
 
  5
 
 514
 
 gene;island
 
 0.06140
 
 0.06160
 
 Not queried
 
  0.7390226
 
       NA
 
       NA
  
 
  
 5
 
   1491377
 
   1491600
 
  ENSEMBL   UCSC 
 
  224
 
  4
 
 31489
 
 gene;island
 
 0.93340
 
 0.91917
 
 Not queried
 
  0.7389713
 
       NA
 
       NA
  
 
  
 16
 
   3074294
 
   3074537
 
  ENSEMBL   UCSC 
 
  244
 
  7
 
 15016
 
 tss
 
 0.02310
 
 0.02763
 
 Not queried
 
  0.7388346
 
       NA
 
       NA
  
 
  
 19
 
  54415702
 
  54416068
 
  ENSEMBL   UCSC 
 
  367
 
  6
 
 21009
 
 tss
 
 0.47439
 
 0.38332
 
 Not queried
 
  0.7388114
 
       NA
 
       NA
  
 
  
 3
 
 171178943
 
 171179043
 
  ENSEMBL   UCSC 
 
  101
 
  4
 
 28675
 
 tss;island
 
 0.01742
 
 0.02133
 
 Not queried
 
  0.7387678
 
       NA
 
       NA
  
 
  
 4
 
  17578564
 
  17578868
 
  ENSEMBL   UCSC 
 
  305
 
  8
 
 30269
 
 tss
 
 0.02941
 
 0.07205
 
 Not queried
 
  0.7387663
 
       NA
 
       NA
  
 
  
 12
 
 120799597
 
 120800066
 
  ENSEMBL   UCSC 
 
  470
 
  4
 
 9325
 
 gene;island
 
 0.22145
 
 0.18335
 
 Not queried
 
  0.7387392
 
       NA
 
       NA
  
 
  
 16
 
   4852825
 
   4853175
 
  ENSEMBL   UCSC 
 
  351
 
  7
 
 15046
 
 tss;island
 
 0.01737
 
 0.02037
 
 Not queried
 
  0.7387191
 
       NA
 
       NA
  
 
  
 17
 
   4045930
 
   4046484
 
  ENSEMBL   UCSC 
 
  555
 
 11
 
 16987
 
 tss;island
 
 0.02264
 
 0.03046
 
 Not queried
 
  0.7386954
 
       NA
 
       NA
  
 
  
 6
 
  32182679
 
  32182745
 
  ENSEMBL   UCSC 
 
   67
 
  4
 
 34032
 
 gene
 
 0.95585
 
 0.93185
 
 Not queried
 
  0.7386181
 
       NA
 
       NA
  
 
  
 19
 
  45805662
 
  45805935
 
  ENSEMBL   UCSC 
 
  274
 
  4
 
 20055
 
 gene;island
 
 0.88641
 
 0.90009
 
 Not queried
 
  0.7385827
 
       NA
 
       NA
  
 
  
 10
 
   1068490
 
   1068763
 
  ENSEMBL   UCSC 
 
  274
 
  6
 
 4439
 
 gene
 
 0.85063
 
 0.88673
 
 Not queried
 
  0.7385538
 
       NA
 
       NA
  
 
  
 16
 
  10912331
 
  10912718
 
  ENSEMBL   UCSC 
 
  388
 
  6
 
 15664
 
 island
 
 0.50475
 
 0.57562
 
 Not queried
 
  0.7384426
 
       NA
 
       NA
  
 
  
 8
 
   6662963
 
   6663401
 
  ENSEMBL   UCSC 
 
  439
 
  4
 
 39988
 
 island
 
 0.74798
 
 0.88046
 
 Not queried
 
  0.7381642
 
       NA
 
       NA
  
 
  
 7
 
  44279049
 
  44279273
 
  ENSEMBL   UCSC 
 
  225
 
  4
 
 36929
 
 gene;island
 
 0.60723
 
 0.74228
 
 Not queried
 
  0.7380592
 
       NA
 
       NA
  
 
  
 16
 
  22386311
 
  22386531
 
  ENSEMBL   UCSC 
 
  221
 
  4
 
 15101
 
 tss;island
 
 0.01949
 
 0.01255
 
 Not queried
 
  0.7379754
 
       NA
 
       NA
  
 
  
 2
 
 189156378
 
 189156549
 
  ENSEMBL   UCSC 
 
  172
 
  4
 
 23738
 
 tss;island
 
 0.01322
 
 0.02253
 
 Not queried
 
  0.7378879
 
       NA
 
       NA
  
 
  
 7
 
  77648378
 
  77648624
 
  ENSEMBL   UCSC 
 
  247
 
  4
 
 37000
 
 gene;island
 
 0.01283
 
 0.01598
 
 Not queried
 
  0.7378180
 
       NA
 
       NA
  
 
  
 3
 
  50283852
 
  50284305
 
  ENSEMBL   UCSC 
 
  454
 
  4
 
 27755
 
 gene;tss
 
 0.43237
 
 0.43532
 
 Not queried
 
  0.7377449
 
       NA
 
       NA
  
 
  
 22
 
  29601559
 
  29601862
 
  ENSEMBL   UCSC 
 
  304
 
  6
 
 27002
 
 tss
 
 0.05898
 
 0.06213
 
 Not queried
 
  0.7376658
 
       NA
 
       NA
  
 
  
 6
 
  31373932
 
  31374206
 
  ENSEMBL   UCSC 
 
  275
 
  4
 
 33809
 
 gene;island
 
 0.60528
 
 0.55869
 
 Not queried
 
  0.7374719
 
       NA
 
       NA
  
 
  
 10
 
    575899
 
    576100
 
  ENSEMBL   UCSC 
 
  202
 
  5
 
 4430
 
 gene;island
 
 0.95346
 
 0.86837
 
 Not queried
 
  0.7374474
 
       NA
 
       NA
  
 
  
 2
 
  70351227
 
  70351524
 
  ENSEMBL   UCSC 
 
  298
 
  4
 
 22684
 
 gene;island
 
 0.02443
 
 0.03998
 
 Not queried
 
  0.7374080
 
       NA
 
       NA
  
 
  
 19
 
  38943334
 
  38943724
 
  ENSEMBL   UCSC 
 
  391
 
  4
 
 19983
 
 gene;island
 
 0.49811
 
 0.36950
 
 Not queried
 
  0.7373876
 
       NA
 
       NA
  
 
  
 1
 
 102462806
 
 102462877
 
  ENSEMBL   UCSC 
 
   72
 
  4
 
 1781
 
 tss
 
 0.39566
 
 0.46070
 
 Not queried
 
  0.7373656
 
       NA
 
       NA
  
 
  
 16
 
  19896246
 
  19896644
 
  ENSEMBL   UCSC 
 
  399
 
  4
 
 15089
 
 tss
 
 0.10828
 
 0.20471
 
 Not queried
 
  0.7373654
 
       NA
 
       NA
  
 
  
 12
 
  47219793
 
  47220092
 
  ENSEMBL   UCSC 
 
  300
 
  9
 
 9634
 
 tss
 
 0.04024
 
 0.02453
 
 Not queried
 
  0.7373431
 
       NA
 
       NA
  
 
  
 6
 
  24667276
 
  24667597
 
  ENSEMBL   UCSC 
 
  322
 
  6
 
 33529
 
 gene
 
 0.05931
 
 0.02331
 
 Not queried
 
  0.7372672
 
       NA
 
       NA
  
 
  
 15
 
  67546975
 
  67547294
 
  ENSEMBL   UCSC 
 
  320
 
  8
 
 13365
 
 gene;tss;island
 
 0.01177
 
 0.01320
 
 Not queried
 
  0.7371975
 
       NA
 
       NA
  
 
  
 3
 
  62363922
 
  62364390
 
  ENSEMBL   UCSC 
 
  469
 
  6
 
 29208
 
 island
 
 0.04840
 
 0.06367
 
 Not queried
 
  0.7371673
 
       NA
 
       NA
  
 
  
 12
 
  75728104
 
  75728458
 
  ENSEMBL   UCSC 
 
  355
 
  5
 
 9855
 
 tss
 
 0.01184
 
 0.01754
 
 Not queried
 
  0.7371186
 
       NA
 
       NA
  
 
  
 3
 
  67705044
 
  67705285
 
  ENSEMBL   UCSC 
 
  242
 
  5
 
 28393
 
 tss
 
 0.04069
 
 0.06603
 
 Not queried
 
  0.7371095
 
       NA
 
       NA
  
 
  
 4
 
   2277576
 
   2277836
 
  ENSEMBL   UCSC 
 
  261
 
  4
 
 29840
 
 gene;island
 
 0.90736
 
 0.93481
 
 Not queried
 
  0.7369679
 
       NA
 
       NA
  
 
  
 17
 
  74721824
 
  74722097
 
  ENSEMBL   UCSC 
 
  274
 
  6
 
 16757
 
 gene;tss;island
 
 0.03555
 
 0.05880
 
 Not queried
 
  0.7369130
 
       NA
 
       NA
  
 
  
 1
 
  25943790
 
  25943891
 
  ENSEMBL   UCSC 
 
  102
 
  5
 
 1369
 
 tss;island
 
 0.03636
 
 0.03696
 
 Not queried
 
  0.7368421
 
       NA
 
       NA
  
 
  
 9
 
  35096654
 
  35096980
 
  ENSEMBL   UCSC 
 
  327
 
  4
 
 40744
 
 tss;island
 
 0.01918
 
 0.03090
 
 Not queried
 
  0.7367907
 
       NA
 
       NA
  
 
  
 14
 
 100258973
 
 100259352
 
  ENSEMBL   UCSC 
 
  380
 
  6
 
 12573
 
 tss;island
 
 0.12722
 
 0.21968
 
 Not queried
 
  0.7367583
 
       NA
 
       NA
  
 
  
 2
 
  98280362
 
  98280854
 
  ENSEMBL   UCSC 
 
  493
 
 12
 
 24512
 
 island
 
 0.01899
 
 0.02749
 
 Not queried
 
  0.7366480
 
       NA
 
       NA
  
 
  
 3
 
  14597418
 
  14597729
 
  ENSEMBL   UCSC 
 
  312
 
  4
 
 28870
 
 island
 
 0.84051
 
 0.83443
 
 Not queried
 
  0.7365777
 
       NA
 
       NA
  
 
  
 1
 
  91869782
 
  91870067
 
  ENSEMBL   UCSC 
 
  286
 
  4
 
 628
 
 gene;island
 
 0.02578
 
 0.03374
 
 Not queried
 
  0.7365674
 
       NA
 
       NA
  
 
  
 6
 
  41007450
 
  41007692
 
  ENSEMBL   UCSC 
 
  243
 
  4
 
 34898
 
 tss
 
 0.78997
 
 0.90778
 
 Not queried
 
  0.7363142
 
       NA
 
       NA
  
 
  
 8
 
  79577796
 
  79578360
 
  ENSEMBL   UCSC 
 
  565
 
 10
 
 40342
 
 island
 
 0.02505
 
 0.02651
 
 Not queried
 
  0.7362706
 
       NA
 
       NA
  
 
  
 11
 
 107798913
 
 107799287
 
  ENSEMBL   UCSC 
 
  375
 
 10
 
 8753
 
 island
 
 0.02548
 
 0.05759
 
 Not queried
 
  0.7362172
 
       NA
 
       NA
  
 
  
 6
 
  31466722
 
  31466859
 
  ENSEMBL   UCSC 
 
  138
 
  5
 
 33817
 
 gene;island
 
 0.07503
 
 0.32175
 
 Not queried
 
  0.7361453
 
       NA
 
       NA
  
 
  
 6
 
  31545252
 
  31545473
 
  ENSEMBL   UCSC 
 
  222
 
  5
 
 33840
 
 gene;island
 
 0.82653
 
 0.77474
 
 Not queried
 
  0.7358412
 
       NA
 
       NA
  
 
  
 22
 
  43583726
 
  43583855
 
  ENSEMBL   UCSC 
 
  130
 
  4
 
 27161
 
 tss;island
 
 0.02220
 
 0.01731
 
 Not queried
 
  0.7358360
 
       NA
 
       NA
  
 
  
 X
 
   9432900
 
   9433105
 
  ENSEMBL   UCSC 
 
  206
 
  6
 
 41178
 
 tss
 
 0.01624
 
 0.03182
 
 Not queried
 
  0.7358144
 
       NA
 
       NA
  
 
  
 1
 
   9711632
 
   9711663
 
  ENSEMBL   UCSC 
 
   32
 
  4
 
 1226
 
 tss;island
 
 0.02521
 
 0.01851
 
 Not queried
 
  0.7357551
 
       NA
 
       NA
  
 
  
 11
 
  62477052
 
  62477624
 
  ENSEMBL   UCSC 
 
  573
 
  9
 
 7340
 
 tss
 
 0.02562
 
 0.05612
 
 Not queried
 
  0.7356465
 
       NA
 
       NA
  
 
  
 7
 
  73133982
 
  73134111
 
  ENSEMBL   UCSC 
 
  130
 
  6
 
 38502
 
 island
 
 0.02531
 
 0.07336
 
 Not queried
 
  0.7355101
 
       NA
 
       NA
  
 
  
 20
 
  21686308
 
  21686728
 
  ENSEMBL   UCSC 
 
  421
 
  4
 
 25336
 
 gene
 
 0.02196
 
 0.03517
 
 Not queried
 
  0.7354627
 
       NA
 
       NA
  
 
  
 19
 
  55996329
 
  55996566
 
  ENSEMBL   UCSC 
 
  238
 
  7
 
 21046
 
 tss
 
 0.03445
 
 0.03929
 
 Not queried
 
  0.7353176
 
       NA
 
       NA
  
 
  
 14
 
 102227915
 
 102227943
 
  ENSEMBL   UCSC 
 
   29
 
  4
 
 12609
 
 tss;island
 
 0.11461
 
 0.20577
 
 Not queried
 
  0.7352558
 
       NA
 
       NA
  
 
  
 13
 
  60970210
 
  60970705
 
  ENSEMBL   UCSC 
 
  496
 
  8
 
 11440
 
 tss;island
 
 0.02769
 
 0.02719
 
 Not queried
 
  0.7352326
 
       NA
 
       NA
  
 
  
 7
 
  72299837
 
  72300134
 
  ENSEMBL   UCSC 
 
  298
 
  6
 
 37564
 
 tss;island
 
 0.01134
 
 0.01839
 
 Not queried
 
  0.7351987
 
       NA
 
       NA
  
 
  
 11
 
  22851383
 
  22851591
 
  ENSEMBL   UCSC 
 
  209
 
  7
 
 7184
 
 tss
 
 0.02889
 
 0.04112
 
 Not queried
 
  0.7351550
 
       NA
 
       NA
  
 
  
 X
 
  72666981
 
  72667379
 
  ENSEMBL   UCSC 
 
  399
 
  7
 
 41871
 
 island
 
 0.63351
 
 0.41616
 
 Not queried
 
  0.7349409
 
       NA
 
       NA
  
 
  
 16
 
  67926607
 
  67927351
 
  ENSEMBL   UCSC 
 
  745
 
  7
 
 16001
 
 island
 
 0.00756
 
 0.02262
 
 Not queried
 
  0.7349321
 
       NA
 
       NA
  
 
  
 11
 
  14666436
 
  14666839
 
  ENSEMBL   UCSC 
 
  404
 
  4
 
 6500
 
 gene;island
 
 0.02122
 
 0.01550
 
 Not queried
 
  0.7348932
 
       NA
 
       NA
  
 
  
 7
 
  73184617
 
  73184671
 
  ENSEMBL   UCSC 
 
   55
 
  6
 
 37576
 
 tss
 
 0.01367
 
 0.02262
 
 Not queried
 
  0.7347068
 
       NA
 
       NA
  
 
  
 5
 
 177026865
 
 177026993
 
  ENSEMBL   UCSC 
 
  129
 
  4
 
 32475
 
 tss
 
 0.03261
 
 0.04632
 
 Not queried
 
  0.7346344
 
       NA
 
       NA
  
 
  
 2
 
 232063073
 
 232063251
 
  ENSEMBL   UCSC 
 
  179
 
  6
 
 23900
 
 tss
 
 0.01887
 
 0.03374
 
 Not queried
 
  0.7346185
 
       NA
 
       NA
  
 
  
 15
 
  69325271
 
  69325560
 
  ENSEMBL   UCSC 
 
  290
 
  5
 
 13369
 
 gene;island
 
 0.82856
 
 0.67804
 
 Not queried
 
  0.7345508
 
       NA
 
       NA
  
 
  
 3
 
 108836693
 
 108836912
 
  ENSEMBL   UCSC 
 
  220
 
  4
 
 27847
 
 gene
 
 0.94819
 
 0.91082
 
 Not queried
 
  0.7344293
 
       NA
 
       NA
  
 
  
 3
 
  27410970
 
  27411065
 
  ENSEMBL   UCSC 
 
   96
 
  4
 
 28153
 
 tss
 
 0.00675
 
 0.01137
 
 Not queried
 
  0.7344156
 
       NA
 
       NA
  
 
  
 1
 
  27693341
 
  27693474
 
  ENSEMBL   UCSC 
 
  134
 
  4
 
 1405
 
 tss
 
 0.02169
 
 0.04152
 
 Not queried
 
  0.7343653
 
       NA
 
       NA
  
 
  
 15
 
  90234067
 
  90234232
 
  ENSEMBL   UCSC 
 
  166
 
  8
 
 13445
 
 gene
 
 0.02504
 
 0.03269
 
 Not queried
 
  0.7342972
 
       NA
 
       NA
  
 
  
 8
 
 145641882
 
 145642243
 
  ENSEMBL   UCSC 
 
  362
 
  7
 
 39458
 
 gene
 
 0.68509
 
 0.80401
 
 Not queried
 
  0.7342656
 
       NA
 
       NA
  
 
  
 X
 
  47077815
 
  47077986
 
  ENSEMBL   UCSC 
 
  172
 
  7
 
 40975
 
 gene;tss;island
 
 0.01204
 
 0.02936
 
 Not queried
 
  0.7340944
 
       NA
 
       NA
  
 
  
 2
 
 119532041
 
 119532574
 
  ENSEMBL   UCSC 
 
  534
 
  7
 
 24605
 
 island
 
 0.01372
 
 0.01571
 
 Not queried
 
  0.7340868
 
       NA
 
       NA
  
 
  
 3
 
  14219850
 
  14220525
 
  ENSEMBL   UCSC 
 
  676
 
 17
 
 28127
 
 tss;island
 
 0.01681
 
 0.01806
 
 Not queried
 
  0.7339955
 
       NA
 
       NA
  
 
  
 7
 
 134832770
 
 134833299
 
  ENSEMBL   UCSC 
 
  530
 
  7
 
 37172
 
 gene
 
 0.71153
 
 0.83329
 
 Not queried
 
  0.7339288
 
       NA
 
       NA
  
 
  
 6
 
  33143721
 
  33143807
 
  ENSEMBL   UCSC 
 
   87
 
  4
 
 34139
 
 gene
 
 0.20234
 
 0.27254
 
 Not queried
 
  0.7339096
 
       NA
 
       NA
  
 
  
 3
 
   9690995
 
   9691379
 
  ENSEMBL   UCSC 
 
  385
 
 10
 
 28815
 
 island
 
 0.02000
 
 0.03367
 
 Not queried
 
  0.7338432
 
       NA
 
       NA
  
 
  
 1
 
  10732290
 
  10733030
 
  ENSEMBL   UCSC 
 
  741
 
 11
 
 215
 
 gene
 
 0.83729
 
 0.92774
 
 Not queried
 
  0.7337937
 
       NA
 
       NA
  
 
  
 14
 
  53173926
 
  53174235
 
  ENSEMBL   UCSC 
 
  310
 
  4
 
 12053
 
 gene
 
 0.02569
 
 0.01339
 
 Not queried
 
  0.7337890
 
       NA
 
       NA
  
 
  
 7
 
 156931759
 
 156932163
 
  ENSEMBL   UCSC 
 
  405
 
  5
 
 37255
 
 gene;island
 
 0.03461
 
 0.02390
 
 Not queried
 
  0.7335376
 
       NA
 
       NA
  
 
  
 16
 
  55512806
 
  55513191
 
  ENSEMBL   UCSC 
 
  386
 
  7
 
 15902
 
 island
 
 0.01743
 
 0.02773
 
 Not queried
 
  0.7334227
 
       NA
 
       NA
  
 
  
 8
 
 143532613
 
 143532922
 
  ENSEMBL   UCSC 
 
  310
 
  4
 
 40568
 
 island
 
 0.05141
 
 0.02553
 
 Not queried
 
  0.7332441
 
       NA
 
       NA
  
 
  
 3
 
 191046380
 
 191046872
 
  ENSEMBL   UCSC 
 
  493
 
  5
 
 28745
 
 tss
 
 0.07694
 
 0.06411
 
 Not queried
 
  0.7331451
 
       NA
 
       NA
  
 
  
 16
 
   1131266
 
   1132015
 
  ENSEMBL   UCSC 
 
  750
 
  8
 
 15483
 
 island
 
 0.67342
 
 0.81631
 
 Not queried
 
  0.7331444
 
       NA
 
       NA
  
 
  
 1
 
  24104423
 
  24104798
 
  ENSEMBL   UCSC 
 
  376
 
  7
 
 1342
 
 tss;island
 
 0.01532
 
 0.02655
 
 Not queried
 
  0.7330738
 
       NA
 
       NA
  
 
  
 2
 
  23913769
 
  23914666
 
  ENSEMBL   UCSC 
 
  898
 
  8
 
 22560
 
 gene;island
 
 0.90779
 
 0.94049
 
 Not queried
 
  0.7329129
 
       NA
 
       NA
  
 
  
 10
 
  23983496
 
  23983686
 
  ENSEMBL   UCSC 
 
  191
 
  5
 
 5594
 
 island
 
 0.07328
 
 0.03995
 
 Not queried
 
  0.7327430
 
       NA
 
       NA
  
 
  
 20
 
  37434158
 
  37434552
 
  ENSEMBL   UCSC 
 
  395
 
  8
 
 26121
 
 island
 
 0.02912
 
 0.02815
 
 Not queried
 
  0.7327368
 
       NA
 
       NA
  
 
  
 6
 
 110501281
 
 110501372
 
  ENSEMBL   UCSC 
 
   92
 
  5
 
 35103
 
 tss;island
 
 0.02155
 
 0.02421
 
 Not queried
 
  0.7324967
 
       NA
 
       NA
  
 
  
 1
 
 156211409
 
 156211896
 
  ENSEMBL   UCSC 
 
  488
 
 10
 
 2007
 
 tss
 
 0.66710
 
 0.81344
 
 Not queried
 
  0.7324565
 
       NA
 
       NA
  
 
  
 7
 
  75931606
 
  75931843
 
  ENSEMBL   UCSC 
 
  238
 
  8
 
 37597
 
 tss
 
 0.09951
 
 0.05169
 
 Not queried
 
  0.7323103
 
       NA
 
       NA
  
 
  
 16
 
  31214307
 
  31214607
 
  ENSEMBL   UCSC 
 
  301
 
  8
 
 15178
 
 tss
 
 0.59466
 
 0.37890
 
 Not queried
 
  0.7322238
 
       NA
 
       NA
  
 
  
 14
 
  94759646
 
  94759873
 
  ENSEMBL   UCSC 
 
  228
 
  4
 
 12555
 
 tss
 
 0.44449
 
 0.62705
 
 Not queried
 
  0.7321082
 
       NA
 
       NA
  
 
  
 13
 
  26623620
 
  26624039
 
  ENSEMBL   UCSC 
 
  420
 
  4
 
 11083
 
 gene;island
 
 0.03477
 
 0.06259
 
 Not queried
 
  0.7320653
 
       NA
 
       NA
  
 
  
 10
 
 135160697
 
 135160792
 
  ENSEMBL   UCSC 
 
   96
 
  5
 
 5444
 
 tss
 
 0.70701
 
 0.63324
 
 Not queried
 
  0.7320615
 
       NA
 
       NA
  
 
  
 3
 
 183852688
 
 183853494
 
  ENSEMBL   UCSC 
 
  807
 
 12
 
 29668
 
 island
 
 0.02079
 
 0.02427
 
 Not queried
 
  0.7320278
 
       NA
 
       NA
  
 
  
 1
 
 204951031
 
 204951336
 
  ENSEMBL   UCSC 
 
  306
 
  4
 
 958
 
 gene;island
 
 0.87729
 
 0.74969
 
 Not queried
 
  0.7320252
 
       NA
 
       NA
  
 
  
 7
 
   1534766
 
   1535141
 
  ENSEMBL   UCSC 
 
  376
 
  5
 
 36695
 
 gene;island
 
 0.85139
 
 0.85969
 
 Not queried
 
  0.7318051
 
       NA
 
       NA
  
 
  
 9
 
 140115806
 
 140116137
 
  ENSEMBL   UCSC 
 
  332
 
  4
 
 40793
 
 tss;island
 
 0.58592
 
 0.42969
 
 Not queried
 
  0.7317751
 
       NA
 
       NA
  
 
  
 13
 
  20437712
 
  20437969
 
  ENSEMBL   UCSC 
 
  258
 
  5
 
 11535
 
 island
 
 0.02583
 
 0.02987
 
 Not queried
 
  0.7316483
 
       NA
 
       NA
  
 
  
 10
 
 112432175
 
 112432418
 
  ENSEMBL   UCSC 
 
  244
 
  4
 
 4741
 
 gene;island
 
 0.03651
 
 0.04234
 
 Not queried
 
  0.7316171
 
       NA
 
       NA
  
 
  
 2
 
 176994142
 
 176994764
 
  ENSEMBL   UCSC 
 
  623
 
  9
 
 24866
 
 island
 
 0.01485
 
 0.02209
 
 Not queried
 
  0.7315526
 
       NA
 
       NA
  
 
  
 3
 
  16646904
 
  16647103
 
  ENSEMBL   UCSC 
 
  200
 
  5
 
 28882
 
 island
 
 0.93831
 
 0.90275
 
 Not queried
 
  0.7314723
 
       NA
 
       NA
  
 
  
 16
 
   1060367
 
   1060765
 
  ENSEMBL   UCSC 
 
  399
 
  5
 
 15475
 
 island
 
 0.77078
 
 0.85113
 
 Not queried
 
  0.7313282
 
       NA
 
       NA
  
 
  
 2
 
  97760606
 
  97760749
 
  ENSEMBL   UCSC 
 
  144
 
  7
 
 23489
 
 tss;island
 
 0.03291
 
 0.01937
 
 Not queried
 
  0.7313090
 
       NA
 
       NA
  
 
  
 18
 
  11821776
 
  11822136
 
  ENSEMBL   UCSC 
 
  361
 
  5
 
 19023
 
 gene;island
 
 0.91720
 
 0.89537
 
 Not queried
 
  0.7309862
 
       NA
 
       NA
  
 
  
 1
 
 240986512
 
 240986898
 
  ENSEMBL   UCSC 
 
  387
 
  5
 
 1105
 
 gene;island
 
 0.88719
 
 0.82574
 
 Not queried
 
  0.7309823
 
       NA
 
       NA
  
 
  
 19
 
  58514497
 
  58514839
 
  ENSEMBL   UCSC 
 
  343
 
  4
 
 22452
 
 island
 
 0.15572
 
 0.12601
 
 Not queried
 
  0.7309565
 
       NA
 
       NA
  
 
  
 6
 
  32178017
 
  32178298
 
  ENSEMBL   UCSC 
 
  282
 
  5
 
 34028
 
 gene
 
 0.76930
 
 0.66768
 
 Not queried
 
  0.7309307
 
       NA
 
       NA
  
 
  
 2
 
 174219209
 
 174219534
 
  ENSEMBL   UCSC 
 
  326
 
  5
 
 23684
 
 tss;island
 
 0.02005
 
 0.01105
 
 Not queried
 
  0.7309069
 
       NA
 
       NA
  
 
  
 12
 
  53661747
 
  53662079
 
  ENSEMBL   UCSC 
 
  333
 
  5
 
 9711
 
 tss;island
 
 0.01725
 
 0.03047
 
 Not queried
 
  0.7309000
 
       NA
 
       NA
  
 
  
 17
 
   4710015
 
   4710044
 
  ENSEMBL   UCSC 
 
   30
 
  4
 
 16999
 
 tss;island
 
 0.03061
 
 0.04237
 
 Not queried
 
  0.7308389
 
       NA
 
       NA
  
 
  
 4
 
  87515467
 
  87515700
 
  ENSEMBL   UCSC 
 
  234
 
  4
 
 30004
 
 gene
 
 0.02688
 
 0.02564
 
 Not queried
 
  0.7307853
 
       NA
 
       NA
  
 
  
 10
 
  71906310
 
  71906761
 
  ENSEMBL   UCSC 
 
  452
 
 10
 
 5794
 
 island
 
 0.01923
 
 0.01526
 
 Not queried
 
  0.7307032
 
       NA
 
       NA
  
 
  
 22
 
  38082108
 
  38082349
 
  ENSEMBL   UCSC 
 
  242
 
  7
 
 27428
 
 island
 
 0.02164
 
 0.05176
 
 Not queried
 
  0.7305223
 
       NA
 
       NA
  
 
  
 12
 
   2788627
 
   2788847
 
  ENSEMBL   UCSC 
 
  221
 
  5
 
 8981
 
 gene;island
 
 0.94281
 
 0.92886
 
 Not queried
 
  0.7305028
 
       NA
 
       NA
  
 
  
 16
 
  67450370
 
  67450810
 
  ENSEMBL   UCSC 
 
  441
 
  6
 
 15271
 
 tss;island
 
 0.08164
 
 0.12318
 
 Not queried
 
  0.7303274
 
       NA
 
       NA
  
 
  
 17
 
  37322330
 
  37322730
 
  ENSEMBL   UCSC 
 
  401
 
  4
 
 18234
 
 island
 
 0.51282
 
 0.42781
 
 Not queried
 
  0.7300801
 
       NA
 
       NA
  
 
  
 20
 
  52790589
 
  52790733
 
  ENSEMBL   UCSC 
 
  145
 
  4
 
 25790
 
 tss;island
 
 0.02338
 
 0.04061
 
 Not queried
 
  0.7300379
 
       NA
 
       NA
  
 
  
 17
 
  37793426
 
  37793717
 
  ENSEMBL   UCSC 
 
  292
 
  5
 
 16515
 
 gene
 
 0.02839
 
 0.02863
 
 Not queried
 
  0.7300265
 
       NA
 
       NA
  
 
  
 17
 
  37793028
 
  37793717
 
  ENSEMBL   UCSC 
 
  690
 
 13
 
 18249
 
 island
 
 0.02839
 
 0.04634
 
 Not queried
 
  0.7300265
 
       NA
 
       NA
  
 
  
 9
 
 131799192
 
 131799655
 
  ENSEMBL   UCSC 
 
  464
 
  5
 
 40890
 
 island
 
 0.13335
 
 0.20019
 
 Not queried
 
  0.7300076
 
       NA
 
       NA
  
 
  
 19
 
  12551876
 
  12552133
 
  ENSEMBL   UCSC 
 
  258
 
  5
 
 21566
 
 island
 
 0.02792
 
 0.04061
 
 Not queried
 
  0.7299632
 
       NA
 
       NA
  
 
  
 6
 
 127441060
 
 127441199
 
  ENSEMBL   UCSC 
 
  140
 
  4
 
 34404
 
 gene;island
 
 0.02380
 
 0.04335
 
 Not queried
 
  0.7299502
 
       NA
 
       NA
  
 
  
 5
 
 154237370
 
 154237720
 
  ENSEMBL   UCSC 
 
  351
 
  6
 
 32392
 
 tss;island
 
 0.01153
 
 0.02419
 
 Not queried
 
  0.7298744
 
       NA
 
       NA
  
 
  
 10
 
  16859453
 
  16859644
 
  ENSEMBL   UCSC 
 
  192
 
  7
 
 4951
 
 tss
 
 0.01303
 
 0.01499
 
 Not queried
 
  0.7298304
 
       NA
 
       NA
  
 
  
 11
 
 123986111
 
 123986166
 
  ENSEMBL   UCSC 
 
   56
 
  4
 
 6932
 
 gene
 
 0.02461
 
 0.02059
 
 Not queried
 
  0.7298118
 
       NA
 
       NA
  
 
  
 2
 
  27712555
 
  27713092
 
  ENSEMBL   UCSC 
 
  538
 
 11
 
 24149
 
 island
 
 0.03264
 
 0.01994
 
 Not queried
 
  0.7296078
 
       NA
 
       NA
  
 
  
 11
 
  60691582
 
  60692078
 
  ENSEMBL   UCSC 
 
  497
 
  8
 
 8295
 
 island
 
 0.04064
 
 0.05986
 
 Not queried
 
  0.7295247
 
       NA
 
       NA
  
 
  
 17
 
    618140
 
    618545
 
  ENSEMBL   UCSC 
 
  406
 
  7
 
 16947
 
 tss
 
 0.01095
 
 0.01965
 
 Not queried
 
  0.7294815
 
       NA
 
       NA
  
 
  
 16
 
  67143780
 
  67143802
 
  ENSEMBL   UCSC 
 
   23
 
  4
 
 15260
 
 tss;island
 
 0.05707
 
 0.12257
 
 Not queried
 
  0.7294157
 
       NA
 
       NA
  
 
  
 18
 
  42259977
 
  42260234
 
  ENSEMBL   UCSC 
 
  258
 
  7
 
 19216
 
 tss;island
 
 0.03158
 
 0.05103
 
 Not queried
 
  0.7294058
 
       NA
 
       NA
  
 
  
 17
 
  48183258
 
  48183477
 
  ENSEMBL   UCSC 
 
  220
 
  5
 
 16645
 
 gene;island
 
 0.84163
 
 0.62551
 
 Not queried
 
  0.7293054
 
       NA
 
       NA
  
 
  
 5
 
  56204834
 
  56205359
 
  ENSEMBL   UCSC 
 
  526
 
  9
 
 32748
 
 island
 
 0.01498
 
 0.02106
 
 Not queried
 
  0.7288528
 
       NA
 
       NA
  
 
  
 7
 
  56031952
 
  56032122
 
  ENSEMBL   UCSC 
 
  171
 
  6
 
 37541
 
 tss;island
 
 0.02705
 
 0.04704
 
 Not queried
 
  0.7287118
 
       NA
 
       NA
  
 
  
 15
 
  27019248
 
  27019448
 
  ENSEMBL   UCSC 
 
  201
 
  4
 
 13512
 
 tss;island
 
 0.14426
 
 0.05678
 
 Not queried
 
  0.7285299
 
       NA
 
       NA
  
 
  
 16
 
   4588414
 
   4588809
 
  ENSEMBL   UCSC 
 
  396
 
  4
 
 14612
 
 gene
 
 0.02027
 
 0.01054
 
 Not queried
 
  0.7284999
 
       NA
 
       NA
  
 
  
 17
 
  56064295
 
  56064928
 
  ENSEMBL   UCSC 
 
  634
 
  7
 
 16671
 
 gene;island
 
 0.01275
 
 0.03077
 
 Not queried
 
  0.7283800
 
       NA
 
       NA
  
 
  
 19
 
   3036221
 
   3036484
 
  ENSEMBL   UCSC 
 
  264
 
  4
 
 19668
 
 gene;island
 
 0.02612
 
 0.03640
 
 Not queried
 
  0.7283672
 
       NA
 
       NA
  
 
  
 7
 
  19147041
 
  19147487
 
  ENSEMBL   UCSC 
 
  447
 
  5
 
 38218
 
 island
 
 0.02883
 
 0.02570
 
 Not queried
 
  0.7283536
 
       NA
 
       NA
  
 
  
 6
 
 152126736
 
 152126938
 
  ENSEMBL   UCSC 
 
  203
 
  5
 
 34447
 
 gene;island
 
 0.46149
 
 0.44877
 
 Not queried
 
  0.7281939
 
       NA
 
       NA
  
 
  
 6
 
  22569543
 
  22569665
 
  ENSEMBL   UCSC 
 
  123
 
  6
 
 34600
 
 tss
 
 0.86510
 
 0.90717
 
 Not queried
 
  0.7281346
 
       NA
 
       NA
  
 
  
 12
 
 123464511
 
 123464932
 
  ENSEMBL   UCSC 
 
  422
 
  6
 
 10912
 
 island
 
 0.07376
 
 0.03910
 
 Not queried
 
  0.7280579
 
       NA
 
       NA
  
 
  
 11
 
  68781976
 
  68782211
 
  ENSEMBL   UCSC 
 
  236
 
  5
 
 7495
 
 tss;island
 
 0.69960
 
 0.73613
 
 Not queried
 
  0.7280275
 
       NA
 
       NA
  
 
  
 22
 
  39096475
 
  39096760
 
  ENSEMBL   UCSC 
 
  286
 
  8
 
 27102
 
 tss
 
 0.04924
 
 0.05161
 
 Not queried
 
  0.7279842
 
       NA
 
       NA
  
 
  
 11
 
 133402497
 
 133402589
 
  ENSEMBL   UCSC 
 
   93
 
  4
 
 7803
 
 tss
 
 0.55680
 
 0.39579
 
 Not queried
 
  0.7277466
 
       NA
 
       NA
  
 
  
 6
 
  43276570
 
  43276781
 
  ENSEMBL   UCSC 
 
  212
 
  8
 
 34932
 
 tss
 
 0.02135
 
 0.02073
 
 Not queried
 
  0.7276815
 
       NA
 
       NA
  
 
  
 15
 
  49255364
 
  49255563
 
  ENSEMBL   UCSC 
 
  200
 
  4
 
 13310
 
 gene
 
 0.04197
 
 0.05735
 
 Not queried
 
  0.7276636
 
       NA
 
       NA
  
 
  
 10
 
 116581112
 
 116581358
 
  ENSEMBL   UCSC 
 
  247
 
  6
 
 5361
 
 tss;island
 
 0.04983
 
 0.04351
 
 Not queried
 
  0.7275892
 
       NA
 
       NA
  
 
  
 7
 
  42276814
 
  42277071
 
  ENSEMBL   UCSC 
 
  258
 
 11
 
 37485
 
 tss;island
 
 0.02068
 
 0.02730
 
 Not queried
 
  0.7275016
 
       NA
 
       NA
  
 
  
 22
 
  38794898
 
  38795061
 
  ENSEMBL   UCSC 
 
  164
 
  8
 
 27445
 
 island
 
 0.02500
 
 0.02118
 
 Not queried
 
  0.7274800
 
       NA
 
       NA
  
 
  
 5
 
  43515194
 
  43515527
 
  ENSEMBL   UCSC 
 
  334
 
  5
 
 31989
 
 tss
 
 0.00948
 
 0.01560
 
 Not queried
 
  0.7273458
 
       NA
 
       NA
  
 
  
 15
 
  52821290
 
  52821541
 
  ENSEMBL   UCSC 
 
  252
 
  6
 
 13652
 
 tss
 
 0.02256
 
 0.03770
 
 Not queried
 
  0.7272702
 
       NA
 
       NA
  
 
  
 17
 
  47074576
 
  47074713
 
  ENSEMBL   UCSC 
 
  138
 
  7
 
 17447
 
 tss
 
 0.02031
 
 0.02380
 
 Not queried
 
  0.7271730
 
       NA
 
       NA
  
 
  
 19
 
  31841161
 
  31841663
 
  ENSEMBL   UCSC 
 
  503
 
  6
 
 20638
 
 tss;island
 
 0.08120
 
 0.10477
 
 Not queried
 
  0.7269981
 
       NA
 
       NA
  
 
  
 2
 
 128433058
 
 128433507
 
  ENSEMBL   UCSC 
 
  450
 
  6
 
 22826
 
 gene;tss;island
 
 0.02255
 
 0.03200
 
 Not queried
 
  0.7269493
 
       NA
 
       NA
  
 
  
 3
 
 172468745
 
 172469117
 
  ENSEMBL   UCSC 
 
  373
 
  5
 
 29625
 
 island
 
 0.03028
 
 0.11344
 
 Not queried
 
  0.7268388
 
       NA
 
       NA
  
 
  
 10
 
 133949683
 
 133950036
 
  ENSEMBL   UCSC 
 
  354
 
  4
 
 4835
 
 gene;island
 
 0.58613
 
 0.70583
 
 Not queried
 
  0.7268329
 
       NA
 
       NA
  
 
  
 17
 
  19617084
 
  19617395
 
  ENSEMBL   UCSC 
 
  312
 
  4
 
 16446
 
 gene;island
 
 0.86201
 
 0.83900
 
 Not queried
 
  0.7268267
 
       NA
 
       NA
  
 
  
 7
 
 158649018
 
 158649327
 
  ENSEMBL   UCSC 
 
  310
 
  9
 
 39092
 
 island
 
 0.01656
 
 0.03384
 
 Not queried
 
  0.7267710
 
       NA
 
       NA
  
 
  
 19
 
   2051251
 
   2051277
 
  ENSEMBL   UCSC 
 
   27
 
  4
 
 20280
 
 tss;island
 
 0.03272
 
 0.04159
 
 Not queried
 
  0.7267246
 
       NA
 
       NA
  
 
  
 3
 
 190040223
 
 190040375
 
  ENSEMBL   UCSC 
 
  153
 
  4
 
 28741
 
 tss
 
 0.01685
 
 0.02955
 
 Not queried
 
  0.7266895
 
       NA
 
       NA
  
 
  
 6
 
  34524095
 
  34524290
 
  ENSEMBL   UCSC 
 
  196
 
  4
 
 34856
 
 tss
 
 0.67993
 
 0.79252
 
 Not queried
 
  0.7266639
 
       NA
 
       NA
  
 
  
 16
 
  31519317
 
  31519685
 
  ENSEMBL   UCSC 
 
  369
 
  4
 
 14709
 
 gene
 
 0.01367
 
 0.01907
 
 Not queried
 
  0.7266439
 
       NA
 
       NA
  
 
  
 2
 
  55746547
 
  55746998
 
  ENSEMBL   UCSC 
 
  452
 
  8
 
 24272
 
 island
 
 0.01465
 
 0.01946
 
 Not queried
 
  0.7266190
 
       NA
 
       NA
  
 
  
 17
 
    157043
 
    157219
 
  ENSEMBL   UCSC 
 
  177
 
  4
 
 16253
 
 gene;island
 
 0.88418
 
 0.84332
 
 Not queried
 
  0.7265652
 
       NA
 
       NA
  
 
  
 7
 
 150652702
 
 150653195
 
  ENSEMBL   UCSC 
 
  494
 
  8
 
 37216
 
 gene;island
 
 0.79478
 
 0.77054
 
 Not queried
 
  0.7264951
 
       NA
 
       NA
  
 
  
 19
 
   1028379
 
   1028780
 
  ENSEMBL   UCSC 
 
  402
 
  4
 
 19605
 
 gene;island
 
 0.09690
 
 0.13230
 
 Not queried
 
  0.7264341
 
       NA
 
       NA
  
 
  
 X
 
 139173266
 
 139173584
 
  ENSEMBL   UCSC 
 
  319
 
  4
 
 42076
 
 island
 
 0.01879
 
 0.03655
 
 Not queried
 
  0.7264268
 
       NA
 
       NA
  
 
  
 12
 
 133066471
 
 133066762
 
  ENSEMBL   UCSC 
 
  292
 
 14
 
 10105
 
 tss;island
 
 0.03147
 
 0.03563
 
 Not queried
 
  0.7264028
 
       NA
 
       NA
  
 
  
 9
 
 136243334
 
 136243698
 
  ENSEMBL   UCSC 
 
  365
 
  4
 
 40726
 
 gene
 
 0.27691
 
 0.14264
 
 Not queried
 
  0.7263668
 
       NA
 
       NA
  
 
  
 19
 
  46296257
 
  46296285
 
  ENSEMBL   UCSC 
 
   29
 
  4
 
 20844
 
 tss;island
 
 0.01277
 
 0.01398
 
 Not queried
 
  0.7262613
 
       NA
 
       NA
  
 
  
 22
 
  33196328
 
  33196627
 
  ENSEMBL   UCSC 
 
  300
 
  5
 
 26809
 
 gene;tss;island
 
 0.81030
 
 0.91697
 
 Not queried
 
  0.7262602
 
       NA
 
       NA
  
 
  
 16
 
  88262974
 
  88263263
 
  ENSEMBL   UCSC 
 
  290
 
  4
 
 16161
 
 island
 
 0.77189
 
 0.83014
 
 Not queried
 
  0.7259562
 
       NA
 
       NA
  
 
  
 5
 
  89854632
 
  89854979
 
  ENSEMBL   UCSC 
 
  348
 
  5
 
 31630
 
 gene
 
 0.02832
 
 0.00972
 
 Not queried
 
  0.7258949
 
       NA
 
       NA
  
 
  
 11
 
  68816030
 
  68816274
 
  ENSEMBL   UCSC 
 
  245
 
  6
 
 7496
 
 tss
 
 0.05324
 
 0.12651
 
 Not queried
 
  0.7258742
 
       NA
 
       NA
  
 
  
 10
 
    685962
 
    686354
 
  ENSEMBL   UCSC 
 
  393
 
  4
 
 4435
 
 gene;island
 
 0.92730
 
 0.86457
 
 Not queried
 
  0.7258348
 
       NA
 
       NA
  
 
  
 15
 
  76603985
 
  76604113
 
  ENSEMBL   UCSC 
 
  129
 
  5
 
 13794
 
 tss
 
 0.04495
 
 0.04824
 
 Not queried
 
  0.7257545
 
       NA
 
       NA
  
 
  
 6
 
   4135575
 
   4135762
 
  ENSEMBL   UCSC 
 
  188
 
  5
 
 33476
 
 gene
 
 0.00748
 
 0.01576
 
 Not queried
 
  0.7257423
 
       NA
 
       NA
  
 
  
 1
 
  28561995
 
  28562373
 
  ENSEMBL   UCSC 
 
  379
 
  5
 
 1422
 
 tss;island
 
 0.00830
 
 0.00544
 
 Not queried
 
  0.7257132
 
       NA
 
       NA
  
 
  
 17
 
   8055756
 
   8055888
 
  ENSEMBL   UCSC 
 
  133
 
  5
 
 17076
 
 tss
 
 0.03728
 
 0.06009
 
 Not queried
 
  0.7256638
 
       NA
 
       NA
  
 
  
 19
 
  38865020
 
  38865080
 
  ENSEMBL   UCSC 
 
   61
 
  6
 
 20717
 
 tss
 
 0.03277
 
 0.02766
 
 Not queried
 
  0.7255913
 
       NA
 
       NA
  
 
  
 13
 
 112724221
 
 112724583
 
  ENSEMBL   UCSC 
 
  363
 
  5
 
 11217
 
 gene;island
 
 0.33378
 
 0.34173
 
 Not queried
 
  0.7255556
 
       NA
 
       NA
  
 
  
 20
 
   4795836
 
   4796176
 
  ENSEMBL   UCSC 
 
  341
 
  7
 
 25312
 
 gene;tss
 
 0.84818
 
 0.93765
 
 Not queried
 
  0.7255552
 
       NA
 
       NA
  
 
  
 4
 
  84456674
 
  84457116
 
  ENSEMBL   UCSC 
 
  443
 
  4
 
 30430
 
 tss;island
 
 0.05377
 
 0.03851
 
 Not queried
 
  0.7255096
 
       NA
 
       NA
  
 
  
 2
 
    306606
 
    307058
 
  ENSEMBL   UCSC 
 
  453
 
  4
 
 23963
 
 island
 
 0.78494
 
 0.73829
 
 Not queried
 
  0.7253951
 
       NA
 
       NA
  
 
  
 1
 
 155247091
 
 155247785
 
  ENSEMBL   UCSC 
 
  695
 
 11
 
 3813
 
 island
 
 0.02817
 
 0.04309
 
 Not queried
 
  0.7253951
 
       NA
 
       NA
  
 
  
 10
 
 135186854
 
 135187068
 
  ENSEMBL   UCSC 
 
  215
 
  4
 
 6341
 
 island
 
 0.02755
 
 0.03509
 
 Not queried
 
  0.7251842
 
       NA
 
       NA
  
 
  
 21
 
  42733729
 
  42733894
 
  ENSEMBL   UCSC 
 
  166
 
  5
 
 26515
 
 tss
 
 0.74175
 
 0.61412
 
 Not queried
 
  0.7250557
 
       NA
 
       NA
  
 
  
 2
 
 220174201
 
 220174251
 
  ENSEMBL   UCSC 
 
   51
 
  4
 
 23866
 
 tss;island
 
 0.48911
 
 0.35295
 
 Not queried
 
  0.7250242
 
       NA
 
       NA
  
 
  
 5
 
  65440440
 
  65440543
 
  ENSEMBL   UCSC 
 
  104
 
  5
 
 31577
 
 gene;tss;island
 
 0.04222
 
 0.06763
 
 Not queried
 
  0.7249520
 
       NA
 
       NA
  
 
  
 X
 
  49056505
 
  49057013
 
  ENSEMBL   UCSC 
 
  509
 
 11
 
 41790
 
 island
 
 0.04881
 
 0.02540
 
 Not queried
 
  0.7249090
 
       NA
 
       NA
  
 
  
 19
 
  10397429
 
  10397642
 
  ENSEMBL   UCSC 
 
  214
 
  5
 
 20407
 
 tss
 
 0.20587
 
 0.09637
 
 Not queried
 
  0.7248936
 
       NA
 
       NA
  
 
  
 2
 
 118943676
 
 118944144
 
  ENSEMBL   UCSC 
 
  469
 
  5
 
 24604
 
 island
 
 0.01219
 
 0.02061
 
 Not queried
 
  0.7248923
 
       NA
 
       NA
  
 
  
 19
 
   7712095
 
   7712539
 
  ENSEMBL   UCSC 
 
  445
 
  4
 
 19749
 
 gene;island
 
 0.88174
 
 0.94047
 
 Not queried
 
  0.7248910
 
       NA
 
       NA
  
 
  
 8
 
 143545214
 
 143545574
 
  ENSEMBL   UCSC 
 
  361
 
  5
 
 40570
 
 island
 
 0.69929
 
 0.73719
 
 Not queried
 
  0.7248800
 
       NA
 
       NA
  
 
  
 6
 
  33756678
 
  33757077
 
  ENSEMBL   UCSC 
 
  400
 
  8
 
 35985
 
 island
 
 0.03076
 
 0.04259
 
 Not queried
 
  0.7248554
 
       NA
 
       NA
  
 
  
 20
 
  30225325
 
  30225685
 
  ENSEMBL   UCSC 
 
  361
 
  6
 
 25625
 
 tss
 
 0.79994
 
 0.89153
 
 Not queried
 
  0.7248163
 
       NA
 
       NA
  
 
  
 19
 
   6516369
 
   6517069
 
  ENSEMBL   UCSC 
 
  701
 
  6
 
 21398
 
 island
 
 0.05613
 
 0.07075
 
 Not queried
 
  0.7245586
 
       NA
 
       NA
  
 
  
 7
 
  10979847
 
  10980008
 
  ENSEMBL   UCSC 
 
  162
 
  7
 
 37375
 
 tss
 
 0.02256
 
 0.01377
 
 Not queried
 
  0.7245363
 
       NA
 
       NA
  
 
  
 19
 
  46438607
 
  46438944
 
  ENSEMBL   UCSC 
 
  338
 
  4
 
 22131
 
 island
 
 0.53405
 
 0.58668
 
 Not queried
 
  0.7244750
 
       NA
 
       NA
  
 
  
 21
 
  35747390
 
  35747882
 
  ENSEMBL   UCSC 
 
  493
 
  9
 
 26618
 
 island
 
 0.02914
 
 0.04899
 
 Not queried
 
  0.7244490
 
       NA
 
       NA
  
 
  
 1
 
 200004529
 
 200004757
 
  ENSEMBL   UCSC 
 
  229
 
  5
 
 930
 
 gene;island
 
 0.04505
 
 0.01990
 
 Not queried
 
  0.7243732
 
       NA
 
       NA
  
 
  
 18
 
  46477127
 
  46477404
 
  ENSEMBL   UCSC 
 
  278
 
  6
 
 19234
 
 tss
 
 0.03031
 
 0.08446
 
 Not queried
 
  0.7242394
 
       NA
 
       NA
  
 
  
 19
 
  11998457
 
  11999148
 
  ENSEMBL   UCSC 
 
  692
 
 14
 
 21550
 
 island
 
 0.02432
 
 0.03315
 
 Not queried
 
  0.7242205
 
       NA
 
       NA
  
 
  
 14
 
 102829091
 
 102829219
 
  ENSEMBL   UCSC 
 
  129
 
  5
 
 12203
 
 gene
 
 0.01383
 
 0.02765
 
 Not queried
 
  0.7241335
 
       NA
 
       NA
  
 
  
 10
 
 105678128
 
 105678266
 
  ENSEMBL   UCSC 
 
  139
 
  6
 
 5324
 
 tss
 
 0.02704
 
 0.04691
 
 Not queried
 
  0.7239304
 
       NA
 
       NA
  
 
  
 11
 
    207155
 
    207881
 
  ENSEMBL   UCSC 
 
  727
 
 11
 
 6983
 
 tss;island
 
 0.02274
 
 0.02716
 
 Not queried
 
  0.7239192
 
       NA
 
       NA
  
 
  
 12
 
  53273196
 
  53273497
 
  ENSEMBL   UCSC 
 
  302
 
  4
 
 10420
 
 island
 
 0.09594
 
 0.21243
 
 Not queried
 
  0.7237376
 
       NA
 
       NA
  
 
  
 16
 
   2932904
 
   2933096
 
  ENSEMBL   UCSC 
 
  193
 
  5
 
 15008
 
 tss;island
 
 0.03002
 
 0.03038
 
 Not queried
 
  0.7236983
 
       NA
 
       NA
  
 
  
 1
 
   6051551
 
   6051640
 
  ENSEMBL   UCSC 
 
   90
 
  4
 
 157
 
 gene;island
 
 0.02568
 
 0.03788
 
 Not queried
 
  0.7235913
 
       NA
 
       NA
  
 
  
 5
 
  65017248
 
  65017496
 
  ENSEMBL   UCSC 
 
  249
 
  4
 
 31575
 
 gene;tss;island
 
 0.01769
 
 0.03544
 
 Not queried
 
  0.7234729
 
       NA
 
       NA
  
 
  
 1
 
 144533778
 
 144533951
 
  ENSEMBL   UCSC 
 
  174
 
  4
 
 3661
 
 island
 
 0.73979
 
 0.61809
 
 Not queried
 
  0.7234530
 
       NA
 
       NA
  
 
  
 X
 
   6146608
 
   6146900
 
  ENSEMBL   UCSC 
 
  293
 
  6
 
 41172
 
 tss;island
 
 0.04998
 
 0.02345
 
 Not queried
 
  0.7233572
 
       NA
 
       NA
  
 
  
 16
 
   1359179
 
   1359454
 
  ENSEMBL   UCSC 
 
  276
 
  7
 
 14532
 
 gene
 
 0.01402
 
 0.02293
 
 Not queried
 
  0.7232794
 
       NA
 
       NA
  
 
  
 22
 
  30938383
 
  30938784
 
  ENSEMBL   UCSC 
 
  402
 
  4
 
 27371
 
 island
 
 0.43664
 
 0.54766
 
 Not queried
 
  0.7231467
 
       NA
 
       NA
  
 
  
 10
 
 102045829
 
 102046368
 
  ENSEMBL   UCSC 
 
  540
 
 12
 
 4699
 
 gene
 
 0.02942
 
 0.07013
 
 Not queried
 
  0.7231254
 
       NA
 
       NA
  
 
  
 14
 
  93170534
 
  93170970
 
  ENSEMBL   UCSC 
 
  437
 
  4
 
 12155
 
 gene
 
 0.85443
 
 0.94082
 
 Not queried
 
  0.7231159
 
       NA
 
       NA
  
 
  
 10
 
 135278717
 
 135279147
 
  ENSEMBL   UCSC 
 
  431
 
  5
 
 4890
 
 gene;island
 
 0.87777
 
 0.81313
 
 Not queried
 
  0.7228083
 
       NA
 
       NA
  
 
  
 7
 
  76609880
 
  76610059
 
  ENSEMBL   UCSC 
 
  180
 
  6
 
 37601
 
 tss
 
 0.83358
 
 0.87988
 
 Not queried
 
  0.7227838
 
       NA
 
       NA
  
 
  
 1
 
  46216513
 
  46216737
 
  ENSEMBL   UCSC 
 
  225
 
  4
 
 1588
 
 tss
 
 0.04170
 
 0.02639
 
 Not queried
 
  0.7227210
 
       NA
 
       NA
  
 
  
 19
 
   9420290
 
   9420631
 
  ENSEMBL   UCSC 
 
  342
 
  6
 
 21459
 
 island
 
 0.01092
 
 0.02105
 
 Not queried
 
  0.7226157
 
       NA
 
       NA
  
 
  
 9
 
 136203187
 
 136203387
 
  ENSEMBL   UCSC 
 
  201
 
  4
 
 40785
 
 tss;island
 
 0.05298
 
 0.07147
 
 Not queried
 
  0.7225792
 
       NA
 
       NA
  
 
  
 13
 
  27828900
 
  27829259
 
  ENSEMBL   UCSC 
 
  360
 
  5
 
 11087
 
 gene;tss;island
 
 0.87202
 
 0.93858
 
 Not queried
 
  0.7225684
 
       NA
 
       NA
  
 
  
 11
 
  46957758
 
  46958558
 
  ENSEMBL   UCSC 
 
  801
 
 12
 
 8235
 
 island
 
 0.02957
 
 0.05115
 
 Not queried
 
  0.7225346
 
       NA
 
       NA
  
 
  
 10
 
  85899064
 
  85899272
 
  ENSEMBL   UCSC 
 
  209
 
  8
 
 5874
 
 island
 
 0.01916
 
 0.02605
 
 Not queried
 
  0.7225183
 
       NA
 
       NA
  
 
  
 19
 
  53636226
 
  53636398
 
  ENSEMBL   UCSC 
 
  173
 
  5
 
 20987
 
 tss
 
 0.02104
 
 0.03375
 
 Not queried
 
  0.7224948
 
       NA
 
       NA
  
 
  
 8
 
 145536028
 
 145536348
 
  ENSEMBL   UCSC 
 
  321
 
  4
 
 39453
 
 gene
 
 0.81833
 
 0.88292
 
 Not queried
 
  0.7222459
 
       NA
 
       NA
  
 
  
 8
 
  20112813
 
  20113188
 
  ENSEMBL   UCSC 
 
  376
 
  7
 
 39513
 
 tss
 
 0.78009
 
 0.85867
 
 Not queried
 
  0.7221933
 
       NA
 
       NA
  
 
  
 6
 
  99395968
 
  99396091
 
  ENSEMBL   UCSC 
 
  124
 
  5
 
 35068
 
 tss;island
 
 0.01617
 
 0.02640
 
 Not queried
 
  0.7220347
 
       NA
 
       NA
  
 
  
 1
 
  27816678
 
  27816827
 
  ENSEMBL   UCSC 
 
  150
 
  5
 
 1409
 
 tss
 
 0.01187
 
 0.01937
 
 Not queried
 
  0.7219207
 
       NA
 
       NA
  
 
  
 2
 
 234215965
 
 234216155
 
  ENSEMBL   UCSC 
 
  191
 
  4
 
 23920
 
 tss
 
 0.90270
 
 0.87401
 
 Not queried
 
  0.7219162
 
       NA
 
       NA
  
 
  
 13
 
 110790284
 
 110790566
 
  ENSEMBL   UCSC 
 
  283
 
  5
 
 11845
 
 island
 
 0.01235
 
 0.01985
 
 Not queried
 
  0.7219008
 
       NA
 
       NA
  
 
  
 11
 
  32851087
 
  32851378
 
  ENSEMBL   UCSC 
 
  292
 
  9
 
 7208
 
 tss
 
 0.01487
 
 0.02875
 
 Not queried
 
  0.7218772
 
       NA
 
       NA
  
 
  
 10
 
 134460304
 
 134460468
 
  ENSEMBL   UCSC 
 
  165
 
  4
 
 4854
 
 gene;island
 
 0.85630
 
 0.80020
 
 Not queried
 
  0.7218069
 
       NA
 
       NA
  
 
  
 19
 
  40561788
 
  40562354
 
  ENSEMBL   UCSC 
 
  567
 
  9
 
 21976
 
 island
 
 0.02976
 
 0.07271
 
 Not queried
 
  0.7217252
 
       NA
 
       NA
  
 
  
 14
 
  58862965
 
  58863097
 
  ENSEMBL   UCSC 
 
  133
 
  4
 
 12068
 
 gene;island
 
 0.03892
 
 0.06436
 
 Not queried
 
  0.7216119
 
       NA
 
       NA
  
 
  
 6
 
  78173200
 
  78173408
 
  ENSEMBL   UCSC 
 
  209
 
  5
 
 35014
 
 tss;island
 
 0.02868
 
 0.03985
 
 Not queried
 
  0.7215832
 
       NA
 
       NA
  
 
  
 15
 
  60771498
 
  60771615
 
  ENSEMBL   UCSC 
 
  118
 
  5
 
 13683
 
 tss
 
 0.02001
 
 0.03257
 
 Not queried
 
  0.7214632
 
       NA
 
       NA
  
 
  
 16
 
  19729622
 
  19729857
 
  ENSEMBL   UCSC 
 
  236
 
  7
 
 14652
 
 gene;tss;island
 
 0.03690
 
 0.05197
 
 Not queried
 
  0.7214575
 
       NA
 
       NA
  
 
  
 5
 
  87963342
 
  87963592
 
  ENSEMBL   UCSC 
 
  251
 
  4
 
 31619
 
 gene
 
 0.03045
 
 0.03314
 
 Not queried
 
  0.7214128
 
       NA
 
       NA
  
 
  
 16
 
    815975
 
    816177
 
  ENSEMBL   UCSC 
 
  203
 
  5
 
 14516
 
 gene
 
 0.71257
 
 0.78493
 
 Not queried
 
  0.7210813
 
       NA
 
       NA
  
 
  
 16
 
  31519317
 
  31519964
 
  ENSEMBL   UCSC 
 
  648
 
  9
 
 15848
 
 island
 
 0.01914
 
 0.02553
 
 Not queried
 
  0.7209921
 
       NA
 
       NA
  
 
  
 7
 
  99156126
 
  99156387
 
  ENSEMBL   UCSC 
 
  262
 
  4
 
 37067
 
 gene
 
 0.01879
 
 0.03383
 
 Not queried
 
  0.7209747
 
       NA
 
       NA
  
 
  
 18
 
  20714217
 
  20714496
 
  ENSEMBL   UCSC 
 
  280
 
  5
 
 19170
 
 tss;island
 
 0.02549
 
 0.03555
 
 Not queried
 
  0.7209451
 
       NA
 
       NA
  
 
  
 4
 
 143767608
 
 143767672
 
  ENSEMBL   UCSC 
 
   65
 
  6
 
 30564
 
 tss
 
 0.04106
 
 0.05968
 
 Not queried
 
  0.7209283
 
       NA
 
       NA
  
 
  
 8
 
 129061048
 
 129061382
 
  ENSEMBL   UCSC 
 
  335
 
  4
 
 39368
 
 gene;tss
 
 0.88113
 
 0.92434
 
 Not queried
 
  0.7208829
 
       NA
 
       NA
  
 
  
 5
 
 156886816
 
 156887005
 
  ENSEMBL   UCSC 
 
  190
 
  6
 
 32397
 
 tss
 
 0.09190
 
 0.06809
 
 Not queried
 
  0.7208091
 
       NA
 
       NA
  
 
  
 4
 
  39979757
 
  39979791
 
  ENSEMBL   UCSC 
 
   35
 
  4
 
 30308
 
 tss;island
 
 0.02174
 
 0.03540
 
 Not queried
 
  0.7207982
 
       NA
 
       NA
  
 
  
 1
 
  36863212
 
  36863738
 
  ENSEMBL   UCSC 
 
  527
 
 11
 
 3053
 
 island
 
 0.02225
 
 0.03281
 
 Not queried
 
  0.7207135
 
       NA
 
       NA
  
 
  
 11
 
  73019337
 
  73019677
 
  ENSEMBL   UCSC 
 
  341
 
  8
 
 8617
 
 island
 
 0.01401
 
 0.01982
 
 Not queried
 
  0.7206991
 
       NA
 
       NA
  
 
  
 14
 
  93673175
 
  93673304
 
  ENSEMBL   UCSC 
 
  130
 
  4
 
 12158
 
 gene;tss;island
 
 0.02728
 
 0.04214
 
 Not queried
 
  0.7206267
 
       NA
 
       NA
  
 
  
 8
 
  48675647
 
  48676092
 
  ENSEMBL   UCSC 
 
  446
 
  7
 
 40208
 
 island
 
 0.77680
 
 0.83758
 
 Not queried
 
  0.7205365
 
       NA
 
       NA
  
 
  
 3
 
  69812798
 
  69812973
 
  ENSEMBL   UCSC 
 
  176
 
  4
 
 27813
 
 gene
 
 0.76519
 
 0.78390
 
 Not queried
 
  0.7205279
 
       NA
 
       NA
  
 
  
 12
 
 133419166
 
 133419329
 
  ENSEMBL   UCSC 
 
  164
 
  4
 
 9440
 
 gene;island
 
 0.78332
 
 0.87291
 
 Not queried
 
  0.7205092
 
       NA
 
       NA
  
 
  
 7
 
  26438234
 
  26438411
 
  ENSEMBL   UCSC 
 
  178
 
  5
 
 38268
 
 island
 
 0.00937
 
 0.01229
 
 Not queried
 
  0.7204134
 
       NA
 
       NA
  
 
  
 17
 
  62341026
 
  62341149
 
  ENSEMBL   UCSC 
 
  124
 
  4
 
 17549
 
 tss;island
 
 0.03199
 
 0.02150
 
 Not queried
 
  0.7203019
 
       NA
 
       NA
  
 
  
 9
 
 134406824
 
 134407190
 
  ENSEMBL   UCSC 
 
  367
 
  4
 
 40783
 
 tss;island
 
 0.72513
 
 0.61263
 
 Not queried
 
  0.7202471
 
       NA
 
       NA
  
 
  
 14
 
  77495072
 
  77495248
 
  ENSEMBL   UCSC 
 
  177
 
  4
 
 12498
 
 tss
 
 0.02263
 
 0.03078
 
 Not queried
 
  0.7199330
 
       NA
 
       NA
  
 
  
 20
 
  30102058
 
  30102125
 
  ENSEMBL   UCSC 
 
   68
 
  6
 
 25622
 
 tss
 
 0.04258
 
 0.08943
 
 Not queried
 
  0.7198825
 
       NA
 
       NA
  
 
  
 6
 
 100016731
 
 100016838
 
  ENSEMBL   UCSC 
 
  108
 
  6
 
 35072
 
 tss
 
 0.02147
 
 0.03415
 
 Not queried
 
  0.7198493
 
       NA
 
       NA
  
 
  
 4
 
  15005434
 
  15005593
 
  ENSEMBL   UCSC 
 
  160
 
  7
 
 30885
 
 island
 
 0.02471
 
 0.03258
 
 Not queried
 
  0.7198483
 
       NA
 
       NA
  
 
  
 21
 
  27543229
 
  27543721
 
  ENSEMBL   UCSC 
 
  493
 
  5
 
 26458
 
 tss
 
 0.02998
 
 0.02586
 
 Not queried
 
  0.7196160
 
       NA
 
       NA
  
 
  
 21
 
  27543045
 
  27543721
 
  ENSEMBL   UCSC 
 
  677
 
  7
 
 26579
 
 island
 
 0.02998
 
 0.02586
 
 Not queried
 
  0.7196160
 
       NA
 
       NA
  
 
  
 3
 
  15140507
 
  15140889
 
  ENSEMBL   UCSC 
 
  383
 
  6
 
 28873
 
 island
 
 0.01664
 
 0.02139
 
 Not queried
 
  0.7195531
 
       NA
 
       NA
  
 
  
 5
 
 176810904
 
 176811305
 
  ENSEMBL   UCSC 
 
  402
 
  6
 
 32466
 
 tss
 
 0.70974
 
 0.56260
 
 Not queried
 
  0.7193295
 
       NA
 
       NA
  
 
  
 12
 
  14926572
 
  14927542
 
  ENSEMBL   UCSC 
 
  971
 
 13
 
 10240
 
 island
 
 0.02625
 
 0.04239
 
 Not queried
 
  0.7193294
 
       NA
 
       NA
  
 
  
 1
 
 204183337
 
 204183655
 
  ENSEMBL   UCSC 
 
  319
 
  7
 
 2193
 
 tss
 
 0.02455
 
 0.03557
 
 Not queried
 
  0.7193008
 
       NA
 
       NA
  
 
  
 2
 
  63274476
 
  63275178
 
  ENSEMBL   UCSC 
 
  703
 
  7
 
 24297
 
 island
 
 0.03346
 
 0.07904
 
 Not queried
 
  0.7192725
 
       NA
 
       NA
  
 
  
 10
 
  38382849
 
  38383250
 
  ENSEMBL   UCSC 
 
  402
 
  9
 
 5019
 
 tss
 
 0.01312
 
 0.02391
 
 Not queried
 
  0.7192570
 
       NA
 
       NA
  
 
  
 2
 
  74756234
 
  74756470
 
  ENSEMBL   UCSC 
 
  237
 
  7
 
 23425
 
 tss
 
 0.03989
 
 0.01691
 
 Not queried
 
  0.7191971
 
       NA
 
       NA
  
 
  
 11
 
   2322286
 
   2323459
 
  ENSEMBL   UCSC 
 
 1174
 
 23
 
 6436
 
 gene;tss
 
 0.57732
 
 0.67166
 
 Not queried
 
  0.7191141
 
       NA
 
       NA
  
 
  
 15
 
  75230280
 
  75230753
 
  ENSEMBL   UCSC 
 
  474
 
  8
 
 14293
 
 island
 
 0.01988
 
 0.02539
 
 Not queried
 
  0.7191025
 
       NA
 
       NA
  
 
  
 6
 
  31937454
 
  31938533
 
  ENSEMBL   UCSC 
 
 1080
 
 17
 
 34792
 
 tss
 
 0.87504
 
 0.93150
 
 Not queried
 
  0.7190455
 
       NA
 
       NA
  
 
  
 19
 
  32836297
 
  32836832
 
  ENSEMBL   UCSC 
 
  536
 
 10
 
 21829
 
 island
 
 0.02793
 
 0.05824
 
 Not queried
 
  0.7190062
 
       NA
 
       NA
  
 
  
 17
 
  28443852
 
  28444188
 
  ENSEMBL   UCSC 
 
  337
 
  8
 
 16471
 
 gene
 
 0.02323
 
 0.02743
 
 Not queried
 
  0.7188710
 
       NA
 
       NA
  
 
  
 7
 
  43351208
 
  43351604
 
  ENSEMBL   UCSC 
 
  397
 
  6
 
 36919
 
 gene
 
 0.94128
 
 0.95257
 
 Not queried
 
  0.7186707
 
       NA
 
       NA
  
 
  
 1
 
 156211409
 
 156212005
 
  ENSEMBL   UCSC 
 
  597
 
 11
 
 3837
 
 island
 
 0.68126
 
 0.79490
 
 Not queried
 
  0.7186638
 
       NA
 
       NA
  
 
  
 2
 
 237120069
 
 237120313
 
  ENSEMBL   UCSC 
 
  245
 
  4
 
 23053
 
 gene;island
 
 0.91990
 
 0.84764
 
 Not queried
 
  0.7185939
 
       NA
 
       NA
  
 
  
 20
 
  44044586
 
  44044709
 
  ENSEMBL   UCSC 
 
  124
 
  6
 
 25736
 
 tss
 
 0.00862
 
 0.02229
 
 Not queried
 
  0.7185598
 
       NA
 
       NA
  
 
  
 7
 
  64254469
 
  64254733
 
  ENSEMBL   UCSC 
 
  265
 
  6
 
 37551
 
 tss
 
 0.08520
 
 0.03796
 
 Not queried
 
  0.7185418
 
       NA
 
       NA
  
 
  
 19
 
   1725535
 
   1725758
 
  ENSEMBL   UCSC 
 
  224
 
  4
 
 21201
 
 island
 
 0.10284
 
 0.04692
 
 Not queried
 
  0.7185358
 
       NA
 
       NA
  
 
  
 5
 
 156351010
 
 156351266
 
  ENSEMBL   UCSC 
 
  257
 
  4
 
 31814
 
 gene
 
 0.78871
 
 0.72696
 
 Not queried
 
  0.7185049
 
       NA
 
       NA
  
 
  
 7
 
  20817859
 
  20818226
 
  ENSEMBL   UCSC 
 
  368
 
  4
 
 38227
 
 island
 
 0.05138
 
 0.02544
 
 Not queried
 
  0.7183722
 
       NA
 
       NA
  
 
  
 11
 
 121970613
 
 121970768
 
  ENSEMBL   UCSC 
 
  156
 
  5
 
 7747
 
 tss
 
 0.03348
 
 0.05366
 
 Not queried
 
  0.7182830
 
       NA
 
       NA
  
 
  
 12
 
  96336486
 
  96336545
 
  ENSEMBL   UCSC 
 
   60
 
  4
 
 9903
 
 tss;island
 
 0.05719
 
 0.03030
 
 Not queried
 
  0.7182761
 
       NA
 
       NA
  
 
  
 2
 
  11810365
 
  11810683
 
  ENSEMBL   UCSC 
 
  319
 
  7
 
 23154
 
 tss
 
 0.29131
 
 0.40588
 
 Not queried
 
  0.7182552
 
       NA
 
       NA
  
 
  
 1
 
  65730339
 
  65730409
 
  ENSEMBL   UCSC 
 
   71
 
  4
 
 1673
 
 tss
 
 0.05092
 
 0.06037
 
 Not queried
 
  0.7182444
 
       NA
 
       NA
  
 
  
 13
 
  79169714
 
  79170627
 
  ENSEMBL   UCSC 
 
  914
 
 10
 
 11763
 
 island
 
 0.03188
 
 0.05315
 
 Not queried
 
  0.7180973
 
       NA
 
       NA
  
 
  
 2
 
 231851704
 
 231852154
 
  ENSEMBL   UCSC 
 
  451
 
  5
 
 25138
 
 island
 
 0.04945
 
 0.07578
 
 Not queried
 
  0.7179664
 
       NA
 
       NA
  
 
  
 19
 
  44809188
 
  44809319
 
  ENSEMBL   UCSC 
 
  132
 
  4
 
 20811
 
 tss
 
 0.01784
 
 0.02500
 
 Not queried
 
  0.7177483
 
       NA
 
       NA
  
 
  
 1
 
 109968980
 
 109969188
 
  ENSEMBL   UCSC 
 
  209
 
  8
 
 3553
 
 island
 
 0.01492
 
 0.02546
 
 Not queried
 
  0.7177285
 
       NA
 
       NA
  
 
  
 12
 
   9446757
 
   9446913
 
  ENSEMBL   UCSC 
 
  157
 
  4
 
 9027
 
 gene
 
 0.93579
 
 0.77632
 
 Not queried
 
  0.7175538
 
       NA
 
       NA
  
 
  
 3
 
  32509018
 
  32509543
 
  ENSEMBL   UCSC 
 
  526
 
  9
 
 28921
 
 island
 
 0.55408
 
 0.62945
 
 Not queried
 
  0.7175097
 
       NA
 
       NA
  
 
  
 15
 
  75073839
 
  75074068
 
  ENSEMBL   UCSC 
 
  230
 
  4
 
 13773
 
 tss;island
 
 0.03511
 
 0.05824
 
 Not queried
 
  0.7174931
 
       NA
 
       NA
  
 
  
 3
 
  75668627
 
  75669109
 
  ENSEMBL   UCSC 
 
  483
 
  4
 
 29245
 
 island
 
 0.34873
 
 0.44681
 
 Not queried
 
  0.7174548
 
       NA
 
       NA
  
 
  
 4
 
  76912131
 
  76912348
 
  ENSEMBL   UCSC 
 
  218
 
  8
 
 30396
 
 tss;island
 
 0.01332
 
 0.02643
 
 Not queried
 
  0.7174311
 
       NA
 
       NA
  
 
  
 2
 
 119607097
 
 119607636
 
  ENSEMBL   UCSC 
 
  540
 
  7
 
 24611
 
 island
 
 0.52924
 
 0.64736
 
 Not queried
 
  0.7173138
 
       NA
 
       NA
  
 
  
 10
 
  52499393
 
  52499571
 
  ENSEMBL   UCSC 
 
  179
 
  4
 
 5065
 
 tss
 
 0.01278
 
 0.01925
 
 Not queried
 
  0.7172422
 
       NA
 
       NA
  
 
  
 2
 
  27434612
 
  27434752
 
  ENSEMBL   UCSC 
 
  141
 
  6
 
 22585
 
 gene;tss;island
 
 0.01899
 
 0.03860
 
 Not queried
 
  0.7171205
 
       NA
 
       NA
  
 
  
 17
 
  66453769
 
  66453919
 
  ENSEMBL   UCSC 
 
  151
 
  4
 
 17575
 
 tss;island
 
 0.02098
 
 0.03108
 
 Not queried
 
  0.7169072
 
       NA
 
       NA
  
 
  
 12
 
  56545428
 
  56545810
 
  ENSEMBL   UCSC 
 
  383
 
  4
 
 9761
 
 tss;island
 
 0.05043
 
 0.03130
 
 Not queried
 
  0.7166910
 
       NA
 
       NA
  
 
  
 17
 
  59940826
 
  59941132
 
  ENSEMBL   UCSC 
 
  307
 
  7
 
 17526
 
 tss
 
 0.00626
 
 0.02138
 
 Not queried
 
  0.7166861
 
       NA
 
       NA
  
 
  
 1
 
 217311511
 
 217311608
 
  ENSEMBL   UCSC 
 
   98
 
  4
 
 2251
 
 tss;island
 
 0.12164
 
 0.07887
 
 Not queried
 
  0.7164780
 
       NA
 
       NA
  
 
  
 7
 
   2442660
 
   2443076
 
  ENSEMBL   UCSC 
 
  417
 
  6
 
 37345
 
 tss;island
 
 0.02700
 
 0.05167
 
 Not queried
 
  0.7162734
 
       NA
 
       NA
  
 
  
 12
 
   6602187
 
   6602654
 
  ENSEMBL   UCSC 
 
  468
 
  7
 
 9488
 
 tss;island
 
 0.00964
 
 0.01812
 
 Not queried
 
  0.7161381
 
       NA
 
       NA
  
 
  
 22
 
  36806414
 
  36806655
 
  ENSEMBL   UCSC 
 
  242
 
  4
 
 27409
 
 island
 
 0.03716
 
 0.02255
 
 Not queried
 
  0.7161063
 
       NA
 
       NA
  
 
  
 15
 
  90118624
 
  90119427
 
  ENSEMBL   UCSC 
 
  804
 
 11
 
 14410
 
 island
 
 0.01905
 
 0.03871
 
 Not queried
 
  0.7157478
 
       NA
 
       NA
  
 
  
 3
 
 173113376
 
 173113576
 
  ENSEMBL   UCSC 
 
  201
 
  4
 
 29626
 
 island
 
 0.03748
 
 0.04278
 
 Not queried
 
  0.7155280
 
       NA
 
       NA
  
 
  
 1
 
 228594250
 
 228594464
 
  ENSEMBL   UCSC 
 
  215
 
  4
 
 1057
 
 gene
 
 0.01025
 
 0.02425
 
 Not queried
 
  0.7155143
 
       NA
 
       NA
  
 
  
 6
 
   5087266
 
   5087749
 
  ENSEMBL   UCSC 
 
  484
 
  4
 
 35335
 
 island
 
 0.90501
 
 0.87112
 
 Not queried
 
  0.7154837
 
       NA
 
       NA
  
 
  
 15
 
  82555336
 
  82555552
 
  ENSEMBL   UCSC 
 
  217
 
  4
 
 13423
 
 gene;tss;island
 
 0.01548
 
 0.02137
 
 Not queried
 
  0.7154208
 
       NA
 
       NA
  
 
  
 1
 
 240255377
 
 240255691
 
  ENSEMBL   UCSC 
 
  315
 
  4
 
 1102
 
 gene;island
 
 0.03462
 
 0.05405
 
 Not queried
 
  0.7152116
 
       NA
 
       NA
  
 
  
 10
 
  30721971
 
  30722286
 
  ENSEMBL   UCSC 
 
  316
 
  4
 
 4995
 
 tss;island
 
 0.09634
 
 0.05369
 
 Not queried
 
  0.7151361
 
       NA
 
       NA
  
 
  
 11
 
 110582732
 
 110583036
 
  ENSEMBL   UCSC 
 
  305
 
  4
 
 6876
 
 gene;island
 
 0.03532
 
 0.05692
 
 Not queried
 
  0.7149807
 
       NA
 
       NA
  
 
  
 15
 
  90319420
 
  90319584
 
  ENSEMBL   UCSC 
 
  165
 
  4
 
 13864
 
 tss
 
 0.58200
 
 0.59620
 
 Not queried
 
  0.7149505
 
       NA
 
       NA
  
 
  
 19
 
  41317837
 
  41318235
 
  ENSEMBL   UCSC 
 
  399
 
  4
 
 22009
 
 island
 
 0.27055
 
 0.19709
 
 Not queried
 
  0.7149086
 
       NA
 
       NA
  
 
  
 1
 
  17308032
 
  17308303
 
  ENSEMBL   UCSC 
 
  272
 
  5
 
 1285
 
 tss;island
 
 0.71150
 
 0.68020
 
 Not queried
 
  0.7148868
 
       NA
 
       NA
  
 
  
 22
 
  38794936
 
  38795061
 
  ENSEMBL   UCSC 
 
  126
 
  6
 
 27096
 
 tss
 
 0.01387
 
 0.02045
 
 Not queried
 
  0.7148809
 
       NA
 
       NA
  
 
  
 6
 
  42018162
 
  42018626
 
  ENSEMBL   UCSC 
 
  465
 
  7
 
 36074
 
 island
 
 0.03264
 
 0.03191
 
 Not queried
 
  0.7148795
 
       NA
 
       NA
  
 
  
 5
 
 132361924
 
 132362247
 
  ENSEMBL   UCSC 
 
  324
 
  5
 
 33044
 
 island
 
 0.02319
 
 0.02061
 
 Not queried
 
  0.7148239
 
       NA
 
       NA
  
 
  
 6
 
  28971902
 
  28972110
 
  ENSEMBL   UCSC 
 
  209
 
  4
 
 33585
 
 gene
 
 0.72900
 
 0.85978
 
 Not queried
 
  0.7147387
 
       NA
 
       NA
  
 
  
 15
 
  42264823
 
  42265079
 
  ENSEMBL   UCSC 
 
  257
 
  7
 
 13585
 
 tss
 
 0.01275
 
 0.02019
 
 Not queried
 
  0.7146307
 
       NA
 
       NA
  
 
  
 1
 
  27189270
 
  27189440
 
  ENSEMBL   UCSC 
 
  171
 
  4
 
 1395
 
 tss;island
 
 0.44126
 
 0.54731
 
 Not queried
 
  0.7145350
 
       NA
 
       NA
  
 
  
 1
 
 110081561
 
 110081965
 
  ENSEMBL   UCSC 
 
  405
 
  4
 
 1803
 
 tss
 
 0.56948
 
 0.65199
 
 Not queried
 
  0.7144055
 
       NA
 
       NA
  
 
  
 X
 
  20159528
 
  20160252
 
  ENSEMBL   UCSC 
 
  725
 
 10
 
 41679
 
 island
 
 0.01501
 
 0.02973
 
 Not queried
 
  0.7143149
 
       NA
 
       NA
  
 
  
 8
 
 104383715
 
 104383971
 
  ENSEMBL   UCSC 
 
  257
 
  6
 
 40453
 
 island
 
 0.00845
 
 0.01208
 
 Not queried
 
  0.7142333
 
       NA
 
       NA
  
 
  
 10
 
  12237758
 
  12237955
 
  ENSEMBL   UCSC 
 
  198
 
  7
 
 4485
 
 gene;tss;island
 
 0.01559
 
 0.02148
 
 Not queried
 
  0.7141718
 
       NA
 
       NA
  
 
  
 3
 
  26664115
 
  26664404
 
  ENSEMBL   UCSC 
 
  290
 
  5
 
 28902
 
 island
 
 0.02193
 
 0.03498
 
 Not queried
 
  0.7140751
 
       NA
 
       NA
  
 
  
 1
 
 247681102
 
 247681931
 
  ENSEMBL   UCSC 
 
  830
 
  8
 
 4405
 
 island
 
 0.67641
 
 0.80688
 
 Not queried
 
  0.7140571
 
       NA
 
       NA
  
 
  
 14
 
  61116782
 
  61117162
 
  ENSEMBL   UCSC 
 
  381
 
  5
 
 12412
 
 tss;island
 
 0.04801
 
 0.07150
 
 Not queried
 
  0.7140169
 
       NA
 
       NA
  
 
  
 4
 
  77355817
 
  77356250
 
  ENSEMBL   UCSC 
 
  434
 
  5
 
 30402
 
 tss
 
 0.04317
 
 0.06196
 
 Not queried
 
  0.7139541
 
       NA
 
       NA
  
 
  
 16
 
   1420086
 
   1420537
 
  ENSEMBL   UCSC 
 
  452
 
  5
 
 14539
 
 gene;island
 
 0.87010
 
 0.91658
 
 Not queried
 
  0.7139276
 
       NA
 
       NA
  
 
  
 15
 
  78384520
 
  78384897
 
  ENSEMBL   UCSC 
 
  378
 
  5
 
 13800
 
 tss
 
 0.82187
 
 0.72161
 
 Not queried
 
  0.7138992
 
       NA
 
       NA
  
 
  
 1
 
  26324332
 
  26324647
 
  ENSEMBL   UCSC 
 
  316
 
  6
 
 335
 
 gene
 
 0.00733
 
 0.00914
 
 Not queried
 
  0.7138887
 
       NA
 
       NA
  
 
  
 19
 
  14063031
 
  14063471
 
  ENSEMBL   UCSC 
 
  441
 
  9
 
 19853
 
 gene;island
 
 0.00996
 
 0.01712
 
 Not queried
 
  0.7138462
 
       NA
 
       NA
  
 
  
 17
 
  43224789
 
  43225229
 
  ENSEMBL   UCSC 
 
  441
 
  5
 
 16595
 
 gene;island
 
 0.01348
 
 0.01343
 
 Not queried
 
  0.7137962
 
       NA
 
       NA
  
 
  
 3
 
  52279902
 
  52280139
 
  ENSEMBL   UCSC 
 
  238
 
  5
 
 28334
 
 tss
 
 0.04138
 
 0.06506
 
 Not queried
 
  0.7136089
 
       NA
 
       NA
  
 
  
 15
 
  90039409
 
  90039794
 
  ENSEMBL   UCSC 
 
  386
 
  4
 
 13441
 
 gene
 
 0.02906
 
 0.06700
 
 Not queried
 
  0.7135810
 
       NA
 
       NA
  
 
  
 5
 
 140019017
 
 140019523
 
  ENSEMBL   UCSC 
 
  507
 
  6
 
 31731
 
 gene
 
 0.02744
 
 0.04226
 
 Not queried
 
  0.7133923
 
       NA
 
       NA
  
 
  
 7
 
 149570987
 
 149571373
 
  ENSEMBL   UCSC 
 
  387
 
 11
 
 37909
 
 tss
 
 0.01740
 
 0.03287
 
 Not queried
 
  0.7132038
 
       NA
 
       NA
  
 
  
 17
 
  79420145
 
  79420436
 
  ENSEMBL   UCSC 
 
  292
 
  5
 
 16878
 
 gene;island
 
 0.78653
 
 0.86329
 
 Not queried
 
  0.7129709
 
       NA
 
       NA
  
 
  
 X
 
   9432900
 
   9433262
 
  ENSEMBL   UCSC 
 
  363
 
  7
 
 40929
 
 gene;island
 
 0.01710
 
 0.03341
 
 Not queried
 
  0.7129619
 
       NA
 
       NA
  
 
  
 17
 
  42019637
 
  42019987
 
  ENSEMBL   UCSC 
 
  351
 
  5
 
 18348
 
 island
 
 0.81943
 
 0.69915
 
 Not queried
 
  0.7128888
 
       NA
 
       NA
  
 
  
 8
 
 143379015
 
 143379277
 
  ENSEMBL   UCSC 
 
  263
 
  4
 
 39395
 
 gene;island
 
 0.76008
 
 0.86058
 
 Not queried
 
  0.7128206
 
       NA
 
       NA
  
 
  
 X
 
  80457442
 
  80457709
 
  ENSEMBL   UCSC 
 
  268
 
  9
 
 41405
 
 tss
 
 0.02535
 
 0.02242
 
 Not queried
 
  0.7127401
 
       NA
 
       NA
  
 
  
 7
 
  94537744
 
  94537893
 
  ENSEMBL   UCSC 
 
  150
 
  7
 
 37658
 
 tss
 
 0.02560
 
 0.03440
 
 Not queried
 
  0.7127307
 
       NA
 
       NA
  
 
  
 12
 
  51420343
 
  51420611
 
  ENSEMBL   UCSC 
 
  269
 
  5
 
 9684
 
 tss;island
 
 0.05303
 
 0.05110
 
 Not queried
 
  0.7127167
 
       NA
 
       NA
  
 
  
 19
 
  41815869
 
  41816051
 
  ENSEMBL   UCSC 
 
  183
 
  5
 
 20769
 
 tss
 
 0.01463
 
 0.02369
 
 Not queried
 
  0.7126897
 
       NA
 
       NA
  
 
  
 16
 
   2570187
 
   2570492
 
  ENSEMBL   UCSC 
 
  306
 
  6
 
 15576
 
 island
 
 0.02624
 
 0.03793
 
 Not queried
 
  0.7126663
 
       NA
 
       NA
  
 
  
 16
 
  69458177
 
  69458746
 
  ENSEMBL   UCSC 
 
  570
 
  9
 
 16028
 
 island
 
 0.01768
 
 0.03765
 
 Not queried
 
  0.7125940
 
       NA
 
       NA
  
 
  
 17
 
  79818530
 
  79818808
 
  ENSEMBL   UCSC 
 
  279
 
  6
 
 18912
 
 island
 
 0.02346
 
 0.02525
 
 Not queried
 
  0.7125101
 
       NA
 
       NA
  
 
  
 X
 
  55515154
 
  55515751
 
  ENSEMBL   UCSC 
 
  598
 
  7
 
 41820
 
 island
 
 0.05328
 
 0.06076
 
 Not queried
 
  0.7124502
 
       NA
 
       NA
  
 
  
 1
 
  75596724
 
  75596930
 
  ENSEMBL   UCSC 
 
  207
 
  4
 
 586
 
 gene;island
 
 0.18524
 
 0.12185
 
 Not queried
 
  0.7124452
 
       NA
 
       NA
  
 
  
 19
 
  47759699
 
  47759811
 
  ENSEMBL   UCSC 
 
  113
 
  5
 
 22155
 
 island
 
 0.03571
 
 0.02782
 
 Not queried
 
  0.7124266
 
       NA
 
       NA
  
 
  
 8
 
   1899943
 
   1900524
 
  ENSEMBL   UCSC 
 
  582
 
  6
 
 39122
 
 gene
 
 0.86487
 
 0.91903
 
 Not queried
 
  0.7123688
 
       NA
 
       NA
  
 
  
 5
 
 169010289
 
 169010600
 
  ENSEMBL   UCSC 
 
  312
 
  5
 
 32422
 
 tss
 
 0.00470
 
 0.00776
 
 Not queried
 
  0.7122814
 
       NA
 
       NA
  
 
  
 16
 
    740182
 
    740572
 
  ENSEMBL   UCSC 
 
  391
 
  7
 
 15446
 
 island
 
 0.04224
 
 0.04515
 
 Not queried
 
  0.7121187
 
       NA
 
       NA
  
 
  
 12
 
  41085901
 
  41086308
 
  ENSEMBL   UCSC 
 
  408
 
  8
 
 9615
 
 tss
 
 0.02825
 
 0.04465
 
 Not queried
 
  0.7120889
 
       NA
 
       NA
  
 
  
 1
 
 235324133
 
 235324437
 
  ENSEMBL   UCSC 
 
  305
 
  5
 
 1085
 
 gene
 
 0.01128
 
 0.01398
 
 Not queried
 
  0.7119682
 
       NA
 
       NA
  
 
  
 17
 
   7166293
 
   7166718
 
  ENSEMBL   UCSC 
 
  426
 
  4
 
 17037
 
 tss;island
 
 0.05636
 
 0.07937
 
 Not queried
 
  0.7119437
 
       NA
 
       NA
  
 
  
 10
 
  32345420
 
  32345553
 
  ENSEMBL   UCSC 
 
  134
 
  7
 
 5003
 
 tss
 
 0.02126
 
 0.03303
 
 Not queried
 
  0.7118211
 
       NA
 
       NA
  
 
  
 16
 
  56666323
 
  56666640
 
  ENSEMBL   UCSC 
 
  318
 
  5
 
 15917
 
 island
 
 0.06509
 
 0.03874
 
 Not queried
 
  0.7117721
 
       NA
 
       NA
  
 
  
 19
 
  42721812
 
  42722189
 
  ENSEMBL   UCSC 
 
  378
 
 10
 
 22034
 
 island
 
 0.03062
 
 0.05552
 
 Not queried
 
  0.7116470
 
       NA
 
       NA
  
 
  
 1
 
  86174607
 
  86174929
 
  ENSEMBL   UCSC 
 
  323
 
  4
 
 1721
 
 tss;island
 
 0.86290
 
 0.80500
 
 Not queried
 
  0.7115928
 
       NA
 
       NA
  
 
  
 12
 
  54982590
 
  54982717
 
  ENSEMBL   UCSC 
 
  128
 
  4
 
 9741
 
 tss;island
 
 0.05218
 
 0.08276
 
 Not queried
 
  0.7115870
 
       NA
 
       NA
  
 
  
 2
 
 223168453
 
 223168941
 
  ENSEMBL   UCSC 
 
  489
 
  6
 
 23014
 
 gene;island
 
 0.14893
 
 0.09546
 
 Not queried
 
  0.7115117
 
       NA
 
       NA
  
 
  
 1
 
 159046773
 
 159047163
 
  ENSEMBL   UCSC 
 
  391
 
  5
 
 2024
 
 tss
 
 0.70679
 
 0.88713
 
 Not queried
 
  0.7114364
 
       NA
 
       NA
  
 
  
 13
 
  95364908
 
  95364993
 
  ENSEMBL   UCSC 
 
   86
 
  5
 
 11473
 
 tss;island
 
 0.01351
 
 0.03011
 
 Not queried
 
  0.7113931
 
       NA
 
       NA
  
 
  
 12
 
 130823838
 
 130824529
 
  ENSEMBL   UCSC 
 
  692
 
  7
 
 9387
 
 gene;island
 
 0.53425
 
 0.62463
 
 Not queried
 
  0.7113579
 
       NA
 
       NA
  
 
  
 14
 
  23425770
 
  23426164
 
  ENSEMBL   UCSC 
 
  395
 
  6
 
 11991
 
 gene;island
 
 0.02417
 
 0.02611
 
 Not queried
 
  0.7113537
 
       NA
 
       NA
  
 
  
 16
 
  58718334
 
  58718971
 
  ENSEMBL   UCSC 
 
  638
 
  9
 
 15949
 
 island
 
 0.07425
 
 0.10641
 
 Not queried
 
  0.7113526
 
       NA
 
       NA
  
 
  
 7
 
 101936230
 
 101936527
 
  ENSEMBL   UCSC 
 
  298
 
  5
 
 37102
 
 gene;island
 
 0.08783
 
 0.05554
 
 Not queried
 
  0.7113266
 
       NA
 
       NA
  
 
  
 13
 
  44544993
 
  44545268
 
  ENSEMBL   UCSC 
 
  276
 
  4
 
 11673
 
 island
 
 0.93533
 
 0.87154
 
 Not queried
 
  0.7112906
 
       NA
 
       NA
  
 
  
 X
 
  69664317
 
  69664617
 
  ENSEMBL   UCSC 
 
  301
 
  6
 
 41368
 
 tss
 
 0.07171
 
 0.06303
 
 Not queried
 
  0.7110608
 
       NA
 
       NA
  
 
  
 16
 
  85878940
 
  85879145
 
  ENSEMBL   UCSC 
 
  206
 
  4
 
 16131
 
 island
 
 0.85800
 
 0.91654
 
 Not queried
 
  0.7108520
 
       NA
 
       NA
  
 
  
 8
 
  22436246
 
  22436458
 
  ENSEMBL   UCSC 
 
  213
 
  4
 
 39526
 
 tss;island
 
 0.20543
 
 0.14207
 
 Not queried
 
  0.7107178
 
       NA
 
       NA
  
 
  
 1
 
  36863509
 
  36863738
 
  ENSEMBL   UCSC 
 
  230
 
  8
 
 1493
 
 tss
 
 0.02139
 
 0.03250
 
 Not queried
 
  0.7106314
 
       NA
 
       NA
  
 
  
 1
 
 149223316
 
 149223634
 
  ENSEMBL   UCSC 
 
  319
 
  4
 
 3695
 
 island
 
 0.07958
 
 0.07524
 
 Not queried
 
  0.7104937
 
       NA
 
       NA
  
 
  
 22
 
  47157964
 
  47158756
 
  ENSEMBL   UCSC 
 
  793
 
  9
 
 27554
 
 island
 
 0.02621
 
 0.02930
 
 Not queried
 
  0.7104794
 
       NA
 
       NA
  
 
  
 3
 
  53164314
 
  53164425
 
  ENSEMBL   UCSC 
 
  112
 
  4
 
 27786
 
 gene
 
 0.01865
 
 0.02733
 
 Not queried
 
  0.7102851
 
       NA
 
       NA
  
 
  
 17
 
  76227227
 
  76227681
 
  ENSEMBL   UCSC 
 
  455
 
  7
 
 18785
 
 island
 
 0.67925
 
 0.46974
 
 Not queried
 
  0.7100486
 
       NA
 
       NA
  
 
  
 17
 
   8066184
 
   8066251
 
  ENSEMBL   UCSC 
 
   68
 
  4
 
 16391
 
 gene;island
 
 0.02924
 
 0.03681
 
 Not queried
 
  0.7100389
 
       NA
 
       NA
  
 
  
 8
 
 145019377
 
 145019839
 
  ENSEMBL   UCSC 
 
  463
 
  9
 
 39438
 
 gene;tss;island
 
 0.68139
 
 0.87100
 
 Not queried
 
  0.7100154
 
       NA
 
       NA
  
 
  
 2
 
  74607507
 
  74607697
 
  ENSEMBL   UCSC 
 
  191
 
  4
 
 23413
 
 tss
 
 0.53733
 
 0.44192
 
 Not queried
 
  0.7099450
 
       NA
 
       NA
  
 
  
 14
 
  29243173
 
  29243762
 
  ENSEMBL   UCSC 
 
  590
 
  7
 
 12024
 
 gene;island
 
 0.05721
 
 0.09030
 
 Not queried
 
  0.7099114
 
       NA
 
       NA
  
 
  
 3
 
  66024549
 
  66024691
 
  ENSEMBL   UCSC 
 
  143
 
  6
 
 28391
 
 tss
 
 0.02261
 
 0.05195
 
 Not queried
 
  0.7098742
 
       NA
 
       NA
  
 
  
 15
 
  73735509
 
  73735694
 
  ENSEMBL   UCSC 
 
  186
 
  4
 
 13383
 
 gene
 
 0.93242
 
 0.89293
 
 Not queried
 
  0.7098407
 
       NA
 
       NA
  
 
  
 15
 
  83680418
 
  83680570
 
  ENSEMBL   UCSC 
 
  153
 
  6
 
 13835
 
 tss
 
 0.01133
 
 0.02609
 
 Not queried
 
  0.7098187
 
       NA
 
       NA
  
 
  
 13
 
  28712342
 
  28712670
 
  ENSEMBL   UCSC 
 
  329
 
  4
 
 11090
 
 gene;island
 
 0.01577
 
 0.01887
 
 Not queried
 
  0.7097708
 
       NA
 
       NA
  
 
  
 4
 
 187646730
 
 187647457
 
  ENSEMBL   UCSC 
 
  728
 
  8
 
 31432
 
 island
 
 0.04725
 
 0.09246
 
 Not queried
 
  0.7097679
 
       NA
 
       NA
  
 
  
 3
 
 197354944
 
 197355164
 
  ENSEMBL   UCSC 
 
  221
 
  5
 
 28778
 
 tss;island
 
 0.05036
 
 0.03085
 
 Not queried
 
  0.7097673
 
       NA
 
       NA
  
 
  
 7
 
   6189539
 
   6189918
 
  ENSEMBL   UCSC 
 
  380
 
  4
 
 36821
 
 gene;island
 
 0.90962
 
 0.94684
 
 Not queried
 
  0.7096594
 
       NA
 
       NA
  
 
  
 2
 
 105858730
 
 105859083
 
  ENSEMBL   UCSC 
 
  354
 
  4
 
 22776
 
 gene;island
 
 0.84769
 
 0.76182
 
 Not queried
 
  0.7096046
 
       NA
 
       NA
  
 
  
 2
 
  64068705
 
  64069010
 
  ENSEMBL   UCSC 
 
  306
 
  7
 
 23352
 
 tss
 
 0.03511
 
 0.03832
 
 Not queried
 
  0.7095821
 
       NA
 
       NA
  
 
  
 15
 
  79724739
 
  79724802
 
  ENSEMBL   UCSC 
 
   64
 
  4
 
 13817
 
 tss;island
 
 0.03187
 
 0.06207
 
 Not queried
 
  0.7091726
 
       NA
 
       NA
  
 
  
 6
 
  71276074
 
  71276548
 
  ENSEMBL   UCSC 
 
  475
 
  8
 
 34994
 
 tss
 
 0.02382
 
 0.03447
 
 Not queried
 
  0.7091426
 
       NA
 
       NA
  
 
  
 11
 
   6947353
 
   6947773
 
  ENSEMBL   UCSC 
 
  421
 
 11
 
 8002
 
 island
 
 0.01511
 
 0.01608
 
 Not queried
 
  0.7091109
 
       NA
 
       NA
  
 
  
 19
 
   1354361
 
   1354765
 
  ENSEMBL   UCSC 
 
  405
 
  6
 
 20263
 
 tss;island
 
 0.29098
 
 0.30199
 
 Not queried
 
  0.7090515
 
       NA
 
       NA
  
 
  
 11
 
  72929368
 
  72929553
 
  ENSEMBL   UCSC 
 
  186
 
  4
 
 6801
 
 gene
 
 0.04770
 
 0.02540
 
 Not queried
 
  0.7090060
 
       NA
 
       NA
  
 
  
 14
 
  75079072
 
  75079491
 
  ENSEMBL   UCSC 
 
  420
 
  6
 
 12480
 
 tss;island
 
 0.02465
 
 0.04113
 
 Not queried
 
  0.7089996
 
       NA
 
       NA
  
 
  
 12
 
  56320915
 
  56320970
 
  ENSEMBL   UCSC 
 
   56
 
  5
 
 9747
 
 tss
 
 0.01814
 
 0.02061
 
 Not queried
 
  0.7088975
 
       NA
 
       NA
  
 
  
 11
 
  86085623
 
  86085757
 
  ENSEMBL   UCSC 
 
  135
 
  4
 
 7588
 
 tss
 
 0.30775
 
 0.46258
 
 Not queried
 
  0.7088848
 
       NA
 
       NA
  
 
  
 1
 
 146713916
 
 146714232
 
  ENSEMBL   UCSC 
 
  317
 
 10
 
 1889
 
 tss
 
 0.03498
 
 0.09038
 
 Not queried
 
  0.7087440
 
       NA
 
       NA
  
 
  
 2
 
  47797415
 
  47797590
 
  ENSEMBL   UCSC 
 
  176
 
  4
 
 24245
 
 island
 
 0.04553
 
 0.06124
 
 Not queried
 
  0.7085259
 
       NA
 
       NA
  
 
  
 19
 
  53794492
 
  53794794
 
  ENSEMBL   UCSC 
 
  303
 
  4
 
 20163
 
 gene
 
 0.96176
 
 0.93492
 
 Not queried
 
  0.7084995
 
       NA
 
       NA
  
 
  
 12
 
  40499623
 
  40500139
 
  ENSEMBL   UCSC 
 
  517
 
  5
 
 10300
 
 island
 
 0.06682
 
 0.02714
 
 Not queried
 
  0.7084739
 
       NA
 
       NA
  
 
  
 2
 
 135809693
 
 135809808
 
  ENSEMBL   UCSC 
 
  116
 
  5
 
 23605
 
 tss
 
 0.07744
 
 0.04642
 
 Not queried
 
  0.7084648
 
       NA
 
       NA
  
 
  
 7
 
 107384386
 
 107384783
 
  ENSEMBL   UCSC 
 
  398
 
  5
 
 37121
 
 gene
 
 0.03606
 
 0.02777
 
 Not queried
 
  0.7084615
 
       NA
 
       NA
  
 
  
 19
 
    913499
 
    913714
 
  ENSEMBL   UCSC 
 
  216
 
  4
 
 20247
 
 tss;island
 
 0.02120
 
 0.02421
 
 Not queried
 
  0.7084438
 
       NA
 
       NA
  
 
  
 17
 
  37025927
 
  37026044
 
  ENSEMBL   UCSC 
 
  118
 
  5
 
 17268
 
 tss
 
 0.03011
 
 0.04828
 
 Not queried
 
  0.7084103
 
       NA
 
       NA
  
 
  
 6
 
  32937207
 
  32937713
 
  ENSEMBL   UCSC 
 
  507
 
 15
 
 34103
 
 gene;tss;island
 
 0.02086
 
 0.02623
 
 Not queried
 
  0.7083402
 
       NA
 
       NA
  
 
  
 10
 
 112630726
 
 112630835
 
  ENSEMBL   UCSC 
 
  110
 
  4
 
 5342
 
 tss;island
 
 0.77032
 
 0.82033
 
 Not queried
 
  0.7083038
 
       NA
 
       NA
  
 
  
 22
 
  20008297
 
  20008614
 
  ENSEMBL   UCSC 
 
  318
 
  5
 
 26931
 
 tss;island
 
 0.01747
 
 0.01667
 
 Not queried
 
  0.7081264
 
       NA
 
       NA
  
 
  
 16
 
   2052269
 
   2052619
 
  ENSEMBL   UCSC 
 
  351
 
  4
 
 14564
 
 gene;island
 
 0.93114
 
 0.93383
 
 Not queried
 
  0.7079211
 
       NA
 
       NA
  
 
  
 4
 
 175444096
 
 175444268
 
  ENSEMBL   UCSC 
 
  173
 
  4
 
 30637
 
 tss;island
 
 0.01621
 
 0.02678
 
 Not queried
 
  0.7078857
 
       NA
 
       NA
  
 
  
 20
 
   1093493
 
   1093802
 
  ENSEMBL   UCSC 
 
  310
 
  6
 
 25493
 
 tss
 
 0.54797
 
 0.65469
 
 Not queried
 
  0.7078803
 
       NA
 
       NA
  
 
  
 6
 
  31937066
 
  31938533
 
  ENSEMBL   UCSC 
 
 1468
 
 26
 
 35817
 
 island
 
 0.87879
 
 0.92997
 
 Not queried
 
  0.7078712
 
       NA
 
       NA
  
 
  
 12
 
  74931289
 
  74931535
 
  ENSEMBL   UCSC 
 
  247
 
  6
 
 9853
 
 tss
 
 0.01680
 
 0.02332
 
 Not queried
 
  0.7078602
 
       NA
 
       NA
  
 
  
 11
 
 115530150
 
 115530590
 
  ENSEMBL   UCSC 
 
  441
 
  6
 
 8805
 
 island
 
 0.03805
 
 0.05466
 
 Not queried
 
  0.7077768
 
       NA
 
       NA
  
 
  
 13
 
  30424821
 
  30424932
 
  ENSEMBL   UCSC 
 
  112
 
  4
 
 11344
 
 tss;island
 
 0.01326
 
 0.01295
 
 Not queried
 
  0.7077282
 
       NA
 
       NA
  
 
  
 1
 
 107683532
 
 107683775
 
  ENSEMBL   UCSC 
 
  244
 
  5
 
 657
 
 gene;island
 
 0.01113
 
 0.01804
 
 Not queried
 
  0.7075006
 
       NA
 
       NA
  
 
  
 17
 
  27717641
 
  27717927
 
  ENSEMBL   UCSC 
 
  287
 
  8
 
 17200
 
 tss
 
 0.01295
 
 0.01889
 
 Not queried
 
  0.7073864
 
       NA
 
       NA
  
 
  
 1
 
   7111184
 
   7111367
 
  ENSEMBL   UCSC 
 
  184
 
  4
 
 184
 
 gene
 
 0.78452
 
 0.86307
 
 Not queried
 
  0.7072657
 
       NA
 
       NA
  
 
  
 2
 
  65454430
 
  65454860
 
  ENSEMBL   UCSC 
 
  431
 
 10
 
 24314
 
 island
 
 0.02514
 
 0.03735
 
 Not queried
 
  0.7072650
 
       NA
 
       NA
  
 
  
 1
 
 156357839
 
 156358408
 
  ENSEMBL   UCSC 
 
  570
 
 10
 
 3843
 
 island
 
 0.56482
 
 0.49040
 
 Not queried
 
  0.7072650
 
       NA
 
       NA
  
 
  
 19
 
  46650855
 
  46651227
 
  ENSEMBL   UCSC 
 
  373
 
  5
 
 20854
 
 tss
 
 0.36857
 
 0.41956
 
 Not queried
 
  0.7072502
 
       NA
 
       NA
  
 
  
 17
 
   7145532
 
   7146054
 
  ENSEMBL   UCSC 
 
  523
 
  9
 
 17893
 
 island
 
 0.01264
 
 0.01322
 
 Not queried
 
  0.7072289
 
       NA
 
       NA
  
 
  
 X
 
  47479436
 
  47479662
 
  ENSEMBL   UCSC 
 
  227
 
  5
 
 41282
 
 tss;island
 
 0.01271
 
 0.04444
 
 Not queried
 
  0.7072101
 
       NA
 
       NA
  
 
  
 13
 
  84456127
 
  84456486
 
  ENSEMBL   UCSC 
 
  360
 
  6
 
 11158
 
 gene
 
 0.01740
 
 0.03294
 
 Not queried
 
  0.7071849
 
       NA
 
       NA
  
 
  
 17
 
  77955167
 
  77955530
 
  ENSEMBL   UCSC 
 
  364
 
  4
 
 16813
 
 gene;island
 
 0.85220
 
 0.89724
 
 Not queried
 
  0.7071007
 
       NA
 
       NA
  
 
  
 10
 
  88024553
 
  88024673
 
  ENSEMBL   UCSC 
 
  121
 
  6
 
 5179
 
 tss
 
 0.60687
 
 0.76735
 
 Not queried
 
  0.7070800
 
       NA
 
       NA
  
 
  
 6
 
  31698047
 
  31698226
 
  ENSEMBL   UCSC 
 
  180
 
  7
 
 34769
 
 tss
 
 0.04805
 
 0.06981
 
 Not queried
 
  0.7070401
 
       NA
 
       NA
  
 
  
 1
 
 100715098
 
 100715402
 
  ENSEMBL   UCSC 
 
  305
 
  4
 
 650
 
 gene
 
 0.01593
 
 0.01642
 
 Not queried
 
  0.7069840
 
       NA
 
       NA
  
 
  
 12
 
  46123553
 
  46123850
 
  ENSEMBL   UCSC 
 
  298
 
  4
 
 10319
 
 island
 
 0.02367
 
 0.04374
 
 Not queried
 
  0.7069567
 
       NA
 
       NA
  
 
  
 7
 
 150745278
 
 150745494
 
  ENSEMBL   UCSC 
 
  217
 
  4
 
 37922
 
 tss
 
 0.71544
 
 0.65649
 
 Not queried
 
  0.7068956
 
       NA
 
       NA
  
 
  
 19
 
  50832552
 
  50832861
 
  ENSEMBL   UCSC 
 
  310
 
  6
 
 22269
 
 island
 
 0.05078
 
 0.06119
 
 Not queried
 
  0.7068246
 
       NA
 
       NA
  
 
  
 6
 
  30138178
 
  30138339
 
  ENSEMBL   UCSC 
 
  162
 
  4
 
 33675
 
 gene;island
 
 0.41617
 
 0.42666
 
 Not queried
 
  0.7067491
 
       NA
 
       NA
  
 
  
 19
 
    603639
 
    603853
 
  ENSEMBL   UCSC 
 
  215
 
  4
 
 19596
 
 gene;island
 
 0.91001
 
 0.86160
 
 Not queried
 
  0.7067141
 
       NA
 
       NA
  
 
  
 11
 
 111847692
 
 111847984
 
  ENSEMBL   UCSC 
 
  293
 
  5
 
 7673
 
 tss
 
 0.03328
 
 0.03313
 
 Not queried
 
  0.7066830
 
       NA
 
       NA
  
 
  
 5
 
 176326365
 
 176326619
 
  ENSEMBL   UCSC 
 
  255
 
  4
 
 32459
 
 tss
 
 0.67440
 
 0.70286
 
 Not queried
 
  0.7066070
 
       NA
 
       NA
  
 
  
 1
 
  10092629
 
  10092702
 
  ENSEMBL   UCSC 
 
   74
 
  4
 
 1231
 
 tss;island
 
 0.03641
 
 0.06245
 
 Not queried
 
  0.7062626
 
       NA
 
       NA
  
 
  
 12
 
  27396706
 
  27397070
 
  ENSEMBL   UCSC 
 
  365
 
  8
 
 9594
 
 tss
 
 0.54651
 
 0.63940
 
 Not queried
 
  0.7062460
 
       NA
 
       NA
  
 
  
 5
 
  65892993
 
  65893538
 
  ENSEMBL   UCSC 
 
  546
 
  5
 
 31578
 
 gene;island
 
 0.04852
 
 0.06183
 
 Not queried
 
  0.7059928
 
       NA
 
       NA
  
 
  
 1
 
 230414987
 
 230415668
 
  ENSEMBL   UCSC 
 
  682
 
  7
 
 1067
 
 gene;island
 
 0.80858
 
 0.90813
 
 Not queried
 
  0.7057533
 
       NA
 
       NA
  
 
  
 3
 
 126373634
 
 126374304
 
  ENSEMBL   UCSC 
 
  671
 
  6
 
 29392
 
 island
 
 0.03280
 
 0.04312
 
 Not queried
 
  0.7057504
 
       NA
 
       NA
  
 
  
 2
 
 121493807
 
 121494043
 
  ENSEMBL   UCSC 
 
  237
 
  5
 
 24631
 
 island
 
 0.01294
 
 0.02054
 
 Not queried
 
  0.7057437
 
       NA
 
       NA
  
 
  
 21
 
  14410329
 
  14410566
 
  ENSEMBL   UCSC 
 
  238
 
  4
 
 26563
 
 island
 
 0.40679
 
 0.53203
 
 Not queried
 
  0.7056062
 
       NA
 
       NA
  
 
  
 13
 
 114875001
 
 114875412
 
  ENSEMBL   UCSC 
 
  412
 
  5
 
 11286
 
 gene;island
 
 0.73063
 
 0.83425
 
 Not queried
 
  0.7055279
 
       NA
 
       NA
  
 
  
 15
 
  65822858
 
  65823204
 
  ENSEMBL   UCSC 
 
  347
 
  5
 
 13353
 
 gene
 
 0.02689
 
 0.03846
 
 Not queried
 
  0.7054893
 
       NA
 
       NA
  
 
  
 20
 
   4702381
 
   4702538
 
  ENSEMBL   UCSC 
 
  158
 
  4
 
 25536
 
 tss
 
 0.55312
 
 0.65515
 
 Not queried
 
  0.7054795
 
       NA
 
       NA
  
 
  
 19
 
  55866305
 
  55866412
 
  ENSEMBL   UCSC 
 
  108
 
  5
 
 20190
 
 gene
 
 0.04513
 
 0.08280
 
 Not queried
 
  0.7054231
 
       NA
 
       NA
  
 
  
 2
 
  65454430
 
  65454775
 
  ENSEMBL   UCSC 
 
  346
 
  9
 
 23358
 
 tss
 
 0.02434
 
 0.02869
 
 Not queried
 
  0.7053760
 
       NA
 
       NA
  
 
  
 20
 
    825050
 
    825268
 
  ENSEMBL   UCSC 
 
  219
 
  5
 
 25491
 
 tss
 
 0.01677
 
 0.02099
 
 Not queried
 
  0.7049551
 
       NA
 
       NA
  
 
  
 1
 
 154744644
 
 154744863
 
  ENSEMBL   UCSC 
 
  220
 
  4
 
 782
 
 gene;island
 
 0.85399
 
 0.78371
 
 Not queried
 
  0.7049001
 
       NA
 
       NA
  
 
  
 17
 
  65040527
 
  65040616
 
  ENSEMBL   UCSC 
 
   90
 
  6
 
 17563
 
 tss
 
 0.85481
 
 0.90108
 
 Not queried
 
  0.7048783
 
       NA
 
       NA
  
 
  
 6
 
 127587654
 
 127587940
 
  ENSEMBL   UCSC 
 
  287
 
  7
 
 35151
 
 tss
 
 0.00917
 
 0.02613
 
 Not queried
 
  0.7046515
 
       NA
 
       NA
  
 
  
 4
 
 141489799
 
 141490428
 
  ENSEMBL   UCSC 
 
  630
 
 12
 
 31273
 
 island
 
 0.04603
 
 0.02187
 
 Not queried
 
  0.7046032
 
       NA
 
       NA
  
 
  
 2
 
  74757141
 
  74757221
 
  ENSEMBL   UCSC 
 
   81
 
  5
 
 22712
 
 gene;tss;island
 
 0.01187
 
 0.03395
 
 Not queried
 
  0.7045321
 
       NA
 
       NA
  
 
  
 4
 
 109571402
 
 109571724
 
  ENSEMBL   UCSC 
 
  323
 
  6
 
 30493
 
 tss
 
 0.03198
 
 0.05658
 
 Not queried
 
  0.7044588
 
       NA
 
       NA
  
 
  
 21
 
  40752339
 
  40752891
 
  ENSEMBL   UCSC 
 
  553
 
  5
 
 26386
 
 gene
 
 0.04465
 
 0.02727
 
 Not queried
 
  0.7043792
 
       NA
 
       NA
  
 
  
 15
 
  56035371
 
  56035616
 
  ENSEMBL   UCSC 
 
  246
 
  6
 
 13657
 
 tss;island
 
 0.02552
 
 0.03748
 
 Not queried
 
  0.7043608
 
       NA
 
       NA
  
 
  
 10
 
  70748213
 
  70748649
 
  ENSEMBL   UCSC 
 
  437
 
 11
 
 5782
 
 island
 
 0.01786
 
 0.03181
 
 Not queried
 
  0.7042619
 
       NA
 
       NA
  
 
  
 2
 
  31805915
 
  31806551
 
  ENSEMBL   UCSC 
 
  637
 
  8
 
 24167
 
 island
 
 0.54576
 
 0.59586
 
 Not queried
 
  0.7042498
 
       NA
 
       NA
  
 
  
 1
 
 203320386
 
 203320541
 
  ENSEMBL   UCSC 
 
  156
 
  4
 
 2188
 
 tss
 
 0.49044
 
 0.42450
 
 Not queried
 
  0.7042235
 
       NA
 
       NA
  
 
  
 10
 
  44701885
 
  44702076
 
  ENSEMBL   UCSC 
 
  192
 
  4
 
 5687
 
 island
 
 0.91196
 
 0.86440
 
 Not queried
 
  0.7041875
 
       NA
 
       NA
  
 
  
 6
 
  32822857
 
  32823292
 
  ENSEMBL   UCSC 
 
  436
 
  5
 
 34092
 
 gene;island
 
 0.42570
 
 0.50189
 
 Not queried
 
  0.7040944
 
       NA
 
       NA
  
 
  
 15
 
  93199037
 
  93199180
 
  ENSEMBL   UCSC 
 
  144
 
  5
 
 13884
 
 tss
 
 0.05008
 
 0.04229
 
 Not queried
 
  0.7040901
 
       NA
 
       NA
  
 
  
 12
 
  52995358
 
  52995634
 
  ENSEMBL   UCSC 
 
  277
 
  4
 
 9704
 
 tss
 
 0.67667
 
 0.59381
 
 Not queried
 
  0.7039888
 
       NA
 
       NA
  
 
  
 1
 
 179545091
 
 179545458
 
  ENSEMBL   UCSC 
 
  368
 
  8
 
 2114
 
 tss
 
 0.07173
 
 0.03411
 
 Not queried
 
  0.7039809
 
       NA
 
       NA
  
 
  
 5
 
 138628911
 
 138629210
 
  ENSEMBL   UCSC 
 
  300
 
  8
 
 31715
 
 gene;tss;island
 
 0.00713
 
 0.01430
 
 Not queried
 
  0.7039407
 
       NA
 
       NA
  
 
  
 4
 
  39978128
 
  39978343
 
  ENSEMBL   UCSC 
 
  216
 
  4
 
 29939
 
 gene;island
 
 0.02241
 
 0.02935
 
 Not queried
 
  0.7038884
 
       NA
 
       NA
  
 
  
 20
 
  62492074
 
  62492380
 
  ENSEMBL   UCSC 
 
  307
 
  4
 
 25864
 
 tss;island
 
 0.58999
 
 0.70157
 
 Not queried
 
  0.7038634
 
       NA
 
       NA
  
 
  
 10
 
  79686265
 
  79686444
 
  ENSEMBL   UCSC 
 
  180
 
  5
 
 5161
 
 tss;island
 
 0.03501
 
 0.05580
 
 Not queried
 
  0.7038373
 
       NA
 
       NA
  
 
  
 1
 
  17338581
 
  17338766
 
  ENSEMBL   UCSC 
 
  186
 
  6
 
 1286
 
 tss
 
 0.04075
 
 0.07214
 
 Not queried
 
  0.7037911
 
       NA
 
       NA
  
 
  
 22
 
  38218945
 
  38219400
 
  ENSEMBL   UCSC 
 
  456
 
  5
 
 27433
 
 island
 
 0.57548
 
 0.68204
 
 Not queried
 
  0.7037857
 
       NA
 
       NA
  
 
  
 2
 
   3605707
 
   3606331
 
  ENSEMBL   UCSC 
 
  625
 
 12
 
 23995
 
 island
 
 0.02353
 
 0.03601
 
 Not queried
 
  0.7037765
 
       NA
 
       NA
  
 
  
 10
 
  28591940
 
  28592238
 
  ENSEMBL   UCSC 
 
  299
 
  4
 
 5619
 
 island
 
 0.01872
 
 0.02514
 
 Not queried
 
  0.7037019
 
       NA
 
       NA
  
 
  
 1
 
  77747875
 
  77748221
 
  ENSEMBL   UCSC 
 
  347
 
  6
 
 1702
 
 tss
 
 0.02509
 
 0.03322
 
 Not queried
 
  0.7036336
 
       NA
 
       NA
  
 
  
 4
 
  89205891
 
  89206201
 
  ENSEMBL   UCSC 
 
  311
 
  7
 
 30444
 
 tss
 
 0.01635
 
 0.03831
 
 Not queried
 
  0.7036177
 
       NA
 
       NA
  
 
  
 15
 
  78441442
 
  78441695
 
  ENSEMBL   UCSC 
 
  254
 
  7
 
 13802
 
 tss
 
 0.01328
 
 0.01681
 
 Not queried
 
  0.7035967
 
       NA
 
       NA
  
 
  
 3
 
 107241636
 
 107241928
 
  ENSEMBL   UCSC 
 
  293
 
  4
 
 29302
 
 island
 
 0.03523
 
 0.05679
 
 Not queried
 
  0.7034908
 
       NA
 
       NA
  
 
  
 15
 
  25123287
 
  25123731
 
  ENSEMBL   UCSC 
 
  445
 
  6
 
 13227
 
 gene;island
 
 0.61252
 
 0.67688
 
 Not queried
 
  0.7034521
 
       NA
 
       NA
  
 
  
 15
 
  75182130
 
  75182373
 
  ENSEMBL   UCSC 
 
  244
 
  5
 
 13776
 
 tss
 
 0.05346
 
 0.08421
 
 Not queried
 
  0.7033643
 
       NA
 
       NA
  
 
  
 15
 
  75182130
 
  75182590
 
  ENSEMBL   UCSC 
 
  461
 
  9
 
 14291
 
 island
 
 0.03538
 
 0.04087
 
 Not queried
 
  0.7033643
 
       NA
 
       NA
  
 
  
 1
 
  92352293
 
  92352481
 
  ENSEMBL   UCSC 
 
  189
 
  4
 
 1740
 
 tss;island
 
 0.03119
 
 0.03206
 
 Not queried
 
  0.7032972
 
       NA
 
       NA
  
 
  
 12
 
 111834929
 
 111835267
 
  ENSEMBL   UCSC 
 
  339
 
  4
 
 10781
 
 island
 
 0.03638
 
 0.05727
 
 Not queried
 
  0.7031942
 
       NA
 
       NA
  
 
  
 11
 
 107436281
 
 107436442
 
  ENSEMBL   UCSC 
 
  162
 
  4
 
 6864
 
 gene
 
 0.01379
 
 0.00923
 
 Not queried
 
  0.7031732
 
       NA
 
       NA
  
 
  
 5
 
 177026865
 
 177027175
 
  ENSEMBL   UCSC 
 
  311
 
  6
 
 33395
 
 island
 
 0.03453
 
 0.04855
 
 Not queried
 
  0.7030808
 
       NA
 
       NA
  
 
  
 16
 
  30022548
 
  30022725
 
  ENSEMBL   UCSC 
 
  178
 
  7
 
 15144
 
 tss;island
 
 0.01162
 
 0.02432
 
 Not queried
 
  0.7030068
 
       NA
 
       NA
  
 
  
 7
 
  23510005
 
  23510123
 
  ENSEMBL   UCSC 
 
  119
 
  5
 
 37407
 
 tss;island
 
 0.01919
 
 0.03770
 
 Not queried
 
  0.7029671
 
       NA
 
       NA
  
 
  
 1
 
 149859456
 
 149859690
 
  ENSEMBL   UCSC 
 
  235
 
  5
 
 1895
 
 tss;island
 
 0.00519
 
 0.00907
 
 Not queried
 
  0.7029132
 
       NA
 
       NA
  
 
  
 1
 
  17381800
 
  17382130
 
  ENSEMBL   UCSC 
 
  331
 
  4
 
 1287
 
 tss;island
 
 0.63179
 
 0.78980
 
 Not queried
 
  0.7028981
 
       NA
 
       NA
  
 
  
 10
 
   8095121
 
   8095363
 
  ENSEMBL   UCSC 
 
  243
 
  4
 
 4475
 
 gene
 
 0.05160
 
 0.04887
 
 Not queried
 
  0.7027929
 
       NA
 
       NA
  
 
  
 5
 
 137688057
 
 137688433
 
  ENSEMBL   UCSC 
 
  377
 
  6
 
 33096
 
 island
 
 0.06500
 
 0.08474
 
 Not queried
 
  0.7026099
 
       NA
 
       NA
  
 
  
 3
 
  27755970
 
  27756421
 
  ENSEMBL   UCSC 
 
  452
 
  6
 
 28905
 
 island
 
 0.04843
 
 0.06778
 
 Not queried
 
  0.7026061
 
       NA
 
       NA
  
 
  
 22
 
  41777504
 
  41777896
 
  ENSEMBL   UCSC 
 
  393
 
  9
 
 26841
 
 gene;tss;island
 
 0.01971
 
 0.03282
 
 Not queried
 
  0.7025678
 
       NA
 
       NA
  
 
  
 6
 
 144328917
 
 144330345
 
  ENSEMBL   UCSC 
 
 1429
 
 22
 
 34433
 
 gene;island
 
 0.12151
 
 0.05156
 
 Not queried
 
  0.7024603
 
       NA
 
       NA
  
 
  
 3
 
 142297737
 
 142298018
 
  ENSEMBL   UCSC 
 
  282
 
  9
 
 28598
 
 tss
 
 0.02015
 
 0.03130
 
 Not queried
 
  0.7024384
 
       NA
 
       NA
  
 
  
 3
 
 142297635
 
 142298018
 
  ENSEMBL   UCSC 
 
  384
 
 11
 
 29508
 
 island
 
 0.02015
 
 0.03130
 
 Not queried
 
  0.7024384
 
       NA
 
       NA
  
 
  
 6
 
  10694545
 
  10694962
 
  ENSEMBL   UCSC 
 
  418
 
  9
 
 33500
 
 gene
 
 0.00986
 
 0.02622
 
 Not queried
 
  0.7024173
 
       NA
 
       NA
  
 
  
 3
 
 195942993
 
 195943366
 
  ENSEMBL   UCSC 
 
  374
 
  5
 
 28766
 
 tss
 
 0.70243
 
 0.73727
 
 Not queried
 
  0.7023056
 
       NA
 
       NA
  
 
  
 7
 
   6098733
 
   6099250
 
  ENSEMBL   UCSC 
 
  518
 
 11
 
 38175
 
 island
 
 0.01183
 
 0.02265
 
 Not queried
 
  0.7022231
 
       NA
 
       NA
  
 
  
 X
 
   3264341
 
   3265089
 
  ENSEMBL   UCSC 
 
  749
 
 12
 
 41619
 
 island
 
 0.02244
 
 0.03892
 
 Not queried
 
  0.7022102
 
       NA
 
       NA
  
 
  
 6
 
 130182452
 
 130182620
 
  ENSEMBL   UCSC 
 
  169
 
  6
 
 35156
 
 tss
 
 0.95417
 
 0.92148
 
 Not queried
 
  0.7021862
 
       NA
 
       NA
  
 
  
 11
 
  69259788
 
  69260314
 
  ENSEMBL   UCSC 
 
  527
 
  6
 
 8566
 
 island
 
 0.74926
 
 0.85368
 
 Not queried
 
  0.7021060
 
       NA
 
       NA
  
 
  
 4
 
 106395029
 
 106395643
 
  ENSEMBL   UCSC 
 
  615
 
 10
 
 31167
 
 island
 
 0.02281
 
 0.03426
 
 Not queried
 
  0.7018697
 
       NA
 
       NA
  
 
  
 1
 
 156783633
 
 156784152
 
  ENSEMBL   UCSC 
 
  520
 
  6
 
 829
 
 gene;island
 
 0.06914
 
 0.04247
 
 Not queried
 
  0.7018485
 
       NA
 
       NA
  
 
  
 11
 
  63741847
 
  63742144
 
  ENSEMBL   UCSC 
 
  298
 
  9
 
 8356
 
 island
 
 0.01369
 
 0.02252
 
 Not queried
 
  0.7017604
 
       NA
 
       NA
  
 
  
 17
 
  79108051
 
  79108344
 
  ENSEMBL   UCSC 
 
  294
 
  6
 
 16863
 
 gene;tss;island
 
 0.83464
 
 0.90265
 
 Not queried
 
  0.7017326
 
       NA
 
       NA
  
 
  
 1
 
  54665846
 
  54666401
 
  ENSEMBL   UCSC 
 
  556
 
  8
 
 546
 
 gene
 
 0.01282
 
 0.02289
 
 Not queried
 
  0.7017235
 
       NA
 
       NA
  
 
  
 17
 
  37886349
 
  37887061
 
  ENSEMBL   UCSC 
 
  713
 
 10
 
 18255
 
 island
 
 0.01537
 
 0.02833
 
 Not queried
 
  0.7016782
 
       NA
 
       NA
  
 
  
 11
 
   1774722
 
   1775039
 
  ENSEMBL   UCSC 
 
  318
 
  4
 
 6413
 
 gene;island
 
 0.79791
 
 0.84403
 
 Not queried
 
  0.7015449
 
       NA
 
       NA
  
 
  
 13
 
  37248078
 
  37248423
 
  ENSEMBL   UCSC 
 
  346
 
  4
 
 11105
 
 gene;island
 
 0.01772
 
 0.02809
 
 Not queried
 
  0.7015075
 
       NA
 
       NA
  
 
  
 11
 
  31832879
 
  31833264
 
  ENSEMBL   UCSC 
 
  386
 
  8
 
 7203
 
 tss
 
 0.05301
 
 0.02512
 
 Not queried
 
  0.7014848
 
       NA
 
       NA
  
 
  
 15
 
  86338211
 
  86338428
 
  ENSEMBL   UCSC 
 
  218
 
  9
 
 13848
 
 tss
 
 0.02106
 
 0.03058
 
 Not queried
 
  0.7013822
 
       NA
 
       NA
  
 
  
 15
 
  86338090
 
  86338428
 
  ENSEMBL   UCSC 
 
  339
 
 11
 
 14386
 
 island
 
 0.02106
 
 0.03125
 
 Not queried
 
  0.7013822
 
       NA
 
       NA
  
 
  
 11
 
  74303294
 
  74303539
 
  ENSEMBL   UCSC 
 
  246
 
  6
 
 7539
 
 tss
 
 0.03580
 
 0.02436
 
 Not queried
 
  0.7013730
 
       NA
 
       NA
  
 
  
 17
 
  46681111
 
  46681401
 
  ENSEMBL   UCSC 
 
  291
 
  6
 
 16621
 
 gene
 
 0.35878
 
 0.40786
 
 Not queried
 
  0.7013688
 
       NA
 
       NA
  
 
  
 1
 
 149144820
 
 149145243
 
  ENSEMBL   UCSC 
 
  424
 
  5
 
 3693
 
 island
 
 0.62689
 
 0.75556
 
 Not queried
 
  0.7013203
 
       NA
 
       NA
  
 
  
 3
 
  52279902
 
  52280329
 
  ENSEMBL   UCSC 
 
  428
 
  6
 
 29138
 
 island
 
 0.04594
 
 0.07654
 
 Not queried
 
  0.7012154
 
       NA
 
       NA
  
 
  
 1
 
 151138249
 
 151138408
 
  ENSEMBL   UCSC 
 
  160
 
  5
 
 747
 
 gene
 
 0.02348
 
 0.03764
 
 Not queried
 
  0.7010699
 
       NA
 
       NA
  
 
  
 1
 
 151138249
 
 151138583
 
  ENSEMBL   UCSC 
 
  335
 
 11
 
 1919
 
 tss
 
 0.02009
 
 0.03328
 
 Not queried
 
  0.7010699
 
       NA
 
       NA
  
 
  
 6
 
  25652381
 
  25652407
 
  ENSEMBL   UCSC 
 
   27
 
  6
 
 34610
 
 tss
 
 0.05802
 
 0.09836
 
 Not queried
 
  0.7010414
 
       NA
 
       NA
  
 
  
 6
 
  25652381
 
  25652815
 
  ENSEMBL   UCSC 
 
  435
 
 10
 
 35436
 
 island
 
 0.04923
 
 0.08304
 
 Not queried
 
  0.7010414
 
       NA
 
       NA
  
 
  
 6
 
  90062296
 
  90062885
 
  ENSEMBL   UCSC 
 
  590
 
  8
 
 36259
 
 island
 
 0.01161
 
 0.03243
 
 Not queried
 
  0.7009934
 
       NA
 
       NA
  
 
  
 3
 
 112931126
 
 112931354
 
  ENSEMBL   UCSC 
 
  229
 
  6
 
 28462
 
 tss
 
 0.02816
 
 0.04258
 
 Not queried
 
  0.7008967
 
       NA
 
       NA
  
 
  
 20
 
   5591818
 
   5591950
 
  ENSEMBL   UCSC 
 
  133
 
  7
 
 25545
 
 tss
 
 0.02826
 
 0.04089
 
 Not queried
 
  0.7007877
 
       NA
 
       NA
  
 
  
 8
 
 145025059
 
 145025178
 
  ENSEMBL   UCSC 
 
  120
 
  5
 
 39902
 
 tss
 
 0.79516
 
 0.90493
 
 Not queried
 
  0.7007710
 
       NA
 
       NA
  
 
  
 5
 
 172483354
 
 172483448
 
  ENSEMBL   UCSC 
 
   95
 
  4
 
 31842
 
 gene;island
 
 0.02890
 
 0.03183
 
 Not queried
 
  0.7007457
 
       NA
 
       NA
  
 
  
 2
 
  96012586
 
  96012803
 
  ENSEMBL   UCSC 
 
  218
 
  5
 
 22739
 
 gene;island
 
 0.15097
 
 0.10675
 
 Not queried
 
  0.7006853
 
       NA
 
       NA
  
 
  
 21
 
  32624145
 
  32624306
 
  ENSEMBL   UCSC 
 
  162
 
  4
 
 26355
 
 gene;island
 
 0.91866
 
 0.86510
 
 Not queried
 
  0.7006687
 
       NA
 
       NA
  
 
  
 2
 
  27069154
 
  27069408
 
  ENSEMBL   UCSC 
 
  255
 
  4
 
 24122
 
 island
 
 0.01345
 
 0.03090
 
 Not queried
 
  0.7006579
 
       NA
 
       NA
  
 
  
 4
 
   1577932
 
   1578221
 
  ENSEMBL   UCSC 
 
  290
 
  6
 
 30743
 
 island
 
 0.93343
 
 0.94088
 
 Not queried
 
  0.7006478
 
       NA
 
       NA
  
 
  
 7
 
 100086099
 
 100086445
 
  ENSEMBL   UCSC 
 
  347
 
  4
 
 37078
 
 gene;island
 
 0.88541
 
 0.91874
 
 Not queried
 
  0.7005900
 
       NA
 
       NA
  
 
  
 20
 
  57607406
 
  57607775
 
  ENSEMBL   UCSC 
 
  370
 
 10
 
 25819
 
 tss
 
 0.01851
 
 0.02653
 
 Not queried
 
  0.7005032
 
       NA
 
       NA
  
 
  
 19
 
  44037015
 
  44037336
 
  ENSEMBL   UCSC 
 
  322
 
  7
 
 20794
 
 tss;island
 
 0.01242
 
 0.01948
 
 Not queried
 
  0.7004377
 
       NA
 
       NA
  
 
  
 2
 
  55509105
 
  55509779
 
  ENSEMBL   UCSC 
 
  675
 
  8
 
 24271
 
 island
 
 0.26825
 
 0.41836
 
 Not queried
 
  0.7004359
 
       NA
 
       NA
  
 
  
 1
 
  92949337
 
  92950836
 
  ENSEMBL   UCSC 
 
 1500
 
 31
 
 634
 
 gene;island
 
 0.02455
 
 0.03595
 
 Not queried
 
  0.7004278
 
       NA
 
       NA
  
 
  
 1
 
  92949447
 
  92950836
 
  ENSEMBL   UCSC 
 
 1390
 
 29
 
 1748
 
 tss
 
 0.02455
 
 0.03595
 
 Not queried
 
  0.7004278
 
       NA
 
       NA
  
 
  
 8
 
 114450065
 
 114450347
 
  ENSEMBL   UCSC 
 
  283
 
  4
 
 39813
 
 tss
 
 0.02436
 
 0.02695
 
 Not queried
 
  0.7003026
 
       NA
 
       NA
  
 
  
 1
 
  21059189
 
  21059745
 
  ENSEMBL   UCSC 
 
  557
 
 10
 
 1315
 
 tss
 
 0.09482
 
 0.12763
 
 Not queried
 
  0.7002127
 
       NA
 
       NA
  
 
  
 1
 
  21058966
 
  21059745
 
  ENSEMBL   UCSC 
 
  780
 
 12
 
 2797
 
 island
 
 0.08533
 
 0.12179
 
 Not queried
 
  0.7002127
 
       NA
 
       NA
  
 
  
 20
 
  25039658
 
  25039812
 
  ENSEMBL   UCSC 
 
  155
 
  5
 
 25605
 
 tss;island
 
 0.27806
 
 0.33749
 
 Not queried
 
  0.7001183
 
       NA
 
       NA
  
 
  
 19
 
   6768010
 
   6768251
 
  ENSEMBL   UCSC 
 
  242
 
  4
 
 20358
 
 tss;island
 
 0.28500
 
 0.35070
 
 Not queried
 
  0.7000379
 
       NA
 
       NA
  
 
  
 19
 
  11038957
 
  11039319
 
  ENSEMBL   UCSC 
 
  363
 
  7
 
 19789
 
 gene
 
 0.02648
 
 0.03537
 
 Not queried
 
  0.6999393
 
       NA
 
       NA
  
 
  
 1
 
 113258059
 
 113258477
 
  ENSEMBL   UCSC 
 
  419
 
  7
 
 1836
 
 tss
 
 0.01109
 
 0.03675
 
 Not queried
 
  0.6999266
 
       NA
 
       NA
  
 
  
 10
 
  95462161
 
  95462699
 
  ENSEMBL   UCSC 
 
  539
 
 15
 
 5938
 
 island
 
 0.01759
 
 0.02157
 
 Not queried
 
  0.6999085
 
       NA
 
       NA
  
 
  
 3
 
  57678924
 
  57679146
 
  ENSEMBL   UCSC 
 
  223
 
  7
 
 28368
 
 tss;island
 
 0.01515
 
 0.07007
 
 Not queried
 
  0.6998491
 
       NA
 
       NA
  
 
  
 1
 
   1476551
 
   1477194
 
  ENSEMBL   UCSC 
 
  644
 
  7
 
 1152
 
 tss;island
 
 0.73303
 
 0.85976
 
 Not queried
 
  0.6998457
 
       NA
 
       NA
  
 
  
 4
 
   1800154
 
   1800448
 
  ENSEMBL   UCSC 
 
  295
 
  4
 
 29830
 
 gene;island
 
 0.91902
 
 0.93973
 
 Not queried
 
  0.6995742
 
       NA
 
       NA
  
 
  
 16
 
  67194867
 
  67195069
 
  ENSEMBL   UCSC 
 
  203
 
  4
 
 14759
 
 gene;tss;island
 
 0.02282
 
 0.01615
 
 Not queried
 
  0.6995138
 
       NA
 
       NA
  
 
  
 5
 
  95297792
 
  95298307
 
  ENSEMBL   UCSC 
 
  516
 
  7
 
 32129
 
 tss
 
 0.02544
 
 0.04347
 
 Not queried
 
  0.6994674
 
       NA
 
       NA
  
 
  
 2
 
 178129680
 
 178130284
 
  ENSEMBL   UCSC 
 
  605
 
  8
 
 23709
 
 tss;island
 
 0.02656
 
 0.02668
 
 Not queried
 
  0.6994229
 
       NA
 
       NA
  
 
  
 8
 
  95835263
 
  95835351
 
  ENSEMBL   UCSC 
 
   89
 
  4
 
 39756
 
 tss
 
 0.00687
 
 0.01121
 
 Not queried
 
  0.6993791
 
       NA
 
       NA
  
 
  
 16
 
    834043
 
    834510
 
  ENSEMBL   UCSC 
 
  468
 
  4
 
 15456
 
 island
 
 0.87837
 
 0.94453
 
 Not queried
 
  0.6992053
 
       NA
 
       NA
  
 
  
 5
 
  54529624
 
  54530436
 
  ENSEMBL   UCSC 
 
  813
 
 10
 
 32006
 
 tss
 
 0.01983
 
 0.03375
 
 Not queried
 
  0.6991352
 
       NA
 
       NA
  
 
  
 8
 
  95487349
 
  95487732
 
  ENSEMBL   UCSC 
 
  384
 
  9
 
 39751
 
 tss
 
 0.01560
 
 0.01966
 
 Not queried
 
  0.6991197
 
       NA
 
       NA
  
 
  
 15
 
 101835872
 
 101836060
 
  ENSEMBL   UCSC 
 
  189
 
  4
 
 13906
 
 tss;island
 
 0.03170
 
 0.01447
 
 Not queried
 
  0.6990193
 
       NA
 
       NA
  
 
  
 17
 
  79670048
 
  79670405
 
  ENSEMBL   UCSC 
 
  358
 
  7
 
 17714
 
 tss
 
 0.01567
 
 0.03026
 
 Not queried
 
  0.6989822
 
       NA
 
       NA
  
 
  
 4
 
   8081109
 
   8081484
 
  ENSEMBL   UCSC 
 
  376
 
  4
 
 29900
 
 gene
 
 0.84507
 
 0.91567
 
 Not queried
 
  0.6988771
 
       NA
 
       NA
  
 
  
 1
 
  20126168
 
  20126875
 
  ENSEMBL   UCSC 
 
  708
 
 10
 
 2781
 
 island
 
 0.03103
 
 0.05233
 
 Not queried
 
  0.6988454
 
       NA
 
       NA
  
 
  
 X
 
  23017887
 
  23018057
 
  ENSEMBL   UCSC 
 
  171
 
  7
 
 41225
 
 tss
 
 0.89075
 
 0.94195
 
 Not queried
 
  0.6986124
 
       NA
 
       NA
  
 
  
 X
 
  24482846
 
  24483294
 
  ENSEMBL   UCSC 
 
  449
 
  9
 
 41231
 
 tss
 
 0.02308
 
 0.01863
 
 Not queried
 
  0.6985391
 
       NA
 
       NA
  
 
  
 10
 
  92979939
 
  92980276
 
  ENSEMBL   UCSC 
 
  338
 
  7
 
 5204
 
 tss
 
 0.05951
 
 0.07699
 
 Not queried
 
  0.6985371
 
       NA
 
       NA
  
 
  
 7
 
  27224092
 
  27224795
 
  ENSEMBL   UCSC 
 
  704
 
  8
 
 36878
 
 gene
 
 0.01828
 
 0.03957
 
 Not queried
 
  0.6984498
 
       NA
 
       NA
  
 
  
 19
 
  44488121
 
  44488342
 
  ENSEMBL   UCSC 
 
  222
 
  8
 
 20802
 
 tss
 
 0.02611
 
 0.03983
 
 Not queried
 
  0.6984303
 
       NA
 
       NA
  
 
  
 7
 
 106505688
 
 106505961
 
  ENSEMBL   UCSC 
 
  274
 
  5
 
 38761
 
 island
 
 0.88012
 
 0.92256
 
 Not queried
 
  0.6984187
 
       NA
 
       NA
  
 
  
 5
 
    443432
 
    443625
 
  ENSEMBL   UCSC 
 
  194
 
  6
 
 31449
 
 gene;tss;island
 
 0.02231
 
 0.03894
 
 Not queried
 
  0.6981695
 
       NA
 
       NA
  
 
  
 15
 
  50716270
 
  50716372
 
  ENSEMBL   UCSC 
 
  103
 
  4
 
 13641
 
 tss;island
 
 0.04479
 
 0.03636
 
 Not queried
 
  0.6980737
 
       NA
 
       NA
  
 
  
 4
 
 101111147
 
 101111427
 
  ENSEMBL   UCSC 
 
  281
 
  4
 
 30023
 
 gene
 
 0.01299
 
 0.02381
 
 Not queried
 
  0.6980662
 
       NA
 
       NA
  
 
  
 20
 
   3214756
 
   3214926
 
  ENSEMBL   UCSC 
 
  171
 
  4
 
 25309
 
 gene;island
 
 0.91360
 
 0.87404
 
 Not queried
 
  0.6980245
 
       NA
 
       NA
  
 
  
 1
 
   8002302
 
   8002698
 
  ENSEMBL   UCSC 
 
  397
 
  5
 
 2624
 
 island
 
 0.05434
 
 0.03813
 
 Not queried
 
  0.6979839
 
       NA
 
       NA
  
 
  
 22
 
  25615317
 
  25615557
 
  ENSEMBL   UCSC 
 
  241
 
  8
 
 26987
 
 tss
 
 0.69130
 
 0.78893
 
 Not queried
 
  0.6978652
 
       NA
 
       NA
  
 
  
 1
 
  47697429
 
  47697663
 
  ENSEMBL   UCSC 
 
  235
 
  4
 
 3225
 
 island
 
 0.08496
 
 0.04647
 
 Not queried
 
  0.6978479
 
       NA
 
       NA
  
 
  
 5
 
 131993614
 
 131993853
 
  ENSEMBL   UCSC 
 
  240
 
  4
 
 32214
 
 tss
 
 0.92475
 
 0.84282
 
 Not queried
 
  0.6977742
 
       NA
 
       NA
  
 
  
 10
 
  95721181
 
  95721442
 
  ENSEMBL   UCSC 
 
  262
 
  4
 
 4670
 
 gene
 
 0.51497
 
 0.29200
 
 Not queried
 
  0.6977593
 
       NA
 
       NA
  
 
  
 5
 
 176778888
 
 176779229
 
  ENSEMBL   UCSC 
 
  342
 
  9
 
 32464
 
 tss
 
 0.01214
 
 0.02123
 
 Not queried
 
  0.6977358
 
       NA
 
       NA
  
 
  
 8
 
 143823734
 
 143824001
 
  ENSEMBL   UCSC 
 
  268
 
  5
 
 40577
 
 island
 
 0.68855
 
 0.57684
 
 Not queried
 
  0.6976354
 
       NA
 
       NA
  
 
  
 1
 
 161953593
 
 161953982
 
  ENSEMBL   UCSC 
 
  390
 
  4
 
 855
 
 gene;island
 
 0.60024
 
 0.76461
 
 Not queried
 
  0.6976091
 
       NA
 
       NA
  
 
  
 11
 
  60674132
 
  60674473
 
  ENSEMBL   UCSC 
 
  342
 
 10
 
 7303
 
 tss;island
 
 0.02006
 
 0.02657
 
 Not queried
 
  0.6976038
 
       NA
 
       NA
  
 
  
 3
 
  33481897
 
  33481969
 
  ENSEMBL   UCSC 
 
   73
 
  4
 
 28172
 
 tss
 
 0.09714
 
 0.15607
 
 Not queried
 
  0.6975995
 
       NA
 
       NA
  
 
  
 8
 
  17103891
 
  17104535
 
  ENSEMBL   UCSC 
 
  645
 
  8
 
 39152
 
 gene;tss;island
 
 0.01625
 
 0.02664
 
 Not queried
 
  0.6975374
 
       NA
 
       NA
  
 
  
 12
 
  41085901
 
  41086365
 
  ENSEMBL   UCSC 
 
  465
 
  9
 
 10301
 
 island
 
 0.02586
 
 0.03982
 
 Not queried
 
  0.6974435
 
       NA
 
       NA
  
 
  
 3
 
 190105524
 
 190105821
 
  ENSEMBL   UCSC 
 
  298
 
  8
 
 28742
 
 tss
 
 0.46386
 
 0.64620
 
 Not queried
 
  0.6973518
 
       NA
 
       NA
  
 
  
 1
 
  15479095
 
  15479474
 
  ENSEMBL   UCSC 
 
  380
 
  4
 
 242
 
 gene
 
 0.04206
 
 0.04106
 
 Not queried
 
  0.6972418
 
       NA
 
       NA
  
 
  
 11
 
  59328249
 
  59328586
 
  ENSEMBL   UCSC 
 
  338
 
  4
 
 8284
 
 island
 
 0.02979
 
 0.04522
 
 Not queried
 
  0.6971900
 
       NA
 
       NA
  
 
  
 8
 
 145619488
 
 145619719
 
  ENSEMBL   UCSC 
 
  232
 
  5
 
 39455
 
 gene;tss;island
 
 0.82623
 
 0.91255
 
 Not queried
 
  0.6971662
 
       NA
 
       NA
  
 
  
 14
 
 105045877
 
 105045992
 
  ENSEMBL   UCSC 
 
  116
 
  4
 
 12631
 
 tss
 
 0.63164
 
 0.70518
 
 Not queried
 
  0.6970858
 
       NA
 
       NA
  
 
  
 4
 
 176922556
 
 176923081
 
  ENSEMBL   UCSC 
 
  526
 
  5
 
 30138
 
 gene;island
 
 0.01640
 
 0.02875
 
 Not queried
 
  0.6968947
 
       NA
 
       NA
  
 
  
 6
 
  53530538
 
  53530628
 
  ENSEMBL   UCSC 
 
   91
 
  5
 
 34977
 
 tss
 
 0.31469
 
 0.23985
 
 Not queried
 
  0.6967022
 
       NA
 
       NA
  
 
  
 1
 
 155270866
 
 155271211
 
  ENSEMBL   UCSC 
 
  346
 
  5
 
 799
 
 gene;tss
 
 0.80529
 
 0.71084
 
 Not queried
 
  0.6966605
 
       NA
 
       NA
  
 
  
 16
 
   4802469
 
   4802990
 
  ENSEMBL   UCSC 
 
  522
 
  5
 
 14616
 
 gene;island
 
 0.91170
 
 0.94541
 
 Not queried
 
  0.6966468
 
       NA
 
       NA
  
 
  
 22
 
  38669073
 
  38669222
 
  ENSEMBL   UCSC 
 
  150
 
  7
 
 27093
 
 tss
 
 0.01119
 
 0.01680
 
 Not queried
 
  0.6965994
 
       NA
 
       NA
  
 
  
 2
 
  74685383
 
  74685531
 
  ENSEMBL   UCSC 
 
  149
 
  6
 
 23418
 
 tss
 
 0.01779
 
 0.02761
 
 Not queried
 
  0.6965446
 
       NA
 
       NA
  
 
  
 10
 
 114206778
 
 114207340
 
  ENSEMBL   UCSC 
 
  563
 
  7
 
 4749
 
 gene
 
 0.01529
 
 0.02637
 
 Not queried
 
  0.6964755
 
       NA
 
       NA
  
 
  
 5
 
   1212434
 
   1212641
 
  ENSEMBL   UCSC 
 
  208
 
  5
 
 31472
 
 gene;island
 
 0.93882
 
 0.79212
 
 Not queried
 
  0.6963790
 
       NA
 
       NA
  
 
  
 17
 
  41560934
 
  41561319
 
  ENSEMBL   UCSC 
 
  386
 
 10
 
 17357
 
 tss
 
 0.01756
 
 0.03110
 
 Not queried
 
  0.6963637
 
       NA
 
       NA
  
 
  
 22
 
  50885288
 
  50885658
 
  ENSEMBL   UCSC 
 
  371
 
  4
 
 26888
 
 gene;island
 
 0.86211
 
 0.89732
 
 Not queried
 
  0.6963634
 
       NA
 
       NA
  
 
  
 2
 
  86564436
 
  86564659
 
  ENSEMBL   UCSC 
 
  224
 
  4
 
 22732
 
 gene;island
 
 0.04602
 
 0.05807
 
 Not queried
 
  0.6962063
 
       NA
 
       NA
  
 
  
 4
 
  89205684
 
  89206201
 
  ENSEMBL   UCSC 
 
  518
 
 10
 
 31119
 
 island
 
 0.01911
 
 0.03195
 
 Not queried
 
  0.6961790
 
       NA
 
       NA
  
 
  
 17
 
  48263150
 
  48263204
 
  ENSEMBL   UCSC 
 
   55
 
  6
 
 16646
 
 gene
 
 0.90603
 
 0.88246
 
 Not queried
 
  0.6961386
 
       NA
 
       NA
  
 
  
 1
 
  65730339
 
  65730494
 
  ENSEMBL   UCSC 
 
  156
 
  6
 
 3346
 
 island
 
 0.04685
 
 0.02936
 
 Not queried
 
  0.6961127
 
       NA
 
       NA
  
 
  
 17
 
  45726922
 
  45727226
 
  ENSEMBL   UCSC 
 
  305
 
  6
 
 17409
 
 tss;island
 
 0.01341
 
 0.01943
 
 Not queried
 
  0.6960482
 
       NA
 
       NA
  
 
  
 19
 
  14676202
 
  14676461
 
  ENSEMBL   UCSC 
 
  260
 
  4
 
 19861
 
 gene;island
 
 0.89580
 
 0.91480
 
 Not queried
 
  0.6959909
 
       NA
 
       NA
  
 
  
 5
 
 132202151
 
 132202281
 
  ENSEMBL   UCSC 
 
  131
 
  4
 
 32222
 
 tss
 
 0.01959
 
 0.02332
 
 Not queried
 
  0.6959357
 
       NA
 
       NA
  
 
  
 10
 
  31609200
 
  31609531
 
  ENSEMBL   UCSC 
 
  332
 
  4
 
 4540
 
 gene;tss;island
 
 0.02334
 
 0.03537
 
 Not queried
 
  0.6959168
 
       NA
 
       NA
  
 
  
 10
 
  85997177
 
  85997517
 
  ENSEMBL   UCSC 
 
  341
 
  4
 
 4643
 
 gene;island
 
 0.57710
 
 0.64147
 
 Not queried
 
  0.6957764
 
       NA
 
       NA
  
 
  
 12
 
  65672405
 
  65672531
 
  ENSEMBL   UCSC 
 
  127
 
  4
 
 10572
 
 island
 
 0.01803
 
 0.03275
 
 Not queried
 
  0.6956200
 
       NA
 
       NA
  
 
  
 19
 
   1490414
 
   1490731
 
  ENSEMBL   UCSC 
 
  318
 
  7
 
 20271
 
 tss;island
 
 0.03115
 
 0.04658
 
 Not queried
 
  0.6953519
 
       NA
 
       NA
  
 
  
 5
 
 150632246
 
 150632592
 
  ENSEMBL   UCSC 
 
  347
 
  6
 
 32380
 
 tss
 
 0.01178
 
 0.01835
 
 Not queried
 
  0.6953388
 
       NA
 
       NA
  
 
  
 16
 
  28550525
 
  28550637
 
  ENSEMBL   UCSC 
 
  113
 
  4
 
 15124
 
 tss
 
 0.64555
 
 0.74676
 
 Not queried
 
  0.6953191
 
       NA
 
       NA
  
 
  
 10
 
 102746998
 
 102747550
 
  ENSEMBL   UCSC 
 
  553
 
 14
 
 4702
 
 gene;tss;island
 
 0.01224
 
 0.02051
 
 Not queried
 
  0.6951194
 
       NA
 
       NA
  
 
  
 3
 
  97483466
 
  97483919
 
  ENSEMBL   UCSC 
 
  454
 
  9
 
 29268
 
 island
 
 0.01327
 
 0.01949
 
 Not queried
 
  0.6951084
 
       NA
 
       NA
  
 
  
 1
 
 145385163
 
 145385771
 
  ENSEMBL   UCSC 
 
  609
 
  8
 
 3667
 
 island
 
 0.86111
 
 0.74550
 
 Not queried
 
  0.6950537
 
       NA
 
       NA
  
 
  
 X
 
 118108506
 
 118108666
 
  ENSEMBL   UCSC 
 
  161
 
  6
 
 41484
 
 tss
 
 0.12820
 
 0.09263
 
 Not queried
 
  0.6950504
 
       NA
 
       NA
  
 
  
 18
 
  43753473
 
  43754049
 
  ENSEMBL   UCSC 
 
  577
 
  9
 
 19454
 
 island
 
 0.02724
 
 0.02567
 
 Not queried
 
  0.6949944
 
       NA
 
       NA
  
 
  
 20
 
  62321059
 
  62321379
 
  ENSEMBL   UCSC 
 
  321
 
  4
 
 25469
 
 gene;island
 
 0.82923
 
 0.89425
 
 Not queried
 
  0.6947171
 
       NA
 
       NA
  
 
  
 5
 
 179636283
 
 179636617
 
  ENSEMBL   UCSC 
 
  335
 
  4
 
 32499
 
 tss;island
 
 0.48875
 
 0.30407
 
 Not queried
 
  0.6944980
 
       NA
 
       NA
  
 
  
 2
 
 238777587
 
 238777806
 
  ENSEMBL   UCSC 
 
  220
 
  4
 
 23056
 
 gene
 
 0.94711
 
 0.87763
 
 Not queried
 
  0.6944792
 
       NA
 
       NA
  
 
  
 17
 
  21187761
 
  21187850
 
  ENSEMBL   UCSC 
 
   90
 
  8
 
 17169
 
 tss;island
 
 0.02204
 
 0.01535
 
 Not queried
 
  0.6944596
 
       NA
 
       NA
  
 
  
 11
 
   1471410
 
   1471699
 
  ENSEMBL   UCSC 
 
  290
 
  6
 
 6402
 
 gene;island
 
 0.90400
 
 0.94224
 
 Not queried
 
  0.6943393
 
       NA
 
       NA
  
 
  
 11
 
 107798913
 
 107799237
 
  ENSEMBL   UCSC 
 
  325
 
  9
 
 7644
 
 tss
 
 0.02426
 
 0.07027
 
 Not queried
 
  0.6943052
 
       NA
 
       NA
  
 
  
 19
 
  34012884
 
  34013247
 
  ENSEMBL   UCSC 
 
  364
 
  7
 
 20649
 
 tss
 
 0.02144
 
 0.03200
 
 Not queried
 
  0.6941223
 
       NA
 
       NA
  
 
  
 6
 
  33179469
 
  33180415
 
  ENSEMBL   UCSC 
 
  947
 
 14
 
 34167
 
 gene;island
 
 0.71053
 
 0.86742
 
 Not queried
 
  0.6940673
 
       NA
 
       NA
  
 
  
 19
 
  16222203
 
  16222446
 
  ENSEMBL   UCSC 
 
  244
 
  4
 
 20531
 
 tss
 
 0.01632
 
 0.00836
 
 Not queried
 
  0.6940038
 
       NA
 
       NA
  
 
  
 6
 
 166891617
 
 166891959
 
  ENSEMBL   UCSC 
 
  343
 
  5
 
 34481
 
 gene
 
 0.96760
 
 0.93273
 
 Not queried
 
  0.6938855
 
       NA
 
       NA
  
 
  
 6
 
 170862455
 
 170862576
 
  ENSEMBL   UCSC 
 
  122
 
  5
 
 35293
 
 tss;island
 
 0.05548
 
 0.03301
 
 Not queried
 
  0.6938676
 
       NA
 
       NA
  
 
  
 12
 
  79258313
 
  79258438
 
  ENSEMBL   UCSC 
 
  126
 
  4
 
 9200
 
 gene;tss
 
 0.01995
 
 0.03207
 
 Not queried
 
  0.6938066
 
       NA
 
       NA
  
 
  
 11
 
   3173868
 
   3174128
 
  ENSEMBL   UCSC 
 
  261
 
  5
 
 6457
 
 gene
 
 0.78609
 
 0.85598
 
 Not queried
 
  0.6936227
 
       NA
 
       NA
  
 
  
 2
 
   1732172
 
   1732454
 
  ENSEMBL   UCSC 
 
  283
 
  4
 
 22498
 
 gene
 
 0.68482
 
 0.77811
 
 Not queried
 
  0.6934963
 
       NA
 
       NA
  
 
  
 6
 
  33177497
 
  33177949
 
  ENSEMBL   UCSC 
 
  453
 
 13
 
 34165
 
 gene;island
 
 0.87480
 
 0.90176
 
 Not queried
 
  0.6932920
 
       NA
 
       NA
  
 
  
 6
 
 148663353
 
 148663964
 
  ENSEMBL   UCSC 
 
  612
 
 12
 
 36488
 
 island
 
 0.01513
 
 0.01727
 
 Not queried
 
  0.6932688
 
       NA
 
       NA
  
 
  
 11
 
  68851580
 
  68851932
 
  ENSEMBL   UCSC 
 
  353
 
  7
 
 6761
 
 gene;island
 
 0.89632
 
 0.87650
 
 Not queried
 
  0.6931121
 
       NA
 
       NA
  
 
  
 19
 
  54415702
 
  54416165
 
  ENSEMBL   UCSC 
 
  464
 
  7
 
 22355
 
 island
 
 0.40974
 
 0.36154
 
 Not queried
 
  0.6931120
 
       NA
 
       NA
  
 
  
 1
 
  29213635
 
  29213863
 
  ENSEMBL   UCSC 
 
  229
 
  7
 
 372
 
 gene;island
 
 0.01773
 
 0.02836
 
 Not queried
 
  0.6930909
 
       NA
 
       NA
  
 
  
 11
 
   8704225
 
   8704501
 
  ENSEMBL   UCSC 
 
  277
 
  4
 
 6479
 
 gene;island
 
 0.03192
 
 0.04823
 
 Not queried
 
  0.6930183
 
       NA
 
       NA
  
 
  
 18
 
  12947517
 
  12947957
 
  ENSEMBL   UCSC 
 
  441
 
  8
 
 19158
 
 tss
 
 0.03613
 
 0.05559
 
 Not queried
 
  0.6930124
 
       NA
 
       NA
  
 
  
 18
 
  12947517
 
  12948029
 
  ENSEMBL   UCSC 
 
  513
 
 10
 
 19374
 
 island
 
 0.03480
 
 0.04746
 
 Not queried
 
  0.6930124
 
       NA
 
       NA
  
 
  
 6
 
  31459698
 
  31460088
 
  ENSEMBL   UCSC 
 
  391
 
  6
 
 35711
 
 island
 
 0.02947
 
 0.03673
 
 Not queried
 
  0.6930090
 
       NA
 
       NA
  
 
  
 X
 
 122993679
 
 122994037
 
  ENSEMBL   UCSC 
 
  359
 
 10
 
 41504
 
 tss
 
 0.01992
 
 0.01867
 
 Not queried
 
  0.6928738
 
       NA
 
       NA
  
 
  
 21
 
  14982080
 
  14982363
 
  ENSEMBL   UCSC 
 
  284
 
  4
 
 26444
 
 tss
 
 0.58972
 
 0.82370
 
 Not queried
 
  0.6928387
 
       NA
 
       NA
  
 
  
 19
 
    407758
 
    408233
 
  ENSEMBL   UCSC 
 
  476
 
  4
 
 19590
 
 gene;island
 
 0.65597
 
 0.79001
 
 Not queried
 
  0.6925701
 
       NA
 
       NA
  
 
  
 22
 
  38082108
 
  38082335
 
  ENSEMBL   UCSC 
 
  228
 
  6
 
 27080
 
 tss
 
 0.03074
 
 0.04270
 
 Not queried
 
  0.6924958
 
       NA
 
       NA
  
 
  
 10
 
  80827237
 
  80827482
 
  ENSEMBL   UCSC 
 
  246
 
  4
 
 5163
 
 tss
 
 0.03232
 
 0.06176
 
 Not queried
 
  0.6920498
 
       NA
 
       NA
  
 
  
 12
 
 123921306
 
 123921793
 
  ENSEMBL   UCSC 
 
  488
 
  8
 
 10075
 
 tss
 
 0.05334
 
 0.04140
 
 Not queried
 
  0.6919989
 
       NA
 
       NA
  
 
  
 7
 
  23146155
 
  23146405
 
  ENSEMBL   UCSC 
 
  251
 
  4
 
 36850
 
 gene;island
 
 0.03142
 
 0.03023
 
 Not queried
 
  0.6919086
 
       NA
 
       NA
  
 
  
 20
 
  36793705
 
  36794002
 
  ENSEMBL   UCSC 
 
  298
 
 10
 
 25692
 
 tss
 
 0.04452
 
 0.06293
 
 Not queried
 
  0.6918158
 
       NA
 
       NA
  
 
  
 20
 
  36793608
 
  36794002
 
  ENSEMBL   UCSC 
 
  395
 
 12
 
 26112
 
 island
 
 0.05157
 
 0.08203
 
 Not queried
 
  0.6918158
 
       NA
 
       NA
  
 
  
 12
 
   1100377
 
   1100388
 
  ENSEMBL   UCSC 
 
   12
 
  4
 
 9457
 
 tss
 
 0.01969
 
 0.02882
 
 Not queried
 
  0.6916953
 
       NA
 
       NA
  
 
  
 12
 
 131356526
 
 131356597
 
  ENSEMBL   UCSC 
 
   72
 
  4
 
 10095
 
 tss
 
 0.02514
 
 0.04161
 
 Not queried
 
  0.6916420
 
       NA
 
       NA
  
 
  
 4
 
 154125208
 
 154125720
 
  ENSEMBL   UCSC 
 
  513
 
  9
 
 30105
 
 gene
 
 0.03553
 
 0.04111
 
 Not queried
 
  0.6914301
 
       NA
 
       NA
  
 
  
 14
 
 104175555
 
 104175847
 
  ENSEMBL   UCSC 
 
  293
 
  4
 
 12213
 
 gene
 
 0.90921
 
 0.93692
 
 Not queried
 
  0.6914129
 
       NA
 
       NA
  
 
  
 19
 
  50321295
 
  50321723
 
  ENSEMBL   UCSC 
 
  429
 
 10
 
 22256
 
 island
 
 0.01013
 
 0.01346
 
 Not queried
 
  0.6913842
 
       NA
 
       NA
  
 
  
 8
 
 144565115
 
 144565335
 
  ENSEMBL   UCSC 
 
  221
 
  4
 
 39416
 
 gene;island
 
 0.88201
 
 0.93552
 
 Not queried
 
  0.6912919
 
       NA
 
       NA
  
 
  
 11
 
 105481283
 
 105481509
 
  ENSEMBL   UCSC 
 
  227
 
  8
 
 6861
 
 gene;island
 
 0.01658
 
 0.02218
 
 Not queried
 
  0.6912902
 
       NA
 
       NA
  
 
  
 2
 
   3605983
 
   3606331
 
  ENSEMBL   UCSC 
 
  349
 
  9
 
 23123
 
 tss
 
 0.02841
 
 0.03316
 
 Not queried
 
  0.6911100
 
       NA
 
       NA
  
 
  
 7
 
 101630546
 
 101630948
 
  ENSEMBL   UCSC 
 
  403
 
  6
 
 37096
 
 gene
 
 0.92987
 
 0.94593
 
 Not queried
 
  0.6910202
 
       NA
 
       NA
  
 
  
 5
 
 137688057
 
 137688255
 
  ENSEMBL   UCSC 
 
  199
 
  4
 
 32261
 
 tss
 
 0.16953
 
 0.22212
 
 Not queried
 
  0.6910190
 
       NA
 
       NA
  
 
  
 6
 
 135375750
 
 135376004
 
  ENSEMBL   UCSC 
 
  255
 
  4
 
 34419
 
 gene
 
 0.02371
 
 0.03367
 
 Not queried
 
  0.6909409
 
       NA
 
       NA
  
 
  
 17
 
   1933006
 
   1933418
 
  ENSEMBL   UCSC 
 
  413
 
  9
 
 16965
 
 tss
 
 0.03774
 
 0.03270
 
 Not queried
 
  0.6908889
 
       NA
 
       NA
  
 
  
 12
 
  95227997
 
  95228136
 
  ENSEMBL   UCSC 
 
  140
 
  6
 
 9894
 
 tss
 
 0.65322
 
 0.82454
 
 Not queried
 
  0.6908455
 
       NA
 
       NA
  
 
  
 17
 
  46124684
 
  46124821
 
  ENSEMBL   UCSC 
 
  138
 
  4
 
 17418
 
 tss;island
 
 0.11958
 
 0.04278
 
 Not queried
 
  0.6907987
 
       NA
 
       NA
  
 
  
 6
 
  28227127
 
  28227482
 
  ENSEMBL   UCSC 
 
  356
 
  4
 
 33564
 
 gene
 
 0.89494
 
 0.93578
 
 Not queried
 
  0.6907971
 
       NA
 
       NA
  
 
  
 6
 
  74171350
 
  74171446
 
  ENSEMBL   UCSC 
 
   97
 
  4
 
 35007
 
 tss
 
 0.02264
 
 0.01410
 
 Not queried
 
  0.6906892
 
       NA
 
       NA
  
 
  
 6
 
  32634245
 
  32634508
 
  ENSEMBL   UCSC 
 
  264
 
  6
 
 35859
 
 island
 
 0.50888
 
 0.33574
 
 Not queried
 
  0.6906579
 
       NA
 
       NA
  
 
  
 20
 
  47662406
 
  47662890
 
  ENSEMBL   UCSC 
 
  485
 
 10
 
 26189
 
 island
 
 0.02122
 
 0.02405
 
 Not queried
 
  0.6906517
 
       NA
 
       NA
  
 
  
 13
 
  52378287
 
  52378744
 
  ENSEMBL   UCSC 
 
  458
 
 10
 
 11425
 
 tss
 
 0.02443
 
 0.04024
 
 Not queried
 
  0.6906280
 
       NA
 
       NA
  
 
  
 1
 
 110881820
 
 110881943
 
  ENSEMBL   UCSC 
 
  124
 
  4
 
 1811
 
 tss
 
 0.01488
 
 0.02694
 
 Not queried
 
  0.6906047
 
       NA
 
       NA
  
 
  
 4
 
 108853204
 
 108853489
 
  ENSEMBL   UCSC 
 
  286
 
  4
 
 30036
 
 gene;island
 
 0.20872
 
 0.26682
 
 Not queried
 
  0.6904485
 
       NA
 
       NA
  
 
  
 19
 
  41018757
 
  41018880
 
  ENSEMBL   UCSC 
 
  124
 
  4
 
 20008
 
 gene;island
 
 0.22439
 
 0.14634
 
 Not queried
 
  0.6903651
 
       NA
 
       NA
  
 
  
 17
 
  38210900
 
  38211345
 
  ENSEMBL   UCSC 
 
  446
 
  7
 
 17288
 
 tss
 
 0.08616
 
 0.03561
 
 Not queried
 
  0.6902549
 
       NA
 
       NA
  
 
  
 17
 
  38210697
 
  38211345
 
  ENSEMBL   UCSC 
 
  649
 
  9
 
 18258
 
 island
 
 0.05324
 
 0.03368
 
 Not queried
 
  0.6902549
 
       NA
 
       NA
  
 
  
 3
 
 123603306
 
 123603618
 
  ENSEMBL   UCSC 
 
  313
 
  6
 
 28504
 
 tss;island
 
 0.01508
 
 0.00782
 
 Not queried
 
  0.6900116
 
       NA
 
       NA
  
 
  
 1
 
 113741483
 
 113741744
 
  ENSEMBL   UCSC 
 
  262
 
  4
 
 3605
 
 island
 
 0.75160
 
 0.89147
 
 Not queried
 
  0.6899140
 
       NA
 
       NA
  
 
  
 5
 
  43557435
 
  43557751
 
  ENSEMBL   UCSC 
 
  317
 
  8
 
 31990
 
 tss;island
 
 0.02264
 
 0.03647
 
 Not queried
 
  0.6898994
 
       NA
 
       NA
  
 
  
 2
 
  98205903
 
  98206249
 
  ENSEMBL   UCSC 
 
  347
 
  4
 
 22756
 
 gene;island
 
 0.02023
 
 0.03048
 
 Not queried
 
  0.6898845
 
       NA
 
       NA
  
 
  
 20
 
  52824106
 
  52824483
 
  ENSEMBL   UCSC 
 
  378
 
  8
 
 25791
 
 tss
 
 0.03119
 
 0.03993
 
 Not queried
 
  0.6898286
 
       NA
 
       NA
  
 
  
 2
 
  98963408
 
  98963768
 
  ENSEMBL   UCSC 
 
  361
 
  4
 
 22764
 
 gene;island
 
 0.10146
 
 0.05586
 
 Not queried
 
  0.6897641
 
       NA
 
       NA
  
 
  
 6
 
  30879515
 
  30880557
 
  ENSEMBL   UCSC 
 
 1043
 
 17
 
 33771
 
 gene;island
 
 0.83702
 
 0.91430
 
 Not queried
 
  0.6897467
 
       NA
 
       NA
  
 
  
 1
 
  38512588
 
  38512829
 
  ENSEMBL   UCSC 
 
  242
 
  5
 
 1512
 
 tss;island
 
 0.02575
 
 0.03416
 
 Not queried
 
  0.6897158
 
       NA
 
       NA
  
 
  
 17
 
  18164253
 
  18164442
 
  ENSEMBL   UCSC 
 
  190
 
  5
 
 16437
 
 gene;island
 
 0.04924
 
 0.06645
 
 Not queried
 
  0.6896298
 
       NA
 
       NA
  
 
  
 4
 
  77135162
 
  77135506
 
  ENSEMBL   UCSC 
 
  345
 
  7
 
 30399
 
 tss
 
 0.04912
 
 0.01894
 
 Not queried
 
  0.6894404
 
       NA
 
       NA
  
 
  
 7
 
  44925080
 
  44925417
 
  ENSEMBL   UCSC 
 
  338
 
  5
 
 37509
 
 tss
 
 0.04304
 
 0.02801
 
 Not queried
 
  0.6894376
 
       NA
 
       NA
  
 
  
 6
 
 116937346
 
 116937499
 
  ENSEMBL   UCSC 
 
  154
 
  8
 
 35127
 
 tss
 
 0.01032
 
 0.02644
 
 Not queried
 
  0.6894134
 
       NA
 
       NA
  
 
  
 X
 
  53111116
 
  53111786
 
  ENSEMBL   UCSC 
 
  671
 
  9
 
 41804
 
 island
 
 0.01667
 
 0.02390
 
 Not queried
 
  0.6893573
 
       NA
 
       NA
  
 
  
 1
 
  16010226
 
  16010724
 
  ENSEMBL   UCSC 
 
  499
 
  5
 
 1272
 
 tss;island
 
 0.03695
 
 0.05826
 
 Not queried
 
  0.6893338
 
       NA
 
       NA
  
 
  
 17
 
  38472961
 
  38473382
 
  ENSEMBL   UCSC 
 
  422
 
  5
 
 16527
 
 gene;island
 
 0.02005
 
 0.01353
 
 Not queried
 
  0.6892573
 
       NA
 
       NA
  
 
  
 17
 
  32907636
 
  32907997
 
  ENSEMBL   UCSC 
 
  362
 
  7
 
 18169
 
 island
 
 0.07593
 
 0.08990
 
 Not queried
 
  0.6892041
 
       NA
 
       NA
  
 
  
 19
 
  49994097
 
  49994372
 
  ENSEMBL   UCSC 
 
  276
 
  4
 
 20914
 
 tss
 
 0.95584
 
 0.93446
 
 Not queried
 
  0.6888126
 
       NA
 
       NA
  
 
  
 11
 
  68518800
 
  68519252
 
  ENSEMBL   UCSC 
 
  453
 
 10
 
 8555
 
 island
 
 0.04688
 
 0.05664
 
 Not queried
 
  0.6886202
 
       NA
 
       NA
  
 
  
 16
 
  11038165
 
  11038308
 
  ENSEMBL   UCSC 
 
  144
 
  4
 
 15059
 
 tss
 
 0.02087
 
 0.03010
 
 Not queried
 
  0.6886143
 
       NA
 
       NA
  
 
  
 19
 
  52800469
 
  52800757
 
  ENSEMBL   UCSC 
 
  289
 
  4
 
 20155
 
 gene
 
 0.04456
 
 0.07193
 
 Not queried
 
  0.6886143
 
       NA
 
       NA
  
 
  
 3
 
  35680791
 
  35680899
 
  ENSEMBL   UCSC 
 
  109
 
  4
 
 28934
 
 island
 
 0.55861
 
 0.45444
 
 Not queried
 
  0.6885167
 
       NA
 
       NA
  
 
  
 22
 
  35653223
 
  35653394
 
  ENSEMBL   UCSC 
 
  172
 
  5
 
 27055
 
 tss;island
 
 0.01028
 
 0.01554
 
 Not queried
 
  0.6885123
 
       NA
 
       NA
  
 
  
 10
 
    711897
 
    712592
 
  ENSEMBL   UCSC 
 
  696
 
  8
 
 4437
 
 gene;island
 
 0.75324
 
 0.86563
 
 Not queried
 
  0.6885099
 
       NA
 
       NA
  
 
  
 14
 
 104940229
 
 104940658
 
  ENSEMBL   UCSC 
 
  430
 
  5
 
 13177
 
 island
 
 0.87494
 
 0.90705
 
 Not queried
 
  0.6884800
 
       NA
 
       NA
  
 
  
 11
 
   8932380
 
   8933103
 
  ENSEMBL   UCSC 
 
  724
 
 10
 
 7109
 
 tss;island
 
 0.02934
 
 0.04383
 
 Not queried
 
  0.6882502
 
       NA
 
       NA
  
 
  
 11
 
  67236227
 
  67237179
 
  ENSEMBL   UCSC 
 
  953
 
 14
 
 8530
 
 island
 
 0.02314
 
 0.04815
 
 Not queried
 
  0.6881644
 
       NA
 
       NA
  
 
  
 19
 
   4153364
 
   4153603
 
  ENSEMBL   UCSC 
 
  240
 
  4
 
 20323
 
 tss
 
 0.11224
 
 0.14706
 
 Not queried
 
  0.6881498
 
       NA
 
       NA
  
 
  
 13
 
  50699716
 
  50699806
 
  ENSEMBL   UCSC 
 
   91
 
  5
 
 11419
 
 tss;island
 
 0.01750
 
 0.03224
 
 Not queried
 
  0.6881368
 
       NA
 
       NA
  
 
  
 17
 
   8055733
 
   8055888
 
  ENSEMBL   UCSC 
 
  156
 
  6
 
 17956
 
 island
 
 0.03725
 
 0.05972
 
 Not queried
 
  0.6880168
 
       NA
 
       NA
  
 
  
 7
 
  91569823
 
  91570105
 
  ENSEMBL   UCSC 
 
  283
 
  7
 
 37637
 
 tss
 
 0.01630
 
 0.01953
 
 Not queried
 
  0.6878650
 
       NA
 
       NA
  
 
  
 6
 
 152623094
 
 152623479
 
  ENSEMBL   UCSC 
 
  386
 
  5
 
 34450
 
 gene;island
 
 0.01538
 
 0.02551
 
 Not queried
 
  0.6877316
 
       NA
 
       NA
  
 
  
 6
 
  33394324
 
  33394537
 
  ENSEMBL   UCSC 
 
  214
 
  4
 
 34221
 
 gene;island
 
 0.13348
 
 0.10264
 
 Not queried
 
  0.6877028
 
       NA
 
       NA
  
 
  
 4
 
 170948564
 
 170948896
 
  ENSEMBL   UCSC 
 
  333
 
  4
 
 30628
 
 tss;island
 
 0.02171
 
 0.02680
 
 Not queried
 
  0.6876828
 
       NA
 
       NA
  
 
  
 6
 
  31746795
 
  31747433
 
  ENSEMBL   UCSC 
 
  639
 
 25
 
 33892
 
 gene
 
 0.84125
 
 0.89338
 
 Not queried
 
  0.6875661
 
       NA
 
       NA
  
 
  
 1
 
  33816020
 
  33816159
 
  ENSEMBL   UCSC 
 
  140
 
  5
 
 403
 
 gene;tss;island
 
 0.03117
 
 0.05792
 
 Not queried
 
  0.6874840
 
       NA
 
       NA
  
 
  
 16
 
  54968727
 
  54969146
 
  ENSEMBL   UCSC 
 
  420
 
  6
 
 15900
 
 island
 
 0.04498
 
 0.05110
 
 Not queried
 
  0.6874598
 
       NA
 
       NA
  
 
  
 19
 
   7894891
 
   7895133
 
  ENSEMBL   UCSC 
 
  243
 
  8
 
 20372
 
 tss
 
 0.01662
 
 0.02830
 
 Not queried
 
  0.6872569
 
       NA
 
       NA
  
 
  
 6
 
  31846899
 
  31847028
 
  ENSEMBL   UCSC 
 
  130
 
  7
 
 34783
 
 tss
 
 0.76852
 
 0.73653
 
 Not queried
 
  0.6871168
 
       NA
 
       NA
  
 
  
 11
 
   2919689
 
   2919808
 
  ENSEMBL   UCSC 
 
  120
 
  4
 
 6450
 
 gene;tss;island
 
 0.61289
 
 0.73918
 
 Not queried
 
  0.6871038
 
       NA
 
       NA
  
 
  
 22
 
  36697013
 
  36697055
 
  ENSEMBL   UCSC 
 
   43
 
  5
 
 26817
 
 gene
 
 0.86826
 
 0.84187
 
 Not queried
 
  0.6870237
 
       NA
 
       NA
  
 
  
 21
 
  46077454
 
  46077731
 
  ENSEMBL   UCSC 
 
  278
 
  6
 
 26418
 
 gene;tss
 
 0.90466
 
 0.92368
 
 Not queried
 
  0.6869316
 
       NA
 
       NA
  
 
  
 3
 
 107810361
 
 107810791
 
  ENSEMBL   UCSC 
 
  431
 
  6
 
 28445
 
 tss;island
 
 0.52257
 
 0.63714
 
 Not queried
 
  0.6868804
 
       NA
 
       NA
  
 
  
 17
 
  73512319
 
  73512583
 
  ENSEMBL   UCSC 
 
  265
 
  7
 
 17621
 
 tss;island
 
 0.02650
 
 0.03744
 
 Not queried
 
  0.6868765
 
       NA
 
       NA
  
 
  
 6
 
 130686592
 
 130686865
 
  ENSEMBL   UCSC 
 
  274
 
  5
 
 36415
 
 island
 
 0.02452
 
 0.02842
 
 Not queried
 
  0.6867780
 
       NA
 
       NA
  
 
  
 2
 
 131512651
 
 131513216
 
  ENSEMBL   UCSC 
 
  566
 
 12
 
 23591
 
 tss;island
 
 0.38654
 
 0.25643
 
 Not queried
 
  0.6867635
 
       NA
 
       NA
  
 
  
 13
 
  22033395
 
  22033473
 
  ENSEMBL   UCSC 
 
   79
 
  4
 
 11561
 
 island
 
 0.03398
 
 0.05981
 
 Not queried
 
  0.6867626
 
       NA
 
       NA
  
 
  
 2
 
 218712695
 
 218712871
 
  ENSEMBL   UCSC 
 
  177
 
  4
 
 22974
 
 gene
 
 0.90406
 
 0.84906
 
 Not queried
 
  0.6867089
 
       NA
 
       NA
  
 
  
 16
 
   3018991
 
   3019192
 
  ENSEMBL   UCSC 
 
  202
 
  4
 
 15011
 
 tss;island
 
 0.03360
 
 0.02876
 
 Not queried
 
  0.6866740
 
       NA
 
       NA
  
 
  
 2
 
 183903302
 
 183903477
 
  ENSEMBL   UCSC 
 
  176
 
  4
 
 23729
 
 tss;island
 
 0.03342
 
 0.03437
 
 Not queried
 
  0.6865884
 
       NA
 
       NA
  
 
  
 21
 
  46875142
 
  46875417
 
  ENSEMBL   UCSC 
 
  276
 
  4
 
 26555
 
 tss
 
 0.81800
 
 0.86358
 
 Not queried
 
  0.6865732
 
       NA
 
       NA
  
 
  
 5
 
 134210707
 
 134210839
 
  ENSEMBL   UCSC 
 
  133
 
  4
 
 31697
 
 gene;island
 
 0.05325
 
 0.03244
 
 Not queried
 
  0.6864672
 
       NA
 
       NA
  
 
  
 1
 
  10714469
 
  10714701
 
  ENSEMBL   UCSC 
 
  233
 
  4
 
 212
 
 gene;island
 
 0.80660
 
 0.87002
 
 Not queried
 
  0.6863428
 
       NA
 
       NA
  
 
  
 7
 
  75988356
 
  75988652
 
  ENSEMBL   UCSC 
 
  297
 
  9
 
 37598
 
 tss
 
 0.02052
 
 0.04092
 
 Not queried
 
  0.6862796
 
       NA
 
       NA
  
 
  
 15
 
  23085583
 
  23085908
 
  ENSEMBL   UCSC 
 
  326
 
  4
 
 13223
 
 gene;island
 
 0.11404
 
 0.07921
 
 Not queried
 
  0.6862459
 
       NA
 
       NA
  
 
  
 13
 
 113495380
 
 113495690
 
  ENSEMBL   UCSC 
 
  311
 
  4
 
 11230
 
 gene;island
 
 0.93364
 
 0.89704
 
 Not queried
 
  0.6861486
 
       NA
 
       NA
  
 
  
 5
 
 127874463
 
 127874587
 
  ENSEMBL   UCSC 
 
  125
 
  4
 
 32200
 
 tss;island
 
 0.01017
 
 0.02030
 
 Not queried
 
  0.6860872
 
       NA
 
       NA
  
 
  
 1
 
 228353591
 
 228353883
 
  ENSEMBL   UCSC 
 
  293
 
  5
 
 1046
 
 gene;island
 
 0.02360
 
 0.03558
 
 Not queried
 
  0.6860603
 
       NA
 
       NA
  
 
  
 1
 
 161171211
 
 161171902
 
  ENSEMBL   UCSC 
 
  692
 
  8
 
 2049
 
 tss
 
 0.51066
 
 0.49778
 
 Not queried
 
  0.6860038
 
       NA
 
       NA
  
 
  
 10
 
 103892309
 
 103892743
 
  ENSEMBL   UCSC 
 
  435
 
 11
 
 5299
 
 tss
 
 0.01987
 
 0.02237
 
 Not queried
 
  0.6859549
 
       NA
 
       NA
  
 
  
 1
 
 212873963
 
 212874153
 
  ENSEMBL   UCSC 
 
  191
 
  4
 
 2242
 
 tss;island
 
 0.04570
 
 0.09189
 
 Not queried
 
  0.6859252
 
       NA
 
       NA
  
 
  
 1
 
 150552348
 
 150552817
 
  ENSEMBL   UCSC 
 
  470
 
  6
 
 1906
 
 tss;island
 
 0.14259
 
 0.08227
 
 Not queried
 
  0.6857073
 
       NA
 
       NA
  
 
  
 19
 
  53757910
 
  53758309
 
  ENSEMBL   UCSC 
 
  400
 
  7
 
 22336
 
 island
 
 0.02095
 
 0.03895
 
 Not queried
 
  0.6855926
 
       NA
 
       NA
  
 
  
 21
 
  44486205
 
  44486452
 
  ENSEMBL   UCSC 
 
  248
 
  4
 
 26401
 
 gene;island
 
 0.94758
 
 0.90953
 
 Not queried
 
  0.6855229
 
       NA
 
       NA
  
 
  
 X
 
 154842296
 
 154842535
 
  ENSEMBL   UCSC 
 
  240
 
  4
 
 41168
 
 gene
 
 0.02234
 
 0.03539
 
 Not queried
 
  0.6854615
 
       NA
 
       NA
  
 
  
 17
 
  28511883
 
  28512115
 
  ENSEMBL   UCSC 
 
  233
 
  4
 
 16472
 
 gene
 
 0.96014
 
 0.97726
 
 Not queried
 
  0.6854215
 
       NA
 
       NA
  
 
  
 5
 
 153863417
 
 153863826
 
  ENSEMBL   UCSC 
 
  410
 
  4
 
 33270
 
 island
 
 0.17141
 
 0.29249
 
 Not queried
 
  0.6853913
 
       NA
 
       NA
  
 
  
 5
 
 131993614
 
 131994061
 
  ENSEMBL   UCSC 
 
  448
 
  6
 
 33033
 
 island
 
 0.92345
 
 0.88333
 
 Not queried
 
  0.6852787
 
       NA
 
       NA
  
 
  
 5
 
 180287559
 
 180287904
 
  ENSEMBL   UCSC 
 
  346
 
  4
 
 33447
 
 island
 
 0.01278
 
 0.03022
 
 Not queried
 
  0.6851441
 
       NA
 
       NA
  
 
  
 1
 
 210501936
 
 210502156
 
  ENSEMBL   UCSC 
 
  221
 
  5
 
 988
 
 gene;tss;island
 
 0.05745
 
 0.07606
 
 Not queried
 
  0.6851000
 
       NA
 
       NA
  
 
  
 1
 
 155100382
 
 155100511
 
  ENSEMBL   UCSC 
 
  130
 
  4
 
 789
 
 gene
 
 0.04809
 
 0.07749
 
 Not queried
 
  0.6850817
 
       NA
 
       NA
  
 
  
 11
 
    560589
 
    560751
 
  ENSEMBL   UCSC 
 
  163
 
  4
 
 6361
 
 gene
 
 0.05184
 
 0.07138
 
 Not queried
 
  0.6849138
 
       NA
 
       NA
  
 
  
 4
 
   3769468
 
   3769810
 
  ENSEMBL   UCSC 
 
  343
 
  4
 
 29868
 
 gene;island
 
 0.21345
 
 0.30375
 
 Not queried
 
  0.6848775
 
       NA
 
       NA
  
 
  
 4
 
 155664941
 
 155665106
 
  ENSEMBL   UCSC 
 
  166
 
  6
 
 30599
 
 tss
 
 0.03745
 
 0.06624
 
 Not queried
 
  0.6848452
 
       NA
 
       NA
  
 
  
 5
 
 139927007
 
 139927271
 
  ENSEMBL   UCSC 
 
  265
 
 10
 
 31728
 
 gene;island
 
 0.00904
 
 0.01716
 
 Not queried
 
  0.6847434
 
       NA
 
       NA
  
 
  
 2
 
  20866107
 
  20866414
 
  ENSEMBL   UCSC 
 
  308
 
  8
 
 23177
 
 tss
 
 0.02456
 
 0.03045
 
 Not queried
 
  0.6847163
 
       NA
 
       NA
  
 
  
 8
 
  98880860
 
  98881351
 
  ENSEMBL   UCSC 
 
  492
 
  9
 
 40415
 
 island
 
 0.03807
 
 0.03822
 
 Not queried
 
  0.6846280
 
       NA
 
       NA
  
 
  
 1
 
   6259145
 
   6259495
 
  ENSEMBL   UCSC 
 
  351
 
  4
 
 161
 
 gene
 
 0.01546
 
 0.01981
 
 Not queried
 
  0.6846258
 
       NA
 
       NA
  
 
  
 1
 
   6259145
 
   6259905
 
  ENSEMBL   UCSC 
 
  761
 
  8
 
 2588
 
 island
 
 0.01271
 
 0.01808
 
 Not queried
 
  0.6846258
 
       NA
 
       NA
  
 
  
 6
 
  33171765
 
  33172398
 
  ENSEMBL   UCSC 
 
  634
 
 10
 
 34830
 
 tss
 
 0.05934
 
 0.09001
 
 Not queried
 
  0.6844168
 
       NA
 
       NA
  
 
  
 6
 
  33171765
 
  33172572
 
  ENSEMBL   UCSC 
 
  808
 
 16
 
 35915
 
 island
 
 0.04988
 
 0.06840
 
 Not queried
 
  0.6844168
 
       NA
 
       NA
  
 
  
 2
 
  27712613
 
  27713092
 
  ENSEMBL   UCSC 
 
  480
 
 10
 
 23233
 
 tss
 
 0.03267
 
 0.02634
 
 Not queried
 
  0.6843116
 
       NA
 
       NA
  
 
  
 3
 
 142314877
 
 142315224
 
  ENSEMBL   UCSC 
 
  348
 
  7
 
 28599
 
 tss
 
 0.02570
 
 0.03443
 
 Not queried
 
  0.6843058
 
       NA
 
       NA
  
 
  
 10
 
  27389392
 
  27389666
 
  ENSEMBL   UCSC 
 
  275
 
  8
 
 5604
 
 island
 
 0.01659
 
 0.01403
 
 Not queried
 
  0.6842826
 
       NA
 
       NA
  
 
  
 15
 
  26109204
 
  26109614
 
  ENSEMBL   UCSC 
 
  411
 
 12
 
 13511
 
 tss;island
 
 0.70139
 
 0.66392
 
 Not queried
 
  0.6841983
 
       NA
 
       NA
  
 
  
 7
 
     82270
 
     82623
 
  ENSEMBL   UCSC 
 
  354
 
  5
 
 37963
 
 island
 
 0.69750
 
 0.77145
 
 Not queried
 
  0.6841725
 
       NA
 
       NA
  
 
  
 19
 
  10046864
 
  10047420
 
  ENSEMBL   UCSC 
 
  557
 
  8
 
 21475
 
 island
 
 0.04130
 
 0.06804
 
 Not queried
 
  0.6841536
 
       NA
 
       NA
  
 
  
 11
 
  94502349
 
  94502879
 
  ENSEMBL   UCSC 
 
  531
 
  8
 
 6850
 
 gene;island
 
 0.02661
 
 0.03466
 
 Not queried
 
  0.6840020
 
       NA
 
       NA
  
 
  
 4
 
   2011006
 
   2011099
 
  ENSEMBL   UCSC 
 
   94
 
  4
 
 30199
 
 tss
 
 0.02025
 
 0.02759
 
 Not queried
 
  0.6839909
 
       NA
 
       NA
  
 
  
 13
 
 114203831
 
 114204079
 
  ENSEMBL   UCSC 
 
  249
 
  4
 
 11265
 
 gene;island
 
 0.83540
 
 0.90173
 
 Not queried
 
  0.6839634
 
       NA
 
       NA
  
 
  
 20
 
  34542109
 
  34542777
 
  ENSEMBL   UCSC 
 
  669
 
  8
 
 26092
 
 island
 
 0.00833
 
 0.01839
 
 Not queried
 
  0.6838900
 
       NA
 
       NA
  
 
  
 15
 
  43802612
 
  43802888
 
  ENSEMBL   UCSC 
 
  277
 
  8
 
 14058
 
 island
 
 0.02201
 
 0.04202
 
 Not queried
 
  0.6838786
 
       NA
 
       NA
  
 
  
 5
 
  75013329
 
  75013682
 
  ENSEMBL   UCSC 
 
  354
 
  7
 
 32072
 
 tss
 
 0.02610
 
 0.05047
 
 Not queried
 
  0.6837874
 
       NA
 
       NA
  
 
  
 5
 
  32585221
 
  32585549
 
  ENSEMBL   UCSC 
 
  329
 
  6
 
 31948
 
 tss
 
 0.01901
 
 0.02598
 
 Not queried
 
  0.6837872
 
       NA
 
       NA
  
 
  
 16
 
  30597058
 
  30597528
 
  ENSEMBL   UCSC 
 
  471
 
  6
 
 15157
 
 tss;island
 
 0.05065
 
 0.02647
 
 Not queried
 
  0.6837498
 
       NA
 
       NA
  
 
  
 8
 
   8085844
 
   8086146
 
  ENSEMBL   UCSC 
 
  303
 
  5
 
 39992
 
 island
 
 0.06390
 
 0.04845
 
 Not queried
 
  0.6837355
 
       NA
 
       NA
  
 
  
 6
 
  15524614
 
  15524865
 
  ENSEMBL   UCSC 
 
  252
 
  4
 
 33519
 
 gene
 
 0.92080
 
 0.92615
 
 Not queried
 
  0.6836699
 
       NA
 
       NA
  
 
  
 3
 
 113666360
 
 113666584
 
  ENSEMBL   UCSC 
 
  225
 
  4
 
 28468
 
 tss;island
 
 0.03515
 
 0.02260
 
 Not queried
 
  0.6836027
 
       NA
 
       NA
  
 
  
 15
 
  72523736
 
  72523932
 
  ENSEMBL   UCSC 
 
  197
 
  4
 
 13746
 
 tss
 
 0.02104
 
 0.03567
 
 Not queried
 
  0.6835837
 
       NA
 
       NA
  
 
  
 11
 
 129683974
 
 129684323
 
  ENSEMBL   UCSC 
 
  350
 
  4
 
 8924
 
 island
 
 0.86795
 
 0.83599
 
 Not queried
 
  0.6835004
 
       NA
 
       NA
  
 
  
 6
 
  32947695
 
  32948526
 
  ENSEMBL   UCSC 
 
  832
 
 15
 
 34109
 
 gene
 
 0.90549
 
 0.95471
 
 Not queried
 
  0.6834669
 
       NA
 
       NA
  
 
  
 2
 
  95825271
 
  95825387
 
  ENSEMBL   UCSC 
 
  117
 
  6
 
 23470
 
 tss;island
 
 0.02603
 
 0.04476
 
 Not queried
 
  0.6833676
 
       NA
 
       NA
  
 
  
 17
 
  73874443
 
  73874637
 
  ENSEMBL   UCSC 
 
  195
 
  5
 
 16744
 
 gene
 
 0.05697
 
 0.08083
 
 Not queried
 
  0.6833553
 
       NA
 
       NA
  
 
  
 17
 
  73874443
 
  73874790
 
  ENSEMBL   UCSC 
 
  348
 
  7
 
 18730
 
 island
 
 0.03977
 
 0.07293
 
 Not queried
 
  0.6833553
 
       NA
 
       NA
  
 
  
 10
 
 102997782
 
 102998117
 
  ENSEMBL   UCSC 
 
  336
 
  6
 
 4714
 
 gene;island
 
 0.74658
 
 0.64144
 
 Not queried
 
  0.6833519
 
       NA
 
       NA
  
 
  
 16
 
  25123200
 
  25123425
 
  ENSEMBL   UCSC 
 
  226
 
  4
 
 14668
 
 gene;island
 
 0.03317
 
 0.05647
 
 Not queried
 
  0.6833159
 
       NA
 
       NA
  
 
  
 5
 
  52405349
 
  52405518
 
  ENSEMBL   UCSC 
 
  170
 
  6
 
 31556
 
 gene
 
 0.00550
 
 0.00878
 
 Not queried
 
  0.6833036
 
       NA
 
       NA
  
 
  
 2
 
  98274951
 
  98275002
 
  ENSEMBL   UCSC 
 
   52
 
  6
 
 22757
 
 gene
 
 0.91141
 
 0.85036
 
 Not queried
 
  0.6833012
 
       NA
 
       NA
  
 
  
 12
 
   2903984
 
   2904111
 
  ENSEMBL   UCSC 
 
  128
 
  8
 
 10134
 
 island
 
 0.00816
 
 0.01644
 
 Not queried
 
  0.6832380
 
       NA
 
       NA
  
 
  
 1
 
  77747875
 
  77748480
 
  ENSEMBL   UCSC 
 
  606
 
  9
 
 593
 
 gene;island
 
 0.02778
 
 0.03539
 
 Not queried
 
  0.6831576
 
       NA
 
       NA
  
 
  
 20
 
  50808543
 
  50809000
 
  ENSEMBL   UCSC 
 
  458
 
 11
 
 25788
 
 tss;island
 
 0.00818
 
 0.01793
 
 Not queried
 
  0.6829243
 
       NA
 
       NA
  
 
  
 11
 
 124932892
 
 124933008
 
  ENSEMBL   UCSC 
 
  117
 
  5
 
 7767
 
 tss
 
 0.02891
 
 0.01821
 
 Not queried
 
  0.6829187
 
       NA
 
       NA
  
 
  
 22
 
  38218945
 
  38219336
 
  ENSEMBL   UCSC 
 
  392
 
  4
 
 27084
 
 tss
 
 0.62994
 
 0.70369
 
 Not queried
 
  0.6829164
 
       NA
 
       NA
  
 
  
 12
 
  54673731
 
  54674114
 
  ENSEMBL   UCSC 
 
  384
 
  8
 
 9734
 
 tss;island
 
 0.02330
 
 0.03681
 
 Not queried
 
  0.6827191
 
       NA
 
       NA
  
 
  
 17
 
  75136249
 
  75136439
 
  ENSEMBL   UCSC 
 
  191
 
  4
 
 17660
 
 tss;island
 
 0.05435
 
 0.10073
 
 Not queried
 
  0.6826754
 
       NA
 
       NA
  
 
  
 17
 
  41560934
 
  41561480
 
  ENSEMBL   UCSC 
 
  547
 
 13
 
 18338
 
 island
 
 0.01916
 
 0.03179
 
 Not queried
 
  0.6825145
 
       NA
 
       NA
  
 
  
 21
 
  30670919
 
  30671158
 
  ENSEMBL   UCSC 
 
  240
 
  7
 
 26462
 
 tss;island
 
 0.01797
 
 0.02136
 
 Not queried
 
  0.6825084
 
       NA
 
       NA
  
 
  
 4
 
   7666052
 
   7666259
 
  ENSEMBL   UCSC 
 
  208
 
  5
 
 29896
 
 gene;island
 
 0.92729
 
 0.94298
 
 Not queried
 
  0.6824491
 
       NA
 
       NA
  
 
  
 17
 
  41116197
 
  41116704
 
  ENSEMBL   UCSC 
 
  508
 
  6
 
 16555
 
 gene;island
 
 0.02838
 
 0.03881
 
 Not queried
 
  0.6824181
 
       NA
 
       NA
  
 
  
 3
 
 139108398
 
 139108740
 
  ENSEMBL   UCSC 
 
  343
 
  8
 
 29493
 
 island
 
 0.02074
 
 0.03260
 
 Not queried
 
  0.6823776
 
       NA
 
       NA
  
 
  
 6
 
  31799311
 
  31799575
 
  ENSEMBL   UCSC 
 
  265
 
  4
 
 35787
 
 island
 
 0.69436
 
 0.79776
 
 Not queried
 
  0.6820294
 
       NA
 
       NA
  
 
  
 X
 
 107683309
 
 107683435
 
  ENSEMBL   UCSC 
 
  127
 
  4
 
 41075
 
 gene;tss
 
 0.02940
 
 0.03349
 
 Not queried
 
  0.6820120
 
       NA
 
       NA
  
 
  
 10
 
  64577216
 
  64577607
 
  ENSEMBL   UCSC 
 
  392
 
  4
 
 4589
 
 gene;tss;island
 
 0.06859
 
 0.11039
 
 Not queried
 
  0.6819624
 
       NA
 
       NA
  
 
  
 22
 
  36013398
 
  36013708
 
  ENSEMBL   UCSC 
 
  311
 
  7
 
 27059
 
 tss
 
 0.69851
 
 0.77028
 
 Not queried
 
  0.6818663
 
       NA
 
       NA
  
 
  
 17
 
  58042029
 
  58042493
 
  ENSEMBL   UCSC 
 
  465
 
 12
 
 18581
 
 island
 
 0.01760
 
 0.02213
 
 Not queried
 
  0.6818472
 
       NA
 
       NA
  
 
  
 17
 
  48474379
 
  48475044
 
  ENSEMBL   UCSC 
 
  666
 
  8
 
 18515
 
 island
 
 0.01067
 
 0.01737
 
 Not queried
 
  0.6817727
 
       NA
 
       NA
  
 
  
 14
 
  67826350
 
  67826504
 
  ENSEMBL   UCSC 
 
  155
 
  4
 
 12090
 
 gene;tss;island
 
 0.02358
 
 0.03389
 
 Not queried
 
  0.6817222
 
       NA
 
       NA
  
 
  
 7
 
 150773226
 
 150773481
 
  ENSEMBL   UCSC 
 
  256
 
  5
 
 37222
 
 gene
 
 0.71496
 
 0.89640
 
 Not queried
 
  0.6816838
 
       NA
 
       NA
  
 
  
 17
 
  14204310
 
  14204485
 
  ENSEMBL   UCSC 
 
  176
 
  5
 
 17113
 
 tss
 
 0.01779
 
 0.02522
 
 Not queried
 
  0.6816805
 
       NA
 
       NA
  
 
  
 6
 
  10398494
 
  10398754
 
  ENSEMBL   UCSC 
 
  261
 
  4
 
 33492
 
 gene;island
 
 0.95371
 
 0.96738
 
 Not queried
 
  0.6816224
 
       NA
 
       NA
  
 
  
 1
 
 113249942
 
 113249994
 
  ENSEMBL   UCSC 
 
   53
 
  4
 
 692
 
 gene
 
 0.04116
 
 0.05514
 
 Not queried
 
  0.6816146
 
       NA
 
       NA
  
 
  
 5
 
 135170000
 
 135170281
 
  ENSEMBL   UCSC 
 
  282
 
  7
 
 32248
 
 tss
 
 0.13561
 
 0.05958
 
 Not queried
 
  0.6814882
 
       NA
 
       NA
  
 
  
 1
 
 150208519
 
 150208726
 
  ENSEMBL   UCSC 
 
  208
 
  8
 
 1899
 
 tss;island
 
 0.01378
 
 0.02041
 
 Not queried
 
  0.6814702
 
       NA
 
       NA
  
 
  
 3
 
  52001778
 
  52002169
 
  ENSEMBL   UCSC 
 
  392
 
  4
 
 28326
 
 tss
 
 0.04475
 
 0.03872
 
 Not queried
 
  0.6814381
 
       NA
 
       NA
  
 
  
 19
 
  36233297
 
  36233469
 
  ENSEMBL   UCSC 
 
  173
 
  5
 
 21879
 
 island
 
 0.82560
 
 0.88361
 
 Not queried
 
  0.6814325
 
       NA
 
       NA
  
 
  
 8
 
 145649356
 
 145649678
 
  ENSEMBL   UCSC 
 
  323
 
  5
 
 39459
 
 gene;island
 
 0.75983
 
 0.88711
 
 Not queried
 
  0.6814215
 
       NA
 
       NA
  
 
  
 8
 
  79577796
 
  79578223
 
  ENSEMBL   UCSC 
 
  428
 
  7
 
 39715
 
 tss
 
 0.04240
 
 0.06432
 
 Not queried
 
  0.6812598
 
       NA
 
       NA
  
 
  
 4
 
 159131324
 
 159131785
 
  ENSEMBL   UCSC 
 
  462
 
  7
 
 31342
 
 island
 
 0.01985
 
 0.03251
 
 Not queried
 
  0.6810416
 
       NA
 
       NA
  
 
  
 12
 
  54070114
 
  54070610
 
  ENSEMBL   UCSC 
 
  497
 
 12
 
 9720
 
 tss
 
 0.08295
 
 0.32945
 
 Not queried
 
  0.6809142
 
       NA
 
       NA
  
 
  
 6
 
 159589874
 
 159590231
 
  ENSEMBL   UCSC 
 
  358
 
  4
 
 35255
 
 tss;island
 
 0.06335
 
 0.05060
 
 Not queried
 
  0.6808964
 
       NA
 
       NA
  
 
  
 3
 
  16924563
 
  16925048
 
  ENSEMBL   UCSC 
 
  486
 
  4
 
 28883
 
 island
 
 0.18993
 
 0.12561
 
 Not queried
 
  0.6807604
 
       NA
 
       NA
  
 
  
 X
 
  50557055
 
  50557283
 
  ENSEMBL   UCSC 
 
  229
 
  4
 
 41322
 
 tss
 
 0.03989
 
 0.02400
 
 Not queried
 
  0.6807491
 
       NA
 
       NA
  
 
  
 19
 
  48103651
 
  48104228
 
  ENSEMBL   UCSC 
 
  578
 
  6
 
 22163
 
 island
 
 0.03949
 
 0.01928
 
 Not queried
 
  0.6807114
 
       NA
 
       NA
  
 
  
 17
 
   3496166
 
   3496570
 
  ENSEMBL   UCSC 
 
  405
 
  4
 
 16301
 
 gene
 
 0.85140
 
 0.80454
 
 Not queried
 
  0.6806332
 
       NA
 
       NA
  
 
  
 5
 
 139040820
 
 139041014
 
  ENSEMBL   UCSC 
 
  195
 
  4
 
 31721
 
 gene;island
 
 0.82296
 
 0.81891
 
 Not queried
 
  0.6804652
 
       NA
 
       NA
  
 
  
 2
 
 127729597
 
 127729962
 
  ENSEMBL   UCSC 
 
  366
 
  5
 
 24644
 
 island
 
 0.03645
 
 0.04271
 
 Not queried
 
  0.6804445
 
       NA
 
       NA
  
 
  
 X
 
  77359739
 
  77359924
 
  ENSEMBL   UCSC 
 
  186
 
  4
 
 41040
 
 gene
 
 0.00887
 
 0.01021
 
 Not queried
 
  0.6804386
 
       NA
 
       NA
  
 
  
 7
 
 107302255
 
 107302510
 
  ENSEMBL   UCSC 
 
  256
 
  7
 
 37120
 
 gene;tss;island
 
 0.02136
 
 0.03379
 
 Not queried
 
  0.6804354
 
       NA
 
       NA
  
 
  
 4
 
  40858965
 
  40859344
 
  ENSEMBL   UCSC 
 
  380
 
  9
 
 29945
 
 gene;island
 
 0.10982
 
 0.06164
 
 Not queried
 
  0.6804014
 
       NA
 
       NA
  
 
  
 17
 
  56232260
 
  56232499
 
  ENSEMBL   UCSC 
 
  240
 
  4
 
 17499
 
 tss
 
 0.38381
 
 0.49803
 
 Not queried
 
  0.6803780
 
       NA
 
       NA
  
 
  
 5
 
  54528989
 
  54530436
 
  ENSEMBL   UCSC 
 
 1448
 
 15
 
 32739
 
 island
 
 0.02017
 
 0.03288
 
 Not queried
 
  0.6803639
 
       NA
 
       NA
  
 
  
 10
 
  43892790
 
  43893015
 
  ENSEMBL   UCSC 
 
  226
 
  8
 
 4557
 
 gene;tss;island
 
 0.03183
 
 0.06497
 
 Not queried
 
  0.6802159
 
       NA
 
       NA
  
 
  
 4
 
  94749865
 
  94750061
 
  ENSEMBL   UCSC 
 
  197
 
  6
 
 30453
 
 tss
 
 0.02405
 
 0.04280
 
 Not queried
 
  0.6801644
 
       NA
 
       NA
  
 
  
 19
 
   1847575
 
   1847834
 
  ENSEMBL   UCSC 
 
  260
 
  4
 
 19631
 
 gene;island
 
 0.04055
 
 0.03982
 
 Not queried
 
  0.6801634
 
       NA
 
       NA
  
 
  
 3
 
  25705530
 
  25705861
 
  ENSEMBL   UCSC 
 
  332
 
  6
 
 28898
 
 island
 
 0.04610
 
 0.07191
 
 Not queried
 
  0.6801133
 
       NA
 
       NA
  
 
  
 3
 
 128206790
 
 128206967
 
  ENSEMBL   UCSC 
 
  178
 
  5
 
 28526
 
 tss
 
 0.01994
 
 0.03156
 
 Not queried
 
  0.6801007
 
       NA
 
       NA
  
 
  
 19
 
  54041163
 
  54041398
 
  ENSEMBL   UCSC 
 
  236
 
  6
 
 20168
 
 gene;island
 
 0.01769
 
 0.01834
 
 Not queried
 
  0.6800186
 
       NA
 
       NA
  
 
  
 3
 
  32859377
 
  32859587
 
  ENSEMBL   UCSC 
 
  211
 
  7
 
 28925
 
 island
 
 0.02118
 
 0.02906
 
 Not queried
 
  0.6799963
 
       NA
 
       NA
  
 
  
 12
 
  64238325
 
  64238538
 
  ENSEMBL   UCSC 
 
  214
 
  7
 
 9814
 
 tss
 
 0.01513
 
 0.02053
 
 Not queried
 
  0.6799406
 
       NA
 
       NA
  
 
  
 12
 
  64238325
 
  64238719
 
  ENSEMBL   UCSC 
 
  395
 
  9
 
 10564
 
 island
 
 0.01513
 
 0.02053
 
 Not queried
 
  0.6799406
 
       NA
 
       NA
  
 
  
 6
 
  32711889
 
  32712103
 
  ENSEMBL   UCSC 
 
  215
 
  4
 
 34063
 
 gene
 
 0.34418
 
 0.22499
 
 Not queried
 
  0.6796836
 
       NA
 
       NA
  
 
  
 10
 
  81154142
 
  81154192
 
  ENSEMBL   UCSC 
 
   51
 
  4
 
 4638
 
 gene
 
 0.83078
 
 0.87093
 
 Not queried
 
  0.6796783
 
       NA
 
       NA
  
 
  
 17
 
  58042144
 
  58042493
 
  ENSEMBL   UCSC 
 
  350
 
 11
 
 17517
 
 tss
 
 0.01772
 
 0.02196
 
 Not queried
 
  0.6796744
 
       NA
 
       NA
  
 
  
 5
 
 148206173
 
 148206412
 
  ENSEMBL   UCSC 
 
  240
 
  4
 
 31797
 
 gene
 
 0.02354
 
 0.04842
 
 Not queried
 
  0.6795583
 
       NA
 
       NA
  
 
  
 2
 
   1497815
 
   1498138
 
  ENSEMBL   UCSC 
 
  324
 
  5
 
 22494
 
 gene;island
 
 0.69638
 
 0.72363
 
 Not queried
 
  0.6795309
 
       NA
 
       NA
  
 
  
 11
 
 111750159
 
 111750412
 
  ENSEMBL   UCSC 
 
  254
 
  4
 
 7670
 
 tss
 
 0.01698
 
 0.04234
 
 Not queried
 
  0.6795135
 
       NA
 
       NA
  
 
  
 14
 
  50052765
 
  50053030
 
  ENSEMBL   UCSC 
 
  266
 
  4
 
 12048
 
 gene
 
 0.01911
 
 0.02736
 
 Not queried
 
  0.6795046
 
       NA
 
       NA
  
 
  
 3
 
  53194945
 
  53195316
 
  ENSEMBL   UCSC 
 
  372
 
  8
 
 29156
 
 island
 
 0.02851
 
 0.03232
 
 Not queried
 
  0.6794687
 
       NA
 
       NA
  
 
  
 6
 
  52535550
 
  52536059
 
  ENSEMBL   UCSC 
 
  510
 
 11
 
 36145
 
 island
 
 0.04644
 
 0.06265
 
 Not queried
 
  0.6794613
 
       NA
 
       NA
  
 
  
 2
 
 220094490
 
 220094626
 
  ENSEMBL   UCSC 
 
  137
 
  7
 
 22989
 
 gene
 
 0.01930
 
 0.02174
 
 Not queried
 
  0.6793809
 
       NA
 
       NA
  
 
  
 10
 
  70166985
 
  70167264
 
  ENSEMBL   UCSC 
 
  280
 
  7
 
 5095
 
 tss;island
 
 0.03527
 
 0.02357
 
 Not queried
 
  0.6793119
 
       NA
 
       NA
  
 
  
 19
 
  39935857
 
  39936306
 
  ENSEMBL   UCSC 
 
  450
 
 10
 
 21963
 
 island
 
 0.01622
 
 0.02408
 
 Not queried
 
  0.6792770
 
       NA
 
       NA
  
 
  
 7
 
  11013274
 
  11013450
 
  ENSEMBL   UCSC 
 
  177
 
  8
 
 37376
 
 tss
 
 0.02000
 
 0.03891
 
 Not queried
 
  0.6791883
 
       NA
 
       NA
  
 
  
 7
 
  11013274
 
  11013963
 
  ENSEMBL   UCSC 
 
  690
 
 14
 
 38199
 
 island
 
 0.01800
 
 0.03184
 
 Not queried
 
  0.6791883
 
       NA
 
       NA
  
 
  
 18
 
  72265164
 
  72265361
 
  ENSEMBL   UCSC 
 
  198
 
  8
 
 19291
 
 tss
 
 0.01083
 
 0.01016
 
 Not queried
 
  0.6791857
 
       NA
 
       NA
  
 
  
 22
 
  22307371
 
  22307684
 
  ENSEMBL   UCSC 
 
  314
 
  6
 
 26961
 
 tss
 
 0.32687
 
 0.23607
 
 Not queried
 
  0.6789924
 
       NA
 
       NA
  
 
  
 19
 
  53662353
 
  53662723
 
  ENSEMBL   UCSC 
 
  371
 
  8
 
 20988
 
 tss
 
 0.01446
 
 0.03827
 
 Not queried
 
  0.6789895
 
       NA
 
       NA
  
 
  
 7
 
  73038888
 
  73039108
 
  ENSEMBL   UCSC 
 
  221
 
  6
 
 37572
 
 tss
 
 0.02206
 
 0.02549
 
 Not queried
 
  0.6789816
 
       NA
 
       NA
  
 
  
 12
 
 123874243
 
 123874466
 
  ENSEMBL   UCSC 
 
  224
 
  4
 
 9356
 
 gene;island
 
 0.03326
 
 0.05485
 
 Not queried
 
  0.6789642
 
       NA
 
       NA
  
 
  
 5
 
 176074273
 
 176074484
 
  ENSEMBL   UCSC 
 
  212
 
  7
 
 33368
 
 island
 
 0.03899
 
 0.04611
 
 Not queried
 
  0.6789027
 
       NA
 
       NA
  
 
  
 7
 
    778962
 
    779185
 
  ENSEMBL   UCSC 
 
  224
 
  4
 
 36656
 
 gene;island
 
 0.85525
 
 0.92070
 
 Not queried
 
  0.6788363
 
       NA
 
       NA
  
 
  
 4
 
    967432
 
    967541
 
  ENSEMBL   UCSC 
 
  110
 
  6
 
 30185
 
 tss
 
 0.01738
 
 0.04266
 
 Not queried
 
  0.6788323
 
       NA
 
       NA
  
 
  
 21
 
  46407926
 
  46408142
 
  ENSEMBL   UCSC 
 
  217
 
  4
 
 26706
 
 island
 
 0.69430
 
 0.81139
 
 Not queried
 
  0.6787653
 
       NA
 
       NA
  
 
  
 21
 
  43528734
 
  43528949
 
  ENSEMBL   UCSC 
 
  216
 
  4
 
 26394
 
 gene;tss;island
 
 0.75801
 
 0.84077
 
 Not queried
 
  0.6785075
 
       NA
 
       NA
  
 
  
 2
 
 220143840
 
 220143980
 
  ENSEMBL   UCSC 
 
  141
 
  4
 
 23864
 
 tss;island
 
 0.01908
 
 0.03337
 
 Not queried
 
  0.6783954
 
       NA
 
       NA
  
 
  
 12
 
 110010952
 
 110011337
 
  ENSEMBL   UCSC 
 
  386
 
  8
 
 9267
 
 gene
 
 0.01684
 
 0.03281
 
 Not queried
 
  0.6783756
 
       NA
 
       NA
  
 
  
 5
 
 140305947
 
 140306458
 
  ENSEMBL   UCSC 
 
  512
 
 10
 
 31747
 
 gene;island
 
 0.01377
 
 0.02918
 
 Not queried
 
  0.6783425
 
       NA
 
       NA
  
 
  
 5
 
 140305947
 
 140306249
 
  ENSEMBL   UCSC 
 
  303
 
  8
 
 32298
 
 tss
 
 0.01118
 
 0.01902
 
 Not queried
 
  0.6783425
 
       NA
 
       NA
  
 
  
 5
 
  77071865
 
  77072171
 
  ENSEMBL   UCSC 
 
  307
 
  4
 
 31599
 
 gene
 
 0.03337
 
 0.02666
 
 Not queried
 
  0.6783161
 
       NA
 
       NA
  
 
  
 1
 
 160231874
 
 160232248
 
  ENSEMBL   UCSC 
 
  375
 
  6
 
 840
 
 gene
 
 0.01740
 
 0.02606
 
 Not queried
 
  0.6783084
 
       NA
 
       NA
  
 
  
 16
 
  69373498
 
  69373674
 
  ENSEMBL   UCSC 
 
  177
 
  4
 
 14791
 
 gene;tss;island
 
 0.01169
 
 0.02515
 
 Not queried
 
  0.6783008
 
       NA
 
       NA
  
 
  
 X
 
   3264695
 
   3265089
 
  ENSEMBL   UCSC 
 
  395
 
  9
 
 41171
 
 tss
 
 0.02481
 
 0.03871
 
 Not queried
 
  0.6781820
 
       NA
 
       NA
  
 
  
 19
 
  11750621
 
  11751104
 
  ENSEMBL   UCSC 
 
  484
 
  5
 
 21543
 
 island
 
 0.02000
 
 0.03229
 
 Not queried
 
  0.6781394
 
       NA
 
       NA
  
 
  
 1
 
 110210684
 
 110211023
 
  ENSEMBL   UCSC 
 
  340
 
  6
 
 671
 
 gene
 
 0.20551
 
 0.10991
 
 Not queried
 
  0.6780957
 
       NA
 
       NA
  
 
  
 4
 
  53588360
 
  53588850
 
  ENSEMBL   UCSC 
 
  491
 
  6
 
 30992
 
 island
 
 0.02072
 
 0.04235
 
 Not queried
 
  0.6780187
 
       NA
 
       NA
  
 
  
 1
 
 231664136
 
 231664364
 
  ENSEMBL   UCSC 
 
  229
 
  8
 
 2320
 
 tss
 
 0.01811
 
 0.02078
 
 Not queried
 
  0.6779132
 
       NA
 
       NA
  
 
  
 10
 
   5060291
 
   5060566
 
  ENSEMBL   UCSC 
 
  276
 
  4
 
 4905
 
 tss
 
 0.87670
 
 0.70915
 
 Not queried
 
  0.6778600
 
       NA
 
       NA
  
 
  
 18
 
  77160030
 
  77160396
 
  ENSEMBL   UCSC 
 
  367
 
  7
 
 19103
 
 gene;island
 
 0.04059
 
 0.05211
 
 Not queried
 
  0.6778378
 
       NA
 
       NA
  
 
  
 3
 
 137893270
 
 137893718
 
  ENSEMBL   UCSC 
 
  449
 
  6
 
 27920
 
 gene
 
 0.01806
 
 0.02238
 
 Not queried
 
  0.6778247
 
       NA
 
       NA
  
 
  
 6
 
  31239653
 
  31240223
 
  ENSEMBL   UCSC 
 
  571
 
  8
 
 35698
 
 island
 
 0.06237
 
 0.11277
 
 Not queried
 
  0.6778176
 
       NA
 
       NA
  
 
  
 8
 
  19460097
 
  19460243
 
  ENSEMBL   UCSC 
 
  147
 
  5
 
 39508
 
 tss
 
 0.13245
 
 0.06370
 
 Not queried
 
  0.6777745
 
       NA
 
       NA
  
 
  
 14
 
  55657967
 
  55658398
 
  ENSEMBL   UCSC 
 
  432
 
  5
 
 12837
 
 island
 
 0.01985
 
 0.03409
 
 Not queried
 
  0.6777550
 
       NA
 
       NA
  
 
  
 17
 
   8151103
 
   8151491
 
  ENSEMBL   UCSC 
 
  389
 
  4
 
 17081
 
 tss
 
 0.00838
 
 0.01623
 
 Not queried
 
  0.6776538
 
       NA
 
       NA
  
 
  
 20
 
  61732467
 
  61732608
 
  ENSEMBL   UCSC 
 
  142
 
  4
 
 25458
 
 gene;tss;island
 
 0.32090
 
 0.41820
 
 Not queried
 
  0.6775690
 
       NA
 
       NA
  
 
  
 1
 
 179846837
 
 179847329
 
  ENSEMBL   UCSC 
 
  493
 
 10
 
 3990
 
 island
 
 0.03143
 
 0.02697
 
 Not queried
 
  0.6774826
 
       NA
 
       NA
  
 
  
 17
 
  78865087
 
  78865755
 
  ENSEMBL   UCSC 
 
  669
 
  7
 
 16847
 
 gene;island
 
 0.69064
 
 0.81546
 
 Not queried
 
  0.6773411
 
       NA
 
       NA
  
 
  
 10
 
   5882304
 
   5882739
 
  ENSEMBL   UCSC 
 
  436
 
  4
 
 5506
 
 island
 
 0.67465
 
 0.79188
 
 Not queried
 
  0.6772397
 
       NA
 
       NA
  
 
  
 19
 
   3480363
 
   3480672
 
  ENSEMBL   UCSC 
 
  310
 
  5
 
 21293
 
 island
 
 0.33808
 
 0.44956
 
 Not queried
 
  0.6771989
 
       NA
 
       NA
  
 
  
 13
 
 114260262
 
 114260546
 
  ENSEMBL   UCSC 
 
  285
 
  4
 
 11266
 
 gene;island
 
 0.93772
 
 0.90586
 
 Not queried
 
  0.6771678
 
       NA
 
       NA
  
 
  
 5
 
 169930841
 
 169931363
 
  ENSEMBL   UCSC 
 
  523
 
  9
 
 31831
 
 gene;island
 
 0.25264
 
 0.12078
 
 Not queried
 
  0.6769919
 
       NA
 
       NA
  
 
  
 8
 
 123964031
 
 123964437
 
  ENSEMBL   UCSC 
 
  407
 
  5
 
 39353
 
 gene
 
 0.86526
 
 0.91089
 
 Not queried
 
  0.6769859
 
       NA
 
       NA
  
 
  
 X
 
  50214405
 
  50214570
 
  ENSEMBL   UCSC 
 
  166
 
  4
 
 41321
 
 tss;island
 
 0.10912
 
 0.08016
 
 Not queried
 
  0.6769144
 
       NA
 
       NA
  
 
  
 8
 
 143373411
 
 143373735
 
  ENSEMBL   UCSC 
 
  325
 
  4
 
 39394
 
 gene
 
 0.68668
 
 0.76759
 
 Not queried
 
  0.6768757
 
       NA
 
       NA
  
 
  
 11
 
 118754280
 
 118754535
 
  ENSEMBL   UCSC 
 
  256
 
  5
 
 7717
 
 tss
 
 0.93630
 
 0.92596
 
 Not queried
 
  0.6768433
 
       NA
 
       NA
  
 
  
 1
 
  21044031
 
  21044207
 
  ENSEMBL   UCSC 
 
  177
 
  4
 
 293
 
 gene
 
 0.06530
 
 0.04891
 
 Not queried
 
  0.6767596
 
       NA
 
       NA
  
 
  
 5
 
 102455846
 
 102456179
 
  ENSEMBL   UCSC 
 
  334
 
  9
 
 32140
 
 tss
 
 0.02009
 
 0.02501
 
 Not queried
 
  0.6767365
 
       NA
 
       NA
  
 
  
 14
 
 105262269
 
 105262406
 
  ENSEMBL   UCSC 
 
  138
 
  5
 
 12634
 
 tss;island
 
 0.07178
 
 0.03992
 
 Not queried
 
  0.6767208
 
       NA
 
       NA
  
 
  
 7
 
   1062652
 
   1062965
 
  ENSEMBL   UCSC 
 
  314
 
  9
 
 36674
 
 gene;island
 
 0.78022
 
 0.80951
 
 Not queried
 
  0.6766453
 
       NA
 
       NA
  
 
  
 17
 
  43138209
 
  43139021
 
  ENSEMBL   UCSC 
 
  813
 
 12
 
 17389
 
 tss;island
 
 0.01889
 
 0.03247
 
 Not queried
 
  0.6765373
 
       NA
 
       NA
  
 
  
 12
 
  50794308
 
  50794574
 
  ENSEMBL   UCSC 
 
  267
 
  7
 
 9680
 
 tss
 
 0.02260
 
 0.03731
 
 Not queried
 
  0.6761284
 
       NA
 
       NA
  
 
  
 13
 
 113992056
 
 113992391
 
  ENSEMBL   UCSC 
 
  336
 
  4
 
 11255
 
 gene
 
 0.84067
 
 0.89145
 
 Not queried
 
  0.6760810
 
       NA
 
       NA
  
 
  
 2
 
 173420763
 
 173421107
 
  ENSEMBL   UCSC 
 
  345
 
  5
 
 24828
 
 island
 
 0.01604
 
 0.03575
 
 Not queried
 
  0.6759696
 
       NA
 
       NA
  
 
  
 17
 
  49008322
 
  49008925
 
  ENSEMBL   UCSC 
 
  604
 
  7
 
 18529
 
 island
 
 0.03672
 
 0.03263
 
 Not queried
 
  0.6759638
 
       NA
 
       NA
  
 
  
 3
 
 119379414
 
 119379792
 
  ENSEMBL   UCSC 
 
  379
 
  4
 
 28481
 
 tss
 
 0.69550
 
 0.77835
 
 Not queried
 
  0.6758951
 
       NA
 
       NA
  
 
  
 3
 
   9594151
 
   9594385
 
  ENSEMBL   UCSC 
 
  235
 
  4
 
 27611
 
 gene;island
 
 0.09471
 
 0.14725
 
 Not queried
 
  0.6758886
 
       NA
 
       NA
  
 
  
 17
 
  37607407
 
  37607663
 
  ENSEMBL   UCSC 
 
  257
 
  7
 
 18241
 
 island
 
 0.00865
 
 0.00638
 
 Not queried
 
  0.6758034
 
       NA
 
       NA
  
 
  
 6
 
 139695356
 
 139696016
 
  ENSEMBL   UCSC 
 
  661
 
 17
 
 35194
 
 tss
 
 0.02322
 
 0.03080
 
 Not queried
 
  0.6756875
 
       NA
 
       NA
  
 
  
 6
 
 139695202
 
 139696016
 
  ENSEMBL   UCSC 
 
  815
 
 19
 
 36459
 
 island
 
 0.02191
 
 0.02964
 
 Not queried
 
  0.6756875
 
       NA
 
       NA
  
 
  
 8
 
  41507621
 
  41507937
 
  ENSEMBL   UCSC 
 
  317
 
  5
 
 40184
 
 island
 
 0.81250
 
 0.87087
 
 Not queried
 
  0.6756545
 
       NA
 
       NA
  
 
  
 6
 
  40995889
 
  40996213
 
  ENSEMBL   UCSC 
 
  325
 
  5
 
 34262
 
 gene;island
 
 0.05437
 
 0.07093
 
 Not queried
 
  0.6756187
 
       NA
 
       NA
  
 
  
 19
 
  54704426
 
  54704704
 
  ENSEMBL   UCSC 
 
  279
 
  8
 
 21022
 
 tss
 
 0.03203
 
 0.07459
 
 Not queried
 
  0.6756166
 
       NA
 
       NA
  
 
  
 15
 
  76016056
 
  76016306
 
  ENSEMBL   UCSC 
 
  251
 
  4
 
 13790
 
 tss
 
 0.52328
 
 0.65462
 
 Not queried
 
  0.6756069
 
       NA
 
       NA
  
 
  
 6
 
  73329988
 
  73330358
 
  ENSEMBL   UCSC 
 
  371
 
  6
 
 36184
 
 island
 
 0.04342
 
 0.08118
 
 Not queried
 
  0.6754055
 
       NA
 
       NA
  
 
  
 20
 
  44993182
 
  44993436
 
  ENSEMBL   UCSC 
 
  255
 
  7
 
 25751
 
 tss
 
 0.03550
 
 0.04000
 
 Not queried
 
  0.6753137
 
       NA
 
       NA
  
 
  
 16
 
  67753310
 
  67753554
 
  ENSEMBL   UCSC 
 
  245
 
  9
 
 15279
 
 tss
 
 0.01505
 
 0.01824
 
 Not queried
 
  0.6752022
 
       NA
 
       NA
  
 
  
 17
 
  80200634
 
  80200974
 
  ENSEMBL   UCSC 
 
  341
 
  4
 
 18945
 
 island
 
 0.87047
 
 0.92268
 
 Not queried
 
  0.6751653
 
       NA
 
       NA
  
 
  
 2
 
   7057153
 
   7057402
 
  ENSEMBL   UCSC 
 
  250
 
  6
 
 23130
 
 tss;island
 
 0.09608
 
 0.05847
 
 Not queried
 
  0.6750899
 
       NA
 
       NA
  
 
  
 19
 
   4471596
 
   4471839
 
  ENSEMBL   UCSC 
 
  244
 
  4
 
 20329
 
 tss;island
 
 0.01731
 
 0.03099
 
 Not queried
 
  0.6750574
 
       NA
 
       NA
  
 
  
 19
 
  54704426
 
  54705194
 
  ENSEMBL   UCSC 
 
  769
 
 12
 
 22369
 
 island
 
 0.02228
 
 0.04409
 
 Not queried
 
  0.6750110
 
       NA
 
       NA
  
 
  
 11
 
  17410182
 
  17410554
 
  ENSEMBL   UCSC 
 
  373
 
  4
 
 6505
 
 gene;island
 
 0.13763
 
 0.21792
 
 Not queried
 
  0.6749257
 
       NA
 
       NA
  
 
  
 21
 
  45079144
 
  45079336
 
  ENSEMBL   UCSC 
 
  193
 
  5
 
 26405
 
 gene;tss;island
 
 0.05208
 
 0.07137
 
 Not queried
 
  0.6748596
 
       NA
 
       NA
  
 
  
 16
 
  73096842
 
  73097142
 
  ENSEMBL   UCSC 
 
  301
 
  6
 
 16052
 
 island
 
 0.02000
 
 0.03776
 
 Not queried
 
  0.6746118
 
       NA
 
       NA
  
 
  
 11
 
  24518149
 
  24518448
 
  ENSEMBL   UCSC 
 
  300
 
  9
 
 7185
 
 tss;island
 
 0.03334
 
 0.04520
 
 Not queried
 
  0.6745278
 
       NA
 
       NA
  
 
  
 10
 
 125767795
 
 125768098
 
  ENSEMBL   UCSC 
 
  304
 
  4
 
 4788
 
 gene
 
 0.87883
 
 0.89888
 
 Not queried
 
  0.6745049
 
       NA
 
       NA
  
 
  
 16
 
  25228569
 
  25228709
 
  ENSEMBL   UCSC 
 
  141
 
  4
 
 14669
 
 gene
 
 0.92191
 
 0.88436
 
 Not queried
 
  0.6744873
 
       NA
 
       NA
  
 
  
 1
 
  85741866
 
  85742210
 
  ENSEMBL   UCSC 
 
  345
 
  4
 
 604
 
 gene;island
 
 0.01859
 
 0.03438
 
 Not queried
 
  0.6744669
 
       NA
 
       NA
  
 
  
 17
 
  37009861
 
  37010237
 
  ENSEMBL   UCSC 
 
  377
 
  5
 
 17267
 
 tss;island
 
 0.01054
 
 0.01808
 
 Not queried
 
  0.6743875
 
       NA
 
       NA
  
 
  
 7
 
 149570931
 
 149571373
 
  ENSEMBL   UCSC 
 
  443
 
 12
 
 37206
 
 gene;island
 
 0.02070
 
 0.02955
 
 Not queried
 
  0.6743421
 
       NA
 
       NA
  
 
  
 1
 
    982189
 
    982491
 
  ENSEMBL   UCSC 
 
  303
 
  4
 
 9
 
 gene;island
 
 0.83239
 
 0.75056
 
 Not queried
 
  0.6742706
 
       NA
 
       NA
  
 
  
 4
 
    851793
 
    852280
 
  ENSEMBL   UCSC 
 
  488
 
  5
 
 29796
 
 gene
 
 0.92430
 
 0.89217
 
 Not queried
 
  0.6742449
 
       NA
 
       NA
  
 
  
 1
 
 202896457
 
 202896601
 
  ENSEMBL   UCSC 
 
  145
 
  4
 
 2181
 
 tss
 
 0.02162
 
 0.02923
 
 Not queried
 
  0.6742344
 
       NA
 
       NA
  
 
  
 11
 
  66383961
 
  66384069
 
  ENSEMBL   UCSC 
 
  109
 
  6
 
 8498
 
 island
 
 0.02761
 
 0.04087
 
 Not queried
 
  0.6742118
 
       NA
 
       NA
  
 
  
 3
 
  55523815
 
  55524048
 
  ENSEMBL   UCSC 
 
  234
 
  4
 
 29174
 
 island
 
 0.06911
 
 0.04841
 
 Not queried
 
  0.6740660
 
       NA
 
       NA
  
 
  
 7
 
  43909044
 
  43909515
 
  ENSEMBL   UCSC 
 
  472
 
 11
 
 38384
 
 island
 
 0.01799
 
 0.02737
 
 Not queried
 
  0.6740486
 
       NA
 
       NA
  
 
  
 11
 
  61129269
 
  61129520
 
  ENSEMBL   UCSC 
 
  252
 
  8
 
 6608
 
 gene;island
 
 0.02540
 
 0.04371
 
 Not queried
 
  0.6739387
 
       NA
 
       NA
  
 
  
 17
 
  80674481
 
  80674520
 
  ENSEMBL   UCSC 
 
   40
 
  5
 
 17746
 
 tss
 
 0.03213
 
 0.05226
 
 Not queried
 
  0.6739151
 
       NA
 
       NA
  
 
  
 6
 
 127587654
 
 127588115
 
  ENSEMBL   UCSC 
 
  462
 
  8
 
 36407
 
 island
 
 0.01058
 
 0.02703
 
 Not queried
 
  0.6739106
 
       NA
 
       NA
  
 
  
 21
 
  43431083
 
  43431529
 
  ENSEMBL   UCSC 
 
  447
 
  4
 
 26521
 
 tss;island
 
 0.11107
 
 0.05660
 
 Not queried
 
  0.6738894
 
       NA
 
       NA
  
 
  
 11
 
  43596920
 
  43597273
 
  ENSEMBL   UCSC 
 
  354
 
  5
 
 8193
 
 island
 
 0.03858
 
 0.02453
 
 Not queried
 
  0.6738484
 
       NA
 
       NA
  
 
  
 14
 
  70346798
 
  70346984
 
  ENSEMBL   UCSC 
 
  187
 
  4
 
 12100
 
 gene;island
 
 0.04790
 
 0.02138
 
 Not queried
 
  0.6738262
 
       NA
 
       NA
  
 
  
 7
 
   5395900
 
   5396436
 
  ENSEMBL   UCSC 
 
  537
 
  5
 
 36810
 
 gene;island
 
 0.94771
 
 0.95143
 
 Not queried
 
  0.6738176
 
       NA
 
       NA
  
 
  
 13
 
  36429936
 
  36430134
 
  ENSEMBL   UCSC 
 
  199
 
  4
 
 11100
 
 gene
 
 0.14333
 
 0.04950
 
 Not queried
 
  0.6738132
 
       NA
 
       NA
  
 
  
 17
 
   7530871
 
   7531394
 
  ENSEMBL   UCSC 
 
  524
 
 13
 
 16370
 
 gene;tss;island
 
 0.01197
 
 0.01965
 
 Not queried
 
  0.6737334
 
       NA
 
       NA
  
 
  
 15
 
  41166042
 
  41166443
 
  ENSEMBL   UCSC 
 
  402
 
  5
 
 13270
 
 gene
 
 0.01920
 
 0.04587
 
 Not queried
 
  0.6737128
 
       NA
 
       NA
  
 
  
 7
 
 107300939
 
 107301048
 
  ENSEMBL   UCSC 
 
  110
 
  5
 
 37764
 
 tss
 
 0.27448
 
 0.23081
 
 Not queried
 
  0.6736596
 
       NA
 
       NA
  
 
  
 22
 
  30639611
 
  30639979
 
  ENSEMBL   UCSC 
 
  369
 
  4
 
 26794
 
 gene;island
 
 0.91717
 
 0.86276
 
 Not queried
 
  0.6736559
 
       NA
 
       NA
  
 
  
 2
 
  69239676
 
  69240197
 
  ENSEMBL   UCSC 
 
  522
 
  8
 
 23365
 
 tss;island
 
 0.02870
 
 0.03643
 
 Not queried
 
  0.6736505
 
       NA
 
       NA
  
 
  
 19
 
  37958346
 
  37958752
 
  ENSEMBL   UCSC 
 
  407
 
  7
 
 20702
 
 tss;island
 
 0.02880
 
 0.01905
 
 Not queried
 
  0.6736476
 
       NA
 
       NA
  
 
  
 5
 
 111496357
 
 111496724
 
  ENSEMBL   UCSC 
 
  368
 
  6
 
 31654
 
 gene
 
 0.03065
 
 0.04988
 
 Not queried
 
  0.6736476
 
       NA
 
       NA
  
 
  
 8
 
 124657935
 
 124658143
 
  ENSEMBL   UCSC 
 
  209
 
  4
 
 39357
 
 gene
 
 0.87453
 
 0.85260
 
 Not queried
 
  0.6734814
 
       NA
 
       NA
  
 
  
 2
 
 242576809
 
 242576858
 
  ENSEMBL   UCSC 
 
   50
 
  4
 
 23954
 
 tss;island
 
 0.02003
 
 0.02620
 
 Not queried
 
  0.6734778
 
       NA
 
       NA
  
 
  
 14
 
  74892986
 
  74893183
 
  ENSEMBL   UCSC 
 
  198
 
  5
 
 12478
 
 tss;island
 
 0.04497
 
 0.05268
 
 Not queried
 
  0.6734756
 
       NA
 
       NA
  
 
  
 11
 
 116694114
 
 116694372
 
  ENSEMBL   UCSC 
 
  259
 
  5
 
 7690
 
 tss
 
 0.51298
 
 0.58090
 
 Not queried
 
  0.6734547
 
       NA
 
       NA
  
 
  
 13
 
  93879303
 
  93879769
 
  ENSEMBL   UCSC 
 
  467
 
  5
 
 11162
 
 gene;island
 
 0.01954
 
 0.02660
 
 Not queried
 
  0.6734289
 
       NA
 
       NA
  
 
  
 12
 
  56727304
 
  56727976
 
  ENSEMBL   UCSC 
 
  673
 
 15
 
 10508
 
 island
 
 0.02891
 
 0.03610
 
 Not queried
 
  0.6734263
 
       NA
 
       NA
  
 
  
 14
 
  50359443
 
  50359703
 
  ENSEMBL   UCSC 
 
  261
 
  6
 
 12356
 
 tss
 
 0.00895
 
 0.01420
 
 Not queried
 
  0.6733994
 
       NA
 
       NA
  
 
  
 18
 
  21083211
 
  21083459
 
  ENSEMBL   UCSC 
 
  249
 
  5
 
 19172
 
 tss
 
 0.01532
 
 0.01997
 
 Not queried
 
  0.6732748
 
       NA
 
       NA
  
 
  
 1
 
  78148262
 
  78148604
 
  ENSEMBL   UCSC 
 
  343
 
  8
 
 3390
 
 island
 
 0.01800
 
 0.03100
 
 Not queried
 
  0.6732380
 
       NA
 
       NA
  
 
  
 18
 
  46476822
 
  46477404
 
  ENSEMBL   UCSC 
 
  583
 
  8
 
 19467
 
 island
 
 0.03031
 
 0.08446
 
 Not queried
 
  0.6732104
 
       NA
 
       NA
  
 
  
 4
 
  77135005
 
  77135506
 
  ENSEMBL   UCSC 
 
  502
 
  8
 
 31062
 
 island
 
 0.03396
 
 0.02017
 
 Not queried
 
  0.6732022
 
       NA
 
       NA
  
 
  
 20
 
  61298068
 
  61298156
 
  ENSEMBL   UCSC 
 
   89
 
  4
 
 25453
 
 gene;tss
 
 0.64824
 
 0.73925
 
 Not queried
 
  0.6731635
 
       NA
 
       NA
  
 
  
 17
 
  40610755
 
  40611088
 
  ENSEMBL   UCSC 
 
  334
 
  9
 
 18305
 
 island
 
 0.01878
 
 0.02299
 
 Not queried
 
  0.6731526
 
       NA
 
       NA
  
 
  
 19
 
  10764351
 
  10764813
 
  ENSEMBL   UCSC 
 
  463
 
 12
 
 20420
 
 tss;island
 
 0.00686
 
 0.01236
 
 Not queried
 
  0.6731238
 
       NA
 
       NA
  
 
  
 6
 
  27280122
 
  27280722
 
  ENSEMBL   UCSC 
 
  601
 
 10
 
 34651
 
 tss
 
 0.02175
 
 0.05250
 
 Not queried
 
  0.6730481
 
       NA
 
       NA
  
 
  
 13
 
 114126676
 
 114127206
 
  ENSEMBL   UCSC 
 
  531
 
  6
 
 11261
 
 gene
 
 0.95868
 
 0.95655
 
 Not queried
 
  0.6727691
 
       NA
 
       NA
  
 
  
 15
 
  68346336
 
  68346669
 
  ENSEMBL   UCSC 
 
  334
 
  7
 
 14228
 
 island
 
 0.01857
 
 0.03703
 
 Not queried
 
  0.6727572
 
       NA
 
       NA
  
 
  
 3
 
  50310484
 
  50311009
 
  ENSEMBL   UCSC 
 
  526
 
  7
 
 27756
 
 gene;island
 
 0.04866
 
 0.03611
 
 Not queried
 
  0.6727155
 
       NA
 
       NA
  
 
  
 6
 
  33262579
 
  33263475
 
  ENSEMBL   UCSC 
 
  897
 
 13
 
 34189
 
 gene;island
 
 0.72520
 
 0.82825
 
 Not queried
 
  0.6725434
 
       NA
 
       NA
  
 
  
 1
 
 145473005
 
 145473392
 
  ENSEMBL   UCSC 
 
  388
 
  6
 
 723
 
 gene;island
 
 0.01819
 
 0.03187
 
 Not queried
 
  0.6724595
 
       NA
 
       NA
  
 
  
 2
 
 239197356
 
 239197782
 
  ENSEMBL   UCSC 
 
  427
 
  4
 
 23935
 
 tss;island
 
 0.07110
 
 0.10842
 
 Not queried
 
  0.6723991
 
       NA
 
       NA
  
 
  
 20
 
   2384240
 
   2384464
 
  ENSEMBL   UCSC 
 
  225
 
  4
 
 25294
 
 gene;island
 
 0.79111
 
 0.68304
 
 Not queried
 
  0.6723811
 
       NA
 
       NA
  
 
  
 4
 
 175204418
 
 175205489
 
  ENSEMBL   UCSC 
 
 1072
 
 20
 
 30136
 
 gene;island
 
 0.01187
 
 0.01912
 
 Not queried
 
  0.6723520
 
       NA
 
       NA
  
 
  
 X
 
 102862559
 
 102863013
 
  ENSEMBL   UCSC 
 
  455
 
 10
 
 41927
 
 island
 
 0.05582
 
 0.02559
 
 Not queried
 
  0.6723086
 
       NA
 
       NA
  
 
  
 6
 
  11771473
 
  11771840
 
  ENSEMBL   UCSC 
 
  368
 
  4
 
 33508
 
 gene
 
 0.70921
 
 0.80624
 
 Not queried
 
  0.6721557
 
       NA
 
       NA
  
 
  
 14
 
 102357944
 
 102358100
 
  ENSEMBL   UCSC 
 
  157
 
  4
 
 12189
 
 gene;island
 
 0.85519
 
 0.90430
 
 Not queried
 
  0.6720756
 
       NA
 
       NA
  
 
  
 11
 
   1316693
 
   1317114
 
  ENSEMBL   UCSC 
 
  422
 
  5
 
 6394
 
 gene;island
 
 0.86779
 
 0.93350
 
 Not queried
 
  0.6720199
 
       NA
 
       NA
  
 
  
 19
 
  10541207
 
  10541478
 
  ENSEMBL   UCSC 
 
  272
 
  4
 
 19776
 
 gene;tss;island
 
 0.13202
 
 0.21334
 
 Not queried
 
  0.6718428
 
       NA
 
       NA
  
 
  
 11
 
 126173650
 
 126174041
 
  ENSEMBL   UCSC 
 
  392
 
  5
 
 6942
 
 gene
 
 0.03644
 
 0.04329
 
 Not queried
 
  0.6717901
 
       NA
 
       NA
  
 
  
 6
 
 110501627
 
 110501899
 
  ENSEMBL   UCSC 
 
  273
 
  4
 
 34379
 
 gene
 
 0.01071
 
 0.01585
 
 Not queried
 
  0.6717450
 
       NA
 
       NA
  
 
  
 17
 
  15847977
 
  15848221
 
  ENSEMBL   UCSC 
 
  245
 
  7
 
 17119
 
 tss
 
 0.03012
 
 0.03463
 
 Not queried
 
  0.6717450
 
       NA
 
       NA
  
 
  
 1
 
  89990806
 
  89991016
 
  ENSEMBL   UCSC 
 
  211
 
  4
 
 619
 
 gene;island
 
 0.03530
 
 0.07194
 
 Not queried
 
  0.6717251
 
       NA
 
       NA
  
 
  
 2
 
 219905959
 
 219906468
 
  ENSEMBL   UCSC 
 
  510
 
 10
 
 25065
 
 island
 
 0.01138
 
 0.01703
 
 Not queried
 
  0.6715681
 
       NA
 
       NA
  
 
  
 7
 
   4872926
 
   4873549
 
  ENSEMBL   UCSC 
 
  624
 
  6
 
 36797
 
 gene;island
 
 0.81288
 
 0.77536
 
 Not queried
 
  0.6714789
 
       NA
 
       NA
  
 
  
 2
 
  43453251
 
  43453558
 
  ENSEMBL   UCSC 
 
  308
 
  5
 
 22620
 
 gene;tss;island
 
 0.02194
 
 0.01389
 
 Not queried
 
  0.6714377
 
       NA
 
       NA
  
 
  
 14
 
  23388406
 
  23388579
 
  ENSEMBL   UCSC 
 
  174
 
  4
 
 12262
 
 tss
 
 0.00941
 
 0.00953
 
 Not queried
 
  0.6713931
 
       NA
 
       NA
  
 
  
 14
 
  23388278
 
  23388579
 
  ENSEMBL   UCSC 
 
  302
 
  6
 
 12678
 
 island
 
 0.01192
 
 0.01124
 
 Not queried
 
  0.6713931
 
       NA
 
       NA
  
 
  
 19
 
  35521622
 
  35521856
 
  ENSEMBL   UCSC 
 
  235
 
  4
 
 19943
 
 gene;island
 
 0.01497
 
 0.02195
 
 Not queried
 
  0.6713762
 
       NA
 
       NA
  
 
  
 21
 
  30671675
 
  30671710
 
  ENSEMBL   UCSC 
 
   36
 
  5
 
 26463
 
 tss
 
 0.02086
 
 0.03281
 
 Not queried
 
  0.6713636
 
       NA
 
       NA
  
 
  
 4
 
  47916636
 
  47917094
 
  ENSEMBL   UCSC 
 
  459
 
  7
 
 30332
 
 tss
 
 0.01546
 
 0.01654
 
 Not queried
 
  0.6713130
 
       NA
 
       NA
  
 
  
 16
 
  75467692
 
  75468057
 
  ENSEMBL   UCSC 
 
  366
 
  6
 
 15331
 
 tss;island
 
 0.01794
 
 0.02897
 
 Not queried
 
  0.6709827
 
       NA
 
       NA
  
 
  
 2
 
  24346215
 
  24346267
 
  ENSEMBL   UCSC 
 
   53
 
  4
 
 23186
 
 tss;island
 
 0.01390
 
 0.02057
 
 Not queried
 
  0.6707292
 
       NA
 
       NA
  
 
  
 20
 
  43150917
 
  43151161
 
  ENSEMBL   UCSC 
 
  245
 
  4
 
 25722
 
 tss;island
 
 0.01025
 
 0.01632
 
 Not queried
 
  0.6705862
 
       NA
 
       NA
  
 
  
 1
 
   1310715
 
   1310978
 
  ENSEMBL   UCSC 
 
  264
 
  4
 
 1145
 
 tss;island
 
 0.02026
 
 0.01777
 
 Not queried
 
  0.6703577
 
       NA
 
       NA
  
 
  
 2
 
 128422179
 
 128422717
 
  ENSEMBL   UCSC 
 
  539
 
  8
 
 23576
 
 tss
 
 0.79719
 
 0.83204
 
 Not queried
 
  0.6701749
 
       NA
 
       NA
  
 
  
 13
 
  73301012
 
  73301231
 
  ENSEMBL   UCSC 
 
  220
 
  5
 
 11149
 
 gene;tss;island
 
 0.06501
 
 0.02006
 
 Not queried
 
  0.6700091
 
       NA
 
       NA
  
 
  
 2
 
 233415416
 
 233416425
 
  ENSEMBL   UCSC 
 
 1010
 
 14
 
 23029
 
 gene
 
 0.02605
 
 0.02844
 
 Not queried
 
  0.6699818
 
       NA
 
       NA
  
 
  
 10
 
  75935758
 
  75936339
 
  ENSEMBL   UCSC 
 
  582
 
  9
 
 4625
 
 gene;tss;island
 
 0.02813
 
 0.02199
 
 Not queried
 
  0.6699404
 
       NA
 
       NA
  
 
  
 X
 
 107068750
 
 107069063
 
  ENSEMBL   UCSC 
 
  314
 
  5
 
 41463
 
 tss;island
 
 0.07910
 
 0.03764
 
 Not queried
 
  0.6699086
 
       NA
 
       NA
  
 
  
 17
 
  72462164
 
  72462441
 
  ENSEMBL   UCSC 
 
  278
 
  4
 
 17594
 
 tss
 
 0.71119
 
 0.57042
 
 Not queried
 
  0.6698890
 
       NA
 
       NA
  
 
  
 14
 
  78083170
 
  78083524
 
  ENSEMBL   UCSC 
 
  355
 
  5
 
 12505
 
 tss
 
 0.02722
 
 0.04992
 
 Not queried
 
  0.6698627
 
       NA
 
       NA
  
 
  
 3
 
  52812367
 
  52812520
 
  ENSEMBL   UCSC 
 
  154
 
  4
 
 27783
 
 gene;tss
 
 0.82621
 
 0.71124
 
 Not queried
 
  0.6697747
 
       NA
 
       NA
  
 
  
 10
 
   7661611
 
   7661796
 
  ENSEMBL   UCSC 
 
  186
 
  5
 
 4471
 
 gene
 
 0.89353
 
 0.92158
 
 Not queried
 
  0.6697559
 
       NA
 
       NA
  
 
  
 7
 
  27135477
 
  27135780
 
  ENSEMBL   UCSC 
 
  304
 
  8
 
 38272
 
 island
 
 0.01972
 
 0.02851
 
 Not queried
 
  0.6697539
 
       NA
 
       NA
  
 
  
 2
 
 232329239
 
 232329264
 
  ENSEMBL   UCSC 
 
   26
 
  5
 
 23902
 
 tss
 
 0.01415
 
 0.03999
 
 Not queried
 
  0.6697325
 
       NA
 
       NA
  
 
  
 2
 
 232329096
 
 232329264
 
  ENSEMBL   UCSC 
 
  169
 
  7
 
 25144
 
 island
 
 0.01415
 
 0.03999
 
 Not queried
 
  0.6697325
 
       NA
 
       NA
  
 
  
 13
 
  52378179
 
  52378744
 
  ENSEMBL   UCSC 
 
  566
 
 11
 
 11722
 
 island
 
 0.02420
 
 0.02921
 
 Not queried
 
  0.6697280
 
       NA
 
       NA
  
 
  
 19
 
  49964960
 
  49965263
 
  ENSEMBL   UCSC 
 
  304
 
  4
 
 20114
 
 gene;island
 
 0.96690
 
 0.94895
 
 Not queried
 
  0.6697208
 
       NA
 
       NA
  
 
  
 3
 
 183903550
 
 183903797
 
  ENSEMBL   UCSC 
 
  248
 
  6
 
 28710
 
 tss
 
 0.02410
 
 0.01715
 
 Not queried
 
  0.6697149
 
       NA
 
       NA
  
 
  
 17
 
  63557887
 
  63558518
 
  ENSEMBL   UCSC 
 
  632
 
  6
 
 17560
 
 tss
 
 0.10089
 
 0.05548
 
 Not queried
 
  0.6696893
 
       NA
 
       NA
  
 
  
 6
 
 133563764
 
 133564066
 
  ENSEMBL   UCSC 
 
  303
 
  4
 
 34412
 
 gene;island
 
 0.07837
 
 0.13342
 
 Not queried
 
  0.6695637
 
       NA
 
       NA
  
 
  
 3
 
 111717682
 
 111718245
 
  ENSEMBL   UCSC 
 
  564
 
  8
 
 27851
 
 gene
 
 0.05612
 
 0.02516
 
 Not queried
 
  0.6695321
 
       NA
 
       NA
  
 
  
 1
 
  33430314
 
  33430890
 
  ENSEMBL   UCSC 
 
  577
 
 11
 
 1464
 
 tss;island
 
 0.01674
 
 0.03741
 
 Not queried
 
  0.6690905
 
       NA
 
       NA
  
 
  
 1
 
   1102276
 
   1102446
 
  ENSEMBL   UCSC 
 
  171
 
  5
 
 1136
 
 tss;island
 
 0.82580
 
 0.77113
 
 Not queried
 
  0.6690403
 
       NA
 
       NA
  
 
  
 15
 
  49913299
 
  49913596
 
  ENSEMBL   UCSC 
 
  298
 
  6
 
 13314
 
 gene
 
 0.01890
 
 0.03674
 
 Not queried
 
  0.6689358
 
       NA
 
       NA
  
 
  
 22
 
  30234213
 
  30234508
 
  ENSEMBL   UCSC 
 
  296
 
  6
 
 27360
 
 island
 
 0.00977
 
 0.01749
 
 Not queried
 
  0.6689329
 
       NA
 
       NA
  
 
  
 12
 
  16506202
 
  16506341
 
  ENSEMBL   UCSC 
 
  140
 
  4
 
 9045
 
 gene;tss
 
 0.66510
 
 0.79761
 
 Not queried
 
  0.6689308
 
       NA
 
       NA
  
 
  
 4
 
  94749865
 
  94750205
 
  ENSEMBL   UCSC 
 
  341
 
  7
 
 31132
 
 island
 
 0.02315
 
 0.04078
 
 Not queried
 
  0.6688967
 
       NA
 
       NA
  
 
  
 15
 
  74907203
 
  74907290
 
  ENSEMBL   UCSC 
 
   88
 
  4
 
 13768
 
 tss
 
 0.01901
 
 0.03660
 
 Not queried
 
  0.6688826
 
       NA
 
       NA
  
 
  
 1
 
 226595334
 
 226596422
 
  ENSEMBL   UCSC 
 
 1089
 
 10
 
 4247
 
 island
 
 0.02726
 
 0.04229
 
 Not queried
 
  0.6688135
 
       NA
 
       NA
  
 
  
 2
 
  30669597
 
  30670025
 
  ENSEMBL   UCSC 
 
  429
 
  7
 
 23248
 
 tss
 
 0.05543
 
 0.03303
 
 Not queried
 
  0.6687392
 
       NA
 
       NA
  
 
  
 1
 
 240775075
 
 240775401
 
  ENSEMBL   UCSC 
 
  327
 
  4
 
 1104
 
 gene
 
 0.01946
 
 0.02936
 
 Not queried
 
  0.6686950
 
       NA
 
       NA
  
 
  
 1
 
 113615856
 
 113616118
 
  ENSEMBL   UCSC 
 
  263
 
  4
 
 696
 
 gene
 
 0.01948
 
 0.02807
 
 Not queried
 
  0.6685050
 
       NA
 
       NA
  
 
  
 2
 
 242987510
 
 242987840
 
  ENSEMBL   UCSC 
 
  331
 
  4
 
 25282
 
 island
 
 0.64963
 
 0.77861
 
 Not queried
 
  0.6684788
 
       NA
 
       NA
  
 
  
 2
 
 242754333
 
 242754756
 
  ENSEMBL   UCSC 
 
  424
 
  5
 
 23110
 
 gene;island
 
 0.89217
 
 0.88505
 
 Not queried
 
  0.6683000
 
       NA
 
       NA
  
 
  
 1
 
 249152672
 
 249153132
 
  ENSEMBL   UCSC 
 
  461
 
  7
 
 1127
 
 gene;island
 
 0.00867
 
 0.01700
 
 Not queried
 
  0.6681341
 
       NA
 
       NA
  
 
  
 1
 
  24513473
 
  24513722
 
  ENSEMBL   UCSC 
 
  250
 
  4
 
 324
 
 gene
 
 0.02311
 
 0.03275
 
 Not queried
 
  0.6679919
 
       NA
 
       NA
  
 
  
 20
 
  47662406
 
  47662828
 
  ENSEMBL   UCSC 
 
  423
 
  9
 
 25764
 
 tss
 
 0.02178
 
 0.02504
 
 Not queried
 
  0.6679905
 
       NA
 
       NA
  
 
  
 11
 
  30038615
 
  30038797
 
  ENSEMBL   UCSC 
 
  183
 
  9
 
 7198
 
 tss;island
 
 0.02216
 
 0.02606
 
 Not queried
 
  0.6679801
 
       NA
 
       NA
  
 
  
 17
 
  19247837
 
  19248009
 
  ENSEMBL   UCSC 
 
  173
 
  8
 
 16443
 
 gene;island
 
 0.85969
 
 0.90838
 
 Not queried
 
  0.6679298
 
       NA
 
       NA
  
 
  
 11
 
 121970496
 
 121970768
 
  ENSEMBL   UCSC 
 
  273
 
  6
 
 6926
 
 gene
 
 0.05011
 
 0.06244
 
 Not queried
 
  0.6678790
 
       NA
 
       NA
  
 
  
 1
 
 161067636
 
 161068138
 
  ENSEMBL   UCSC 
 
  503
 
  9
 
 2041
 
 tss
 
 0.01461
 
 0.02181
 
 Not queried
 
  0.6677688
 
       NA
 
       NA
  
 
  
 4
 
 155664941
 
 155665249
 
  ENSEMBL   UCSC 
 
  309
 
  7
 
 31333
 
 island
 
 0.03378
 
 0.05380
 
 Not queried
 
  0.6677600
 
       NA
 
       NA
  
 
  
 7
 
  27142100
 
  27142810
 
  ENSEMBL   UCSC 
 
  711
 
 15
 
 38275
 
 island
 
 0.04121
 
 0.06460
 
 Not queried
 
  0.6676406
 
       NA
 
       NA
  
 
  
 1
 
 154244759
 
 154244960
 
  ENSEMBL   UCSC 
 
  202
 
  7
 
 1957
 
 tss;island
 
 0.01597
 
 0.02907
 
 Not queried
 
  0.6676109
 
       NA
 
       NA
  
 
  
 22
 
  39712956
 
  39713086
 
  ENSEMBL   UCSC 
 
  131
 
  6
 
 26835
 
 gene;tss;island
 
 0.57713
 
 0.71503
 
 Not queried
 
  0.6675248
 
       NA
 
       NA
  
 
  
 2
 
  48132755
 
  48133053
 
  ENSEMBL   UCSC 
 
  299
 
  5
 
 24248
 
 island
 
 0.01247
 
 0.03517
 
 Not queried
 
  0.6674527
 
       NA
 
       NA
  
 
  
 19
 
  46088010
 
  46088450
 
  ENSEMBL   UCSC 
 
  441
 
 12
 
 22110
 
 island
 
 0.00623
 
 0.01234
 
 Not queried
 
  0.6674027
 
       NA
 
       NA
  
 
  
 4
 
  96760945
 
  96761231
 
  ENSEMBL   UCSC 
 
  287
 
  5
 
 30459
 
 tss
 
 0.88243
 
 0.92260
 
 Not queried
 
  0.6673814
 
       NA
 
       NA
  
 
  
 16
 
  89044523
 
  89044882
 
  ENSEMBL   UCSC 
 
  360
 
  5
 
 15388
 
 tss
 
 0.58572
 
 0.68697
 
 Not queried
 
  0.6672993
 
       NA
 
       NA
  
 
  
 12
 
  97300803
 
  97300881
 
  ENSEMBL   UCSC 
 
   79
 
  7
 
 9908
 
 tss;island
 
 0.01726
 
 0.05744
 
 Not queried
 
  0.6671759
 
       NA
 
       NA
  
 
  
 11
 
  46298891
 
  46299204
 
  ENSEMBL   UCSC 
 
  314
 
  5
 
 7252
 
 tss
 
 0.03204
 
 0.04241
 
 Not queried
 
  0.6671315
 
       NA
 
       NA
  
 
  
 19
 
  52531703
 
  52531956
 
  ENSEMBL   UCSC 
 
  254
 
  6
 
 20968
 
 tss
 
 0.02260
 
 0.03043
 
 Not queried
 
  0.6670645
 
       NA
 
       NA
  
 
  
 19
 
  52531438
 
  52531956
 
  ENSEMBL   UCSC 
 
  519
 
  8
 
 22312
 
 island
 
 0.01569
 
 0.02783
 
 Not queried
 
  0.6670645
 
       NA
 
       NA
  
 
  
 15
 
  79055416
 
  79055693
 
  ENSEMBL   UCSC 
 
  278
 
  4
 
 13411
 
 gene;island
 
 0.69183
 
 0.73871
 
 Not queried
 
  0.6670432
 
       NA
 
       NA
  
 
  
 5
 
  40679636
 
  40679966
 
  ENSEMBL   UCSC 
 
  331
 
  8
 
 31975
 
 tss
 
 0.01742
 
 0.02736
 
 Not queried
 
  0.6670384
 
       NA
 
       NA
  
 
  
 14
 
  92980026
 
  92980103
 
  ENSEMBL   UCSC 
 
   78
 
  4
 
 12539
 
 tss
 
 0.04084
 
 0.03930
 
 Not queried
 
  0.6670330
 
       NA
 
       NA
  
 
  
 16
 
  58498456
 
  58498754
 
  ENSEMBL   UCSC 
 
  299
 
  7
 
 15243
 
 tss
 
 0.03885
 
 0.07078
 
 Not queried
 
  0.6668160
 
       NA
 
       NA
  
 
  
 17
 
    862212
 
    862384
 
  ENSEMBL   UCSC 
 
  173
 
  4
 
 16265
 
 gene
 
 0.94991
 
 0.96863
 
 Not queried
 
  0.6667632
 
       NA
 
       NA
  
 
  
 11
 
   2018088
 
   2018247
 
  ENSEMBL   UCSC 
 
  160
 
  7
 
 7033
 
 tss
 
 0.81957
 
 0.74418
 
 Not queried
 
  0.6667620
 
       NA
 
       NA
  
 
  
 2
 
 192543570
 
 192543908
 
  ENSEMBL   UCSC 
 
  339
 
  4
 
 22927
 
 gene;island
 
 0.11663
 
 0.12634
 
 Not queried
 
  0.6667396
 
       NA
 
       NA
  
 
  
 15
 
  74833297
 
  74833399
 
  ENSEMBL   UCSC 
 
  103
 
  4
 
 13767
 
 tss;island
 
 0.01978
 
 0.01953
 
 Not queried
 
  0.6666457
 
       NA
 
       NA
  
 
  
 2
 
  10281159
 
  10281345
 
  ENSEMBL   UCSC 
 
  187
 
  5
 
 23141
 
 tss
 
 0.79090
 
 0.85747
 
 Not queried
 
  0.6666242
 
       NA
 
       NA
  
 
  
 X
 
  47004051
 
  47004911
 
  ENSEMBL   UCSC 
 
  861
 
 17
 
 40973
 
 gene;tss;island
 
 0.01141
 
 0.01486
 
 Not queried
 
  0.6665871
 
       NA
 
       NA
  
 
  
 15
 
  25430614
 
  25430671
 
  ENSEMBL   UCSC 
 
   58
 
  4
 
 13501
 
 tss
 
 0.85923
 
 0.81465
 
 Not queried
 
  0.6665557
 
       NA
 
       NA
  
 
  
 5
 
 122110867
 
 122111354
 
  ENSEMBL   UCSC 
 
  488
 
  5
 
 31671
 
 gene
 
 0.03554
 
 0.04386
 
 Not queried
 
  0.6664861
 
       NA
 
       NA
  
 
  
 1
 
   2318621
 
   2318918
 
  ENSEMBL   UCSC 
 
  298
 
  4
 
 80
 
 gene;island
 
 0.80731
 
 0.83139
 
 Not queried
 
  0.6663759
 
       NA
 
       NA
  
 
  
 X
 
  48541820
 
  48542179
 
  ENSEMBL   UCSC 
 
  360
 
  4
 
 41294
 
 tss
 
 0.67389
 
 0.76703
 
 Not queried
 
  0.6662317
 
       NA
 
       NA
  
 
  
 3
 
   3168142
 
   3168585
 
  ENSEMBL   UCSC 
 
  444
 
  9
 
 28070
 
 tss
 
 0.01998
 
 0.01905
 
 Not queried
 
  0.6661358
 
       NA
 
       NA
  
 
  
 10
 
 118033581
 
 118034031
 
  ENSEMBL   UCSC 
 
  451
 
  4
 
 5366
 
 tss;island
 
 0.02904
 
 0.05230
 
 Not queried
 
  0.6661238
 
       NA
 
       NA
  
 
  
 17
 
  19266095
 
  19266474
 
  ENSEMBL   UCSC 
 
  380
 
 10
 
 17154
 
 tss
 
 0.02414
 
 0.03730
 
 Not queried
 
  0.6661230
 
       NA
 
       NA
  
 
  
 17
 
  19265900
 
  19266474
 
  ENSEMBL   UCSC 
 
  575
 
 12
 
 18062
 
 island
 
 0.02494
 
 0.03730
 
 Not queried
 
  0.6661230
 
       NA
 
       NA
  
 
  
 11
 
  85358760
 
  85359218
 
  ENSEMBL   UCSC 
 
  459
 
  9
 
 8681
 
 island
 
 0.01756
 
 0.01912
 
 Not queried
 
  0.6661022
 
       NA
 
       NA
  
 
  
 6
 
  33288180
 
  33289280
 
  ENSEMBL   UCSC 
 
 1101
 
 36
 
 34203
 
 gene;island
 
 0.86876
 
 0.90731
 
 Not queried
 
  0.6660922
 
       NA
 
       NA
  
 
  
 19
 
   1479291
 
   1479573
 
  ENSEMBL   UCSC 
 
  283
 
 10
 
 20270
 
 tss
 
 0.02659
 
 0.03989
 
 Not queried
 
  0.6660461
 
       NA
 
       NA
  
 
  
 4
 
  25657365
 
  25657429
 
  ENSEMBL   UCSC 
 
   65
 
  5
 
 30287
 
 tss
 
 0.02531
 
 0.05051
 
 Not queried
 
  0.6659485
 
       NA
 
       NA
  
 
  
 4
 
  25657365
 
  25657628
 
  ENSEMBL   UCSC 
 
  264
 
  7
 
 30912
 
 island
 
 0.02531
 
 0.05051
 
 Not queried
 
  0.6659485
 
       NA
 
       NA
  
 
  
 8
 
 124084365
 
 124084822
 
  ENSEMBL   UCSC 
 
  458
 
  6
 
 39832
 
 tss;island
 
 0.01160
 
 0.04071
 
 Not queried
 
  0.6659474
 
       NA
 
       NA
  
 
  
 21
 
  43221380
 
  43221841
 
  ENSEMBL   UCSC 
 
  462
 
  5
 
 26390
 
 gene;island
 
 0.82171
 
 0.82839
 
 Not queried
 
  0.6658226
 
       NA
 
       NA
  
 
  
 6
 
 100054469
 
 100055173
 
  ENSEMBL   UCSC 
 
  705
 
 11
 
 36295
 
 island
 
 0.12543
 
 0.29567
 
 Not queried
 
  0.6658155
 
       NA
 
       NA
  
 
  
 10
 
  50970428
 
  50970649
 
  ENSEMBL   UCSC 
 
  222
 
  8
 
 5060
 
 tss
 
 0.02182
 
 0.02583
 
 Not queried
 
  0.6657767
 
       NA
 
       NA
  
 
  
 12
 
  54402697
 
  54402888
 
  ENSEMBL   UCSC 
 
  192
 
  6
 
 9727
 
 tss
 
 0.01699
 
 0.03431
 
 Not queried
 
  0.6657664
 
       NA
 
       NA
  
 
  
 2
 
  30144547
 
  30144838
 
  ENSEMBL   UCSC 
 
  292
 
  6
 
 23245
 
 tss
 
 0.01142
 
 0.01922
 
 Not queried
 
  0.6655634
 
       NA
 
       NA
  
 
  
 4
 
  57301960
 
  57302115
 
  ENSEMBL   UCSC 
 
  156
 
  6
 
 29963
 
 gene;tss;island
 
 0.01714
 
 0.01530
 
 Not queried
 
  0.6655342
 
       NA
 
       NA
  
 
  
 1
 
 147400503
 
 147400607
 
  ENSEMBL   UCSC 
 
  105
 
  4
 
 3687
 
 island
 
 0.01188
 
 0.02657
 
 Not queried
 
  0.6655149
 
       NA
 
       NA
  
 
  
 4
 
  55991782
 
  55991943
 
  ENSEMBL   UCSC 
 
  162
 
  6
 
 30355
 
 tss
 
 0.02997
 
 0.08016
 
 Not queried
 
  0.6655088
 
       NA
 
       NA
  
 
  
 17
 
  77030221
 
  77030546
 
  ENSEMBL   UCSC 
 
  326
 
  5
 
 16798
 
 gene
 
 0.47554
 
 0.63738
 
 Not queried
 
  0.6654946
 
       NA
 
       NA
  
 
  
 14
 
  93215038
 
  93215209
 
  ENSEMBL   UCSC 
 
  172
 
  6
 
 12540
 
 tss
 
 0.02901
 
 0.04053
 
 Not queried
 
  0.6653778
 
       NA
 
       NA
  
 
  
 17
 
  37307911
 
  37308278
 
  ENSEMBL   UCSC 
 
  368
 
  6
 
 17270
 
 tss;island
 
 0.05235
 
 0.03588
 
 Not queried
 
  0.6653448
 
       NA
 
       NA
  
 
  
 3
 
 169378675
 
 169379010
 
  ENSEMBL   UCSC 
 
  336
 
  5
 
 27979
 
 gene;island
 
 0.09599
 
 0.04697
 
 Not queried
 
  0.6652687
 
       NA
 
       NA
  
 
  
 3
 
   2139977
 
   2140256
 
  ENSEMBL   UCSC 
 
  280
 
  4
 
 28789
 
 island
 
 0.02607
 
 0.06411
 
 Not queried
 
  0.6651886
 
       NA
 
       NA
  
 
  
 19
 
  16999576
 
  16999953
 
  ENSEMBL   UCSC 
 
  378
 
  7
 
 21690
 
 island
 
 0.75827
 
 0.78270
 
 Not queried
 
  0.6649966
 
       NA
 
       NA
  
 
  
 11
 
 130786525
 
 130786720
 
  ENSEMBL   UCSC 
 
  196
 
  7
 
 7799
 
 tss
 
 0.02402
 
 0.01595
 
 Not queried
 
  0.6649945
 
       NA
 
       NA
  
 
  
 3
 
 107809956
 
 107810145
 
  ENSEMBL   UCSC 
 
  190
 
  4
 
 28444
 
 tss;island
 
 0.01722
 
 0.01998
 
 Not queried
 
  0.6649205
 
       NA
 
       NA
  
 
  
 3
 
 142681401
 
 142681704
 
  ENSEMBL   UCSC 
 
  304
 
  4
 
 27935
 
 gene;island
 
 0.92291
 
 0.82264
 
 Not queried
 
  0.6646677
 
       NA
 
       NA
  
 
  
 1
 
 166845374
 
 166845800
 
  ENSEMBL   UCSC 
 
  427
 
 11
 
 3927
 
 island
 
 0.01696
 
 0.03222
 
 Not queried
 
  0.6646349
 
       NA
 
       NA
  
 
  
 16
 
  50058938
 
  50059188
 
  ENSEMBL   UCSC 
 
  251
 
  7
 
 15878
 
 island
 
 0.02568
 
 0.04300
 
 Not queried
 
  0.6646181
 
       NA
 
       NA
  
 
  
 2
 
 236293335
 
 236293610
 
  ENSEMBL   UCSC 
 
  276
 
  5
 
 25179
 
 island
 
 0.75562
 
 0.68065
 
 Not queried
 
  0.6646173
 
       NA
 
       NA
  
 
  
 17
 
  27279400
 
  27279763
 
  ENSEMBL   UCSC 
 
  364
 
  4
 
 17196
 
 tss;island
 
 0.05591
 
 0.04270
 
 Not queried
 
  0.6645875
 
       NA
 
       NA
  
 
  
 10
 
  98945775
 
  98946238
 
  ENSEMBL   UCSC 
 
  464
 
 10
 
 5240
 
 tss
 
 0.04659
 
 0.05053
 
 Not queried
 
  0.6645112
 
       NA
 
       NA
  
 
  
 16
 
  50581707
 
  50582193
 
  ENSEMBL   UCSC 
 
  487
 
  5
 
 15198
 
 tss;island
 
 0.02055
 
 0.02862
 
 Not queried
 
  0.6644913
 
       NA
 
       NA
  
 
  
 15
 
  52311035
 
  52311631
 
  ENSEMBL   UCSC 
 
  597
 
  6
 
 14118
 
 island
 
 0.05072
 
 0.04765
 
 Not queried
 
  0.6643515
 
       NA
 
       NA
  
 
  
 12
 
  69863677
 
  69864352
 
  ENSEMBL   UCSC 
 
  676
 
  9
 
 10588
 
 island
 
 0.01652
 
 0.02541
 
 Not queried
 
  0.6643427
 
       NA
 
       NA
  
 
  
 17
 
  16342463
 
  16343134
 
  ENSEMBL   UCSC 
 
  672
 
 13
 
 16423
 
 gene;tss;island
 
 0.01349
 
 0.01781
 
 Not queried
 
  0.6642461
 
       NA
 
       NA
  
 
  
 12
 
 124069168
 
 124069758
 
  ENSEMBL   UCSC 
 
  591
 
  5
 
 9358
 
 gene
 
 0.00667
 
 0.01057
 
 Not queried
 
  0.6641659
 
       NA
 
       NA
  
 
  
 15
 
  62358751
 
  62359275
 
  ENSEMBL   UCSC 
 
  525
 
  7
 
 14170
 
 island
 
 0.04833
 
 0.04775
 
 Not queried
 
  0.6640549
 
       NA
 
       NA
  
 
  
 6
 
 166419479
 
 166419943
 
  ENSEMBL   UCSC 
 
  465
 
  6
 
 36563
 
 island
 
 0.06431
 
 0.03670
 
 Not queried
 
  0.6640079
 
       NA
 
       NA
  
 
  
 11
 
  16759900
 
  16760677
 
  ENSEMBL   UCSC 
 
  778
 
 16
 
 8071
 
 island
 
 0.02518
 
 0.04364
 
 Not queried
 
  0.6639900
 
       NA
 
       NA
  
 
  
 3
 
 197676755
 
 197676828
 
  ENSEMBL   UCSC 
 
   74
 
  4
 
 28066
 
 gene;tss
 
 0.00747
 
 0.01525
 
 Not queried
 
  0.6639806
 
       NA
 
       NA
  
 
  
 11
 
 124543402
 
 124543660
 
  ENSEMBL   UCSC 
 
  259
 
  6
 
 6933
 
 gene
 
 0.01417
 
 0.03002
 
 Not queried
 
  0.6638545
 
       NA
 
       NA
  
 
  
 2
 
  70417854
 
  70418309
 
  ENSEMBL   UCSC 
 
  456
 
 10
 
 24340
 
 island
 
 0.02439
 
 0.03735
 
 Not queried
 
  0.6635863
 
       NA
 
       NA
  
 
  
 4
 
    646293
 
    646577
 
  ENSEMBL   UCSC 
 
  285
 
  5
 
 29785
 
 gene;tss;island
 
 0.92553
 
 0.95028
 
 Not queried
 
  0.6635401
 
       NA
 
       NA
  
 
  
 1
 
  92683403
 
  92683554
 
  ENSEMBL   UCSC 
 
  152
 
  5
 
 1745
 
 tss
 
 0.93051
 
 0.86796
 
 Not queried
 
  0.6634934
 
       NA
 
       NA
  
 
  
 19
 
  12250778
 
  12251131
 
  ENSEMBL   UCSC 
 
  354
 
  4
 
 19816
 
 gene
 
 0.01676
 
 0.03491
 
 Not queried
 
  0.6634779
 
       NA
 
       NA
  
 
  
 8
 
 117886887
 
 117887318
 
  ENSEMBL   UCSC 
 
  432
 
 11
 
 40487
 
 island
 
 0.01662
 
 0.03065
 
 Not queried
 
  0.6634239
 
       NA
 
       NA
  
 
  
 8
 
  56436393
 
  56436771
 
  ENSEMBL   UCSC 
 
  379
 
  5
 
 39247
 
 gene
 
 0.91634
 
 0.94432
 
 Not queried
 
  0.6633644
 
       NA
 
       NA
  
 
  
 4
 
   4389509
 
   4389918
 
  ENSEMBL   UCSC 
 
  410
 
  4
 
 29870
 
 gene;island
 
 0.09388
 
 0.16963
 
 Not queried
 
  0.6633258
 
       NA
 
       NA
  
 
  
 4
 
 158141449
 
 158141570
 
  ENSEMBL   UCSC 
 
  122
 
  5
 
 30606
 
 tss;island
 
 0.03132
 
 0.12667
 
 Not queried
 
  0.6633069
 
       NA
 
       NA
  
 
  
 4
 
 178230851
 
 178230972
 
  ENSEMBL   UCSC 
 
  122
 
  7
 
 30642
 
 tss
 
 0.01609
 
 0.02487
 
 Not queried
 
  0.6632274
 
       NA
 
       NA
  
 
  
 14
 
 100111183
 
 100111392
 
  ENSEMBL   UCSC 
 
  210
 
  8
 
 12571
 
 tss;island
 
 0.20930
 
 0.26247
 
 Not queried
 
  0.6631986
 
       NA
 
       NA
  
 
  
 2
 
  96990762
 
  96991327
 
  ENSEMBL   UCSC 
 
  566
 
  9
 
 23477
 
 tss;island
 
 0.08185
 
 0.04109
 
 Not queried
 
  0.6631355
 
       NA
 
       NA
  
 
  
 16
 
  56691551
 
  56691849
 
  ENSEMBL   UCSC 
 
  299
 
  7
 
 15219
 
 tss
 
 0.01476
 
 0.04047
 
 Not queried
 
  0.6631058
 
       NA
 
       NA
  
 
  
 1
 
  27319836
 
  27320132
 
  ENSEMBL   UCSC 
 
  297
 
  6
 
 1399
 
 tss
 
 0.01041
 
 0.01197
 
 Not queried
 
  0.6630331
 
       NA
 
       NA
  
 
  
 19
 
   1449857
 
   1450023
 
  ENSEMBL   UCSC 
 
  167
 
  5
 
 20269
 
 tss;island
 
 0.05166
 
 0.08391
 
 Not queried
 
  0.6630143
 
       NA
 
       NA
  
 
  
 1
 
 145476687
 
 145477023
 
  ENSEMBL   UCSC 
 
  337
 
  5
 
 1881
 
 tss
 
 0.01662
 
 0.04661
 
 Not queried
 
  0.6629931
 
       NA
 
       NA
  
 
  
 3
 
 124303268
 
 124303745
 
  ENSEMBL   UCSC 
 
  478
 
  5
 
 27881
 
 gene;island
 
 0.15586
 
 0.06286
 
 Not queried
 
  0.6629833
 
       NA
 
       NA
  
 
  
 22
 
  50963825
 
  50964432
 
  ENSEMBL   UCSC 
 
  608
 
 10
 
 26889
 
 gene;island
 
 0.01194
 
 0.02074
 
 Not queried
 
  0.6629757
 
       NA
 
       NA
  
 
  
 17
 
  78856675
 
  78857238
 
  ENSEMBL   UCSC 
 
  564
 
  5
 
 16845
 
 gene;island
 
 0.89256
 
 0.93563
 
 Not queried
 
  0.6629468
 
       NA
 
       NA
  
 
  
 2
 
 241807708
 
 241808113
 
  ENSEMBL   UCSC 
 
  406
 
  6
 
 23944
 
 tss
 
 0.56737
 
 0.71053
 
 Not queried
 
  0.6629323
 
       NA
 
       NA
  
 
  
 14
 
  38064456
 
  38064532
 
  ENSEMBL   UCSC 
 
   77
 
  5
 
 12768
 
 island
 
 0.01607
 
 0.02521
 
 Not queried
 
  0.6629224
 
       NA
 
       NA
  
 
  
 14
 
  36295485
 
  36295590
 
  ENSEMBL   UCSC 
 
  106
 
  4
 
 12328
 
 tss
 
 0.02835
 
 0.04137
 
 Not queried
 
  0.6628364
 
       NA
 
       NA
  
 
  
 1
 
 201924337
 
 201924583
 
  ENSEMBL   UCSC 
 
  247
 
  7
 
 2167
 
 tss;island
 
 0.02693
 
 0.01718
 
 Not queried
 
  0.6628138
 
       NA
 
       NA
  
 
  
 10
 
 102973432
 
 102973678
 
  ENSEMBL   UCSC 
 
  247
 
  5
 
 6037
 
 island
 
 0.01745
 
 0.03476
 
 Not queried
 
  0.6628101
 
       NA
 
       NA
  
 
  
 19
 
  50145283
 
  50145354
 
  ENSEMBL   UCSC 
 
   72
 
  4
 
 20920
 
 tss;island
 
 0.01811
 
 0.02144
 
 Not queried
 
  0.6627895
 
       NA
 
       NA
  
 
  
 X
 
 153664862
 
 153665103
 
  ENSEMBL   UCSC 
 
  242
 
  4
 
 41600
 
 tss;island
 
 0.04781
 
 0.02903
 
 Not queried
 
  0.6627678
 
       NA
 
       NA
  
 
  
 19
 
  56110871
 
  56111502
 
  ENSEMBL   UCSC 
 
  632
 
  7
 
 21047
 
 tss;island
 
 0.03201
 
 0.02976
 
 Not queried
 
  0.6626291
 
       NA
 
       NA
  
 
  
 5
 
 140683196
 
 140683946
 
  ENSEMBL   UCSC 
 
  751
 
 11
 
 33167
 
 island
 
 0.86611
 
 0.86310
 
 Not queried
 
  0.6625932
 
       NA
 
       NA
  
 
  
 5
 
 178450457
 
 178450763
 
  ENSEMBL   UCSC 
 
  307
 
  5
 
 32487
 
 tss
 
 0.02497
 
 0.03928
 
 Not queried
 
  0.6625833
 
       NA
 
       NA
  
 
  
 11
 
    422107
 
    422260
 
  ENSEMBL   UCSC 
 
  154
 
  4
 
 6355
 
 gene;island
 
 0.83649
 
 0.88135
 
 Not queried
 
  0.6624691
 
       NA
 
       NA
  
 
  
 19
 
  57901022
 
  57901516
 
  ENSEMBL   UCSC 
 
  495
 
  7
 
 22431
 
 island
 
 0.02778
 
 0.05499
 
 Not queried
 
  0.6624184
 
       NA
 
       NA
  
 
  
 1
 
  39456418
 
  39456833
 
  ENSEMBL   UCSC 
 
  416
 
  6
 
 1516
 
 tss;island
 
 0.02306
 
 0.03309
 
 Not queried
 
  0.6623338
 
       NA
 
       NA
  
 
  
 8
 
  23021325
 
  23021690
 
  ENSEMBL   UCSC 
 
  366
 
  6
 
 40082
 
 island
 
 0.07405
 
 0.04338
 
 Not queried
 
  0.6623315
 
       NA
 
       NA
  
 
  
 6
 
 148663353
 
 148663680
 
  ENSEMBL   UCSC 
 
  328
 
 10
 
 35216
 
 tss
 
 0.01311
 
 0.01589
 
 Not queried
 
  0.6623155
 
       NA
 
       NA
  
 
  
 12
 
   4433587
 
   4433983
 
  ENSEMBL   UCSC 
 
  397
 
  5
 
 8990
 
 gene;island
 
 0.91252
 
 0.94228
 
 Not queried
 
  0.6623005
 
       NA
 
       NA
  
 
  
 4
 
   2010926
 
   2011099
 
  ENSEMBL   UCSC 
 
  174
 
  5
 
 30757
 
 island
 
 0.01841
 
 0.02321
 
 Not queried
 
  0.6621765
 
       NA
 
       NA
  
 
  
 19
 
   1103660
 
   1103806
 
  ENSEMBL   UCSC 
 
  147
 
  4
 
 20254
 
 tss;island
 
 0.03220
 
 0.03575
 
 Not queried
 
  0.6621384
 
       NA
 
       NA
  
 
  
 5
 
 131562848
 
 131563099
 
  ENSEMBL   UCSC 
 
  252
 
  4
 
 31688
 
 gene;island
 
 0.06529
 
 0.05525
 
 Not queried
 
  0.6621150
 
       NA
 
       NA
  
 
  
 17
 
   8295971
 
   8296319
 
  ENSEMBL   UCSC 
 
  349
 
  4
 
 16396
 
 gene;island
 
 0.84431
 
 0.75156
 
 Not queried
 
  0.6621135
 
       NA
 
       NA
  
 
  
 14
 
  77278949
 
  77279508
 
  ENSEMBL   UCSC 
 
  560
 
  7
 
 12997
 
 island
 
 0.03266
 
 0.06039
 
 Not queried
 
  0.6620699
 
       NA
 
       NA
  
 
  
 2
 
 201981814
 
 201982020
 
  ENSEMBL   UCSC 
 
  207
 
  4
 
 22949
 
 gene;island
 
 0.00823
 
 0.00763
 
 Not queried
 
  0.6620335
 
       NA
 
       NA
  
 
  
 2
 
 128381653
 
 128381877
 
  ENSEMBL   UCSC 
 
  225
 
  5
 
 22824
 
 gene;island
 
 0.86023
 
 0.79180
 
 Not queried
 
  0.6619789
 
       NA
 
       NA
  
 
  
 14
 
  88851247
 
  88851995
 
  ENSEMBL   UCSC 
 
  749
 
 10
 
 12515
 
 tss;island
 
 0.00893
 
 0.02437
 
 Not queried
 
  0.6619543
 
       NA
 
       NA
  
 
  
 1
 
 192778168
 
 192778396
 
  ENSEMBL   UCSC 
 
  229
 
  4
 
 924
 
 gene
 
 0.00952
 
 0.01648
 
 Not queried
 
  0.6618398
 
       NA
 
       NA
  
 
  
 10
 
 106014296
 
 106014624
 
  ENSEMBL   UCSC 
 
  329
 
  9
 
 5329
 
 tss;island
 
 0.04033
 
 0.05057
 
 Not queried
 
  0.6616737
 
       NA
 
       NA
  
 
  
 14
 
  36295485
 
  36296022
 
  ENSEMBL   UCSC 
 
  538
 
  9
 
 12755
 
 island
 
 0.01668
 
 0.02641
 
 Not queried
 
  0.6616329
 
       NA
 
       NA
  
 
  
 1
 
  24742249
 
  24742672
 
  ENSEMBL   UCSC 
 
  424
 
  4
 
 326
 
 gene
 
 0.02433
 
 0.03095
 
 Not queried
 
  0.6616191
 
       NA
 
       NA
  
 
  
 1
 
  24741723
 
  24742672
 
  ENSEMBL   UCSC 
 
  950
 
 12
 
 2853
 
 island
 
 0.03278
 
 0.02916
 
 Not queried
 
  0.6616191
 
       NA
 
       NA
  
 
  
 17
 
  72209469
 
  72209802
 
  ENSEMBL   UCSC 
 
  334
 
  7
 
 17587
 
 tss;island
 
 0.01634
 
 0.02625
 
 Not queried
 
  0.6615942
 
       NA
 
       NA
  
 
  
 1
 
   7844775
 
   7845237
 
  ENSEMBL   UCSC 
 
  463
 
  4
 
 188
 
 gene
 
 0.82499
 
 0.86524
 
 Not queried
 
  0.6613394
 
       NA
 
       NA
  
 
  
 1
 
  40203334
 
  40203715
 
  ENSEMBL   UCSC 
 
  382
 
  4
 
 1524
 
 tss;island
 
 0.81419
 
 0.84972
 
 Not queried
 
  0.6612957
 
       NA
 
       NA
  
 
  
 15
 
  83478255
 
  83478541
 
  ENSEMBL   UCSC 
 
  287
 
  5
 
 13426
 
 gene;island
 
 0.02017
 
 0.03551
 
 Not queried
 
  0.6611141
 
       NA
 
       NA
  
 
  
 10
 
  28821190
 
  28821381
 
  ENSEMBL   UCSC 
 
  192
 
  6
 
 4990
 
 tss;island
 
 0.01961
 
 0.03414
 
 Not queried
 
  0.6609395
 
       NA
 
       NA
  
 
  
 10
 
 106113175
 
 106113447
 
  ENSEMBL   UCSC 
 
  273
 
  5
 
 5332
 
 tss
 
 0.02935
 
 0.04088
 
 Not queried
 
  0.6608614
 
       NA
 
       NA
  
 
  
 2
 
 169746958
 
 169747227
 
  ENSEMBL   UCSC 
 
  270
 
  8
 
 23662
 
 tss
 
 0.01909
 
 0.01898
 
 Not queried
 
  0.6607924
 
       NA
 
       NA
  
 
  
 1
 
  36788387
 
  36788627
 
  ENSEMBL   UCSC 
 
  241
 
  4
 
 426
 
 gene;island
 
 0.42523
 
 0.53905
 
 Not queried
 
  0.6607315
 
       NA
 
       NA
  
 
  
 11
 
  61447487
 
  61447866
 
  ENSEMBL   UCSC 
 
  380
 
  5
 
 7315
 
 tss
 
 0.01345
 
 0.02110
 
 Not queried
 
  0.6605907
 
       NA
 
       NA
  
 
  
 3
 
  14444035
 
  14444175
 
  ENSEMBL   UCSC 
 
  141
 
  6
 
 28869
 
 island
 
 0.04162
 
 0.07185
 
 Not queried
 
  0.6605899
 
       NA
 
       NA
  
 
  
 1
 
 109505929
 
 109506288
 
  ENSEMBL   UCSC 
 
  360
 
  6
 
 3540
 
 island
 
 0.02264
 
 0.03136
 
 Not queried
 
  0.6605134
 
       NA
 
       NA
  
 
  
 12
 
  66524311
 
  66524943
 
  ENSEMBL   UCSC 
 
  633
 
 10
 
 10575
 
 island
 
 0.01201
 
 0.02248
 
 Not queried
 
  0.6603444
 
       NA
 
       NA
  
 
  
 4
 
  74734714
 
  74735101
 
  ENSEMBL   UCSC 
 
  388
 
  9
 
 30383
 
 tss
 
 0.04431
 
 0.03285
 
 Not queried
 
  0.6601837
 
       NA
 
       NA
  
 
  
 5
 
  31531945
 
  31532200
 
  ENSEMBL   UCSC 
 
  256
 
  7
 
 31519
 
 gene
 
 0.01633
 
 0.03309
 
 Not queried
 
  0.6600548
 
       NA
 
       NA
  
 
  
 5
 
  31531945
 
  31532653
 
  ENSEMBL   UCSC 
 
  709
 
 15
 
 31945
 
 tss;island
 
 0.01633
 
 0.03213
 
 Not queried
 
  0.6600548
 
       NA
 
       NA
  
 
  
 20
 
   3218145
 
   3218579
 
  ENSEMBL   UCSC 
 
  435
 
  9
 
 25928
 
 island
 
 0.51177
 
 0.46704
 
 Not queried
 
  0.6600287
 
       NA
 
       NA
  
 
  
 4
 
  15376072
 
  15376215
 
  ENSEMBL   UCSC 
 
  144
 
  8
 
 29913
 
 gene
 
 0.94826
 
 0.96046
 
 Not queried
 
  0.6599943
 
       NA
 
       NA
  
 
  
 1
 
 200378963
 
 200379364
 
  ENSEMBL   UCSC 
 
  402
 
  6
 
 4052
 
 island
 
 0.03230
 
 0.05010
 
 Not queried
 
  0.6599588
 
       NA
 
       NA
  
 
  
 11
 
  15671597
 
  15671931
 
  ENSEMBL   UCSC 
 
  335
 
  5
 
 8063
 
 island
 
 0.03389
 
 0.02903
 
 Not queried
 
  0.6599331
 
       NA
 
       NA
  
 
  
 14
 
  50359443
 
  50359978
 
  ENSEMBL   UCSC 
 
  536
 
  9
 
 12800
 
 island
 
 0.01255
 
 0.02391
 
 Not queried
 
  0.6599283
 
       NA
 
       NA
  
 
  
 19
 
  41768003
 
  41768357
 
  ENSEMBL   UCSC 
 
  355
 
  5
 
 20767
 
 tss
 
 0.03733
 
 0.06236
 
 Not queried
 
  0.6597489
 
       NA
 
       NA
  
 
  
 19
 
   6502101
 
   6502557
 
  ENSEMBL   UCSC 
 
  457
 
  7
 
 21397
 
 island
 
 0.02425
 
 0.04960
 
 Not queried
 
  0.6597153
 
       NA
 
       NA
  
 
  
 12
 
  75904963
 
  75905681
 
  ENSEMBL   UCSC 
 
  719
 
 14
 
 10610
 
 island
 
 0.01957
 
 0.02605
 
 Not queried
 
  0.6595535
 
       NA
 
       NA
  
 
  
 20
 
  42142224
 
  42143502
 
  ENSEMBL   UCSC 
 
 1279
 
 28
 
 26134
 
 island
 
 0.03344
 
 0.04409
 
 Not queried
 
  0.6595141
 
       NA
 
       NA
  
 
  
 4
 
   4858340
 
   4858814
 
  ENSEMBL   UCSC 
 
  475
 
  5
 
 30816
 
 island
 
 0.02543
 
 0.02627
 
 Not queried
 
  0.6595030
 
       NA
 
       NA
  
 
  
 16
 
  51184786
 
  51185461
 
  ENSEMBL   UCSC 
 
  676
 
 15
 
 15200
 
 tss;island
 
 0.02438
 
 0.02963
 
 Not queried
 
  0.6594862
 
       NA
 
       NA
  
 
  
 X
 
  71497035
 
  71497424
 
  ENSEMBL   UCSC 
 
  390
 
  6
 
 41867
 
 island
 
 0.01595
 
 0.02512
 
 Not queried
 
  0.6594355
 
       NA
 
       NA
  
 
  
 6
 
  31937066
 
  31937470
 
  ENSEMBL   UCSC 
 
  405
 
 12
 
 33942
 
 gene
 
 0.88149
 
 0.92147
 
 Not queried
 
  0.6593659
 
       NA
 
       NA
  
 
  
 19
 
  19249194
 
  19249481
 
  ENSEMBL   UCSC 
 
  288
 
  7
 
 21769
 
 island
 
 0.01050
 
 0.03199
 
 Not queried
 
  0.6593600
 
       NA
 
       NA
  
 
  
 16
 
   1146277
 
   1146545
 
  ENSEMBL   UCSC 
 
  269
 
  4
 
 14957
 
 tss
 
 0.82790
 
 0.90345
 
 Not queried
 
  0.6593536
 
       NA
 
       NA
  
 
  
 3
 
  16555288
 
  16555620
 
  ENSEMBL   UCSC 
 
  333
 
  9
 
 28135
 
 tss;island
 
 0.02478
 
 0.05939
 
 Not queried
 
  0.6593472
 
       NA
 
       NA
  
 
  
 5
 
  43040505
 
  43040621
 
  ENSEMBL   UCSC 
 
  117
 
  6
 
 31983
 
 tss
 
 0.01591
 
 0.01626
 
 Not queried
 
  0.6592576
 
       NA
 
       NA
  
 
  
 15
 
  80215929
 
  80216491
 
  ENSEMBL   UCSC 
 
  563
 
  8
 
 13420
 
 gene;island
 
 0.01455
 
 0.02634
 
 Not queried
 
  0.6592208
 
       NA
 
       NA
  
 
  
 7
 
  63505584
 
  63505871
 
  ENSEMBL   UCSC 
 
  288
 
  7
 
 38458
 
 island
 
 0.22276
 
 0.34560
 
 Not queried
 
  0.6591030
 
       NA
 
       NA
  
 
  
 6
 
 150463775
 
 150464265
 
  ENSEMBL   UCSC 
 
  491
 
  8
 
 36506
 
 island
 
 0.04221
 
 0.04862
 
 Not queried
 
  0.6590131
 
       NA
 
       NA
  
 
  
 1
 
  36851558
 
  36851860
 
  ENSEMBL   UCSC 
 
  303
 
  7
 
 1492
 
 tss;island
 
 0.03138
 
 0.05463
 
 Not queried
 
  0.6589739
 
       NA
 
       NA
  
 
  
 17
 
  79818544
 
  79818808
 
  ENSEMBL   UCSC 
 
  265
 
  5
 
 17717
 
 tss
 
 0.02927
 
 0.02258
 
 Not queried
 
  0.6588967
 
       NA
 
       NA
  
 
  
 6
 
 137366322
 
 137366545
 
  ENSEMBL   UCSC 
 
  224
 
  7
 
 35183
 
 tss
 
 0.92011
 
 0.88008
 
 Not queried
 
  0.6588685
 
       NA
 
       NA
  
 
  
 13
 
 114067889
 
 114068181
 
  ENSEMBL   UCSC 
 
  293
 
  4
 
 11935
 
 island
 
 0.72349
 
 0.74632
 
 Not queried
 
  0.6588677
 
       NA
 
       NA
  
 
  
 13
 
 110961069
 
 110961606
 
  ENSEMBL   UCSC 
 
  538
 
  8
 
 11199
 
 gene;island
 
 0.06361
 
 0.14716
 
 Not queried
 
  0.6587309
 
       NA
 
       NA
  
 
  
 12
 
  10364834
 
  10365082
 
  ENSEMBL   UCSC 
 
  249
 
  4
 
 9535
 
 tss;island
 
 0.03238
 
 0.05062
 
 Not queried
 
  0.6587306
 
       NA
 
       NA
  
 
  
 4
 
 156297854
 
 156298427
 
  ENSEMBL   UCSC 
 
  574
 
 12
 
 31336
 
 island
 
 0.03278
 
 0.04443
 
 Not queried
 
  0.6587024
 
       NA
 
       NA
  
 
  
 2
 
   1895643
 
   1895961
 
  ENSEMBL   UCSC 
 
  319
 
  5
 
 22503
 
 gene;island
 
 0.74604
 
 0.89586
 
 Not queried
 
  0.6586136
 
       NA
 
       NA
  
 
  
 1
 
  21616619
 
  21617100
 
  ENSEMBL   UCSC 
 
  482
 
  5
 
 294
 
 gene;island
 
 0.01596
 
 0.02921
 
 Not queried
 
  0.6583502
 
       NA
 
       NA
  
 
  
 17
 
  18761289
 
  18761479
 
  ENSEMBL   UCSC 
 
  191
 
  7
 
 17149
 
 tss;island
 
 0.01081
 
 0.01994
 
 Not queried
 
  0.6581065
 
       NA
 
       NA
  
 
  
 3
 
 128206703
 
 128206967
 
  ENSEMBL   UCSC 
 
  265
 
  6
 
 27895
 
 gene;island
 
 0.02295
 
 0.03567
 
 Not queried
 
  0.6579335
 
       NA
 
       NA
  
 
  
 11
 
 124747075
 
 124747263
 
  ENSEMBL   UCSC 
 
  189
 
  4
 
 6935
 
 gene;island
 
 0.31917
 
 0.45257
 
 Not queried
 
  0.6579200
 
       NA
 
       NA
  
 
  
 15
 
  32321637
 
  32321944
 
  ENSEMBL   UCSC 
 
  308
 
  4
 
 13522
 
 tss;island
 
 0.03263
 
 0.02936
 
 Not queried
 
  0.6579145
 
       NA
 
       NA
  
 
  
 5
 
 173738161
 
 173738479
 
  ENSEMBL   UCSC 
 
  319
 
  4
 
 33351
 
 island
 
 0.37994
 
 0.61895
 
 Not queried
 
  0.6579114
 
       NA
 
       NA
  
 
  
 8
 
  25042340
 
  25042630
 
  ENSEMBL   UCSC 
 
  291
 
  4
 
 39185
 
 gene;island
 
 0.03220
 
 0.05051
 
 Not queried
 
  0.6579016
 
       NA
 
       NA
  
 
  
 21
 
  44394280
 
  44394503
 
  ENSEMBL   UCSC 
 
  224
 
  4
 
 26531
 
 tss;island
 
 0.01555
 
 0.03614
 
 Not queried
 
  0.6578571
 
       NA
 
       NA
  
 
  
 7
 
  79764176
 
  79764387
 
  ENSEMBL   UCSC 
 
  212
 
  4
 
 37004
 
 gene;island
 
 0.02381
 
 0.04603
 
 Not queried
 
  0.6578204
 
       NA
 
       NA
  
 
  
 11
 
  64901879
 
  64902218
 
  ENSEMBL   UCSC 
 
  340
 
  8
 
 8418
 
 island
 
 0.01806
 
 0.02178
 
 Not queried
 
  0.6577799
 
       NA
 
       NA
  
 
  
 5
 
 145562370
 
 145562699
 
  ENSEMBL   UCSC 
 
  330
 
  8
 
 32346
 
 tss
 
 0.01402
 
 0.02531
 
 Not queried
 
  0.6577085
 
       NA
 
       NA
  
 
  
 12
 
 122018375
 
 122019117
 
  ENSEMBL   UCSC 
 
  743
 
 14
 
 10048
 
 tss
 
 0.03232
 
 0.01982
 
 Not queried
 
  0.6576382
 
       NA
 
       NA
  
 
  
 1
 
  76251458
 
  76252133
 
  ENSEMBL   UCSC 
 
  676
 
 11
 
 1697
 
 tss
 
 0.01094
 
 0.01451
 
 Not queried
 
  0.6576122
 
       NA
 
       NA
  
 
  
 12
 
   6798753
 
   6799136
 
  ENSEMBL   UCSC 
 
  384
 
  9
 
 9497
 
 tss;island
 
 0.02069
 
 0.02909
 
 Not queried
 
  0.6575015
 
       NA
 
       NA
  
 
  
 2
 
 132919534
 
 132919865
 
  ENSEMBL   UCSC 
 
  332
 
  5
 
 23598
 
 tss
 
 0.48501
 
 0.63459
 
 Not queried
 
  0.6574970
 
       NA
 
       NA
  
 
  
 12
 
  56862350
 
  56862729
 
  ENSEMBL   UCSC 
 
  380
 
  5
 
 9155
 
 gene
 
 0.01601
 
 0.02367
 
 Not queried
 
  0.6573255
 
       NA
 
       NA
  
 
  
 5
 
 156886816
 
 156887129
 
  ENSEMBL   UCSC 
 
  314
 
  7
 
 33279
 
 island
 
 0.08574
 
 0.05965
 
 Not queried
 
  0.6573178
 
       NA
 
       NA
  
 
  
 3
 
  50374670
 
  50375735
 
  ENSEMBL   UCSC 
 
 1066
 
 25
 
 27763
 
 gene;island
 
 0.04791
 
 0.03092
 
 Not queried
 
  0.6572898
 
       NA
 
       NA
  
 
  
 8
 
   1792023
 
   1792249
 
  ENSEMBL   UCSC 
 
  227
 
  4
 
 39115
 
 gene
 
 0.93295
 
 0.92265
 
 Not queried
 
  0.6572884
 
       NA
 
       NA
  
 
  
 14
 
  89020645
 
  89021477
 
  ENSEMBL   UCSC 
 
  833
 
 11
 
 13030
 
 island
 
 0.02969
 
 0.04687
 
 Not queried
 
  0.6570489
 
       NA
 
       NA
  
 
  
 10
 
   6130787
 
   6130867
 
  ENSEMBL   UCSC 
 
   81
 
  5
 
 4915
 
 tss;island
 
 0.02451
 
 0.03254
 
 Not queried
 
  0.6570440
 
       NA
 
       NA
  
 
  
 12
 
 125223502
 
 125223679
 
  ENSEMBL   UCSC 
 
  178
 
  4
 
 10947
 
 island
 
 0.80222
 
 0.82962
 
 Not queried
 
  0.6569549
 
       NA
 
       NA
  
 
  
 1
 
  44115487
 
  44115742
 
  ENSEMBL   UCSC 
 
  256
 
  6
 
 1561
 
 tss
 
 0.02324
 
 0.03391
 
 Not queried
 
  0.6568676
 
       NA
 
       NA
  
 
  
 8
 
  17270347
 
  17270608
 
  ENSEMBL   UCSC 
 
  262
 
  4
 
 39153
 
 gene;island
 
 0.03471
 
 0.03136
 
 Not queried
 
  0.6568412
 
       NA
 
       NA
  
 
  
 X
 
  44732013
 
  44732417
 
  ENSEMBL   UCSC 
 
  405
 
  8
 
 41268
 
 tss
 
 0.01941
 
 0.02817
 
 Not queried
 
  0.6568096
 
       NA
 
       NA
  
 
  
 6
 
  44695195
 
  44695536
 
  ENSEMBL   UCSC 
 
  342
 
  5
 
 36118
 
 island
 
 0.76197
 
 0.81142
 
 Not queried
 
  0.6566534
 
       NA
 
       NA
  
 
  
 8
 
  95566223
 
  95566670
 
  ENSEMBL   UCSC 
 
  448
 
  4
 
 39753
 
 tss;island
 
 0.01404
 
 0.02847
 
 Not queried
 
  0.6566522
 
       NA
 
       NA
  
 
  
 7
 
  27170241
 
  27170394
 
  ENSEMBL   UCSC 
 
  154
 
  4
 
 36870
 
 gene
 
 0.01704
 
 0.02687
 
 Not queried
 
  0.6566224
 
       NA
 
       NA
  
 
  
 12
 
  12878752
 
  12878887
 
  ENSEMBL   UCSC 
 
  136
 
  6
 
 10228
 
 island
 
 0.01071
 
 0.01685
 
 Not queried
 
  0.6565171
 
       NA
 
       NA
  
 
  
 7
 
  63505584
 
  63505811
 
  ENSEMBL   UCSC 
 
  228
 
  6
 
 37548
 
 tss
 
 0.22353
 
 0.34413
 
 Not queried
 
  0.6564911
 
       NA
 
       NA
  
 
  
 1
 
   1177703
 
   1178245
 
  ENSEMBL   UCSC 
 
  543
 
  6
 
 2413
 
 island
 
 0.80996
 
 0.86054
 
 Not queried
 
  0.6564044
 
       NA
 
       NA
  
 
  
 10
 
 131263962
 
 131264846
 
  ENSEMBL   UCSC 
 
  885
 
 12
 
 5421
 
 tss;island
 
 0.67157
 
 0.84085
 
 Not queried
 
  0.6563031
 
       NA
 
       NA
  
 
  
 10
 
  75545269
 
  75545507
 
  ENSEMBL   UCSC 
 
  239
 
  6
 
 5148
 
 tss
 
 0.01546
 
 0.02411
 
 Not queried
 
  0.6562406
 
       NA
 
       NA
  
 
  
 17
 
  27493467
 
  27493798
 
  ENSEMBL   UCSC 
 
  332
 
  4
 
 16469
 
 gene;island
 
 0.87263
 
 0.83982
 
 Not queried
 
  0.6562342
 
       NA
 
       NA
  
 
  
 12
 
  38710408
 
  38710504
 
  ENSEMBL   UCSC 
 
   97
 
  6
 
 9610
 
 tss
 
 0.00949
 
 0.01285
 
 Not queried
 
  0.6561187
 
       NA
 
       NA
  
 
  
 3
 
  50242325
 
  50242754
 
  ENSEMBL   UCSC 
 
  430
 
  5
 
 29095
 
 island
 
 0.06077
 
 0.03211
 
 Not queried
 
  0.6560099
 
       NA
 
       NA
  
 
  
 3
 
 133524362
 
 133525182
 
  ENSEMBL   UCSC 
 
  821
 
  9
 
 29449
 
 island
 
 0.01752
 
 0.02503
 
 Not queried
 
  0.6560036
 
       NA
 
       NA
  
 
  
 4
 
 123300224
 
 123300788
 
  ENSEMBL   UCSC 
 
  565
 
 12
 
 30072
 
 gene;island
 
 0.89897
 
 0.87787
 
 Not queried
 
  0.6559775
 
       NA
 
       NA
  
 
  
 6
 
  84562778
 
  84563098
 
  ENSEMBL   UCSC 
 
  321
 
  9
 
 36230
 
 island
 
 0.02074
 
 0.03035
 
 Not queried
 
  0.6559605
 
       NA
 
       NA
  
 
  
 7
 
 128049508
 
 128049980
 
  ENSEMBL   UCSC 
 
  473
 
  7
 
 37150
 
 gene
 
 0.02006
 
 0.01398
 
 Not queried
 
  0.6559356
 
       NA
 
       NA
  
 
  
 19
 
  21203359
 
  21203493
 
  ENSEMBL   UCSC 
 
  135
 
  6
 
 20622
 
 tss
 
 0.12970
 
 0.06892
 
 Not queried
 
  0.6558774
 
       NA
 
       NA
  
 
  
 2
 
  17699740
 
  17699980
 
  ENSEMBL   UCSC 
 
  241
 
  7
 
 23161
 
 tss
 
 0.01800
 
 0.03334
 
 Not queried
 
  0.6558260
 
       NA
 
       NA
  
 
  
 19
 
    917326
 
    917813
 
  ENSEMBL   UCSC 
 
  488
 
  5
 
 21148
 
 island
 
 0.10634
 
 0.04762
 
 Not queried
 
  0.6557980
 
       NA
 
       NA
  
 
  
 15
 
  65341703
 
  65341995
 
  ENSEMBL   UCSC 
 
  293
 
  5
 
 13349
 
 gene
 
 0.01310
 
 0.02094
 
 Not queried
 
  0.6557051
 
       NA
 
       NA
  
 
  
 3
 
 118753705
 
 118753871
 
  ENSEMBL   UCSC 
 
  167
 
  5
 
 28472
 
 tss
 
 0.01984
 
 0.02821
 
 Not queried
 
  0.6556703
 
       NA
 
       NA
  
 
  
 10
 
  43866966
 
  43867071
 
  ENSEMBL   UCSC 
 
  106
 
  5
 
 5029
 
 tss
 
 0.76016
 
 0.60016
 
 Not queried
 
  0.6556380
 
       NA
 
       NA
  
 
  
 1
 
    981164
 
    981566
 
  ENSEMBL   UCSC 
 
  403
 
  4
 
 8
 
 gene;island
 
 0.93160
 
 0.89813
 
 Not queried
 
  0.6556161
 
       NA
 
       NA
  
 
  
 17
 
  37729644
 
  37730450
 
  ENSEMBL   UCSC 
 
  807
 
  7
 
 18243
 
 island
 
 0.02967
 
 0.01650
 
 Not queried
 
  0.6555581
 
       NA
 
       NA
  
 
  
 1
 
  24018356
 
  24018520
 
  ENSEMBL   UCSC 
 
  165
 
  4
 
 317
 
 gene;island
 
 0.02602
 
 0.04871
 
 Not queried
 
  0.6554938
 
       NA
 
       NA
  
 
  
 6
 
  31687914
 
  31688428
 
  ENSEMBL   UCSC 
 
  515
 
  6
 
 33878
 
 gene;island
 
 0.70043
 
 0.59444
 
 Not queried
 
  0.6554852
 
       NA
 
       NA
  
 
  
 19
 
  58459097
 
  58459358
 
  ENSEMBL   UCSC 
 
  262
 
  8
 
 21093
 
 tss
 
 0.00932
 
 0.01819
 
 Not queried
 
  0.6554693
 
       NA
 
       NA
  
 
  
 20
 
   3149249
 
   3149495
 
  ENSEMBL   UCSC 
 
  247
 
  6
 
 25521
 
 tss;island
 
 0.37254
 
 0.31601
 
 Not queried
 
  0.6554441
 
       NA
 
       NA
  
 
  
 6
 
 160220921
 
 160221433
 
  ENSEMBL   UCSC 
 
  513
 
 10
 
 36547
 
 island
 
 0.95047
 
 0.91931
 
 Not queried
 
  0.6554410
 
       NA
 
       NA
  
 
  
 1
 
 155265026
 
 155265378
 
  ENSEMBL   UCSC 
 
  353
 
  4
 
 798
 
 gene;island
 
 0.36721
 
 0.47639
 
 Not queried
 
  0.6554277
 
       NA
 
       NA
  
 
  
 11
 
  66278004
 
  66278117
 
  ENSEMBL   UCSC 
 
  114
 
  6
 
 7450
 
 tss
 
 0.02501
 
 0.03132
 
 Not queried
 
  0.6553852
 
       NA
 
       NA
  
 
  
 20
 
  62103400
 
  62103627
 
  ENSEMBL   UCSC 
 
  228
 
  4
 
 25462
 
 gene;island
 
 0.02733
 
 0.02326
 
 Not queried
 
  0.6553254
 
       NA
 
       NA
  
 
  
 19
 
  43918868
 
  43919028
 
  ENSEMBL   UCSC 
 
  161
 
  6
 
 20792
 
 tss
 
 0.86985
 
 0.79086
 
 Not queried
 
  0.6552679
 
       NA
 
       NA
  
 
  
 3
 
 128294970
 
 128295141
 
  ENSEMBL   UCSC 
 
  172
 
  6
 
 28527
 
 tss
 
 0.68464
 
 0.74853
 
 Not queried
 
  0.6552462
 
       NA
 
       NA
  
 
  
 1
 
  92494898
 
  92495083
 
  ENSEMBL   UCSC 
 
  186
 
  4
 
 1743
 
 tss;island
 
 0.32922
 
 0.45872
 
 Not queried
 
  0.6550247
 
       NA
 
       NA
  
 
  
 6
 
 161575229
 
 161575667
 
  ENSEMBL   UCSC 
 
  439
 
  7
 
 34472
 
 gene
 
 0.92928
 
 0.86293
 
 Not queried
 
  0.6549276
 
       NA
 
       NA
  
 
  
 6
 
  31540399
 
  31540461
 
  ENSEMBL   UCSC 
 
   63
 
  6
 
 33838
 
 gene
 
 0.90097
 
 0.82431
 
 Not queried
 
  0.6548996
 
       NA
 
       NA
  
 
  
 17
 
  73452251
 
  73452602
 
  ENSEMBL   UCSC 
 
  352
 
  6
 
 17619
 
 tss
 
 0.01793
 
 0.03344
 
 Not queried
 
  0.6548582
 
       NA
 
       NA
  
 
  
 16
 
    699218
 
    699500
 
  ENSEMBL   UCSC 
 
  283
 
  6
 
 15438
 
 island
 
 0.83115
 
 0.92523
 
 Not queried
 
  0.6547770
 
       NA
 
       NA
  
 
  
 5
 
 138940474
 
 138940718
 
  ENSEMBL   UCSC 
 
  245
 
  7
 
 32275
 
 tss;island
 
 0.01266
 
 0.03726
 
 Not queried
 
  0.6546973
 
       NA
 
       NA
  
 
  
 12
 
  56842950
 
  56843596
 
  ENSEMBL   UCSC 
 
  647
 
 10
 
 10510
 
 island
 
 0.07303
 
 0.02788
 
 Not queried
 
  0.6546006
 
       NA
 
       NA
  
 
  
 7
 
  47294004
 
  47294330
 
  ENSEMBL   UCSC 
 
  327
 
  5
 
 38416
 
 island
 
 0.03351
 
 0.04251
 
 Not queried
 
  0.6545919
 
       NA
 
       NA
  
 
  
 10
 
  51784543
 
  51784888
 
  ENSEMBL   UCSC 
 
  346
 
  4
 
 5723
 
 island
 
 0.52952
 
 0.65758
 
 Not queried
 
  0.6544926
 
       NA
 
       NA
  
 
  
 7
 
 140098427
 
 140098579
 
  ENSEMBL   UCSC 
 
  153
 
  4
 
 37872
 
 tss
 
 0.10587
 
 0.12289
 
 Not queried
 
  0.6544398
 
       NA
 
       NA
  
 
  
 7
 
  94537584
 
  94537893
 
  ENSEMBL   UCSC 
 
  310
 
  8
 
 37031
 
 gene;island
 
 0.03248
 
 0.03483
 
 Not queried
 
  0.6543705
 
       NA
 
       NA
  
 
  
 4
 
   2251938
 
   2252266
 
  ENSEMBL   UCSC 
 
  329
 
  4
 
 29838
 
 gene
 
 0.78675
 
 0.86833
 
 Not queried
 
  0.6543701
 
       NA
 
       NA
  
 
  
 15
 
  63796130
 
  63796746
 
  ENSEMBL   UCSC 
 
  617
 
 10
 
 13695
 
 tss
 
 0.01855
 
 0.02726
 
 Not queried
 
  0.6542767
 
       NA
 
       NA
  
 
  
 19
 
  10828819
 
  10829423
 
  ENSEMBL   UCSC 
 
  605
 
 16
 
 19787
 
 gene
 
 0.02267
 
 0.03790
 
 Not queried
 
  0.6542631
 
       NA
 
       NA
  
 
  
 19
 
  10828487
 
  10829423
 
  ENSEMBL   UCSC 
 
  937
 
 20
 
 21510
 
 island
 
 0.01888
 
 0.02897
 
 Not queried
 
  0.6542631
 
       NA
 
       NA
  
 
  
 7
 
   2049947
 
   2050170
 
  ENSEMBL   UCSC 
 
  224
 
  4
 
 36722
 
 gene
 
 0.91994
 
 0.95905
 
 Not queried
 
  0.6542612
 
       NA
 
       NA
  
 
  
 7
 
  43151725
 
  43152125
 
  ENSEMBL   UCSC 
 
  401
 
  6
 
 37490
 
 tss
 
 0.41924
 
 0.36039
 
 Not queried
 
  0.6541770
 
       NA
 
       NA
  
 
  
 13
 
  26625089
 
  26625396
 
  ENSEMBL   UCSC 
 
  308
 
  7
 
 11587
 
 island
 
 0.05801
 
 0.08058
 
 Not queried
 
  0.6541625
 
       NA
 
       NA
  
 
  
 6
 
 168393930
 
 168394198
 
  ENSEMBL   UCSC 
 
  269
 
  5
 
 36591
 
 island
 
 0.94014
 
 0.96246
 
 Not queried
 
  0.6540731
 
       NA
 
       NA
  
 
  
 8
 
 142350506
 
 142350581
 
  ENSEMBL   UCSC 
 
   76
 
  4
 
 39865
 
 tss
 
 0.89095
 
 0.93383
 
 Not queried
 
  0.6540426
 
       NA
 
       NA
  
 
  
 7
 
 119912636
 
 119912900
 
  ENSEMBL   UCSC 
 
  265
 
  5
 
 37794
 
 tss;island
 
 0.06531
 
 0.04268
 
 Not queried
 
  0.6539910
 
       NA
 
       NA
  
 
  
 4
 
  85504029
 
  85504053
 
  ENSEMBL   UCSC 
 
   25
 
  5
 
 30433
 
 tss;island
 
 0.03445
 
 0.05315
 
 Not queried
 
  0.6539832
 
       NA
 
       NA
  
 
  
 17
 
  40428759
 
  40428970
 
  ENSEMBL   UCSC 
 
  212
 
  4
 
 17327
 
 tss;island
 
 0.11004
 
 0.04985
 
 Not queried
 
  0.6539811
 
       NA
 
       NA
  
 
  
 7
 
 121955208
 
 121955894
 
  ENSEMBL   UCSC 
 
  687
 
  6
 
 38809
 
 island
 
 0.17357
 
 0.05433
 
 Not queried
 
  0.6539707
 
       NA
 
       NA
  
 
  
 2
 
 129659633
 
 129659946
 
  ENSEMBL   UCSC 
 
  314
 
  4
 
 24673
 
 island
 
 0.75322
 
 0.66422
 
 Not queried
 
  0.6539575
 
       NA
 
       NA
  
 
  
 8
 
  61086176
 
  61086268
 
  ENSEMBL   UCSC 
 
   93
 
  4
 
 40263
 
 island
 
 0.59444
 
 0.71548
 
 Not queried
 
  0.6538724
 
       NA
 
       NA
  
 
  
 X
 
  69282642
 
  69283055
 
  ENSEMBL   UCSC 
 
  414
 
  4
 
 41013
 
 gene;island
 
 0.87393
 
 0.88353
 
 Not queried
 
  0.6538611
 
       NA
 
       NA
  
 
  
 12
 
 121647128
 
 121647217
 
  ENSEMBL   UCSC 
 
   90
 
  5
 
 10044
 
 tss;island
 
 0.74801
 
 0.84600
 
 Not queried
 
  0.6536723
 
       NA
 
       NA
  
 
  
 5
 
  41925120
 
  41925229
 
  ENSEMBL   UCSC 
 
  110
 
  5
 
 31981
 
 tss
 
 0.03172
 
 0.02093
 
 Not queried
 
  0.6536582
 
       NA
 
       NA
  
 
  
 5
 
  41925120
 
  41925485
 
  ENSEMBL   UCSC 
 
  366
 
  7
 
 32695
 
 island
 
 0.03055
 
 0.02093
 
 Not queried
 
  0.6536582
 
       NA
 
       NA
  
 
  
 11
 
  57267101
 
  57267244
 
  ENSEMBL   UCSC 
 
  144
 
  4
 
 6596
 
 gene;island
 
 0.05857
 
 0.07480
 
 Not queried
 
  0.6536468
 
       NA
 
       NA
  
 
  
 10
 
  72200612
 
  72201008
 
  ENSEMBL   UCSC 
 
  397
 
  4
 
 4605
 
 gene;island
 
 0.53857
 
 0.37746
 
 Not queried
 
  0.6536269
 
       NA
 
       NA
  
 
  
 5
 
 172305889
 
 172306136
 
  ENSEMBL   UCSC 
 
  248
 
  6
 
 31837
 
 gene
 
 0.89803
 
 0.92815
 
 Not queried
 
  0.6534759
 
       NA
 
       NA
  
 
  
 10
 
   7661622
 
   7661796
 
  ENSEMBL   UCSC 
 
  175
 
  4
 
 4921
 
 tss
 
 0.90901
 
 0.93421
 
 Not queried
 
  0.6533517
 
       NA
 
       NA
  
 
  
 11
 
  67159072
 
  67159421
 
  ENSEMBL   UCSC 
 
  350
 
  8
 
 7468
 
 tss
 
 0.00838
 
 0.01432
 
 Not queried
 
  0.6531708
 
       NA
 
       NA
  
 
  
 15
 
  40331526
 
  40331866
 
  ENSEMBL   UCSC 
 
  341
 
  7
 
 13552
 
 tss
 
 0.01483
 
 0.02065
 
 Not queried
 
  0.6531673
 
       NA
 
       NA
  
 
  
 13
 
 114103452
 
 114103796
 
  ENSEMBL   UCSC 
 
  345
 
  7
 
 11258
 
 gene
 
 0.65112
 
 0.53498
 
 Not queried
 
  0.6531522
 
       NA
 
       NA
  
 
  
 14
 
 102026230
 
 102026555
 
  ENSEMBL   UCSC 
 
  326
 
 10
 
 12607
 
 tss
 
 0.12366
 
 0.05860
 
 Not queried
 
  0.6531088
 
       NA
 
       NA
  
 
  
 6
 
  30614632
 
  30614796
 
  ENSEMBL   UCSC 
 
  165
 
  8
 
 34714
 
 tss
 
 0.02140
 
 0.03246
 
 Not queried
 
  0.6530364
 
       NA
 
       NA
  
 
  
 10
 
  23479993
 
  23480316
 
  ENSEMBL   UCSC 
 
  324
 
  6
 
 4970
 
 tss;island
 
 0.13606
 
 0.10173
 
 Not queried
 
  0.6529737
 
       NA
 
       NA
  
 
  
 1
 
   2846836
 
   2847220
 
  ENSEMBL   UCSC 
 
  385
 
  5
 
 2516
 
 island
 
 0.76915
 
 0.86577
 
 Not queried
 
  0.6529733
 
       NA
 
       NA
  
 
  
 17
 
  79849429
 
  79850075
 
  ENSEMBL   UCSC 
 
  647
 
 14
 
 16893
 
 gene;tss;island
 
 0.01314
 
 0.02578
 
 Not queried
 
  0.6529684
 
       NA
 
       NA
  
 
  
 3
 
 196295218
 
 196295373
 
  ENSEMBL   UCSC 
 
  156
 
  4
 
 28057
 
 gene
 
 0.03331
 
 0.04469
 
 Not queried
 
  0.6529635
 
       NA
 
       NA
  
 
  
 1
 
  40348903
 
  40349329
 
  ENSEMBL   UCSC 
 
  427
 
  6
 
 3103
 
 island
 
 0.03141
 
 0.05340
 
 Not queried
 
  0.6529371
 
       NA
 
       NA
  
 
  
 6
 
  89827912
 
  89828172
 
  ENSEMBL   UCSC 
 
  261
 
  4
 
 35053
 
 tss;island
 
 0.16222
 
 0.03252
 
 Not queried
 
  0.6529196
 
       NA
 
       NA
  
 
  
 19
 
   4007529
 
   4007604
 
  ENSEMBL   UCSC 
 
   76
 
  4
 
 20321
 
 tss;island
 
 0.01641
 
 0.01820
 
 Not queried
 
  0.6529079
 
       NA
 
       NA
  
 
  
 13
 
 112668130
 
 112668501
 
  ENSEMBL   UCSC 
 
  372
 
  6
 
 11876
 
 island
 
 0.91546
 
 0.93349
 
 Not queried
 
  0.6528959
 
       NA
 
       NA
  
 
  
 2
 
 110371607
 
 110371903
 
  ENSEMBL   UCSC 
 
  297
 
  9
 
 23531
 
 tss;island
 
 0.03591
 
 0.04588
 
 Not queried
 
  0.6528667
 
       NA
 
       NA
  
 
  
 4
 
   6711592
 
   6711995
 
  ENSEMBL   UCSC 
 
  404
 
  8
 
 30843
 
 island
 
 0.04438
 
 0.02720
 
 Not queried
 
  0.6528472
 
       NA
 
       NA
  
 
  
 3
 
 187463496
 
 187463617
 
  ENSEMBL   UCSC 
 
  122
 
  5
 
 28736
 
 tss
 
 0.01149
 
 0.02293
 
 Not queried
 
  0.6528074
 
       NA
 
       NA
  
 
  
 7
 
    223445
 
    223896
 
  ENSEMBL   UCSC 
 
  452
 
  4
 
 36643
 
 gene;island
 
 0.87232
 
 0.90229
 
 Not queried
 
  0.6527742
 
       NA
 
       NA
  
 
  
 10
 
  79789146
 
  79789430
 
  ENSEMBL   UCSC 
 
  285
 
  6
 
 5856
 
 island
 
 0.03139
 
 0.06321
 
 Not queried
 
  0.6526966
 
       NA
 
       NA
  
 
  
 7
 
  20825216
 
  20825862
 
  ENSEMBL   UCSC 
 
  647
 
  7
 
 36846
 
 gene;island
 
 0.01391
 
 0.02583
 
 Not queried
 
  0.6525843
 
       NA
 
       NA
  
 
  
 13
 
 113474153
 
 113474413
 
  ENSEMBL   UCSC 
 
  261
 
  5
 
 11228
 
 gene;island
 
 0.84518
 
 0.87957
 
 Not queried
 
  0.6525291
 
       NA
 
       NA
  
 
  
 22
 
  38453075
 
  38453489
 
  ENSEMBL   UCSC 
 
  415
 
 10
 
 27437
 
 island
 
 0.02999
 
 0.05114
 
 Not queried
 
  0.6524341
 
       NA
 
       NA
  
 
  
 11
 
  66336125
 
  66336293
 
  ENSEMBL   UCSC 
 
  169
 
  6
 
 7453
 
 tss
 
 0.81174
 
 0.92811
 
 Not queried
 
  0.6524147
 
       NA
 
       NA
  
 
  
 17
 
   8079766
 
   8079962
 
  ENSEMBL   UCSC 
 
  197
 
  7
 
 17078
 
 tss
 
 0.01320
 
 0.02281
 
 Not queried
 
  0.6523482
 
       NA
 
       NA
  
 
  
 2
 
 147344914
 
 147345337
 
  ENSEMBL   UCSC 
 
  424
 
  5
 
 22855
 
 gene;island
 
 0.85947
 
 0.92401
 
 Not queried
 
  0.6523463
 
       NA
 
       NA
  
 
  
 12
 
  74931289
 
  74931760
 
  ENSEMBL   UCSC 
 
  472
 
  9
 
 10603
 
 island
 
 0.01656
 
 0.02302
 
 Not queried
 
  0.6522554
 
       NA
 
       NA
  
 
  
 8
 
  67686767
 
  67687060
 
  ENSEMBL   UCSC 
 
  294
 
  5
 
 39262
 
 gene;tss;island
 
 0.03825
 
 0.02005
 
 Not queried
 
  0.6522345
 
       NA
 
       NA
  
 
  
 18
 
   6284379
 
   6284570
 
  ENSEMBL   UCSC 
 
  192
 
  5
 
 19017
 
 gene;island
 
 0.94896
 
 0.88169
 
 Not queried
 
  0.6520357
 
       NA
 
       NA
  
 
  
 20
 
  60813424
 
  60813456
 
  ENSEMBL   UCSC 
 
   33
 
  4
 
 25828
 
 tss
 
 0.04125
 
 0.05990
 
 Not queried
 
  0.6519680
 
       NA
 
       NA
  
 
  
 20
 
  60813424
 
  60813777
 
  ENSEMBL   UCSC 
 
  354
 
  6
 
 26275
 
 island
 
 0.03017
 
 0.04862
 
 Not queried
 
  0.6519680
 
       NA
 
       NA
  
 
  
 19
 
  39138026
 
  39138322
 
  ENSEMBL   UCSC 
 
  297
 
  7
 
 20722
 
 tss
 
 0.04516
 
 0.03478
 
 Not queried
 
  0.6519230
 
       NA
 
       NA
  
 
  
 2
 
   7017571
 
   7017787
 
  ENSEMBL   UCSC 
 
  217
 
  4
 
 23129
 
 tss
 
 0.59753
 
 0.70008
 
 Not queried
 
  0.6518975
 
       NA
 
       NA
  
 
  
 1
 
  40839445
 
  40839764
 
  ENSEMBL   UCSC 
 
  320
 
  4
 
 3115
 
 island
 
 0.04162
 
 0.05161
 
 Not queried
 
  0.6517779
 
       NA
 
       NA
  
 
  
 3
 
 154042025
 
 154042278
 
  ENSEMBL   UCSC 
 
  254
 
  5
 
 27959
 
 gene
 
 0.01783
 
 0.03238
 
 Not queried
 
  0.6517769
 
       NA
 
       NA
  
 
  
 1
 
   1114912
 
   1115070
 
  ENSEMBL   UCSC 
 
  159
 
  4
 
 1140
 
 tss
 
 0.90662
 
 0.87671
 
 Not queried
 
  0.6516524
 
       NA
 
       NA
  
 
  
 14
 
  34420292
 
  34420683
 
  ENSEMBL   UCSC 
 
  392
 
  8
 
 12317
 
 tss
 
 0.02412
 
 0.03202
 
 Not queried
 
  0.6516481
 
       NA
 
       NA
  
 
  
 5
 
 177659919
 
 177660036
 
  ENSEMBL   UCSC 
 
  118
 
  4
 
 32479
 
 tss;island
 
 0.21667
 
 0.13619
 
 Not queried
 
  0.6515917
 
       NA
 
       NA
  
 
  
 10
 
  15902539
 
  15902887
 
  ENSEMBL   UCSC 
 
  349
 
  6
 
 4949
 
 tss;island
 
 0.28703
 
 0.13903
 
 Not queried
 
  0.6515638
 
       NA
 
       NA
  
 
  
 3
 
 139258658
 
 139258948
 
  ENSEMBL   UCSC 
 
  291
 
  8
 
 29496
 
 island
 
 0.04415
 
 0.04750
 
 Not queried
 
  0.6514842
 
       NA
 
       NA
  
 
  
 2
 
 219920195
 
 219920517
 
  ENSEMBL   UCSC 
 
  323
 
  4
 
 22987
 
 gene;island
 
 0.71087
 
 0.60550
 
 Not queried
 
  0.6514682
 
       NA
 
       NA
  
 
  
 3
 
 123987591
 
 123988013
 
  ENSEMBL   UCSC 
 
  423
 
  6
 
 27879
 
 gene;island
 
 0.83056
 
 0.74992
 
 Not queried
 
  0.6514101
 
       NA
 
       NA
  
 
  
 1
 
  10532618
 
  10532886
 
  ENSEMBL   UCSC 
 
  269
 
  8
 
 1236
 
 tss
 
 0.00930
 
 0.01624
 
 Not queried
 
  0.6513616
 
       NA
 
       NA
  
 
  
 12
 
  51984943
 
  51985016
 
  ENSEMBL   UCSC 
 
   74
 
  5
 
 9693
 
 tss
 
 0.03832
 
 0.05179
 
 Not queried
 
  0.6513547
 
       NA
 
       NA
  
 
  
 13
 
  20735236
 
  20735532
 
  ENSEMBL   UCSC 
 
  297
 
  7
 
 11299
 
 tss
 
 0.04535
 
 0.02247
 
 Not queried
 
  0.6513527
 
       NA
 
       NA
  
 
  
 15
 
  41952235
 
  41952827
 
  ENSEMBL   UCSC 
 
  593
 
  8
 
 14038
 
 island
 
 0.03643
 
 0.02540
 
 Not queried
 
  0.6512986
 
       NA
 
       NA
  
 
  
 1
 
  89357317
 
  89357426
 
  ENSEMBL   UCSC 
 
  110
 
  4
 
 1733
 
 tss
 
 0.02972
 
 0.01175
 
 Not queried
 
  0.6512805
 
       NA
 
       NA
  
 
  
 15
 
  75660973
 
  75661354
 
  ENSEMBL   UCSC 
 
  382
 
  9
 
 13783
 
 tss
 
 0.01069
 
 0.01916
 
 Not queried
 
  0.6512198
 
       NA
 
       NA
  
 
  
 10
 
    695844
 
    696063
 
  ENSEMBL   UCSC 
 
  220
 
  6
 
 4436
 
 gene
 
 0.86184
 
 0.92348
 
 Not queried
 
  0.6511109
 
       NA
 
       NA
  
 
  
 10
 
 102504022
 
 102504297
 
  ENSEMBL   UCSC 
 
  276
 
  5
 
 5279
 
 tss;island
 
 0.01870
 
 0.04381
 
 Not queried
 
  0.6510863
 
       NA
 
       NA
  
 
  
 1
 
 228674817
 
 228674966
 
  ENSEMBL   UCSC 
 
  150
 
  5
 
 2304
 
 tss
 
 0.01803
 
 0.02849
 
 Not queried
 
  0.6510635
 
       NA
 
       NA
  
 
  
 1
 
 228674817
 
 228675139
 
  ENSEMBL   UCSC 
 
  323
 
  7
 
 4291
 
 island
 
 0.02557
 
 0.03425
 
 Not queried
 
  0.6510635
 
       NA
 
       NA
  
 
  
 1
 
  40420503
 
  40421002
 
  ENSEMBL   UCSC 
 
  500
 
 13
 
 3105
 
 island
 
 0.08792
 
 0.05900
 
 Not queried
 
  0.6508766
 
       NA
 
       NA
  
 
  
 2
 
 220432815
 
 220432956
 
  ENSEMBL   UCSC 
 
  142
 
  4
 
 23005
 
 gene;island
 
 0.85321
 
 0.82753
 
 Not queried
 
  0.6508568
 
       NA
 
       NA
  
 
  
 X
 
  77359355
 
  77359613
 
  ENSEMBL   UCSC 
 
  259
 
  9
 
 41402
 
 tss
 
 0.00879
 
 0.01600
 
 Not queried
 
  0.6508093
 
       NA
 
       NA
  
 
  
 X
 
  77359355
 
  77359924
 
  ENSEMBL   UCSC 
 
  570
 
 13
 
 41887
 
 island
 
 0.00879
 
 0.01407
 
 Not queried
 
  0.6508093
 
       NA
 
       NA
  
 
  
 11
 
  65150083
 
  65150416
 
  ENSEMBL   UCSC 
 
  334
 
  5
 
 8429
 
 island
 
 0.70774
 
 0.66534
 
 Not queried
 
  0.6507172
 
       NA
 
       NA
  
 
  
 2
 
  87035609
 
  87035758
 
  ENSEMBL   UCSC 
 
  150
 
  7
 
 23458
 
 tss
 
 0.01324
 
 0.03492
 
 Not queried
 
  0.6505622
 
       NA
 
       NA
  
 
  
 4
 
  77172861
 
  77173197
 
  ENSEMBL   UCSC 
 
  337
 
  4
 
 29982
 
 gene
 
 0.04627
 
 0.03795
 
 Not queried
 
  0.6505266
 
       NA
 
       NA
  
 
  
 2
 
 232651086
 
 232651151
 
  ENSEMBL   UCSC 
 
   66
 
  6
 
 23907
 
 tss
 
 0.01523
 
 0.01495
 
 Not queried
 
  0.6505202
 
       NA
 
       NA
  
 
  
 4
 
  72052798
 
  72053040
 
  ENSEMBL   UCSC 
 
  243
 
  5
 
 31038
 
 island
 
 0.02302
 
 0.02393
 
 Not queried
 
  0.6501938
 
       NA
 
       NA
  
 
  
 1
 
    917642
 
    917892
 
  ENSEMBL   UCSC 
 
  251
 
  5
 
 1133
 
 tss;island
 
 0.55913
 
 0.71434
 
 Not queried
 
  0.6501438
 
       NA
 
       NA
  
 
  
 11
 
   9335949
 
   9336131
 
  ENSEMBL   UCSC 
 
  183
 
  4
 
 6486
 
 gene;island
 
 0.01633
 
 0.02272
 
 Not queried
 
  0.6501174
 
       NA
 
       NA
  
 
  
 17
 
   2324794
 
   2325143
 
  ENSEMBL   UCSC 
 
  350
 
  5
 
 16296
 
 gene
 
 0.68503
 
 0.80940
 
 Not queried
 
  0.6501156
 
       NA
 
       NA
  
 
  
 2
 
  69614107
 
  69614473
 
  ENSEMBL   UCSC 
 
  367
 
  9
 
 24329
 
 island
 
 0.01358
 
 0.01392
 
 Not queried
 
  0.6501075
 
       NA
 
       NA
  
 
  
 5
 
  44808792
 
  44808992
 
  ENSEMBL   UCSC 
 
  201
 
  6
 
 31994
 
 tss
 
 0.01338
 
 0.02765
 
 Not queried
 
  0.6500221
 
       NA
 
       NA
  
 
  
 4
 
 147558089
 
 147558544
 
  ENSEMBL   UCSC 
 
  456
 
  4
 
 31297
 
 island
 
 0.03759
 
 0.15451
 
 Not queried
 
  0.6499307
 
       NA
 
       NA
  
 
  
 19
 
  50379799
 
  50380022
 
  ENSEMBL   UCSC 
 
  224
 
  4
 
 20130
 
 gene;tss;island
 
 0.01286
 
 0.01135
 
 Not queried
 
  0.6499145
 
       NA
 
       NA
  
 
  
 19
 
  22817176
 
  22817496
 
  ENSEMBL   UCSC 
 
  321
 
  4
 
 19930
 
 gene
 
 0.08439
 
 0.09881
 
 Not queried
 
  0.6498689
 
       NA
 
       NA
  
 
  
 17
 
   8021572
 
   8022575
 
  ENSEMBL   UCSC 
 
 1004
 
 14
 
 17951
 
 island
 
 0.03016
 
 0.03521
 
 Not queried
 
  0.6498332
 
       NA
 
       NA
  
 
  
 X
 
 134156561
 
 134156809
 
  ENSEMBL   UCSC 
 
  249
 
  6
 
 42046
 
 island
 
 0.03017
 
 0.03168
 
 Not queried
 
  0.6497163
 
       NA
 
       NA
  
 
  
 2
 
 220117599
 
 220117945
 
  ENSEMBL   UCSC 
 
  347
 
  7
 
 22992
 
 gene;tss;island
 
 0.40339
 
 0.49672
 
 Not queried
 
  0.6496384
 
       NA
 
       NA
  
 
  
 14
 
 102829091
 
 102829271
 
  ENSEMBL   UCSC 
 
  181
 
  8
 
 12616
 
 tss;island
 
 0.02218
 
 0.03239
 
 Not queried
 
  0.6495282
 
       NA
 
       NA
  
 
  
 1
 
 161171211
 
 161172049
 
  ENSEMBL   UCSC 
 
  839
 
  9
 
 850
 
 gene;island
 
 0.35273
 
 0.38208
 
 Not queried
 
  0.6494904
 
       NA
 
       NA
  
 
  
 4
 
  37455280
 
  37455499
 
  ENSEMBL   UCSC 
 
  220
 
  7
 
 30294
 
 tss;island
 
 0.02834
 
 0.10393
 
 Not queried
 
  0.6494675
 
       NA
 
       NA
  
 
  
 18
 
  52495404
 
  52496070
 
  ENSEMBL   UCSC 
 
  667
 
 11
 
 19491
 
 island
 
 0.03341
 
 0.05287
 
 Not queried
 
  0.6494447
 
       NA
 
       NA
  
 
  
 12
 
  52579502
 
  52579837
 
  ENSEMBL   UCSC 
 
  336
 
  4
 
 9109
 
 gene
 
 0.54100
 
 0.67487
 
 Not queried
 
  0.6494184
 
       NA
 
       NA
  
 
  
 6
 
 109703809
 
 109704021
 
  ENSEMBL   UCSC 
 
  213
 
  7
 
 35099
 
 tss;island
 
 0.01311
 
 0.02737
 
 Not queried
 
  0.6493826
 
       NA
 
       NA
  
 
  
 3
 
  55517496
 
  55517853
 
  ENSEMBL   UCSC 
 
  358
 
  4
 
 27796
 
 gene;island
 
 0.79816
 
 0.92442
 
 Not queried
 
  0.6493768
 
       NA
 
       NA
  
 
  
 5
 
 102455768
 
 102456179
 
  ENSEMBL   UCSC 
 
  412
 
 10
 
 32927
 
 island
 
 0.02021
 
 0.02613
 
 Not queried
 
  0.6493389
 
       NA
 
       NA
  
 
  
 6
 
   6588693
 
   6589075
 
  ENSEMBL   UCSC 
 
  383
 
  5
 
 33484
 
 gene
 
 0.72260
 
 0.73091
 
 Not queried
 
  0.6493229
 
       NA
 
       NA
  
 
  
 11
 
   2593857
 
   2594153
 
  ENSEMBL   UCSC 
 
  297
 
  4
 
 6445
 
 gene;island
 
 0.68323
 
 0.77173
 
 Not queried
 
  0.6493191
 
       NA
 
       NA
  
 
  
 1
 
   1108820
 
   1109244
 
  ENSEMBL   UCSC 
 
  425
 
  6
 
 1139
 
 tss
 
 0.75676
 
 0.82058
 
 Not queried
 
  0.6492896
 
       NA
 
       NA
  
 
  
 2
 
  37311377
 
  37311892
 
  ENSEMBL   UCSC 
 
  516
 
 10
 
 23265
 
 tss;island
 
 0.01541
 
 0.01834
 
 Not queried
 
  0.6492831
 
       NA
 
       NA
  
 
  
 12
 
  48397568
 
  48398056
 
  ENSEMBL   UCSC 
 
  489
 
  4
 
 9083
 
 gene;island
 
 0.20940
 
 0.21730
 
 Not queried
 
  0.6491394
 
       NA
 
       NA
  
 
  
 20
 
  45523098
 
  45523187
 
  ENSEMBL   UCSC 
 
   90
 
  4
 
 25759
 
 tss
 
 0.02245
 
 0.04290
 
 Not queried
 
  0.6491191
 
       NA
 
       NA
  
 
  
 16
 
  87958145
 
  87958493
 
  ENSEMBL   UCSC 
 
  349
 
  4
 
 14864
 
 gene
 
 0.91473
 
 0.87768
 
 Not queried
 
  0.6490898
 
       NA
 
       NA
  
 
  
 4
 
   1736942
 
   1737129
 
  ENSEMBL   UCSC 
 
  188
 
  4
 
 29829
 
 gene;island
 
 0.96303
 
 0.92742
 
 Not queried
 
  0.6490884
 
       NA
 
       NA
  
 
  
 20
 
  54919037
 
  54919410
 
  ENSEMBL   UCSC 
 
  374
 
  6
 
 26231
 
 island
 
 0.07523
 
 0.04108
 
 Not queried
 
  0.6490570
 
       NA
 
       NA
  
 
  
 10
 
  99609716
 
  99609982
 
  ENSEMBL   UCSC 
 
  267
 
  7
 
 5257
 
 tss
 
 0.05249
 
 0.05008
 
 Not queried
 
  0.6490400
 
       NA
 
       NA
  
 
  
 12
 
   6677610
 
   6677729
 
  ENSEMBL   UCSC 
 
  120
 
  5
 
 9492
 
 tss;island
 
 0.02953
 
 0.05239
 
 Not queried
 
  0.6490317
 
       NA
 
       NA
  
 
  
 17
 
  65362673
 
  65363007
 
  ENSEMBL   UCSC 
 
  335
 
  9
 
 18645
 
 island
 
 0.01469
 
 0.02450
 
 Not queried
 
  0.6489456
 
       NA
 
       NA
  
 
  
 22
 
  50493126
 
  50493212
 
  ENSEMBL   UCSC 
 
   87
 
  4
 
 27201
 
 tss
 
 0.94724
 
 0.90785
 
 Not queried
 
  0.6488794
 
       NA
 
       NA
  
 
  
 15
 
  37387304
 
  37387577
 
  ENSEMBL   UCSC 
 
  274
 
  5
 
 13254
 
 gene;island
 
 0.06404
 
 0.06530
 
 Not queried
 
  0.6488698
 
       NA
 
       NA
  
 
  
 6
 
  32032689
 
  32033006
 
  ENSEMBL   UCSC 
 
  318
 
 10
 
 33966
 
 gene
 
 0.80369
 
 0.65834
 
 Not queried
 
  0.6485973
 
       NA
 
       NA
  
 
  
 12
 
  65152772
 
  65153209
 
  ENSEMBL   UCSC 
 
  438
 
  5
 
 9179
 
 gene
 
 0.02438
 
 0.03914
 
 Not queried
 
  0.6485706
 
       NA
 
       NA
  
 
  
 1
 
  61522716
 
  61523012
 
  ENSEMBL   UCSC 
 
  297
 
  4
 
 3322
 
 island
 
 0.02714
 
 0.04852
 
 Not queried
 
  0.6485631
 
       NA
 
       NA
  
 
  
 11
 
  45906914
 
  45907154
 
  ENSEMBL   UCSC 
 
  241
 
  6
 
 7249
 
 tss;island
 
 0.06177
 
 0.08677
 
 Not queried
 
  0.6484959
 
       NA
 
       NA
  
 
  
 1
 
   6453807
 
   6454339
 
  ENSEMBL   UCSC 
 
  533
 
 10
 
 2598
 
 island
 
 0.03401
 
 0.05571
 
 Not queried
 
  0.6483353
 
       NA
 
       NA
  
 
  
 X
 
  23801333
 
  23801656
 
  ENSEMBL   UCSC 
 
  324
 
  4
 
 40949
 
 gene
 
 0.01771
 
 0.02580
 
 Not queried
 
  0.6483298
 
       NA
 
       NA
  
 
  
 10
 
  70091418
 
  70091938
 
  ENSEMBL   UCSC 
 
  521
 
 11
 
 4592
 
 gene;island
 
 0.02476
 
 0.02543
 
 Not queried
 
  0.6482490
 
       NA
 
       NA
  
 
  
 10
 
  70091418
 
  70091743
 
  ENSEMBL   UCSC 
 
  326
 
  9
 
 5093
 
 tss
 
 0.03559
 
 0.03710
 
 Not queried
 
  0.6482490
 
       NA
 
       NA
  
 
  
 19
 
  39694477
 
  39695237
 
  ENSEMBL   UCSC 
 
  761
 
  9
 
 21956
 
 island
 
 0.79758
 
 0.88189
 
 Not queried
 
  0.6482435
 
       NA
 
       NA
  
 
  
 7
 
  26240901
 
  26241126
 
  ENSEMBL   UCSC 
 
  226
 
  7
 
 37418
 
 tss;island
 
 0.02681
 
 0.01727
 
 Not queried
 
  0.6482414
 
       NA
 
       NA
  
 
  
 6
 
  33235504
 
  33235977
 
  ENSEMBL   UCSC 
 
  474
 
  9
 
 34174
 
 gene;island
 
 0.87563
 
 0.87415
 
 Not queried
 
  0.6482116
 
       NA
 
       NA
  
 
  
 7
 
  23719453
 
  23719682
 
  ENSEMBL   UCSC 
 
  230
 
  7
 
 37410
 
 tss
 
 0.43725
 
 0.34207
 
 Not queried
 
  0.6481934
 
       NA
 
       NA
  
 
  
 10
 
  99329961
 
  99330203
 
  ENSEMBL   UCSC 
 
  243
 
  5
 
 4683
 
 gene;island
 
 0.79454
 
 0.86481
 
 Not queried
 
  0.6481152
 
       NA
 
       NA
  
 
  
 11
 
 120434710
 
 120435056
 
  ENSEMBL   UCSC 
 
  347
 
  4
 
 8872
 
 island
 
 0.14283
 
 0.10547
 
 Not queried
 
  0.6480517
 
       NA
 
       NA
  
 
  
 6
 
  33272880
 
  33273128
 
  ENSEMBL   UCSC 
 
  249
 
  5
 
 34197
 
 gene
 
 0.66326
 
 0.56974
 
 Not queried
 
  0.6479709
 
       NA
 
       NA
  
 
  
 3
 
 169490965
 
 169491284
 
  ENSEMBL   UCSC 
 
  320
 
  4
 
 27981
 
 gene
 
 0.01907
 
 0.03020
 
 Not queried
 
  0.6478536
 
       NA
 
       NA
  
 
  
 3
 
 183735660
 
 183736171
 
  ENSEMBL   UCSC 
 
  512
 
  9
 
 29666
 
 island
 
 0.02560
 
 0.04460
 
 Not queried
 
  0.6477301
 
       NA
 
       NA
  
 
  
 11
 
  20408536
 
  20408972
 
  ENSEMBL   UCSC 
 
  437
 
  8
 
 7177
 
 tss;island
 
 0.00839
 
 0.01648
 
 Not queried
 
  0.6476979
 
       NA
 
       NA
  
 
  
 4
 
   2278261
 
   2278594
 
  ENSEMBL   UCSC 
 
  334
 
  5
 
 29841
 
 gene;island
 
 0.79053
 
 0.85534
 
 Not queried
 
  0.6476877
 
       NA
 
       NA
  
 
  
 22
 
  29784624
 
  29784813
 
  ENSEMBL   UCSC 
 
  190
 
  8
 
 27006
 
 tss
 
 0.01584
 
 0.02640
 
 Not queried
 
  0.6476782
 
       NA
 
       NA
  
 
  
 13
 
  51825774
 
  51826086
 
  ENSEMBL   UCSC 
 
  313
 
  4
 
 11142
 
 gene;island
 
 0.82595
 
 0.86564
 
 Not queried
 
  0.6476652
 
       NA
 
       NA
  
 
  
 2
 
  96012586
 
  96012735
 
  ENSEMBL   UCSC 
 
  150
 
  4
 
 23472
 
 tss
 
 0.09294
 
 0.06305
 
 Not queried
 
  0.6475505
 
       NA
 
       NA
  
 
  
 1
 
 145096479
 
 145096849
 
  ENSEMBL   UCSC 
 
  371
 
  5
 
 720
 
 gene
 
 0.02710
 
 0.01777
 
 Not queried
 
  0.6475132
 
       NA
 
       NA
  
 
  
 1
 
 153931119
 
 153931566
 
  ENSEMBL   UCSC 
 
  448
 
  7
 
 1952
 
 tss;island
 
 0.03985
 
 0.05657
 
 Not queried
 
  0.6474871
 
       NA
 
       NA
  
 
  
 19
 
  42806843
 
  42807187
 
  ENSEMBL   UCSC 
 
  345
 
  9
 
 20032
 
 gene;tss;island
 
 0.02364
 
 0.06284
 
 Not queried
 
  0.6473524
 
       NA
 
       NA
  
 
  
 1
 
 246930936
 
 246931160
 
  ENSEMBL   UCSC 
 
  225
 
  4
 
 1120
 
 gene
 
 0.92772
 
 0.96437
 
 Not queried
 
  0.6469729
 
       NA
 
       NA
  
 
  
 7
 
   4824387
 
   4824800
 
  ENSEMBL   UCSC 
 
  414
 
  4
 
 36791
 
 gene;island
 
 0.79573
 
 0.86204
 
 Not queried
 
  0.6468459
 
       NA
 
       NA
  
 
  
 1
 
 181452618
 
 181452839
 
  ENSEMBL   UCSC 
 
  222
 
  4
 
 4006
 
 island
 
 0.18616
 
 0.25710
 
 Not queried
 
  0.6467874
 
       NA
 
       NA
  
 
  
 6
 
   4021316
 
   4021498
 
  ENSEMBL   UCSC 
 
  183
 
  7
 
 34538
 
 tss
 
 0.03640
 
 0.03680
 
 Not queried
 
  0.6467400
 
       NA
 
       NA
  
 
  
 6
 
   4021316
 
   4021748
 
  ENSEMBL   UCSC 
 
  433
 
  9
 
 35327
 
 island
 
 0.03640
 
 0.03992
 
 Not queried
 
  0.6467400
 
       NA
 
       NA
  
 
  
 4
 
 185354977
 
 185355309
 
  ENSEMBL   UCSC 
 
  333
 
  4
 
 30155
 
 gene
 
 0.90343
 
 0.93189
 
 Not queried
 
  0.6467018
 
       NA
 
       NA
  
 
  
 2
 
 177012117
 
 177012706
 
  ENSEMBL   UCSC 
 
  590
 
  7
 
 24868
 
 island
 
 0.73193
 
 0.67811
 
 Not queried
 
  0.6466196
 
       NA
 
       NA
  
 
  
 5
 
 122180749
 
 122181105
 
  ENSEMBL   UCSC 
 
  357
 
  8
 
 32187
 
 tss;island
 
 0.01233
 
 0.02151
 
 Not queried
 
  0.6464269
 
       NA
 
       NA
  
 
  
 1
 
 244013668
 
 244014210
 
  ENSEMBL   UCSC 
 
  543
 
  6
 
 4376
 
 island
 
 0.02489
 
 0.03842
 
 Not queried
 
  0.6464194
 
       NA
 
       NA
  
 
  
 3
 
 172167445
 
 172167644
 
  ENSEMBL   UCSC 
 
  200
 
  4
 
 28678
 
 tss
 
 0.04953
 
 0.09117
 
 Not queried
 
  0.6463514
 
       NA
 
       NA
  
 
  
 2
 
  38892846
 
  38892958
 
  ENSEMBL   UCSC 
 
  113
 
  4
 
 23274
 
 tss
 
 0.02397
 
 0.02416
 
 Not queried
 
  0.6463371
 
       NA
 
       NA
  
 
  
 7
 
 157502370
 
 157502620
 
  ENSEMBL   UCSC 
 
  251
 
  4
 
 37277
 
 gene;island
 
 0.69009
 
 0.76778
 
 Not queried
 
  0.6462318
 
       NA
 
       NA
  
 
  
 6
 
  30619047
 
  30619242
 
  ENSEMBL   UCSC 
 
  196
 
 11
 
 33731
 
 gene;island
 
 0.87445
 
 0.89857
 
 Not queried
 
  0.6461827
 
       NA
 
       NA
  
 
  
 10
 
  73078578
 
  73078971
 
  ENSEMBL   UCSC 
 
  394
 
  6
 
 5125
 
 tss
 
 0.14394
 
 0.12248
 
 Not queried
 
  0.6461535
 
       NA
 
       NA
  
 
  
 2
 
  69001665
 
  69002378
 
  ENSEMBL   UCSC 
 
  714
 
 11
 
 22680
 
 gene
 
 0.64723
 
 0.70730
 
 Not queried
 
  0.6461003
 
       NA
 
       NA
  
 
  
 2
 
  69001665
 
  69001870
 
  ENSEMBL   UCSC 
 
  206
 
  7
 
 23364
 
 tss
 
 0.67716
 
 0.72788
 
 Not queried
 
  0.6461003
 
       NA
 
       NA
  
 
  
 1
 
 155247091
 
 155247361
 
  ENSEMBL   UCSC 
 
  271
 
  7
 
 1989
 
 tss
 
 0.02817
 
 0.04274
 
 Not queried
 
  0.6459505
 
       NA
 
       NA
  
 
  
 1
 
   3157287
 
   3157510
 
  ENSEMBL   UCSC 
 
  224
 
  4
 
 110
 
 gene;island
 
 0.86729
 
 0.91755
 
 Not queried
 
  0.6459408
 
       NA
 
       NA
  
 
  
 8
 
 110657174
 
 110657197
 
  ENSEMBL   UCSC 
 
   24
 
  5
 
 39809
 
 tss
 
 0.01523
 
 0.01225
 
 Not queried
 
  0.6459068
 
       NA
 
       NA
  
 
  
 17
 
   1028673
 
   1028931
 
  ENSEMBL   UCSC 
 
  259
 
  4
 
 16273
 
 gene;island
 
 0.89735
 
 0.87769
 
 Not queried
 
  0.6458542
 
       NA
 
       NA
  
 
  
 10
 
 133955398
 
 133955570
 
  ENSEMBL   UCSC 
 
  173
 
  5
 
 4838
 
 gene;island
 
 0.88556
 
 0.88151
 
 Not queried
 
  0.6458434
 
       NA
 
       NA
  
 
  
 3
 
 185655795
 
 185656289
 
  ENSEMBL   UCSC 
 
  495
 
  6
 
 29700
 
 island
 
 0.25585
 
 0.19475
 
 Not queried
 
  0.6457856
 
       NA
 
       NA
  
 
  
 4
 
  68411268
 
  68411531
 
  ENSEMBL   UCSC 
 
  264
 
  8
 
 30371
 
 tss
 
 0.02417
 
 0.04002
 
 Not queried
 
  0.6457779
 
       NA
 
       NA
  
 
  
 4
 
  68411154
 
  68411531
 
  ENSEMBL   UCSC 
 
  378
 
 10
 
 31028
 
 island
 
 0.02196
 
 0.03116
 
 Not queried
 
  0.6457779
 
       NA
 
       NA
  
 
  
 2
 
 172778734
 
 172778879
 
  ENSEMBL   UCSC 
 
  146
 
  5
 
 23677
 
 tss
 
 0.02671
 
 0.03675
 
 Not queried
 
  0.6457391
 
       NA
 
       NA
  
 
  
 1
 
 109204168
 
 109204325
 
  ENSEMBL   UCSC 
 
  158
 
  5
 
 1787
 
 tss;island
 
 0.02178
 
 0.03364
 
 Not queried
 
  0.6457298
 
       NA
 
       NA
  
 
  
 6
 
   8064493
 
   8064800
 
  ENSEMBL   UCSC 
 
  308
 
  7
 
 35354
 
 island
 
 0.02212
 
 0.02246
 
 Not queried
 
  0.6457293
 
       NA
 
       NA
  
 
  
 12
 
  62586267
 
  62586892
 
  ENSEMBL   UCSC 
 
  626
 
  8
 
 10554
 
 island
 
 0.12197
 
 0.03371
 
 Not queried
 
  0.6457214
 
       NA
 
       NA
  
 
  
 3
 
 182817381
 
 182817626
 
  ENSEMBL   UCSC 
 
  246
 
  7
 
 28699
 
 tss
 
 0.02806
 
 0.05346
 
 Not queried
 
  0.6455097
 
       NA
 
       NA
  
 
  
 X
 
  95939631
 
  95939864
 
  ENSEMBL   UCSC 
 
  234
 
  4
 
 41898
 
 island
 
 0.03839
 
 0.02753
 
 Not queried
 
  0.6454786
 
       NA
 
       NA
  
 
  
 7
 
 130126153
 
 130126637
 
  ENSEMBL   UCSC 
 
  485
 
  6
 
 37160
 
 gene
 
 0.02336
 
 0.01489
 
 Not queried
 
  0.6454351
 
       NA
 
       NA
  
 
  
 7
 
 130125200
 
 130126637
 
  ENSEMBL   UCSC 
 
 1438
 
 18
 
 38858
 
 island
 
 0.15014
 
 0.04049
 
 Not queried
 
  0.6454351
 
       NA
 
       NA
  
 
  
 13
 
  96204493
 
  96204978
 
  ENSEMBL   UCSC 
 
  486
 
 10
 
 11165
 
 gene;island
 
 0.03060
 
 0.05157
 
 Not queried
 
  0.6453114
 
       NA
 
       NA
  
 
  
 2
 
  74685383
 
  74685602
 
  ENSEMBL   UCSC 
 
  220
 
  8
 
 24396
 
 island
 
 0.02188
 
 0.01739
 
 Not queried
 
  0.6453060
 
       NA
 
       NA
  
 
  
 2
 
  27651135
 
  27651571
 
  ENSEMBL   UCSC 
 
  437
 
  6
 
 24146
 
 island
 
 0.01779
 
 0.02267
 
 Not queried
 
  0.6452113
 
       NA
 
       NA
  
 
  
 15
 
  65197883
 
  65198005
 
  ENSEMBL   UCSC 
 
  123
 
  4
 
 14193
 
 island
 
 0.38137
 
 0.47857
 
 Not queried
 
  0.6451922
 
       NA
 
       NA
  
 
  
 1
 
 109288968
 
 109289113
 
  ENSEMBL   UCSC 
 
  146
 
  6
 
 1789
 
 tss
 
 0.02341
 
 0.02813
 
 Not queried
 
  0.6451125
 
       NA
 
       NA
  
 
  
 7
 
  19748663
 
  19748896
 
  ENSEMBL   UCSC 
 
  234
 
  8
 
 37397
 
 tss
 
 0.01826
 
 0.02765
 
 Not queried
 
  0.6450725
 
       NA
 
       NA
  
 
  
 X
 
  38660266
 
  38660667
 
  ENSEMBL   UCSC 
 
  402
 
  8
 
 41259
 
 tss
 
 0.06284
 
 0.03958
 
 Not queried
 
  0.6450225
 
       NA
 
       NA
  
 
  
 11
 
  67407037
 
  67407224
 
  ENSEMBL   UCSC 
 
  188
 
  5
 
 7478
 
 tss
 
 0.84353
 
 0.77464
 
 Not queried
 
  0.6449559
 
       NA
 
       NA
  
 
  
 7
 
 138818186
 
 138818439
 
  ENSEMBL   UCSC 
 
  254
 
  6
 
 37864
 
 tss
 
 0.01685
 
 0.01166
 
 Not queried
 
  0.6448121
 
       NA
 
       NA
  
 
  
 7
 
  84569142
 
  84569518
 
  ENSEMBL   UCSC 
 
  377
 
  4
 
 38563
 
 island
 
 0.61461
 
 0.72938
 
 Not queried
 
  0.6447175
 
       NA
 
       NA
  
 
  
 7
 
 157791721
 
 157792100
 
  ENSEMBL   UCSC 
 
  380
 
  4
 
 37292
 
 gene
 
 0.89259
 
 0.93980
 
 Not queried
 
  0.6446871
 
       NA
 
       NA
  
 
  
 2
 
  74645353
 
  74645769
 
  ENSEMBL   UCSC 
 
  417
 
  6
 
 23415
 
 tss;island
 
 0.43193
 
 0.65173
 
 Not queried
 
  0.6445926
 
       NA
 
       NA
  
 
  
 19
 
   6739524
 
   6740123
 
  ENSEMBL   UCSC 
 
  600
 
  6
 
 21403
 
 island
 
 0.02589
 
 0.03897
 
 Not queried
 
  0.6445729
 
       NA
 
       NA
  
 
  
 17
 
  54893255
 
  54893317
 
  ENSEMBL   UCSC 
 
   63
 
  4
 
 17488
 
 tss
 
 0.92170
 
 0.89720
 
 Not queried
 
  0.6445658
 
       NA
 
       NA
  
 
  
 5
 
  89705234
 
  89705579
 
  ENSEMBL   UCSC 
 
  346
 
  4
 
 31627
 
 gene
 
 0.01573
 
 0.01964
 
 Not queried
 
  0.6444671
 
       NA
 
       NA
  
 
  
 1
 
 156084490
 
 156084750
 
  ENSEMBL   UCSC 
 
  261
 
  5
 
 812
 
 gene
 
 0.01532
 
 0.02461
 
 Not queried
 
  0.6444483
 
       NA
 
       NA
  
 
  
 4
 
  87281281
 
  87281511
 
  ENSEMBL   UCSC 
 
  231
 
  4
 
 30003
 
 gene
 
 0.21979
 
 0.12747
 
 Not queried
 
  0.6443369
 
       NA
 
       NA
  
 
  
 6
 
 100903561
 
 100904055
 
  ENSEMBL   UCSC 
 
  495
 
  7
 
 34366
 
 gene;island
 
 0.27713
 
 0.14564
 
 Not queried
 
  0.6442633
 
       NA
 
       NA
  
 
  
 6
 
 170582184
 
 170582543
 
  ENSEMBL   UCSC 
 
  360
 
  4
 
 36624
 
 island
 
 0.85164
 
 0.72020
 
 Not queried
 
  0.6442002
 
       NA
 
       NA
  
 
  
 10
 
 131591484
 
 131591629
 
  ENSEMBL   UCSC 
 
  146
 
  4
 
 6241
 
 island
 
 0.72413
 
 0.82796
 
 Not queried
 
  0.6441667
 
       NA
 
       NA
  
 
  
 5
 
 176449346
 
 176449687
 
  ENSEMBL   UCSC 
 
  342
 
  9
 
 32461
 
 tss
 
 0.01277
 
 0.02682
 
 Not queried
 
  0.6441460
 
       NA
 
       NA
  
 
  
 5
 
 176449346
 
 176450179
 
  ENSEMBL   UCSC 
 
  834
 
 13
 
 33374
 
 island
 
 0.01495
 
 0.02750
 
 Not queried
 
  0.6441460
 
       NA
 
       NA
  
 
  
 2
 
  61245136
 
  61245510
 
  ENSEMBL   UCSC 
 
  375
 
  8
 
 22659
 
 gene
 
 0.03081
 
 0.03949
 
 Not queried
 
  0.6440410
 
       NA
 
       NA
  
 
  
 11
 
  33722319
 
  33722598
 
  ENSEMBL   UCSC 
 
  280
 
  7
 
 7214
 
 tss
 
 0.04852
 
 0.02728
 
 Not queried
 
  0.6439799
 
       NA
 
       NA
  
 
  
 7
 
   1022841
 
   1023156
 
  ENSEMBL   UCSC 
 
  316
 
  5
 
 36668
 
 gene
 
 0.75330
 
 0.74689
 
 Not queried
 
  0.6439485
 
       NA
 
       NA
  
 
  
 7
 
   1022471
 
   1023156
 
  ENSEMBL   UCSC 
 
  686
 
 11
 
 37999
 
 island
 
 0.78916
 
 0.86789
 
 Not queried
 
  0.6439485
 
       NA
 
       NA
  
 
  
 5
 
 111496931
 
 111497173
 
  ENSEMBL   UCSC 
 
  243
 
  6
 
 31655
 
 gene;tss;island
 
 0.01683
 
 0.01878
 
 Not queried
 
  0.6439401
 
       NA
 
       NA
  
 
  
 15
 
  63889093
 
  63889538
 
  ENSEMBL   UCSC 
 
  446
 
  5
 
 13696
 
 tss
 
 0.54371
 
 0.67815
 
 Not queried
 
  0.6439342
 
       NA
 
       NA
  
 
  
 7
 
 122526824
 
 122526940
 
  ENSEMBL   UCSC 
 
  117
 
  6
 
 37802
 
 tss
 
 0.02512
 
 0.03042
 
 Not queried
 
  0.6438346
 
       NA
 
       NA
  
 
  
 14
 
  20773935
 
  20774101
 
  ENSEMBL   UCSC 
 
  167
 
  4
 
 11975
 
 gene
 
 0.02582
 
 0.05128
 
 Not queried
 
  0.6436985
 
       NA
 
       NA
  
 
  
 20
 
  21685834
 
  21686728
 
  ENSEMBL   UCSC 
 
  895
 
 14
 
 26002
 
 island
 
 0.04584
 
 0.10095
 
 Not queried
 
  0.6436624
 
       NA
 
       NA
  
 
  
 15
 
  81282230
 
  81282586
 
  ENSEMBL   UCSC 
 
  357
 
  6
 
 13823
 
 tss
 
 0.03206
 
 0.04002
 
 Not queried
 
  0.6435608
 
       NA
 
       NA
  
 
  
 15
 
  63674108
 
  63674276
 
  ENSEMBL   UCSC 
 
  169
 
  6
 
 13694
 
 tss;island
 
 0.06247
 
 0.05727
 
 Not queried
 
  0.6435575
 
       NA
 
       NA
  
 
  
 6
 
  33135753
 
  33136023
 
  ENSEMBL   UCSC 
 
  271
 
  6
 
 34133
 
 gene
 
 0.72236
 
 0.59945
 
 Not queried
 
  0.6435416
 
       NA
 
       NA
  
 
  
 11
 
  43902062
 
  43902343
 
  ENSEMBL   UCSC 
 
  282
 
  8
 
 7235
 
 tss
 
 0.42429
 
 0.50340
 
 Not queried
 
  0.6434350
 
       NA
 
       NA
  
 
  
 17
 
  29158893
 
  29158999
 
  ENSEMBL   UCSC 
 
  107
 
  6
 
 17210
 
 tss
 
 0.00746
 
 0.01294
 
 Not queried
 
  0.6433522
 
       NA
 
       NA
  
 
  
 5
 
  92956474
 
  92957098
 
  ENSEMBL   UCSC 
 
  625
 
 10
 
 31634
 
 gene;island
 
 0.01060
 
 0.02186
 
 Not queried
 
  0.6431741
 
       NA
 
       NA
  
 
  
 16
 
  58498456
 
  58498818
 
  ENSEMBL   UCSC 
 
  363
 
  8
 
 14742
 
 gene;island
 
 0.04305
 
 0.07371
 
 Not queried
 
  0.6429795
 
       NA
 
       NA
  
 
  
 6
 
  35265308
 
  35265568
 
  ENSEMBL   UCSC 
 
  261
 
  9
 
 34864
 
 tss
 
 0.03374
 
 0.09321
 
 Not queried
 
  0.6429050
 
       NA
 
       NA
  
 
  
 12
 
 106751060
 
 106751428
 
  ENSEMBL   UCSC 
 
  369
 
  7
 
 9943
 
 tss;island
 
 0.01680
 
 0.02599
 
 Not queried
 
  0.6428580
 
       NA
 
       NA
  
 
  
 1
 
   1655803
 
   1655942
 
  ENSEMBL   UCSC 
 
  140
 
  4
 
 1156
 
 tss
 
 0.02653
 
 0.03740
 
 Not queried
 
  0.6428390
 
       NA
 
       NA
  
 
  
 13
 
  20806477
 
  20806813
 
  ENSEMBL   UCSC 
 
  337
 
  8
 
 11303
 
 tss;island
 
 0.24212
 
 0.07048
 
 Not queried
 
  0.6428020
 
       NA
 
       NA
  
 
  
 14
 
  60952405
 
  60952945
 
  ENSEMBL   UCSC 
 
  541
 
  6
 
 12874
 
 island
 
 0.03899
 
 0.09297
 
 Not queried
 
  0.6427796
 
       NA
 
       NA
  
 
  
 17
 
   8023961
 
   8024253
 
  ENSEMBL   UCSC 
 
  293
 
  4
 
 16390
 
 gene;island
 
 0.14360
 
 0.11974
 
 Not queried
 
  0.6426420
 
       NA
 
       NA
  
 
  
 2
 
 139259034
 
 139259163
 
  ENSEMBL   UCSC 
 
  130
 
  5
 
 23614
 
 tss;island
 
 0.02005
 
 0.02331
 
 Not queried
 
  0.6426259
 
       NA
 
       NA
  
 
  
 1
 
  78148360
 
  78148604
 
  ENSEMBL   UCSC 
 
  245
 
  7
 
 1703
 
 tss
 
 0.01877
 
 0.03088
 
 Not queried
 
  0.6425494
 
       NA
 
       NA
  
 
  
 19
 
  58728390
 
  58728865
 
  ENSEMBL   UCSC 
 
  476
 
  5
 
 22461
 
 island
 
 0.71919
 
 0.81175
 
 Not queried
 
  0.6424697
 
       NA
 
       NA
  
 
  
 6
 
  28874479
 
  28874754
 
  ENSEMBL   UCSC 
 
  276
 
  4
 
 33577
 
 gene
 
 0.94400
 
 0.96407
 
 Not queried
 
  0.6424407
 
       NA
 
       NA
  
 
  
 6
 
  32151346
 
  32151882
 
  ENSEMBL   UCSC 
 
  537
 
  8
 
 34015
 
 gene
 
 0.75711
 
 0.89903
 
 Not queried
 
  0.6424218
 
       NA
 
       NA
  
 
  
 12
 
  16758692
 
  16759029
 
  ENSEMBL   UCSC 
 
  338
 
  6
 
 9047
 
 gene
 
 0.02776
 
 0.02497
 
 Not queried
 
  0.6424097
 
       NA
 
       NA
  
 
  
 6
 
  32977983
 
  32978775
 
  ENSEMBL   UCSC 
 
  793
 
 16
 
 34823
 
 tss;island
 
 0.84837
 
 0.63305
 
 Not queried
 
  0.6423144
 
       NA
 
       NA
  
 
  
 19
 
  50316212
 
  50316878
 
  ENSEMBL   UCSC 
 
  667
 
 11
 
 22255
 
 island
 
 0.03507
 
 0.03128
 
 Not queried
 
  0.6422340
 
       NA
 
       NA
  
 
  
 17
 
  19436770
 
  19437157
 
  ENSEMBL   UCSC 
 
  388
 
  7
 
 17159
 
 tss
 
 0.07300
 
 0.03192
 
 Not queried
 
  0.6420180
 
       NA
 
       NA
  
 
  
 14
 
  68283326
 
  68283527
 
  ENSEMBL   UCSC 
 
  202
 
  4
 
 12448
 
 tss
 
 0.01574
 
 0.02169
 
 Not queried
 
  0.6418991
 
       NA
 
       NA
  
 
  
 1
 
 234350263
 
 234350515
 
  ENSEMBL   UCSC 
 
  253
 
  4
 
 1081
 
 gene;island
 
 0.05022
 
 0.07429
 
 Not queried
 
  0.6418634
 
       NA
 
       NA
  
 
  
 9
 
 139908753
 
 139909161
 
  ENSEMBL   UCSC 
 
  409
 
  4
 
 40731
 
 gene;island
 
 0.83241
 
 0.77363
 
 Not queried
 
  0.6418548
 
       NA
 
       NA
  
 
  
 7
 
  56119384
 
  56119637
 
  ENSEMBL   UCSC 
 
  254
 
  4
 
 36958
 
 gene
 
 0.01960
 
 0.03197
 
 Not queried
 
  0.6416392
 
       NA
 
       NA
  
 
  
 2
 
  55459572
 
  55460177
 
  ENSEMBL   UCSC 
 
  606
 
 13
 
 22651
 
 gene;tss
 
 0.01984
 
 0.02416
 
 Not queried
 
  0.6416353
 
       NA
 
       NA
  
 
  
 7
 
    640338
 
    640603
 
  ENSEMBL   UCSC 
 
  266
 
  4
 
 36652
 
 gene;island
 
 0.90840
 
 0.95574
 
 Not queried
 
  0.6414659
 
       NA
 
       NA
  
 
  
 15
 
  60289239
 
  60289532
 
  ENSEMBL   UCSC 
 
  294
 
  4
 
 14159
 
 island
 
 0.02972
 
 0.04191
 
 Not queried
 
  0.6414602
 
       NA
 
       NA
  
 
  
 17
 
  65040527
 
  65040983
 
  ENSEMBL   UCSC 
 
  457
 
 10
 
 18643
 
 island
 
 0.80398
 
 0.87260
 
 Not queried
 
  0.6413822
 
       NA
 
       NA
  
 
  
 2
 
 152956034
 
 152956135
 
  ENSEMBL   UCSC 
 
  102
 
  4
 
 23625
 
 tss;island
 
 0.03020
 
 0.04698
 
 Not queried
 
  0.6413520
 
       NA
 
       NA
  
 
  
 14
 
  23298687
 
  23299085
 
  ENSEMBL   UCSC 
 
  399
 
  9
 
 12260
 
 tss
 
 0.01253
 
 0.02639
 
 Not queried
 
  0.6412371
 
       NA
 
       NA
  
 
  
 19
 
    633625
 
    633785
 
  ENSEMBL   UCSC 
 
  161
 
  8
 
 20238
 
 tss
 
 0.03664
 
 0.05263
 
 Not queried
 
  0.6411139
 
       NA
 
       NA
  
 
  
 19
 
  14800530
 
  14800772
 
  ENSEMBL   UCSC 
 
  243
 
  5
 
 20513
 
 tss;island
 
 0.01629
 
 0.01929
 
 Not queried
 
  0.6410762
 
       NA
 
       NA
  
 
  
 X
 
 105855176
 
 105855386
 
  ENSEMBL   UCSC 
 
  211
 
  4
 
 41069
 
 gene
 
 0.02175
 
 0.01375
 
 Not queried
 
  0.6409663
 
       NA
 
       NA
  
 
  
 5
 
  88179235
 
  88179539
 
  ENSEMBL   UCSC 
 
  305
 
  6
 
 31625
 
 gene;island
 
 0.01877
 
 0.02489
 
 Not queried
 
  0.6408835
 
       NA
 
       NA
  
 
  
 5
 
  88179379
 
  88179539
 
  ENSEMBL   UCSC 
 
  161
 
  4
 
 32110
 
 tss
 
 0.01516
 
 0.02355
 
 Not queried
 
  0.6408835
 
       NA
 
       NA
  
 
  
 15
 
  90294471
 
  90294776
 
  ENSEMBL   UCSC 
 
  306
 
  8
 
 14413
 
 island
 
 0.07294
 
 0.09464
 
 Not queried
 
  0.6408707
 
       NA
 
       NA
  
 
  
 4
 
 146018582
 
 146019130
 
  ENSEMBL   UCSC 
 
  549
 
 10
 
 30097
 
 gene;tss;island
 
 0.02015
 
 0.02832
 
 Not queried
 
  0.6408295
 
       NA
 
       NA
  
 
  
 17
 
  76227227
 
  76227386
 
  ENSEMBL   UCSC 
 
  160
 
  4
 
 17669
 
 tss
 
 0.64264
 
 0.56347
 
 Not queried
 
  0.6408290
 
       NA
 
       NA
  
 
  
 11
 
  76858648
 
  76859056
 
  ENSEMBL   UCSC 
 
  409
 
  6
 
 6821
 
 gene;island
 
 0.21955
 
 0.28651
 
 Not queried
 
  0.6408144
 
       NA
 
       NA
  
 
  
 4
 
  89378005
 
  89378224
 
  ENSEMBL   UCSC 
 
  220
 
  5
 
 30445
 
 tss
 
 0.01928
 
 0.03906
 
 Not queried
 
  0.6408115
 
       NA
 
       NA
  
 
  
 4
 
  89378005
 
  89378460
 
  ENSEMBL   UCSC 
 
  456
 
  7
 
 31121
 
 island
 
 0.01928
 
 0.02975
 
 Not queried
 
  0.6408115
 
       NA
 
       NA
  
 
  
 11
 
   1483731
 
   1483973
 
  ENSEMBL   UCSC 
 
  243
 
  4
 
 7905
 
 island
 
 0.87055
 
 0.90582
 
 Not queried
 
  0.6407698
 
       NA
 
       NA
  
 
  
 3
 
  29322342
 
  29322474
 
  ENSEMBL   UCSC 
 
  133
 
  4
 
 28157
 
 tss
 
 0.02288
 
 0.02198
 
 Not queried
 
  0.6407125
 
       NA
 
       NA
  
 
  
 12
 
 123717888
 
 123718415
 
  ENSEMBL   UCSC 
 
  528
 
  9
 
 9355
 
 gene
 
 0.01821
 
 0.02284
 
 Not queried
 
  0.6406122
 
       NA
 
       NA
  
 
  
 6
 
  26225258
 
  26225539
 
  ENSEMBL   UCSC 
 
  282
 
  5
 
 35465
 
 island
 
 0.20880
 
 0.08916
 
 Not queried
 
  0.6405995
 
       NA
 
       NA
  
 
  
 19
 
  49496473
 
  49496578
 
  ENSEMBL   UCSC 
 
  106
 
  4
 
 20105
 
 gene
 
 0.02492
 
 0.03140
 
 Not queried
 
  0.6405909
 
       NA
 
       NA
  
 
  
 18
 
  46064816
 
  46065292
 
  ENSEMBL   UCSC 
 
  477
 
  6
 
 19233
 
 tss;island
 
 0.07933
 
 0.04659
 
 Not queried
 
  0.6404966
 
       NA
 
       NA
  
 
  
 5
 
 132200490
 
 132200666
 
  ENSEMBL   UCSC 
 
  177
 
  4
 
 32220
 
 tss
 
 0.96270
 
 0.94587
 
 Not queried
 
  0.6404129
 
       NA
 
       NA
  
 
  
 13
 
  41345473
 
  41345689
 
  ENSEMBL   UCSC 
 
  217
 
  8
 
 11377
 
 tss
 
 0.01479
 
 0.02265
 
 Not queried
 
  0.6403799
 
       NA
 
       NA
  
 
  
 3
 
 136471376
 
 136471803
 
  ENSEMBL   UCSC 
 
  428
 
  5
 
 28570
 
 tss;island
 
 0.01714
 
 0.02498
 
 Not queried
 
  0.6403439
 
       NA
 
       NA
  
 
  
 4
 
  17615782
 
  17616265
 
  ENSEMBL   UCSC 
 
  484
 
 10
 
 30270
 
 tss
 
 0.01570
 
 0.02071
 
 Not queried
 
  0.6402141
 
       NA
 
       NA
  
 
  
 1
 
 109969051
 
 109969188
 
  ENSEMBL   UCSC 
 
  138
 
  7
 
 1800
 
 tss
 
 0.01378
 
 0.02337
 
 Not queried
 
  0.6401334
 
       NA
 
       NA
  
 
  
 5
 
 153825063
 
 153825431
 
  ENSEMBL   UCSC 
 
  369
 
  5
 
 32388
 
 tss
 
 0.03539
 
 0.03306
 
 Not queried
 
  0.6400286
 
       NA
 
       NA
  
 
  
 4
 
   3309966
 
   3310191
 
  ENSEMBL   UCSC 
 
  226
 
  4
 
 30787
 
 island
 
 0.93922
 
 0.96075
 
 Not queried
 
  0.6399518
 
       NA
 
       NA
  
 
  
 22
 
  24372913
 
  24373054
 
  ENSEMBL   UCSC 
 
  142
 
  5
 
 26974
 
 tss
 
 0.01685
 
 0.03472
 
 Not queried
 
  0.6399359
 
       NA
 
       NA
  
 
  
 8
 
 142368026
 
 142368332
 
  ENSEMBL   UCSC 
 
  307
 
  6
 
 39389
 
 gene;island
 
 0.77008
 
 0.84158
 
 Not queried
 
  0.6399159
 
       NA
 
       NA
  
 
  
 13
 
 115000149
 
 115000275
 
  ENSEMBL   UCSC 
 
  127
 
  8
 
 11529
 
 tss
 
 0.02235
 
 0.02870
 
 Not queried
 
  0.6398447
 
       NA
 
       NA
  
 
  
 4
 
 113152573
 
 113152877
 
  ENSEMBL   UCSC 
 
  305
 
  7
 
 30503
 
 tss;island
 
 0.01665
 
 0.02504
 
 Not queried
 
  0.6398418
 
       NA
 
       NA
  
 
  
 6
 
  32184829
 
  32185340
 
  ENSEMBL   UCSC 
 
  512
 
  9
 
 34034
 
 gene
 
 0.64672
 
 0.60917
 
 Not queried
 
  0.6397585
 
       NA
 
       NA
  
 
  
 17
 
  77984086
 
  77984481
 
  ENSEMBL   UCSC 
 
  396
 
  5
 
 16815
 
 gene;island
 
 0.82417
 
 0.80654
 
 Not queried
 
  0.6396700
 
       NA
 
       NA
  
 
  
 6
 
  33175613
 
  33176267
 
  ENSEMBL   UCSC 
 
  655
 
 11
 
 34832
 
 tss
 
 0.02985
 
 0.02263
 
 Not queried
 
  0.6396154
 
       NA
 
       NA
  
 
  
 2
 
  97405364
 
  97405809
 
  ENSEMBL   UCSC 
 
  446
 
  5
 
 22748
 
 gene
 
 0.02708
 
 0.04955
 
 Not queried
 
  0.6395084
 
       NA
 
       NA
  
 
  
 16
 
  30937154
 
  30937214
 
  ENSEMBL   UCSC 
 
   61
 
  4
 
 14696
 
 gene;island
 
 0.83906
 
 0.91111
 
 Not queried
 
  0.6394789
 
       NA
 
       NA
  
 
  
 12
 
  57588031
 
  57588243
 
  ENSEMBL   UCSC 
 
  213
 
  7
 
 9786
 
 tss
 
 0.88000
 
 0.84016
 
 Not queried
 
  0.6394508
 
       NA
 
       NA
  
 
  
 6
 
  52529024
 
  52529191
 
  ENSEMBL   UCSC 
 
  168
 
  6
 
 34970
 
 tss
 
 0.01429
 
 0.02061
 
 Not queried
 
  0.6394460
 
       NA
 
       NA
  
 
  
 6
 
  74019562
 
  74020018
 
  ENSEMBL   UCSC 
 
  457
 
  5
 
 34322
 
 gene
 
 0.03388
 
 0.01779
 
 Not queried
 
  0.6394224
 
       NA
 
       NA
  
 
  
 10
 
 131812411
 
 131812878
 
  ENSEMBL   UCSC 
 
  468
 
  6
 
 6250
 
 island
 
 0.79275
 
 0.87313
 
 Not queried
 
  0.6394103
 
       NA
 
       NA
  
 
  
 11
 
  64135650
 
  64136263
 
  ENSEMBL   UCSC 
 
  614
 
 11
 
 6669
 
 gene;island
 
 0.83889
 
 0.89346
 
 Not queried
 
  0.6393780
 
       NA
 
       NA
  
 
  
 11
 
  64135650
 
  64136024
 
  ENSEMBL   UCSC 
 
  375
 
  9
 
 7378
 
 tss
 
 0.84290
 
 0.91624
 
 Not queried
 
  0.6393780
 
       NA
 
       NA
  
 
  
 14
 
  97208114
 
  97208333
 
  ENSEMBL   UCSC 
 
  220
 
  4
 
 13091
 
 island
 
 0.36003
 
 0.13037
 
 Not queried
 
  0.6393652
 
       NA
 
       NA
  
 
  
 10
 
  13141779
 
  13141924
 
  ENSEMBL   UCSC 
 
  146
 
  6
 
 4938
 
 tss;island
 
 0.02783
 
 0.03283
 
 Not queried
 
  0.6391449
 
       NA
 
       NA
  
 
  
 19
 
   5455176
 
   5455638
 
  ENSEMBL   UCSC 
 
  463
 
  8
 
 21368
 
 island
 
 0.92349
 
 0.84564
 
 Not queried
 
  0.6391206
 
       NA
 
       NA
  
 
  
 19
 
  55996329
 
  55996652
 
  ENSEMBL   UCSC 
 
  324
 
  8
 
 22392
 
 island
 
 0.03182
 
 0.03779
 
 Not queried
 
  0.6390451
 
       NA
 
       NA
  
 
  
 X
 
 129039711
 
 129039863
 
  ENSEMBL   UCSC 
 
  153
 
  4
 
 41513
 
 tss;island
 
 0.01433
 
 0.02100
 
 Not queried
 
  0.6389702
 
       NA
 
       NA
  
 
  
 16
 
   2021690
 
   2021951
 
  ENSEMBL   UCSC 
 
  262
 
  8
 
 14982
 
 tss
 
 0.02994
 
 0.03366
 
 Not queried
 
  0.6389041
 
       NA
 
       NA
  
 
  
 1
 
   1272398
 
   1272559
 
  ENSEMBL   UCSC 
 
  162
 
  4
 
 32
 
 gene;island
 
 0.94316
 
 0.90506
 
 Not queried
 
  0.6386840
 
       NA
 
       NA
  
 
  
 2
 
 200323017
 
 200323432
 
  ENSEMBL   UCSC 
 
  416
 
  5
 
 22937
 
 gene;island
 
 0.01900
 
 0.02927
 
 Not queried
 
  0.6386156
 
       NA
 
       NA
  
 
  
 16
 
   2653222
 
   2653306
 
  ENSEMBL   UCSC 
 
   85
 
  7
 
 15001
 
 tss
 
 0.05923
 
 0.02500
 
 Not queried
 
  0.6385551
 
       NA
 
       NA
  
 
  
 6
 
  33172442
 
  33172572
 
  ENSEMBL   UCSC 
 
  131
 
  6
 
 34162
 
 gene
 
 0.03330
 
 0.03867
 
 Not queried
 
  0.6384353
 
       NA
 
       NA
  
 
  
 11
 
  60738971
 
  60739019
 
  ENSEMBL   UCSC 
 
   49
 
  4
 
 7306
 
 tss
 
 0.72617
 
 0.83748
 
 Not queried
 
  0.6383893
 
       NA
 
       NA
  
 
  
 12
 
 110337835
 
 110338662
 
  ENSEMBL   UCSC 
 
  828
 
 15
 
 10761
 
 island
 
 0.01758
 
 0.02710
 
 Not queried
 
  0.6381434
 
       NA
 
       NA
  
 
  
 6
 
 170101828
 
 170102133
 
  ENSEMBL   UCSC 
 
  306
 
  5
 
 34510
 
 gene
 
 0.01996
 
 0.01291
 
 Not queried
 
  0.6381334
 
       NA
 
       NA
  
 
  
 16
 
  57570661
 
  57570819
 
  ENSEMBL   UCSC 
 
  159
 
  4
 
 15234
 
 tss;island
 
 0.02195
 
 0.02621
 
 Not queried
 
  0.6381216
 
       NA
 
       NA
  
 
  
 11
 
 124805912
 
 124806173
 
  ENSEMBL   UCSC 
 
  262
 
  4
 
 6936
 
 gene
 
 0.57350
 
 0.43142
 
 Not queried
 
  0.6380331
 
       NA
 
       NA
  
 
  
 13
 
 102068234
 
 102068650
 
  ENSEMBL   UCSC 
 
  417
 
  5
 
 11184
 
 gene;island
 
 0.07778
 
 0.04031
 
 Not queried
 
  0.6380273
 
       NA
 
       NA
  
 
  
 X
 
  38660266
 
  38660704
 
  ENSEMBL   UCSC 
 
  439
 
  9
 
 41722
 
 island
 
 0.06393
 
 0.04265
 
 Not queried
 
  0.6379969
 
       NA
 
       NA
  
 
  
 8
 
 145026305
 
 145026613
 
  ENSEMBL   UCSC 
 
  309
 
  4
 
 39440
 
 gene;island
 
 0.03174
 
 0.03878
 
 Not queried
 
  0.6379921
 
       NA
 
       NA
  
 
  
 11
 
 121322456
 
 121322712
 
  ENSEMBL   UCSC 
 
  257
 
  4
 
 7746
 
 tss;island
 
 0.02019
 
 0.01980
 
 Not queried
 
  0.6379781
 
       NA
 
       NA
  
 
  
 8
 
  70404514
 
  70404928
 
  ENSEMBL   UCSC 
 
  415
 
  7
 
 39266
 
 gene;tss
 
 0.20972
 
 0.10820
 
 Not queried
 
  0.6379649
 
       NA
 
       NA
  
 
  
 3
 
 101546486
 
 101546859
 
  ENSEMBL   UCSC 
 
  374
 
  6
 
 27841
 
 gene
 
 0.04090
 
 0.06754
 
 Not queried
 
  0.6378996
 
       NA
 
       NA
  
 
  
 7
 
  27146237
 
  27146445
 
  ENSEMBL   UCSC 
 
  209
 
  4
 
 36866
 
 gene;island
 
 0.01743
 
 0.01958
 
 Not queried
 
  0.6377291
 
       NA
 
       NA
  
 
  
 14
 
  99947303
 
  99947768
 
  ENSEMBL   UCSC 
 
  466
 
  9
 
 12570
 
 tss;island
 
 0.02478
 
 0.03670
 
 Not queried
 
  0.6377097
 
       NA
 
       NA
  
 
  
 19
 
  49149549
 
  49149933
 
  ENSEMBL   UCSC 
 
  385
 
  5
 
 20098
 
 gene;tss
 
 0.02217
 
 0.02115
 
 Not queried
 
  0.6376272
 
       NA
 
       NA
  
 
  
 18
 
  61034324
 
  61034655
 
  ENSEMBL   UCSC 
 
  332
 
  6
 
 19520
 
 island
 
 0.02401
 
 0.02184
 
 Not queried
 
  0.6374608
 
       NA
 
       NA
  
 
  
 4
 
  53578129
 
  53578664
 
  ENSEMBL   UCSC 
 
  536
 
 11
 
 30345
 
 tss;island
 
 0.02236
 
 0.03174
 
 Not queried
 
  0.6372970
 
       NA
 
       NA
  
 
  
 17
 
  40075284
 
  40075472
 
  ENSEMBL   UCSC 
 
  189
 
  6
 
 17315
 
 tss
 
 0.01534
 
 0.02329
 
 Not queried
 
  0.6372685
 
       NA
 
       NA
  
 
  
 1
 
  47779610
 
  47780210
 
  ENSEMBL   UCSC 
 
  601
 
 13
 
 3227
 
 island
 
 0.02120
 
 0.03724
 
 Not queried
 
  0.6372519
 
       NA
 
       NA
  
 
  
 15
 
  63796130
 
  63796908
 
  ENSEMBL   UCSC 
 
  779
 
 12
 
 14180
 
 island
 
 0.02097
 
 0.03008
 
 Not queried
 
  0.6372271
 
       NA
 
       NA
  
 
  
 11
 
  16760157
 
  16760677
 
  ENSEMBL   UCSC 
 
  521
 
  7
 
 6504
 
 gene
 
 0.02522
 
 0.04719
 
 Not queried
 
  0.6372226
 
       NA
 
       NA
  
 
  
 11
 
 123525662
 
 123525714
 
  ENSEMBL   UCSC 
 
   53
 
  4
 
 7757
 
 tss;island
 
 0.02653
 
 0.05205
 
 Not queried
 
  0.6371006
 
       NA
 
       NA
  
 
  
 12
 
 132698353
 
 132698786
 
  ENSEMBL   UCSC 
 
  434
 
  4
 
 9409
 
 gene;island
 
 0.71929
 
 0.77571
 
 Not queried
 
  0.6370673
 
       NA
 
       NA
  
 
  
 6
 
   5994956
 
   5995150
 
  ENSEMBL   UCSC 
 
  195
 
  4
 
 35338
 
 island
 
 0.74356
 
 0.65855
 
 Not queried
 
  0.6369963
 
       NA
 
       NA
  
 
  
 19
 
  46417551
 
  46417734
 
  ENSEMBL   UCSC 
 
  184
 
  4
 
 20071
 
 gene;island
 
 0.85055
 
 0.82460
 
 Not queried
 
  0.6369715
 
       NA
 
       NA
  
 
  
 17
 
  21496125
 
  21496299
 
  ENSEMBL   UCSC 
 
  175
 
  4
 
 18094
 
 island
 
 0.78922
 
 0.76778
 
 Not queried
 
  0.6368555
 
       NA
 
       NA
  
 
  
 8
 
  71581601
 
  71581788
 
  ENSEMBL   UCSC 
 
  188
 
  6
 
 39271
 
 gene
 
 0.44002
 
 0.15362
 
 Not queried
 
  0.6368290
 
       NA
 
       NA
  
 
  
 17
 
  76719591
 
  76719640
 
  ENSEMBL   UCSC 
 
   50
 
  4
 
 16789
 
 gene
 
 0.05794
 
 0.11384
 
 Not queried
 
  0.6368094
 
       NA
 
       NA
  
 
  
 11
 
  70244230
 
  70244470
 
  ENSEMBL   UCSC 
 
  241
 
  5
 
 7507
 
 tss;island
 
 0.01358
 
 0.01248
 
 Not queried
 
  0.6367570
 
       NA
 
       NA
  
 
  
 14
 
 101495604
 
 101495910
 
  ENSEMBL   UCSC 
 
  307
 
  6
 
 12594
 
 tss
 
 0.88068
 
 0.91438
 
 Not queried
 
  0.6367463
 
       NA
 
       NA
  
 
  
 1
 
  38478417
 
  38478702
 
  ENSEMBL   UCSC 
 
  286
 
  6
 
 441
 
 gene
 
 0.03676
 
 0.05544
 
 Not queried
 
  0.6367440
 
       NA
 
       NA
  
 
  
 11
 
    976950
 
    977280
 
  ENSEMBL   UCSC 
 
  331
 
  4
 
 6380
 
 gene;island
 
 0.83435
 
 0.88184
 
 Not queried
 
  0.6367174
 
       NA
 
       NA
  
 
  
 3
 
 137905836
 
 137906401
 
  ENSEMBL   UCSC 
 
  566
 
 10
 
 29475
 
 island
 
 0.01554
 
 0.02195
 
 Not queried
 
  0.6366741
 
       NA
 
       NA
  
 
  
 15
 
  66161519
 
  66162152
 
  ENSEMBL   UCSC 
 
  634
 
 11
 
 14210
 
 island
 
 0.01422
 
 0.02693
 
 Not queried
 
  0.6365836
 
       NA
 
       NA
  
 
  
 17
 
  60501257
 
  60501650
 
  ENSEMBL   UCSC 
 
  394
 
  4
 
 16687
 
 gene
 
 0.01541
 
 0.02481
 
 Not queried
 
  0.6365814
 
       NA
 
       NA
  
 
  
 3
 
  37034840
 
  37035399
 
  ENSEMBL   UCSC 
 
  560
 
 19
 
 27675
 
 gene
 
 0.03789
 
 0.02386
 
 Not queried
 
  0.6364519
 
       NA
 
       NA
  
 
  
 20
 
  21000809
 
  21001266
 
  ENSEMBL   UCSC 
 
  458
 
  4
 
 25997
 
 island
 
 0.05186
 
 0.10663
 
 Not queried
 
  0.6360949
 
       NA
 
       NA
  
 
  
 12
 
  89747417
 
  89747739
 
  ENSEMBL   UCSC 
 
  323
 
  4
 
 9882
 
 tss;island
 
 0.07907
 
 0.07995
 
 Not queried
 
  0.6360869
 
       NA
 
       NA
  
 
  
 7
 
   5271480
 
   5271655
 
  ENSEMBL   UCSC 
 
  176
 
  4
 
 36805
 
 gene;island
 
 0.90587
 
 0.93329
 
 Not queried
 
  0.6360174
 
       NA
 
       NA
  
 
  
 12
 
 129279936
 
 129280449
 
  ENSEMBL   UCSC 
 
  514
 
  6
 
 9383
 
 gene;island
 
 0.84527
 
 0.92127
 
 Not queried
 
  0.6359622
 
       NA
 
       NA
  
 
  
 19
 
  54210147
 
  54210518
 
  ENSEMBL   UCSC 
 
  372
 
  4
 
 20999
 
 tss
 
 0.83849
 
 0.78217
 
 Not queried
 
  0.6359379
 
       NA
 
       NA
  
 
  
 4
 
 146100596
 
 146101151
 
  ENSEMBL   UCSC 
 
  556
 
  7
 
 31288
 
 island
 
 0.01238
 
 0.02007
 
 Not queried
 
  0.6359231
 
       NA
 
       NA
  
 
  
 13
 
 100258524
 
 100258760
 
  ENSEMBL   UCSC 
 
  237
 
  5
 
 11486
 
 tss
 
 0.04234
 
 0.02579
 
 Not queried
 
  0.6359072
 
       NA
 
       NA
  
 
  
 6
 
  41215806
 
  41216090
 
  ENSEMBL   UCSC 
 
  285
 
  4
 
 34904
 
 tss
 
 0.41922
 
 0.31908
 
 Not queried
 
  0.6358940
 
       NA
 
       NA
  
 
  
 16
 
  69458177
 
  69458483
 
  ENSEMBL   UCSC 
 
  307
 
  7
 
 15302
 
 tss
 
 0.01806
 
 0.03930
 
 Not queried
 
  0.6355829
 
       NA
 
       NA
  
 
  
 9
 
  98784231
 
  98784441
 
  ENSEMBL   UCSC 
 
  211
 
  4
 
 40759
 
 tss;island
 
 0.06305
 
 0.06020
 
 Not queried
 
  0.6355237
 
       NA
 
       NA
  
 
  
 13
 
  34391699
 
  34392163
 
  ENSEMBL   UCSC 
 
  465
 
  8
 
 11360
 
 tss
 
 0.04271
 
 0.03157
 
 Not queried
 
  0.6352376
 
       NA
 
       NA
  
 
  
 18
 
  42530692
 
  42531132
 
  ENSEMBL   UCSC 
 
  441
 
  8
 
 19063
 
 gene
 
 0.90746
 
 0.92177
 
 Not queried
 
  0.6352236
 
       NA
 
       NA
  
 
  
 6
 
  30644681
 
  30644832
 
  ENSEMBL   UCSC 
 
  152
 
  4
 
 33736
 
 gene;island
 
 0.79715
 
 0.87739
 
 Not queried
 
  0.6350536
 
       NA
 
       NA
  
 
  
 10
 
  81838185
 
  81839181
 
  ENSEMBL   UCSC 
 
  997
 
 17
 
 4639
 
 gene;island
 
 0.02659
 
 0.03157
 
 Not queried
 
  0.6350134
 
       NA
 
       NA
  
 
  
 19
 
   6459960
 
   6460274
 
  ENSEMBL   UCSC 
 
  315
 
  6
 
 20353
 
 tss;island
 
 0.01603
 
 0.02243
 
 Not queried
 
  0.6348953
 
       NA
 
       NA
  
 
  
 14
 
  20937107
 
  20937494
 
  ENSEMBL   UCSC 
 
  388
 
  6
 
 12246
 
 tss
 
 0.07874
 
 0.08047
 
 Not queried
 
  0.6347188
 
       NA
 
       NA
  
 
  
 6
 
  37787045
 
  37787209
 
  ENSEMBL   UCSC 
 
  165
 
  4
 
 34885
 
 tss;island
 
 0.02510
 
 0.04459
 
 Not queried
 
  0.6346904
 
       NA
 
       NA
  
 
  
 1
 
  21022984
 
  21023254
 
  ENSEMBL   UCSC 
 
  271
 
  4
 
 291
 
 gene;island
 
 0.01483
 
 0.01326
 
 Not queried
 
  0.6346288
 
       NA
 
       NA
  
 
  
 19
 
  41935153
 
  41935467
 
  ENSEMBL   UCSC 
 
  315
 
  4
 
 20775
 
 tss
 
 0.90927
 
 0.94110
 
 Not queried
 
  0.6345203
 
       NA
 
       NA
  
 
  
 2
 
 217236425
 
 217236650
 
  ENSEMBL   UCSC 
 
  226
 
  4
 
 22973
 
 gene
 
 0.03265
 
 0.07771
 
 Not queried
 
  0.6345152
 
       NA
 
       NA
  
 
  
 14
 
  73493715
 
  73494282
 
  ENSEMBL   UCSC 
 
  568
 
 12
 
 12954
 
 island
 
 0.01914
 
 0.03057
 
 Not queried
 
  0.6345043
 
       NA
 
       NA
  
 
  
 7
 
   1032865
 
   1033176
 
  ENSEMBL   UCSC 
 
  312
 
  4
 
 38000
 
 island
 
 0.05094
 
 0.11169
 
 Not queried
 
  0.6344914
 
       NA
 
       NA
  
 
  
 16
 
  30124803
 
  30125403
 
  ENSEMBL   UCSC 
 
  601
 
  8
 
 15149
 
 tss
 
 0.79487
 
 0.85107
 
 Not queried
 
  0.6344803
 
       NA
 
       NA
  
 
  
 6
 
 100016130
 
 100016838
 
  ENSEMBL   UCSC 
 
  709
 
 10
 
 36293
 
 island
 
 0.02808
 
 0.04696
 
 Not queried
 
  0.6344667
 
       NA
 
       NA
  
 
  
 1
 
 120612147
 
 120612404
 
  ENSEMBL   UCSC 
 
  258
 
  4
 
 3655
 
 island
 
 0.02386
 
 0.03662
 
 Not queried
 
  0.6344586
 
       NA
 
       NA
  
 
  
 6
 
 117002099
 
 117002360
 
  ENSEMBL   UCSC 
 
  262
 
  8
 
 35128
 
 tss
 
 0.01367
 
 0.01820
 
 Not queried
 
  0.6343354
 
       NA
 
       NA
  
 
  
 5
 
 112824614
 
 112824920
 
  ENSEMBL   UCSC 
 
  307
 
  6
 
 32160
 
 tss
 
 0.54039
 
 0.62852
 
 Not queried
 
  0.6342478
 
       NA
 
       NA
  
 
  
 10
 
  52499747
 
  52500187
 
  ENSEMBL   UCSC 
 
  441
 
  5
 
 4574
 
 gene
 
 0.00972
 
 0.03183
 
 Not queried
 
  0.6342319
 
       NA
 
       NA
  
 
  
 10
 
  52499393
 
  52500187
 
  ENSEMBL   UCSC 
 
  795
 
  9
 
 5726
 
 island
 
 0.00972
 
 0.02718
 
 Not queried
 
  0.6342319
 
       NA
 
       NA
  
 
  
 19
 
  11565385
 
  11565683
 
  ENSEMBL   UCSC 
 
  299
 
  6
 
 19807
 
 gene;island
 
 0.83593
 
 0.87391
 
 Not queried
 
  0.6341752
 
       NA
 
       NA
  
 
  
 3
 
  21792857
 
  21792991
 
  ENSEMBL   UCSC 
 
  135
 
  4
 
 28140
 
 tss
 
 0.03022
 
 0.02101
 
 Not queried
 
  0.6341578
 
       NA
 
       NA
  
 
  
 17
 
  75136725
 
  75136989
 
  ENSEMBL   UCSC 
 
  265
 
  7
 
 17661
 
 tss
 
 0.02359
 
 0.05251
 
 Not queried
 
  0.6341543
 
       NA
 
       NA
  
 
  
 17
 
  75136725
 
  75137144
 
  ENSEMBL   UCSC 
 
  420
 
  9
 
 18767
 
 island
 
 0.02345
 
 0.04024
 
 Not queried
 
  0.6341543
 
       NA
 
       NA
  
 
  
 8
 
  88884985
 
  88885254
 
  ENSEMBL   UCSC 
 
  270
 
  4
 
 39292
 
 gene;island
 
 0.74055
 
 0.86421
 
 Not queried
 
  0.6340183
 
       NA
 
       NA
  
 
  
 1
 
  71546313
 
  71547110
 
  ENSEMBL   UCSC 
 
  798
 
 10
 
 3376
 
 island
 
 0.02268
 
 0.02562
 
 Not queried
 
  0.6338752
 
       NA
 
       NA
  
 
  
 17
 
  66243584
 
  66243669
 
  ENSEMBL   UCSC 
 
   86
 
  4
 
 17571
 
 tss;island
 
 0.03624
 
 0.04852
 
 Not queried
 
  0.6338219
 
       NA
 
       NA
  
 
  
 6
 
  30303537
 
  30303902
 
  ENSEMBL   UCSC 
 
  366
 
 12
 
 33701
 
 gene
 
 0.78489
 
 0.86830
 
 Not queried
 
  0.6337991
 
       NA
 
       NA
  
 
  
 12
 
  93963179
 
  93963555
 
  ENSEMBL   UCSC 
 
  377
 
 10
 
 9891
 
 tss
 
 0.01531
 
 0.03131
 
 Not queried
 
  0.6337398
 
       NA
 
       NA
  
 
  
 12
 
  93963179
 
  93963603
 
  ENSEMBL   UCSC 
 
  425
 
 12
 
 10648
 
 island
 
 0.01791
 
 0.03131
 
 Not queried
 
  0.6337398
 
       NA
 
       NA
  
 
  
 16
 
  31085507
 
  31085762
 
  ENSEMBL   UCSC 
 
  256
 
  8
 
 15174
 
 tss;island
 
 0.01846
 
 0.02527
 
 Not queried
 
  0.6336986
 
       NA
 
       NA
  
 
  
 7
 
 150755072
 
 150755981
 
  ENSEMBL   UCSC 
 
  910
 
 20
 
 37923
 
 tss
 
 0.04966
 
 0.02774
 
 Not queried
 
  0.6336982
 
       NA
 
       NA
  
 
  
 1
 
  22213801
 
  22214106
 
  ENSEMBL   UCSC 
 
  306
 
  5
 
 303
 
 gene;island
 
 0.90036
 
 0.83171
 
 Not queried
 
  0.6336945
 
       NA
 
       NA
  
 
  
 6
 
  31629900
 
  31630923
 
  ENSEMBL   UCSC 
 
 1024
 
 10
 
 33867
 
 gene;island
 
 0.89786
 
 0.92826
 
 Not queried
 
  0.6336917
 
       NA
 
       NA
  
 
  
 10
 
   1120434
 
   1120831
 
  ENSEMBL   UCSC 
 
  398
 
  4
 
 4440
 
 gene
 
 0.76857
 
 0.91848
 
 Not queried
 
  0.6335499
 
       NA
 
       NA
  
 
  
 15
 
  25684203
 
  25684849
 
  ENSEMBL   UCSC 
 
  647
 
 13
 
 13508
 
 tss
 
 0.04260
 
 0.03486
 
 Not queried
 
  0.6333640
 
       NA
 
       NA
  
 
  
 6
 
 100062488
 
 100062855
 
  ENSEMBL   UCSC 
 
  368
 
  5
 
 34365
 
 gene;island
 
 0.03464
 
 0.04644
 
 Not queried
 
  0.6333598
 
       NA
 
       NA
  
 
  
 1
 
  35496792
 
  35497655
 
  ENSEMBL   UCSC 
 
  864
 
  8
 
 3026
 
 island
 
 0.02665
 
 0.03651
 
 Not queried
 
  0.6333372
 
       NA
 
       NA
  
 
  
 1
 
  32670916
 
  32671135
 
  ENSEMBL   UCSC 
 
  220
 
  7
 
 1451
 
 tss
 
 0.01899
 
 0.05024
 
 Not queried
 
  0.6332124
 
       NA
 
       NA
  
 
  
 13
 
  84456127
 
  84456722
 
  ENSEMBL   UCSC 
 
  596
 
  9
 
 11777
 
 island
 
 0.02686
 
 0.05207
 
 Not queried
 
  0.6331521
 
       NA
 
       NA
  
 
  
 15
 
  52821013
 
  52821541
 
  ENSEMBL   UCSC 
 
  529
 
  8
 
 14122
 
 island
 
 0.03576
 
 0.05420
 
 Not queried
 
  0.6331491
 
       NA
 
       NA
  
 
  
 4
 
  20702045
 
  20702377
 
  ENSEMBL   UCSC 
 
  333
 
  4
 
 29919
 
 gene
 
 0.01217
 
 0.01343
 
 Not queried
 
  0.6330735
 
       NA
 
       NA
  
 
  
 10
 
 134491205
 
 134491593
 
  ENSEMBL   UCSC 
 
  389
 
  5
 
 4855
 
 gene
 
 0.85655
 
 0.90253
 
 Not queried
 
  0.6329457
 
       NA
 
       NA
  
 
  
 1
 
 154909529
 
 154909991
 
  ENSEMBL   UCSC 
 
  463
 
  8
 
 1966
 
 tss
 
 0.02196
 
 0.03310
 
 Not queried
 
  0.6328293
 
       NA
 
       NA
  
 
  
 19
 
   2476077
 
   2476226
 
  ENSEMBL   UCSC 
 
  150
 
  4
 
 21251
 
 island
 
 0.00989
 
 0.01435
 
 Not queried
 
  0.6327056
 
       NA
 
       NA
  
 
  
 2
 
 201983199
 
 201983452
 
  ENSEMBL   UCSC 
 
  254
 
  8
 
 22950
 
 gene;island
 
 0.00926
 
 0.01568
 
 Not queried
 
  0.6326882
 
       NA
 
       NA
  
 
  
 2
 
 201983199
 
 201983303
 
  ENSEMBL   UCSC 
 
  105
 
  6
 
 23777
 
 tss
 
 0.00926
 
 0.01568
 
 Not queried
 
  0.6326882
 
       NA
 
       NA
  
 
  
 11
 
  47290613
 
  47291501
 
  ENSEMBL   UCSC 
 
  889
 
 14
 
 7265
 
 tss;island
 
 0.04240
 
 0.05839
 
 Not queried
 
  0.6326578
 
       NA
 
       NA
  
 
  
 7
 
 131241256
 
 131241396
 
  ENSEMBL   UCSC 
 
  141
 
  4
 
 38866
 
 island
 
 0.02550
 
 0.03439
 
 Not queried
 
  0.6326527
 
       NA
 
       NA
  
 
  
 7
 
   3019159
 
   3019382
 
  ENSEMBL   UCSC 
 
  224
 
  4
 
 36759
 
 gene
 
 0.74901
 
 0.85146
 
 Not queried
 
  0.6326314
 
       NA
 
       NA
  
 
  
 20
 
  43374239
 
  43374406
 
  ENSEMBL   UCSC 
 
  168
 
  8
 
 25724
 
 tss;island
 
 0.21527
 
 0.16853
 
 Not queried
 
  0.6325974
 
       NA
 
       NA
  
 
  
 19
 
  10381577
 
  10382048
 
  ENSEMBL   UCSC 
 
  472
 
  4
 
 19775
 
 gene
 
 0.05123
 
 0.03532
 
 Not queried
 
  0.6325279
 
       NA
 
       NA
  
 
  
 21
 
  34602557
 
  34602948
 
  ENSEMBL   UCSC 
 
  392
 
  5
 
 26362
 
 gene;island
 
 0.03166
 
 0.04070
 
 Not queried
 
  0.6324976
 
       NA
 
       NA
  
 
  
 6
 
  57036894
 
  57037099
 
  ENSEMBL   UCSC 
 
  206
 
  5
 
 34985
 
 tss
 
 0.01380
 
 0.02123
 
 Not queried
 
  0.6324845
 
       NA
 
       NA
  
 
  
 6
 
  69344406
 
  69344702
 
  ENSEMBL   UCSC 
 
  297
 
  5
 
 34991
 
 tss;island
 
 0.06927
 
 0.06024
 
 Not queried
 
  0.6324673
 
       NA
 
       NA
  
 
  
 1
 
  77684965
 
  77685094
 
  ENSEMBL   UCSC 
 
  130
 
  4
 
 592
 
 gene
 
 0.02722
 
 0.03427
 
 Not queried
 
  0.6323742
 
       NA
 
       NA
  
 
  
 1
 
  77684965
 
  77685251
 
  ENSEMBL   UCSC 
 
  287
 
  8
 
 3388
 
 island
 
 0.02081
 
 0.03411
 
 Not queried
 
  0.6323742
 
       NA
 
       NA
  
 
  
 18
 
  77676008
 
  77676321
 
  ENSEMBL   UCSC 
 
  314
 
  4
 
 19112
 
 gene;island
 
 0.92729
 
 0.94014
 
 Not queried
 
  0.6323621
 
       NA
 
       NA
  
 
  
 2
 
  69664772
 
  69664869
 
  ENSEMBL   UCSC 
 
   98
 
  5
 
 23368
 
 tss
 
 0.02237
 
 0.01392
 
 Not queried
 
  0.6323131
 
       NA
 
       NA
  
 
  
 X
 
  10124797
 
  10124977
 
  ENSEMBL   UCSC 
 
  181
 
  4
 
 41180
 
 tss
 
 0.01529
 
 0.02613
 
 Not queried
 
  0.6322744
 
       NA
 
       NA
  
 
  
 2
 
 136742688
 
 136743046
 
  ENSEMBL   UCSC 
 
  359
 
  4
 
 22848
 
 gene;island
 
 0.01556
 
 0.02819
 
 Not queried
 
  0.6322679
 
       NA
 
       NA
  
 
  
 3
 
  58291814
 
  58291905
 
  ENSEMBL   UCSC 
 
   92
 
  5
 
 28372
 
 tss
 
 0.02579
 
 0.06309
 
 Not queried
 
  0.6322415
 
       NA
 
       NA
  
 
  
 15
 
  76552451
 
  76552771
 
  ENSEMBL   UCSC 
 
  321
 
  5
 
 13405
 
 gene
 
 0.88570
 
 0.77165
 
 Not queried
 
  0.6322024
 
       NA
 
       NA
  
 
  
 X
 
   9431111
 
   9431326
 
  ENSEMBL   UCSC 
 
  216
 
  6
 
 41177
 
 tss;island
 
 0.02175
 
 0.02965
 
 Not queried
 
  0.6321993
 
       NA
 
       NA
  
 
  
 14
 
  90722653
 
  90722861
 
  ENSEMBL   UCSC 
 
  209
 
  5
 
 12525
 
 tss
 
 0.06889
 
 0.06322
 
 Not queried
 
  0.6321628
 
       NA
 
       NA
  
 
  
 19
 
  58220295
 
  58220837
 
  ENSEMBL   UCSC 
 
  543
 
 10
 
 22443
 
 island
 
 0.02209
 
 0.01951
 
 Not queried
 
  0.6321618
 
       NA
 
       NA
  
 
  
 20
 
  61885850
 
  61886313
 
  ENSEMBL   UCSC 
 
  464
 
  7
 
 25461
 
 gene;island
 
 0.05530
 
 0.05375
 
 Not queried
 
  0.6321606
 
       NA
 
       NA
  
 
  
 19
 
  56136057
 
  56136215
 
  ENSEMBL   UCSC 
 
  159
 
  7
 
 21048
 
 tss
 
 0.02786
 
 0.04253
 
 Not queried
 
  0.6320421
 
       NA
 
       NA
  
 
  
 6
 
   7590278
 
   7590591
 
  ENSEMBL   UCSC 
 
  314
 
  6
 
 35351
 
 island
 
 0.01922
 
 0.01815
 
 Not queried
 
  0.6320205
 
       NA
 
       NA
  
 
  
 17
 
   8151011
 
   8151491
 
  ENSEMBL   UCSC 
 
  481
 
  5
 
 17965
 
 island
 
 0.00993
 
 0.02191
 
 Not queried
 
  0.6319455
 
       NA
 
       NA
  
 
  
 22
 
  28314999
 
  28315683
 
  ENSEMBL   UCSC 
 
  685
 
  8
 
 26997
 
 tss;island
 
 0.01806
 
 0.04862
 
 Not queried
 
  0.6319089
 
       NA
 
       NA
  
 
  
 14
 
 101498206
 
 101498317
 
  ENSEMBL   UCSC 
 
  112
 
  5
 
 12595
 
 tss
 
 0.86068
 
 0.90542
 
 Not queried
 
  0.6319085
 
       NA
 
       NA
  
 
  
 10
 
 134540246
 
 134540624
 
  ENSEMBL   UCSC 
 
  379
 
  4
 
 4861
 
 gene;island
 
 0.93817
 
 0.96071
 
 Not queried
 
  0.6319066
 
       NA
 
       NA
  
 
  
 6
 
 117002099
 
 117002400
 
  ENSEMBL   UCSC 
 
  302
 
  9
 
 36370
 
 island
 
 0.01212
 
 0.01774
 
 Not queried
 
  0.6319011
 
       NA
 
       NA
  
 
  
 1
 
 228296932
 
 228297275
 
  ENSEMBL   UCSC 
 
  344
 
  8
 
 4265
 
 island
 
 0.01979
 
 0.03361
 
 Not queried
 
  0.6318116
 
       NA
 
       NA
  
 
  
 17
 
  16120399
 
  16120465
 
  ENSEMBL   UCSC 
 
   67
 
  5
 
 17122
 
 tss
 
 0.02394
 
 0.02369
 
 Not queried
 
  0.6318084
 
       NA
 
       NA
  
 
  
 2
 
 192109990
 
 192110021
 
  ENSEMBL   UCSC 
 
   32
 
  4
 
 23751
 
 tss;island
 
 0.00745
 
 0.01094
 
 Not queried
 
  0.6317324
 
       NA
 
       NA
  
 
  
 18
 
  47792917
 
  47793175
 
  ENSEMBL   UCSC 
 
  259
 
  6
 
 19241
 
 tss
 
 0.01204
 
 0.02745
 
 Not queried
 
  0.6315627
 
       NA
 
       NA
  
 
  
 19
 
   5699060
 
   5699451
 
  ENSEMBL   UCSC 
 
  392
 
  4
 
 19718
 
 gene;island
 
 0.84976
 
 0.90295
 
 Not queried
 
  0.6314860
 
       NA
 
       NA
  
 
  
 5
 
 141705351
 
 141705539
 
  ENSEMBL   UCSC 
 
  189
 
  5
 
 32338
 
 tss;island
 
 0.02193
 
 0.03282
 
 Not queried
 
  0.6314451
 
       NA
 
       NA
  
 
  
 4
 
 100870323
 
 100870750
 
  ENSEMBL   UCSC 
 
  428
 
  6
 
 30022
 
 gene;tss;island
 
 0.03914
 
 0.03256
 
 Not queried
 
  0.6313026
 
       NA
 
       NA
  
 
  
 X
 
  30327545
 
  30327819
 
  ENSEMBL   UCSC 
 
  275
 
  8
 
 41244
 
 tss
 
 0.05805
 
 0.04614
 
 Not queried
 
  0.6312976
 
       NA
 
       NA
  
 
  
 20
 
  21378045
 
  21378278
 
  ENSEMBL   UCSC 
 
  234
 
  8
 
 26000
 
 island
 
 0.02152
 
 0.03434
 
 Not queried
 
  0.6312270
 
       NA
 
       NA
  
 
  
 1
 
 228613073
 
 228613260
 
  ENSEMBL   UCSC 
 
  188
 
  6
 
 2301
 
 tss
 
 0.90755
 
 0.86832
 
 Not queried
 
  0.6311898
 
       NA
 
       NA
  
 
  
 11
 
 105480771
 
 105480979
 
  ENSEMBL   UCSC 
 
  209
 
  5
 
 7632
 
 tss;island
 
 0.12074
 
 0.07401
 
 Not queried
 
  0.6311888
 
       NA
 
       NA
  
 
  
 1
 
 202679089
 
 202679191
 
  ENSEMBL   UCSC 
 
  103
 
  4
 
 944
 
 gene;island
 
 0.03095
 
 0.03002
 
 Not queried
 
  0.6311478
 
       NA
 
       NA
  
 
  
 7
 
   1570060
 
   1570407
 
  ENSEMBL   UCSC 
 
  348
 
  6
 
 38049
 
 island
 
 0.04130
 
 0.07969
 
 Not queried
 
  0.6311217
 
       NA
 
       NA
  
 
  
 6
 
  25732093
 
  25732239
 
  ENSEMBL   UCSC 
 
  147
 
  4
 
 35438
 
 island
 
 0.84441
 
 0.77249
 
 Not queried
 
  0.6309026
 
       NA
 
       NA
  
 
  
 10
 
 111767584
 
 111767867
 
  ENSEMBL   UCSC 
 
  284
 
  7
 
 4738
 
 gene;island
 
 0.04253
 
 0.04705
 
 Not queried
 
  0.6308937
 
       NA
 
       NA
  
 
  
 X
 
  15353660
 
  15354150
 
  ENSEMBL   UCSC 
 
  491
 
 10
 
 41197
 
 tss;island
 
 0.02186
 
 0.03393
 
 Not queried
 
  0.6308315
 
       NA
 
       NA
  
 
  
 8
 
  40755380
 
  40755559
 
  ENSEMBL   UCSC 
 
  180
 
  5
 
 39602
 
 tss
 
 0.03763
 
 0.03486
 
 Not queried
 
  0.6305491
 
       NA
 
       NA
  
 
  
 8
 
  40755165
 
  40755559
 
  ENSEMBL   UCSC 
 
  395
 
  7
 
 40175
 
 island
 
 0.03291
 
 0.02125
 
 Not queried
 
  0.6305491
 
       NA
 
       NA
  
 
  
 18
 
  77631625
 
  77631756
 
  ENSEMBL   UCSC 
 
  132
 
  4
 
 19110
 
 gene;island
 
 0.80600
 
 0.90031
 
 Not queried
 
  0.6305420
 
       NA
 
       NA
  
 
  
 19
 
  49496473
 
  49497177
 
  ENSEMBL   UCSC 
 
  705
 
 13
 
 20899
 
 tss;island
 
 0.02854
 
 0.04344
 
 Not queried
 
  0.6305198
 
       NA
 
       NA
  
 
  
 6
 
  42988922
 
  42989367
 
  ENSEMBL   UCSC 
 
  446
 
 10
 
 34925
 
 tss;island
 
 0.01894
 
 0.02654
 
 Not queried
 
  0.6304969
 
       NA
 
       NA
  
 
  
 16
 
  22012456
 
  22012743
 
  ENSEMBL   UCSC 
 
  288
 
  7
 
 15098
 
 tss
 
 0.06488
 
 0.04047
 
 Not queried
 
  0.6304060
 
       NA
 
       NA
  
 
  
 11
 
 111637186
 
 111637613
 
  ENSEMBL   UCSC 
 
  428
 
  9
 
 7667
 
 tss
 
 0.01065
 
 0.01895
 
 Not queried
 
  0.6303720
 
       NA
 
       NA
  
 
  
 11
 
 111636955
 
 111637613
 
  ENSEMBL   UCSC 
 
  659
 
 11
 
 8780
 
 island
 
 0.01065
 
 0.01950
 
 Not queried
 
  0.6303720
 
       NA
 
       NA
  
 
  
 11
 
  67236733
 
  67237179
 
  ENSEMBL   UCSC 
 
  447
 
  9
 
 7473
 
 tss
 
 0.04391
 
 0.05047
 
 Not queried
 
  0.6302810
 
       NA
 
       NA
  
 
  
 11
 
  65337543
 
  65337913
 
  ENSEMBL   UCSC 
 
  371
 
  5
 
 7411
 
 tss
 
 0.00981
 
 0.01093
 
 Not queried
 
  0.6302183
 
       NA
 
       NA
  
 
  
 5
 
   6378647
 
   6378882
 
  ENSEMBL   UCSC 
 
  236
 
  7
 
 31922
 
 tss
 
 0.02243
 
 0.01025
 
 Not queried
 
  0.6302118
 
       NA
 
       NA
  
 
  
 15
 
  52030001
 
  52030199
 
  ENSEMBL   UCSC 
 
  199
 
  4
 
 13322
 
 gene;island
 
 0.08155
 
 0.03930
 
 Not queried
 
  0.6301567
 
       NA
 
       NA
  
 
  
 11
 
  43902062
 
  43902557
 
  ENSEMBL   UCSC 
 
  496
 
 11
 
 8197
 
 island
 
 0.41738
 
 0.49234
 
 Not queried
 
  0.6301427
 
       NA
 
       NA
  
 
  
 8
 
  95653174
 
  95653368
 
  ENSEMBL   UCSC 
 
  195
 
  5
 
 40395
 
 island
 
 0.04509
 
 0.06422
 
 Not queried
 
  0.6301416
 
       NA
 
       NA
  
 
  
 6
 
  31513198
 
  31513306
 
  ENSEMBL   UCSC 
 
  109
 
  5
 
 33833
 
 gene;tss;island
 
 0.52981
 
 0.57453
 
 Not queried
 
  0.6300047
 
       NA
 
       NA
  
 
  
 3
 
  27410627
 
  27411065
 
  ENSEMBL   UCSC 
 
  439
 
  6
 
 28903
 
 island
 
 0.01828
 
 0.02880
 
 Not queried
 
  0.6299551
 
       NA
 
       NA
  
 
  
 15
 
  25513277
 
  25513654
 
  ENSEMBL   UCSC 
 
  378
 
  4
 
 13507
 
 tss
 
 0.05924
 
 0.14420
 
 Not queried
 
  0.6299487
 
       NA
 
       NA
  
 
  
 1
 
 179545073
 
 179545458
 
  ENSEMBL   UCSC 
 
  386
 
  9
 
 3986
 
 island
 
 0.06961
 
 0.02379
 
 Not queried
 
  0.6298050
 
       NA
 
       NA
  
 
  
 2
 
  15701235
 
  15701450
 
  ENSEMBL   UCSC 
 
  216
 
  6
 
 22543
 
 gene
 
 0.03405
 
 0.05401
 
 Not queried
 
  0.6297487
 
       NA
 
       NA
  
 
  
 1
 
 236925843
 
 236925965
 
  ENSEMBL   UCSC 
 
  123
 
  4
 
 1098
 
 gene
 
 0.94553
 
 0.96133
 
 Not queried
 
  0.6297176
 
       NA
 
       NA
  
 
  
 12
 
 128245397
 
 128245800
 
  ENSEMBL   UCSC 
 
  404
 
  6
 
 10958
 
 island
 
 0.59109
 
 0.51509
 
 Not queried
 
  0.6296769
 
       NA
 
       NA
  
 
  
 3
 
 187463294
 
 187463617
 
  ENSEMBL   UCSC 
 
  324
 
  7
 
 29719
 
 island
 
 0.02099
 
 0.02293
 
 Not queried
 
  0.6295849
 
       NA
 
       NA
  
 
  
 11
 
 111169795
 
 111169961
 
  ENSEMBL   UCSC 
 
  167
 
  5
 
 7660
 
 tss
 
 0.02190
 
 0.02767
 
 Not queried
 
  0.6294697
 
       NA
 
       NA
  
 
  
 5
 
 132148706
 
 132149015
 
  ENSEMBL   UCSC 
 
  310
 
  7
 
 32219
 
 tss
 
 0.01811
 
 0.03389
 
 Not queried
 
  0.6293315
 
       NA
 
       NA
  
 
  
 19
 
  55574425
 
  55574733
 
  ENSEMBL   UCSC 
 
  309
 
  7
 
 20187
 
 gene;island
 
 0.01953
 
 0.03403
 
 Not queried
 
  0.6293215
 
       NA
 
       NA
  
 
  
 16
 
  55542668
 
  55543166
 
  ENSEMBL   UCSC 
 
  499
 
  9
 
 15904
 
 island
 
 0.03259
 
 0.03653
 
 Not queried
 
  0.6293131
 
       NA
 
       NA
  
 
  
 1
 
 160068471
 
 160068842
 
  ENSEMBL   UCSC 
 
  372
 
 13
 
 2032
 
 tss
 
 0.05053
 
 0.13482
 
 Not queried
 
  0.6293051
 
       NA
 
       NA
  
 
  
 12
 
  15373987
 
  15374609
 
  ENSEMBL   UCSC 
 
  623
 
  9
 
 10242
 
 island
 
 0.02654
 
 0.02872
 
 Not queried
 
  0.6292590
 
       NA
 
       NA
  
 
  
 10
 
  73975914
 
  73976264
 
  ENSEMBL   UCSC 
 
  351
 
  4
 
 4614
 
 gene;tss;island
 
 0.02599
 
 0.03472
 
 Not queried
 
  0.6291914
 
       NA
 
       NA
  
 
  
 7
 
 158649018
 
 158649243
 
  ENSEMBL   UCSC 
 
  226
 
  7
 
 37960
 
 tss
 
 0.02214
 
 0.02628
 
 Not queried
 
  0.6291715
 
       NA
 
       NA
  
 
  
 3
 
 121264903
 
 121264942
 
  ENSEMBL   UCSC 
 
   40
 
  4
 
 28488
 
 tss
 
 0.01776
 
 0.01323
 
 Not queried
 
  0.6291363
 
       NA
 
       NA
  
 
  
 19
 
  59084789
 
  59085608
 
  ENSEMBL   UCSC 
 
  820
 
 16
 
 20230
 
 gene;island
 
 0.02110
 
 0.05972
 
 Not queried
 
  0.6291288
 
       NA
 
       NA
  
 
  
 1
 
  33625328
 
  33625438
 
  ENSEMBL   UCSC 
 
  111
 
  4
 
 402
 
 gene;island
 
 0.85053
 
 0.78725
 
 Not queried
 
  0.6291211
 
       NA
 
       NA
  
 
  
 17
 
   3539439
 
   3539754
 
  ENSEMBL   UCSC 
 
  316
 
  8
 
 16981
 
 tss;island
 
 0.02103
 
 0.03106
 
 Not queried
 
  0.6290948
 
       NA
 
       NA
  
 
  
 9
 
 139377930
 
 139378129
 
  ENSEMBL   UCSC 
 
  200
 
  4
 
 40790
 
 tss;island
 
 0.01823
 
 0.03740
 
 Not queried
 
  0.6290559
 
       NA
 
       NA
  
 
  
 12
 
  56882314
 
  56882693
 
  ENSEMBL   UCSC 
 
  380
 
  7
 
 9775
 
 tss;island
 
 0.06925
 
 0.04066
 
 Not queried
 
  0.6290533
 
       NA
 
       NA
  
 
  
 19
 
  18335182
 
  18335429
 
  ENSEMBL   UCSC 
 
  248
 
  5
 
 19896
 
 gene;island
 
 0.01490
 
 0.02957
 
 Not queried
 
  0.6290518
 
       NA
 
       NA
  
 
  
 20
 
   3218393
 
   3218579
 
  ENSEMBL   UCSC 
 
  187
 
  7
 
 25524
 
 tss
 
 0.44367
 
 0.46560
 
 Not queried
 
  0.6289952
 
       NA
 
       NA
  
 
  
 2
 
 176986261
 
 176986659
 
  ENSEMBL   UCSC 
 
  399
 
  4
 
 23698
 
 tss;island
 
 0.50065
 
 0.39420
 
 Not queried
 
  0.6289932
 
       NA
 
       NA
  
 
  
 1
 
 245026373
 
 245026741
 
  ENSEMBL   UCSC 
 
  369
 
  5
 
 1112
 
 gene;island
 
 0.04064
 
 0.06148
 
 Not queried
 
  0.6289906
 
       NA
 
       NA
  
 
  
 6
 
  50803820
 
  50803945
 
  ENSEMBL   UCSC 
 
  126
 
  4
 
 34300
 
 gene
 
 0.58344
 
 0.44926
 
 Not queried
 
  0.6289900
 
       NA
 
       NA
  
 
  
 3
 
  48701524
 
  48701770
 
  ENSEMBL   UCSC 
 
  247
 
  5
 
 28264
 
 tss;island
 
 0.02399
 
 0.02396
 
 Not queried
 
  0.6289236
 
       NA
 
       NA
  
 
  
 7
 
  75115627
 
  75115859
 
  ENSEMBL   UCSC 
 
  233
 
  8
 
 37588
 
 tss
 
 0.02883
 
 0.01863
 
 Not queried
 
  0.6289114
 
       NA
 
       NA
  
 
  
 7
 
 107300939
 
 107301288
 
  ENSEMBL   UCSC 
 
  350
 
  7
 
 37119
 
 gene;island
 
 0.21773
 
 0.23081
 
 Not queried
 
  0.6288873
 
       NA
 
       NA
  
 
  
 1
 
 214557726
 
 214557928
 
  ENSEMBL   UCSC 
 
  203
 
  4
 
 1004
 
 gene;island
 
 0.97053
 
 0.91269
 
 Not queried
 
  0.6288146
 
       NA
 
       NA
  
 
  
 13
 
  23471764
 
  23471968
 
  ENSEMBL   UCSC 
 
  205
 
  4
 
 11565
 
 island
 
 0.91197
 
 0.92538
 
 Not queried
 
  0.6288047
 
       NA
 
       NA
  
 
  
 19
 
  51071307
 
  51071328
 
  ENSEMBL   UCSC 
 
   22
 
  4
 
 20945
 
 tss
 
 0.07693
 
 0.12096
 
 Not queried
 
  0.6286865
 
       NA
 
       NA
  
 
  
 6
 
  34123215
 
  34123609
 
  ENSEMBL   UCSC 
 
  395
 
  5
 
 35989
 
 island
 
 0.76200
 
 0.69082
 
 Not queried
 
  0.6286516
 
       NA
 
       NA
  
 
  
 5
 
 131563492
 
 131564050
 
  ENSEMBL   UCSC 
 
  559
 
 10
 
 32208
 
 tss;island
 
 0.01405
 
 0.03676
 
 Not queried
 
  0.6286155
 
       NA
 
       NA
  
 
  
 1
 
  90228963
 
  90229095
 
  ENSEMBL   UCSC 
 
  133
 
  4
 
 3444
 
 island
 
 0.07295
 
 0.03933
 
 Not queried
 
  0.6285665
 
       NA
 
       NA
  
 
  
 17
 
  27507501
 
  27507678
 
  ENSEMBL   UCSC 
 
  178
 
  8
 
 17198
 
 tss;island
 
 0.04087
 
 0.02316
 
 Not queried
 
  0.6285519
 
       NA
 
       NA
  
 
  
 6
 
  32078398
 
  32078624
 
  ENSEMBL   UCSC 
 
  227
 
  8
 
 34798
 
 tss
 
 0.56374
 
 0.72926
 
 Not queried
 
  0.6284783
 
       NA
 
       NA
  
 
  
 19
 
   7984832
 
   7985219
 
  ENSEMBL   UCSC 
 
  388
 
  8
 
 20375
 
 tss;island
 
 0.04599
 
 0.03684
 
 Not queried
 
  0.6284771
 
       NA
 
       NA
  
 
  
 15
 
  35086890
 
  35086985
 
  ENSEMBL   UCSC 
 
   96
 
  4
 
 13251
 
 gene
 
 0.95053
 
 0.91371
 
 Not queried
 
  0.6283764
 
       NA
 
       NA
  
 
  
 12
 
  70759665
 
  70760024
 
  ENSEMBL   UCSC 
 
  360
 
  6
 
 9842
 
 tss
 
 0.01381
 
 0.03872
 
 Not queried
 
  0.6283234
 
       NA
 
       NA
  
 
  
 5
 
    462141
 
    462522
 
  ENSEMBL   UCSC 
 
  382
 
  5
 
 31450
 
 gene;island
 
 0.92520
 
 0.95148
 
 Not queried
 
  0.6281715
 
       NA
 
       NA
  
 
  
 4
 
  41936852
 
  41937034
 
  ENSEMBL   UCSC 
 
  183
 
  4
 
 30317
 
 tss
 
 0.25985
 
 0.28186
 
 Not queried
 
  0.6280939
 
       NA
 
       NA
  
 
  
 11
 
  46958255
 
  46958558
 
  ENSEMBL   UCSC 
 
  304
 
  4
 
 6585
 
 gene
 
 0.03823
 
 0.06124
 
 Not queried
 
  0.6280841
 
       NA
 
       NA
  
 
  
 6
 
  31782675
 
  31782761
 
  ENSEMBL   UCSC 
 
   87
 
  4
 
 33904
 
 gene
 
 0.64788
 
 0.64344
 
 Not queried
 
  0.6280705
 
       NA
 
       NA
  
 
  
 11
 
 120195717
 
 120195905
 
  ENSEMBL   UCSC 
 
  189
 
  5
 
 7740
 
 tss;island
 
 0.08978
 
 0.06000
 
 Not queried
 
  0.6280622
 
       NA
 
       NA
  
 
  
 6
 
  35995246
 
  35995679
 
  ENSEMBL   UCSC 
 
  434
 
 12
 
 36018
 
 island
 
 0.01640
 
 0.02599
 
 Not queried
 
  0.6280354
 
       NA
 
       NA
  
 
  
 7
 
   1543936
 
   1544333
 
  ENSEMBL   UCSC 
 
  398
 
  6
 
 38048
 
 island
 
 0.01427
 
 0.02032
 
 Not queried
 
  0.6280135
 
       NA
 
       NA
  
 
  
 12
 
 131589169
 
 131589455
 
  ENSEMBL   UCSC 
 
  287
 
  5
 
 9400
 
 gene
 
 0.34355
 
 0.35829
 
 Not queried
 
  0.6279766
 
       NA
 
       NA
  
 
  
 17
 
  42785825
 
  42785946
 
  ENSEMBL   UCSC 
 
  122
 
  5
 
 17381
 
 tss
 
 0.00541
 
 0.00874
 
 Not queried
 
  0.6278606
 
       NA
 
       NA
  
 
  
 17
 
  42785825
 
  42786055
 
  ENSEMBL   UCSC 
 
  231
 
  7
 
 18374
 
 island
 
 0.00554
 
 0.00955
 
 Not queried
 
  0.6278606
 
       NA
 
       NA
  
 
  
 6
 
 147727982
 
 147728290
 
  ENSEMBL   UCSC 
 
  309
 
  5
 
 36485
 
 island
 
 0.94173
 
 0.91539
 
 Not queried
 
  0.6278038
 
       NA
 
       NA
  
 
  
 2
 
 216878340
 
 216878510
 
  ENSEMBL   UCSC 
 
  171
 
  7
 
 25031
 
 island
 
 0.01481
 
 0.03731
 
 Not queried
 
  0.6277678
 
       NA
 
       NA
  
 
  
 17
 
  80668611
 
  80668944
 
  ENSEMBL   UCSC 
 
  334
 
  5
 
 18973
 
 island
 
 0.58646
 
 0.67570
 
 Not queried
 
  0.6276422
 
       NA
 
       NA
  
 
  
 4
 
  77997182
 
  77997201
 
  ENSEMBL   UCSC 
 
   20
 
  4
 
 30405
 
 tss;island
 
 0.00897
 
 0.01319
 
 Not queried
 
  0.6276377
 
       NA
 
       NA
  
 
  
 6
 
 167031273
 
 167031411
 
  ENSEMBL   UCSC 
 
  139
 
  4
 
 34484
 
 gene;island
 
 0.95133
 
 0.97168
 
 Not queried
 
  0.6276090
 
       NA
 
       NA
  
 
  
 5
 
 145826525
 
 145826831
 
  ENSEMBL   UCSC 
 
  307
 
  6
 
 32348
 
 tss
 
 0.01867
 
 0.01746
 
 Not queried
 
  0.6275839
 
       NA
 
       NA
  
 
  
 10
 
 119134981
 
 119135296
 
  ENSEMBL   UCSC 
 
  316
 
  5
 
 5373
 
 tss
 
 0.01511
 
 0.02420
 
 Not queried
 
  0.6275828
 
       NA
 
       NA
  
 
  
 19
 
   1851750
 
   1852165
 
  ENSEMBL   UCSC 
 
  416
 
  5
 
 21212
 
 island
 
 0.86974
 
 0.84988
 
 Not queried
 
  0.6275010
 
       NA
 
       NA
  
 
  
 12
 
  72667326
 
  72667707
[truncated: 3,110,195 more chars]
